# Increased transmissibility and global spread of SARS-CoV-2 variants of concern as at June 2021

**DOI:** 10.2807/1560-7917.ES.2021.26.24.2100509

**Published:** 2021-06-17

**Authors:** Finlay Campbell, Brett Archer, Henry Laurenson-Schafer, Yuka Jinnai, Franck Konings, Neale Batra, Boris Pavlin, Katelijn Vandemaele, Maria D Van Kerkhove, Thibaut Jombart, Oliver Morgan, Olivier le Polain de Waroux

**Affiliations:** 1 Health Emergencies Programme, World Health Organization, Geneva, Switzerland; 2 London School of Hygiene and Tropical Medicine, London, United Kingdom; 3 Imperial College London, London, United Kingdom

**Keywords:** SARS-CoV-2, COVID-19, Variants of concern, Variants of interest

## Abstract

We present a global analysis of the spread of recently emerged SARS-CoV-2 variants and estimate changes in effective reproduction numbers at country-specific level using sequence data from GISAID. Nearly all investigated countries demonstrated rapid replacement of previously circulating lineages by the World Health Organization-designated variants of concern, with estimated transmissibility increases of 29% (95% CI: 24–33), 25% (95% CI: 20–30), 38% (95% CI: 29–48) and 97% (95% CI: 76–117), respectively, for B.1.1.7, B.1.351, P.1 and B.1.617.2.

Recent months have seen the emergence and rapid spread of severe acute respiratory syndrome coronavirus 2 (SARS-CoV-2) variants associated with increased transmissibility, including the World Health Organization (WHO)-designated variants of concern (VOC) Alpha (hereafter referred to using the Phylogenetic Assignment of Named Global Outbreak (Pango) lineage designation B.1.1.7), Beta (B.1.351), Gamma (P.1) and Delta (B.1.617.2), as well as multiple variants of interest (VOI) [1]. By 3 June 2021, B.1.1.7 had been reported from at least 160 countries, B.1.351 from 113 countries, P.1 from 64 countries and B.1.617.2 from 62 countries [1]. We present an analysis of the effective reproduction number and global spread of SARS-CoV-2 variants with data available by 3 June 2021.

## Effective reproduction number estimates

We analysed 1,722,652 SARS-CoV-2 sequences uploaded to the Global Initiative On Sharing All Influenza Data (GISAID) hCoV-19 database [2], considering only VOC or VOI reported at least 25 times in at least three countries (see Supplementary Tables S1 and S2 for sequence numbers per variant per country). GISAID sequences used for this work are acknowledged in Supplement 2. We used a multinomial logistic model of competitive growth to estimate the effective reproduction number of each variant relative to that of the non-VOC/VOI viral population for each reporting country. We assumed that the generation time of VOC/VOI remained unchanged compared with previously circulating variants. Further details on the methods, as well as an exploration of the sensitivity of our results to the assumption of an unchanged generation time, can be found in the Supplementary Material.

**Figure 1:**
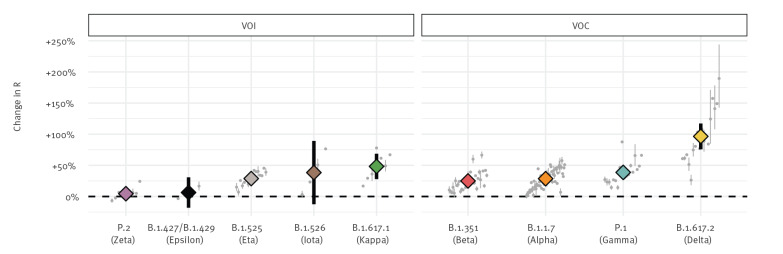
Estimated change in effective reproduction number of SARS-CoV-2 variants relative to non-variants, 64 countries, data until 3 June 2021

**Figure 2:**
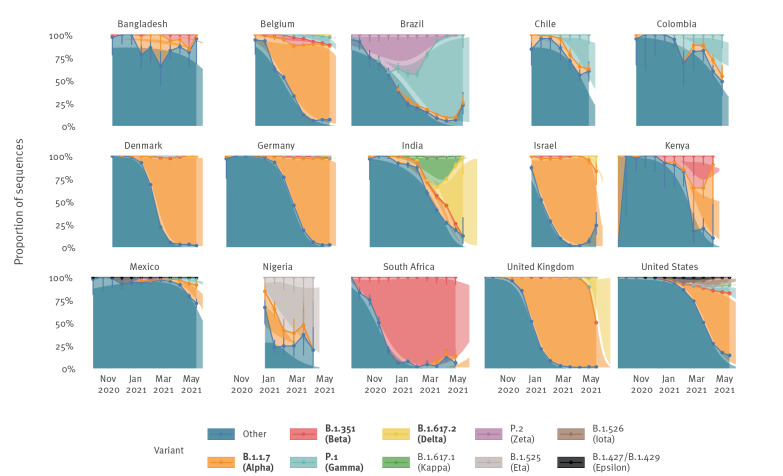
Empirical and modelled variant proportions of SARS-CoV-2 variants over time, 15 countries, data until 3 June 2021

Despite differences between countries, our analysis showed a statistically significant increase in the pooled mean effective reproduction number relative to non-VOC/VOI of B.1.1.7 at 29% (95% confidence interval (CI): 24–33), B.1.351 at 25% (95% CI: 20–30), P.1 at 38% (95% CI: 29–48) and B.1.617.2 at 97% (95% CI: 76–117) (Figure 1). Of the six variants currently designated as VOI, five were considered in our analysis and among these, only B.1.617.1 and B.1.525 demonstrated a statistically significant increase in the effective reproduction number of 48% (95% CI: 28–69) and 29% (95% CI: 23–35), respectively. In line with these estimates, our results showed rapid replacement of previously circulating variants by VOC/VOI in nearly all countries; of the 64 countries considered in this analysis, we estimate VOC/VOI to be the most frequently circulating lineage on the last day of available data in 52 countries, the most common variants being B.1.1.7 (40 countries) and B.1.617.2 (India, Singapore, United Kingdom and Australia) (Figure 2, Supplementary Figures S1 and S2).

R: effective reproduction number; SARS-CoV-2: severe acute respiratory syndrome coronavirus 2; VOC: variant of concern; VOI: variant of interest.

Points and lines represent the mean and 95% confidence intervals. Coloured: pooled estimate (random effects model); grey: individual country estimates. GISAID sequences used for this work are acknowledged in Supplement 2.

SARS-CoV-2: severe acute respiratory syndrome coronavirus 2.

Points and lines represent stacked empirical variant proportions, coloured areas between lines represent absolute empirical proportions, error bars represent multinomial 95% confidence intervals. Smoothed coloured areas represent stacked modelled variant proportions, white areas represent 95% confidence intervals of the modelled estimates. Darker shaded areas therefore represent overlap between empirical and modelled estimates. VOC are indicated in bold text, VOI in plain text.

Countries were selected to represent different geographical regions; estimates for all countries are provided in the Supplementary Figures S1 and S2). Countries, territories and areas as reported in GISAID do not imply the expression of any opinion whatsoever on the part of World Health Organization concerning the legal status of any country, territory or area or of its authorities. GISAID sequences used for this work are acknowledged in Supplement 2.

**Figure 3:**
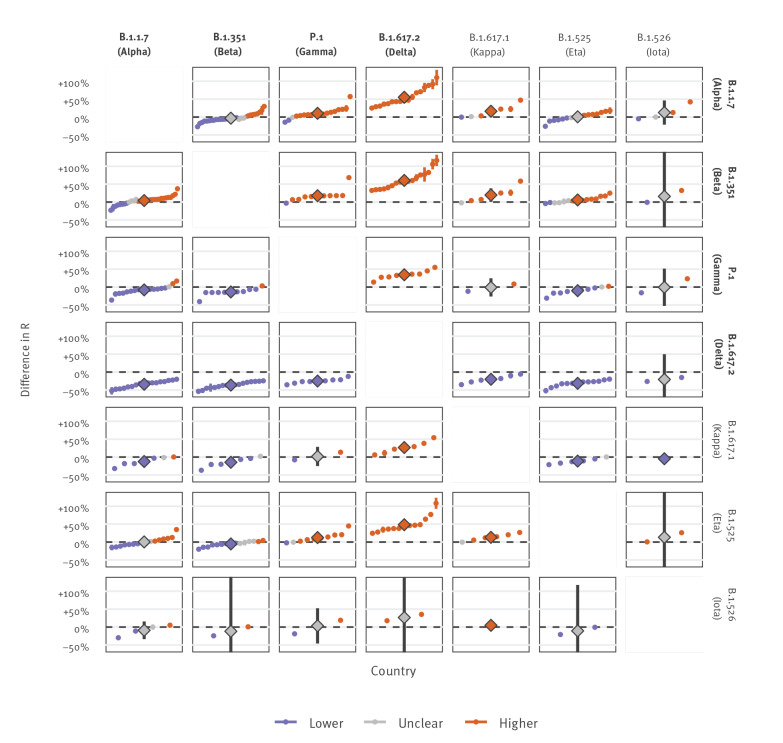
Effective reproduction number of SARS-CoV-2 variants of concern/interest compared against each other, 64 countries, data until 3 June 2021

Given the widespread co-circulation of VOC/VOI, we also compared the effective reproduction numbers of these variants against each in order to estimate the nature of future competitive growth between them (Figure 3, excluding P.2 and B.1.427/B.1.429). Notably, the pooled mean difference in the effective production number between the VOC B.1.1.7 and B.1.351 was small at 4% (95% CI: 0–8), while P.1 demonstrated an increase relative to B.1.1.7 and B.1.351 of 10% (95% CI: 3–17) and 17% (95% CI: 6–30). Given these estimates, the longer-term trends of competitive growth between these three VOC remain unclear.

R: effective reproduction number; VOC: variant of concern; VOI: variant of interest.

Small points and thin lines represent country-specific mean and 95% confidence intervals of the change in the effective reproduction number of the variant indicated in the top row relative to the variant indicated in the column on the right (confidence intervals are too narrow to be visible for some estimates). Diamonds and thick lines represent the mean and 95% confidence of the pooled estimate across all countries (random effects model). VOC are indicated in bold text, VOI in plain text. GISAID sequences used for this work are acknowledged in Supplement 2.

In contrast, the rapid observed growth of B.1.617.2 suggests a clear competitive advantage compared with B.1.1.7, B.1.351 and P.1, with estimated increases in the effective reproduction number of 55% (95% CI: 43–68), 60% (95% CI: 48–73) and 34% (95% CI: 26–43) respectively.

## Ethical statement

Ethical approval was not required for this study.

## Discussion

In this analysis we have highlighted the global spread of SARS-CoV-2 variants and estimated their relative transmission rates. Given our estimates and all other factors remaining constant, B.1.617.2 is expected to rapidly outcompete other variants and become the dominant circulating lineage over the coming months.

These estimates must be considered in light of potential sources of bias in estimating effective reproduction numbers from proportions calculated from GISAID data. Firstly, individual country estimates are likely to be biased as data are often not representative of the variants circulating in the country and the metadata required to account for this are generally not available. Although sequences labelled as being obtained from incoming travellers were assigned to the country of departure in these analyses, bias could still have been introduced if reported sequences disproportionately represented clusters and regions with known circulation of a given variant. This applies especially to B.1.617.1 and B.1.617.2, which have only recently been declared VOC/VOI and are subject to targeted sequencing for example in the United Kingdom (UK) [3].

Secondly, bias can be introduced by founder effects where variants introduced into populations with locally elevated transmission can increase in proportion at a national level without a bona fide transmission advantage. Once again, this bias is most likely to affect the B.1.617 sublineages, as these variants have only recently been introduced in most countries and may still be circulating in the founder demographic, while variants introduced earlier are likely to be circulating in the wider population. Given these two sources of bias, our estimates for B.1.617.1 and B.167.2 probably represent an upper limit on the true value.

Furthermore, we assume that the delays between sampling and submission to GISAID are independent of the variant; if this is not the case, variant proportions in recent GISAID data will be biased towards variants with shorter submission delays. Finally, the country weightings in the pooled transmissibility estimates consider only the number of sequences submitted and not the size of the actual epidemics in each country, biasing estimates towards countries with higher sequencing capacity.

However, in spite of these limitations, the average trends observed across a number of countries with different sequencing strategies and epidemiological contexts are likely to be robust to these sources of bias and representative of true increases in the effective reproduction number, consistent with results from epidemiological studies from various countries [4,5]; our transmissibility estimates are therefore most reliable for variants reported in many countries (see Supplementary Tables S1 and S2). For B.1.617.2, our estimate of a 55% (95% CI: 43–68) increase in the effective reproduction number compared with B.1.1.7 is marginally greater than but has overlapping CI with the increase in the secondary attack rate of 42% (95% CI: 36–48) estimated from epidemiological investigations in the UK [6].

It is important to note that our analysis cannot distinguish between a genuine increase in transmissibility (i.e. the basic reproduction number) and immune evasion as explanations for higher effective reproduction numbers. For variants with relevant levels of immune evasion, as potentially observed for B.1.351 [7], the future nature of competitive growth with other variants will depend on the immune context, both infection- and vaccine-derived, of each country under consideration.

The more rapid growth and widespread prevalence of VOC pose challenges to the control of SARS-CoV-2 worldwide, especially with the recent emergence of B.1.617.2. Despite the emergence and rapid replacement by more transmissible VOC, several countries have successfully reduced SARS-CoV-2 transmission with the use of available and proven public health and social measures (PHSM). Evidence has shown that the higher transmissibility of VOC has required increases in the duration or stringency of PHSM (as elaborated in the WHO interim guidance [8]) in order to achieve the same levels of reduction as before VOC circulation [9]. The increased transmissibility of VOC will probably also lead to a higher community immunity threshold, which may additionally mean that PHSM may need to be maintained for longer periods of time as vaccines are being rolled out. As the virus continues to evolve, the degree of protection offered by the different vaccines against future VOC/VOI remains unclear; vaccination coverage targets themselves may need to be revised [10]. Lastly, given that higher transmissibility has increased case numbers in countries where VOC are circulating and the fact that some VOC are suggested to be associated with higher rates of hospitalisation and mortality [11], the burden on healthcare systems per coronavirus disease (COVID-19) case is likely to increase, although this effect will depend on vaccination coverage and efficacy.

The convergent evolution of mutations thought to be associated with higher transmissibility or immune escape in VOC (e.g. N501Y, E484K) highlights the fact that variants will probably continue to emerge under selective pressures such as PHSM and population immunity [7]. The emergence of new variants threatens the effectiveness of vaccines and requires constant evaluation of available diagnostic, therapeutic, PHSM and vaccination strategies as the COVID-19 pandemic continues. The WHO has established a SARS-CoV-2 Virus Evolution Working group to critically evaluate variants and a Global Risk Assessment and Monitoring Framework for SARS-CoV-2 variants to harmonise the decision-making processes for assessing the impact of VOC on public health and medical interventions [12].

## Conclusion

Rapid replacement means that epidemiological assessment of new VOI must be conducted quickly and regularly if PHSM are to continue to reduce the spread of SARS-CoV-2. Given limitations and inherent delays in detecting emerging variants and investigating their phenotypic impacts, the use and adjustment of PHSM should continue to be informed by traditional epidemiological surveillance. Critically, the capacity to detect and respond to new variants requires continued efforts to enhance surveillance and sequencing capacity globally. Enhanced efforts are needed to ensure better geographical representativeness of available SARS-CoV-2 sequences and the rapid sharing of sequences and metadata for analysis and to inform public health decision making. 

## Supplementary Data

1146000 Supplement

## Supplementary Data

2383000 Supplement2

## References

[1] World Health Organization (WHO). COVID-19 weekly epidemiological update, 1 June 2021. Geneva: WHO; 2021. Available from: https://apps.who.int/iris/handle/10665/341622

[2] Elbe S Buckland-Merrett G. Data, disease and diplomacy: GISAID’s innovative contribution to global health. Glob Chall. 2017;1(1):33-46. 10.1002/gch2.1018 31565258 PMC6607375

[3] Department of Health and Social Care. New measures to boost response to the B1.617.2 variant. Press release. London: GOV.UK; 13 May 2021. Available from: https://www.gov.uk/government/news/new-measures-to-boost-response-to-the-b16172-variant

[4] Centers for Disease Control and Prevention (CDC). COVID data tracker. Atlanta: CDC. [Accessed: 13 Apr 2021]. Available from: https://covid.cdc.gov/covid-data-tracker

[5] Public Health England (PHE). Investigation of novel SARS-CoV-2 variants of concern: technical briefings. London: PHE; 2021. [Accessed: 13 Apr 2021]. Available from: https://www.gov.uk/government/publications/investigation-of-novel-sars-cov-2-variant-variant-of-concern-20201201

[6] Public Health England (PHE). SARS-CoV-2 variants of concern and variants under investigation in England. Technical briefing 15. London: PHE; 11 June 2021. Available from: https://assets.publishing.service.gov.uk/government/uploads/system/uploads/attachment_data/file/993198/Variants_of_Concern_VOC_Technical_Briefing.pdf

[7] Madhi SA Baillie V Cutland CL Voysey M Koen AL Fairlie L Efficacy of the ChAdOx1 nCoV-19 Covid-19 vaccine against the B.1.351 variant. N Engl J Med. 2021;384(20):1885-98. 10.1056/NEJMoa2102214 33725432 PMC7993410

[8] World Health Organization (WHO). Considerations for implementing and adjusting public health and social measures in the context of COVID-19: interim guidance, 4 November 2020. Geneva: WHO; 2020. Available from: https://apps.who.int/iris/handle/10665/336374

[9] Davies NG Abbott S Barnard RC Jarvis CI Kucharski AJ Munday JD Estimated transmissibility and impact of SARS-CoV-2 lineage B.1.1.7 in England. Science. 2021;372(6538):eabg3055. 10.1126/science.abg3055 33658326 PMC8128288

[10] Hodgson D Flasche S Jit M Kucharski AJ CMMID COVID-19 Working Group Centre for Mathematical Modelling of Infectious Disease (CMMID) COVID-19 Working Group. The potential for vaccination-induced herd immunity against the SARS-CoV-2 B.1.1.7 variant. Euro Surveill. 2021;26(20):2100428. 10.2807/1560-7917.ES.2021.26.20.2100428 34018481 PMC8138959

[11] Davies NG Jarvis CI Edmunds WJ Jewell NP Diaz-Ordaz K Keogh RH Increased mortality in community-tested cases of SARS-CoV-2 lineage B.1.1.7. Nature. 2021;593(7858):270-4. 10.1038/s41586-021-03426-1 33723411 PMC9170116

[12] World Health Organization (WHO). COVID-19 weekly epidemiological update, 13 April 2021. Geneva: WHO; 2021. Available from: https://apps.who.int/iris/handle/10665/340826

