# Supplementary material for: Increased transmissibility and global spread of SARS-CoV-2 variants of concern as at June 2021
Source: Euro Surveill. 2021 Jun 17;26(24):2100509. doi: 10.2807/1560-7917.ES.2021.26.24.2100509 (PMC8212592; doi:10.2807/1560-7917.ES.2021.26.24.2100509)
Supplement: Supplement [file 21-00509_CAMPBELL_supplement.pdf]

Supplementary Materials

This supplementary material is hosted by Eurosurveillance *as supporting information alongside the article "Increased transmissibility and global spread of SARS-CoV-2 variants of concern", on behalf of the authors, who remain responsible for the accuracy and appropriateness of the content. The same standards for ethics, copyright, attributions and permissions as for the article apply. Supplements are not edited by* Eurosurveillance *and the journal is not responsible for the maintenance of any links or email addresses provided therein.*

Supplementary Text

Supplementary Text 1: Estimating growth rates

The number of individuals infected by a given variant v on day t under a simple logistic growth model is given by:

$$I_v(t) = p_v e^{r_v t}$$

where r_v is the growth rate and p_v the number of infectious individuals on day $t = 0$ for variant v. We note r_{wt} the growth rate of non-VOC/VOI variants, referred to as "wild-type". The transmissibility of a variant v can be modelled using an additive term Δ_v so that its growth rate is defined as r_{wt} + Δv and we have:

$$I_v(t) = p_v e^{(r_{wt}+\Delta_v)t}$$

The proportion among cases of a variant v at time t, and therefore the probability of a sequence S_i randomly sampled at time T_i being variant v, is given by:

$$Pr(S_i = v) = \frac{p_v e^{(r_{wt}+\Delta_v)T_i}}{\sum_{j=1}^{N_V} p_j e^{(r_{wt}+\Delta_j)T_i}}$$

where N_v is the total number of variants circulating in the population. This can be simplified as follows:

$$Pr(S_i = v) = \frac{p_v e^{(r_{wt}+\Delta_v)T_i}}{p_{wt} e^{r_{wt}T_i} + \sum_{j=2}^{N_V} p_j e^{(r_{wt}+\Delta_j)T_i}}$$

$$Pr(S_i = v) = \frac{\frac{p_v}{p_{wt}} e^{\Delta_v T_i}}{1 + \sum_{j=2}^{N_V} \frac{p_j}{p_{wt}} e^{\Delta_j T_i}}$$

$$Pr(S_i = v) = \frac{e^{\log\left(\frac{p_v}{p_{wt}}\right)+\Delta_i T_i}}{1 + \sum_{j=2}^{N_V} e^{\log\left(\frac{p_j}{p_{wt}}\right)+\Delta_j T_i}}$$

This model can be fitted using multinomial logistic regression, where the observed categorical outcome is the variant v of a given sequence S_i and the single explanatory variable is the time of sampling T_i.

For multinomial logistic regression with a single explanatory variable $x_{1,i}$, the linear predictor function of the probability that observation i has outcome v is given by:

$$f(v,i) = \beta_{0,v} + \beta_{1,v} x_{1,i}$$

which, given a logit link function, defines the absolute probability that the observation (i.e. the sequence) S_i has outcome v, if the outcome $v = 1 = wt$ is chosen as the "pivot", as:

$$Pr(S_i = v) = \frac{e^{\beta_{0,v}+\beta_{1,v}x_{1,i}}}{1+\sum_{j=2}^{N_V} e^{\beta_{0,j}+\beta_{1,j}x_{1,i}}}$$

By using the time of sampling T_i as the explanatory variable $x_{1,i}$, it can be seen that the coefficients of the multinomial logistic regression relate to the logistic growth model as follows:

$$\beta_{0,v} = \log\left(\frac{p_v}{p_{wt}}\right)$$

$$\beta_{1,v} = \Delta_{\mathrm{v}}$$

This regression model was implemented and fitted using the *multinom* function from the **nnet** package in the R statistical software (R Project for Statistical Computing, r-project.org).

Supplementary Text 2: Estimating reproduction numbers

To relate estimated differences in growth rates to changes in the reproduction number R and generation time distribution, we used the formulation described by Park *et al.*[1] which assumes a Gamma distributed generation time distribution parameterized by the mean $\bar{G}$ and squared coefficient of variation κ:

$$R = (1+\kappa\bar{G}r)^{1/\kappa}$$

The reproduction number R_v of a variant v can be defined relative to the growth rate of the wild-type strain r_{wt} and the difference in growth rates Δ_{v}:

$$R_v = \left(1+\kappa_v\overline{G_v}(r_{wt}+\Delta_v)\right)^{1/\kappa_v}$$

This equation can be re-defined using the equivalence:

$$r_{wt} = \frac{R_{wt}^{\kappa_{wt}}-1}{\kappa_{wt}\overline{G_{wt}}}$$

to generate:

$$R_v = \left(1+\kappa_v\overline{G_v}\left(\frac{R_{wt}^{\kappa_{wt}}-1}{\kappa_{wt}\overline{G_{wt}}}+\Delta_v\right)\right)^{1/\kappa_v}$$

which simplifies to:

$$R_v = \left(1+\kappa_v\overline{G_v}\Delta_v+\left(R_{wt}^{\kappa_{wt}}-1\right)\frac{\kappa_v\overline{G_v}}{\kappa_{wt}\overline{G_{wt}}}\right)^{1/\kappa_v}$$

The increase in the reproduction number of a variant v relative to the wild-type strain is therefore:

$$\frac{R_v}{R_{wt}}-1 = \frac{\left(1+\kappa_v\overline{G_v}\Delta_v+\left(R_{wt}^{\kappa_{wt}}-1\right)\frac{\kappa_v\overline{G_v}}{\kappa_{wt}\overline{G_{wt}}}\right)^{1/\kappa_v}}{R_{wt}}-1$$

Under the assumption that the coefficient of variation of the generation time distribution is the same between variants in that $\kappa_v = \kappa_{wt}$:

$$\frac{R_v}{R_{wt}} - 1 = \frac{\left(1 + \kappa_{wt}\overline{G_v}\Delta_v + \left(R_{wt}^{\kappa_{wt}} - 1\right)\frac{\overline{G_v}}{\overline{G_{wt}}}\right)^{1/\kappa_{wt}}}{R_{wt}} - 1$$

This formulation shows that the ratio of reproduction numbers is dependent on the value of R_{wt}. However, the value of R_{wt} cannot be directly estimated during the period of variant co-circulation as only the composite reproduction number R_{tot}, defined as average reproduction number across the circulating variants weighted by variant prevalence, is observed.

R_{wt} can be calculated from R_{tot} with knowledge of the variant proportions over time, which are directly estimated in the multinomial regression model and given by $Pr(S_i = v)$, the differences in growth rates Δ_v also estimated above, the wild-type generation time distribution (i.e. the mean $\overline{G_{wt}}$ and the squared coefficient of variation κ_{wt}), and the mean generation time of the variant $\overline{G_v}$, which is unknown but can be explored in sensitivity analyses:

$$R_{tot} = \Pr(S_i = wt)\, R_{wt} + \sum_{j=2}^{N_V} \Pr(S_i = j)\left(1 + \kappa_{wt}\overline{G_J}\Delta_j + \left(R_{wt}^{\kappa_{wt}} - 1\right)\frac{\overline{G_J}}{\overline{G_{wt}}}\right)^{1/\kappa_{wt}}$$

This equivalence was solved numerically for R_{wt} using daily, country-specific estimates of R_{tot} taken from publicly available sources.[2] Daily estimates of R_{wt} were used to calculate the daily relative increase in the reproduction number for all variants, which was averaged across the period for which $0.01 < \Pr(S_i = wt) < 0.99$.

The wild-type generation time distribution was assumed to have a mean $\overline{G_{wt}}$ of 5.0 days and a standard deviation of 1.9 days[3], giving a squared coefficient of variation $\kappa_{wt} = 0.14$. Sensitivity of R_v estimates to changes in the mean generation $\overline{G_v}$ time of the variants were explored using a range from -30% to +30% relative to wild-type (i.e. 3.5 days to 6.5 days).

Supplementary Text 3: Model fitting

The multinomial growth model was fitted to countries with at least 250 sequences submitted since 1 November 2020. Variants were included if at least 25 sequences were submitted by a given country and were designated as non-VOC/VOI otherwise. Variant sublineages were grouped into the parent lineage (e.g. B.1.525.1 was classified as B.1.525). The country of exposure was used instead of the country of reporting to exclude targeted sequencing of incoming travelers.

Supplementary References

1. Park SW, Champredon D, Weitz JS, Dushoff J. A practical generation-interval-based approach to inferring the strength of epidemics from their speed. Epidemics. 2019 Jun 1;27:12–8.

2. Abbott S, Bennett C, Hickson J, Allen J, Sherratt K, Funk S. National Reproduction Number (R) Based on Reported Cases [Internet]. Harvard Dataverse; 2021 [cited 2021 Apr 9]. Available from: https://dataverse.harvard.edu/dataset.xhtml?persistentId=doi:10.7910/DVN/ER7GJH

3. Ferretti L, Wymant C, Kendall M, Zhao L, Nurtay A, Abeler-Dörner L, et al. Quantifying SARS-CoV-2 transmission suggests epidemic control with digital contact tracing. Science [Internet]. 2020 May 8 [cited 2021 Mar 11];368(6491). Available from: https://science.sciencemag.org/content/368/6491/eabb6936

Supplementary Figures

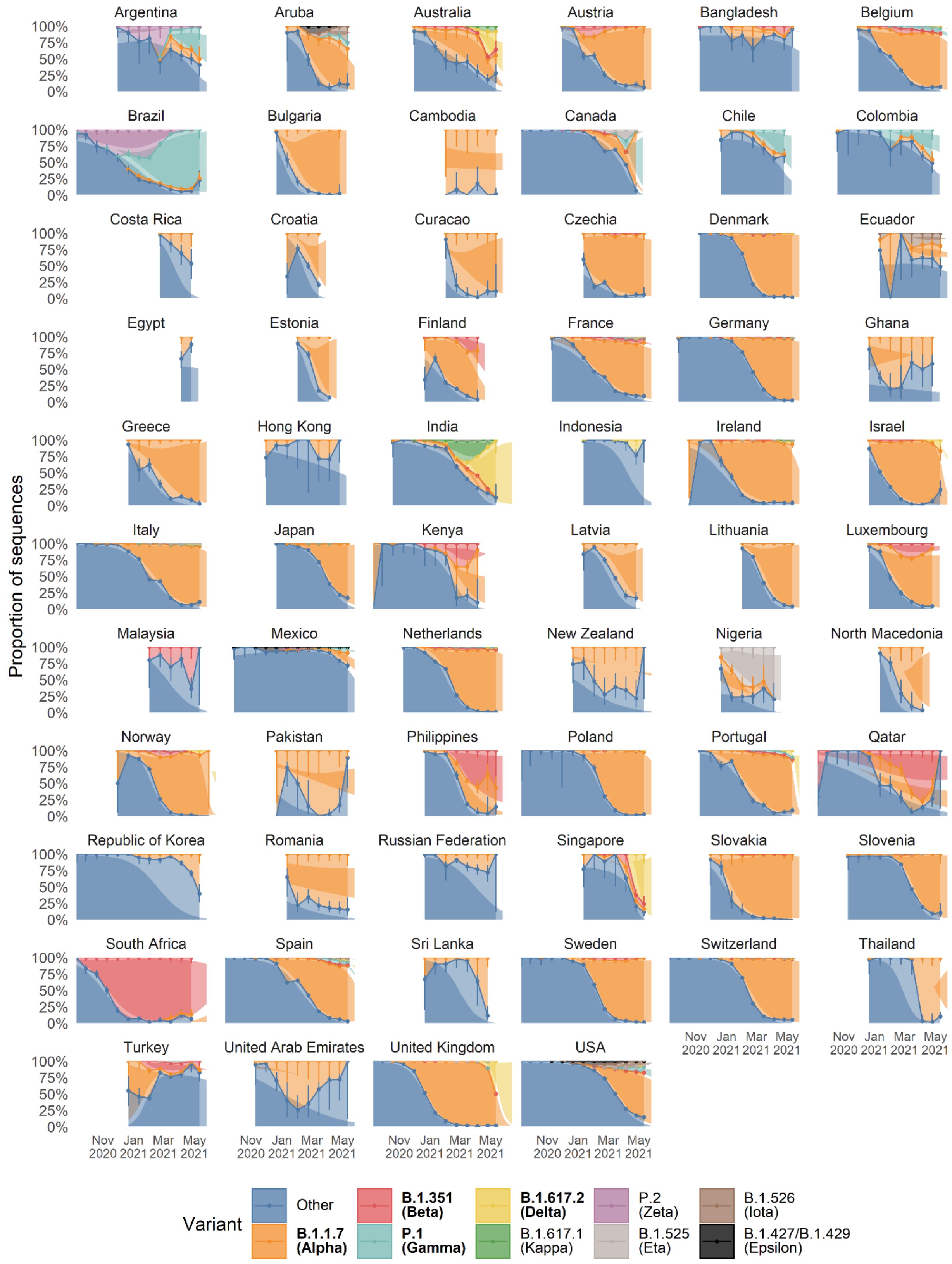

Supplementary Figure S1. Empirical and modelled variant proportions over time for selected countries. Points and lines represent stacked empirical variant proportions, coloured areas between lines represent absolute empirical proportions, error bars represent multinomial 95% confidence intervals. Smoothed coloured areas represent stacked modelled variant proportions, white areas represent 95% confidence intervals of the modelled estimates. Darker shaded areas therefore represent overlap between empirical and modelled estimates. VOCs are indicated in bold text, VOIs in plain text. Countries, territories and areas as reported in GISAID do not imply the expression of any opinion whatsoever on the part of WHO concerning the legal status of any country, territory or area or of its authorities.

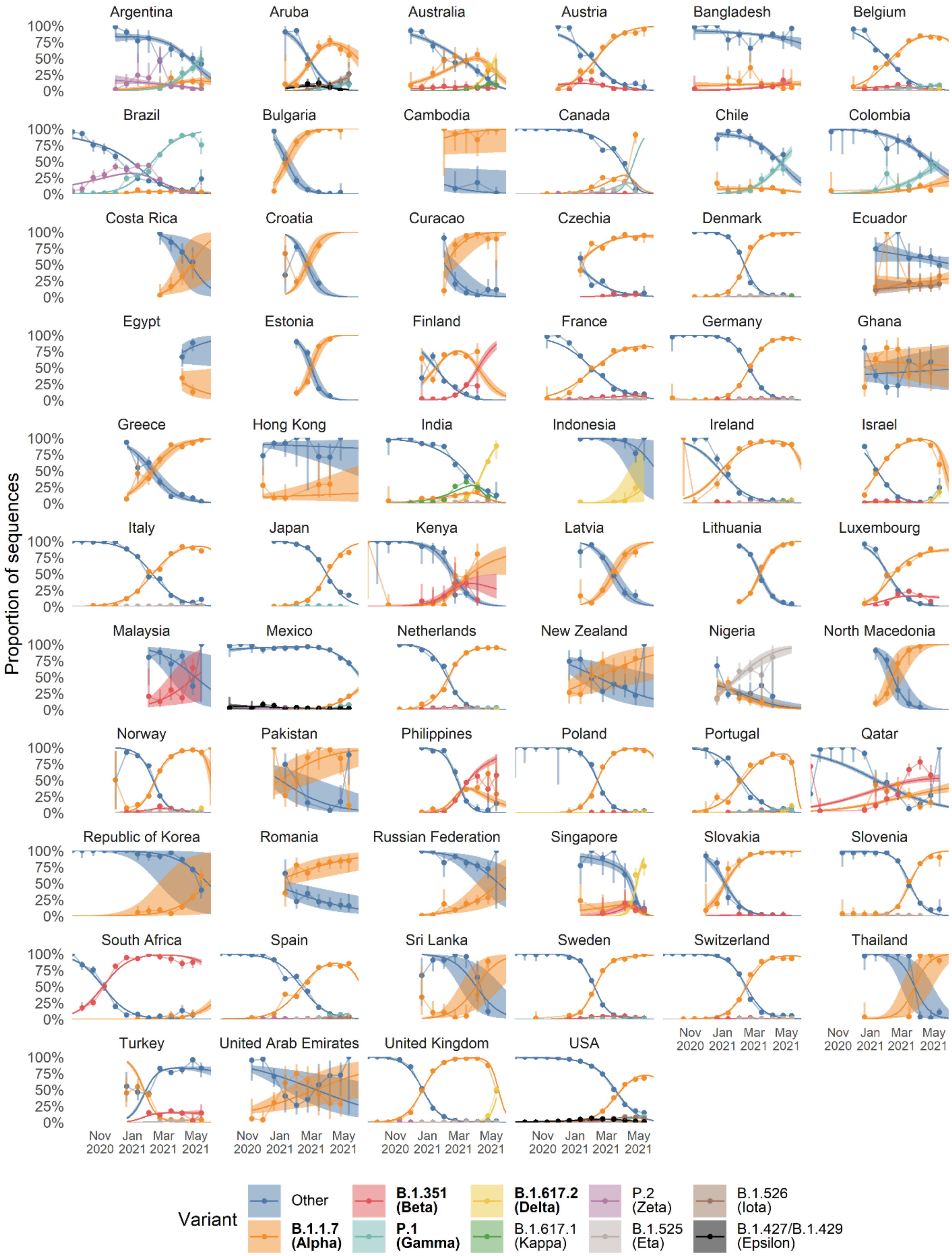

Argentina
Aruba
Australia
Austria
Bangladesh
Belgium
Brazil
Bulgaria
Cambodia
Canada
Chile
Colombia
Costa Rica
Croatia
Curacao
Czechia
Denmark
Ecuador
Egypt
Estonia
Finland
France
Germany
Ghana
Greece
Hong Kong
India
Indonesia
Ireland
Israel
Italy
Japan
Kenya
Latvia
Lithuania
Luxembourg
Malaysia
Mexico
Netherlands
New Zealand
Nigeria
North Macedonia
Norway
Pakistan
Philippines
Poland
Portugal
Qatar
Republic of Korea
Romania
Russian Federation
Singapore
Slovakia
Slovenia
South Africa
Spain
Sri Lanka
Sweden
Switzerland
Thailand
Turkey
United Arab Emirates
United Kingdom
USA
Proportion of sequences
100%
75%
50%
25%
0%
Nov 2020
Jan 2021
Mar 2021
May 2021
Variant
Other
B.1.1.7 (Alpha)
B.1.351 (Beta)
P.1 (Gamma)
B.1.617.2 (Delta)
B.1.617.1 (Kappa)
P.2 (Zeta)
B.1.525 (Eta)
B.1.526 (Iota)
B.1.427/B.1.429 (Epsilon)

Supplementary Figure S2. Empirical and modelled variant proportions over time for countries, territories or areas. Points and lines represent empirical variant proportions, error bars represent multinomial 95% confidence intervals. Smoothed lines represent modelled variant proportions, coloured areas represent 95% confidence intervals. VOCs are indicated in bold text, VOIs in plain text. Countries, territories and areas as reported in GISAID do not imply the expression of any opinion whatsoever on the part of WHO concerning the legal status of any country, territory or area or of its authorities.

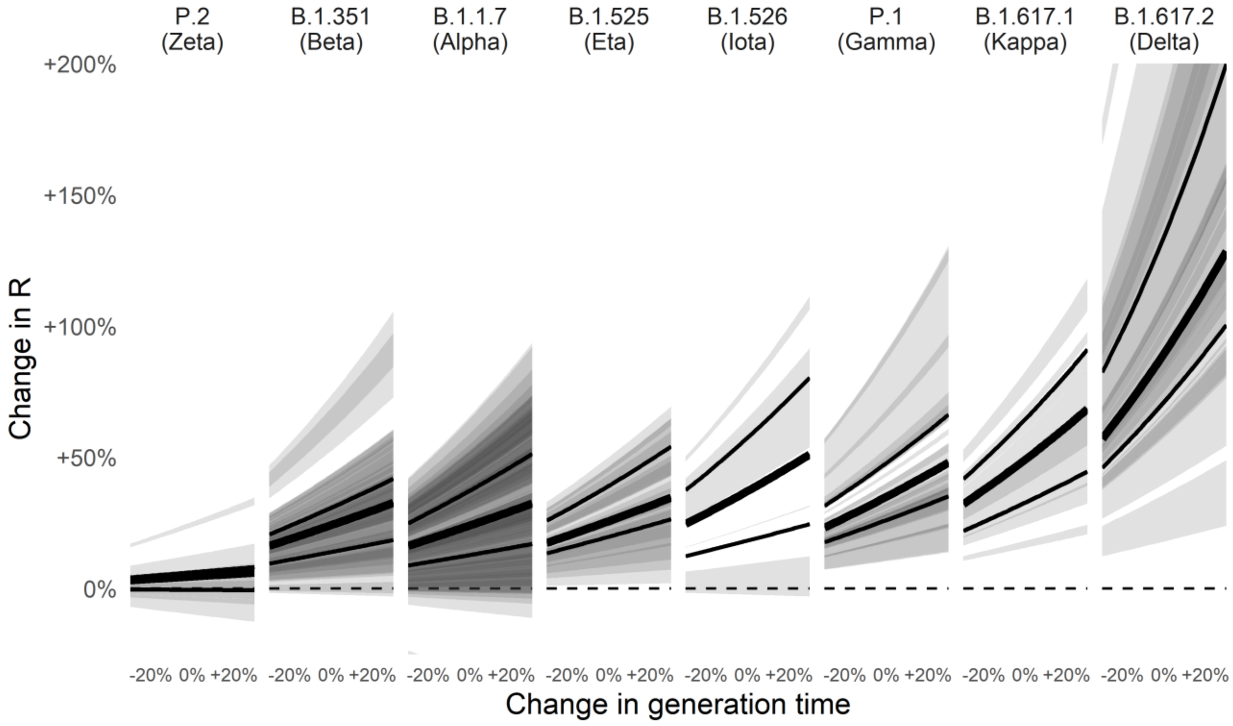

Supplementary Figure S3. Sensitivity of variant transmissibility estimates to changes in the mean generation time. Shaded areas represent 95% confidence intervals of individual country estimates, solid lines represent the median values of the central estimate and 95% confidence intervals.

Supplementary Table 1: VOC sequence numbers by country

B.1.1.7 (Alpha) [n = 764,911]		B.1.351 (Beta) [n = 17,973]	P.1 (Gamma) [n = 22,144]	B.1.617.2 (Delta) [n = 16,020]
United Kingdom (243,804)	Turkey (573)	South Africa (3,695)	USA (11,660)	United Kingdom (11,354)
USA (151,037)	Romania (504)	Germany (1,996)	Brazil (6,337)	India (1,934)
Germany (86,487)	Aruba (480)	Sweden (1,990)	Belgium (913)	USA (1,329)
Denmark (46,635)	Mexico (437)	France (1,754)	Spain (451)	Germany (347)
Sweden (41,027)	Australia (388)	USA (1,718)	Canada (418)	Singapore (144)
France (23,589)	Thailand (382)	Belgium (871)	Netherlands (377)	Belgium (118)
Netherlands (20,308)	Republic of Korea (322)	Philippines (741)	France (340)	Australia (108)
Italy (18,050)	Brazil (308)	Netherlands (651)	Chile (251)	Ireland (99)
Switzerland (17,447)	Curacao (285)	Luxembourg (640)	Italy (203)	Denmark (78)
Belgium (13,100)	Cambodia (223)	United Kingdom (616)	Argentina (181)	France (78)
Spain (12,448)	Pakistan (199)	Turkey (527)	Germany (163)	Italy (72)
Japan (11,077)	Qatar (189)	Qatar (388)	Colombia (160)	Portugal (67)
Poland (11,046)	North Macedonia (179)	Norway (346)	Mexico (153)	Spain (67)
Ireland (10,660)	Ghana (168)	Finland (295)	Portugal (138)	Switzerland (54)
Israel (7,545)	Russian Federation (166)	Austria (251)	United Kingdom (135)	Netherlands (41)
Norway (5,918)	Nigeria (138)	Israel (229)	Switzerland (93)	Israel (36)
Lithuania (5,772)	New Zealand (123)	Spain (188)	Japan (77)	Poland (34)
Slovenia (5,638)	Argentina (95)	Switzerland (178)	Sweden (61)	Indonesia (30)
Portugal (3,950)	United Arab Emirates (88)	India (105)	Aruba (33)	Norway (30)
Luxembourg (2,960)	Sri Lanka (83)	Denmark (98)		
Finland (2,436)	Bangladesh (82)	Canada (93)		
Slovakia (2,355)	Kenya (79)	Portugal (88)		
Greece (2,332)	Chile (78)	Malaysia (74)		
Czechia (2,297)	Ecuador (65)	Ireland (68)		
Austria (2,179)	Colombia (55)	Kenya (68)		
Bulgaria (2,179)	Costa Rica (48)	Czechia (56)		
Canada (1,786)	Egypt (43)	Australia (54)		
Latvia (1,204)	Hong Kong (33)	Bangladesh (39)		
Estonia (1,164)	Singapore (29)	Italy (36)		
India (1,113)	South Africa (28)	Poland (36)		
Croatia (837)		Slovenia (31)		
Philippines (661)		Singapore (27)		

Supplementary Table 2: VOI sequence numbers by country

B.1.617.1 (Kappa) [n = 2,608]
India (1,813)
United Kingdom (367)
USA (233)
Germany (74)
Ireland (58)
Australia (36)
Denmark (27)

P.2 (Zeta) [n = 3,072]
Brazil (1,496)
USA (1,221)
France (112)
Argentina (66)
United Kingdom (52)
Spain (37)
Canada (33)
Netherlands (28)
Mexico (27)

B.1.525 (Eta) [n = 4,877]
USA (1,019)
Canada (726)
Denmark (611)
Germany (585)
France (457)
United Kingdom (428)
Italy (256)
Nigeria (204)
Spain (129)
India (80)
Norway (80)
Ireland (55)
Belgium (54)
Netherlands (50)
Slovenia (50)
Switzerland (50)
Turkey (43)

B.1.427/B.1.429 (Epsilon) [n = 12,737]
USA (12,512)
Mexico (176)
Aruba (49)

B.1.526 (Iota) [n = 19,896]
USA (19,716)
Aruba (71)
Spain (62)
Ecuador (47)
