# Supplementary material for: Increased transmissibility and global spread of SARS-CoV-2 variants of concern as at June 2021
Source: Euro Surveill. 2021 Jun 17;26(24):2100509. doi: 10.2807/1560-7917.ES.2021.26.24.2100509 (PMC8212592; doi:10.2807/1560-7917.ES.2021.26.24.2100509)
Supplement: Supplement2 [file 21-00509_CAMPBELL_supplement2.pdf]

We gratefully acknowledge the following Authors from the Originating laboratories responsible for obtaining the specimens, as well as the Submitting laboratories where the genome data were generated and shared via GISAID, on which this research is based.

All Submitters of data may be contacted directly via www.gisaid.org

Authors are sorted alphabetically.

Accession ID	Originating Laboratory	Submitting Laboratory	Authors
EPI_ISL_1446651 to 1446666, EPI_ISL_1446671, EPI_ISL_1528479 to 1528492	"AK State Public Health Lab, State Health Department"	Centers for Disease Control and Prevention Division of Viral Diseases, Pathogen Discovery	Alison Laufer Halpin; Ben L. Rambo-Martin; Clinton R. Paden; Dakota Howard; Darlene Wagner; Dave Wentworth; Dhwani Batra; Jasmine Padilla; Justin Lee; Katie Dillon; Krista Queen; Kristen Knipe; Kristine Lacek; Mark Burroughs; Matthew Schmerer; Mili Sheth; Peter Cook; Sam Shepard; Sarah Nobles; Shoshona Le; Suxiang Tong; Vivien Dugan; Yvette Unoarumhi
EPI_ISL_1446456, EPI_ISL_1446459, EPI_ISL_1446462, EPI_ISL_1446465, EPI_ISL_1446468, EPI_ISL_1446471, EPI_ISL_1446473, EPI_ISL_1528291 to 1528293			
see above	"AR Dept. of Health-PHL, Molecular Diagnostics"	Centers for Disease Control and Prevention Division of Viral Diseases, Pathogen Discovery	Alison Laufer Halpin; Ben L. Rambo-Martin; Clinton R. Paden; Dakota Howard; Darlene Wagner; Dave Wentworth; Dhwani Batra; Jasmine Padilla; Justin Lee; Katie Dillon; Krista Queen; Kristen Knipe; Kristine Lacek; Mark Burroughs; Matthew Schmerer; Mili Sheth; Peter Cook; Sam Shepard; Sarah Nobles; Shoshona Le; Suxiang Tong; Vivien Dugan; Yvette Unoarumhi
EPI_ISL_1509928 to 1509929, EPI_ISL_1509937	"AR Dept. of Health-PHL, Molecular Diagnostics"	Genomics and Discovery, Respiratory Viruses Branch, Division of Viral Diseases, Centers for Disease Control and Prevention	Adam Retchless; Anna Kelleher; Anna Montmayeur; Anna Uehara; Brian Lynch; Clinton R. Paden; Haibin Wang; Han Jia Justin Ng; Jing Zhang; Justin Lee; Krista Queen; Mark Burroughs; Peter Cook; Rachel Marine; Suxiang Tong; Yan Li; Ying Tao
EPI_ISL_1528419	"AZ SPHL, Arizona Department of Health Services"	Centers for Disease Control and Prevention Division of Viral Diseases, Pathogen Discovery	Alison Laufer Halpin; Ben L. Rambo-Martin; Clinton R. Paden; Dakota Howard; Darlene Wagner; Dave Wentworth; Dhwani Batra; Jasmine Padilla; Justin Lee; Katie Dillon; Krista Queen; Kristen Knipe; Kristine Lacek; Mark Burroughs; Matthew Schmerer; Mili Sheth; Peter Cook; Sam Shepard; Sarah Nobles; Shoshona Le; Suxiang Tong; Vivien Dugan; Yvette Unoarumhi
EPI_ISL_1446773 to 1446786, EPI_ISL_1446788 to 1446789, EPI_ISL_1446791 to 1446802, EPI_ISL_1446840 to 1446845, EPI_ISL_1447018	"CDPH, Viral and Rickettsial Disease Laboratory"	Centers for Disease Control and Prevention Division of Viral Diseases, Pathogen Discovery	Alison Laufer Halpin; Ben L. Rambo-Martin; Clinton R. Paden; Dakota Howard; Darlene Wagner; Dave Wentworth; Dhwani Batra; Jasmine Padilla; Justin Lee; Katie Dillon; Krista Queen; Kristen Knipe; Kristine Lacek; Mark Burroughs; Matthew Schmerer; Mili Sheth; Peter Cook; Sam Shepard; Sarah Nobles; Shoshona Le; Suxiang Tong; Vivien Dugan; Yvette Unoarumhi
EPI_ISL_1491516 to 1491517, EPI_ISL_1491522	"CDPH, Viral and Rickettsial Disease Laboratory"	Genomics and Discovery, Respiratory Viruses Branch, Division of Viral Diseases, Centers for Disease Control and Prevention	Anna Kelleher; Anna Uehara; Brian Lynch; Clinton R. Paden; Haibin Wang; Han Jia Justin Ng; Jing Zhang; Krista Queen; Peter Cook; Suxiang Tong; Yan Li; Ying Tao
EPI_ISL_1528339 to 1528362	"CO Dept. of Public Health and Environment, Lab Services Division"	Centers for Disease Control and Prevention Division of Viral Diseases, Pathogen Discovery	Alison Laufer Halpin; Ben L. Rambo-Martin; Clinton R. Paden; Dakota Howard; Darlene Wagner; Dave Wentworth; Dhwani Batra; Jasmine Padilla; Justin Lee; Katie Dillon; Krista Queen; Kristen Knipe; Kristine Lacek; Mark Burroughs; Matthew Schmerer; Mili Sheth; Peter Cook; Sam Shepard; Sarah Nobles; Shoshona Le; Suxiang Tong; Vivien Dugan; Yvette Unoarumhi
EPI_ISL_1394769 to 1394770, EPI_ISL_1394772 to 1394773, EPI_ISL_1394776 to 1394777, EPI_ISL_1394780, EPI_ISL_1394782, EPI_ISL_1394784, EPI_ISL_1394786 to 1394787, EPI_ISL_1394792, EPI_ISL_1394795 to 1394799, EPI_ISL_1394804, EPI_ISL_1394808, EPI_ISL_1394811, EPI_ISL_1394835			
see above	"InMedica"	Lithuanian University of Health Sciences Hospital, Department of Genetics and Molecular Medicine	Astra Vitkauskiene; Darius Cereskevicius; Inga Nasvytiene; Mantas Sarauskas; Marius Sukys; Rasa Ugenskiene; Renaldas Jurkevicius; Rima Vainoriene; Zivile Zemeckiene
EPI_ISL_1446640, EPI_ISL_1446721 to 1446722, EPI_ISL_1446869 to 1446871, EPI_ISL_1446873, EPI_ISL_1446875, EPI_ISL_1446879, EPI_ISL_1446881, EPI_ISL_1446883, EPI_ISL_1446889 to 1446899, EPI_ISL_1446902, EPI_ISL_1446906, EPI_ISL_1446909, EPI_ISL_1446986, EPI_ISL_1528272 to 1528274			
see above	"MN PHL Division, Minnesota Department of Health"	Centers for Disease Control and Prevention Division of Viral Diseases, Pathogen Discovery	Alison Laufer Halpin; Ben L. Rambo-Martin; Clinton R. Paden; Dakota Howard; Darlene Wagner; Dave Wentworth; Dhwani Batra; Jasmine Padilla; Justin Lee; Katie Dillon; Krista Queen; Kristen Knipe; Kristine Lacek; Mark Burroughs; Matthew Schmerer; Mili Sheth; Peter Cook; Sam Shepard; Sarah Nobles; Shoshona Le; Suxiang Tong; Vivien Dugan; Yvette Unoarumhi
EPI_ISL_1446386, EPI_ISL_1446389, EPI_ISL_1446392, EPI_ISL_1446395, EPI_ISL_1446398, EPI_ISL_1446401, EPI_ISL_1446404, EPI_ISL_1446406, EPI_ISL_1446409, EPI_ISL_1446632 to 1446633, EPI_ISL_1446635 to 1446639, EPI_ISL_1446868, EPI_ISL_1446874, EPI_ISL_1446876, EPI_ISL_1446878, EPI_ISL_1446884 to 1446886, EPI_ISL_1446888, EPI_ISL_1446901, EPI_ISL_1446903, EPI_ISL_1446907 to 1446908, EPI_ISL_1446912, EPI_ISL_1528378 to 1528385			
see above	"NM Dept. Health, Scientific Laboratory Division "	Centers for Disease Control and Prevention Division of Viral Diseases, Pathogen Discovery	Alison Laufer Halpin; Ben L. Rambo-Martin; Clinton R. Paden; Dakota Howard; Darlene Wagner; Dave Wentworth; Dhwani Batra; Jasmine Padilla; Justin Lee; Katie Dillon; Krista Queen; Kristen Knipe; Kristine Lacek; Mark Burroughs; Matthew Schmerer; Mili Sheth; Peter Cook; Sam Shepard; Sarah Nobles; Shoshona Le; Suxiang Tong; Vivien Dugan; Yvette Unoarumhi
EPI_ISL_1446676 to 1446677, EPI_ISL_1447053	"NYSDOH Wadsworth Center, Virology Lab"	Centers for Disease Control and Prevention Division of Viral Diseases, Pathogen Discovery	Alison Laufer Halpin; Ben L. Rambo-Martin; Clinton R. Paden; Dakota Howard; Darlene Wagner; Dave Wentworth; Dhwani Batra; Jasmine Padilla; Justin Lee; Katie Dillon; Krista Queen; Kristen Knipe; Kristine Lacek; Mark Burroughs; Matthew Schmerer; Mili Sheth; Peter Cook; Sam Shepard; Sarah Nobles; Shoshona Le; Suxiang Tong; Vivien Dugan; Yvette Unoarumhi
EPI_ISL_1446543 to 1446546	"OK Public Health Laboratory, Oklahoma State DOH"	Centers for Disease Control and Prevention Division of Viral Diseases, Pathogen Discovery	Alison Laufer Halpin; Ben L. Rambo-Martin; Clinton R. Paden; Dakota Howard; Darlene Wagner; Dave Wentworth; Dhwani Batra; Jasmine Padilla; Justin Lee; Katie Dillon; Krista Queen; Kristen Knipe; Kristine Lacek; Mark Burroughs; Matthew Schmerer; Mili Sheth; Peter Cook; Sam Shepard; Sarah Nobles; Shoshona Le; Suxiang Tong; Vivien Dugan; Yvette Unoarumhi
EPI_ISL_1509943	"OK Public Health Laboratory, Oklahoma State DOH"	Genomics and Discovery, Respiratory Viruses Branch, Division of Viral Diseases, Centers for Disease Control and Prevention	Adam Retchless; Anna Kelleher; Anna Montmayeur; Anna Uehara; Brian Lynch; Clinton R. Paden; Haibin Wang; Han Jia Justin Ng; Jing Zhang; Justin Lee; Krista Queen; Mark Burroughs; Peter Cook; Rachel Marine; Suxiang Tong; Yan Li; Ying Tao
EPI_ISL_1446701, EPI_ISL_1446807 to 1446808, EPI_ISL_1447019 to 1447026, EPI_ISL_1528506 to 1528507	"PA Department of Health, Bureau of Laboratories"	Centers for Disease Control and Prevention Division of Viral Diseases, Pathogen Discovery	Alison Laufer Halpin; Ben L. Rambo-Martin; Clinton R. Paden; Dakota Howard; Darlene Wagner; Dave Wentworth; Dhwani Batra; Jasmine Padilla; Justin Lee; Katie Dillon; Krista Queen; Kristen Knipe; Kristine Lacek; Mark Burroughs; Matthew Schmerer; Mili Sheth; Peter Cook; Sam Shepard; Sarah Nobles; Shoshona Le; Suxiang Tong; Vivien Dugan; Yvette Unoarumhi
EPI_ISL_1408890, EPI_ISL_1448525, EPI_ISL_1478203	"PA Department of Health, Bureau of Laboratories"	Genomics and Discovery, Respiratory Viruses Branch, Division of Viral Diseases, Centers for Disease Control and Prevention	Anna Kelleher; Anna Uehara; Brian Lynch; Clinton R. Paden; Haibin Wang; Han Jia Justin Ng; Jing Zhang; Krista Queen; Peter Cook; Suxiang Tong; Yan Li; Ying Tao
EPI_ISL_1455671, EPI_ISL_1457126	"Stefan S. Nicolau"Institute of Virology	"Stefan S. Nicolau"Institute of Virology	Adriana Plesa; Alina Nastasie; Ana Iulia Neagu; Anca Botezatu; Camelia Sultana; Carmen Cristina Diaconu; Coralia Bleotu; Cristina Mambet; Denisa Dragu; Gabriela Anton; Ioana Pitica; Iulia Virginia Iancu; Laura Grecu; Laura Necula; Lilia Matei; Marinela Bostan; Mihaela Economescu; Mirela Mihaila; Saviana Nedeianu; Simona Ruta
EPI_ISL_1446641 to 1446650, EPI_ISL_1446738, EPI_ISL_1528397 to 1528398	"TX DSHS, Lab Services Section MC 1947"	Centers for Disease Control and Prevention Division of Viral Diseases, Pathogen Discovery	Alison Laufer Halpin; Ben L. Rambo-Martin; Clinton R. Paden; Dakota Howard; Darlene Wagner; Dave Wentworth; Dhwani Batra; Jasmine Padilla; Justin Lee; Katie Dillon; Krista Queen; Kristen Knipe; Kristine Lacek; Mark Burroughs; Matthew Schmerer; Mili Sheth; Peter Cook; Sam Shepard; Sarah Nobles; Shoshona Le; Suxiang Tong; Vivien Dugan; Yvette Unoarumhi
EPI_ISL_1524996 to 1524998	#VALUE!	Labo Klinische Biologie, UZA	Basil Britto Xavier; Christine Lammens; Herman Goossens; Jasmine Coppens; Marie Le Mercier; Veerle Matheeussen
EPI_ISL_1448509	1) Laboratorio Cerba 2) Università Milano-Bicocca 3) TaskForce Covid19-Ceinge Regione Campania	TaskForce Covid19-Ceinge Regione Campania	2) Ettore Capoluongo (3) Alessio Colombo (2) Adelaide Bussini (1) Nicola Perrelli (2) Silvia Cavalli (2) Alice Urbani (2) Giovanni Paolella (3) Veronica Ferrucci (3) Rossella Tufano (3) Angelo Boccia (3) Massimo Zollo (3) 1) Università Milano-Bicocca 2) Laboratorio Cerba 3) TaskForce Covid19-Ceinge Regione Campania; Francesco Broccolo (1
EPI_ISL_1410538, EPI_ISL_1410540, EPI_ISL_1410543, EPI_ISL_1410545, EPI_ISL_1410548, EPI_ISL_1410551, EPI_ISL_1410553, EPI_ISL_1410556, EPI_ISL_1410558, EPI_ISL_1410561, EPI_ISL_1410564, EPI_ISL_1410566, EPI_ISL_1410569, EPI_ISL_1410571, EPI_ISL_1410574, EPI_ISL_1410577, EPI_ISL_1410579, EPI_ISL_1410582, EPI_ISL_1410585, EPI_ISL_1410587, EPI_ISL_1410590, EPI_ISL_1410592, EPI_ISL_1410595, EPI_ISL_1410598, EPI_ISL_1410600, EPI_ISL_1410603, EPI_ISL_1410605, EPI_ISL_1410608, EPI_ISL_1410611, EPI_ISL_1410613, EPI_ISL_1410616, EPI_ISL_1410618, EPI_ISL_1410621, EPI_ISL_1410624, EPI_ISL_1410626, EPI_ISL_1410629, EPI_ISL_1410631, EPI_ISL_1410634, EPI_ISL_1410637, EPI_ISL_1410640, EPI_ISL_1410643, EPI_ISL_1410645, EPI_ISL_1410648, EPI_ISL_1410650, EPI_ISL_1410653, EPI_ISL_1410655, EPI_ISL_1410658, EPI_ISL_1410660, EPI_ISL_1410663, EPI_ISL_1410666, EPI_ISL_1410669, EPI_ISL_1410671, EPI_ISL_1410674, EPI_ISL_1410677, EPI_ISL_1410679, EPI_ISL_1410682, EPI_ISL_1410685, EPI_ISL_1410687, EPI_ISL_1410690, EPI_ISL_1410693, EPI_ISL_1410696, EPI_ISL_1410699, EPI_ISL_1410701, EPI_ISL_1410705, EPI_ISL_1410707, EPI_ISL_1410709, EPI_ISL_1410712, EPI_ISL_1410715, EPI_ISL_1410717, EPI_ISL_1410720, EPI_ISL_1410723, EPI_ISL_1410725, EPI_ISL_1410728, EPI_ISL_1410731, EPI_ISL_1410733, EPI_ISL_1410736, EPI_ISL_1410738, EPI_ISL_1410741, EPI_ISL_1410743, EPI_ISL_1410746, EPI_ISL_1410749, EPI_ISL_1410751, EPI_ISL_1410753, EPI_ISL_1410756, EPI_ISL_1410758,			

EPI_ISL_1410761, EPI_ISL_1410764, EPI_ISL_1410767, EPI_ISL_1410769, EPI_ISL_1410772, EPI_ISL_1410774, EPI_ISL_1410776, EPI_ISL_1410779, EPI_ISL_1410782, EPI_ISL_1410785, EPI_ISL_1411509 to 1411554, EPI_ISL_1411557, EPI_ISL_1411559, EPI_ISL_1411562, EPI_ISL_1411564, EPI_ISL_1411566, EPI_ISL_1411568, EPI_ISL_1411571, EPI_ISL_1411574, EPI_ISL_1411576, EPI_ISL_1411578, EPI_ISL_1411580, EPI_ISL_1411583, EPI_ISL_1411585, EPI_ISL_1411588, EPI_ISL_1411590, EPI_ISL_1411593, EPI_ISL_1411595, EPI_ISL_1411598, EPI_ISL_1411601, EPI_ISL_1411604, EPI_ISL_1411606, EPI_ISL_1411609, EPI_ISL_1411611, EPI_ISL_1411614, EPI_ISL_1411616, EPI_ISL_1411619, EPI_ISL_1411622, EPI_ISL_1411624, EPI_ISL_1411626, EPI_ISL_1411629, EPI_ISL_1411631, EPI_ISL_1411633, EPI_ISL_1411636			
see above	1. Glowny Inspektorat Sanitarny. 2. Diagnostyka. Laboratoria Medyczne.	1. ViroGenetics - BSL3 Laboratory of Virology, Malopolska Centre of Biotechnology, Jagiellonian University; 2. genXone SA, Research & Development Laboratory	Aleksandra Gidlewicz; Anna Brylak; Gromowski, T.; Grzegorz Nowicki; Jakub Grabowski; Karol Szeszko; Kowalski, M.; Krzysztof Pyrc; Labaj; Lukasz Krych; Maciej Sykulski; Mazur-Panasiuk, N.; Michal Kaszuba; Michal Kowalski; Natalia Drweska-Matelska; Natalia Mazur-Panasiuk; P.P.; Pawel P Labaj; Piotr Szulc; Pyrc, K.; Sylwia Januszczak; Szulc, P.; Tomasz Gromowski
EPI_ISL_1396558 to 1396633	1. Glowny Inspektorat Sanitarny. 2. Diagnostyka. Laboratoria Medyczne.	1. ViroGenetics - BSL3 Laboratory of Virology, Maopolska Centre of Biotechnology, Jagiellonian University; 2. Diagtron Laboratoria Lukasz Rabalski	Andrzej Horban; Krzysztof Pyrc; Lukasz Rabalski; Maciej Kosinski; Natalia Derewonko; Natalia Mazur-Panasiuk; Piotr Zabek; Sylwia Januszczak; Tomasz Dyda; Tomasz Gromowski
EPI_ISL_1402655 to 1402706, EPI_ISL_1406315 to 1406386, EPI_ISL_1502583 to 1502730, EPI_ISL_1502732 to 1502794, EPI_ISL_1524757 to 1524770	1. Glowny Inspektorat Sanitarny. 2. Diagnostyka. Laboratoria Medyczne.	1. ViroGenetics - BSL3 Laboratory of Virology, Maopolska Centre of Biotechnology, Jagiellonian University; 2. genXone SA, Research & Development Laboratory	Aleksandra Gidlewicz; Anna Brylak; Gromowski, T.; Grzegorz Nowicki; Jakub Grabowski; Karol Szeszko; Kowalski, M.; Labaj; Maciej Sykulski; Mazur-Panasiuk, N.; Micha Kaszuba; Natalia Drweska-Matelska; P.P.; Pyrc, K.; Sylwia Januszczak; Szulc, P.; ukasz Krych
EPI_ISL_1402473 to 1402539, EPI_ISL_1497679 to 1497765, EPI_ISL_1499116 to 1499200, EPI_ISL_1499206 to 1499296, EPI_ISL_1499301 to 1499363, EPI_ISL_1500087 to 1500150	1. Glowny Inspektorat Sanitarny. 2. Diagnostyka. Laboratoria Medyczne.	1. ViroGenetics - BSL3 Laboratory of Virology, Maopolska Centre of Biotechnology, Jagiellonian University; 2. genXone SA, Research & Development Laboratory	Aleksandra Gidlewicz; Anna Brylak; Gromowski, T.; Grzegorz Nowicki; Jakub Grabowski; Karol Szeszko; Kowalski, M.; Labaj; Maciej Sykulski; Mazur-Panasiuk, N.; Micha Kaszuba; Natalia Drweska-Matelska; P.P.; Pyrc, K.; Sylwia Januszczak; Szulc, P.; ukasz Krych
EPI_ISL_451971 to 451987	1. ViroGenetics - BSL3 Laboratory of Virology, Maopolska Centre of Biotechnology, Jagiellonian University; 2. II Department of Internal Medicine, Faculty of Medicine, Jagiellonian University Medical College; 3. DIAGNOSTYKA Ltd.	1. ViroGenetics - BSL3 Laboratory of Virology, Maopolska Centre of Biotechnology, Jagiellonian University; 2. II Department of Internal Medicine, Faculty of Medicine, Jagiellonian University Medical College.	Jakub Swadba; Krzysztof Pyr; Marcin Surmiak; Marek Sanak; Marta Rogalska-Kupiec; Monika Gsecka-Czapla; Pawe P abaj; Wojciech Branicki
EPI_ISL_455440 to 455453, EPI_ISL_492066 to 492073	1. ViroGenetics - BSL3 Laboratory of Virology, Maopolska Centre of Biotechnology, Jagiellonian University; 2. II Department of Internal Medicine, Faculty of Medicine, Jagiellonian University Medical College; 3. Narodowy Instytut Zdrowia Publicznego - Pastwowy Zakad Higieny (NIZP-PZH)	1. ViroGenetics - BSL3 Laboratory of Virology, Maopolska Centre of Biotechnology, Jagiellonian University; 2. II Department of Internal Medicine, Faculty of Medicine, Jagiellonian University Medical College; 3. Narodowy Instytut Zdrowia Publicznego - Pastwowy Zakad Higieny (NIZP-PZH).	Agnieszka Koakowska-Kulesza; Aleksandra A. Zasada; Aleksandra Milewska; Ewelina Hallman-Szeliska; Katarzyna Owczarek; Katarzyna Pancer; Katarzyna Zacharczuk; Krzysztof Pyr; Magdalena Rzeczkowska; Marek Sanak; Natalia Wolaniuk; Pawe P abaj; Tomasz Wokowicz; Wojciech Branicki
EPI_ISL_1547375, EPI_ISL_1547388 to 1547392	1.AO Universitaria 'S. Giovanni di Dio e Ruggi D'Aragona, Scuola Medica Salernitana' Hospital / 2.UOC di Virologia e Microbiologia, Università della Campania 'L. Vanvitelli' / 3.AO Universitaria 'Federico II' Napoli Hospital / 4.AORN 'San Giuseppe Moscati' Avellino Hospital / 5.AO 'San Pio - presidio G. Rummo' Benevento Hospital / 6.AO 'Sant'Anna e San Sebastiano' Caserta Hospital / 7.PO 'Maria Santissima Addolorata' Eboli Hospital / 8.Biogem Istituto di Ricerche Genetiche / 9. U.O.C. di Genetica Medica e di Laboratorio A.O.R.N., Azienda Ospedaliera di Rilievo Nazionale Antonio Cardarelli, Napoli / 10. Centro di riferimento Oncologico della Basilicata (IRCCS-CROB), Rionero in Vulture (PZ) / 11. Presidio Ospedaliero di Agropoli, Agropoli (SA).	1. Genome Research Center for Health (CRGS) / 2. Laboratory of Molecular Medicine and Genomics(LMMGe) / 3. Center for Research in Pure and Applied Mathematics (CRMPA)	Alessandro Weisz; Alessandro Weisz (Corresponding Author); Alessia Cossu; Andreina Baj; Aniello Gentile; Annamaria Salvati; Antonello Saccomanno; Arnolfo Petruzziello; Assunta Sellitto; Caivano Antonella; Carlo Ferravante; D'Auria Fiorella; De Luca Luciana.; Domenico Memoli; Domenico Palumbo; Edmondo Adorisio; Elena Alexandrova; Emilia Vaccaro; Fau; Fausto Sessa; Francesca Marciano; Francesca Rizzo; Francesca Rizzo (Corresponding Author); Francesco Curcio; Gianluigi Franci; Giorgio Dirani; Giorgio Giurato; Giorgio Giurato (Corresponding Author); Giovanni Nassa; Giovanni Pecoraro; Giuseppe Fenza; Giuseppe Portella; Giusy Giordano; Gregorio Goffredi; Ilaria Terenzi; Jessica Lamberti; Maddalena Schioppa; Marcello Ametrano; Maria Grazia Foti; Maria Landi; Marianna Scrima; Mariano Fiorenza; Mariarosaria Ingino; Marina Tarsitano; Massimiliano Chetta; Massimiliano Galdiero; Maurizio Fumi; Michela Iacobellis; Michele Caraglia; Michele Cennamo; Morena D'Avenia; Oriana Strianese; Pasquale Pagliano; Rita Greco; Roberta Tarallo; Rosanna Piluscio; Silvia Zanoli; Simona Semprini; Sonia Amabile; Stefania Marzinotto; Teresa Rocco; Valeria Mirici Cappa; Vincenzo Rocco; Viola Melone; Vittoria Letizia; Vittorio Sambri; Ylenia D'Agostino
EPI_ISL_1534286, EPI_ISL_1534290, EPI_ISL_1534296, EPI_ISL_1534327 to 1534330, EPI_ISL_1534374, EPI_ISL_1534401, EPI_ISL_1534418, EPI_ISL_1534429			
see above	2 Military Hospital wc MAA	NHLS/UCT	Arash Iranzadeh; Bruna Galvao; Carolyn Williamson; Deelan Doolabh; Diana Hardie; Emmanuel SJ; Innocent Mudau; Kruger Marais; Lynn Tyers; Marvin Hsiao; Stephen Korsman; Tegally H; de Oliveira T
EPI_ISL_450444	20 Dongda Street, Fengtai District, Beijing, Beijing 100071, China	Dept. OPA, Beijing Institute of Microbiology and Epidemiology	Fan, H.; Fang; Gong; J.H.; L.Q. and Liu, W.; Qi; R.Z.; X.A.; Zhang; Zheng, K.; Zheng, W.
EPI_ISL_1447961	20210213546	TIGEM	Antonio Grimaldi Patrizia Annunziata Francesco Panariello Biancamaria Pierri Claudia Tiberio Valentina Bouche Chiara Colantuono Maria Concetta Cuomo Denise Di Concilio Lucio Di Filippo Anna Manfredi Marcello Salvi Antonio Limone Luigi Atripaldi Pellegrino Cerino Andrea Ballabio Davide Cacchiarelli
EPI_ISL_1447950	20210213554	TIGEM	Antonio Grimaldi Patrizia Annunziata Francesco Panariello Biancamaria Pierri Claudia Tiberio Valentina Bouche Chiara Colantuono Maria Concetta Cuomo Denise Di Concilio Lucio Di Filippo Anna Manfredi Marcello Salvi Antonio Limone Luigi Atripaldi Pellegrino Cerino Andrea Ballabio Davide Cacchiarelli
EPI_ISL_1447951	20210213565	TIGEM	Antonio Grimaldi Patrizia Annunziata Francesco Panariello Biancamaria Pierri Claudia Tiberio Valentina Bouche Chiara Colantuono Maria Concetta Cuomo Denise Di Concilio Lucio Di Filippo Anna Manfredi Marcello Salvi Antonio Limone Luigi Atripaldi Pellegrino Cerino Andrea Ballabio Davide Cacchiarelli
EPI_ISL_1447958	20210213566	TIGEM	Antonio Grimaldi Patrizia Annunziata Francesco Panariello Biancamaria Pierri Claudia Tiberio Valentina Bouche Chiara Colantuono Maria Concetta Cuomo Denise Di Concilio Lucio Di Filippo Anna Manfredi Marcello Salvi Antonio Limone Luigi Atripaldi Pellegrino Cerino Andrea Ballabio Davide Cacchiarelli
EPI_ISL_1447957	20210213570	TIGEM	Antonio Grimaldi Patrizia Annunziata Francesco Panariello Biancamaria Pierri Claudia Tiberio Valentina Bouche Chiara Colantuono Maria Concetta Cuomo Denise Di Concilio Lucio Di Filippo Anna Manfredi Marcello Salvi Antonio Limone Luigi Atripaldi Pellegrino Cerino Andrea Ballabio Davide Cacchiarelli
EPI_ISL_1447953	20210213607	TIGEM	Antonio Grimaldi Patrizia Annunziata Francesco Panariello Biancamaria Pierri Claudia Tiberio Valentina Bouche Chiara Colantuono Maria Concetta Cuomo Denise Di Concilio Lucio Di Filippo Anna Manfredi Marcello Salvi Antonio Limone Luigi Atripaldi Pellegrino Cerino Andrea Ballabio Davide Cacchiarelli
EPI_ISL_1447952	20210217579	TIGEM	Antonio Grimaldi Patrizia Annunziata Francesco Panariello Biancamaria Pierri Claudia Tiberio Valentina Bouche Chiara Colantuono Maria Concetta Cuomo Denise Di Concilio Lucio Di Filippo Anna Manfredi Marcello Salvi Antonio Limone Luigi Atripaldi Pellegrino Cerino Andrea Ballabio Davide Cacchiarelli
EPI_ISL_1447943	20210218154	TIGEM	Antonio Grimaldi Patrizia Annunziata Francesco Panariello Biancamaria Pierri Claudia Tiberio Valentina Bouche Chiara Colantuono Maria Concetta Cuomo Denise Di Concilio Lucio Di Filippo Anna Manfredi Marcello Salvi Antonio Limone Luigi Atripaldi Pellegrino Cerino Andrea Ballabio Davide Cacchiarelli
EPI_ISL_1447948	20210218178	TIGEM	Antonio Grimaldi Patrizia Annunziata Francesco Panariello Biancamaria Pierri Claudia Tiberio Valentina Bouche Chiara Colantuono Maria Concetta Cuomo Denise Di Concilio Lucio Di Filippo Anna Manfredi Marcello Salvi Antonio Limone Luigi Atripaldi Pellegrino Cerino Andrea Ballabio Davide Cacchiarelli
EPI_ISL_1447970	20210218285	TIGEM	Antonio Grimaldi Patrizia Annunziata Francesco Panariello Biancamaria Pierri Claudia Tiberio Valentina Bouche Chiara Colantuono Maria Concetta Cuomo Denise Di Concilio Lucio Di Filippo Anna Manfredi Marcello Salvi Antonio Limone Luigi Atripaldi Pellegrino Cerino Andrea Ballabio Davide Cacchiarelli
EPI_ISL_1447960	20210218311	TIGEM	Antonio Grimaldi Patrizia Annunziata Francesco Panariello Biancamaria Pierri Claudia Tiberio Valentina Bouche Chiara Colantuono Maria Concetta Cuomo Denise Di Concilio Lucio Di Filippo Anna Manfredi Marcello Salvi Antonio Limone Luigi Atripaldi Pellegrino Cerino Andrea Ballabio Davide

			Cacchiarelli
EPI_ISL_1447956	20210218316	TIGEM	Antonio Grimaldi Patrizia Annunziata Francesco Panariello Biancamaria Pierri Claudia Tiberio Valentina Bouche Chiara Colantuono Maria Concetta Cuomo Denise Di Concilio Lucio Di Filippo Anna Manfredi Marcello Salvi Antonio Limone Luigi Atripaldi Pellegrino Cerino Andrea Ballabio Davide Cacchiarelli
EPI_ISL_1447959	20210218322	TIGEM	Antonio Grimaldi Patrizia Annunziata Francesco Panariello Biancamaria Pierri Claudia Tiberio Valentina Bouche Chiara Colantuono Maria Concetta Cuomo Denise Di Concilio Lucio Di Filippo Anna Manfredi Marcello Salvi Antonio Limone Luigi Atripaldi Pellegrino Cerino Andrea Ballabio Davide Cacchiarelli
EPI_ISL_1447971	20210218329	TIGEM	Antonio Grimaldi Patrizia Annunziata Francesco Panariello Biancamaria Pierri Claudia Tiberio Valentina Bouche Chiara Colantuono Maria Concetta Cuomo Denise Di Concilio Lucio Di Filippo Anna Manfredi Marcello Salvi Antonio Limone Luigi Atripaldi Pellegrino Cerino Andrea Ballabio Davide Cacchiarelli
EPI_ISL_1447954	20210218337	TIGEM	Antonio Grimaldi Patrizia Annunziata Francesco Panariello Biancamaria Pierri Claudia Tiberio Valentina Bouche Chiara Colantuono Maria Concetta Cuomo Denise Di Concilio Lucio Di Filippo Anna Manfredi Marcello Salvi Antonio Limone Luigi Atripaldi Pellegrino Cerino Andrea Ballabio Davide Cacchiarelli
EPI_ISL_1447955	20210218343	TIGEM	Antonio Grimaldi Patrizia Annunziata Francesco Panariello Biancamaria Pierri Claudia Tiberio Valentina Bouche Chiara Colantuono Maria Concetta Cuomo Denise Di Concilio Lucio Di Filippo Anna Manfredi Marcello Salvi Antonio Limone Luigi Atripaldi Pellegrino Cerino Andrea Ballabio Davide Cacchiarelli
EPI_ISL_1447967	20210218375	TIGEM	Antonio Grimaldi Patrizia Annunziata Francesco Panariello Biancamaria Pierri Claudia Tiberio Valentina Bouche Chiara Colantuono Maria Concetta Cuomo Denise Di Concilio Lucio Di Filippo Anna Manfredi Marcello Salvi Antonio Limone Luigi Atripaldi Pellegrino Cerino Andrea Ballabio Davide Cacchiarelli
EPI_ISL_1447968	20210300057	TIGEM	Antonio Grimaldi Patrizia Annunziata Francesco Panariello Biancamaria Pierri Claudia Tiberio Valentina Bouche Chiara Colantuono Maria Concetta Cuomo Denise Di Concilio Lucio Di Filippo Anna Manfredi Marcello Salvi Antonio Limone Luigi Atripaldi Pellegrino Cerino Andrea Ballabio Davide Cacchiarelli
EPI_ISL_1447969	20210300061	TIGEM	Antonio Grimaldi Patrizia Annunziata Francesco Panariello Biancamaria Pierri Claudia Tiberio Valentina Bouche Chiara Colantuono Maria Concetta Cuomo Denise Di Concilio Lucio Di Filippo Anna Manfredi Marcello Salvi Antonio Limone Luigi Atripaldi Pellegrino Cerino Andrea Ballabio Davide Cacchiarelli
EPI_ISL_1447966	20210300066	TIGEM	Antonio Grimaldi Patrizia Annunziata Francesco Panariello Biancamaria Pierri Claudia Tiberio Valentina Bouche Chiara Colantuono Maria Concetta Cuomo Denise Di Concilio Lucio Di Filippo Anna Manfredi Marcello Salvi Antonio Limone Luigi Atripaldi Pellegrino Cerino Andrea Ballabio Davide Cacchiarelli
EPI_ISL_1447981	20210300406	TIGEM	Antonio Grimaldi Patrizia Annunziata Francesco Panariello Biancamaria Pierri Claudia Tiberio Valentina Bouche Chiara Colantuono Maria Concetta Cuomo Denise Di Concilio Lucio Di Filippo Anna Manfredi Marcello Salvi Antonio Limone Luigi Atripaldi Pellegrino Cerino Andrea Ballabio Davide Cacchiarelli
EPI_ISL_1447988	20210300533	TIGEM	Antonio Grimaldi Patrizia Annunziata Francesco Panariello Biancamaria Pierri Claudia Tiberio Valentina Bouche Chiara Colantuono Maria Concetta Cuomo Denise Di Concilio Lucio Di Filippo Anna Manfredi Marcello Salvi Antonio Limone Luigi Atripaldi Pellegrino Cerino Andrea Ballabio Davide Cacchiarelli
EPI_ISL_1447973	20210300708	TIGEM	Antonio Grimaldi Patrizia Annunziata Francesco Panariello Biancamaria Pierri Claudia Tiberio Valentina Bouche Chiara Colantuono Maria Concetta Cuomo Denise Di Concilio Lucio Di Filippo Anna Manfredi Marcello Salvi Antonio Limone Luigi Atripaldi Pellegrino Cerino Andrea Ballabio Davide Cacchiarelli
EPI_ISL_1447989	20210300725	TIGEM	Antonio Grimaldi Patrizia Annunziata Francesco Panariello Biancamaria Pierri Claudia Tiberio Valentina Bouche Chiara Colantuono Maria Concetta Cuomo Denise Di Concilio Lucio Di Filippo Anna Manfredi Marcello Salvi Antonio Limone Luigi Atripaldi Pellegrino Cerino Andrea Ballabio Davide Cacchiarelli
EPI_ISL_1447987	20210300728	TIGEM	Antonio Grimaldi Patrizia Annunziata Francesco Panariello Biancamaria Pierri Claudia Tiberio Valentina Bouche Chiara Colantuono Maria Concetta Cuomo Denise Di Concilio Lucio Di Filippo Anna Manfredi Marcello Salvi Antonio Limone Luigi Atripaldi Pellegrino Cerino Andrea Ballabio Davide Cacchiarelli
EPI_ISL_1447972	20210300761	TIGEM	Antonio Grimaldi Patrizia Annunziata Francesco Panariello Biancamaria Pierri Claudia Tiberio Valentina Bouche Chiara Colantuono Maria Concetta Cuomo Denise Di Concilio Lucio Di Filippo Anna Manfredi Marcello Salvi Antonio Limone Luigi Atripaldi Pellegrino Cerino Andrea Ballabio Davide Cacchiarelli
EPI_ISL_1447983	20210300801	TIGEM	Antonio Grimaldi Patrizia Annunziata Francesco Panariello Biancamaria Pierri Claudia Tiberio Valentina Bouche Chiara Colantuono Maria Concetta Cuomo Denise Di Concilio Lucio Di Filippo Anna Manfredi Marcello Salvi Antonio Limone Luigi Atripaldi Pellegrino Cerino Andrea Ballabio Davide Cacchiarelli
EPI_ISL_1447984	20210300812	TIGEM	Antonio Grimaldi Patrizia Annunziata Francesco Panariello Biancamaria Pierri Claudia Tiberio Valentina Bouche Chiara Colantuono Maria Concetta Cuomo Denise Di Concilio Lucio Di Filippo Anna Manfredi Marcello Salvi Antonio Limone Luigi Atripaldi Pellegrino Cerino Andrea Ballabio Davide Cacchiarelli
EPI_ISL_1447962	20210300850	TIGEM	Antonio Grimaldi Patrizia Annunziata Francesco Panariello Biancamaria Pierri Claudia Tiberio Valentina Bouche Chiara Colantuono Maria Concetta Cuomo Denise Di Concilio Lucio Di Filippo Anna Manfredi Marcello Salvi Antonio Limone Luigi Atripaldi Pellegrino Cerino Andrea Ballabio Davide Cacchiarelli
EPI_ISL_1447978	20210300851	TIGEM	Antonio Grimaldi Patrizia Annunziata Francesco Panariello Biancamaria Pierri Claudia Tiberio Valentina Bouche Chiara Colantuono Maria Concetta Cuomo Denise Di Concilio Lucio Di Filippo Anna Manfredi Marcello Salvi Antonio Limone Luigi Atripaldi Pellegrino Cerino Andrea Ballabio Davide Cacchiarelli
EPI_ISL_1447979	20210300858	TIGEM	Antonio Grimaldi Patrizia Annunziata Francesco Panariello Biancamaria Pierri Claudia Tiberio Valentina Bouche Chiara Colantuono Maria Concetta Cuomo Denise Di Concilio Lucio Di Filippo Anna Manfredi Marcello Salvi Antonio Limone Luigi Atripaldi Pellegrino Cerino Andrea Ballabio Davide Cacchiarelli
EPI_ISL_1447985	20210300868	TIGEM	Antonio Grimaldi Patrizia Annunziata Francesco Panariello Biancamaria Pierri Claudia Tiberio Valentina Bouche Chiara Colantuono Maria Concetta Cuomo Denise Di Concilio Lucio Di Filippo Anna Manfredi Marcello Salvi Antonio Limone Luigi Atripaldi Pellegrino Cerino Andrea Ballabio Davide Cacchiarelli
EPI_ISL_1447965	20210300911	TIGEM	Antonio Grimaldi Patrizia Annunziata Francesco Panariello Biancamaria Pierri Claudia Tiberio Valentina Bouche Chiara Colantuono Maria Concetta Cuomo Denise Di Concilio Lucio Di Filippo Anna Manfredi Marcello Salvi Antonio Limone Luigi Atripaldi Pellegrino Cerino Andrea Ballabio Davide Cacchiarelli
EPI_ISL_1447974	20210301612	TIGEM	Antonio Grimaldi Patrizia Annunziata Francesco Panariello Biancamaria Pierri Claudia Tiberio Valentina Bouche Chiara Colantuono Maria Concetta Cuomo Denise Di Concilio Lucio Di Filippo Anna Manfredi Marcello Salvi Antonio Limone Luigi Atripaldi Pellegrino Cerino Andrea Ballabio Davide Cacchiarelli
EPI_ISL_1447964	20210301624	TIGEM	Antonio Grimaldi Patrizia Annunziata Francesco Panariello Biancamaria Pierri Claudia Tiberio Valentina Bouche Chiara Colantuono Maria Concetta Cuomo Denise Di Concilio Lucio Di Filippo Anna Manfredi Marcello Salvi Antonio Limone Luigi Atripaldi Pellegrino Cerino Andrea Ballabio Davide Cacchiarelli
EPI_ISL_1447963	20210301640	TIGEM	Antonio Grimaldi Patrizia Annunziata Francesco Panariello Biancamaria Pierri Claudia Tiberio Valentina Bouche Chiara Colantuono Maria Concetta Cuomo Denise Di Concilio Lucio Di Filippo Anna Manfredi Marcello Salvi Antonio Limone Luigi Atripaldi Pellegrino Cerino Andrea Ballabio Davide

			Cacchiarelli
EPI_ISL_1447986	20210301662	TIGEM	Antonio Grimaldi Patrizia Annunziata Francesco Panariello Biancamaria Pierri Claudia Tiberio Valentina Bouche Chiara Colantuono Maria Concetta Cuomo Denise Di Concilio Lucio Di Filippo Anna Manfredi Marcello Salvi Antonio Limone Luigi Atripaldi Pellegrino Cerino Andrea Ballabio Davide Cacchiarelli
EPI_ISL_1447980	20210301675	TIGEM	Antonio Grimaldi Patrizia Annunziata Francesco Panariello Biancamaria Pierri Claudia Tiberio Valentina Bouche Chiara Colantuono Maria Concetta Cuomo Denise Di Concilio Lucio Di Filippo Anna Manfredi Marcello Salvi Antonio Limone Luigi Atripaldi Pellegrino Cerino Andrea Ballabio Davide Cacchiarelli
EPI_ISL_1447941	20210302039	TIGEM	Antonio Grimaldi Patrizia Annunziata Francesco Panariello Biancamaria Pierri Claudia Tiberio Valentina Bouche Chiara Colantuono Maria Concetta Cuomo Denise Di Concilio Lucio Di Filippo Anna Manfredi Marcello Salvi Antonio Limone Luigi Atripaldi Pellegrino Cerino Andrea Ballabio Davide Cacchiarelli
EPI_ISL_1448010	20210302199	TIGEM	Antonio Grimaldi Patrizia Annunziata Francesco Panariello Biancamaria Pierri Claudia Tiberio Valentina Bouche Chiara Colantuono Maria Concetta Cuomo Denise Di Concilio Lucio Di Filippo Anna Manfredi Marcello Salvi Antonio Limone Luigi Atripaldi Pellegrino Cerino Andrea Ballabio Davide Cacchiarelli
EPI_ISL_1447937	20210302215	TIGEM	Antonio Grimaldi Patrizia Annunziata Francesco Panariello Biancamaria Pierri Claudia Tiberio Valentina Bouche Chiara Colantuono Maria Concetta Cuomo Denise Di Concilio Lucio Di Filippo Anna Manfredi Marcello Salvi Antonio Limone Luigi Atripaldi Pellegrino Cerino Andrea Ballabio Davide Cacchiarelli
EPI_ISL_1447940	20210302217	TIGEM	Antonio Grimaldi Patrizia Annunziata Francesco Panariello Biancamaria Pierri Claudia Tiberio Valentina Bouche Chiara Colantuono Maria Concetta Cuomo Denise Di Concilio Lucio Di Filippo Anna Manfredi Marcello Salvi Antonio Limone Luigi Atripaldi Pellegrino Cerino Andrea Ballabio Davide Cacchiarelli
EPI_ISL_1447936	20210302233	TIGEM	Antonio Grimaldi Patrizia Annunziata Francesco Panariello Biancamaria Pierri Claudia Tiberio Valentina Bouche Chiara Colantuono Maria Concetta Cuomo Denise Di Concilio Lucio Di Filippo Anna Manfredi Marcello Salvi Antonio Limone Luigi Atripaldi Pellegrino Cerino Andrea Ballabio Davide Cacchiarelli
EPI_ISL_1447949	20210302239	TIGEM	Antonio Grimaldi Patrizia Annunziata Francesco Panariello Biancamaria Pierri Claudia Tiberio Valentina Bouche Chiara Colantuono Maria Concetta Cuomo Denise Di Concilio Lucio Di Filippo Anna Manfredi Marcello Salvi Antonio Limone Luigi Atripaldi Pellegrino Cerino Andrea Ballabio Davide Cacchiarelli
EPI_ISL_1447942	20210302244	TIGEM	Antonio Grimaldi Patrizia Annunziata Francesco Panariello Biancamaria Pierri Claudia Tiberio Valentina Bouche Chiara Colantuono Maria Concetta Cuomo Denise Di Concilio Lucio Di Filippo Anna Manfredi Marcello Salvi Antonio Limone Luigi Atripaldi Pellegrino Cerino Andrea Ballabio Davide Cacchiarelli
EPI_ISL_1447947	20210302250	TIGEM	Antonio Grimaldi Patrizia Annunziata Francesco Panariello Biancamaria Pierri Claudia Tiberio Valentina Bouche Chiara Colantuono Maria Concetta Cuomo Denise Di Concilio Lucio Di Filippo Anna Manfredi Marcello Salvi Antonio Limone Luigi Atripaldi Pellegrino Cerino Andrea Ballabio Davide Cacchiarelli
EPI_ISL_1447946	20210302266	TIGEM	Antonio Grimaldi Patrizia Annunziata Francesco Panariello Biancamaria Pierri Claudia Tiberio Valentina Bouche Chiara Colantuono Maria Concetta Cuomo Denise Di Concilio Lucio Di Filippo Anna Manfredi Marcello Salvi Antonio Limone Luigi Atripaldi Pellegrino Cerino Andrea Ballabio Davide Cacchiarelli
EPI_ISL_1447938	20210302281	TIGEM	Antonio Grimaldi Patrizia Annunziata Francesco Panariello Biancamaria Pierri Claudia Tiberio Valentina Bouche Chiara Colantuono Maria Concetta Cuomo Denise Di Concilio Lucio Di Filippo Anna Manfredi Marcello Salvi Antonio Limone Luigi Atripaldi Pellegrino Cerino Andrea Ballabio Davide Cacchiarelli
EPI_ISL_1447939	20210302302	TIGEM	Antonio Grimaldi Patrizia Annunziata Francesco Panariello Biancamaria Pierri Claudia Tiberio Valentina Bouche Chiara Colantuono Maria Concetta Cuomo Denise Di Concilio Lucio Di Filippo Anna Manfredi Marcello Salvi Antonio Limone Luigi Atripaldi Pellegrino Cerino Andrea Ballabio Davide Cacchiarelli
EPI_ISL_1447944	20210302922	TIGEM	Antonio Grimaldi Patrizia Annunziata Francesco Panariello Biancamaria Pierri Claudia Tiberio Valentina Bouche Chiara Colantuono Maria Concetta Cuomo Denise Di Concilio Lucio Di Filippo Anna Manfredi Marcello Salvi Antonio Limone Luigi Atripaldi Pellegrino Cerino Andrea Ballabio Davide Cacchiarelli
EPI_ISL_1447931	20210302924	TIGEM	Antonio Grimaldi Patrizia Annunziata Francesco Panariello Biancamaria Pierri Claudia Tiberio Valentina Bouche Chiara Colantuono Maria Concetta Cuomo Denise Di Concilio Lucio Di Filippo Anna Manfredi Marcello Salvi Antonio Limone Luigi Atripaldi Pellegrino Cerino Andrea Ballabio Davide Cacchiarelli
EPI_ISL_1447934	20210302925	TIGEM	Antonio Grimaldi Patrizia Annunziata Francesco Panariello Biancamaria Pierri Claudia Tiberio Valentina Bouche Chiara Colantuono Maria Concetta Cuomo Denise Di Concilio Lucio Di Filippo Anna Manfredi Marcello Salvi Antonio Limone Luigi Atripaldi Pellegrino Cerino Andrea Ballabio Davide Cacchiarelli
EPI_ISL_1447930	20210302931	TIGEM	Antonio Grimaldi Patrizia Annunziata Francesco Panariello Biancamaria Pierri Claudia Tiberio Valentina Bouche Chiara Colantuono Maria Concetta Cuomo Denise Di Concilio Lucio Di Filippo Anna Manfredi Marcello Salvi Antonio Limone Luigi Atripaldi Pellegrino Cerino Andrea Ballabio Davide Cacchiarelli
EPI_ISL_1447932	20210302944	TIGEM	Antonio Grimaldi Patrizia Annunziata Francesco Panariello Biancamaria Pierri Claudia Tiberio Valentina Bouche Chiara Colantuono Maria Concetta Cuomo Denise Di Concilio Lucio Di Filippo Anna Manfredi Marcello Salvi Antonio Limone Luigi Atripaldi Pellegrino Cerino Andrea Ballabio Davide Cacchiarelli
EPI_ISL_1447929	20210302964	TIGEM	Antonio Grimaldi Patrizia Annunziata Francesco Panariello Biancamaria Pierri Claudia Tiberio Valentina Bouche Chiara Colantuono Maria Concetta Cuomo Denise Di Concilio Lucio Di Filippo Anna Manfredi Marcello Salvi Antonio Limone Luigi Atripaldi Pellegrino Cerino Andrea Ballabio Davide Cacchiarelli
EPI_ISL_1447933	20210302988	TIGEM	Antonio Grimaldi Patrizia Annunziata Francesco Panariello Biancamaria Pierri Claudia Tiberio Valentina Bouche Chiara Colantuono Maria Concetta Cuomo Denise Di Concilio Lucio Di Filippo Anna Manfredi Marcello Salvi Antonio Limone Luigi Atripaldi Pellegrino Cerino Andrea Ballabio Davide Cacchiarelli
EPI_ISL_1447945	20210303025	TIGEM	Antonio Grimaldi Patrizia Annunziata Francesco Panariello Biancamaria Pierri Claudia Tiberio Valentina Bouche Chiara Colantuono Maria Concetta Cuomo Denise Di Concilio Lucio Di Filippo Anna Manfredi Marcello Salvi Antonio Limone Luigi Atripaldi Pellegrino Cerino Andrea Ballabio Davide Cacchiarelli
EPI_ISL_1447928	20210303033	TIGEM	Antonio Grimaldi Patrizia Annunziata Francesco Panariello Biancamaria Pierri Claudia Tiberio Valentina Bouche Chiara Colantuono Maria Concetta Cuomo Denise Di Concilio Lucio Di Filippo Anna Manfredi Marcello Salvi Antonio Limone Luigi Atripaldi Pellegrino Cerino Andrea Ballabio Davide Cacchiarelli
EPI_ISL_1447926	20210303049	TIGEM	Antonio Grimaldi Patrizia Annunziata Francesco Panariello Biancamaria Pierri Claudia Tiberio Valentina Bouche Chiara Colantuono Maria Concetta Cuomo Denise Di Concilio Lucio Di Filippo Anna Manfredi Marcello Salvi Antonio Limone Luigi Atripaldi Pellegrino Cerino Andrea Ballabio Davide Cacchiarelli
EPI_ISL_1447935	20210303140	TIGEM	Antonio Grimaldi Patrizia Annunziata Francesco Panariello Biancamaria Pierri Claudia Tiberio Valentina Bouche Chiara Colantuono Maria Concetta Cuomo Denise Di Concilio Lucio Di Filippo Anna Manfredi Marcello Salvi Antonio Limone Luigi Atripaldi Pellegrino Cerino Andrea Ballabio Davide Cacchiarelli
EPI_ISL_1447927	20210303149	TIGEM	Antonio Grimaldi Patrizia Annunziata Francesco Panariello Biancamaria Pierri Claudia Tiberio Valentina Bouche Chiara Colantuono Maria Concetta Cuomo Denise Di Concilio Lucio Di Filippo Anna Manfredi Marcello Salvi Antonio Limone Luigi Atripaldi Pellegrino Cerino Andrea Ballabio Davide

			Cacchiarelli
EPI_ISL_1448008	20210303778	TIGEM	Antonio Grimaldi Patrizia Annunziata Francesco Panariello Biancamaria Pierri Claudia Tiberio Valentina Bouche Chiara Colantuono Maria Concetta Cuomo Denise Di Concilio Lucio Di Filippo Anna Manfredi Marcello Salvi Antonio Limone Luigi Atripaldi Pellegrino Cerino Andrea Ballabio Davide Cacchiarelli
EPI_ISL_1448004	20210303790	TIGEM	Antonio Grimaldi Patrizia Annunziata Francesco Panariello Biancamaria Pierri Claudia Tiberio Valentina Bouche Chiara Colantuono Maria Concetta Cuomo Denise Di Concilio Lucio Di Filippo Anna Manfredi Marcello Salvi Antonio Limone Luigi Atripaldi Pellegrino Cerino Andrea Ballabio Davide Cacchiarelli
EPI_ISL_1447994	20210303793	TIGEM	Antonio Grimaldi Patrizia Annunziata Francesco Panariello Biancamaria Pierri Claudia Tiberio Valentina Bouche Chiara Colantuono Maria Concetta Cuomo Denise Di Concilio Lucio Di Filippo Anna Manfredi Marcello Salvi Antonio Limone Luigi Atripaldi Pellegrino Cerino Andrea Ballabio Davide Cacchiarelli
EPI_ISL_1447990	20210303795	TIGEM	Antonio Grimaldi Patrizia Annunziata Francesco Panariello Biancamaria Pierri Claudia Tiberio Valentina Bouche Chiara Colantuono Maria Concetta Cuomo Denise Di Concilio Lucio Di Filippo Anna Manfredi Marcello Salvi Antonio Limone Luigi Atripaldi Pellegrino Cerino Andrea Ballabio Davide Cacchiarelli
EPI_ISL_1448003	20210303842	TIGEM	Antonio Grimaldi Patrizia Annunziata Francesco Panariello Biancamaria Pierri Claudia Tiberio Valentina Bouche Chiara Colantuono Maria Concetta Cuomo Denise Di Concilio Lucio Di Filippo Anna Manfredi Marcello Salvi Antonio Limone Luigi Atripaldi Pellegrino Cerino Andrea Ballabio Davide Cacchiarelli
EPI_ISL_1447996	20210303844	TIGEM	Antonio Grimaldi Patrizia Annunziata Francesco Panariello Biancamaria Pierri Claudia Tiberio Valentina Bouche Chiara Colantuono Maria Concetta Cuomo Denise Di Concilio Lucio Di Filippo Anna Manfredi Marcello Salvi Antonio Limone Luigi Atripaldi Pellegrino Cerino Andrea Ballabio Davide Cacchiarelli
EPI_ISL_1448007	20210303845	TIGEM	Antonio Grimaldi Patrizia Annunziata Francesco Panariello Biancamaria Pierri Claudia Tiberio Valentina Bouche Chiara Colantuono Maria Concetta Cuomo Denise Di Concilio Lucio Di Filippo Anna Manfredi Marcello Salvi Antonio Limone Luigi Atripaldi Pellegrino Cerino Andrea Ballabio Davide Cacchiarelli
EPI_ISL_1447991	20210303889	TIGEM	Antonio Grimaldi Patrizia Annunziata Francesco Panariello Biancamaria Pierri Claudia Tiberio Valentina Bouche Chiara Colantuono Maria Concetta Cuomo Denise Di Concilio Lucio Di Filippo Anna Manfredi Marcello Salvi Antonio Limone Luigi Atripaldi Pellegrino Cerino Andrea Ballabio Davide Cacchiarelli
EPI_ISL_1448013	20210303909	TIGEM	Antonio Grimaldi Patrizia Annunziata Francesco Panariello Biancamaria Pierri Claudia Tiberio Valentina Bouche Chiara Colantuono Maria Concetta Cuomo Denise Di Concilio Lucio Di Filippo Anna Manfredi Marcello Salvi Antonio Limone Luigi Atripaldi Pellegrino Cerino Andrea Ballabio Davide Cacchiarelli
EPI_ISL_1448009	20210303943	TIGEM	Antonio Grimaldi Patrizia Annunziata Francesco Panariello Biancamaria Pierri Claudia Tiberio Valentina Bouche Chiara Colantuono Maria Concetta Cuomo Denise Di Concilio Lucio Di Filippo Anna Manfredi Marcello Salvi Antonio Limone Luigi Atripaldi Pellegrino Cerino Andrea Ballabio Davide Cacchiarelli
EPI_ISL_1447999	20210303971	TIGEM	Antonio Grimaldi Patrizia Annunziata Francesco Panariello Biancamaria Pierri Claudia Tiberio Valentina Bouche Chiara Colantuono Maria Concetta Cuomo Denise Di Concilio Lucio Di Filippo Anna Manfredi Marcello Salvi Antonio Limone Luigi Atripaldi Pellegrino Cerino Andrea Ballabio Davide Cacchiarelli
EPI_ISL_1447997	20210303976	TIGEM	Antonio Grimaldi Patrizia Annunziata Francesco Panariello Biancamaria Pierri Claudia Tiberio Valentina Bouche Chiara Colantuono Maria Concetta Cuomo Denise Di Concilio Lucio Di Filippo Anna Manfredi Marcello Salvi Antonio Limone Luigi Atripaldi Pellegrino Cerino Andrea Ballabio Davide Cacchiarelli
EPI_ISL_1447995	20210303977	TIGEM	Antonio Grimaldi Patrizia Annunziata Francesco Panariello Biancamaria Pierri Claudia Tiberio Valentina Bouche Chiara Colantuono Maria Concetta Cuomo Denise Di Concilio Lucio Di Filippo Anna Manfredi Marcello Salvi Antonio Limone Luigi Atripaldi Pellegrino Cerino Andrea Ballabio Davide Cacchiarelli
EPI_ISL_1447998	20210303978	TIGEM	Antonio Grimaldi Patrizia Annunziata Francesco Panariello Biancamaria Pierri Claudia Tiberio Valentina Bouche Chiara Colantuono Maria Concetta Cuomo Denise Di Concilio Lucio Di Filippo Anna Manfredi Marcello Salvi Antonio Limone Luigi Atripaldi Pellegrino Cerino Andrea Ballabio Davide Cacchiarelli
EPI_ISL_1448012	20210304041	TIGEM	Antonio Grimaldi Patrizia Annunziata Francesco Panariello Biancamaria Pierri Claudia Tiberio Valentina Bouche Chiara Colantuono Maria Concetta Cuomo Denise Di Concilio Lucio Di Filippo Anna Manfredi Marcello Salvi Antonio Limone Luigi Atripaldi Pellegrino Cerino Andrea Ballabio Davide Cacchiarelli
EPI_ISL_1448014	20210304324	TIGEM	Antonio Grimaldi Patrizia Annunziata Francesco Panariello Biancamaria Pierri Claudia Tiberio Valentina Bouche Chiara Colantuono Maria Concetta Cuomo Denise Di Concilio Lucio Di Filippo Anna Manfredi Marcello Salvi Antonio Limone Luigi Atripaldi Pellegrino Cerino Andrea Ballabio Davide Cacchiarelli
EPI_ISL_1448006	20210304339	TIGEM	Antonio Grimaldi Patrizia Annunziata Francesco Panariello Biancamaria Pierri Claudia Tiberio Valentina Bouche Chiara Colantuono Maria Concetta Cuomo Denise Di Concilio Lucio Di Filippo Anna Manfredi Marcello Salvi Antonio Limone Luigi Atripaldi Pellegrino Cerino Andrea Ballabio Davide Cacchiarelli
EPI_ISL_1448005	20210304373	TIGEM	Antonio Grimaldi Patrizia Annunziata Francesco Panariello Biancamaria Pierri Claudia Tiberio Valentina Bouche Chiara Colantuono Maria Concetta Cuomo Denise Di Concilio Lucio Di Filippo Anna Manfredi Marcello Salvi Antonio Limone Luigi Atripaldi Pellegrino Cerino Andrea Ballabio Davide Cacchiarelli
EPI_ISL_1448011	20210304393	TIGEM	Antonio Grimaldi Patrizia Annunziata Francesco Panariello Biancamaria Pierri Claudia Tiberio Valentina Bouche Chiara Colantuono Maria Concetta Cuomo Denise Di Concilio Lucio Di Filippo Anna Manfredi Marcello Salvi Antonio Limone Luigi Atripaldi Pellegrino Cerino Andrea Ballabio Davide Cacchiarelli
EPI_ISL_1448000	20210304422	TIGEM	Antonio Grimaldi Patrizia Annunziata Francesco Panariello Biancamaria Pierri Claudia Tiberio Valentina Bouche Chiara Colantuono Maria Concetta Cuomo Denise Di Concilio Lucio Di Filippo Anna Manfredi Marcello Salvi Antonio Limone Luigi Atripaldi Pellegrino Cerino Andrea Ballabio Davide Cacchiarelli
EPI_ISL_1447993	20210304470	TIGEM	Antonio Grimaldi Patrizia Annunziata Francesco Panariello Biancamaria Pierri Claudia Tiberio Valentina Bouche Chiara Colantuono Maria Concetta Cuomo Denise Di Concilio Lucio Di Filippo Anna Manfredi Marcello Salvi Antonio Limone Luigi Atripaldi Pellegrino Cerino Andrea Ballabio Davide Cacchiarelli
EPI_ISL_1448002	20210304543	TIGEM	Antonio Grimaldi Patrizia Annunziata Francesco Panariello Biancamaria Pierri Claudia Tiberio Valentina Bouche Chiara Colantuono Maria Concetta Cuomo Denise Di Concilio Lucio Di Filippo Anna Manfredi Marcello Salvi Antonio Limone Luigi Atripaldi Pellegrino Cerino Andrea Ballabio Davide Cacchiarelli
EPI_ISL_1448015	20210304551	TIGEM	Antonio Grimaldi Patrizia Annunziata Francesco Panariello Biancamaria Pierri Claudia Tiberio Valentina Bouche Chiara Colantuono Maria Concetta Cuomo Denise Di Concilio Lucio Di Filippo Anna Manfredi Marcello Salvi Antonio Limone Luigi Atripaldi Pellegrino Cerino Andrea Ballabio Davide Cacchiarelli
EPI_ISL_1448001	20210304568	TIGEM	Antonio Grimaldi Patrizia Annunziata Francesco Panariello Biancamaria Pierri Claudia Tiberio Valentina Bouche Chiara Colantuono Maria Concetta Cuomo Denise Di Concilio Lucio Di Filippo Anna Manfredi Marcello Salvi Antonio Limone Luigi Atripaldi Pellegrino Cerino Andrea Ballabio Davide Cacchiarelli
EPI_ISL_1447992	20210304674	TIGEM	Antonio Grimaldi Patrizia Annunziata Francesco Panariello Biancamaria Pierri Claudia Tiberio Valentina Bouche Chiara Colantuono Maria Concetta Cuomo Denise Di Concilio Lucio Di Filippo Anna Manfredi Marcello Salvi Antonio Limone Luigi Atripaldi Pellegrino Cerino Andrea Ballabio Davide

			Cacchiarelli
EPI_ISL_1447975	20210305370	TIGEM	Antonio Grimaldi Patrizia Annunziata Francesco Panariello Biancamaria Pierri Claudia Tiberio Valentina Bouche Chiara Colantuono Maria Concetta Cuomo Denise Di Concilio Lucio Di Filippo Anna Manfredi Marcello Salvi Antonio Limone Luigi Atripaldi Pellegrino Cerino Andrea Ballabio Davide Cacchiarelli
EPI_ISL_1447976	20210305554	TIGEM	Antonio Grimaldi Patrizia Annunziata Francesco Panariello Biancamaria Pierri Claudia Tiberio Valentina Bouche Chiara Colantuono Maria Concetta Cuomo Denise Di Concilio Lucio Di Filippo Anna Manfredi Marcello Salvi Antonio Limone Luigi Atripaldi Pellegrino Cerino Andrea Ballabio Davide Cacchiarelli
EPI_ISL_1447977	20210305752	TIGEM	Antonio Grimaldi Patrizia Annunziata Francesco Panariello Biancamaria Pierri Claudia Tiberio Valentina Bouche Chiara Colantuono Maria Concetta Cuomo Denise Di Concilio Lucio Di Filippo Anna Manfredi Marcello Salvi Antonio Limone Luigi Atripaldi Pellegrino Cerino Andrea Ballabio Davide Cacchiarelli
EPI_ISL_1495749 to 1495759	3. Medizinische Abteilung, Hanusch Krankenhaus	Bergthaler laboratory, CeMM Research Center for Molecular Medicine of the Austrian Academy of Sciences	Andreas Bergthaler; Anna Schedl; Bekir Erguner; Benedikt Agerer; Christoph Bock; Fabian Amman; Jan Laine; Lukas Endler; Maelle Le Moing; Martin Senekowitsch; Michael Schuster; Petr Triska; Thomas Penz
EPI_ISL_1529005	4919 MacCorkle Ave SE, Charleston, WV 25304	WVU and Marshall University Combined Genomics Core Facilities	"James Denvir; Peter Perrotta; Peter Stoilov; Ryan Percifield"; Wesley Kimble
EPI_ISL_1529003	4920 MacCorkle Ave SE, Charleston, WV 25304	WVU and Marshall University Combined Genomics Core Facilities	"James Denvir; Peter Perrotta; Peter Stoilov; Ryan Percifield"; Wesley Kimble
EPI_ISL_1528876	4923 MacCorkle Ave SE, Charleston, WV 25304	WVU and Marshall University Combined Genomics Core Facilities	"James Denvir; Peter Perrotta; Peter Stoilov; Ryan Percifield"; Wesley Kimble
EPI_ISL_1528900	4924 MacCorkle Ave SE, Charleston, WV 25304	WVU and Marshall University Combined Genomics Core Facilities	"James Denvir; Peter Perrotta; Peter Stoilov; Ryan Percifield"; Wesley Kimble
EPI_ISL_1528871	4926 MacCorkle Ave SE, Charleston, WV 25304	WVU and Marshall University Combined Genomics Core Facilities	"James Denvir; Peter Perrotta; Peter Stoilov; Ryan Percifield"; Wesley Kimble
EPI_ISL_1528899	4927 MacCorkle Ave SE, Charleston, WV 25304	WVU and Marshall University Combined Genomics Core Facilities	"James Denvir; Peter Perrotta; Peter Stoilov; Ryan Percifield"; Wesley Kimble
EPI_ISL_1528894	4929 MacCorkle Ave SE, Charleston, WV 25304	WVU and Marshall University Combined Genomics Core Facilities	"James Denvir; Peter Perrotta; Peter Stoilov; Ryan Percifield"; Wesley Kimble
EPI_ISL_1528902	4930 MacCorkle Ave SE, Charleston, WV 25304	WVU and Marshall University Combined Genomics Core Facilities	"James Denvir; Peter Perrotta; Peter Stoilov; Ryan Percifield"; Wesley Kimble
EPI_ISL_1528896	4931 MacCorkle Ave SE, Charleston, WV 25304	WVU and Marshall University Combined Genomics Core Facilities	"James Denvir; Peter Perrotta; Peter Stoilov; Ryan Percifield"; Wesley Kimble
EPI_ISL_1528877	4932 MacCorkle Ave SE, Charleston, WV 25304	WVU and Marshall University Combined Genomics Core Facilities	"James Denvir; Peter Perrotta; Peter Stoilov; Ryan Percifield"; Wesley Kimble
EPI_ISL_1528886	4933 MacCorkle Ave SE, Charleston, WV 25304	WVU and Marshall University Combined Genomics Core Facilities	"James Denvir; Peter Perrotta; Peter Stoilov; Ryan Percifield"; Wesley Kimble
EPI_ISL_1528897	4934 MacCorkle Ave SE, Charleston, WV 25304	WVU and Marshall University Combined Genomics Core Facilities	"James Denvir; Peter Perrotta; Peter Stoilov; Ryan Percifield"; Wesley Kimble
EPI_ISL_1528873	4935 MacCorkle Ave SE, Charleston, WV 25304	WVU and Marshall University Combined Genomics Core Facilities	"James Denvir; Peter Perrotta; Peter Stoilov; Ryan Percifield"; Wesley Kimble
EPI_ISL_1528892	4936 MacCorkle Ave SE, Charleston, WV 25304	WVU and Marshall University Combined Genomics Core Facilities	"James Denvir; Peter Perrotta; Peter Stoilov; Ryan Percifield"; Wesley Kimble
EPI_ISL_1528895	4937 MacCorkle Ave SE, Charleston, WV 25304	WVU and Marshall University Combined Genomics Core Facilities	"James Denvir; Peter Perrotta; Peter Stoilov; Ryan Percifield"; Wesley Kimble
EPI_ISL_1528908	4938 MacCorkle Ave SE, Charleston, WV 25304	WVU and Marshall University Combined Genomics Core Facilities	"James Denvir; Peter Perrotta; Peter Stoilov; Ryan Percifield"; Wesley Kimble
EPI_ISL_1528898	4940 MacCorkle Ave SE, Charleston, WV 25304	WVU and Marshall University Combined Genomics Core Facilities	"James Denvir; Peter Perrotta; Peter Stoilov; Ryan Percifield"; Wesley Kimble
EPI_ISL_1528874	4944 MacCorkle Ave SE, Charleston, WV 25304	WVU and Marshall University Combined Genomics Core Facilities	"James Denvir; Peter Perrotta; Peter Stoilov; Ryan Percifield"; Wesley Kimble
EPI_ISL_1528872	4945 MacCorkle Ave SE, Charleston, WV 25304	WVU and Marshall University Combined Genomics Core Facilities	"James Denvir; Peter Perrotta; Peter Stoilov; Ryan Percifield"; Wesley Kimble
EPI_ISL_1528893	4948 MacCorkle Ave SE, Charleston, WV 25304	WVU and Marshall University Combined Genomics Core Facilities	"James Denvir; Peter Perrotta; Peter Stoilov; Ryan Percifield"; Wesley Kimble
EPI_ISL_1528920	4949 MacCorkle Ave SE, Charleston, WV 25304	WVU and Marshall University Combined Genomics Core Facilities	"James Denvir; Peter Perrotta; Peter Stoilov; Ryan Percifield"; Wesley Kimble
EPI_ISL_1528901	4950 MacCorkle Ave SE, Charleston, WV 25304	WVU and Marshall University Combined Genomics Core Facilities	"James Denvir; Peter Perrotta; Peter Stoilov; Ryan Percifield"; Wesley Kimble
EPI_ISL_1528909	4951 MacCorkle Ave SE, Charleston, WV 25304	WVU and Marshall University Combined Genomics Core Facilities	"James Denvir; Peter Perrotta; Peter Stoilov; Ryan Percifield"; Wesley Kimble
EPI_ISL_1528888	4952 MacCorkle Ave SE, Charleston, WV 25304	WVU and Marshall University Combined Genomics Core Facilities	"James Denvir; Peter Perrotta; Peter Stoilov; Ryan Percifield"; Wesley Kimble
EPI_ISL_1528875	4954 MacCorkle Ave SE, Charleston, WV 25304	WVU and Marshall University Combined Genomics Core Facilities	"James Denvir; Peter Perrotta; Peter Stoilov; Ryan Percifield"; Wesley Kimble
EPI_ISL_1528907	4956 MacCorkle Ave SE, Charleston, WV 25304	WVU and Marshall University Combined Genomics Core Facilities	"James Denvir; Peter Perrotta; Peter Stoilov; Ryan Percifield"; Wesley Kimble
EPI_ISL_1528921	4957 MacCorkle Ave SE, Charleston, WV 25304	WVU and Marshall University Combined Genomics Core Facilities	"James Denvir; Peter Perrotta; Peter Stoilov; Ryan Percifield"; Wesley Kimble
EPI_ISL_1528889	4958 MacCorkle Ave SE, Charleston, WV 25304	WVU and Marshall University Combined Genomics Core Facilities	"James Denvir; Peter Perrotta; Peter Stoilov; Ryan Percifield"; Wesley Kimble
EPI_ISL_1528905	4959 MacCorkle Ave SE, Charleston, WV 25304	WVU and Marshall University Combined Genomics Core Facilities	"James Denvir; Peter Perrotta; Peter Stoilov; Ryan Percifield"; Wesley Kimble
EPI_ISL_1528903	4960 MacCorkle Ave SE, Charleston, WV 25304	WVU and Marshall University Combined Genomics Core	"James Denvir; Peter Perrotta; Peter Stoilov; Ryan Percifield"; Wesley Kimble

		Facilities	
EPI_ISL_513348 to 513349, EPI_ISL_513402, EPI_ISL_513413, EPI_ISL_526186, EPI_ISL_593646 to 593648	4Cyte Pathology	NSW Health Pathology - Institute of Clinical Pathology and Medical Research; Westmead Hospital; University of Sydney	CIDM-PH et al.
EPI_ISL_1492578	ACISMOM	INMI Lazzaro Spallanzani IRCCS	A Di Caro; B Bartolini; CEM Gruber; E Giombini; F Messina; F Santini; G Bonfiglio; I Giambini; M Rueca; MM Todini; MR Capobianchi; O Butera
EPI_ISL_455042 to 455043, EPI_ISL_455050, EPI_ISL_455074	ACT Pathology	NSW Health Pathology - Institute of Clinical Pathology and Medical Research; Westmead Hospital; University of Sydney	CIDM-PH et al.
EPI_ISL_498468 to 498548	ACT Pathology	Schwessinger Lab	Ashley Jones; Benjamin Schwessinger; Craig Kennedy; Karina Kennedy; Kevin Murray; Megan McDonald; Ming-Dao Chia; Robert Lanfear; Robyn N Hall
EPI_ISL_427711, EPI_ISL_427717 to 427719, EPI_ISL_427721	ACT Pathology, The Canberra Hospital	NSW Health Pathology - Institute of Clinical Pathology and Medical Research; Westmead Hospital; University of Sydney	Arnott A; Bachmann N; Basile K; Byun R; Carter I; Chang S; Chen SC; Draper J; Dwyer DE for the 2019-nCoV Study Group; Eden JS; Gall M; Gray K; Holmes EC; Kok J; Lam C; Maddocks S; O'Sullivan MV; Propenko M; Rockett R; Sadsad R; Sim E; Sintchenko V; Sorrell T; Timms V
EPI_ISL_451540, EPI_ISL_451588, EPI_ISL_451590 to 451593, EPI_ISL_451595 to 451596, EPI_ISL_451598, EPI_ISL_451612	ACT pathology	NSW Health Pathology - Institute of Clinical Pathology and Medical Research; Westmead Hospital; University of Sydney	CIDM-PH et al.
EPI_ISL_1397424 to 1397443, EPI_ISL_1397711, EPI_ISL_1397721, EPI_ISL_1397723, EPI_ISL_1397731, EPI_ISL_1397733 to 1397735, EPI_ISL_1397738 to 1397739, EPI_ISL_1397741, EPI_ISL_1397743, EPI_ISL_1397745 to 1397751, EPI_ISL_1397766			
see above	ADIRONDACK MEDICAL CENTER	Wadsworth Center, New York State Department of Health	Alexis Russel; Daryl M. Lamson; Erasmus Schneider; Erica Lasek-Nesselquist; John Kelly; Jonathan Plitnick; Kirsten St. George; Matthew Shudt; Melissa A Leisner; Navjot Singh
EPI_ISL_1420644, EPI_ISL_1420646, EPI_ISL_1420703, EPI_ISL_1420751	AHEPA, Laboratory of Microbiology	Greek Genome Center, Biomedical Research Foundation of the Academy of Athens (BRFAA)	Dimitrios Thanos; Emmanouil Athanasiadis; Ioannis Vatsellas; Katerina Zoi; Lemonia Skoura; Theodoros Loupis
EPI_ISL_602622 to 602631, EPI_ISL_605784	AHRI-Sigal	KRISP, KZN Research Innovation and Sequencing Platform	Cele S; Gazy I; Giandhari J; Karim F; Pillay S; Sigal A; Sigl A; Sigla; Tegally H; Wilkinson E; de Oliveira T
EPI_ISL_1494955 to 1494956	AIDALAB S.A.S	Instituto Nacional de Salud- Dirección de Investigación en Salud Pública	Carlos Franco-Muñoz; Carmen Osorio; Diana Malo; Diego A. Álvarez-Díaz; Diego Andrés Prada; Gerardo Santamaría; Hector Alejandro Ruiz-Moreno; Jhonnatan Reales-González; Juan Camilo Martinez; Julian Naizaque; Katherine Laiton-Donato; Lisseth Pardo; Magdalena Wiesner; Marcela Mercado-Reyes; Maria T. Herrera-Sepúlveda; Marta Lopez Blanco; Martha Lucia Ospina Martinez; Paola Rojas; Sergio Gomez; Sheryll Corchuelo; Ángela Alarcon Cruz
EPI_ISL_1446595 to 1446597, EPI_ISL_1446601, EPI_ISL_1446855 to 1446856, EPI_ISL_1446944, EPI_ISL_1447002, EPI_ISL_1528478	AL Dept. of Public Health Bureau of Clinical Laboratories	Centers for Disease Control and Prevention Division of Viral Diseases, Pathogen Discovery	Alison Laufer Halpin; Ben L. Rambo-Martin; Clinton R. Paden; Dakota Howard; Darlene Wagner; Dave Wentworth; Dhwani Batra; Jasmine Padilla; Justin Lee; Katie Dillon; Krista Queen; Kristen Knipe; Kristine Lacek; Mark Burroughs; Matthew Schmerer; Mili Sheth; Peter Cook; Sam Shepard; Sarah Nobles; Shoshona Le; Suxiang Tong; Vivien Dugan; Yvette Unoarumhi
EPI_ISL_1447205, EPI_ISL_1447239 to 1447240, EPI_ISL_1447242, EPI_ISL_1447277	ALAB Grudzieniec	1. National Institute of Public Health - National Institute of Hygiene; 2. Eurofins Genomics Europe Sequencing GmbH	ECDC COVID-19 WGS support team; Eurofins Genomics Europe Sequencing Team; Gierczyki Rafa; Sadkowska-Todys Magorzata; Wokowicz Tomasz; Zacharczuk Katarzyna
EPI_ISL_1538840 to 1538841	ALAB laboratoria sp. z o. o. Laboratorium Analiz Lekarskich	1. National Institute of Public Health - National Institute of Hygiene; 2. Eurofins Genomics Europe Sequencing GmbH	ECDC COVID-19 WGS support team; Eurofins Genomics Europe Sequencing Team; Gierczyki Rafa; Sadkowska-Todys Magorzata; Wokowicz Tomasz; Zacharczuk Katarzyna
EPI_ISL_1433035, EPI_ISL_1433037, EPI_ISL_1433094, EPI_ISL_1433134, EPI_ISL_1433219, EPI_ISL_1433248, EPI_ISL_1433260, EPI_ISL_1433276, EPI_ISL_1438687, EPI_ISL_1438712, EPI_ISL_1438726, EPI_ISL_1438731, EPI_ISL_1438778, EPI_ISL_1438797 to 1438799, EPI_ISL_1438815, EPI_ISL_1438843, EPI_ISL_1438865, EPI_ISL_1438880, EPI_ISL_1438929, EPI_ISL_1438958, EPI_ISL_1438974, EPI_ISL_1438983, EPI_ISL_1438985, EPI_ISL_1438995, EPI_ISL_1439007, EPI_ISL_1439018			
see above	ALB FILS KLINIKEN GmbH - Institut für Laboratoriumsmedizin Klinik am Eichert Göppingen	Robert Koch Institute	
EPI_ISL_1397542 to 1397545, EPI_ISL_1397551, EPI_ISL_1397707 to 1397710, EPI_ISL_1397712 to 1397720, EPI_ISL_1397722, EPI_ISL_1397724 to 1397730, EPI_ISL_1397732, EPI_ISL_1397736 to 1397737, EPI_ISL_1397740, EPI_ISL_1397742, EPI_ISL_1397744, EPI_ISL_1397752 to 1397765, EPI_ISL_1397767, EPI_ISL_1397834 to 1397835, EPI_ISL_1397837, EPI_ISL_1397843, EPI_ISL_1397853 to 1397856, EPI_ISL_1397858, EPI_ISL_1397861, EPI_ISL_1397866 to 1397869, EPI_ISL_1397961, EPI_ISL_1398021 to 1398027, EPI_ISL_1398147 to 1398153, EPI_ISL_1398274 to 1398283, EPI_ISL_1461295 to 1461299, EPI_ISL_1462506, EPI_ISL_1462508, EPI_ISL_1465615 to 1465616, EPI_ISL_1465634 to 1465636, EPI_ISL_1465639, EPI_ISL_1482455 to 1482469			
see above	ALBANY MEDICAL CENTER	Wadsworth Center, New York State Department of Health	Alexis Russel; Alexis Russell; Catharine Prussing; Daryl M. Lamson; Erasmus Schneider; Erica Lasek-Nesselquist; John Kelly; Jonathan Plitnick; Kirsten St. George; Matthew Shudt; Melissa A Leisner; Navjot Singh
EPI_ISL_1499457 to 1499473	ALBANY MEDICAL CENTER HOSPITAL CLINICAL LABORATORIES	Wadsworth Center, New York State Department of Health	Alexis Russel; Daryl M. Lamson; Erasmus Schneider; Erica Lasek-Nesselquist; John Kelly; Jonathan Plitnick; Kirsten St. George; Matthew Shudt; Melissa A Leisner; Navjot Singh
EPI_ISL_471546	AMA DR Jose Soares Hungria	Instituto Adolfo Lutz, Interdiciplinary Procedures Center, Strategic Laboratory	Claudia Regina Gonçalves; Claudio Tavares Sacchi; Erica Valessa Ramos Gomes
EPI_ISL_523989	AMA Jardim Joamar	Instituto Adolfo Lutz, Interdiciplinary Procedures Center, Strategic Laboratory	Claudia Regina Gonçalves; Claudio Tavares Sacchi; Erica Valessa Ramos Gomes
EPI_ISL_523990	AMA Jardim Peri	Instituto Adolfo Lutz, Interdiciplinary Procedures Center, Strategic Laboratory	Claudia Regina Gonçalves; Claudio Tavares Sacchi; Erica Valessa Ramos Gomes
EPI_ISL_1445264, EPI_ISL_1445266 to 1445267, EPI_ISL_1445273 to 1445274	AMBULATORIO MEDICO DE ESPECIALIDADES DE PERUIBE	Instituto Butantan / Mendelics	Antonio Jorge Martins; Bibiana Santos; Claudia Renata dos Santos Barros; David Schlesinger; Debora Botequio Moretti; Dimas Tadeu Covas; Elaine Cristina Marqueze; Elaine Vieira dos Santos; Erika Freitas; Evandra Strazza Rodrigues; Flavia Aburjaile; José Salvatore Leister Patané; João Paulo Kitajima; Luiz Carlos Junior de Alcantara; Maria Carolina Elias; Marta Giovanetti; Rafael dos Santos Bezerra; Raul Machado Neto; Ricardo Haddad; Rodrigo Tocantins Calado.; Sandra Coccuzzo Sampaio; Simone Kashima; Svetoslav Nanev Slavov; Vagner Fonseca; Vincent Louis Viala
EPI_ISL_467432 to 467435, EPI_ISL_467449 to 467474	AMPATH-DBN	KRISP, KZN Research Innovation and Sequencing Platform	Chimukangara B; Giandhari J; Khan S; Lessells R; Mdlalose K; Pillay S; Tegally H; Wilkinson E; York D; de Oliveira T
EPI_ISL_458150	ANOUAL	ANOUAL	Azami Nawfel; Benhida Rachid; Chenaoui Mohamed; El Aliani Aissam; El Ansari Fatima Zahra; Fekkak Jamal; Jouali Farah; Kasmi Yassine; Kitane Driss Lahlou; Loukman Salma; Marchoudi Nabila
EPI_ISL_1547799 to 1547800	AP SSO	CSIR-Centre for Cellular and Molecular Biology - INSACOG	Amareshwar Vodapalli; Ara Sreenivas; B Himasri; Divya Tej Sowpati; Karthik Bharadwaj Tallapaka; Krishna Khairnar; Lamuk Zaveri; Onkar Kulkarni; Rakesh K Mishra; Sharath Chandra Thota; Shreekant Verma; Sofia Banu; Viswagithe S L
EPI_ISL_1516317 to 1516321	AR Dept. of Health-PHL, Molecular Diagnostics	Centers for Disease Control and Prevention Division of Viral Diseases, Pathogen Discovery	Alison Laufer Halpin; Ben L. Rambo-Martin; Clinton R. Paden; Dakota Howard; Darlene Wagner; Dave Wentworth; Dhwani Batra; Jasmine Padilla; Justin Lee; Katie Dillon; Krista Queen; Kristen Knipe; Kristine Lacek; Mark Burroughs; Matthew Schmerer; Mili Sheth; Peter Cook; Sam Shepard; Sarah Nobles; Shoshona Le; Suxiang Tong; Vivien Dugan; Yvette Unoarumhi
EPI_ISL_450800, EPI_ISL_509663 to 509668, EPI_ISL_509670 to 509685, EPI_ISL_527632 to 527657, EPI_ISL_535650 to 535661, EPI_ISL_576196 to 576197	AR Dept. of Health-Public Health Lab	Pathogen Discovery, Respiratory Viruses Branch, Division of Viral Diseases, Centers for Disease Control and Prevention	Anna Montmayeur; Anna Uehara; Bettina Bankamp; Brian Lynch; Clinton Paden; Clinton R. Paden; Haibin Wang; Jing Zhang; Krista Queen; Peter Cook; Rachel Marine; Suxiang Tong; Yan Li; Ying Tao; Zachary Weiner
EPI_ISL_418000 to 418001, EPI_ISL_418004	ARS Algarve - Laboratório Laura Ayres	Instituto Nacional de Saude (INSA)	Guiomar et al
EPI_ISL_1527024	AS Belén Flores	Inciensa, Instituto Costarricense de Investigación y Enseñanza en Nutrición y Salud	Barboza-Arguedas E & Centeno-Miranda M; Pérez-Corrales C
EPI_ISL_445326, EPI_ISL_445363, EPI_ISL_445367	ASISTENCIA PUBLICA DR.ALEJANDRO DEL RIO	Instituto de Salud Publica de Chile	Alejandra Acevedo; Andrés E Castillo; Bárbara Parra; Carolina Tambley; Gabriel Leal; Jaime Lagos; Jorge Fernandez; Loredana Arata; Patricia Bustos; Paz Tapia; Rodrigo Fasce; Winston Andrade
EPI_ISL_1557254 to 1558513, EPI_ISL_1558515 to 1558518, EPI_ISL_1558520	ASL Napoli 1 Centro	AMES Centro Polidiagnostico Strumentale S.r.l.	"Giovanni Savarese; Antonella Di Carlo; Antonio Fico"; Eloisa Evangelista; Luigi D'Amore; Luisa Circelli; Maurizio D'Amora; Monica Ianniello; Nadia Petrillo; Raffaella Ruggiero; Roberto Sirica

EPI_ISL_542098 to 542277, EPI_ISL_542400 to 542443	ASST GOM Niguarda	Dep. Of Oncology and Hemato-Oncology University of Milan	Antonio Piralla; Carlo Federico Perno; Chiara Vismara; Claudia Alteri; Elisa Matarazzo; Fausto Baldanti; Federica Giardina; Federica Novazzi; Luna Colagrossi; Maria Antonello; Massimo Puoti; Monica Tallarita; Oscar Massimiliano Epis; Roberto Fumagalli; Silvia Renica; Stefano Gaiarsa; Valentino Costabile; Valeria Cento
EPI_ISL_1500446 to 1500447	ASST della Brianza	Laboratory of Clinical Microbiology, Virology and Bioemergencies, ASST Fatebenefratelli Sacco - Sacco Hospital	Alberto Rizzo; Alessandro Mancon; Fiorenza Bracchitta; Luca Rizzuto; Maria Rita Gismondo; Valeria Micheli
EPI_ISL_1433783, EPI_ISL_1433787, EPI_ISL_1433900, EPI_ISL_1433906, EPI_ISL_1433910, EPI_ISL_1433912, EPI_ISL_1433917, EPI_ISL_1433925, EPI_ISL_1433927, EPI_ISL_1433929, EPI_ISL_1434063, EPI_ISL_1434065, EPI_ISL_1434067, EPI_ISL_1434069, EPI_ISL_1434190 to 1434191, EPI_ISL_1434193, EPI_ISL_1434198 to 1434199, EPI_ISL_1457765			
see above	ASTRALAB LIMOGES	CNR Virus des Infections Respiratoires - France SUD	Antonin Bal; Bruno Lina; Gregory Destras; Gwendolyne Burfin; Hadrien Regue; Laurence Josset; Martine Valette; Quentin Semanas
EPI_ISL_1418241, EPI_ISL_1461136 to 1461137	AX BIO OCEAN	CNR Virus des Infections Respiratoires - France SUD	Antonin Bal; Bruno Lina; Gregory Destras; Gwendolyne Burfin; Hadrien Regue; Laurence Josset; Martine Valette; Quentin Semanas
EPI_ISL_420784	AZ Department of Health Services	Pathogen Discovery, Respiratory Viruses Branch, Division of Viral Diseases, Centers for Disease Control and Prevention	Alison S. Laufer Halpin; Anne Uehara; Christopher A. Elkins; Clinton R. Paden; Haibin Wang; Jasmine Padilla; Jing Zhang; Justin Lee; Krista Queen; Mary S. Keckler; Rachel Marine; Suxiang Tong; Yan Li; Ying Tao
EPI_ISL_1403384, EPI_ISL_1403422 to 1403426, EPI_ISL_1403497, EPI_ISL_1403499, EPI_ISL_1403607 to 1403608, EPI_ISL_1403612, EPI_ISL_1403615 to 1403623, EPI_ISL_1403644 to 1403645, EPI_ISL_1403665, EPI_ISL_1456121, EPI_ISL_1456450, EPI_ISL_1457196, EPI_ISL_1457409, EPI_ISL_1457771 to 1457772, EPI_ISL_1457774 to 1457782, EPI_ISL_1457784 to 1457791			
see above	AZ Klina	AZ Klina	Carl Vael; Carl Vael - Lynsey Berckmans; Carl Vael- Lynsey Berckmans; Lynsey Berckmans
EPI_ISL_424848 to 424849, EPI_ISL_452118	AZ SPHL, Arizona Department of Health Services	Pathogen Discovery, Respiratory Viruses Branch, Division of Viral Diseases, Centers for Disease Control and Prevention	Alison S. Laufer Halpin; Anna Montmayeur; Anna Uehara; Christopher A. Elkins; Clinton R. Paden; Haibin Wang; Jing Zhang; Krista Queen; Mary S. Keckler; Rachel Marine; Suxiang Tong; Yan Li; Ying Tao; Zachary Weiner
EPI_ISL_426483, EPI_ISL_426485, EPI_ISL_426512 to 426519, EPI_ISL_426527 to 426531, EPI_ISL_426537 to 426555, EPI_ISL_426558 to 426569, EPI_ISL_427271 to 427272			
see above	AZ SPHL, Arizona Department of Health Services	TGen North	Darrin Lemmer; Dave Engelthaler; Jolene Bowers; Megan Folkerts
EPI_ISL_1402411 to 1402419, EPI_ISL_1402421 to 1402422, EPI_ISL_1408901, EPI_ISL_1408903, EPI_ISL_1408905	AZ Sint-Jan	AZ SINT-JAN BRUGGE	Jorn Hellemans; Laurien Hoornaert; Marijke Reynders; Patrick Descheemaeker; Thomas Van Landschoot
EPI_ISL_1402407 to 1402410, EPI_ISL_1402420, EPI_ISL_1408899 to 1408900, EPI_ISL_1408902, EPI_ISL_1408904, EPI_ISL_1408906	AZ Zeno	AZ SINT-JAN BRUGGE	Jorn Hellemans; Laurien Hoornaert; Marijke Reynders; Patrick Descheemaeker; Thomas Van Landschoot
EPI_ISL_1389354 to 1389395, EPI_ISL_1390731 to 1390767, EPI_ISL_1404395 to 1404434, EPI_ISL_1469122 to 1469158, EPI_ISL_1534709 to 1534748	AZDelta	AZDelta	Dieter De Smet; Geert Martens
EPI_ISL_1388566	Abklärungs- und _Teststation BL	University Hospital Basel, Clinical Bacteriology	Adrian Egli; Alfredo Mari; Hans Hirsch; Helena MB Seth-Smith; Julia Bielicki; Karoline Leuzinger; Madlen Stange; Manuel Battegay; Simon Fuchs; Tim Roloff
EPI_ISL_517616, EPI_ISL_517621 to 517662, EPI_ISL_518799 to 518818	Academic Hospital Paramaribo	Erasmus Medical Center	Bas Oude Munnink; Dion Gajadin; Ed Ijzerman; Emmanuelle Munger; Gary Gummels; Ingrid Krishnadath; Lycke Woittiez; Marion Koopmans; Mireille Van de Veer; Princes Wongsowidjojo; Radjesh Ori; Rohma Banwari; Stephen Vreden
EPI_ISL_476135	Achima Care Fristadens VC	The Public Health Agency of Sweden	Anna Risberg; Anna-Malin Linde; Karin Tegmark-Wisell; Maria Lind Karlberg; Mattias Haukland; Mia Brytting; Olov Svartstrom; Oskar Karlsson Lindsjo; Petra Edquist; Reza Advani; Sandra Broddesson
EPI_ISL_509412 to 509417	Acibadem Labcell Cellular Therapy Laboratory	Acibadem Mehmet Ali Aydinlar University School of Medicine, Medical Genetics Department	Bulut Yurtsever; Cihan Tastan; Derya Dilek Kancagi; Ercument Ovali; Gozde Sir Karakus; Gunseli Bayram Akcapinar; Ilayda Sahin; Ozden Hatirnaz Ng; Ozkan Ozdemir; Sezer Akyoney; Ugur Ozbek
EPI_ISL_458083	Adi Husada Undaan Hospital	Institute of Tropical Disease, Universitas Airlangga	Aldise M Nastri; Gatot Soegiarto; Irawati Marga; Jezzy R Dewantari; Kazufumi Shimizu; Krisnoadi Rahardjo; Laksmi Wulandari; Maria I Lusida; Mitsuhiro Nishimura; Resti Yudhawati; Retno A Setyoningrum; Rima R Prasetya; Soetjipto; Yasuko Mori; Yohko K Shimizu
EPI_ISL_514253	Advanced Biotechnology Laboratory	Genomic Research Lab, BCSIR	Abu Sayeed Mohammad Mahmud; Barna Goswami; Eshrar Osman; Eunus Ali; Hossain Uddin Shekhar; Iffat Jahan; M. Aftab Uddin; Md. Ahashan Habib; Md. Bayejid Hosen; Md. Murshed Hasan Sarkar; Md. Saddam Hossain; Md. Salim Khan; Mohammad Samir Uzzaman; Salek Ahmed Sajib; Shahina Akter; Tanjina Akhter Banu; Utpal Chandra Ray
EPI_ISL_1445276 to 1445977, EPI_ISL_1445980, EPI_ISL_1445983, EPI_ISL_1445987, EPI_ISL_1445990, EPI_ISL_1445993, EPI_ISL_1445996, EPI_ISL_1445999, EPI_ISL_1446002, EPI_ISL_1446005, EPI_ISL_1446008, EPI_ISL_1446011, EPI_ISL_1446013, EPI_ISL_1446016, EPI_ISL_1446019, EPI_ISL_1446022, EPI_ISL_1446025, EPI_ISL_1446029, EPI_ISL_1446032, EPI_ISL_1446035, EPI_ISL_1446038, EPI_ISL_1446041, EPI_ISL_1446044, EPI_ISL_1446047, EPI_ISL_1446050, EPI_ISL_1446053, EPI_ISL_1446057, EPI_ISL_1446060, EPI_ISL_1446063, EPI_ISL_1446066, EPI_ISL_1446069, EPI_ISL_1446071, EPI_ISL_1446075, EPI_ISL_1446077, EPI_ISL_1446081 to 1446193, EPI_ISL_1479441 to 1479682, EPI_ISL_1479802 to 1479869, EPI_ISL_1491733 to 1491949, EPI_ISL_1493494 to 1493571, EPI_ISL_1513139 to 1513630, EPI_ISL_1525019 to 1525088, EPI_ISL_1525090 to 1525248, EPI_ISL_1550389 to 1550505, EPI_ISL_1550507 to 1550655, EPI_ISL_1550657 to 1550793, EPI_ISL_1550795 to 1550877, EPI_ISL_1550963 to 1550971, EPI_ISL_1550973 to 1551027, EPI_ISL_1551029 to 1551201, EPI_ISL_1551203 to 1551361, EPI_ISL_1551363, EPI_ISL_1551366, EPI_ISL_1551369, EPI_ISL_1551371, EPI_ISL_1551374 to 1551451, EPI_ISL_1551453 to 1551555, EPI_ISL_1551557 to 1551614, EPI_ISL_1551616 to 1551621, EPI_ISL_1551623 to 1551642, EPI_ISL_1559627 to 1559659, EPI_ISL_1559700 to 1559704, EPI_ISL_1559706 to 1559725, EPI_ISL_1559727 to 1559739, EPI_ISL_1559741, EPI_ISL_1559744 to 1559747, EPI_ISL_1559749 to 1559765, EPI_ISL_1559767 to 1559779, EPI_ISL_1559800 to 1559833, EPI_ISL_1559835 to 1559838, EPI_ISL_1559840, EPI_ISL_1559842 to 1559851, EPI_ISL_1559853 to 1559878, EPI_ISL_1559880 to 1559938, EPI_ISL_1559940 to 1559968, EPI_ISL_1559970 to 1559973, EPI_ISL_1559975 to 1559985, EPI_ISL_1559987 to 1559999, EPI_ISL_1560010 to 1560029, EPI_ISL_1560040 to 1560049, EPI_ISL_1560090 to 1560099, EPI_ISL_1560127 to 1560133, EPI_ISL_1560135 to 1560160, EPI_ISL_1560162 to 1560174, EPI_ISL_1560176 to 1560232, EPI_ISL_1560234 to 1560270, EPI_ISL_1560272 to 1560273, EPI_ISL_1560275 to 1560280, EPI_ISL_1560282 to 1560298, EPI_ISL_1560300 to 1560307, EPI_ISL_1560309 to 1560389, EPI_ISL_1560391 to 1560398, EPI_ISL_1560400, EPI_ISL_1560402 to 1560419, EPI_ISL_1560421 to 1560431, EPI_ISL_1560433, EPI_ISL_1560435 to 1560442, EPI_ISL_1560444 to 1560445, EPI_ISL_1560447 to 1560453, EPI_ISL_1560455 to 1560461, EPI_ISL_1560465, EPI_ISL_1560468 to 1560498, EPI_ISL_1560500 to 1560515, EPI_ISL_1560517 to 1560520, EPI_ISL_1560522 to 1560555, EPI_ISL_1560557 to 1560585, EPI_ISL_1560587 to 1560592, EPI_ISL_1560594 to 1560621, EPI_ISL_1560623 to 1560625, EPI_ISL_1560627 to 1560634, EPI_ISL_1560636 to 1560666, EPI_ISL_1560668 to 1560680, EPI_ISL_1560682 to 1560688, EPI_ISL_1560690 to 1560698, EPI_ISL_1560700 to 1560716, EPI_ISL_1560718 to 1560734, EPI_ISL_1560736 to 1560765, EPI_ISL_1560767 to 1560785, EPI_ISL_1560787 to 1560830, EPI_ISL_1560832 to 1560857, EPI_ISL_1560859 to 1560914, EPI_ISL_1560916 to 1560927, EPI_ISL_1560929 to 1560954, EPI_ISL_1560956 to 1560958, EPI_ISL_1560960 to 1560965, EPI_ISL_1560967 to 1560987, EPI_ISL_1560989 to 1561005, EPI_ISL_1561007 to 1561018, EPI_ISL_1561020, EPI_ISL_1561022 to 1561026, EPI_ISL_1561029 to 1561045, EPI_ISL_1561048 to 1561049, EPI_ISL_1561051 to 1561056, EPI_ISL_1561058, EPI_ISL_1561060 to 1561069, EPI_ISL_1561071 to 1561075, EPI_ISL_1561077 to 1561081, EPI_ISL_1561083 to 1561109, EPI_ISL_1561111 to 1561113, EPI_ISL_1561115 to 1561127, EPI_ISL_1561129 to 1561143, EPI_ISL_1561145 to 1561181, EPI_ISL_1561183 to 1561197, EPI_ISL_1561199 to 1561201, EPI_ISL_1561203 to 1561236, EPI_ISL_1561239 to 1561263, EPI_ISL_1561265 to 1561283, EPI_ISL_1561285 to 1561295, EPI_ISL_1561297 to 1561322, EPI_ISL_1561324 to 1561335, EPI_ISL_1561337 to 1561349, EPI_ISL_1561351 to 1561395, EPI_ISL_1561398 to 1561415, EPI_ISL_1561417 to 1561427, EPI_ISL_1561429 to 1561435, EPI_ISL_1561437 to 1561455, EPI_ISL_1561457 to 1561469, EPI_ISL_1561471 to 1561472, EPI_ISL_1561475 to 1561508, EPI_ISL_1561510 to 1561512, EPI_ISL_1561514 to 1561579, EPI_ISL_1561581 to 1561603, EPI_ISL_1561605 to 1561606, EPI_ISL_1561608 to 1561635, EPI_ISL_1561637 to 1561695, EPI_ISL_1561697 to 1561737, EPI_ISL_1561739 to 1561743, EPI_ISL_1561745 to 1561747, EPI_ISL_1561749 to 1561761, EPI_ISL_1561763 to 1561773, EPI_ISL_1561775 to 1561788, EPI_ISL_1561791 to 1561792, EPI_ISL_1561794 to 1561819, EPI_ISL_1561821 to 1561822, EPI_ISL_1561824 to 1561848, EPI_ISL_1561850 to 1561855, EPI_ISL_1561857 to 1561865, EPI_ISL_1561867 to 1561875, EPI_ISL_1561877 to 1561881, EPI_ISL_1561883 to 1561893, EPI_ISL_1561896 to 1561920, EPI_ISL_1561922 to 1561935, EPI_ISL_1561937 to 1561969, EPI_ISL_1561972 to 1561993, EPI_ISL_1561995 to 1561999, EPI_ISL_1562001 to 1562008, EPI_ISL_1562010 to 1562013, EPI_ISL_1562015 to 1562038, EPI_ISL_1562042 to 1562068, EPI_ISL_1562070 to 1562074, EPI_ISL_1562076 to 1562083, EPI_ISL_1562086, EPI_ISL_1562088 to 1562101, EPI_ISL_1562103 to 1562104, EPI_ISL_1562106 to 1562119, EPI_ISL_1562121 to 1562153, EPI_ISL_1562155 to 1562201, EPI_ISL_1562203 to 1562233, EPI_ISL_1562235 to 1562271, EPI_ISL_1562273 to 1562319, EPI_ISL_1562321 to 1562328, EPI_ISL_1562331, EPI_ISL_1562333 to 1562361, EPI_ISL_1562364 to 1562407, EPI_ISL_1562409, EPI_ISL_1562411 to 1562425, EPI_ISL_1562427 to 1562442, EPI_ISL_1562444 to 1562503, EPI_ISL_1562505, EPI_ISL_1562507 to 1562520, EPI_ISL_1562522 to 1562554, EPI_ISL_1562556 to 1562594, EPI_ISL_1562596 to 1562598, EPI_ISL_1562600 to 1562643, EPI_ISL_1562645 to 1562671, EPI_ISL_1562673 to 1562677, EPI_ISL_1562679 to 1562761, EPI_ISL_1562763 to 1562766, EPI_ISL_1562768 to 1562823, EPI_ISL_1562825 to 1562841, EPI_ISL_1562843 to 1562885, EPI_ISL_1562887 to 1562915, EPI_ISL_1562917 to 1562920, EPI_ISL_1562922 to 1562938, EPI_ISL_1562940, EPI_ISL_1562943 to 1562959, EPI_ISL_1562961 to 1562962, EPI_ISL_1562964 to 1562968, EPI_ISL_1562970 to 1562981, EPI_ISL_1562983 to 1562987, EPI_ISL_1562989 to 1562997, EPI_ISL_1562999 to 1563000, EPI_ISL_1563002 to 1563019, EPI_ISL_1563022 to 1563066, EPI_ISL_1563068 to 1563107, EPI_ISL_1563110 to 1563124, EPI_ISL_1563126			
see above	Aegis Sciences Corporation	Centers for Disease Control and Prevention Division of Viral Diseases, Pathogen Discovery	Adrian Paskey; Alec Vest; Benjamin Rambo-Martin; Christopher Gulvick; Clinton R. Paden; Cyndi Clark; Dakota Howard; Darlene Wagner; Dhwani Batra; Dillon Nall; Duncan MacCannell; Ethan Sanders; Holly Houdeshell; Jason Caravas; Kara Moser; Matthew Hardison; Matthew Schmerer; Ola Kvalvaag; Patrick Campbell; Peter W. Cook; Rob Case; Scott Sammons; Shatavia Morrison; Shaun Westlund; Vikramsinha Ghorpade; Yvette Unoarumhi
EPI_ISL_528934 to 528949	Agenzia di Tutela della Salute di Bergamo	Istituto Zooprofilattico Sperimentale dell'Abruzzo e Molise "G.Caporale"	Ancora M; Cammà C; Curini V; Di Domenico M; Di Pasquale A; Lorusso A; Mangone I; Marcacci M; Puglia I; Rinaldi A; Savini G.
EPI_ISL_445244	Akbiomed lab	Tejgaon College bmb lab	Md.Abdul kaium; Md.Easin Arafat
EPI_ISL_420134, EPI_ISL_420136, EPI_ISL_420139, EPI_ISL_420311 to 420312, EPI_ISL_500779 to 500783, EPI_ISL_500793 to 500796, EPI_ISL_549083 to 549084, EPI_ISL_549089 to 549091, EPI_ISL_549169 to 549171, EPI_ISL_590889 to 590892, EPI_ISL_590951 to 590952, EPI_ISL_1385963, EPI_ISL_1385966, EPI_ISL_1385969, EPI_ISL_1385971 to 1385972, EPI_ISL_1385974 to 1385976, EPI_ISL_1385979, EPI_ISL_1385984, EPI_ISL_1385987, EPI_ISL_1385990, EPI_ISL_1385993, EPI_ISL_1490202 to 1490203, EPI_ISL_1490205 to 1490214, EPI_ISL_1490216 to 1490218, EPI_ISL_1490278, EPI_ISL_1490284, EPI_ISL_1490291, EPI_ISL_1490296, EPI_ISL_1490299 to 1490300, EPI_ISL_1490303, EPI_ISL_1490338, EPI_ISL_1490382, EPI_ISL_1490385, EPI_ISL_1490392, EPI_ISL_1490394, EPI_ISL_1490420, EPI_ISL_1490423, EPI_ISL_1490425, EPI_ISL_1490427, EPI_ISL_1490460, EPI_ISL_1490467, EPI_ISL_1490503 to 1490505,			

EPI_ISL_1490507 to 1490510, EPI_ISL_1490513 to 1490514, EPI_ISL_1490526 to 1490529, EPI_ISL_1490531 to 1490533, EPI_ISL_1490557 to 1490563, EPI_ISL_1490565, EPI_ISL_1490567 to 1490568, EPI_ISL_1490588, EPI_ISL_1490590 to 1490594, EPI_ISL_1490596 to 1490597, EPI_ISL_1490602, EPI_ISL_1490608, EPI_ISL_1490626, EPI_ISL_1490631, EPI_ISL_1547507 to 1547510, EPI_ISL_1547514, EPI_ISL_1547517, EPI_ISL_1547533 to 1547543, EPI_ISL_1547546, EPI_ISL_1547584, EPI_ISL_1547589 to 1547592, EPI_ISL_1547602, EPI_ISL_1547604, EPI_ISL_1547608, EPI_ISL_1547611, EPI_ISL_1547616, EPI_ISL_1547621, EPI_ISL_1547624 to 1547626, EPI_ISL_1547649 to 1547651, EPI_ISL_1547653 to 1547654, EPI_ISL_1547657 to 1547662			
see above	Akershus University Hospital, Department for Microbiology and Infectious Disease Control	Norwegian Institute of Public Health, Department of Virology	Atiya R Ali; Debech Nadia; Engebretsen Serina Beate; Garcia Llorente Ignacio; Hilde Elshaug; Hilde Synnøve Vollan; Hilde Vollan; Jon Bråte; Kamilla Heddeland Instefjord; Karoline Bragstad; Kathrine Stene-Johansen; Marie Paulsen Madsen; Olav Hungnes; Pedersen Benedikte Nevjen; Rasmus Riis Kopperud
EPI_ISL_480204	Akita City Public Health Center	Pathogen Genomics Center, National Institute of Infectious Diseases	Hajime Kamiya; Kentaro Itokawa; Koichi Ito; Makoto Kuroda; Masanori Hashino; Motoi Suzuki; Rina Tanaka; Tsuyoshi Sekizuka
EPI_ISL_1394096 to 1394100, EPI_ISL_1538592, EPI_ISL_1538857, EPI_ISL_1538928	Alab Laboratoria Sp. z o. o.	1. National Institute of Public Health - National Institute of Hygiene; 2. Eurofins Genomics Europe Sequencing GmbH	ECDC COVID-19 WGS support team; Eurofins Genomics Europe Sequencing Team; Gierczyki Rafa; Sadkowska-Todys Magorzata; Wokowicz Tomasz; Zacharczuk Katarzyna
EPI_ISL_509688	Alabama Department of Public Health Bureau of Clinical Laboratories	Pathogen Discovery, Respiratory Viruses Branch, Division of Viral Diseases, Centers for Disease Control and Prevention	Anna Uehara; Clinton Paden; Haibin Wang; Jing Zhang; Krista Queen; Suxiang Tong; Yan Li; Ying Tao
EPI_ISL_454607 to 454613, EPI_ISL_468357 to 468387, EPI_ISL_1477170, EPI_ISL_1477175, EPI_ISL_1477178, EPI_ISL_1477199, EPI_ISL_1477216, EPI_ISL_1477228, EPI_ISL_1477243 to 1477244, EPI_ISL_1477251 to 1477252, EPI_ISL_1477259 to 1477260, EPI_ISL_1477262, EPI_ISL_1477268 to 1477271, EPI_ISL_1477279, EPI_ISL_1477287 to 1477288, EPI_ISL_1477292 to 1477293, EPI_ISL_1477295, EPI_ISL_1477301, EPI_ISL_1477308 to 1477309, EPI_ISL_1477313, EPI_ISL_1477318, EPI_ISL_1477322, EPI_ISL_1477328 to 1477329, EPI_ISL_1477335, EPI_ISL_1477337, EPI_ISL_1477343, EPI_ISL_1477352, EPI_ISL_1477359, EPI_ISL_1477364, EPI_ISL_1477367, EPI_ISL_1477371, EPI_ISL_1477373, EPI_ISL_1477377, EPI_ISL_1477380, EPI_ISL_1477382, EPI_ISL_1477385, EPI_ISL_1477388, EPI_ISL_1477405 to 1477406, EPI_ISL_1477410 to 1477411, EPI_ISL_1477413 to 1477415, EPI_ISL_1477432 to 1477433, EPI_ISL_1477435, EPI_ISL_1477446 to 1477447, EPI_ISL_1477462, EPI_ISL_1477466, EPI_ISL_1477468, EPI_ISL_1477483, EPI_ISL_1477487, EPI_ISL_1477489, EPI_ISL_1477491, EPI_ISL_1477497, EPI_ISL_1477499, EPI_ISL_1477520, EPI_ISL_1477545 to 1477547, EPI_ISL_1477554, EPI_ISL_1477557, EPI_ISL_1477565, EPI_ISL_1477569, EPI_ISL_1477584, EPI_ISL_1477594 to 1477595, EPI_ISL_1477605, EPI_ISL_1477617, EPI_ISL_1477627, EPI_ISL_1477630, EPI_ISL_1477632, EPI_ISL_1477639, EPI_ISL_1477647, EPI_ISL_1477649, EPI_ISL_1477657, EPI_ISL_1477668 to 1477669, EPI_ISL_1477671 to 1477672, EPI_ISL_1477674, EPI_ISL_1477683 to 1477684, EPI_ISL_1477686, EPI_ISL_1477688, EPI_ISL_1477691, EPI_ISL_1477696, EPI_ISL_1477701 to 1477703, EPI_ISL_1477708, EPI_ISL_1477710, EPI_ISL_1477712, EPI_ISL_1477720, EPI_ISL_1477733, EPI_ISL_1477735, EPI_ISL_1477741, EPI_ISL_1477747, EPI_ISL_1477751, EPI_ISL_1477753 to 1477754, EPI_ISL_1477757 to 1477758, EPI_ISL_1477766, EPI_ISL_1477771 to 1477773, EPI_ISL_1477775, EPI_ISL_1477779, EPI_ISL_1477783 to 1477784, EPI_ISL_1477791, EPI_ISL_1477801, EPI_ISL_1477804 to 1477805, EPI_ISL_1477807, EPI_ISL_1477809, EPI_ISL_1477820, EPI_ISL_1477823 to 1477825, EPI_ISL_1477828, EPI_ISL_1477832, EPI_ISL_1477841, EPI_ISL_1477847, EPI_ISL_1477857 to 1477858, EPI_ISL_1477862, EPI_ISL_1477865, EPI_ISL_1477880, EPI_ISL_1477886 to 1477887, EPI_ISL_1477889, EPI_ISL_1477896, EPI_ISL_1477899 to 1477900, EPI_ISL_1477904 to 1477906, EPI_ISL_1477914 to 1477915, EPI_ISL_1477919, EPI_ISL_1477922, EPI_ISL_1477928, EPI_ISL_1477932 to 1477933, EPI_ISL_1477936, EPI_ISL_1477941 to 1477942, EPI_ISL_1477945, EPI_ISL_1477948			
see above	Alameda County Public Health Lab	Chan-Zuckerberg Biohub	CZB Cliahub Consortium
EPI_ISL_420303 to 420306, EPI_ISL_424346 to 424347, EPI_ISL_427619 to 427622, EPI_ISL_431013 to 431019, EPI_ISL_435441 to 435444, EPI_ISL_436464, EPI_ISL_437437, EPI_ISL_437513 to 437518, EPI_ISL_437873, EPI_ISL_476898 to 476899, EPI_ISL_492049 to 492063, EPI_ISL_492087, EPI_ISL_512135 to 512157, EPI_ISL_522396 to 522405, EPI_ISL_525758 to 525760, EPI_ISL_528485 to 528537, EPI_ISL_560554 to 560566, EPI_ISL_576111 to 576112, EPI_ISL_586243 to 586266, EPI_ISL_602559 to 602561, EPI_ISL_1483586 to 1483699, EPI_ISL_1529075 to 1529098			
see above	Alaska State Virology Laboratory	Alaska State Virology Laboratory	Bortz E; Chen; Chen J et al with Pathogenomics group Dagdag R; Chen, J.; DeRonde, S.; Deuling, H.; Drown DM; Elva House; George W; J; Jack Chen; Kovalenko A; Lisa Smith; Milton E; Ph.D.; Redlinger M; Stephanie DeRonde
EPI_ISL_1534281, EPI_ISL_1534336	Albertinia Clinic wc AAP	NHLS/UCT	Arash Iranzadeh; Bruna Galvao; Carolyn Williamson; Deelan Doolabh; Diana Hardie; Emmanuel SJ; Innocent Mudau; Kruger Marais; Lynn Tyers; Marvin Hsiao; Stephen Korsman; Tegally H; de Oliveira T
EPI_ISL_1433679 to 1433684, EPI_ISL_1433686, EPI_ISL_1433688 to 1433692, EPI_ISL_1433694 to 1433696, EPI_ISL_1433698 to 1433735	Alexianer DaKS GmbH (eigenes IT-Tochterunternehmen der Alexianer Krankenhäuser)	Robert Koch Institute	
EPI_ISL_1385807, EPI_ISL_1385811	Alfa Diagnostica LLC	ONCOGENE LLC	ONCOGENE LLC
EPI_ISL_1385804	Alfa Diagnostica, Republic of Moldova	ONCOGENE LLC	ONCOGENE LLC
EPI_ISL_1443683 to 1443698	Algemeen Medisch Labo	Labo Klinische Biologie, UZA	Basil Britto Xavier; Christine Lammens; Herman Goossens; Jasmine Coppens; Marie Le Mercier; Veerle Matheeussen
EPI_ISL_1416762 to 1416763, EPI_ISL_1416769 to 1416770, EPI_ISL_1416776, EPI_ISL_1416781, EPI_ISL_1416795 to 1416796, EPI_ISL_1416800, EPI_ISL_1416804, EPI_ISL_1455660, EPI_ISL_1455663			
see above	Algemeen Medisch Laboratorium (AML)	UAntwerp, Laboratory of Medical Microbiology	Basil Britto Xavier; Christine Lammens; Herman Goossens; Jasmine Coppens; Marie Le Mercier; Veerle Matheeussen
EPI_ISL_508156 to 508206	All india institute of Medical Sciences Rishikesh	National Institute of Biomedical Genomics	Amit Mangla; Arindam Maitra; Deepjyoti Kalita; Ravi Kant; Saumitra Das
EPI_ISL_511908 to 511922	All india institute of Medical Sciences Rishikesh	National Institute of Biomedical Genomics - DBT's PAN-INDIA 1000 SARS--CoV-2 RNA Genome Sequencing Consortium	Amit Mangla; Arindam Maitra; Deepjyoti Kalita; Ravi Kant; Saumitra Das
EPI_ISL_514647, EPI_ISL_514649, EPI_ISL_514651	Allina Health Laboratory	Minnesota Department of Health, Public Health Laboratory	Jacob Garfin; Matt Plumb; and Xiong Wang
EPI_ISL_1534288, EPI_ISL_1534371, EPI_ISL_1534384, EPI_ISL_1534454	Alma CDC wc AHC	NHLS/UCT	Arash Iranzadeh; Bruna Galvao; Carolyn Williamson; Deelan Doolabh; Diana Hardie; Emmanuel SJ; Innocent Mudau; Kruger Marais; Lynn Tyers; Marvin Hsiao; Stephen Korsman; Tegally H; de Oliveira T
EPI_ISL_528538	Alsafar	Alsafar	Andreas Henschel; Ernesto Damiani; Gihan Elsir Ahmed Daw Elbait; Guan Tay; Habiba Alsafar; Rifat; Samuel Feng
EPI_ISL_528686 to 528721	Alsafar - Khalifa University Abu Dhabi	Alsafar - Khalifa University Abu Dhabi	Andreas Henschel; Ernesto Damiani; Gihan Daw Elbait; Guan Tay; Habiba Alsafar; Rifat Hamoudi; Samuel Feng
EPI_ISL_1468667 to 1468670, EPI_ISL_1468673 to 1468675, EPI_ISL_1468680 to 1468682, EPI_ISL_1468684, EPI_ISL_1468688 to 1468689, EPI_ISL_1468692 to 1468693, EPI_ISL_1468695, EPI_ISL_1468697 to 1468699, EPI_ISL_1468701 to 1468702, EPI_ISL_1468704, EPI_ISL_1468706 to 1468708, EPI_ISL_1468710, EPI_ISL_1468712 to 1468716, EPI_ISL_1468718, EPI_ISL_1468720 to 1468721, EPI_ISL_1468723, EPI_ISL_1468726 to 1468729, EPI_ISL_1468732 to 1468734, EPI_ISL_1468737, EPI_ISL_1468740, EPI_ISL_1468748 to 1468749, EPI_ISL_1468752 to 1468753, EPI_ISL_1468763, EPI_ISL_1468765, EPI_ISL_1468767, EPI_ISL_1468769 to 1468773, EPI_ISL_1468776, EPI_ISL_1468778 to 1468780, EPI_ISL_1468783 to 1468788, EPI_ISL_1468790, EPI_ISL_1468792, EPI_ISL_1468795, EPI_ISL_1468797 to 1468798, EPI_ISL_1468800, EPI_ISL_1468802, EPI_ISL_1468804 to 1468809, EPI_ISL_1468811 to 1468813, EPI_ISL_1468820 to 1468828, EPI_ISL_1483053 to 1483054			
see above	Altius Institute	Seattle Flu Study	Alex Nguyen; Amanda Adler; Andrew Meuser; Barry R. Lutz; Benjamin Pelle; Caitlin R. Wolf; Chris D. Frazar; Clem Green; Daniel Bates; Deborah A. Nickerson; Elisabeth Brandstetter; Erica Ryke; Hannah Petersen; Helen Y. Chu; Jacob Rodriguez; Janet A. Englund; Jay Shendure; Jessica Halow; John Stamatoyannopoulos; Joshua Richards; Jover Lee; Julia Wald; Kairsten Fay; Kirsten Lacombe; Kneshay Harper; Lea M. Starita; Mark J. Rieder; Matt Hartman; Matthew Richardson; Matthew Thompson; Melissa Truong; Michael Boeckh; Michael Famulare; Misja Ilcisin; Muhammad Halimun; Olivia Waltner; Peter D. Han; Rebecca Bruders; Ryan Alexander; Sadie Patraw; Sofia Olsson; Stephanie DeBaun; Thomas R. Sibley; Tobias Ragoczy; Trevor Bedford; Truong Nguyen
EPI_ISL_1520155	Alverno Laboratories	Illinois Department of Public Health - Chicago Lab	Ira Heimler; Vineet K. Dhiman
EPI_ISL_515544, EPI_ISL_523984, EPI_ISL_523986	Ama Dr Jose Soares Hungria	Instituto Adolfo Lutz, Interdiciplinary Procedures Center, Strategic Laboratory	Claudia Regina Gonçalves; Claudio Tavares Sacchi; Erica Valessa Ramos Gomes
EPI_ISL_1534405	Amalienstein Clinic wc AMS	NHLS/UCT	Arash Iranzadeh; Bruna Galvao; Carolyn Williamson; Deelan Doolabh; Diana Hardie; Emmanuel SJ; Innocent Mudau; Kruger Marais; Lynn Tyers; Marvin Hsiao; Stephen Korsman; Tegally H; de Oliveira T
EPI_ISL_569864 to 569886	Amedeo di savoia	Crosetto lab, Karolinska Institutet, SciLifeLab	Anna Sapino; Luuk Harbers; Maria Grazia Milia; Michele Simonetti; Nicola Crosetto; Ning Zhang; Valeria Ghisetti
EPI_ISL_456656	American Type Culture Collection Inc. (ATCC)	American Type Culture Collection Inc. (ATCC)	A.M.; Benton, B.; Chu; D.K.W.; Flores, B.; Frank; J.S.M.; King; M. and Rashid, S.; M.A.; N.P.; Parker; Peiris; Puthuveetil; Riojas; S.P.
EPI_ISL_414648, EPI_ISL_416457	Andersen Lab, The Scripps Research Institute	Andersen Lab, The Scripps Research Institute	Catie Anderson; Emily Spender; Karthik Gangavarapu; Kristian Andersen; Laura Nicholson; Mark Zeller; Raphaelle Klitting; Refugio Robles-Sikisaka; Sarah Topol
EPI_ISL_429991, EPI_ISL_430016	Andersen lab at Scripps Research	Andersen lab at Scripps Research	SEARCH Alliance San Diego
EPI_ISL_450818	Aneby VC	The Public Health Agency of Sweden	Anna Risberg; Anna-Malin Linde; Karin Tegmark-Wisell; Ken Granath; Maria Lind Karlberg; Mia Brytting; Olov Svartstrom; Oskar Karlsson Lindsjo; Theresa Enkirch
EPI_ISL_1384637 to 1384640, EPI_ISL_1510085, EPI_ISL_1510089, EPI_ISL_1510091, EPI_ISL_1510099, EPI_ISL_1510161 to 1510191, EPI_ISL_1510197 to 1510201, EPI_ISL_1510205 to 1510206, EPI_ISL_1510230 to 1510255			
see above	Anteja laboratorija (UAB Diagnostikos laboratorija)	Vilnius University Hospital Santaros Klinikos, Center of	Daniel Naumovas; Dovile Ezerskyte; Gytis Dudas; Ingrida Olendraite; Laimonas Griskevicius; Ligita Raugaite; Mindaugas Stoskus; Monika Katenaite;

		Laboratory Medicine	Rimvydas Norvilas
EPI_ISL_582127 to 582132	Antwerp University Hospital	Institute of Tropical Medicine	Colin Anthony; Philippe Selhorst
EPI_ISL_1443004 to 1443020, EPI_ISL_1443022 to 1443023, EPI_ISL_1510651 to 1510652	Arcispedale Santa Maria Nuova, Autoimmunità, Allergologia e Biotecnologie Innovative	Istituto Zooprofilattico Sperimentale della Lombardia e dell'Emilia Romagna (IZSLER), Risk Analysis and Genomic Epidemiology Unit	Alessandro Zerbini; Erika Scaltriti; Ilaria Menozzi; Lucia Belloni; Marina Morganti; Stefania Croci; Stefano Pongolini
EPI_ISL_1386123 to 1386126, EPI_ISL_1386129 to 1386133, EPI_ISL_1386224 to 1386234	Area Biologia Molecolare Istituto Zooprofilattico Sperimentale della Sicilia	Area Biologia Molecolare Istituto Zooprofilattico Sperimentale della Sicilia	BRUNO Federica; BRUNO Gabriella; CASTELLI Germano; COLLURA Rosaria; PIAZZA Angela; REALE Stefano; SCIBETTA Silvia; VITALE Fabrizio
EPI_ISL_512663 to 512664, EPI_ISL_512669, EPI_ISL_527740, EPI_ISL_527749	Area De Salud Alajuela Norte - Clinica Dr. Marcial Rodriguez	Inciensa, Instituto Costarricense de Investigación y Enseñanza en Nutrición y Salud	Adriana Godinez & Melany Calderon; Claudio Soto-Garita; Estela Cordero; Francisco Duarte; Hebleen Porras
EPI_ISL_527756	Area De Salud Aserri	Inciensa, Instituto Costarricense de Investigación y Enseñanza en Nutrición y Salud	Adriana Godinez & Melany Calderon; Claudio Soto-Garita; Estela Cordero; Francisco Duarte; Hebleen Porras
EPI_ISL_512668, EPI_ISL_527745, EPI_ISL_527748, EPI_ISL_527751	Area De Salud Corredores	Inciensa, Instituto Costarricense de Investigación y Enseñanza en Nutrición y Salud	Adriana Godinez & Melany Calderon; Claudio Soto-Garita; Estela Cordero; Francisco Duarte; Hebleen Porras
EPI_ISL_512653	Area De Salud Desamparados 1 - Clinica Dr. Marcial Fallas [Grifo Alto/Desampara	Inciensa, Instituto Costarricense de Investigación y Enseñanza en Nutrición y Salud	Adriana Godinez & Melany Calderon; Claudio Soto-Garita; Estela Cordero; Francisco Duarte; Hebleen Porras
EPI_ISL_512659	Area De Salud Fortuna	Inciensa, Instituto Costarricense de Investigación y Enseñanza en Nutrición y Salud	Adriana Godinez & Melany Calderon; Claudio Soto-Garita; Estela Cordero; Francisco Duarte; Hebleen Porras
EPI_ISL_527757	Area De Salud Goicoechea 1	Inciensa, Instituto Costarricense de Investigación y Enseñanza en Nutrición y Salud	Adriana Godinez & Melany Calderon; Claudio Soto-Garita; Estela Cordero; Francisco Duarte; Hebleen Porras
EPI_ISL_512662, EPI_ISL_512666 to 512667, EPI_ISL_512671, EPI_ISL_527746 to 527747, EPI_ISL_527760	Area De Salud La Cruz	Inciensa, Instituto Costarricense de Investigación y Enseñanza en Nutrición y Salud	Adriana Godinez & Melany Calderon; Claudio Soto-Garita; Estela Cordero; Francisco Duarte; Hebleen Porras
EPI_ISL_512660 to 512661	Area De Salud Los Chiles	Inciensa, Instituto Costarricense de Investigación y Enseñanza en Nutrición y Salud	Adriana Godinez & Melany Calderon; Claudio Soto-Garita; Estela Cordero; Francisco Duarte; Hebleen Porras
EPI_ISL_512658	Area De Salud Orotina-San Mateo [Orotina/Alajuela]	Inciensa, Instituto Costarricense de Investigación y Enseñanza en Nutrición y Salud	Adriana Godinez & Melany Calderon; Claudio Soto-Garita; Estela Cordero; Francisco Duarte; Hebleen Porras
EPI_ISL_512656	Area De Salud Pavas (Coopesalud) [Pavas/San Jose]	Inciensa, Instituto Costarricense de Investigación y Enseñanza en Nutrición y Salud	Adriana Godinez & Melany Calderon; Claudio Soto-Garita; Estela Cordero; Francisco Duarte; Hebleen Porras
EPI_ISL_512657	Area De Salud Tibas-Uruca-Merced - Clinica Dr. Clorito Picado [Tibas/San Jose]	Inciensa, Instituto Costarricense de Investigación y Enseñanza en Nutrición y Salud	Adriana Godinez & Melany Calderon; Claudio Soto-Garita; Estela Cordero; Francisco Duarte; Hebleen Porras
EPI_ISL_491446	Area de Salud Alajuela Central	Inciensa, Instituto Costarricense de Investigación y Enseñanza en Nutrición y Salud	Adriana Godinez & Melany Calderon; Claudio Soto-Garita; Estela Cordero; Francisco Duarte; Hebleen Brenes
EPI_ISL_434533, EPI_ISL_434535, EPI_ISL_491449	Area de Salud Alajuela Sur	Inciensa, Instituto Costarricense de Investigación y Enseñanza en Nutrición y Salud	Adriana Godinez & Melany Calderon; Claudio Soto-Garita; Estela Cordero; Francisco Duarte; Hebleen Brenes; Hebleen Porras
EPI_ISL_491437, EPI_ISL_491444	Area de Salud Escazu (Coopesana)	Inciensa, Instituto Costarricense de Investigación y Enseñanza en Nutrición y Salud	Adriana Godinez & Melany Calderon; Claudio Soto-Garita; Estela Cordero; Francisco Duarte; Hebleen Brenes
EPI_ISL_491457	Area de Salud Los Santos	Inciensa, Instituto Costarricense de Investigación y Enseñanza en Nutrición y Salud	Adriana Godinez & Melany Calderon; Claudio Soto-Garita; Estela Cordero; Francisco Duarte; Hebleen Brenes
EPI_ISL_491445	Area de Salud Mata Redonda	Inciensa, Instituto Costarricense de Investigación y Enseñanza en Nutrición y Salud	Adriana Godinez & Melany Calderon; Claudio Soto-Garita; Estela Cordero; Francisco Duarte; Hebleen Brenes
EPI_ISL_434539	Area de Salud Orotina	Inciensa, Instituto Costarricense de Investigación y Enseñanza en Nutrición y Salud	Adriana Godinez & Melany Calderon; Claudio Soto-Garita; Estela Cordero; Francisco Duarte; Hebleen Porras
EPI_ISL_1483700	Area of Virology, Serology and Virology Division (SAVID), New South Wales Health Pathology Randwick	Area of Virology, Serology and Virology Division (SAVID), New South Wales Health Pathology Randwick	Au, J.; Bull, R.; Deveson, I.; Foster, C.; Jean, T.; Rawlinson, W.; Ruiz Silva, M.; Van Haal, S.
EPI_ISL_500596 to 500706, EPI_ISL_500717, EPI_ISL_509492 to 509523, EPI_ISL_527008 to 527064, EPI_ISL_527179 to 527180, EPI_ISL_527400, EPI_ISL_530334 to 530340, EPI_ISL_537286 to 537287, EPI_ISL_539327, EPI_ISL_569858, EPI_ISL_577597, EPI_ISL_577604, EPI_ISL_586571			
see above	Area of Virology, Serology and Virology Division (SAViD), New South Wales Health Pathology Randwick	Area of Virology, Serology and Virology Division (SAViD), New South Wales Health Pathology Randwick	Bull, R.; Deveson, I.; Rawlinson; Rawlinson, W.; Van Hal, S.; W
EPI_ISL_1406433, EPI_ISL_1406435, EPI_ISL_1424504 to 1424508, EPI_ISL_1494718 to 1494723, EPI_ISL_1516747 to 1516748, EPI_ISL_1543924 to 1543927	Area of Virology, Serology and Virology Division (SAViD), New South Wales Health Pathology Randwick	Virology Research Laboratory; Area of Virology, Serology and Virology Division (SAViD), New South Wales Health Pathology Randwick	Au, J.; Bull, R.; Deveson, I.; Foster, C.; Rawlinson, W.; Ruiz Silva, M.; Van Hal, S.; Wong, M.
EPI_ISL_406223	Arizona Department of Health Services	Pathogen Discovery, Respiratory Viruses Branch, Division of Viral Diseases, Centers for Disease Control and Prevention	Anna Uehara; Brett L. Whitaker; Brian Lynch; Clinton R. Paden; Janna' R. Murray; Jing Zhang; Krista Queen; Lijuan Wang; Senthil Kumar K. Sakthivel; Shifaq Kamili; Stephen Lindstrom; Susan I. Gerber; Suxiang Tong; Xiaoyan Lu; Yan Li; Ying Tao
EPI_ISL_1443779 to 1443801, EPI_ISL_1447063 to 1447073, EPI_ISL_1503299 to 1503308	Arizona State Public Health Laboratory	Arizona State Public Health Laboratory	Jessica Escobar; Katherine Fullerton; Linda Getsinger; Nobuko Fukushima; Stacy White; Trung Huynh; Victor Waddell
EPI_ISL_1402540, EPI_ISL_1403385, EPI_ISL_1405918 to 1405924, EPI_ISL_1423515, EPI_ISL_1423934 to 1423944, EPI_ISL_1423946 to 1423976, EPI_ISL_1483573 to 1483585, EPI_ISL_1532306 to 1532584			
see above	Arizona State University	Arizona State University	Efrem S. Lim; Emily A. Kaelin; Joshua LaBaer; Joy M. Blain; Kristina Buss; LaRinda A. Holland; Neal W Woodbury; Nicholas J. Mellor; Peter T. Skidmore; Rabia Maqsood; Valerie Harris; Vel Murugan
EPI_ISL_424668 to 424669, EPI_ISL_424671, EPI_ISL_467372 to 467373	Arizona State University Health Services	Arizona State University	Arvind Varsani; Bereket Estifanos; Brenda G. Hogue; Efrem S. Lim; Emily A. Kaelin; Jason Steel; LaRinda A. Holland; Lily I. Wu; Matthew Scotch; Nicholas J. Mellor; Peter T. Skidmore; Rabia Maqsood; Rolf U. Halden
EPI_ISL_1436622, EPI_ISL_1436624, EPI_ISL_1436626, EPI_ISL_1436628 to 1436629, EPI_ISL_1436631, EPI_ISL_1436633, EPI_ISL_1436635, EPI_ISL_1436637, EPI_ISL_1436639, EPI_ISL_1436641, EPI_ISL_1436643, EPI_ISL_1436645, EPI_ISL_1436647, EPI_ISL_1436649, EPI_ISL_1436651, EPI_ISL_1436653, EPI_ISL_1436656, EPI_ISL_1436658, EPI_ISL_1436660, EPI_ISL_1436662, EPI_ISL_1436664, EPI_ISL_1436666, EPI_ISL_1436668, EPI_ISL_1436670, EPI_ISL_1436673, EPI_ISL_1436831, EPI_ISL_1436833, EPI_ISL_1436835, EPI_ISL_1436838, EPI_ISL_1436840, EPI_ISL_1436842, EPI_ISL_1436844, EPI_ISL_1436846, EPI_ISL_1436848, EPI_ISL_1436861, EPI_ISL_1436864, EPI_ISL_1436866, EPI_ISL_1436868, EPI_ISL_1436870, EPI_ISL_1436872, EPI_ISL_1436874, EPI_ISL_1436876, EPI_ISL_1436878, EPI_ISL_1436880, EPI_ISL_1436883, EPI_ISL_1436885 to 1436886, EPI_ISL_1436888, EPI_ISL_1436891 to 1436892, EPI_ISL_1436895, EPI_ISL_1436897, EPI_ISL_1436899, EPI_ISL_1437041, EPI_ISL_1437690, EPI_ISL_1437692, EPI_ISL_1437694, EPI_ISL_1437696, EPI_ISL_1437698, EPI_ISL_1437713, EPI_ISL_1437718, EPI_ISL_1437720, EPI_ISL_1437729, EPI_ISL_1437734, EPI_ISL_1437736, EPI_ISL_1438441, EPI_ISL_1559592 to 1559611			
see above	Arlon	Plateforme de testing Namuroise	; Céline Maschietto; Degosserie Jonathan; Denis Olivier; Mullier François; Otto Gaetan
EPI_ISL_496518 to 496520, EPI_ISL_496529, EPI_ISL_496533, EPI_ISL_496537 to 496545, EPI_ISL_496602, EPI_ISL_497880 to 497887	Armed Forces Medical College	National Centre For Cell Science	Arvind Sahu; DBT's PAN-INDIA 1000 SARS-CoV2 RNA genome sequencing consortium; Dhiraj Paul; Girdhari Lal; Janesh Kumar; Kavita Bala Anand; Kunal Jani; Maharashtra COVID-19 Study Group; Manoj Kumar Bhat; Murlidhar Tambe; Radha Chauhan; Rajesh Karyakarte; Rajiv Mohan Gupta; Santosh Karade; Shelinder Pal Singh Shergill; Sourav Sen; Suvarna Joshi; Vasudevan Seshadri; Yogesh S Shouche
EPI_ISL_1389311, EPI_ISL_1465611 to 1465614, EPI_ISL_1516923 to 1516924, EPI_ISL_1517089, EPI_ISL_1517091 to 1517092, EPI_ISL_1517094, EPI_ISL_1517464 to 1517467, EPI_ISL_1517479 to 1517482, EPI_ISL_1528183 to 1528185, EPI_ISL_1528199 to 1528201, EPI_ISL_1528218, EPI_ISL_1528235			
see above	Armies	National Reference Center for Viruses of Respiratory	Amaury Vaysse; Angela Brisebarre; Camille Capel; Christophe Malabat; Corinne Maufrais; Damien Mornico; Desroches Marine; Etienne Simon-Lorière;

		Infections, Institut Pasteur, Paris	Frédéric Lemoine; Louise Lefrançois; Marine Desroches; Marion Barbet; Maud Vanpeene; Méline Bizard; Sylvie Behillil; Sylvie van der Werf; Vincent Enouf
EPI_ISL_457826	Army Medical Center - Scientific Department	Army Medical and Veterinary Research Center	Anna Anselmo; Antonella Fortunato; Florigio Lista; Francesco Giordani; Giovanni Faggioni; Nino D'Amore; Riccardo De Sanctis; Silvia Fillo; Vanessa Vera Fain
EPI_ISL_457825	Army Medical Research Center - Scientific Department	Army Medical and Veterinary Research Center	Anna Anselmo; Antonella Fortunato; Florigio Lista; Francesco Giordani; Giovanni Faggioni; Nino D'Amore; Riccardo De Sanctis; Silvia Fillo; Vanessa Vera Fain
EPI_ISL_539809 to 539812, EPI_ISL_539821	Asiaworld Expo Command Post	Hong Kong Department of Health	Alan K.L. Tsang; Dominic N.C. Tsang; Edman T.K. Lam; Peter C.W. Yip; Rickjason C.W. Chan
EPI_ISL_1524868	Assistenza Domiciliare Integrata (ADI) TERAMO TERAMO(TERAMO)	Istituto Zooprofilattico Sperimentale dell'Abruzzo e Molise "G. Caporale"	Ancora M; Calistri P; Cammà C; Caporale M; Curini V; Delli Compagni E; Di Domenico M; Di Lollo Valeria; Di Pasquale A; Lorusso A; Mangone I; Marcacci M; Puglia I; Rinaldi A; Savini G; Scialabba S
EPI_ISL_1498263, EPI_ISL_1498585, EPI_ISL_1498821 to 1498825, EPI_ISL_1498916, EPI_ISL_1498919, EPI_ISL_1499020, EPI_ISL_1499105, EPI_ISL_1499114, EPI_ISL_1499201 to 1499205, EPI_ISL_1499297 to 1499300			
see above	Associação Fundo de Incentivo à Pesquisa (AFIP)	Associação Fundo de Incentivo à Pesquisa (AFIP)	Debora R. Ramadan; Erika Rodrigues de Oliveira; Juliana Nogueira Martins Rodrigues; Priscila Farias Tempaku; Sergio Tufik.; Soraya Sgambatti de Andrade
EPI_ISL_1498380	Associação Fundo de Incentivo à Pesquisa (AFIP).	Associação Fundo de Incentivo à Pesquisa (AFIP).	Debora R. Ramadan; Erika Rodrigues de Oliveira; Juliana Nogueira Martins Rodrigues; Priscila Farias Tempaku; Sergio Tufik.; Soraya Sgambatti de Andrade
EPI_ISL_1533436, EPI_ISL_1533443 to 1533444, EPI_ISL_1533446 to 1533447, EPI_ISL_1533449 to 1533450, EPI_ISL_1533452 to 1533530, EPI_ISL_1533539 to 1533563, EPI_ISL_1559385			
see above	Atlanta VA Medical Center	Genomics and Discovery, Respiratory Viruses Branch, Division of Viral Diseases, Centers for Disease Control and Prevention	Adam Retchless; Anna Kelleher; Anna Montmayeur; Anna Uehara; Brian Lynch; Clinton R. Paden; Haibin Wang; Han Jia Justin Ng; Jing Zhang; Justin Lee; Krista Queen; Mark Burroughs; Peter Cook; Rachel Marine; Suxiang Tong; Yan Li; Ying Tao
EPI_ISL_413490, EPI_ISL_416519, EPI_ISL_416526	Auckland Hospital	Institute of Environmental Science and Research (ESR)	Erasmus Smit; Gary McAuliffe; Joep de Ligt; Lauren Jelly; Matt Storey; Matthew Blakiston; Sally Roberts; Xiaoyun Ren
EPI_ISL_569616	Aurora County Clinic	South Dakota Public Health Laboratory	Jacob Garfin; Matt Plumb; Xiong Wang; and Chris Carlson
EPI_ISL_513340, EPI_ISL_526166 to 526169, EPI_ISL_593649	Austech Medical Laboratories	NSW Health Pathology - Institute of Clinical Pathology and Medical Research; Westmead Hospital; University of Sydney	CIDM-PH et al.
EPI_ISL_451486, EPI_ISL_451574, EPI_ISL_451599, EPI_ISL_513331, EPI_ISL_544966 to 544967, EPI_ISL_544988, EPI_ISL_545024, EPI_ISL_547597, EPI_ISL_591484			
see above	Australian Clinical Labs	NSW Health Pathology - Institute of Clinical Pathology and Medical Research; Westmead Hospital; University of Sydney	CIDM-PH et al.
EPI_ISL_526212	Australian Clinical Labs (formerly Healthscope Pathology)	NSW Health Pathology - Institute of Clinical Pathology and Medical Research; Westmead Hospital; University of Sydney	CIDM-PH et al.
EPI_ISL_475830 to 475886, EPI_ISL_583573 to 583578, EPI_ISL_583630 to 583690, EPI_ISL_583883 to 583892, EPI_ISL_1495407 to 1495465, EPI_ISL_1495627 to 1495718	Austrian Agency for Health and Food Safety (AGES)	Bergthaler laboratory, CeMM Research Center for Molecular Medicine of the Austrian Academy of Sciences	Adi Steinrigl; Alexander Lercher; Alexandra Popa; Andreas Bergthaler; Anna Schedl; Bekir Erguner; Benedikt Agerer; Christian Paar; Christoph Bock; Daniela Schmid; Dorothee von Laer; Elisabeth Puchhammer-Stoeckl; Fabian Amman; Franz Allerberger; Gernot Walder; Gregor Hörmann; Guenter Weiss; Gunther Vogl; Henrique Colaco; Jakob-Wendelin Genger; Jan Laine; Judith Aberle; Kinga Rigler-Hohenwarter; Lukas Endler; Maelle Le Moing; Manfred Nairz; Mark Smyth; Martin Senekowitsch; Michael Schuster; Peter Hufnagl; Peter Obrist; Petr Triska; Rainer Gattringer; Sabine Sussitz-Rack; Stephan Aberle; Thomas Penz; Wegene Borena
EPI_ISL_569610, EPI_ISL_569624	Avera McKennan Hospital	South Dakota Public Health Laboratory	Jacob Garfin; Matt Plumb; Xiong Wang; and Chris Carlson
EPI_ISL_507963 to 507972, EPI_ISL_514645	Avera Mckennan Laboratory	Minnesota Department of Health, Public Health Laboratory	Jacob Garfin; Matt Plumb; and Xiong Wang
EPI_ISL_1524739	Azienda Ospedaliera San Camillo Forlanini	INMI Lazzaro Spallanzani IRCCS	A D'Agostino; A Di Caro; B Bartolini; CEM Gruber; E Giombini; F Basile; F Messina; F Santini; G Bonfiglio; G Parisi; M Rueca; MR Capobianchi; O Butera
EPI_ISL_1492573, EPI_ISL_1496001	Azienda Ospedaliera San Giovanni Addolorata	INMI Lazzaro Spallanzani IRCCS	A Di Caro; B Bartolini; CEM Gruber; E Giombini; F Messina; F Santini; G Bonfiglio; M Gaudio; M Rueca; MR Capobianchi; O Butera; PM Placanica
EPI_ISL_1390769, EPI_ISL_1394744 to 1394748, EPI_ISL_1499474 to 1499503, EPI_ISL_1499621 to 1499627, EPI_ISL_1524709 to 1524710	Azienda Ospedaliera Terni	Istituto Zooprofilattico Sperimentale dell'Abruzzo e Molise "G. Caporale"	Ancora M; Calistri P; Cammà C; Caporale M; Curini V; Delli Compagni E; Di Domenico M; Di Lollo V; Di Lollo Valeria; Di Pasquale A; Lorusso A; Mangone I; Marcacci M; Palumbo M; Puglia I; Rinaldi A; Savini G; Scaccetti A; Scialabba S
EPI_ISL_1524660 to 1524663	Azienda Ospedaliera Universitaria Integrata Verona	Istituto Zooprofilattico Sperimentale delle Venezie	Adelaide Milani; Alessia Schivo; Alice Fusaro; Ambra Pastori; Annalisa Salviato; Antonia Ricci; Calogero Terregino; Edoardo Giussani; Elisa Palumbo; Erika Giorgia Quaranta; Isabella Monne; Luca Tassoni
EPI_ISL_1524498 to 1524506	Azienda Ospedaliera Universitaria Integrata Verona	Istituto di Genomica Applicata; Istituto Zooprofilattico Sperimentale delle Venezie	Alessia Schivo; Alice Fusaro; Ambra Pastori; Annalisa Salviato; Antonia Ricci; Calogero Terregino; Davide Scaglione; Edoardo Giussani; Eleonora Paparelli; Erika Giorgia Quaranta; Francesca Bruno; Gabriele Magris; Irena Jurman; Isabella Monne; Luca Tassoni; Maria Varotto; Michele Morgante; Silvia Ormelli; Valeria D'Amico; Vera Vendramin
EPI_ISL_1442147, EPI_ISL_1442149, EPI_ISL_1442151, EPI_ISL_1442153, EPI_ISL_1442155, EPI_ISL_1442162, EPI_ISL_1442166, EPI_ISL_1442169, EPI_ISL_1442174, EPI_ISL_1442179, EPI_ISL_1442185, EPI_ISL_1442187, EPI_ISL_1442190, EPI_ISL_1442192, EPI_ISL_1442194, EPI_ISL_1442196, EPI_ISL_1442198, EPI_ISL_1442200, EPI_ISL_1442202, EPI_ISL_1442207, EPI_ISL_1442216			
see above	Azienda Ospedaliero - Universitaria di Modena Policlinico - Virologia e Microbiologia Molecolare	Istituto Zooprofilattico Sperimentale della Lombardia e dell'Emilia Romagna (IZSLER), Risk Analysis and Genomic Epidemiology Unit	Erika Scaltriti; Giulia Fregni Serpini; Ilaria Menozzi; Marina Morganti; Monica Pecorari; Stefano Pongolini; William Gennari
EPI_ISL_1442426, EPI_ISL_1442433, EPI_ISL_1442438, EPI_ISL_1442440, EPI_ISL_1442442, EPI_ISL_1442445, EPI_ISL_1442447 to 1442448, EPI_ISL_1442451, EPI_ISL_1442453, EPI_ISL_1442455, EPI_ISL_1442462			
see above	Azienda Sanitaria Locale di Piacenza - Presidio Ospedaliero - Laboratorio di Microbiologia	Istituto Zooprofilattico Sperimentale della Lombardia e dell'Emilia Romagna (IZSLER), Risk Analysis and Genomic Epidemiology Unit	Erika Scaltriti; Ilaria Menozzi; Lo Cascio Giuliana; Marina Morganti; Peroni Irene; Schiavo Roberta; Stefano Pongolini
EPI_ISL_1386112, EPI_ISL_1386115 to 1386117	Azienda Sanitaria Locale di Piacenza - Presidio Ospedaliero - Laboratorio di Microbiologia	U.O. Microbiologia, Laboratorio Unico Centro Servizi - AUSL della Romagna	Giorgio Dirani; Lo Cascio Giuliana; Schiavo Roberta; Silvia Zannoli; Vittorio Sambri
EPI_ISL_1489740 to 1489741, EPI_ISL_1489743 to 1489757, EPI_ISL_1489759 to 1489834, EPI_ISL_1523544 to 1523583, EPI_ISL_1523585 to 1523639, EPI_ISL_1545669 to 1545673, EPI_ISL_1545675 to 1545703, EPI_ISL_1545705 to 1545763			
see above	Azienda Sanitaria dell'Alto Adige Laboratorio Aziendale di Microbiologia e Virologia	Istituto di Genomica Applicata	Davide Scaglione; Eleonora Paparelli; Elisa Masi; Elisabetta Giacobazzi; Elisabetta Pagani; Gabriele Magris; Irena Jurman; Irene Bianconi; Michele Morgante; Stefanie Wieser; Vera Vendramin
EPI_ISL_1394739 to 1394743	Azienda USL Umbria 2	Istituto Zooprofilattico Sperimentale dell'Abruzzo e Molise "G. Caporale"	Ancora M; Calistri P; Cammà C; Caporale M; Curini V; Di Domenico M; Di Lollo Valeria; Di Pasquale A; Lorusso A; Mangone I; Marcacci M; Pistoni E; Proietti A; Puglia I; Rinaldi A; Savini G; Scialabba S
EPI_ISL_496521 to 496523, EPI_ISL_496527 to 496528, EPI_ISL_496530, EPI_ISL_496534, EPI_ISL_496546 to 496554, EPI_ISL_497888 to 497891	B.J. Govt. Medical College	National Centre For Cell Science	Arvind Sahu; DBT's PAN-INDIA 1000 SARS-CoV2 RNA genome sequencing consortium; Dhiraj Paul; Girdhari Lal; Janesh Kumar; Kavita Bala Anand; Kunal Jani; Maharashtra COVID-19 Study Group; Manoj Kumar Bhat; Murlidhar Tambe; Radha Chauhan; Rajesh Karyakarte; Rajiv Mohan Gupta; Santosh Karade; Shelinder Pal Singh Shergill; Sourav Sen; Suvarna Joshi; Vasudevan Seshadri; Yogesh S Shouche
EPI_ISL_435049 to 435054, EPI_ISL_437445 to 437454, EPI_ISL_444456 to 444481, EPI_ISL_447030 to 447046, EPI_ISL_458086 to 458102, EPI_ISL_461483 to 461506, EPI_ISL_467041 to 467054, EPI_ISL_469024 to 469028, EPI_ISL_495014 to 495019			
see above	B.J. Medical College and Civil hospital	Gujarat Biotechnology Research Centre	; A M Kadri; Afzal Ansari; Akanksha Verma; Amit Kanani; Anjali Rajwar; Ankit Hinsu; Apurvasinh Puvar; Armi Chaudhari; Bhavesh Modi; Bhavya Jindal; Binita Aring; Camellia Chakraborty; Chaitanya Joshi; Dhaval Vaghela; Dinesh Kumar; Dipa Kinariwala; Dipeshwari Shewale; Disha Patel; Fenil Patel; Gaurishankar Shrimali; Geeta Vaghela; Harsh Bakshi; Janvi Raval; Kairavi Joshi; Kamlesh J Upadhyay; Komal Patel; Labdhi Pandya; Madhvi Joshi; Maharshi Pandya; Monika Gandhi; Neelam Nathani; Neeta Khandelwal; Neha Rajpara; Nidhi Patel; Nidhi Sood; Nikha Trivedi; Nirav Mungalpara; Nitin

			Savaliya; Pinal Trivedi; Pooja P Doshi; Pragya Sharma; Pranay Shah; Pritesh Sabara; Priti Pandita; Priyanka P Vatsa; R D Dixit; Raghawendra Kumar; Ramesh Pandit; Ramesh Patel; Sanjay Kapadia; Sharmistha Majumdar; Siddhant Kumar; Snehal Bagatharia; Sonia Barve; Tejas Shah; Umang Mishra; Vasudha Sharma; Zarna Patel; Zuber Saiyed
EPI_ISL_512058 to 512065, EPI_ISL_514581 to 514609, EPI_ISL_524713 to 524729, EPI_ISL_525421 to 525422, EPI_ISL_586513 to 586530, EPI_ISL_590689, EPI_ISL_1544110 to 1544117			
see above	B.J. Medical College and Civil hospital, Ahmedabad	Gujarat Biotechnology Research Centre	A M Kadri; Afzal Ansari; Apurvasinh Puvar; Chaitanya Joshi; Dinesh Kumar; Dipa Kinariwala; Harsh Bakshi; Janvi Raval; Kamlesh J Upadhyay; Komal Patel; Labdhi Pandya; Madhvi Joshi; Maharshi Pandya; Monika Gandhi; Nidhi Patel; Nikha Trivedi; Nitesh Shah; Nitin Savaliya; Pinal Trivedi; Pranay Shah; R D Dixit; Raghawendra Kumar; Ramesh Pandit; Sanjay Kapadia; Sonal Sharma; Twinkle Soni; Umang Mishra; Zarna Patel; Zuber Saiyed
EPI_ISL_1385820	BANGALORE MEDICAL COLLEGE AND RESEARCH INSTITUTE	INSACOG-KA, NIMHANS	Anita S Desai; Anson Kunjumon George; Chitra Pattabiraman; Darshan Sreenivas; Nakka Vijay Kiran Reddy; Pramada Prasad; V Ravi
EPI_ISL_1534704 to 1534705	BBLK Surabaya	National Institute of Health Research and Development	Arie Ardiansyah Nugraha; Hana Apsari Pawestri; Hartanti Dian Ikawati; Kartika Dewi Puspa; Krisna Pangesti; Nelly Puspandari; Subangkit; Vivi Setiawaty
EPI_ISL_1385817 to 1385819, EPI_ISL_1385821 to 1385824, EPI_ISL_1385826 to 1385828, EPI_ISL_1385837 to 1385838, EPI_ISL_1396654	BBMP Urban PHC	INSACOG-KA, NIMHANS	Anita S Desai; Anson Kunjumon George; Chitra Pattabiraman; Darshan Sreenivas; Nakka Vijay Kiran Reddy; Pramada Prasad; V Ravi
EPI_ISL_1469239	BBTKL PP Jakarta	National Institute of Health Research and Development	Arie Ardiansyah Nugraha; Hana Apsari Pawestri; Hartanti Dian Ikawati; Kartika Dewi Puspa; Krisna Pangesti; Nelly Puspandari; Subangkit; Vivi Setiawaty
EPI_ISL_412965, EPI_ISL_415577 to 415590, EPI_ISL_418816 to 418859	BCCDC Public Health Laboratory	BCCDC Public Health Laboratory	Choi; Gilmour; Harrigan; Hoang; Kamelian; Krajden; Lapointe; Lee; Levett; Li; Loman; Prystajecky; Quick; Sekirov; Snutch; Tyson
EPI_ISL_486395, EPI_ISL_515933, EPI_ISL_515937, EPI_ISL_516075	BIMS	Department of Neurovirology, National Institute of Mental Health and Neuroscience (NIMHANS)	Anita Desai; Chitra Pattabiraman; Harsha PK; Manjunatha Venkataswamy; Pramada Prasad; Ravi Vasanthapuram; Risha Rasheed; Shafeeq S Hameed; Vijayalakshmi Reddy
EPI_ISL_1397694 to 1397699, EPI_ISL_1397912 to 1397913	BIO-REFERENCE LABORATORIES	Wadsworth Center, New York State Department of Health	Alexis Russel; Daryl M. Lamson; Erasmus Schneider; Erica Lasek-Nesselquist; John Kelly; Jonathan Plitnick; Kirsten St. George; Matthew Shudt; Melissa A Leisner; Navjot Singh
EPI_ISL_1418249 to 1418251, EPI_ISL_1461078, EPI_ISL_1461080, EPI_ISL_1461082, EPI_ISL_1461085, EPI_ISL_1461087, EPI_ISL_1461089, EPI_ISL_1461091 to 1461092, EPI_ISL_1461094, EPI_ISL_1461096, EPI_ISL_1461098, EPI_ISL_1461100 to 1461101, EPI_ISL_1461103, EPI_ISL_1461105, EPI_ISL_1461107 to 1461108			
see above	BIOMNIS LYON	CNR Virus des Infections Respiratoires - France SUD	Antonin Bal; Bruno Lina; Gregory Destras; Gwendolyne Burfin; Hadrien Regue; Laurence Josset; Martine Valette; Quentin Semanas
EPI_ISL_1396844 to 1396882	BIOPATH	Department of Virology, Henri Mondor University Hospital, Assistance Publique Hôpitaux de Paris, Université Paris-Est Créteil, INSERM U955	Alexandre Soulier; Christophe Rodriguez; Elisabeth Trawinski; Guillaume Gricourt; Jean-Michel Pawlotsky; Melissa N'Debi; Slim Fourati; Vanessa Demontant
EPI_ISL_1461124 to 1461130, EPI_ISL_1461132 to 1461134	BIOSELLAL	CNR Virus des Infections Respiratoires - France SUD	Antonin Bal; Bruno Lina; Gregory Destras; Gwendolyne Burfin; Hadrien Regue; Laurence Josset; Martine Valette; Quentin Semanas
EPI_ISL_1482408 to 1482409, EPI_ISL_1482446 to 1482453	BOSTON HEART DIAGNOSTICS CORP	Wadsworth Center, New York State Department of Health	Alexis Russell; Catharine Prussing; Daryl M. Lamson; Erasmus Schneider; Erica Lasek-Nesselquist; John Kelly; Jonathan Plitnick; Kirsten St. George; Matthew Shudt; Melissa A Leisner; Navjot Singh
EPI_ISL_500716	BSL3 Lab Pendik Veterinary Control Institute	Department of Medicinal Genetics, Bursa Uluda University, Faculty of medicine By Sehime Gülsün Temel, Adem Alemdar, Kadir Yeilba	Ahmet SAIT; Cumhur ADIAY; Fahriye SARAC; Hakan ENUL; Kadir YESILBAG; Mustafa HASOKSUZ; Oguz KARABEY; Orbay SAYI; Osman ERGANIS; Serdar UZAR
EPI_ISL_491476	BSL3 Lab, Pendik Veterinary Control Enstitue	Genomic Laboratory (GLAB), Istanbul Technical University	Ahmet SAIT; Cumhur ADIAY; Fahriye SARAC; Hakan ENUL; Kadir YESILBAG; Mustafa HASOKSUZ; Oguz KARABEY; Orbay SAYI; Osman ERGANIS; Serdar UZAR
EPI_ISL_574619 to 574623	BTKLPP Kelas I Manado	Eijkman Institute for Molecular Biology, Ministry of Research and Technology/National Agency for Research and Innovation	Amin Soebandrio; David H Muljono; Edison Johar; Frilasita A Yudhaputri; Herawati Sudoyo; Hidayat Trimarsanto; Iskandar A Adnan; Khin Saw Myint; Safarina G Malik; Willy Agustine
EPI_ISL_1534690 to 1534696	BTKLPP kelas 1 Batam	National Institute of Health Research and Development	Arie Ardiansyah Nugraha; Budi Santosa; Hana Apsari Pawestri; Hartanti Dian Ikawati; Ida Neni Haryanti; Ismail; Kartika Dewi Puspa; Krisna Pangesti; Nelly Puspandari; Subangkit; Vivi Setiawaty
EPI_ISL_445362	BUPA SERVICIOS CLINICOS S.A	Instituto de Salud Publica de Chile	Alejandra Acevedo; Andrés E Castillo; Bárbara Parra; Carolina Tambley; Gabriel Leal; Jaime Lagos; Jorge Fernandez; Loredana Arata; Patricia Bustos; Paz Tapia; Rodrigo Fasce; Winston Andrade
EPI_ISL_538506 to 538507, EPI_ISL_538512	Balai Penelitian dan Pengembangan Biomedis Papua	National Institute of Health Research and Development	A; AA; HA; HD; HML; Hutapea; Ikawati; KD; KNA; M; Nugraha; Oktavian; Paisal; Pangesti; Pasaribu; Pawestri; Puspa; Setiawaty, V.; Soekarso; Subangkit; T
EPI_ISL_1394766, EPI_ISL_1394768, EPI_ISL_1394778, EPI_ISL_1394791, EPI_ISL_1394810, EPI_ISL_1394815, EPI_ISL_1394819, EPI_ISL_1394823, EPI_ISL_1394830, EPI_ISL_1540543, EPI_ISL_1540548, EPI_ISL_1540551, EPI_ISL_1540560, EPI_ISL_1540562 to 1540563, EPI_ISL_1540570 to 1540572, EPI_ISL_1540582			
see above	Baltic Medics	Lithuanian University of Health Sciences Hospital, Department of Genetics and Molecular Medicine	Astra Vitkauskiene; Darius Cereskevicius; Inga Nasvytiene; Mantas Sarauskas; Marius Sukys; Rasa Ugenskiene; Renaldas Jurkevicius; Rima Vainoriene; Zivile Zemeckiene
EPI_ISL_403963	Bamrasnaradura Hospital	1. Department of Medical Sciences, Ministry of Public Health, Thailand 2. Thai Red Cross Emerging Infectious Diseases - Health Science Centre 3. Department of Disease Control, Ministry of Public Health, Thailand	Buathong; Chittaganpitch; Malinee; Mekha; Nanthawan; Okada; Parnmen; Phuygun; Pilailuk; Rome; Siripaporn; Sittiporn; Sunthareeya; Supaporn; Thanadachakul; Thanutsapa; Wacharapluesadee; Waicharoen; Warawan; Wongboot
EPI_ISL_434693 to 434694, EPI_ISL_434696	Bamrasnaradura hospital	National Institute of Health. Department of medical Sciences, Ministry of Public Health, Thailand	Chittaganpitch; Malinee; Okada; Parnmen; Phuygun; Pilailuk; Siripaporn; Sittiporn; Sunthareeya; Thanadachakul; Thanutsapa; Waicharoen; Warawan; Wongboot
EPI_ISL_469048, EPI_ISL_475026 to 475029, EPI_ISL_476867 to 476868	Banas Medical College and Research Institute	Gujarat Biotechnology Research Centre	A M Kadri; Afzal Ansari; Ankit Hinsu; Apurvasinh Puvar; Chaitanya Joshi; Dinesh Kumar; Fenil Patel; Harsh Bakshi; Janvi Raval; Komal Patel; Labdhi Pandya; Madhvi Joshi; Maharshi Pandya; Monika Gandhi; Neelam Nathani; Neha Rajpara; Nidhi Patel; Nikha Trivedi; Nitin Savaliya; Pinal Trivedi; Pritesh Sabara; Priti Pandita; R D Dixit; Radhika Khara; Raghawendra Kumar; Snehal Bagatharia; Sunil R Joshi; Tejas Shah; Viren s Doshi; Zarna Patel; Zuber Saiyed
EPI_ISL_450339 to 450345	Bangladesh Institute of Tropical & Infectious Diseases, COVID-19 Testing Laboratory	Basic and Applied Research on Jute Project	A S M Anwarul Huq; AMAM Zonaed Siddiki; Eaftekhar Ahmed Rana; Emdadul Mannan Emdad; Goutam Buddha Das; M A Hassan Chowdhury; Md. Monjurul Alam; Md. Nazmul Haq Rony; Md. Sabbir Hossain; Md. Samiul Haque; Md. Shahidul Islam; Md. Shakeel Ahmed; Md. Sharifur Rahman; Paritous Kumar Biswas; Rasel Ahmed; Shah Md Tamim Kabir
EPI_ISL_539886	Barnakuten	The Public Health Agency of Sweden	Anna Risberg; Anna-Malin Linde; Karin Tegmark-Wisell; Maria Lind Karlberg; Mattias Haukland; Mia Brytting; Olov Svartstrom; Oskar Karlsson Lindsjo; Reza Advani; Sandra Broddesson; Theresa Enkirch
EPI_ISL_1391410	Barts Health NHS Trust	Barts Health NHS TRust	BROAD; Beatrix; CUTINO-MOGUEL; Claire; David; Dola; Esin; HARRINGTON; KARAA; KELE; KULASEGARAN-SHYLINI; Maria-Teresa; OWOYEMI; Raghavendran
EPI_ISL_1391409, EPI_ISL_1391411 to 1391412, EPI_ISL_1391414	Barts Health NHS Trust	Barts Health NHS Trust	BROAD; Beatrix; CUTINO-MOGUEL; Claire; David; Dola; Esin; HARRINGTON; KARAA; KELE; KULASEGARAN-SHYLINI; Maria-Teresa; OWOYEMI; Raghavendran
EPI_ISL_1474801 to 1474828	Barts Health NHS Trust	COVID-19 Genomics UK (COG-UK) Consortium	BROAD; Beatrix; CUTINO-MOGUEL; Claire; David; Dola; HARRINGTON; KELE; KULASEGARAN-SHYLINI; Maria-Teresa; OWOYEMI; Raghavendran
EPI_ISL_541752, EPI_ISL_541754 to 541755, EPI_ISL_541757 to 541766, EPI_ISL_541768 to 541772, EPI_ISL_541775 to 541783	Barts Health NHS Trust	Wellcome Sanger Institute for the COVID-19 Genomics UK (COG-UK) consortium	Beatrix Kele; Cordelia Langford; David Harrington and Alex Alderton; David K. Jackson; Dominic Kwiatkowski; Ewan Harrison; Ian Johnston; John Sillitoe on behalf of the Wellcome Sanger Institute COVID-19 Surveillance Team; Mark Hopkins; Roberto Amato; Sonia Goncalves; Teresa Cutino-Moguel
EPI_ISL_1538414	Basurto University Hospital	Biocruces	Ana de la Hoz; Estibaliz Ugalde Zarraga; José Luís Díaz de Tuesta del Arco; Mikel Gallego Rodrigo; Mikel Urrutikoetxea-Gutierrez; Mª Carmen Nieto Toboso

EPI_ISL_1538413	Basurto University Hospital Microbiology Lab	Biocruces	Ana de la Hoz; Estibalez Ugalde Zarrraga; Jose Luis Díaz de Tuesta del Arco; Mikel Gallego Rodrigo; Mikel Urrutikoetxea-Gutierrez; Mª Carmen Nieto Toboso
EPI_ISL_444022, EPI_ISL_445078 to 445084, EPI_ISL_501167 to 501174, EPI_ISL_513294	Baylor College of Medicine	Baylor College of Medicine: HGSC	David Henke; Donna Muzny; Erin Nicholson; George Weissenberger; Ginger Metcalf; Harsha Doddapaneni; Hsu Chao; Hua Shen; Joseph F. Petrosino; Kavya Kottapalli; Kristi L. Hoffman; Matthew C. Ross; Matthew Wong; Pedro Piedra; Qingchang Meng; Richard Sucgang; Sara J.J. Cregeen; Sejal Salvi; Tulin Ayvaz; Vasanthi Avadhanula; Vipin Menon; Yimiti Meiheerguli; Zeineen Momin
EPI_ISL_1400800	Baylor Esoteric + Molecular Lab	Baylor Esoteric + Molecular Lab	Dawn Richards; Elizabeth Forrester
EPI_ISL_1443344, EPI_ISL_1443512, EPI_ISL_1443601, EPI_ISL_1489935, EPI_ISL_1489939, EPI_ISL_1490190, EPI_ISL_1490222, EPI_ISL_1490227, EPI_ISL_1490251, EPI_ISL_1490652, EPI_ISL_1490660, EPI_ISL_1490667, EPI_ISL_1490711, EPI_ISL_1490716, EPI_ISL_1490749, EPI_ISL_1490817, EPI_ISL_1490897, EPI_ISL_1491054, EPI_ISL_1491361, EPI_ISL_1491366, EPI_ISL_1491387 to 1491388, EPI_ISL_1493105, EPI_ISL_1493109, EPI_ISL_1493123, EPI_ISL_1497766, EPI_ISL_1498094, EPI_ISL_1498096			
see above	Baylor Esoteric and Molecular Lab	Baylor Esoteric and Molecular Lab	Elizabeth Forrester and Dawn Richards
EPI_ISL_1391413	Baylor Scott & White-Irving	Baylor Scott & White-Temple	Ari Rao; Caitlin Maloney; Kimberly Walker; Linden Morales; Marcus Volz; Shelby Hendrickson
EPI_ISL_1391416, EPI_ISL_1391421, EPI_ISL_1391621	Baylor Scott & White-Temple	Baylor Scott & White-Temple	Ari Rao; Caitlin Maloney; Kimberly Walker; Linden Morales; Marcus Volz; Shelby Hendrickson
EPI_ISL_1391415	Baylor Scott & White-Waxahachie	Baylor Scott & White-Temple	Ari Rao; Caitlin Maloney; Kimberly Walker; Linden Morales; Marcus Volz; Shelby Hendrickson
EPI_ISL_412386	Beijing Ditan Hospital, Capital Medical University	National Institute for Communicable Disease Control and Prevention, Chinese Center for Disease Control and Prevention	Biao Kan; Chuansheng Li; Fangfang Jin; Haijian Zhou; Haofeng Xiong; Hebing Guo; Huizhu Wang; Jianbo Tan; Jie Gong; Jingjing Hao; Jingyuan Liu; Lili Gao; Lin Pu; Ming Zhang; Pan Xiang; Ruihong Li; Xiaoping Chen; Xin Lu; Xinmin Xu; Yajie Wang; Yanwen Xiong; Yao Sun; Yufeng Liu
EPI_ISL_424355 to 424360	Beijing Institute of Microbiology and Epidemiology	Beijing Institute of Microbiology and Epidemiology	B. and Cui, Y.; Fan, H.; Guo, Y.; Hou, J.; Li; Mi, Z.; Mu, J.; Qin, E.; Song, Y.; Teng, Y.; Wu, Y.; Xu, Z.; Yang, R.; Yong, Y.; Zhang, X.
EPI_ISL_509711 to 509714	Belize Ministry of Health	Pathogen Discovery, Respiratory Viruses Branch, Division of Viral Diseases, Centers for Disease Control and Prevention	Anna Uehara; Clinton Paden; Haibin Wang; Jing Zhang; Krista Queen; Suxiang Tong; Yan Li; Ying Tao
EPI_ISL_569609	Bennet County Hospital	South Dakota Public Health Laboratory	Jacob Garfin; Matt Plumb; Xiong Wang; and Chris Carlson
EPI_ISL_1423010 to 1423014, EPI_ISL_1423112 to 1423124, EPI_ISL_1423203 to 1423210, EPI_ISL_1423213 to 1423227, EPI_ISL_1423246 to 1423256, EPI_ISL_1423326 to 1423340, EPI_ISL_1528579 to 1528600, EPI_ISL_1528745 to 1528755, EPI_ISL_1529029 to 1529074			
see above	Berkeley Medical Center	WVU and Marshall University Combined Genomics Core Facilities	"James Denvir; Peter Perrotta; Peter Stoilov; Ryan Percifield"; Wesley Kimble
EPI_ISL_430843	Bethany Hospital	Research Institute for Tropical Medicine	Bautista; Brunker, K.; C.S.; C.T.; D.L.; Demetria; E.S.; F.G.M.; I.A.P.; Manalo; Medado; Mercado; O.J.T.; Onza; Polotan
EPI_ISL_569618 to 569619	Bethel Lutheran Home	South Dakota Public Health Laboratory	Jacob Garfin; Matt Plumb; Xiong Wang; and Chris Carlson
EPI_ISL_457824	Bezmialem Vakif University, Dept Microbiology, Medical School, Fatih, Istanbul, Turkey	Bezmialem Vakif University, Medical School & Beykoz Institute of Life Sciences & Biotechnology	Bilge Sumbul; Elif Karaaslan; Filiz Guney; Mehmet Z. Doymaz; Merve Kalkan; Nesibe Cetin
EPI_ISL_1388112, EPI_ISL_1388230, EPI_ISL_1388355 to 1388356, EPI_ISL_1388457, EPI_ISL_1388536, EPI_ISL_1388542, EPI_ISL_1388544, EPI_ISL_1388546 to 1388548, EPI_ISL_1388606, EPI_ISL_1388608, EPI_ISL_1388610, EPI_ISL_1388612, EPI_ISL_1388614, EPI_ISL_1388616, EPI_ISL_1388657, EPI_ISL_1388687, EPI_ISL_1388689, EPI_ISL_1388701, EPI_ISL_1388703, EPI_ISL_1388705, EPI_ISL_1388842			
see above	Bioanalytika AG	University Hospital Basel, Clinical Bacteriology	Adrian Egli; Adrian Härri; Alfredo Mari; Hans Hirsch; Helena MB Seth-Smith; Julia Bielicki; Karoline Leuzinger; Madlen Stange; Manuel Battegay; Tim Roloff
EPI_ISL_1502568 to 1502582	Biochemistry and Molecular Biology Department-Faculty of Medicine, Al-Quds University	Biochemistry and Molecular Biology Department-Faculty of Medicine, Al-Quds University	Al-Jawabreh, A.; Dumaidi, K.; Ereqat, S.; Nasereddin, A.
EPI_ISL_1389139 to 1389142, EPI_ISL_1389144 to 1389155, EPI_ISL_1476741, EPI_ISL_1476746 to 1476747, EPI_ISL_1476752, EPI_ISL_1476754, EPI_ISL_1476756 to 1476759, EPI_ISL_1476762 to 1476813, EPI_ISL_1476815 to 1476850, EPI_ISL_1476853 to 1476858, EPI_ISL_1476860, EPI_ISL_1476867, EPI_ISL_1476872, EPI_ISL_1476874, EPI_ISL_1476878, EPI_ISL_1476880, EPI_ISL_1476892, EPI_ISL_1476895 to 1476897, EPI_ISL_1476899 to 1476900			
see above	Bioinformatics and Biostatistics Lab, Advanced Sequencing Facility	COVID-19 Genomics UK (COG-UK) Consortium	Aengus Stewart; Chelsea Sawyer; Harshil Patel; Jerome Nicod; Laura Cubitt; Margaret Crawford
EPI_ISL_429992 to 430009, EPI_ISL_430011 to 430015, EPI_ISL_434516, EPI_ISL_450186 to 450189	Biolab Diagnostic Laboratories	Andersen lab at Scripps Research	Ahmad Tibi; Amid Abdelnour with SEARCH Alliance San Diego; Issa Abu-Dayyeh; Lama Hussein; Lina Mohammad; Zein Naber
EPI_ISL_1404588, EPI_ISL_1404614, EPI_ISL_1405965, EPI_ISL_1406143, EPI_ISL_1406151, EPI_ISL_1406179, EPI_ISL_1406183, EPI_ISL_1406189, EPI_ISL_1406195, EPI_ISL_1406202, EPI_ISL_1406239			
see above	Biolab Diagnostic Laboratories	Biolab Diagnostic Laboratories	Ahmad Tibi; Amid Abdelnour; Badia Saddedin; Eiad Atwa; Issa Abu-Dayyeh; Lama Hussein; Shayma Ali
EPI_ISL_527007	Biological Prevention, Army	Biological Prevention, Army	A.E.; A.F.; A.M.; Ageez; B.E.; Elhoseiny; Gad; Harty; M.D.; M.F.; M.G.; Seadawy; Shabaan; Shamel
EPI_ISL_526975 to 527006	Biological prevention, army	Biological prevention, army	A.E.; A.F.; A.M.; Ageez; B.E.; Elhoseiny; Elhosieny; Gad; Harty; M.D.; M.F.; M.G.; Seadawy; Shabaan; Shamel
EPI_ISL_510526, EPI_ISL_510532	Biological prevention, army	Biological prevention, army	A.A.; A.F.; A.M. and Soliman; Ali; Amer; B.E.; B.S.; ELnabrawy; ElGohary; Elhoseiny; Elhoseny; Elnagdy; Elnakeeb; Gad; H.A.; Harty; Hassan; Kandeil; Karam; M.A.; M.D.; M.F.; M.G.; M.M. and Gad; Raouf; Seadawy; Shamel; T.A.; W.A.; Y.A.; k.E.
EPI_ISL_1524330 to 1524381	Biology Department, College of Science, Al Muthanna University and Public Health Laboratory, Al-Muthanna Health Directorate	Department of Virology, Faculty of Medicine, University of Helsinki, Helsinki, Finland generated and submitted to GISAID	Alaa Hameed; Ali Jasim; Hussein Alburkat; Murad Munahi; Nihad Al-Rashedi; Olli Vapalahti; Tarja Sironen; Teemu Smura
EPI_ISL_582029 to 582030	Biology Department, College of Science, Al-Muthanna University	International Centre for Genetic Engineering and Biotechnology (ICGEB) and ARGO Open Lab Platform	Alessandro Marcello; Danilo Licastro; Nihad Al-Rashedi; Simeone Dal Monego; Sreejith Rajasekharan
EPI_ISL_505003	Biology Dpt	Microbiology and Infections Diseases	Annabelle Garnier; Audrey Ferrier-Rembert; Clarisse Vigne; Emilie Tessier; Emmanuelle Billon-Denis; Flora Nolent; Isabelle Drouet; Jean-Nicolas Tournier; Jessica Denis; Laurence Cheutin; Noémie Verguet; Olivier Ferraris; Olivier Gorgé
EPI_ISL_506041	Biology Dpt, HIA Percy	Microbiology and Infectious Diseases Dpt	Annabelle Garnier; Audrey Ferrier-Rembert; Clarisse Vigne; Emilie Tessier; Emmanuelle Billon-Denis; Flora Nolent; Isabelle Drouet; Jean-Nicolas Tournier; Jessica Denis; Laurence Cheutin; Noémie Verguet; Olivier Ferraris; Olivier Gorgé
EPI_ISL_1388384, EPI_ISL_1388807, EPI_ISL_1388809 to 1388810, EPI_ISL_1388812 to 1388813	Biolytix AG	University Hospital Basel, Clinical Bacteriology	Adrian Egli; Alfredo Mari; Hans Hirsch; Helena MB Seth-Smith; Julia Bielicki; Karoline Leuzinger; Madlen Stange; Manuel Battegay; Simon Fuchs; Tim Roloff
EPI_ISL_1420638 to 1420643, EPI_ISL_1420645, EPI_ISL_1420647 to 1420659, EPI_ISL_1420661 to 1420702, EPI_ISL_1420704 to 1420723, EPI_ISL_1420733 to 1420750, EPI_ISL_1420752 to 1420786			
see above	Biomedical Research Foundation of the Academy of Athens (BRFAA)	Greek Genome Center, Biomedical Research Foundation of the Academy of Athens (BRFAA)	Dimitrios Thanos; Emmanouil Athanasiadis; Ioannis Vatsellas; Katerina Zoi; Theodoros Loupis
EPI_ISL_516079 to 516088	Biomedical Sciences and Public Health, Polytechnic University of Marche	Biomedical Sciences and Public Health, Polytechnic University of Marche	Alessandrini, F.; Bagnarelli, P.; Caucci, S.; Di Sante, L.; Melchionda, F.; Menzo, S.; Onofri, V.; Tagliabracci, A.; Turchi, C.
EPI_ISL_1498381 to 1498584, EPI_ISL_1498586 to 1498820, EPI_ISL_1522575, EPI_ISL_1522594, EPI_ISL_1522599, EPI_ISL_1522602, EPI_ISL_1522605, EPI_ISL_1522613, EPI_ISL_1522622, EPI_ISL_1522633, EPI_ISL_1522636, EPI_ISL_1522653 to 1522654, EPI_ISL_1522657, EPI_ISL_1522667, EPI_ISL_1522674, EPI_ISL_1522676, EPI_ISL_1522678, EPI_ISL_1522708, EPI_ISL_1522714 to 1522715, EPI_ISL_1522728, EPI_ISL_1522730, EPI_ISL_1522733, EPI_ISL_1522737, EPI_ISL_1522739 to 1522740, EPI_ISL_1522743 to 1522744, EPI_ISL_1522747 to 1522748, EPI_ISL_1522754, EPI_ISL_1522758, EPI_ISL_1522769 to 1522770, EPI_ISL_1522781, EPI_ISL_1522783 to 1522785, EPI_ISL_1522801 to 1522839, EPI_ISL_1523027 to 1523039, EPI_ISL_1523133, EPI_ISL_1523135 to 1523136, EPI_ISL_1523154, EPI_ISL_1523162, EPI_ISL_1523186, EPI_ISL_1523194, EPI_ISL_1523200, EPI_ISL_1523205, EPI_ISL_1523209, EPI_ISL_1523212, EPI_ISL_1523215 to 1523218, EPI_ISL_1523224 to 1523251, EPI_ISL_1523345 to 1523346			
see above	BioneXt Lab	Laboratoire national de sante, Microbiology, Microbial Genomics Platform	Anke Wienecke-Baldacchino; Catherine Ragimbeau; Fatu Djabi; Jessica Tapp; Lise Pignon; Raoul Salmon; Tamir Abdelrahman; Thibault Ferrandon

EPI_ISL_458287	Biosafety Department PCL3	Biosafety Department PCL3	A. and El Kabbaj, S.; Lemriss, S.; Souiri
EPI_ISL_1432939, EPI_ISL_1432943, EPI_ISL_1432945, EPI_ISL_1432952, EPI_ISL_1432954, EPI_ISL_1432958, EPI_ISL_1432963, EPI_ISL_1432970, EPI_ISL_1432978, EPI_ISL_1432991, EPI_ISL_1433000, EPI_ISL_1433002, EPI_ISL_1433004, EPI_ISL_1433015, EPI_ISL_1433017, EPI_ISL_1433019, EPI_ISL_1433023, EPI_ISL_1433048, EPI_ISL_1433050, EPI_ISL_1433052, EPI_ISL_1433059, EPI_ISL_1433061 to 1433066, EPI_ISL_1433069, EPI_ISL_1433071, EPI_ISL_1433073 to 1433074, EPI_ISL_1433080, EPI_ISL_1433086, EPI_ISL_1433088, EPI_ISL_1433090, EPI_ISL_1433097, EPI_ISL_1433101, EPI_ISL_1433106, EPI_ISL_1433115 to 1433116, EPI_ISL_1433119, EPI_ISL_1433128, EPI_ISL_1433133, EPI_ISL_1433137 to 1433139, EPI_ISL_1433145, EPI_ISL_1433149 to 1433152, EPI_ISL_1433155 to 1433157, EPI_ISL_1433160 to 1433161, EPI_ISL_1433163, EPI_ISL_1433172 to 1433173, EPI_ISL_1433177 to 1433178, EPI_ISL_1433180 to 1433183, EPI_ISL_1433185 to 1433186, EPI_ISL_1433194, EPI_ISL_1433196, EPI_ISL_1433198 to 1433199, EPI_ISL_1433205, EPI_ISL_1433210 to 1433212, EPI_ISL_1433217 to 1433218, EPI_ISL_1433221, EPI_ISL_1433224, EPI_ISL_1433232, EPI_ISL_1433234, EPI_ISL_1433236, EPI_ISL_1433240, EPI_ISL_1433244 to 1433245, EPI_ISL_1433247, EPI_ISL_1433249 to 1433250, EPI_ISL_1433253, EPI_ISL_1433255 to 1433256, EPI_ISL_1433262, EPI_ISL_1433267, EPI_ISL_1433271, EPI_ISL_1433273 to 1433274, EPI_ISL_1433278, EPI_ISL_1433280, EPI_ISL_1433282 to 1433283, EPI_ISL_1433300, EPI_ISL_1433305, EPI_ISL_1433308 to 1433309, EPI_ISL_1433311, EPI_ISL_1433321, EPI_ISL_1433323, EPI_ISL_1433325, EPI_ISL_1433327, EPI_ISL_1433329, EPI_ISL_1433334, EPI_ISL_1433340, EPI_ISL_1433360, EPI_ISL_1433362, EPI_ISL_1433372, EPI_ISL_1433374, EPI_ISL_1433376, EPI_ISL_1436034, EPI_ISL_1436305, EPI_ISL_1436307, EPI_ISL_1438686, EPI_ISL_1438693, EPI_ISL_1438696 to 1438699, EPI_ISL_1438704 to 1438705, EPI_ISL_1438710 to 1438711, EPI_ISL_1438717, EPI_ISL_1438721, EPI_ISL_1438723 to 1438724, EPI_ISL_1438729 to 1438730, EPI_ISL_1438732, EPI_ISL_1438739 to 1438741, EPI_ISL_1438743 to 1438744, EPI_ISL_1438746 to 1438747, EPI_ISL_1438749 to 1438751, EPI_ISL_1438753 to 1438758, EPI_ISL_1438764 to 1438765, EPI_ISL_1438767 to 1438768, EPI_ISL_1438770, EPI_ISL_1438773 to 1438777, EPI_ISL_1438781 to 1438782, EPI_ISL_1438788, EPI_ISL_1438790, EPI_ISL_1438792 to 1438794, EPI_ISL_1438796, EPI_ISL_1438800 to 1438804, EPI_ISL_1438807, EPI_ISL_1438810, EPI_ISL_1438816, EPI_ISL_1438818, EPI_ISL_1438820, EPI_ISL_1438822, EPI_ISL_1438827 to 1438828, EPI_ISL_1438830, EPI_ISL_1438832, EPI_ISL_1438837, EPI_ISL_1438839 to 1438840, EPI_ISL_1438844, EPI_ISL_1438848, EPI_ISL_1438850, EPI_ISL_1438853, EPI_ISL_1438857 to 1438858, EPI_ISL_1438860, EPI_ISL_1438864, EPI_ISL_1438866, EPI_ISL_1438868, EPI_ISL_1438873, EPI_ISL_1438875 to 1438877, EPI_ISL_1438881, EPI_ISL_1438883, EPI_ISL_1438885 to 1438886, EPI_ISL_1438888, EPI_ISL_1438890 to 1438891, EPI_ISL_1438895, EPI_ISL_1438898 to 1438899, EPI_ISL_1438902, EPI_ISL_1438906, EPI_ISL_1438911, EPI_ISL_1438913, EPI_ISL_1438915 to 1438916, EPI_ISL_1438921 to 1438923, EPI_ISL_1438930, EPI_ISL_1438933, EPI_ISL_1438936, EPI_ISL_1438942, EPI_ISL_1438944 to 1438945, EPI_ISL_1438948 to 1438953, EPI_ISL_1438956 to 1438957, EPI_ISL_1438960, EPI_ISL_1438962 to 1438968, EPI_ISL_1438970, EPI_ISL_1438975, EPI_ISL_1438977 to 1438979, EPI_ISL_1438981 to 1438982, EPI_ISL_1438987 to 1438988, EPI_ISL_1438990, EPI_ISL_1438993 to 1438994, EPI_ISL_1438996, EPI_ISL_1438999 to 1439000, EPI_ISL_1439006, EPI_ISL_1439013, EPI_ISL_1439015, EPI_ISL_1439023, EPI_ISL_1439026, EPI_ISL_1439035 to 1439037, EPI_ISL_1439039, EPI_ISL_1439047, EPI_ISL_1439057 to 1439058, EPI_ISL_1439067, EPI_ISL_1439076, EPI_ISL_1439081 to 1439082, EPI_ISL_1439086, EPI_ISL_1439089 to 1439090, EPI_ISL_1439094, EPI_ISL_1439105, EPI_ISL_1439111, EPI_ISL_1439115, EPI_ISL_1439117, EPI_ISL_1439123, EPI_ISL_1439132, EPI_ISL_1439139 to 1439140, EPI_ISL_1439143, EPI_ISL_1439150 to 1439151, EPI_ISL_1439153, EPI_ISL_1439156, EPI_ISL_1439166 to 1439169, EPI_ISL_1439179, EPI_ISL_1439181 to 1439182, EPI_ISL_1439185, EPI_ISL_1439187 to 1439188, EPI_ISL_1439190 to 1439191, EPI_ISL_1439196, EPI_ISL_1439198, EPI_ISL_1439205 to 1439206, EPI_ISL_1439214 to 1439215, EPI_ISL_1439223, EPI_ISL_1439227, EPI_ISL_1439231, EPI_ISL_1439242, EPI_ISL_1439251, EPI_ISL_1439255, EPI_ISL_1439257 to 1439259, EPI_ISL_1439261 to 1439263, EPI_ISL_1439274, EPI_ISL_1439276, EPI_ISL_1439280, EPI_ISL_1439295, EPI_ISL_1439309, EPI_ISL_1439311, EPI_ISL_1439319, EPI_ISL_1439325 to 1439326, EPI_ISL_1439330, EPI_ISL_1439336 to 1439338, EPI_ISL_1439350, EPI_ISL_1439358 to 1439360, EPI_ISL_1439364			
see above	Bioscientia Labor Wermsdorf	Robert Koch Institute	
EPI_ISL_1432947, EPI_ISL_1432982, EPI_ISL_1432995, EPI_ISL_1433067, EPI_ISL_1433087, EPI_ISL_1433092, EPI_ISL_1433117, EPI_ISL_1433121, EPI_ISL_1433129, EPI_ISL_1433136, EPI_ISL_1433144, EPI_ISL_1433179, EPI_ISL_1433192, EPI_ISL_1433209, EPI_ISL_1433213, EPI_ISL_1433230, EPI_ISL_1433286, EPI_ISL_1433338, EPI_ISL_1439158, EPI_ISL_1439237, EPI_ISL_1439243, EPI_ISL_1439327, EPI_ISL_1439340, EPI_ISL_1439344			
see above	Bioscientia MVZ Labor Karlsruhe GmbH	Robert Koch Institute	
EPI_ISL_450813, EPI_ISL_475540	Bla Kustens halsocentral	The Public Health Agency of Sweden	Anna Risberg; Anna-Malin Linde; Karin Tegmark-Wisell; Maria Lind Karlberg; Mattias Haukland; Mia Brytting; Olof Norrby; Olov Svartstrom; Oskar Karlsson Lindsjo; Reza Advani; Sandra Broddesson; Theresa Enkirch
EPI_ISL_1534276, EPI_ISL_1534449	Bongolethu Clinic wc BLC	NHLS/UCT	Arash Iranzadeh; Bruna Galvao; Carolyn Williamson; Deelan Doolabh; Diana Hardie; Emmanuel SJ; Innocent Mudau; Kruger Marais; Lynn Tyers; Marvin Hsiao; Stephen Korsman; Tegally H; de Oliveira T
EPI_ISL_596453	Booali laboratory, Qom, Iran. Department of Virology, School of Public Health, Tehran University of Medical Sciences, Tehran, Iran.	Genetics Research Center, University of Social Welfare and Rehabilitation Sciences	Ali Jafarpour; Azam Ghaziasadi; Hossein Najmbadi; Khadijeh Jalalvand; Kimia Kahrizi; Marzieh Mohseni; Mohammad Khazeni; Seyed Amir Momeni; Seyed Mohammad Jazayeri; Seyedeh elham Mortazavi; Zohreh Fattahi
EPI_ISL_1393841 to 1393862	Borlamed Medyczne Laboratorium Diagnostyczne	1. National Institute of Public Health - National Institute of Hygiene; 2. Eurofins Genomics Europe Sequencing GmbH	ECDC COVID-19 WGS support team; Eurofins Genomics Europe Sequencing Team; Gierczyki Rafa; Sadkowska-Todys Magorzata; Wokowicz Tomasz; Zacharczuk Katarzyna
EPI_ISL_1516827 to 1516881, EPI_ISL_1524873 to 1524885, EPI_ISL_1532841 to 1532854	Botswana Harvard HIV Reference Laboratory	Botswana Harvard HIV Reference Laboratory	Boitumelo Zuze; Botshelo Radibe; David Lawrence; Dorcas Maruapula; Joseph Makhema; Legodile Kooepile; Mosepele Mosepele; Roger Shapiro; Shahin Lockman; Sikhulile Dorcas Maruapula; Sikhulile Wonderful T. Choga; Simani Gaseitsiwe; Thongbotho Mphoyakgosi; Wonderful T. Choga
EPI_ISL_437433	Bozeman Health Deaconess Hospital	Wiedenheft lab, Montana State University	Anna Nemudraia; Artem Nemudryi; Blake Wiedenheft; Kevin Surya; Murat Buyukyoruk; Royce Wilkinson; Tanner Wiegand
EPI_ISL_437434	Bozeman Water Reclamation Facility	Wiedenheft lab, Montana State University	Anna Nemudraia; Artem Nemudryi; Blake Wiedenheft; Kevin Surya; Murat Buyukyoruk; Royce Wilkinson; Tanner Wiegand
EPI_ISL_1534363	Bredasdorp Clinic wc BDC	NHLS/UCT	Arash Iranzadeh; Bruna Galvao; Carolyn Williamson; Deelan Doolabh; Diana Hardie; Emmanuel SJ; Innocent Mudau; Kruger Marais; Lynn Tyers; Marvin Hsiao; Stephen Korsman; Tegally H; de Oliveira T
EPI_ISL_420799 to 420801	Brian D. Allgood Army Community Hospital	Pathogen Discovery, Respiratory Viruses Branch, Division of Viral Diseases, Centers for Disease Control and Prevention	Alison S. Laufer Halpin; Anne Uehara; Christopher A. Elkins; Clinton R. Paden; Haibin Wang; Jasmine Padilla; Jing Zhang; Justin Lee; Krista Queen; Mary S. Keckler; Rachel Marine; Suxiang Tong; Yan Li; Ying Tao
EPI_ISL_593478 to 593480, EPI_ISL_593553 to 593558	Brigham and Women's Hospital	Jonathan Li Laboratory	James Regan; Jonathan Z. Li; Manish C. Choudhary
EPI_ISL_1407254 to 1407333, EPI_ISL_1413141, EPI_ISL_1413144, EPI_ISL_1413147, EPI_ISL_1413150, EPI_ISL_1413152, EPI_ISL_1413154, EPI_ISL_1413157, EPI_ISL_1413160, EPI_ISL_1413163, EPI_ISL_1413165, EPI_ISL_1413168, EPI_ISL_1413171, EPI_ISL_1413173, EPI_ISL_1413176, EPI_ISL_1413179, EPI_ISL_1413181, EPI_ISL_1413184, EPI_ISL_1413187, EPI_ISL_1413191, EPI_ISL_1413193, EPI_ISL_1413196, EPI_ISL_1413199, EPI_ISL_1413202, EPI_ISL_1413204, EPI_ISL_1413207, EPI_ISL_1413209, EPI_ISL_1413212, EPI_ISL_1413215, EPI_ISL_1413218, EPI_ISL_1413221, EPI_ISL_1413224, EPI_ISL_1413226, EPI_ISL_1413229, EPI_ISL_1413232, EPI_ISL_1413235, EPI_ISL_1413238, EPI_ISL_1413241, EPI_ISL_1413243, EPI_ISL_1413246, EPI_ISL_1413249, EPI_ISL_1413251, EPI_ISL_1413254, EPI_ISL_1413257, EPI_ISL_1413260, EPI_ISL_1413263, EPI_ISL_1413266, EPI_ISL_1413269, EPI_ISL_1413272, EPI_ISL_1413274, EPI_ISL_1413277, EPI_ISL_1413280, EPI_ISL_1413282, EPI_ISL_1413285, EPI_ISL_1413287, EPI_ISL_1413290, EPI_ISL_1413293, EPI_ISL_1413296, EPI_ISL_1413299, EPI_ISL_1413302, EPI_ISL_1413304, EPI_ISL_1413307, EPI_ISL_1413309, EPI_ISL_1413311, EPI_ISL_1413314, EPI_ISL_1413316, EPI_ISL_1413319, EPI_ISL_1413322, EPI_ISL_1413325, EPI_ISL_1413328, EPI_ISL_1413330, EPI_ISL_1413333, EPI_ISL_1413336, EPI_ISL_1413339, EPI_ISL_1413341, EPI_ISL_1413344, EPI_ISL_1413347, EPI_ISL_1413350, EPI_ISL_1413352, EPI_ISL_1413355, EPI_ISL_1413358, EPI_ISL_1413361, EPI_ISL_1413363, EPI_ISL_1413366, EPI_ISL_1413369, EPI_ISL_1413372, EPI_ISL_1413374, EPI_ISL_1413377, EPI_ISL_1413380, EPI_ISL_1413382, EPI_ISL_1413385, EPI_ISL_1413388, EPI_ISL_1413390, EPI_ISL_1413393, EPI_ISL_1413396, EPI_ISL_1413399, EPI_ISL_1413401, EPI_ISL_1413404, EPI_ISL_1413407, EPI_ISL_1413410, EPI_ISL_1413413, EPI_ISL_1413416, EPI_ISL_1413418, EPI_ISL_1413421, EPI_ISL_1413424, EPI_ISL_1413426, EPI_ISL_1413429, EPI_ISL_1413431, EPI_ISL_1413434, EPI_ISL_1413437, EPI_ISL_1413439, EPI_ISL_1413442, EPI_ISL_1413445, EPI_ISL_1413447, EPI_ISL_1413450, EPI_ISL_1413452, EPI_ISL_1413455, EPI_ISL_1413458, EPI_ISL_1413460, EPI_ISL_1413463, EPI_ISL_1413465, EPI_ISL_1413468, EPI_ISL_1413471, EPI_ISL_1413473, EPI_ISL_1413476, EPI_ISL_1413479, EPI_ISL_1413482, EPI_ISL_1413485, EPI_ISL_1413488, EPI_ISL_1413490, EPI_ISL_1413493, EPI_ISL_1413496, EPI_ISL_1413498, EPI_ISL_1413501, EPI_ISL_1413504, EPI_ISL_1413506, EPI_ISL_1413509, EPI_ISL_1413511, EPI_ISL_1413789, EPI_ISL_1413792, EPI_ISL_1413794, EPI_ISL_1413797, EPI_ISL_1413799, EPI_ISL_1413803, EPI_ISL_1413805, EPI_ISL_1413807, EPI_ISL_1413810, EPI_ISL_1413813, EPI_ISL_1413815, EPI_ISL_1413818, EPI_ISL_1413821, EPI_ISL_1413823, EPI_ISL_1413826, EPI_ISL_1413829, EPI_ISL_1413831, EPI_ISL_1413835, EPI_ISL_1413837, EPI_ISL_1413840, EPI_ISL_1413842, EPI_ISL_1413845, EPI_ISL_1413848, EPI_ISL_1413851, EPI_ISL_1413853, EPI_ISL_1413856, EPI_ISL_1413859, EPI_ISL_1413861, EPI_ISL_1413864, EPI_ISL_1516446 to 1516597, EPI_ISL_1516665 to 1516745			
see above	Broad Institute Clinical Research Sequencing Platform	Infectious Disease Program, Broad Institute of Harvard and MIT	Adams, G.; B.L.; B.W.; Bauer, M.; Birren; Blumenstiel, B.; Brown, C.; Carter, A.; Chaluvadi, S.; D.J.; DeFelice, M.; DeRuff, K.; Dodge, S.; Gabriel, S.; Gallagher, G.; Gladden-Young, A.; Granger, B.; J.E.; K.J.; Lagerborg, K.; Larkin, K.; Lee, M.; Lemieux; Lennon, N.; Loreth, C.; Madoff, L.; McGovern, S.; Meldrim, J.; Normandin, E.; P.C.; Park; Pearlman, L.; Reilly, S.; Rudy, M.; Sabeti; Siddle; Smole, S.; Tomkins-Tinch, C.; Vicente, G.; and MacInnis
EPI_ISL_1534396	Brooklyn Chest Hospital wc BCH	NHLS/UCT	Arash Iranzadeh; Bruna Galvao; Carolyn Williamson; Deelan Doolabh; Diana Hardie; Emmanuel SJ; Innocent Mudau; Kruger Marais; Lynn Tyers; Marvin Hsiao; Stephen Korsman; Tegally H; de Oliveira T
EPI_ISL_603248, EPI_ISL_605791	Brotman Baty Institute for Precision Medicine	Brotman Baty Institute for Precision Medicine	Benjamin Pelle; Caitlin R. Wolf; Chris D. Frazar; Christina M. Lockwood; Deborah A. Nickerson; Erica Ryke; Helen Y. Chu; Jay Shendure; Jennifer K. Logue; Jover Lee; Lea M. Starita; Mark J. Rieder; Peter D. Han; Trevor Bedford
EPI_ISL_1501128 to 1501136, EPI_ISL_1501170 to 1501173, EPI_ISL_1501211 to 1501217, EPI_ISL_1501258 to 1501267	Broussais	HEGP - Laboratoire de Virologie	David Veyer
EPI_ISL_1394042 to 1394062, EPI_ISL_1425544	Bruss	1. National Institute of Public Health - National Institute of Hygiene; 2. Eurofins Genomics Europe Sequencing GmbH	ECDC COVID-19 WGS support team; Eurofins Genomics Europe Sequencing Team; Gierczyki Rafa; Sadkowska-Todys Magorzata; Wokowicz Tomasz; Zacharczuk Katarzyna
EPI_ISL_414520 to 414521	Bundeswehr Institute of Microbiology	Bundeswehr Institute of Microbiology	Markus H Antwerpen and Roman Wölfel; Mathias C Walter
EPI_ISL_424981 to 424982, EPI_ISL_424984 to 424986, EPI_ISL_424988 to 424990, EPI_ISL_424992	Bureau of Laboratories, Michigan Department of Health and Human Services	Michigan Department of Health and Human Services	Blankenship HM; Riner D; Soehnlen MK
EPI_ISL_450832	Byjorden vardcentral	The Public Health Agency of Sweden	Anna Risberg; Anna-Malin Linde; Karin Tegmark-Wisell; Maria Lind Karlberg; Mia Brytting; Olov Svartstrom; Oskar Karlsson Lindsjo; Pernilla Brunman; Theresa Enkirch
EPI_ISL_524476	Bülach Hospital	Institute of Medical Virology, University of Zurich	Alexandra Trkola; Andrea Zbinden; Fiona Steiner; Gabriela Ziltener; Jon Huder; Jürg Böni; Maryam Zaheri; Michael Huber; Patrick Redli; Riccarda Capaul; Stefan Schmutz; Verena Kufner
EPI_ISL_445320	C.C.SALUD FAMILIAR PADRE FELIX DONOSO G.	Instituto de Salud Publica de Chile	Alejandra Acevedo; Andrés E Castillo; Bárbara Parra; Carolyna Tambley; Gabriel Leal; Jaime Lagos; Jorge Fernandez; Loredana Arata; Patricia Bustos; Paz Tapia; Rodrigo Fasce; Winston Andrade
EPI_ISL_445318	C.DE SALUD FAMILIAR PABLO NERUDA	Instituto de Salud Publica de Chile	Alejandra Acevedo; Andrés E Castillo; Bárbara Parra; Carolyna Tambley; Gabriel Leal; Jaime Lagos; Jorge Fernandez; Loredana Arata; Patricia Bustos;

			Paz Tapia; Rodrigo Fasce; Winston Andrade
EPI_ISL_539531	C.H.U Nuestra Señora de Candelaria	Instituto de Salud Carlos III	A. Monzón; F. Casas; I; I. Jiménez; Iglesias-Caballero; M. Camarero; M. Cuesta; M. González-Esguevillas; M. Molinero Calamita; M. Zaballos; O. Díez; P. Jiménez; S. Juliá; S. Pozo; S. Varona
EPI_ISL_1477167 to 1477168, EPI_ISL_1477171, EPI_ISL_1477177, EPI_ISL_1477179, EPI_ISL_1477181 to 1477182, EPI_ISL_1477184 to 1477188, EPI_ISL_1477190 to 1477194, EPI_ISL_1477196 to 1477198, EPI_ISL_1477200 to 1477205, EPI_ISL_1477209 to 1477215, EPI_ISL_1477217, EPI_ISL_1477219, EPI_ISL_1477221 to 1477223, EPI_ISL_1477225, EPI_ISL_1477229 to 1477233, EPI_ISL_1477235, EPI_ISL_1477237 to 1477238, EPI_ISL_1477240 to 1477242, EPI_ISL_1477245 to 1477246, EPI_ISL_1477248, EPI_ISL_1477250, EPI_ISL_1477253 to 1477254, EPI_ISL_1477256, EPI_ISL_1477258, EPI_ISL_1477261, EPI_ISL_1477263, EPI_ISL_1477265 to 1477267, EPI_ISL_1477273 to 1477275, EPI_ISL_1477278, EPI_ISL_1477281, EPI_ISL_1477283, EPI_ISL_1477285 to 1477286, EPI_ISL_1477289, EPI_ISL_1477291, EPI_ISL_1477297 to 1477298, EPI_ISL_1477300, EPI_ISL_1477302 to 1477305, EPI_ISL_1477307, EPI_ISL_1477310 to 1477311, EPI_ISL_1477314 to 1477317, EPI_ISL_1477319 to 1477321, EPI_ISL_1477323 to 1477324, EPI_ISL_1477327, EPI_ISL_1477330 to 1477331, EPI_ISL_1477333, EPI_ISL_1477336, EPI_ISL_1477338, EPI_ISL_1477342, EPI_ISL_1477344 to 1477345, EPI_ISL_1477347, EPI_ISL_1477349 to 1477351, EPI_ISL_1477353, EPI_ISL_1477356 to 1477358, EPI_ISL_1477360 to 1477363, EPI_ISL_1477365 to 1477366, EPI_ISL_1477368 to 1477370, EPI_ISL_1477372, EPI_ISL_1477374, EPI_ISL_1477378, EPI_ISL_1477383 to 1477384, EPI_ISL_1477386 to 1477387, EPI_ISL_1477390 to 1477393, EPI_ISL_1477395, EPI_ISL_1477398, EPI_ISL_1477400, EPI_ISL_1477402 to 1477404, EPI_ISL_1477407, EPI_ISL_1477409, EPI_ISL_1477416, EPI_ISL_1477418 to 1477429, EPI_ISL_1477431, EPI_ISL_1477434, EPI_ISL_1477437 to 1477440, EPI_ISL_1477442, EPI_ISL_1477444, EPI_ISL_1477448 to 1477453, EPI_ISL_1477456 to 1477460, EPI_ISL_1477463 to 1477465, EPI_ISL_1477467, EPI_ISL_1477469 to 1477482, EPI_ISL_1477485, EPI_ISL_1477488, EPI_ISL_1477490, EPI_ISL_1477492 to 1477493, EPI_ISL_1477500, EPI_ISL_1477502 to 1477506, EPI_ISL_1477508 to 1477509, EPI_ISL_1477511 to 1477517, EPI_ISL_1477521 to 1477528, EPI_ISL_1477531 to 1477533, EPI_ISL_1477536 to 1477539, EPI_ISL_1477542 to 1477544, EPI_ISL_1477548 to 1477549, EPI_ISL_1477551, EPI_ISL_1477553, EPI_ISL_1477555 to 1477556, EPI_ISL_1477559 to 1477563, EPI_ISL_1477567 to 1477568, EPI_ISL_1477570 to 1477571, EPI_ISL_1477573 to 1477574, EPI_ISL_1477576 to 1477577, EPI_ISL_1477579 to 1477580, EPI_ISL_1477583, EPI_ISL_1477585 to 1477589, EPI_ISL_1477591 to 1477593, EPI_ISL_1477596 to 1477597, EPI_ISL_1477599 to 1477602, EPI_ISL_1477604, EPI_ISL_1477606 to 1477610, EPI_ISL_1477612 to 1477616, EPI_ISL_1477618 to 1477621, EPI_ISL_1477623 to 1477625, EPI_ISL_1477628 to 1477629, EPI_ISL_1477631, EPI_ISL_1477633, EPI_ISL_1477635 to 1477636, EPI_ISL_1477638, EPI_ISL_1477640 to 1477643, EPI_ISL_1477645 to 1477646, EPI_ISL_1477648, EPI_ISL_1477650 to 1477654, EPI_ISL_1477656, EPI_ISL_1477658 to 1477662, EPI_ISL_1477665 to 1477667, EPI_ISL_1477673, EPI_ISL_1477678, EPI_ISL_1477680, EPI_ISL_1477687, EPI_ISL_1477692 to 1477694, EPI_ISL_1477700, EPI_ISL_1477705, EPI_ISL_1477707, EPI_ISL_1477709, EPI_ISL_1477711, EPI_ISL_1477715, EPI_ISL_1477717 to 1477719, EPI_ISL_1477721, EPI_ISL_1477723, EPI_ISL_1477725, EPI_ISL_1477728, EPI_ISL_1477736, EPI_ISL_1477740, EPI_ISL_1477742, EPI_ISL_1477756, EPI_ISL_1477759, EPI_ISL_1477763, EPI_ISL_1477769, EPI_ISL_1477777 to 1477778, EPI_ISL_1477786 to 1477787, EPI_ISL_1477792, EPI_ISL_1477796, EPI_ISL_1477798, EPI_ISL_1477803, EPI_ISL_1477808, EPI_ISL_1477811 to 1477812, EPI_ISL_1477819, EPI_ISL_1477830 to 1477831, EPI_ISL_1477837 to 1477838, EPI_ISL_1477840, EPI_ISL_1477848, EPI_ISL_1477852 to 1477853, EPI_ISL_1477855, EPI_ISL_1477868, EPI_ISL_1477873, EPI_ISL_1477875, EPI_ISL_1477877, EPI_ISL_1477883 to 1477884, EPI_ISL_1477890 to 1477891, EPI_ISL_1477897, EPI_ISL_1477901, EPI_ISL_1477903, EPI_ISL_1477908, EPI_ISL_1477913, EPI_ISL_1477917, EPI_ISL_1477920 to 1477921, EPI_ISL_1477924, EPI_ISL_1477929, EPI_ISL_1477934, EPI_ISL_1477938, EPI_ISL_1477940, EPI_ISL_1477949, EPI_ISL_1477959, EPI_ISL_1477964, EPI_ISL_1477968 to 1477969, EPI_ISL_1477971, EPI_ISL_1477978, EPI_ISL_1477984 to 1477985, EPI_ISL_1477988 to 1477989, EPI_ISL_1477994, EPI_ISL_1477996, EPI_ISL_1478001, EPI_ISL_1478004, EPI_ISL_1478014, EPI_ISL_1478016, EPI_ISL_1478021, EPI_ISL_1478027, EPI_ISL_1478029, EPI_ISL_1478033, EPI_ISL_1478042, EPI_ISL_1478051, EPI_ISL_1478056, EPI_ISL_1478060, EPI_ISL_1478064, EPI_ISL_1478068, EPI_ISL_1478071, EPI_ISL_1478073, EPI_ISL_1478076, EPI_ISL_1478079, EPI_ISL_1478085, EPI_ISL_1478100, EPI_ISL_1478104, EPI_ISL_1478109, EPI_ISL_1478112 to 1478116, EPI_ISL_1478119, EPI_ISL_1478127, EPI_ISL_1478130, EPI_ISL_1478132, EPI_ISL_1478137, EPI_ISL_1478142, EPI_ISL_1478144, EPI_ISL_1478146, EPI_ISL_1478148, EPI_ISL_1478151, EPI_ISL_1478158, EPI_ISL_1478162, EPI_ISL_1478166 to 1478168, EPI_ISL_1478173, EPI_ISL_1478178, EPI_ISL_1478183 to 1478185, EPI_ISL_1478191, EPI_ISL_1478194, EPI_ISL_1478201, EPI_ISL_1529100, EPI_ISL_1529104 to 1529105, EPI_ISL_1529111 to 1529112, EPI_ISL_1529114 to 1529118, EPI_ISL_1529120, EPI_ISL_1529125 to 1529126, EPI_ISL_1529130, EPI_ISL_1529132, EPI_ISL_1529136, EPI_ISL_1529139 to 1529143, EPI_ISL_1529145 to 1529146, EPI_ISL_1529150, EPI_ISL_1529153, EPI_ISL_1529156 to 1529157, EPI_ISL_1529159 to 1529160, EPI_ISL_1529167, EPI_ISL_1529169 to 1529170, EPI_ISL_1529172, EPI_ISL_1529174 to 1529175, EPI_ISL_1529178, EPI_ISL_1529180, EPI_ISL_1529182, EPI_ISL_1529184, EPI_ISL_1529187, EPI_ISL_1529189, EPI_ISL_1529192, EPI_ISL_1529197 to 1529201, EPI_ISL_1529203, EPI_ISL_1529214 to 1529215, EPI_ISL_1529219 to 1529220, EPI_ISL_1529222 to 1529223, EPI_ISL_1529230 to 1529231, EPI_ISL_1529233, EPI_ISL_1529242 to 1529244, EPI_ISL_1529249, EPI_ISL_1529254, EPI_ISL_1529257, EPI_ISL_1529260, EPI_ISL_1529264 to 1529266, EPI_ISL_1529274, EPI_ISL_1529276 to 1529278, EPI_ISL_1529280 to 1529281, EPI_ISL_1529286, EPI_ISL_1529288 to 1529291, EPI_ISL_1529294, EPI_ISL_1529299, EPI_ISL_1529303, EPI_ISL_1529306 to 1529307, EPI_ISL_1529312 to 1529315, EPI_ISL_1529319, EPI_ISL_1529321, EPI_ISL_1529324 to 1529326, EPI_ISL_1529332 to 1529333, EPI_ISL_1529335, EPI_ISL_1529337 to 1529339, EPI_ISL_1529356 to 1529357, EPI_ISL_1529359 to 1529363, EPI_ISL_1529366, EPI_ISL_1529369			
see above	CA DPH Viral and Rickettsial Disease Laboratory	Chan-Zuckerberg Biohub	CZB Cliahub Consortium
EPI_ISL_576177	CA, CDPH, Viral and Rickettsial Disease Laboratory	Pathogen Discovery, Respiratory Viruses Branch, Division of Viral Diseases, Centers for Disease Control and Prevention	Anna Uehara; Brian Lynch; Clinton R. Paden; Haibin Wang; Jing Zhang; Krista Queen; Peter Cook; Suxiang Tong; Yan Li; Ying Tao
EPI_ISL_1446714 to 1446715, EPI_ISL_1447049 to 1447051, EPI_ISL_1516361	CA-Los Angeles County Public Health Laboratory	Centers for Disease Control and Prevention Division of Viral Diseases, Pathogen Discovery	Alison Laufer Halpin; Ben L. Rambo-Martin; Clinton R. Paden; Dakota Howard; Darlene Wagner; Dave Wentworth; Dhwani Batra; Jasmine Padilla; Justin Lee; Katie Dillon; Krista Queen; Kristen Knipe; Kristine Lacek; Mark Burroughs; Matthew Schmerer; Mili Sheth; Peter Cook; Sam Shepard; Sarah Nobles; Shoshona Le; Suxiang Tong; Vivien Dugan; Yvette Unoarumhi
EPI_ISL_1553808	CAP DE L'ESQUIROL	Banc de Sang i Teixits	Carlos Hobeich; Francisco Vidal; Irene Corrales; Lorena Ramírez; Maria Glòria Soria; Natália Comes; Nina Borràs; Noemí Gonzalez; Silvia Sauleda
EPI_ISL_1553801 to 1553802, EPI_ISL_1553806 to 1553807	CAP EL REMEI	Banc de Sang i Teixits	Carlos Hobeich; Francisco Vidal; Irene Corrales; Lorena Ramírez; Maria Glòria Soria; Natália Comes; Nina Borràs; Noemí Gonzalez; Silvia Sauleda
EPI_ISL_1553804, EPI_ISL_1553809, EPI_ISL_1553818, EPI_ISL_1553826, EPI_ISL_1553828, EPI_ISL_1553830, EPI_ISL_1553843, EPI_ISL_1553846			
see above	CAP MANLLEU	Banc de Sang i Teixits	Carlos Hobeich; Francisco Vidal; Irene Corrales; Lorena Ramírez; Maria Glòria Soria; Natália Comes; Nina Borràs; Noemí Gonzalez; Silvia Sauleda
EPI_ISL_1553805	CAP PRATS DE LLUÇANÈS	Banc de Sang i Teixits	Carlos Hobeich; Francisco Vidal; Irene Corrales; Lorena Ramírez; Maria Glòria Soria; Natália Comes; Nina Borràs; Noemí Gonzalez; Silvia Sauleda
EPI_ISL_1553815 to 1553816, EPI_ISL_1553842	CAP RODA DE TER	Banc de Sang i Teixits	Carlos Hobeich; Francisco Vidal; Irene Corrales; Lorena Ramírez; Maria Glòria Soria; Natália Comes; Nina Borràs; Noemí Gonzalez; Silvia Sauleda
EPI_ISL_1553803, EPI_ISL_1553847	CAP ROGER DE FLOR	Banc de Sang i Teixits	Carlos Hobeich; Francisco Vidal; Irene Corrales; Lorena Ramírez; Maria Glòria Soria; Natália Comes; Nina Borràs; Noemí Gonzalez; Silvia Sauleda
EPI_ISL_1553832	CAP SAGRADA FAMILIA	Banc de Sang i Teixits	Carlos Hobeich; Francisco Vidal; Irene Corrales; Lorena Ramírez; Maria Glòria Soria; Natália Comes; Nina Borràs; Noemí Gonzalez; Silvia Sauleda
EPI_ISL_1553821 to 1553822, EPI_ISL_1553840	CAP SANT HIPÒLIT DE VOLTREGÀ	Banc de Sang i Teixits	Carlos Hobeich; Francisco Vidal; Irene Corrales; Lorena Ramírez; Maria Glòria Soria; Natália Comes; Nina Borràs; Noemí Gonzalez; Silvia Sauleda
EPI_ISL_1553838	CAP SANT JULIÀ DE VILATORTA	Banc de Sang i Teixits	Carlos Hobeich; Francisco Vidal; Irene Corrales; Lorena Ramírez; Maria Glòria Soria; Natália Comes; Nina Borràs; Noemí Gonzalez; Silvia Sauleda
EPI_ISL_1553839	CAP SANT PERE DE TORELLÓ	Banc de Sang i Teixits	Carlos Hobeich; Francisco Vidal; Irene Corrales; Lorena Ramírez; Maria Glòria Soria; Natália Comes; Nina Borràs; Noemí Gonzalez; Silvia Sauleda
EPI_ISL_1553823	CAP SANT QUIRZE DE BESORA	Banc de Sang i Teixits	Carlos Hobeich; Francisco Vidal; Irene Corrales; Lorena Ramírez; Maria Glòria Soria; Natália Comes; Nina Borràs; Noemí Gonzalez; Silvia Sauleda
EPI_ISL_1553810, EPI_ISL_1553817, EPI_ISL_1553824, EPI_ISL_1553829, EPI_ISL_1553833	CAP TONA	Banc de Sang i Teixits	Carlos Hobeich; Francisco Vidal; Irene Corrales; Lorena Ramírez; Maria Glòria Soria; Natália Comes; Nina Borràs; Noemí Gonzalez; Silvia Sauleda
EPI_ISL_1553800, EPI_ISL_1553811, EPI_ISL_1553819, EPI_ISL_1553825, EPI_ISL_1553827, EPI_ISL_1553841, EPI_ISL_1553844 to 1553845			
see above	CAP TORELLÓ	Banc de Sang i Teixits	Carlos Hobeich; Francisco Vidal; Irene Corrales; Lorena Ramírez; Maria Glòria Soria; Natália Comes; Nina Borràs; Noemí Gonzalez; Silvia Sauleda
EPI_ISL_1553812 to 1553813, EPI_ISL_1553831, EPI_ISL_1553834 to 1553837	CAP VIC NORD	Banc de Sang i Teixits	Carlos Hobeich; Francisco Vidal; Irene Corrales; Lorena Ramírez; Maria Glòria Soria; Natália Comes; Nina Borràs; Noemí Gonzalez; Silvia Sauleda
EPI_ISL_467516	CAPRISA	KRISP, KZN Research Innovation and Sequencing Platform	Chimukangara B; Giandhari J; Khan S; Lessells R; Mdlalose K; Pillay S; Tegally H; Wilkinson E; York D; de Oliveira T
EPI_ISL_586549 to 586550, EPI_ISL_586555 to 586561, EPI_ISL_590690	CCC,Veraval	Gujarat Biotechnology Research Centre	A M Kadri; Afzal Ansari; Apurvasinh Puvar; Chaitanya Joshi; Dinesh Kumar; Harsh Bakshi; Janvi Raval; Jignesh Parmar; Jitendra Bamrotia; Komal Patel; Labdhi Pandya; Madhvi Joshi; Maharshi Pandya; Monika Gandhi; Nidhi Patel; Nikha Trivedi; Nitin Savaliya; Pinal Trivedi; R D Dixit; Raghawendra Kumar; Zarna Patel; Zuber Saiyed
EPI_ISL_1461204	CDP CH THONON	CNR Virus des Infections Respiratoires - France SUD	Antonin Bal; Bruno Lina; Gregory Destras; Gwendolyne Burfin; Hadrien Regue; Laurence Josset; Martine Valette; Quentin Semanas
EPI_ISL_1406589, EPI_ISL_1406649, EPI_ISL_1483772, EPI_ISL_1547441 to 1547444	CDP COVID MONTLUCON	CHU Clermont-Ferrand, service de virologie	Bisseux Maxime; Combes Patricia; Henquell Cécile; Mirand Audrey
EPI_ISL_1418301	CDP PIERRE OUDOT	CNR Virus des Infections Respiratoires - France SUD	Antonin Bal; Bruno Lina; Gregory Destras; Gwendolyne Burfin; Hadrien Regue; Laurence Josset; Martine Valette; Quentin Semanas
EPI_ISL_535662 to 535715	CDPH, Microbial Diseases Laboratory	Pathogen Discovery, Respiratory Viruses Branch, Division of Viral Diseases, Centers for Disease Control and Prevention	Anna Uehara; Brian Lynch; Clinton R. Paden; Haibin Wang; Jing Zhang; Krista Queen; Rachel Marine; Suxiang Tong; Yan Li; Ying Tao
EPI_ISL_1483240	CDPH, Viral and Rickettsial Disease Laboratory	Pathogen Discovery, Respiratory Viruses Branch, Division of Viral Diseases, Centers for Disease Control and Prevention	Anna Kelleher; Anna Uehara; Brian Lynch; Clinton R. Paden; Haibin Wang; Han Jia Justin Ng; Jing Zhang; Krista Queen; Peter Cook; Suxiang Tong; Yan Li; Ying Tao
EPI_ISL_486852	CDRI/SGPGI	CSIR-CDRI/SGPGI	Dharam Veer Singh; Rahul Vishvkarma; Rajender Singh; Ravishankar Ramachandran; Saumya Sarkar; Tapas Kumar Kundu; Uday Ghoshal; Ujjala Ghoshal
EPI_ISL_1461168 to 1461178	CEDIBIO UNILAB TOULOUSE	CNR Virus des Infections Respiratoires - France SUD	Antonin Bal; Bruno Lina; Gregory Destras; Gwendolyne Burfin; Hadrien Regue; Laurence Josset; Martine Valette; Quentin Semanas
EPI_ISL_605780 to 605782	CEIRS Data Processing and Coordinating Center, St. Jude Center of Excellence for Influenza Research and Surveillance (CEIRS)	CEIRS Data Processing and Coordinating Center, St. Jude Center of Excellence for Influenza Research and Surveillance (CEIRS)	A.E.; Ali; El-Guindy; El-Sayes, M.; El-Shesheny, R.; El-Taweel, A.; Gomaa, M.; Kamel; Kandeil, A.; Kayali, G.; Kayed; Kutkat, O.; M.A.; M.N.; Mahmoud; Mahrous, N.; Moatasim, Y.; Mostafa, A.; N.M.; Naguib, A.; Roshdy; S.H.; Shehata, M.; Showky, S.; W.H.; Webby, R.
EPI_ISL_1418248	CENTRE HOSPITALIER BELLEVUE	CNR Virus des Infections Respiratoires - France SUD	Antonin Bal; Bruno Lina; Gregory Destras; Gwendolyne Burfin; Hadrien Regue; Laurence Josset; Martine Valette; Quentin Semanas
EPI_ISL_1418243, EPI_ISL_1461056, EPI_ISL_1461058, EPI_ISL_1526376 to 1526377, EPI_ISL_1526446, EPI_ISL_1526450 to 1526452, EPI_ISL_1526454 to 1526455			

see above	CENTRE HOSPITALIER DE BOURG EN BRESSE	CNR Virus des Infections Respiratoires - France SUD	Antonin Bal; Bruno Lina; Gregory Destras; Gwendolyne Burfin; Hadrien Regue; Laurence Josset; Martine Valette; Quentin Semanas
EPI_ISL_1418204, EPI_ISL_1418206, EPI_ISL_1418208, EPI_ISL_1433820, EPI_ISL_1461013, EPI_ISL_1461017 to 1461018, EPI_ISL_1461046 to 1461048, EPI_ISL_1526107 to 1526108, EPI_ISL_1526167, EPI_ISL_1526196, EPI_ISL_1526253 to 1526254, EPI_ISL_1526369, EPI_ISL_1526392 to 1526394			
see above	CENTRE HOSPITALIER DU HAUT BUGEY	CNR Virus des Infections Respiratoires - France SUD	Antonin Bal; Bruno Lina; Gregory Destras; Gwendolyne Burfin; Hadrien Regue; Laurence Josset; Martine Valette; Quentin Semanas
EPI_ISL_1461165	CENTRE HOSPITALIER E. BOREL	CNR Virus des Infections Respiratoires - France SUD	Antonin Bal; Bruno Lina; Gregory Destras; Gwendolyne Burfin; Hadrien Regue; Laurence Josset; Martine Valette; Quentin Semanas
EPI_ISL_1418230, EPI_ISL_1418240, EPI_ISL_1461020 to 1461021, EPI_ISL_1461039, EPI_ISL_1461122, EPI_ISL_1461142 to 1461144, EPI_ISL_1461152, EPI_ISL_1526075, EPI_ISL_1526105, EPI_ISL_1526127, EPI_ISL_1526162, EPI_ISL_1526183, EPI_ISL_1526202 to 1526203, EPI_ISL_1526359, EPI_ISL_1526374, EPI_ISL_1526406 to 1526409, EPI_ISL_1526412, EPI_ISL_1526447 to 1526449			
see above	CENTRE HOSPITALIER LUCIEN HUSSEL	CNR Virus des Infections Respiratoires - France SUD	Antonin Bal; Bruno Lina; Gregory Destras; Gwendolyne Burfin; Hadrien Regue; Laurence Josset; Martine Valette; Quentin Semanas
EPI_ISL_1433793, EPI_ISL_1434243, EPI_ISL_1461033, EPI_ISL_1461045, EPI_ISL_1461148, EPI_ISL_1526094, EPI_ISL_1526113, EPI_ISL_1526142, EPI_ISL_1526177, EPI_ISL_1526220, EPI_ISL_1526244, EPI_ISL_1526248, EPI_ISL_1526258, EPI_ISL_1526277, EPI_ISL_1526328, EPI_ISL_1526355, EPI_ISL_1526424 to 1526425, EPI_ISL_1526453			
see above	CENTRE HOSPITALIER PIERRE OUDOT	CNR Virus des Infections Respiratoires - France SUD	Antonin Bal; Bruno Lina; Gregory Destras; Gwendolyne Burfin; Hadrien Regue; Laurence Josset; Martine Valette; Quentin Semanas
EPI_ISL_1461071	CENTRE HOSPITALIER POITIERS	CNR Virus des Infections Respiratoires - France SUD	Antonin Bal; Bruno Lina; Gregory Destras; Gwendolyne Burfin; Hadrien Regue; Laurence Josset; Martine Valette; Quentin Semanas
EPI_ISL_1418247, EPI_ISL_1461008, EPI_ISL_1461037, EPI_ISL_1461041, EPI_ISL_1461072, EPI_ISL_1461166 to 1461167, EPI_ISL_1526120 to 1526122, EPI_ISL_1526155 to 1526156, EPI_ISL_1526170 to 1526173, EPI_ISL_1526201, EPI_ISL_1526204 to 1526207, EPI_ISL_1526350, EPI_ISL_1526368, EPI_ISL_1526420, EPI_ISL_1526441 to 1526445			
see above	CENTRE HOSPITALIER ST JOSEPH ST LUC	CNR Virus des Infections Respiratoires - France SUD	Antonin Bal; Bruno Lina; Gregory Destras; Gwendolyne Burfin; Hadrien Regue; Laurence Josset; Martine Valette; Quentin Semanas
EPI_ISL_1418256	CENTRE HOSPITALIER SUD GIRONDE	CNR Virus des Infections Respiratoires - France SUD	Antonin Bal; Bruno Lina; Gregory Destras; Gwendolyne Burfin; Hadrien Regue; Laurence Josset; Martine Valette; Quentin Semanas
EPI_ISL_1469658	CENTRO DE ESPECIALIDADES TRIUNFO	Epiclin	Ana Paula Muterle; Carolina Comerlato; Eliana Márcia Da Ros Wendland; Fernando Hayashi Sant'Anna; Janira Prichula; Juliana Comerlato
EPI_ISL_1511237	CENTRO DE MILITAR DE VETERINARIA	Instituto de Salud Carlos III	A. Monzón; DANIEL; F. Casas; I. Jiménez; I.FERNANDEZ MOREIRA; Iglesias-Caballero; M. Sandonís; P. Zaballos; S. Camarero; S. Cuesta; S. Pozo; S. Varona; V. Vázquez-Morón
EPI_ISL_1445147 to 1445152	CENTRO DE REFERENCIA DO IDOSO DR HUMBERTO MENDES DE CARVALHO	Instituto Butantan / Mendelics	Antonio Jorge Martins; Bibiana Santos; Claudia Renata dos Santos Barros; David Schlesinger; Debora Botequio Moretti; Dimas Tadeu Covas; Elaine Cristina Marqueze; Elaine Vieira dos Santos; Erika Freitas; Evandra Strazza Rodrigues; Flavia Aburjaile; José Salvatore Leister Patané; João Paulo Kitajima; Luiz Carlos Junior de Alcantara; Maria Carolina Elias; Marta Giovanetti; Rafael dos Santos Bezerra; Raul Machado Neto; Ricardo Haddad; Rodrigo Tocantins Calado.; Sandra Coccuzzo Sampaio; Simone Kashima; Svetoslav Nanev Slavov; Vagner Fonseca; Vincent Louis Viala
EPI_ISL_1469596, EPI_ISL_1469642, EPI_ISL_1469730, EPI_ISL_1469732, EPI_ISL_1469737, EPI_ISL_1469742, EPI_ISL_1479125			
see above	CENTRO DE REFERENCIA EM SINDROMES GRIPAIS	Epiclin	Ana Paula Muterle; Carolina Comerlato; Eliana Márcia Da Ros Wendland; Fernando Hayashi Sant'Anna; Janira Prichula; Juliana Comerlato
EPI_ISL_1445195	CENTRO DE SAUDE DE BORA	Instituto Butantan / Mendelics	Antonio Jorge Martins; Bibiana Santos; Claudia Renata dos Santos Barros; David Schlesinger; Debora Botequio Moretti; Dimas Tadeu Covas; Elaine Cristina Marqueze; Elaine Vieira dos Santos; Erika Freitas; Evandra Strazza Rodrigues; Flavia Aburjaile; José Salvatore Leister Patané; João Paulo Kitajima; Luiz Carlos Junior de Alcantara; Maria Carolina Elias; Marta Giovanetti; Rafael dos Santos Bezerra; Raul Machado Neto; Ricardo Haddad; Rodrigo Tocantins Calado.; Sandra Coccuzzo Sampaio; Simone Kashima; Svetoslav Nanev Slavov; Vagner Fonseca; Vincent Louis Viala
EPI_ISL_1445197	CENTRO DE SAUDE DE JULIO MESQUITA	Instituto Butantan / Mendelics	Antonio Jorge Martins; Bibiana Santos; Claudia Renata dos Santos Barros; David Schlesinger; Debora Botequio Moretti; Dimas Tadeu Covas; Elaine Cristina Marqueze; Elaine Vieira dos Santos; Erika Freitas; Evandra Strazza Rodrigues; Flavia Aburjaile; José Salvatore Leister Patané; João Paulo Kitajima; Luiz Carlos Junior de Alcantara; Maria Carolina Elias; Marta Giovanetti; Rafael dos Santos Bezerra; Raul Machado Neto; Ricardo Haddad; Rodrigo Tocantins Calado.; Sandra Coccuzzo Sampaio; Simone Kashima; Svetoslav Nanev Slavov; Vagner Fonseca; Vincent Louis Viala
EPI_ISL_1469662, EPI_ISL_1469706	CENTRO DE SAUDE DR BRUNO CASSEL	Epiclin	Ana Paula Muterle; Carolina Comerlato; Eliana Márcia Da Ros Wendland; Fernando Hayashi Sant'Anna; Janira Prichula; Juliana Comerlato
EPI_ISL_1445075 to 1445079	CENTRO DE SAUDE II DR GABRIEL MESQUITA VARGEM GDE DO SUL	Instituto Butantan / Mendelics	Antonio Jorge Martins; Bibiana Santos; Claudia Renata dos Santos Barros; David Schlesinger; Debora Botequio Moretti; Dimas Tadeu Covas; Elaine Cristina Marqueze; Elaine Vieira dos Santos; Erika Freitas; Evandra Strazza Rodrigues; Flavia Aburjaile; José Salvatore Leister Patané; João Paulo Kitajima; Luiz Carlos Junior de Alcantara; Maria Carolina Elias; Marta Giovanetti; Rafael dos Santos Bezerra; Raul Machado Neto; Ricardo Haddad; Rodrigo Tocantins Calado.; Sandra Coccuzzo Sampaio; Simone Kashima; Svetoslav Nanev Slavov; Vagner Fonseca; Vincent Louis Viala
EPI_ISL_1445086, EPI_ISL_1445089	CENTRO DE SAUDE II MAIRINQUE MAIRINQUE	Instituto Butantan / Mendelics	Antonio Jorge Martins; Bibiana Santos; Claudia Renata dos Santos Barros; David Schlesinger; Debora Botequio Moretti; Dimas Tadeu Covas; Elaine Cristina Marqueze; Elaine Vieira dos Santos; Erika Freitas; Evandra Strazza Rodrigues; Flavia Aburjaile; José Salvatore Leister Patané; João Paulo Kitajima; Luiz Carlos Junior de Alcantara; Maria Carolina Elias; Marta Giovanetti; Rafael dos Santos Bezerra; Raul Machado Neto; Ricardo Haddad; Rodrigo Tocantins Calado.; Sandra Coccuzzo Sampaio; Simone Kashima; Svetoslav Nanev Slavov; Vagner Fonseca; Vincent Louis Viala
EPI_ISL_1445199	CENTRO DE SAUDE III SALES OLIVEIRA	Instituto Butantan / Mendelics	Antonio Jorge Martins; Bibiana Santos; Claudia Renata dos Santos Barros; David Schlesinger; Debora Botequio Moretti; Dimas Tadeu Covas; Elaine Cristina Marqueze; Elaine Vieira dos Santos; Erika Freitas; Evandra Strazza Rodrigues; Flavia Aburjaile; José Salvatore Leister Patané; João Paulo Kitajima; Luiz Carlos Junior de Alcantara; Maria Carolina Elias; Marta Giovanetti; Rafael dos Santos Bezerra; Raul Machado Neto; Ricardo Haddad; Rodrigo Tocantins Calado.; Sandra Coccuzzo Sampaio; Simone Kashima; Svetoslav Nanev Slavov; Vagner Fonseca; Vincent Louis Viala
EPI_ISL_1445081	CENTRO DE SAUDE SAO ROQUE DR JOSE CARVALHO BRITO	Instituto Butantan / Mendelics	Antonio Jorge Martins; Bibiana Santos; Claudia Renata dos Santos Barros; David Schlesinger; Debora Botequio Moretti; Dimas Tadeu Covas; Elaine Cristina Marqueze; Elaine Vieira dos Santos; Erika Freitas; Evandra Strazza Rodrigues; Flavia Aburjaile; José Salvatore Leister Patané; João Paulo Kitajima; Luiz Carlos Junior de Alcantara; Maria Carolina Elias; Marta Giovanetti; Rafael dos Santos Bezerra; Raul Machado Neto; Ricardo Haddad; Rodrigo Tocantins Calado.; Sandra Coccuzzo Sampaio; Simone Kashima; Svetoslav Nanev Slavov; Vagner Fonseca; Vincent Louis Viala
EPI_ISL_1469574, EPI_ISL_1469628, EPI_ISL_1469682, EPI_ISL_1469698	CENTRO DE SERVICOS ESPECIALIZADOS SANTA RITA DE CASSIA	Epiclin	Ana Paula Muterle; Carolina Comerlato; Eliana Márcia Da Ros Wendland; Fernando Hayashi Sant'Anna; Janira Prichula; Juliana Comerlato
EPI_ISL_1511224 to 1511225	CENTRO MILITAR DE VETERINARIA	Instituto de Salud Carlos III	A. Monzón; DANIEL; F. Casas; I. FERNANDEZ MOREIRA; I. Jiménez; M. Camarero; P. Zaballos; S. Cuesta; S. Iglesias-Caballero; S. Pozo; S. Varona; Sandonís; V. Vázquez-Morón
EPI_ISL_1469584, EPI_ISL_1469629	CENTRO MUNICIPAL DE SAUDE DE ROLANTE	Epiclin	Ana Paula Muterle; Carolina Comerlato; Eliana Márcia Da Ros Wendland; Fernando Hayashi Sant'Anna; Janira Prichula; Juliana Comerlato
EPI_ISL_445266 to 445267	CENTRO ONCOLOGICO DEL NORTE	Instituto de Salud Publica de Chile	Alejandra Acevedo; Andrés E Castillo; Bárbara Parra; Carolina Tambley; Gabriel Leal; Jaime Lagos; Jorge Fernandez; Loredana Arata; Patricia Bustos; Paz Tapia; Rodrigo Fasce; Winston Andrade
EPI_ISL_1461004	CERBALLIANCE CHARENTES SAINTES	CNR Virus des Infections Respiratoires - France SUD	Antonin Bal; Bruno Lina; Gregory Destras; Gwendolyne Burfin; Hadrien Regue; Laurence Josset; Martine Valette; Quentin Semanas
EPI_ISL_445316	CESFAM BALMACEDA DE RENCA	Instituto de Salud Publica de Chile	Alejandra Acevedo; Andrés E Castillo; Bárbara Parra; Carolina Tambley; Gabriel Leal; Jaime Lagos; Jorge Fernandez; Loredana Arata; Patricia Bustos; Paz Tapia; Rodrigo Fasce; Winston Andrade
EPI_ISL_1520166	CGH Medical Center	Illinois Department of Public Health - Chicago Lab	Ira Heimler; Vineet K. Dhiman
EPI_ISL_1478855, EPI_ISL_1553252 to 1553260	CGH Medical Center	Illinois Department of Public Health - Springfield Lab	Bryan Sim; Gordon McCall
EPI_ISL_1461074	CH ALBI	CNR Virus des Infections Respiratoires - France SUD	Antonin Bal; Bruno Lina; Gregory Destras; Gwendolyne Burfin; Hadrien Regue; Laurence Josset; Martine Valette; Quentin Semanas
EPI_ISL_1418257 to 1418259, EPI_ISL_1526351	CH ANTIBES - JUAN LES PINS	CNR Virus des Infections Respiratoires - France SUD	Antonin Bal; Bruno Lina; Gregory Destras; Gwendolyne Burfin; Hadrien Regue; Laurence Josset; Martine Valette; Quentin Semanas
EPI_ISL_1526071	CH ARDECHE NORD	CNR Virus des Infections Respiratoires - France SUD	Antonin Bal; Bruno Lina; Gregory Destras; Gwendolyne Burfin; Hadrien Regue; Laurence Josset; Martine Valette; Quentin Semanas
EPI_ISL_421455, EPI_ISL_421479 to 421480, EPI_ISL_421485 to 421486	CH Barreiro Montijo	Instituto Nacional de Saude (INSA)	Guiomar et al
EPI_ISL_1396708 to 1396710, EPI_ISL_1396739	CH Béthune	CHU Lille - Laboratoire de Virologie	AIT YAHYA Emilie; ALIDJINOU Enagnon Kazali; BOCKET Laurence; CREPIN Michel; DEMAY Christophe; ENGELMANN Ilka; GEFFROY Sandrine; GUIGON Aurélie; LAMBERT Valérie; LAZREK Mouna; NOBILLIAUX Florian; PREVOST Brigitte; TCHANTCHOU NJOSSE YANICK; THUILLIER Caroline; TINEZ Claire

EPI_ISL_1433837	CH CAHORS	CNR Virus des Infections Respiratoires - France SUD	Antonin Bal; Bruno Lina; Gregory Destras; Gwendolyne Burfin; Hadrien Regue; Laurence Josset; Martine Valette; Quentin Semanas
EPI_ISL_1396682 to 1396684, EPI_ISL_1396704	CH Calais	CHU Lille - Laboratoire de Virologie	AIT YAHYA Emilie; ALIDJINOU Enagnon Kazali; BOCKET Laurence; CREPIN Michel; DEMAY Christophe; ENGELMANN Ilka; GEFFROY Sandrine; GUIGON Aurélie; LAMBERT Valérie; LAZREK Mouna; NOBILLIAUX Florian; PREVOST Brigitte; TCHANTCHOU NJOSSE YANICK; THUILLIER Caroline; TINEZ Claire
EPI_ISL_420041, EPI_ISL_420049 to 420050, EPI_ISL_420056 to 420057, EPI_ISL_421500, EPI_ISL_421509 to 421511, EPI_ISL_428353, EPI_ISL_428359 to 428360, EPI_ISL_443309, EPI_ISL_443316			
see above	CH Compiègne Laboratoire de Biologie	National Reference Center for Viruses of Respiratory Infections, Institut Pasteur, Paris	Angela Brisebarre; Etienne Simon-Lorière; Flora Donati; Marion Barbet; Maud Vanpeene; Mélanie Albert; Méline Bizard; Olivia Raulin; Raulin Olivia; Sylvie Behillil; Sylvie van der Werf; Vincent Enouf
EPI_ISL_1396678, EPI_ISL_1396693, EPI_ISL_1396711 to 1396712, EPI_ISL_1396720, EPI_ISL_1396738	CH Douai	CHU Lille - Laboratoire de Virologie	AIT YAHYA Emilie; ALIDJINOU Enagnon Kazali; BOCKET Laurence; CREPIN Michel; DEMAY Christophe; ENGELMANN Ilka; GEFFROY Sandrine; GUIGON Aurélie; LAMBERT Valérie; LAZREK Mouna; NOBILLIAUX Florian; PREVOST Brigitte; TCHANTCHOU NJOSSE YANICK; THUILLIER Caroline; TINEZ Claire
EPI_ISL_1396675, EPI_ISL_1396703, EPI_ISL_1396735, EPI_ISL_1396740	CH Dunkerque	CHU Lille - Laboratoire de Virologie	AIT YAHYA Emilie; ALIDJINOU Enagnon Kazali; BOCKET Laurence; CREPIN Michel; DEMAY Christophe; ENGELMANN Ilka; GEFFROY Sandrine; GUIGON Aurélie; LAMBERT Valérie; LAZREK Mouna; NOBILLIAUX Florian; PREVOST Brigitte; TCHANTCHOU NJOSSE YANICK; THUILLIER Caroline; TINEZ Claire
EPI_ISL_1461027, EPI_ISL_1461073, EPI_ISL_1461135, EPI_ISL_1461153	CH GIVORS	CNR Virus des Infections Respiratoires - France SUD	Antonin Bal; Bruno Lina; Gregory Destras; Gwendolyne Burfin; Hadrien Regue; Laurence Josset; Martine Valette; Quentin Semanas
EPI_ISL_416493, EPI_ISL_420044, EPI_ISL_420053, EPI_ISL_428350	CH Jean de Navarre Laboratoire de Biologie	National Reference Center for Viruses of Respiratory Infections, Institut Pasteur, Paris	Angela Brisebarre; Etienne Simon-Lorière; Flora Donati; Marion Barbet; Maud Vanpeene; Mélanie Albert; Méline Bizard; Mélnie Albert; Sylvie Behillil; Sylvie van der Werf; Vincent Enouf
EPI_ISL_428358, EPI_ISL_428366	CH Jeanne de Navarre Laboratoire de Biologie	National Reference Center for Viruses of Respiratory Infections, Institut Pasteur, Paris	Angela Brisebarre; Etienne Simon-Lorière; Flora Donati; Marion Barbet; Maud Vanpeene; Mélanie Albert; Méline Bizard; Sylvie Behillil; Sylvie van der Werf; Vincent Enouf
EPI_ISL_1396685	CH Lens	CHU Lille - Laboratoire de Virologie	AIT YAHYA Emilie; ALIDJINOU Enagnon Kazali; BOCKET Laurence; CREPIN Michel; DEMAY Christophe; ENGELMANN Ilka; GEFFROY Sandrine; GUIGON Aurélie; LAMBERT Valérie; LAZREK Mouna; NOBILLIAUX Florian; PREVOST Brigitte; TCHANTCHOU NJOSSE YANICK; THUILLIER Caroline; TINEZ Claire
EPI_ISL_1434014, EPI_ISL_1434201, EPI_ISL_1434203, EPI_ISL_1434257	CH METROPOLE SAVOIE	CNR Virus des Infections Respiratoires - France SUD	Antonin Bal; Bruno Lina; Gregory Destras; Gwendolyne Burfin; Hadrien Regue; Laurence Josset; Martine Valette; Quentin Semanas
EPI_ISL_1433935, EPI_ISL_1433937, EPI_ISL_1434077	CH MOULINS YZEURE	CNR Virus des Infections Respiratoires - France SUD	Antonin Bal; Bruno Lina; Gregory Destras; Gwendolyne Burfin; Hadrien Regue; Laurence Josset; Martine Valette; Quentin Semanas
EPI_ISL_1434195	CH NIORT	CNR Virus des Infections Respiratoires - France SUD	Antonin Bal; Bruno Lina; Gregory Destras; Gwendolyne Burfin; Hadrien Regue; Laurence Josset; Martine Valette; Quentin Semanas
EPI_ISL_1396679 to 1396680, EPI_ISL_1396713	CH Roubaix	CHU Lille - Laboratoire de Virologie	AIT YAHYA Emilie; ALIDJINOU Enagnon Kazali; BOCKET Laurence; CREPIN Michel; DEMAY Christophe; ENGELMANN Ilka; GEFFROY Sandrine; GUIGON Aurélie; LAMBERT Valérie; LAZREK Mouna; NOBILLIAUX Florian; PREVOST Brigitte; TCHANTCHOU NJOSSE YANICK; THUILLIER Caroline; TINEZ Claire
EPI_ISL_1434044, EPI_ISL_1457763	CH SAINTONGES	CNR Virus des Infections Respiratoires - France SUD	Antonin Bal; Bruno Lina; Gregory Destras; Gwendolyne Burfin; Hadrien Regue; Laurence Josset; Martine Valette; Quentin Semanas
EPI_ISL_1396676 to 1396677, EPI_ISL_1396691 to 1396692, EPI_ISL_1396721	CH Saint-Philibert	CHU Lille - Laboratoire de Virologie	AIT YAHYA Emilie; ALIDJINOU Enagnon Kazali; BOCKET Laurence; CREPIN Michel; DEMAY Christophe; ENGELMANN Ilka; GEFFROY Sandrine; GUIGON Aurélie; LAMBERT Valérie; LAZREK Mouna; NOBILLIAUX Florian; PREVOST Brigitte; TCHANTCHOU NJOSSE YANICK; THUILLIER Caroline; TINEZ Claire
EPI_ISL_1396681, EPI_ISL_1396689, EPI_ISL_1396718	CH Seclin	CHU Lille - Laboratoire de Virologie	AIT YAHYA Emilie; ALIDJINOU Enagnon Kazali; BOCKET Laurence; CREPIN Michel; DEMAY Christophe; ENGELMANN Ilka; GEFFROY Sandrine; GUIGON Aurélie; LAMBERT Valérie; LAZREK Mouna; NOBILLIAUX Florian; PREVOST Brigitte; TCHANTCHOU NJOSSE YANICK; THUILLIER Caroline; TINEZ Claire
EPI_ISL_1396690	CH Tourcoing	CHU Lille - Laboratoire de Virologie	AIT YAHYA Emilie; ALIDJINOU Enagnon Kazali; BOCKET Laurence; CREPIN Michel; DEMAY Christophe; ENGELMANN Ilka; GEFFROY Sandrine; GUIGON Aurélie; LAMBERT Valérie; LAZREK Mouna; NOBILLIAUX Florian; PREVOST Brigitte; TCHANTCHOU NJOSSE YANICK; THUILLIER Caroline; TINEZ Claire
EPI_ISL_1418260 to 1418261	CH VALENCE	CNR Virus des Infections Respiratoires - France SUD	Antonin Bal; Bruno Lina; Gregory Destras; Gwendolyne Burfin; Hadrien Regue; Laurence Josset; Martine Valette; Quentin Semanas
EPI_ISL_421482 to 421484	CH VN Gaia - Espinho	Instituto Nacional de Saude (INSA)	Guiomar et al
EPI_ISL_1396736 to 1396737	CH Valenciennes	CHU Lille - Laboratoire de Virologie	AIT YAHYA Emilie; ALIDJINOU Enagnon Kazali; BOCKET Laurence; CREPIN Michel; DEMAY Christophe; ENGELMANN Ilka; GEFFROY Sandrine; GUIGON Aurélie; LAMBERT Valérie; LAZREK Mouna; NOBILLIAUX Florian; PREVOST Brigitte; TCHANTCHOU NJOSSE YANICK; THUILLIER Caroline; TINEZ Claire
EPI_ISL_1526097, EPI_ISL_1526102, EPI_ISL_1526112, EPI_ISL_1526123, EPI_ISL_1526128, EPI_ISL_1526135, EPI_ISL_1526138 to 1526139, EPI_ISL_1526161, EPI_ISL_1526168, EPI_ISL_1526188, EPI_ISL_1526217, EPI_ISL_1526225, EPI_ISL_1526299, EPI_ISL_1526341, EPI_ISL_1526363, EPI_ISL_1526383, EPI_ISL_1526404			
see above	CH de GIVORS	CNR Virus des Infections Respiratoires - France SUD	Antonin Bal; Bruno Lina; Gregory Destras; Gwendolyne Burfin; Hadrien Regue; Laurence Josset; Martine Valette; Quentin Semanas
EPI_ISL_1396829 to 1396833	CH. CHARLES NICOLLE	Department of Virology, Henri Mondor University Hospital, Assistance Publique Hôpitaux de Paris, Université Paris-Est Créteil, INSERM U955	Alexandre Soulier; Christophe Rodriguez; Elisabeth Trawinski; Guillaume Gricourt; Jean-Michel Pawlotsky; Melissa N'Debi; Slim Fourati; Vanessa Demontant
EPI_ISL_1396812	CH. CLEMENCEAU	Department of Virology, Henri Mondor University Hospital, Assistance Publique Hôpitaux de Paris, Université Paris-Est Créteil, INSERM U955	Alexandre Soulier; Christophe Rodriguez; Elisabeth Trawinski; Guillaume Gricourt; Jean-Michel Pawlotsky; Melissa N'Debi; Slim Fourati; Vanessa Demontant
EPI_ISL_1404509 to 1404510	CH. ROBERT DEBRE	Department of Virology, Henri Mondor University Hospital, Assistance Publique Hôpitaux de Paris, Université Paris-Est Créteil, INSERM U955	Alexandre Soulier; Christophe Rodriguez; Elisabeth Trawinski; Guillaume Gricourt; Jean-Michel Pawlotsky; Melissa N'Debi; Slim Fourati; Vanessa Demontant
EPI_ISL_1396837 to 1396843, EPI_ISL_1396884 to 1396890, EPI_ISL_1404463 to 1404474, EPI_ISL_1404477 to 1404481, EPI_ISL_1404565	CH.INTERCOMMUNAL DE CRETEIL	Department of Virology, Henri Mondor University Hospital, Assistance Publique Hôpitaux de Paris, Université Paris-Est Créteil, INSERM U955	Alexandre Soulier; Christophe Rodriguez; Elisabeth Trawinski; Guillaume Gricourt; Jean-Michel Pawlotsky; Melissa N'Debi; Slim Fourati; Vanessa Demontant
EPI_ISL_1396892, EPI_ISL_1404451 to 1404458	CH.de LONS LE SAUNIER	Department of Virology, Henri Mondor University Hospital, Assistance Publique Hôpitaux de Paris, Université Paris-Est Créteil, INSERM U955	Alexandre Soulier; Christophe Rodriguez; Elisabeth Trawinski; Guillaume Gricourt; Jean-Michel Pawlotsky; Melissa N'Debi; Slim Fourati; Vanessa Demontant
EPI_ISL_418006	CHBarreiro Montijo	Instituto Nacional de Saude (INSA)	Guiomar et al
EPI_ISL_1392648 to 1392649	CHD Vendée	CHU Nantes Virology	Audrey Rodallec; Berthe-Marie Imbert-Marcille; Cecile Le Boterff; Celine Bressollette-Bodin; Thomas Drumel
EPI_ISL_1404461 to 1404462	CHI VILLENEUVE ST GEORGES	Department of Virology, Henri Mondor University Hospital, Assistance Publique Hôpitaux de Paris, Université Paris-Est Créteil, INSERM U955	Alexandre Soulier; Christophe Rodriguez; Elisabeth Trawinski; Guillaume Gricourt; Jean-Michel Pawlotsky; Melissa N'Debi; Slim Fourati; Vanessa Demontant
EPI_ISL_418017	CHMT	Instituto Nacional de Saude (INSA)	Guiomar et al
EPI_ISL_1492732	CHR de La Citadelle	GIGA Medical Genomics	Bouchra Boujemla; Cécile Meex; Keith Durkin; Maria Artesi; Marie-Pierre Hayette; Nathalie Renotte; Pierrette Melin; Raphaël Boreux; Sébastien Bontems; Vincent Bours

EPI_ISL_1494954	CHRISTUS SINERGIA - Clínica Farallones	Instituto Nacional de Salud- Dirección de Investigación en Salud Pública	Carlos Franco-Muñoz; Carmen Osorio; Diana Malo; Diego A. Álvarez-Díaz; Diego Andrés Prada; Gerardo Santamaría; Hector Alejandro Ruiz-Moreno; Jhonnatan Reales-González; Juan Camilo Martinez; Julian Naizaque; Katherine Laiton-Donato; Lisseth Pardo; Magdalena Wiesner; Marcela Mercado-Reyes; Maria T. Herrera-Sepúlveda; Marta Lopez Blanco; Martha Lucia Ospina Martinez; Paola Rojas; Sergio Gomez; Sheryll Corchuelo; Ángela Alarcon Cruz
EPI_ISL_418222	CHRU Bretonneau - Serv. Bacterio-Virol.	National Reference Center for Viruses of Respiratory Infections, Institut Pasteur, Paris	Angela Brisebarre; Etienne Simon-Lorière; Fabiana Gambaro; Flora Donati; Julien Marlet; Marion Barbet; Maud Vanpeene; Mélanie Albert; Méline Bizard; Sylvie Behillil; Sylvie van der Werf; Vincent Enouf
EPI_ISL_416502 to 416513, EPI_ISL_443289 to 443294	CHRU Pontchaillou - Laboratoire de Virologie	National Reference Center for Viruses of Respiratory Infections, Institut Pasteur, Paris	Angela Brisebarre; Etienne Simon-Lorière; Flora Donati; Gisèle Lagathu; Marion Barbet; Maud Vanpeene; Mélanie Albert; Méline Bizard; Mélnie Albert; Sylvie Behillil; Sylvie van der Werf; Vincent Enouf
EPI_ISL_613545 to 613559, EPI_ISL_614281	CHRU Pontchaillou - Laboratoire de Virologie 2, rue Henri Le Guilloux	National Reference Center for Viruses of Respiratory Infections, Institut Pasteur, Paris	Angela Brisebarre; Camille Capel; Etienne Simon-Lorière; Gisèle Lagathu; Marion Barbet; Maud Vanpeene; Méline Bizard; Sylvie Behillil; Sylvie van der Werf; Vincent Enouf
EPI_ISL_418027, EPI_ISL_421453, EPI_ISL_421464 to 421465	CHTMAD	Instituto Nacional de Saude (INSA)	Guiomar et al
EPI_ISL_418219, EPI_ISL_443265 to 443283	CHU - Hôpital Cavale Blanche - Labo. de Virologie	National Reference Center for Viruses of Respiratory Infections, Institut Pasteur, Paris	Angela Brisebarre; Etienne Simon-Lorière; Fabiana Gambaro; Flora Donati; Léa Pilorge; Marion Barbet; Maud Vanpeene; Mélanie Albert; Méline Bizard; Sylvie Behillil; Sylvie van der Werf; Vincent Enouf
EPI_ISL_1433814, EPI_ISL_1433816, EPI_ISL_1433841, EPI_ISL_1433843, EPI_ISL_1433847, EPI_ISL_1433849, EPI_ISL_1433851, EPI_ISL_1433853, EPI_ISL_1433855, EPI_ISL_1433857, EPI_ISL_1433859, EPI_ISL_1433861, EPI_ISL_1433865, EPI_ISL_1433869, EPI_ISL_1433871, EPI_ISL_1433873, EPI_ISL_1433997, EPI_ISL_1433999, EPI_ISL_1434001, EPI_ISL_1457757 to 1457758			
see above	CHU BORDEAUX - GH ST ANDRE	CNR Virus des Infections Respiratoires - France SUD	Antonin Bal; Bruno Lina; Gregory Destras; Gwendolyne Burfin; Hadrien Regue; Laurence Josset; Martine Valette; Quentin Semanas
EPI_ISL_1433894, EPI_ISL_1434018, EPI_ISL_1434021, EPI_ISL_1434025, EPI_ISL_1434029 to 1434030, EPI_ISL_1434035, EPI_ISL_1434037, EPI_ISL_1434239			
see above	CHU CLERMONT FERRAND	CNR Virus des Infections Respiratoires - France SUD	Antonin Bal; Bruno Lina; Gregory Destras; Gwendolyne Burfin; Hadrien Regue; Laurence Josset; Martine Valette; Quentin Semanas
EPI_ISL_418002	CHU Coimbra	Instituto Nacional de Saude (INSA)	Guiomar et al
EPI_ISL_418005	CHU Coimbra - Pediátrico	Instituto Nacional de Saude (INSA)	Guiomar et al
EPI_ISL_1433791, EPI_ISL_1433795, EPI_ISL_1433797, EPI_ISL_1433799, EPI_ISL_1433834, EPI_ISL_1433839, EPI_ISL_1433845, EPI_ISL_1433863, EPI_ISL_1433867, EPI_ISL_1434186, EPI_ISL_1434188, EPI_ISL_1434219, EPI_ISL_1434221, EPI_ISL_1434247, EPI_ISL_1434249, EPI_ISL_1434252			
see above	CHU GRENOBLE	CNR Virus des Infections Respiratoires - France SUD	Antonin Bal; Bruno Lina; Gregory Destras; Gwendolyne Burfin; Hadrien Regue; Laurence Josset; Martine Valette; Quentin Semanas
EPI_ISL_416751 to 416752	CHU Gabriel Montpied	CNR Virus des Infections Respiratoires - France SUD	Alexandre; Antonin; Bal; Bouscambert-Duchamp; Brengel-Pesce; Bruno.; Cheynet; Destras; Florence; Gaymard; Gregory; Josset; Karen; Laurence; Lina; Martine; Maude; Morfin-Sherpa; Valette; Valérie
EPI_ISL_1433931, EPI_ISL_1433933, EPI_ISL_1434071, EPI_ISL_1434073, EPI_ISL_1434075	CHU LIMOGES	CNR Virus des Infections Respiratoires - France SUD	Antonin Bal; Bruno Lina; Gregory Destras; Gwendolyne Burfin; Hadrien Regue; Laurence Josset; Martine Valette; Quentin Semanas
EPI_ISL_1396667 to 1396674, EPI_ISL_1396686 to 1396688, EPI_ISL_1396694 to 1396702, EPI_ISL_1396705 to 1396707, EPI_ISL_1396714 to 1396717, EPI_ISL_1396719, EPI_ISL_1396722 to 1396734			
see above	CHU Lille	CHU Lille - Laboratoire de Virologie	AIT YAHYA Emilie; ALIDJINOU Enagnon Kazali; BOCKET Laurence; CREPIN Michel; DEMAY Christophe; ENGELMANN Ilka; GEFFROY Sandrine; GUIGON Aurélie; LAMBERT Valérie; LAZREK Mouna; NOBILLIAUX Florian; PREVOST Brigitte; TCHANTCHOU NJOSSE YANICK; THUILLIER Caroline; TINEZ Claire
EPI_ISL_1433892, EPI_ISL_1433896, EPI_ISL_1434017, EPI_ISL_1434023, EPI_ISL_1434027, EPI_ISL_1434032, EPI_ISL_1457761			
see above	CHU MONTAUBAN	CNR Virus des Infections Respiratoires - France SUD	Antonin Bal; Bruno Lina; Gregory Destras; Gwendolyne Burfin; Hadrien Regue; Laurence Josset; Martine Valette; Quentin Semanas
EPI_ISL_1461640 to 1461648, EPI_ISL_1524907 to 1524912	CHU NIMES	CHU NIMES	Agathe Boudet; Marie-josée Carles; Sophie Bravo; Stephan Robin
EPI_ISL_1392647	CHU Nantes Virology	CHU Nantes Virology	Audrey Rodallec; Berthe-Marie Imbert-Marcille; Celine Bressollette-Bodin; Thomas Drumel
EPI_ISL_1461191 to 1461194	CHU POITIERS	CNR Virus des Infections Respiratoires - France SUD	Antonin Bal; Bruno Lina; Gregory Destras; Gwendolyne Burfin; Hadrien Regue; Laurence Josset; Martine Valette; Quentin Semanas
EPI_ISL_1409708	CHU Pontchaillou	CHU Pontchaillou	DE TAYRAC Marie; DENOUAL Florent; DUFOUR Marie José; ETCHEVERRY Amandine; GALIBERT Marie Dominique; GROLHIER Claire; JAGLINE Steven; PRONIER Charlotte; SASSI Mohamed; THIBAULT Vincent
EPI_ISL_591099, EPI_ISL_591541 to 591547, EPI_ISL_593855 to 593901, EPI_ISL_603216 to 603220, EPI_ISL_1420815 to 1420821, EPI_ISL_1425079, EPI_ISL_1448425, EPI_ISL_1511115 to 1511120			
see above	CHU Purpan - Laboratoire de Virologie - Institut Fédératif de Biologie	CHU Purpan - Laboratoire de Virologie - Institut Fédératif de Biologie	Agnès Harter; Boyer P.; Carcenac R.; Dubois M.; Harter A.; Izopet J.; Jacques Izopet; Justine Latour; Latour J.; Martine Dubois; Nicolas Jeanne; Noémie Ranger; Pauline Boyer; Pauline Tremeaux; Ranger N.; Romain Carcenac; Tremeaux P.
EPI_ISL_434626 to 434635, EPI_ISL_482879 to 482889	CHU Purpan - Laboratoire de Virologie - Institut Fédératif de Biologie	Laboratoire de virologie - École Nationale Vétérinaire de Toulouse	Guillaume Croville; Jacques Izopet; Jean-Luc Guérin
EPI_ISL_1526231 to 1526243, EPI_ISL_1526245 to 1526247	CHU REUNION	CNR Virus des Infections Respiratoires - France SUD	Antonin Bal; Bruno Lina; Gregory Destras; Gwendolyne Burfin; Hadrien Regue; Laurence Josset; Martine Valette; Quentin Semanas
EPI_ISL_1433785, EPI_ISL_1433789, EPI_ISL_1433824, EPI_ISL_1433826, EPI_ISL_1433828, EPI_ISL_1433830, EPI_ISL_1433832 to 1433833, EPI_ISL_1433836, EPI_ISL_1433875, EPI_ISL_1433989, EPI_ISL_1433991, EPI_ISL_1433993, EPI_ISL_1433995, EPI_ISL_1461015 to 1461016, EPI_ISL_1461181, EPI_ISL_1461188, EPI_ISL_1461190			
see above	CHU ST ETIENNE HOPITAL NORD	CNR Virus des Infections Respiratoires - France SUD	Antonin Bal; Bruno Lina; Gregory Destras; Gwendolyne Burfin; Hadrien Regue; Laurence Josset; Martine Valette; Quentin Semanas
EPI_ISL_535732, EPI_ISL_535801	CHU Sainte-Justine	Laboratoire de santé publique du Québec	Guillaume Bourque; Ioannis Ragoussis; Jesse Shapiro; Mark Lathrop and Michel Roger; Mark Lathrop and Michel Roger on behalf of the CoVSeQ research group; Sandrine Moreira
EPI_ISL_1461111 to 1461112, EPI_ISL_1461114, EPI_ISL_1461116 to 1461120	CHU TOULOUSE	CNR Virus des Infections Respiratoires - France SUD	Antonin Bal; Bruno Lina; Gregory Destras; Gwendolyne Burfin; Hadrien Regue; Laurence Josset; Martine Valette; Quentin Semanas
EPI_ISL_443261 to 443264	CHU de Dijon - Laboratoire de Virologie	National Reference Center for Viruses of Respiratory Infections, Institut Pasteur, Paris	Angela Brisebarre; Etienne Simon-Lorière; Flora Donati; Jean-Baptiste Bour; Marion Barbet; Maud Vanpeene; Mélanie Albert; Méline Bizard; Sylvie Behillil; Sylvie van der Werf; Vincent Enouf
EPI_ISL_418024	CHUA - Faro	Instituto Nacional de Saude (INSA)	Guiomar et al
EPI_ISL_1448431 to 1448508	CHUGA-IBP-laboratoire de Virologie	IBP-laboratoire de virologie	Anne Signori -Schmuck; Anne-Karen Faure; Aurélie Truffot; Benjamin Nemoz; Julien Andréani; Julien Lupo; Léa Ponderand; Pascal Poignard; Patrice Morand; Raphaël Germi; Sylvie Larrat
EPI_ISL_535736 to 535737, EPI_ISL_535741, EPI_ISL_535772 to 535775, EPI_ISL_535781 to 535782, EPI_ISL_535784 to 535785, EPI_ISL_535822 to 535825, EPI_ISL_535828			
see above	CHUL-LABO MULTI / MICRO	Laboratoire de santé publique du Québec	Guillaume Bourque; Ioannis Ragoussis; Jesse Shapiro; Mark Lathrop and Michel Roger; Mark Lathrop and Michel Roger on behalf of the CoVSeQ research group; Sandrine Moreira
EPI_ISL_417988, EPI_ISL_417990 to 417991, EPI_ISL_417994 to 417996, EPI_ISL_418010 to 418016	CHULC - H Curry Cabral	Instituto Nacional de Saude (INSA)	Guiomar et al
EPI_ISL_417992 to 417993	CHULC - H D Estefania	Instituto Nacional de Saude (INSA)	Guiomar et al
EPI_ISL_535726, EPI_ISL_535729 to 535730, EPI_ISL_535735, EPI_ISL_535740, EPI_ISL_535753 to 535754, EPI_ISL_535760, EPI_ISL_535783, EPI_ISL_535789, EPI_ISL_535802, EPI_ISL_535806, EPI_ISL_535814, EPI_ISL_535826 to 535827, EPI_ISL_535830, EPI_ISL_535832			
see above	CHUM - Microbiologie - Hôpital Saint-Luc	Laboratoire de santé publique du Québec	Guillaume Bourque; Ioannis Ragoussis; Jesse Shapiro; Mark Lathrop and Michel Roger; Mark Lathrop and Michel Roger on behalf of the CoVSeQ

			research group; Sandrine Moreira
EPI_ISL_1517297 to 1517300, EPI_ISL_1517304 to 1517305, EPI_ISL_1517307 to 1517320, EPI_ISL_1517322 to 1517334, EPI_ISL_1517336 to 1517337, EPI_ISL_1517340 to 1517357, EPI_ISL_1517359 to 1517372, EPI_ISL_1517374 to 1517383, EPI_ISL_1545184, EPI_ISL_1545187, EPI_ISL_1545190 to 1545197, EPI_ISL_1545199 to 1545200, EPI_ISL_1545202 to 1545206, EPI_ISL_1545208 to 1545220, EPI_ISL_1545222, EPI_ISL_1545224 to 1545231, EPI_ISL_1545233 to 1545235, EPI_ISL_1545237 to 1545247, EPI_ISL_1545257			
see above	CHUV	Laboratory of genomics and metagenomics	Claire Bertelli; Damien Jacot; Gilbert Greub; SÃ©bastien Aeby; Sébastien Aeby; Trestan Pillonel
EPI_ISL_1403082 to 1403087, EPI_ISL_1403089 to 1403092, EPI_ISL_1443187 to 1443189, EPI_ISL_1443191 to 1443195, EPI_ISL_1448527 to 1448528, EPI_ISL_1451467, EPI_ISL_1451470, EPI_ISL_1451473, EPI_ISL_1451476, EPI_ISL_1451479, EPI_ISL_1451482, EPI_ISL_1451485, EPI_ISL_1451488, EPI_ISL_1451491, EPI_ISL_1451494, EPI_ISL_1454688, EPI_ISL_1545445 to 1545450, EPI_ISL_1545632 to 1545641, EPI_ISL_1545643 to 1545667			
see above	CHWAPI - SITE NOTRE DAME	Institut de Pathologie et Genetique (IPG)	Jérémie Gras; Pascale Hilbert
EPI_ISL_487446 to 487466	CICM-Mali	Bundeswehr Institut of Microbiology	Antwerpen; Bestehorn-Willmann; Dürr; Heitzer; Kouriba; Maiga; Quedraogo; Rehn; Sangaré; Sogodogo; Traoré; Walter; Wölfel; Zimmermann
EPI_ISL_445245, EPI_ISL_445248, EPI_ISL_445250, EPI_ISL_445253 to 445255, EPI_ISL_445258, EPI_ISL_445260	CLINICA ALEMANA DE SANTIAGO S.A.	Instituto de Salud Publica de Chile	Alejandra Acevedo; Andrés E Castillo; Bárbara Parra; Carolina Tambley; Gabriel Leal; Jaime Lagos; Jorge Fernandez; Loredana Arata; Patricia Bustos; Paz Tapia; Rodrigo Fasce; Winston Andrade
EPI_ISL_445272, EPI_ISL_445285, EPI_ISL_445287	CLINICA CIUDAD DEL MAR	Instituto de Salud Publica de Chile	Alejandra Acevedo; Andrés E Castillo; Bárbara Parra; Carolina Tambley; Gabriel Leal; Jaime Lagos; Jorge Fernandez; Loredana Arata; Patricia Bustos; Paz Tapia; Rodrigo Fasce; Winston Andrade
EPI_ISL_445297	CLINICA INTEGRAL S.A.	Instituto de Salud Publica de Chile	Alejandra Acevedo; Andrés E Castillo; Bárbara Parra; Carolina Tambley; Gabriel Leal; Jaime Lagos; Jorge Fernandez; Loredana Arata; Patricia Bustos; Paz Tapia; Rodrigo Fasce; Winston Andrade
EPI_ISL_445256, EPI_ISL_445259	CLINICA LAS CONDES S.A.	Instituto de Salud Publica de Chile	Alejandra Acevedo; Andrés E Castillo; Bárbara Parra; Carolina Tambley; Gabriel Leal; Jaime Lagos; Jorge Fernandez; Loredana Arata; Patricia Bustos; Paz Tapia; Rodrigo Fasce; Winston Andrade
EPI_ISL_445282 to 445283, EPI_ISL_445290 to 445292, EPI_ISL_445299	CLINICA MAGALLANES S.A.	Instituto de Salud Publica de Chile	Alejandra Acevedo; Andrés E Castillo; Bárbara Parra; Carolina Tambley; Gabriel Leal; Jaime Lagos; Jorge Fernandez; Loredana Arata; Patricia Bustos; Paz Tapia; Rodrigo Fasce; Winston Andrade
EPI_ISL_445264	CLINICA REDSALUD VITACURA.	Instituto de Salud Publica de Chile	Alejandra Acevedo; Andrés E Castillo; Bárbara Parra; Carolina Tambley; Gabriel Leal; Jaime Lagos; Jorge Fernandez; Loredana Arata; Patricia Bustos; Paz Tapia; Rodrigo Fasce; Winston Andrade
EPI_ISL_445249	CLINICA SANTA MARIA S.A.	Instituto de Salud Publica de Chile	Alejandra Acevedo; Andrés E Castillo; Bárbara Parra; Carolina Tambley; Gabriel Leal; Jaime Lagos; Jorge Fernandez; Loredana Arata; Patricia Bustos; Paz Tapia; Rodrigo Fasce; Winston Andrade
EPI_ISL_445257, EPI_ISL_445262	CLINICA TABANCURA	Instituto de Salud Publica de Chile	Alejandra Acevedo; Andrés E Castillo; Bárbara Parra; Carolina Tambley; Gabriel Leal; Jaime Lagos; Jorge Fernandez; Loredana Arata; Patricia Bustos; Paz Tapia; Rodrigo Fasce; Winston Andrade
EPI_ISL_445306, EPI_ISL_445312, EPI_ISL_445315, EPI_ISL_445361	CLINICA UC SAN CARLOS DE APOQUINDO	Instituto de Salud Publica de Chile	Alejandra Acevedo; Andrés E Castillo; Bárbara Parra; Carolina Tambley; Gabriel Leal; Jaime Lagos; Jorge Fernandez; Loredana Arata; Patricia Bustos; Paz Tapia; Rodrigo Fasce; Winston Andrade
EPI_ISL_445334	CLINICA UNIVERSITARIA DE PUERTO MONTT S.A.	Instituto de Salud Publica de Chile	Alejandra Acevedo; Andrés E Castillo; Bárbara Parra; Carolina Tambley; Gabriel Leal; Jaime Lagos; Jorge Fernandez; Loredana Arata; Patricia Bustos; Paz Tapia; Rodrigo Fasce; Winston Andrade
EPI_ISL_445330	CLINICA VESPUCIO S. A.	Instituto de Salud Publica de Chile	Alejandra Acevedo; Andrés E Castillo; Bárbara Parra; Carolina Tambley; Gabriel Leal; Jaime Lagos; Jorge Fernandez; Loredana Arata; Patricia Bustos; Paz Tapia; Rodrigo Fasce; Winston Andrade
EPI_ISL_536310	CLSC et Centre d'Hébergement la Petite-Nation	Laboratoire de santé publique du Québec	Guillaume Bourque; Ioannis Ragoussis; Jesse Shapiro; Mark Lathrop and Michel Roger on behalf of the CoVSeQ research group; Sandrine Moreira
EPI_ISL_1392800 to 1392806, EPI_ISL_1402337 to 1402353, EPI_ISL_1420787	CMBG FN Brno	CMBG FN Brno	Bezdicek M.; Lengerova M.; Svaton J.
EPI_ISL_420043, EPI_ISL_420061	CMIP	National Reference Center for Viruses of Respiratory Infections, Institut Pasteur, Paris	Angela Brisebarre; Etienne Simon-Lorière; Flora Donati; Marion Barbet; Maud Vanpeene; Mélanie Albert; Méline Bizard; Sylvie Behillil; Sylvie van der Werf; Vincent Enouf
EPI_ISL_539492, EPI_ISL_547584, EPI_ISL_560320 to 560321	CMS, Roorkee	CSIR-Institute of Microbial Technology	Amandeep Kaur; Anu Singh; Ashwani Kumar; Debarghya Ghose; Dipak Dutta; Harsh Goar; Kanika Bansal; Navin Baid; Poushali Chakraborty; Prabhu B. Patil; Rajesh Kumar Mishra; Sanjeet Kumar; Sanjeev Khosla
EPI_ISL_410486, EPI_ISL_416745 to 416746, EPI_ISL_508912 to 508930, EPI_ISL_508933, EPI_ISL_508937, EPI_ISL_508939 to 508940, EPI_ISL_508942, EPI_ISL_508945, EPI_ISL_508948, EPI_ISL_508951, EPI_ISL_508953 to 508957, EPI_ISL_508961 to 508965, EPI_ISL_508967, EPI_ISL_508969 to 508974, EPI_ISL_508976 to 508977, EPI_ISL_508979 to 508997, EPI_ISL_508999 to 509002, EPI_ISL_525536 to 525538, EPI_ISL_525540 to 525543, EPI_ISL_578176 to 578177, EPI_ISL_582110 to 582120, EPI_ISL_582122, EPI_ISL_582508, EPI_ISL_1418187 to 1418191, EPI_ISL_1418193, EPI_ISL_1418201 to 1418203, EPI_ISL_1418209 to 1418217, EPI_ISL_1418219, EPI_ISL_1418229, EPI_ISL_1418231 to 1418239, EPI_ISL_1418242, EPI_ISL_1418244 to 1418246, EPI_ISL_1418262 to 1418300, EPI_ISL_1418302 to 1418307, EPI_ISL_1433804, EPI_ISL_1433822, EPI_ISL_1433983, EPI_ISL_1433985, EPI_ISL_1433987, EPI_ISL_1434245, EPI_ISL_1461003, EPI_ISL_1461005 to 1461007, EPI_ISL_1461009 to 1461012, EPI_ISL_1461014, EPI_ISL_1461019, EPI_ISL_1461022 to 1461026, EPI_ISL_1461028 to 1461032, EPI_ISL_1461034 to 1461036, EPI_ISL_1461038, EPI_ISL_1461040, EPI_ISL_1461042 to 1461044, EPI_ISL_1461049 to 1461051, EPI_ISL_1461053 to 1461055, EPI_ISL_1461057, EPI_ISL_1461059 to 1461070, EPI_ISL_1461075 to 1461076, EPI_ISL_1461109, EPI_ISL_1461121, EPI_ISL_1461123, EPI_ISL_1461131, EPI_ISL_1461138 to 1461141, EPI_ISL_1461145 to 1461147, EPI_ISL_1461149 to 1461151, EPI_ISL_1461154 to 1461164, EPI_ISL_1461195 to 1461203, EPI_ISL_1461205 to 1461245, EPI_ISL_1461247 to 1461252, EPI_ISL_1526072 to 1526074, EPI_ISL_1526076 to 1526093, EPI_ISL_1526095 to 1526096, EPI_ISL_1526098 to 1526101, EPI_ISL_1526103 to 1526104, EPI_ISL_1526106, EPI_ISL_1526109 to 1526111, EPI_ISL_1526114 to 1526119, EPI_ISL_1526124 to 1526126, EPI_ISL_1526129 to 1526134, EPI_ISL_1526136 to 1526137, EPI_ISL_1526140 to 1526141, EPI_ISL_1526143 to 1526154, EPI_ISL_1526157 to 1526160, EPI_ISL_1526163 to 1526166, EPI_ISL_1526169, EPI_ISL_1526174 to 1526176, EPI_ISL_1526178 to 1526182, EPI_ISL_1526184 to 1526187, EPI_ISL_1526189 to 1526195, EPI_ISL_1526197 to 1526200, EPI_ISL_1526208 to 1526216, EPI_ISL_1526218 to 1526219, EPI_ISL_1526221 to 1526224, EPI_ISL_1526226 to 1526230, EPI_ISL_1526249 to 1526252, EPI_ISL_1526255 to 1526257, EPI_ISL_1526259 to 1526263, EPI_ISL_1526265 to 1526267, EPI_ISL_1526272, EPI_ISL_1526281, EPI_ISL_1526287, EPI_ISL_1526313, EPI_ISL_1526317, EPI_ISL_1526327, EPI_ISL_1526329 to 1526340, EPI_ISL_1526342 to 1526343, EPI_ISL_1526346 to 1526349, EPI_ISL_1526352 to 1526354, EPI_ISL_1526356 to 1526358, EPI_ISL_1526360 to 1526362, EPI_ISL_1526364 to 1526367, EPI_ISL_1526370 to 1526373, EPI_ISL_1526375, EPI_ISL_1526378 to 1526382, EPI_ISL_1526384 to 1526388, EPI_ISL_1526391, EPI_ISL_1526401 to 1526403, EPI_ISL_1526405, EPI_ISL_1526410 to 1526411, EPI_ISL_1526413 to 1526419, EPI_ISL_1526421 to 1526423, EPI_ISL_1526426 to 1526440, EPI_ISL_1526456 to 1526566, EPI_ISL_1526568 to 1526584, EPI_ISL_1526586 to 1526605			
see above	CNR Virus des Infections Respiratoires - France SUD	CNR Virus des Infections Respiratoires - France SUD	Alexandre; Alexandre Gaymard; Antonin; Antonin Bal; Bal; Bouscambert-Duchamp; Brengel-Pesce; Bruno Lina; Bruno.; Carine Moustaud; Cheynet; Destras; Emilie Frobert; Florence; Florence Morfin-Sherpa; Gaymard; Gregory; Gregory Destras; Gregory Queromes; Gwendolyne Burfin; Hadrien Regue; Hadrien Règue; Josset; Karen; Laurence; Laurence Josset; Lina; Martine; Martine Valette; Maude; Maude Bouscambert-Duchamp; Morfin-Sherpa; Quentin Semanas; Raphaelle Lamy; Solenne Brun; Valette; Valérie
EPI_ISL_452127 to 452130	CO Department of Public Health and Environment	Pathogen Discovery, Respiratory Viruses Branch, Division of Viral Diseases, Centers for Disease Control and Prevention	Alison S. Laufer Halpin; Anna Montmayeur; Anna Uehara; Christopher A. Elkins; Clinton R. Paden; Haibin Wang; Jing Zhang; Krista Queen; Mary S. Keckler; Rachel Marine; Suxiang Tong; Yan Li; Ying Tao; Zachary Weiner
EPI_ISL_416994, EPI_ISL_418248 to 418249	COMPLEJO ASISTENCIAL UNIVERSITARIO DE BURGOS	Instituto de Salud Carlos III	A. Monzón; F. Casas; G. Hospital: ------; I. Jiménez; I. Megias-Lobon G.; Iglesias-Caballero; M. Camarero; M. Camarero S. Pozo F. Casas I. Jiménez P. Jiménez M. Zaballos A. Monzón; M. Cuesta; M. González-Esguevillas; M. Molinero Calamita; M. Zaballos; P. Jiménez; S. Juliá; S. Juliá M. Cuesta I. Megias Lobón; S. Pozo; S. Varona
EPI_ISL_1445109 to 1445110, EPI_ISL_1445114 to 1445118	COMPLEXO HOSPITALAR OURO VERDE DE CAMPINAS	Instituto Butantan / Mendelics	Antonio Jorge Martins; Bibiana Santos; Claudia Renata dos Santos Barros; David Schlesinger; Debora Botequio Moretti; Dimas Tadeu Covas; Elaine Cristina Marqueze; Elaine Vieira dos Santos; Erika Freitas; Evandra Strazza Rodrigues; Flavia Aburjaile; José Salvatore Leister Patané; João Paulo Kitajima; Luiz Carlos Junior de Alcantara; Maria Carolina Elias; Marta Giovanetti; Rafael dos Santos Bezerra; Raul Machado Neto; Ricardo Haddad; Rodrigo Tocantins Calado.; Sandra Coccuzzo Sampaio; Simone Kashima; Svetoslav Nanev Slavov; Vagner Fonseca; Vincent Louis Viala
EPI_ISL_1509967, EPI_ISL_1509973, EPI_ISL_1509978, EPI_ISL_1509988, EPI_ISL_1509997 to 1509998, EPI_ISL_1510032, EPI_ISL_1510708 to 1510711, EPI_ISL_1510713 to 1510718, EPI_ISL_1510730 to 1510735, EPI_ISL_1510744 to 1510746, EPI_ISL_1511218 to 1511221, EPI_ISL_1511223			
see above	CONSEJERIA DE SANIDAD Y ASUNTOS SOCIALES	Instituto de Salud Carlos III	A. Monzón; F. Casas; GONZALO; I. GUTIERREZ AVILA; I. Jiménez; I.GUTIERREZ AVILA; Iglesias-Caballero; M. Camarero; M. Sandonís; P. Zaballos; S. Camarero; S. Cuesta; S. Iglesias-Caballero; S. Pozo; S. Varona; Sandonís; V. Camarero; V. Vázquez-Morón; Vázquez-Morón
EPI_ISL_434538	COOPESAIN	Inciensa, Instituto Costarricense de Investigación y Enseñanza en Nutrición y Salud	Adriana Godinez & Melany Calderon; Claudio Soto-Garita; Estela Cordero; Francisco Duarte; Hebleen Porras
EPI_ISL_1527013	COOPESAIN-TIBÁS	Inciensa, Instituto Costarricense de Investigación y Enseñanza en Nutrición y Salud	Barboza-Arguedas E & Flores-León G; Pérez-Corrales C
EPI_ISL_1527014	COOPESIBA BARVA	Inciensa, Instituto Costarricense de Investigación y Enseñanza en Nutrición y Salud	Barboza-Arguedas E & Kellerman-Montero K; Pérez-Corrales C
EPI_ISL_1469604, EPI_ISL_1469608, EPI_ISL_1469635 to 1469636, EPI_ISL_1469641, EPI_ISL_1469715, EPI_ISL_1469735, EPI_ISL_1469782			

see above	COORDENADORIA GERAL DE VIGILANCIA EM SAUDE	Epiclin	Ana Paula Muterle; Carolina Comerlato; Eliana Márcia Da Ros Wendland; Fernando Hayashi Sant'Anna; Janira Prichula; Juliana Comerlato
EPI_ISL_1424478 to 1424502	COVID Outreach Wood River	UW Virology Lab	Alexander Greninger; Hong Xie; Keith R. Jerome; Lasata Shrestha; Meei-Li Huang; Michelle Lin; Pavitra Roychoudhury; Ryan Cole; Shah Mohamed Bakhash; Tom Archie
EPI_ISL_450841	COVID-19 Laboratory	DNA Solution Ltd	ABM Khademul Islam; AHM Nurun Nabi; Abu Sufian; Gazi Nurun Nahar; Habibul Bari Shozib; Haseena Khan; Imran Khan; Latiful Bari; M Anwar Hossain.; MA Malek; Mamun Ahmed; Md Imdadul Hoque; Md Ismail Hosen; Md Mizanur Rahman; Mohammad Riazul Islam; Nazmul Ahsan; Richard Malo; Sabita Rezwana Rahman; Sabrina Moriom Elius; Shahryar Nabi; Sharif Akhteruzzaman; Zeba Islam Seraj
EPI_ISL_450842 to 450843	COVID-19 Laboratory	DNA Solution Ltd.	ABM Khademul Islam; AHM Nurun Nabi; Abu Sufian; Gazi Nurun Nahar; Habibul Bari Shozib; Haseena Khan; Imran Khan; Latiful Bari; M Anwar Hossain.; MA Malek; Mamun Ahmed; Md Imdadul Hoque; Md Ismail Hosen; Md Mizanur Rahman; Mohammad Riazul Islam; Nazmul Ahsan; Richard Malo; Sabita Rezwana Rahman; Sabrina Moriom Elius; Shahryar Nabi; Sharif Akhteruzzaman; Zeba Islam Seraj
EPI_ISL_450840	COVID-19 Laboratory	DNA Solution Ltd. L-5	ABM Khademul Islam; AHM Nurun Nabi; Abu Sufian; Gazi Nurun Nahar; Habibul Bari Shozib; Haseena Khan; Imran Khan; Latiful Bari; M Anwar Hossain.; MA Malek; Mamun Ahmed; Md Imdadul Hoque; Md Ismail Hosen; Md Mizanur Rahman; Mohammad Riazul Islam; Nazmul Ahsan; Richard Malo; Sabita Rezwana Rahman; Sabrina Moriom Elius; Shahryar Nabi; Sharif Akhteruzzaman; Zeba Islam Seraj
EPI_ISL_450839	COVID-19 Laboratory Centre for Advanced Research in Sciences (CARS), University of Dhaka, Dhaka-1000, Bangladesh	DNA Solution Ltd	ABM Khademul Islam; AHM Nurun Nabi; Abu Sufian; Gazi Nurun Nahar; Habibul Bari Shozib; Haseena Khan; Imran Khan; Latiful Bari; M Anwar Hossain.; MA Malek; Mamun Ahmed; Md Imdadul Hoque; Md Ismail Hosen; Md Mizanur Rahman; Mohammad Riazul Islam; Nazmul Ahsan; Richard Malo; Sabita Rezwana Rahman; Sabrina Moriom Elius; Shahryar Nabi; Sharif Akhteruzzaman; Zeba Islam Seraj
EPI_ISL_412981	CR&WISCO GENERAL HOSPITAL	Hubei Provincial Center for Disease Control and Prevention	Bin Fang; Bo Yang; Bo Yu; Faxian Zhan; Guojun Ye; Jing Li; Junqiang Xu; Kun Cai; Linlin Liu; Xiang Li; Xiao Yu; Xixiang Huo; Yongzhong Jiang.
EPI_ISL_1493593	CS II Dr Jahyr de Paula Ribeiro Guara	Instituto Adolfo Lutz, Interdiciplinary Procedures Center, Strategic Laboratory	Caio Vinicius Dias Lopes; Claudia Regina Gonçalves; Claudio Tavares Sacchi; Erica Valessa Ramos Gomes; Karoline Rodrigues Campos
EPI_ISL_1493589, EPI_ISL_1494923	CS II Dr Jose Ferreira Telles	Instituto Adolfo Lutz, Interdiciplinary Procedures Center, Strategic Laboratory	Caio Vinicius Dias Lopes; Claudia Regina Gonçalves; Claudio Tavares Sacchi; Erica Valessa Ramos Gomes; Karoline Rodrigues Campos
EPI_ISL_1493580, EPI_ISL_1493582	CS II Dr Miguel Vitaliano Orlandia	Instituto Adolfo Lutz, Interdiciplinary Procedures Center, Strategic Laboratory	Caio Vinicius Dias Lopes; Claudia Regina Gonçalves; Claudio Tavares Sacchi; Erica Valessa Ramos Gomes; Karoline Rodrigues Campos
EPI_ISL_574593, EPI_ISL_574596, EPI_ISL_583494	CS II Dr. Antonio Vicoso Moreira de Rezende Sumare	Instituto Adolfo Lutz, Interdiciplinary Procedures Center, Strategic Laboratory	Claudia Regina Gonçalves; Claudio Tavares Sacchi; Erica Valessa Ramos Gomes; Karoline Rodrigues Campos
EPI_ISL_1445142 to 1445146	CS II EGIDIO BRUNHARA MORRO AGUDO	Instituto Butantan / Mendelics	Antonio Jorge Martins; Bibiana Santos; Claudia Renata dos Santos Barros; David Schlesinger; Debora Botequio Moretti; Dimas Tadeu Covas; Elaine Cristina Marqueze; Elaine Vieira dos Santos; Erika Freitas; Evandra Strazza Rodrigues; Flavia Aburjaile; José Salvatore Leister Patané; João Paulo Kitajima; Luiz Carlos Junior de Alcantara; Maria Carolina Elias; Marta Giovanetti; Rafael dos Santos Bezerra; Raul Machado Neto; Ricardo Haddad; Rodrigo Tocantins Calado.; Sandra Coccuzzo Sampaio; Simone Kashima; Svetoslav Nanev Slavov; Vagner Fonseca; Vincent Louis Viala
EPI_ISL_1493587 to 1493588	CS III de Patrocinio Paulista	Instituto Adolfo Lutz, Interdiciplinary Procedures Center, Strategic Laboratory	Caio Vinicius Dias Lopes; Claudia Regina Gonçalves; Claudio Tavares Sacchi; Erica Valessa Ramos Gomes; Karoline Rodrigues Campos
EPI_ISL_486853	CSIR-CDRI/SGPGI	CSIR-CDRI/SGPGI	Dharam Veer Singh; Rahul Vishvkarma; Rajender Singh; Ravishankar Ramachandran; Saumya Sarkar; Tapas Kumar Kundu; Uday Ghoshal; Ujjala Ghoshal
EPI_ISL_447556 to 447583, EPI_ISL_447847 to 447866, EPI_ISL_450326 to 450332, EPI_ISL_458045 to 458065, EPI_ISL_458070 to 458077, EPI_ISL_458080, EPI_ISL_458298, EPI_ISL_471585 to 471646, EPI_ISL_495161 to 495273, EPI_ISL_528823 to 528869, EPI_ISL_539616 to 539775, EPI_ISL_1398943 to 1398954			
see above	CSIR-Centre for Cellular and Molecular Biology	CSIR-Centre for Cellular and Molecular Biology	Ajay Sarawagi; Amrutha H C; Ananga Ghosh; Annapoorna P Karthyayani; Archana Bharadwaj Siva; B Himasri; Blessy B John; Debabrata Jana; Debrya Saha; Deepak Kumar; Devi Prasad Vijayashankar; Devi Prasad Vijayashankara; Dhiviya Vedagiri; Disha Nanda; Divya Das; Divya Gupta; Divya Tej Sowpati; G. Aditya Kumar; Gangumala Srinivas Reddy; Gokulan C G; Gunjan Purohit; Hanuman Tulashiram Kale; Jotin Gogoi; Kakade Aishwarya Arun; Karthik Bharadwaj Tallapaka; Kezia J Ann; Koushick Sivakumar; Krishnan Harinivas Harshan; Lamuk Zaveri; M Soujanya Reddy; M Soujanya Reddy Rakesh K Mishra; Manish Bhattacharjee; Namami Gaur; Nikhil Hajirnis; Onkar Kulkarni; Pankaj Kumar; Payel Mukherjee; Peddapuvala Sai Uday Kiran; Peddapuvala Sai Uday Kiran Rakesh K Mishra; Pooja Ramesh Gupta; Prachand Issarapu; Pratheusa Maccha; Preethi Jampala; Preethi Jampala Rakesh K Mishra; Priya Singh; Priyanka Pant; Purushotham Vodnala; Radhika Khandelwal; Rajan Kumar Jha; Rajkanwar Nathawat; Rakesh K Mishra; Ravi Prasad Mukku; Renu Sudhakar; Roshan Maku Venkata; Sakshi Shambhavi; Santosh Kumar Kuncha; Shagufta Khan; Sharada Ravi Iyer; Shemin Mansuri; Shraddha Vijay Lahoti; Sofia Banu; Somesh Gorde; Sonu Uday; Sudipta Mondal; Sujoy Deb; Sulagana Mukherjee; Swati Bayyana; Swetha Sundar; Tulasi Nagabandi; Umesh Kumar; Unis Ahmad Bhat; Vishal Sah; Viswagithe S L; Zeba Rizvi; Zuberwasim Sayyad
EPI_ISL_569859 to 569863, EPI_ISL_578080 to 578081, EPI_ISL_578168, EPI_ISL_578175, EPI_ISL_578178 to 578184, EPI_ISL_581449 to 581451, EPI_ISL_581494 to 581497, EPI_ISL_581499 to 581504, EPI_ISL_582028			
see above	CSIR-Indian Institute of Chemical Biology, MEDICA Supercpecialty Hospital Kolkata	CSIR-Indian Institute of Chemical Biology, MEDICA Supercpecialty Hospital Kolkata	Abhishake Lahiri; Debaleena Bhowmik; Dr. Partha Chakrabarti; Dr. Arpita Ghosh Mitra; Dr. Aviral Roy; Dr. Aviral Roy; Dr. AviralRoy; Dr. Partha Chakrabarti; Dr. Rajesh Pandey; Dr. Saikat Chakrabarti; Dr. Sandip Paul; Dr. Soumen Saha; Dr.Partha Chakrabarti; Priyanka Mallick; Sujay Krishna Maity
EPI_ISL_1547802 to 1547805	CSIR-National Environmental Engineering Research Institute	CSIR-Centre for Cellular and Molecular Biology - INSACOG	Amareshwar Vodapalli; Ara Sreenivas; B Himasri; Divya Tej Sowpati; Karthik Bharadwaj Tallapaka; Krishna Khairnar; Lamuk Zaveri; Onkar Kulkarni; Rakesh K Mishra; Sharath Chandra Thota; Shreekant Verma; Sofia Banu; Viswagithe S L
EPI_ISL_1393834 to 1393839, EPI_ISL_1538575, EPI_ISL_1538915, EPI_ISL_1539185	CSK MSWiA Warszawa	1. National Institute of Public Health - National Institute of Hygiene; 2. Eurofins Genomics Europe Sequencing GmbH	ECDC COVID-19 WGS support team; Eurofins Genomics Europe Sequencing Team; Gierczyki Rafa; Sadkowska-Todys Magorzata; Wokowicz Tomasz; Zacharczuk Katarzyna
EPI_ISL_450306, EPI_ISL_465685, EPI_ISL_465687, EPI_ISL_465692, EPI_ISL_535799 to 535800, EPI_ISL_535813, EPI_ISL_535815, EPI_ISL_535841, EPI_ISL_535846, EPI_ISL_535864, EPI_ISL_535876 to 535877, EPI_ISL_535884, EPI_ISL_535902, EPI_ISL_535910, EPI_ISL_535925, EPI_ISL_535928, EPI_ISL_535946, EPI_ISL_535967, EPI_ISL_535984, EPI_ISL_536027 to 536028, EPI_ISL_536040, EPI_ISL_536042, EPI_ISL_536044, EPI_ISL_536110, EPI_ISL_536150, EPI_ISL_536158, EPI_ISL_536160 to 536161, EPI_ISL_536164, EPI_ISL_536166, EPI_ISL_536172, EPI_ISL_536181, EPI_ISL_536197, EPI_ISL_536213, EPI_ISL_536215 to 536217, EPI_ISL_536235, EPI_ISL_536237, EPI_ISL_536243, EPI_ISL_536256, EPI_ISL_536258, EPI_ISL_536272, EPI_ISL_536278, EPI_ISL_536286, EPI_ISL_536299, EPI_ISL_536301 to 536302, EPI_ISL_536304, EPI_ISL_536321, EPI_ISL_536344 to 536345, EPI_ISL_536361, EPI_ISL_536382 to 536383			
see above	CSSS Haut-Richelieu/Rouville (Hôpital)	Laboratoire de santé publique du Québec	Guillaume Bourque; Ioannis Ragoussis; Jesse Shapiro; Mark Lathrop and Michel Roger; Mark Lathrop and Michel Roger on behalf of the CoVSeQ research group; Sandrine Moreira
EPI_ISL_536109	CSSS de Port-Cartier	Laboratoire de santé publique du Québec	Guillaume Bourque; Ioannis Ragoussis; Jesse Shapiro; Mark Lathrop and Michel Roger on behalf of the CoVSeQ research group; Sandrine Moreira
EPI_ISL_535970, EPI_ISL_536206, EPI_ISL_536260	CSSS de la Minganie	Laboratoire de santé publique du Québec	Guillaume Bourque; Ioannis Ragoussis; Jesse Shapiro; Mark Lathrop and Michel Roger; Mark Lathrop and Michel Roger on behalf of the CoVSeQ research group; Sandrine Moreira
EPI_ISL_1446620, EPI_ISL_1446745, EPI_ISL_1516364 to 1516367	CT-Dr. Katherine A. Kelley State Public Health Lab	Centers for Disease Control and Prevention Division of Viral Diseases, Pathogen Discovery	Alison Laufer Halpin; Ben L. Rambo-Martin; Clinton R. Paden; Dakota Howard; Darlene Wagner; Dave Wentworth; Dhwani Batra; Jasmine Padilla; Justin Lee; Katie Dillon; Krista Queen; Kristen Knipe; Kristine Lacek; Mark Burroughs; Matthew Schmerer; Mili Sheth; Peter Cook; Sam Shepard; Sarah Nobles; Shoshona Le; Suxiang Tong; Vivien Dugan; Yvette Unoarumhi
EPI_ISL_426416, EPI_ISL_454642, EPI_ISL_454645	CT-Dr. Katherine A. Kelley State Public Health Lab	Pathogen Discovery, Respiratory Viruses Branch, Division of Viral Diseases, Centers for Disease Control and Prevention	Alison S. Laufer Halpin; Anna Uehara; Bettina Bankamp; Christopher A. Elkins; Clinton R. Paden; Haibin Wang; Jing Zhang; Krista Queen; Mary S. Keckler; Rachel Marine; Suxiang Tong; Yan Li; Ying Tao; Zachary Weiner
EPI_ISL_468314, EPI_ISL_583503	CTA Centro de Testagem e Aconselhamento	Instituto Adolfo Lutz, Interdiciplinary Procedures Center, Strategic Laboratory	Claudia Regina Gonçalves; Claudio Tavares Sacchi; Erica Valessa Ramos Gomes; Karoline Rodrigues Campos
EPI_ISL_445303	CTRO.DE SALUD FAMILIAR DR. RAUL YAZIGI	Instituto de Salud Publica de Chile	Alejandra Acevedo; Andrés E Castillo; Bárbara Parra; Carolina Tambley; Gabriel Leal; Jaime Lagos; Jorge Fernandez; Loredana Arata; Patricia Bustos; Paz Tapia; Rodrigo Fasce; Winston Andrade
EPI_ISL_451935, EPI_ISL_452140, EPI_ISL_452142, EPI_ISL_452148 to 452152, EPI_ISL_1469959	CUB Hopital Erasme Laboratoire d'Anatomie Pathologique	CUB Hopital Erasme Laboratoire d'Anatomie Pathologique	Dr Nicky D'Haene; Dr. Nicky D'Haene; Dr.Nikcy D'Haene; Isabelle Salmon; Nicky D'Haene; Nikcy D'Haene; Prof. Isabelle Salmon
EPI_ISL_535749, EPI_ISL_535761, EPI_ISL_535764 to 535765, EPI_ISL_535811,	CUSM-Site Glen-LAB Microbiologie	Laboratoire de santé publique du Québec	Guillaume Bourque; Ioannis Ragoussis; Jesse Shapiro; Mark Lathrop and Michel Roger; Mark Lathrop and Michel Roger on behalf of the CoVSeQ research group; Sandrine Moreira

EPI_ISL_536337, EPI_ISL_536365 to 536366			
EPI_ISL_515938 to 515942	CV RAMAN HOSPITAL	Department of Neurovirology, National Institute of Mental Health and Neuroscience (NIMHANS)	Anita Desai; Chitra Pattabiraman; Harsha PK; Manjunatha Venkataswamy; Pramada Prasad; Ravi Vasanthapuram; Risha Rasheed; Shafeeq S Hameed; Vijayalakshmi Reddy
EPI_ISL_486383, EPI_ISL_486881	CV Raman Hospital	Department of Neurovirology, National Institute of Mental Health and Neuroscience (NIMHANS)	Anita Desai; Chitra Pattabiraman; Harsha PK; Manjunatha Venkataswamy; Ravi Vasanthapuram; Risha Rasheed; Shafeeq S Hameed; Vijayalakshmi Reddy
EPI_ISL_428364, EPI_ISL_428367, EPI_ISL_443300 to 443302, EPI_ISL_443306, EPI_ISL_443311 to 443313, EPI_ISL_443317	Cabinet Médical	National Reference Center for Viruses of Respiratory Infections, Institut Pasteur, Paris	Angela Brisebarre; Etienne Simon-Lorière; Flora Donati; Marion Barbet; Maud Vanpeene; Mélanie Albert; Méline Bizard; Sylvie Behillil; Sylvie van der Werf; Vincent Enouf
EPI_ISL_1443190, EPI_ISL_1545642	Cabinet Priv	Institut de Pathologie et Genetique (IPG)	Jérémie Gras; Pascale Hilbert
EPI_ISL_418235	Cabinet médical	National Reference Center for Viruses of Respiratory Infections, Institut Pasteur, Paris	Angela Brisebarre; Etienne Simon-Lorière; Flora Donati; Marion Barbet; Maud Vanpeene; Mélanie Albert; Méline Bizard; Sylvie Behillil; Sylvie van der Werf; Vincent Enouf
EPI_ISL_418812 to 418813, EPI_ISL_429807, EPI_ISL_429817 to 429820, EPI_ISL_482474 to 482475, EPI_ISL_482478, EPI_ISL_482480 to 482484	Cadham Provincial Laboratory	National Microbiology Laboratory	; Anna Majer; David Alexander; Elsie Grudeski; Gary Van Domselaar; Grace Seo; Jared Bullard; Jennifer Tanner; Kerry Dust; Kristyn Burak; Matthew Gilmour; Morag Graham; Natalie Knox; Nathalie Bastien; Paul Van Caeseele; Philip Mabon; Rhiannon Huzarewich; Russell Mandes; Russell Mandez; Shari Tyson; Timothy Booth; Yan Li
EPI_ISL_582242 to 582249, EPI_ISL_582251 to 582286, EPI_ISL_582288 to 582306, EPI_ISL_582308, EPI_ISL_582311 to 582343, EPI_ISL_582345 to 582347, EPI_ISL_582349 to 582397, EPI_ISL_582399 to 582414, EPI_ISL_582416 to 582420, EPI_ISL_582422 to 582443, EPI_ISL_582445 to 582507			
see above	Cadham Provincial Laboratory	National Microbiology Laboratory (NML)	Anna Majer; Anneliese Landgraff; CanCOGeN's metadata curation team; Darian Hole; David Alexander; Elsie Grudeski; Gary Van Domselaar; Grace Seo; Jared Bullard; Jennifer Tanner; Kerry Dust; Madison Chapel; Morag Graham; Natalie Knox; Nathalie Bastien; Paul Van Caeseele; Philip Mabon; Public Health Agency of Canada CanCOGeN team; Rhiannon Huzarewich; Russell Mandes; Shari Tyson; Timothy Booth; Yan Li
EPI_ISL_444794	Cairns Hospital	Public Health Virology Laboratory	Alyssa Pyke; Amanda De Jong; Andrew Van Den Hurk; Bixing Huang; Carmel Taylor; David Warrilow; Doris Genge; Elisabeth Gamez; Glen Hewitson; Ian Maxwell Mackay; Inga Sultana; Jamie McMahon; Jean Barcelon; Judy Northill; Mitchell Finger; Natalie Simpson; Neelima Nair; Peter Burtonclay; Peter Moore; Sarah Wheatley; Sean Moody; Sonja Hall-Mendelin; Timothy Gardam; and Frederick Moore
EPI_ISL_408009 to 408010	California Department of Health	Pathogen Discovery, Respiratory Viruses Branch, Division of Viral Diseases, Centers for Dieases Control and Prevention	Anna Uehara; Brett L. Whitaker; Brian Lynch; Clinton Paden; Janna' R. Murray; Jing Zhang; Krista Queen; Lijuan Wang; Senthil Kumar K. Sakthivel; Shifaq Kamili; Stephen Lindstrom; Susan I. Gerber; Suxiang Tong; Xiaoyan Lu; Yan Li; Ying Tao
EPI_ISL_408008	California Department of Health	Pathogen Discovery, Respiratory Viruses Branch, Division of Viral Diseases, Centers for Disease Control and Prevention	Anna Uehara; Brett L. Whitaker; Brian Lynch; Clinton Paden; Janna' R. Murray; Jing Zhang; Krista Queen; Lijuan Wang; Senthil Kumar K. Sakthivel; Shifaq Kamili; Stephen Lindstrom; Susan I. Gerber; Suxiang Tong; Xiaoyan Lu; Yan Li; Ying Tao
EPI_ISL_515894 to 515929	California Department of Public Health	California Department of Public Health	CDPH IDLB COVIDNet
EPI_ISL_413930	California Department of Public Health	Chiu Laboratory UCSF-Abbott Viral Diagnostics and Discovery Center University of California, San Francisco	Alicia Sotomayor-Gonzalez; Allan Gopez; Chao-Yang Pan; Debra A. Wadford; Guixia Yu; Hugo Guevara; Scot Federman; Steve Miller; Wei Gu; Xianding Deng; and Charles Y. Chiu
EPI_ISL_413557 to 413559, EPI_ISL_413561, EPI_ISL_413922, EPI_ISL_413924 to 413926, EPI_ISL_413928, EPI_ISL_413931, EPI_ISL_417322 to 417329, EPI_ISL_429875 to 429876, EPI_ISL_429881			
see above	California Department of Public Health	Chiu Laboratory, University of California, San Francisco	Chao-Yang Pan; Debra A. Wadford; Hugo Guevara; Scot Federman; Wei Gu; Xianding Deng; and Charles Y. Chiu
EPI_ISL_406034, EPI_ISL_406036, EPI_ISL_410044, EPI_ISL_411954 to 411955	California Department of Public Health	Pathogen Discovery, Respiratory Viruses Branch, Division of Viral Diseases, Centers for Dieases Control and Prevention	Anna Uehara; Brett L. Whitaker; Brian Lynch; Clinton R. Paden; Haibin Wang; Janna' R. Murray; Jing Zhang; Krista Queen; Lijuan Wang; Senthil Kumar K. Sakthivel; Shifaq Kamili; Stephen Lindstrom; Susan I. Gerber; Suxiang Tong; Xiaoyan Lu; Yan Li; Ying Tao
EPI_ISL_412862, EPI_ISL_419554, EPI_ISL_594458	California Department of Public Health	Pathogen Discovery, Respiratory Viruses Branch, Division of Viral Diseases, Centers for Disease Control and Prevention	Anna Uehara; Brett L. Whitaker; Brian Lynch; Clinton Paden; Clinton R. Paden; Haibin Wang; Janna' R. Murray; Jasmine Padilla; Jing Zhang; Julu Bhatnagar; Justin Lee; Krista Queen; Lijuan Wang; Senthil Kumar K. Sakthivel; Shifaq Kamili; Stephen Lindstrom; Susan I. Gerber; Suxiang Tong; Xiaoyan Lu; Yan Li; Ying Tao
EPI_ISL_418865	California Department of Public Health	University of California, San Francisco	Chao-Yang Pan; Debra A. Wadford; Hugo Guevara; Scot Federman; Wei Gu; Xianding Deng; and Charles Y. Chiu
EPI_ISL_1534294, EPI_ISL_1534383, EPI_ISL_1534404	Calitzdorp (Bergsig) Clinic wc CDC	NHLS/UCT	Arash Iranzadeh; Bruna Galvao; Carolyn Williamson; Deelan Doolabh; Diana Hardie; Emmanuel SJ; Innocent Mudau; Kruger Marais; Lynn Tyers; Marvin Hsiao; Stephen Korsman; Tegally H; de Oliveira T
EPI_ISL_447594	Caloundra Hospital	Public Health Virology Laboratory	Alyssa Pyke; Amanda De Jong; Andrew Van Den Hurk; Bixing Huang; Carmel Taylor; David Warrilow; Doris Genge; Elisabeth Gamez; Glen Hewitson; Ian Maxwell Mackay; Inga Sultana; Jamie McMahon; Jean Barcelon; Judy Northill; Mitchell Finger; Natalie Simpson; Neelima Nair; Peter Burtonclay; Peter Moore; Sarah Wheatley; Sean Moody; Sonja Hall-Mendelin; Timothy Gardam; and Frederick Moore
EPI_ISL_1532800, EPI_ISL_1532803, EPI_ISL_1532812	Cambodian National Public Health Laboratory, National Institute of Public Health	Virology Unit, Institut Pasteur du Cambodge	Chau Darapheak; Chin Savuth; Erik A Karlsson; Kraing Sidonn; Leakhena Pum; Ly Sovann; Sokhoun Yann; Teyputita Ou; Veasna Duong; Yi Sengdoeurn
EPI_ISL_1423125, EPI_ISL_1423127	Camden Clark Medical Center	WVU and Marshall University Combined Genomics Core Facilities	"James Denvir; Peter Perrotta; Peter Stoilov; Ryan Percifield"; Wesley Kimble
EPI_ISL_576371 to 576373	Cancer Biology Department, National Cancer Institute	Cancer Biology Department, National Cancer Institute	A.N.; Abouelhoda, M.; Ahmed; H.K.; Hafez; Hamdy; M.M.; M.S.; O.S.; Soliman; Zekri
EPI_ISL_447054	Cantacuzino National Military-Medical Institute for Research and Development	Cantacuzino Institute	A.Cretu; L.Ustea; M.Lazar
EPI_ISL_456321 to 456322, EPI_ISL_456348 to 456375, EPI_ISL_536792, EPI_ISL_547980 to 547985, EPI_ISL_548116 to 548118, EPI_ISL_548129, EPI_ISL_548134, EPI_ISL_548138 to 548140, EPI_ISL_579060 to 579092, EPI_ISL_579220 to 579227, EPI_ISL_579426 to 579452, EPI_ISL_579454 to 579471, EPI_ISL_579473 to 579506			
see above	Canterbury Health Laboratories	Institute of Environmental Science and Research (ESR)	Anja Werno; Antje van der Linden; Arlo Upton; Chris Mansell; David Hammer; Dragana Drinkovic; Erasmus Smit; Gary McAuliffe; Hana Sofia Andersson; Hermes Perez; James Ussher; Jill Sherwood; Jing Wang; Joep de Ligt; Josh Freeman; Julia Howard; Juliet Elvy; Lauren Jelly; Mary DeAlmeida; Matt Blakiston; Matt Storey; Matthew Rogers; Max Bloomfield; Michael Addidle; Michelle Balm; Muhammad Faisal; Nikki Freed; Olin Silander; Sally Roberts; Sarah Jefferies; Sharmini Muttaiyah; Susan Morpeth; Susan Taylor; Timothy Blackmore; Vani Sathyendran; Veronica Playle; Virginia Hope; Xiaoyun Ren
EPI_ISL_524478	Cantonal Hospital Winterthur	Institute of Medical Virology, University of Zurich	Alexandra Trkola; Andrea Zbinden; Fiona Steiner; Gabriela Ziltener; Jon Huder; Jürg Böni; Maryam Zaheri; Michael Huber; Patrick Redli; Riccarda Capaul; Stefan Schmutz; Verena Kufner
EPI_ISL_534230 to 534233, EPI_ISL_560981	Capio S:t Gorans sjukhus	The Public Health Agency of Sweden	Anna Risberg; Anna-Malin Linde; Karin Tegmark-Wisell; Maria Lind Karlberg; Mattias Haukland; Mia Brytting; Olov Svartstrom; Oskar Karlsson Lindsjo; Petra Edquist; Reza Advani; Sandra Broddesson
EPI_ISL_491472	Cardinal Santos Medical Center	Research Institute for Tropical Medicine	Catalino Demetria; Criselda Bautista; Daria Manalo; Edelwisa Mercado; Francisco Gerardo Polotan; Inez Andrea Medado; Kirstyn Brunker; Ma. Angelica Tujan; Othoniel Jan Onza
EPI_ISL_583504 to 583505	Casa de Saude Stella Maris	Instituto Adolfo Lutz, Interdiciplinary Procedures Center, Strategic Laboratory	Claudia Regina Gonçalves; Claudio Tavares Sacchi; Erica Valessa Ramos Gomes; Karoline Rodrigues Campos
EPI_ISL_1478887, EPI_ISL_1478908 to 1478909	Cass County Health Department	Illinois Department of Public Health - Springfield Lab	Bryan Sim; Gordon McCall
EPI_ISL_467809, EPI_ISL_475574 to 475716	Cedars-Sinai Medical Center, Department of Pathology & Laboratory Medicine, Molecular Pathology Laboratory	Cedars-Sinai Medical Center, Molecular Pathology Laboratory of Department of Pathology & Laboratory Medicine and Genomic Core	Brian Davis; Eric Vail; Jasmine T Plummer; Jean Lopategui; Jianbo Song; John Paul Govindavari; Jong Taek Kim; Stephanie Chen; Wenjuan Zhang
EPI_ISL_1393658, EPI_ISL_1538438, EPI_ISL_1538553, EPI_ISL_1538760, EPI_ISL_1538871, EPI_ISL_1538900 to 1538901, EPI_ISL_1539033, EPI_ISL_1539145, EPI_ISL_1539181			
see above	Cell-t	1. National Institute of Public Health - National Institute of Hygiene; 2. Eurofins Genomics Europe Sequencing GmbH	ECDC COVID-19 WGS support team; Eurofins Genomics Europe Sequencing Team; Gierczyki Rafa; Sadkowska-Todys Magorzata; Wokowicz Tomasz; Zacharczuk Katarzyna

EPI_ISL_450413	Center for Diagnostics, Institute of Medical Microbiology, Virology and Hygiene	University Medical Center Hamburg-Eppendorf	Huang, J.; Pfefferle; S. and Fischer, N.
EPI_ISL_459856 to 459864, EPI_ISL_468752, EPI_ISL_468760	Center for Genome Regulation (CRG)	Center for Mathematical Modeling and Center for Genome Regulation. Santiago, Chile	Allende ML; Gaete A; González M.; Maass A; Palma R; Travisany D; Urra C; Varas M
EPI_ISL_479663 to 479675, EPI_ISL_481283	Center for Genomics and System Biology, New York University	Center for Genomics and System Biology, New York University	Banakis, S.; Borenstein; Cornelius, A.; D. and Ghedin, E.; E.S.; Fleming, A.; Ghedin, E.; Gresham; Gresham, D.; Herati, R.; Johnson, K.; Khalfan, M.; Kottkamp, A.; M.J.; Mulligan; Raabe, V.; Roder, A.; Samanovic, M.; Ulrich, R.
EPI_ISL_516783 to 516785, EPI_ISL_522439 to 522453, EPI_ISL_522455 to 522464, EPI_ISL_522467 to 522473, EPI_ISL_522476, EPI_ISL_522479 to 522483, EPI_ISL_522485, EPI_ISL_522491 to 522497, EPI_ISL_522502, EPI_ISL_522505, EPI_ISL_522508, EPI_ISL_522510, EPI_ISL_522515 to 522516, EPI_ISL_522518, EPI_ISL_526730 to 526731, EPI_ISL_526734 to 526746			
see above	Center for Laboratory Control of Infectious Diseases, Korea Centers for Diseases Control and Prevention	Center for Laboratory Control of Infectious Diseases, Korea Centers for Diseases Control and Prevention	Ae Kyung Park; Eunkyung Shin; Heui Man Kim; Jeong-Min Kim; Jin Sun No; Junyoung Kim; Myung Guk Han; Yoon-Seok Chung
EPI_ISL_1546044, EPI_ISL_1546046, EPI_ISL_1546048, EPI_ISL_1546051, EPI_ISL_1546053, EPI_ISL_1546057, EPI_ISL_1546060, EPI_ISL_1546062, EPI_ISL_1546064, EPI_ISL_1546066, EPI_ISL_1546068, EPI_ISL_1546070, EPI_ISL_1546073, EPI_ISL_1546075, EPI_ISL_1546077, EPI_ISL_1546079, EPI_ISL_1546082, EPI_ISL_1546084, EPI_ISL_1546086 to 1546091, EPI_ISL_1546094, EPI_ISL_1546096, EPI_ISL_1546100, EPI_ISL_1546104, EPI_ISL_1546106, EPI_ISL_1546109, EPI_ISL_1546111, EPI_ISL_1546113 to 1546114, EPI_ISL_1546117, EPI_ISL_1546119, EPI_ISL_1546121, EPI_ISL_1546123, EPI_ISL_1546125, EPI_ISL_1546127, EPI_ISL_1546130, EPI_ISL_1546132, EPI_ISL_1546134, EPI_ISL_1546136, EPI_ISL_1546138			
see above	Center for Laboratory Medicine St. Gallen	Center for Laboratory Medicine St. Gallen	Yannick Gerth
EPI_ISL_493137, EPI_ISL_493139	Center for Research and Innovation, Faculty of Medical Technology, Mahidol University	Center for Research and Innovation, Faculty of Medical Technology, Mahidol University	Anek Mungaomklang; Hatairat Lerdsamran; Jarunee Prasertsopon; Kamolthip Atsawawaranunt; Kantima Sangsiriwut; Nattakan Thinpan; Pilaipan Puthavathana; Prabda Praphasiri; Somrak Sirikhetkon; Tipsuda Chanmanee
EPI_ISL_419654 to 419674, EPI_ISL_437993 to 438128, EPI_ISL_475770 to 475812, EPI_ISL_583563 to 583571, EPI_ISL_583692 to 583726, EPI_ISL_583869 to 583882	Center for Virology, Medical University of Vienna	Bergthaler laboratory, CeMM Research Center for Molecular Medicine of the Austrian Academy of Sciences	Adi Steinrigl; Alexander Lercher; Alexandra Popa; Andreas Bergthaler; Benedikt Agerer; Christian Paar; Christoph Bock; Daniela Schmid; Dorothee von Laer; Elisabeth Puchhammer-Stoeckl; Elisabeth Puchhammer-Stöckl; Franz Allerberger; Gernot Walder; Gregor Hörmann; Guenter Weiss; Gunther Vogl; Henrique Colaco; Jakob-Wendelin Genger; Jan Laine; Judith Aberle; Kinga Rigler-Hohenwarter; Lukas Endler; Manfred Nairz; Mark Smyth; Martin Senekowitsch; Michael Schuster; Peter Hufnagl; Peter Obrist; Rainer Gattringer; Sabine Sussitz-Rack; Stephan Aberle; Thomas Penz; Wegene Borena
EPI_ISL_516414 to 516425	Center for public health - Skopje	Research Center for Genetic Engineering and Biotechnology "Georgi D. Efremov" , Macedonian Academy of Sciences and Arts	RCGEB - MASA
EPI_ISL_1400492, EPI_ISL_1400525, EPI_ISL_1400552	Center of Hygiene and Epidemiology in Belgorod Region	WHO National Influenza Centre Russian Federation	Andrey Komissarov; Anna Ivanova; Artem Fadeev; Daria Danilenko; Dmitry Bazhenov; Dmitry Lioznov; Elena Nabieva; Georgii Bazykin; Ksenia Safina; Kseniya Komissarova; Lyudmila Berdinskikh; Maria Pisareva; Maria Timofeeva; Tamila Musaeva; Veronika Eder
EPI_ISL_523950	Center of Medical Microbiology, Virology, and Hospital Hygiene, University of Duesseldorf	Center of Medical Microbiology, Virology, and Hospital Hygiene, Heinrich Heine University Düsseldorf	Alexander Dilthey; Andreas Walker; Daniel Strelow; Hendrik Streeck; Jessica Nicolai; Jörg Timm; Klaus Pfeffer; Malte Kohns Vasconcelos; Marek Korencak; Maximilian Damagnez; Tobias Wienemann; Torsten Houwaart
EPI_ISL_413488, EPI_ISL_414497 to 414499, EPI_ISL_414504 to 414509, EPI_ISL_414574, EPI_ISL_417457 to 417468, EPI_ISL_419541 to 419552, EPI_ISL_425120 to 425140, EPI_ISL_452180, EPI_ISL_523927 to 523929, EPI_ISL_523931 to 523949, EPI_ISL_602510 to 602517, EPI_ISL_602533 to 602552			
see above	Center of Medical Microbiology, Virology, and Hospital Hygiene, University of Duesseldorf	Center of Medical Microbiology, Virology, and Hospital Hygiene, University of Duesseldorf	Alexander Dilthey; Andreas Walker; Bjorn-Erik Jensen; Björn-Erik Jensen; Björn-ErikJensen; Daniel Strelow; Detlef Kindgen-Milles; Hendrik Streeck; Jessica Nicolai; Jorg Timm; Jörg Timm; Klaus Pfeffer; Lisanna Hülse; Malte Kohns Vasconcelos; Marcel Andree; Marek Korencak; Maximilian Damagnez; Nadine Lübke; Ortwin Adams; Sandra Hauka; Tina Senff; Tobias Wienemann; Torsten Feldt; Torsten Houwaart
EPI_ISL_523930	Center of Medical Microbiology, Virology, and Hospital Hygiene, University of Duesseldorf	Universitätstr.1 40225 Düsseldorf Germany	Alexander Dilthey; Andreas Walker; Daniel Strelow; Hendrik Streeck; Jessica Nicolai; Jörg Timm; Klaus Pfeffer; Malte Kohns Vasconcelos; Marek Korencak; Maximilian Damagnez; Tobias Wienemann; Torsten Houwaart
EPI_ISL_430820	Center of Scientific Excellence for Influenza Viruses, National Research Centre (NRC), Egypt.	Center of Scientific Excellence for Influenza Viruses, National Research Centre (NRC), Egypt.	Abo Shama; Ahmed E Kayed; Ahmed El-Taweel; Ahmed Kandeil; Ahmed Mostafa; Amal Naguib; M Noura; Mahmoud Shehata; Mina Kamel; Mohamed Ahmed Ali; Mohamed El Sayes; Mokhtar Gomaa; Nancy M. El Guindy; Omnia Kutkat; Rabeh El-Shesheny; Sara Mahmoud; Shymaa Showky Ahmed; Wael Roshdy; Yassmin Moatasim
EPI_ISL_430819	Center of Scientific Excellence for Influenza Viruses,National Research Centre (NRC), Egypt.	Center of Scientific Excellence for Influenza Viruses,National Research Centre (NRC), Egypt.	Abo Shama; Ahmed E Kayed; Ahmed El-Taweel; Ahmed Kandeil; Ahmed Mostafa; Amal Naguib; M Noura; Mahmoud Shehata; Mina Kamel; Mohamed Ahmed Ali; Mohamed El Sayes; Mokhtar Gomaa; Nancy M. El Guindy; Omnia Kutkat; Rabeh El-Shesheny; Sara Mahmoud; Shymaa Showky Ahmed; Wael Roshdy; Yassmin Moatasim
EPI_ISL_429852 to 429855, EPI_ISL_495459	Centers for Disease Control and Prevention of Lishui	Department of InspectionCenters for Disease Control and Prevention of Lishui	Ji Jiansong; Ji Qiaoying; Wang Xiaoguang; Ye Bifeng; Ye Ling
EPI_ISL_539495	Centers for Disease Control and Prevention, Dengue Branch	Centers for Disease Control and Prevention, Dengue Branch	Betzabel Flores; Diego Sainz de la Peña; Gabriela Paz-Bailey; Gilberto A. Santiago; Glenda Gonzalez; Janice Perez; Jorge Bertran; Jorge L. Munoz-Jordan; Keyla Charriez; Vanessa Rivera-Amill
EPI_ISL_406031, EPI_ISL_420082 to 420085, EPI_ISL_421641, EPI_ISL_421651, EPI_ISL_428488 to 428491, EPI_ISL_429882 to 429884	Centers for Disease Control, R.O.C. (Taiwan)	Centers for Disease Control, R.O.C. (Taiwan)	Ji-Rong Yang; Jung-Jung Mu; Ming-Tsan Liu; Ming-Tsan-Liu; Shu-Ying Li; Yu-Chi Lin; Yu-Chi-Lin
EPI_ISL_457750, EPI_ISL_459962 to 459964	Centogene AG	Centogene AG	Dr. Krishna Kumar Kandaswamy; Prof. Dr. Peter Bauer
EPI_ISL_1479160 to 1479201	Centracare Laboratory Services	Minnesota Department of Health, Public Health Laboratory	Alexandra Lorentz; Jacob Garfin; Matt Plumb; and Xiong Wang
EPI_ISL_455583, EPI_ISL_455594	Central Chest Institute of Thailand	National Institute of Health. Department of medical Sciences, Ministry of Public Health, Thailand	Chittaganpitch; Malinee; Okada; Parnmen; Phuygun; Pilailuk; Siripaporn; Sittiporn; Sunthareeya; Thanadachakul; Thanutsapa; Waicharoen; Warawan; Wongboot
EPI_ISL_1520156 to 1520157	Central DuPage Hospital	Illinois Department of Public Health - Chicago Lab	Ira Heimler; Vineet K. Dhiman
EPI_ISL_529032	Central Molecular Microbiology Laboratory and Next Generation Sequencing Reference Laboratory, Clinical and Chemical Pathology Department, Faculty of Medicine, CAIRO UNIVERSITY	Next Generation Sequencing Reference Laboratory, Faculty of Medicine, CAIRO UNIVERSITY and The Center for Genome and Microbiome Research, Faculty of Pharmacy, CAIRO UNIVERSITY	May Abdelfattah; May Sherif Soliman; Ramy Karam Aziz
EPI_ISL_529031	Central Molecular Microbiology Laboratory, Clinical and Chemical Pathology Department, Faculty of Medicine, CAIRO UNIVERSITY	Next Generation Sequencing Reference Laboratory, Faculty of Medicine, Cairo University and The Center for Genome and Microbiome Research, Faculty of Pharmacy, CAIRO UNIVERSITY	May Abdelfattah; May Sherif Soliman; Ramy Karam Aziz
EPI_ISL_429664 to 429703	Central Public Health Laboratory/Octávio Magalhães Institute (IOM) from the Ezequiel Dias Foundation (FUNED)	Instituto Octávio Magalhães / Fundação Ezequiel Dias (IOM/Funed)	Joilson Xavier; Luiz Carlos Junior Alcantara; Marcos Vinícius Silva; Marluce Aparecida Assunção Oliveira; Marta Giovanetti; Talita Adelino; Vagner Fonseca
EPI_ISL_447250 to 447251	Central Virology Laboratory	Central Virology Laboratory	Danit Sofer; Efrat Bucris; Ella Mendelson; Michal Mandelboim; Neta Zuckerman; Oran Erster; Orna Mor
EPI_ISL_419211	Central Virology Laboratory	Israel Institute for Biological Research	Adi Beth-Din; Anat Zvi; Boaz Politi; Dana Stein; Einat Vitner; Gadi Segal; Gili Regev-Yochay; Hadas Tamir; Hagit Achdout; Inbar Cohen-Gihon; Lilach Cherry; Michal Mandelboim; Nir Paran; Ofir Israeli; Ohad Shifman; Oran Erster; Orly Laskar; Sharon Melamed; Shay Weiss; Shmuel C. Shapira; Shmuel Yitzhaki; Tomer Israely; Yfat Yahalom Ronen
EPI_ISL_430842	Central chest Institute of Thailand	National Institute of Health. Department of medical Sciences, Ministry of Public Health, Thailand	Chittaganpitch; Malinee; Okada; Parnmen; Phuygun; Pilailuk; Siripaporn; Sittiporn; Sunthareeya; Thanadachakul; Thanutsapa; Waicharoen; Warawan; Wongboot
EPI_ISL_486410, EPI_ISL_486417	Centrala laboratorija	Latvian Biomedical Research and Study Centre	Ivars Silamielis; Jana Oste; Jnis Kloviš; Kaspars Megnis; Marta Priedte; Monta Ustinova; Stella Lapia; Uga Dumpis; Vita Rovte; ikita Zrelovs
EPI_ISL_548256	Centralsjukhuset	The Public Health Agency of Sweden	Anna Risberg; Anna-Malin Linde; Karin Tegmark-Wisell; Maria Lind Karlberg; Mattias Haukland; Mia Brytting; Olov Svartstrom; Oskar Karlsson Lindsjo; Petra Edquist; Reza Advani; Sandra Broddesson
EPI_ISL_1406454, EPI_ISL_1406472, EPI_ISL_1406486, EPI_ISL_1406585, EPI_ISL_1406590, EPI_ISL_1406594, EPI_ISL_1406599, EPI_ISL_1406606, EPI_ISL_1406616, EPI_ISL_1406619, EPI_ISL_1406639 to 1406640, EPI_ISL_1406656, EPI_ISL_1406664, EPI_ISL_1483780 to 1483782, EPI_ISL_1547454			

see above	Centre De Prelevement COVID RIOM	CHU Clermont-Ferrand, service de virologie	Bisseux Maxime; Combes Patricia; Henquell Cécile; Mirand Audrey
EPI_ISL_1483771, EPI_ISL_1547456	Centre De Prélévement COVID VICHY	CHU Clermont-Ferrand, service de virologie	Bisseux Maxime; Combes Patricia; Henquell Cécile; Mirand Audrey
EPI_ISL_1406459, EPI_ISL_1406498, EPI_ISL_1406507, EPI_ISL_1406518, EPI_ISL_1406549, EPI_ISL_1406597, EPI_ISL_1406652, EPI_ISL_1406657, EPI_ISL_1490235			
see above	Centre De Prélévement COVID VICHY	CHU Clermont-Ferrand, service de virologie	Bisseux Maxime; Combes Patricia; Henquell Cécile; Mirand Audrey
EPI_ISL_457999 to 458000	Centre For Biotechnology Research and Development	Centre For Biotechnology Research and Development	C.N. and Michuki; D.K.; G.N.; J.O.; Kimotho, J.; Matoke-Muhia; Matoke-Muhia, D.; Muuo; Ochwoto, M.; S.L.; S.N.; Symeker; Waruhiu; Zablon
EPI_ISL_414624, EPI_ISL_416494	Centre Hositalier Universitaire de Rouen Laboratoire de Virologie	National Reference Center for Viruses of Respiratory Infections, Institut Pasteur, Paris	Angela Brisebarre; Etienne Simon-Lorière; Flora Donati; Flora Donati Vincent Enouf; Jean-Christophe Plantier; Marion Barbet; Maud Vanpeene; Méline Bizard; Mélnie Albert; Sylvie Behillil; Sylvie van der Werf; Vincent Enouf
EPI_ISL_508958, EPI_ISL_509012	Centre Hospitalier Alpes Leman	CNR Virus des Infections Respiratoires - France SUD	Alexandre Gaymard; Antonin Bal; Bruno Lina; Carine Moustaud; Florence Morfin-Sherpa; Gregory Destras; Gwendolyne Burfin; Laurence Josset; Martine Valette; Maude Bouscambert-Duchamp; Raphaelle Lamy; Solenne Brun
EPI_ISL_414627 to 414630, EPI_ISL_414634 to 414638, EPI_ISL_415653 to 415654, EPI_ISL_416495 to 416497, EPI_ISL_418218, EPI_ISL_418220 to 418221, EPI_ISL_418223 to 418225, EPI_ISL_418227 to 418228, EPI_ISL_418231, EPI_ISL_418236 to 418239, EPI_ISL_429968			
see above	Centre Hospitalier Compiègne Laboratoire de Biologie	National Reference Center for Viruses of Respiratory Infections, Institut Pasteur, Paris	Angela Brisebarre; Etienne Simon-Lorière; Fabiana Gambaro; Flora Donati; Flora Donati Vincent Enouf; Marion Barbet; Maud Vanpeene; Mélanie Albert; Méline Bizard; Mélnie Albert; Raulin Olivia; Sylvie Behillil; Sylvie van der Werf; Vincent Enouf
EPI_ISL_418428, EPI_ISL_508947	Centre Hospitalier Lucien Hussel	CNR Virus des Infections Respiratoires - France SUD	Alexandre Gaymard; Antonin Bal; Bruno Lina; Carine Moustaud; Florence Morfin-Sherpa; Gregory Destras; Gwendolyne Burfin; Laurence Josset; Martine Valette; Maude Bouscambert-Duchamp; Raphaelle Lamy; Solenne Brun
EPI_ISL_1406575, EPI_ISL_1406650, EPI_ISL_1483754, EPI_ISL_1547426, EPI_ISL_1547428, EPI_ISL_1547452	Centre Hospitalier Paul Ardier	CHU Clermont-Ferrand, service de virologie	Bisseux Maxime; Combes Patricia; Henquell Cécile; Mirand Audrey
EPI_ISL_508966, EPI_ISL_508975, EPI_ISL_508998	Centre Hospitalier Pierre Oudot	CNR Virus des Infections Respiratoires - France SUD	Alexandre Gaymard; Antonin Bal; Bruno Lina; Carine Moustaud; Florence Morfin-Sherpa; Gregory Destras; Gwendolyne Burfin; Laurence Josset; Martine Valette; Maude Bouscambert-Duchamp; Raphaelle Lamy; Solenne Brun
EPI_ISL_1406443, EPI_ISL_1406447, EPI_ISL_1406471, EPI_ISL_1406487, EPI_ISL_1406499, EPI_ISL_1406528 to 1406529, EPI_ISL_1406588, EPI_ISL_1406603, EPI_ISL_1406634, EPI_ISL_1406670, EPI_ISL_1483729, EPI_ISL_1483752, EPI_ISL_1483756, EPI_ISL_1547429, EPI_ISL_1547434, EPI_ISL_1547460			
see above	Centre Hospitalier RIOM	CHU Clermont-Ferrand, service de virologie	Bisseux Maxime; Combes Patricia; Henquell Cécile; Mirand Audrey
EPI_ISL_414633	Centre Hospitalier René Dubois Laboratoire de Microbiologie - Bât A	National Reference Center for Viruses of Respiratory Infections, Institut Pasteur, Paris	Angela Brisebarre; Flora Donati Vincent Enouf; Marion Barbet; Maud Vanpeene; Méline Bizard; Mélnie Albert; Pascale Martres; Sylvie Behillil; Sylvie van der Werf
EPI_ISL_414625	Centre Hospitalier Régional Universitaire de Nantes Laboratoire de Virologie	National Reference Center for Viruses of Respiratory Infections, Institut Pasteur, Paris	Angela Brisebarre; Flora Donati Vincent Enouf; Marianne Coste-Burel; Marion Barbet; Maud Vanpeene; Méline Bizard; Mélnie Albert; Sylvie Behillil; Sylvie van der Werf
EPI_ISL_535787 to 535788, EPI_ISL_535790, EPI_ISL_535842 to 535844, EPI_ISL_535870 to 535871, EPI_ISL_535894 to 535896, EPI_ISL_535907, EPI_ISL_535931 to 535935, EPI_ISL_535955, EPI_ISL_536002, EPI_ISL_536005 to 536006, EPI_ISL_536009, EPI_ISL_536012, EPI_ISL_536014 to 536016, EPI_ISL_536047 to 536048, EPI_ISL_536050, EPI_ISL_536052 to 536054, EPI_ISL_536059 to 536060, EPI_ISL_536063, EPI_ISL_536073 to 536074, EPI_ISL_536076, EPI_ISL_536079, EPI_ISL_536119 to 536120, EPI_ISL_536123, EPI_ISL_536126, EPI_ISL_536129 to 536130, EPI_ISL_536133 to 536134, EPI_ISL_536138, EPI_ISL_536151, EPI_ISL_536153 to 536154, EPI_ISL_536381, EPI_ISL_560415			
see above	Centre Hospitalier Régional de Lanaudière	Laboratoire de santé publique du Québec	Guillaume Bourque; Ioannis Ragoussis; Jesse Shapiro; Mark Lathrop and Michel Roger; Mark Lathrop and Michel Roger on behalf of the CoVSeQ research group; Sandrine Moreira
EPI_ISL_418418 to 418419, EPI_ISL_420617, EPI_ISL_508879 to 508880, EPI_ISL_508959 to 508960	Centre Hospitalier Saint Joseph Saint Luc	CNR Virus des Infections Respiratoires - France SUD	Alexandre Gaymard; Antonin Bal; Bruno Lina; Carine Moustaud; Florence Morfin-Sherpa; Gregory Destras; Gwendolyne Burfin; Laurence Josset; Martine Valette; Maude Bouscambert-Duchamp; Raphaelle Lamy; Solenne Brun
EPI_ISL_1406437 to 1406438, EPI_ISL_1406440 to 1406441, EPI_ISL_1406444 to 1406445, EPI_ISL_1406448 to 1406451, EPI_ISL_1406455, EPI_ISL_1406457, EPI_ISL_1406460, EPI_ISL_1406462, EPI_ISL_1406464 to 1406470, EPI_ISL_1406474 to 1406476, EPI_ISL_1406479 to 1406483, EPI_ISL_1406485, EPI_ISL_1406488 to 1406496, EPI_ISL_1406501 to 1406506, EPI_ISL_1406508 to 1406511, EPI_ISL_1406513 to 1406517, EPI_ISL_1406519 to 1406527, EPI_ISL_1406532, EPI_ISL_1406534, EPI_ISL_1406536 to 1406539, EPI_ISL_1406541 to 1406542, EPI_ISL_1406544, EPI_ISL_1406546 to 1406548, EPI_ISL_1406550 to 1406570, EPI_ISL_1406572 to 1406573, EPI_ISL_1406576 to 1406584, EPI_ISL_1406586, EPI_ISL_1406591, EPI_ISL_1406595, EPI_ISL_1406600, EPI_ISL_1406602, EPI_ISL_1406604 to 1406605, EPI_ISL_1406607, EPI_ISL_1406609 to 1406610, EPI_ISL_1406612 to 1406615, EPI_ISL_1406617, EPI_ISL_1406620 to 1406622, EPI_ISL_1406624 to 1406629, EPI_ISL_1406631 to 1406633, EPI_ISL_1406635 to 1406636, EPI_ISL_1406638, EPI_ISL_1406641 to 1406648, EPI_ISL_1406653 to 1406655, EPI_ISL_1406658 to 1406659, EPI_ISL_1406663, EPI_ISL_1406666 to 1406668, EPI_ISL_1406675, EPI_ISL_1406680, EPI_ISL_1406683 to 1406687, EPI_ISL_1483708 to 1483709, EPI_ISL_1483711 to 1483718, EPI_ISL_1483721 to 1483723, EPI_ISL_1483725, EPI_ISL_1483727 to 1483728, EPI_ISL_1483730 to 1483737, EPI_ISL_1483739, EPI_ISL_1483742 to 1483747, EPI_ISL_1483749 to 1483751, EPI_ISL_1483753, EPI_ISL_1483755, EPI_ISL_1483757 to 1483758, EPI_ISL_1483762 to 1483768, EPI_ISL_1483773 to 1483776, EPI_ISL_1483783 to 1483787, EPI_ISL_1490230, EPI_ISL_1490232, EPI_ISL_1490236, EPI_ISL_1490238 to 1490243, EPI_ISL_1492562 to 1492563, EPI_ISL_1547393 to 1547396, EPI_ISL_1547399, EPI_ISL_1547401 to 1547406, EPI_ISL_1547408 to 1547411, EPI_ISL_1547413 to 1547416, EPI_ISL_1547418 to 1547420, EPI_ISL_1547422 to 1547425, EPI_ISL_1547430 to 1547433, EPI_ISL_1547435 to 1547440, EPI_ISL_1547445 to 1547446, EPI_ISL_1547448 to 1547451, EPI_ISL_1547455, EPI_ISL_1547457 to 1547458, EPI_ISL_1547463 to 1547464, EPI_ISL_1547468, EPI_ISL_1547471			
see above	Centre Hospitalier Universitaire Clermont-Ferrand	CHU Clermont-Ferrand, service de virologie	Bisseux Maxime; Combes Patricia; Henquell Cécile; Mirand Audrey
EPI_ISL_416757, EPI_ISL_417340, EPI_ISL_418426, EPI_ISL_419183, EPI_ISL_419185 to 419186, EPI_ISL_420620, EPI_ISL_508938, EPI_ISL_508944, EPI_ISL_508968, EPI_ISL_525539, EPI_ISL_582121			
see above	Centre Hospitalier de Bourg en Bresse	CNR Virus des Infections Respiratoires - France SUD	Alexandre; Alexandre Gaymard; Antonin; Antonin Bal; Bal; Bouscambert-Duchamp; Brengel-Pesce; Bruno Lina; Bruno.; Carine Moustaud; Cheynet; Destras; Florence; Florence Morfin-Sherpa; Gaymard; Gregory; Gregory Destras; Gwendolyne Burfin; Hadrien Règue; Josset; Karen; Laurence; Laurence Josset; Lina; Martine; Martine Valette; Maude; Maude Bouscambert-Duchamp; Morfin-Sherpa; Raphaelle Lamy; Solenne Brun; Valette; Valérie
EPI_ISL_417338, EPI_ISL_418413, EPI_ISL_419174 to 419176, EPI_ISL_419187 to 419188, EPI_ISL_420612 to 420614, EPI_ISL_508875 to 508876, EPI_ISL_508941, EPI_ISL_508943, EPI_ISL_508946, EPI_ISL_508949 to 508950, EPI_ISL_508952, EPI_ISL_509005			
see above	Centre Hospitalier de Macon	CNR Virus des Infections Respiratoires - France SUD	Alexandre Gaymard; Antonin Bal; Bruno Lina; Carine Moustaud; Florence Morfin-Sherpa; Gregory Destras; Gwendolyne Burfin; Laurence Josset; Martine Valette; Maude Bouscambert-Duchamp; Raphaelle Lamy; Solenne Brun
EPI_ISL_416749, EPI_ISL_418414 to 418415, EPI_ISL_418417, EPI_ISL_419168, EPI_ISL_508881	Centre Hospitalier de Valence	CNR Virus des Infections Respiratoires - France SUD	Alexandre; Alexandre Gaymard; Antonin; Antonin Bal; Bal; Bouscambert-Duchamp; Brengel-Pesce; Bruno Lina; Bruno.; Carine Moustaud; Cheynet; Destras; Florence; Florence Morfin-Sherpa; Gaymard; Gregory; Gregory Destras; Gwendolyne Burfin; Josset; Karen; Laurence; Laurence Josset; Lina; Martine; Martine Valette; Maude; Maude Bouscambert-Duchamp; Morfin-Sherpa; Raphaelle Lamy; Solenne Brun; Valette; Valérie
EPI_ISL_508932, EPI_ISL_508978, EPI_ISL_509006, EPI_ISL_509015	Centre Hospitalier de Villefranche	CNR Virus des Infections Respiratoires - France SUD	Alexandre Gaymard; Antonin Bal; Bruno Lina; Carine Moustaud; Florence Morfin-Sherpa; Gregory Destras; Gwendolyne Burfin; Laurence Josset; Martine Valette; Maude Bouscambert-Duchamp; Raphaelle Lamy; Solenne Brun
EPI_ISL_418412	Centre Hospitalier des Vals d'Ardeche	CNR Virus des Infections Respiratoires - France SUD	Alexandre Gaymard; Antonin Bal; Bruno Lina; Carine Moustaud; Florence Morfin-Sherpa; Gregory Destras; Gwendolyne Burfin; Laurence Josset; Martine Valette; Maude Bouscambert-Duchamp; Raphaelle Lamy; Solenne Brun
EPI_ISL_509016	Centre Hospitalier du Haut-Bugey	CNR Virus des Infections Respiratoires - France SUD	Alexandre Gaymard; Antonin Bal; Bruno Lina; Carine Moustaud; Florence Morfin-Sherpa; Gregory Destras; Gwendolyne Burfin; Laurence Josset; Martine Valette; Maude Bouscambert-Duchamp; Raphaelle Lamy; Solenne Brun
EPI_ISL_1477063 to 1477128, EPI_ISL_1522577, EPI_ISL_1522581, EPI_ISL_1522583, EPI_ISL_1522589, EPI_ISL_1522625, EPI_ISL_1522628, EPI_ISL_1522638, EPI_ISL_1522647, EPI_ISL_1522664, EPI_ISL_1522690, EPI_ISL_1522703, EPI_ISL_1522705, EPI_ISL_1522723 to 1522724, EPI_ISL_1522726, EPI_ISL_1522786 to 1522787, EPI_ISL_1522840 to 1522847, EPI_ISL_1523040 to 1523046, EPI_ISL_1523115, EPI_ISL_1523122, EPI_ISL_1523134, EPI_ISL_1523190, EPI_ISL_1523252 to 1523255			
see above	Centre Hospitalier du Nord	Laboratoire national de sante, Microbiology, Microbial Genomics Platform	Anke Wienecke-Baldacchino; Catherine Ragimbeau; Fatiha Boulmerka; Fatu Djabi; Jessica Tapp; Lise Pignon; Raoul Salmon; Tamir Abdelrahman
EPI_ISL_1406540, EPI_ISL_1406637, EPI_ISL_1406681, EPI_ISL_1490237	Centre Jean Perrin	CHU Clermont-Ferrand, service de virologie	Bisseux Maxime; Combes Patricia; Henquell Cécile; Mirand Audrey
EPI_ISL_512873	Centre Pasteur of Cameroun	Virology Service, Centre Pasteur of Cameroun	Richard Njouom and Serge Alain SADEUH-Mba
EPI_ISL_1495973	Centre de Biologie humaine CHU Amiens Laboratoire de Virologie 80000 Amiens	National Reference Center for Viruses of Respiratory Infections, Institut Pasteur, Paris	Angela Brisebarre; Camille Capel; Castelain Sandrine; Etienne Simon-Lorière; Louise Lefrançois; Marion Barbet; Maud Vanpeene; Méline Bizard; Sylvie Behillil; Sylvie van der Werf; Vincent Enouf
EPI_ISL_482820	Centre de Recerca en Sanitat Animal (IRTA-CReSA)	IrsiCaixa AIDS Research Lab	A. Valencia; B. Clotet; C. Avila-Nieto; E. Vidal; G. Cantero; I. Blanco; J. Blanco; J. Carrillo; J. Rodon; J. Segalés; J. Vergara-Alert; M. Noguera-Julián; M. Parera; M. Puig; M.T. Terrón; N. Izquierdo-Useros; S. Cruz; V. Guallar
EPI_ISL_539573 to 539576	Centre de Recherches Medicales de Lambarene (CERMEL)	Department of Emerging Infectious Diseases, Institute of	Akim A. Adegnika; Bertrand Lell; Haruka Abe; Jiro Yasuda; Rodrigue Bikangui; Yuri Ushijima

		Tropical Medicine, Nagasaki University	
EPI_ISL_535845, EPI_ISL_535939 to 535940, EPI_ISL_560413 to 560414	Centre de SSS D'Arthabaska-et-de-l'érable - Hôtel-Dieu	Laboratoire de santé publique du Québec	Guillaume Bourque; Ioannis Ragoussis; Jesse Shapiro; Mark Lathrop and Michel Roger; Mark Lathrop and Michel Roger on behalf of the CoVSeQ research group; Sandrine Moreira
EPI_ISL_535724, EPI_ISL_535742 to 535743	Centre de SSS La Pommeraie	Laboratoire de santé publique du Québec	Guillaume Bourque; Ioannis Ragoussis; Jesse Shapiro; Mark Lathrop and Michel Roger; Mark Lathrop and Michel Roger on behalf of the CoVSeQ research group; Sandrine Moreira
EPI_ISL_535722, EPI_ISL_535747 to 535748, EPI_ISL_535849, EPI_ISL_535916, EPI_ISL_535976 to 535977, EPI_ISL_535979, EPI_ISL_536007, EPI_ISL_536031, EPI_ISL_536034 to 536036, EPI_ISL_536038			
see above	Centre de SSS de Trois-Rivières	Laboratoire de santé publique du Québec	Guillaume Bourque; Ioannis Ragoussis; Jesse Shapiro; Mark Lathrop and Michel Roger; Mark Lathrop and Michel Roger on behalf of the CoVSeQ research group; Sandrine Moreira
EPI_ISL_535727, EPI_ISL_535776 to 535780, EPI_ISL_535803 to 535805, EPI_ISL_535816, EPI_ISL_535854 to 535855	Centre de SSS de la Haute-Yamaska	Laboratoire de santé publique du Québec	Guillaume Bourque; Ioannis Ragoussis; Jesse Shapiro; Mark Lathrop and Michel Roger; Mark Lathrop and Michel Roger on behalf of the CoVSeQ research group; Sandrine Moreira
EPI_ISL_471456 to 471460	Centre de Virologie des Maladies Tropicales	Functional Genomic Platform/Service Analyses Biologique/UATRS/ Centre National Pour la Recherche Scientifique Et Technique (CNRST)	Abdelillah LARAQUI; Abdelkader LAATIRIS; Ahmed REGGAD; Elmostafa EL FAHIME; Farida HILALI; Hicham EL ANNAZ; Idriss-Amine LAHLOU; Khalid ENNIBI; Marouane MELLOUL; Mly Abdelaziz ELALAOUI; Mostafa ELOUENNASS; Nadia TOUIL; Rachid ABI; Rida TAGAJDID; Safae ELKOCHRI; Sanaa ALAOUI-Amine; Tahar BAJJOU; Yassine SEKHSOKH; Youssef AKHOUAD; Zohour KASMY
EPI_ISL_535808	Centre de santé Chibougamau	Laboratoire de santé publique du Québec	Guillaume Bourque; Ioannis Ragoussis; Jesse Shapiro; Mark Lathrop and Michel Roger; Sandrine Moreira
EPI_ISL_443310	Centre de santé Filieris	National Reference Center for Viruses of Respiratory Infections, Institut Pasteur, Paris	Angela Brisebarre; Etienne Simon-Lorière; Flora Donati; Marion Barbet; Maud Vanpeene; Mélanie Albert; Méline Bizard; Sylvie Behillil; Sylvie van der Werf; Vincent Enouf
EPI_ISL_465697, EPI_ISL_536353	Centre de santé Inuulitsivik	Laboratoire de santé publique du Québec	Guillaume Bourque; Ioannis Ragoussis; Jesse Shapiro; Mark Lathrop and Michel Roger on behalf of the CoVSeQ research group; Sandrine Moreira
EPI_ISL_483916 to 484217	Centre for Clinical Infection and Diagnostics Research and Genomics Innovation Unit, Guy's and St. Thomas' NHS Trust	COVID-19 Genomics UK (COG-UK) Consortium	Ali Raza Awan; Chloe Fisher; Jonathan Edgeworth; Luke Snell; Penny Cliff; Rahul Batra
EPI_ISL_560649 to 560740, EPI_ISL_561363 to 561369, EPI_ISL_561373 to 561374	Centre for Clinical Infection and Diagnostics Research and Genomics Innovation Unit, Guy's and St. Thomas' NHS Trust	Centre for Clinical Infection and Diagnostics Research and Genomics Innovation Unit, Guy's and St. Thomas' NHS Trust	Ali Raza Awan; Chloe Fisher; Jonathan Edgeworth; Luke Snell; Rahul Batra
EPI_ISL_428670 to 428673, EPI_ISL_525476, EPI_ISL_525478 to 525479, EPI_ISL_525481, EPI_ISL_525486, EPI_ISL_525488 to 525489	Centre for Dengue Research	Centre for Dengue Research	Ananda Wijewickrama; Chandima Jeewandara; Damayanthi Idampitiya; Damayanthi Tdampitiya; Deshni Jayathilaka; Dinuka Ariyaratne; Dinuka Ariyatane; Dinuka Guruge; Diyanath Ranasinghe; Eranga Narangoda; Gathsaurie Neelika Malavige; Laksiri Gomes; Malika Karunaratne; Neelika Malaige; Neelika Malavige; Ruwan Wijayamuni
EPI_ISL_525474	Centre for Dengue Research	Centre for Dengue Research, USJ, SL	Chandima Jeewandara; Deshni Jayathilaka; Dinuka Ariyaratne; Dinuka Guruge; Diyanath Ranasinghe; Gathsaurie Neelika Malavige; Laksiri Gomes; Ruwan Wijayamuni
EPI_ISL_1533803 to 1533851	Centre for Dengue Research and AICBU, Department of Immunology and Molecular Medicine	Centre for Dengue Research and AICBU, Department of Immunology and Molecular Medicine	Chandima Jeewandara; Deshan Madhusanka; Deshni Jayathilaka; Dinuka Ariyaratne; Diyanath Ranasinghe; Gathsaurie Neelika Malavige; Laksiri Gomes
EPI_ISL_602564	Centre for Dengue Research, Department of Immunology and Molecular Medicine	Centre for Dengue Research	Ananda Wijewickrama; Chandima Jeewandara; Deshni Jayathilaka; Dinuka Ariyaratne; Diyanath Ranasinghe; Gathsaurie Neelika Malavige; Laksiri Gomes; Malika Karunaratne
EPI_ISL_602566 to 602576	Centre for Dengue Research, Department of Immunology and Molecular Medicine	Centre for Dengue Research, Department of Immunology and Molecular Medicine	Ananda Wijewickrama; Chandima Jeewandara; Deshni Jayathilaka; Dinuka Ariyaratne; Dinuka Guruge; Diyanath Ranasinghe; Gathsaurie Neelika Malavige; Laksiri Gomes; Malika Karunaratne; Ruwan Wiajayamuni
EPI_ISL_602565	Centre for Dengue Research, Department of Immunology and Molecular Medicine	Centre for Dengue Research, Department of Immunology and Molecular Medicine,	Chandima Jeewandara; Deshni Jayathilaka; Dinuka Ariyaratne; Dinuka Guruge; Diyanath Ranasinghe; Gathsaurie Neelika Malavige; Laksiri Gomes; Ruwan Wiajayamuni
EPI_ISL_453666 to 453695, EPI_ISL_461970 to 461981, EPI_ISL_461983 to 461997, EPI_ISL_475239 to 475265, EPI_ISL_475267 to 475293, EPI_ISL_475295 to 475303, EPI_ISL_475306 to 475341, EPI_ISL_479175 to 479181, EPI_ISL_479183 to 479194, EPI_ISL_484407 to 484408, EPI_ISL_484410 to 484432, EPI_ISL_493615 to 493623, EPI_ISL_493625 to 493665, EPI_ISL_499270 to 499329, EPI_ISL_507131 to 507157, EPI_ISL_512384 to 512388, EPI_ISL_512392 to 512435, EPI_ISL_514455 to 514457, EPI_ISL_514459 to 514466, EPI_ISL_514468 to 514513, EPI_ISL_517510 to 517530, EPI_ISL_517532 to 517541, EPI_ISL_526433 to 526436, EPI_ISL_529272, EPI_ISL_529303, EPI_ISL_529341 to 529352, EPI_ISL_529373 to 529377, EPI_ISL_529431 to 529432, EPI_ISL_529465, EPI_ISL_529481, EPI_ISL_529491, EPI_ISL_529511, EPI_ISL_529525, EPI_ISL_529668 to 529674, EPI_ISL_535024 to 535025, EPI_ISL_535027 to 535042, EPI_ISL_549341, EPI_ISL_549343, EPI_ISL_549360, EPI_ISL_549363 to 549364, EPI_ISL_549366, EPI_ISL_549460 to 549476, EPI_ISL_559983, EPI_ISL_572715 to 572716, EPI_ISL_572721, EPI_ISL_573253 to 573265, EPI_ISL_573870, EPI_ISL_577040 to 577067, EPI_ISL_577193 to 577205, EPI_ISL_577207 to 577219, EPI_ISL_577299 to 577345, EPI_ISL_577381 to 577382, EPI_ISL_577385 to 577400, EPI_ISL_577545 to 577546, EPI_ISL_584168 to 584187, EPI_ISL_584193 to 584195, EPI_ISL_584200 to 584206, EPI_ISL_584212 to 584216, EPI_ISL_584809 to 584810, EPI_ISL_595100 to 595114, EPI_ISL_611526, EPI_ISL_611532, EPI_ISL_611535, EPI_ISL_611537 to 611538, EPI_ISL_611555, EPI_ISL_611557, EPI_ISL_611585, EPI_ISL_611589 to 611590, EPI_ISL_611592, EPI_ISL_611638 to 611639, EPI_ISL_611689, EPI_ISL_611754 to 611755, EPI_ISL_611761, EPI_ISL_611763, EPI_ISL_611771 to 611773, EPI_ISL_611841, EPI_ISL_611916, EPI_ISL_611925, EPI_ISL_611927 to 611928, EPI_ISL_611933 to 611934, EPI_ISL_611957 to 611960, EPI_ISL_611962, EPI_ISL_611971, EPI_ISL_611983, EPI_ISL_611998, EPI_ISL_612068 to 612069, EPI_ISL_612082, EPI_ISL_612089, EPI_ISL_612092, EPI_ISL_612094, EPI_ISL_613282 to 613298, EPI_ISL_613302 to 613306, EPI_ISL_1386409, EPI_ISL_1386932, EPI_ISL_1388643, EPI_ISL_1388646 to 1388647, EPI_ISL_1388649, EPI_ISL_1388651, EPI_ISL_1388660 to 1388661, EPI_ISL_1388664 to 1388665, EPI_ISL_1388667, EPI_ISL_1388670, EPI_ISL_1388672, EPI_ISL_1388674, EPI_ISL_1388676 to 1388677, EPI_ISL_1388680, EPI_ISL_1388682, EPI_ISL_1388684, EPI_ISL_1388686, EPI_ISL_1388688, EPI_ISL_1388690, EPI_ISL_1388692, EPI_ISL_1388694, EPI_ISL_1388696, EPI_ISL_1388698, EPI_ISL_1388700, EPI_ISL_1388702, EPI_ISL_1388704, EPI_ISL_1388706, EPI_ISL_1388708, EPI_ISL_1388710, EPI_ISL_1388712, EPI_ISL_1388714 to 1388715, EPI_ISL_1388717, EPI_ISL_1388719, EPI_ISL_1388721, EPI_ISL_1388724 to 1388725, EPI_ISL_1388728, EPI_ISL_1388730, EPI_ISL_1388732, EPI_ISL_1388734, EPI_ISL_1388736, EPI_ISL_1388738 to 1388739, EPI_ISL_1388743, EPI_ISL_1388745, EPI_ISL_1388747, EPI_ISL_1388749, EPI_ISL_1388752, EPI_ISL_1388754, EPI_ISL_1388756, EPI_ISL_1388758, EPI_ISL_1388760, EPI_ISL_1388764, EPI_ISL_1388766, EPI_ISL_1388768, EPI_ISL_1388770, EPI_ISL_1388772, EPI_ISL_1388775, EPI_ISL_1388777, EPI_ISL_1388779, EPI_ISL_1388782, EPI_ISL_1388784, EPI_ISL_1388786, EPI_ISL_1388788, EPI_ISL_1388790, EPI_ISL_1388792, EPI_ISL_1388794, EPI_ISL_1388796, EPI_ISL_1388798, EPI_ISL_1388800, EPI_ISL_1388802, EPI_ISL_1388804, EPI_ISL_1388806, EPI_ISL_1388808, EPI_ISL_1388863, EPI_ISL_1388865, EPI_ISL_1388867, EPI_ISL_1388870, EPI_ISL_1388872, EPI_ISL_1388874, EPI_ISL_1388876, EPI_ISL_1388878, EPI_ISL_1388880, EPI_ISL_1388882, EPI_ISL_1388884, EPI_ISL_1388886, EPI_ISL_1388888, EPI_ISL_1388890 to 1388891, EPI_ISL_1388893, EPI_ISL_1388896 to 1388897, EPI_ISL_1388899, EPI_ISL_1388901, EPI_ISL_1388904, EPI_ISL_1388906, EPI_ISL_1388908, EPI_ISL_1388910, EPI_ISL_1388912, EPI_ISL_1388914, EPI_ISL_1388917, EPI_ISL_1388919, EPI_ISL_1388921, EPI_ISL_1388923, EPI_ISL_1388925, EPI_ISL_1388927, EPI_ISL_1388929, EPI_ISL_1388931, EPI_ISL_1476549 to 1476565, EPI_ISL_1476665, EPI_ISL_1476672 to 1476685, EPI_ISL_1547230 to 1547258, EPI_ISL_1547300, EPI_ISL_1547304 to 1547309			
see above	Centre for Enzyme Innovation, University of Portsmouth / Translational Research Laboratory, Portsmouth Hospitals NHS Trust	COVID-19 Genomics UK (COG-UK) Consortium	; Allyson Lloyd; Angela Beckett; Anoop Chauhan; Christopher Fearn; Ethan Butcher; Garry Scarlett; Kate Cook; Katie Loveson; Kelly Bicknell; Robert Impey; Salman Goudarzi; Samuel Robson; Sarah Wyllie; Scott Elliott; Sharon Glaysher; Yann Bourgeois
EPI_ISL_453665	Centre for Enzyme Innovation, University of Portsmouth / Translational Research Laboratory, Portsmouth Hospitals NHS Trust	University of Portsmouth	; Allyson Lloyd; Angela Beckett; Anoop Chauhan; Ethan Butcher; Garry Scarlett; Kelly Bicknell; Robert Impey; Samuel Robson; Sarah Wyllie; Scott Elliott; Sharon Glaysher; Yann Bourgeois
EPI_ISL_413550	Centre for Human and Zoonotic Virology (CHAZVY), College of Medicine University of Lagos/Lagos University Teaching Hospital (LUTH), part of the Laboratory Network of the Nigeria Centre for Disease Control (NCDC)	African Centre of Excellence for Genomics of Infectious Diseases (ACEGID), Redeemer's University, Ede, Osun State, Nigeria	Ajogbasile F.V.; Folarin O.A.; Ihekweazu C. Happi C.T.; Kayode A.; Oguzie J.; Oluniyi P.E.
EPI_ISL_413596, EPI_ISL_413598 to 413599	Centre for Infectious Diseases and Microbiology - Public Health	NSW Health Pathology - Institute of Clinical Pathology and Medical Research; Westmead Hospital; University of Sydney	Carter I; Chen SC; Eden J-S; Gall; Gray K; Holmes EC; Kok J and Dwyer DE for the 2019-nCoV Study Group*; Lam C; M; Maddocks S; O'Sullivan MV; Rahman H; Rockett R; Sintchenko V; Timms; V
EPI_ISL_407893, EPI_ISL_408976, EPI_ISL_412975, EPI_ISL_413213 to 413214, EPI_ISL_413594 to 413595, EPI_ISL_417030	Centre for Infectious Diseases and Microbiology Laboratory Services	NSW Health Pathology - Institute of Clinical Pathology and Medical Research; Westmead Hospital; University of Sydney	; A; Alicia; Carter I; Chen SC; Eden J-S; Gall; Gray K; Holmes EC; Kok J and Dwyer DE for the 2019-nCoV Study Group; Kok J and Dwyer DE for the 2019-nCoV Study Group*; Lam C; M; Maddocks S; O'Sullivan MV; Rahman H; Rockett R; Sadsad R; Sintchenko V; Timms; V
EPI_ISL_417383 to 417412, EPI_ISL_427643 to 427710, EPI_ISL_427712 to 427716, EPI_ISL_427720, EPI_ISL_427722 to 427808	Centre for Infectious Diseases and Microbiology Public Health	NSW Health Pathology - Institute of Clinical Pathology and Medical Research; Westmead Hospital; University of Sydney	Arnott A; Arnott A and Sadsad R for the 2019-nCoV Study Group; Bachmann N; Basile K; Byun R; Carter I; Carter I and Rahman H for the 2019-nCoV Study Group; Chang S; Chen SC; Chen SC and Maddocks S for the 2019-nCoV Study Group; Draper J; Dwyer DE; Dwyer DE and Rockett R for the 2019-nCoV Study Group; Dwyer DE for the 2019-nCoV Study Group; Eden J-S; Eden J-S and Lam C for the 2019-nCoV Study Group; Eden JS; Gall M; Gall M and Arnott A for the 2019-nCoV Study Group; Gray K; Gray K and Timms V for the 2019-nCoV Study Group; Holmes EC; Holmes EC and O'Sullivan MV for the 2019-nCoV Study Group; Kok J; Kok J and Dwyer DE for the 2019-nCoV Study Group; Lam C; Lam C and Gray K for the 2019-nCoV Study Group; Maddocks S; Maddocks S and Kok J for the 2019-nCoV Study Group; O'Sullivan MV; O'Sullivan MV and Sintchenko V for the 2019-nCoV Study Group; Propenko M; Rahman H; Rahman H and Holmes EC for the 2019-nCoV Study Group; Rockett R; Rockett R and Eden J-S for the 2019-nCoV Study Group; Sadsad R; Sadsad R and Carter I for the 2019-nCoV Study Group; Sim E; Sintchenko V; Sintchenko V and Chen SC for the 2019-nCoV Study Group; Sorrell T; Timms V; Timms V and Gall M for the 2019-nCoV Study Group; V
EPI_ISL_413597	Centre for Infectious Diseases and Microbiology- Public Health	NSW Health Pathology - Institute of Clinical Pathology and	Carter I; Chen SC; Eden J-S; Gall; Gray K; Holmes EC; Kok J and Dwyer DE for the 2019-nCoV Study Group*; Lam C; M; Maddocks S; O'Sullivan MV;

		Medical Research; Westmead Hospital; University of Sydney	Rahman H; Rockett R; Sintchenko V; Timms; V
EPI_ISL_450302 to 450303, EPI_ISL_465688 to 465690, EPI_ISL_535960, EPI_ISL_535980 to 535981, EPI_ISL_536000, EPI_ISL_536004, EPI_ISL_536023, EPI_ISL_536043, EPI_ISL_536045, EPI_ISL_536080, EPI_ISL_536104, EPI_ISL_536141 to 536142, EPI_ISL_536145, EPI_ISL_536155 to 536156, EPI_ISL_536159, EPI_ISL_536162, EPI_ISL_536182 to 536184, EPI_ISL_536211 to 536212, EPI_ISL_536218 to 536221, EPI_ISL_536224, EPI_ISL_536232, EPI_ISL_536240 to 536241, EPI_ISL_536255, EPI_ISL_536283, EPI_ISL_536315, EPI_ISL_536325, EPI_ISL_536329, EPI_ISL_536348, EPI_ISL_536368 to 536370, EPI_ISL_536376 to 536378			
see above	Centre hospitalier Anna-Laberge	Laboratoire de santé publique du Québec	Guillaume Bourque; Ioannis Ragoussis; Jesse Shapiro; Mark Lathrop and Michel Roger; Mark Lathrop and Michel Roger on behalf of the CoVSeQ research group; Sandrine Moreira
EPI_ISL_1406456, EPI_ISL_1406477, EPI_ISL_1406512, EPI_ISL_1406533, EPI_ISL_1406592, EPI_ISL_1406601, EPI_ISL_1406608, EPI_ISL_1406618, EPI_ISL_1483719 to 1483720, EPI_ISL_1483726, EPI_ISL_1547397 to 1547398, EPI_ISL_1547407, EPI_ISL_1547417, EPI_ISL_1547461			
see above	Centre hospitalier D'AURILLAC	CHU Clermont-Ferrand, service de virologie	Bisseux Maxime; Combes Patricia; Henquell Cécile; Mirand Audrey
EPI_ISL_508934 to 508936	Centre hospitalier Métropole Savoie	CNR Virus des Infections Respiratoires - France SUD	Alexandre Gaymard; Antonin Bal; Bruno Lina; Carine Moustaud; Florence Morfin-Sherpa; Gregory Destras; Gwendolyne Burfin; Laurence Josset; Martine Valette; Maude Bouscambert-Duchamp; Raphaelle Lamy; Solenne Brun
EPI_ISL_535746, EPI_ISL_535767 to 535768	Centre hospitalier de St-Mary	Laboratoire de santé publique du Québec	Guillaume Bourque; Ioannis Ragoussis; Jesse Shapiro; Mark Lathrop and Michel Roger; Sandrine Moreira
EPI_ISL_535807, EPI_ISL_535872 to 535873, EPI_ISL_535958 to 535959, EPI_ISL_536115, EPI_ISL_536199, EPI_ISL_536204	Centre hospitalier régional du Grand Portage	Laboratoire de santé publique du Québec	Guillaume Bourque; Ioannis Ragoussis; Jesse Shapiro; Mark Lathrop and Michel Roger; Mark Lathrop and Michel Roger on behalf of the CoVSeQ research group; Sandrine Moreira
EPI_ISL_413647, EPI_ISL_417997 to 417999	Centro Hospital do Porto, E.P.E. - H. Geral de Santo Antonio	Instituto Nacional de Saude (INSA)	Guiomar et al; Helena Ramos; Inês Costa; Joana Isidro; Joana Mendonça; João Paulo Gomes; Luís Vieira; Pedro Pechirra; Raquel Guiomar; Vítor Borges
EPI_ISL_1494714 to 1494716, EPI_ISL_1494757 to 1494811, EPI_ISL_1494875 to 1494914	Centro Hospitalar de Entre o Douro e Vouga (CHEDV)	Institute of Biomedicine (IBiMED), Universidade de Aveiro	Gabriela Moura; Miguel Pinheiro and Manuel Santos; Patricia Arinto; Sofia Marques
EPI_ISL_1494812 to 1494874, EPI_ISL_1516760 to 1516761	Centro Hospitalar do Baixo Vouga (CHBV)	Institute of Biomedicine (IBiMED), Universidade de Aveiro	Gabriela Moura; Miguel Pinheiro and Manuel Santos; Patricia Arinto; Sofia Marques
EPI_ISL_417986 to 417987, EPI_ISL_417989	Centro Hospitalar e Universitario de Sao Joao, Porto	Instituto Nacional de Saude (INSA)	Guiomar et al
EPI_ISL_413648	Centro Hospitalar e Universitário de Sao Joao, Porto	Instituto Nacional de Saude (INSA)	Inês Costa; Joana Isidro; Joana Mendonça; João Paulo Gomes; João Tiago Guimarães; Luís Vieira; Pedro Pechirra; Raquel Guiomar; Vítor Borges
EPI_ISL_515547	Centro Medico da Policia Militar do Estado de Sao Paulo	Instituto Adolfo Lutz, Interdiciplinary Procedures Center, Strategic Laboratory	Claudia Regina Gonçalves; Claudio Tavares Sacchi; Erica Valessa Ramos Gomes
EPI_ISL_1469655, EPI_ISL_1469776	Centro Municipal de Saúde de Rolante	Epiclin	Ana Paula Muterle; Carolina Comerlato; Eliana Márcia Da Ros Wendland; Fernando Hayashi Sant'Anna; Janira Prichula; Juliana Comerlato
EPI_ISL_512670, EPI_ISL_527742, EPI_ISL_527755	Centro Nacional De Rehabilitacion Humberto Araya Rojas (Cenare)	Inciensa, Instituto Costarricense de Investigación y Enseñanza en Nutrición y Salud	Adriana Godinez & Melany Calderon; Claudio Soto-Garita; Estela Cordero; Francisco Duarte; Hebleen Porras
EPI_ISL_1494948	Centro de Atención y Diagnóstico de Enfermedades Infecciosas	Instituto Nacional de Salud- Dirección de Investigación en Salud Pública	Carlos Franco-Muñoz; Carmen Osorio; Diana Malo; Diego A. Álvarez-Díaz; Diego Andrés Prada; Gerardo Santamaría; Hector Alejandro Ruiz-Moreno; Jhonnatan Reales-González; Juan Camilo Martinez; Julian Naizaque; Katherine Laiton-Donato; Lisseth Pardo; Magdalena Wiesner; Marcela Mercado-Reyes; Maria T. Herrera-Sepúlveda; Marta Lopez Blanco; Martha Lucia Ospina Martinez; Paola Rojas; Sergio Gomez; Sheryll Corchuelo; Ángela Alarcon Cruz
EPI_ISL_1468452	Centro de Atendimento COVID	Instituto Adolfo Lutz, Interdiciplinary Procedures Center, Strategic Laboratory	Caio Vinicius Dias Lopes; Claudia Regina Gonçalves; Claudio Tavares Sacchi; Erica Valessa Ramos Gomes; Karoline Rodrigues Campos
EPI_ISL_483065, EPI_ISL_509430 to 509435, EPI_ISL_510536, EPI_ISL_529139	Centro de Desenvolvimento Tecnologico em Saude, Fundacao Oswaldo Cruz	Centro de Desenvolvimento Tecnologico em Saude, Fundacao Oswaldo Cruz	A.D.; Barroso; C.Q.; C.Q. and Medeiros; De Paula; F.B.; Ferreira; Fintelman-Rodrigues, N.; Gregorio; J.S.; M.A.; M.L.; Medeiros; Oliveira; S.P.; Sacramento; Saraiva; Souza; T.M.; Tschoeke, D.
EPI_ISL_510541, EPI_ISL_529140	Centro de Desenvolvimento Tecnologico em Saude, Fundacao Oswaldo Cruz	Centro de Desenvolvimento Tecnologico em Saude, Fundacao Oswaldo Cruz	A.D.; C.Q.; C.Q. and Medeiros; De Paula; F.B.; Ferreira; Fintelman-Rodrigues, N.; M.A.; Medeiros; Sacramento; Saraiva; Souza; T.M.
EPI_ISL_1531807 to 1531941	Centro de Diagnostico COVID-19 UABC Tijuana	Andersen lab at Scripps Research	German Ibarra; Jonathan Vincent Baena; Jorge Luis Jimenez Niebla; Manuel Sanchez Alavez; Oscar Efren Zazueta Fierro; SEARCH Alliance San Diego with Idanya Rubi Serafin Higuera
EPI_ISL_1469729, EPI_ISL_1469779	Centro de Especialidades Triunfo	Epiclin	Ana Paula Muterle; Carolina Comerlato; Eliana Márcia Da Ros Wendland; Fernando Hayashi Sant'Anna; Janira Prichula; Juliana Comerlato
EPI_ISL_1469108 to 1469119	Centro de Investigacion en Ciencias de la Salud y Biomedicina	CINVESTAV	Andreu Comas; Sofia Bernal
EPI_ISL_491933 to 491935, EPI_ISL_491941, EPI_ISL_527818 to 527819, EPI_ISL_594118	Centro de Investigaciones, Universidad de Especialidades Espíritu Santo	Institute of Microbiology, Universidad San Francisco de Quito	Belén Prado-Vivar; Bernardo Gutiérrez; Derly Andrade; Edith Lopez; Fernando Espinoza; Gabriel Morey; Gabriel Trueba; Jose Pedro Barberan; Juan Carlos Fernandez; Juan José Guadalupe; Michelle Grunauer; Monica Becerra-Wong; Patricio Rojas-Silva; Paúl Cárdenas; Ruben Armas; Sully Márquez; Verónica Barragán
EPI_ISL_468761 to 468764, EPI_ISL_500369 to 500372, EPI_ISL_500374 to 500381, EPI_ISL_500383, EPI_ISL_500385 to 500458, EPI_ISL_537382 to 537466, EPI_ISL_537811 to 537873, EPI_ISL_1534182 to 1534244			
see above	Centro de Investigación Biomédica de La Rioja - Hospital San Pedro Logroño	SeqCOVID-SPAIN consortium/IBV(CSIC)	José Manuel Azcona Gutiérrez; María Pilar Bea Escudero; María de Toro; Miriam Blasco Alberdi and SeqCOVID-SPAIN consortium
EPI_ISL_1494674, EPI_ISL_1494717	Centro de Investigación en Ciencias de la Salud y Biomedicina, U.A.S.L.P	Centro de Investigación en Ciencias de la Salud y Biomedicina, U.A.S.L.P	MD PhD Sofía Bernal Silva
EPI_ISL_1500471, EPI_ISL_1500515, EPI_ISL_1500639, EPI_ISL_1500866	Centro de Investigación en Ciencias de la Salud y Biomedicina, U.A.S.L.P.	Centro de Investigación en Ciencias de la Salud y Biomedicina, U.A.S.L.P.	MD PhD Sofía Bernal Silva
EPI_ISL_1494724 to 1494730	Centro de Investigación en Ciencias de la Salud y Biomedicina, U.A.S.L.P.	Centro de Investigación en Ciencias de la Salud y Biomedicina, U.A.S.L.P.	MD PhD Sofía Bernal Silva
EPI_ISL_1469603, EPI_ISL_1469667, EPI_ISL_1469741, EPI_ISL_1469749, EPI_ISL_1469777, EPI_ISL_1469806	Centro de Referência em Síndromes Gripais	Epiclin	Ana Paula Muterle; Carolina Comerlato; Eliana Márcia Da Ros Wendland; Fernando Hayashi Sant'Anna; Janira Prichula; Juliana Comerlato
EPI_ISL_583491	Centro de Saude Esf IV Zona Rual Domingos de SJ Rio Pardo	Instituto Adolfo Lutz, Interdiciplinary Procedures Center, Strategic Laboratory	Claudia Regina Gonçalves; Claudio Tavares Sacchi; Erica Valessa Ramos Gomes; Karoline Rodrigues Campos
EPI_ISL_471543, EPI_ISL_583500	Centro de Saude I Tacito Leite de Carvalho e Silva	Instituto Adolfo Lutz, Interdiciplinary Procedures Center, Strategic Laboratory	Claudia Regina Gonçalves; Claudio Tavares Sacchi; Erica Valessa Ramos Gomes; Karoline Rodrigues Campos
EPI_ISL_1493572, EPI_ISL_1493591	Centro de Saude II Dr Alcides Facundo Arroyo	Instituto Adolfo Lutz, Interdiciplinary Procedures Center, Strategic Laboratory	Caio Vinicius Dias Lopes; Claudia Regina Gonçalves; Claudio Tavares Sacchi; Erica Valessa Ramos Gomes; Karoline Rodrigues Campos
EPI_ISL_1520129 to 1520137	Centro de Saude II Dr Jose Paione Mococa	Instituto Adolfo Lutz, Interdiciplinary Procedures Center, Strategic Laboratory	Caio Vinicius Dias Lopes; Claudia Regina Gonçalves; Claudio Tavares Sacchi; Erica Valessa Ramos Gomes; Karoline Rodrigues Campos
EPI_ISL_1520117 to 1520128	Centro de Saude II Dr Jose de Felipe Espito Santo do Pinhal SP	Instituto Adolfo Lutz, Interdiciplinary Procedures Center, Strategic Laboratory	Caio Vinicius Dias Lopes; Claudia Regina Gonçalves; Claudio Tavares Sacchi; Erica Valessa Ramos Gomes; Karoline Rodrigues Campos
EPI_ISL_1533689 to 1533691	Centro de Saude II Dr. Jose Paione Mococa	Instituto Adolfo Lutz, Interdiciplinary Procedures Center, Strategic Laboratory	Caio Vinicius Dias Lopes; Claudia Regina Gonçalves; Claudio Tavares Sacchi; Erica Valessa Ramos Gomes; Karoline Rodrigues Campos; Leonardo Jose Tadeu de Araujo
EPI_ISL_1468411, EPI_ISL_1468426 to 1468427, EPI_ISL_1468429 to 1468430, EPI_ISL_1468463, EPI_ISL_1468472 to 1468473	Centro de Saude II Matao	Instituto Adolfo Lutz, Interdiciplinary Procedures Center, Strategic Laboratory	Caio Vinicius Dias Lopes; Claudia Regina Gonçalves; Claudio Tavares Sacchi; Erica Valessa Ramos Gomes; Karoline Rodrigues Campos

EPI_ISL_1469821	Centro de Saúde Bruno Cassel	Epiclin	Ana Paula Muterle; Carolina Comerlato; Eliana Márcia Da Ros Wendland; Fernando Hayashi Sant'Anna; Janira Prichula; Juliana Comerlato
EPI_ISL_1469557	Centro de Serviços Especializados Santa Rita	Epiclin	Ana Paula Muterle; Carolina Comerlato; Eliana Márcia Da Ros Wendland; Fernando Hayashi Sant'Anna; Janira Prichula; Juliana Comerlato
EPI_ISL_1396412 to 1396424, EPI_ISL_1396426 to 1396427, EPI_ISL_1396429 to 1396518	Centro de Tecnología en Salud Pública de la Universidad Nacional de Rosario	Laboratorio Mixto de Biotecnología Acuática (LMBA) on behalf of 'Proyecto Argentino Interinstitucional de genomica de SARS-CoV-2' (PAIS Consortium)	Adriana Giri; Agustina Cerri; Ana Cavatorta; Ana Paletta; Diego Chouhy; Elisa Bolatti; Elizabeth Tapia (argenTAG); Federico Remes Lenicov; Flavio Spetale; Gastón Viarengo; Ignacio García Labari; Javier Murillo; Joaquín Ezpeleta; Julian Acosta; Laura Angelone; Leandro Ciappina; María Re; Pablo Casal; Pilar Bulacio; Silvana Spinelli; Silvia Arranz; Sofía Lavista Llanos; Vanina Villanova; Victoria Posner
EPI_ISL_468305, EPI_ISL_468307	Centro de Vigilancia a Saude de Diadema	Instituto Adolfo Lutz, Interdiciplinary Procedures Center, Strategic Laboratory	Claudia Regina Gonçalves; Claudio Tavares Sacchi; Erica Valessa Ramos Gomes
EPI_ISL_1392889 to 1392891	Centrum Badawczo-Rozwojowe Invicta (Invicta sp. z o.o.)	Diagtron Laboratoria	Celina Cybulska; Karolina Gackowska; Krzysztof Lukaszczuk; Lukasz Rabalski; Maciej Kosinski; Natalia Derewonko
EPI_ISL_1393778 to 1393779, EPI_ISL_1393793, EPI_ISL_1393802 to 1393804, EPI_ISL_1538617 to 1538619, EPI_ISL_1538963	Centrum Medycyny Klinicznej dr n. med. Karol Majewski	1. National Institute of Public Health - National Institute of Hygiene; 2. Eurofins Genomics Europe Sequencing GmbH	ECDC COVID-19 WGS support team; Eurofins Genomics Europe Sequencing Team; Gierczyki Rafa; Sadkowska-Todys Magorzata; Wokowicz Tomasz; Zacharczuk Katarzyna
EPI_ISL_1394224 to 1394227, EPI_ISL_1394229 to 1394230	Centrum Medycyny Klinicznej dr n. med. Karol Majewski Di MEDICAL	1. National Institute of Public Health - National Institute of Hygiene; 2. Eurofins Genomics Europe Sequencing GmbH	ECDC COVID-19 WGS support team; Eurofins Genomics Europe Sequencing Team; Gierczyki Rafa; Sadkowska-Todys Magorzata; Wokowicz Tomasz; Zacharczuk Katarzyna
EPI_ISL_1394228	Centrum Medycyny Klinicznej dr n. med. Karol Majewski Di MEDICALi	1. National Institute of Public Health - National Institute of Hygiene; 2. Eurofins Genomics Europe Sequencing GmbH	ECDC COVID-19 WGS support team; Eurofins Genomics Europe Sequencing Team; Gierczyki Rafa; Sadkowska-Todys Magorzata; Wokowicz Tomasz; Zacharczuk Katarzyna
EPI_ISL_1538614	Centrum Medycyny Klinicznej dr n. med.Karol Majewski -DiMedical	1. National Institute of Public Health - National Institute of Hygiene; 2. Eurofins Genomics Europe Sequencing GmbH	ECDC COVID-19 WGS support team; Eurofins Genomics Europe Sequencing Team; Gierczyki Rafa; Sadkowska-Todys Magorzata; Wokowicz Tomasz; Zacharczuk Katarzyna
EPI_ISL_1393805 to 1393807, EPI_ISL_1394203 to 1394209, EPI_ISL_1394212 to 1394213, EPI_ISL_1394218 to 1394221, EPI_ISL_1538464, EPI_ISL_1538527, EPI_ISL_1538535, EPI_ISL_1538612 to 1538613, EPI_ISL_1538615 to 1538616, EPI_ISL_1538620 to 1538624, EPI_ISL_1538786 to 1538787, EPI_ISL_1538794 to 1538795, EPI_ISL_1538864, EPI_ISL_1538962, EPI_ISL_1538964, EPI_ISL_1539053, EPI_ISL_1539117 to 1539120, EPI_ISL_1539130 to 1539132, EPI_ISL_1539176, EPI_ISL_1539202			
see above	Centrum Medycyny Klinicznej dr n.med. Karol Majewski - DiMedical	1. National Institute of Public Health - National Institute of Hygiene; 2. Eurofins Genomics Europe Sequencing GmbH	ECDC COVID-19 WGS support team; Eurofins Genomics Europe Sequencing Team; Gierczyki Rafa; Sadkowska-Todys Magorzata; Wokowicz Tomasz; Zacharczuk Katarzyna
EPI_ISL_1393727 to 1393738, EPI_ISL_1394283 to 1394289, EPI_ISL_1425552 to 1425553, EPI_ISL_1538440	Centrum Medyczne MEDYK Sp. z o.o. ,Sp.k. Zakad Diagnostyki Medycznej	1. National Institute of Public Health - National Institute of Hygiene; 2. Eurofins Genomics Europe Sequencing GmbH	ECDC COVID-19 WGS support team; Eurofins Genomics Europe Sequencing Team; Gierczyki Rafa; Sadkowska-Todys Magorzata; Wokowicz Tomasz; Zacharczuk Katarzyna
EPI_ISL_450520 to 450524	Centrl Laboratorija	Latvian Biomedical Research and Study Centre	Ivars Silamielis; Jana Oste; Jnis Kloviš; Kaspars Megnis; Marta Priedte; Monta Ustinova; Stella Lapia; Uga Dumpis; Vita Rovte; ikita Zrelovs
EPI_ISL_486390 to 486391, EPI_ISL_486411 to 486416, EPI_ISL_486418 to 486421, EPI_ISL_486437, EPI_ISL_492988 to 492992, EPI_ISL_501286 to 501289, EPI_ISL_501808, EPI_ISL_501817, EPI_ISL_501895 to 501896, EPI_ISL_501915, EPI_ISL_501922, EPI_ISL_515196, EPI_ISL_534200 to 534202, EPI_ISL_534206 to 534208, EPI_ISL_534210 to 534211, EPI_ISL_534220, EPI_ISL_534222 to 534223			
see above	Centrl laboratorija	Latvian Biomedical Research and Study Centre	Ivars Silamielis; Jana Oste; Jnis Kloviš; Jnis Pjalkovskis; Kaspars Megnis; Marta Priedte; Monta Ustinova; Stella Lapia; Uga Dumpis; Vita Rovte; ikita Zrelovs
EPI_ISL_1404144	Cerballiance IDFEst	Cerba Lab	Benazra M; Haim-Boukobza S; Lecorche E; Olivi M; Roquebert B; Trombert-Paolantoni S
EPI_ISL_1547506	Cerballiance Pont Audemer	Cerba Lab	Haïm-Boukobza S; Lecorche E; Leriche A; Prigent A; Roquebert B; Trombert-Paolantoni S; Verdurme L
EPI_ISL_1403780	Cerballiance site les Clayes	Cerba Lab	Benazra M; Haim-Boukobza S; Lecorche E; Olivi M; Roquebert B; Trombert-Paolantoni S
EPI_ISL_1404374	Cerballliance Les Clayes	Cerba Lab	Benazra M; Haim-Boukobza S; Lecorche E; Olivi M; Trombert-Paolantoni S; Zimmer S
EPI_ISL_516629 to 516645	Charite Universitatsmedizin Berlin, Institut fur Virologie/Labor Berlin	Charite Universitatsmedizin Berlin, Institut fur Virologie/Labor Berlin	Barbara Muhlemann; Christian Drosten; Julia Schneider; Jörn Beheim-Schwarzbach; Talitha Veith; Terry Jones; Victor M Corman
EPI_ISL_406862	Charité Universitätsmedizin Berlin, Institute of Virology; Institut für Mikrobiologie der Bundeswehr, Munich	Charité Universitätsmedizin Berlin, Institute of Virology	Barbara Mühlemann; Christian Drosten; Julia Schneider; Markus Antwerpen; Roman Wölfel; Talitha Veith; Victor M Corman
EPI_ISL_1435333 to 1435340	Chemische und Veterinäruntersuchungsamt Münsterland-Emscher-Lippe	Robert Koch Institute	
EPI_ISL_569612 to 569613	Cheyenne River Health Center	South Dakota Public Health Laboratory	Jacob Garfin; Matt Plumb; Xiong Wang; and Chris Carlson
EPI_ISL_419297 to 419298, EPI_ISL_419309 to 419311, EPI_ISL_480004 to 480014, EPI_ISL_1426653 to 1426658, EPI_ISL_1427071 to 1427104, EPI_ISL_1427717 to 1427718, EPI_ISL_1428288 to 1428313, EPI_ISL_1431615, EPI_ISL_1431642 to 1431708, EPI_ISL_1431726, EPI_ISL_1431749 to 1431764, EPI_ISL_1431824 to 1431828, EPI_ISL_1432028, EPI_ISL_1432030, EPI_ISL_1432032, EPI_ISL_1432034 to 1432035, EPI_ISL_1432037, EPI_ISL_1432039, EPI_ISL_1432041, EPI_ISL_1432043, EPI_ISL_1432045, EPI_ISL_1432047, EPI_ISL_1432049, EPI_ISL_1432051, EPI_ISL_1432054, EPI_ISL_1432056, EPI_ISL_1432058, EPI_ISL_1432060, EPI_ISL_1432063 to 1432064, EPI_ISL_1432067, EPI_ISL_1432069, EPI_ISL_1432071, EPI_ISL_1432073, EPI_ISL_1432076 to 1432077, EPI_ISL_1432079, EPI_ISL_1432081, EPI_ISL_1432083			
see above	Chiba Prefectural Institute of Public Health	Pathogen Genomics Center, National Institute of Infectious Diseases	Hajime Kamiya; Kentaro Itokawa; Makoto Kuroda; Masakatsu Taira; Masanori Hashino; Motoi Suzuki; Rina Tanaka; Tsuyoshi Sekizuka; Yushi Hachisu
EPI_ISL_468070 to 468078, EPI_ISL_477125 to 477140, EPI_ISL_492028 to 492031, EPI_ISL_1469964 to 1469986	Child Health Research Foundation	Child Health Research Foundation	Afroza Akter Tanni; CHRF Bangladesh Genomics Team; Hafizur Rahman; Maksuda Islam; Md Hafizur Rahman; Md Saiful Islam Sajib; Roly Malaker; Samir K Saha; Senjuti Saha; Syed Muktadir Al Sium
EPI_ISL_437912	Child Health Research Foundation	Child Health Research Lab	Maksuda Islam; Md Hafizur Rahman; Md Hasanuzzaman; Md Saiful Islam Sajib; Md Shahidul Islam; Roly Malaker; Samir K Saha; Senjuti Saha; Zabed B Ahmed
EPI_ISL_513343 to 513344	Children Westmead Hospital	NSW Health Pathology - Institute of Clinical Pathology and Medical Research; Westmead Hospital; University of Sydney	CIDM-PH et al.
EPI_ISL_507962	Children's Hospitals and Clinics of Minnesota	Minnesota Department of Health, Public Health Laboratory	Jacob Garfin; Matt Plumb; and Xiong Wang
EPI_ISL_451548, EPI_ISL_451594, EPI_ISL_451605, EPI_ISL_455068	Childrens Hospital Westmead	NSW Health Pathology - Institute of Clinical Pathology and Medical Research; Westmead Hospital; University of Sydney	CIDM-PH et al.
EPI_ISL_430722 to 430746	Chinese PLA Institute for Disease Control and Prevention	Chinese PLA Institute for Disease Control and Prevention	Lizhong Li; Peng LiJinhui Li
EPI_ISL_417316, EPI_ISL_417330 to 417332, EPI_ISL_429877 to 429878	Chiu Laboratory, University of California, San Francisco	Chiu Laboratory, University of California, San Francisco	Scot Federman; Wei Gu; Xianding Deng; and Charles Y. Chiu
EPI_ISL_443315	Château de la Source	National Reference Center for Viruses of Respiratory Infections, Institut Pasteur, Paris	Angela Brisebarre; Etienne Simon-Lorière; Flora Donati; Marion Barbet; Maud Vanpeene; Mélanie Albert; Méline Bizard; Sylvie Behillil; Sylvie van der Werf; Vincent Enouf
EPI_ISL_1398367, EPI_ISL_1398565, EPI_ISL_1398932, EPI_ISL_1398939, EPI_ISL_1398942, EPI_ISL_1398955, EPI_ISL_1398983			
see above	Cianjur Public Health	West Java Health Laboratory; School of Life Sciences and Technology, Institut Teknologi Bandung	Azzania Fibriani; Cut Nur Cinthia Alamanda; Ema Rahmawati; Isak Solihin; Kamila Tania; Karimatu Khoirunnisa; Miftahul Faridl; Rifky Waluyajati Rachman; Rini Robiani; Ryan Bayusantika Ristandi
EPI_ISL_491115	Cicin-Sain Lab	Cicin-Sain Lab	Kathrin Eschke; Luka Cicin-Sain; M. Zeeshan Chaudhry; Yeonsu Kim
EPI_ISL_1534697 to 1534700	Ciputra Hospital Citra Garden City	National Institute of Health Research and Development	Arie Ardiansyah Nugraha; Hana Apsari Pawestri; Hartanti Dian Ikawati; Kartika Dewi Puspa; Krisna Pangesti; Nelly Puspandari; Subangkit; Vivi Setiawaty
EPI_ISL_454646	City of El Paso Department of Public Health Laboratory	Pathogen Discovery, Respiratory Viruses Branch, Division of Viral Diseases, Centers for Disease Control and Prevention	Anna Uehara; Clinton R. Paden; Haibin Wang; Jing Zhang; Krista Queen; Michael Bowen; Suxiang Tong; Yan Li; Ying Tao; Zachary Weiner

EPI_ISL_1493367 to 1493388, EPI_ISL_1494731 to 1494756	City of Milwaukee Health Department Laboratory	City of Milwaukee Health Department Laboratory	Sanjib Bhattacharyya
EPI_ISL_547585 to 547593, EPI_ISL_560318, EPI_ISL_560322 to 560324, EPI_ISL_561337 to 561343	Civil Hospital, Panchkula	CSIR-Institute of Microbial Technology	Amandeep Kaur; Anu Singh; Ashwani Kumar; Debarghya Ghose; Dipak Dutta; Harsh Goar; Kanika Bansal; Navin Baid; Poushali Chakraborty; Prabhu B. Patil; Rajesh Kumar Mishra; Sanjeet Kumar; Sanjeev Khosla
EPI_ISL_539483 to 539490, EPI_ISL_561344	Civil Hospital, Rupnagar	CSIR-Institute of Microbial Technology	Amandeep Kaur; Anu Singh; Ashwani Kumar; Debarghya Ghose; Dipak Dutta; Harsh Goar; Kanika Bansal; Navin Baid; Poushali Chakraborty; Prabhu B. Patil; Rajesh Kumar Mishra; Sanjeet Kumar; Sanjeev Khosla
EPI_ISL_1424882 to 1425075, EPI_ISL_1435646, EPI_ISL_1435649, EPI_ISL_1435651, EPI_ISL_1435653, EPI_ISL_1435655, EPI_ISL_1435657, EPI_ISL_1435660, EPI_ISL_1435662			
see above	Clalit Health Services Laboratories, Israel	Stern Lab	Stern Lab
EPI_ISL_1510648 to 1510650	Clincal Reference Laboratory	Kansas Health and Environmental Lab	Ben Olsen; Jonathan Barnell; Mike Grose; and Phil Adam
EPI_ISL_1534264	Clinic-in-Asla	NHLS/UCT	Arash Iranzadeh; Bruna Galvao; Carolyn Williamson; Deelan Doolabh; Diana Hardie; Innocent Mudau; Kruger Marais; Lynn Tyers; Marvin Hsiao; Stephen Korsman
EPI_ISL_414579	Clinica Alemana de Santiago, Chile	Instituto de Salud Publica de Chile	Alejandra Acevedo; Andrés E. Castillo; Bárbara Parra; Carolina Tambley; Gabriel Leal; Gisselle Barra; Jaime Lagos; Javier Tognarelli; Jorge Fernández.; Loredana Arata; Patricia Bustos; Paz Tapia; Rodrigo Fasce; Soledad Ulloa; Winston Andrade
EPI_ISL_527759	Clinica Biblica	Inciensa, Instituto Costarricense de Investigación y Enseñanza en Nutrición y Salud	Adriana Godinez & Melany Calderon; Claudio Soto-Garita; Estela Cordero; Francisco Duarte; Hebleen Porras
EPI_ISL_414580	Clinica Santa Maria, Santiago, Chile	Instituto de Salud Publica de Chile	Alejandra Acevedo; Andrés E. Castillo; Bárbara Parra; Carolina Tambley; Gabriel Leal; Gisselle Barra; Jaime Lagos; Javier Tognarelli; Jorge Fernández.; Loredana Arata; Patricia Bustos; Paz Tapia; Rodrigo Fasce; Soledad Ulloa; Winston Andrade
EPI_ISL_1494958	Clinica Universitaria Medicina Integral - Laboratorio Molecular	Instituto Nacional de Salud- Dirección de Investigación en Salud Pública	Carlos Franco-Muñoz; Carmen Osorio; Diana Malo; Diego A. Álvarez-Díaz; Diego Andrés Prada; Gerardo Santamaría; Hector Alejandro Ruiz-Moreno; Jhonnatan Reales-González; Juan Camilo Martinez; Julian Naizaque; Katherine Laiton-Donato; Lisseth Pardo; Magdalena Wiesner; Marcela Mercado-Reyes; Maria T. Herrera-Sepúlveda; Marta Lopez Blanco; Martha Lucia Ospina Martinez; Paola Rojas; Sergio Gomez; Sheryll Corchuelo; Ángela Alarcon Cruz
EPI_ISL_462450 to 462476	Clinical Center, University of Sarajevo	Charite Universitatsmedizin Berlin, Institute of Virology	Almedina Hadzihasanovic-Moro; Amela Dedeic-Ljubovic; Barbara Muehlemann; Christian Drosten; Irma Salimovic-Besic; Jorn Beheim-Schwarzbach; Julia Schneider; Selma Mutevelic; Suzana Arapcic; Talitha Veith; Terry Jones; Victor M Corman
EPI_ISL_421279, EPI_ISL_421281, EPI_ISL_437455 to 437458	Clinical Diagnostics Laboratory, Diagnostic & Experimental Pathology, Lilly Research Laboratories	Clinical Diagnostics Laboratory, Diagnostic & Experimental Pathology, Lilly Research Laboratories	Andrew Schade; Angie Fulford; Erin Wray; Jeff Fill; Joe Oakley; John Calley; John McElwee; Leslie ONeill Reising; Leslie O'Neill Reising; Mayuri Vaidya; Pat Finnegan; Phil Ebert; Rachael Redmond; Sam McNeely; Tim Holzer
EPI_ISL_516426 to 516431	Clinical Hospital - Shtip	Research Center for Genetic Engineering and Biotechnology "Georgi D. Efremov" , Macedonian Academy of Sciences and Arts	RCGEB - MASA
EPI_ISL_1418324	Clinical Infectious Diseases Hospital Constana	National Institute of Infectious Diseases-Prof. Dr. Matei Bals Molecular Diagnostics Laboratory	Corina Casangiu; Dan Otelea; Ionelia Nicolae; Leontina Banica; Marius Surleac; Ovidiu Vlaicu; Simona Paraschiv
EPI_ISL_424352	Clinical Laboratory, Fuyang City Center for Disease Control and Prevention	Clinical Laboratory, Fuyang City Center for Disease Control and Prevention	Ge, B.
EPI_ISL_450506	Clinical Laboratory, Hospital Israelita Albert Einstein	Clinical Laboratory, Hospital Israelita Albert Einstein	Amgarten, D.; Araujo; C.L.P.; D.B.; D.B.L.; Durigon; E.L. and Pinho; J.R.R.; Machado; Malta, F.; Mangueira; R.A.F.; R.R.G.; Santana; de Oliveira
EPI_ISL_416432	Clinical Microbiology Lab	Infectious Disease Research Department, King Abdullah International Medical Research Center (KAIMRC)	Abdulrahman Alswaji; Liliane Okdah; Majed Alghoribi; Michel Doumith; Sadeem Alhayli; Sameera Al Johani
EPI_ISL_447419 to 447469	Clinical Microbiology Laboratory, Sheba Medical Center	Stern Lab	Stern Lab
EPI_ISL_447383 to 447406, EPI_ISL_447417 to 447418	Clinical Microbiology Laboratory, The Baruch Padeh Medical Center, Poriya	Stern Lab	Stern Lab
EPI_ISL_483570	Clinical Microbiology Laboratory- Basurto University Hospita	Biocruces-Bizkaia	Ana Belén Belén de la Hoz; Estibaliz Ugalde-Zarraga; José Luis Díaz de Tuesta del Arco; Matxalen Vidal-García; Mikel J. Urrutikoetxea-Gutierrez; Mº Carmen Nieto Toboso
EPI_ISL_483566, EPI_ISL_483571 to 483573, EPI_ISL_486876, EPI_ISL_489833 to 489835, EPI_ISL_490202 to 490204, EPI_ISL_490977	Clinical Microbiology Laboratory- Basurto University Hospital	Biocruces-Bizkaia	Ana Belén Belén de la Hoz; Estibaliz Ugalde-Zarraga; José Luis Díaz de Tuesta del Arco; Matxalen Vidal-García; Mikel J. Urrutikoetxea-Gutierrez; Mº Carmen Nieto Toboso
EPI_ISL_1385842 to 1385953, EPI_ISL_1468475 to 1468531, EPI_ISL_1534017 to 1534018, EPI_ISL_1534020 to 1534033, EPI_ISL_1534035 to 1534070, EPI_ISL_1534072 to 1534073, EPI_ISL_1534076 to 1534081, EPI_ISL_1534083 to 1534095, EPI_ISL_1534097 to 1534122, EPI_ISL_1534124 to 1534133, EPI_ISL_1534136 to 1534159			
see above	Clinical Microbiology, Infection Prevention and Control	Section for Molecular Diagnostics	Björn Hallström; Jonas Björkman
EPI_ISL_1443293 to 1443336	Clinical Molecular Microbiology Laboratory, UNC Hospital	Dirk Dittmer	Angelica Juarez; Blossom Damania.; Brent A. Eason; Cameroon Grant; Carolina Caro-Vegas; Cecilia Thompson; Dirk Dittmer; Evelyn Hoffman; Jason Wong; Justin T. Landis; Linda Pluta; Melissa B. Miller; Patricio Cano; Razia Moorad; Ryan McNamara; Shawn Hawken
EPI_ISL_1418311 to 1418312	Clinical Pneumology Hospital Iai	National Institute of Infectious Diseases-Prof. Dr. Matei Bals Molecular Diagnostics Laboratory	Corina Casangiu; Dan Otelea; Ionelia Nicolae; Leontina Banica; Marius Surleac; Ovidiu Vlaicu; Simona Paraschiv
EPI_ISL_1396634 to 1396646, EPI_ISL_1503231	Clinical Reference Laboratory	Kansas Health and Environmental Lab	Ben Olsen; Jonathan Barnell; Mike Grose; and Phil Adam
EPI_ISL_578697, EPI_ISL_605799 to 605816	Clinical Virology Laboratory, Institute of Liver and Biliary Sciences	ILBS - IGIB	Abhishek Padhi; Ekta Gupta; Jaswinder Singh Maras; Reshu Agarwal; Sheetalnath Rooge; Shridhar Sivasubbu; Shvetank Sharma; Vinod Scaria
EPI_ISL_447311 to 447330	Clinical Virology Laboratory, Soroka Medical Center and the Faculty of Health Sciences, Ben-Gurion University of the Negev	Stern Lab	Stern Lab
EPI_ISL_447331 to 447382, EPI_ISL_447407 to 447416	Clinical Virology Unit, Hadassah Hebrew University Medical Center	Stern Lab	Stern Lab
EPI_ISL_418230	Clinique AVERAY LA BROUSTE, Med. Polyvalente	National Reference Center for Viruses of Respiratory Infections, Institut Pasteur, Paris	Angela Brisebarre; Elsa Ngwem; Etienne Simon-Lorière; Flora Donati; Marion Barbet; Maud Vanpeene; Mélanie Albert; Méline Bizard; Sylvie Behillil; Sylvie van der Werf; Vincent Enouf
EPI_ISL_1527016, EPI_ISL_1527022	Clínica Clorito Picado	Inciensa, Instituto Costarricense de Investigación y Enseñanza en Nutrición y Salud	Barboza-Arguedas E & Salas-Abarca P; Pérez-Corrales C
EPI_ISL_1494957	Clínica Primavera	Instituto Nacional de Salud- Dirección de Investigación en Salud Pública	Carlos Franco-Muñoz; Carmen Osorio; Diana Malo; Diego A. Álvarez-Díaz; Diego Andrés Prada; Gerardo Santamaría; Hector Alejandro Ruiz-Moreno; Jhonnatan Reales-González; Juan Camilo Martinez; Julian Naizaque; Katherine Laiton-Donato; Lisseth Pardo; Magdalena Wiesner; Marcela Mercado-Reyes; Maria T. Herrera-Sepúlveda; Marta Lopez Blanco; Martha Lucia Ospina Martinez; Paola Rojas; Sergio Gomez; Sheryll Corchuelo; Ángela Alarcon Cruz
EPI_ISL_452472 to 452543, EPI_ISL_538082 to 538117, EPI_ISL_538608 to 538612	Clínica Universidad de Navarra. Servicio de Enfermedades Infecciosas y Microbiología clínica	SeqCOVID-SPAIN consortium/IBV(CSIC)	Jose Luis del Pozo and SeqCOVID-SPAIN consortium; Mirian Fernández-Alonso
EPI_ISL_1534273 to 1534274, EPI_ISL_1534298, EPI_ISL_1534340,	CoVid WC Garden Route	NHLS/UCT	Arash Iranzadeh; Bruna Galvao; Carolyn Williamson; Deelan Doolabh; Diana Hardie; Emmanuel SJ; Innocent Mudau; Kruger Marais; Lynn Tyers; Marvin Hsiao; Stephen Korsman; Tegally H; de Oliveira T

EPI_ISL_1534355, EPI_ISL_1534395			
EPI_ISL_479662, EPI_ISL_591070	College of Veterinary Medicine, Chungnam National University	College of Veterinary Medicine, Chungnam National University	S.H. and Jang, Y.; Seo; Seo, S.
EPI_ISL_1539238 to 1540417	Colorado Department of Public Health and Environment	Colorado Department of Public Health and Environment	Diana Ir; Emily A. Travanty; Laura Bankers; Molly C. Hetherington-Rauth; Sarah Elizabeth Totten; Shannon Ely; Shannon R. Matzinger
EPI_ISL_527401 to 527488	Colorado State University - Ebel Lab	Colorado State University - Ebel Lab	Greg Ebel et al.
EPI_ISL_1395726 to 1395749, EPI_ISL_1397636 to 1397649, EPI_ISL_1397824 to 1397828, EPI_ISL_1397886 to 1397906, EPI_ISL_1398044 to 1398047, EPI_ISL_1398144 to 1398146, EPI_ISL_1398163 to 1398203, EPI_ISL_1465633, EPI_ISL_1465693 to 1465695, EPI_ISL_1465803 to 1465804, EPI_ISL_1482277, EPI_ISL_1482410			
see above	Columbia University Irving Medical Center	Wadsworth Center, New York State Department of Health	Alexis Russel; Alexis Russell; Catharine Prussing; Daryl M. Lamson; Erasmus Schneider; Erica Lasek-Nesselquist; John Kelly; Jonathan Plitnick; Kirsten St. George; Matthew Shudt; Melissa A Leisner; Navjot Singh
EPI_ISL_1494952 to 1494953	Comfandi	Instituto Nacional de Salud- Dirección de Investigación en Salud Pública	Carlos Franco-Muñoz; Carmen Osorio; Diana Malo; Diego A. Álvarez-Díaz; Diego Andrés Prada; Gerardo Santamaría; Hector Alejandro Ruiz-Moreno; Jhonnatan Reales-González; Juan Camilo Martinez; Julian Naizaque; Katherine Laiton-Donato; Lisseth Pardo; Magdalena Wiesner; Marcela Mercado-Reyes; Maria T. Herrera-Sepúlveda; Marta Lopez Blanco; Martha Lucia Ospina Martinez; Paola Rojas; Sergio Gomez; Sheryll Corchuelo; Ángela Alarcon Cruz
EPI_ISL_1516188 to 1516196	Commonwealth Healthcare Center	Centers for Disease Control and Prevention Division of Viral Diseases, Pathogen Discovery	Alison Laufer Halpin; Ben L. Rambo-Martin; Clinton R. Paden; Dakota Howard; Darlene Wagner; Dave Wentworth; Dhwani Batra; Jasmine Padilla; Justin Lee; Katie Dillon; Krista Queen; Kristen Knipe; Kristine Lacek; Mark Burroughs; Matthew Schmerer; Mili Sheth; Peter Cook; Sam Shepard; Sarah Nobles; Shoshona Le; Suxiang Tong; Vivien Dugan; Yvette Unoarumhi
EPI_ISL_539822 to 539823	Communicable Disease Branch	Hong Kong Department of Health	Alan K.L. Tsang; Dominic N.C. Tsang; Edman T.K. Lam; Peter C.W. Yip; Rickjason C.W. Chan
EPI_ISL_485401, EPI_ISL_510528, EPI_ISL_510531	Communicable Disease Laboratory, Public Health Directorate	Communicable Disease Laboratory, Public Health Directorate	Al Wasti; Al-Taif; Al-Wasti, H.; AlWasti, H.; Altaif, Z.; H. and AlTaif, Z.; Shehab, F.; Z. and Shehab, F.; Zaed, A.
EPI_ISL_455328 to 455331, EPI_ISL_539563 to 539564, EPI_ISL_578194	Complejo Hospitalario Universitario La Coruna	Instituto de Salud Carlos III	A. Monzón; F. Casas; I; I. Jiménez; Iglesias-Caballero; J. López; M. Camarero; M. Cuesta; M. González-Esguevillas; M. Molinero Calamita; M. Zaballos; M.A Canizares; M.I Paz; P. Jiménez; S. Juliá; S. Pozo; S. Varona
EPI_ISL_474798 to 474831, EPI_ISL_474837 to 474840, EPI_ISL_474847 to 474849, EPI_ISL_474853, EPI_ISL_474901 to 474906, EPI_ISL_474919, EPI_ISL_474921, EPI_ISL_474933, EPI_ISL_474940 to 474941, EPI_ISL_474945 to 474947, EPI_ISL_474951 to 474956, EPI_ISL_500157 to 500159, EPI_ISL_500166, EPI_ISL_500207, EPI_ISL_500218			
see above	Complejo Hospitalario Universitario de Albacete	SeqCOVID-SPAIN consortium/IBV(CSIC)	Caridad Sainz de Baranda Camino and SeqCOVID-SPAIN consortium; Encarnacion Simarro Córdoba; Julia Lozano Serra; Lorena Robles Fonseca; Monica Parra Grandes
EPI_ISL_1478755 to 1478756	Complejo Hospitalario Universitario de Pontevedra	Microbiology Department. Complexo Hospitalario Universitario de Vigo	Campello M; Perez S; Regueiro B; Trigo M
EPI_ISL_455333	Complejo Hospitalario Universitario de Santiago	Instituto de Salud Carlos III	A. Monzón; F. Casas; I; I. Jiménez; Iglesias-Caballero; J. Llovo; M. Camarero; M. Cuesta; M. González-Esguevillas; M. Molinero Calamita; M. Zaballos; P. Jiménez; S. Juliá; S. Pozo; S. Varona
EPI_ISL_537380 to 537381	Complejo Hospitalario Universitario de Vigo	SeqCOVID-SPAIN consortium/IBV(CSIC)	Benito Regueiro and SeqCOVID-SPAIN consortium
EPI_ISL_539569 to 539572	Complejo Hospitalario de Navarra	Instituto de Salud Carlos III	A. Monzón; F. Casas; I; I. Jiménez; Iglesias-Caballero; J. López; M. Camarero; M. Cuesta; M. González-Esguevillas; M. Molinero Calamita; M. Zaballos; P. Jiménez; S. Juliá; S. Pozo; S. Varona
EPI_ISL_455334 to 455335, EPI_ISL_578195	Complejo Hospitalario de Orense	Instituto de Salud Carlos III	A. Monzón; F. Casas; I; I. Jiménez; Iglesias-Caballero; M. Camarero; M. Cuesta; M. García; M. González-Esguevillas; M. Molinero Calamita; M. Paz; M. Zaballos; P. Jiménez; S. Juliá; S. Pozo; S. Varona
EPI_ISL_583497	Complexo Hospitalar Ouro Verde de Campinas	Instituto Adolfo Lutz, Interdiciplinary Procedures Center, Strategic Laboratory	Claudia Regina Gonçalves; Claudio Tavares Sacchi; Erica Valessa Ramos Gomes; Karoline Rodrigues Campos
EPI_ISL_523970	Conjunto Hospitalar do Mandaqui	Instituto Adolfo Lutz, Interdiciplinary Procedures Center, Strategic Laboratory	Claudia Regina Gonçalves; Claudio Tavares Sacchi; Erica Valessa Ramos Gomes
EPI_ISL_416416 to 416419, EPI_ISL_435710 to 435719, EPI_ISL_527738, EPI_ISL_527761 to 527785	Connecticut State Department of Public Health	Grubaugh Lab - Yale School of Public Health	Adam Moore; Akiko Iwasaki; Albert Ko; Alice Lu; Allison Nelson; Anderson Brito; Anne Wyllie; Anthony Muyombwe; Arnau Casanovas; Catherine Muenker; Chaney Kalinich; Chantal Vogels; Charlese Dela Cruz; Cole Jensen; Ellen Foxman; Isabel Ott; Jafar Razeq; Joseph Fauver; Maria Tokuyama; Marie-Louise Landry; Mary Petrone; Nagarjuna Cheemarla; Nathan Grubaugh; Patrick Wong; Peiwen Lu; Richard Martinello; Saad Omer; Shelli Farhadian; Tara Alpert
EPI_ISL_536222, EPI_ISL_536288	Conseil Cri de la SSS de la Baie-James	Laboratoire de santé publique du Québec	Guillaume Bourque; Ioannis Ragoussis; Jesse Shapiro; Mark Lathrop and Michel Roger on behalf of the CoVSeQ research group; Sandrine Moreira
EPI_ISL_455327, EPI_ISL_539526 to 539530	Consejeria de Sanidad y Asuntos Sociales	Instituto de Salud Carlos III	A. Monzón; F. Casas; G. Gutiérrez; I; I. Jiménez; Iglesias-Caballero; M. Camarero; M. Cuesta; M. González-Esguevillas; M. Molinero Calamita; M. Zaballos; P. Jiménez; S. Juliá; S. Pozo; S. Varona
EPI_ISL_1477675 to 1477676, EPI_ISL_1477679, EPI_ISL_1477685, EPI_ISL_1477689, EPI_ISL_1477697 to 1477698, EPI_ISL_1477704, EPI_ISL_1477713 to 1477714, EPI_ISL_1477716, EPI_ISL_1477724, EPI_ISL_1477732, EPI_ISL_1477737 to 1477739, EPI_ISL_1477743 to 1477744, EPI_ISL_1477746, EPI_ISL_1477748, EPI_ISL_1477760 to 1477761, EPI_ISL_1477764 to 1477765, EPI_ISL_1477770, EPI_ISL_1477774, EPI_ISL_1477781 to 1477782, EPI_ISL_1477785, EPI_ISL_1477788 to 1477789, EPI_ISL_1477794 to 1477795, EPI_ISL_1477802, EPI_ISL_1477806, EPI_ISL_1477810, EPI_ISL_1477813, EPI_ISL_1477815 to 1477816, EPI_ISL_1477821, EPI_ISL_1477826 to 1477827, EPI_ISL_1477829, EPI_ISL_1477833 to 1477835, EPI_ISL_1477843 to 1477844, EPI_ISL_1477849, EPI_ISL_1477851, EPI_ISL_1477861, EPI_ISL_1477864, EPI_ISL_1477866 to 1477867, EPI_ISL_1477869 to 1477870, EPI_ISL_1477872, EPI_ISL_1477876, EPI_ISL_1477894, EPI_ISL_1477898, EPI_ISL_1477910 to 1477911, EPI_ISL_1477916, EPI_ISL_1477918, EPI_ISL_1477927, EPI_ISL_1477930 to 1477931, EPI_ISL_1477939, EPI_ISL_1477944, EPI_ISL_1477946 to 1477947, EPI_ISL_1477950, EPI_ISL_1477952 to 1477953, EPI_ISL_1477956 to 1477957, EPI_ISL_1477960, EPI_ISL_1477962, EPI_ISL_1477966, EPI_ISL_1477972, EPI_ISL_1477977, EPI_ISL_1477980, EPI_ISL_1477990, EPI_ISL_1477993, EPI_ISL_1477995, EPI_ISL_1477998 to 1478000, EPI_ISL_1478002 to 1478003, EPI_ISL_1478005, EPI_ISL_1478007 to 1478008, EPI_ISL_1478011, EPI_ISL_1478017, EPI_ISL_1478020, EPI_ISL_1478022 to 1478023, EPI_ISL_1478032, EPI_ISL_1478034 to 1478037, EPI_ISL_1478039 to 1478040, EPI_ISL_1478043, EPI_ISL_1478047, EPI_ISL_1478049, EPI_ISL_1478052 to 1478053, EPI_ISL_1478057, EPI_ISL_1478069, EPI_ISL_1478072, EPI_ISL_1478078, EPI_ISL_1478086, EPI_ISL_1478088, EPI_ISL_1478090 to 1478091, EPI_ISL_1478098 to 1478099, EPI_ISL_1478101, EPI_ISL_1478108, EPI_ISL_1478111, EPI_ISL_1478117, EPI_ISL_1478120, EPI_ISL_1478122, EPI_ISL_1478125 to 1478126, EPI_ISL_1478128, EPI_ISL_1478131, EPI_ISL_1478133, EPI_ISL_1478139, EPI_ISL_1478141, EPI_ISL_1478145, EPI_ISL_1478147, EPI_ISL_1478154 to 1478155, EPI_ISL_1478157, EPI_ISL_1478160, EPI_ISL_1478180 to 1478182, EPI_ISL_1478187 to 1478189, EPI_ISL_1478193, EPI_ISL_1478196 to 1478197, EPI_ISL_1478199, EPI_ISL_1529103, EPI_ISL_1529122 to 1529124, EPI_ISL_1529131, EPI_ISL_1529133 to 1529135, EPI_ISL_1529144, EPI_ISL_1529149, EPI_ISL_1529155, EPI_ISL_1529163 to 1529166, EPI_ISL_1529171, EPI_ISL_1529176 to 1529177, EPI_ISL_1529179, EPI_ISL_1529181, EPI_ISL_1529185 to 1529186, EPI_ISL_1529188, EPI_ISL_1529190, EPI_ISL_1529194 to 1529196, EPI_ISL_1529202, EPI_ISL_1529204, EPI_ISL_1529209 to 1529212, EPI_ISL_1529216 to 1529217, EPI_ISL_1529227 to 1529229, EPI_ISL_1529235 to 1529236, EPI_ISL_1529239, EPI_ISL_1529247, EPI_ISL_1529250 to 1529251, EPI_ISL_1529253, EPI_ISL_1529261, EPI_ISL_1529269, EPI_ISL_1529271 to 1529272, EPI_ISL_1529275, EPI_ISL_1529282, EPI_ISL_1529296 to 1529298, EPI_ISL_1529300 to 1529302, EPI_ISL_1529304 to 1529305, EPI_ISL_1529308, EPI_ISL_1529310, EPI_ISL_1529318, EPI_ISL_1529320, EPI_ISL_1529322 to 1529323, EPI_ISL_1529327 to 1529328, EPI_ISL_1529330 to 1529331, EPI_ISL_1529334, EPI_ISL_1529340, EPI_ISL_1529345 to 1529346, EPI_ISL_1529348, EPI_ISL_1529350, EPI_ISL_1529352 to 1529355, EPI_ISL_1529358, EPI_ISL_1529364 to 1529365, EPI_ISL_1529367 to 1529368			
see above	Contra Costa County Public Health Lab	Chan-Zuckerberg Biohub	CZB Cliahub Consortium
EPI_ISL_468615 to 468655, EPI_ISL_548491 to 548492, EPI_ISL_548495, EPI_ISL_548499, EPI_ISL_548501, EPI_ISL_548506 to 548508, EPI_ISL_548510, EPI_ISL_548515 to 548516, EPI_ISL_548520, EPI_ISL_548524, EPI_ISL_548527 to 548528, EPI_ISL_548531, EPI_ISL_548534 to 548535, EPI_ISL_548537 to 548540, EPI_ISL_548545, EPI_ISL_548548 to 548549, EPI_ISL_548552, EPI_ISL_548554, EPI_ISL_548556 to 548558, EPI_ISL_548561, EPI_ISL_548564 to 548567, EPI_ISL_548569, EPI_ISL_548572 to 548575, EPI_ISL_548577, EPI_ISL_548579			
see above	Contra Costa Public Health Lab	Chan-Zuckerberg Biohub	CZB Cliahub Consortium
EPI_ISL_1534334, EPI_ISL_1534399	Conville CDC wc CVC	NHLS/UCT	Arash Iranzadeh; Bruna Galvao; Carolyn Williamson; Deelan Doolabh; Diana Hardie; Emmanuel SJ; Innocent Mudau; Kruger Marais; Lynn Tyers; Marvin Hsiao; Stephen Korsman; Tegally H; de Oliveira T
EPI_ISL_1469766	Coordenadoria Geral de Vigilância em Saúde · Vigilância em Saúde	Epiclin	Ana Paula Muterle; Carolina Comerlato; Eliana Márcia Da Ros Wendland; Fernando Hayashi Sant'Anna; Janira Prichula; Juliana Comerlato
EPI_ISL_1439696 to 1439707	Corona-Testzentrum ifp Institut für Produktqualität GmbH	Robert Koch Institute	
EPI_ISL_514751	CoronaNet Lab- TaskForce Regione Campania, CEINGE Biotecnologie Avanzate, Via G. Salvatore	CoronaNet Lab- TaskForce Regione Campania, CEINGE Biotecnologie Avanzate, Via G. Salvatore	Asadzadeh, F.; Atripaldi, L.; Bianchi, M.; Boccia, A.; Borriello, G.; Brandi, S.; Castaldo, G.; Cerino, R.; Chiariotti, L.; Comegna, M.; Ferrucci, V.; Fusco, G.; H.Y.; J.H.; J.M.; Jung; K.S. and Kim; Kong, Dy.; Lee; Marrone, L.; Paolella, G.; Pascarella, S.; Siciliano, R.; Tiberio, C.; Viscardi, M.; Yun; Zollo, M.
EPI_ISL_1553234	Corssing Healthcare	Illinois Department of Public Health - Springfield Lab	Bryan Sim; Gordon McCall
EPI_ISL_1418323	County Emergency Hospital Piteti	National Institute of Infectious Diseases-Prof. Dr. Matei Bals Molecular Diagnostics Laboratory	Corina Casangiu; Dan Otelea; Ionelia Nicolae; Leontina Banica; Marius Surleac; Ovidiu Vlaicu; Simona Paraschiv
EPI_ISL_454635	County Of San Luis Obispo Public Health Laboratory	Chan-Zuckerberg Biohub	CZB Cliahub Consortium

EPI_ISL_468388 to 468437, EPI_ISL_548265, EPI_ISL_548268 to 548269, EPI_ISL_548280 to 548281, EPI_ISL_548284, EPI_ISL_548286, EPI_ISL_548288, EPI_ISL_548292, EPI_ISL_548294 to 548295, EPI_ISL_548304, EPI_ISL_548311, EPI_ISL_548316, EPI_ISL_548322, EPI_ISL_548325, EPI_ISL_548329 to 548330, EPI_ISL_548335 to 548336, EPI_ISL_548347, EPI_ISL_548350 to 548351, EPI_ISL_548357, EPI_ISL_548359 to 548361, EPI_ISL_548365 to 548366, EPI_ISL_548369 to 548370, EPI_ISL_548391, EPI_ISL_548425, EPI_ISL_548428, EPI_ISL_548436, EPI_ISL_548453, EPI_ISL_548457, EPI_ISL_548493, EPI_ISL_548502, EPI_ISL_548504, EPI_ISL_548517, EPI_ISL_548519, EPI_ISL_548521, EPI_ISL_548532, EPI_ISL_548542, EPI_ISL_548544, EPI_ISL_548560, EPI_ISL_548603, EPI_ISL_548631, EPI_ISL_548644, EPI_ISL_548663, EPI_ISL_548671, EPI_ISL_1477436, EPI_ISL_1477454, EPI_ISL_1477530, EPI_ISL_1477550, EPI_ISL_1477564, EPI_ISL_1477626, EPI_ISL_1477634, EPI_ISL_1477965, EPI_ISL_1477992, EPI_ISL_1478006, EPI_ISL_1478018, EPI_ISL_1478025, EPI_ISL_1478044 to 1478045, EPI_ISL_1478061, EPI_ISL_1478070, EPI_ISL_1478074, EPI_ISL_1478080, EPI_ISL_1478118, EPI_ISL_1478136, EPI_ISL_1478174, EPI_ISL_1478179, EPI_ISL_1478195, EPI_ISL_1478198, EPI_ISL_1529127 to 1529129, EPI_ISL_1529147, EPI_ISL_1529161, EPI_ISL_1529168, EPI_ISL_1529208, EPI_ISL_1529213, EPI_ISL_1529225, EPI_ISL_1529232, EPI_ISL_1529237, EPI_ISL_1529246, EPI_ISL_1529248, EPI_ISL_1529255 to 1529256, EPI_ISL_1529268, EPI_ISL_1529270, EPI_ISL_1529279, EPI_ISL_1529285, EPI_ISL_1529292 to 1529293, EPI_ISL_1529311, EPI_ISL_1529329, EPI_ISL_1529341 to 1529343			
see above	County of San Luis Obispo Public Health Laboratory	Chan-Zuckerberg Biohub	CZB Cliahub Consortium
EPI_ISL_437043 to 437088, EPI_ISL_444023 to 444026	County of Santa Clara Public Health	Chan-Zuckerberg Biohub	CZB Cliahub Consortium
EPI_ISL_436641 to 436683, EPI_ISL_454653 to 454689, EPI_ISL_468345 to 468356, EPI_ISL_486115 to 486119, EPI_ISL_513773 to 513788, EPI_ISL_548264, EPI_ISL_548266 to 548267, EPI_ISL_548277, EPI_ISL_548279, EPI_ISL_548282, EPI_ISL_548290, EPI_ISL_548299, EPI_ISL_548302 to 548303, EPI_ISL_548307, EPI_ISL_548310, EPI_ISL_548314, EPI_ISL_548319 to 548321, EPI_ISL_548324, EPI_ISL_548327, EPI_ISL_548331, EPI_ISL_548333, EPI_ISL_548337 to 548345, EPI_ISL_548371, EPI_ISL_548379, EPI_ISL_548384 to 548385, EPI_ISL_548388, EPI_ISL_548392, EPI_ISL_548394 to 548396, EPI_ISL_548399, EPI_ISL_548401, EPI_ISL_548403, EPI_ISL_548407, EPI_ISL_548415, EPI_ISL_548417, EPI_ISL_548419 to 548423, EPI_ISL_548426, EPI_ISL_548429, EPI_ISL_548432 to 548434, EPI_ISL_548437, EPI_ISL_548446, EPI_ISL_548448, EPI_ISL_548451, EPI_ISL_548466, EPI_ISL_548468, EPI_ISL_548470, EPI_ISL_548472, EPI_ISL_548478, EPI_ISL_548482, EPI_ISL_548494, EPI_ISL_548496 to 548498, EPI_ISL_548500, EPI_ISL_548503, EPI_ISL_548509, EPI_ISL_548511 to 548512, EPI_ISL_548514, EPI_ISL_548518, EPI_ISL_548523, EPI_ISL_548525 to 548526, EPI_ISL_548529 to 548530, EPI_ISL_548533, EPI_ISL_548536, EPI_ISL_548541, EPI_ISL_548543, EPI_ISL_548546 to 548547, EPI_ISL_548550 to 548551, EPI_ISL_548553, EPI_ISL_548559, EPI_ISL_548562 to 548563, EPI_ISL_548568, EPI_ISL_548570, EPI_ISL_548576, EPI_ISL_548578, EPI_ISL_548582 to 548584, EPI_ISL_548588, EPI_ISL_548593, EPI_ISL_548595 to 548598, EPI_ISL_548600, EPI_ISL_548602, EPI_ISL_548604 to 548607, EPI_ISL_548610, EPI_ISL_548612, EPI_ISL_548616 to 548619, EPI_ISL_548621 to 548622, EPI_ISL_548624, EPI_ISL_548629 to 548630, EPI_ISL_548632 to 548633, EPI_ISL_548636, EPI_ISL_548639, EPI_ISL_548642 to 548643, EPI_ISL_548645, EPI_ISL_548647, EPI_ISL_548649 to 548650, EPI_ISL_548652, EPI_ISL_548655 to 548659, EPI_ISL_548661, EPI_ISL_548664, EPI_ISL_548669 to 548670, EPI_ISL_548672 to 548673, EPI_ISL_548676, EPI_ISL_548678 to 548679, EPI_ISL_582851 to 582958			
see above	County of Santa Clara Public Health Department	Chan-Zuckerberg Biohub	CZB Cliahub Consortium
EPI_ISL_1534338, EPI_ISL_1534419	Crags Clinic wc CRG	NHLS/UCT	Arash Iranzadeh; Bruna Galvao; Carolyn Williamson; Deelan Doolabh; Diana Hardie; Emmanuel SJ; Innocent Mudau; Kruger Marais; Lynn Tyers; Marvin Hsiao; Stephen Korsman; Tegally H; de Oliveira T
EPI_ISL_1435898, EPI_ISL_1435904, EPI_ISL_1435906, EPI_ISL_1435911, EPI_ISL_1435915, EPI_ISL_1435926, EPI_ISL_1435928, EPI_ISL_1435931, EPI_ISL_1435935, EPI_ISL_1435973, EPI_ISL_1435975, EPI_ISL_1435977, EPI_ISL_1435979, EPI_ISL_1435981, EPI_ISL_1435983, EPI_ISL_1435985, EPI_ISL_1435987, EPI_ISL_1435989, EPI_ISL_1435991 to 1435992, EPI_ISL_1436009, EPI_ISL_1436011, EPI_ISL_1436015, EPI_ISL_1436017, EPI_ISL_1436020, EPI_ISL_1436035, EPI_ISL_1436037, EPI_ISL_1436050, EPI_ISL_1436052, EPI_ISL_1436054, EPI_ISL_1436056, EPI_ISL_1436058, EPI_ISL_1443513 to 1443538, EPI_ISL_1499569 to 1499571			
see above	Croatian Institute of Public Health	Croatian Institute of Public Health	Irena Tabain; Ivana Ferenak
EPI_ISL_454602	Croatian Institute of Public Health	University of Zagreb, Centre for research and knowledge transfer in biotechnology	Anamarija Slovic; Irena Tabain; Jelena Ivancic Jelecki; Tatjana Vilibic-Cavlek
EPI_ISL_1436048	Croatian Institute of Public health	Croatian Institute of Public Health	Irena Tabain; Ivana Ferenak
EPI_ISL_1443274, EPI_ISL_1443289, EPI_ISL_1478857, EPI_ISL_1478910 to 1478911, EPI_ISL_1478913 to 1478918, EPI_ISL_1500204 to 1500205, EPI_ISL_1500232 to 1500234, EPI_ISL_1553184			
see above	Crossing Healthcare	Illinois Department of Public Health - Springfield Lab	Bryan Sim; Gordon McCall
EPI_ISL_1522293, EPI_ISL_1522295, EPI_ISL_1522297, EPI_ISL_1522299, EPI_ISL_1522302, EPI_ISL_1522304, EPI_ISL_1522306, EPI_ISL_1522309, EPI_ISL_1522311, EPI_ISL_1522313, EPI_ISL_1522316, EPI_ISL_1522318, EPI_ISL_1522321, EPI_ISL_1522324, EPI_ISL_1522326, EPI_ISL_1522328, EPI_ISL_1522331, EPI_ISL_1522333, EPI_ISL_1522336, EPI_ISL_1522339, EPI_ISL_1522341, EPI_ISL_1522343, EPI_ISL_1522346, EPI_ISL_1522349, EPI_ISL_1522351, EPI_ISL_1522354, EPI_ISL_1522357, EPI_ISL_1522359, EPI_ISL_1522361, EPI_ISL_1522363, EPI_ISL_1522365, EPI_ISL_1522367 to 1522381			
see above	Curative	New Mexico Department of Health Scientific Laboratory	Anastacia Griego-Fisher; D'eldra Malone; Ellie Johnson; Jennifer Benoit; Linda Salazar; Ratheesh Rajan
EPI_ISL_1503991	Curative Labs	Los Angeles County PHL	P. Hemarajata et al.
EPI_ISL_1392907 to 1392915, EPI_ISL_1396554 to 1396555, EPI_ISL_1447084, EPI_ISL_1461634 to 1461637, EPI_ISL_1494554 to 1494556, EPI_ISL_1510636 to 1510640, EPI_ISL_1510647			
see above	Cytocheck Laboratory	Kansas Health and Environmental Lab	Ben Olsen; Jonathan Barnell; Mike Grose; and Phil Adam
EPI_ISL_1534270, EPI_ISL_1534284 to 1534285, EPI_ISL_1534297, EPI_ISL_1534436, EPI_ISL_1534439 to 1534440, EPI_ISL_1534460	D'Almeida Clinic wc DAL	NHLS/UCT	Arash Iranzadeh; Bruna Galvao; Carolyn Williamson; Deelan Doolabh; Diana Hardie; Emmanuel SJ; Innocent Mudau; Kruger Marais; Lynn Tyers; Marvin Hsiao; Stephen Korsman; Tegally H; de Oliveira T
EPI_ISL_476171 to 476202, EPI_ISL_476209 to 476216, EPI_ISL_476218 to 476219, EPI_ISL_476224 to 476229, EPI_ISL_476232, EPI_ISL_476278 to 476336, EPI_ISL_476350 to 476371			
see above	DB Diagnósticos do Brasil	Instituto de Medicina Tropical da Univesidade de São Paulo	Camila Alves Maia da Silva; Darlan da Silva Candido; Erika Regina Manuli; Ester Sabino; Flavia Cristina da Silva Sales; Giula Magalhaes Ferreira; Jaqueline Goes de Jesus; Julien Theze; Mariana Severo Ramundo; Nuno Faria; Samples: Nelson Gaburo Jr; Sequencing: Ingra Morales Claro; Thais de Moura Coletti
EPI_ISL_594460	DC Department of Forensic Sciences	Pathogen Discovery, Respiratory Viruses Branch, Division of Viral Diseases, Centers for Disease Control and Prevention	Anna Uehara; Clinton Paden; Haibin Wang; Jing Zhang; Julu Bhatnagar; Krista Queen; Suxiang Tong; Yan Li; Ying Tao
EPI_ISL_436040 to 436043	DC Public Health Lab Dept of Forensic Science	Pathogen Discovery, Respiratory Viruses Branch, Division of Viral Diseases, Centers for Disease Control and Prevention	Anna Uehara; Bettina Bankamp; Clinton R. Paden; Haibin Wang; Jing Zhang; Krista Queen; Suxiang Tong; Yan Li; Ying Tao; Zachary Weiner
EPI_ISL_1446970 to 1446973, EPI_ISL_1447048, EPI_ISL_1528442 to 1528443	DC Public Health Lab/ Dept. of Forensic Sciences	Centers for Disease Control and Prevention Division of Viral Diseases, Pathogen Discovery	Alison Laufer Halpin; Ben L. Rambo-Martin; Clinton R. Paden; Dakota Howard; Darlene Wagner; Dave Wentworth; Dhwani Batra; Jasmine Padilla; Justin Lee; Katie Dillon; Krista Queen; Kristen Knipe; Kristine Lacek; Mark Burroughs; Matthew Schmerer; Mili Sheth; Peter Cook; Sam Shepard; Sarah Nobles; Shoshona Le; Suxiang Tong; Vivien Dugan; Yvette Unoarumhi
EPI_ISL_1391372 to 1391392, EPI_ISL_1396407, EPI_ISL_1403613 to 1403614, EPI_ISL_1404576 to 1404587, EPI_ISL_1411004 to 1411007, EPI_ISL_1482632 to 1482646, EPI_ISL_1491523 to 1491524, EPI_ISL_1502075 to 1502086, EPI_ISL_1515798 to 1515799, EPI_ISL_1516749 to 1516752, EPI_ISL_1551362, EPI_ISL_1551364 to 1551365, EPI_ISL_1551367 to 1551368, EPI_ISL_1551370, EPI_ISL_1551372 to 1551373			
see above	DC Public Health Lab/ Dept. of Forensic Sciences	DC Public Health Lab/ Dept. of Forensic Sciences	Brittany Hamilton; Connie Maza; David Payne; Elizabeth Zelaya; Janis Doss; Jocelyn Hauser; Monica Mann; Sarah Scott; Scott Nguyen
EPI_ISL_424852, EPI_ISL_447840, EPI_ISL_576178	DC Public Health Lab/ Dept. of Forensic Sciences	Pathogen Discovery, Respiratory Viruses Branch, Division of Viral Diseases, Centers for Disease Control and Prevention	Alison S. Laufer Halpin; Anna Uehara; Brian Lynch; Christopher A. Elkins; Clinton R. Paden; Haibin Wang; Jasmine Padilla; Jing Zhang; Justin Lee; Krista Queen; Mary S. Keckler; Peter Cook; Rachel Marine; Suxiang Tong; Yan Li; Ying Tao
EPI_ISL_1446607 to 1446619, EPI_ISL_1528303 to 1528307	DE Public Health Laboratory	Centers for Disease Control and Prevention Division of Viral Diseases, Pathogen Discovery	Alison Laufer Halpin; Ben L. Rambo-Martin; Clinton R. Paden; Dakota Howard; Darlene Wagner; Dave Wentworth; Dhwani Batra; Jasmine Padilla; Justin Lee; Katie Dillon; Krista Queen; Kristen Knipe; Kristine Lacek; Mark Burroughs; Matthew Schmerer; Mili Sheth; Peter Cook; Sam Shepard; Sarah Nobles; Shoshona Le; Suxiang Tong; Vivien Dugan; Yvette Unoarumhi
EPI_ISL_1445175	DEPARTAMENTO DE SAUDE COLETIVA	Instituto Butantan / Mendelics	Antonio Jorge Martins; Bibiana Santos; Claudia Renata dos Santos Barros; David Schlesinger; Debora Botequio Moretti; Dimas Tadeu Covas; Elaine Cristina Marqueze; Elaine Vieira dos Santos; Erika Freitas; Evandra Strazza Rodrigues; Flavia Aburjaile; José Salvatore Leister Patané; João Paulo Kitajima; Luiz Carlos Junior de Alcantara; Maria Carolina Elias; Marta Giovanetti; Rafael dos Santos Bezerra; Raul Machado Neto; Ricardo Haddad; Rodrigo Tocantins Calado.; Sandra Coccuzzo Sampaio; Simone Kashima; Svetoslav Nanev Slavov; Vagner Fonseca; Vincent Louis Viala
EPI_ISL_486384, EPI_ISL_486387 to 486389, EPI_ISL_486401 to 486403, EPI_ISL_486405 to 486409, EPI_ISL_515932, EPI_ISL_515934 to 515936, EPI_ISL_515945 to 515949, EPI_ISL_515954 to 515955, EPI_ISL_515957 to 515959, EPI_ISL_515961 to 515962, EPI_ISL_515965, EPI_ISL_583914			
see above	DH	Department of Neurovirology, National Institute of Mental Health and Neuroscience (NIMHANS)	Anita Desai; Chitra Pattabiraman; Harsha PK; Manjunatha Venkataswamy; Pramada Prasad; Ravi Vasanthapuram; Risha Rasheed; Shafeeq S Hameed; Vijayalakshmi Reddy
EPI_ISL_1391403	DIP. PREV. AVEZZANO SERVIZIO DI IGIENE EPIDEMIOLOGIA E SANITA' PUBBLICA	Istituto Zooprofilattico Sperimentale dell'Abruzzo e Molise "G. Caporale"	Ancora M; Calistri P; Cammà C; Caporale M; Curini V; Di Domenico M; Di Lollo Valeria; Di Pasquale A; Lorusso A; Mangone I; Marcacci M; Puglia I; Rinaldi A; Savini G; Scialabba S
EPI_ISL_1391238, EPI_ISL_1391241 to 1391247, EPI_ISL_1392667 to 1392669, EPI_ISL_1392672 to 1392673, EPI_ISL_1392675 to 1392679, EPI_ISL_1392711 to 1392712, EPI_ISL_1403512 to 1403513, EPI_ISL_1403541, EPI_ISL_1403544, EPI_ISL_1403546, EPI_ISL_1403584, EPI_ISL_1403601, EPI_ISL_1502475 to 1502479, EPI_ISL_1502481, EPI_ISL_1502512, EPI_ISL_1502514 to 1502516, EPI_ISL_1502519 to 1502521			
see above	DIP. PREV. AVEZZANO SERVIZIO DI IGIENE EPIDEMIOLOGIA E SANITA' PUBBLICA AVEZZANO(L'AQUILA)	Istituto Zooprofilattico Sperimentale dell'Abruzzo e Molise "G. Caporale"	Ancora M; Calistri P; Cammà C; Caporale M; Curini V; Delli Compagni E; Di Domenico M; Di Lollo Valeria; Di Pasquale A; Lorusso A; Mangone I; Marcacci M; Puglia I; Rinaldi A; Savini G; Scialabba S
EPI_ISL_1469558, EPI_ISL_1469566, EPI_ISL_1469579, EPI_ISL_1469590, EPI_ISL_1469594, EPI_ISL_1469611, EPI_ISL_1469633, EPI_ISL_1469637 to 1469638, EPI_ISL_1469652, EPI_ISL_1469659, EPI_ISL_1469663, EPI_ISL_1469674 to 1469675, EPI_ISL_1469677, EPI_ISL_1469679, EPI_ISL_1469681,			

EPI_ISL_1469684, EPI_ISL_1469686, EPI_ISL_1469688, EPI_ISL_1469692, EPI_ISL_1469710, EPI_ISL_1469725, EPI_ISL_1469731, EPI_ISL_1469743, EPI_ISL_1469760, EPI_ISL_1469764, EPI_ISL_1469772, EPI_ISL_1469781, EPI_ISL_1469784, EPI_ISL_1469791, EPI_ISL_1469798, EPI_ISL_1479119, EPI_ISL_1479121			
see above	DIRETORIA DE VIGILANCIA EM SAUDE	Epiclin	Ana Paula Muterle; Carolina Comerlato; Eliana Márcia Da Ros Wendland; Fernando Hayashi Sant'Anna; Janira Prichula; Juliana Comerlato
EPI_ISL_1394006 to 1394009, EPI_ISL_1538518, EPI_ISL_1538536, EPI_ISL_1538554, EPI_ISL_1538735 to 1538736, EPI_ISL_1539018, EPI_ISL_1539182, EPI_ISL_1539217			
see above	DL OBM WSSE w Lublinie	1. National Institute of Public Health - National Institute of Hygiene; 2. Eurofins Genomics Europe Sequencing GmbH	ECDC COVID-19 WGS support team; Eurofins Genomics Europe Sequencing Team; Gierczyki Rafa; Sadkowska-Todys Magorzata; Wokowicz Tomasz; Zacharczuk Katarzyna
EPI_ISL_445213	DNA Solution Ltd	DNA Solution Ltd	Abdul Khaleque; Abu Sufian; Hasan Ul Haider; Kazi Nadim Hasan; MSM Chowdhury; Mala Khan; Mamudul Hasan Razu; Md. Imran Khan; Mizanur Rahman; Mohammad Fazle Alam Rabbi; Mohammed Nafiz Imtiaz Polol
EPI_ISL_445214 to 445217	DNA Solution Ltd.	DNA Solution Ltd.	Abdul Khaleque; Abu Sufian; Hasan Ul Haider; Kazi Nadim Hasan; MSM Chowdhury; Mala Khan; Mamudul Hasan Razu; Md. Imran Khan; Mizanur Rahman; Mohammad Fazle Alam Rabbi; Mohammed Nafiz Imtiaz Polol
EPI_ISL_1435783, EPI_ISL_1435785, EPI_ISL_1435787, EPI_ISL_1435789, EPI_ISL_1435791 to 1435792, EPI_ISL_1435794, EPI_ISL_1435796, EPI_ISL_1435798, EPI_ISL_1435800			
see above	DNALAB Sdn. Bhd.	Malaysia Genome Institute	Azrin Ahmad; Enizza Kasim; Irni Suhayu Sapian; Mohd Faizal Abu Bakar; Mohd Noor Mat Isa; Nor Azfa Johari.; Nurhezreen Md Iqbal; Shamsidar Sopie; Siti Noraini Othman; Wong Yong Wee; Yusuf Muhammad Noor
EPI_ISL_1447101, EPI_ISL_1447109, EPI_ISL_1447116, EPI_ISL_1447129 to 1447130, EPI_ISL_1447134, EPI_ISL_1447167 to 1447168, EPI_ISL_1447186			
see above	DOHMH Central Harlem	New York City Public Health Laboratory	Jade Wang; et al.
EPI_ISL_1447093, EPI_ISL_1447096, EPI_ISL_1447118, EPI_ISL_1447123, EPI_ISL_1447133, EPI_ISL_1447156 to 1447157, EPI_ISL_1447169, EPI_ISL_1447193			
see above	DOHMH Chelsea	New York City Public Health Laboratory	Jade Wang; et al.
EPI_ISL_1447088, EPI_ISL_1447095, EPI_ISL_1447126, EPI_ISL_1447136 to 1447137, EPI_ISL_1447139 to 1447142, EPI_ISL_1447144, EPI_ISL_1447147, EPI_ISL_1447155, EPI_ISL_1447160, EPI_ISL_1447165, EPI_ISL_1447171 to 1447172, EPI_ISL_1447185			
see above	DOHMH Corona	New York City Public Health Laboratory	Jade Wang; et al.
EPI_ISL_1447089, EPI_ISL_1447113, EPI_ISL_1447128, EPI_ISL_1447148, EPI_ISL_1447161 to 1447162, EPI_ISL_1447166, EPI_ISL_1447170, EPI_ISL_1447191			
see above	DOHMH Crown Heights	New York City Public Health Laboratory	Jade Wang; et al.
EPI_ISL_1447098, EPI_ISL_1447112, EPI_ISL_1447115, EPI_ISL_1447127, EPI_ISL_1447135, EPI_ISL_1447176, EPI_ISL_1447187			
see above	DOHMH Fort Greene	New York City Public Health Laboratory	Jade Wang; et al.
EPI_ISL_1447092, EPI_ISL_1447094, EPI_ISL_1447103, EPI_ISL_1447106, EPI_ISL_1447117, EPI_ISL_1447120 to 1447122, EPI_ISL_1447124 to 1447125, EPI_ISL_1447131 to 1447132, EPI_ISL_1447146, EPI_ISL_1447151 to 1447153, EPI_ISL_1447158 to 1447159, EPI_ISL_1447163 to 1447164, EPI_ISL_1447175, EPI_ISL_1447179 to 1447180, EPI_ISL_1447188 to 1447190, EPI_ISL_1447192			
see above	DOHMH Jamaica	New York City Public Health Laboratory	Jade Wang; et al.
EPI_ISL_1447087, EPI_ISL_1447090, EPI_ISL_1447097, EPI_ISL_1447108, EPI_ISL_1447110, EPI_ISL_1447181	DOHMH Morrisania	New York City Public Health Laboratory	Jade Wang; et al.
EPI_ISL_1447099, EPI_ISL_1447107, EPI_ISL_1447145, EPI_ISL_1447149 to 1447150, EPI_ISL_1447184	DOHMH PHL	New York City Public Health Laboratory	Jade Wang; et al.
EPI_ISL_1447105, EPI_ISL_1447154, EPI_ISL_1447173	DOHMH Riverside	New York City Public Health Laboratory	Jade Wang; et al.
EPI_ISL_1534421	DP Marais Hospital wc DPM	NHLS/UCT	Arash Iranzadeh; Bruna Galvao; Carolyn Williamson; Deelan Doolabh; Diana Hardie; Emmanuel SJ; Innocent Mudau; Kruger Marais; Lynn Tyers; Marvin Hsiao; Stephen Korsman; Tegally H; de Oliveira T
EPI_ISL_1511139 to 1511197	DPHL	Delaware Public Health Lab	Rebecca Savage
EPI_ISL_1433803, EPI_ISL_1433806, EPI_ISL_1433808, EPI_ISL_1433810, EPI_ISL_1433880, EPI_ISL_1433882, EPI_ISL_1433884, EPI_ISL_1433886, EPI_ISL_1433909, EPI_ISL_1433939, EPI_ISL_1433941, EPI_ISL_1433943, EPI_ISL_1433945, EPI_ISL_1433947, EPI_ISL_1433949, EPI_ISL_1433951, EPI_ISL_1433953, EPI_ISL_1433955, EPI_ISL_1433957, EPI_ISL_1433981, EPI_ISL_1434008, EPI_ISL_1434010, EPI_ISL_1434012, EPI_ISL_1434039, EPI_ISL_1434041 to 1434042, EPI_ISL_1434046, EPI_ISL_1434049, EPI_ISL_1434051, EPI_ISL_1434053, EPI_ISL_1434055, EPI_ISL_1434057, EPI_ISL_1434059, EPI_ISL_1434079, EPI_ISL_1434081, EPI_ISL_1434083, EPI_ISL_1434085, EPI_ISL_1434087, EPI_ISL_1434089, EPI_ISL_1434091, EPI_ISL_1434093, EPI_ISL_1434095, EPI_ISL_1434098 to 1434099, EPI_ISL_1434101, EPI_ISL_1434103, EPI_ISL_1434105, EPI_ISL_1434107, EPI_ISL_1434109, EPI_ISL_1434111 to 1434112, EPI_ISL_1434115 to 1434116, EPI_ISL_1434118, EPI_ISL_1434120, EPI_ISL_1434122, EPI_ISL_1434124, EPI_ISL_1434126, EPI_ISL_1434128 to 1434129, EPI_ISL_1434132, EPI_ISL_1434134, EPI_ISL_1434136, EPI_ISL_1434138, EPI_ISL_1434140, EPI_ISL_1434142, EPI_ISL_1434217, EPI_ISL_1434235, EPI_ISL_1434237, EPI_ISL_1434256, EPI_ISL_1434266, EPI_ISL_1434268, EPI_ISL_1457760, EPI_ISL_1457768 to 1457769			
see above	DYOMEDEA SAUVEGARDE LYON	CNR Virus des Infections Respiratoires - France SUD	Antonin Bal; Bruno Lina; Gregory Destras; Gwendolyne Burfin; Hadrien Regue; Laurence Josset; Martine Valette; Quentin Semanas
EPI_ISL_416542 to 416543, EPI_ISL_421652	Dasman Diabetes Institute	Dasman Diabetes Institute	Ebaa Al-Ozairi; Ebaa AlOzairi; Fahd Al-Mulla; Motasem Melhem; Qais Al-Duwairi; Rasheeba Iqbal; Sara Al-Qabandi; Sumi John
EPI_ISL_416541	Dasman Diabetes Institute and Virology Laboratory Ministry of Health	Dasman Diabetes Institute	Ebaa AlOzairi; Fahd Al-Mulla; Motasem Melhem; Qais Al-Duwairi; Rasheeba Iqbal; Sara Al-Qabandi; Sumi John
EPI_ISL_1534365	De Rust (Blommenek) Clinic wc DRC	NHLS/UCT	Arash Iranzadeh; Bruna Galvao; Carolyn Williamson; Deelan Doolabh; Diana Hardie; Emmanuel SJ; Innocent Mudau; Kruger Marais; Lynn Tyers; Marvin Hsiao; Stephen Korsman; Tegally H; de Oliveira T
EPI_ISL_1524383	Debswana Orapa Mine Hospital Laboratory	Botswana Harvard HIV Reference Laboratory	Boitumelo Zuze; Botshelo Radibe; David Lawrence; Dorcas Maruapula; Joseph Makhema; Koketso Maotwe; Legodile Kooepile; Lesedi Magama; Mosepele Mosepele; Roger Shapiro; Shahin Lockman; Sikhulile Moyo; Simani Gaseitsiwe; Thongbotho Mphoyakgosi; Wonderful T. Choga
EPI_ISL_1500237, EPI_ISL_1553187	Decatur Memorial Hospital	Illinois Department of Public Health - Springfield Lab	Bryan Sim; Gordon McCall
EPI_ISL_476022	Defence Research & Development Establishment	Defence Research & Development Establishment	Ambuj Shrivastava; Jyoti S Kumar; Paban Kumar Dash; Shashi Sharma; Sushil Kumar Sharma
EPI_ISL_476023, EPI_ISL_476840, EPI_ISL_476842, EPI_ISL_476844, EPI_ISL_476846, EPI_ISL_476848 to 476850, EPI_ISL_476852 to 476854, EPI_ISL_476883 to 476896			
see above	Defence Research & Development Establishment (DRDE)	Defence Research & Development Establishment (DRDE)	Ambuj Shrivastava; Jyoti S. Kumar; Paban Kumar Dash; Shashi Sharma; Sushil Kumar Sharma
EPI_ISL_560307, EPI_ISL_560406, EPI_ISL_560567, EPI_ISL_560647 to 560648, EPI_ISL_560741 to 560742, EPI_ISL_561335 to 561336, EPI_ISL_561345 to 561353, EPI_ISL_561356 to 561360, EPI_ISL_561362, EPI_ISL_565830 to 565832, EPI_ISL_566084, EPI_ISL_566108 to 566109, EPI_ISL_574581, EPI_ISL_574584 to 574586, EPI_ISL_574599, EPI_ISL_575335, EPI_ISL_576150 to 576169, EPI_ISL_576222 to 576227, EPI_ISL_582133, EPI_ISL_583246 to 583248, EPI_ISL_583994 to 583995, EPI_ISL_584013 to 584014, EPI_ISL_593937 to 593981			
see above	Delaware Public Health Lab	Delaware Public Health Lab	Gregory Hovan
EPI_ISL_529147 to 529148	Democritus University of Thrace, Department of Medicine	Democritus University of Thrace, Department of Medicine	Bampali, M.; Dovrolis, N.; Froukala, E.; Gatzidou, E.; Karakasiliotis, I.; Kassela, K.; Spanakis, N.; Stavropoulou, A.; Tsakris, A.; Veletza, S.
EPI_ISL_430796 to 430798, EPI_ISL_430802, EPI_ISL_430805 to 430806, EPI_ISL_430808	Departamento de Biología y genética molecular, IACA Laboratorios.	Área de Secuenciación del Laboratorio de Virología del Hospital de Niños Dr. Ricardo Gutierrez on behalf of 'Proyecto Argentino Interinstitucional de genomica de SARS-CoV-2' (PAIS Consortium)	A; AS; E; Goya; LE; Lusso; MI; MS; Masciovecchio MV; Mistchenko; Nabaes Jodar; Natale; S; Streitenberger ER; Suárez; Tittarelli; Valinotto; Viegas, M.
EPI_ISL_444493	Departamento de Laboratorios de Salud Publica (DLSP, Division Epidemiologia, Ministerio de Salud Publica)	Facultad de Ciencias (Sección Genética Evolutiva, Sección Virología).	Arbiza; Calleros, L.; Chiparelli, H.; Coppola, L.; Delfraro, A.; Frabasile, S.; Fuques, E.; Goni, N.; Grecco, S.; J. and Perez, R.; Panzera, Y.; Ramos, N.; Ramos, V.; Techera, C.
EPI_ISL_508615 to 508686, EPI_ISL_1423762 to 1423792, EPI_ISL_1423849 to 1423857	Departamento de Microbiología, CDB, Hospital Clínic, Barcelona	SeqCOVID-SPAIN consortium/IBV(CSIC)	Aida Peiró and SeqCOVID-SPAIN consortium; Andrea Vergara; Elisa Rubio; Jéssica Navero; Mikel Martínez
EPI_ISL_603022, EPI_ISL_603034	Departamento de Vigilância à Saúde	Instituto Adolfo Lutz, Interdiciplinary Procedures Center, Strategic Laboratory	Claudia Regina Gonçalves; Claudio Tavares Sacchi; Erica Valessa Ramos Gomes; Karoline Rodrigues Campos

EPI_ISL_516922 to 516933	Department for Molecular Diagnostics, Centre for Medical Microbiology, Institute of Public Health of Montenegro	Charite Universitatsmedizin Berlin, Institut fur Virologie	Barbara Muehlemann; Christian Drosten; Julia Schneider; Jörn Beheim-Schwarzbach; Marija Govedarica and Danijela Vujoševi; Talitha Veith; Terry Jones; Victor M Corman
EPI_ISL_420140, EPI_ISL_420142, EPI_ISL_420144	Department for Virology, Molecular Biology and Genome Research, R. G. Lugar Center for Public Health Research, National Center for Disease Control and Public Health (NCDC) of Georgia.	Department for Virology, Molecular Biology and Genome Research, R. G. Lugar Center for Public Health Research, National Center for Disease Control and Public Health (NCDC) of Georgia.	Adam Kotorashvili; Amiran Gamkrelidze; Amiran Gamkrelidze.; Ana Papkiauri; Ann Machablishvili; Anna Kasradze; Davit Tsaguria; Ekaterine Khmaladze; Ekaterine Zangaladze; Ekaterine Zhghenti; Giorgi Tomashvili; Gvantsa Brachveli; Gvantsa Chanturia; Irma Burjanadze; Ketevan Sidamonidze; Khatuna Zakhashvili; Lela Sabadze; Lela Urushadze; Magda Dgebuadze; Maia Alkhazashvili; Mari Gavashelidze; Mariam Zakalashvili; Marine Murtskhvaladze; Meri Pantsulaia; Nato Kotaria; Nino Berishvili; Paata Imnadze; Roena Sukhiashvili; Tamar Jashiashvili; Tata Imnadze; Tea Tevdoradze
EPI_ISL_447055 to 447056, EPI_ISL_470876 to 470877, EPI_ISL_471529, EPI_ISL_477169, EPI_ISL_481380, EPI_ISL_481483	Department for Virology, Molecular Biology and Genome Research, R. G. Lugar Center for Public Health Research, National Center for Disease Control and Public Health (NCDC) of Georgia.	Department for Virology, Molecular Biology and Genome Research, R. G. Lugar Center for Public Health Research, National Center for Disease Control and Public Health (NCDC) of Georgia.	Adam Kotorashvili; Amiran Gamkrelidze.; Ana Papkiauri; Ann Machablishvili; Anna Kasradze; Davit Tsaguria; Ekaterine Khmaladze; Ekaterine Zangaladze; Ekaterine Zhghenti; Giorgi Tomashvili; Gvantsa Brachveli; Gvantsa Chanturia; Irma Burjanadze; Ketevan Sidamonidze; Khatuna Zakhashvili; Lela Sabadze; Lela Urushadze; Magda Dgebuadze; Maia Alkhazashvili; Mari Gavashelidze; Mariam Zakalashvili; Marine Murtskhvaladze; Meri Pantsulaia; Nato Kotaria; Nino Berishvili; Paata Imnadze; Roena Sukhiashvili; Tamar Jashiashvili; Tata Imnadze; Tea Tevdoradze
EPI_ISL_515082 to 515112	Department of Biochemistry, Cell and Molecular Biology	WACCBIP, University of Ghana	A.K.; Adu, B.; Amenga-Etego; Ampofo, W.; Amuzu; Anang; Arjarquah, A.; Asante, I.; Awandare; Bediako, Y.; Boatemaa, L.; Bonney, E.; Bonney, K.; C.M.; D.S.; Eshun, M.; G.A.; G.B.; J.K.; J.M.; Kotey, E.; Kumordjie, S.; Kyei; L.N.; Magnussen, V.; Morang'a; Mutungi; Ngoi; Quashie, P.; Tei-Maya, F.
EPI_ISL_429239	Department of Clinical Laboratory, the First People's Hospital of Yunnan Province	Department of Clinical Laboratory, the First People's Hospital of Yunnan Province	Guiqian Zhang; Xin Fan; Ya Xu; Yi Sun; Yu Zhang; Ziqin Dian
EPI_ISL_416997, EPI_ISL_417004 to 417006, EPI_ISL_417008 to 417009, EPI_ISL_417011 to 417023, EPI_ISL_417025, EPI_ISL_418623 to 418666, EPI_ISL_421182 to 421214, EPI_ISL_424628 to 424665, EPI_ISL_427340 to 427390, EPI_ISL_447120 to 447162, EPI_ISL_455958 to 455979, EPI_ISL_471427 to 471437, EPI_ISL_484693 to 484708, EPI_ISL_498127 to 498151, EPI_ISL_498628 to 498629, EPI_ISL_515055 to 515081, EPI_ISL_540469 to 540578, EPI_ISL_581575 to 581667, EPI_ISL_582027, EPI_ISL_1425566 to 1425567, EPI_ISL_1492720 to 1492730, EPI_ISL_1492837 to 1492841, EPI_ISL_1492904 to 1492924, EPI_ISL_1533936			
see above	Department of Clinical Microbiology	GIGA Medical Genomics	Artesi Maria; Axelle Chaslain; Bontems Sébastien; Boreux Raphaël; Bouchra Boujemla; Bours Vincent.; Cecile Meex; Celine Fombellida-Lopez; Cécile Meex; Céline Fombellida-Lopez; Durkin Keith; Hayette Marie-Pierre; Keith Durkin; Maria Artesi; Marie-Pierre Hayette; Meex Cécile; Melin Pierrette; Nathalie Renotte; Pierrette Melin; Raphael Boreux; Raphaël Boreux; Sebastien Bontems; Sébastien Bontems; Vincent Bours; Vincent Bours.
EPI_ISL_429262 to 429318, EPI_ISL_429320 to 429332, EPI_ISL_451988 to 452100	Department of Clinical Microbiology, Copenhagen University Hospital, Hvidovre, Kettegaard Alle 30, 2650 Hvidovre.	Albertsen lab, Department of Chemistry and Bioscience, Aalborg University, Denmark	Rasmus Kirkegaard
EPI_ISL_417183 to 417184, EPI_ISL_417187 to 417188, EPI_ISL_417190, EPI_ISL_417193, EPI_ISL_417195, EPI_ISL_417197, EPI_ISL_417199, EPI_ISL_418815, EPI_ISL_419213 to 419216, EPI_ISL_419218 to 419219, EPI_ISL_419224 to 419229, EPI_ISL_419232, EPI_ISL_419241, EPI_ISL_419243 to 419252, EPI_ISL_420455			
see above	Department of Clinical Pathology, Pamela Youde Nethersole Eastern Hospital	Department of Health Technology and Informatics, Faculty of Health and Social Science, The Hong Kong Polytechnic University	Alan Ka-Lun WU; Alex Yat-Man HO; Barry Kin-Chung WONG; Chong-Yee YAU; David Ho-Keung SHUM; Eugene Yuk-Keung TSO; Gilman Kit-Hang SIU; Hiu-Yin LAO; Kam-Tong YIP; Kam-Tong Yip; Kenneth Siu-Sing LEUNG; Kingsley King-Gee TAM; Kit-Man SIN; Kitty Sau-Chun FUNG; Kwok-Cheung LUNG; Lam-Kwong LEE; Man-Chun CHAN; Ming-Pan CHOI; Miranda Chong-Yee YAU; Raymond Wai-To LIU; Sandy Ka-Yee CHAU; Shea Ping YIP; Tak-Lun QUE; Tak-Lun Que; Timothy Ting-Leung NG; Wai-Shing LEUNG; Wing Cheong YAM; Wing-Kin TO; Yuk-Yung NG
EPI_ISL_419242	Department of Clinical Pathology, Tuen Mun Hospital	Department of Health Technology and Informatics, Faculty of Health and Social Science, The Hong Kong Polytechnic University	Alan Ka-Lun WU; Alex Yat-Man HO; Barry Kin-Chung WONG; David Ho-Keung SHUM; Eugene Yuk-Keung TSO; Gilman Kit-Hang SIU; Hiu-Yin LAO; Kam-Tong YIP; Kenneth Siu-Sing LEUNG; Kingsley King-Gee TAM; Kit-Man SIN; Kitty Sau-Chun FUNG; Kwok-Cheung LUNG; Lam-Kwong LEE; Man-Chun CHAN; Ming-Pan CHOI; Miranda Chong-Yee YAU; Raymond Wai-To LIU; Sandy Ka-Yee CHAU; Shea Ping YIP; Tak-Lun QUE; Timothy Ting-Leung NG; Wai-Shing LEUNG; Wing Cheong YAM; Wing-Kin TO; Yuk-Yung NG
EPI_ISL_419231	Department of Clinical Pathology, Tuen Mun Hospital, 23 Tsing Chung Koon Road, Tuen Mun, N.T.	Department of Health Technology and Informatics, Faculty of Health and Social Science, The Hong Kong Polytechnic University	Alan Ka-Lun WU; Alex Yat-Man HO; Barry Kin-Chung WONG; David Ho-Keung SHUM; Eugene Yuk-Keung TSO; Gilman Kit-Hang SIU; Hiu-Yin LAO; Kam-Tong YIP; Kenneth Siu-Sing LEUNG; Kingsley King-Gee TAM; Kit-Man SIN; Kitty Sau-Chun FUNG; Kwok-Cheung LUNG; Lam-Kwong LEE; Man-Chun CHAN; Ming-Pan CHOI; Miranda Chong-Yee YAU; Raymond Wai-To LIU; Sandy Ka-Yee CHAU; Shea Ping YIP; Tak-Lun QUE; Timothy Ting-Leung NG; Wai-Shing LEUNG; Wing Cheong YAM; Wing-Kin TO; Yuk-Yung NG
EPI_ISL_481251 to 481252, EPI_ISL_481254 to 481255, EPI_ISL_481257 to 481261, EPI_ISL_481263	Department of Emerging Infectious Diseases, Institute of Tropical Medicine, Nagasaki University	Department of Emerging Infectious Diseases, Institute of Tropical Medicine, Nagasaki University	Haruka Abe; Jiro Yasuda; Rokusuke Yoshikawa; Yuichiro Furusato
EPI_ISL_507206 to 507215	Department of Experimental Modeling and Pathogenesis of Infectious Diseases	WHO National Influenza Centre Russian Federation	Andrey Komissarov; Anna Ivanova; Artem Fadeev; Daria Danilenko; Mariia Sergeeva
EPI_ISL_487276	Department of Food Safety, Nutrition and Veterinary public health, Istituto Superiore di Sanita'	Department of Biomedical, Surgical and Dental Sciences and Department of Biomedical Sciences for Health	Anselmi, G.; Basilico, N.; Binda, S.; D'Alessandro, S.; Delbue, S.; Ferrante, P.; Galli, C.; Parapini, S.; Pariani, E.; Primache, V.; Signorini, L.
EPI_ISL_1538415 to 1538417, EPI_ISL_1538432, EPI_ISL_1541553, EPI_ISL_1542073, EPI_ISL_1542918, EPI_ISL_1545269 to 1545270, EPI_ISL_1546389, EPI_ISL_1547360, EPI_ISL_1547365, EPI_ISL_1547371 to 1547372, EPI_ISL_1547828, EPI_ISL_1548044, EPI_ISL_1548059, EPI_ISL_1548072, EPI_ISL_1549238, EPI_ISL_1550055, EPI_ISL_1550506, EPI_ISL_1563632 to 1563634			
see above	Department of Genetic Engineering and Biotechnology, Shahjalal University of Science and Technology	Genomic Research Lab, BCSIR	Abu Sayeed Mohammad Mahmud; Ajit Ghosh; Barna Goswami; Eshrar Osman; G. M. Nurnabi Azad Jewel; G. M. Nurnabi Azad Jewel; Iffat Jahan; Md. Ahasan Habib; Md. Akkas Ali; Md. Asraful Jahan; Md. Fahmid Hossain Bhuiyan; Md. Hammadul Hoque; Md. Kamrul Islam; Md. Murshed Hasan Sarkar; Md. Nazmul Hasan; Md. Saddam Hossain; Md. Salim Khan; Md. Shamsul Haque Prodhan; Mohammad Mohi Uddin; Mohammad Samir Uzzaman; Shahina Akter; Tanjina Akhter Banu
EPI_ISL_610163 to 610226	Department of Health Technology and Informatics, The Hong Kong Polytechnic University	Department of Health Technology and Informatics, The Hong Kong Polytechnic University	A.K.-L.; A.Y.-M.; B.K.-C.; C.T.-M.; Chan; Chau; D.H.-K.; Fung; G.K.-H.; H.-Y.; Ho; J.S.-L.; K.-T.; K.K.-G.; K.S.-C.; K.S.-S.; L.-K.; Lai; Lao; Lee; Leung; Luk, K.; M.C.-Y.; Ng; Que; S.K.-Y.; S.P.; Shum; Siu; T.-L.; T.T.-L.; Tam; To; W.-K.; W.C.; Wong; Wu; Y.W.-M.; Yam; Yau; Yip
EPI_ISL_1472376 to 1472377	Department of Health Technology and Informatics, The Hong Kong Polytechnic University	Department of Health Technology and Informatics, The Hong Kong Polytechnic University	A.K.-L.; C.T.-M.; Chan; D.S.-H.; G.K.-H.; H.-Y.; J.S.-L.; K.K.-G.; K.S.-S.; L.-K.; Lao; Lee; Leung; Lo; M.C.-Y. and Lai; Ng; Siu; T.T.-L.; Tam; W.-H.; Wong; Wu; Y.W.-M.; Yau
EPI_ISL_417444	Department of Healthcare Biotechnology, National University of Sciences and Technology (NUST)	Department of Healthcare Biotechnology, National University of Sciences and Technology (NUST)	Corman; Ghani, E.; H.A.; Janjua; Javed, A.; Niazi; S.K.; Saqib, M.; V.M. and Zohaib, A.
EPI_ISL_1447102, EPI_ISL_1447174, EPI_ISL_1447182	Department of Homeless Services	New York City Public Health Laboratory	Jade Wang; et al.
EPI_ISL_529151 to 529154, EPI_ISL_529162 to 529166	Department of Immunology, The Scripps Research Institute	Andersen lab at Scripps Research	Allison Smither; Antoinette Bell; Arnaud Drouin; Austin; B. with SEARCH Alliance San Diego; Basler, T.; Dahlene Fusco; Gilberto Sabino-Santos; I. with SEARCH Alliance San Diego; Kaylynn Genemaras; Lilia Melnik; Mchardy; Patricia Snarski; Quigley, M.; Robert Garry with SEARCH Alliance San Diego; Shephard, J.; Stefanski, E.
EPI_ISL_524435 to 524436	Department of Immunology, The Scripps Research Institute	Department of Immunology, The Scripps Research Institute	Bell, A.; Drouin, A.; Fusco, D.; Garry, R.; Genemaras, K.; J.H.; Melnik, L.; Pride, D.; S.D.; SEARCH Alliance; Sabino-Santos, G.; Shin; Smither, A.; Snarski, P.
EPI_ISL_522407 to 522409	Department of Infection Prevention and Infectious Diseases, University Hospital Regensburg	Department of Infection Prevention and Infectious Diseases, University Hospital Regensburg	Fritsch, J.; Holzmann, T.; Schneider-Brachert, W.
EPI_ISL_513298 to 513307	Department of Infection Prevention and Infectious Diseases, University Hospital Regensburg	University Hospital Regensburg	Fritsch, J.; Holzmann, T.; Schneider-Brachert, W.
EPI_ISL_605929 to 605930	Department of Infectious Disease Prevention and Control, Henan Provincial Center for Disease Control and Prevention	Department of Infectious Disease Prevention and Control, Henan Provincial Center for Disease Control and Prevention	Guo, W.; Hu, X.; Huang, X.; Li, D.; Li, X.; Lu, S.; Wu, B.; Ye, Y.
EPI_ISL_568556 to 568576, EPI_ISL_1447321 to 1447328	Department of Infectious Diseases and Immunology, National Hospital Organization Nagoya Medical Center	Clinical Research Center, National Hospital Organization Nagoya Medical Center	Hirotaka Ode; Kazuhiro Matsuoka; Mai Kubota; Masakazu Matsuda; Mayumi Imahashi; Miho Nakasuji; Mikiko Mori; Nakasuji Miho; Yasumasa Iwatani; Yoshihiro Nakata; Yoshiyuki Yokomaku
EPI_ISL_524474, EPI_ISL_524480 to 524481	Department of Infectious Diseases, Cantonal Hospital Baden	Institute of Medical Virology, University of Zurich	Alexandra Trkola; Andrea Zbinden; Fiona Steiner; Gabriela Ziltener; Jon Huder; Jürg Böni; Maryam Zaheri; Michael Huber; Patrick Redli; Riccarda Capaul; Stefan Schmutz; Verena Kufner
EPI_ISL_457699 to 457700, EPI_ISL_457721, EPI_ISL_457724, EPI_ISL_457728, EPI_ISL_457732, EPI_ISL_457736, EPI_ISL_457749			

see above	Department of Infectious Diseases, Istituto Superiore di Sanità, Roma , Italy	Army Medical and Veterinary Research Center	Alessandra Lo Presti; Anna Anselmo; Antonella Fortunato; Antonella Marchi; Concetta Fabiani Silvia Fillo; Concetta Fabiani Silvia Fillo; Eleonora Benedetti; Florigio Lista; Francesco Giordani; Giovanni Faggioni; Nino D'Amore; Paola Stefanelli; Riccardo De Sanctis; Stefano Fiore; Vanessa Vera Fain
EPI_ISL_412973	Department of Infectious Diseases, Istituto Superiore di Sanità, Roma , Italy	Virology Laboratory, Scientific Department, Army Medical Center	Andrea Ciammaruconi; Anna Anselmo; Antonella Fortunato; Antonella Marchi; Concetta Fabiani; Eleonora Benedetti; Florigio Lista; Giovanni Faggioni; Paola Stefanelli; Riccardo De Santis; Silvia Fillo; Stefano Fiore; Stefano Palomba
EPI_ISL_412974	Department of Infectious Diseases, Istituto Superiore di Sanità, Rome, Italy	Virology Laboratory, Scientific Department, Army Medical Center	Andrea Ciammaruconi; Antonella Fortunato; Antonella Marchi; Concetta Fabiani; Eleonora Benedetti; Filippo Molinari; Florigio Lista; Giancarlo Petralito; Giovanni Faggioni; Paola Stefanelli; Riccardo De Santis; Silvia Fillo; Stefano Fiore
EPI_ISL_1425080 to 1425540	Department of Infectious Diseases, Kobe Institute of Health	Department of Infectious Diseases, Kobe Institute of Health	Kentaro Itokawa; Makoto Kuroda; Masanori Hashino; Noriko Nakanishi; Rina Tanaka; Ryohei Nomoto; Tomotada Iwamoto; Tsuyoshi Sekizuka
EPI_ISL_479821 to 479822, EPI_ISL_479855 to 479861, EPI_ISL_479868, EPI_ISL_479986 to 479989, EPI_ISL_480090 to 480102, EPI_ISL_480205 to 480207, EPI_ISL_480209 to 480220, EPI_ISL_1429588			
see above	Department of Infectious Diseases, Kobe Institute of Health	Pathogen Genomics Center, National Institute of Infectious Diseases	Hajime Kamiya; Kentaro Itokawa; Makoto Kuroda; Masanori Hashino; Motoi Suzuki; Rina Tanaka; Ryohei Nomoto; Tsuyoshi Sekizuka
EPI_ISL_411218 to 411220	Department of Infectious and Tropical Diseases, Bichat Claude Bernard Hospital, Paris	Laboratoire Virpath, CIRI U111, UCBL1, INSERM, CNRS, ENS Lyon	Alexandre Gaymard; Aurélien Traversier; Bruno Lina; Catherine Legras-Lachuer; Julien Fouret; Manuel Rosa-Calatrava; Olivier Terrier; Xavier Lescure; Yazdan Yazdanpanah
EPI_ISL_406596 to 406597, EPI_ISL_408430, EPI_ISL_410720, EPI_ISL_410984	Department of Infectious and Tropical Diseases, Bichat Claude Bernard Hospital, Paris	National Reference Center for Viruses of Respiratory Infections, Institut Pasteur, Paris	Angela Brisebarre; Flora Donati; Marion Barbet; Maud Vanpeene; Mélanie Albert; Méline Bizard; Sylvie Behillil; Sylvie van der Werf; Vincent Enouf; Xavier Lescure; Xavier Lescure.; Yazdan Yazdanpanah
EPI_ISL_485398	Department of Internal Medicine, College of Medicine, Chosun University	Department of Internal Medicine, College of Medicine, Chosun University	D.-M.; Kim
EPI_ISL_413019 to 413020	Department of Internal Medicine, Triemli Hospital	Institute of Medical Virology, University of Zurich	Alexandra Trkola; Andrea Zbinden; Fiona Steiner; Gerhard Eich; Jon Huder; Jürg Böni; Maryam Zaheri; Michael Huber; Patrick Redli; Riccarda Capaul; Stefan Schmutz; Verena Kufner
EPI_ISL_476805 to 476812, EPI_ISL_476815	Department of Laboratory Medicine Tan Tock Seng Hospital	Department of Laboratory Medicine Tan Tock Seng Hospital	Barkham TMS; Chen YYC; Li C; Maurer-Stroh S; Nagarajan N; Sessions OM; Tang WY; Zair X
EPI_ISL_477172, EPI_ISL_477174 to 477175, EPI_ISL_477177 to 477178, EPI_ISL_477180, EPI_ISL_477182, EPI_ISL_477184, EPI_ISL_477187 to 477192, EPI_ISL_479482 to 479492, EPI_ISL_492978 to 492979			
see above	Department of Laboratory Medicine Tan Tock Seng Hospital	Department of Laboratory Medicine Tan Tock Seng Hospital	Barkham TMS; Chen YYC; Li C; Maurer-Stroh S; Nagarajan N; Sessions OM; Tang WY; Zair X
EPI_ISL_516801 to 516805	Department of Laboratory Medicine, Tan Tock Seng Hospital	Department of Laboratory Medicine, Tan Tock Seng Hospital	Barkham TMS; Chen YYC; Li C; Maurer-Stroh S; Nagarajan N; Sessions OM; Tang WY; Zair X
EPI_ISL_1509003 to 1509094	Department of Laboratory Medicine, Clinical Center, National Institutes of Health	Laboratory of Parasitic Diseases, Systems Genomics Section, National Institute of Allergy and Infectious Diseases, National Institutes of Health	Allison Roder; Elodie Ghedin; Heike Bailin; Jennifer Kwan; Jessica McCormick-Ell; Jung-ho Youn; Matthew Chung; Michael Bell; Rachel Mercado; Sanchita Das; Stephanie Banakis; Tara Palmore; Wei Wang
EPI_ISL_1495154 to 1495165, EPI_ISL_1495286 to 1495353, EPI_ISL_1495466 to 1495527, EPI_ISL_1495810 to 1495866	Department of Laboratory Medicine, Division of Clinical Virology, University of Medicine, Vienna	Bergthaler laboratory, CeMM Research Center for Molecular Medicine of the Austrian Academy of Sciences	Andreas Bergthaler; Anna Schedl; Bekir Erguner; Benedikt Agerer; Christoph Bock; Fabian Amman; Jan Laine; Lukas Endler; Maelle Le Moing; Martin Senekowitsch; Michael Schuster; Petr Triska; Thomas Penz
EPI_ISL_408489, EPI_ISL_410218, EPI_ISL_413592, EPI_ISL_422407 to 422422, EPI_ISL_447614 to 447622, EPI_ISL_463007, EPI_ISL_534336			
see above	Department of Laboratory Medicine, National Taiwan University Hospital	Microbial Genomics Core Lab, National Taiwan University Centers of Genomic and Precision Medicine	Chiao-Ling Li; Pei-Jer Chen; Shan-Chwen Chang; Shiou-Hwei Yeh; Sui-Yuan Chang; Ya-Yun Lai; You-Yu Lin
EPI_ISL_476795 to 476797, EPI_ISL_476813 to 476814, EPI_ISL_476816 to 476821, EPI_ISL_507002 to 507006, EPI_ISL_538435 to 538497	Department of Laboratory Medicine, Tan Tock Seng Hospital	Department of Laboratory Medicine, Tan Tock Seng Hospital	Barkham TMS; Chen YYC; Li C; Lim JX; Maurer-Stroh S; Nagarajan N; Sessions OM; Tang WY; Zair X
EPI_ISL_477170	Department of Laboratory, Medicine Tan Tock Seng Hospital	Department of Laboratory Medicine Tan Tock Seng Hospital	Barkham TMS; Chen YYC; Li C; Maurer-Stroh S; Nagarajan N; Sessions OM; Tang WY; Zair X
EPI_ISL_477171	Department of Laboratory Medicine Tan Tock Seng Hospital	Department of Laboratory, Medicine Tan Tock Seng Hospital	Barkham TMS; Chen YYC; Li C; Maurer-Stroh S; Nagarajan N; Sessions OM; Tang WY; Zair X
EPI_ISL_591012, EPI_ISL_591018 to 591019, EPI_ISL_1385962, EPI_ISL_1385965, EPI_ISL_1385968, EPI_ISL_1385991, EPI_ISL_1490377, EPI_ISL_1547612, EPI_ISL_1547618, EPI_ISL_1547623, EPI_ISL_1547633			
see above	Department of Medical Microbiology - section Molde, Molde Hospital	Norwegian Institute of Public Health, Department of Virology	Atiya R Ali; Debech Nadia; Engebretsen Serina Beate; Garcia Llorente Ignacio; Hilde Elshaug; Hilde Vollan; Jon Bråte; Kamilla Heddeland Instefjord; Karoline Bragstad; Kathrine Stene-Johansen; Marie Paulsen Madsen; Olav Hungnes; Pedersen Benedikte Nevjen; Rasmus Riis Kopperud
EPI_ISL_454416	Department of Medical Microbiology, Leiden University Medical Center	Department of Medical Microbiology, Leiden University Medical Center	Dalebout; E.J.; J.C.; J.J. and Sidorov, I.; N.S.; Ogando; Snijder; T.J.; Zevenhoven; de Vries
EPI_ISL_591007, EPI_ISL_591014, EPI_ISL_1490457, EPI_ISL_1490461, EPI_ISL_1547563, EPI_ISL_1547567 to 1547569	Department of Medical Microbiology, St. Olavs hospital	Norwegian Institute of Public Health, Department of Virology	Atiya R Ali; Debech Nadia; Engebretsen Serina Beate; Garcia Llorente Ignacio; Hilde Elshaug; Hilde Vollan; Jon Bråte; Kamilla Heddeland Instefjord; Karoline Bragstad; Kathrine Stene-Johansen; Marie Paulsen Madsen; Olav Hungnes; Pedersen Benedikte Nevjen; Rasmus Riis Kopperud
EPI_ISL_417917	Department of Medical Microbiology, University Malaya Medical Centre	Department of Medical Microbiology	Adeeba KAMARULZAMAN; Chee Kuan WONG; Cindy Shuan Ju TEH; I-Ching SAM; Sasheela PONNAMPALAVANAR; Sharifah Faridah SYED OMAR; University Malaya Medical Centre COVID Team; Vijayan MUNUSAMY; Yoke Fun Chan; Yoong Min CHONG
EPI_ISL_417918 to 417920, EPI_ISL_501176 to 501228, EPI_ISL_506996 to 507000	Department of Medical Microbiology, University Malaya Medical Centre	Department of Medical Microbiology, Faculty of Medicine, University of Malaya	Adeeba KAMARULZAMAN; Chee Kuan WONG; Cindy Shuan Ju TEH; Fadhil Hadi JAMALUDDIN; Han Ming GAN; I-Ching SAM; Jennifer Chong; Sasheela PONNAMPALAVANAR; Sharifah Faridah SYED OMAR; University Malaya Medical Centre COVID Team; Vijayan MUNUSAMY; Yoke Fun CHAN; Yoke Fun Chan; Yoong Min CHONG
EPI_ISL_488840, EPI_ISL_488845, EPI_ISL_488847, EPI_ISL_488853 to 488856, EPI_ISL_488861, EPI_ISL_488863 to 488864, EPI_ISL_488872, EPI_ISL_492836, EPI_ISL_492838 to 492839, EPI_ISL_492841 to 492842, EPI_ISL_492848 to 492849, EPI_ISL_492853 to 492855, EPI_ISL_492857 to 492861, EPI_ISL_492866 to 492867, EPI_ISL_492870 to 492871, EPI_ISL_492873, EPI_ISL_492875, EPI_ISL_492878, EPI_ISL_492881, EPI_ISL_492884, EPI_ISL_492886, EPI_ISL_492889, EPI_ISL_492891 to 492893, EPI_ISL_492895 to 492900, EPI_ISL_492903 to 492904, EPI_ISL_492907, EPI_ISL_492909 to 492910, EPI_ISL_492912 to 492913, EPI_ISL_492915, EPI_ISL_495081 to 495096, EPI_ISL_501557, EPI_ISL_501562, EPI_ISL_501602, EPI_ISL_501613			
see above	Department of Medical Microbiology, Western Sussex Hospitals NHS Foundation Trust, St Richard's Hospital	Wellcome Sanger Institute for the COVID-19 Genomics UK (COG-UK) consortium	Cordelia Langford; David K. Jackson; Dominic Kwiatkowski; Ewan Harrison; Ian Johnston; John Sillitoe on behalf of the Wellcome Sanger Institute COVID-19 Surveillance Team (http://www.sanger.ac.uk/covid-team); Jonathan Lewis; Manasa Mutingwende; Michelle Erkiert; Olga Podplomyk; Paul Randell and Alex Alderton; Roberto Amato; Sarah Lowdon; Sonia Goncalves
EPI_ISL_512844	Department of Medical Research	DMR_Myanmar	Aung Kyaw Kyaw; Aung Zaw Latt; Hlaing Myat Thu; Hnin Ohnmar Soe; Htin Lin; Kay Thi Aye; Lai Lai San; Myat Htut Nyunt; Nan Aye Thida Oo; Ni Ni Zaw; Phyu Win Ei; Su Mon Win; Theingi Win Myat; Wah Wah Aung; Yi Yi Kyaw; Zaw Than Htun
EPI_ISL_454733	Department of Medical, Biotechnologies University of Siena	Department of Medical, Biotechnologies University of Siena	Anichini, G.; Cusi; G. and Santoro, F.; Gandolfo, C.; M.G.; Pinzauti, D.; Pozzi
EPI_ISL_528809 to 528822	Department of Medicine, Gandhi hospital, Hyderabad	CSIR-Centre for Cellular and Molecular Biology	Ajay Sarawagi; Archana Bharadwaj Siva; Dhiviya Vedagiri; Divya Gupta; Divya Tej Sowpati; G. Aditya Kumar; Gangumala Srinivas Reddy; Karthik Bharadwaj Tallapaka; Koushick Sivakumar; Krishnan Harinivas Harshan; Lamuk Zaveri; M Soujanya Reddy; Namami Gaur; Nikhil Hajirnis; Onkar Kulkarni; Payel Mukherjee; Pooja Ramesh Gupta; Pratheusa Maccha; Priya Singh; Priyanka Pant; Purushotham Vodnala; Rajan Kumar Jha; Rajarao Mesipogu; Rajkanwar Nathawat; Rakesh K Mishra; Renu Sudhakar; Sakshi Shambhavi; Santosh Kumar Kuncha; Shagufta Khan; Shraddha Vijay Lahoti; Sofia Banu; Somesh Gorde; Sujoy Deb; Swati Bayyana; Thrilok Chander Bingi; Tulasi Nagabandi; Umesh Kumar; Unis Ahmad Bhat; Vinayasekhar Aedula; Vishal Sah
EPI_ISL_437438 to 437444, EPI_ISL_475030 to 475046, EPI_ISL_476869 to 476882, EPI_ISL_477183, EPI_ISL_495070 to 495080, EPI_ISL_500947 to 500950, EPI_ISL_512070 to 512071			
see above	Department of MicroBiology, Government Medical College, Surat	Gujarat Biotechnology Research Centre	A M Kadri; Afzal Ansari; Akanksha Verma; Amit Kanani; Amit gamit; Anjali Rajwar; Ankit Hinsu; Apurvasinh Puvar; Armi Chaudhari; Bhavesh Modi; Bhavya Jindal; Binita Aring; Chaitanya Joshi; Dinesh Kumar; Dipa Kinariwala; Dipeshwari Shewale; Disha Patel; Fenil Patel; Gaurishankar Shrimali; Geeta Vaghela; Harsh Bakshi; Janvi Raval; Kairavi Joshi; Kamlesh J Upadhyay; Komal Patel; Labdhi Pandya; Madhvi Joshi; Maharshi Pandya; Monika Gandhi; Naresh Chauhan; Neelam Nathani; Neeta Khandelwal; Neha Rajpara; Nidhi Patel; Nidhi Sood; Nikha Trivedi; Nitin Savaliya; Pinal Trivedi; Pooja P Doshi; Pragya Sharma; Pranay Shah; Pritesh Sabara; Priti Pandita; Priyanka P Vatsa; R D Dixit; Raghawendra Kumar; Ramesh Pandit; Sharmistha Majumdar; Snehal Bagatharia; Sonia Barve; Summaiya Mullan; Tejas Shah; Zarna Patel; Zuber Saiyed

EPI_ISL_431102	Department of MicroBiology,Gandhi Medical College and Hospital,Secendrabad,Hyderabad,India	Department of Microbiology, Gandhi Medical College and Hospital, Secendrabad, Hyderabad	Amit A. Upadhyay; Anand Kumar K; Kalyani Putty; Muttineni Radhakrishna; Nagamani K; Pankaj Singh D; Raja Rao M; Rama Amara; Ravikumar P; Steven E. Bosinger; Sunitha P; Thrilok Chander B
EPI_ISL_416314 to 416315	Department of Microbiology, Faculty of Medicine, The Chinese University of Hong Kong, Hong Kong SAR, China	Department of Microbiology, Faculty of Medicine, Chinese University of Hong Kong, Hong Kong SAR, China	Paul KS Chan; Zigui Chen
EPI_ISL_431103, EPI_ISL_431117	Department of Microbiology, Gandhi Medical College and Hospital, Secendrabad, Hyderabad, India	Department of Microbiology, Gandhi Medical College and Hospital, Secendrabad, Hyderabad, India	Amit A. Upadhyay; Anand Kumar K; Kalyani Putty; Muttineni Radhakrishna; Nagamani K; Pankaj Singh D; Raja Rao M; Rama Amara; Ravikumar P; Steven E. Bosinger; Steven E.Bosinger; Sunitha P; Thrilok Chander B
EPI_ISL_483850 to 483879	Department of Microbiology, Government Medical College, Surat	Gujarat Biotechnology Research Centre	A M Kadri; Afzal Ansari; Amit gamit; Apurvasinh Puvar; Chaitanya Joshi; Dinesh Kumar; Harsh Bakshi; Janvi Raval; Komal Patel; Labdhi Pandya; Madhvi Joshi; Maharshi Pandya; Monika Gandhi; Naresh Chauhan; Nidhi Patel; Nikha Trivedi; Nitin Savaliya; Pinal Trivedi; R D Dixit; Raghawendra Kumar; Summaiya Mullan; Zarna Patel; Zuber Saiyed
EPI_ISL_413017 to 413018, EPI_ISL_413514, EPI_ISL_413516	Department of Microbiology, Institute for Viral Diseases, College of Medicine, Korea University	Department of Microbiology, Institute for Viral Diseases, College of Medicine, Korea University	Changmin Kang; Cui Chunguang; Dong Min Kim; Gee Eun Lee; Gee eun Lee; Hee Jin Cheong; Heedo Park; Jeonghun Kim; Ji Yun Noh; Jin Gu Yoon; Jin Il Kim; Joon Young Song; Joon-Yong Bae; Jungmin Lee; Juyoung Cho; Kyeong-ryeol Shin; Man-Seong Park; Woo Joo Kim
EPI_ISL_420531 to 420534, EPI_ISL_420536 to 420539	Department of Microbiology, PathWest QEII Medical Centre	Department of Microbiology, PathWest QEII Medical Centre	Avram Levy; Chisha Sikazwe; David Speers and David Smith; Jurissa Lang
EPI_ISL_497768 to 497870, EPI_ISL_498269 to 498271, EPI_ISL_510538 to 510540, EPI_ISL_516798 to 516799	Department of Microbiology, The University of Hong Kong	Department of Microbiology, The University of Hong Kong	Kelvin K.W. To; Kwok-Yung Yuen
EPI_ISL_1540501 to 1540533, EPI_ISL_1544108	Department of Microbiology, University Hospital Motol	Department of Microbiology, University Hospital Motol	Ales Briksi; Katerina Chuda; Katerina Polackova; Klara Krivankova; Miroslav Zajac; Ondrej Cinek; Pavel Drevinek; Petr Hubacek
EPI_ISL_1495362 to 1495383, EPI_ISL_1495602 to 1495625, EPI_ISL_1495894 to 1495919	Department of Microbiology, University Innsbruck	Bergthaler laboratory, CeMM Research Center for Molecular Medicine of the Austrian Academy of Sciences	Andreas Bergthaler; Anna Schedl; Bekir Erguner; Benedikt Agerer; Christoph Bock; Fabian Amman; Jan Laine; Lukas Endler; Maelle Le Moing; Martin Senekowitsch; Michael Schuster; Petr Triska; Thomas Penz
EPI_ISL_438138	Department of Microbiology,Gandhi Medical College and Hospital	Department of Microbiology, Gandhi Medical College and Hospital Secendrabad, Hyderbad, India	Amit A. Upadhyay; Anand Kumar K; Kalyani Putty; Muttineni Radhakrishna; Nagamani K; Pankaj Singh D; Raja Rao Mesipogu; Rama Amara; Ravikumar P; Steven Bosinger; Sunitha P; Thrilok Chander B
EPI_ISL_437626	Department of Microbiology,Gandhi Medical College and Hospital	Department of Veterinary Biotechnalogy, College of Veterinary Science, Rajendranagar, PV Narsimha Rao Telengana Veterinary University	Amit A. Upadhyay; Anand Kumar K; Kalyani Putty; Muttineni Radhakrishna; Nagamani K; Pankaj Singh D; Raja Rao M; Rama Amara; Ravikumar P; Steven Bosinger; Sunitha P; Thrilok Chander B
EPI_ISL_431101	Department of Microbiology,Gandhi Medical College and Hospital	Virus Research Laboratory, Department of Zoology, Osmania University,Hyderabad,India	Amit A. Upadhyay Steven E. Bosinger; Anand Kumar K; Kalyani Putty; Muttineni Radhakrishna; Nagamani K; Pankaj Singh D; Raja Rao M; Rama Amara; Ravikumar P; Sunitha P; Thrilok Chander B
EPI_ISL_438139	Department of Microbiology,Gandhi Medical College and Hospital,Hyderabad	Virus Research Laboratory, Department of Zoology, Osmania University, Hyderabad, India	Amit A. Upadhyay; Anand Kumar K; Kalyani Putty; Muttineni Radhakrishna; Nagamani K; Pankaj Singh D; Raja Rao M; Rama Amara; Ravikumar P; Steven Bosinger; Sunitha P; Thrilok Chander B
EPI_ISL_463741 to 463748	Department of Molecular Virology, Cyprus Institute of Neurology and Genetics	Department of Molecular Virology, Cyprus Institute of Neurology and Genetics	Christina Christodoulou; Christina Tryfonos; Dana Koptides; George Krashias; Jan Richter; Stavros Bashiardes
EPI_ISL_1472367 to 1472371	Department of Pathology, National Institute of Infectious Diseases	Department of Pathology, National Institute of Infectious Diseases	Harutaka Katano; Kentaro Itokawa; Makoto Kuroda; Masanori Hashino; Rina Tanaka; Tadaki Suzuki; Tsuyoshi Sekizuka
EPI_ISL_417176 to 417179, EPI_ISL_419217, EPI_ISL_419222 to 419223	Department of Pathology, Princess Margaret Hospital	Department of Health Technology and Informatics, Faculty of Health and Social Science, The Hong Kong Polytechnic University	Alan Ka-Lun WU; Alex Yat-Man HO; Barry Kin-Chung WONG; David Ho-Keung SHUM; Eugene Yuk-Keung TSO; Gilman Kit-Hang SIU; Hiu-Yin LAO; Kam-Tong YIP; Kam-Tong Yip; Kenneth Siu-Sing LEUNG; Kingsley King-Gee TAM; Kit-Man SIN; Kitty Sau-Chun FUNG; Kwok-Cheung LUNG; Lam-Kwong LEE; Man-Chun CHAN; Ming-Pan CHOI; Miranda Chong-Yee YAU; Raymond Wai-To LIU; Sandy Ka-Yee CHAU; Shea Ping YIP; Tak-Lun QUE; Tak-Lun Que; Timothy Ting-Leung NG; Wai-Shing LEUNG; Wing Cheong YAM; Wing-Kin TO; Yuk-Yung NG
EPI_ISL_594185 to 594188, EPI_ISL_596451 to 596452, EPI_ISL_596455	Department of Pathology, School of Medicine, Imam Khomeini Hospital, Tehran University of Medical Sciences	Genetics Research Center, University of Social Welfare and Rehabilitation Sciences	Ali Jafarpour; Alireza Abdollahi; Azam Ghaziasad; Azam Ghaziasadi; Azar Hadadi; Hossein Najmabadi; Khadijeh Jalalvand; Kimia Kahrizi; Marzieh Mohseni; Reza Najafipour; Saber Soltani; Seyed Mohammad Jazayeri; Seyedeh elham Mortazavi; Zohreh Fattahi
EPI_ISL_582033	Department of Pathology, School of Medicine, Imam Khomeini Hospital, Tehran University of Medical Sciences	Genetics Research Center. University Of Social Welfare And Rehabilitation Sciences	Ali Jafarpour; Alireza Abdollahi; Azam Ghaziasad; Azam Ghaziasadi; Azar Hadadi; Hossein Najmabadi; Khadijeh Jalalvand; Kimia Kahrizi; Marzieh Mohseni; Reza Najafipour; Saber Soltani; Seyed Mohammad Jazayeri; Seyedeh elham Mortazavi; Zohreh Fattahi
EPI_ISL_413459	Department of Pathology, Toshima Hospital	Pathogen Genomics Center, National Institute of Infectious Diseases	Haruka Nishioka; Harutaka Katano; Ippei Miyamoto; Ja-Mun Chong; Jun Yamazaki; Kentaro Itokawa; Makoto Kuroda; Masahiro Sano; Minoru Tobiume; Noriko Nakajima; Tadaki Suzuki; Takuya Adachi; Tsuyoshi Sekizuka; Yuko Sato
EPI_ISL_417180 to 417182, EPI_ISL_417185, EPI_ISL_419221, EPI_ISL_419253	Department of Pathology, United Christian Hospital	Department of Health Technology and Informatics, Faculty of Health and Social Science, The Hong Kong Polytechnic University	Alan Ka-Lun WU; Alex Yat-Man HO; Barry Kin-Chung WONG; David Ho-Keung SHUM; Eugene Yuk-Keung TSO; Gilman Kit-Hang SIU; Hiu-Yin LAO; Kam-Tong YIP; Kam-Tong Yip; Kenneth Siu-Sing LEUNG; Kingsley King-Gee TAM; Kit-Man SIN; Kitty Sau-Chun FUNG; Kwok-Cheung LUNG; Lam-Kwong LEE; Man-Chun CHAN; Ming-Pan CHOI; Miranda Chong-Yee YAU; Raymond Wai-To LIU; Sandy Ka-Yee CHAU; Shea Ping YIP; Tak-Lun QUE; Tak-Lun Que; Timothy Ting-Leung NG; Wai-Shing LEUNG; Wing Cheong YAM; Wing-Kin TO; Yuk-Yung NG
EPI_ISL_425231 to 425257, EPI_ISL_425260, EPI_ISL_425262 to 425273, EPI_ISL_425275 to 425279, EPI_ISL_425281 to 425345, EPI_ISL_425347 to 425371, EPI_ISL_425373 to 425385, EPI_ISL_425387 to 425404, EPI_ISL_425406 to 425409, EPI_ISL_425411 to 425418, EPI_ISL_425420 to 425421, EPI_ISL_425423 to 425438, EPI_ISL_425441 to 425461, EPI_ISL_433466 to 433487, EPI_ISL_433489 to 433497, EPI_ISL_433666 to 433686, EPI_ISL_433688 to 433707, EPI_ISL_433709 to 433715, EPI_ISL_433717 to 433739, EPI_ISL_433741 to 433746, EPI_ISL_433748 to 433765, EPI_ISL_433767 to 433806, EPI_ISL_433808 to 433837, EPI_ISL_433839 to 433971, EPI_ISL_433974 to 433983, EPI_ISL_433986 to 433997, EPI_ISL_433999 to 434006, EPI_ISL_434008 to 434062, EPI_ISL_438550 to 438571, EPI_ISL_438573 to 438610, EPI_ISL_438612 to 438654, EPI_ISL_438656 to 438687, EPI_ISL_438689 to 438748, EPI_ISL_444313 to 444342, EPI_ISL_444344, EPI_ISL_444346 to 444348, EPI_ISL_444350 to 444354, EPI_ISL_444356 to 444374, EPI_ISL_444376 to 444382, EPI_ISL_444384 to 444408, EPI_ISL_444410 to 444433, EPI_ISL_444435 to 444443, EPI_ISL_444446 to 444453, EPI_ISL_447945 to 447983, EPI_ISL_447987 to 448013, EPI_ISL_448015 to 448029, EPI_ISL_448031 to 448037, EPI_ISL_448039 to 448071, EPI_ISL_448074 to 448084, EPI_ISL_448086 to 448090, EPI_ISL_448092, EPI_ISL_448096 to 448104, EPI_ISL_448106 to 448115, EPI_ISL_452857 to 452901, EPI_ISL_452903 to 452906, EPI_ISL_452910 to 452930, EPI_ISL_452932 to 452949, EPI_ISL_452951 to 452954, EPI_ISL_452956 to 453005, EPI_ISL_456677 to 456682, EPI_ISL_456686, EPI_ISL_456688, EPI_ISL_456690, EPI_ISL_456692, EPI_ISL_456695 to 456706, EPI_ISL_456708 to 456733, EPI_ISL_456735 to 456755, EPI_ISL_461546 to 461563, EPI_ISL_461565 to 461588, EPI_ISL_473451 to 473493, EPI_ISL_473495 to 473507, EPI_ISL_477783 to 477785, EPI_ISL_477787 to 477819, EPI_ISL_483913 to 483915, EPI_ISL_490317 to 490329, EPI_ISL_499853, EPI_ISL_499855, EPI_ISL_499905, EPI_ISL_499908, EPI_ISL_499966, EPI_ISL_499968 to 499973, EPI_ISL_512330 to 512335, EPI_ISL_514443 to 514451, EPI_ISL_516990 to 517000, EPI_ISL_529224 to 529225, EPI_ISL_529236, EPI_ISL_529257, EPI_ISL_529261, EPI_ISL_529265, EPI_ISL_529269, EPI_ISL_529283, EPI_ISL_529299, EPI_ISL_529445, EPI_ISL_529524, EPI_ISL_529656 to 529660, EPI_ISL_549374, EPI_ISL_549386, EPI_ISL_549388 to 549394, EPI_ISL_572471 to 572472, EPI_ISL_572484, EPI_ISL_572519, EPI_ISL_572524, EPI_ISL_572541, EPI_ISL_572592, EPI_ISL_572616, EPI_ISL_572642, EPI_ISL_572779, EPI_ISL_572792, EPI_ISL_572851 to 572852, EPI_ISL_572862 to 572868, EPI_ISL_573138 to 573151, EPI_ISL_573153, EPI_ISL_576897 to 576902, EPI_ISL_576906 to 576919, EPI_ISL_584283, EPI_ISL_584286 to 584291, EPI_ISL_584294 to 584302, EPI_ISL_584304 to 584307, EPI_ISL_584309 to 584314, EPI_ISL_584316 to 584318, EPI_ISL_584321 to 584323, EPI_ISL_611593, EPI_ISL_611751, EPI_ISL_611938, EPI_ISL_611964 to 611967, EPI_ISL_612007, EPI_ISL_612034, EPI_ISL_612099, EPI_ISL_612106, EPI_ISL_612123, EPI_ISL_612186, EPI_ISL_612262 to 612283, EPI_ISL_1386571 to 1386575, EPI_ISL_1386577 to 1386581, EPI_ISL_1386583, EPI_ISL_1386586, EPI_ISL_1386588 to 1386589, EPI_ISL_1474564 to 1474573, EPI_ISL_1545847 to 1545851			
see above	Department of Pathology, University of Cambridge	COVID-19 Genomics UK (COG-UK) Consortium	Aminu S. Jahun; Anna Yakovleva; Charlotte J. Houldcroft; Fahad A Khokhar; Grant Hall; Ian Goodfellow; Iliana Georgana; Laura G Caller; Luke W Meredith; M. Estee Torok; M. Estée Török; M. Est©e Trk; Malte Pinckert; Martin D. Curran; Myra Hosmillo; Rhys Izuagbe; Sarah L. Caddy; Surendra Parmar; Theresa Feltwell; William L. Hamilton; Yasmin Chaudhry
EPI_ISL_458571, EPI_ISL_459173, EPI_ISL_459241, EPI_ISL_459243, EPI_ISL_459258, EPI_ISL_524590, EPI_ISL_524595			
see above	Department of Pathology, University of Cambridge	Wellcome Sanger Institute for the COVID-19 Genomics UK (COG-UK) Consortium	Aminu S. Jahun; Anna Yakovleva; Charlotte J. Houldcroft; Cordelia Langford; David K. Jackson; Dominic Kwiatkowski; Ewan Harrison; Fahad A Khokhar; Grant Hall; Ian Goodfellow; Ian Johnston; John Sillitoe on behalf of the Wellcome Sanger Institute COVID-19 Surveillance Team; Laura G Caller; Luke W Meredith; M. Estée Török; Martin D. Curran; Myra Hosmillo; Roberto Amato; Sarah L. Caddy; Sonia Goncalves; Theresa Feltwell; William L. Hamilton; and Alex Alderton
EPI_ISL_438248 to 438545, EPI_ISL_439368 to 439664, EPI_ISL_439863 to 439952, EPI_ISL_439954 to 439956, EPI_ISL_439958, EPI_ISL_439961, EPI_ISL_439963, EPI_ISL_439968, EPI_ISL_439970, EPI_ISL_439975, EPI_ISL_439977, EPI_ISL_439980, EPI_ISL_439987, EPI_ISL_439989 to 439991, EPI_ISL_439993 to 439997, EPI_ISL_439999, EPI_ISL_440003 to 440004, EPI_ISL_440007, EPI_ISL_440009 to 440012, EPI_ISL_440014 to 440015, EPI_ISL_440017, EPI_ISL_440019, EPI_ISL_440024 to 440025, EPI_ISL_440030, EPI_ISL_440034, EPI_ISL_440040 to 440041, EPI_ISL_440044 to 440049, EPI_ISL_440053, EPI_ISL_440055, EPI_ISL_440057, EPI_ISL_440059 to 440062, EPI_ISL_440064, EPI_ISL_440066 to 440067, EPI_ISL_440069, EPI_ISL_440072 to 440080, EPI_ISL_440082 to 440083, EPI_ISL_440085 to 440086, EPI_ISL_440088 to 440090, EPI_ISL_440096 to 440097, EPI_ISL_440099, EPI_ISL_440104, EPI_ISL_440107 to 440112, EPI_ISL_440117, EPI_ISL_440120 to 440122, EPI_ISL_440125, EPI_ISL_440129, EPI_ISL_440132 to 440133, EPI_ISL_440138, EPI_ISL_440143 to 440144, EPI_ISL_440146 to 440147, EPI_ISL_440150, EPI_ISL_440152, EPI_ISL_440155, EPI_ISL_440159, EPI_ISL_440161, EPI_ISL_440163 to 440164, EPI_ISL_440166, EPI_ISL_440169 to 440171, EPI_ISL_440173, EPI_ISL_440176 to 440177, EPI_ISL_440179 to 440186, EPI_ISL_440188, EPI_ISL_440193, EPI_ISL_440195, EPI_ISL_440197, EPI_ISL_440202 to 440203, EPI_ISL_440207 to 440209, EPI_ISL_440211, EPI_ISL_440213, EPI_ISL_440217, EPI_ISL_440223, EPI_ISL_440226, EPI_ISL_440230 to 440231, EPI_ISL_440234, EPI_ISL_440237, EPI_ISL_440239 to 440240, EPI_ISL_440244, EPI_ISL_440246 to 440247, EPI_ISL_440254, EPI_ISL_440257 to 440261, EPI_ISL_440263 to 440265, EPI_ISL_440267 to 440268, EPI_ISL_440270 to 440271, EPI_ISL_440278 to 440285, EPI_ISL_440287 to 440290, EPI_ISL_440293, EPI_ISL_440295 to 440297, EPI_ISL_440300 to 440622, EPI_ISL_440810 to 440851, EPI_ISL_441052 to 441349, EPI_ISL_441547 to 441658, EPI_ISL_441660 to 441661, EPI_ISL_441664 to 441669, EPI_ISL_441671 to 441674, EPI_ISL_441677 to 441684, EPI_ISL_441686 to 441689, EPI_ISL_441692, EPI_ISL_441695, EPI_ISL_441700 to 441703, EPI_ISL_441705 to 441707, EPI_ISL_441709 to 441711, EPI_ISL_441713, EPI_ISL_441715, EPI_ISL_441718, EPI_ISL_441720 to 441729, EPI_ISL_441731 to 441732, EPI_ISL_441734 to 441736, EPI_ISL_441738 to 441746, EPI_ISL_441748 to 441750, EPI_ISL_441752, EPI_ISL_441754 to 441760, EPI_ISL_441762 to 441764, EPI_ISL_441767 to 441769, EPI_ISL_441772 to 441774, EPI_ISL_441777 to 441780, EPI_ISL_441782 to 441846, EPI_ISL_442045 to 442343, EPI_ISL_442524 to 442623, EPI_ISL_442811 to 443118, EPI_ISL_443318 to 443685, EPI_ISL_458516 to 458570, EPI_ISL_458572 to 458577, EPI_ISL_459166 to 459172, EPI_ISL_459174 to 459240, EPI_ISL_459242, EPI_ISL_459244 to 459257, EPI_ISL_459259 to 459297, EPI_ISL_459304 to 459323, EPI_ISL_459410 to 459504, EPI_ISL_469891 to 469913, EPI_ISL_470090 to 470355, EPI_ISL_470358 to 470528, EPI_ISL_470531 to			

470533, EPI_ISL_470535 to 470538, EPI_ISL_489381 to 489390, EPI_ISL_489394 to 489395, EPI_ISL_489397 to 489400, EPI_ISL_489402 to 489406, EPI_ISL_489409 to 489412, EPI_ISL_489414 to 489417, EPI_ISL_489419 to 489428, EPI_ISL_489430 to 489441, EPI_ISL_489443 to 489448, EPI_ISL_489450 to 489454, EPI_ISL_489456 to 489469, EPI_ISL_489471 to 489479, EPI_ISL_489481 to 489488, EPI_ISL_489490 to 489522, EPI_ISL_489524 to 489529, EPI_ISL_489531 to 489535, EPI_ISL_489538 to 489542, EPI_ISL_489544 to 489546, EPI_ISL_489548 to 489566, EPI_ISL_489568 to 489577, EPI_ISL_492189 to 492194, EPI_ISL_492196, EPI_ISL_492198 to 492201, EPI_ISL_492204 to 492206, EPI_ISL_511959, EPI_ISL_511970, EPI_ISL_511982, EPI_ISL_524493, EPI_ISL_524495, EPI_ISL_524497 to 524498, EPI_ISL_524501 to 524503, EPI_ISL_524505 to 524506, EPI_ISL_524508 to 524509, EPI_ISL_524515, EPI_ISL_524521 to 524522, EPI_ISL_524525, EPI_ISL_524528 to 524531, EPI_ISL_524537 to 524538, EPI_ISL_524541, EPI_ISL_524544, EPI_ISL_524546, EPI_ISL_524548, EPI_ISL_524551, EPI_ISL_524553, EPI_ISL_524558, EPI_ISL_524560, EPI_ISL_524564, EPI_ISL_524568, EPI_ISL_524570, EPI_ISL_524573 to 524589, EPI_ISL_524591 to 524594, EPI_ISL_524596 to 524618, EPI_ISL_591076 to 591082, EPI_ISL_591091 to 591093			
see above	Department of Pathology, University of Cambridge	Wellcome Sanger Institute for the COVID-19 Genomics UK (COG-UK) consortium	Alex Alderton; Aminu S. Jahun; Anna Yakovleva; Charlotte J. Houldcroft; Cordelia Langford; David K. Jackson; Dominic Kwiatkowski; Ewan Harrison; Fahad A Khokhar; Grant Hall; Ian Goodfellow; Ian Johnston; John Sillitoe on behalf of the Wellcome Sanger Institute COVID-19 Surveillance Team; John Sillitoe on behalf of the Wellcome Sanger Institute COVID-19 Surveillance Team (http://www.sanger.ac.uk/covid-team); Laura G Caller; Luke W Meredith; M. Estée Török; Martin D. Curran; Myra Hosmillo; Roberto Amato; Sarah L. Caddy; Sonia Goncalves; Theresa Feltwell; William L. Hamilton; and Alex Alderton
EPI_ISL_1435600, EPI_ISL_1435602, EPI_ISL_1435604, EPI_ISL_1435606	Department of Public Health Microbiology Ljubljana, National Laboratory for Health, Environment and Food	Department for Public Health Microbiology Ljubljana, National Laboratory for Health, Environment and Food	José Gonçalves; Katarina Prosenc; Martin Bosilj; Metka Paragi; Tom Koritnik
EPI_ISL_1401986 to 1402336, EPI_ISL_1435579, EPI_ISL_1435581, EPI_ISL_1435583, EPI_ISL_1435585, EPI_ISL_1435587, EPI_ISL_1435589, EPI_ISL_1435591, EPI_ISL_1435593, EPI_ISL_1435595 to 1435596, EPI_ISL_1435598, EPI_ISL_1435608, EPI_ISL_1435610, EPI_ISL_1435612, EPI_ISL_1435614, EPI_ISL_1435616			
see above	Department of Public Health Microbiology Ljubljana, National Laboratory for Health, Environment and Food	Department of Public Health Microbiology Ljubljana, National Laboratory for Health, Environment and Food	José Gonçalves; Katarina Prosenc; Martin Bosilj; Metka Paragi; Tom Koritnik
EPI_ISL_576146 to 576149	Department of Respiratory & Other Viral Infections of L.V. Gromashevsky Institute of Epidemiology & Infectious Diseases NAMS of Ukraine	Department of Respiratory & Other Viral Infections of L.V. Gromashevsky Institute of Epidemiology & Infectious Diseases NAMS of Ukraine, JSC "Farmak"	Alla Mironenko; Andriy Goy; Ihor Kravchuk; Larysa Radchenko; Liudmyla Bolotova; Nataliia Teteriuk
EPI_ISL_582509	Department of Respiratory and other Viral Infections of L.V.Gromashevsky Institute of Epidemiology & Infectious Diseases NAMS of Ukraine	Department of Respiratory and other Viral Infections of L.V.Gromashevsky Institute of Epidemiology & Infectious Diseases NAMS of Ukraine, JSC "Farmak"	Alla Mironenko; Andriy Goy; Ihor Kravchuk; Larysa Radchenko; Ludmyla Bolotova; Nataliia Teteriuk
EPI_ISL_582510	Department of Respiratory and other Viral Infections of L.V.Gromashevsky Institute of Epidemiology & Infectious Diseases NAMS of Ukraine	Department of Respiratory and other Viral Infections of L.V.Gromashevsky Institute of Epidemiology & Infectious Diseases NAMS of Ukrain, JSC "Farmak"	Alla Mironenko; Andriy Goy; Ihor Kravchuk; Larysa Radchenko; Ludmyla Bolotova; Nataliia Teteriuk
EPI_ISL_582511 to 582513	Department of Respiratory and other Viral Infections of L.V.Gromashevsky Institute of Epidemiology & Infectious Diseases NAMS of Ukrain	Department of Respiratory and other Viral Infections of L.V.Gromashevsky Institute of Epidemiology & Infectious Diseases NAMS of Ukrain, JSC "Farmak"	Alla Mironenko; Andriy Goy; Ihor Kravchuk; Larysa Radchenko; Ludmyla Bolotova; Nataliia Teteriuk
EPI_ISL_487275	Department of Veterinary Pathology, University of Liege - FARAH	Department of Veterinary Pathology, University of Liege - FARAH	A.-S.; Clercx, C.; Desmecht, D.; Eloit, M.; Escriou, N.; Garigliany, M.; Giet, D.; Huon, C.; Vanlaere; van der Werf, S.
EPI_ISL_450198 to 450200, EPI_ISL_450203 to 450204, EPI_ISL_450207 to 450209, EPI_ISL_1385784 to 1385786, EPI_ISL_1385789 to 1385791, EPI_ISL_1385794, EPI_ISL_1385796, EPI_ISL_1385798, EPI_ISL_1385800, EPI_ISL_1385802, EPI_ISL_1385806, EPI_ISL_1385808, EPI_ISL_1385810, EPI_ISL_1385812, EPI_ISL_1397958 to 1397960, EPI_ISL_1398039, EPI_ISL_1406391, EPI_ISL_1406393 to 1406395, EPI_ISL_1406397, EPI_ISL_1406399 to 1406401			
see above	Department of Virology	Department of Virology	Aamer Ikram; Abdul Ahad; Ackermann, N.; Antwerpen, M.; Austin Leach; Bengs, K.; Berger, A.; Boehm, S.; Boehmer; Boender; Buchholz, U.; Cai, W.; Corman; D.V.; Dangel, A.; Drosten, C.; Eberle, U.; Fingerle, V.; Grahl, A.; Haas, W.; Hamouda, O.; Hoch, M.; Hoermansdorfer, S.; Ippisch, S.; Joel Montgomery; John Klena; Jones; Katz, K.; Ketan Patel; Konrad, R.; Liebl, B.; M.M.; Marosevic; Massab Umair; Melissa Mobley; Muehlemann, B.; Muhammad Salman; Muller, N.; Nazish Badar; Poertner, K.; Protzer, U.; Reich, A.; Rexroth, U.; Sana Tamim; Schneider, J.; Shannon Whitmer; Sing, A.; T.C.; T.S.; Treis, B.; V.M.; Veith, T.; Walter, M.; Wicklein, B.; Woelfel, R.; Woudenberg, T.; Zaira Rehman; Zapf, A.; Zeitlmann, N.; an der Heiden, M.
EPI_ISL_407084, EPI_ISL_529135	Department of Virology III, National Institute of Infectious Diseases	Pathogen Genomics Center, National Institute of Infectious Diseases	Kazuya Shirato; Kentaro Itokawa; Makoto Kuroda; Makoto Takeda; Masanori Hashino; Naganori Nao; Rina Tanaka; Shinji Watanabe; Shutoku Matsuyama; Takaji Wakita; Tsuyoshi Sekizuka
EPI_ISL_413602 to 413605, EPI_ISL_414640 to 414646, EPI_ISL_418385 to 418411, EPI_ISL_481513 to 481715, EPI_ISL_481717 to 481740, EPI_ISL_1496711 to 1497278, EPI_ISL_1497280 to 1497285, EPI_ISL_1548040 to 1548041			
see above	Department of Virology and Immunology, University of Helsinki and Helsinki University Hospital, Huslab Finland	Department of Virology, Faculty of Medicine, University of Helsinki, Helsinki, Finland	Essi Korhonen; Hanna Jarva; Hanna Liimatainen; Hannimari Kallio-Kokko; Harri Kangas; Hussein Alburkat; Jenni Virtanen; Maija Lappalainen; Maija Suvanto; Olli Vapalahti; Pekka Ellonen; Phuoc Truong; Ravi Kant; Sari Hannula; Satu Kurkela; Teemu Smura
EPI_ISL_1558595 to 1558614	Department of Virology, Istituto Zooprofilattico Sperimentale del Lazio e della Toscana (IZSLT)	Department of General Diagnostics; Department of Virology; Istituto Zooprofilattico Sperimentale del Lazio e della Toscana (IZSLT)	Alessia Franco; Antonella Cersini; Antonio Battisti.; Elena L. Diaconu; Fabiola Feltrin; Giuseppe Manna; Patricia Alba; Raffaela Conti; Teresa Scicluna; Virginia Carfora
EPI_ISL_468161, EPI_ISL_468163	Department of Virology, Public Health Laboratories Division, National Institute of Health	Department of Virology, Public Health Laboratories Division, National Institute of Health	Aamer Ikram; Adnan Khurshid; John Klena; Massab Umair; Muhammad Salman; Nazish Badar; Shannon Whitmer
EPI_ISL_515974	Department of Virus and Microbiological Special Diagnostics, Statens Serum Institut	Albertsen lab, Department of Chemistry and Bioscience, Aalborg University, Denmark	Danish Corona Danica Consortia
EPI_ISL_429333 to 429590, EPI_ISL_436962 to 437042, EPI_ISL_437627 to 437683, EPI_ISL_444817 to 444968, EPI_ISL_452101 to 452102	Department of Virus and Microbiological Special Diagnostics, Statens Serum Institut, Copenhagen, Denmark, Artillerivej 5, 2300 Copenahgen S	Albertsen lab, Department of Chemistry and Bioscience, Aalborg University, Denmark	Rasmus Kirkegaard
EPI_ISL_613541 to 613544, EPI_ISL_614403 to 614762, EPI_ISL_614764 to 614888, EPI_ISL_615166 to 620405, EPI_ISL_620762 to 622714	Department of Virus and Microbiological Special Diagnostics, Statens Serum Institut, Denmark	Albertsen lab, Department of Chemistry and Bioscience, Aalborg University, Denmark	Danish Corona Genome Consortia; Danish Covid-19 Genome Consortia
EPI_ISL_415647, EPI_ISL_416140 to 416142	Department of Virus and Microbiological Special diagnostics, Statens Serum Institut, Copenhagen, Denmark.	Statens Serum Institute	Anders Fomsgaard; Maiken Worsoe Rosenstierne; Morten Rasmussen
EPI_ISL_415646, EPI_ISL_415648, EPI_ISL_416143 to 416144, EPI_ISL_416153	Department of Virus and Microbiological Special diagnostics, Statens Serum Institut, Copenhagen, Denmark.	ViFU	Anders Fomsgaard; Maiken Worsoe Rosenstierne; Morten Rasmussen
EPI_ISL_413485	Department of microbiology laboratory,Anhui Provincial Center for Disease Control and Prevention	Department of microbiology laboratory,Anhui Provincial Center for Disease Control and Prevention	Bin Su; Jun He; Junling Yu; Qingqing Chen; Weidong Li; Weiwei Li; Yinglu Ge; Yong Sun; Yonglin Shi; Yuan Yuan; Zhirong Liu; Zhuhui Zhang
EPI_ISL_496482	Dept. Infectious, Tropical Diseases & Microbiology, IRCCS Sacro Cuore Don Calabria Hospital	1) Dept. Infectious, Tropical Diseases & Microbiology, IRCCS Sacro Cuore Don Calabria Hospital; 2) Centro Piattaforme Tecnologiche, University of Verona; 3) Dept. Neurosciences, Biomedicine and Movement Sciences, University of Verona.	1) Antonio Mori; 2) Monica Castellucci and Francesca Griggio; 3) Giovanni Malerba; Chiara Piubelli; Elena Pomari; Michela Deiana
EPI_ISL_449789 to 449794, EPI_ISL_590877, EPI_ISL_590953 to 590977, EPI_ISL_591021, EPI_ISL_1490197 to 1490198, EPI_ISL_1547512 to 1547513, EPI_ISL_1547520, EPI_ISL_1547522			
see above	Dept. of Medical Microbiology, Stavanger University Hospital, Helse Stavanger HF	Norwegian Institute of Public Health, Department of Virology	Atiya R Ali; Debech Nadia; Engebretsen Serina Beate; Garcia Llorente Ignacio; Hilde Elshaug; Hilde Vollan; Iren Löhr; Jon Bråte; Kamilla Heddeland Instefjord; Karoline Bragstad; Kathrine Stene-Johansen; Marie Paulsen Madsen; Olav Hungnes; Pedersen Benedikte Nevjen; Rasmus Riis Kopperud
EPI_ISL_447837	Dept. of Medical Microbiology, Stavanger University Hospital, Helse Stavanger HF,	Norwegian Institute of Public Health, Department of Virology	Hilde Elshaug; Kamilla Heddeland Instefjord; Karoline Bragstad; Kathrine Stene-Johansen; Olav Hungnes; Rasmus Riis Kopperud
EPI_ISL_410531 to 410532	Dept. of Pathology, National Institute of Infectious Diseases	Pathogen Genomics Center, National Institute of Infectious Diseases	Harutaka Katano; Hideki Hasegawa; Kazuya Shirato; Makoto Kuroda; Makoto Takeda; Motoi Suzuki; Naganori Nao; Shutoku Matsuyama; Tadaki Suzuki; Takaji Wakita; Tsuyoshi Sekizuka

EPI_ISL_408665 to 408667, EPI_ISL_408669	Dept. of Virology III, National Institute of Infectious Diseases	Pathogen Genomics Center, National Institute of Infectious Diseases	Kazuya Shirato; Makoto Kuroda; Makoto Takeda; Naganori Nao; Shutoku Matsuyama; Tsuyoshi Sekizuka
EPI_ISL_459911 to 459925, EPI_ISL_459931 to 459952	Devki Devi Foundation, a unit of Max Healthcare	CSIR-IGIB/Max	Anurag Agrawal; Bansidhar Tarai; Bharathram Uppili; Mitali Mukerji; Mohammed Faruq; Nishu Tyagi; Pooja Sharma; Poonam Das; Rajesh Pandey#; Samreen Siddiqui; Saruchi Wadhwa; Sujeet Jha; Vinita Jha; Vivekanand A
EPI_ISL_1394222 to 1394223	Di MEDICAL	1. National Institute of Public Health - National Institute of Hygiene; 2. Eurofins Genomics Europe Sequencing GmbH	ECDC COVID-19 WGS support team; Eurofins Genomics Europe Sequencing Team; Gierczyki Rafa; Sadkowska-Todys Magorzata; Wokowicz Tomasz; Zacharczuk Katarzyna
EPI_ISL_1393795 to 1393797, EPI_ISL_1394210 to 1394211	DiMedical	1. National Institute of Public Health - National Institute of Hygiene; 2. Eurofins Genomics Europe Sequencing GmbH	ECDC COVID-19 WGS support team; Eurofins Genomics Europe Sequencing Team; Gierczyki Rafa; Sadkowska-Todys Magorzata; Wokowicz Tomasz; Zacharczuk Katarzyna
EPI_ISL_1559626	Diagnostic Genomics Laboratory	Diagnostic Genomics Laboratory	Carolyn Banister; Diego Altomoare; Fadi Abboodi; Helmut Albrecht; Ji (Emily) Hao; Michael Shtutman; Phillip Buckhaults
EPI_ISL_1447329 to 1447362, EPI_ISL_1469232 to 1469237, EPI_ISL_1533034 to 1533101	Diagnostic and Research Center of Infectious Diseases, Medical Faculty, Andalas University	Diagnostic and Research Center of Infectious Diseases, Medical Faculty, Andalas University	Andani Eka Putra; Ayu Novita Trisnawati; Dede Rahman Agustian; Desmawati; Dessy Arisanty; Fauzul Azhim; Gestina Aliska; Ikhwan R. Sudji; Juane Plantika Menra; Linosefa; Mutia Lailani; Nia Ayuni Putri; Nita Afriani; SM Rezvi; Sekar Asri Tresnaningtyas; Siskalil Fahma; Syafrizayanti; Syandrez Prima Putra; Yolani Syaputri
EPI_ISL_437197 to 437203, EPI_ISL_437298 to 437303	Diagnostic- and Research Institute of Pathology, Medical University of Graz	Diagnostic- and Research Institute of Pathology, Medical University of Graz	Gregor Gorkiewicz; Karl Kashofer; Martin Zacharias; Peter Regitnig
EPI_ISL_1435840, EPI_ISL_1439526 to 1439530, EPI_ISL_1439532 to 1439534, EPI_ISL_1439536 to 1439577, EPI_ISL_1439579, EPI_ISL_1439581 to 1439583, EPI_ISL_1439585 to 1439588, EPI_ISL_1439591 to 1439592, EPI_ISL_1439594 to 1439596, EPI_ISL_1439598 to 1439624, EPI_ISL_1440967 to 1440979, EPI_ISL_1440981 to 1440982, EPI_ISL_1440984 to 1440986, EPI_ISL_1440988 to 1441034, EPI_ISL_1441036 to 1441040, EPI_ISL_1441042 to 1441051, EPI_ISL_1441053 to 1441058, EPI_ISL_1441060, EPI_ISL_1441062 to 1441073, EPI_ISL_1441075, EPI_ISL_1441077 to 1441080, EPI_ISL_1441082 to 1441087, EPI_ISL_1441089 to 1441098, EPI_ISL_1441101 to 1441110, EPI_ISL_1441113 to 1441114, EPI_ISL_1441116 to 1441125, EPI_ISL_1441128 to 1441130, EPI_ISL_1441132, EPI_ISL_1441134 to 1441136, EPI_ISL_1441138 to 1441139, EPI_ISL_1441141 to 1441146, EPI_ISL_1441148 to 1441157			
see above	Diagnosticum - Labor Neukirchen	Robert Koch Institute	
EPI_ISL_1538842 to 1538843, EPI_ISL_1539097 to 1539101	Diagnostyka	1. National Institute of Public Health - National Institute of Hygiene; 2. Eurofins Genomics Europe Sequencing GmbH	ECDC COVID-19 WGS support team; Eurofins Genomics Europe Sequencing Team; Gierczyki Rafa; Sadkowska-Todys Magorzata; Wokowicz Tomasz; Zacharczuk Katarzyna
EPI_ISL_1447200, EPI_ISL_1447206 to 1447210, EPI_ISL_1447243 to 1447249, EPI_ISL_1447278	Diagnostyka Sp. Z o.o.	1. National Institute of Public Health - National Institute of Hygiene; 2. Eurofins Genomics Europe Sequencing GmbH	ECDC COVID-19 WGS support team; Eurofins Genomics Europe Sequencing Team; Gierczyki Rafa; Sadkowska-Todys Magorzata; Wokowicz Tomasz; Zacharczuk Katarzyna
EPI_ISL_1394164 to 1394174	Diagnostyka Sp. z o. o Kraków	1. National Institute of Public Health - National Institute of Hygiene; 2. Eurofins Genomics Europe Sequencing GmbH	ECDC COVID-19 WGS support team; Eurofins Genomics Europe Sequencing Team; Gierczyki Rafa; Sadkowska-Todys Magorzata; Wokowicz Tomasz; Zacharczuk Katarzyna
EPI_ISL_1393783 to 1393784, EPI_ISL_1393789, EPI_ISL_1394231 to 1394232	Diagnostyka Sp. z o. o. ód	1. National Institute of Public Health - National Institute of Hygiene; 2. Eurofins Genomics Europe Sequencing GmbH	ECDC COVID-19 WGS support team; Eurofins Genomics Europe Sequencing Team; Gierczyki Rafa; Sadkowska-Todys Magorzata; Wokowicz Tomasz; Zacharczuk Katarzyna
EPI_ISL_1538639 to 1538642, EPI_ISL_1538868, EPI_ISL_1538971 to 1538977	Diagnostyka Sp. z o.o	1. National Institute of Public Health - National Institute of Hygiene; 2. Eurofins Genomics Europe Sequencing GmbH	ECDC COVID-19 WGS support team; Eurofins Genomics Europe Sequencing Team; Gierczyki Rafa; Sadkowska-Todys Magorzata; Wokowicz Tomasz; Zacharczuk Katarzyna
EPI_ISL_1394114 to 1394118, EPI_ISL_1425564, EPI_ISL_1538525, EPI_ISL_1538643 to 1538644, EPI_ISL_1538978 to 1538981	Diagnostyka Sp. z o.o.	1. National Institute of Public Health - National Institute of Hygiene; 2. Eurofins Genomics Europe Sequencing GmbH	ECDC COVID-19 WGS support team; Eurofins Genomics Europe Sequencing Team; Gierczyki Rafa; Sadkowska-Todys Magorzata; Wokowicz Tomasz; Zacharczuk Katarzyna
EPI_ISL_1538550 to 1538552, EPI_ISL_1538763 to 1538764, EPI_ISL_1538897 to 1538899, EPI_ISL_1539034 to 1539036, EPI_ISL_1539154, EPI_ISL_1539180	Diagnostyka Sp. z o.o. Gdask	1. National Institute of Public Health - National Institute of Hygiene; 2. Eurofins Genomics Europe Sequencing GmbH	ECDC COVID-19 WGS support team; Eurofins Genomics Europe Sequencing Team; Gierczyki Rafa; Sadkowska-Todys Magorzata; Wokowicz Tomasz; Zacharczuk Katarzyna
EPI_ISL_1393923 to 1393932, EPI_ISL_1394247 to 1394254, EPI_ISL_1425550, EPI_ISL_1425558, EPI_ISL_1538598 to 1538599, EPI_ISL_1538938 to 1538942, EPI_ISL_1539201			
see above	Diagnostyka Sp.z o.o.	1. National Institute of Public Health - National Institute of Hygiene; 2. Eurofins Genomics Europe Sequencing GmbH	ECDC COVID-19 WGS support team; Eurofins Genomics Europe Sequencing Team; Gierczyki Rafa; Sadkowska-Todys Magorzata; Wokowicz Tomasz; Zacharczuk Katarzyna
EPI_ISL_1435285 to 1435291	Dianovis GmbH Greiz	Robert Koch Institute	
EPI_ISL_1393798 to 1393800, EPI_ISL_1394214 to 1394217, EPI_ISL_1425549	Dimedical	1. National Institute of Public Health - National Institute of Hygiene; 2. Eurofins Genomics Europe Sequencing GmbH	ECDC COVID-19 WGS support team; Eurofins Genomics Europe Sequencing Team; Gierczyki Rafa; Sadkowska-Todys Magorzata; Wokowicz Tomasz; Zacharczuk Katarzyna
EPI_ISL_452234, EPI_ISL_475512, EPI_ISL_475562 to 475563	Din Klinik	The Public Health Agency of Sweden	Anna Risberg; Anna-Malin Linde; Helene Warnborg; Karin Tegmark-Wisell; Maria Lind Karlberg; Mattias Haukland; Mia Brytting; Olov Svartstrom; Oskar Karlsson Lindsjo; Reza Advani; Sandra Broddesson; Theresa Enkirch
EPI_ISL_1469250	Dinas Kesehatan Blitar	National Institute of Health Research and Development	Arie Ardiansyah Nugraha; Hana Apsari Pawestri; Hartanti Dian Ikawati; Kartika Dewi Puspa; Krisna Pangesti; Nelly Puspandari; Subangkit; Vivi Setiawaty
EPI_ISL_1469240, EPI_ISL_1469246, EPI_ISL_1469271 to 1469275, EPI_ISL_1469284	Dinas Kesehatan Kabupaten Ponorogo	National Institute of Health Research and Development	Arie Ardiansyah Nugraha; Hana Apsari Pawestri; Hartanti Dian Ikawati; Kartika Dewi Puspa; Krisna Pangesti; Nelly Puspandari; Subangkit; Vivi Setiawaty
EPI_ISL_528753	Dinkes Kota Bogor	School of Life Sciences and Technology & School of Pharmacy-Institut Teknologi Bandung; Molecular Genetics Laboratory-Faculty of Medicine-Universitas Padjadjaran; Laboratorium Kesehatan Provinsi Jawa Barat	Agung Eru Wibowo; Azzania Fibriani; Catur Riani; Cut Nur Cinthia Alamanda; Davin H. E. Setiamarga; Ema Rahmawati; Hammam Riza; Hesti Lina Wiraswati; Husna Nugrahapraja; Irvan Faizal; Lia Faridah; Marselina Irasonia Tan; Rifky Waluyajati Rachman; Rizki Mardian; Ryan Bayusantika Ristandi; Savira Ekawardhani; Sony Solistia Wirawan; Tarwadi; Yunia Sribudiani
EPI_ISL_528748	Dinkes Provinsi Jawa Barat	School of Life Sciences and Technology & School of Pharmacy-Institut Teknologi Bandung; Molecular Genetics Laboratory-Faculty of Medicine-Universitas Padjadjaran; Laboratorium Kesehatan Provinsi Jawa Barat	Adelina Khristiani Rahayu; Agung Eru Wibowo; Azzania Fibriani; Catur Riani; Cut Nur Cinthia Alamanda; Ema Rahmawati; Gusti Ayu Prani Pradani; Hammam Riza; Hesti Lina Wiraswati; Husna Nugrahapraja; Irvan Faizal; Lia Faridah; Marselina Irasonia Tan; Rifky Waluyajati Rachman; Ryan Bayusantika Ristandi; Savira Ekawardhani; Sony Solistia Wirawan; Tarwadi; Yunia Sribudiani
EPI_ISL_467374	Dinkes Samarinda	Eijkman Institute for Molecular Biology, Ministry of Research and Technology/National Agency for Research and Innovation	Amin Soebandrio; David H Muljono; Edison Johar; Frilasita A Yudhaputri; Hidayat Trimarsanto; Khin Saw Myint; Safarina G Malik
EPI_ISL_591327 to 591340	Dipartimento di Biotecnologie Mediche, University of Siena	Dipartimento di Biotecnologie Mediche, University of Siena	Anichini, G.; COVID; Cusi; Gandolfo, C.; M.G.; Pinzauti, D.; Pozzi, G.; Santoro, F.
EPI_ISL_1509923	Dipartimento di Medicina e Chirurgia Universita' degli Studi dell'Insubria Laboratorio di Microbiologia Medica Varese Italia	Laboratorio di Microbiologia e Virologia, Università Vita-Salute San Raffaele, Milano	Elena Criscuolo; Enzo Boeri; Fabrizio Maggi; Massimo Clementi; Matteo Castelli; Michela Sampaolo; Nicasio Mancini and Nicola Clementi; Roberta Antonia Diotti; Roberto Ferrarese
EPI_ISL_1533724	Diretoria Municipal de Saude	Instituto Adolfo Lutz, Interdiciplinary Procedures Center, Strategic Laboratory	Caio Vinicius Dias Lopes; Claudia Regina Gonçalves; Claudio Tavares Sacchi; Erica Valessa Ramos Gomes; Karoline Rodrigues Campos; Leonardo Jose Tadeu de Araujo
EPI_ISL_1469553, EPI_ISL_1469560, EPI_ISL_1469592, EPI_ISL_1469598, EPI_ISL_1469605, EPI_ISL_1469609 to 1469610, EPI_ISL_1469616, EPI_ISL_1469618, EPI_ISL_1469623, EPI_ISL_1469625, EPI_ISL_1469627, EPI_ISL_1469632, EPI_ISL_1469634, EPI_ISL_1469644, EPI_ISL_1469650, EPI_ISL_1469656, EPI_ISL_1469669, EPI_ISL_1469672, EPI_ISL_1469700, EPI_ISL_1469702, EPI_ISL_1469708, EPI_ISL_1469728, EPI_ISL_1469748, EPI_ISL_1469753, EPI_ISL_1469756, EPI_ISL_1469761, EPI_ISL_1469769, EPI_ISL_1469775, EPI_ISL_1469783, EPI_ISL_1469786, EPI_ISL_1469792, EPI_ISL_1469796 to 1469797, EPI_ISL_1469802, EPI_ISL_1469808, EPI_ISL_1469816, EPI_ISL_1469819, EPI_ISL_1469823, EPI_ISL_1469829, EPI_ISL_1469832, EPI_ISL_1469835 to 1469836, EPI_ISL_1469841, EPI_ISL_1469848, EPI_ISL_1479120			
see above	Diretoria de Vigilância em Saúde	Epiclin	Ana Paula Muterle; Carolina Comerlato; Eliana Márcia Da Ros Wendland; Fernando Hayashi Sant'Anna; Janira Prichula; Juliana Comerlato
EPI_ISL_524428 to 524432	Dirk Dittmer	Dirk Dittmer	A.B.; A.G.; Bailey; C.P.; Caro-Vegas; Dittmer, D.; Eason; J.T.; Juarez, A.; L.J.; Landis; M.B.; McNamara; Miller; Moorad, R.; Pluta; R.P.; Seltzer; T.A.; Thompson, C.; Vahrson; W. and Villamor, F.
EPI_ISL_504185, EPI_ISL_515466	Discovery DNA	Discovery DNA	Alice Li; Aneal Khan; Desmond Koo; Dustin Hittel; Leo Dimnik; Marina Kerr
EPI_ISL_1534373	District 6 CDC	NHLS/UCT	Arash Iranzadeh; Bruna Galvao; Carolyn Williamson; Deelan Doolabh; Diana Hardie; Emmanuel SJ; Innocent Mudau; Kruger Marais; Lynn Tyers; Marvin

			Hsiao; Stephen Korsman; Tegally H; de Oliveira T
EPI_ISL_436137 to 436141, EPI_ISL_436156 to 436157	District Surveillance Unit	Department of Neurovirology, National Institute of Mental Health and Neuroscience (NIMHANS)	Anita Desai; Chitra Pattabiraman; Harsha PK; Manjunatha Venkataswamy; Ravi Vasanthapuram; Risha Rasheed; Shafeeq S Hameed; Vijayalakshmi Reddy
EPI_ISL_428479 to 428487, EPI_ISL_486382	District Surveillence Unit	Department of Neurovirology, National Institute of Mental Health and Neuroscience (NIMHANS)	Anita Desai; Chitra Pattabiraman; Harsha PK; Manjunatha Venkataswamy; Ravi Vasanthapuram; Risha Rasheed; Shafeeq S Hameed; Vijayalakshmi Reddy
EPI_ISL_534312	Distrito Sanitario Sul	Instituto Adolfo Lutz, Interdiciplinary Procedures Center, Strategic Laboratory	Claudia Regina Gonçalves; Claudio Tavares Sacchi; Erica Valessa Ramos Gomes
EPI_ISL_583499	Distrito Sanitario Sul Campinas	Instituto Adolfo Lutz, Interdiciplinary Procedures Center, Strategic Laboratory	Claudia Regina Gonçalves; Claudio Tavares Sacchi; Erica Valessa Ramos Gomes; Karoline Rodrigues Campos
EPI_ISL_1489527, EPI_ISL_1489529 to 1489656, EPI_ISL_1489942 to 1490164, EPI_ISL_1490167 to 1490184	Division of Emerging Infectious Diseases, Bureau of Infectious Diseases Diagnosis Control, Korea Disease Control and Prevention Agency	Division of Emerging Infectious Diseases, Bureau of Infectious Diseases Diagnosis Control, Korea Disease Control and Prevention Agency	Ae Kyung Park; Chae Young Lee; Eun-Jin Kim; Heui Man Kim; Il-Hwan Kim; Jeong-Ah Kim; Jeong-Min Kim; Jin Sun No
EPI_ISL_1491561 to 1491569, EPI_ISL_1491572	Division of Epidemiology	COVID-19 Network Investigations (CONI) Alliance	Angkana Huang; Anthony R. Jones; Araya Keskesorn; Arporn Wangwiwatsin; Arthicha Wongkumma; Atika Awae; Bhakbhoom Panthan; Chattiwutti Tanabat; Chodmanee Sukunpun; Chonticha Klungtong; Duangkamon Loesbanluechai; Ekawat Pasomsub; Elizabeth Batty; Insee Sensorn; Janjira Thaipadungpanit; Juraiwan Ungsathaphornphon; Kamonchanok Chuayjan; Kawinna Kerdsalung; Khajohn Joonlasak; Kingkan Rakmanee; Krittikorn Kumpornsin; Namfon Kotanan; Pantila Taweewigyakarn; Prangsiri Nalaem; Prapai Kreerat; Rattanapong Kompao; Rusmanira Khwankerd; Saraiya Chete; Sawanya Chantutanon; Sawvanit Lamun; Sitipatimoh Mina; Stefan Fernandez; Thanat Chookajorn; Theerarat Kochakarn; Treewat Watthanachockchai; Wachirapun Chainontee; Wasun Chantratita; Wudtichai Manasatienkij
EPI_ISL_485603	Division of Infectious Disease	Steininger Lab	Christoph Steininger; Ingeborg Klymiuk; Jakob Thannesberger; Lorenz Schubert; Nicolas Rascovan; Oliver Robak
EPI_ISL_508864 to 508870, EPI_ISL_509222	Division of Infectious Diseases and Hospital Epidemiology, University Hospital Zürich	Institute of Medical Virology, University of Zurich	Aline Wolfensberger; Dana Weissberg; Hugo Sax; Irene A. Abela; Jürg Böni; Maryam Zaheri; Michael Huber; Peter W. Schreiber; Silvana K. Rampini; Verena Kufner
EPI_ISL_413513, EPI_ISL_413515	Division of Infectious Diseases, Department of Internal Medicine, Korea University College of Medicine	Department of Microbiology, Institute for Viral Diseases, College of Medicine, Korea University	Changmin Kang; Cui Chunguang; Gee Eun Lee; Hee Jin Cheong; Heedo Park; Jeonghun Kim; Ji Yun Noh; Jin Gu Yoon; Jin Il Kim; Joon Young Song; Joon-Yong Bae; Jungmin Lee; Juyoung Cho; Kyeong-ryeol Shin; Man-Seong Park; Woo Joo Kim
EPI_ISL_413022 to 413024	Division of Infectious Diseases, University Hospital Zurich	Institute of Medical Virology, University of Zurich	Alexandra Trkola; Andrea Zbinden; Fiona Steiner; Gabriela Ziltener; Jon Huder; Jürg Böni; Maryam Zaheri; Michael Huber; Patrick Redli; Riccarda Capaul; Roberto Speck; Stefan Schmutz; Verena Kufner
EPI_ISL_524475, EPI_ISL_524477, EPI_ISL_524483, EPI_ISL_524485	Division of Infectious Diseases, University Hospital Zürich	Institute of Medical Virology, University of Zurich	Alexandra Trkola; Andrea Zbinden; Fiona Steiner; Gabriela Ziltener; Jon Huder; Jürg Böni; Maryam Zaheri; Michael Huber; Patrick Redli; Riccarda Capaul; Stefan Schmutz; Verena Kufner
EPI_ISL_1502129 to 1502198	Division of Medical Virology, National Health Laboratory Service (NHLS), Tygerberg Hospital / Stellenbosch University	Division of Medical Virology, Stellenbosch University and NHLS Tygerberg Hospital	Bronwyn Kleinhans; Gert van Zyl; Susan Engelbrecht; Wolfgang Preiser
EPI_ISL_412869 to 412873, EPI_ISL_425117 to 425118, EPI_ISL_426163 to 426164, EPI_ISL_426166 to 426169, EPI_ISL_426171, EPI_ISL_426173, EPI_ISL_426180 to 426183, EPI_ISL_426187, EPI_ISL_427809 to 427813, EPI_ISL_471425 to 471426, EPI_ISL_471438 to 471455, EPI_ISL_481370 to 481379, EPI_ISL_497951, EPI_ISL_497953 to 498053, EPI_ISL_506947 to 506995, EPI_ISL_510547 to 510688, EPI_ISL_514756 to 515054, EPI_ISL_522454, EPI_ISL_522465 to 522466, EPI_ISL_522474 to 522475, EPI_ISL_522477 to 522478, EPI_ISL_522484, EPI_ISL_522486 to 522490, EPI_ISL_522498 to 522501, EPI_ISL_522503 to 522504, EPI_ISL_522506 to 522507, EPI_ISL_522509, EPI_ISL_522511 to 522514, EPI_ISL_522517, EPI_ISL_522519 to 522544, EPI_ISL_526700 to 526729, EPI_ISL_526732 to 526733			
see above	Division of Viral Diseases, Center for Laboratory Control of Infectious Diseases, Korea Centers for Diseases Control and Prevention	Division of Viral Diseases, Center for Laboratory Control of Infectious Diseases, Korea Centers for Diseases Control and Prevention	Daesang Lee; Dong Hyun Song; Heui Man Kim; Hye-Jun Jo; Jeong-Min Kim; Jun-Sub Kim; Junhyeong Jang; Mi-Seon Kim; Myung Guk Han; Namjoo Lee; Sang Hee Woo; Sehee Park; Seong Tae Jeong; Yoon-Seok Chung
EPI_ISL_513357, EPI_ISL_513389	Douglas Hanly Moir	NSW Health Pathology - Institute of Clinical Pathology and Medical Research; Westmead Hospital; University of Sydney	CIDM-PH et al.
EPI_ISL_451601, EPI_ISL_455051 to 455053, EPI_ISL_455065	Douglas Hanly Moir Pathology	NSW Health Pathology - Institute of Clinical Pathology and Medical Research; Westmead Hospital; University of Sydney	CIDM-PH et al.
EPI_ISL_498773, EPI_ISL_526153, EPI_ISL_526213 to 526214, EPI_ISL_545027, EPI_ISL_593650 to 593651	Douglass Hanly Moir Pathology	NSW Health Pathology - Institute of Clinical Pathology and Medical Research; Westmead Hospital; University of Sydney	CIDM-PH et al.
EPI_ISL_1545263 to 1545265	Dr. Bard	Laboratory of genomics and metagenomics	Claire Bertelli; Damien Jacot; Gilbert Greub; Sébastien Aeby; Trestan Pillonel
EPI_ISL_1545260 to 1545261	Dr. Borel	Laboratory of genomics and metagenomics	Claire Bertelli; Damien Jacot; Gilbert Greub; Sébastien Aeby; Trestan Pillonel
EPI_ISL_1457773	Dr. Carol Davila Central Military Emergency University Hospital	"Stefan S. Nicolau"Institute of Virology	Adriana Plesa; Alina Nastasie; Ana Iulia Neagu; Anca Botezatu; Camelia Sultana; Carmen Cristina Diaconu; Codruta-Georgiana Carp; Coralia Bleotu; Cristina Mambet; Denisa Dragu; Gabriela Anton; Ioana Pitica; Iulia Virginia Iancu; Laura Grecu; Laura Necula; Lilia Matei; Marinela Bostan; Mihaela Economescu; Mirela Mihaila; Saviana Nedeianu; Simona Ruta
EPI_ISL_418811, EPI_ISL_429806, EPI_ISL_429811, EPI_ISL_429816, EPI_ISL_479625 to 479651, EPI_ISL_481742 to 481758, EPI_ISL_482471 to 482473			
see above	Dr. Georges-L.-Dumont University Hospital Centre	National Microbiology Laboratory	Anna Majer; Elsie Grudeski; Gary Van Domselaar; Grace Seo; Guillaume Desnoyers; Jennifer Tanner; Kristyn Burak; Matthew Gilmour; Morag Graham; Natalie Knox; Nathalie Bastien; Philip Mabon; Rhiannon Huzarewich; Richard Garceau; Russell Mandes; Shari Tyson; Timothy Booth; Yan Li
EPI_ISL_583727 to 583852	Dr. Gernot Walder GmbH	Bergthaler laboratory, CeMM Research Center for Molecular Medicine of the Austrian Academy of Sciences	Adi Steinrigl; Alexander Lercher; Alexandra Popa; Andreas Bergthaler; Benedikt Agerer; Christian Paar; Christoph Bock; Daniela Schmid; Dorothee von Laer; Elisabeth Puchhammer-Stoeckl; Franz Allerberger; Gernot Walder; Gregor Hörmann; Guenter Weiss; Gunther Vogl; Henrique Colaco; Jakob-Wendelin Genger; Jan Laine; Judith Aberle; Kinga Rigler-Hohenwarter; Lukas Endler; Manfred Nairz; Mark Smyth; Martin Senekowitsch; Michael Schuster; Peter Hufnagl; Peter Obrist; Rainer Gattringer; Sabine Sussitz-Rack; Stephan Aberle; Thomas Penz; Wegene Borena
EPI_ISL_528752	Dr. H. A. Rotinsulu Lung Hospital	School of Pharmacy & School of Life Sciences and Technology - Institut Teknologi Bandung; Molecular Genetics Laboratory-Faculty of Medicine-Universitas Padjadjaran; Laboratorium Kesehatan Provinsi Jawa Barat	Adelina Khristiani Rahayu; Agung Eru Wibowo; Azzania Fibriani; Catur Riani; Cut Nur Cinthia Alamanda; Ema Rahmawati; Gusti Ayu Prani Pradani; Hammam Riza; Hesti Lina Wiraswati; Husna Nugrahapraja; Irvan Faizal; Lia Faridah; Marselina Irasonia Tan; Rifky Waluyajati Rachman; Ryan Bayusantika Ristandi; Savira Ekawardhani; Sony Solistia Wirawan; Tarwadi; Yunia Sribudiani
EPI_ISL_469043 to 469047, EPI_ISL_475056 to 475057, EPI_ISL_495067 to 495069	Dr. N. D. Desai Medical College & Hospital	Gujarat Biotechnology Research Centre	A M Kadri; Afzal Ansari; Akanksha Verma; Ankit Hinsu; Apurvasinh Puvar; Bhavya Jindal; Chaitanya Joshi; Dinesh Kumar; Harsh Bakshi; J G Buch; Janvi Raval; Jigar Gusani; Komal Patel; Labdhi Pandya; Maharshi Pandya; Monika Gandhi; Neha Rajpara; Nidhi Patel; Nikha Trivedi; Nitin Savaliya; Pinal Trivedi; Pooja P Doshi; Pritesh Sabara; Priti Pandita; Priyanka P Vatsa; R D Dixit; Raghawendra Kumar; Snehal Bagatharia; Supreet Prabhu; Tejas Shah; Zarna Patel; Zuber Saiyed
EPI_ISL_512075 to 512077, EPI_ISL_514435 to 514437, EPI_ISL_524732	Dr. RSS Hospital, Modasa	Gujarat Biotechnology Research Centre	A M Kadri; Afzal Ansari; Apurvasinh Puvar; Chaitanya Joshi; Dinesh Kumar; Harsh Bakshi; Harsh Chaudhari; Janvi Raval; Komal Patel; Labdhi Pandya; Madhvi Joshi; Maharshi Pandya; Monika Gandhi; Neena Doshi; Nidhi Patel; Nikha Trivedi; Nitin Savaliya; Pinal Trivedi; R D Dixit; Raghawendra Kumar; Varsha Godbole; Zarna Patel; Zuber Saiyed
EPI_ISL_495012	Dr. Tony Mazzulli Microbiologist-in-Chief	Dr. Jeff Wrana, Senior Investigator	Bryn Hazlett; Christine Bruce; Dan Trcka; Javier Hernandez; Jeff Wrana; Jess Shen; Jessica Bourke; Kin Chan; Laurence Pelletier; Marie-Ming Aynaud; Seda Barutcu; Sue Poutanen; Tony Mazzulli
EPI_ISL_494970, EPI_ISL_495013	Dr. Tony Mazzulli Microbiologist-in-Chief	Dr. Jeff Wrana, Senior Investigator	Bryn Hazlett; Christine Bruce; Dan Trcka; Javier Hernandez; Jeff Wrana; Jess Shen; Jessica Bourke; Kin Chan; Laurence Pelletier; Marie-Ming Aynaud; Seda Barutcu; Sue Poutanen; Tony Mazzulli
EPI_ISL_417211 to 417212	Dunedin Hospital	University of Otago	B. Lawley; J. Grant; J. Ussher; M.E. Quiñones-Mateu; R. Harfoot
EPI_ISL_414423 to 414446, EPI_ISL_414448 to 414471, EPI_ISL_414529 to 414541, EPI_ISL_414544 to 414566, EPI_ISL_415460 to 415463, EPI_ISL_415465 to 415492, EPI_ISL_415495 to 415498, EPI_ISL_415500 to 415506, EPI_ISL_415508, EPI_ISL_415510 to 415516, EPI_ISL_415524 to 415535, EPI_ISL_422566 to 422635, EPI_ISL_422637 to 422881, EPI_ISL_422885 to 422899, EPI_ISL_422901 to 422960, EPI_ISL_423034, EPI_ISL_455112 to 455180, EPI_ISL_455194 to 455200, EPI_ISL_455202 to 455222, EPI_ISL_455226 to 455258, EPI_ISL_455261, EPI_ISL_455263 to 455307, EPI_ISL_460636 to 460705, EPI_ISL_460714 to 460721, EPI_ISL_460723 to 460738, EPI_ISL_460753 to 460768, EPI_ISL_460770 to 460771, EPI_ISL_460773 to 460821, EPI_ISL_460823 to 460890, EPI_ISL_460892 to 461050, EPI_ISL_461061 to 461087, EPI_ISL_461101 to 461111, EPI_ISL_461113 to 461159, EPI_ISL_461161, EPI_ISL_461163 to 461167,			

EPI_ISL_461172, EPI_ISL_461177, EPI_ISL_461179, EPI_ISL_461181 to 461189, EPI_ISL_461193 to 461199, EPI_ISL_461201 to 461202, EPI_ISL_461204 to 461232, EPI_ISL_461234 to 461373, EPI_ISL_461375 to 461381, EPI_ISL_461383 to 461391, EPI_ISL_461394, EPI_ISL_461396 to 461398, EPI_ISL_522987 to 523212, EPI_ISL_523235 to 523273, EPI_ISL_523275 to 523291, EPI_ISL_523301 to 523309, EPI_ISL_523332, EPI_ISL_523334 to 523385, EPI_ISL_523401 to 523499, EPI_ISL_523501 to 523517, EPI_ISL_523522 to 523526, EPI_ISL_523528 to 523558, EPI_ISL_523568 to 523578, EPI_ISL_523595 to 523611, EPI_ISL_523613 to 523657, EPI_ISL_523659 to 523809, EPI_ISL_574779 to 574781, EPI_ISL_574786 to 574789, EPI_ISL_577743 to 577747, EPI_ISL_577749 to 577803, EPI_ISL_577805, EPI_ISL_577807 to 577844, EPI_ISL_577847 to 577850, EPI_ISL_577853 to 577866, EPI_ISL_577872 to 577882, EPI_ISL_577886 to 577905, EPI_ISL_577908 to 577916, EPI_ISL_577926 to 577944, EPI_ISL_577948 to 577954, EPI_ISL_577964 to 578000, EPI_ISL_578004 to 578071, EPI_ISL_578073 to 578079			
see above	Dutch COVID-19 response team	Erasmus Medical Center	Anne van der Linden; Annemiek van der Eijk; Aura Timen; Bas Oude Munnink; Claudia Schapendonk; Corien Swaan; Corine GeurtsvanKessel; David Nieuwenhuijse; Emmanuelle Munger; Irina Chestakova; Jeroen van Kampen; Jolanda Voermans; Madelief Mollers; Manon Haverkate; Marion Koopmans; Marjan Boter; Mark Pronk; Mart Stein; OH consortium; Pascal Lexmond; Reina Sikkema; Richard Molenkamp; Sandra Kengne Kamga Mobou; Stefan van Nieuwkoop; Theo Bestebroer; on behalf of the Dutch national COVID-19 response team.
EPI_ISL_1394665 to 1394737, EPI_ISL_1443441 to 1443511, EPI_ISL_1509640 to 1509696, EPI_ISL_1509702 to 1509716, EPI_ISL_1509718 to 1509719, EPI_ISL_1558521 to 1558593	Dutch COVID-19 response team	Medical Microbiology, Maastricht University Medical Centre	Brian van der Veer*; Carmen Reumkens; Christian Hoebe; Erik Beuken; Jozef Dingemans*; Lieke van Alphen; Paul Savelkoul
EPI_ISL_454750 to 454767, EPI_ISL_454769 to 454794, EPI_ISL_547445 to 547453, EPI_ISL_547455 to 547458, EPI_ISL_547460 to 547465, EPI_ISL_547469 to 547470, EPI_ISL_547473 to 547486, EPI_ISL_547491 to 547499, EPI_ISL_547501 to 547509, EPI_ISL_547511, EPI_ISL_547513 to 547522, EPI_ISL_547524 to 547529, EPI_ISL_547531 to 547534, EPI_ISL_547536 to 547539, EPI_ISL_547541, EPI_ISL_547557 to 547560, EPI_ISL_1455675 to 1456120, EPI_ISL_1456122 to 1456449, EPI_ISL_1456451 to 1457125, EPI_ISL_1457127 to 1457195, EPI_ISL_1457197 to 1457408, EPI_ISL_1457410 to 1457756, EPI_ISL_1498153 to 1498261, EPI_ISL_1520441 to 1522236			
see above	Dutch COVID-19 response team	National Institute for Public Health and the Environment (RIVM)	Adam Meijer; AnneMarie van den Brandt; Annelies Kroneman; Bas van der Veer; Chantal Reusken; Dennis Schmitz; Dirk Eggink; Euníce Then; Florian Zwagemaker; Harry Vennema; James Groot; Jeroen Cremer; Jolienke Hardeman; Karim Hajji; Kim Freriks; Linda van de Nes; Lisa Wijsman; Lynn Aarts; Melissa van Tuil; Pieter Overduin; Robert Kohl; Ryanne Jaarsma; Sanne Bos; Sharon van den Brink; Sjoerd Kuiling; on behalf of the national COVID-19 response team
EPI_ISL_1534299, EPI_ISL_1534387	Dysselsdorp Clinic wc DDC	NHLS/UCT	Arash Iranzadeh; Bruna Galvao; Carolyn Williamson; Deelan Doolabh; Diana Hardie; Emmanuel SJ; Innocent Mudau; Kruger Marais; Lynn Tyers; Marvin Hsiao; Stephen Korsman; Tegally H; de Oliveira T
EPI_ISL_1447201	Dzia Diagnostyki Laboratoryjnej Wojewódzki Szpital Specjalistyczny im.J.Gromkowskiego	1. National Institute of Public Health - National Institute of Hygiene; 2. Eurofins Genomics Europe Sequencing GmbH	ECDC COVID-19 WGS support team; Eurofins Genomics Europe Sequencing Team; Gierczyki Rafa; Sadkowska-Todys Magorzata; Wokowicz Tomasz; Zacharczuk Katarzyna
EPI_ISL_1393659 to 1393664, EPI_ISL_1538474, EPI_ISL_1538885 to 1538888, EPI_ISL_1538998 to 1539001, EPI_ISL_1539174	Dzia Diagnostyki Laboratoryjnej SPZOZ	1. National Institute of Public Health - National Institute of Hygiene; 2. Eurofins Genomics Europe Sequencing GmbH	ECDC COVID-19 WGS support team; Eurofins Genomics Europe Sequencing Team; Gierczyki Rafa; Sadkowska-Todys Magorzata; Wokowicz Tomasz; Zacharczuk Katarzyna
EPI_ISL_1538529	Dzia Diagnostyki Laboratoryjnej Wojewódzki Szpital Specjalistyczny im.J.Gromkowskiego	1. National Institute of Public Health - National Institute of Hygiene; 2. Eurofins Genomics Europe Sequencing GmbH	ECDC COVID-19 WGS support team; Eurofins Genomics Europe Sequencing Team; Gierczyki Rafa; Sadkowska-Todys Magorzata; Wokowicz Tomasz; Zacharczuk Katarzyna
EPI_ISL_1538927	Dzia Diagnostyki Laboratoryjnej Wojewódzkiego Szpitala Obserwacyjno-Zakanego w Bydgoszczy	1. National Institute of Public Health - National Institute of Hygiene; 2. Eurofins Genomics Europe Sequencing GmbH	ECDC COVID-19 WGS support team; Eurofins Genomics Europe Sequencing Team; Gierczyki Rafa; Sadkowska-Todys Magorzata; Wokowicz Tomasz; Zacharczuk Katarzyna
EPI_ISL_1538837, EPI_ISL_1539094	Dziecicy Szpital Kliniczny im. Józefa Polikarpa Brudziskiego UCK WUM w Warszawie	1. National Institute of Public Health - National Institute of Hygiene; 2. Eurofins Genomics Europe Sequencing GmbH	ECDC COVID-19 WGS support team; Eurofins Genomics Europe Sequencing Team; Gierczyki Rafa; Sadkowska-Todys Magorzata; Wokowicz Tomasz; Zacharczuk Katarzyna
EPI_ISL_421653 to 421656, EPI_ISL_426285 to 426289, EPI_ISL_450518 to 450519, EPI_ISL_492993 to 493000, EPI_ISL_501275, EPI_ISL_501284 to 501285, EPI_ISL_501823, EPI_ISL_501829, EPI_ISL_501833, EPI_ISL_501839, EPI_ISL_501849, EPI_ISL_501894, EPI_ISL_501929, EPI_ISL_501936, EPI_ISL_512313 to 512314, EPI_ISL_512645 to 512646, EPI_ISL_515187 to 515195, EPI_ISL_534199, EPI_ISL_534203, EPI_ISL_534212 to 534216, EPI_ISL_534218 to 534219, EPI_ISL_534221			
see above	E. Gulbja Laboratorija	Latvian Biomedical Research and Study Centre	Dmitrijs Perminovs; Ivars Silamielis; Jnis Kloviš; Jnis Pjalkovskis; Kaspars Megnis; Mikus Gavars; Monta Ustinova; Uga Dumpis; Vita Rovte; ikita Zrelovs
EPI_ISL_486438	E. Gulbja laboratorija	Latvian Biomedical Research and Study Centre	Dmitrijs Perminovs; Ivars Silamielis; Jnis Kloviš; Kaspars Megnis; Mikus Gavars; Monta Ustinova; Uga Dumpis; Vita Rovte; ikita Zrelovs
EPI_ISL_434540	EBAIS Concepción Norte	Inciensa, Instituto Costarricense de Investigación y Enseñanza en Nutrición y Salud	Adriana Godinez & Melany Calderon; Claudio Soto-Garita; Estela Cordero; Francisco Duarte; Hebleen Porras
EPI_ISL_1545248 to 1545250, EPI_ISL_1545252 to 1545253, EPI_ISL_1545255 to 1545256	EHC MORGES	Laboratory of genomics and metagenomics	Claire Bertelli; Damien Jacot; Gilbert Greub; Sébastien Aeby; Trestan Pillonel
EPI_ISL_1517303, EPI_ISL_1545185 to 1545186, EPI_ISL_1545189, EPI_ISL_1545258	EHNV	Laboratory of genomics and metagenomics	Claire Bertelli; Damien Jacot; Gilbert Greub; SÃ©bastien Aeby; Sébastien Aeby; Trestan Pillonel
EPI_ISL_1406674	EHPAD	CHU Clermont-Ferrand, service de virologie	Bisseux Maxime; Combes Patricia; Henquell Cécile; Mirand Audrey
EPI_ISL_418226	EHPAD - Résidences les Cèdres	National Reference Center for Viruses of Respiratory Infections, Institut Pasteur, Paris	Angela Brisebarre; Etienne Simon-Lorière; Flora Donati; Marion Barbet; Maud Vanpeene; Mélanie Albert; Méline Bizard; Sylvie Behillil; Sylvie van der Werf; Vincent Enouf
EPI_ISL_1433876, EPI_ISL_1433878, EPI_ISL_1434002, EPI_ISL_1434004, EPI_ISL_1434006, EPI_ISL_1434223, EPI_ISL_1434254, EPI_ISL_1457759			
see above	EPSILAB TALON VEZERONCE CURTIN	CNR Virus des Infections Respiratoires - France SUD	Antonin Bal; Bruno Lina; Gregory Destras; Gwendolyne Burfin; Hadrien Regue; Laurence Josset; Martine Valette; Quentin Semanas
EPI_ISL_1494949	ESE. SAN ISIDRO DEL MUNICIPIO DE TONA	Instituto Nacional de Salud- Dirección de Investigación en Salud Pública	Carlos Franco-Muñoz; Carmen Osorio; Diana Malo; Diego A. Álvarez-Díaz; Diego Andrés Prada; Gerardo Santamaría; Hector Alejandro Ruiz-Moreno; Jhonnatan Reales-González; Juan Camilo Martinez; Julian Naizaque; Katherine Laiton-Donato; Lisseth Pardo; Magdalena Wiesner; Marcela Mercado-Reyes; Maria T. Herrera-Sepúlveda; Marta Lopez Blanco; Martha Lucia Ospina Martinez; Paola Rojas; Sergio Gomez; Sheryll Corchuelo; Ángela Alarcon Cruz
EPI_ISL_515943 to 515944	ESIC	Department of Neurovirology, National Institute of Mental Health and Neuroscience (NIMHANS)	Anita Desai; Chitra Pattabiraman; Harsha PK; Manjunatha Venkataswamy; Pramada Prasad; Ravi Vasanthapuram; Risha Rasheed; Shafeeq S Hameed; Vijayalakshmi Reddy
EPI_ISL_1400801	ETSU Clinical Lab	Baylor Esoteric + Molecular Lab	Dawn Richards; Elizabeth Forrester
EPI_ISL_1418205, EPI_ISL_1418207	EUROFINS CBM 69 LA SOIE	CNR Virus des Infections Respiratoires - France SUD	Antonin Bal; Bruno Lina; Gregory Destras; Gwendolyne Burfin; Hadrien Regue; Laurence Josset; Martine Valette; Quentin Semanas
EPI_ISL_593481, EPI_ISL_593483, EPI_ISL_593486 to 593487, EPI_ISL_593489 to 593499, EPI_ISL_593501 to 593504, EPI_ISL_593506 to 593509, EPI_ISL_593511 to 593515, EPI_ISL_593517 to 593519, EPI_ISL_593523 to 593529, EPI_ISL_593531 to 593534, EPI_ISL_593536 to 593541, EPI_ISL_593543 to 593551			
see above	Eastern Ontario Regional Laboratory Association	McMaster University	Ahmed Draia; Andrew G. McArthur; Emily Panousis; Hooman Derakhshani; Jalees Nasir; Leanne Mortimer; Robert Slinger
EPI_ISL_437349	Ecole nationale vétérinaire d'Alfort-laboratoire de santé animale Anses UMR 1161 de virologie ENVA-Anses-INRAE	Institut Pasteur CIBU-ERI	Bernard Klonjkowski; Corinne Sailleau; Marine Dumarest; Sophie Le Poder; Stéphan Zientara
EPI_ISL_468044 to 468062, EPI_ISL_475722 to 475724, EPI_ISL_477161, EPI_ISL_478672, EPI_ISL_479686 to 479735, EPI_ISL_524426 to 524427, EPI_ISL_529141 to 529145			
see above	Egyptian National Cancer Institute (ENCI)	Egyptian National Cancer Institute (ENCI)	A.A.; A.N.; Abdel Rahman N; Abdel Rahman N.; Abdelhamid, W.; Abouelhoda; Abouelhoda, M.; Ahmed; Ali; Ali, M.; Amer; Bahnassy; Elkhateeb; Elsissy; Ezzelarab; Gad, A.; H.K.; Hafez; Hamdy; Hassan; Hassan, W.; K.E.; Khattab; M.A.; M.H.; M.M.; M.S.; Mahmoud; Mohamed; O.S.; Raouf, A.; S.M.; Samir, M.; Soliman; W.A.; Zekri
EPI_ISL_469275	Egyptian National Cancer Institute (ENCI)	Human Genome Center	A.A.; Abdel Rahman N; Abdelhamid, W.; Abouelhoda; Ahmed; Ali, M.; Amer; Bahnassy; Elkhateeb; Elsissy; Ezzelarab; Gad, A.; H.K.; Hafez; Hamdy; Hassan, W.; K.E.; M.H.; M.M.; M.S.; Mohamed; O.S.; Raouf, A.; S.M.; Samir, M.; Soliman; Zekri
EPI_ISL_434652	Ektorps Vardcentral	The Public Health Agency of Sweden	Anna Risberg; Anna-Malin Linde; Eva Espmark; Karin Tegmark-Wisell; Maria Lind Karlberg; Mia Brytting; Olov Svartstrom; Oskar Karlsson Lindsjo; Theresa Enkirch
EPI_ISL_1483789 to 1483794, EPI_ISL_1483800 to 1483801, EPI_ISL_1532124 to 1532126	El Camino Hospital	Santa Clara County Public Health Laboratory	Santa Clara County Public Health Department
EPI_ISL_1495719 to 1495748, EPI_ISL_1495920	Elling group, Institute of Molecular Biotechnology (IMBA)	Bergthaler laboratory, CeMM Research Center for Molecular	Andreas Bergthaler; Anna Schedl; Bekir Erguner; Benedikt Agerer; Christoph Bock; Fabian Amman; Jan Laine; Lukas Endler; Maelle Le Moing; Martin

to 1495969		Medicine of the Austrian Academy of Sciences	Senekowitsch; Michael Schuster; Petr Triska; Thomas Penz
EPI_ISL_1398364, EPI_ISL_1398925, EPI_ISL_1398937	Emam Ali Hospital	Razi Vaccine and Serum Research Institute	Akbar Khorasani; Amir Kaffashi; Jiabin Huang; Mohammad Hossein Fallah Mehrabadi; Mohsen Bashashati; Mohsen Lotfi; Morteza Taghizadeh; S Reza Banihashemi; Sayed Hamidreza Mozhgan; Sayed Hamidreza Mozhgani
EPI_ISL_486854	Emergency County Hospital Suceava	Stefan cel Mare, University Metagenomics lab	Lobiuc Andrei et al.
EPI_ISL_486855	Emergency county Hospital Suceava	"Stefan cel Mare" University Metagenomics Lab	Lobiuc Andrei et al.
EPI_ISL_454690, EPI_ISL_455355 to 455361, EPI_ISL_1503955 to 1503970, EPI_ISL_1503972 to 1503978	Emory Molecular Diagnostics Laboratory, Emory Healthcare	Piantadosi Lab, Emory Department of Pathology	Ahmed Babiker; Anne Piantadosi
EPI_ISL_424350 to 424351, EPI_ISL_468718	Environmental and Global Health	Environmental and Global Health	Alam, M.; C.J.; Elbadry; Gibson; J.A.; J.C.; J.G.; J.G. Jr. and Lednicky; L.H.; M.A.; Merck; Morris; Stephenson; Subramaniam, K.; T.B.; Waltzek
EPI_ISL_524433 to 524434	Environmental and Global Health, University of Florida - Gainesville	University of Florida	Alam; C.J.; Elbadry; Gibson; J.A.; J.C.; J.G. Jr.; Lednicky; M.A.; M.M.; Morris; Stephenson; Subramaniam, K.; T.B.; Waltzek
EPI_ISL_1400985 to 1401069, EPI_ISL_1444909 to 1445019	Erie County Public Health (ECPHL)	University at Buffalo Genomics and Bioinformatics Core	Alyssa Pohlman; Amanda Boccolucci; Brandon Marzullo; Donald Yergeau; Jennifer Surtees; Jonathan Bard; Natalie Lamb; Norma Nowak
EPI_ISL_514648	Essentia Health-St. Joseph's Medical Center	Minnesota Department of Health, Public Health Laboratory	Jacob Garfin; Matt Plumb; and Xiong Wang
EPI_ISL_514642, EPI_ISL_603004 to 603005, EPI_ISL_603012 to 603014, EPI_ISL_603016 to 603017	Essentia Health-St. Mary's Medical Center	Minnesota Department of Health, Public Health Laboratory	Alexandra Lorentz; Jacob Garfin; Matt Plumb; and Xiong Wang
EPI_ISL_1479139	Essesntia Health- Holy Trinity Hospital	Minnesota Department of Health, Public Health Laboratory	Alexandra Lorentz; Jacob Garfin; Matt Plumb; and Xiong Wang
EPI_ISL_614265 to 614280	Eurofins	National Reference Center for Viruses of Respiratory Infections, Institut Pasteur, Paris	Angela Brisebarre; Camille Capel; Etienne Simon-Lorière; Marion Barbet; Maud Vanpeene; Méline Bizard; Sylvie Behillil; Sylvie van der Werf; Vincent Enouf
EPI_ISL_1404923 to 1404996, EPI_ISL_1532585 to 1532651	Eurofins Diatherix	Hudsonalpha Genome Sequencing Center	Ada Stewart; Jane Grimwood; Jenell Webber; Jennifer Cart; John Lovell; Joshua Stough; Leslie Malone; Lori H. Handley; Melissa Williams; Stefan Brzezinski; Teresa Jones; and Jeremy Schmutz
EPI_ISL_1432751, EPI_ISL_1432753, EPI_ISL_1432755, EPI_ISL_1432757, EPI_ISL_1432760, EPI_ISL_1432762, EPI_ISL_1432764, EPI_ISL_1432766, EPI_ISL_1432768, EPI_ISL_1432770, EPI_ISL_1432772, EPI_ISL_1432774, EPI_ISL_1432777, EPI_ISL_1432779, EPI_ISL_1432781, EPI_ISL_1432783, EPI_ISL_1432785, EPI_ISL_1432787, EPI_ISL_1432790, EPI_ISL_1432792, EPI_ISL_1432794, EPI_ISL_1432796, EPI_ISL_1432799 to 1432800, EPI_ISL_1432803, EPI_ISL_1432805, EPI_ISL_1432807, EPI_ISL_1432809, EPI_ISL_1432814, EPI_ISL_1432816, EPI_ISL_1432818, EPI_ISL_1432820, EPI_ISL_1432822, EPI_ISL_1432824, EPI_ISL_1432827, EPI_ISL_1432829, EPI_ISL_1432831, EPI_ISL_1432833, EPI_ISL_1432836, EPI_ISL_1432838, EPI_ISL_1432840, EPI_ISL_1432843 to 1432844, EPI_ISL_1432847, EPI_ISL_1432849, EPI_ISL_1432851, EPI_ISL_1432853, EPI_ISL_1432855, EPI_ISL_1432857, EPI_ISL_1432859, EPI_ISL_1432861, EPI_ISL_1432864, EPI_ISL_1432866, EPI_ISL_1432869, EPI_ISL_1432871, EPI_ISL_1432873, EPI_ISL_1432875, EPI_ISL_1432878, EPI_ISL_1432880, EPI_ISL_1432882, EPI_ISL_1432884, EPI_ISL_1432886, EPI_ISL_1432889, EPI_ISL_1432891, EPI_ISL_1432893, EPI_ISL_1432895, EPI_ISL_1432898, EPI_ISL_1432902, EPI_ISL_1432905, EPI_ISL_1432907, EPI_ISL_1432909, EPI_ISL_1432911, EPI_ISL_1432915, EPI_ISL_1432918, EPI_ISL_1432920, EPI_ISL_1432922, EPI_ISL_1432924			
see above	Eurofins LifeCodexx GmbH	Robert Koch Institute	
EPI_ISL_450873 to 450874	Evandro Chagas Institute	Evandro Chagas Institute	A.M.; Barbagelata; E.C.; E.M.A.; Ferreira; G.M.R; J.A.; Junior; L.C.; L.S.; M.C.; Martins; P.S.; Santos; Silva; Sousa; Sousa Junior; Viana; W.D.C.; da Silva
EPI_ISL_602282 to 602303	Evangelisches Klinikum Bethel, Institut für Laboratoriumsmedizin, Mikrobiologie und Hygiene	Bielefeld University	Alexander Sczyrba; Christiane Scherer; David Brandt; Jörn Kalinowski; Levin-Joe Klages; Marina Simunovic; Markus Haak; Svenja Vinke; Tobias Busche
EPI_ISL_548966 to 548971	Expo2020 Emergency Center	Agiomix	Cengiz Yakicier; Cherif Ben Hamada; Rashid Mohammed; Tamer Degheidy; Walaa Allam; Walid Dridi
EPI_ISL_1446999 to 1447001, EPI_ISL_1447047, EPI_ISL_1528314 to 1528320	FL Bur. of Public Health Laboratories-Jacksonville	Centers for Disease Control and Prevention Division of Viral Diseases, Pathogen Discovery	Alison Laufer Halpin; Ben L. Rambo-Martin; Clinton R. Paden; Dakota Howard; Darlene Wagner; Dave Wentworth; Dhwani Batra; Jasmine Padilla; Justin Lee; Katie Dillon; Krista Queen; Kristen Knipe; Kristine Lacek; Mark Burroughs; Matthew Schmerer; Mili Sheth; Peter Cook; Sam Shepard; Sarah Nobles; Shoshona Le; Suxiang Tong; Vivien Dugan; Yvette Unoarumhi
EPI_ISL_424856 to 424857	FL Bur. of Public Health Laboratories-Jacksonville	Pathogen Discovery, Respiratory Viruses Branch, Division of Viral Diseases, Centers for Disease Control and Prevention	Alison S. Laufer Halpin; Anna Uehara; Christopher A. Elkins; Clinton R. Paden; Haibin Wang; Jing Zhang; Krista Queen; Mary S. Keckler; Rachel Marine; Suxiang Tong; Yan Li; Ying Tao
EPI_ISL_420785	FL Bureau of Health Laboratories Tampa	Pathogen Discovery, Respiratory Viruses Branch, Division of Viral Diseases, Centers for Disease Control and Prevention	Alison S. Laufer Halpin; Anne Uehara; Christopher A. Elkins; Clinton R. Paden; Haibin Wang; Jasmine Padilla; Jing Zhang; Justin Lee; Krista Queen; Mary S. Keckler; Rachel Marine; Suxiang Tong; Yan Li; Ying Tao
EPI_ISL_452110 to 452111, EPI_ISL_452132, EPI_ISL_594461, EPI_ISL_594465	FL Bureau of Public Health Laboratories	Pathogen Discovery, Respiratory Viruses Branch, Division of Viral Diseases, Centers for Disease Control and Prevention	Alison S. Laufer Halpin; Anna Montmayeur; Anna Uehara; Christopher A. Elkins; Clinton Paden; Clinton R. Paden; Haibin Wang; Jing Zhang; Julu Bhatnagar; Krista Queen; Mary S. Keckler; Rachel Marine; Suxiang Tong; Yan Li; Ying Tao; Zachary Weiner
EPI_ISL_1447054	FL Bureau of Public Health Laboratories-Miami	Centers for Disease Control and Prevention Division of Viral Diseases, Pathogen Discovery	Alison Laufer Halpin; Ben L. Rambo-Martin; Clinton R. Paden; Dakota Howard; Darlene Wagner; Dave Wentworth; Dhwani Batra; Jasmine Padilla; Justin Lee; Katie Dillon; Krista Queen; Kristen Knipe; Kristine Lacek; Mark Burroughs; Matthew Schmerer; Mili Sheth; Peter Cook; Sam Shepard; Sarah Nobles; Shoshona Le; Suxiang Tong; Vivien Dugan; Yvette Unoarumhi
EPI_ISL_424853 to 424854	FL Bureau of Public Health Laboratories-Miami	Pathogen Discovery, Respiratory Viruses Branch, Division of Viral Diseases, Centers for Disease Control and Prevention	Alison S. Laufer Halpin; Anna Uehara; Christopher A. Elkins; Clinton R. Paden; Haibin Wang; Jing Zhang; Krista Queen; Mary S. Keckler; Rachel Marine; Suxiang Tong; Yan Li; Ying Tao
EPI_ISL_1446987 to 1446988, EPI_ISL_1528284 to 1528285	FL Bureau of Public Health Laboratories-Tampa	Centers for Disease Control and Prevention Division of Viral Diseases, Pathogen Discovery	Alison Laufer Halpin; Ben L. Rambo-Martin; Clinton R. Paden; Dakota Howard; Darlene Wagner; Dave Wentworth; Dhwani Batra; Jasmine Padilla; Justin Lee; Katie Dillon; Krista Queen; Kristen Knipe; Kristine Lacek; Mark Burroughs; Matthew Schmerer; Mili Sheth; Peter Cook; Sam Shepard; Sarah Nobles; Shoshona Le; Suxiang Tong; Vivien Dugan; Yvette Unoarumhi
EPI_ISL_419559 to 419560, EPI_ISL_424855, EPI_ISL_447841	FL Bureau of Public Health Laboratories-Tampa	Pathogen Discovery, Respiratory Viruses Branch, Division of Viral Diseases, Centers for Disease Control and Prevention	Alison S. Laufer Halpin; Anna Uehara; Christopher A. Elkins; Clinton R. Paden; Haibin Wang; Jasmine Padilla; Jing Zhang; Justin Lee; Krista Queen; Mary S. Keckler; Rachel Marine; Suxiang Tong; Yan Li; Ying Tao
EPI_ISL_576173	FL Department of Health-Bureau of Epidemiology	Pathogen Discovery, Respiratory Viruses Branch, Division of Viral Diseases, Centers for Disease Control and Prevention	Anna Uehara; Brian Lynch; Clinton R. Paden; Haibin Wang; Jing Zhang; Krista Queen; Peter Cook; Suxiang Tong; Yan Li; Ying Tao
EPI_ISL_428851 to 428852	FSBSI "Chumakov Federal Scientific Center for Research and Development of Immune-and-Biological Products of Russian Academy of Sciences"	FSBSI "Chumakov Federal Scientific Center for Research and Development of Immune-and-Biological Products of Russian Academy of Sciences" & NRC "Kurchatov institute"	Anastasia Berestovskaya; Anastasia Piniaeva; Anna Shishova; Aydar Ishmukhametov; Denis Protsenko; Egor Prokhorchuk; Georgy Ignatyev; Liubov Kozlovskaya; Mikhail Rychev
EPI_ISL_1469555, EPI_ISL_1469572, EPI_ISL_1469612, EPI_ISL_1469620, EPI_ISL_1469630, EPI_ISL_1469651, EPI_ISL_1469661, EPI_ISL_1469785			
see above	FUNDACAO DE SAUDE PUBLICA DE NOVO HAMBURGO FSNH	Epiclin	Ana Paula Muterle; Carolina Comerlato; Eliana Márcia Da Ros Wendland; Fernando Hayashi Sant'Anna; Janira Prichula; Juliana Comerlato
EPI_ISL_1469665, EPI_ISL_1469689, EPI_ISL_1469693, EPI_ISL_1469704, EPI_ISL_1469723	FUNDACAO DE SAUDE PUBLICA SAO CAMILO DE ESTEIO	Epiclin	Ana Paula Muterle; Carolina Comerlato; Eliana Márcia Da Ros Wendland; Fernando Hayashi Sant'Anna; Janira Prichula; Juliana Comerlato
EPI_ISL_1469549	FUNDACAO HOSPITALAR DE SAPUCAIA DO SUL	Epiclin	Ana Paula Muterle; Carolina Comerlato; Eliana Márcia Da Ros Wendland; Fernando Hayashi Sant'Anna; Janira Prichula; Juliana Comerlato
EPI_ISL_1469586	FUNDACAO HOSPITALAR SAO JOSE	Epiclin	Ana Paula Muterle; Carolina Comerlato; Eliana Márcia Da Ros Wendland; Fernando Hayashi Sant'Anna; Janira Prichula; Juliana Comerlato
EPI_ISL_445277	FUNDACION DE SALUD EL TENIENTE	Instituto de Salud Publica de Chile	Alejandra Acevedo; Andrés E Castillo; Bárbara Parra; Carolina Tambley; Gabriel Leal; Jaime Lagos; Jorge Fernandez; Loredana Arata; Patricia Bustos; Paz Tapia; Rodrigo Fasce; Winston Andrade
EPI_ISL_417010, EPI_ISL_418252	FUNDACION JIMENEZ DIAZ	Instituto de Salud Carlos III	A. Monzón; F. Casas; I. Fernández Roblas, R.; I. Jiménez; Iglesias-Caballero; M. Camarero; M. Cuesta; M. González-Esguevillas; M. Molinero Calamita; M. Zaballos; P. Jiménez; S. Juliá; S. Pozo; S. Varona
EPI_ISL_468747 to 468751	Facultad de Medicina UC	Center for Mathematical Modeling and Center for Genome Regulation. Santiago, Chile	Allende ML; Ferres M.; Gaete A; González M; Maass A; Palma R; Travisany D; Urra C; Varas M

EPI_ISL_1491576 to 1491583, EPI_ISL_1538420, EPI_ISL_1538429 to 1538431, EPI_ISL_1545668	Faculty of Medicine Vajira Hospital	COVID-19 Network Investigations (CONI) Alliance	Ampan Vimonvattana; Anan Manomaipiboon; Angkana Huang; Anthony R. Jones; Arporn Wangwiwatsin; Bhakbhoom Panthan; Chayanit Phutthanu; Chonticha Klungtong; Duangkamon Loesbanluechai; Ekawat Pasomsub; Elizabeth Batty; Insee Sensorn; Jakravoot Maneerit; Janjira Thaipadungpanit; Khajohn Joonlasak; Kingkan Rakmanee; Krittikorn Kumpornsin; Namfon Kotanan; Stefan Fernandez; Sunisa Dongphooyao; Thanat Chookajorn; Theerarat Kochakarn; Tonsan Hansirisathit; Treewat Watthanachockchai; Uraporn Phumisantiphong; Wasun Chantratita; Wipawee Thongsopa; Wudtichai Manasatienkij
EPI_ISL_510533 to 510534	Faculty of Medicine, Bursa Uludag University	Bursa Uludag University	Alemdar, A.; S.G.; Saglik, I.; Temel; Özemri Sa, .
EPI_ISL_526686 to 526688, EPI_ISL_548243 to 548244	Faith Laboratory, Immunology Institute, Icahn School of Medicine at Mount Sinai	van Bakel Laboratory, Genetics and Genomics Sciences, Icahn School of Medicine at Mount Sinai	Alexandra Livanos; Alice Chen-Liaw; Ana S. Gonzalez-Reiche; Benjamin R. tenOever; Daisy Hoagland; Divya Jha; Fatima Amanat; Florian Krammer; Francesca Cossarini; Gerold Bongers; Graham J. Britton; Gustavo Martinez-Delgado; Harm Van Bakel; Ilaria Mogno; Joseph Eggers; Lauren Tal Grinspan; Marla C. Dubinsky; Matthew P. Spindler; Michael Tankelevich; Miriam Merad; Rebekah E. Dixon; Saurabh Mehandru and Jeremiah J. Faith; Sophia Sui; Tamar Plitt
EPI_ISL_1416191	Fakultas Kedokteran Universitas Sumatera Utara	National Institute of Health Research and Development	Arie Ardiansyah Nugraha; Hana Apsari Pawestri; Hartanti Dian Ikawati; Kartika Dewi Puspa; Krisna Nur Andriana Pangesti; Nelly Puspandari; Subangkit; Vivi Setiawaty
EPI_ISL_1534701 to 1534703	Fakultas kedokteran Universitas Sumatra Utara	National Institute of Health Research and Development	Arie Ardiansyah Nugraha; Hana Apsari Pawestri; Hartanti Dian Ikawati; Kartika Dewi Puspa; Krisna Pangesti; Nelly Puspandari; Subangkit; Vivi Setiawaty
EPI_ISL_1534260, EPI_ISL_1534262, EPI_ISL_1534266, EPI_ISL_1534278, EPI_ISL_1534307, EPI_ISL_1534331, EPI_ISL_1534380, EPI_ISL_1534400, EPI_ISL_1534442, EPI_ISL_1534461			
see above	False Bay Hospital wc FBH	NHLS/UCT	Arash Iranzadeh; Bruna Galvao; Carolyn Williamson; Deelan Doolabh; Diana Hardie; Emmanuel SJ; Innocent Mudau; Kruger Marais; Lynn Tyers; Marvin Hsiao; Stephen Korsman; Tegally H; de Oliveira T
EPI_ISL_536400	Fareham Creek Veterinary Surgery	MRC-University of Glasgow Centre for Virus Research	Ana Da Silva Filipe; Andrew Stevenson; Brian J Willett; Bryn Tennant; Daniel G Streicker; Daniel Goldfarb; Darcy Herrity; David L Robertson and William Weir; Dawn Dunbar; Emma MacDonald; Fiona Howie; Frazer Bell; Ilaria Epifano; Lynn Stevenson; Margaret J Hosie; Michael McDonald; Natasha Johnson; Pablo R Murcia; Richard J Orton; Ruth F Jarrett; Vanessa Herder
EPI_ISL_526935 to 526948, EPI_ISL_614295 to 614317	Faroese National Reference Laboratory for Fish and Animal Diseases	Faroese National Reference Laboratory for Fish and Animal Diseases	Debes Hammershaimb Christiansen; Maria Marjunardóttir Dahl; Petra Elisabeth Petersen
EPI_ISL_451963 to 451970	Federal Budget Institution of Science, State Research Center for Applied Microbiology & Biotechnology	Federal Budget Institution of Science, State Research Center for Applied Microbiology & Biotechnology	Abaimova A; Bakhteeva I; Blagodatskikh S; Bogun A; Borzilov A; Chekan L; Chernysh S; Denisenko E; Dentovskaya S; Detushev K; Detusheva E; Dyatlov I; Firstova V; Frolov V; Fursov M; Fursova N; Galkina E; Gapelchenkova T; Goncharova J; Gorbatov A; Hlyntseva A; Ivanov S; Kalmantayev T; Kalmantayeva O; Kanashenko M; Kartsev N; Kartseva A; Khomyakov A; Khramov M; Kislichkina A; Kolchanova A; Koroleva-Ushakova A; Kosilova I; Krasilnikova E; Kuzin V; Kuzina E; Makarova M; Marin M; Novikova T; Platonov M; Podkopaev Y; Ryabko A; Shaikhutdinova R; Shemyakin I; Shishkina L; Silkina M; Sizova A; Skryabin Y; Slukin P; Slukina N; Solomentsev V; Solovieva A; Teymurazov M; Timofeev V; Titareva G; Trunyakova A; Tyurin E; Vagayskaya A; Zeninskaya N; Zhumakaev R
EPI_ISL_436505 to 436563, EPI_ISL_445003 to 445050, EPI_ISL_450844 to 450872, EPI_ISL_467667, EPI_ISL_480790 to 480951, EPI_ISL_489710 to 489832, EPI_ISL_495330 to 495410, EPI_ISL_508708 to 508763, EPI_ISL_508765 to 508809, EPI_ISL_508811 to 508861, EPI_ISL_509716 to 509798, EPI_ISL_512107, EPI_ISL_512547 to 512596, EPI_ISL_512676 to 512677, EPI_ISL_514139 to 514225, EPI_ISL_517771 to 517957, EPI_ISL_526553 to 526685, EPI_ISL_541338, EPI_ISL_549178 to 549270, EPI_ISL_568587 to 568685, EPI_ISL_568876, EPI_ISL_594277 to 594393, EPI_ISL_594466, EPI_ISL_613712 to 613950, EPI_ISL_614247, EPI_ISL_1530990 to 1531416, EPI_ISL_1559588 to 1559591			
see above	Florida Bureau of Public Health Laboratories	Florida Bureau of Public Health Laboratories	Jason Blanton; S. and Blanton, J.; Sarah Schmedes; Schmedes
EPI_ISL_541139 to 541331	Florida Bureau of Public Health Laboratories, Florida Department of Health	Florida Bureau of Public Health Laboratories, Florida Department of Health	Blanton, J.; Schmedes, S.
EPI_ISL_1497542	Flow Diagnostics Lab	Los Angeles County PHL	P. Hemarajata et al.
EPI_ISL_1413763, EPI_ISL_1413766, EPI_ISL_1516746	Flow Health	Infectious Disease Program, Broad Institute of Harvard and MIT	Adams, G.; B.L.; B.W.; Bauer, M.; Birren; Carter, A.; Chaluvadi, S.; D.J.; DeRuff, K.; Gallagher, G.; Gladden-Young, A.; J.E.; K.J.; Lagerborg, K.; Lemieux; Loreth, C.; MacInnis; Normandin, E.; P.C.; Park; Reilly, S.; Rudy, M.; Siddle; Smole, S.; Tomkins-Tinch, C.; and Sabeti
EPI_ISL_470882, EPI_ISL_500776 to 500778, EPI_ISL_590883 to 590884, EPI_ISL_590899, EPI_ISL_590981, EPI_ISL_590985, EPI_ISL_591008, EPI_ISL_1490307, EPI_ISL_1490645, EPI_ISL_1547638 to 1547643			
see above	Foerde Hospital, Department of Microbiology	Norwegian Institute of Public Health, Department of Virology	Atiya R Ali; Debech Nadia; Engebretsen Serina Beate; Garcia Llorente Ignacio; Hilde Elshaug; Hilde Vollan; Jon Bråte; Kamilla Heddeland Instefjord; Karoline Bragstad; Kathrine Stene-Johansen; Marie Paulsen Madsen; Olav Hungnes; Pedersen Benedikte Nevjen; Rasmus Riis Kopperud
EPI_ISL_455903, EPI_ISL_475152, EPI_ISL_476139	Folkhalsomyndigheten	The Public Health Agency of Sweden	Anna Risberg; Anna-Malin Linde; Karin Tegmark-Wisell; Maria Lind Karlberg; Mattias Haukland; Olov Svartstrom; Oskar Karlsson Lindsjo; Petra Edquist; Reza Advani; Sandra Broddesson; Shamam Muradrasoli
EPI_ISL_434650, EPI_ISL_475536, EPI_ISL_475541	Follinge Halsocentral	The Public Health Agency of Sweden	Anna Risberg; Anna-Malin Linde; Karin Tegmark-Wisell; Kerstin Persson Moberg; Maria Lind Karlberg; Mattias Haukland; Mia Brytting; Olov Svartstrom; Oskar Karlsson Lindsjo; Reza Advani; Sandra Broddesson; Theresa Enkirch
EPI_ISL_581455, EPI_ISL_581487, EPI_ISL_581489 to 581493	Fondation Congolaise pour la recherche medicale (FCRM)	NGS Competence Center Tübingen, Institut für Medizinische Mikrobiologie und Hygiene, Universitätsklinikum Tübingen	Angel Angelov
EPI_ISL_420145	Forde Hospital Department of Microbiology	Norwegian Institute of Public Health, Department of Virology	Hilde Elshaug; Kamilla Heddeland Instefjord; Karoline Bragstad; Kathrine Stene-Johansen; Olav Hungnes
EPI_ISL_413565	Foundation Pamm	Erasmus Medical Center	Anne van der Linden; Annemiek van der Eijk; Aura Timen; Bas Oude Munnink; Claudia Schapendonk; Corien Swaan; Corine GeurtsvanKessel; David Nieuwenhuijse; Irina Chestakova; Jeroen van Kampen; Jolanda Voermans; Madelief Mollers; Manon Haverkate; Marion Koopmans; Mark Pronk; Mart Stein; Pascal Lexmond; Reina Sikkema; Richard Molenkamp; Sandra Kengne Kamga Mobou; on behalf of the Dutch national COVID-19 response team.
EPI_ISL_411060, EPI_ISL_411066, EPI_ISL_431118, EPI_ISL_431180, EPI_ISL_431240, EPI_ISL_431292, EPI_ISL_431779 to 431785			
see above	Fujian Center for Disease Control and Prevention	Fujian Center for Disease Control and Prevention	Chen Wei; He Wenxiang; Huang Zhimiao; Lin Qi; Weng Yuwei; Zhang Yanhua
EPI_ISL_479967 to 479978, EPI_ISL_480120 to 480168, EPI_ISL_480225	Fukui Prefectural Institute of Public Health and Environmental Science	Pathogen Genomics Center, National Institute of Infectious Diseases	Hajime Kamiya; Kentaro Itokawa; Makoto Kuroda; Masanori Hashino; Miho Toho; Motoi Suzuki; Rina Tanaka; Tsuyoshi Sekizuka
EPI_ISL_1472113 to 1472273, EPI_ISL_1472338 to 1472339	Fukuoka Institute of Health and Environmental Sciences	Fukuoka Institute of Health and Environmental Sciences	Akira Ohishi; Asako Nakamura; Chiharu Katamune; Hiroaki Shigemura; Kentaro Itokawa; Makoto Kuroda; Masanori Hashino; Mitsuhiro Hamasaki; Rina Tanaka; Saori Ueda; Takayuki Kobayashi; Tsuyoshi Sekizuka; Yoshiki Etoh; Yuki Ashizuka
EPI_ISL_1457792 to 1458819, EPI_ISL_1458844 to 1458845, EPI_ISL_1458847, EPI_ISL_1458850 to 1458859, EPI_ISL_1458900 to 1458939, EPI_ISL_1458965, EPI_ISL_1458968, EPI_ISL_1458970 to 1458979, EPI_ISL_1459012 to 1459013, EPI_ISL_1459016 to 1459029, EPI_ISL_1459070 to 1459109, EPI_ISL_1459133 to 1459134, EPI_ISL_1459137 to 1459149, EPI_ISL_1459190 to 1459229, EPI_ISL_1459256 to 1459269, EPI_ISL_1459292 to 1459508, EPI_ISL_1459529 to 1460729, EPI_ISL_1481549 to 1482273, EPI_ISL_1525267 to 1525903, EPI_ISL_1554950 to 1554951, EPI_ISL_1554953 to 1554954, EPI_ISL_1554956 to 1554957, EPI_ISL_1554959 to 1554960, EPI_ISL_1554962 to 1555117, EPI_ISL_1555119, EPI_ISL_1555121 to 1555289, EPI_ISL_1555291 to 1555382, EPI_ISL_1555384 to 1555625, EPI_ISL_1555628 to 1555708, EPI_ISL_1555710 to 1556023, EPI_ISL_1556025 to 1556110, EPI_ISL_1556112 to 1556400, EPI_ISL_1556402 to 1556416, EPI_ISL_1556418 to 1556827, EPI_ISL_1556829 to 1556909, EPI_ISL_1556911 to 1556914, EPI_ISL_1556916 to 1557001, EPI_ISL_1557003 to 1557066, EPI_ISL_1557068 to 1557218			
see above	Fulgent Genetics	Centers for Disease Control and Prevention Division of Viral Diseases, Pathogen Discovery	Adrian Paskey; Becky Tsai; Benafsh Sapra; Benjamin Rambo-Martin; Christopher Gulvick; Clinton R. Paden; Dakota Howard; Darlene Wagner; Dhwani Batra; Doreen Ng; Duncan MacCannell; Harry Gao; James Xie; Jason Caravas; John Gao; Joseph Fierro; Kara Moser; Matthew Schmerer; Mickey Li; Peter W. Cook; Scott Sammons; Shatavia Morrison; Yan Meng; Yvette Unoarumhi
EPI_ISL_492074	Functional Genomics Core University of South Carolina / Prisma Health-Midlands	Functional Genomics Core, University of South Carolina,	Alyssa Clay-Glimour; B.Celia Cui; Diego Altomare; Hao Ji; Helmut Albrecht; Mengqian Chen; Michael Shtutman; Michael Wyatt; Phillip Buckhaults
EPI_ISL_491481	Functional Genomics Core University of South Carolina / Prisma Health-Midlands	Functional Genomics Core, Center For Targeted Therapeutics,	Alyssa Clay-Glimour; B.Celia Cui; Diego Altomare; Hao Ji; Helmut Albrecht; Mengqian Chen; Michael Shtutman; Michael Wyatt; Phillip Buckhaults
EPI_ISL_493209 to 493212, EPI_ISL_493427, EPI_ISL_494553 to 494556, EPI_ISL_494558	Functional Genomics Core University of South Carolina / Prisma Health-Midlands	Functional Genomics Core, University of South Carolina	Alyssa Clay-Glimour; B.Celia Cui; Diego Altomare; Hao Ji; Helmut Albrecht; Mengqian Chen; Michael Shtutman; Michael Wyatt; Phillip Buckhaults
EPI_ISL_493129 to 493130, EPI_ISL_493426	Functional Genomics Core University of South Carolina / Prisma Health-Midlands	Functional Genomics Core, University of South Carolina,	Alyssa Clay-Glimour; B.Celia Cui; Diego Altomare; Hao Ji; Helmut Albrecht; Mengqian Chen; Michael Shtutman; Michael Wyatt; Phillip Buckhaults
EPI_ISL_1494951	Fundacion Cardioinfantil	Instituto Nacional de Salud- Dirección de Investigación en Salud Pública	Carlos Franco-Muñoz; Carmen Osorio; Diana Malo; Diego A. Álvarez-Díaz; Diego Andrés Prada; Gerardo Santamaría; Hector Alejandro Ruiz-Moreno; Jhonnatan Reales-González; Juan Camilo Martinez; Julian Naizaque; Katherine Laiton-Donato; Lisseth Pardo; Magdalena Wiesner; Marcela

			Mercado-Reyes; Maria T. Herrera-Sepúlveda; Marta Lopez Blanco; Martha Lucia Ospina Martinez; Paola Rojas; Sergio Gomez; Sheryll Corchuelo; Ángela Alarcon Cruz
EPI_ISL_419235 to 419237	Fundacion Jimenez Diaz	Instituto de Salud Carlos III	Camarero, S.; Casas, I.; Cuesta, I.; Fernández, R.; González-Esguevillas, M.; Iglesias-Caballero, M.; Jiménez, M.; Jiménez, P.; Juliá, M.; Molinero Calamita, M.; Monzón, S.; Pozo, F.; Varona, S.; Zaballos, A.
EPI_ISL_462447 to 462449	Fundació Lluita contra la SIDA (FLSida)/Hospital Universitari Germans Trias i Pujol	IrsiCaixa AIDS Research Lab	Bonaventura Clotet; Joaquim Segalés; Jorge Carrillo; Julia Blanco; Lidia Ruiz; Marc Corbacho; Marc Noguera-Julian; Maria Pilar Armengol; Maria Ubals; Mariona Parera; Nuria Izquierdo; Oriol Mitjà; Roger Paredes
EPI_ISL_462478	Fundación Jiménez Díaz	Instituto de Salud Carlos III	A. Monzón; F. Casas; I; I. Jiménez; Iglesias-Caballero; M. Camarero; M. Cuesta; M. González-Esguevillas; M. Molinero Calamita; M. Zaballos; P. Jiménez; R. Fernández; S. Juliá; S. Pozo; S. Varona
EPI_ISL_1479122	Fundação Hospitalar São José	Epiclin	Ana Paula Muterle; Carolina Comerlato; Eliana Márcia Da Ros Wendland; Fernando Hayashi Sant'Anna; Janira Prichula; Juliana Comerlato
EPI_ISL_1469585, EPI_ISL_1469606, EPI_ISL_1469617, EPI_ISL_1469619, EPI_ISL_1469654, EPI_ISL_1469713, EPI_ISL_1469724, EPI_ISL_1469752, EPI_ISL_1469787, EPI_ISL_1469805, EPI_ISL_1469809, EPI_ISL_1469817, EPI_ISL_1469831, EPI_ISL_1469845			
see above	Fundação Hospitalar de Sapucaia do Sul	Epiclin	Ana Paula Muterle; Carolina Comerlato; Eliana Márcia Da Ros Wendland; Fernando Hayashi Sant'Anna; Janira Prichula; Juliana Comerlato
EPI_ISL_1469554, EPI_ISL_1469561, EPI_ISL_1469569, EPI_ISL_1469640, EPI_ISL_1469691, EPI_ISL_1469837, EPI_ISL_1469850			
see above	Fundação de Saúde Pública São Camilo de Esteio	Epiclin	Ana Paula Muterle; Carolina Comerlato; Eliana Márcia Da Ros Wendland; Fernando Hayashi Sant'Anna; Janira Prichula; Juliana Comerlato
EPI_ISL_1469595, EPI_ISL_1469597, EPI_ISL_1469613, EPI_ISL_1469622, EPI_ISL_1469626, EPI_ISL_1469653, EPI_ISL_1469673, EPI_ISL_1469767, EPI_ISL_1469770, EPI_ISL_1469800, EPI_ISL_1469826, EPI_ISL_1469828, EPI_ISL_1479123, EPI_ISL_1479131 to 1479132			
see above	Fundação de Saúde Pública de Novo Hamburgo	Epiclin	Ana Paula Muterle; Carolina Comerlato; Eliana Márcia Da Ros Wendland; Fernando Hayashi Sant'Anna; Janira Prichula; Juliana Comerlato
EPI_ISL_420141, EPI_ISL_420146, EPI_ISL_420313, EPI_ISL_449787 to 449788, EPI_ISL_481208, EPI_ISL_493356 to 493380, EPI_ISL_500768 to 500774, EPI_ISL_500784 to 500790, EPI_ISL_549027 to 549028, EPI_ISL_549038, EPI_ISL_549048 to 549049, EPI_ISL_549053 to 549058, EPI_ISL_549060 to 549069, EPI_ISL_549071 to 549080, EPI_ISL_549082, EPI_ISL_549088, EPI_ISL_549112, EPI_ISL_549119 to 549122, EPI_ISL_549124, EPI_ISL_549132, EPI_ISL_549143, EPI_ISL_549154, EPI_ISL_549165, EPI_ISL_549168, EPI_ISL_590881, EPI_ISL_590898, EPI_ISL_590901 to 590903, EPI_ISL_590912, EPI_ISL_1385977, EPI_ISL_1385980 to 1385983, EPI_ISL_1385985, EPI_ISL_1385988, EPI_ISL_1385992, EPI_ISL_1402968, EPI_ISL_1490261 to 1490262, EPI_ISL_1490281, EPI_ISL_1490287, EPI_ISL_1490295, EPI_ISL_1490297, EPI_ISL_1490311, EPI_ISL_1490315, EPI_ISL_1490320 to 1490323, EPI_ISL_1490325, EPI_ISL_1490330 to 1490331, EPI_ISL_1490340, EPI_ISL_1490342, EPI_ISL_1490344 to 1490345, EPI_ISL_1490347, EPI_ISL_1490350 to 1490353, EPI_ISL_1490366, EPI_ISL_1490398 to 1490399, EPI_ISL_1490413, EPI_ISL_1490415, EPI_ISL_1490421 to 1490422, EPI_ISL_1490428, EPI_ISL_1490430 to 1490431, EPI_ISL_1490453, EPI_ISL_1490455, EPI_ISL_1490464, EPI_ISL_1490466, EPI_ISL_1490468 to 1490469, EPI_ISL_1490471 to 1490472, EPI_ISL_1490474, EPI_ISL_1490476 to 1490477, EPI_ISL_1490480 to 1490491, EPI_ISL_1490502, EPI_ISL_1490506, EPI_ISL_1490511, EPI_ISL_1490515 to 1490516, EPI_ISL_1490534 to 1490540, EPI_ISL_1490542, EPI_ISL_1490544 to 1490547, EPI_ISL_1490549 to 1490553, EPI_ISL_1490564, EPI_ISL_1490574 to 1490575, EPI_ISL_1490582, EPI_ISL_1490600, EPI_ISL_1490606, EPI_ISL_1490619 to 1490620, EPI_ISL_1490623, EPI_ISL_1490625, EPI_ISL_1490634, EPI_ISL_1547521, EPI_ISL_1547523 to 1547532, EPI_ISL_1547553 to 1547554, EPI_ISL_1547596 to 1547601, EPI_ISL_1547606 to 1547607, EPI_ISL_1547610, EPI_ISL_1547614, EPI_ISL_1547617, EPI_ISL_1547635 to 1547637, EPI_ISL_1547663 to 1547664, EPI_ISL_1547667, EPI_ISL_1547669 to 1547670, EPI_ISL_1547672, EPI_ISL_1547674, EPI_ISL_1547677 to 1547678, EPI_ISL_1547683, EPI_ISL_1547688, EPI_ISL_1547690 to 1547691, EPI_ISL_1547693 to 1547704			
see above	Furst Medical Laboratory	Norwegian Institute of Public Health, Department of Virology	Atiya R Ali; Debech Nadia; Engebretsen Serina Beate; Garcia Llorente Ignacio; Hilde Elshaug; Hilde Synnøve Vollan; Hilde Vollan; Jon Bråte; Kamilla Heddeland Instefjord; Karoline Bragstad; Kathrine Stene-Johansen; Marie Paulsen Madsen; Olav Hungnes; Pedersen Benedikte Nevjen; Rasmus Riis Kopperud
EPI_ISL_522549 to 522550	Félix Guyon Hospital	UMR PIMIT Université de La Réunion	Camille Lebarbenchon; David Wilkinson; Patrick Mavingui
EPI_ISL_1404484	G.H.E.F.Grand Hôpital EST Francilien	Department of Virology, Henri Mondor University Hospital, Assistance Publique Hôpitaux de Paris, Université Paris-Est Créteil, INSERM U955	Alexandre Soulier; Christophe Rodriguez; Elisabeth Trawinski; Guillaume Gricourt; Jean-Michel Pawlotsky; Melissa N'Debi; Slim Fourati; Vanessa Demontant
EPI_ISL_1422562 to 1422563, EPI_ISL_1422565 to 1422566, EPI_ISL_1422568 to 1422569, EPI_ISL_1422571 to 1422572, EPI_ISL_1422574 to 1422575, EPI_ISL_1422577 to 1422578, EPI_ISL_1422580 to 1422581, EPI_ISL_1422583 to 1422584, EPI_ISL_1422586 to 1422587, EPI_ISL_1422589 to 1422590, EPI_ISL_1443802 to 1443818, EPI_ISL_1527444 to 1527463			
see above	GA Department of Public Health	GA Department of Public Health	Aliyah Fields; Cynthia Dixey; Jonathan Edwards; Stacy Reeves; Taylor Smith; Tonia Parrott
EPI_ISL_420786 to 420788	GA Department of Public Health	Pathogen Discovery, Respiratory Viruses Branch, Division of Viral Diseases, Centers for Disease Control and Prevention	Alison S. Laufer Halpin; Anne Uehara; Christopher A. Elkins; Clinton R. Paden; Haibin Wang; Jasmine Padilla; Jing Zhang; Justin Lee; Krista Queen; Mary S. Keckler; Rachel Marine; Suxiang Tong; Yan Li; Ying Tao
EPI_ISL_1446809 to 1446824, EPI_ISL_1446864 to 1446867, EPI_ISL_1446872, EPI_ISL_1446877, EPI_ISL_1446880, EPI_ISL_1446882, EPI_ISL_1446887, EPI_ISL_1446900, EPI_ISL_1446904 to 1446905, EPI_ISL_1446910 to 1446911, EPI_ISL_1447003 to 1447011, EPI_ISL_1447055 to 1447058, EPI_ISL_1528521 to 1528530			
see above	GA Department of Public Health Laboratory	Centers for Disease Control and Prevention Division of Viral Diseases, Pathogen Discovery	Alison Laufer Halpin; Ben L. Rambo-Martin; Clinton R. Paden; Dakota Howard; Darlene Wagner; Dave Wentworth; Dhwani Batra; Jasmine Padilla; Justin Lee; Katie Dillon; Krista Queen; Kristen Knipe; Kristine Lacek; Mark Burroughs; Matthew Schmerer; Mili Sheth; Peter Cook; Sam Shepard; Sarah Nobles; Shoshona Le; Suxiang Tong; Vivien Dugan; Yvette Unoarumhi
EPI_ISL_1408891, EPI_ISL_1408895	GA Department of Public Health Laboratory	Genomics and Discovery, Respiratory Viruses Branch, Division of Viral Diseases, Centers for Disease Control and Prevention	Anna Kelleher; Anna Uehara; Brian Lynch; Clinton R. Paden; Haibin Wang; Han Jia Justin Ng; Jing Zhang; Krista Queen; Peter Cook; Suxiang Tong; Yan Li; Ying Tao
EPI_ISL_419556 to 419557, EPI_ISL_424858 to 424859, EPI_ISL_424861, EPI_ISL_424864, EPI_ISL_426417, EPI_ISL_527658 to 527665, EPI_ISL_566085 to 566107, EPI_ISL_576189 to 576195			
see above	GA Department of Public Health Laboratory	Pathogen Discovery, Respiratory Viruses Branch, Division of Viral Diseases, Centers for Disease Control and Prevention	Alison S. Laufer Halpin; Anna Montmayeur; Anna Uehara; Brian Lynch; Christopher A. Elkins; Clinton R. Paden; Haibin Wang; Jasmine Padilla; Jing Zhang; Justin Lee; Krista Queen; Mary S. Keckler; Peter Cook; Rachel Marine; Suxiang Tong; Yan Li; Ying Tao
EPI_ISL_475058 to 475059, EPI_ISL_495065 to 495066	GAIMS & G K General Hospital	Gujarat Biotechnology Research Centre	; A M Kadri; Afzal Ansari; Apurvasinh Puvar; Babulal Babhoria; Chaitanya Joshi; Dinesh Kumar; Harsh Bakshi; Hitesh Assudani; Janvi Raval; Komal Patel; Labdhi Pandya; Madhvi Joshi; Maharshi Pandya; Monika Gandhi; Neha Rajpara; Nidhi Patel; Nikha Trivedi; Nitin Savaliya; Pinal Trivedi; Pragya Sharma; Pritesh Sabara; R D Dixit; Raghawendra Kumar; Snehal Bagatharia; Zarna Patel; Zuber Saiyed
EPI_ISL_586569 to 586570	GCRI,Ahmedabad	Gujarat Biotechnology Research Centre	A M Kadri; Afzal Ansari; Apurvasinh Puvar; Chaitanya Joshi; Dinesh Kumar; Harsh Bakshi; Harsha Panchal; Janvi Raval; Komal Patel; Labdhi Pandya; Madhvi Joshi; Maharshi Pandya; Monika Gandhi; Nidhi Patel; Nikha Trivedi; Nitin Savaliya; Pinal Trivedi; R D Dixit; Raghawendra Kumar; Shashank Pandya; Zarna Patel; Zuber Saiyed
EPI_ISL_1461246, EPI_ISL_1526567, EPI_ISL_1526585	GH BOURG CENTRE PRELEVEMENT	CNR Virus des Infections Respiratoires - France SUD	Antonin Bal; Bruno Lina; Gregory Destras; Gwendolyne Burfin; Hadrien Regue; Laurence Josset; Martine Valette; Quentin Semanas
EPI_ISL_418416, EPI_ISL_508878, EPI_ISL_509003 to 509004	GH Les Portes du Sud	CNR Virus des Infections Respiratoires - France SUD	Alexandre Gaymard; Antonin Bal; Bruno Lina; Carine Moustaud; Florence Morfin-Sherpa; Gregory Destras; Gwendolyne Burfin; Laurence Josset; Martine Valette; Maude Bouscambert-Duchamp; Raphaelle Lamy; Solenne Brun
EPI_ISL_428351 to 428352, EPI_ISL_428363	GH Nord Essonne Service de Biologie clinique	National Reference Center for Viruses of Respiratory Infections, Institut Pasteur, Paris	Angela Brisebarre; Etienne Simon-Lorière; Flora Donati; Marion Barbet; Maud Vanpeene; Mélanie Albert; Méline Bizard; Sylvie Behillil; Sylvie van der Werf; Vincent Enouf
EPI_ISL_1517294 to 1517296	GHOL	Laboratory of genomics and metagenomics	Claire Bertelli; Damien Jacot; Gilbert Greub; SÃ©bastien Aeby; Trestan Pillonel
EPI_ISL_1397546 to 1397549, EPI_ISL_1397962 to 1397965, EPI_ISL_1398154 to 1398155, EPI_ISL_1398270, EPI_ISL_1461293, EPI_ISL_1465617, EPI_ISL_1465853 to 1465856, EPI_ISL_1482333 to 1482335			
see above	GLENS FALLS HOSPITAL LABORATORY	Wadsworth Center, New York State Department of Health	Alexis Russel; Alexis Russell; Catharine Prussing; Daryl M. Lamson; Erasmus Schneider; Erica Lasek-Nesselquist; John Kelly; Jonathan Plitnick; Kirsten St. George; Matthew Shudt; Melissa A Leisner; Navjot Singh
EPI_ISL_586562 to 586566	GMERS GMERS Medical College and Hospital, Junagadh	Gujarat Biotechnology Research Centre	A M Kadri; Afzal Ansari; Apurvasinh Puvar; Bhavesh Bagda; Chaitanya Joshi; Dinesh Kumar; Harsh Bakshi; Janvi Raval; Komal Patel; Labdhi Pandya; Madhvi Joshi; Maharshi Pandya; Monika Gandhi; Nidhi Patel; Nikha Trivedi; Nitin Savaliya; Pinal Trivedi; R D Dixit; Raghawendra Kumar; Ravi Dedaniya; Zarna Patel; Zuber Saiyed
EPI_ISL_469037 to 469042, EPI_ISL_475047 to 475055	GMERS Medical College & Hospital	Gujarat Biotechnology Research Centre	A M Kadri; Afzal Ansari; Ankit Hinsu; Apurvasinh Puvar; Armi Chaudhari; Bhavya Jindal; Chaitanya Joshi; Dinesh Kumar; Fenil Patel; Harsh Bakshi; Janvi Raval; Komal Patel; Labdhi Pandya; Madhvi Joshi; Maharshi Pandya; Meenakshi Shah; Monika Gandhi; Neelam Nathani; Neena Doshi; Neha Rajpara; Nidhi Patel; Nitin Savaliya; Pinal Trivedi; Pooja P Doshi; Pragya Sharma; Pritesh Sabara; Priyanka P Vatsa; R D Dixit; Raghawendra Kumar; Snehal Bagatharia; Tejas Shah; Varsha Godbole; Zarna Patel; Zuber Saiyed
EPI_ISL_476855 to 476862, EPI_ISL_495026 to 495062, EPI_ISL_500946, EPI_ISL_514610,	GMERS Medical College & Hospital, Gotri, Vadodara	Gujarat Biotechnology Research Centre	; A M Kadri; Afzal Ansari; Apurvasinh Puvar; Chaitanya Joshi; Dinesh Kumar; Harsh Bakshi; Janvi Raval; Kamlesh J Upadhyay; Komal Patel; Labdhi Pandya; Madhvi Joshi; Maharshi Pandya; Meenakshi Shah; Monika Gandhi; Neena Doshi; Nidhi Patel; Nikha Trivedi; Nitin Savaliya; Pinal Trivedi; Pranay

EPI_ISL_524730 to 524731			Shah; R D Dixit; Raghawendra Kumar; Sanjay Kapadia; Varsha Godbole; Zarna Patel; Zuber Saiyed
EPI_ISL_495063 to 495064	GMERS Medical College & Hospital, Himmatnagar	Gujarat Biotechnology Research Centre	; A M Kadri; Afzal Ansari; Apurvasinh Puvar; Chaitanya Joshi; Dinesh Kumar; Harsh Bakshi; Himanshu Khatri; Janvi Raval; Komal Patel; Labdhi Pandya; Madhvi Joshi; Maharshi Pandya; Mayur Gandhi; Monika Gandhi; Nidhi Patel; Nikha Trivedi; Nitin Savaliya; Pinal Trivedi; R D Dixit; Raghawendra Kumar; Zarna Patel; Zuber Saiyed
EPI_ISL_483823 to 483828	GMERS Medical College Himmatnagar	Gujarat Biotechnology Research Centre	A M Kadri; Afzal Ansari; Apurvasinh Puvar; Chaitanya Joshi; Dinesh Kumar; Harsh Bakshi; Himanshu Khatri; Janvi Raval; Komal Patel; Labdhi Pandya; Madhvi Joshi; Maharshi Pandya; Mayur Gandhi; Monika Gandhi; Nidhi Patel; Nikha Trivedi; Nitin Savaliya; Pinal Trivedi; R D Dixit; Raghawendra Kumar; Zarna Patel; Zuber Saiyed
EPI_ISL_524733 to 524734, EPI_ISL_524752 to 524766	GMERS Medical College and Hospital, Dharpur, Patan	Gujarat Biotechnology Research Centre	A M Kadri; A N Parmar; Afzal Ansari; Apurvasinh Puvar; Chaitanya Joshi; Dinesh Kumar; Harsh Bakshi; Janvi Raval; Komal Patel; Labdhi Pandya; Madhvi Joshi; Maharshi Pandya; Monika Gandhi; Nidhi Patel; Nikha Trivedi; Nitin Savaliya; Pinal Trivedi; R D Dixit; Raghawendra Kumar; Zarna Patel; Zuber Saiyed
EPI_ISL_447047 to 447053, EPI_ISL_447547 to 447555, EPI_ISL_467029 to 467038, EPI_ISL_476863 to 476866, EPI_ISL_483820, EPI_ISL_514611 to 514612, EPI_ISL_524737 to 524745, EPI_ISL_525419 to 525420			
see above	GMERS Medical College and Hospital, Gandhinagar	Gujarat Biotechnology Research Centre	A M Kadri; Afzal Ansari; Akanksha Verma; Amit Kanani; Anjali Rajwar; Ankit Hinsu; Apurvasinh Puvar; Armi Chaudhari; Bharti Rajani; Bhavesh Modi; Bhavya Jindal; Binita Aring; Chaitanya Joshi; Dinesh Kumar; Dipa Kinariwala; Dipeshwari Shewale; Disha Patel; Fenil Patel; Gaurishankar Shrimali; Geeta Vaghela; Harsh Bakshi; Janvi Raval; Kairavi Joshi; Kamlesh J Upadhyay; Komal Patel; Labdhi Pandya; Madhvi Joshi; Maharshi Pandya; Monika Gandhi; Neelam Nathani; Neeta Khandelwal; Neha Rajpara; Nidhi Patel; Nidhi Sood; Nikha Trivedi; Nitin Savaliya; Pinal Trivedi; Pooja P Doshi; Pragya Sharma; Pranay Shah; Pritesh Sabara; Priti Pandita; Priyanka P Vatsa; R D Dixit; Raghawendra Kumar; Ramesh Pandit; Sanjay Kapadia; Seema Bhatt; Sharmistha Majumdar; Snehal Bagatharia; Sonia Barve; Tejas Shah; Zarna Patel; Zuber Saiyed
EPI_ISL_1544133 to 1544134	GMERS, Medical College, Gandhinagar	Gujarat Biotechnology Research Centre	Chaitanya Joshi; Dinesh Kumar; Gaurishankar Shrimali; Janvi Raval; Madhvi Joshi; Nitesh Shah; Nitin Savaliya; Ramesh Pandit; Sonal Sharma; Twinkle Soni; Umang Mishra; Zarna Patel; Zuber Saiyed
EPI_ISL_1465848, EPI_ISL_1465858	GOOD SAMARITAN HOSPITAL LABORATORY	Wadsworth Center, New York State Department of Health	Alexis Russell; Catharine Prussing; Daryl M. Lamson; Erasmus Schneider; Erica Lasek-Nesselquist; John Kelly; Jonathan Plitnick; Kirsten St. George; Matthew Shudt; Melissa A Leisner; Navjot Singh
EPI_ISL_583426, EPI_ISL_583429, EPI_ISL_583434 to 583436, EPI_ISL_583441 to 583442, EPI_ISL_583452 to 583454, EPI_ISL_583458 to 583459, EPI_ISL_583463 to 583464, EPI_ISL_583467, EPI_ISL_583471, EPI_ISL_583473, EPI_ISL_583477			
see above	Garcia-Sastre Laboratory, Department of Microbiology, Icahn School of Medicine at Mount Sinai	van Bakel Laboratory, Genetics and Genomics Sciences, Icahn School of Medicine at Mount Sinai	Adolfo García-Sastre; Adriana van de Guchte; Ajay Obla; Ana S. Gonzalez-Reiche; Genovefa Papanicolaou; Gunjan Shah; Harm van Bakel; Jayeeta Dutta; Judith Aberg; Kent Sepkowitz; Miguel-Angel Perales; Ngolela Esther Babady; Sadaf Aslam; Teresa Aydillo; Tobias Hohl; Zenab Khan; and Mini Kamboj
EPI_ISL_534249 to 534251	Gavle Sjukhus	The Public Health Agency of Sweden	Anna Risberg; Anna-Malin Linde; Karin Tegmark-Wisell; Maria Lind Karlberg; Mattias Haukland; Mia Brytting; Olov Svartstrom; Oskar Karlsson Lindsjo; Petra Edquist; Reza Advani; Sandra Broddesson
EPI_ISL_475110, EPI_ISL_475115, EPI_ISL_582801, EPI_ISL_582809, EPI_ISL_582834, EPI_ISL_615101 to 615103	Gavle klinisk mikrobiologi	The Public Health Agency of Sweden	Anna Risberg; Anna-Malin Linde; Karin Tegmark-Wisell; Maria Lind Karlberg; Mattias Haukland; Mia Brytting; Olov Svartstrom; Oskar Karlsson Lindsjo; Petra Edquist; Reza Advani; Sandra Broddesson; Shamam Muradrasoli
EPI_ISL_420855, EPI_ISL_420876 to 420877, EPI_ISL_430064 to 430066	Geelong Centre for Emerging Infectious Diseases	Geelong Centre for Emerging Infectious Diseases	Alexandersen S.; Bhatta T.R.; Chamings A.; Chamings, A.; Raj Bhatta T.
EPI_ISL_1393874 to 1393875, EPI_ISL_1447260, EPI_ISL_1447281, EPI_ISL_1538682	GenXOne SA	1. National Institute of Public Health - National Institute of Hygiene; 2. Eurofins Genomics Europe Sequencing GmbH	ECDC COVID-19 WGS support team; Eurofins Genomics Europe Sequencing Team; Gierczyki Rafa; Sadkowska-Todys Magorzata; Wokowicz Tomasz; Zacharczuk Katarzyna
EPI_ISL_1538761 to 1538762, EPI_ISL_1538861	GeneMe	1. National Institute of Public Health - National Institute of Hygiene; 2. Eurofins Genomics Europe Sequencing GmbH	ECDC COVID-19 WGS support team; Eurofins Genomics Europe Sequencing Team; Gierczyki Rafa; Sadkowska-Todys Magorzata; Wokowicz Tomasz; Zacharczuk Katarzyna
EPI_ISL_467300	General Hospital "Abdulah Nakas"	Alea Genetic Center	Damir Marjanovic; Dino Pecar; Enis Kandic; Lana Salihefendic; Nihad Fejzic; Rijad Konjhodzic; Sead Jazic; Teufik Goletic
EPI_ISL_516413	General Hospital - Kumanovo	Research Center for Genetic Engineering and Biotechnology "Georgi D. Efremov" , Macedonian Academy of Sciences and Arts	RCGEB - MASA
EPI_ISL_514354	General Hospital - Prilep	Research Center for Genetic Engineering and Biotechnology "Georgi D. Efremov" , Macedonian Academy of Sciences and Arts	RCGEB - MASA
EPI_ISL_586531 to 586535	General Hospital,Chhotaudepur	Gujarat Biotechnology Research Centre	A M Kadri; Afzal Ansari; Apurvasinh Puvar; Arti Thakur; Chaitanya Joshi; Dharak Pandya; Dinesh Kumar; Harsh Bakshi; Janvi Raval; Komal Patel; Labdhi Pandya; Madhvi Joshi; Maharshi Pandya; Monika Gandhi; Nidhi Patel; Nikha Trivedi; Nitin Savaliya; Pinal Trivedi; R D Dixit; Raghawendra Kumar; Yogesh Parmar; Zarna Patel; Zuber Saiyed
EPI_ISL_586546 to 586548	General Hospital,Godhra	Gujarat Biotechnology Research Centre	A M Kadri; Afzal Ansari; Apurvasinh Puvar; Chaitanya Joshi; Dinesh Kumar; Harsh Bakshi; Janvi Raval; Komal Patel; Labdhi Pandya; Madhvi Joshi; Maharshi Pandya; Monika Gandhi; Neela Katara; Nidhi Patel; Nikha Trivedi; Nitin Savaliya; Pinal Gandhi; Pinal Trivedi; R D Dixit; Raghawendra Kumar; Sangeeta Kumar; Zarna Patel; Zuber Saiyed
EPI_ISL_430846	General Intensive Care Unit, Raymond Poincaré Hospital (AP-HP), Lab Inflammation & Infection, U1173 University Paris Saclay-UVSQ/INSERM, Garches, France.	Institut Pasteur, Laboratory for Urgent Response to biological Threats	Annane Djillali; Caro Valérie; Manuguerra Jean-Claude; Vanhomwegen Jessica
EPI_ISL_614282 to 614293	General practitioner	National Reference Center for Viruses of Respiratory Infections, Institut Pasteur, Paris	Angela Brisebarre; Camille Capel; Etienne Simon-Lorière; Marion Barbet; Maud Vanpeene; Méline Bizard; Sylvie Behillil; Sylvie van der Werf; Vincent Enouf
EPI_ISL_1470415 to 1470559, EPI_ISL_1534544 to 1534609, EPI_ISL_1540996 to 1541072	Genetica Molecular and Subdepartamento de Virologia ISP Chile	Instituto de Salud Publica de Chile	Andres Castillo; Barbara Parra; Gisselle Barra; Jaime Lagos; Javier Tognarelli; Jorge Fernandez; Karen Orostica; Loredana Arata; Patricia Bustos; Rodrigo Fasce
EPI_ISL_1530875 to 1530876	Genome Analysis Center, Kamma Memorial Hospital	Genome Analysis Center, Kamma Memorial Hospital	Hanako Yazawa; Hiroshi Kamma; Jun Ishii; Kaori Watanabe; Michiaki Masuda; Satoko Soma; Takuya Yazawa
EPI_ISL_475570 to 475571, EPI_ISL_475573, EPI_ISL_605783	Genome Center	Genome Center	A. S. M. Rubayet- Ul- Alam; Ashok Kumar; Chakrabortty Atonu; Habiba Ibnat; Hassan M. Al-Emran; M. Rafiul Islam; M. Shaminur Rahman; Md Nur Kabidul Azam; Md. Ali Ahasan Setu; Md. Aminul Islam; Md. Anwar Hossain; Md. Hasan al Pramanik; Md. Iqbal Kabir Jahid; Md. Iqbal Kabir Jahid Md. Anwar Hossain; Md. Muzahidul Islam; Md. Nazmul Hasan; Md. Shazid Hasan; Md. Taijul Islam; Md. Tanvir Islam; Md. Zannat Ali; Mithun Talukder Md. Tawyabur; Najmuj Sakib; Ovinu Kibria Islam; Pravas Chandra Roy; Proshanto Kumar Das; Provakar Mondol; Ruhul Amin; S. M. Tanjil Shah; Selina Akter; Shireen Nigar; Shohanur Rahaman; Shovon Lal Sarkar; Sourav Dutta; Tanay Chakrovarty
EPI_ISL_427391, EPI_ISL_428346, EPI_ISL_428368, EPI_ISL_480228 to 480292	Genomic Laboratory (GLAB) (Conjoint lab of Health Directorate of Istanbul and Istanbul Technical University)	Genomic Laboratory (GLAB), Istanbul Technical University	Arzu Irvem; Betsi Köse; Bugra Agaoglu; Elifnaz Çelik; Gizem Alkurt; Gizem Dinler Doganay; Ilker Karacan; Jale Yildiz; Levent Doganay; Mehtap Aydn; Ozlem Akgun Dogan; Tugba Kizilboga Akgun; Yasemin Kendir Demirkol
EPI_ISL_1492576	Genomic Medicine Laboratory, IRCCS Santa Lucia Foundation	INMI Lazzaro Spallanzani IRCCS	A Di Caro; B Bartolini; CEM Gruber; E Giardina; E Giombini; F Messina; F Santini; G Bonfiglio; M Rueca; MR Capobianchi; O Butera
EPI_ISL_512774	Genomic Research Lab, BCSIR	Bangladesh Council of Scientific and Industrial Research	A. K. M. Shamsuzzaman; Abu Sayeed Mohammad Mahmud; Asish Kumar Ghosh; Barna Goswami; Eshrar Osman; Iffat Jahan; Mahmuda Yeasmin; Md. Ahasan Habib; Md. Maruf Ahmed Molla; Md. Murshed Hasan Sarkar; Md. Saddam Hossain; Md. Salim Khan; Mohammad Samir Uzzaman; Salek Ahmed Sajib; Shahina Akter; Sheikh Md. Selim Al Din; Tanjina Akhter Banu; Tasnim Nafisa; Utpal Chandra Ray
EPI_ISL_483688	Genomic Research Lab, BCSIR	Genomic Research Lab, BCSIR	A. K. M. Shamsuzzaman; Abu Sayeed Mohammad Mahmud; Asish Kumar Ghosh; Barna Goswami; Eshrar Osman; Iffat Jahan; Mahmuda Yeasmin; Md. Ahasan Habib; Md. Maruf Ahmed Molla; Md. Murshed Hasan Sarkar; Md. Saddam Hossain; Md. Salim Khan; Mohammad Samir Uzzaman; Salek Ahmed Sajib; Shahina Akter; Sheikh Md. Selim Al Din; Tanjina Akhter Banu; Tasnim Nafisa; Utpal Chandra Ray
EPI_ISL_1511643	Genomic and molecular Biology Group, A.C.Camargo Cancer	Laboratory of Bioinformatics and Computational Biology,	Alexandre Defelicibus; Dirce Carraro; Giovana Torrezan; Israel Tojal

	Center	A.C.Camargo Cancer Center	
EPI_ISL_436715 to 436717	Genomics and Computational Biology Lab, Scientific Research Institute of Physical-Chemical Medicine, FMBA of Russia	Genomics and Computational Biology Lab, Scientific Research Institute of Physical-Chemical Medicine, FMBA of Russia	A. Manolov; A. Pavlenko; D. Fedorov; K. Klimina; O. Guskova; V. Govorun and E. Ilina; V. Veselovsky
EPI_ISL_1534341, EPI_ISL_1534423, EPI_ISL_1534431, EPI_ISL_1534433, EPI_ISL_1534444	George Hospital wc GRH	NHLS/UCT	Arash Iranzadeh; Bruna Galvao; Carolyn Williamson; Deelan Doolabh; Diana Hardie; Emmanuel SJ; Innocent Mudau; Kruger Marais; Lynn Tyers; Marvin Hsiao; Stephen Korsman; Tegally H; de Oliveira T
EPI_ISL_450801, EPI_ISL_452120, EPI_ISL_452125, EPI_ISL_509662	Georgia Department of Health	Pathogen Discovery, Respiratory Viruses Branch, Division of Viral Diseases, Centers for Disease Control and Prevention	Alison S. Laufer Halpin; Anna Montmayeur; Anna Uehara; Bettina Bankamp; Christopher A. Elkins; Clinton R. Paden; Haibin Wang; Jing Zhang; Krista Queen; Mary S. Keckler; Rachel Marine; Suxiang Tong; Yan Li; Ying Tao; Zachary Weiner
EPI_ISL_594456	Georgia Public Health Laboratory	Pathogen Discovery, Respiratory Viruses Branch, Division of Viral Diseases, Centers for Disease Control and Prevention	Anna Uehara; Clinton Paden; Haibin Wang; Jing Zhang; Julu Bhatnagar; Krista Queen; Suxiang Tong; Yan Li; Ying Tao
EPI_ISL_539524	Gerencia de Asistencia Sanitaria de Soria	Instituto de Salud Carlos III	A. Monzón; C. Aldea; F. Casas; I; I. Jiménez; Iglesias-Caballero; M. Camarero; M. Cuesta; M. González-Esguevillas; M. Molinero Calamita; M. Zaballos; P. Jiménez; S. Juliá; S. Pozo; S. Varona
EPI_ISL_539557	Gerencia del área de salud de Badajoz, Llerena y Zafra	Instituto de Salud Carlos III	A. Monzón; C. Pazos; F. Casas; I; I. Jiménez; Iglesias-Caballero; M. Camarero; M. Cuesta; M. González-Esguevillas; M. Molinero Calamita; M. Zaballos; P. Jiménez; S. Juliá; S. Pozo; S. Varona
EPI_ISL_480083 to 480089	Gifu Prefectural Institute of Public Health and Environmental Sciences	Pathogen Genomics Center, National Institute of Infectious Diseases	Hajime Kamiya; Kentaro Itokawa; Makoto Kuroda; Masanori Hashino; Motoi Suzuki; Rina Tanaka; Tsuyoshi Sekizuka; Yoshihiko Kameyama
EPI_ISL_1529099, EPI_ISL_1529370, EPI_ISL_1529535, EPI_ISL_1529952	Global Health Research Complex (GHRC)	Texas A&M Institute for Genomic Sciences and Society (TIGSS)	Allison Ficht; Benjamin W. Neuman; David Threadgill; Drew Hillhouse; Kurt A. Zuelke; Melissa M. Kahl-Mcdonagh; Sankar P. Chaki; Sierra Guidry; Wesley Brashear
EPI_ISL_1528826	Global Health Research Complex (GHRC)	TxGen	Allison Ficht; Benjamin W. Neuman; Charlie D. Johnson; Joshua Hill; Kurt A. Zuelke; Marcel Brun; Melissa M. Kahl-Mcdonagh; Richard Metz; Sankar P. Chaki; Sierra Guidry
EPI_ISL_452218 to 452223	Goethe University Hospital Frankfurt	Institute for Medical Virology, Goethe University Hospital Frankfurt	Annemarie Berger; Björn Rotter; Denisa Bojkova; Jindrich Cinatl; Klaus Hoffmeier; Sandra Westhaus; Sandra Ciesek; Sebastian Hoehl; Tuna Toptan; and Marek Widera
EPI_ISL_1508978 to 1508983, EPI_ISL_1508987, EPI_ISL_1532118 to 1532123	Good Samaritan Hospital	Santa Clara County Public Health Laboratory	Santa Clara County Public Health Department
EPI_ISL_1482470	Good Samaritan Hospital Laboratory	Wadsworth Center, New York State Department of Health	Alexis Russell; Catharine Prussing; Daryl M. Lamson; Erasmus Schneider; Erica Lasek-Nesselquist; John Kelly; Jonathan Plitnick; Kirsten St. George; Matthew Shudt; Melissa A Leisner; Navjot Singh
EPI_ISL_415152	Gorgas Memorial Institute for Health Studies	Gorgas Memorial Institute for Health Studies	Alexander A. Martinez.; Ambar Moreno; Claudia Gonzalez; Danilo Franco; Elimelec Valdespino; Juan M. Pascale; Leyda Abrego; Oris Chavarria; Sandra Lopez-Verges; Yamilka Diaz
EPI_ISL_496603 to 496915, EPI_ISL_1502842 to 1502860, EPI_ISL_1502862 to 1502863, EPI_ISL_1502866, EPI_ISL_1502869 to 1503122, EPI_ISL_1503130 to 1503142	Gorgas Memorial Laboratory of Health Studies	Gorgas Memorial Laboratory of Health Studies	Alexander A Martinez; Castillo Jorge; Claudia Gonzalez Sandra Lopez-Verges; Danilo Franco; Franco Danilo; Gonzalez Claudia; Jessica Gondola; Leyda Abrego; Lopez-Verges Sandra; Marlenne Castillo; Martinez Alexander; Moreno Ambar; Moreno Brechla; Oris Chavarria; Ortiz Alma
EPI_ISL_524735 to 524736, EPI_ISL_586542 to 586545	Government General Hospital, Jam Khambhaliya, Devbhoomi Dwarka	Gujarat Biotechnology Research Centre	A M Kadri; Afzal Ansari; Apurvasinh Puvar; Chaitanya Joshi; Dinesh Kumar; Harish Matani; Harsh Bakshi; Janvi Raval; Komal Patel; Labdhi Pandya; Madhvi Joshi; Maharshi Pandya; Monika Gandhi; Nidhi Patel; Nikha Trivedi; Nitin Savaliya; Pinal Trivedi; R D Dixit; Raghawendra Kumar; Zarna Patel; Zuber Saiyed
EPI_ISL_586551 to 586554, EPI_ISL_590691 to 590692	Government Hospital,Veraval	Gujarat Biotechnology Research Centre	A M Kadri; Afzal Ansari; Apurvasinh Puvar; Chaitanya Joshi; Dinesh Kumar; Harsh Bakshi; Janvi Raval; Jignesh Parmar; Jitendra Bamrotia; Komal Patel; Labdhi Pandya; Madhvi Joshi; Maharshi Pandya; Monika Gandhi; Nidhi Patel; Nikha Trivedi; Nitin Savaliya; Pinal Trivedi; R D Dixit; Raghawendra Kumar; Zarna Patel; Zuber Saiyed
EPI_ISL_508207 to 508286	Government Medical College	National Institute of Biomedical Genomics	Arindam Maitra; Dhaval Khatri; Jyoti Iravane; Maitrik Dave; Saumitra Das
EPI_ISL_511930 to 511941	Government Medical College	National Institute of Biomedical Genomics - DBT's PAN-INDIA 1000 SARS--CoV-2 RNA Genome Sequencing Consortium	Arindam Maitra; Dhaval Khatri; Jyoti Iravane; Maitrik Dave; Saumitra Das
EPI_ISL_1544179 to 1544195	Government Medical College (GMC), Surat	Gujarat Biotechnology Research Centre	Chaitanya Joshi; Dinesh Kumar; Janvi Raval; Madhvi Joshi; Neeta Khandelwal; Nitesh Shah; Nitin Savaliya; Ramesh Pandit; Sonal Sharma; Twinkle Soni; Umang Mishra; Zarna Patel; Zuber Saiyed
EPI_ISL_495020 to 495025	Government Medical College, Bhavnagar	Gujarat Biotechnology Research Centre	A M Kadri; Afzal Ansari; Apurvasinh Puvar; Chaitanya Joshi; Dinesh Kumar; Harsh Bakshi; Janvi Raval; Kairavi Desai; Komal Patel; Labdhi Pandya; Madhvi Joshi; Maharshi Pandya; Monika Gandhi; Nidhi Patel; Nikha Trivedi; Nitin Savaliya; Pinal Trivedi; R D Dixit; Raghawendra Kumar; Saklin Malek; Shirish Patel; Zarna Patel; Zuber Saiyed
EPI_ISL_451154 to 451163, EPI_ISL_455017 to 455027, EPI_ISL_461478 to 461480, EPI_ISL_467039 to 467040, EPI_ISL_469029 to 469036, EPI_ISL_483821 to 483822	Government Medical College, Vadodara	Gujarat Biotechnology Research Centre	; A M Kadri; Afzal Ansari; Akanksha Verma; Amit Kanani; Ankit Hinsu; Apurvasinh Puvar; Armi Chaudhari; Bhavesh Modi; Bhavya Jindal; Camellia Chakraborty; Chaitanya Joshi; Dinesh Kumar; Fenil Patel; Gaurishankar Shrimali; Harsh Bakshi; Janvi Raval; Komal Patel; Labdhi Pandya; Madhvi Joshi; Maharshi Pandya; Manish Pattani; Meenakshi Shah; Monika Gandhi; Neelam Nathani; Neena Doshi; Nidhi Patel; Nikha Trivedi; Nitin Savaliya; Pinal Trivedi; Pooja P Doshi; Pragya Sharma; Pritesh Sabara; Priti Pandita; Priyanka P Vatsa; R D Dixit; R N Daveshwar; Raghawendra Kumar; Ramesh Pandit; Sharmistha Majumdar; Siddhant Kumar; Snehal Bagatharia; Tanuja Javadekar; Tejas Shah; Umang Mishra; Varsha Godbole; Zarna Patel; Zuber Saiyed
EPI_ISL_450781 to 450784	Government Medical College-Bhavnagar	Gujarat Biotechnology Research Centre	A M Kadri; Afzal Ansari; Akanksha Verma; Amit Kanani; Ankit Hinsu; Apurvasinh Puvar; Bhavesh Modi; Chaitanya Joshi; Dinesh Kumar; Gaurishankar Shrimali; Janvi Raval; Kairavi Desai; Komal Patel; Labdhi Pandya; Madhvi Joshi; Maharshi Pandya; Monika Gandhi; Neha Rajpara; Nidhi Patel; Nitin Savaliya; Pinal Trivedi; Pritesh Sabara; Priti Pandita; R D Dixit; Raghawendra Kumar; Ramesh Pandit; Saklain Malek; Shirish Patel; Snehal Bagatharia; Tejas Shah; Zarna Patel; Zuber Saiyed
EPI_ISL_1503921 to 1503954, EPI_ISL_1503971	Grady Memorial Hospital, Clinical Microbiology Laboratory	Piantadosi Lab, Emory Department of Pathology	Anne Piantadosi; Yun F (Wayne) Wang
EPI_ISL_1423341	Grafton City Hospital	WVU and Marshall University Combined Genomics Core Facilities	"James Denvir; Peter Perrotta; Peter Stoilov; Ryan Percifield"; Wesley Kimble
EPI_ISL_1470375, EPI_ISL_1470395, EPI_ISL_1491342, EPI_ISL_1491367	Gravity Diagnostics	Kentucky State Public Health Lab	Joshua Tobias; Karim George; Matthew Johnson; Rachel Zinner; Rhonda Lucas; Stephanie Lunn; Vaneet Arora; William Grooms
EPI_ISL_1534247 to 1534259, EPI_ISL_1534268, EPI_ISL_1534283, EPI_ISL_1534291, EPI_ISL_1534302, EPI_ISL_1534304, EPI_ISL_1534306, EPI_ISL_1534308 to 1534310, EPI_ISL_1534313, EPI_ISL_1534315 to 1534320, EPI_ISL_1534322 to 1534326, EPI_ISL_1534345 to 1534352, EPI_ISL_1534362, EPI_ISL_1534364, EPI_ISL_1534366, EPI_ISL_1534369, EPI_ISL_1534376 to 1534377, EPI_ISL_1534382, EPI_ISL_1534389 to 1534394, EPI_ISL_1534406 to 1534408, EPI_ISL_1534411 to 1534416, EPI_ISL_1534450 to 1534453, EPI_ISL_1534455, EPI_ISL_1534457, EPI_ISL_1534459			
see above	Groote Schuur Hospital wc GSH	NHLS/UCT	Arash Iranzadeh; Bruna Galvao; Carolyn Williamson; Deelan Doolabh; Diana Hardie; Emmanuel SJ; Innocent Mudau; Kruger Marais; Lynn Tyers; Marvin Hsiao; Stephen Korsman; Tegally H; de Oliveira T
EPI_ISL_1396813 to 1396822, EPI_ISL_1396824 to 1396828, EPI_ISL_1396834 to 1396836	Groupe LCD	Department of Virology, Henri Mondor University Hospital, Assistance Publique Hôpitaux de Paris, Université Paris-Est Créteil, INSERM U955	Alexandre Soulier; Christophe Rodriguez; Elisabeth Trawinski; Guillaume Gricourt; Jean-Michel Pawlotsky; Melissa N'Debi; Slim Fourati; Vanessa Demontant
EPI_ISL_1447673 to 1447676, EPI_ISL_1509270 to 1509271	Grubaugh Lab - Yale School of Public Health	Grubaugh Lab - Yale School of Public Health	Anderson Brito; Annie Watkins; Chaney Kalinich; Chantal Vogels; Isabell Ott; Jessica Rothman; Joseph Fauver; Mallery Breban; Mary Petrone; Nathan Grubaugh; Tara Alpert
EPI_ISL_447734 to 447754	Grupo de Investigaciones Microbiológicas-UR (GIMUR), Departamento de Biología, Facultad de Ciencias Naturales, Universidad del Rosario, Bogotá, Colombia	Grupo de Investigaciones Microbiológicas-UR (GIMUR), Departamento de Biología, Facultad de Ciencias Naturales, Universidad del Rosario, Bogotá, Colombia Instituto Nacional	Adriana Castillo; Alberto Paniz-Mondolfi; Ana S. Gonzalez-Reiche; Angelica Rico; Aníbal A. Teherán; Carolina Florez; Carolina Hernandez; David Martínez; Emilia Mia Sordillo; Esther C. Barros; Harm van Bakel; Jesús E. Jaimes; Juan David Ramírez; Laura Vega; Lisseth Pardo; Marina Muñoz; Martha L. Ospina; Matthew M. Hernandez; Nathalia Ballesteros; Sergio Castañeda; Sergio Gomez; Viviana Simon

		de Salud, Bogotá, Colombia Icahn School of Medicine at Mount Sinai, New York, USA	
EPI_ISL_406531	Guangdong Provincial Center for Diseases Contorl and Prevention; Guangdong Provinical Public Health	Guangdong Provincial Center for Disease Control and Prevention	Baisheng Li; Changwen Ke; Feng Ruan; Guanhao He; Haojie Zhong; Huihong Deng; Jianfeng He; Jianpeng Xiao; Jianxiang Geng; Jianxiong Hu; Jie Wu; Jing Lu; Lifeng Lin; Lijun Liang; Lirong Zou; Min Kang; Qi Zhu; Shujiang Mei; Songjian Xiao; Tao Liu; Tie Song; Weilin Zeng; Wenjun Ma; Xing Li; Xiujuan Tang; Xue Zhuang; Xuguang Chen; Ying Wang; Yingchao Song; Yingtao Zhang; Yuhuang Liao; Zhe Liu
EPI_ISL_406533	Guangdong Provincial Center for Diseases Contorl and Prevention; Guangdong Provinical Public Health	Guangdong Provincial Center for Diseases Control and Prevention	Baisheng Li; Changwen Ke; Feng Ruan; Guanhao He; Haojie Zhong; Huihong Deng; Jianfeng He; Jianpeng Xiao; Jianxiang Geng; Jianxiong Hu; Jie Wu; Jing Lu; Lifeng Lin; Lijun Liang; Lirong Zou; Min Kang; Qi Zhu; Shujiang Mei; Songjian Xiao; Tao Liu; Tie Song; Weilin Zeng; Wenjun Ma; Xing Li; Xiujuan Tang; Xue Zhuang; Xuguang Chen; Ying Wang; Yingchao Song; Yingtao Zhang; Yuhuang Liao; Zhe Liu
EPI_ISL_403932 to 403937	Guangdong Provincial Center for Diseases Control and Prevention; Guangdong Provinical Public Health	Department of Microbiology, Guangdong Provincial Center for Diseases Control and Prevention	Baisheng Li; Changwen Ke; Feng Ruan; Guanhao He; Haojie Zhong; Huihong Deng; Jianfeng He; Jianpeng Xiao; Jianxiang Geng; Jianxiong Hu; Jie Wu; Jing Lu; Lifeng Lin; Lijun Liang; Lirong Zou; Min Kang; Qi Zhu; Shujiang Mei; Songjian Xiao; Tao Liu; Tie Song; Weilin Zeng; Wenjun Ma; Xing Li; Xiujuan Tang; Xue Zhuang; Xuguang Chen; Ying Wang; Yingchao Song; Yingtao Zhang; Yuhuang Liao; Zhe Liu
EPI_ISL_406534 to 406536	Guangdong Provincial Center for Diseases Control and Prevention; Guangdong Provinical Public Health	Guangdong Provincial Center for Diseases Control and Prevention	Baisheng Li; Changwen Ke; Feng Ruan; Guanhao He; Haojie Zhong; Huihong Deng; Jianfeng He; Jianpeng Xiao; Jianxiang Geng; Jianxiong Hu; Jie Wu; Jing Lu; Lifeng Lin; Lijun Liang; Lirong Zou; Min Kang; Qi Zhu; Shujiang Mei; Songjian Xiao; Tao Liu; Tie Song; Weilin Zeng; Wenjun Ma; Xing Li; Xiujuan Tang; Xue Zhuang; Xuguang Chen; Ying Wang; Yingchao Song; Yingtao Zhang; Yuhuang Liao; Zhe Liu
EPI_ISL_406538	Guangdong Provincial Center for Diseases Control and Prevention;Guangdong Provincial Institute of Public Health	Guangdong Provincial Center for Diseases Control and Prevention	Baisheng Li; Changwen Ke; Feng Ruan; Guanhao He; Haojie Zhong; Huihong Deng; Jianfeng He; Jianpeng Xiao; Jianxiang Geng; Jianxiong Hu; Jie Wu; Jing Lu; Lifeng Lin; Lijun Liang; Lirong Zou; Min Kang; Qi Zhu; Shujiang Mei; Songjian Xiao; Tao Liu; Tie Song; Weilin Zeng; Wenjun Ma; Xing Li; Xiujuan Tang; Xue Zhuang; Xuguang Chen; Ying Wang; Yingchao Song; Yingtao Zhang; Yuhuang Liao; Zhe Liu
EPI_ISL_428440 to 428478	Guangdong Provincial Center for Diseases Control and Prevention;Guangdong Provincial Institute of Public Health	School of Public Health, The University of Hong Kong	Bosheng Li; Hanri Zeng; Haogao Gu; Hui-Ling Yen; Jie Wu; Leo L.M. Poon; Lijun Liang; Tie Song; Yao Hu; Yingchao Song; Zhencui Li
EPI_ISL_413850 to 413902	Guangdong Provincial Institution of Public Health, Guangdong Provinical Center for Disease Control and Prevention	Guangdong Provincial Institution of Public Health	Andrew Rambaut; Bo Peng; Changwen Ke; Chuming Liang; Huanying Zheng; Huifang Lin; Jing Lu; Jingju Peng; Jiufeng Sun; Josh Quick; Juan Su; Kang Min; Kuibiao Li; Lilian Zeng; Liu Zhe; Louis du Plessis; Minfeng Liang; Moritz Kraemer; Nick Loman; Nuno Faria; Oliver Pybus; Pingping Zhou; Qianlin Xiong; Ru bai; Ruilin Sun; Runyu Yuan; Sarah François; Shisong Fang; Song Tie; Tao Liu; Verity Hill; Wei Li; Wenjun Ma; Wenzhe Su; Xi Tang; Jing Lu
EPI_ISL_444969	Guangzhou Eighth People's Hospital (Jiahe Sector)	Institute of Human Virology, Zhongshan School of Medicine, Sun Yat-sen University	Bingfeng Liu; Fang Li; Fei Yu; Feng Huang; Fengyu Hu; Hui Zhang; Huimin Fan; Jun Liu; Junsong Zhang; Kai Deng; Mang Shi; Ruosu Ying; Ting Pan; Xu Zhang; Yiwen Zhang
EPI_ISL_509695 to 509703, EPI_ISL_509710	Guatemala Ministry of Public Health	Pathogen Discovery, Respiratory Viruses Branch, Division of Viral Diseases, Centers for Disease Control and Prevention	Anna Uehara; Clinton Paden; Haibin Wang; Jing Zhang; Krista Queen; Suxiang Tong; Yan Li; Ying Tao
EPI_ISL_1534397, EPI_ISL_1534410	Guguletu CHC wc GDH	NHLS/UCT	Arash Iranzadeh; Bruna Galvao; Carolyn Williamson; Deelan Doolabh; Diana Hardie; Emmanuel SJ; Innocent Mudau; Kruger Marais; Lynn Tyers; Marvin Hsiao; Stephen Korsman; Tegally H; de Oliveira T
EPI_ISL_435055 to 435056, EPI_ISL_444482 to 444486, EPI_ISL_447534 to 447546, EPI_ISL_458103 to 458113	Gujarat Biotechnology Research Centre	Gujarat Biotechnology Research Centre	; A M Kadri; Afzal Ansari; Akanksha Verma; Amit Kanani; Anjali Rajwal; Anjali Rajwar; Ankit Hinsu; Apurvasinh Puvar; Armi Chaudhari; Bhavesh Modi; Bhavya Jindal; Binita Aring; Camellia Chakraborty; Chaitanya Joshi; Dinesh Kumar; Dipa Kinariwala; Dipeshwari Shewale; Disha Patel; Gaurishankar Shrimali; Geeta Vaghela; Janvi Raval; Kairavi Joshi; Kamlesh J Upadhyay; Komal Patel; Labdhi Pandya; Madhvi Joshi; Maharshi Pandya; Monika Gandhi; Neelam Nathani; Neeta Khandelwal; Neha Rajpara; Nidhi Patel; Nidhi Sood; Nitin Savaliya; Pinal Trivedi; Pooja P Doshi; Pragya Sharma; Pranay Shah; Pritesh Sabara; Priti Pandita; Priyanka P Vatsa; R D Dixit; Raghawendra Kumar; Ramesh Pandit; Sharmistha Majumdar; Siddhant Kumar; Snehal Bagatharia; Sonia Barve; Tejas Shah; Umang Mishra; Zarna Patel; Zuber Saiyed
EPI_ISL_455573 to 455574, EPI_ISL_455580 to 455582, EPI_ISL_471157, EPI_ISL_489904, EPI_ISL_489956 to 489958, EPI_ISL_547608 to 547610, EPI_ISL_547666 to 547676, EPI_ISL_547683 to 547684, EPI_ISL_547705, EPI_ISL_1465767 to 1465793, EPI_ISL_1483051 to 1483052			
see above	Gundersen Clinical Microbiology Laboratory	Kabara Cancer Research Institute	Craig S. Richmond; Paraic A. Kenny
EPI_ISL_418185 to 418186	Gundersen Molecular Diagnostic Laboratory	Kabara Cancer Research Institute	Craig S. Richmond & Paraic A. Kenny
EPI_ISL_418184, EPI_ISL_418187 to 418189, EPI_ISL_419651 to 419652, EPI_ISL_422453, EPI_ISL_422459, EPI_ISL_422461 to 422465, EPI_ISL_426159 to 426161, EPI_ISL_429843 to 429848, EPI_ISL_435394 to 435402, EPI_ISL_436891 to 436900, EPI_ISL_455568 to 455572, EPI_ISL_455575 to 455579, EPI_ISL_471144 to 471156, EPI_ISL_489896 to 489903, EPI_ISL_489905 to 489955, EPI_ISL_547602 to 547607, EPI_ISL_547611 to 547665, EPI_ISL_547677 to 547682, EPI_ISL_547685 to 547704, EPI_ISL_547706 to 547869			
see above	Gundersen Molecular Diagnostics Laboratory	Kabara Cancer Research Institute	Craig S. Richmond; Craig S. Richmond & Paraic A. Kenny; Paraic A. Kenny
EPI_ISL_1442956 to 1442992, EPI_ISL_1448408	Gunma Prefectural Institute of Public Health and Environmental Sciences	Gunma Prefectural Institute of Public Health and Environmental Sciences	Daisuke Shinoda; Hiroyuki Tsukagoshi; Kentaro Itokawa; Makoto Kuroda; Mariko Saito; Masanori Hashino; Rina Tanaka; Tsuyoshi Sekizuka
EPI_ISL_479850 to 479854, EPI_ISL_479896 to 479901, EPI_ISL_480015 to 480020, EPI_ISL_480169 to 480177, EPI_ISL_1425568, EPI_ISL_1425701 to 1425702, EPI_ISL_1426142 to 1426157, EPI_ISL_1427569, EPI_ISL_1427672, EPI_ISL_1427689 to 1427692, EPI_ISL_1427719 to 1427728, EPI_ISL_1428601, EPI_ISL_1430572 to 1430575, EPI_ISL_1431736, EPI_ISL_1431802 to 1431804			
see above	Gunma Prefectural Institute of Public Health and Environmental Sciences	Pathogen Genomics Center, National Institute of Infectious Diseases	; Chikako Shimokawa; Daisuke Kobayashi; Eunsil Park; Hajime Kamiya; Hiroyuki Tsukagoshi; Hussein H. Aly; Kentaro Itokawa; Kento Fukano; Makoto Kuroda; Masanori Hashino; Motoi Suzuki; Rina Tanaka; Takanobu Kato; Tsuguto Fujimoto; Tsuyoshi Sekizuka; Yasutaka Hoshino; Yoshihiro Kaku
EPI_ISL_421454, EPI_ISL_421458, EPI_ISL_421481, EPI_ISL_421487, EPI_ISL_421491	H Beatriz Angelo	Instituto Nacional de Saude (INSA)	Guiomar et al
EPI_ISL_418003, EPI_ISL_418007 to 418008, EPI_ISL_418019 to 418022	H Braga	Instituto Nacional de Saude (INSA)	Guiomar et al
EPI_ISL_421457	H Dr Nelio Mendonca - Funchal	Instituto Nacional de Saude (INSA)	Guiomar et al
EPI_ISL_418026, EPI_ISL_421449, EPI_ISL_421459 to 421461	H Dr. Nelio Mendonca - Funchal	Instituto Nacional de Saude (INSA)	Guiomar et al
EPI_ISL_418023, EPI_ISL_421466 to 421467	H Evora	Instituto Nacional de Saude (INSA)	Guiomar et al
EPI_ISL_418018	H Garcia de Orta	Instituto Nacional de Saude (INSA)	Guiomar et al
EPI_ISL_421446 to 421448	H Guimaraes	Instituto Nacional de Saude (INSA)	Guiomar et al
EPI_ISL_418025, EPI_ISL_421462, EPI_ISL_421468 to 421471, EPI_ISL_421488, EPI_ISL_421490, EPI_ISL_421492, EPI_ISL_421495			
see above	H Santarem	Instituto Nacional de Saude (INSA)	Guiomar et al
EPI_ISL_455587	H.R.H. Maha Chakri Sirindhorn Medical Center	National Institute of Health. Department of medical Sciences, Ministry of Public Health, Thailand	Chittaganpitch; Malinee; Okada; Parnmen; Phuygun; Pilailuk; Siripaporn; Sittiporn; Sunthareeya; Thanadachakul; Thanutsapa; Waicharoen; Warawan; Wongboot
EPI_ISL_1404131 to 1404142	HCRC	HCRC	Janneke Cox; Jolien Van Cleemput; Laurens Lambrechts; Linos Vandekerckhove; Nick Vereecke; Piet Maes; Sebastiaan Theuns
EPI_ISL_1500949 to 1501127, EPI_ISL_1501137 to 1501169, EPI_ISL_1501174 to 1501210, EPI_ISL_1501218 to 1501257	HEGP - Laboratoire de Virologie	HEGP - Laboratoire de Virologie	David Veyer
EPI_ISL_596250 to 596263, EPI_ISL_596268 to 596317, EPI_ISL_596357 to 596377	HELIX LCC	WHO National Influenza Centre Russian Federation	Andrey Komissarov; Anna Ivanova; Artem Fadeev; Daria Danilenko; Dmitry Bazhenov; Kseniya Komissarova
EPI_ISL_602329 to 602411, EPI_ISL_602417 to 602461, EPI_ISL_1400490, EPI_ISL_1400493, EPI_ISL_1400495, EPI_ISL_1400498, EPI_ISL_1400501 to 1400503, EPI_ISL_1400505, EPI_ISL_1400509 to 1400513, EPI_ISL_1400515 to 1400516, EPI_ISL_1400518 to 1400521, EPI_ISL_1400526 to 1400528, EPI_ISL_1400530 to 1400531, EPI_ISL_1400541 to 1400545, EPI_ISL_1400553 to 1400557, EPI_ISL_1400562 to 1400568, EPI_ISL_1400571, EPI_ISL_1491588, EPI_ISL_1491593, EPI_ISL_1491597 to 1491602, EPI_ISL_1491604, EPI_ISL_1491608 to 1491613, EPI_ISL_1491615 to 1491616, EPI_ISL_1491618 to 1491627, EPI_ISL_1491671 to 1491732			
see above	HELIX LLC	WHO National Influenza Centre Russian Federation	Alexey Masharsky; Andrey Komissarov; Anna Ivanova; Artem Fadeev; Daria Danilenko; Dmitry Bazhenov; Dmitry Lioznov; Elena Nabieva; Georgii

			Bazykin; Ksenia Safina; Kseniya Komissarova; Maria Baturova; Maria Pisareva; Maria Timofeeva; Mikhail Bakaev; Tamila Musaeva; Veronika Eder
EPI_ISL_426420 to 426421	HI Dept. of Health, State Laboratories Division	Pathogen Discovery, Respiratory Viruses Branch, Division of Viral Diseases, Centers for Disease Control and Prevention	Alison S. Laufer Halpin; Anna Uehara; Christopher A. Elkins; Clinton R. Paden; Haibin Wang; Jing Zhang; Krista Queen; Mary S. Keckler; Rachel Marine; Suxiang Tong; Yan Li; Ying Tao
EPI_ISL_1406453, EPI_ISL_1406478, EPI_ISL_1406587, EPI_ISL_1406630, EPI_ISL_1406679, EPI_ISL_1483707, EPI_ISL_1483738, EPI_ISL_1490244, EPI_ISL_1547400, EPI_ISL_1547421, EPI_ISL_1547427, EPI_ISL_1547469			
see above	HOPITAL DE MONTLUCON	CHU Clermont-Ferrand, service de virologie	Bisseux Maxime; Combes Patricia; Henquell Cécile; Mirand Audrey
EPI_ISL_1406439, EPI_ISL_1406442, EPI_ISL_1406452, EPI_ISL_1406458, EPI_ISL_1406484, EPI_ISL_1406596, EPI_ISL_1406611, EPI_ISL_1406623, EPI_ISL_1406651, EPI_ISL_1406660 to 1406662, EPI_ISL_1406665, EPI_ISL_1406669, EPI_ISL_1406671, EPI_ISL_1406673, EPI_ISL_1406676 to 1406678, EPI_ISL_1406682, EPI_ISL_1406688, EPI_ISL_1483710, EPI_ISL_1483724, EPI_ISL_1483740 to 1483741, EPI_ISL_1483760, EPI_ISL_1483769 to 1483770, EPI_ISL_1490233 to 1490234, EPI_ISL_1490245, EPI_ISL_1492561, EPI_ISL_1547447, EPI_ISL_1547459, EPI_ISL_1547462, EPI_ISL_1547465 to 1547467, EPI_ISL_1547470			
see above	HOPITAL DE VICHY	CHU Clermont-Ferrand, service de virologie	Bisseux Maxime; Combes Patricia; Henquell Cécile; Mirand Audrey
EPI_ISL_1406461, EPI_ISL_1406473, EPI_ISL_1406497, EPI_ISL_1406500, EPI_ISL_1406530 to 1406531, EPI_ISL_1406535, EPI_ISL_1406543, EPI_ISL_1406545, EPI_ISL_1406571, EPI_ISL_1406574, EPI_ISL_1406593, EPI_ISL_1406672, EPI_ISL_1483759, EPI_ISL_1483761, EPI_ISL_1483777 to 1483779, EPI_ISL_1490229, EPI_ISL_1547412, EPI_ISL_1547453			
see above	HOPITAL DU MONT DORE	CHU Clermont-Ferrand, service de virologie	Bisseux Maxime; Combes Patricia; Henquell Cécile; Mirand Audrey
EPI_ISL_1526395	HOPITAL INSTRUCTION DES ARMEES LAVERAN	CNR Virus des Infections Respiratoires - France SUD	Antonin Bal; Bruno Lina; Gregory Destras; Gwendolyne Burfin; Hadrien Regue; Laurence Josset; Martine Valette; Quentin Semanas
EPI_ISL_445359	HOSP. SANTIAGO ORIENTE DR. LUIS TISNE B.	Instituto de Salud Publica de Chile	Alejandra Acevedo; Andrés E Castillo; Bárbara Parra; Carolina Tambley; Gabriel Leal; Jaime Lagos; Jorge Fernandez; Loredana Arata; Patricia Bustos; Paz Tapia; Rodrigo Fasce; Winston Andrade
EPI_ISL_445323	HOSP.ENFERMEDADES INFECCIOSAS	Instituto de Salud Publica de Chile	Alejandra Acevedo; Andrés E Castillo; Bárbara Parra; Carolina Tambley; Gabriel Leal; Jaime Lagos; Jorge Fernandez; Loredana Arata; Patricia Bustos; Paz Tapia; Rodrigo Fasce; Winston Andrade
EPI_ISL_1461113, EPI_ISL_1461115	HOSPILAB AGEN	CNR Virus des Infections Respiratoires - France SUD	Antonin Bal; Bruno Lina; Gregory Destras; Gwendolyne Burfin; Hadrien Regue; Laurence Josset; Martine Valette; Quentin Semanas
EPI_ISL_1469813	HOSPITAL BOM JESUS	Epiclin	Ana Paula Muterle; Carolina Comerlato; Eliana Márcia Da Ros Wendland; Fernando Hayashi Sant'Anna; Janira Prichula; Juliana Comerlato
EPI_ISL_1510005	HOSPITAL CLINIC	Instituto de Salud Carlos III	A. Monzón; F. Casas; I. Jiménez; I.VILA ESTAPE; JORDI; M. Sandonís; P. Zaballos; S. Cuesta; S. Iglesias-Caballero; S. Pozo; S. Varona; V. Camarero; Vázquez-Morón
EPI_ISL_445281	HOSPITAL CLINICO DEL SUR	Instituto de Salud Publica de Chile	Alejandra Acevedo; Andrés E Castillo; Bárbara Parra; Carolina Tambley; Gabriel Leal; Jaime Lagos; Jorge Fernandez; Loredana Arata; Patricia Bustos; Paz Tapia; Rodrigo Fasce; Winston Andrade
EPI_ISL_445275 to 445276	HOSPITAL CLINICO FUSAT	Instituto de Salud Publica de Chile	Alejandra Acevedo; Andrés E Castillo; Bárbara Parra; Carolina Tambley; Gabriel Leal; Jaime Lagos; Jorge Fernandez; Loredana Arata; Patricia Bustos; Paz Tapia; Rodrigo Fasce; Winston Andrade
EPI_ISL_1509963 to 1509964, EPI_ISL_1509974 to 1509977, EPI_ISL_1509995, EPI_ISL_1510002 to 1510003, EPI_ISL_1510010 to 1510012, EPI_ISL_1511226 to 1511233	HOSPITAL COMARCAL DE MELILLA	Instituto de Salud Carlos III	A. Monzón; F. Casas; I. Jiménez; I. ROMAN SOTO; I.ROMAN SOTO; Iglesias-Caballero; M. Camarero; M. Sandonís; P. Zaballos; S. Camarero; S. Cuesta; S. Iglesias-Caballero; S. Pozo; S. Varona; SERGIO; Sandonís; V. Camarero; V. Vázquez-Morón; Vázquez-Morón
EPI_ISL_445378	HOSPITAL DE BULNES	Instituto de Salud Publica de Chile	Alejandra Acevedo; Andrés E Castillo; Bárbara Parra; Carolina Tambley; Gabriel Leal; Jaime Lagos; Jorge Fernandez; Loredana Arata; Patricia Bustos; Paz Tapia; Rodrigo Fasce; Winston Andrade
EPI_ISL_445335	HOSPITAL DE CALBUCO	Instituto de Salud Publica de Chile	Alejandra Acevedo; Andrés E Castillo; Bárbara Parra; Carolina Tambley; Gabriel Leal; Jaime Lagos; Jorge Fernandez; Loredana Arata; Patricia Bustos; Paz Tapia; Rodrigo Fasce; Winston Andrade
EPI_ISL_1469705, EPI_ISL_1469839	HOSPITAL DE CAMPO BOM DR LAURO REUS	Epiclin	Ana Paula Muterle; Carolina Comerlato; Eliana Márcia Da Ros Wendland; Fernando Hayashi Sant'Anna; Janira Prichula; Juliana Comerlato
EPI_ISL_445251, EPI_ISL_445305, EPI_ISL_445354, EPI_ISL_445369 to 445370	HOSPITAL DE CARABINEROS	Instituto de Salud Publica de Chile	Alejandra Acevedo; Andrés E Castillo; Bárbara Parra; Carolina Tambley; Gabriel Leal; Jaime Lagos; Jorge Fernandez; Loredana Arata; Patricia Bustos; Paz Tapia; Rodrigo Fasce; Winston Andrade
EPI_ISL_419238	HOSPITAL DE CRUCES.	Instituto de Salud Carlos III	A. Monzón; F. Casas; I. Aranzamendi, M.; I. Jiménez; Iglesias-Caballero; M. Camarero; M. Cuesta; M. González-Esguevillas; M. Molinero Calamita; M. Zaballos; P. Jiménez; S. Juliá; S. Pozo; S. Varona
EPI_ISL_1509996	HOSPITAL DE LA VEGA LORENZO GUIRAO	Instituto de Salud Carlos III	A. Monzón; F. Casas; I. Jiménez; I.ALVAREZ MELGARES DE AGUILAR; Iglesias-Caballero; JOAQUIN; M. Sandonís; P. Zaballos; S. Camarero; S. Cuesta; S. Pozo; S. Varona; V. Vázquez-Morón
EPI_ISL_445300	HOSPITAL DE RANCAGUA	Instituto de Salud Publica de Chile	Alejandra Acevedo; Andrés E Castillo; Bárbara Parra; Carolina Tambley; Gabriel Leal; Jaime Lagos; Jorge Fernandez; Loredana Arata; Patricia Bustos; Paz Tapia; Rodrigo Fasce; Winston Andrade
EPI_ISL_445298	HOSPITAL DE SAN FERNANDO	Instituto de Salud Publica de Chile	Alejandra Acevedo; Andrés E Castillo; Bárbara Parra; Carolina Tambley; Gabriel Leal; Jaime Lagos; Jorge Fernandez; Loredana Arata; Patricia Bustos; Paz Tapia; Rodrigo Fasce; Winston Andrade
EPI_ISL_1553814, EPI_ISL_1553820	HOSPITAL DE SANTA CATERINA	Banc de Sang i Teixits	Carlos Hobeich; Francisco Vidal; Irene Corrales; Lorena Ramírez; Maria Glòria Soria; Natália Comes; Nina Borràs; Noemí Gonzalez; Silvia Sauleda
EPI_ISL_445352, EPI_ISL_445360, EPI_ISL_445368	HOSPITAL DEL PROFESOR	Instituto de Salud Publica de Chile	Alejandra Acevedo; Andrés E Castillo; Bárbara Parra; Carolina Tambley; Gabriel Leal; Jaime Lagos; Jorge Fernandez; Loredana Arata; Patricia Bustos; Paz Tapia; Rodrigo Fasce; Winston Andrade
EPI_ISL_1494944	HOSPITAL DEPARTAMENTAL SAN VICENTE DE PAUL	Instituto Nacional de Salud- Dirección de Investigación en Salud Pública	Carlos Franco-Muñoz; Carmen Osorio; Diana Malo; Diego A. Álvarez-Díaz; Diego Andrés Prada; Gerardo Santamaría; Hector Alejandro Ruiz-Moreno; Jhonnatan Reales-González; Juan Camilo Martinez; Julian Naizaque; Katherine Laiton-Donato; Lisseth Pardo; Magdalena Wiesner; Marcela Mercado-Reyes; Maria T. Herrera-Sepúlveda; Marta Lopez Blanco; Martha Lucia Ospina Martinez; Paola Rojas; Sergio Gomez; Sheryll Corchuelo; Ángela Alarcon Cruz
EPI_ISL_445270, EPI_ISL_445338 to 445348, EPI_ISL_447119	HOSPITAL DR.HERNAN HENRIQUEZ ARAVENA	Instituto de Salud Publica de Chile	Alejandra Acevedo; Andrés E Castillo; Bárbara Parra; Carolina Tambley; Gabriel Leal; Jaime Lagos; Jorge Fernandez; Loredana Arata; Patricia Bustos; Paz Tapia; Rodrigo Fasce; Winston Andrade
EPI_ISL_445310, EPI_ISL_445325, EPI_ISL_445329, EPI_ISL_445365 to 445366, EPI_ISL_445371	HOSPITAL DR.SOTERO DEL RIO	Instituto de Salud Publica de Chile	Alejandra Acevedo; Andrés E Castillo; Bárbara Parra; Carolina Tambley; Gabriel Leal; Jaime Lagos; Jorge Fernandez; Loredana Arata; Patricia Bustos; Paz Tapia; Rodrigo Fasce; Winston Andrade
EPI_ISL_1445200	HOSPITAL E MATERNIDADE NOSSA SENHORA DA AJUDA	Instituto Butantan / Mendelics	Antonio Jorge Martins; Bibiana Santos; Claudia Renata dos Santos Barros; David Schlesinger; Debora Botequio Moretti; Dimas Tadeu Covas; Elaine Cristina Marqueze; Elaine Vieira dos Santos; Erika Freitas; Evandra Strazza Rodrigues; Flavia Aburjaile; José Salvatore Leister Patané; João Paulo Kitajima; Luiz Carlos Junior de Alcantara; Maria Carolina Elias; Marta Giovanetti; Rafael dos Santos Bezerra; Raul Machado Neto; Ricardo Haddad; Rodrigo Tocantins Calado.; Sandra Coccuzzo Sampaio; Simone Kashima; Svetoslav Nanev Slavov; Vagner Fonseca; Vincent Louis Viala
EPI_ISL_445308, EPI_ISL_445313 to 445314, EPI_ISL_445364	HOSPITAL EL CARMEN DR.LUIS VALENTIN F.	Instituto de Salud Publica de Chile	Alejandra Acevedo; Andrés E Castillo; Bárbara Parra; Carolina Tambley; Gabriel Leal; Jaime Lagos; Jorge Fernandez; Loredana Arata; Patricia Bustos; Paz Tapia; Rodrigo Fasce; Winston Andrade
EPI_ISL_445319, EPI_ISL_445321	HOSPITAL FELIX BULNES	Instituto de Salud Publica de Chile	Alejandra Acevedo; Andrés E Castillo; Bárbara Parra; Carolina Tambley; Gabriel Leal; Jaime Lagos; Jorge Fernandez; Loredana Arata; Patricia Bustos; Paz Tapia; Rodrigo Fasce; Winston Andrade
EPI_ISL_445372	HOSPITAL FF.AA. "CIRUJANO C. GUZMAN	Instituto de Salud Publica de Chile	Alejandra Acevedo; Andrés E Castillo; Bárbara Parra; Carolina Tambley; Gabriel Leal; Jaime Lagos; Jorge Fernandez; Loredana Arata; Patricia Bustos; Paz Tapia; Rodrigo Fasce; Winston Andrade
EPI_ISL_1511216 to 1511217	HOSPITAL GENERAL DE LANZAROTE	Instituto de Salud Carlos III	A. Monzón; F. Casas; I. Jiménez; I.COPADO CARRETERO; M. Sandonís; P. Zaballos; RODOLFO; S. Cuesta; S. Iglesias-Caballero; S. Pozo; S. Varona; V. Camarero; Vázquez-Morón
EPI_ISL_418247	HOSPITAL GENERAL DE SEGOVIA	Instituto de Salud Carlos III	A. Monzón; F. Casas; I. Hernando-Real S.; I. Jiménez; Iglesias-Caballero; M. Camarero; M. Cuesta; M. González-Esguevillas; M. Molinero Calamita; M. Zaballos; P. Jiménez; S. Juliá; S. Pozo; S. Varona

EPI_ISL_1510004, EPI_ISL_1510687	HOSPITAL GENERAL UNIVERSITARIO REINA SOFIA	Instituto de Salud Carlos III	A. Monzón; F. Casas; I. Jiménez; I.NUÑEZ TRIGUEROS; Iglesias-Caballero; M. Sandonís; Mª LUZ; P. Zaballos; S. Camarero; S. Cuesta; S. Iglesias-Caballero; S. Pozo; S. Varona; V. Camarero; V. Vázquez-Morón; Vázquez-Morón
EPI_ISL_445286, EPI_ISL_445337	HOSPITAL HANGA ROA	Instituto de Salud Publica de Chile	Alejandra Acevedo; Andrés E Castillo; Bárbara Parra; Carolina Tambley; Gabriel Leal; Jaime Lagos; Jorge Fernandez; Loredana Arata; Patricia Bustos; Paz Tapia; Rodrigo Fasce; Winston Andrade
EPI_ISL_445331 to 445332	HOSPITAL HERMINDA MARTIN CHILLAN	Instituto de Salud Publica de Chile	Alejandra Acevedo; Andrés E Castillo; Bárbara Parra; Carolina Tambley; Gabriel Leal; Jaime Lagos; Jorge Fernandez; Loredana Arata; Patricia Bustos; Paz Tapia; Rodrigo Fasce; Winston Andrade
EPI_ISL_445336	HOSPITAL LAS HIGUERAS DE TALCAHUANO	Instituto de Salud Publica de Chile	Alejandra Acevedo; Andrés E Castillo; Bárbara Parra; Carolina Tambley; Gabriel Leal; Jaime Lagos; Jorge Fernandez; Loredana Arata; Patricia Bustos; Paz Tapia; Rodrigo Fasce; Winston Andrade
EPI_ISL_1509961 to 1509962	HOSPITAL LOS ARCOS	Instituto de Salud Carlos III	A. Monzón; F. Casas; I. Jiménez; I.CAMARA SIMON; M. Sandonís; MARGARITA; P. Zaballos; S. Cuesta; S. Iglesias-Caballero; S. Pozo; S. Varona; V. Camarero; Vázquez-Morón
EPI_ISL_1469746	HOSPITAL MONTENEGRO	Epiclin	Ana Paula Muterle; Carolina Comerlato; Eliana Márcia Da Ros Wendland; Fernando Hayashi Sant'Anna; Janira Prichula; Juliana Comerlato
EPI_ISL_1445173	HOSPITAL MUNICIPAL DE IBIUNA IBIUNA SP	Instituto Butantan / Mendelics	Antonio Jorge Martins; Bibiana Santos; Claudia Renata dos Santos Barros; David Schlesinger; Debora Botequio Moretti; Dimas Tadeu Covas; Elaine Cristina Marqueze; Elaine Vieira dos Santos; Erika Freitas; Evandra Strazza Rodrigues; Flavia Aburjaile; José Salvatore Leister Patané; João Paulo Kitajima; Luiz Carlos Junior de Alcantara; Maria Carolina Elias; Marta Giovanetti; Rafael dos Santos Bezerra; Raul Machado Neto; Ricardo Haddad; Rodrigo Tocantins Calado.; Sandra Coccuzzo Sampaio; Simone Kashima; Svetoslav Nanev Slavov; Vagner Fonseca; Vincent Louis Viala
EPI_ISL_1469624, EPI_ISL_1469631, EPI_ISL_1469699, EPI_ISL_1469759	HOSPITAL MUNICIPAL GETULIO VARGAS	Epiclin	Ana Paula Muterle; Carolina Comerlato; Eliana Márcia Da Ros Wendland; Fernando Hayashi Sant'Anna; Janira Prichula; Juliana Comerlato
EPI_ISL_445289, EPI_ISL_445301	HOSPITAL NAVAL PUERTO WILLIAMS	Instituto de Salud Publica de Chile	Alejandra Acevedo; Andrés E Castillo; Bárbara Parra; Carolina Tambley; Gabriel Leal; Jaime Lagos; Jorge Fernandez; Loredana Arata; Patricia Bustos; Paz Tapia; Rodrigo Fasce; Winston Andrade
EPI_ISL_445327, EPI_ISL_445353	HOSPITAL PADRE HURTADO	Instituto de Salud Publica de Chile	Alejandra Acevedo; Andrés E Castillo; Bárbara Parra; Carolina Tambley; Gabriel Leal; Jaime Lagos; Jorge Fernandez; Loredana Arata; Patricia Bustos; Paz Tapia; Rodrigo Fasce; Winston Andrade
EPI_ISL_445246	HOSPITAL PUERTO MONTT	Instituto de Salud Publica de Chile	Alejandra Acevedo; Andrés E Castillo; Bárbara Parra; Carolina Tambley; Gabriel Leal; Jaime Lagos; Jorge Fernandez; Loredana Arata; Patricia Bustos; Paz Tapia; Rodrigo Fasce; Winston Andrade
EPI_ISL_1511222	HOSPITAL RAFAEL MENDEZ	Instituto de Salud Carlos III	A. Monzón; EVA; F. Casas; I. CASCALES ALCOLEA; I. Jiménez; M. Camarero; P. Zaballos; S. Cuesta; S. Iglesias-Caballero; S. Pozo; S. Varona; Sandonís; V. Vázquez-Morón
EPI_ISL_445268 to 445269, EPI_ISL_445280, EPI_ISL_445284, EPI_ISL_445288, EPI_ISL_445293 to 445295	HOSPITAL REG.LAUTARO NAVARRO AVARIA	Instituto de Salud Publica de Chile	Alejandra Acevedo; Andrés E Castillo; Bárbara Parra; Carolina Tambley; Gabriel Leal; Jaime Lagos; Jorge Fernandez; Loredana Arata; Patricia Bustos; Paz Tapia; Rodrigo Fasce; Winston Andrade
EPI_ISL_445296	HOSPITAL REGIONAL DE COYHAIQUE	Instituto de Salud Publica de Chile	Alejandra Acevedo; Andrés E Castillo; Bárbara Parra; Carolina Tambley; Gabriel Leal; Jaime Lagos; Jorge Fernandez; Loredana Arata; Patricia Bustos; Paz Tapia; Rodrigo Fasce; Winston Andrade
EPI_ISL_1445083, EPI_ISL_1445181	HOSPITAL REGIONAL DE ITAPETININGA	Instituto Butantan / Mendelics	Antonio Jorge Martins; Bibiana Santos; Claudia Renata dos Santos Barros; David Schlesinger; Debora Botequio Moretti; Dimas Tadeu Covas; Elaine Cristina Marqueze; Elaine Vieira dos Santos; Erika Freitas; Evandra Strazza Rodrigues; Flavia Aburjaile; José Salvatore Leister Patané; João Paulo Kitajima; Luiz Carlos Junior de Alcantara; Maria Carolina Elias; Marta Giovanetti; Rafael dos Santos Bezerra; Raul Machado Neto; Ricardo Haddad; Rodrigo Tocantins Calado.; Sandra Coccuzzo Sampaio; Simone Kashima; Svetoslav Nanev Slavov; Vagner Fonseca; Vincent Louis Viala
EPI_ISL_593772 to 593776	HOSPITAL REGIONAL LAMBAYEQUE	GENOMA MAYOR	Franklin R. Aguilar-Gamboa; Heber Silva-Díaz; Juan J. Bonifacio-Briceño; Luis M. López-Serquén; Luis Salcedo-Mejía; Marco E. Mechán-Llontop and Juan P. Cárdenas.; Percy O. Tullume-Vergara; Ramsés Salas-Asencios
EPI_ISL_445307	HOSPITAL SAN JOSE DE MAIPO	Instituto de Salud Publica de Chile	Alejandra Acevedo; Andrés E Castillo; Bárbara Parra; Carolina Tambley; Gabriel Leal; Jaime Lagos; Jorge Fernandez; Loredana Arata; Patricia Bustos; Paz Tapia; Rodrigo Fasce; Winston Andrade
EPI_ISL_445304, EPI_ISL_445349 to 445351, EPI_ISL_445373 to 445377, EPI_ISL_449800	HOSPITAL SAN JUAN DE DIOS	Instituto de Salud Publica de Chile	Alejandra Acevedo; Andrés E Castillo; Bárbara Parra; Carolina Tambley; Gabriel Leal; Jaime Lagos; Jorge Fernandez; Loredana Arata; Patricia Bustos; Paz Tapia; Rodrigo Fasce; Winston Andrade
EPI_ISL_1510080 to 1510081	HOSPITAL SAN PEDRO DE ALCANTARA - C.D. 05	Instituto de Salud Carlos III	A. Monzón; F. Casas; GUADALUPE; I. Jiménez; I.RODRIGUEZ RODRIGUEZ; Iglesias-Caballero; M. Sandonís; P. Zaballos; S. Camarero; S. Cuesta; S. Pozo; S. Varona; V. Vázquez-Morón
EPI_ISL_417007	HOSPITAL SANTA MARIA NAI	Instituto de Salud Carlos III	A. Monzón; I. García Costa, J.; Iglesias-Caballero; M. Camarero S. Pozo F. Casas I. Jiménez; M. Cuesta; M. González-Esguevillas; M. Molinero Calamita; M. Zaballos; P. Jiménez; S. Juliá; S. Varona
EPI_ISL_1469575, EPI_ISL_1469578, EPI_ISL_1469643, EPI_ISL_1469648, EPI_ISL_1469676, EPI_ISL_1469685, EPI_ISL_1469738, EPI_ISL_1469744, EPI_ISL_1469834, EPI_ISL_1479128, EPI_ISL_1479133			
see above	HOSPITAL SAO FRANCISCO DE ASSIS	Epiclin	Ana Paula Muterle; Carolina Comerlato; Eliana Márcia Da Ros Wendland; Fernando Hayashi Sant'Anna; Janira Prichula; Juliana Comerlato
EPI_ISL_1469726	HOSPITAL SAPIRANGA	Epiclin	Ana Paula Muterle; Carolina Comerlato; Eliana Márcia Da Ros Wendland; Fernando Hayashi Sant'Anna; Janira Prichula; Juliana Comerlato
EPI_ISL_418253, EPI_ISL_419240, EPI_ISL_419709	HOSPITAL TXAGORRITXU	Instituto de Salud Carlos III	A. Monzón; C; F. Casas; I. Gomez-Gonzalez C.; I. Gómez; I. Gómez, C.; I. Jiménez; Iglesias-Caballero; M. Camarero; M. Camarero S. Pozo F. Casas I. Jiménez; M. Cuesta; M. González-Esguevillas; M. Molinero Calamita; M. Zaballos; P. Jiménez; S. Juliá; S. Pozo; S. Varona
EPI_ISL_1509960, EPI_ISL_1509972, EPI_ISL_1509990 to 1509994, EPI_ISL_1510007 to 1510008, EPI_ISL_1510041 to 1510079	HOSPITAL UNIV. MIGUEL SERVET	Instituto de Salud Carlos III	A. Monzón; ANA; ANA MARIA; F. Casas; I. Jiménez; I. MARTINEZ SAPIÑA; I. MILAGRO BEAMONTE; I.MILAGRO BEAMONTE; Iglesias-Caballero; M. Camarero; M. Sandonís; P. Zaballos; S. Camarero; S. Cuesta; S. Iglesias-Caballero; S. Pozo; S. Varona; Sandonís; V. Camarero; V. Vázquez-Morón; Vázquez-Morón
EPI_ISL_1509999 to 1510001, EPI_ISL_1510024 to 1510031, EPI_ISL_1510719 to 1510729	HOSPITAL UNIVERSITARIO DE CANARIAS(GESTION ECONOMICA)	Instituto de Salud Carlos III	A. Monzón; BEATRIZ; F. Casas; I. CASTRO HERNANDEZ; I. Jiménez; I.CASTRO HERNANDEZ; M. Camarero; M. Sandonís; P. Zaballos; S. Cuesta; S. Iglesias-Caballero; S. Pozo; S. Varona; Sandonís; V. Camarero; V. Vázquez-Morón; Vázquez-Morón
EPI_ISL_1511234 to 1511236	HOSPITAL UNIVERSITARIO DE CEUTA	Instituto de Salud Carlos III	A. Monzón; F. Casas; I. Jiménez; I.HIJANO VILLEGAS; Iglesias-Caballero; M. Sandonís; P. Zaballos; S. Camarero; S. Cuesta; S. Pozo; S. Varona; SALOME; V. Vázquez-Morón
EPI_ISL_1510009	HOSPITAL UNIVERSITARIO DE GETAFE	Instituto de Salud Carlos III	A. Monzón; F. Casas; I. Jiménez; I.GARCIA BERMEJO; ISABEL; M. Sandonís; P. Zaballos; S. Cuesta; S. Iglesias-Caballero; S. Pozo; S. Varona; V. Camarero; Vázquez-Morón
EPI_ISL_1510712	HOSPITAL UNIVERSITARIO DE GUADALAJARA	Instituto de Salud Carlos III	A. Monzón; ALEJANDRO; F. Casas; I. GONZALEZ PRAETORIUS; I. Jiménez; M. Camarero; P. Zaballos; S. Cuesta; S. Iglesias-Caballero; S. Pozo; S. Varona; Sandonís; V. Vázquez-Morón
EPI_ISL_1510688 to 1510707	HOSPITAL UNIVERSITARIO INSULAR DE GRAN CANARIA	Instituto de Salud Carlos III	A. Monzón; CRISTOBAL; F. Casas; I. Jiménez; I.DEL ROSARIO QUINTANA; M. Sandonís; P. Zaballos; S. Cuesta; S. Iglesias-Caballero; S. Pozo; S. Varona; V. Camarero; Vázquez-Morón
EPI_ISL_418251	HOSPITAL UNIVERSITARIO LA PAZ	Instituto de Salud Carlos III	A. Monzón; F. Casas; I. Jiménez; I. Romero P.; Iglesias-Caballero; M. Camarero; M. Cuesta; M. González-Esguevillas; M. Molinero Calamita; M. Zaballos; P. Jiménez; S. Juliá; S. Pozo; S. Varona
EPI_ISL_1509965 to 1509966, EPI_ISL_1509968 to 1509971, EPI_ISL_1509979 to 1509987, EPI_ISL_1509989, EPI_ISL_1510013 to 1510023, EPI_ISL_1510033 to 1510039, EPI_ISL_1510736 to 1510743, EPI_ISL_1510747 to 1510757, EPI_ISL_1511209 to 1511215			
see above	HOSPITAL UNIVERSITARIO VIRGEN DE LA ARRIXACA	Instituto de Salud Carlos III	A. Monzón; F. Casas; I. Jiménez; I. MORENO PARRADO; I.MORENO PARRADO; Iglesias-Caballero; LAURA; M. Camarero; M. Sandonís; P. Zaballos; S. Camarero; S. Cuesta; S. Iglesias-Caballero; S. Pozo; S. Varona; Sandonís; V. Camarero; V. Vázquez-Morón; Vázquez-Morón
EPI_ISL_418243 to 418244	HOSPITAL UNIVERSITARIO VIRGEN DE LAS NIEVES	Instituto de Salud Carlos III	A. Monzón; F. Casas; I. Jiménez; I. Sanbonmatsu S.; Iglesias-Caballero; M. Camarero; M. Cuesta; M. González-Esguevillas; M. Molinero Calamita; M. Zaballos; P. Jiménez; S. Juliá; S. Pozo; S. Varona
EPI_ISL_1510040	HOSPITAL VIRGEN DE ALTAGRACIA	Instituto de Salud Carlos III	A. Monzón; ANA; F. Casas; I. Jiménez; I. SANCHEZ MAROTO LOZANO; M. Camarero; P. Zaballos; S. Cuesta; S. Iglesias-Caballero; S. Pozo; S. Varona; Sandonís; V. Vázquez-Morón

EPI_ISL_430847	HS mikrobiologi virus	The Public Health Agency of Sweden	Anna Risberg; Anna-Malin Linde; Karin Tegmark-Wisell; Maria Lind Karlberg; Olov Svartstrom; Oskar Karlsson Lindsjo; Shaman Muradrasoli; Zhibing Yun
EPI_ISL_418009, EPI_ISL_421463, EPI_ISL_421489, EPI_ISL_421493 to 421494	HSE Ilha Terceira - Angra do Heroismo	Instituto Nacional de Saude (INSA)	Guiomar et al
EPI_ISL_412971	HUS Diagnostiikkakeskus, Hallinto	Department of Virology Faculty of Medicine, Medicum University of Helsinki	Hannimari Kallio-Kokko; Olli Vapalahti; Suvi Kuivanen; Teemu Smura
EPI_ISL_454436 to 454446, EPI_ISL_455902, EPI_ISL_469073 to 469074, EPI_ISL_475093 to 475094, EPI_ISL_475096 to 475098, EPI_ISL_475100, EPI_ISL_475114, EPI_ISL_475116 to 475117, EPI_ISL_475120 to 475121, EPI_ISL_475123, EPI_ISL_475125, EPI_ISL_475156 to 475159, EPI_ISL_475164, EPI_ISL_475548, EPI_ISL_475553, EPI_ISL_475556 to 475557, EPI_ISL_510831, EPI_ISL_510866, EPI_ISL_548247, EPI_ISL_548249, EPI_ISL_582803 to 582805, EPI_ISL_594148, EPI_ISL_615104 to 615107			
see above	Halmstad klinisk mikrobiologi	The Public Health Agency of Sweden	Anna Risberg; Anna-Malin Linde; Karin Tegmark-Wisell; Maria Lind Karlberg; Mattias Haukland; Mia Brytting; Olov Svartstrom; Oskar Karlsson Lindsjo; Petra Edquist; Reza Advani; Sandra Broddesson; Shamam Muradrasoli; Shaman Muradrasoli
EPI_ISL_407313	Hangzhou Center for Disease Control and Prevention	Hangzhou Center for Disease Control and Prevention	Haoqiu Wang; Hua Yu; Jun Li; Lingfeng Mao; Qingxin Kong; Shuchang Chen; Xin Qian; Xinfen Yu; Xuchu Wang; Zhou Sun
EPI_ISL_418510	Hangzhou Center for Disease Control and Prevention	Insepction Center of Hangzhou Center for Disease Control and Prevention	Li jun; Pan jingcao; Wang haoqiu; Yu hua; Yu xinfeng
EPI_ISL_418506	Hangzhou Center for Disease Control and Prevention	Inspection Center of Hanghzou Center for Disease Control and Prevention	Li jun; Pan jingcao; Wang haoqiu; Yu hua; Yu xinfeng
EPI_ISL_418441 to 418442, EPI_ISL_418502 to 418504, EPI_ISL_418507 to 418509, EPI_ISL_418511 to 418515	Hangzhou Center for Disease Control and Prevention	Inspection Center of Hangzhou Center for Disease Control and Prevention	Li jun; Pan jingcao; Wang haoqiu; Yu hua; Yu xinfeng
EPI_ISL_406970	Hangzhou Center for Disease and Control Microbiology Lab	Hangzhou Center for Disease and Control Microbiology Lab	Li Jun; Wang Haoqiu; Yu Hua; Yu Xinfeng
EPI_ISL_421221 to 421236, EPI_ISL_482575 to 482586	Hangzhou Center for Diseases Control and Prevention	Hangzhou Center for Diseases Control and Prevention	Haoqiu Wang; Hua Yu; Jun Li; Junfang Chen; Lingfeng Mao; Shuchang Chen; Xin Qian; Xinfen Yu; Xuchu Wang; Zhou Sun
EPI_ISL_1524889 to 1524895, EPI_ISL_1524906	Hannover Medical School, Institute of Virology	Hannover Medical School, Institute of Virology	Lars Steinbrück
EPI_ISL_1534314, EPI_ISL_1534437	Hanover Park CHC wc HPH	NHLS/UCT	Arash Iranzadeh; Bruna Galvao; Carolyn Williamson; Deelan Doolabh; Diana Hardie; Emmanuel SJ; Innocent Mudau; Kruger Marais; Lynn Tyers; Marvin Hsiao; Stephen Korsman; Tegally H; de Oliveira T
EPI_ISL_413025	Harborview Medical Center	UW Virology Lab	Alexander Greninger; Arun Nalla; Hong Xie; Keith Jerome; Pavitra Roychoudhury
EPI_ISL_413487	Harborview Medical Center	University of Washington Virology Lab	Alexander Greninger; Arun Nalla; Hong Xie; Keith Jerome; Pavitra Roychoudhury
EPI_ISL_1490543	Haugesund Hospital, laboratory for Medical Microbiology	Norwegian Institute of Public Health, Department of Virology	Atiya R Ali; Debech Nadia; Engebretsen Serina Beate; Garcia Llorente Ignacio; Hilde Elshaug; Hilde Vollan; Jon Bråte; Kamilla Heddeland Instefjord; Karoline Bragstad; Kathrine Stene-Johansen; Marie Paulsen Madsen; Olav Hungnes; Pedersen Benedikte Nevjen; Rasmus Riis Kopperud
EPI_ISL_1490268, EPI_ISL_1490271, EPI_ISL_1490524 to 1490525, EPI_ISL_1490530, EPI_ISL_1547561, EPI_ISL_1547564, EPI_ISL_1547566			
see above	Haukeland University Hospital, Dept. of Microbiology	Norwegian Institute of Public Health, Department of Virology	Atiya R Ali; Debech Nadia; Engebretsen Serina Beate; Garcia Llorente Ignacio; Hilde Elshaug; Hilde Vollan; Jon Bråte; Kamilla Heddeland Instefjord; Karoline Bragstad; Kathrine Stene-Johansen; Marie Paulsen Madsen; Olav Hungnes; Pedersen Benedikte Nevjen; Rasmus Riis Kopperud
EPI_ISL_429256	Health Sciences Technology Park, Avicena, 8, 18016 Granada. Spain	Sequencing and Bioinformatics Service FISABIO-Public Health	Almudena Rojas; Joaquín Mendoza; Pablo Mendoza
EPI_ISL_541942	Health and Environmental Research Institute of Gwangju	Health & Environment Institute of Gwangju	Ji-eun Lee; Min Ji Kim
EPI_ISL_540993 to 541007	Health and Environmental Research Institute of Gwangju Metropolitan city	Health and Environmental Research Institute of Gwangju Metropolitan city	Ji-eun Lee; Min Ji Kim
EPI_ISL_1478906	Heartland Healthcare Center - Henry	Illinois Department of Public Health - Springfield Lab	Bryan Sim; Gordon McCall
EPI_ISL_1534357, EPI_ISL_1534379, EPI_ISL_1534398	Heidelberg Clinic wc HBC	NHLS/UCT	Arash Iranzadeh; Bruna Galvao; Carolyn Williamson; Deelan Doolabh; Diana Hardie; Emmanuel SJ; Innocent Mudau; Kruger Marais; Lynn Tyers; Marvin Hsiao; Stephen Korsman; Tegally H; de Oliveira T
EPI_ISL_1534402	Heideveld CDC wc HVP	NHLS/UCT	Arash Iranzadeh; Bruna Galvao; Carolyn Williamson; Deelan Doolabh; Diana Hardie; Emmanuel SJ; Innocent Mudau; Kruger Marais; Lynn Tyers; Marvin Hsiao; Stephen Korsman; Tegally H; de Oliveira T
EPI_ISL_1534359, EPI_ISL_1534446, EPI_ISL_1534448	Heideveld Emergency Centre	NHLS/UCT	Arash Iranzadeh; Bruna Galvao; Carolyn Williamson; Deelan Doolabh; Diana Hardie; Emmanuel SJ; Innocent Mudau; Kruger Marais; Lynn Tyers; Marvin Hsiao; Stephen Korsman; Tegally H; de Oliveira T
EPI_ISL_1439466	Helios Universitätsklinikum Wuppertal: Universität Witten/Herdecke: Institut für Medizinische Labordiagnostik	Robert Koch Institute	
EPI_ISL_1391699 to 1391999, EPI_ISL_1420833 to 1420972, EPI_ISL_1420974, EPI_ISL_1420977, EPI_ISL_1420980, EPI_ISL_1420982, EPI_ISL_1420985, EPI_ISL_1420989, EPI_ISL_1420992, EPI_ISL_1420995, EPI_ISL_1420998, EPI_ISL_1421002, EPI_ISL_1421005, EPI_ISL_1421008, EPI_ISL_1421011, EPI_ISL_1421014, EPI_ISL_1421017, EPI_ISL_1421020, EPI_ISL_1421024, EPI_ISL_1421027, EPI_ISL_1421030, EPI_ISL_1421033, EPI_ISL_1421036, EPI_ISL_1421039, EPI_ISL_1421042, EPI_ISL_1421045, EPI_ISL_1421047, EPI_ISL_1421050, EPI_ISL_1421054, EPI_ISL_1421057, EPI_ISL_1421060, EPI_ISL_1421063, EPI_ISL_1421066, EPI_ISL_1421069, EPI_ISL_1421071, EPI_ISL_1421075, EPI_ISL_1421078, EPI_ISL_1421081, EPI_ISL_1421084, EPI_ISL_1421087, EPI_ISL_1421090, EPI_ISL_1421093, EPI_ISL_1421096, EPI_ISL_1421099, EPI_ISL_1421102, EPI_ISL_1421105, EPI_ISL_1421108, EPI_ISL_1421111, EPI_ISL_1421114, EPI_ISL_1421117, EPI_ISL_1421120, EPI_ISL_1421123, EPI_ISL_1421126, EPI_ISL_1421128, EPI_ISL_1421131, EPI_ISL_1421134, EPI_ISL_1421138, EPI_ISL_1421140, EPI_ISL_1421143, EPI_ISL_1421146, EPI_ISL_1421148, EPI_ISL_1421151, EPI_ISL_1421154, EPI_ISL_1421158, EPI_ISL_1421161, EPI_ISL_1421163, EPI_ISL_1421166, EPI_ISL_1421169, EPI_ISL_1421172, EPI_ISL_1421175, EPI_ISL_1421179, EPI_ISL_1421182, EPI_ISL_1421185, EPI_ISL_1421187, EPI_ISL_1421190, EPI_ISL_1421193, EPI_ISL_1421196, EPI_ISL_1421199, EPI_ISL_1421202, EPI_ISL_1421205, EPI_ISL_1421208, EPI_ISL_1421211, EPI_ISL_1421214, EPI_ISL_1421217, EPI_ISL_1421220, EPI_ISL_1421223, EPI_ISL_1421226, EPI_ISL_1421228 to 1421229, EPI_ISL_1421233, EPI_ISL_1421236, EPI_ISL_1421239, EPI_ISL_1421242, EPI_ISL_1421245, EPI_ISL_1421248, EPI_ISL_1421251, EPI_ISL_1421254, EPI_ISL_1421257, EPI_ISL_1421259, EPI_ISL_1421261 to 1421262, EPI_ISL_1421264, EPI_ISL_1421266, EPI_ISL_1421269, EPI_ISL_1421272, EPI_ISL_1421275, EPI_ISL_1421278, EPI_ISL_1421281, EPI_ISL_1421284, EPI_ISL_1421287, EPI_ISL_1421290, EPI_ISL_1421293, EPI_ISL_1421296, EPI_ISL_1421299 to 1421300, EPI_ISL_1421303, EPI_ISL_1421306, EPI_ISL_1421309, EPI_ISL_1421312, EPI_ISL_1421315, EPI_ISL_1421327 to 1421328, EPI_ISL_1421330, EPI_ISL_1421332, EPI_ISL_1421334 to 1421335, EPI_ISL_1421337, EPI_ISL_1421339, EPI_ISL_1421341 to 1421342, EPI_ISL_1421345, EPI_ISL_1421347, EPI_ISL_1421349 to 1421350, EPI_ISL_1421353, EPI_ISL_1421356, EPI_ISL_1421358, EPI_ISL_1421361, EPI_ISL_1421364, EPI_ISL_1421367, EPI_ISL_1421371, EPI_ISL_1421374, EPI_ISL_1421377 to 1421378, EPI_ISL_1421380 to 1421381, EPI_ISL_1421383, EPI_ISL_1421385, EPI_ISL_1421387 to 1421388, EPI_ISL_1421390, EPI_ISL_1421392, EPI_ISL_1421394, EPI_ISL_1421396, EPI_ISL_1421400, EPI_ISL_1421403, EPI_ISL_1421406, EPI_ISL_1421409, EPI_ISL_1421412, EPI_ISL_1421415, EPI_ISL_1421418, EPI_ISL_1421421, EPI_ISL_1421424, EPI_ISL_1421427, EPI_ISL_1421430, EPI_ISL_1421434, EPI_ISL_1421437, EPI_ISL_1421440, EPI_ISL_1421443, EPI_ISL_1421446, EPI_ISL_1421449, EPI_ISL_1421452, EPI_ISL_1421455, EPI_ISL_1421458, EPI_ISL_1421461, EPI_ISL_1421464, EPI_ISL_1421467, EPI_ISL_1421470, EPI_ISL_1421473 to 1421474, EPI_ISL_1421476, EPI_ISL_1421478 to 1421479, EPI_ISL_1421481, EPI_ISL_1421483, EPI_ISL_1421485, EPI_ISL_1421488, EPI_ISL_1421491, EPI_ISL_1421494, EPI_ISL_1421497, EPI_ISL_1421500, EPI_ISL_1421503, EPI_ISL_1421506, EPI_ISL_1421509, EPI_ISL_1421511, EPI_ISL_1421514, EPI_ISL_1421517, EPI_ISL_1421521, EPI_ISL_1421523, EPI_ISL_1421526, EPI_ISL_1421529, EPI_ISL_1421532, EPI_ISL_1421535, EPI_ISL_1421539, EPI_ISL_1421542, EPI_ISL_1421545, EPI_ISL_1421548, EPI_ISL_1421551, EPI_ISL_1421554 to 1421555, EPI_ISL_1421558, EPI_ISL_1421561, EPI_ISL_1421564, EPI_ISL_1421567, EPI_ISL_1421569, EPI_ISL_1421572, EPI_ISL_1421575, EPI_ISL_1421578, EPI_ISL_1421581, EPI_ISL_1421584, EPI_ISL_1421587, EPI_ISL_1421590, EPI_ISL_1421593, EPI_ISL_1421596 to 1421597, EPI_ISL_1421600, EPI_ISL_1421603, EPI_ISL_1421605, EPI_ISL_1421609, EPI_ISL_1421611 to 1421612, EPI_ISL_1421614, EPI_ISL_1421616, EPI_ISL_1421619, EPI_ISL_1421623, EPI_ISL_1421626, EPI_ISL_1421629, EPI_ISL_1421632, EPI_ISL_1421634, EPI_ISL_1421638, EPI_ISL_1421641, EPI_ISL_1421644, EPI_ISL_1421646, EPI_ISL_1421649, EPI_ISL_1421652, EPI_ISL_1421655, EPI_ISL_1421658, EPI_ISL_1421661, EPI_ISL_1421664, EPI_ISL_1421667, EPI_ISL_1421670, EPI_ISL_1421673, EPI_ISL_1421676, EPI_ISL_1421679, EPI_ISL_1421682, EPI_ISL_1421685, EPI_ISL_1421688, EPI_ISL_1421691, EPI_ISL_1421694, EPI_ISL_1421697, EPI_ISL_1421700, EPI_ISL_1421704, EPI_ISL_1421707, EPI_ISL_1421710, EPI_ISL_1421713, EPI_ISL_1421716, EPI_ISL_1421718 to 1421754, EPI_ISL_1443819 to 1443868, EPI_ISL_1443911 to 1444108, EPI_ISL_1444110, EPI_ISL_1444113, EPI_ISL_1444117, EPI_ISL_1444120, EPI_ISL_1444123, EPI_ISL_1444127, EPI_ISL_1444130, EPI_ISL_1444133, EPI_ISL_1444136, EPI_ISL_1444139, EPI_ISL_1444142, EPI_ISL_1444145, EPI_ISL_1444152, EPI_ISL_1444155, EPI_ISL_1444158, EPI_ISL_1444162, EPI_ISL_1444165, EPI_ISL_1444167 to 1444261, EPI_ISL_1460786 to 1461002, EPI_ISL_1479202 to 1479440, EPI_ISL_1480007 to 1480520, EPI_ISL_1493722 to 1493938, EPI_ISL_1511860 to 1511919, EPI_ISL_1511921 to 1512182, EPI_ISL_1512184 to 1512496, EPI_ISL_1512498 to 1512744, EPI_ISL_1553264 to 1553490, EPI_ISL_1553492 to 1553554, EPI_ISL_1553556 to 1553758, EPI_ISL_1553760 to 1553799, EPI_ISL_1553848 to 1554651, EPI_ISL_1554653 to 1554684, EPI_ISL_1554686 to 1554785, EPI_ISL_1554787 to 1554848, EPI_ISL_1554850 to 1554886			
see above	Helix/Illumina	Centers for Disease Control and Prevention Division of Viral Diseases, Pathogen Discovery	Adrian Paskey; Alexandre Bolze; Ary Ascencio; Ben L. Rambo-Martin; Benjamin Rambo-Martin; Brad Sickler; Charlotte Rivera-Garcia; Christine Tran; Christopher Gulvick; Clinton R. Paden; Dakota Howard; Darlene Wagner; David Becker; Dhwani Batra; Duncan MacCannell; Efren Sandoval; Eileen de Feo; Elizabeth Cirulli; Eric Allen; Geraint Levan; James Lu; Jan Antico; Jason Caravas; Jason Nguyen; Jimmy Ramirez; Jingtao Liu; Kara Moser; Kelly Schiabor Barrett; Kim Gietzen; Magnus Isaksson; Marc Laurent; Matthew Schmerer; Matthew Tolentino; Nicole L. Washington; Peter W. Cook; Phil Febbo; Ryan Cho; Scott Sammons; Shannon Wickline; Shatavia Morrison; Sherry Wang; Simon White; Summer Galloway; Suxiang Tong; Tyler Cassens; William Lee; Yvette Unoarumhi
EPI_ISL_451310 to 451311, EPI_ISL_487370, EPI_ISL_487377, EPI_ISL_487379, EPI_ISL_487381, EPI_ISL_501231 to 501238, EPI_ISL_501241 to 501242, EPI_ISL_501246, EPI_ISL_501248 to 501251			
see above	Hellenic Pasteur Institute, National Influenza Reference laboratory of Southern Greece & Unit of Bioinformatics and Applied Genomics	Hellenic Pasteur Institute, National Influenza Reference laboratory of Southern Greece & Unit of Bioinformatics and Applied Genomics	Andreas Mentis; Androniki Voulgari-Kokota; Antonios Kalliaropoulos; Aspasia Kontou; Athanasios Kossyvakis; Evangelidou Maria; Horefti Elina; Timokratis Karamitros; Vasiliki Pogka

EPI_ISL_501230	Hellenic Pasteur Institute, Public Health Laboratories	Hellenic Pasteur Institute, National Influenza Reference laboratory of Southern Greece & Unit of Bioinformatics and Applied Genomics	Andreas Mentis; Androniki Voulgari-Kokota; Antonios Kalliaropoulos; Aspasia Kontou; Athanasios Kossyvakis; Evangelidou Maria; Horefti Elina; Timokratis Karamitros; Vasiliki Pogka
EPI_ISL_430469	Hellenic Pasteur Institute, Public Health Laboratories	Hellenic Pasteur Institute, Public Health Laboratories, Unit of Bioinformatics and Applied Genomics	Andreas Mentis; Androniki Voulgari-Kokota; Antonios Kalliaropoulos; Aspasia Kontou; Athanasios Kossyvakis; Evangelidou Maria; Horefti Elina; Timokratis Karamitros; Vasiliki Pogka
EPI_ISL_428232 to 428236	Hematology Laboratory, Section of Molecular Diagnostics, University Clinical Centre, Medical University of Gdansk	Department of Virology, Faculty of Medicine, University of Helsinki, Helsinki, Finland	Aneta Szulc; Maciej Grzybek; Marlena Robakowska; Olli Vapalahti; Teemu Smura
EPI_ISL_450525 to 450530, EPI_ISL_512296 to 512303	Hematology Laboratory, Section of Molecular Diagnostics, University Clinical Centre, Medical University of Gdansk	Department of Virology, Faculty of Medicine, University of Helsinki, Helsinki, Finland	Aneta Szulc; Ewa Miosz; Maciej Grzybek; Marlena Robakowska; Olli Vapalahti; Teemu Smura
EPI_ISL_451648 to 451654	Hematology Laboratory, Section of Molecular Diagnostics, University Clinical Centre, Medical University of Gdansk	Laboratory of Recombinant Vaccines	Adam Sodol; Aneta Szulc; Boguslaw Szewczyk; Ewa Milosz; Krystyna Bienkowska-Szewczyk; Krzysztof Lewandowski; Lukasz Rabalski; Marlena Robakowska
EPI_ISL_530169 to 530171	Hennepin County Medical Center	Minnesota Department of Health, Public Health Laboratory	Jacob Garfin; Matt Plumb; and Xiong Wang
EPI_ISL_470568 to 470588, EPI_ISL_470598 to 470614, EPI_ISL_470651 to 470655	Hermes Pardini	Bioinformatics Laboratory / LNCC	Alexandra Gerber; Amilcar Tanuri; Ana Paula Guimarães; CADDE-group; Carolina Voloch; Ester Cerdeira Sabino; Filipe Romero; Ingra Morales Claro; Jaqueline Goes de Jesus; Laboratorio Hermes Pardini; Laboratorio Simile; Luiz Gonzaga Paula de Almeida; Mariane Talon; Nuno Rodrigues Faria; Renato Santana Aguiar e Ana Tereza Vasconcelos; Ronaldo da Silva Francisco Junior; Terezinha Marta Pereira; working group UFMG; working group UFRJ; Átila Duque Rossi
EPI_ISL_1534275, EPI_ISL_1534358	Hillside Clinic wc BFW	NHLS/UCT	Arash Iranzadeh; Bruna Galvao; Carolyn Williamson; Deelan Doolabh; Diana Hardie; Emmanuel SJ; Innocent Mudau; Kruger Marais; Lynn Tyers; Marvin Hsiao; Stephen Korsman; Tegally H; de Oliveira T
EPI_ISL_479903 to 479912	Himeji City Institute of Environment and Health	Pathogen Genomics Center, National Institute of Infectious Diseases	Hajime Kamiya; Kentaro Itokawa; Makoto Kuroda; Masanori Hashino; Motoi Suzuki; Rina Tanaka; Tsuyoshi Sekizuka
EPI_ISL_480178 to 480179	Hiroshima City Institute of Public Health	Pathogen Genomics Center, National Institute of Infectious Diseases	Hajime Kamiya; Kentaro Itokawa; Kota Noritsune; Makoto Kuroda; Masanori Hashino; Motoi Suzuki; Rina Tanaka; Tsuyoshi Sekizuka
EPI_ISL_498774 to 498775, EPI_ISL_513318 to 513320, EPI_ISL_544960, EPI_ISL_544975, EPI_ISL_544987, EPI_ISL_545007, EPI_ISL_545034 to 545036, EPI_ISL_547600, EPI_ISL_591485 to 591486, EPI_ISL_593652			
see above	Histopath	NSW Health Pathology - Institute of Clinical Pathology and Medical Research; Westmead Hospital; University of Sydney	CIDM-PH et al.
EPI_ISL_1472274 to 1472337	Hokkaido Institute of Public Health	Hokkaido Institute of Public Health	Hiroki Yamaguchi; Kazuhiro Okubo; Kentaro Itokawa; Makoto Kuroda; Masanori Hashino; Rina Tanaka; Tetsuya Ikeda; Tsuyoshi Sekizuka; Yuta Ohno
EPI_ISL_479812 to 479820	Hokkaido Institute of Public Health	Pathogen Genomics Center, National Institute of Infectious Diseases	Hajime Kamiya; Kentaro Itokawa; Makoto Kuroda; Masanori Hashino; Motoi Suzuki; Rika Komagome; Rina Tanaka; Tsuyoshi Sekizuka
EPI_ISL_412028, EPI_ISL_412030	Hong Kong Department of Health	School of Public Health, The University of Hon g Kong	Daniel K.W. Chu; Dominic N.C. Tsang; Leo L.M. Poon; Malik Peiris
EPI_ISL_414517, EPI_ISL_414519, EPI_ISL_414527 to 414528, EPI_ISL_414567, EPI_ISL_414569, EPI_ISL_414571, EPI_ISL_476801 to 476804			
see above	Hong Kong Department of Health	School of Public Health, The University of Hong Kong	Daniel K.W. Chu; Dominic N.C. Tsang; Leo L.M. Poon; Malik Peiris
EPI_ISL_412029	Hong Kong Department of Health	The University of Hong Kong	Daniel K.W. Chu; Dominic N.C. Tsang; Leo L.M. Poon; Malik Peiris
EPI_ISL_1478858, EPI_ISL_1500208 to 1500210, EPI_ISL_1553185	Hopedale Medical Complex	Illinois Department of Public Health - Springfield Lab	Bryan Sim; Gordon McCall
EPI_ISL_560581 to 560590	Hopital	National Reference Center for Viruses of Respiratory Infections, Institut Pasteur, Paris	Etienne Simon-Lorière; Fabiana Gambaro; Maud Vanpeene; Sylvie Behillil; Sylvie van der Werf; Vincent Enouf
EPI_ISL_418427	Hopital Privé de l'Est Lyonnais	CNR Virus des Infections Respiratoires - France SUD	Alexandre Gaymard; Antonin Bal; Bruno Lina; Carine Moustaud; Florence Morfin-Sherpa; Gregory Destras; Gwendolyne Burfin; Laurence Josset; Martine Valette; Maude Bouscambert-Duchamp; Raphaelle Lamy; Solenne Brun
EPI_ISL_418229	Hopital franco britannique - Laboratoire	National Reference Center for Viruses of Respiratory Infections, Institut Pasteur, Paris	Angela Brisebarre; Etienne Simon-Lorière; Flora Donati; Marianne Asso Bonnet; Marion Barbet; Maud Vanpeene; Mélanie Albert; Méline Bizard; Sylvie Behillil; Sylvie van der Werf; Vincent Enouf
EPI_ISL_416501	Hopital franco britannique - Service des Urgences	National Reference Center for Viruses of Respiratory Infections, Institut Pasteur, Paris	Angela Brisebarre; Etienne Simon-Lorière; Flora Donati; Marion Barbet; Maud Vanpeene; Méline Bizard; Mélnie Albert; Sylvie Behillil; Sylvie van der Werf; Vincent Enouf
EPI_ISL_434666	Hornefors Halsocentral	The Public Health Agency of Sweden	Anna Risberg; Anna-Malin Linde; Camilla Eiback; Karin Tegmark-Wisell; Maria Lind Karlberg; Mia Brytting; Olov Svartstrom; Oskar Karlsson Lindsjo; Theresa Enkirch
EPI_ISL_1533707	Hosp Mun Planalto Waldomiro de Paula	Instituto Adolfo Lutz, Interdiciplinary Procedures Center, Strategic Laboratory	Caio Vinicius Dias Lopes; Claudia Regina Gonçalves; Claudio Tavares Sacchi; Erica Valessa Ramos Gomes; Karoline Rodrigues Campos; Leonardo Jose Tadeu de Araujo
EPI_ISL_471562, EPI_ISL_471581 to 471582, EPI_ISL_515564, EPI_ISL_523977	Hosp. Municipal Prof. Dr. Alípio Corrêa Netto	Instituto Adolfo Lutz, Interdiciplinary Procedures Center, Strategic Laboratory	Claudia Regina Gonçalves; Claudio Tavares Sacchi; Erica Valessa Ramos Gomes
EPI_ISL_560598 to 560636, EPI_ISL_560643 to 560646, EPI_ISL_1389163 to 1389164, EPI_ISL_1389182 to 1389195, EPI_ISL_1389254 to 1389256, EPI_ISL_1389267, EPI_ISL_1389287 to 1389288, EPI_ISL_1389307 to 1389310, EPI_ISL_1389319, EPI_ISL_1389344, EPI_ISL_1389353, EPI_ISL_1416811, EPI_ISL_1416823 to 1416832, EPI_ISL_1416850 to 1416858, EPI_ISL_1416907, EPI_ISL_1416909 to 1416951, EPI_ISL_1416998 to 1417014, EPI_ISL_1417024 to 1417025, EPI_ISL_1417052 to 1417066, EPI_ISL_1417095 to 1417111, EPI_ISL_1443869 to 1443872, EPI_ISL_1443881 to 1443889, EPI_ISL_1443895, EPI_ISL_1443897 to 1443900, EPI_ISL_1443905 to 1443907, EPI_ISL_1465535 to 1465536, EPI_ISL_1465538 to 1465540, EPI_ISL_1465610, EPI_ISL_1489729, EPI_ISL_1489737 to 1489738, EPI_ISL_1516891 to 1516897, EPI_ISL_1516899 to 1516917, EPI_ISL_1516920 to 1516922, EPI_ISL_1516925 to 1516941, EPI_ISL_1516958 to 1516960, EPI_ISL_1517003 to 1517005, EPI_ISL_1517007 to 1517009, EPI_ISL_1517011 to 1517012, EPI_ISL_1517014 to 1517015, EPI_ISL_1517017 to 1517019, EPI_ISL_1517021 to 1517023, EPI_ISL_1517025 to 1517027, EPI_ISL_1517029 to 1517032, EPI_ISL_1517034 to 1517035, EPI_ISL_1517054 to 1517055, EPI_ISL_1517057 to 1517059, EPI_ISL_1517061 to 1517062, EPI_ISL_1517064 to 1517065, EPI_ISL_1517067 to 1517068, EPI_ISL_1517070, EPI_ISL_1517077, EPI_ISL_1517079 to 1517080, EPI_ISL_1517082, EPI_ISL_1517084, EPI_ISL_1517095, EPI_ISL_1517097 to 1517099, EPI_ISL_1517101, EPI_ISL_1517121, EPI_ISL_1517123 to 1517124, EPI_ISL_1517126 to 1517127, EPI_ISL_1517129 to 1517130, EPI_ISL_1517132 to 1517133, EPI_ISL_1517135 to 1517136, EPI_ISL_1517138 to 1517140, EPI_ISL_1517142 to 1517143, EPI_ISL_1517145 to 1517146, EPI_ISL_1517148 to 1517150, EPI_ISL_1517152 to 1517154, EPI_ISL_1517156, EPI_ISL_1517158 to 1517159, EPI_ISL_1517161 to 1517163, EPI_ISL_1517165 to 1517166, EPI_ISL_1517168 to 1517170, EPI_ISL_1517172 to 1517174, EPI_ISL_1517176 to 1517178, EPI_ISL_1517472 to 1517478, EPI_ISL_1517483 to 1517488, EPI_ISL_1517490 to 1517493, EPI_ISL_1517495, EPI_ISL_1525261 to 1525266, EPI_ISL_1526778 to 1526783, EPI_ISL_1526785 to 1526788, EPI_ISL_1526793 to 1526794, EPI_ISL_1528181 to 1528182, EPI_ISL_1528186 to 1528192, EPI_ISL_1528194 to 1528198, EPI_ISL_1528203 to 1528210, EPI_ISL_1528214, EPI_ISL_1528219 to 1528220, EPI_ISL_1528222, EPI_ISL_1528229 to 1528230, EPI_ISL_1528232 to 1528234, EPI_ISL_1528236, EPI_ISL_1528239 to 1528241, EPI_ISL_1540445, EPI_ISL_1540447, EPI_ISL_1540449 to 1540450, EPI_ISL_1554888 to 1554903, EPI_ISL_1554905 to 1554949, EPI_ISL_1554952, EPI_ISL_1554955, EPI_ISL_1554958, EPI_ISL_1554961			
see above	Hospital	National Reference Center for Viruses of Respiratory Infections, Institut Pasteur, Paris	Adeline Baron; Adrien Pain; Alain Delepine; Alexandra Ducancelle; Amaury Vaysse; Angela Brisebarre; Anne Cady; Anne Holstein; Ben Hadj Yahia; Camille Capel; Catherine Coignard; Christophe Malabat; CléMence Guillaume; Corinne Maufrais; CéLine Bressollette; CéLine Ramanantsoa; Damien Mornico; Dominique Descamps; Dorval I.; Ducancelle Alexandra; Eleha Guillotel; Emmanuelle Permal; Eric Farfour; Esther Gyde; Etienne Simon-Lorière; Fabiana Gambaro; Farfour Eric; Febreau Christine; FrançOise Jouble; FrançOise Lunel Fabiani; FréDéRique Canis; Frédéric Lemoine; Gael Millot; Guillotel Eleha; Guinard JéRome; H Bisiau; Haciba Moudjahed; Hochart A; Ilka Engelmann; Isabelle Woercel; Jean Baptiste Bour; Jean-Baptiste Bour; Jean-FrançOis Comes; JéRome Guinard; Lechat; Leruez-Ville Marianne; Louise Lefrançois; LéA Pilorge; Marion Barbet; Martres Pascale; Maud Vanpeene; Mj Dufour; Mj Dufour; Méline Bizard; Nabil Gastli; Odile Morvan; Odile Petsaris; OphéLie Said-Delattre; Pascale Martres; Patricia Stoessel-Thouvenin; Pierre Lechat; Pilorge LéA; Rajaonarison; Ronan Pichard; S. Van Agt; Sellam; Serazin ValéRie; Sophie Chalmin; StéPhanie Guyomard-Rabenirina; Sylvaine Bastian; Sylvie Behillil; Sylvie van der Werf; SéVerine Haouisee; Thibault Guinoiseau; ValéRie Serazin; Victoire; Victoire Baillet; Vincent Enouf
EPI_ISL_434366 to 434386, EPI_ISL_450724 to 450737	Hospital AZ Rivierenland	Institute of Tropical Medicine	; Colin Anthony; Philippe Selhorst
EPI_ISL_1443196 to 1443198	Hospital Aliança	Hospital São Rafael - IDOR	Aquiles Assunção Camelier; Aurea Angelica Paste; Bruno Solano de Freitas Souza; Carolina Kymie Vasques Nonaka; Elves A.P. Maciel; Isadora Cristina de Siqueira; Karoline Almeida Félix de Sousa; Margarida Celia L. C. Neves; Tiago Gräf; Victor Costa Araujo; Yasmin Santos Freitas Macêdo
EPI_ISL_1469707	Hospital Bom Jesus	Epiclin	Ana Paula Muterle; Carolina Comerlato; Eliana Márcia Da Ros Wendland; Fernando Hayashi Sant'Anna; Janira Prichula; Juliana Comerlato

EPI_ISL_471554	Hospital Bosque da Saúde	Instituto Adolfo Lutz, Interdiciplinary Procedures Center, Strategic Laboratory	Claudia Regina Gonçalves; Claudio Tavares Sacchi; Erica Valessa Ramos Gomes
EPI_ISL_491439	Hospital Calderon Guardia	Inciensa, Instituto Costarricense de Investigación y Enseñanza en Nutrición y Salud	Adriana Godinez & Melany Calderon; Claudio Soto-Garita; Estela Cordero; Francisco Duarte; Hebleen Brenes
EPI_ISL_539559 to 539560	Hospital Campo Arañuelo	Instituto de Salud Carlos III	A. Monzón; F. Casas; G. Rodríguez; I; I. Jiménez; Iglesias-Caballero; J. López; M. Camarero; M. Cuesta; M. González-Esguevillas; M. Molinero Calamita; M. Zaballos; P. Jiménez; S. Juliá; S. Pozo; S. Varona
EPI_ISL_1383812 to 1384046, EPI_ISL_1522579, EPI_ISL_1522582, EPI_ISL_1522597 to 1522598, EPI_ISL_1522607, EPI_ISL_1522623, EPI_ISL_1522631, EPI_ISL_1522634, EPI_ISL_1522642, EPI_ISL_1522660, EPI_ISL_1522672, EPI_ISL_1522677, EPI_ISL_1522688, EPI_ISL_1522700, EPI_ISL_1522711 to 1522712, EPI_ISL_1522717, EPI_ISL_1522725, EPI_ISL_1522742, EPI_ISL_1522772, EPI_ISL_1522779, EPI_ISL_1522848 to 1522893, EPI_ISL_1523047 to 1523061, EPI_ISL_1523127, EPI_ISL_1523131, EPI_ISL_1523144, EPI_ISL_1523147, EPI_ISL_1523151 to 1523152, EPI_ISL_1523157, EPI_ISL_1523159, EPI_ISL_1523161, EPI_ISL_1523166 to 1523167, EPI_ISL_1523170, EPI_ISL_1523176 to 1523177, EPI_ISL_1523179, EPI_ISL_1523185, EPI_ISL_1523188, EPI_ISL_1523196 to 1523198, EPI_ISL_1523202, EPI_ISL_1523206, EPI_ISL_1523219 to 1523220, EPI_ISL_1523256 to 1523275, EPI_ISL_1523347 to 1523352			
see above	Hospital Center Emile Mayrisch	Laboratoire national de sante, Microbiology, Microbial Genomics Platform	Anke Wienecke-Baldacchino; Catherine Ragimbeau; Cynthia Oxacelay; Fatu Djabi; Jessica Tapp; Lise Pignon; Raoul Salmon; Tamir Abdelrahman
EPI_ISL_1384047 to 1384149, EPI_ISL_1522574, EPI_ISL_1522620, EPI_ISL_1522630, EPI_ISL_1522635, EPI_ISL_1522637, EPI_ISL_1522648, EPI_ISL_1522655, EPI_ISL_1522662, EPI_ISL_1522682, EPI_ISL_1522687, EPI_ISL_1522694, EPI_ISL_1522741, EPI_ISL_1522750, EPI_ISL_1522753, EPI_ISL_1522773, EPI_ISL_1522775, EPI_ISL_1522777, EPI_ISL_1522788, EPI_ISL_1522894 to 1522904, EPI_ISL_1523062 to 1523069, EPI_ISL_1523112, EPI_ISL_1523126, EPI_ISL_1523139, EPI_ISL_1523153, EPI_ISL_1523156, EPI_ISL_1523160, EPI_ISL_1523163 to 1523164, EPI_ISL_1523174, EPI_ISL_1523189, EPI_ISL_1523193, EPI_ISL_1523195, EPI_ISL_1523204, EPI_ISL_1523207, EPI_ISL_1523213, EPI_ISL_1523222, EPI_ISL_1523276 to 1523288			
see above	Hospital Center Luxembourg	Laboratoire national de sante, Microbiology, Microbial Genomics Platform	Anke Wienecke-Baldacchino; Catherine Ragimbeau; Fatu Djabi; Jean-Hugues Francois; Jessica Tapp; Lise Pignon; Michel Kohnen; Raoul Salmon; Tamir Abdelrahman
EPI_ISL_1396364 to 1396373	Hospital Central Mendoza	Nodo de Secuenciación Tierra del Fuego - Hospital Regional Ushuaia - Centro Austral De Investigaciones Científicas - Universidad Nacional De Tierra Del Fuego on behalf of 'Proyecto Argentino Interinstitucional de genomica de SARS-CoV-2' (PAIS Consortium)	Aguirre Carolina; Alberto Carena; Alejandro Ezequiel Rojas; Andrea Falaschi; Andrea Naser; Bosio Lia; Carlos Espul; Cristina Fernanda Nardi; Fernando Gallego; Héctor Horacio Cuello; Ivan Dario Gramundi; Luciano Lima; Maria Belen Peralta Roca; María Liliana Videla; Pablo Rico; Patricia Robledo; Santiago Guillermo Ceballos; Valeria Fontana
EPI_ISL_574580	Hospital Cidade Tiradentes Carmen Prudente	Instituto Adolfo Lutz, Interdiciplinary Procedures Center, Strategic Laboratory	Claudia Regina Gonçalves; Claudio Tavares Sacchi; Erica Valessa Ramos Gomes; Karoline Rodrigues Campos
EPI_ISL_527743 to 527744	Hospital Cima	Inciensa, Instituto Costarricense de Investigación y Enseñanza en Nutrición y Salud	Adriana Godinez & Melany Calderon; Claudio Soto-Garita; Estela Cordero; Francisco Duarte; Hebleen Porras
EPI_ISL_462479	Hospital Clinic	Instituto de Salud Carlos III	A. Monzón; F. Casas; I; I. Jiménez; Iglesias-Caballero; M. Camarero; M. Cuesta; M. González-Esguevillas; M. Molinero Calamita; M. Zaballos; M.A Marcos; P. Jiménez; S. Juliá; S. Pozo; S. Varona
EPI_ISL_491441 to 491442, EPI_ISL_491455	Hospital Clinica Biblica	Inciensa, Instituto Costarricense de Investigación y Enseñanza en Nutrición y Salud	Adriana Godinez & Melany Calderon; Claudio Soto-Garita; Estela Cordero; Francisco Duarte; Hebleen Brenes
EPI_ISL_539533 to 539556	Hospital Clínic	Instituto de Salud Carlos III	A. Monzón; F. Casas; I; I. Jiménez; Iglesias-Caballero; M. Camarero; M. Cuesta; M. González-Esguevillas; M. Molinero Calamita; M. Zaballos; M.A Marcos; P. Jiménez; S. Juliá; S. Pozo; S. Varona
EPI_ISL_480315 to 480320	Hospital Clínica Bíblica	Charité Virology-University of Costa Rica	Andres Moreira-Soto; Eugenia Corrales-Aguilar; Ignacio Postigo-Hidalgo; Jan Felix Drexler; Karla Sofía Gutiérrez
EPI_ISL_539500 to 539502, EPI_ISL_539509 to 539512, EPI_ISL_578190 to 578191	Hospital Clínico Universitario Lozano Blesa	Instituto de Salud Carlos III	A. Monzón; F. Casas; I; I. Jiménez; Iglesias-Caballero; M. Camarero; M. Cuesta; M. González-Esguevillas; M. Molinero Calamita; M. Zaballos; P. Jiménez; R. Benito; S. Juliá; S. Pozo; S. Varona
EPI_ISL_1423919 to 1423925	Hospital Clínico Universitario Virgen de la Arrixaca (Murcia)	SeqCOVID-SPAIN consortium/IBV(CSIC)	Antonio Moreno Docón; Genoveva Yagüe and SeqCOVID-SPAIN consortium; Manuel Segovia Hernández
EPI_ISL_467262 to 467297, EPI_ISL_500162, EPI_ISL_500167, EPI_ISL_500209, EPI_ISL_500219, EPI_ISL_500221, EPI_ISL_510268 to 510304			
see above	Hospital Clínico Universitario de Santiago de Compostela	SeqCOVID-SPAIN consortium/IBV(CSIC)	Amparo Coira Nieto; Antonio Aguilera Guirao; Gema Barbeito Castiñeiras; José Javier Costa Alcalde; Mª Luisa Pérez del Molino Bernal; Rocio Trastoy Pena and SeqCOVID-SPAIN consortium
EPI_ISL_541037 to 541066	Hospital Clínico Universitario de Santiago de Compostela	SeqCOVID-SPAIN consortium/Institute of Biomedicine of Valencia, IBV-CSIC	Amparo Coira Nieto; Antonio Aguilera Guirao; Gema Barbeito Castiñeiras; José Javier Costa Alcalde; Mª Luisa Pérez del Molino Bernal; Rocio Trastoy Pena and SeqCOVID-SPAIN consortium
EPI_ISL_455344 to 455349, EPI_ISL_539567 to 539568	Hospital Comarcal de Melilla	Instituto de Salud Carlos III	A. Monzón; C. Ezpeleta; F. Casas; I; I. Jiménez; I. Pérez; Iglesias-Caballero; J. López; M. Camarero; M. Cuesta; M. González-Esguevillas; M. Molinero Calamita; M. Zaballos; P. Jiménez; S. Juliá; S. Pozo; S. Varona
EPI_ISL_462477, EPI_ISL_539499	Hospital Costa del Sol	Instituto de Salud Carlos III	A. Monzón; F. Casas; F. Fernández; I; I. Jiménez; Iglesias-Caballero; M. Camarero; M. Cuesta; M. González-Esguevillas; M. Molinero Calamita; M. Zaballos; P. Jiménez; S. Juliá; S. Pozo; S. Varona
EPI_ISL_512665	Hospital De Las Mujeres Dr. Adolfo Carit	Inciensa, Instituto Costarricense de Investigación y Enseñanza en Nutrición y Salud	Adriana Godinez & Melany Calderon; Claudio Soto-Garita; Estela Cordero; Francisco Duarte; Hebleen Porras
EPI_ISL_512655, EPI_ISL_512672 to 512675, EPI_ISL_527750	Hospital De Niños Dr. Carlos Saenz Herrera [San Jose/San Jose]	Inciensa, Instituto Costarricense de Investigación y Enseñanza en Nutrición y Salud	Adriana Godinez & Melany Calderon; Claudio Soto-Garita; Estela Cordero; Francisco Duarte; Hebleen Porras
EPI_ISL_1469562, EPI_ISL_1469581, EPI_ISL_1469711, EPI_ISL_1469734, EPI_ISL_1469794 to 1469795	Hospital Dia e Pronto Atendimento	Epiclin	Ana Paula Muterle; Carolina Comerlato; Eliana Márcia Da Ros Wendland; Fernando Hayashi Sant'Anna; Janira Prichula; Juliana Comerlato
EPI_ISL_574591 to 574592, EPI_ISL_603030	Hospital Domingos Leonardo Ceravolo Presidente Prudente	Instituto Adolfo Lutz, Interdiciplinary Procedures Center, Strategic Laboratory	Claudia Regina Gonçalves; Claudio Tavares Sacchi; Erica Valessa Ramos Gomes; Karoline Rodrigues Campos
EPI_ISL_547578	Hospital Doutor Domingos Leonardo Cerávolo	Instituto Adolfo Lutz, Interdiciplinary Procedures Center, Strategic Laboratory	Claudia Regina Gonçalves; Claudio Tavares Sacchi; Erica Valessa Ramos Gomes; Karoline Rodrigues Campos
EPI_ISL_527752	Hospital Dr. Rafael A. Calderon Guardia	Inciensa, Instituto Costarricense de Investigación y Enseñanza en Nutrición y Salud	Adriana Godinez & Melany Calderon; Claudio Soto-Garita; Estela Cordero; Francisco Duarte; Hebleen Porras
EPI_ISL_512654	Hospital Dr. Rafael A. Calderon Guardia [San Jose/San Jose]	Inciensa, Instituto Costarricense de Investigación y Enseñanza en Nutrición y Salud	Adriana Godinez & Melany Calderon; Claudio Soto-Garita; Estela Cordero; Francisco Duarte; Hebleen Porras
EPI_ISL_574594	Hospital Escola da Universidade de Taubate	Instituto Adolfo Lutz, Interdiciplinary Procedures Center, Strategic Laboratory	Claudia Regina Gonçalves; Claudio Tavares Sacchi; Erica Valessa Ramos Gomes; Karoline Rodrigues Campos
EPI_ISL_1396425, EPI_ISL_1396428	Hospital Español de Rosario	Laboratorio Mixto de Biotecnología Acuática (LMBA) on behalf of 'Proyecto Argentino Interinstitucional de genomica de SARS-CoV-2' (PAIS Consortium)	Adriana Giri; Agustina Cerri; Ana Cavatorta; Ana Paletta; Diego Chouhy; Elisa Bolatti; Elizabeth Tapia (argenTAG); Federico Remes Lenicov; Flavio Spetale; Gastón Viarengo; Ignacio García Labari; Javier Murillo; Joaquín Ezpeleta; Julian Acosta; Laura Angelone; Leandro Ciappina; María Re; Pablo Casal; Pilar Bulacio; Silvana Spinelli; Silvia Arranz; Sofía Lavista Llanos; Vanina Villanova; Victoria Posner
EPI_ISL_574588, EPI_ISL_583490	Hospital Estadual Sumare	Instituto Adolfo Lutz, Interdiciplinary Procedures Center, Strategic Laboratory	Claudia Regina Gonçalves; Claudio Tavares Sacchi; Erica Valessa Ramos Gomes; Karoline Rodrigues Campos
EPI_ISL_1533726	Hospital Estadual de Campanha Covid 19 Barradas	Instituto Adolfo Lutz, Interdiciplinary Procedures Center, Strategic Laboratory	Caio Vinicius Dias Lopes; Claudia Regina Gonçalves; Claudio Tavares Sacchi; Erica Valessa Ramos Gomes; Karoline Rodrigues Campos; Leonardo Jose Tadeu de Araujo
EPI_ISL_583501	Hospital Estadual de CampanhaCOVID 19 Barradas	Instituto Adolfo Lutz, Interdiciplinary Procedures Center, Strategic Laboratory	Claudia Regina Gonçalves; Claudio Tavares Sacchi; Erica Valessa Ramos Gomes; Karoline Rodrigues Campos
EPI_ISL_1468412, EPI_ISL_1468443,	Hospital Estadual de Mirandopolis	Instituto Adolfo Lutz, Interdiciplinary Procedures Center,	Caio Vinicius Dias Lopes; Claudia Regina Gonçalves; Claudio Tavares Sacchi; Erica Valessa Ramos Gomes; Karoline Rodrigues Campos

EPI_ISL_1498917		Strategic Laboratory	
EPI_ISL_1533700	Hospital Estadual de Sapopemba Sao Paulo	Instituto Adolfo Lutz, Interdiciplinary Procedures Center, Strategic Laboratory	Caio Vinicius Dias Lopes; Claudia Regina Gonçalves; Claudio Tavares Sacchi; Erica Valessa Ramos Gomes; Karoline Rodrigues Campos; Leonardo Jose Tadeu de Araujo
EPI_ISL_1533697	Hospital Estadual de Vila Alpina	Instituto Adolfo Lutz, Interdiciplinary Procedures Center, Strategic Laboratory	Caio Vinicius Dias Lopes; Claudia Regina Gonçalves; Claudio Tavares Sacchi; Erica Valessa Ramos Gomes; Karoline Rodrigues Campos; Leonardo Jose Tadeu de Araujo
EPI_ISL_1533701, EPI_ISL_1533709 to 1533710	Hospital Estadual de Vila Alpina Org Social Seconci Sao Paulo	Instituto Adolfo Lutz, Interdiciplinary Procedures Center, Strategic Laboratory	Caio Vinicius Dias Lopes; Claudia Regina Gonçalves; Claudio Tavares Sacchi; Erica Valessa Ramos Gomes; Karoline Rodrigues Campos; Leonardo Jose Tadeu de Araujo
EPI_ISL_491443, EPI_ISL_491447	Hospital Fernando Escalante Pradilla	Inciensa, Instituto Costarricense de Investigación y Enseñanza en Nutrición y Salud	Adriana Godinez & Melany Calderon; Claudio Soto-Garita; Estela Cordero; Francisco Duarte; Hebleen Brenes
EPI_ISL_476496, EPI_ISL_476561	Hospital Garrahan	Héritas	Andrea Mangano; Cristian Rohr; Dalmacio Pereyra; Fabian Fay; Maria Florencia Fernandez; Martin Vazquez; Mauricio Grisolia; Roberta Crespo
EPI_ISL_578186	Hospital General Juan Ramón Jiménez	Instituto de Salud Carlos III	A. Monzón; F. Casas; I; I. Jiménez; Iglesias-Caballero; J. Saavedra; M. Camarero; M. Cuesta; M. González-Esguevillas; M. Molinero Calamita; M. Zaballos; P. Jiménez; S. Juliá; S. Pozo; S. Varona
EPI_ISL_424731	Hospital General Regional No.66, Ciudad Juárez, Chihuahua.	Laboratorio Central de Epidemiología-DLVIE / Laboratorio de Secuenciación-Centro de Instrumentos. Instituto Mexicano del Seguro Social	Araiza-Rodríguez A; Arias C; Barrera-Badillo G; Becerril-Vargas E; Borja-Aburto VH.; Boukadida C; Garcés-Ayala F; García-Andrade LA; Gaytán-Cervantes FJ; González-Bonilla CR; González-Torres C; Grajales-Muñiz C; Grande R; Hernández-Rivas L; Ibarra-González V; Isa P; Ledesma-Barrientos F; López-Martínez I; Martínez-Orozco JA; Mendieta-Condado E; Muñoz-Medina JE; Mújica-Sánchez M; Pulido F. López S; Ramos-Cervantes P; Ramírez-González JE; Ruiz-Palacios GM; Sánchez A; Taboada B; Vázquez G; Vázquez-Pérez JA; de León-Garduño AP; Ávila-Ríos S
EPI_ISL_1401465 to 1401472, EPI_ISL_1476942 to 1476986, EPI_ISL_1477050 to 1477062, EPI_ISL_1511203 to 1511208, EPI_ISL_1524803 to 1524850, EPI_ISL_1547363, EPI_ISL_1547368 to 1547369			
see above	Hospital General Universitario Gregorio Marañón	Hospital General Universitario Gregorio Marañón	Cristina Rodriguez-Grande; Darío García de Viedma; Laura Pérez-Lago; Patricia Muñoz; Pedro Sola Campoy; Pilar Catalán; Sergio Buenestado Serrano
EPI_ISL_467184 to 467261, EPI_ISL_481041 to 481109, EPI_ISL_510103 to 510151, EPI_ISL_510165 to 510245, EPI_ISL_541878 to 541941, EPI_ISL_582812 to 582832, EPI_ISL_582843 to 582844			
see above	Hospital General Universitario Gregorio Marañón	SeqCOVID-SPAIN consortium/IBV(CSIC)	Darío García de Viedma; Darío García de Viedma and SeqCOVID-SPAIN consortium; Jon Sicilia; Julia Suárez; Laura Pérez-Lago; Marta Herranz; Patricia Muñoz; Patricia Muñoz and SeqCOVID-SPAIN consortium; Pilar Catalán
EPI_ISL_1441419, EPI_ISL_1441421, EPI_ISL_1441423 to 1441424, EPI_ISL_1441426, EPI_ISL_1441429, EPI_ISL_1441431, EPI_ISL_1441433, EPI_ISL_1441435 to 1441436, EPI_ISL_1441438, EPI_ISL_1441441 to 1441442, EPI_ISL_1441445, EPI_ISL_1441447, EPI_ISL_1441449 to 1441450, EPI_ISL_1441452, EPI_ISL_1441454, EPI_ISL_1441456, EPI_ISL_1441458, EPI_ISL_1441460, EPI_ISL_1441462, EPI_ISL_1441464, EPI_ISL_1441466, EPI_ISL_1441468, EPI_ISL_1441470 to 1441471, EPI_ISL_1441474 to 1441475, EPI_ISL_1441477, EPI_ISL_1441480, EPI_ISL_1441482, EPI_ISL_1441484 to 1441485, EPI_ISL_1441487, EPI_ISL_1441489, EPI_ISL_1441491, EPI_ISL_1441493, EPI_ISL_1441495, EPI_ISL_1441497, EPI_ISL_1441499, EPI_ISL_1441504, EPI_ISL_1441509, EPI_ISL_1441514, EPI_ISL_1441518, EPI_ISL_1441523, EPI_ISL_1441527, EPI_ISL_1441533, EPI_ISL_1441538 to 1441539, EPI_ISL_1441541, EPI_ISL_1441543, EPI_ISL_1441545, EPI_ISL_1441547, EPI_ISL_1441552, EPI_ISL_1441554, EPI_ISL_1441556, EPI_ISL_1441558, EPI_ISL_1441560, EPI_ISL_1441562, EPI_ISL_1441564, EPI_ISL_1441566, EPI_ISL_1441569, EPI_ISL_1441573, EPI_ISL_1441578, EPI_ISL_1441583, EPI_ISL_1441588, EPI_ISL_1441594, EPI_ISL_1441596, EPI_ISL_1441598, EPI_ISL_1441600			
see above	Hospital General Universitario de Alicante - Instituto de Investigación Sanitaria y Biomédica de Alicante	SeqCOVID-SPAIN consortium/IBV(CSIC)	Carmen Molina Pardines and SeqCOVID-SPAIN consortium; Maripaz Ventero Martín
EPI_ISL_539523	Hospital General de Segovia	Instituto de Salud Carlos III	A. Monzón; F. Casas; I; I. Jiménez; Iglesias-Caballero; M. Camarero; M. Cuesta; M. González-Esguevillas; M. Molinero Calamita; M. Zaballos; P. Jiménez; S. Hernando; S. Juliá; S. Pozo; S. Varona
EPI_ISL_418245 to 418246	Hospital General y Universitario de Guadalajara	Instituto de Salud Carlos III	A. Monzón; F. Casas; I. Gonzalez-Praetorius A.; I. Jiménez; Iglesias-Caballero; M. Camarero; M. Cuesta; M. González-Esguevillas; M. Molinero Calamita; M. Zaballos; P. Jiménez; S. Juliá; S. Pozo; S. Varona
EPI_ISL_471541, EPI_ISL_523971	Hospital Geral Santa Marcelina	Instituto Adolfo Lutz, Interdiciplinary Procedures Center, Strategic Laboratory	Claudia Regina Gonçalves; Claudio Tavares Sacchi; Erica Valessa Ramos Gomes
EPI_ISL_1533725	Hospital Geral de Guarulhos	Instituto Adolfo Lutz, Interdiciplinary Procedures Center, Strategic Laboratory	Caio Vinicius Dias Lopes; Claudia Regina Gonçalves; Claudio Tavares Sacchi; Erica Valessa Ramos Gomes; Karoline Rodrigues Campos; Leonardo Jose Tadeu de Araujo
EPI_ISL_534317	Hospital Geral de Itapevi	Instituto Adolfo Lutz, Interdiciplinary Procedures Center, Strategic Laboratory	Claudia Regina Gonçalves; Claudio Tavares Sacchi; Erica Valessa Ramos Gomes
EPI_ISL_1533723	Hospital Geral de Itaquaquecetuba	Instituto Adolfo Lutz, Interdiciplinary Procedures Center, Strategic Laboratory	Caio Vinicius Dias Lopes; Claudia Regina Gonçalves; Claudio Tavares Sacchi; Erica Valessa Ramos Gomes; Karoline Rodrigues Campos; Leonardo Jose Tadeu de Araujo
EPI_ISL_603037	Hospital Geral de Pedreira	Instituto Adolfo Lutz, Interdiciplinary Procedures Center, Strategic Laboratory	Claudia Regina Gonçalves; Claudio Tavares Sacchi; Erica Valessa Ramos Gomes; Karoline Rodrigues Campos
EPI_ISL_515555	Hospital Geral de Vila Nova Cachoeirinha	Instituto Adolfo Lutz, Interdiciplinary Procedures Center, Strategic Laboratory	Claudia Regina Gonçalves; Claudio Tavares Sacchi; Erica Valessa Ramos Gomes
EPI_ISL_574595	Hospital Geral de Vila Penteado Dr. Jose Pamgella	Instituto Adolfo Lutz, Interdiciplinary Procedures Center, Strategic Laboratory	Claudia Regina Gonçalves; Claudio Tavares Sacchi; Erica Valessa Ramos Gomes; Karoline Rodrigues Campos
EPI_ISL_1527012, EPI_ISL_1527015, EPI_ISL_1527021	Hospital Guápiles	Inciensa, Instituto Costarricense de Investigación y Enseñanza en Nutrición y Salud	Barboza-Arguedas E & Cerdas-Quesada C; Pérez-Corrales C
EPI_ISL_471267 to 471268, EPI_ISL_481244 to 481248, EPI_ISL_574431	Hospital IESS Babahoyo	Institute of Microbiology, Universidad San Francisco de Quito	Belén Prado-Vivar; Bernardo Gutiérrez; Carla Torres; Fernanda Zurita; Francisco Cordova; Gabriel Trueba; Juan José Guadalupe; Killen Briones-Claudette; Killen Briones-Zamora; Michelle Grunauer; Monica Becerra-Wong; Ninfa Henriquez; Patricio Rojas-Silva; Paúl Cárdenas; Sully Márquez; Verónica Barragán
EPI_ISL_412964	Hospital Israelita Albert Einstein	Instituto Adolfo Lutz Interdisciplinary Procedures Center Strategic Laboratory	Andrew Rambaut; Claudia Regina Gonçalves; Claudio Tavares Sacchi; Daniela Bernardes Borges da Silva; Ester Cerdeira Sabino; Flávia Cristina da Silva Sales; Ingra Morales Claro; Jaqueline Goes de Jesus; Joshua Quick; Maria do Carmo; Nicholas James Loman; Nuno Rodrigues Faria; Sampaio Tavares Timenetsky
EPI_ISL_414014, EPI_ISL_416033 to 416034	Hospital Israelita Albert Einstein	Instituto Adolfo Lutz, Interdiciplinary Procedures Center, Strategic Laboratory	Adriana Bugno; Adriano Abbud; Carlos Henrique Camargo; Claudia Regina Gonçalves; Claudio Tavares Sacchi; Daniela Bernardes Borges da Silva; Erica Valessa Ramos Gomes; Ester Cerdeira Sabino; Fabiana Cristina Pereira dos Santos; Katia Correia dos Santos; Maria do Carmo Sampaio Tavares Timenetsky; Maria do Carmo Sampaito Tavares Timenetsky; Simone Guadagnucci Morillo; Terezinha Maria de Paiva
EPI_ISL_413016	Hospital Israelita Albert Einstein	Instituto Adolfo Lutz, Interdisciplinary Procedures Center, Strategic Laboratory	Andrew Rambaut; Claudia Regina Gonçalves; Claudio Tavares Sacchi; Ester Cerdeira Sabino; Fabiana Cristina Pereira dos Santos; Flávia Cristina da Silva Sales; Ingra Morales Claro; Jaqueline Goes de Jesus; Joshua Quick; Maria do Carmo Sampaio Tavares Timenetsky; Nicholas James Loman; Nuno Rodrigues Faria
EPI_ISL_523957	Hospital Itamaraty	Instituto Adolfo Lutz, Interdiciplinary Procedures Center, Strategic Laboratory	Claudia Regina Gonçalves; Claudio Tavares Sacchi; Erica Valessa Ramos Gomes
EPI_ISL_1482628	Hospital Margarita Maza de Juárez	Microbial Genomics Laboratory	Alejandra Garcia-Gasca; Bruno Gomez-Gil; Daniel Fregoso-Rueda; Julissa Enciso-Ibarra
EPI_ISL_527741	Hospital Metropolitano	Inciensa, Instituto Costarricense de Investigación y Enseñanza en Nutrición y Salud	Adriana Godinez & Melany Calderon; Claudio Soto-Garita; Estela Cordero; Francisco Duarte; Hebleen Porras
EPI_ISL_524462	Hospital Metropolitano	Instituto Adolfo Lutz, Interdiciplinary Procedures Center, Strategic Laboratory	Claudia Regina Gonçalves; Claudio Tavares Sacchi; Erica Valessa Ramos Gomes
EPI_ISL_458236, EPI_ISL_480312 to 480314	Hospital Mexico	Charité Virology-University of Costa Rica	Andres Moreira-Soto; Eugenia Corrales-Aguilar; Ignacio Postigo-Hidalgo; Jan Felix Drexler; Teresita Somogyi
EPI_ISL_527739, EPI_ISL_527758	Hospital Mexico [San Jose/San Jose]	Inciensa, Instituto Costarricense de Investigación y Enseñanza en Nutrición y Salud	Adriana Godinez & Melany Calderon; Claudio Soto-Garita; Estela Cordero; Francisco Duarte; Hebleen Porras

EPI_ISL_1406245, EPI_ISL_1406390	Hospital Monte Naranco	Laboratorio de Virología HUCA	Abreu F; Alvarez-Arguelles ME; Boga JA; Castelló C; Costales I; Coto E; Gómez de Oña J; Martín-Rodríguez G; Melón S; Perez-Martínez Z; Rojo S; Sandoval M
EPI_ISL_515541, EPI_ISL_515561	Hospital Montemagno	Instituto Adolfo Lutz, Interdiciplinary Procedures Center, Strategic Laboratory	Claudia Regina Gonçalves; Claudio Tavares Sacchi; Erica Valessa Ramos Gomes
EPI_ISL_1469599	Hospital Montenegro	Epiclin	Ana Paula Muterle; Carolina Comerlato; Eliana Márcia Da Ros Wendland; Fernando Hayashi Sant'Anna; Janira Prichula; Juliana Comerlato
EPI_ISL_534324	Hospital Mun Ver Jose Storopolli	Instituto Adolfo Lutz, Interdiciplinary Procedures Center, Strategic Laboratory	Claudia Regina Gonçalves; Claudio Tavares Sacchi; Erica Valessa Ramos Gomes
EPI_ISL_534318	Hospital Municipal Antonio Giglio	Instituto Adolfo Lutz, Interdiciplinary Procedures Center, Strategic Laboratory	Claudia Regina Gonçalves; Claudio Tavares Sacchi; Erica Valessa Ramos Gomes
EPI_ISL_547571	Hospital Municipal Antônio Giglio	Instituto Adolfo Lutz, Interdiciplinary Procedures Center, Strategic Laboratory	Claudia Regina Gonçalves; Claudio Tavares Sacchi; Erica Valessa Ramos Gomes; Karoline Rodrigues Campos
EPI_ISL_471549, EPI_ISL_523991 to 523992, EPI_ISL_527869	Hospital Municipal Carmen Prudente	Instituto Adolfo Lutz, Interdiciplinary Procedures Center, Strategic Laboratory	Claudia Regina Gonçalves; Claudio Tavares Sacchi; Erica Valessa Ramos Gomes
EPI_ISL_515562	Hospital Municipal Doutor Alexandre Zaio	Instituto Adolfo Lutz, Interdiciplinary Procedures Center, Strategic Laboratory	Claudia Regina Gonçalves; Claudio Tavares Sacchi; Erica Valessa Ramos Gomes
EPI_ISL_468311 to 468312	Hospital Municipal Dr Ignacio Proenca de Gouvea	Instituto Adolfo Lutz, Interdiciplinary Procedures Center, Strategic Laboratory	Claudia Regina Gonçalves; Claudio Tavares Sacchi; Erica Valessa Ramos Gomes
EPI_ISL_1533695	Hospital Municipal Dr Mario Gatti	Instituto Adolfo Lutz, Interdiciplinary Procedures Center, Strategic Laboratory	Caio Vinicius Dias Lopes; Claudia Regina Gonçalves; Claudio Tavares Sacchi; Erica Valessa Ramos Gomes; Karoline Rodrigues Campos; Leonardo Jose Tadeu de Araujo
EPI_ISL_1533708	Hospital Municipal Dr Mario Gatti Campinas	Instituto Adolfo Lutz, Interdiciplinary Procedures Center, Strategic Laboratory	Caio Vinicius Dias Lopes; Claudia Regina Gonçalves; Claudio Tavares Sacchi; Erica Valessa Ramos Gomes; Karoline Rodrigues Campos; Leonardo Jose Tadeu de Araujo
EPI_ISL_515521	Hospital Municipal Dr Waldemar Tebaldi	Instituto Adolfo Lutz, Interdiciplinary Procedures Center, Strategic Laboratory	Claudia Regina Gonçalves; Claudio Tavares Sacchi; Erica Valessa Ramos Gomes
EPI_ISL_523985	Hospital Municipal Dr. Benedicto Montenegro	Instituto Adolfo Lutz, Interdiciplinary Procedures Center, Strategic Laboratory	Claudia Regina Gonçalves; Claudio Tavares Sacchi; Erica Valessa Ramos Gomes
EPI_ISL_515553, EPI_ISL_574577, EPI_ISL_574579	Hospital Municipal Dr. Ignacio Proença de Gouvea	Instituto Adolfo Lutz, Interdiciplinary Procedures Center, Strategic Laboratory	Claudia Regina Gonçalves; Claudio Tavares Sacchi; Erica Valessa Ramos Gomes; Karoline Rodrigues Campos
EPI_ISL_515548, EPI_ISL_515563, EPI_ISL_574582, EPI_ISL_574589	Hospital Municipal Dr. Jose Soares Hungria	Instituto Adolfo Lutz, Interdiciplinary Procedures Center, Strategic Laboratory	Claudia Regina Gonçalves; Claudio Tavares Sacchi; Erica Valessa Ramos Gomes; Karoline Rodrigues Campos
EPI_ISL_515557, EPI_ISL_524467	Hospital Municipal Dr. Moysés Deutsch	Instituto Adolfo Lutz, Interdiciplinary Procedures Center, Strategic Laboratory	Claudia Regina Gonçalves; Claudio Tavares Sacchi; Erica Valessa Ramos Gomes
EPI_ISL_583498	Hospital Municipal Dr. Waldemar Tebaldi	Instituto Adolfo Lutz, Interdiciplinary Procedures Center, Strategic Laboratory	Claudia Regina Gonçalves; Claudio Tavares Sacchi; Erica Valessa Ramos Gomes; Karoline Rodrigues Campos
EPI_ISL_1469754, EPI_ISL_1469778, EPI_ISL_1469811	Hospital Municipal Getúlio Vargas	Epiclin	Ana Paula Muterle; Carolina Comerlato; Eliana Márcia Da Ros Wendland; Fernando Hayashi Sant'Anna; Janira Prichula; Juliana Comerlato
EPI_ISL_527870, EPI_ISL_574578, EPI_ISL_603029, EPI_ISL_603039	Hospital Municipal Mário Gatti	Instituto Adolfo Lutz, Interdiciplinary Procedures Center, Strategic Laboratory	Claudia Regina Gonçalves; Claudio Tavares Sacchi; Erica Valessa Ramos Gomes; Karoline Rodrigues Campos
EPI_ISL_527856	Hospital Municipal Prof. Waldomiro de Paula	Instituto Adolfo Lutz, Interdiciplinary Procedures Center, Strategic Laboratory	Claudia Regina Gonçalves; Claudio Tavares Sacchi; Erica Valessa Ramos Gomes
EPI_ISL_1520110 to 1520112	Hospital Municipal Reynaldo Guerra Cajati	Instituto Adolfo Lutz, Interdiciplinary Procedures Center, Strategic Laboratory	Caio Vinicius Dias Lopes; Claudia Regina Gonçalves; Claudio Tavares Sacchi; Erica Valessa Ramos Gomes; Karoline Rodrigues Campos
EPI_ISL_603028	Hospital Municipal Santa Ana	Instituto Adolfo Lutz, Interdiciplinary Procedures Center, Strategic Laboratory	Claudia Regina Gonçalves; Claudio Tavares Sacchi; Erica Valessa Ramos Gomes; Karoline Rodrigues Campos
EPI_ISL_524468, EPI_ISL_527859	Hospital Municipal Vereador Jose Storopolli	Instituto Adolfo Lutz, Interdiciplinary Procedures Center, Strategic Laboratory	Claudia Regina Gonçalves; Claudio Tavares Sacchi; Erica Valessa Ramos Gomes
EPI_ISL_471647	Hospital Municipal de Barueri Dr. Francisco Moran	Instituto Adolfo Lutz, Interdiciplinary Procedures Center, Strategic Laboratory	Claudia Regina Gonçalves; Claudio Tavares Sacchi; Erica Valessa Ramos Gomes
EPI_ISL_527860	Hospital Municipal de Parelheiros Josanias Castanha Braga	Instituto Adolfo Lutz, Interdiciplinary Procedures Center, Strategic Laboratory	Claudia Regina Gonçalves; Claudio Tavares Sacchi; Erica Valessa Ramos Gomes
EPI_ISL_1533696	Hospital Municipal de Pedreira	Instituto Adolfo Lutz, Interdiciplinary Procedures Center, Strategic Laboratory	Caio Vinicius Dias Lopes; Claudia Regina Gonçalves; Claudio Tavares Sacchi; Erica Valessa Ramos Gomes; Karoline Rodrigues Campos; Leonardo Jose Tadeu de Araujo
EPI_ISL_527862	Hospital Municipal de Urgência	Instituto Adolfo Lutz, Interdiciplinary Procedures Center, Strategic Laboratory	Claudia Regina Gonçalves; Claudio Tavares Sacchi; Erica Valessa Ramos Gomes
EPI_ISL_468308, EPI_ISL_468315, EPI_ISL_515520, EPI_ISL_515526, EPI_ISL_515546, EPI_ISL_515551 to 515552, EPI_ISL_523955, EPI_ISL_523974, EPI_ISL_523976, EPI_ISL_527863			
see above	Hospital Municipal do Tatuape Carmino Caricchio	Instituto Adolfo Lutz, Interdiciplinary Procedures Center, Strategic Laboratory	Claudia Regina Gonçalves; Claudio Tavares Sacchi; Erica Valessa Ramos Gomes
EPI_ISL_491451, EPI_ISL_491453	Hospital México	Inciensa, Instituto Costarricense de Investigación y Enseñanza en Nutrición y Salud	Adriana Godinez & Melany Calderon; Claudio Soto-Garita; Estela Cordero; Francisco Duarte; Hebleen Brenes
EPI_ISL_480322 to 480327	Hospital Nacional de Niños	Charité Virology-University of Costa Rica	Andrei Montero Bonilla; Andres Moreira-Soto; Cristian Pérez Corrales; Eugenia Corrales-Aguilar; Ignacio Postigo-Hidalgo; Jan Felix Drexler
EPI_ISL_1469591, EPI_ISL_1469762, EPI_ISL_1469833, EPI_ISL_1469846, EPI_ISL_1469851	Hospital Nossa Senhora das Graças	Epiclin	Ana Paula Muterle; Carolina Comerlato; Eliana Márcia Da Ros Wendland; Fernando Hayashi Sant'Anna; Janira Prichula; Juliana Comerlato
EPI_ISL_539496	Hospital Nostra Senyora de Meritxell	Instituto de Salud Carlos III	A. Monzón; F. Casas; F. Fernández; I; I. Jiménez; Iglesias-Caballero; M. Camarero; M. Cuesta; M. González-Esguevillas; M. Molinero Calamita; M. Zaballos; P. Jiménez; S. Juliá; S. Pozo; S. Varona
EPI_ISL_1396374 to 1396382	Hospital Notti Mendoza	Nodo de Secuenciación Tierra del Fuego - Hospital Regional Ushuaia - Centro Austral De Investigaciones Científicas - Universidad Nacional De Tierra Del Fuego on behalf of 'Proyecto Argentino Interinstitucional de genomica de SARS-CoV-2' (PAIS Consortium)	Adriana Recabarren; Aguirre Carolina; Alberto Carena; Alejandro Ezequiel Rojas; Andrea Falaschi; Bosio Lia; Carlos Espul; Clara Pott Godoy; Cristina Fernanda Nardi; Daiana Lang; Fernando Gallego; Ivan Dario Gramundi; Maria Belen Peralta Roca; Noelia Cuglia; Patricia Robledo; Sandra Grucci; Santiago Guillermo Ceballos; Vanina Eibar
EPI_ISL_539525	Hospital Nuestra Señora de Sonsoles	Instituto de Salud Carlos III	A. Monzón; A. San Pedro; F. Casas; I; I. Jiménez; Iglesias-Caballero; M. Camarero; M. Cuesta; M. González-Esguevillas; M. Molinero Calamita; M. Zaballos; P. Jiménez; S. Juliá; S. Pozo; S. Varona

EPI_ISL_471269 to 471271	Hospital Oncológico Solca Núcleo de Quito	Institute of Microbiology, Universidad San Francisco de Quito	Belén Prado-Vivar; Bernardo Gutiérrez; Gabriel Trueba; Grace Salazar; Juan José Guadalupe; Marcos Di Stefano; Michelle Grunauer; Patricio Rojas-Silva; Paúl Cárdenas; Sully Márquez; Verónica Barragán
EPI_ISL_1395808 to 1395811	Hospital Pediatrico Dr Avelino Castelan, Hospital 4 de Junio Dr Ramon Carrillo	Grupo de Genómica y Bioinformática del Instituto de Investigación de la Cadena Láctea CONICET-INTA on behalf of 'Proyecto Argentino Interinstitucional de genomica de SARS-CoV-2' (PAIS Consortium)	AF; Amadio; Andrea Piedra Buena; Antonieta Cayré; Eberhardt; Gabriela Jurasek; Gili Andrea; Irazoqui; Laura Lescano; Lopez María Cecilia; MF; Marino Goia; Mayra Mendoza
EPI_ISL_419386 to 419387	Hospital Prof. Doutor Fernando Fonseca, EPE	Instituto Gulbenkian de Ciência	Cathy Paulino; Joao Sobral; João Costa; Ricardo Leite; Susana Ladeiro
EPI_ISL_1405917	Hospital Queen Elizabeth	Institute for Medical Research, Infectious Disease Research Centre, National Institutes of Health, Ministry of Health Malaysia	Azizan MA; Kamel K; Suppiah J; Thayan R
EPI_ISL_1469697, EPI_ISL_1469799, EPI_ISL_1469824, EPI_ISL_1479127	Hospital Regina	Epiclin	Ana Paula Muterle; Carolina Comerlato; Eliana Márcia Da Ros Wendland; Fernando Hayashi Sant'Anna; Janira Prichula; Juliana Comerlato
EPI_ISL_1502818 to 1502820, EPI_ISL_1502824 to 1502825, EPI_ISL_1502835 to 1502836, EPI_ISL_1502867, EPI_ISL_1503123 to 1503129	Hospital Regional Anita Moreno Los Santos	Gorgas Memorial Laboratory of Health Studies	Best Dayana; Castillo Jorge; Espino Juliee; Franco Danilo; Gonzalez Claudia; Jessica Gondola; Leyda Abrego; Lopez-Verges Sandra; Marlenne Castillo; Martinez Alexander; Moreno Ambar; Moreno Brechla; Oris Chavarria; Ortiz Alma
EPI_ISL_1502821 to 1502823, EPI_ISL_1502826 to 1502834, EPI_ISL_1502837 to 1502841, EPI_ISL_1502861, EPI_ISL_1502864 to 1502865, EPI_ISL_1502868	Hospital Regional Dr. Luis "Chicho" Fábrega	Gorgas Memorial Laboratory of Health Studies	Castillo Jorge; Franco Danilo; Gonzalez Claudia; Herrera Genarino; Herrera Melissa; Jessica Gondola; Leyda Abrego; Lopez-Verges Sandra; Marlenne Castillo; Martinez Alexander; Moreno Ambar; Moreno Brechla; Oris Chavarria; Ortiz Alma
EPI_ISL_527857	Hospital Regional Vale do Ribeira	Instituto Adolfo Lutz, Interdiciplinary Procedures Center, Strategic Laboratory	Claudia Regina Gonçalves; Claudio Tavares Sacchi; Erica Valessa Ramos Gomes
EPI_ISL_523956	Hospital Regional de Assis	Instituto Adolfo Lutz, Interdiciplinary Procedures Center, Strategic Laboratory	Claudia Regina Gonçalves; Claudio Tavares Sacchi; Erica Valessa Ramos Gomes
EPI_ISL_524463	Hospital Regional de Cotia	Instituto Adolfo Lutz, Interdiciplinary Procedures Center, Strategic Laboratory	Claudia Regina Gonçalves; Claudio Tavares Sacchi; Erica Valessa Ramos Gomes
EPI_ISL_491450, EPI_ISL_491454, EPI_ISL_491456	Hospital San Juan de Dios	Inciensa, Instituto Costarricense de Investigación y Enseñanza en Nutrición y Salud	Adriana Godinez & Melany Calderon; Claudio Soto-Garita; Estela Cordero; Francisco Duarte; Hebleen Brenes
EPI_ISL_419234, EPI_ISL_455336 to 455343, EPI_ISL_578196 to 578201	Hospital San Pedro	Instituto de Salud Carlos III	A. Monzón; Alonso, C.; C. Alonso; Camarero, S.; Casas, I.; Cuesta, I.; F. Casas; González-Esguevillas, M.; I; I. Jiménez; Iglesias-Caballero; Iglesias-Caballero, M.; J.M Azcona; Jiménez, M.; Jiménez, P.; Juliá, M.; M. Blasco; M. Camarero; M. Cuesta; M. González-Esguevillas; M. Molinero Calamita; M. Zaballos; Molinero Calamita, M.; Monzón, S.; P. Jiménez; Pozo, F.; S. Juliá; S. Pozo; S. Varona; Varona, S.; Zaballos, A.
EPI_ISL_539558, EPI_ISL_539561 to 539562	Hospital San Pedro de Alcántara	Instituto de Salud Carlos III	A. Monzón; E. Cerro; F. Casas; I; I. Jiménez; Iglesias-Caballero; J. López; M. Camarero; M. Cuesta; M. González-Esguevillas; M. Molinero Calamita; M. Zaballos; M.A Cañizares; P. Jiménez; S. Juliá; S. Pozo; S. Varona
EPI_ISL_510305 to 510332, EPI_ISL_613410 to 613411, EPI_ISL_1423681 to 1423695	Hospital San Pedro de Alcántara (Cáceres)	SeqCOVID-SPAIN consortium/IBV(CSIC)	Cristina Muñoz Cuevas; Guadalupe Rodriguez Rodriguez and SeqCOVID-SPAIN consortium; Guadalupe Rodríguez Rodríguez and SeqCOVID-SPAIN consortium
EPI_ISL_491438, EPI_ISL_491440, EPI_ISL_491448, EPI_ISL_491452	Hospital San Rafael de Alajuela	Inciensa, Instituto Costarricense de Investigación y Enseñanza en Nutrición y Salud	Adriana Godinez & Melany Calderon; Claudio Soto-Garita; Estela Cordero; Francisco Duarte; Hebleen Brenes
EPI_ISL_527753 to 527754	Hospital San Vicente De Paul	Inciensa, Instituto Costarricense de Investigación y Enseñanza en Nutrición y Salud	Adriana Godinez & Melany Calderon; Claudio Soto-Garita; Estela Cordero; Francisco Duarte; Hebleen Porras
EPI_ISL_434536	Hospital San Vicente de Paul	Inciensa, Instituto Costarricense de Investigación y Enseñanza en Nutrición y Salud	Adriana Godinez & Melany Calderon; Claudio Soto-Garita; Estela Cordero; Francisco Duarte; Hebleen Porras
EPI_ISL_471552, EPI_ISL_523967	Hospital Sancta Maggiore	Instituto Adolfo Lutz, Interdiciplinary Procedures Center, Strategic Laboratory	Claudia Regina Gonçalves; Claudio Tavares Sacchi; Erica Valessa Ramos Gomes
EPI_ISL_603036	Hospital Santa Ana	Instituto Adolfo Lutz, Interdiciplinary Procedures Center, Strategic Laboratory	Claudia Regina Gonçalves; Claudio Tavares Sacchi; Erica Valessa Ramos Gomes; Karoline Rodrigues Campos
EPI_ISL_515527, EPI_ISL_1533711	Hospital Santa Clara	Instituto Adolfo Lutz, Interdiciplinary Procedures Center, Strategic Laboratory	Caio Vinicius Dias Lopes; Claudia Regina Gonçalves; Claudio Tavares Sacchi; Erica Valessa Ramos Gomes; Karoline Rodrigues Campos; Leonardo Jose Tadeu de Araujo
EPI_ISL_1520113	Hospital Santo Antonio de Juquia Juquia	Instituto Adolfo Lutz, Interdiciplinary Procedures Center, Strategic Laboratory	Caio Vinicius Dias Lopes; Claudia Regina Gonçalves; Claudio Tavares Sacchi; Erica Valessa Ramos Gomes; Karoline Rodrigues Campos
EPI_ISL_1493586, EPI_ISL_1493590	Hospital Sao Marcos da Samamorro Agudo	Instituto Adolfo Lutz, Interdiciplinary Procedures Center, Strategic Laboratory	Caio Vinicius Dias Lopes; Claudia Regina Gonçalves; Claudio Tavares Sacchi; Erica Valessa Ramos Gomes; Karoline Rodrigues Campos
EPI_ISL_468310	Hospital Sao Paulo de Ensino da UNIFESP	Instituto Adolfo Lutz, Interdiciplinary Procedures Center, Strategic Laboratory	Claudia Regina Gonçalves; Claudio Tavares Sacchi; Erica Valessa Ramos Gomes
EPI_ISL_471545, EPI_ISL_471551, EPI_ISL_515528, EPI_ISL_515545, EPI_ISL_515559 to 515560, EPI_ISL_523969, EPI_ISL_523981, EPI_ISL_523988			
see above	Hospital Sao Paulo de Ensino da Unifesp	Instituto Adolfo Lutz, Interdiciplinary Procedures Center, Strategic Laboratory	Claudia Regina Gonçalves; Claudio Tavares Sacchi; Erica Valessa Ramos Gomes
EPI_ISL_1469587, EPI_ISL_1469593, EPI_ISL_1469701, EPI_ISL_1469801, EPI_ISL_1469820, EPI_ISL_1469840, EPI_ISL_1469843			
see above	Hospital Sapiranga	Epiclin	Ana Paula Muterle; Carolina Comerlato; Eliana Márcia Da Ros Wendland; Fernando Hayashi Sant'Anna; Janira Prichula; Juliana Comerlato
EPI_ISL_524484	Hospital Schwyz	Institute of Medical Virology, University of Zurich	Alexandra Trkola; Andrea Zbinden; Fiona Steiner; Gabriela Ziltener; Jon Huder; Jürg Böni; Maryam Zaheri; Michael Huber; Patrick Redli; Riccarda Capaul; Stefan Schmutz; Verena Kufner
EPI_ISL_1482627, EPI_ISL_1482629 to 1482631, EPI_ISL_1491363 to 1491365	Hospital Sharp	Microbial Genomics Laboratory	Alejandra Garcia-Gasca; Bruno Gomez-Gil; Daniel Fregoso-Rueda; Julissa Enciso-Ibarra
EPI_ISL_1469670, EPI_ISL_1469717, EPI_ISL_1469739, EPI_ISL_1469750, EPI_ISL_1469755, EPI_ISL_1469789, EPI_ISL_1469807, EPI_ISL_1469818, EPI_ISL_1469822, EPI_ISL_1469830, EPI_ISL_1469842			
see above	Hospital São Francisco de Assis	Epiclin	Ana Paula Muterle; Carolina Comerlato; Eliana Márcia Da Ros Wendland; Fernando Hayashi Sant'Anna; Janira Prichula; Juliana Comerlato
EPI_ISL_414015 to 414017	Hospital São Joaquim Beneficencia Portuguesa	Instituto Adolfo Lutz, Interdiciplinary Procedures Center, Strategic Laboratory	Audrey Cilli; Carlos Henrique Camargo; Claudia Regina Gonçalves; Claudio Tavares Sacchi; Daniela Bernardes Borges da Silva; Ester Cerdeira Sabino; Fabiana Cristina Pereira dos Santos; Fabiana Cristina Pereira dos Santos Terezinha Maria de Paiva; Maria do Carmo Sampaito Tavares Timenetsky; SimoneGuadagnucci Morillo; Terezinha Maria de Paiva
EPI_ISL_455350 to 455351	Hospital Txagorritxu	Instituto de Salud Carlos III	A. Monzón; C. Gómez; F. Casas; I; I. Jiménez; Iglesias-Caballero; M. Camarero; M. Cuesta; M. González-Esguevillas; M. Molinero Calamita; M. Zaballos; P. Jiménez; S. Juliá; S. Pozo; S. Varona
EPI_ISL_1469668	Hospital Unimed Vale do Caí	Epiclin	Ana Paula Muterle; Carolina Comerlato; Eliana Márcia Da Ros Wendland; Fernando Hayashi Sant'Anna; Janira Prichula; Juliana Comerlato
EPI_ISL_1394460 to 1394499	Hospital Universitari Arnau de Vilanova	Hospital Universitari Vall d'Hebron - Vall d'Hebron Institut de	Andrés Antón; Ariadna Rando; Carla Castillo; Cristina Andrés; Damir Garcia-Cehic; Josep F Abril; Josep Quer; Juliana Esperalba; Maria Carmen Martin;

		Recerca	Maria Gema Codina; Maria Piñana; Tomàs Pumarola
EPI_ISL_1423903 to 1423918	Hospital Universitari Arnau de Vilanova de Lleida	SeqCOVID-SPAIN consortium/IBV(CSIC)	Alba Bellés Bellés; Albert Bernet Sánchez; Eric López González; Iván Prats Sánchez; Mercè García González and SeqCOVID-SPAIN consortium
EPI_ISL_483059	Hospital Universitari Germans Trias i Pujol	IrsiCaixa AIDS Research Lab	A. Valencia; B. Clotet; C. Avila-Nieto; E. Vidal; G. Cantero; I. Blanco; J. Blanco; J. Carrillo; J. Rodon; J. Segalés; J. Vergara-Alert; M. Noguera-Julián; M. Parera; M. Puig; M.T. Terrón; N. Izquierdo-Useros; S. Cruz; V. Guallar
EPI_ISL_535575, EPI_ISL_538552	Hospital Universitari Germans Trias i Pujol(HUGTiP)/Fundació Lluita contra la SIDA (FLSida)	IrsiCaixa AIDS Research Lab	Bonaventura Clotet; Ester Ballana; Joaquim Segalés; Jorge Carrillo; Julia Blanco; Lidia Ruiz; Marc Noguera-Julian; Maria Pilar Armengol; Mariona Parera; Marta Massanella; Nuria Izquierdo; Roger Paredes
EPI_ISL_418268, EPI_ISL_471472, EPI_ISL_510689	Hospital Universitari Germans Trias i Pujol(HUGTiP)/Fundació Lluita contra la SIDA (FLSida)/IRTA-CReSA	IrsiCaixa AIDS Research Lab	Albert Bensaid; Bonaventura Clotet; Joaquim Segalés; Jordi Rodón; Jorge Carrillo; Julia Blanco; Julia Vergara; Lidia Ruiz; Marc Noguera-Julian; Nuria Izquierdo; Pilar Armengol; Roger Paredes
EPI_ISL_516193	Hospital Universitari Germans Trias i Pujol.	IrsiCaixa AIDS Research Lab	Bonaventura Clotet; Ester Ballana; Joaquim Segalés; Jorge Carrillo; Julia Blanco; Lidia Ruiz; Marc Noguera-Julian; Maria Pilar Armengol; Mariona Parera; Marta Massanella; Nuria Izquierdo; Roger Paredes
EPI_ISL_1394500 to 1394505	Hospital Universitari Joan XXIII de Tarragona	Hospital Universitari Vall d'Hebron - Vall d'Hebron Institut de Recerca	Andrés Antón; Ariadna Rando; Carla Castillo; Cristina Andrés; Damir Garcia-Cehic; Josep F Abril; Josep Quer; Juliana Esperalba; Maria Carmen Martin; Maria Gema Codina; Maria Piñana; Tomàs Pumarola
EPI_ISL_418860 to 418861, EPI_ISL_444971 to 444990, EPI_ISL_447532 to 447533, EPI_ISL_450337, EPI_ISL_458130 to 458132, EPI_ISL_1391098 to 1391202, EPI_ISL_1394415 to 1394459			
see above	Hospital Universitari Vall d'Hebron - Vall d'Hebron Institut de Recerca	Hospital Universitari Vall d'Hebron - Vall d'Hebron Institut de Recerca	Andrés Antón; Ariadna Rando; Carla Castillo; Cristina Andrés; DAmir Garcia-Cehic; Damir Garcia-Cehic; Dàmir Garcia-Cehic; Josep F Abril; Josep Gregori; Josep Quer; Juliana Esperalba; Maria Carmen Martin; Maria Carmen Martín; Maria Gema Codina; Maria Piñana; Mercedes Guerrero-Murillo; Tomàs Pumarola
EPI_ISL_538003 to 538054, EPI_ISL_539245	Hospital Universitari i Politècnic La Fe de València	SeqCOVID-SPAIN consortium/IBV(CSIC)	Ana Gil Brusola; Eva González Barbera; José Luis López Hontangas and SeqCOVID-SPAIN consortium; María Dolores Gómez Ruiz; Salvador Giner Almaraz
EPI_ISL_421171 to 421180, EPI_ISL_428683 to 428711, EPI_ISL_430718, EPI_ISL_529972 to 530024, EPI_ISL_534327 to 534329	Hospital Universitario 12 de Octubre	Hospital Universitario 12 de Octubre	Elias Dahdouh; Esther Viedma; Fernando Lázaro; Jesús Mingorance; Juan Carlos Galán; Julio García; Ma Dolores Folgueira; Mª Dolores Folgueira; Natalia Stella; Rafael Cantón; Rafael Delgado; Raúl Recio; Sara González
EPI_ISL_417952, EPI_ISL_417954, EPI_ISL_417956 to 417957, EPI_ISL_417961, EPI_ISL_417963, EPI_ISL_417967, EPI_ISL_418182			
see above	Hospital Universitario 12 de Octubre	Hospital Universitario La Paz	Elias Dahdouh; Esther Viedma; Fernando Lázaro; Jesús Mingorance; Juan Carlos Galán; Julio García; Mª Dolores Folgueira; Natalia Stella; Rafael Cantón; Rafael Delgado; Sara González
EPI_ISL_467092 to 467183, EPI_ISL_500228 to 500286, EPI_ISL_509616 to 509618, EPI_ISL_537874 to 537945, EPI_ISL_538118, EPI_ISL_538231 to 538232	Hospital Universitario Araba. Vitoria-Gasteiz	SeqCOVID-SPAIN consortium/IBV(CSIC)	Amaia Aguirre Quiñonero; Andrés Canut Blasco and SeqCOVID-SPAIN consortium; Andrés Canut Blasco. and SeqCOVID-SPAIN consortium; Carmen Gómez González; Ma Concepción Lecaroz Agara; Ma Rosario Almela Ferrer; Marina Fernández Torres; Mª Concepción Lecaroz Agara; Mª Rosario Almela Ferrer; Silvia Hernáez Crespo
EPI_ISL_452692 to 452786	Hospital Universitario Araba. Vitoria-Gasteiz,	SeqCOVID-SPAIN consortium/IBV(CSIC)	Amaia Aguirre Quiñonero; Andrés Canut Blasco and SeqCOVID-SPAIN consortium; Carmen Gómez González; Maria Concepción Lecaroz Agara; Maria Rosario Almela Ferrer; Marina Fernández Torres; Silvia Hernáez Crespo
EPI_ISL_1538412	Hospital Universitario Basurto Clinical Microbiology Service	Biocruces Bizkaia	Ana Belen de la Hoz; Estibaliza Ugalde Zarraga; Jose Luis Díaz de Tuesta del Arco; Mikel Gallego Rodrigo; Mikel Joseba Urrutikoetxea-Gutierrez; Mª Carmen Nieto Toboso
EPI_ISL_455326	Hospital Universitario Insular de Gran Canaria	Instituto de Salud Carlos III	A. Hernández; A. Monzón; F. Casas; I; I. Jiménez; Iglesias-Caballero; M. Camarero; M. Cuesta; M. González-Esguevillas; M. Molinero Calamita; M. Zaballos; P. Jiménez; S. Juliá; S. Pozo; S. Varona
EPI_ISL_428674 to 428682, EPI_ISL_430719 to 430721, EPI_ISL_435144	Hospital Universitario La Paz	Hospital Universitario 12 de Octubre	Elias Dahdouh; Esther Viedma; Fernando Lázaro; Jesús Mingorance; Juan Carlos Galán; Julio García; Mª Dolores Folgueira; Natalia Stella; Rafael Cantón; Rafael Delgado; Raúl Recio; Sara González
EPI_ISL_417969, EPI_ISL_417972, EPI_ISL_417975, EPI_ISL_417978, EPI_ISL_530025 to 530093, EPI_ISL_534330 to 534333	Hospital Universitario La Paz	Hospital Universitario La Paz	Elias Dahdouh; Esther Viedma; Fernando Lázaro; Jesús Mingorance; Juan Carlos Galán; Julio García; Ma Dolores Folgueira; María Rodríguez; Mª Dolores Folgueira; Natalia Stella; Rafael Cantón; Rafael Delgado; Raúl Recio; Sara González
EPI_ISL_537809 to 537810	Hospital Universitario Marqués de Valdecilla (Santander), Servicio de Microbiología	SeqCOVID-SPAIN consortium/IBV(CSIC)	Daniel Pablo Marcos; Jesús Rodríguez Lozano; Jose Manuel Méndez Legaza; María Eliecer Cano García; María Siller Ruiz and SeqCOVID-SPAIN consortium; Mónica Gozalo Margüello
EPI_ISL_538690 to 538747, EPI_ISL_582091 to 582109, EPI_ISL_1423593 to 1423632, EPI_ISL_1423816 to 1423848	Hospital Universitario Marqués de Valdecilla - IDIVAL (Santander, Cantabria)	SeqCOVID-SPAIN consortium/IBV(CSIC)	Daniel Pablo Marcos; Jesús Rodríguez Rodríguez; Jose Manuel Méndez Legaza; María Eliecer Cano García; María Siller Ruiz and SeqCOVID-SPAIN consortium; Mónica Gozalo Margüello
EPI_ISL_539503 to 539508, EPI_ISL_539513 to 539519, EPI_ISL_578189, EPI_ISL_578192 to 578193	Hospital Universitario Miguel Servet	Instituto de Salud Carlos III	A. Monzón; A. Rezusta; F. Casas; I; I. Jiménez; Iglesias-Caballero; M. Camarero; M. Cuesta; M. González-Esguevillas; M. Molinero Calamita; M. Zaballos; P. Jiménez; R. Benito; S. Juliá; S. Pozo; S. Varona
EPI_ISL_452453 to 452471, EPI_ISL_467064 to 467085	Hospital Universitario Puerta del Mar de Cádiz - INIBICA	SeqCOVID-SPAIN consortium/IBV(CSIC)	Fátima-Galán-Sánchez; Manuel Rodríguez-Iglesias and SeqCOVID-SPAIN consortium; Manuel Rodrí-guez-Iglesias and SeqCOVID-SPAIN consortium; Salud Rodríguez-Pallares
EPI_ISL_530094 to 530099	Hospital Universitario Ramón y Cajal	Hospital Universitario 12 de Octubre	Elias Dahdouh; Esther Viedma; Fernando Lázaro; Jesús Mingorance; Juan Carlos Galán; Julio García; Mª Dolores Folgueira; Natalia Stella; Rafael Cantón; Rafael Delgado; Raúl Recio; Sara González
EPI_ISL_417979 to 417981, EPI_ISL_530100 to 530119, EPI_ISL_534334	Hospital Universitario Ramón y Cajal	Hospital Universitario La Paz	Elias Dahdouh; Esther Viedma; Fernando Lázaro; Jesús Mingorance; Juan Carlos Galán; Julio García; Ma Dolores Folgueira; María Rodríguez; Mª Dolores Folgueira; Natalia Stella; Rafael Cantón; Rafael Delgado; Raúl Recio; Sara González
EPI_ISL_419230	Hospital Universitario Virgen de las Nieves	Instituto de Salud Carlos III	Camarero, S.; Casas, I.; Cuesta, I.; González-Esguevillas, M.; Iglesias-Caballero, M.; Jiménez, M.; Jiménez, P.; Juliá, M.; Molinero Calamita, M.; Monzón, S.; Pozo, F.; Sanbonmatsu, S.; Varona, S.; Zaballos, A.
EPI_ISL_467059 to 467063, EPI_ISL_474797, EPI_ISL_474832 to 474836, EPI_ISL_474841 to 474846, EPI_ISL_474850 to 474852, EPI_ISL_474854 to 474900, EPI_ISL_474907 to 474909, EPI_ISL_474920, EPI_ISL_474922 to 474932, EPI_ISL_474934 to 474939, EPI_ISL_474942 to 474944, EPI_ISL_474948 to 474950, EPI_ISL_474957, EPI_ISL_500160 to 500161, EPI_ISL_500163 to 500165, EPI_ISL_500168 to 500206, EPI_ISL_500208, EPI_ISL_500210 to 500217, EPI_ISL_500220, EPI_ISL_500222 to 500227, EPI_ISL_510424 to 510460, EPI_ISL_538672 to 538689, EPI_ISL_1423926 to 1423933			
see above	Hospital Universitario Virgen de las Nieves de Granada-SAS	SeqCOVID-SPAIN consortium/IBV(CSIC)	Irene Pedrosa Corral; José M. Navarro-Marí and SeqCOVID-SPAIN consortium; Mercedes Pérez Ruiz; Sara Sanbonmatsu Gámez
EPI_ISL_468318, EPI_ISL_468321, EPI_ISL_547574	Hospital Universitario da USP	Instituto Adolfo Lutz, Interdiciplinary Procedures Center, Strategic Laboratory	Claudia Regina Gonçalves; Claudio Tavares Sacchi; Erica Valessa Ramos Gomes; Karoline Rodrigues Campos
EPI_ISL_471539, EPI_ISL_1533702, EPI_ISL_1533712, EPI_ISL_1533727	Hospital Universitario da USP Sao Paulo	Instituto Adolfo Lutz, Interdiciplinary Procedures Center, Strategic Laboratory	Caio Vinicius Dias Lopes; Claudia Regina Gonçalves; Claudio Tavares Sacchi; Erica Valessa Ramos Gomes; Karoline Rodrigues Campos; Leonardo Jose Tadeu de Araujo
EPI_ISL_534314	Hospital Universitario da USP de SP	Instituto Adolfo Lutz, Interdiciplinary Procedures Center, Strategic Laboratory	Claudia Regina Gonçalves; Claudio Tavares Sacchi; Erica Valessa Ramos Gomes
EPI_ISL_419233, EPI_ISL_455325, EPI_ISL_539532	Hospital Universitario de Canarias	Instituto de Salud Carlos III	A. Monzón; B. Castro; Camarero, S.; Casas, I.; Castro, B.; Cuesta, I.; F. Casas; González-Esguevillas, M.; I; I. Jiménez; Iglesias-Caballero; Iglesias-Caballero, M.; Jiménez, M.; Jiménez, P.; Juliá, M.; M. Camarero; M. Cuesta; M. González-Esguevillas; M. Molinero Calamita; M. Zaballos; Molinero Calamita, M.; Monzón, S.; P. Jiménez; Pozo, F.; S. Juliá; S. Pozo; S. Varona; Varona, S.; Zaballos, A.
EPI_ISL_539520 to 539522	Hospital Universitario de Ceuta	Instituto de Salud Carlos III	A. Monzón; F. Casas; G. Sánchez; I; I. Jiménez; Iglesias-Caballero; J. López; M. Camarero; M. Cuesta; M. González-Esguevillas; M. Molinero Calamita; M. Zaballos; P. Jiménez; S. Juliá; S. Pozo; S. Varona
EPI_ISL_467086 to 467091, EPI_ISL_474910 to	Hospital Universitario de Gran Canaria Dr. Negrín	SeqCOVID-SPAIN consortium/IBV(CSIC)	Ana Bordes Benítez and SeqCOVID-SPAIN consortium; Francisco J. Chamizo López; M. Carmen Pérez González

474918, EPI_ISL_537691 to 537728, EPI_ISL_537788 to 537808, EPI_ISL_561370 to 561372, EPI_ISL_1470297 to 1470374			
EPI_ISL_539246 to 539283	Hospital Universitario de La Ribera (Alzira, València)	SeqCOVID-SPAIN consortium/IBV(CSIC)	Julia González and SeqCOVID-SPAIN consortium; Olalla Martínez Macias
EPI_ISL_1469570, EPI_ISL_1469773, EPI_ISL_1469815	Hospital Universitário	Epiclin	Ana Paula Muterle; Carolina Comerlato; Eliana Márcia Da Ros Wendland; Fernando Hayashi Sant'Anna; Janira Prichula; Juliana Comerlato
EPI_ISL_1469774, EPI_ISL_1469793, EPI_ISL_1469844, EPI_ISL_1469849	Hospital Universitário de Canoas	Epiclin	Ana Paula Muterle; Carolina Comerlato; Eliana Márcia Da Ros Wendland; Fernando Hayashi Sant'Anna; Janira Prichula; Juliana Comerlato
EPI_ISL_1399257	Hospital Valle del Nalon	Laboratorio de Virología HUCA	Abreu F; Alvarez-Arguelles ME; Boga JA; Castelló C; Costales I; Coto E; Gómez de Oña J; Martín-Rodríguez G; Melón S; Perez-Martínez Z; Rojo S; Sandoval M
EPI_ISL_1399248, EPI_ISL_1406198, EPI_ISL_1406407	Hospital Valle del Nalón	Laboratorio de Virología HUCA	Abreu F; Alvarez-Arguelles ME; Boga JA; Castelló C; Costales I; Coto E; Gómez de Oña J; Martín-Rodríguez G; Melón S; Perez-Martínez Z; Rojo S; Sandoval M
EPI_ISL_455315 to 455322, EPI_ISL_455324, EPI_ISL_539497 to 539498, EPI_ISL_578187 to 578188	Hospital Virgen de las Nieves	Instituto de Salud Carlos III	A. Monzón; F. Casas; I; I. Jiménez; Iglesias-Caballero; J. Lepe; J.M Navarro; M. Camarero; M. Cuesta; M. González-Esguevillas; M. Molinero Calamita; M. Zaballos; P. Jiménez; S. Juliá; S. Pozo; S. Sanbonmatsu; S. Varona
EPI_ISL_455314, EPI_ISL_455323	Hospital Virgen del Rocio	Instituto de Salud Carlos III	A. Monzón; F. Casas; I; I. Jiménez; Iglesias-Caballero; J. Lepe; M. Camarero; M. Cuesta; M. González-Esguevillas; M. Molinero Calamita; M. Zaballos; P. Jiménez; S. Juliá; S. Pozo; S. Varona
EPI_ISL_476203 to 476208, EPI_ISL_476217, EPI_ISL_476222 to 476223, EPI_ISL_476237 to 476277, EPI_ISL_476372 to 476386, EPI_ISL_476428 to 476490	Hospital da Clínicas da Faculdade de Medicina da Universidade de São Paulo	Instituto de Medicina Tropical da Univesidade de São Paulo	Camila Alves Maia da Silva; Carolina S. Lazar; Cecilia Salete Alencar; Darlan da Silva Candido; Erika Regina Manuli; Ester Sabino; Flavia Cristina da Silva Sales; Giula Magalhaes Ferreira; Jaqueline Goes de Jesus; Julien Theze; Mariana Severo Ramundo; Nuno Faria; Samples: Ingra Morales Claro; Sequencing: Ingra Morales Claro; Silvia F. Costa; Thais de Moura Coletti
EPI_ISL_534313	Hospital da Sta Casa de Sto Amaro	Instituto Adolfo Lutz, Interdiciplinary Procedures Center, Strategic Laboratory	Claudia Regina Gonçalves; Claudio Tavares Sacchi; Erica Valessa Ramos Gomes
EPI_ISL_1533699	Hospital das Clinicas Luzia de Pinho Melo	Instituto Adolfo Lutz, Interdiciplinary Procedures Center, Strategic Laboratory	Caio Vinicius Dias Lopes; Claudia Regina Gonçalves; Claudio Tavares Sacchi; Erica Valessa Ramos Gomes; Karoline Rodrigues Campos; Leonardo Jose Tadeu de Araujo
EPI_ISL_511577 to 511578	Hospital de Braga	Instituto Nacional de Saude (INSA)	Borges et al
EPI_ISL_1406278 to 1406279, EPI_ISL_1406432, EPI_ISL_1406463	Hospital de Cabueñes	Laboratorio de Virología HUCA	Abreu F; Alvarez-Arguelles ME; Boga JA; Castelló C; Costales I; Coto E; Gómez de Oña J; Martín-Rodríguez G; Melón S; Perez-Martínez Z; Rojo S; Sandoval M
EPI_ISL_455352 to 455354	Hospital de Cruces	Instituto de Salud Carlos III	A. Monzón; F. Casas; I; I. Jiménez; Iglesias-Caballero; M. Aranzamendi; M. Camarero; M. Cuesta; M. González-Esguevillas; M. Molinero Calamita; M. Zaballos; P. Jiménez; S. Juliá; S. Pozo; S. Varona
EPI_ISL_476563, EPI_ISL_476565, EPI_ISL_476567 to 476568, EPI_ISL_476571, EPI_ISL_476573, EPI_ISL_615121	Hospital de Pediatría "Prof. Dr. Juan P Garrahan"	Héritas	Andrea Mangano; Bianca Brun; Cristian Rohr; Dalmacio Pereyra; Fabian Fay; Maria Florencia Fernandez; Martin Vazquez; Mauricio Grisolia; Priscila Aldabe; Roberta Crespo
EPI_ISL_1469600, EPI_ISL_1469771, EPI_ISL_1469827	Hospital de Portão	Epiclin	Ana Paula Muterle; Carolina Comerlato; Eliana Márcia Da Ros Wendland; Fernando Hayashi Sant'Anna; Janira Prichula; Juliana Comerlato
EPI_ISL_414577 to 414578	Hospital de Talca, Chile	Instituto de Salud Publica de Chile	Alejandra Acevedo; Andrés E. Castillo; Bárbara Parra; Carolina Tambley; Gabriel Leal; Gisselle Barra; Jaime Lagos; Javier Tognarelli; Jorge Fernández.; Loredana Arata; Patricia Bustos; Paz Tapia; Rodrigo Fasce; Soledad Ulloa; Winston Andrade
EPI_ISL_510246 to 510267	Hospital de la Santa Creu i Sant Pau. Servicio de Microbiología	SeqCOVID-SPAIN consortium/IBV(CSIC)	Elisenda Miró and SeqCOVID-SPAIN consortium; Ferran Navarro; Núria Rabella
EPI_ISL_541067 to 541081	Hospital de la Santa Creu i Sant Pau. Servicio de Microbiología	SeqCOVID-SPAIN consortium/Institute of Biomedicine of Valencia, IBV-CSIC	Elisenda Miró and SeqCOVID-SPAIN consortium; Ferran Navarro; Núria Rabella
EPI_ISL_1406283 to 1406285, EPI_ISL_1406411, EPI_ISL_1406431	Hospital del Oriente de Asturias	Laboratorio de Virología HUCA	Abreu F; Alvarez-Arguelles ME; Boga JA; Castelló C; Costales I; Coto E; Gómez de Oña J; Martín-Rodríguez G; Melón S; Perez-Martínez Z; Rojo S; Sandoval M
EPI_ISL_1399247	Hospital del Valle del Nalón	Laboratorio de Virología HUCA	Abreu F; Alvarez-Arguelles ME; Boga JA; Castelló C; Costales I; Coto E; Gómez de Oña J; Martín-Rodríguez G; Melón S; Perez-Martínez Z; Rojo S; Sandoval M
EPI_ISL_534319 to 534320	Hospital do Serv Pub ESTAFCO Morato de Oliveira	Instituto Adolfo Lutz, Interdiciplinary Procedures Center, Strategic Laboratory	Claudia Regina Gonçalves; Claudio Tavares Sacchi; Erica Valessa Ramos Gomes
EPI_ISL_471548, EPI_ISL_515565, EPI_ISL_523965, EPI_ISL_523972, EPI_ISL_523978, EPI_ISL_523982, EPI_ISL_524470			
see above	Hospital do Servidor Público Estadual Francisco Morato de Oliveira	Instituto Adolfo Lutz, Interdiciplinary Procedures Center, Strategic Laboratory	Claudia Regina Gonçalves; Claudio Tavares Sacchi; Erica Valessa Ramos Gomes
EPI_ISL_527861	Hospital e Maternidade Celso Pierro	Instituto Adolfo Lutz, Interdiciplinary Procedures Center, Strategic Laboratory	01246-1301; 355 - Brazil; Av. Dr. Arnaldo; Cerqueira Cesar; São Paulo - SP
EPI_ISL_547577	Hospital e Maternidade Nossa Senhora das Graças	Instituto Adolfo Lutz, Interdiciplinary Procedures Center, Strategic Laboratory	Claudia Regina Gonçalves; Claudio Tavares Sacchi; Erica Valessa Ramos Gomes; Karoline Rodrigues Campos
EPI_ISL_527865	Hospital e Maternidade São Cristóvão	Instituto Adolfo Lutz, Interdiciplinary Procedures Center, Strategic Laboratory	Claudia Regina Gonçalves; Claudio Tavares Sacchi; Erica Valessa Ramos Gomes
EPI_ISL_527868	Hospital e Maternidade do Braz	Instituto Adolfo Lutz, Interdiciplinary Procedures Center, Strategic Laboratory	Claudia Regina Gonçalves; Claudio Tavares Sacchi; Erica Valessa Ramos Gomes
EPI_ISL_534323	Hospital e Pronto Socorro Comunitario Vila Yolanda	Instituto Adolfo Lutz, Interdiciplinary Procedures Center, Strategic Laboratory	Claudia Regina Gonçalves; Claudio Tavares Sacchi; Erica Valessa Ramos Gomes
EPI_ISL_527864	Hospital e Pronto Socorro Comunitário Vila Iolanda	Instituto Adolfo Lutz, Interdiciplinary Procedures Center, Strategic Laboratory	Claudia Regina Gonçalves; Claudio Tavares Sacchi; Erica Valessa Ramos Gomes
EPI_ISL_1394290 to 1394337	Hospital for Infectious Diseases, Molecular Diagnostics Laboratory, Warsaw, Poland	1. Virogenetics Laboratory of Virology, Maopolska Centre of Biotechnology, Jagiellonian University. 2. Intercollegiate Faculty of Biotechnology University of Gdansk and Medical University of Gdansk	Andrzej Horban; Aneta Kopacz; Karolina Gackowska; Krystyna Bienkowska-Szewczyk; Krzysztof Pyrc; Lukasz Rabalski; Maciej Kosinski; Natalia Mazur-Panasiuk; Piotr Zabek; Tomasz Dyda
EPI_ISL_458023 to 458028, EPI_ISL_498255 to 498261	Hospital for Tropical Diseases	COVID-19 Network Investigations (CONI) Alliance	Akanitt Jittmittraphap; Angkana Huang; Anthony R. Jones; Arporn Wangwiwatsin; Bhakbhoom Panthan; Chonticha Klungtong; Ekawat Pasomsub; Elizabeth Batty; Insee Sensorn; Janjira Thaipadungpanit; Khajohn Joonsalak; Kingkan Rakmanee; Krittikorn Kumpornsin; Namfon Kotanan; Nantarat Chantawat; Pornsawan Leangwutiwong; Stefan Fernandez; Thanat Chookajorn; Theerarat Kochakarn; Treewat Watthanachockchai; Wasun Chantratita; Weena Janwitthayanan; Wudtichai Manasatienkij
EPI_ISL_417486 to 417487, EPI_ISL_420147, EPI_ISL_471173, EPI_ISL_471176, EPI_ISL_481206, EPI_ISL_493384, EPI_ISL_500775, EPI_ISL_500791 to 500792, EPI_ISL_500797 to 500799, EPI_ISL_549036 to 549037, EPI_ISL_549052, EPI_ISL_549167, EPI_ISL_590904 to 590907, EPI_ISL_590915, EPI_ISL_590936 to 590937, EPI_ISL_590984, EPI_ISL_1490219, EPI_ISL_1490282, EPI_ISL_1490285, EPI_ISL_1490288 to 1490289, EPI_ISL_1490292 to 1490293, EPI_ISL_1490333, EPI_ISL_1490335, EPI_ISL_1490357, EPI_ISL_1490365, EPI_ISL_1490400 to 1490401, EPI_ISL_1490403, EPI_ISL_1490405, EPI_ISL_1490598,			

EPI_ISL_1490601, EPI_ISL_1490603, EPI_ISL_1490610, EPI_ISL_1490628 to 1490629, EPI_ISL_1547511, EPI_ISL_1547575 to 1547583, EPI_ISL_1547585 to 1547588			
see above	Hospital of Southern Norway - Kristiansand, Department of Medical Microbiology	Norwegian Institute of Public Health, Department of Virology	Atiya R Ali; Debech Nadia; Engebretsen Serina Beate; Garcia Llorente Ignacio; Hilde Elshaug; Hilde Synnøve Vollan; Hilde Vollan; Jon Bråte; Kamilla Heddeland Instefjord; Karoline Bragstad; Kathrine Stene-Johansen; Marie Paulsen Madsen; Olav Hungnes; Pedersen Benedikte Nevjen; Rasmus Riis Kopperud
EPI_ISL_1402977 to 1403048, EPI_ISL_1511403 to 1511602	Hospital of the University of Pennsylvania Molecular Pathology Lab	Bushman Lab - University of Pennsylvania	Abigail Glascock; Aoife M. Roche; Arupa Ganguly; Ayannah S. Fitzgerald; Brendan Kelly; Jevon Graham-Wooten; John Everett; Kyle Rodino; Layla A. Khatib; Mike Feldman; Pascha Hokama; Ronald G. Collman and Frederic Bushman; Samantha A. Whiteside; Scott Sherrill-Mix; Shantan Reddy; Young Hwang
EPI_ISL_593915	Hospital, Amneville	National Reference Center for Viruses of Respiratory Infections, Institut Pasteur, Paris	Etienne Simon-Lorière; Fabiana Gambaro; Maud Vanpeene; Sylvie Behillil; Sylvie van der Werf; Vincent Enouf
EPI_ISL_593903	Hospital, Argenteuil	National Reference Center for Viruses of Respiratory Infections, Institut Pasteur, Paris	Etienne Simon-Lorière; Fabiana Gambaro; Maud Vanpeene; Sylvie Behillil; Sylvie van der Werf; Vincent Enouf
EPI_ISL_593910	Hospital, Boulogne-Billancourt	National Reference Center for Viruses of Respiratory Infections, Institut Pasteur, Paris	Etienne Simon-Lorière; Fabiana Gambaro; Maud Vanpeene; Sylvie Behillil; Sylvie van der Werf; Vincent Enouf
EPI_ISL_593921	Hospital, Fameck	National Reference Center for Viruses of Respiratory Infections, Institut Pasteur, Paris	Etienne Simon-Lorière; Fabiana Gambaro; Maud Vanpeene; Sylvie Behillil; Sylvie van der Werf; Vincent Enouf
EPI_ISL_593918	Hospital, Joeuf	National Reference Center for Viruses of Respiratory Infections, Institut Pasteur, Paris	Etienne Simon-Lorière; Fabiana Gambaro; Maud Vanpeene; Sylvie Behillil; Sylvie van der Werf; Vincent Enouf
EPI_ISL_593911	Hospital, Le Chesnay Cédex	National Reference Center for Viruses of Respiratory Infections, Institut Pasteur, Paris	Etienne Simon-Lorière; Fabiana Gambaro; Maud Vanpeene; Sylvie Behillil; Sylvie van der Werf; Vincent Enouf
EPI_ISL_593922 to 593923	Hospital, Maizières les Metz	National Reference Center for Viruses of Respiratory Infections, Institut Pasteur, Paris	Etienne Simon-Lorière; Fabiana Gambaro; Maud Vanpeene; Sylvie Behillil; Sylvie van der Werf; Vincent Enouf
EPI_ISL_593912	Hospital, Marange Silvange	National Reference Center for Viruses of Respiratory Infections, Institut Pasteur, Paris	Etienne Simon-Lorière; Fabiana Gambaro; Maud Vanpeene; Sylvie Behillil; Sylvie van der Werf; Vincent Enouf
EPI_ISL_593914, EPI_ISL_593920	Hospital, Metz	National Reference Center for Viruses of Respiratory Infections, Institut Pasteur, Paris	Etienne Simon-Lorière; Fabiana Gambaro; Maud Vanpeene; Sylvie Behillil; Sylvie van der Werf; Vincent Enouf
EPI_ISL_593917	Hospital, Montigny les Metz	National Reference Center for Viruses of Respiratory Infections, Institut Pasteur, Paris	Etienne Simon-Lorière; Fabiana Gambaro; Maud Vanpeene; Sylvie Behillil; Sylvie van der Werf; Vincent Enouf
EPI_ISL_593919	Hospital, Ottonville	National Reference Center for Viruses of Respiratory Infections, Institut Pasteur, Paris	Etienne Simon-Lorière; Fabiana Gambaro; Maud Vanpeene; Sylvie Behillil; Sylvie van der Werf; Vincent Enouf
EPI_ISL_593916	Hospital, Sarreguemines	National Reference Center for Viruses of Respiratory Infections, Institut Pasteur, Paris	Etienne Simon-Lorière; Fabiana Gambaro; Maud Vanpeene; Sylvie Behillil; Sylvie van der Werf; Vincent Enouf
EPI_ISL_593913	Hospital, Talange	National Reference Center for Viruses of Respiratory Infections, Institut Pasteur, Paris	Etienne Simon-Lorière; Fabiana Gambaro; Maud Vanpeene; Sylvie Behillil; Sylvie van der Werf; Vincent Enouf
EPI_ISL_1406196	Hospìtal del Oriente de Asturias	Laboratorio de Virología HUCA	Abreu F; Alvarez-Arguelles ME; Boga JA; Castelló C; Costales I; Coto E; Gómez de Oña J; Martín-Rodríguez G; Melón S; Perez-Martínez Z; Rojo S; Sandoval M
EPI_ISL_1479097 to 1479118	Houston Health Dept.	Houston Health Dept.	Adolpho Lara; Pamela Brown; Ryker Penn; Yanlai Lai
EPI_ISL_434712 to 435031, EPI_ISL_542487 to 542934, EPI_ISL_543007 to 544954, EPI_ISL_545051 to 545574, EPI_ISL_545581 to 545953, EPI_ISL_545958 to 546435, EPI_ISL_546437 to 546934, EPI_ISL_546937 to 547432			
see above	Houston Methodist Hospital	Houston Methodist Hospital	Chia-Wei Chou; Concepcion C. Cantu; Daniel Boutz; David W. Bernard; Ghazaleh Eskandari; Hakon Jonsson; Heather Hendrickson; Hoang A. T. Nguyen; Hung-Che Kuo; Ilya J. Finkelstein; J. Hunter Long; James J. Davis; Jason S. McLellan; Jimmy Gollihar; Jule Goike; Kamyab Javanmardi; Kari Stefansson; Layne Pruitt; Marcus Nguyen; Matthew Ojeda Saavedra; Maulik Shukla; Muthiah Kumaraswami; Paul A. Christensen; Prasanti Yerramilli; Randall J. Olsen; S. Wesley Long; Sishir Subedi; and James M. Musser
EPI_ISL_450819, EPI_ISL_469059, EPI_ISL_469068	Hovas Askim Familjelakare och BVC	The Public Health Agency of Sweden	Anna Risberg; Anna Wendel; Anna-Malin Linde; Karin Tegmark-Wisell; Maria Lind Karlberg; Mattias Haukland; Mia Brytting; Olov Svartstrom; Oskar Karlsson Lindsjo; Petra Edquist; Reza Advani; Sandra Broddesson; Shamam Muradrasoli; Theresa Enkirch
EPI_ISL_452237, EPI_ISL_452240, EPI_ISL_469062, EPI_ISL_469064 to 469065, EPI_ISL_475513, EPI_ISL_475522 to 475523, EPI_ISL_475525, EPI_ISL_475567			
see above	Huddinge VC	The Public Health Agency of Sweden	Anders Johansson; Anna Risberg; Anna-Malin Linde; Karin Tegmark-Wisell; Maria Lind Karlberg; Mattias Haukland; Mia Brytting; Olov Svartstrom; Oskar Karlsson Lindsjo; Petra Edquist; Reza Advani; Sandra Broddesson; Shamam Muradrasoli; Theresa Enkirch
EPI_ISL_424349, EPI_ISL_437512, EPI_ISL_445088	Human Genetic Research Center, Kawsar Biotech Company	Human Genetic Research Center, Kawsar Biotech Company	Abbasalipour Bashash, M.; Abbasalipour, M.; F. and Haghdoost, Y.; Firoozi, M.; Hosseini; Jadaliha; Kehsvar, Y.; Keshvar, Y.; Khateri, E.; Khosravi; M. and Yazdani, R.; M.A.; Mostafavi Jabbari, S.; Pourtavakoli, S.; R. and Hoseini, F.; Sabeghi, S.; Zeinali; Zeinali, S.
EPI_ISL_454636 to 454641, EPI_ISL_468438 to 468461, EPI_ISL_486280 to 486286, EPI_ISL_513840 to 513855, EPI_ISL_583046 to 583170, EPI_ISL_1477169, EPI_ISL_1477172 to 1477174, EPI_ISL_1477176, EPI_ISL_1477180, EPI_ISL_1477183, EPI_ISL_1477189, EPI_ISL_1477195, EPI_ISL_1477206 to 1477208, EPI_ISL_1477218, EPI_ISL_1477220, EPI_ISL_1477224, EPI_ISL_1477226 to 1477227, EPI_ISL_1477234, EPI_ISL_1477236, EPI_ISL_1477239, EPI_ISL_1477247, EPI_ISL_1477249, EPI_ISL_1477255, EPI_ISL_1477257, EPI_ISL_1477264, EPI_ISL_1477272, EPI_ISL_1477276 to 1477277, EPI_ISL_1477280, EPI_ISL_1477282, EPI_ISL_1477284, EPI_ISL_1477290, EPI_ISL_1477294, EPI_ISL_1477296, EPI_ISL_1477299, EPI_ISL_1477306, EPI_ISL_1477312, EPI_ISL_1477325 to 1477326, EPI_ISL_1477332, EPI_ISL_1477334, EPI_ISL_1477339 to 1477341, EPI_ISL_1477346, EPI_ISL_1477348, EPI_ISL_1477354 to 1477355, EPI_ISL_1477375 to 1477376, EPI_ISL_1477379, EPI_ISL_1477381, EPI_ISL_1477389, EPI_ISL_1477394, EPI_ISL_1477396 to 1477397, EPI_ISL_1477399, EPI_ISL_1477401, EPI_ISL_1477408, EPI_ISL_1477412, EPI_ISL_1477417, EPI_ISL_1477430, EPI_ISL_1477951, EPI_ISL_1477954 to 1477955, EPI_ISL_1477970, EPI_ISL_1477973, EPI_ISL_1477975 to 1477976, EPI_ISL_1477981, EPI_ISL_1477987, EPI_ISL_1477991, EPI_ISL_1477997, EPI_ISL_1478009 to 1478010, EPI_ISL_1478015, EPI_ISL_1478026, EPI_ISL_1478041, EPI_ISL_1478054 to 1478055, EPI_ISL_1478058, EPI_ISL_1478062 to 1478063, EPI_ISL_1478065 to 1478067, EPI_ISL_1478075, EPI_ISL_1478077, EPI_ISL_1478082 to 1478083, EPI_ISL_1478093 to 1478094, EPI_ISL_1478096, EPI_ISL_1478105, EPI_ISL_1478107, EPI_ISL_1478121, EPI_ISL_1478134 to 1478135, EPI_ISL_1478138, EPI_ISL_1478140, EPI_ISL_1478143, EPI_ISL_1478150, EPI_ISL_1478156, EPI_ISL_1478159, EPI_ISL_1478163 to 1478165, EPI_ISL_1478169, EPI_ISL_1478172, EPI_ISL_1478176 to 1478177, EPI_ISL_1478186, EPI_ISL_1478190			
see above	Humboldt County Public Health Laboratory	Chan-Zuckerberg Biohub	CZB Cliahub Consortium
EPI_ISL_526215 to 526238	Hungarian Defence Forces Military Medical Centre	National Laboratory of Virology, Szentágothai Research Centre	Balázs Somogyi; Bálint Eszenyi; Endre Gábor Tóth; Ferenc Jakab; Gábor Kemenesi
EPI_ISL_1497544 to 1497547, EPI_ISL_1506116	Huntington Memorial Hospital	Los Angeles County PHL	P. Hemarajata et al.
EPI_ISL_529961	Husada Utama Hospital	Institute of Tropical Disease, Universitas Airlangga	Aldise M Nastri; Didi Dewanto; Gatot Soegiarto; Jezzy R Dewantari; Kazufumi Shimizu; Krisnoadi Rahardjo; Laksmi Wulandari; Maria I Lusida; Mitsuhiro Nishimura; Resti Yudhawati; Retno A Setyoningrum; Rima R Prasetya; Soetjipto; Yasuko Mori; Yohko K Shimizu
EPI_ISL_450814, EPI_ISL_452241, EPI_ISL_455103	Huslakarna Varmbadhuset Varberg	The Public Health Agency of Sweden	Anna Risberg; Anna-Malin Linde; Johanna Hilmersson; Karin Tegmark-Wisell; Maria Lind Karlberg; Mia Brytting; Olov Svartstrom; Oskar Karlsson Lindsjo; Theresa Enkirch
EPI_ISL_1396893	HÃ´pital Robert Schuman-Laboratoire	Department of Virology, Henri Mondor University Hospital, Assistance Publique Hôpitaux de Paris, Université Paris-Est Créteil, INSERM U955	Alexandre Soulier; Christophe Rodriguez; Elisabeth Trawinski; Guillaume Gricourt; Jean-Michel Pawlotsky; Melissa N'Debi; Slim Fourati; Vanessa Demontant
EPI_ISL_1404482 to 1404483, EPI_ISL_1404573 to 1404575	Hôpital Armand-Trousseau	Department of Virology, Henri Mondor University Hospital, Assistance Publique Hôpitaux de Paris, Université Paris-Est Créteil, INSERM U955	Alexandre Soulier; Christophe Rodriguez; Elisabeth Trawinski; Guillaume Gricourt; Jean-Michel Pawlotsky; Melissa N'Debi; Slim Fourati; Vanessa Demontant
EPI_ISL_1404511 to 1404516, EPI_ISL_1404566 to 1404572	Hôpital Avicenne	Department of Virology, Henri Mondor University Hospital, Assistance Publique Hôpitaux de Paris, Université Paris-Est Créteil, INSERM U955	Alexandre Soulier; Christophe Rodriguez; Elisabeth Trawinski; Guillaume Gricourt; Jean-Michel Pawlotsky; Melissa N'Debi; Slim Fourati; Vanessa Demontant

EPI_ISL_450304 to 450305, EPI_ISL_450308, EPI_ISL_450310, EPI_ISL_450316, EPI_ISL_465681 to 465682, EPI_ISL_465694, EPI_ISL_465698 to 465699, EPI_ISL_535738, EPI_ISL_535796, EPI_ISL_535819, EPI_ISL_535848, EPI_ISL_535851, EPI_ISL_535853, EPI_ISL_535866, EPI_ISL_535869, EPI_ISL_535874, EPI_ISL_535888, EPI_ISL_535890 to 535891, EPI_ISL_535920 to 535923, EPI_ISL_535927, EPI_ISL_535944, EPI_ISL_535949 to 535950, EPI_ISL_535952 to 535953, EPI_ISL_535957, EPI_ISL_535961 to 535962, EPI_ISL_535966, EPI_ISL_535972 to 535973, EPI_ISL_535983, EPI_ISL_536017 to 536018, EPI_ISL_536020, EPI_ISL_536024 to 536025, EPI_ISL_536075, EPI_ISL_536077 to 536078, EPI_ISL_536081 to 536082, EPI_ISL_536087 to 536091, EPI_ISL_536093 to 536094, EPI_ISL_536100, EPI_ISL_536146 to 536149, EPI_ISL_536185 to 536187, EPI_ISL_536191, EPI_ISL_536194, EPI_ISL_536203, EPI_ISL_536214, EPI_ISL_536223, EPI_ISL_536225, EPI_ISL_536233, EPI_ISL_536247, EPI_ISL_536268 to 536270, EPI_ISL_536279 to 536282, EPI_ISL_536284 to 536285, EPI_ISL_536290, EPI_ISL_536292 to 536294, EPI_ISL_536296 to 536298, EPI_ISL_536313 to 536314, EPI_ISL_536316 to 536317, EPI_ISL_536326 to 536328, EPI_ISL_536331, EPI_ISL_536336, EPI_ISL_536338 to 536343, EPI_ISL_536350, EPI_ISL_536371 to 536373			
see above	Hôpital Charles-LeMoyne	Laboratoire de santé publique du Québec	Guillaume Bourque; Ioannis Ragoussis; Jesse Shapiro; Mark Lathrop and Michel Roger; Mark Lathrop and Michel Roger on behalf of the CoVSeQ research group; Sandrine Moreira
EPI_ISL_535839	Hôpital Cité de la Santé	Laboratoire de santé publique du Québec	Guillaume Bourque; Ioannis Ragoussis; Jesse Shapiro; Mark Lathrop and Michel Roger; Sandrine Moreira
EPI_ISL_535792	Hôpital Fleurimont	Laboratoire de santé publique du Québec	Guillaume Bourque; Ioannis Ragoussis; Jesse Shapiro; Mark Lathrop and Michel Roger; Sandrine Moreira
EPI_ISL_1396741 to 1396749, EPI_ISL_1396751 to 1396752, EPI_ISL_1396754 to 1396780, EPI_ISL_1396782 to 1396785, EPI_ISL_1396787 to 1396792, EPI_ISL_1396795 to 1396796, EPI_ISL_1396798 to 1396809, EPI_ISL_1404485 to 1404508, EPI_ISL_1404517 to 1404564			
see above	Hôpital Henri Mondor	Department of Virology, Henri Mondor University Hospital, Assistance Publique Hôpitaux de Paris, Université Paris-Est Créteil, INSERM U955	Alexandre Soulier; Christophe Rodriguez; Elisabeth Trawinski; Guillaume Gricourt; Jean-Michel Pawlotsky; Melissa N'Debi; Slim Fourati; Vanessa Demontant
EPI_ISL_535717, EPI_ISL_535797, EPI_ISL_535912, EPI_ISL_535915, EPI_ISL_536013, EPI_ISL_536029, EPI_ISL_536055, EPI_ISL_536177, EPI_ISL_536180, EPI_ISL_536271, EPI_ISL_536330, EPI_ISL_536379			
see above	Hôpital Honoré-Mercier	Laboratoire de santé publique du Québec	Guillaume Bourque; Ioannis Ragoussis; Jesse Shapiro; Mark Lathrop and Michel Roger; Mark Lathrop and Michel Roger on behalf of the CoVSeQ research group; Sandrine Moreira
EPI_ISL_415650	Hôpital Instruction des Armées - BEGIN	National Reference Center for Viruses of Respiratory Infections, Institut Pasteur, Paris	Angela Brisebarre; Christine Bigaillon; Flora Donati Vincent Enouf; Marion Barbet; Maud Vanpeene; Méline Bizard; Mélnie Albert; Sylvie Behillil; Sylvie van der Werf
EPI_ISL_535745, EPI_ISL_535994	Hôpital Laurentien	Laboratoire de santé publique du Québec	Guillaume Bourque; Ioannis Ragoussis; Jesse Shapiro; Mark Lathrop and Michel Roger; Mark Lathrop and Michel Roger on behalf of the CoVSeQ research group; Sandrine Moreira
EPI_ISL_536108	Hôpital Le Royer	Laboratoire de santé publique du Québec	Guillaume Bourque; Ioannis Ragoussis; Jesse Shapiro; Mark Lathrop and Michel Roger on behalf of the CoVSeQ research group; Sandrine Moreira
EPI_ISL_535755, EPI_ISL_535817, EPI_ISL_535831	Hôpital Maisonneuve-Rosemont	Laboratoire de santé publique du Québec	Guillaume Bourque; Ioannis Ragoussis; Jesse Shapiro; Mark Lathrop and Michel Roger; Sandrine Moreira
EPI_ISL_443295 to 443299	Hôpital Necker - Enfants - Malades Laboratoire de Virologie	National Reference Center for Viruses of Respiratory Infections, Institut Pasteur, Paris	Angela Brisebarre; Etienne Simon-Lorière; Flora Donati; Marianne Leruez-Ville; Marion Barbet; Maud Vanpeene; Mélanie Albert; Méline Bizard; Sylvie Behillil; Sylvie van der Werf; Vincent Enouf
EPI_ISL_1404459 to 1404460	Hôpital Necker-Enfants malades	Department of Virology, Henri Mondor University Hospital, Assistance Publique Hôpitaux de Paris, Université Paris-Est Créteil, INSERM U955	Alexandre Soulier; Christophe Rodriguez; Elisabeth Trawinski; Guillaume Gricourt; Jean-Michel Pawlotsky; Melissa N'Debi; Slim Fourati; Vanessa Demontant
EPI_ISL_535721, EPI_ISL_535795	Hôpital Notre-Dame	Laboratoire de santé publique du Québec	Guillaume Bourque; Ioannis Ragoussis; Jesse Shapiro; Mark Lathrop and Michel Roger; Mark Lathrop and Michel Roger on behalf of the CoVSeQ research group; Sandrine Moreira
EPI_ISL_450309, EPI_ISL_450314 to 450315, EPI_ISL_450317 to 450318, EPI_ISL_465695 to 465696, EPI_ISL_465700, EPI_ISL_465702 to 465703, EPI_ISL_535718, EPI_ISL_535733 to 535734, EPI_ISL_535763, EPI_ISL_535786, EPI_ISL_535818, EPI_ISL_535840, EPI_ISL_535857, EPI_ISL_535879, EPI_ISL_535908 to 535909, EPI_ISL_535948, EPI_ISL_536021, EPI_ISL_536039, EPI_ISL_536041, EPI_ISL_536092, EPI_ISL_536096, EPI_ISL_536099, EPI_ISL_536103, EPI_ISL_536173 to 536176, EPI_ISL_536178 to 536179, EPI_ISL_536188 to 536189, EPI_ISL_536195 to 536196, EPI_ISL_536229, EPI_ISL_536231, EPI_ISL_536234, EPI_ISL_536236, EPI_ISL_536245, EPI_ISL_536253, EPI_ISL_536276 to 536277, EPI_ISL_536295, EPI_ISL_536300, EPI_ISL_536303, EPI_ISL_536305 to 536309, EPI_ISL_536322 to 536324, EPI_ISL_536332 to 536335, EPI_ISL_536346 to 536347, EPI_ISL_536351 to 536352, EPI_ISL_536360, EPI_ISL_536374 to 536375, EPI_ISL_536380, EPI_ISL_536388 to 536389, EPI_ISL_536391, EPI_ISL_536395, EPI_ISL_536397			
see above	Hôpital Pierre-Boucher	Laboratoire de santé publique du Québec	Guillaume Bourque; Ioannis Ragoussis; Jesse Shapiro; Mark Lathrop and Michel Roger; Mark Lathrop and Michel Roger on behalf of the CoVSeQ research group; Sandrine Moreira
EPI_ISL_535731, EPI_ISL_535752, EPI_ISL_535762, EPI_ISL_535766, EPI_ISL_535770 to 535771, EPI_ISL_535850, EPI_ISL_535861 to 535863, EPI_ISL_535881 to 535883, EPI_ISL_535892 to 535893, EPI_ISL_535936, EPI_ISL_535938, EPI_ISL_535954, EPI_ISL_535956, EPI_ISL_536061 to 536062, EPI_ISL_536064 to 536068, EPI_ISL_536070, EPI_ISL_536083 to 536086, EPI_ISL_536125, EPI_ISL_536128, EPI_ISL_536131, EPI_ISL_536137, EPI_ISL_536139 to 536140, EPI_ISL_536143 to 536144, EPI_ISL_536152, EPI_ISL_536157			
see above	Hôpital Pierre-Le Gardeur	Laboratoire de santé publique du Québec	Guillaume Bourque; Ioannis Ragoussis; Jesse Shapiro; Mark Lathrop and Michel Roger; Mark Lathrop and Michel Roger on behalf of the CoVSeQ research group; Sandrine Moreira
EPI_ISL_414631 to 414632	Hôpital Robert Debré Laboratoire de Virologie	National Reference Center for Viruses of Respiratory Infections, Institut Pasteur, Paris	Angela Brisebarre; Flora Donati Vincent Enouf; Laurent Andreoletti; Marion Barbet; Maud Vanpeene; Méline Bizard; Mélnie Albert; Sylvie Behillil; Sylvie van der Werf
EPI_ISL_535810, EPI_ISL_535859 to 535860, EPI_ISL_535897 to 535901, EPI_ISL_535969, EPI_ISL_536003, EPI_ISL_536010 to 536011, EPI_ISL_536056 to 536057, EPI_ISL_536252			
see above	Hôpital Sainte-Croix	Laboratoire de santé publique du Québec	Guillaume Bourque; Ioannis Ragoussis; Jesse Shapiro; Mark Lathrop and Michel Roger; Mark Lathrop and Michel Roger on behalf of the CoVSeQ research group; Sandrine Moreira
EPI_ISL_535963, EPI_ISL_536046	Hôpital d'Alma	Laboratoire de santé publique du Québec	Guillaume Bourque; Ioannis Ragoussis; Jesse Shapiro; Mark Lathrop and Michel Roger on behalf of the CoVSeQ research group; Sandrine Moreira
EPI_ISL_536265	Hôpital de Chandler	Laboratoire de santé publique du Québec	Guillaume Bourque; Ioannis Ragoussis; Jesse Shapiro; Mark Lathrop and Michel Roger on behalf of the CoVSeQ research group; Sandrine Moreira
EPI_ISL_535829, EPI_ISL_535905 to 535906, EPI_ISL_535964 to 535965, EPI_ISL_536019, EPI_ISL_536107	Hôpital de Chicoutimi	Laboratoire de santé publique du Québec	Guillaume Bourque; Ioannis Ragoussis; Jesse Shapiro; Mark Lathrop and Michel Roger; Mark Lathrop and Michel Roger on behalf of the CoVSeQ research group; Sandrine Moreira
EPI_ISL_465691, EPI_ISL_535858, EPI_ISL_536266, EPI_ISL_536311, EPI_ISL_536384	Hôpital de Gatineau	Laboratoire de santé publique du Québec	Guillaume Bourque; Ioannis Ragoussis; Jesse Shapiro; Mark Lathrop and Michel Roger on behalf of the CoVSeQ research group; Sandrine Moreira
EPI_ISL_465686, EPI_ISL_535794, EPI_ISL_535809, EPI_ISL_535886 to 535887, EPI_ISL_535911, EPI_ISL_535989, EPI_ISL_535993, EPI_ISL_535995, EPI_ISL_536051, EPI_ISL_536117 to 536118, EPI_ISL_536122, EPI_ISL_536124, EPI_ISL_536127, EPI_ISL_536132, EPI_ISL_536135 to 536136, EPI_ISL_536168 to 536169, EPI_ISL_536207 to 536210, EPI_ISL_536230, EPI_ISL_536238 to 536239, EPI_ISL_536242, EPI_ISL_536244, EPI_ISL_536267, EPI_ISL_536275, EPI_ISL_536291, EPI_ISL_536312, EPI_ISL_536363 to 536364, EPI_ISL_536367, EPI_ISL_536385 to 536387, EPI_ISL_536392 to 536394, EPI_ISL_536396			
see above	Hôpital de Hull	Laboratoire de santé publique du Québec	Guillaume Bourque; Ioannis Ragoussis; Jesse Shapiro; Mark Lathrop and Michel Roger; Mark Lathrop and Michel Roger on behalf of the CoVSeQ research group; Sandrine Moreira
EPI_ISL_535744	Hôpital de Lasalle	Laboratoire de santé publique du Québec	Guillaume Bourque; Ioannis Ragoussis; Jesse Shapiro; Mark Lathrop and Michel Roger; Sandrine Moreira
EPI_ISL_465683 to 465684, EPI_ISL_535885, EPI_ISL_536111 to 536112, EPI_ISL_536262, EPI_ISL_536289	Hôpital de Maria	Laboratoire de santé publique du Québec	Guillaume Bourque; Ioannis Ragoussis; Jesse Shapiro; Mark Lathrop and Michel Roger on behalf of the CoVSeQ research group; Sandrine Moreira
EPI_ISL_535945	Hôpital de Mont-Laurier	Laboratoire de santé publique du Québec	Guillaume Bourque; Ioannis Ragoussis; Jesse Shapiro; Mark Lathrop and Michel Roger; Sandrine Moreira
EPI_ISL_536171	Hôpital de Papineau	Laboratoire de santé publique du Québec	Guillaume Bourque; Ioannis Ragoussis; Jesse Shapiro; Mark Lathrop and Michel Roger on behalf of the CoVSeQ research group; Sandrine Moreira
EPI_ISL_535985 to 535987	Hôpital de Rouyn-Noranda	Laboratoire de santé publique du Québec	Guillaume Bourque; Ioannis Ragoussis; Jesse Shapiro; Mark Lathrop and Michel Roger on behalf of the CoVSeQ research group; Sandrine Moreira
EPI_ISL_535793, EPI_ISL_535865, EPI_ISL_535867, EPI_ISL_535878, EPI_ISL_535889, EPI_ISL_535924, EPI_ISL_535926, EPI_ISL_535929 to 535930, EPI_ISL_535941 to 535943, EPI_ISL_535947, EPI_ISL_535951, EPI_ISL_535982, EPI_ISL_536008, EPI_ISL_536049, EPI_ISL_536058, EPI_ISL_536069, EPI_ISL_536071 to 536072			
see above	Hôpital de Saint-Eustache	Laboratoire de santé publique du Québec	Guillaume Bourque; Ioannis Ragoussis; Jesse Shapiro; Mark Lathrop and Michel Roger; Mark Lathrop and Michel Roger on behalf of the CoVSeQ research group; Sandrine Moreira
EPI_ISL_535716, EPI_ISL_535723, EPI_ISL_535728, EPI_ISL_535856	Hôpital de Verdun	Laboratoire de santé publique du Québec	Guillaume Bourque; Ioannis Ragoussis; Jesse Shapiro; Mark Lathrop and Michel Roger; Mark Lathrop and Michel Roger on behalf of the CoVSeQ research group; Sandrine Moreira

EPI_ISL_535739	Hôpital de l'Enfant-Jésus	Laboratoire de santé publique du Québec	Guillaume Bourque; Ioannis Ragoussis; Jesse Shapiro; Mark Lathrop and Michel Roger; Sandrine Moreira
EPI_ISL_535914, EPI_ISL_535937, EPI_ISL_535968, EPI_ISL_535971, EPI_ISL_535974 to 535975, EPI_ISL_535978, EPI_ISL_535996 to 535999, EPI_ISL_536001, EPI_ISL_536030, EPI_ISL_536032 to 536033, EPI_ISL_536037, EPI_ISL_536246, EPI_ISL_536248 to 536251			
see above	Hôpital du Centre-de-la-Mauricie	Laboratoire de santé publique du Québec	Guillaume Bourque; Ioannis Ragoussis; Jesse Shapiro; Mark Lathrop and Michel Roger; Mark Lathrop and Michel Roger on behalf of the CoVSeQ research group; Sandrine Moreira
EPI_ISL_535812	Hôpital du Sacré-Coeur de Montréal	Laboratoire de santé publique du Québec	Guillaume Bourque; Ioannis Ragoussis; Jesse Shapiro; Mark Lathrop and Michel Roger on behalf of the CoVSeQ research group; Sandrine Moreira
EPI_ISL_450307, EPI_ISL_450311 to 450313, EPI_ISL_465679 to 465680, EPI_ISL_465693, EPI_ISL_465701, EPI_ISL_535847, EPI_ISL_535868, EPI_ISL_535880, EPI_ISL_535913, EPI_ISL_535917 to 535919, EPI_ISL_535988, EPI_ISL_536026, EPI_ISL_536095, EPI_ISL_536097 to 536098, EPI_ISL_536102, EPI_ISL_536105, EPI_ISL_536163, EPI_ISL_536165, EPI_ISL_536167, EPI_ISL_536170, EPI_ISL_536190, EPI_ISL_536192 to 536193, EPI_ISL_536198, EPI_ISL_536200 to 536202, EPI_ISL_536226 to 536228, EPI_ISL_536254, EPI_ISL_536257, EPI_ISL_536259, EPI_ISL_536261, EPI_ISL_536273 to 536274, EPI_ISL_536287, EPI_ISL_536318 to 536320, EPI_ISL_536349, EPI_ISL_536354 to 536359, EPI_ISL_536362, EPI_ISL_536390			
see above	Hôpital du Suroît	Laboratoire de santé publique du Québec	Guillaume Bourque; Ioannis Ragoussis; Jesse Shapiro; Mark Lathrop and Michel Roger; Mark Lathrop and Michel Roger on behalf of the CoVSeQ research group; Sandrine Moreira
EPI_ISL_535991, EPI_ISL_536113 to 536114	Hôpital et Centre d'Hébergement de Sept-Iles	Laboratoire de santé publique du Québec	Guillaume Bourque; Ioannis Ragoussis; Jesse Shapiro; Mark Lathrop and Michel Roger on behalf of the CoVSeQ research group; Sandrine Moreira
EPI_ISL_535719 to 535720, EPI_ISL_535756 to 535757, EPI_ISL_535769	Hôpital général Juif	Laboratoire de santé publique du Québec	Guillaume Bourque; Ioannis Ragoussis; Jesse Shapiro; Mark Lathrop and Michel Roger; Mark Lathrop and Michel Roger on behalf of the CoVSeQ research group; Sandrine Moreira
EPI_ISL_535852, EPI_ISL_535875	Hôpital général du Lakeshore	Laboratoire de santé publique du Québec	Guillaume Bourque; Ioannis Ragoussis; Jesse Shapiro; Mark Lathrop and Michel Roger; Mark Lathrop and Michel Roger on behalf of the CoVSeQ research group; Sandrine Moreira
EPI_ISL_536106, EPI_ISL_536205	Hôpital régional de Rimouski	Laboratoire de santé publique du Québec	Guillaume Bourque; Ioannis Ragoussis; Jesse Shapiro; Mark Lathrop and Michel Roger on behalf of the CoVSeQ research group; Sandrine Moreira
EPI_ISL_535791, EPI_ISL_535798, EPI_ISL_535837 to 535838, EPI_ISL_536022	Hôpital régional de Saint-Jérôme	Laboratoire de santé publique du Québec	Guillaume Bourque; Ioannis Ragoussis; Jesse Shapiro; Mark Lathrop and Michel Roger; Mark Lathrop and Michel Roger on behalf of the CoVSeQ research group; Sandrine Moreira
EPI_ISL_536101	Hôpital, CLSC et Centre d'Hébergement de Roberval	Laboratoire de santé publique du Québec	Guillaume Bourque; Ioannis Ragoussis; Jesse Shapiro; Mark Lathrop and Michel Roger on behalf of the CoVSeQ research group; Sandrine Moreira
EPI_ISL_1477129 to 1477166, EPI_ISL_1522587, EPI_ISL_1522606, EPI_ISL_1522615, EPI_ISL_1522627, EPI_ISL_1522651, EPI_ISL_1522661, EPI_ISL_1522689, EPI_ISL_1522699, EPI_ISL_1522701, EPI_ISL_1522706, EPI_ISL_1522760, EPI_ISL_1522789 to 1522790, EPI_ISL_1522905 to 1522915, EPI_ISL_1523070, EPI_ISL_1523142, EPI_ISL_1523149 to 1523150, EPI_ISL_1523158, EPI_ISL_1523172, EPI_ISL_1523187, EPI_ISL_1523289, EPI_ISL_1523353 to 1523357			
see above	Hôpitaux Robert Schuman	Laboratoire national de sante, Microbiology, Microbial Genomics Platform	Alain Hakim; Anke Wienecke-Baldacchino; Catherine Ragimbeau; Fatu Djabi; Jessica Tapp; Lise Pignon; Nawfal Faik; Raoul Salmon; Tamir Abdelrahman
EPI_ISL_535725, EPI_ISL_535750 to 535751, EPI_ISL_535758 to 535759, EPI_ISL_535820 to 535821, EPI_ISL_535833 to 535836	Hôtel-Dieu de Lévis	Laboratoire de santé publique du Québec	Guillaume Bourque; Ioannis Ragoussis; Jesse Shapiro; Mark Lathrop and Michel Roger; Mark Lathrop and Michel Roger on behalf of the CoVSeQ research group; Sandrine Moreira
EPI_ISL_535903 to 535904, EPI_ISL_535990, EPI_ISL_535992, EPI_ISL_536116, EPI_ISL_536121	Hôtel-Dieu de Sorel	Laboratoire de santé publique du Québec	Guillaume Bourque; Ioannis Ragoussis; Jesse Shapiro; Mark Lathrop and Michel Roger; Mark Lathrop and Michel Roger on behalf of the CoVSeQ research group; Sandrine Moreira
EPI_ISL_1391024 to 1391028	I.R.C.C.S. "S. De Bellis" - Ente Ospedaliero	Istituto Zooprofilattico Sperimentale della Puglia e della Basilicata	Bianco A.; Capozzi L.; Del Sambro L.; Lippolis A.; Notarnicola M.; Parisi A.; Simone D.
EPI_ISL_1446602 to 1446606, EPI_ISL_1446728, EPI_ISL_1446941, EPI_ISL_1516424 to 1516431, EPI_ISL_1528387 to 1528396	IA State Hygienic Laboratory	Centers for Disease Control and Prevention Division of Viral Diseases, Pathogen Discovery	Alison Laufer Halpin; Ben L. Rambo-Martin; Clinton R. Paden; Dakota Howard; Darlene Wagner; Dave Wentworth; Dhwani Batra; Jasmine Padilla; Justin Lee; Katie Dillon; Krista Queen; Kristen Knipe; Kristine Lacek; Mark Burroughs; Matthew Schmerer; Mili Sheth; Peter Cook; Sam Shepard; Sarah Nobles; Shoshona Le; Suxiang Tong; Vivien Dugan; Yvette Unoarumhi
EPI_ISL_424865, EPI_ISL_424895 to 424900, EPI_ISL_447842	IA State Hygienic Laboratory	Pathogen Discovery, Respiratory Viruses Branch, Division of Viral Diseases, Centers for Disease Control and Prevention	Alison S. Laufer Halpin; Anna Uehara; Christopher A. Elkins; Clinton R. Paden; Haibin Wang; Jasmine Padilla; Jing Zhang; Justin Lee; Krista Queen; Mary S. Keckler; Rachel Marine; Suxiang Tong; Yan Li; Ying Tao
EPI_ISL_452787 to 452795	ICAR-National Institute of High Security Animal Diseases	ICAR-National Institute of High Security Animal Diseases	Anamika Mishra; Ashutosh Aasdev; Ashwin Ashok Raut; Atul Kumar Pateriya; Chakradhar Tosh; Chandan Kumar Dubey; Dipesh Kumar Nayak; Fateh Singh; G Venkatesh; Harshad Murugkar; Katherukamem Rajukumar; Manoj Kumar; Meghna Tripathi; Naveen Kumar; Niranjan Mishra; Pradeep Gandhale; Pushpendra Namdeo; Pushpendra Singh; Richa Sood; Rupal Singh; Sandeep Bhatia; Sandeep Kumar Jhade; Senmannan Kalaiyarasu; Sethil Kumar D; Shanmugasundaram Nagarajan; Shashi Bhushan Sudhakar; Suman Kumari Shah; Suman Mishra; Sushil Tripathi; Vijendra Pal Singh; Vishnupriya Patil
EPI_ISL_1517301	ICH-SION	Laboratory of genomics and metagenomics	Claire Bertelli; Damien Jacot; Gilbert Greub; SÃ©bastien Aeby; Trestan Pillonel
EPI_ISL_430464 to 430468, EPI_ISL_437536 to 437539, EPI_ISL_455640 to 455679, EPI_ISL_508441 to 508488	ICMR-National Institute of Cholera and Enteric Diseases	National Institute of Biomedical Genomics	Ananya Chatterjee; Arindam Maitra; Hasina Banu; Mamta Chawla Sarkar; Saumitra Das; Shanta Dutta; Sreedhar Chinnaswamy
EPI_ISL_511899	ICMR-National Institute of Cholera and Enteric Diseases	National Institute of Biomedical Genomics - DBT's PAN-INDIA 1000 SARS--CoV-2 RNA Genome Sequencing Consortium	Ananya Chatterjee; Arindam Maitra; Hasina Banu; Mamta Chawla Sarkar; Saumitra Das; Shanta Dutta; Sreedhar Chinnaswamy
EPI_ISL_1446977 to 1446978	ID Bureau of Laboratories	Centers for Disease Control and Prevention Division of Viral Diseases, Pathogen Discovery	Alison Laufer Halpin; Ben L. Rambo-Martin; Clinton R. Paden; Dakota Howard; Darlene Wagner; Dave Wentworth; Dhwani Batra; Jasmine Padilla; Justin Lee; Katie Dillon; Krista Queen; Kristen Knipe; Kristine Lacek; Mark Burroughs; Matthew Schmerer; Mili Sheth; Peter Cook; Sam Shepard; Sarah Nobles; Shoshona Le; Suxiang Tong; Vivien Dugan; Yvette Unoarumhi
EPI_ISL_539491, EPI_ISL_560319	IDSP unit, Dehradun	CSIR-Institute of Microbial Technology	Amandeep Kaur; Anu Singh; Ashwani Kumar; Debarghya Ghose; Dipak Dutta; Harsh Goar; Kanika Bansal; Navin Baid; Poushali Chakraborty; Prabhu B. Patil; Rajesh Kumar Mishra; Sanjeet Kumar; Sanjeev Khosla
EPI_ISL_569614	IHS Fort Yates Hospital	South Dakota Public Health Laboratory	Jacob Garfin; Matt Plumb; Xiong Wang; and Chris Carlson
EPI_ISL_528685	IIP Institute of Genomics and Integrative Biology	IIP Institute of Genomics and Integrative Biology	Ajit Shewale; Akshay Kanakan; Anurag Agrawal; Azka Khan; Bharathram Uppili; Mahesh S. Dhar; Mohammed Faruq; Partha Rakshit; Pooja Sharma; RadhaKrishnan VS; Rajesh Pandey; Robin Marwal; Sandhya Kabra; Simmi Tiwari; Sujeet Singh; Tushar Nale; Vivekanand A
EPI_ISL_1446979	IL Department of Public Health Chicago Laboratory	Centers for Disease Control and Prevention Division of Viral Diseases, Pathogen Discovery	Alison Laufer Halpin; Ben L. Rambo-Martin; Clinton R. Paden; Dakota Howard; Darlene Wagner; Dave Wentworth; Dhwani Batra; Jasmine Padilla; Justin Lee; Katie Dillon; Krista Queen; Kristen Knipe; Kristine Lacek; Mark Burroughs; Matthew Schmerer; Mili Sheth; Peter Cook; Sam Shepard; Sarah Nobles; Shoshona Le; Suxiang Tong; Vivien Dugan; Yvette Unoarumhi
EPI_ISL_1509925, EPI_ISL_1509933 to 1509934, EPI_ISL_1509938, EPI_ISL_1509940, EPI_ISL_1509947 to 1509950	IL Department of Public Health Chicago Laboratory	Genomics and Discovery, Respiratory Viruses Branch, Division of Viral Diseases, Centers for Disease Control and Prevention	Adam Retchless; Anna Kelleher; Anna Montmayeur; Anna Uehara; Brian Lynch; Clinton R. Paden; Haibin Wang; Han Jia Justin Ng; Jing Zhang; Justin Lee; Krista Queen; Mark Burroughs; Peter Cook; Rachel Marine; Suxiang Tong; Yan Li; Ying Tao
EPI_ISL_404253, EPI_ISL_410045	IL Department of Public Health Chicago Laboratory	Pathogen Discovery, Respiratory Viruses Branch, Division of Viral Diseases, Centers for Dieases Control and Prevention	Anna Uehara; Brett L. Whitaker; Brian Lynch; Clinton R. Paden; Janna' R. Murray; Jing Zhang; Krista Queen; Lijuan Wang; Senthil Kumar K. Sakthivel; Shifaq Kamili; Stephen Lindstrom; Susan I. Gerber; Suxiang Tong; Xiaoyan Lu; Yan Li; Ying Tao
EPI_ISL_424850 to 424851, EPI_ISL_452134	IL Department of Public Health Chicago Laboratory	Pathogen Discovery, Respiratory Viruses Branch, Division of Viral Diseases, Centers for Disease Control and Prevention	Alison S. Laufer Halpin; Anna Montmayeur; Anna Uehara; Christopher A. Elkins; Clinton R. Paden; Haibin Wang; Jing Zhang; Krista Queen; Mary S. Keckler; Rachel Marine; Suxiang Tong; Yan Li; Ying Tao; Zachary Weiner
EPI_ISL_1500236, EPI_ISL_1500238	IL Department of Veterans Affairs	Illinois Department of Public Health - Springfield Lab	Bryan Sim; Gordon McCall
EPI_ISL_1446583 to 1446590, EPI_ISL_1446744, EPI_ISL_1446928 to 1446931, EPI_ISL_1528365 to 1528373	IL Dept. of Public Health Springfield Laboratory	Centers for Disease Control and Prevention Division of Viral Diseases, Pathogen Discovery	Alison Laufer Halpin; Ben L. Rambo-Martin; Clinton R. Paden; Dakota Howard; Darlene Wagner; Dave Wentworth; Dhwani Batra; Jasmine Padilla; Justin Lee; Katie Dillon; Krista Queen; Kristen Knipe; Kristine Lacek; Mark Burroughs; Matthew Schmerer; Mili Sheth; Peter Cook; Sam Shepard; Sarah Nobles; Shoshona Le; Suxiang Tong; Vivien Dugan; Yvette Unoarumhi
EPI_ISL_1408896, EPI_ISL_1448511, EPI_ISL_1448513 to 1448515, EPI_ISL_1448517, EPI_ISL_1448520 to 1448521, EPI_ISL_1448526, EPI_ISL_1509931, EPI_ISL_1509935, EPI_ISL_1509939, EPI_ISL_1509944 to 1509945			

see above	IL Dept. of Public Health Springfield Laboratory	Genomics and Discovery, Respiratory Viruses Branch, Division of Viral Diseases, Centers for Disease Control and Prevention	Adam Retchless; Anna Kelleher; Anna Montmayeur; Anna Uehara; Brian Lynch; Clinton R. Paden; Haibin Wang; Han Jia Justin Ng; Jing Zhang; Justin Lee; Krista Queen; Mark Burroughs; Peter Cook; Rachel Marine; Suxiang Tong; Yan Li; Ying Tao
EPI_ISL_1495760 to 1495761	ILV Kärnten	Bergthaler laboratory, CeMM Research Center for Molecular Medicine of the Austrian Academy of Sciences	Andreas Bergthaler; Anna Schedl; Bekir Erguner; Benedikt Agerer; Christoph Bock; Fabian Amman; Jan Laine; Lukas Endler; Maelle Le Moing; Martin Senekowitsch; Michael Schuster; Petr Triska; Thomas Penz
EPI_ISL_445379	IMALAB- HOSPITAL FACH	Instituto de Salud Publica de Chile	Alejandra Acevedo; Andrés E Castillo; Bárbara Parra; Carolina Tambley; Gabriel Leal; Jaime Lagos; Jorge Fernandez; Loredana Arata; Patricia Bustos; Paz Tapia; Rodrigo Fasce; Winston Andrade
EPI_ISL_1435843, EPI_ISL_1435858, EPI_ISL_1435864, EPI_ISL_1435868, EPI_ISL_1435871 to 1435872, EPI_ISL_1435874, EPI_ISL_1435877, EPI_ISL_1435890, EPI_ISL_1435895, EPI_ISL_1435897, EPI_ISL_1435901, EPI_ISL_1439897			
see above	IMD - Institut für Medizinische Diagnostik Berlin-Potsdam	Robert Koch Institute	
EPI_ISL_1435841, EPI_ISL_1435844 to 1435845, EPI_ISL_1435847, EPI_ISL_1435849 to 1435850, EPI_ISL_1435852, EPI_ISL_1435855 to 1435857, EPI_ISL_1435859 to 1435863, EPI_ISL_1435866 to 1435867, EPI_ISL_1435870, EPI_ISL_1435875, EPI_ISL_1435880, EPI_ISL_1435882 to 1435883, EPI_ISL_1435885, EPI_ISL_1435887, EPI_ISL_1435889, EPI_ISL_1435894, EPI_ISL_1435899, EPI_ISL_1439879, EPI_ISL_1439881, EPI_ISL_1439888, EPI_ISL_1439900			
see above	IMD - Labor Oderland	Robert Koch Institute	
EPI_ISL_1435854, EPI_ISL_1435873, EPI_ISL_1435876, EPI_ISL_1435891, EPI_ISL_1435893	IMD - MVZ Labor Greifswald	Robert Koch Institute	
EPI_ISL_1435842, EPI_ISL_1435846, EPI_ISL_1435848, EPI_ISL_1435851, EPI_ISL_1435853, EPI_ISL_1435865, EPI_ISL_1435869, EPI_ISL_1435878 to 1435879, EPI_ISL_1435881, EPI_ISL_1435884, EPI_ISL_1435886, EPI_ISL_1435892, EPI_ISL_1439870 to 1439872, EPI_ISL_1439876, EPI_ISL_1439882 to 1439885, EPI_ISL_1439889 to 1439892, EPI_ISL_1439894 to 1439895, EPI_ISL_1439898 to 1439899			
see above	IMD - MVZ Labor Martinsried	Robert Koch Institute	
EPI_ISL_1439873 to 1439875, EPI_ISL_1439878, EPI_ISL_1439887, EPI_ISL_1439893	IMD - Medizinisches Labor Rostock	Robert Koch Institute	
EPI_ISL_1433505, EPI_ISL_1433507, EPI_ISL_1433511, EPI_ISL_1433513, EPI_ISL_1433515, EPI_ISL_1433533, EPI_ISL_1433537, EPI_ISL_1433549, EPI_ISL_1433553, EPI_ISL_1433574, EPI_ISL_1433579 to 1433580, EPI_ISL_1433582, EPI_ISL_1433586, EPI_ISL_1433590, EPI_ISL_1433592, EPI_ISL_1439368, EPI_ISL_1439370, EPI_ISL_1439372, EPI_ISL_1439374, EPI_ISL_1439376, EPI_ISL_1439378 to 1439379, EPI_ISL_1439382, EPI_ISL_1439385 to 1439386, EPI_ISL_1439391, EPI_ISL_1439393, EPI_ISL_1439396, EPI_ISL_1439402 to 1439404, EPI_ISL_1439406 to 1439407, EPI_ISL_1439409 to 1439410, EPI_ISL_1439412 to 1439415, EPI_ISL_1439417 to 1439422, EPI_ISL_1439424, EPI_ISL_1439426 to 1439431, EPI_ISL_1439434, EPI_ISL_1439436			
see above	IMD Labor Frankfurt	Robert Koch Institute	
EPI_ISL_1495581 to 1495601	IMP - Research Institute of Molecular Pathology	Bergthaler laboratory, CeMM Research Center for Molecular Medicine of the Austrian Academy of Sciences	Andreas Bergthaler; Anna Schedl; Bekir Erguner; Benedikt Agerer; Christoph Bock; Fabian Amman; Jan Laine; Lukas Endler; Maelle Le Moing; Martin Senekowitsch; Michael Schuster; Petr Triska; Thomas Penz
EPI_ISL_1446516, EPI_ISL_1446519, EPI_ISL_1446522, EPI_ISL_1446524, EPI_ISL_1446527, EPI_ISL_1446530, EPI_ISL_1446533, EPI_ISL_1446535, EPI_ISL_1446538, EPI_ISL_1446846 to 1446854, EPI_ISL_1516432 to 1516444, EPI_ISL_1528424 to 1528431			
see above	IN State Department of Health Laboratory Services	Centers for Disease Control and Prevention Division of Viral Diseases, Pathogen Discovery	Alison Laufer Halpin; Ben L. Rambo-Martin; Clinton R. Paden; Dakota Howard; Darlene Wagner; Dave Wentworth; Dhwani Batra; Jasmine Padilla; Justin Lee; Katie Dillon; Krista Queen; Kristen Knipe; Kristine Lacek; Mark Burroughs; Matthew Schmerer; Mili Sheth; Peter Cook; Sam Shepard; Sarah Nobles; Shoshona Le; Suxiang Tong; Vivien Dugan; Yvette Unoarumhi
EPI_ISL_1448516, EPI_ISL_1448524	IN State Department of Health Laboratory Services	Genomics and Discovery, Respiratory Viruses Branch, Division of Viral Diseases, Centers for Disease Control and Prevention	Anna Kelleher; Anna Uehara; Brian Lynch; Clinton R. Paden; Haibin Wang; Han Jia Justin Ng; Jing Zhang; Krista Queen; Peter Cook; Suxiang Tong; Yan Li; Ying Tao
EPI_ISL_1394840 to 1394857, EPI_ISL_1396656 to 1396666, EPI_ISL_1400444 to 1400460, EPI_ISL_1400472 to 1400488, EPI_ISL_1403051 to 1403081, EPI_ISL_1404145 to 1404168, EPI_ISL_1404436 to 1404450, EPI_ISL_1415061 to 1415085, EPI_ISL_1416193 to 1416221, EPI_ISL_1416223 to 1416255, EPI_ISL_1490750 to 1490792, EPI_ISL_1490818 to 1490860, EPI_ISL_1527942 to 1527953, EPI_ISL_1528242 to 1528265, EPI_ISL_1563635 to 1563659, EPI_ISL_1563661 to 1563664, EPI_ISL_1563666 to 1563669, EPI_ISL_1563671 to 1563674, EPI_ISL_1563676 to 1563677			
see above	IN State Department of Health Laboratory Services	IN State Department of Health Laboratory Services	Ankita Kashikar; Brian Pope; Cassandra Campion; Jamie Yeadon; Kyle Brownlee; Lixia Liu; Mark Glazier; Melissa Hindenlang
EPI_ISL_424866, EPI_ISL_426422 to 426424, EPI_ISL_452131, EPI_ISL_452135 to 452136, EPI_ISL_527666, EPI_ISL_540431	IN State Department of Health Laboratory Services	Pathogen Discovery, Respiratory Viruses Branch, Division of Viral Diseases, Centers for Disease Control and Prevention	Alison S. Laufer Halpin; Anna Montmayeur; Anna Uehara; Brian Lynch; Christopher A. Elkins; Clinton R. Paden; Haibin Wang; Jing Zhang; Krista Queen; Mary S. Keckler; Rachel Marine; Suxiang Tong; Yan Li; Ying Tao; Zachary Weiner
EPI_ISL_419255, EPI_ISL_493328 to 493331, EPI_ISL_590693 to 590698, EPI_ISL_603137 to 603187, EPI_ISL_609989 to 609999, EPI_ISL_1455669, EPI_ISL_1464620, EPI_ISL_1492572, EPI_ISL_1496002, EPI_ISL_1524727 to 1524728, EPI_ISL_1524737 to 1524738, EPI_ISL_1524743 to 1524752			
see above	INMI Lazzaro Spallanzani IRCCS	INMI Lazzaro Spallanzani IRCCS	A Di Caro; Annunziata Tamburro; Antonino Di Caro; B Bartolini; Barbara Bartolini; Beatrice Valli; C.E.M Gruber; CEM Gruber; Cesare E. M. Gruber; Cesare E.M. Gruber; Concetta Castilletti; Daniele Lapa; E Giombini; Eleonora Lalle; Emanuela Giombini; F Messina; F Santini; Fabrizio Carletti; Francesca Colavita; Francesco Messina; Francesco Vairo; Fulvia Pimpinelli; G Bonfiglio; Giuseppe Ippolito.; Giuseppina Cappiello; M Rueca; MR Capobianchi; Marcello Meledandri; Maria Letizia Schiavone; Maria R. Capobianchi; Mariarosa Gaddi; Martina Rueca; O Butera; Patrizia Massarelli; Simone Lanini
EPI_ISL_410545 to 410546, EPI_ISL_417921 to 417923, EPI_ISL_419254, EPI_ISL_424342 to 424344	INMI Lazzaro Spallanzani IRCCS	Laboratory of Virology, INMI Lazzaro Spallanzani IRCCS	Antonino Di Caro; Barbara Bartolini; Cesare E. M. Gruber; Cesare Ernesto Maria Gruber; Concetta Castilletti; Daniele Lapa; Eleonora Lalle; Emanuela Giombini; Emanuele Nicastri; Fabrizio Carletti; Francesca Colavita; Francesco Messina; Francesco Vairo; Giulia Matusali; Giuseppe Ippolito; Giuseppe Ippolito.; Licia Bordi; Maria R. Capobianchi; Maria Rosaria Capobianchi; Martina Rueca
EPI_ISL_1419053 to 1419115	INSACOG-Mizoram	National Institute of Biomedical Genomics - INSACOG	Arindam Maitra; Gracy Laldinmawii; N Senthil Kumar; Nidhan Kumar Biswas; Saumitra Das; Sreedhar Chinnaswamy; Swagnik Roy
EPI_ISL_1419116 to 1419142	INSACOG-Sikkim	National Institute of Biomedical Genomics - INSACOG	Arindam Maitra; Kaden Zangmu Bhutia; Nidhan Kumar Biswas; Saumitra Das; Sreedhar Chinnaswamy Tshering Ongmu Bhutia; Tara D Sharma
EPI_ISL_1419048 to 1419052, EPI_ISL_1419143 to 1419336, EPI_ISL_1419338 to 1419924	INSACOG-WB	National Institute of Biomedical Genomics - INSACOG	Ajay Chakraborti; Arindam Maitra; Bhaswati Bandyopadhyay; Nidhan Kumar Biswas; Saumitra Das; Sreedhar Chinnaswamy; Tamal Ghosh
EPI_ISL_1461179 to 1461180, EPI_ISL_1526344 to 1526345	INSTITUT BERGONIE	CNR Virus des Infections Respiratoires - France SUD	Antonin Bal; Bruno Lina; Gregory Destras; Gwendolyne Burfin; Hadrien Regue; Laurence Josset; Martine Valette; Quentin Semanas
EPI_ISL_445302	INSTITUTO MEDICO LEGAL	Instituto de Salud Publica de Chile	Alejandra Acevedo; Andrés E Castillo; Bárbara Parra; Carolina Tambley; Gabriel Leal; Jaime Lagos; Jorge Fernandez; Loredana Arata; Patricia Bustos; Paz Tapia; Rodrigo Fasce; Winston Andrade
EPI_ISL_1399230, EPI_ISL_1399246, EPI_ISL_1406249, EPI_ISL_1497611 to 1497612	INT Fondazione Pascale	INT Fondazione Pascale	INT Fondazione Pascale
EPI_ISL_445357	INTEGRAMEDICA CENTROS MEDICOS S.A.	Instituto de Salud Publica de Chile	Alejandra Acevedo; Andrés E Castillo; Bárbara Parra; Carolina Tambley; Gabriel Leal; Jaime Lagos; Jorge Fernandez; Loredana Arata; Patricia Bustos; Paz Tapia; Rodrigo Fasce; Winston Andrade
EPI_ISL_445261	INTEGRAMEDICA LAB. CLINICO LTDA.	Instituto de Salud Publica de Chile	Alejandra Acevedo; Andrés E Castillo; Bárbara Parra; Carolina Tambley; Gabriel Leal; Jaime Lagos; Jorge Fernandez; Loredana Arata; Patricia Bustos; Paz Tapia; Rodrigo Fasce; Winston Andrade
EPI_ISL_445324	INTEGRAMEDICA S.A	Instituto de Salud Publica de Chile	Alejandra Acevedo; Andrés E Castillo; Bárbara Parra; Carolina Tambley; Gabriel Leal; Jaime Lagos; Jorge Fernandez; Loredana Arata; Patricia Bustos; Paz Tapia; Rodrigo Fasce; Winston Andrade
EPI_ISL_492980 to 492987	IRCCS Sacro Cuore Don Calabria Hospital, Department of Infectious, Tropical Diseases & Microbiology	University of Verona, Department of Biotechnology	Antonio Mori; Chiara Degli Esposti; Chiara Piubelli; Cristina Beltrami; Elena Pomari; Emanuela Cosentino; Giulia Lopatriello; Luca Marcolungo; Massimo Delledonne; Michela Deiana
EPI_ISL_1496000	IRCCS San Raffaele	INMI Lazzaro Spallanzani IRCCS	A Di Caro; B Bartolini; CEM Gruber; D Limongi; D Russo; E Giombini; F Ciatti; F Messina; F Santini; G Bonfiglio; M Rueca; MR Capobianchi; O Butera
EPI_ISL_480961, EPI_ISL_480975, EPI_ISL_480981, EPI_ISL_480989, EPI_ISL_480995, EPI_ISL_481003, EPI_ISL_481017, EPI_ISL_481025, EPI_ISL_481029, EPI_ISL_481034 to 481035			
see above	ISGlobal, Institut de Salut Global de Barcelona	SeqCOVID-SPAIN consortium/IBV(CSIC)	Alberto L Garcia-Basteiro; Alfredo Mayor; Carlota Dobaño; Gemma Moncunill; Pau Cisteró and SeqCOVID-SPAIN consortium
EPI_ISL_1465857	ITHACA COLLEGE LABORATORY HAMMOND HEALTH	Wadsworth Center, New York State Department of Health	Alexis Russell; Catharine Prussing; Daryl M. Lamson; Erasmus Schneider; Erica Lasek-Nesselquist; John Kelly; Jonathan Plitnick; Kirsten St. George;

	CENTER		Matthew Shudt; Melissa A Leisner; Navjot Singh
EPI_ISL_572320 to 572324, EPI_ISL_584069, EPI_ISL_584071 to 584072	IZSM	IZSM	Giovanna Fusco; Lorena Cardillo; Maurizio Viscardi
EPI_ISL_1390377 to 1390394, EPI_ISL_1390396 to 1390545, EPI_ISL_1390547 to 1390680, EPI_ISL_1390682 to 1390689, EPI_ISL_1390716 to 1390730, EPI_ISL_1396408, EPI_ISL_1447677 to 1447690, EPI_ISL_1447692 to 1447925, EPI_ISL_1509300 to 1509309, EPI_ISL_1509311 to 1509636			
see above	IZSM	TIGEM	Andrea Ballabio; Anna Manfredi; Antonio Grimaldi; Antonio Grimaldi Patrizia Annunziata Francesco Panariello Biancamaria Pierri Claudia Tiberio Valentina Bouche Chiara Colantuono Maria Concetta Cuomo Denise Di Concilio Lucio Di Filippo Anna Manfredi Marcello Salvi Antonio Limone Luigi Atripaldi Pellegrino Cerino Andrea Ballabio Davide Cacchiarelli; Antonio Limone Luigi Atripaldi Pellegrino Cerino; Biancamaria Pierri Claudia Tiberio Valentina Bouche; Chiara Colantuono; Davide Cacchiarelli; Denise Di Concilio; Francesco Panariello; Lucio Di Filippo; Marcello Salvi; Maria Concetta Cuomo; Patrizia Annunziata
EPI_ISL_1434418, EPI_ISL_1434420, EPI_ISL_1434422, EPI_ISL_1434424, EPI_ISL_1434426, EPI_ISL_1434428, EPI_ISL_1434430, EPI_ISL_1434432, EPI_ISL_1434434, EPI_ISL_1434436, EPI_ISL_1434438, EPI_ISL_1434440, EPI_ISL_1434442, EPI_ISL_1434445, EPI_ISL_1434447, EPI_ISL_1434449, EPI_ISL_1434451, EPI_ISL_1434453, EPI_ISL_1434455, EPI_ISL_1434457, EPI_ISL_1434459 to 1434460, EPI_ISL_1434462, EPI_ISL_1434464, EPI_ISL_1434466, EPI_ISL_1434468, EPI_ISL_1434470, EPI_ISL_1434472, EPI_ISL_1434474, EPI_ISL_1434476, EPI_ISL_1434478, EPI_ISL_1434480, EPI_ISL_1434482, EPI_ISL_1434484, EPI_ISL_1434486, EPI_ISL_1434488, EPI_ISL_1434490 to 1434491, EPI_ISL_1434493, EPI_ISL_1434495, EPI_ISL_1434497, EPI_ISL_1434499, EPI_ISL_1434501, EPI_ISL_1434503, EPI_ISL_1434505, EPI_ISL_1434507, EPI_ISL_1434509, EPI_ISL_1434511, EPI_ISL_1434513, EPI_ISL_1434515, EPI_ISL_1434517, EPI_ISL_1434519, EPI_ISL_1434522 to 1434523, EPI_ISL_1434525, EPI_ISL_1434527, EPI_ISL_1434529, EPI_ISL_1434531, EPI_ISL_1434533, EPI_ISL_1434535, EPI_ISL_1434537, EPI_ISL_1434539, EPI_ISL_1434541, EPI_ISL_1434543, EPI_ISL_1434545, EPI_ISL_1434547, EPI_ISL_1434549, EPI_ISL_1434551, EPI_ISL_1434553, EPI_ISL_1434555, EPI_ISL_1434557 to 1434558, EPI_ISL_1434560, EPI_ISL_1434562, EPI_ISL_1434564 to 1434566, EPI_ISL_1434569, EPI_ISL_1434571, EPI_ISL_1434573, EPI_ISL_1434575, EPI_ISL_1434577, EPI_ISL_1434579 to 1434580, EPI_ISL_1434583, EPI_ISL_1434585, EPI_ISL_1434587, EPI_ISL_1434589, EPI_ISL_1434591, EPI_ISL_1434593, EPI_ISL_1434595, EPI_ISL_1434597, EPI_ISL_1434599, EPI_ISL_1434602, EPI_ISL_1434604, EPI_ISL_1434606, EPI_ISL_1434608, EPI_ISL_1434610, EPI_ISL_1434612, EPI_ISL_1434614, EPI_ISL_1434616, EPI_ISL_1434618, EPI_ISL_1434621, EPI_ISL_1434623, EPI_ISL_1434625, EPI_ISL_1434627 to 1434630, EPI_ISL_1434632, EPI_ISL_1434634, EPI_ISL_1434636, EPI_ISL_1434638, EPI_ISL_1434640, EPI_ISL_1434643 to 1434644, EPI_ISL_1434646, EPI_ISL_1434648, EPI_ISL_1434650, EPI_ISL_1434652, EPI_ISL_1434654, EPI_ISL_1434656, EPI_ISL_1434658, EPI_ISL_1434660, EPI_ISL_1434662, EPI_ISL_1434665, EPI_ISL_1434667, EPI_ISL_1434669 to 1434670, EPI_ISL_1434673, EPI_ISL_1434675, EPI_ISL_1434677, EPI_ISL_1434679 to 1434680, EPI_ISL_1434682, EPI_ISL_1434684, EPI_ISL_1434687, EPI_ISL_1434689, EPI_ISL_1434691, EPI_ISL_1434693, EPI_ISL_1434695, EPI_ISL_1434697, EPI_ISL_1434699, EPI_ISL_1434701, EPI_ISL_1434703, EPI_ISL_1434705, EPI_ISL_1434707, EPI_ISL_1434709, EPI_ISL_1434711, EPI_ISL_1434713, EPI_ISL_1434715, EPI_ISL_1434717, EPI_ISL_1434719, EPI_ISL_1434722, EPI_ISL_1434724, EPI_ISL_1434726, EPI_ISL_1434728, EPI_ISL_1434730, EPI_ISL_1434732, EPI_ISL_1434734, EPI_ISL_1434736, EPI_ISL_1434738 to 1434741, EPI_ISL_1434743, EPI_ISL_1434745, EPI_ISL_1434747, EPI_ISL_1434749, EPI_ISL_1434751, EPI_ISL_1434753 to 1434754, EPI_ISL_1434756, EPI_ISL_1434759 to 1434760, EPI_ISL_1434762 to 1434764, EPI_ISL_1434766, EPI_ISL_1434768, EPI_ISL_1434770, EPI_ISL_1434772, EPI_ISL_1434774, EPI_ISL_1434776, EPI_ISL_1434779, EPI_ISL_1434781 to 1434782, EPI_ISL_1434784, EPI_ISL_1434786, EPI_ISL_1434788, EPI_ISL_1434790, EPI_ISL_1434792, EPI_ISL_1434794, EPI_ISL_1434797, EPI_ISL_1434799, EPI_ISL_1434801, EPI_ISL_1434803 to 1434804, EPI_ISL_1434806, EPI_ISL_1434808, EPI_ISL_1434811, EPI_ISL_1434813, EPI_ISL_1434815, EPI_ISL_1434817, EPI_ISL_1434819, EPI_ISL_1434821, EPI_ISL_1434823, EPI_ISL_1434825, EPI_ISL_1434827, EPI_ISL_1434830, EPI_ISL_1434832 to 1434833, EPI_ISL_1434836, EPI_ISL_1434838, EPI_ISL_1434840, EPI_ISL_1434842, EPI_ISL_1434844, EPI_ISL_1434846, EPI_ISL_1434848, EPI_ISL_1434850, EPI_ISL_1434852, EPI_ISL_1434854 to 1434855, EPI_ISL_1434858, EPI_ISL_1434860, EPI_ISL_1434862, EPI_ISL_1434864, EPI_ISL_1434866, EPI_ISL_1434868, EPI_ISL_1434870, EPI_ISL_1434872, EPI_ISL_1434875 to 1434876, EPI_ISL_1434878, EPI_ISL_1434880, EPI_ISL_1434882, EPI_ISL_1434885, EPI_ISL_1434887, EPI_ISL_1434889, EPI_ISL_1434891, EPI_ISL_1434893, EPI_ISL_1434895, EPI_ISL_1434897, EPI_ISL_1434899, EPI_ISL_1434901, EPI_ISL_1434903, EPI_ISL_1434905, EPI_ISL_1434907, EPI_ISL_1434909, EPI_ISL_1434912, EPI_ISL_1434914, EPI_ISL_1434916, EPI_ISL_1434918, EPI_ISL_1434920, EPI_ISL_1434922, EPI_ISL_1434924, EPI_ISL_1434926, EPI_ISL_1434928, EPI_ISL_1434930, EPI_ISL_1434932, EPI_ISL_1434934, EPI_ISL_1434936, EPI_ISL_1434938, EPI_ISL_1434940, EPI_ISL_1434942, EPI_ISL_1434944, EPI_ISL_1434946, EPI_ISL_1434949 to 1434950, EPI_ISL_1434952, EPI_ISL_1434954, EPI_ISL_1434956, EPI_ISL_1434959, EPI_ISL_1434961, EPI_ISL_1434963, EPI_ISL_1434965, EPI_ISL_1434967, EPI_ISL_1434969, EPI_ISL_1434971, EPI_ISL_1434973, EPI_ISL_1434975, EPI_ISL_1434977, EPI_ISL_1434979, EPI_ISL_1434981, EPI_ISL_1434983, EPI_ISL_1434985, EPI_ISL_1434987, EPI_ISL_1434990, EPI_ISL_1434992, EPI_ISL_1434994, EPI_ISL_1434996, EPI_ISL_1434998, EPI_ISL_1435000, EPI_ISL_1435002, EPI_ISL_1435004, EPI_ISL_1435006, EPI_ISL_1435008, EPI_ISL_1435010, EPI_ISL_1435012, EPI_ISL_1435015, EPI_ISL_1435017, EPI_ISL_1435019, EPI_ISL_1435021, EPI_ISL_1435024, EPI_ISL_1435026, EPI_ISL_1435028, EPI_ISL_1435030, EPI_ISL_1435032, EPI_ISL_1435034, EPI_ISL_1435036, EPI_ISL_1435038, EPI_ISL_1435040, EPI_ISL_1435043, EPI_ISL_1435045, EPI_ISL_1435047 to 1435048, EPI_ISL_1435051, EPI_ISL_1435053, EPI_ISL_1435056, EPI_ISL_1435058, EPI_ISL_1435060, EPI_ISL_1435062, EPI_ISL_1435064, EPI_ISL_1435066 to 1435069, EPI_ISL_1435071, EPI_ISL_1435073, EPI_ISL_1435075, EPI_ISL_1435077, EPI_ISL_1435079, EPI_ISL_1435081, EPI_ISL_1435083, EPI_ISL_1435085, EPI_ISL_1435088, EPI_ISL_1435090, EPI_ISL_1435092, EPI_ISL_1435094, EPI_ISL_1435096, EPI_ISL_1435098, EPI_ISL_1435100, EPI_ISL_1435102, EPI_ISL_1435104, EPI_ISL_1435106, EPI_ISL_1435108, EPI_ISL_1435110, EPI_ISL_1435112, EPI_ISL_1435114, EPI_ISL_1435116, EPI_ISL_1435119, EPI_ISL_1435121, EPI_ISL_1435123, EPI_ISL_1435125, EPI_ISL_1435127, EPI_ISL_1435129, EPI_ISL_1435131, EPI_ISL_1435133, EPI_ISL_1435135, EPI_ISL_1435137, EPI_ISL_1435139, EPI_ISL_1435141, EPI_ISL_1435143, EPI_ISL_1435145, EPI_ISL_1435147, EPI_ISL_1435149 to 1435152, EPI_ISL_1435155 to 1435156, EPI_ISL_1435158, EPI_ISL_1435161, EPI_ISL_1435163, EPI_ISL_1435165, EPI_ISL_1435167, EPI_ISL_1435169, EPI_ISL_1435171, EPI_ISL_1435173, EPI_ISL_1435175, EPI_ISL_1435177, EPI_ISL_1435179, EPI_ISL_1435181, EPI_ISL_1435183, EPI_ISL_1435185, EPI_ISL_1435187, EPI_ISL_1435189, EPI_ISL_1435191, EPI_ISL_1435193, EPI_ISL_1435195, EPI_ISL_1435198, EPI_ISL_1435200, EPI_ISL_1435202, EPI_ISL_1435204, EPI_ISL_1435206, EPI_ISL_1435208, EPI_ISL_1435210, EPI_ISL_1435212, EPI_ISL_1435214, EPI_ISL_1435217 to 1435220, EPI_ISL_1435223 to 1435224, EPI_ISL_1435227, EPI_ISL_1435229, EPI_ISL_1435231, EPI_ISL_1435233, EPI_ISL_1435235, EPI_ISL_1435237, EPI_ISL_1435239, EPI_ISL_1435241, EPI_ISL_1435243, EPI_ISL_1435245, EPI_ISL_1435247, EPI_ISL_1435249, EPI_ISL_1435251, EPI_ISL_1435253, EPI_ISL_1435256, EPI_ISL_1435258, EPI_ISL_1435260, EPI_ISL_1435262, EPI_ISL_1435264, EPI_ISL_1435266, EPI_ISL_1435268, EPI_ISL_1435270			
see above	Ibaraki Prefectural Institute of Public Health	Ibaraki Prefectural Institute of Public Health	Keiko Goto; Kentaro Itokawa; Makoto Kuroda; Masanori Hashino; Rina Tanaka; Tsuyoshi Sekizuka
EPI_ISL_479936 to 479943, EPI_ISL_479985, EPI_ISL_480021 to 480029, EPI_ISL_480180 to 480189, EPI_ISL_1433287, EPI_ISL_1433289, EPI_ISL_1433291, EPI_ISL_1433375, EPI_ISL_1433377, EPI_ISL_1433379, EPI_ISL_1433381, EPI_ISL_1433383, EPI_ISL_1433385, EPI_ISL_1433387, EPI_ISL_1433389, EPI_ISL_1433546, EPI_ISL_1433548, EPI_ISL_1433550, EPI_ISL_1433552, EPI_ISL_1433554, EPI_ISL_1433556, EPI_ISL_1433558, EPI_ISL_1433560, EPI_ISL_1433562, EPI_ISL_1433564, EPI_ISL_1433566, EPI_ISL_1433568, EPI_ISL_1433570, EPI_ISL_1433573, EPI_ISL_1433575, EPI_ISL_1433577 to 1433578, EPI_ISL_1433581, EPI_ISL_1433583, EPI_ISL_1433585, EPI_ISL_1433587, EPI_ISL_1433589, EPI_ISL_1433591, EPI_ISL_1433593, EPI_ISL_1433595 to 1433596, EPI_ISL_1433598, EPI_ISL_1433600, EPI_ISL_1433602, EPI_ISL_1433604, EPI_ISL_1433606, EPI_ISL_1433609, EPI_ISL_1433611, EPI_ISL_1433613, EPI_ISL_1433615 to 1433616, EPI_ISL_1433618, EPI_ISL_1433620, EPI_ISL_1433622, EPI_ISL_1433624, EPI_ISL_1433626, EPI_ISL_1433628, EPI_ISL_1433630, EPI_ISL_1433632, EPI_ISL_1433635 to 1433636, EPI_ISL_1433639, EPI_ISL_1433641, EPI_ISL_1433643, EPI_ISL_1433645, EPI_ISL_1433647 to 1433648, EPI_ISL_1433650, EPI_ISL_1433652, EPI_ISL_1433654, EPI_ISL_1433656, EPI_ISL_1433658, EPI_ISL_1433660, EPI_ISL_1433663 to 1433664, EPI_ISL_1433667			
see above	Ibaraki Prefectural Institute of Public Health	Pathogen Genomics Center, National Institute of Infectious Diseases	Hajime Kamiya; Keiko Goto; Kentaro Itokawa; Makoto Kuroda; Masanori Hashino; Motoi Suzuki; Rina Tanaka; Tsuyoshi Sekizuka
EPI_ISL_1405711 to 1405728, EPI_ISL_1492620 to 1492636, EPI_ISL_1510260 to 1510277, EPI_ISL_1532066 to 1532088	Idaho Bureau of Laboratories	Idaho Bureau of Laboratories	"R. Beukelman; Aimee Ceniseros; Christopher Ball; Christopher Ball"; Matthew Charles Burns; R. Beukelman; Robert L. Voermans
EPI_ISL_1443290	Il Department of Veterans Affairs	Illinois Department of Public Health - Springfield Lab	Bryan Sim; Gordon McCall
EPI_ISL_1418313	Ilfov County Emergency Clinical Hospital	National Institute of Infectious Diseases-Prof. Dr. Matei Bals Molecular Diagnostics Laboratory	Corina Casangiu; Dan Otelea; Ionelia Nicolae; Leontina Banica; Marius Surleac; Ovidiu Vlaicu; Simona Paraschiv
EPI_ISL_1494032 to 1494549	Illinois Department of Public Health	Gagnon Lab, Southern Illinois University	Keith Gagnon
EPI_ISL_420789 to 420790	Illinois Department of Public Health Chicago Laboratory	Pathogen Discovery, Respiratory Viruses Branch, Division of Viral Diseases, Centers for Disease Control and Prevention	Alison S. Laufer Halpin; Anne Uehara; Christopher A. Elkins; Clinton R. Paden; Haibin Wang; Jasmine Padilla; Jing Zhang; Justin Lee; Krista Queen; Mary S. Keckler; Rachel Marine; Suxiang Tong; Yan Li; Ying Tao
EPI_ISL_1478927	Illinois State University	Illinois Department of Public Health - Springfield Lab	Bryan Sim; Gordon McCall
EPI_ISL_1553244	Illinois Valley Community Hospital	Illinois Department of Public Health - Springfield Lab	Bryan Sim; Gordon McCall
EPI_ISL_1464601 to 1464619, EPI_ISL_1525254, EPI_ISL_1525259 to 1525260	Imeda Hospital	Imelda Hospital	Dagmar Obbels; Hanne Valgaeren; Johan Frans
EPI_ISL_528746	Immanuel Hospital	Molecular Genetics Laboratory-Faculty of Medicine-Universitas Padjadjaran; School of Life Sciences and Technology & School of Pharmacy-Institut Teknologi Bandung; Laboratorium Kesehatan Provinsi Jawa Barat	Agung Eru Wibowo; Azzania Fibriani; Catur Riani; Cut Nur Cinthia Alamanda; Ema Rahmawati; Fensi Amalina; Hammam Riza; Hesti Lina Wiraswati; Husna Nugrahapraja; Irvan Faizal; Lia Faridah; Marselina Irasonia Tan; Mas Rizky A.A. Syamsunarno; Rifky Waluyajati Rachman; Ryan Bayusantika Ristandi; Savira Ekawardhani; Sony Solistia Wirawan; Tarwadi; Tri Hanggono Achmad; Yunia Sribudiani
EPI_ISL_481110 to 481205	Immunogenomics lab, Institute of Life Sciences, Bhubaneswar	Immunogenomics lab, Institute of Life Sciences, Bhubaneswar	Aditi Chatterjee; Ajay Parida; Ajit Singh; Amol Ratnakar Suryawanshi; Ankita Datey; Arup Ghosh; Atimukta Jha; Auromira Khuntia; Bharati Singh; DBT's PAN-INDIA 1000 SARS-CoV2 RNA genome sequencing consortium; Deepika Singh; Dileep Vasudeva; Dileep Vasudevan; Ghulam Hussain Syed; Manasi Priyadarshini; Naga Jogayya Kothakota; Neha Singh; Orissa COVID-19 Study Group; P. Sushree Shyamli; Punit Prasad; Rahul Das; Rajeeb Swain; Rupesh Dash; Shanti Senapati; Sivaram Krishna; Sneha Dutta; Soma Chattopadhyay; Soumyajit Ghosh; Sunil Raghav; Swati Madhulika; Tsheten Sheroa; Tushar K. Beuria; Viplov K. Biswas
EPI_ISL_475572	Imperial College London	Imperial College London	Jie Zhou; Wendy Barclay
EPI_ISL_1491362	Imágenes Diagnósticas de Mazatlán	Microbial Genomics Laboratory	Alejandra Garcia-Gasca; Bruno Gomez-Gil; Daniel Fregoso-Rueda; Julissa Enciso-Ibarra
EPI_ISL_1517387 to 1517434	Inciensa, Instituto Costarricense de Investigación y Enseñanza en Nutrición y Salud	Inciensa, Instituto Costarricense de Investigación y Enseñanza en Nutrición y Salud	Barboza-Arguedas E & Blanco-Delgado C; Barboza-Arguedas E & Centeno-Miranda M; Barboza-Arguedas E & Jimenez-Mora A; Barboza-Arguedas E & Kellerman-Montero K; Cristian Peréz-Corrales; Cristian Peréz-Corrales & Barboza-Arguedas E; Cristian Peréz-Corrales & Valeria Peralta-Barquero; Valeria Peralta-Barquero & Blanco-Delgado C; Valeria Peralta-Barquero & Centeno-Miranda MJ; Valeria Peralta-Barquero & César Cerdas Quesada; Valeria Peralta-Barquero & Gallegos-Carrillo B; Valeria Peralta-Barquero & Kellerman-Montero K; Valeria Peralta-Barquero & Mauricio Bolaños; Valeria Peralta-Barquero & Patricia Salas; Valeria Peralta-Barquero & Salas-Abarca P
EPI_ISL_476702 to 476704	Incubadora Venezolana de Ciencia, Venezuela	Incubadora Venezolana de Ciencia, Venezuela / Instituto Nacional de Salud, Bogotá, Colombia / Grupo de Investigaciones Microbiológicas-UR (GIMUR), Departamento de Biología, Facultad de Ciencias Naturales, Universidad del Rosario, Bogotá, Colombia / Icahn School of Medicine at Mount Sinai, New York, USA	Alberto Paniz-Mondolfi; Ana S. Gonzalez-Reiche; Angelica Rico; Aníbal A. Teherán; Carolina Florez; Carolina Hernández; Emilia Mia Sordillo; Esther C. Barros; Harm van Bakel; Jesús E. Jaimes; Juan David Ramírez; Lisseth Pardo; Lourdes Delgado; Luis Perez-Garcia; Marina Muñoz; Matthew M. Hernandez; Sergio Gomez; Viviana Simon

EPI_ISL_413522	Indian Council of Medical Research - National Institute of Virology	National Influenza Center, Indian Council of Medical Research - National Institute of Virology	Choudhary ML; Potdar V; Shete-Aich A; Yadav PD
EPI_ISL_413523	Indian Council of Medical Research-National Institute of Virology	National Influenza Center, Indian Council of Medical Research-National Institute of Virology	Choudhary ML; Potdar V; Shete-Aich A; Yadav PD
EPI_ISL_508287 to 508337	Indian Institute of Science	National Institute of Biomedical Genomics	Amit Singh; Arindam Maitra; Bharath K Sundararaj; Deepak K Saini; Harsha Raheja; N. Srinivasan; Saumitra Das
EPI_ISL_511945 to 511954, EPI_ISL_518031 to 518032	Indian Institute of Science	National Institute of Biomedical Genomics - DBT's PAN-INDIA 1000 SARS--CoV-2 RNA Genome Sequencing Consortium	Amit Singh; Arindam Maitra; Bharath K Sundararaj; Deepak K Saini; Harsha Raheja; N. Srinivasan; Saumitra Das
EPI_ISL_1525002 to 1525018	Indiana State Department of Health	Quantigen Biosciences	Paul J Childress
EPI_ISL_1401566	Indiana State Health Department	Quantigen Biosciences	Paul J Childress
EPI_ISL_413518 to 413521	Infectious Disease Control Center, Center for Disease Control and Prevention of PLA	Infectious Disease Control Center, Center for Disease Control and Prevention of PLA	Li; Li, J.; Li, L.; Li, Z.; P. and Li, P.; Qiu, S.; Song, H.
EPI_ISL_596454	Infectious Disease and Tropical Medicine Research Center, Resistant Tuberculosis Institute, Zahedan University of Medical Sciences, Zahedan, Iran.	Genetics Research Center, University of Social Welfare and Rehabilitation Sciences	Ali Jafarpour; Azam Ghaziasadi; Ebrahim Kord; Hossein Najmabadi; Khadijeh Jalalvand; Kimia Kahrizi; Marzieh Mohseni; Seyed Mohammad Hashemi-Shahri; Seyed Mohammad Jazayeri; Seyedeh elham Mortazavi; Zohreh Fattahi
EPI_ISL_1502814 to 1502817, EPI_ISL_1503143	Infectious Diseases Department , Instituto Nacional de Ciencias Medicas y Nutrición	Instituto Nacional de Ciencias Medicas y Nutricion Infectious Diseases	Diana Paola Davalos Martinez; Fernando Arteaga Cabello; Fernando Ledesma Barrientos; Guillermo M. Ruiz-Palacios; Luis Alberto Garcia Andrade; Luz Elena Cervantes Villar; Miriam Arciniega Fuentes; Pilar Ramos Cervantes; Violeta Ibarra Gonzalez
EPI_ISL_1418316	Infectious Diseases Hospital Galati	National Institute of Infectious Diseases-Prof. Dr. Matei Bals Molecular Diagnostics Laboratory	Corina Casangiu; Dan Otelea; Ionelia Nicolae; Leontina Banica; Marius Surleac; Ovidiu Vlaicu; Simona Paraschiv
EPI_ISL_538514 to 538521	Infectious Diseases, North Carolina State Laboratory of Public Health COVID-19 Response Team	Infectious Diseases, North Carolina State Laboratory of Public Health COVID-19 Response Team	Chase, K.
EPI_ISL_538522 to 538523	Infectious Diseases, North Carolina State Laboratory of Public Health COVID-19 Response Team	North Carolina State Laboratory of Public Health	Chase, K.
EPI_ISL_496339 to 496376	Infectolab	Andersen lab at Scripps Research	Carlos A. Cota Haros; Octavio Renteria Pacheco; SEARCH Alliance San Diego with Samuel Navarro Alvarez
EPI_ISL_1512746 to 1513138	Infinity Biologix	Centers for Disease Control and Prevention Division of Viral Diseases, Pathogen Discovery	Adrian Paskey; Benjamin Rambo-Martin; Chirayu Goswami; Christian Bixby; Christopher Gulvick; Clinton R. Paden; Dakota Howard; Darlene Wagner; Dhwani Batra; Duncan MacCannell; Jason Caravas; Jonathan Schultz; Kara Moser; Matthew Schmerer; Peter W. Cook; Robin Grimwood; Russ Hager; Scott Sammons; Shatavia Morrison; Yihe Wang; Yvette Unoarumhi
EPI_ISL_541970	Influenza Centre, University of Bergen	Norwegian Institute of Public Health, Department of Virology	Bjørn Blomberg; Fan Zhou; Hilde Elshaug; Hilde Synnøve Vollan; Kamilla Heddeland Instefjord; Karl A Brokstad; Karoline Bragstad; Kathrine Stene-Johansen; Olav Hungnes; Rasmus Riis Kopperud; Rebecca J Cox
EPI_ISL_1448424	Influenza Virus Research Center, National Institute of Infectious Diseases	Influenza Virus Research Center, National Institute of Infectious Diseases	Hideka Miura; Kentaro Itokawa; Kiyoko Okamoto; Kumiko Araki; Makoto Kuroda; Masanori Hashino; Rina Tanaka; Seiichiro Fujisaki; Shinichiro Hirai; Tsuyoshi Sekizuka
EPI_ISL_470900 to 470904	Influenza etiology and epidemiology laboratory	Pathogenic Microorganisms Variability Laboratory	Alexander Gintsburg; Alexey Shchetinin; Andrey Botikov; Anna Ignatjeva; Daria Grousova; Denis Logunov; Elena Burtseva; Elena Shidlovskaya; Evgeniya Mukasheva; Inna Dolzhikova; Kirill Krasnoslobotsev; Ludmila Kolobukhina; Maria Nikiforova; Nadezhda Kuznetsova; Svetlana Smetanina; Svetlana Trushakova; Vladimir Gushchin
EPI_ISL_469057	Inger Landgren	The Public Health Agency of Sweden	Anna Risberg; Anna-Malin Linde; Karin Tegmark-Wisell; Maria Lind Karlberg; Mattias Haukland; Olov Svartstrom; Oskar Karlsson Lindsjo; Petra Edquist; Reza Advani; Sandra Broddesson; Shamam Muradrasoli
EPI_ISL_1395812 to 1395839	Inmunología del Hospital Perrando e Instituto de Medicina Regional de la UNNE	Grupo de Genómica y Bioinformática del Instituto de Investigación de la Cadena Láctea CONICET-INTA on behalf of 'Proyecto Argentino Interinstitucional de genomica de SARS-CoV-2' (PAIS Consortium)	AF; Amadio; Antonieta Cayré; Eberhardt; Gerardo Deluca; Gustavo Giusiano; Horacio Lucero; Irazoqui; Laura Lescano; MF; Marcelo Marin; María Delia Foussal; María Verónica Gómez; Natalia Andrea Ayala
EPI_ISL_1490193, EPI_ISL_1490200 to 1490201, EPI_ISL_1490265, EPI_ISL_1490294, EPI_ISL_1490298, EPI_ISL_1490396, EPI_ISL_1490402, EPI_ISL_1490426, EPI_ISL_1490441, EPI_ISL_1490541, EPI_ISL_1490548, EPI_ISL_1490583, EPI_ISL_1490624, EPI_ISL_1490648, EPI_ISL_1547644 to 1547645			
see above	Innlandet Hospital Trust, Division Lillehammer, Department for Medical Microbiology	Norwegian Institute of Public Health, Department of Virology	Atiya R Ali; Debech Nadia; Engebretsen Serina Beate; Garcia Llorente Ignacio; Hilde Elshaug; Hilde Vollan; Jon Bråte; Kamilla Heddeland Instefjord; Karoline Bragstad; Kathrine Stene-Johansen; Marie Paulsen Madsen; Olav Hungnes; Pedersen Benedikte Nevjen; Rasmus Riis Kopperud
EPI_ISL_467946 to 467949, EPI_ISL_495122 to 495160, EPI_ISL_511805 to 511851, EPI_ISL_511862, EPI_ISL_569887 to 569948, EPI_ISL_576518 to 576556	Innovative Genomics Institute, UC Berkeley	Innovative Genomics Institute, UC Berkeley	Haridha Shivram; Justin Choi; Liana Lareau; Phil Frankino; Shana McDevitt; Stacia Wyman
EPI_ISL_466648	Innovative Genomics Institute, UCB	Innovative Genomics Institute, UCB	Haridha Shivram; Justin Choi; Liana Lareau; Shana McDevitt; Stacia Wyman
EPI_ISL_418215	Instirut Pasteur Dakar	Institut Pasteur de Dakar	Amadou Alpha Sall; Ndongo Dia; Ousmane Faye
EPI_ISL_1396810 to 1396811	Institut Louis MalardÃ©	Department of Virology, Henri Mondor University Hospital, Assistance Publique Hôpitaux de Paris, Université Paris-Est Créteil, INSERM U955	Alexandre Soulier; Christophe Rodriguez; Elisabeth Trawinski; Guillaume Gricourt; Jean-Michel Pawlotsky; Melissa N'Debi; Slim Fourati; Vanessa Demontant
EPI_ISL_1404475 to 1404476	Institut Louis Malardé	Department of Virology, Henri Mondor University Hospital, Assistance Publique Hôpitaux de Paris, Université Paris-Est Créteil, INSERM U955	Alexandre Soulier; Christophe Rodriguez; Elisabeth Trawinski; Guillaume Gricourt; Jean-Michel Pawlotsky; Melissa N'Debi; Slim Fourati; Vanessa Demontant
EPI_ISL_416498, EPI_ISL_428355 to 428357	Institut Médico légal- Hop R. Poincaré	National Reference Center for Viruses of Respiratory Infections, Institut Pasteur, Paris	Angela Brisebarre; Etienne Simon-Lorière; Flora Donati; Marion Barbet; Maud Vanpeene; Mélanie Albert; Méline Bizard; Mélnie Albert; Sylvie Behillil; Sylvie van der Werf; Vincent Enouf
EPI_ISL_1404615, EPI_ISL_1433132, EPI_ISL_1434443, EPI_ISL_1435763, EPI_ISL_1436817, EPI_ISL_1437749, EPI_ISL_1438551, EPI_ISL_1439647, EPI_ISL_1440879			
see above	Institut National d'Hygiène	Unité Mixte Internationale TransVIHMI (UMI 233 IRD - U1175 INSERM - Université de Montpellier) IRD (Institut de recherche pour le développement)	Abla A. KONOU; Adodo SADJI; Ahidjo AYOUBA; Akoélé SILIADIN; Alassane OURO-MEDELI; Amivi EHLAN; Améyo DORKENOO; Anoumou DAGNRA; Christelle BUTEL; Déléma MABA; Eric DELAPORTE; Issaka Maman; Kokou TEGUENI; Laetitia SERRANO; Martine PEETERS; Messanh DOUFFAN; Mireille PRINCE-DAVID; Mounerou SALOU; Sidonie A.M.KAGNISSODE; Sika DOSSIM; Wembo A. HALATOKO
EPI_ISL_1415293, EPI_ISL_1415425, EPI_ISL_1415428, EPI_ISL_1416165, EPI_ISL_1416192, EPI_ISL_1416222, EPI_ISL_1442993, EPI_ISL_1443000 to 1443003			
see above	Institut National d'hygiène	"Unité Mixte Internationale TransVIHMI (UMI 233 IRD - U1175 INSERM - Université de Montpellier) IRD (Institut de recherche pour le développement)"	Abla A. KONOU; Adodo SADJI; Ahidjo AYOUBA; Akoélé SILIADIN; Alassane OURO-MEDELI; Amivi EHLAN; Améyo DORKENOO; Anoumou DAGNRA; Christelle BUTEL; Déléma MABA; Eric DELAPORTE; Issaka Maman; Kokou TEGUENI; Laetitia SERRANO; Martine PEETERS; Messanh DOUFFAN; Mireille PRINCE-DAVID; Mounerou SALOU; Sidonie A.M.KAGNISSODE; Sika DOSSIM; Wembo A. HALATOKO
EPI_ISL_1508953, EPI_ISL_1508958, EPI_ISL_1508960, EPI_ISL_1508988, EPI_ISL_1508998, EPI_ISL_1509002, EPI_ISL_1509095, EPI_ISL_1509097, EPI_ISL_1509099, EPI_ISL_1509101			
see above	Institut National d'hygiène	Unité Mixte Internationale TransVIHMI (UMI 233 IRD - U1175 INSERM - Université de Montpellier) IRD (Institut de recherche pour le développement)	Abla A. KONOU; Adodo SADJI; Ahidjo AYOUBA; Akoélé SILIADIN; Alassane OURO-MEDELI; Amivi EHLAN; Améyo DORKENOO; Anoumou DAGNRA; Christelle BUTEL; Déléma MABA; Eric DELAPORTE; Issaka Maman; Kokou TEGUENI; Koku AGBODEKA; Laetitia SERRANO; Martine PEETERS; Messanh DOUFFAN; Mireille PRINCE-DAVID; Mounerou SALOU; Sidonie A.M.KAGNISSODE; Sika DOSSIM; Syntyche DEVATCHAGNI; Wembo A. HALATOKO
EPI_ISL_1406144, EPI_ISL_1406177, EPI_ISL_1445275, EPI_ISL_1500948,	Institut National d'Hygiène (INH)	Unité Mixte Internationale TransVIHMI (UMI 233 IRD - U1175 INSERM - Université de Montpellier)IRD (Institut de recherche	Abla A. KONOU; Adodo SADJI; Ahidjo AYOUBA; Akoélé SILIADIN; Alassane OURO-MEDELI; Amivi EHLAN; Améyo DORKENOO; Anoumou DAGNRA; Christelle BUTEL; Déléma MABA; Eric DELAPORTE; Issaka Maman; Kokou TEGUENI; Laetitia SERRANO; Martine PEETERS; Messanh DOUFFAN;

EPI_ISL_1501268		pour le développement)	Mireille PRINCE-DAVID; Mounerou SALOU; Sidonie A.M.KAGNISSODE; Sika DOSSIM; Wembo A. HALATOKO
EPI_ISL_485712	Institut Pasteur	Institut Pasteur de Dakar	Amadou Alpha Sall.; Mamadou Malado Jallow; Mamadou diop; Marie Henriette Dior Ndione; Moussa Moise Diagne; Ndongo Dia; Ousmane Faye; Safietou Sanke
EPI_ISL_418206 to 418214, EPI_ISL_418216 to 418217, EPI_ISL_420069 to 420074, EPI_ISL_420076 to 420079, EPI_ISL_476148 to 476151, EPI_ISL_476491 to 476495, EPI_ISL_476497, EPI_ISL_476514 to 476516, EPI_ISL_476558, EPI_ISL_476560, EPI_ISL_476562, EPI_ISL_476564, EPI_ISL_476569 to 476570, EPI_ISL_476572, EPI_ISL_476574, EPI_ISL_477141 to 477159, EPI_ISL_480554, EPI_ISL_480556, EPI_ISL_480782 to 480783, EPI_ISL_480786 to 480789, EPI_ISL_481220, EPI_ISL_481234 to 481240, EPI_ISL_481243, EPI_ISL_482874 to 482878, EPI_ISL_485635, EPI_ISL_485708, EPI_ISL_485710 to 485711, EPI_ISL_485713, EPI_ISL_485715 to 485717, EPI_ISL_486857 to 486873			
see above	Institut Pasteur Dakar	Institut Pasteur de Dakar	Amadou Alpha Sall; Amadou Alpha Sall.; Amadou Alpha sall; Amadou alpha Sall; Mamadou Diop; Mamadou Malado Jallow; Mamadou diop; Mamadou malado Jallow; Marie Henriette Dior Ndione; Moussa Moise; Moussa Moise Diagne; Ndongo Dia; Ousmane Faye; Ousmane faye; Safietou Sanke; Safietou Sankhe; mamadou Diop
EPI_ISL_498229 to 498252	Institut Pasteur de Dakar	Institut Pasteur de Dakar	Amadou Alpha Sall.; Mamadou Diop; Mamadou Malado Jallow; Marie Henriette Dior Ndione; Moussa Moise Diagne; Ndongo Dia; Ousmane Faye; Safietou Sankhe Mbengue
EPI_ISL_613419 to 613456	Institut Pasteur de la Guadeloupe	Institut Pasteur de la Guadeloupe	Angela Brisebarre; Antoine Talarmin; Camille Capel; Cherina Fleming; Etienne Simon-Lorière; Marion Barbet; Maud Vanpeene; Méline Bizard; Radjin Steingrover; Stéphanie Guyomard; Sylvie Behillil; Sylvie van der Werf; Sébastien Breurec; Vincent Enouf
EPI_ISL_459965 to 459984	Institut Pasteur du Maroc	Institut Pasteur du Maroc	Abdellah Faouzi; Anass Abbad; Anderrahmane Maaroufi; Angela Brisebarre; Camille Capel; Etienne Simon-Lorière; Jalal Nourlil; Latifa Anga; Marion Barbet; Maud Vanpeene; Mjid Eloualid; Méline Bizard; Sylvie Behillil; Sylvie van der Werf; Vincent Enouf
EPI_ISL_1403088	Institut de Pathologie et Genetique (IPG)	Institut de Pathologie et Genetique (IPG)	Jérémie Gras; Pascale Hilbert
EPI_ISL_416747 to 416748, EPI_ISL_416750, EPI_ISL_416753 to 416754, EPI_ISL_416756, EPI_ISL_416758	Institut des Agents Infectieux (IAI) Hospices Civils de Lyon	CNR Virus des Infections Respiratoires - France SUD	Alexandre; Antonin; Bal; Bouscambert-Duchamp; Brengel-Pesce; Bruno.; Cheynet; Destras; Florence; Gaymard; Gregory; Josset; Karen; Laurence; Lina; Martine; Maude; Morfin-Sherpa; Valette; Valérie
EPI_ISL_417333 to 417337, EPI_ISL_417339, EPI_ISL_418420 to 418425, EPI_ISL_418429 to 418432, EPI_ISL_419169 to 419173, EPI_ISL_419177 to 419182, EPI_ISL_419184, EPI_ISL_420604 to 420611, EPI_ISL_420615 to 420616, EPI_ISL_420618 to 420619, EPI_ISL_420621 to 420625, EPI_ISL_508871 to 508874, EPI_ISL_508877, EPI_ISL_508882 to 508911, EPI_ISL_509007 to 509011, EPI_ISL_509013 to 509014			
see above	Institut des Agents Infectieux (IAI), Hospices Civils de Lyon	CNR Virus des Infections Respiratoires - France SUD	Alexandre Gaymard; Antonin Bal; Bruno Lina; Carine Moustaud; Florence Morfin-Sherpa; Gregory Destras; Gwendolyne Burfin; Laurence Josset; Martine Valette; Maude Bouscambert-Duchamp; Raphaelle Lamy; Solenne Brun
EPI_ISL_1439748 to 1439749	Institut für Medizinische Mikrobiologie - Universitätsmedizin Göttingen	Robert Koch Institute	
EPI_ISL_1489931	Institut für Medizinische Virologie, Universitätsklinikum Frankfurt	Charité Universitätsmedizin Berlin, Institute of Virology	Barbara Mühlemann; Christian Drosten; Christiane Pallas; Julia Schneider; Julia Tesch; Jörn Beheim-Schwarzbach; Marek Widera; Niko Kohmer; Sandra Ciesek; Talitha Veith; Terry Jones; Tobias Bleicker; Tuna Toptan Grabmair; Victor M Corman
EPI_ISL_437913 to 437932, EPI_ISL_437974 to 437992, EPI_ISL_475763, EPI_ISL_475769, EPI_ISL_475813 to 475829, EPI_ISL_475916 to 475928, EPI_ISL_583572, EPI_ISL_583600 to 583616, EPI_ISL_583691			
see above	Institut für Virologie am Department für Hygiene, Mikrobiologie und Public Health	Bergthaler laboratory, CeMM Research Center for Molecular Medicine of the Austrian Academy of Sciences	Adi Steinrigl; Alexander Lercher; Alexandra Popa; Andreas Bergthaler; Benedikt Agerer; Christian Paar; Christoph Bock; Daniela Schmid; Dorothee von Laer; Elisabeth Puchhammer-Stoeckl; Franz Allerberger; Gernot Walder; Gregor Hörmann; Guenter Weiss; Gunther Vogl; Henrique Colaco; Jakob-Wendelin Genger; Jan Laine; Judith Aberle; Kinga Rigler-Hohenwarter; Lukas Endler; Manfred Nairz; Mark Smyth; Martin Senekowitsch; Michael Schuster; Peter Hufnagl; Peter Obrist; Rainer Gattringer; Sabine Sussitz-Rack; Stephan Aberle; Thomas Penz; Wegene Borena
EPI_ISL_485809 to 485813, EPI_ISL_508687 to 508704	Institut für Virologie und Epidemiologie der Viruskrankheiten, Universitätsklinikum Tübingen	NGS Competence Center Tübingen, Institut für Medizinische Mikrobiologie und Hygiene, Universitätsklinikum Tübingen	Angel Angelov; Angelov at al.; Angelov et al.
EPI_ISL_420075, EPI_ISL_476566	Institut pasteur Dakar	Institut Pasteur de Dakar	Amadou Alpha Sall; Mamadou Diop; Moussa Moise Diagne; Ndongo Dia; Ousmane Faye
EPI_ISL_1498126 to 1498127, EPI_ISL_1498131 to 1498132, EPI_ISL_1498136 to 1498141, EPI_ISL_1498145, EPI_ISL_1498149 to 1498151	Institute for Developing Science and Health Initiatives (ideSHi)	Institute for Developing Science and Health Initiatives (ideSHi)	Fidausi Qadri; Hassan Afrad; Sadia Rahman; Tahmina Shirin
EPI_ISL_574790 to 574862	Institute for Infectious Diseases, University of Bern	Institute for Infectious Diseases, University of Bern	Alban Ramette; Christian Baumann; Cora Sägesser; Franziska Suter-Riniker; Michel C Koch; Miguel A Terrazos Miani; Peter Keller; Stephen L Leib
EPI_ISL_583853 to 583868	Institute for Laboratory Diagnostics and Microbiology, Klinikum Klagenfurt am Worthersee	Bergthaler laboratory, CeMM Research Center for Molecular Medicine of the Austrian Academy of Sciences	Adi Steinrigl; Alexander Lercher; Alexandra Popa; Andreas Bergthaler; Benedikt Agerer; Christian Paar; Christoph Bock; Daniela Schmid; Dorothee von Laer; Elisabeth Puchhammer-Stoeckl; Franz Allerberger; Gernot Walder; Gregor Hörmann; Guenter Weiss; Gunther Vogl; Henrique Colaco; Jakob-Wendelin Genger; Jan Laine; Judith Aberle; Kinga Rigler-Hohenwarter; Lukas Endler; Manfred Nairz; Mark Smyth; Martin Senekowitsch; Michael Schuster; Peter Hufnagl; Peter Obrist; Rainer Gattringer; Sabine Sussitz-Rack; Stephan Aberle; Thomas Penz; Wegene Borena
EPI_ISL_1495274 to 1495285, EPI_ISL_1495354 to 1495361, EPI_ISL_1495543 to 1495550, EPI_ISL_1495801 to 1495809	Institute for Laboratory Diagnostics and Microbiology, Klinikum Klagenfurt am Wörthersee	Bergthaler laboratory, CeMM Research Center for Molecular Medicine of the Austrian Academy of Sciences	Andreas Bergthaler; Anna Schedl; Bekir Erguner; Benedikt Agerer; Christoph Bock; Fabian Amman; Jan Laine; Lukas Endler; Maelle Le Moing; Martin Senekowitsch; Michael Schuster; Petr Triska; Thomas Penz
EPI_ISL_455790 to 455793	Institute for Medical Research, Infectious Disease Research Centre, National Institutes of Health, Ministry of Health Malaysia	Malaysia Genome Institute	Azrin Ahmad; Enizza Kasim; Irni Suhayu Sapian; Jeyanthi Suppiah; Mohd Faizal Abu Bakar; Mohd Noor Mat Isa; Norazah Ahmad; Nurhezreen Md Iqbal; Ravindran Thayan; Shahrul Hisham Zainal Ariffin; Shamsidar Sopie; Siti Noraini Othman; Tahir Aris; Yusuf Muhammad Noor; Zarina Mohd Zawawi
EPI_ISL_430439	Institute for Medical Research, Infectious Disease Research Centre, National Institutes of Health, Ministry of Health Malaysia	Institute for Medical Research Infectious Disease Research Centre, National Institutes of Health, Ministry of Health Malaysia	Abdul-Wahid.M-Z; Ahmad.N; Azizan.M-A; Hisham.H-A; Kalyanasundram.J; Mat-Sharani.S; Mohd-Zain.R; Mohd-Zawawi.Z; Suppiah.J; Tan.L-P; Thayan.R
EPI_ISL_459957	Institute for Medical Research, Infectious Disease Research Centre, National Institutes of Health, Minis	Institute for Medical Research, Infectious Disease Research Centre, National Institutes of Health, Minis	Ellan K; Kalyanasundram J; Kamel KA; Mohd-Zain R; Mohd-Zawawi Z; Suppiah J; Thayan R
EPI_ISL_459953	Institute for Medical Research, Infectious Disease Research Centre, National Institutes of Health, Ministry of Health Malaysia	Institute for Medical Research Infectious Disease Research Centre, National Institutes of Health, Ministry of Health Malaysia	Ellan K; Kalyanasundram J; Kamel KA; Mohd-Zain R; Mohd-Zawawi Z; Suppiah J; Thayan R
EPI_ISL_430440 to 430444, EPI_ISL_459954 to 459956, EPI_ISL_489992 to 489994, EPI_ISL_490014 to 490016, EPI_ISL_490047 to 490048, EPI_ISL_490089 to 490103, EPI_ISL_596449, EPI_ISL_1405966, EPI_ISL_1406078, EPI_ISL_1406146, EPI_ISL_1406148, EPI_ISL_1406153, EPI_ISL_1406178, EPI_ISL_1406185, EPI_ISL_1406187, EPI_ISL_1406192, EPI_ISL_1406194, EPI_ISL_1406197, EPI_ISL_1406199, EPI_ISL_1406201, EPI_ISL_1406204, EPI_ISL_1406242, EPI_ISL_1406244, EPI_ISL_1406247, EPI_ISL_1406250, EPI_ISL_1406274, EPI_ISL_1406277, EPI_ISL_1406281, EPI_ISL_1406286, EPI_ISL_1406292, EPI_ISL_1406294, EPI_ISL_1424064 to 1424065, EPI_ISL_1424130, EPI_ISL_1424143, EPI_ISL_1424466 to 1424468, EPI_ISL_1424470 to 1424471, EPI_ISL_1424510, EPI_ISL_1424513, EPI_ISL_1424518			
see above	Institute for Medical Research, Infectious Disease Research Centre, National Institutes of Health, Ministry of Health Malaysia	Institute for Medical Research, Infectious Disease Research Centre, National Institutes of Health, Ministry of Health Malaysia	Abdul-Wahid.M-Z; Ahmad.N; Azizan.M-A; Ellan K; Hisham.H-A; Kalyanasundram J; Kalyanasundram.J; Kamel K; Kamel KA; Mat-Sharani.S; Mohd Zawawi Z; Mohd-Zain R; Mohd-Zain.R; Mohd-Zawawi Z; Mohd-Zawawi.Z; Ramly N; Robert F; Suppiah J; Suppiah.J; Tan.L-P; Tengku-Abd-Rashid.T-R; Tengku-Rogayah.TAR; Thayan R; Thayan.R
EPI_ISL_583579 to 583598	Institute for Medical and Chemical Laboratory Diagnostics, Kepler Universitätsklinikum	Bergthaler laboratory, CeMM Research Center for Molecular Medicine of the Austrian Academy of Sciences	Adi Steinrigl; Alexander Lercher; Alexandra Popa; Andreas Bergthaler; Benedikt Agerer; Christian Paar; Christoph Bock; Daniela Schmid; Dorothee von Laer; Elisabeth Puchhammer-Stoeckl; Franz Allerberger; Gernot Walder; Gregor Hörmann; Guenter Weiss; Gunther Vogl; Henrique Colaco; Jakob-Wendelin Genger; Jan Laine; Judith Aberle; Kinga Rigler-Hohenwarter; Lukas Endler; Manfred Nairz; Mark Smyth; Martin Senekowitsch; Michael Schuster; Peter Hufnagl; Peter Obrist; Rainer Gattringer; Sabine Sussitz-Rack; Stephan Aberle; Thomas Penz; Wegene Borena
EPI_ISL_454605 to 454606, EPI_ISL_455566 to 455567, EPI_ISL_468591, EPI_ISL_468656	Institute for Public Health	Laboratory for advanced genomics	Filip Roki; Igor Jurak; Lovro Trgovec-Greif; Neven Sui; Oliver Vugrek; Tomislav Rukavina
EPI_ISL_492075 to 492086	Institute for Public Health of the Republic of North Macedonia	Charite Universitatsmedizin Berlin, Institute of Virology	Barbara Muhlemann; Christian Drosten; Elizabeta Jancheska; Golubinka Bosevska; Joern Beheim-Schwarzbach; Julia Schneider; Maja Kuzmanovska; Talitha Veith; Terry Jones; Victor M Corman
EPI_ISL_477168, EPI_ISL_477205 to 477246, EPI_ISL_477248 to 477251, EPI_ISL_477255 to 477262, EPI_ISL_479736 to 479755, EPI_ISL_480293 to 480296, EPI_ISL_486673			

see above	Institute for Stem Cell Science and Regenerative Medicine	National Centre for Biological Sciences	Aswin Seshasayee; Awadhesh Pandit; Dasaradhi Palakodeti; Farhan Ali; Mohak Sharda; Shah-e-Jahan Gulzar; Srikar Krishna; Uma Ramakrishnan; Vanessa Molin Paynter; Varadha Sundarmurthy
EPI_ISL_1491531 to 1491554, EPI_ISL_1491570 to 1491571, EPI_ISL_1491574, EPI_ISL_1511124 to 1511138, EPI_ISL_1538418 to 1538419, EPI_ISL_1538421 to 1538428	Institute for Urban Disease Control and Prevention	COVID-19 Network Investigations (CONI) Alliance	Amornmas Kongklieng; Angkana Huang; Anthony R. Jones; Arporn Wangwiwatsin; Bhakbhoom Panthan; Chonticha Klungtong; Duangkamon Loesbanluechai; Ekawat Pasomsub; Elizabeth Batty; Insee Sensorn; Janjira Thaipadungpanit; Kamolthip Atsawawaranunt; Khajohn Joonlasak; Kingkan Rakmanee; Krittikorn Kumpornsin; Namfon Kotanan; Prayuth Kaewmalang; Pukkaporn Parnwijitkul; Stefan Fernandez; Thanat Chookajorn; Theerarat Kochakarn; Treewat Watthanachockchai; Vichan Pawun; Wasun Chantratita; Wudtichai Manasatienkij
EPI_ISL_572330 to 572331, EPI_ISL_572333, EPI_ISL_572397, EPI_ISL_573152, EPI_ISL_574259	Institute for Virology, University Hospital Duesseldorf, Medical Faculty, Heinrich-Heine-University Duesseldorf	Institute for Virology, University Hospital Duesseldorf, Medical Faculty, Heinrich-Heine-University Duesseldorf	; Alexander Killer; Andreas Walker; Annemarie Mohring; Anselm Kunstein; Ansgar Schulz; Björn Jensen; Caroline Klindt; Edwin Bölke; Gerald Antoch; Heiner Schaal; Jennifer Neubert; Johannes Bode; Johannes C. Fischer; Jörg Timm; Lisa Müller; Maximilian Damagnez; Nadine Lübke; Ortwin Adams; Philipp Albrecht; Philipp Ostermann; Saskia Elben; Tina Senff; Tom Lüdde; Torsten Feldt; Verena Keitel
EPI_ISL_602462 to 602509, EPI_ISL_602518 to 602532	Institute for Virology, University Hospital Essen	Center of Medical Microbiology, Virology, and Hospital Hygiene, University of Duesseldorf	Alexander Dilthey; Andreas Walker; Daniel Strelow; Jessica Nicolai; Jörg Timm; Klaus Pfeffer; Lisanna Hülse; Malte Kohns Vasconcelos; Maximilian Damagnez; Nadine Lübke; Olympia E. Anastasiou; Tobias Wienemann; Torsten Houwaart; Ulf Dittmer
EPI_ISL_1495272 to 1495273, EPI_ISL_1495384 to 1495406, EPI_ISL_1495551 to 1495573, EPI_ISL_1495867 to 1495893	Institute for Water Quality and Resource Management, Technical University Vienna	Bergthaler laboratory, CeMM Research Center for Molecular Medicine of the Austrian Academy of Sciences	Andreas Bergthaler; Anna Schedl; Bekir Erguner; Benedikt Agerer; Christoph Bock; Fabian Amman; Jan Laine; Lukas Endler; Maelle Le Moing; Martin Senekowitsch; Michael Schuster; Petr Triska; Thomas Penz
EPI_ISL_418988	Institute information KU Leuven, Clinical and Epidemiological Virology	Institute information KU Leuven, Clinical and Epidemiological Virology	Bert Vanmechelen; Joan Marti-Carreras; Piet Maes; Tony Wawina
EPI_ISL_525472 to 525473	Institute of Clinical Microbiology and Hygiene, University Hospital Regensburg	Institute of Clinical Microbiology and Hygiene, University Hospital Regensburg	Hiergeist, A.
EPI_ISL_539333 to 539339	Institute of Disease Control and Prevention, People's Liberation Army	Institute of Disease Control and Prevention, People's Liberation Army	Li, P.; Qiu, S.
EPI_ISL_600428 to 600446, EPI_ISL_600449, EPI_ISL_600451, EPI_ISL_600454, EPI_ISL_600456, EPI_ISL_600459, EPI_ISL_600461, EPI_ISL_600463, EPI_ISL_600466, EPI_ISL_600468, EPI_ISL_600471, EPI_ISL_600473, EPI_ISL_600476, EPI_ISL_600478, EPI_ISL_600480, EPI_ISL_600484, EPI_ISL_600487, EPI_ISL_600489, EPI_ISL_600492, EPI_ISL_600495, EPI_ISL_600498, EPI_ISL_600500, EPI_ISL_600502, EPI_ISL_600505, EPI_ISL_600507, EPI_ISL_600510, EPI_ISL_600512, EPI_ISL_600515, EPI_ISL_600517, EPI_ISL_600519, EPI_ISL_600522, EPI_ISL_600524, EPI_ISL_600527, EPI_ISL_600530, EPI_ISL_600533, EPI_ISL_600535, EPI_ISL_600538, EPI_ISL_600541, EPI_ISL_600543, EPI_ISL_600546, EPI_ISL_600549, EPI_ISL_600552, EPI_ISL_600554, EPI_ISL_600557, EPI_ISL_600559, EPI_ISL_600562, EPI_ISL_600564, EPI_ISL_600567, EPI_ISL_600570			
see above	Institute of Epidemiology Disease Control And Research	Institute for Developing Science and Health Initiatives	Firadausi Qadri; Lauren Cowley; Md. Mahfuz-Al-mamun; Mokibul Hassan Afrad; Sadia Isfat Ara Rahman; Tahmina Shirin
EPI_ISL_1498128 to 1498130, EPI_ISL_1498133 to 1498135, EPI_ISL_1498142 to 1498144, EPI_ISL_1498146 to 1498148, EPI_ISL_1498152	Institute of Epidemiology, Disease Control and Research (IEDCR)	Institute for Developing Science and Health Initiatives (ideSHi)	Fidausi Qadri; Hassan Afrad; Sadia Rahman; Tahmina Shirin
EPI_ISL_462480, EPI_ISL_485399, EPI_ISL_582031 to 582032	Institute of Human Genetics, Polish Academy of Sciences	Institute of Human Genetics, Polish Academy of Sciences	Adam Ustaszewski; Andrzej Pawski; Emilia Lis; Ewa Zitkiewicz; Marta Kaczmarek-Ry; Micha Witt; Szymon Hryhorowicz
EPI_ISL_485400	Institute of Human Genetics, Polish Academy of Sciences	Institute of Human Genetics, Polish Academy of Sciences,	Adam Ustaszewski; Andrzej Pawski; Emilia Lis; Ewa Zitkiewicz; Marta Kaczmarek-Ry; Micha Witt; Szymon Hryhorowicz
EPI_ISL_450294	Institute of Human Genetics, Polish Academy of Sciences Sanitary and Epidemiological Station in Pozna	Institute of Human Genetics, Polish Academy of Sciences	Adam Ustaszewski; Andrzej Pawski; Emilia Lis; Marta Kaczmarek-Ry; Micha Witt; Szymon Hryhorowicz
EPI_ISL_450295	Institute of Human Genetics, Polish Academy of Sciences; Sanitary and Epidemiological Station in Pozna	Institute of Human Genetics, Polish Academy of Sciences	Adam Ustaszewski; Andrzej Pawski; Emilia Lis; Marta Kaczmarek-Ry; Micha Witt; Szymon Hryhorowicz
EPI_ISL_450338	Institute of Human Genetics, Polish Academy of Sciences; Sanitary and Epidemiological Station in Pozna	Institute of Human Genetics, Polish Academy of Sciences	Adam Ustaszewski; Andrzej Pawski; Emilia Lis; Marta Kaczmarek-Ry; Micha Witt; Szymon Hryhorowicz
EPI_ISL_463010 to 463093	Institute of Life Sciences, Bhubaneswar	Immunogenomics lab, Institute of Life Sciences, Bhubaneswar	Ajay Parida; Aliva Minz; Arup Ghosh; Atimukta Jha; Avula Kiran; DBT's PAN-INDIA 1000 SARS-CoV2 RNA genome sequencing consortium; Debyashrita Barik; Deepak Singh; Eshna Laha; Ghulam Hussain Syed; Hiren G. Dodia; ILS COVID-19 TEAM; Jeky Chawla; Kaushik Sen; Kautilya Kumar Jena; Manasi Priyadarshini; Neha Singh; O. P. Shriwas; Orissa COVID-19 Study Group; Parej Nath; Priyanka Mohapatra; Punit Prasad; Rajeeb Swain; Rina Yadav; Rupesh Dash; Saiket De; Sanchari Chatterjee; Sandhya Suranjika; Satya Ranjan Sahu; Shamima Ansari; Shanti Senapati; Shuchi Smita; Sifu Agarwal; Soma Chattopadhyay; Sunil Raghav; Supriya Suman; Swati Madhulika; Tushar K. Beuria; Viplov K. Biswas
EPI_ISL_527871	Institute of Medical Biology, Chinese Academy of Medical Sciences and Peking Union Medical College	Institute of Medical Biology, Chinese Academy of Medical Sciences and Peking Union Medical College	Chen, H.; Fan, S.; G. and Li, Q.; Jiang; Liao, Y.; Liu, L.; Wang, L.; Xie, Z.; Xu, X.; Zheng, H.; Zhou, X.
EPI_ISL_590823 to 590836	Institute of Medical Virology, University of Zurich	Institute of Medical Virology, University of Zurich	Alexandra Trkola; Benjamin G. Hale; Idoia Busnadiego; Irene Abela; Marie O. Pohl; Maryam Zaheri; Michael Huber; Silke Stertz; Stefan Schmutz; Verena Kufner
EPI_ISL_422563 to 422565	Institute of Microbiology Universidad San Francisco de Quito	Institute of Microbiology Universidad San Francisco de Quito	Alejandra Ramones; Belen Prado-Vivar; Bernardo Gutierrez; Edison Ligña; Francisco Mora; Franklin Espinoza; Gabriel Trueba; Jorge Reyes; Juan Gaviria; Juan Jose Guadalupe; Michelle Grunauer; Patricio Rojas-Silva; Paul Cardenas; Sully Marquez; Veronica Barragan
EPI_ISL_420294 to 420295, EPI_ISL_420541, EPI_ISL_1385787 to 1385788, EPI_ISL_1403338 to 1403383, EPI_ISL_1403387 to 1403421, EPI_ISL_1403427 to 1403496, EPI_ISL_1406287 to 1406290, EPI_ISL_1406434, EPI_ISL_1406436, EPI_ISL_1490187, EPI_ISL_1495062 to 1495110, EPI_ISL_1496003, EPI_ISL_1498920 to 1499019, EPI_ISL_1499021 to 1499104, EPI_ISL_1509888 to 1509922, EPI_ISL_1510936 to 1511114, EPI_ISL_1516890			
see above	Institute of Microbiology and Immunology, Faculty of Medicine, University of Ljubljana	Institute of Microbiology and Immunology, Faculty of Medicine, University of Ljubljana	Alen Sulji; Andraž Celar; Dominika Šturm; Doroteja Vlaj; Lucijan Skubic; Mario Poljak; Matic Brvar; Miša Korva; Patricija Pozvek; Samo Zakotnik; Tatjana Avši - Županc; Tatjana Avši - Županc; Tomaž Mark Zorec; Špela Pleh
EPI_ISL_548942 to 548946	Institute of Microbiology, University of Veterinary and Animal sciences	Institute of Microbiology, University of Veterinary and Animal sciences	Ali; Altaf, I.; Anwar, M.; Ashraf; Asif, A.; Attique; Awan; Aziz; Bhatti; Cheema; Fazal, S.; Hassan, S.; Khan; Khan, N.; M.A.; M.B.; M.M.; M.S.; M.T.; M.U.; M.W.; M.Z.; Mehmood, A.; Mukhtar, N.; N.A.; Nawaz, M.; Rafique, S.; Rana; Raza, S.; S.Q.; S.Z.; Sardar, N.; Sarwar, H.; Shabbir; Shah; Tahir, Z.; Yaqub, T.; Younis
EPI_ISL_417482, EPI_ISL_477014 to 477016, EPI_ISL_486842 to 486851, EPI_ISL_491932, EPI_ISL_491936 to 491940, EPI_ISL_516648 to 516652, EPI_ISL_525430 to 525438, EPI_ISL_527809 to 527817, EPI_ISL_539785 to 539793			
see above	Institute of Microbiology, Universidad San Francisco de Quito	Institute of Microbiology, Universidad San Francisco de Quito	Alejandra Ramones; Andrea Macias; Anita Garcia; Belen Prado-Vivar; Belén Prado-Vivar; Bernardo Gutierrez; Bernardo Gutiérrez; Carla Torres; Carlos Guerrero; Carlos Mena; Carlos Tobar; Carolina Pacheco; Damaris Zandoya; DamarisSandoya; Dayron Brossard; Diego Egas; Eddy Chavez; Edison Ligña; Edy Quizhpe; Eulalia Pazmiño; Fausto Maldonado; Francisco Mora; Francisco Rodriguez; Franklin Espinoza; Gabriel Trueba; Geovanny Carzola; Jonathan Araujo; Jorge Luis Velez; Jorge Reyes; Juan Gaviria; Juan Jose Guadalupe; Juan José Guadalupe; Karina Barragan; Khurram Mahbbob; Ligia Briceño; Manuel Jibaja; Michelle Grunauer; Milton Tobar; Monica Becerra-Wong; Nabih Dahik; Patricio Rojas-Silva; Paul Cardenas; Paúl Cárdenas; Prado-Vivar; Rene Bracho; Rosario Erazo; Stalin Castillo; Stephanie Arregui; Sully Marquez; Sully Márquez; Veronica Barragan; Verónica Barragán; Yomara Napa
EPI_ISL_1443641 to 1443665	Institute of Microbiology, Universidad San Francisco de Quito	Omics Sciences Laboratory	; Andrea Cunguan; Belén Prado-Vivar; Bernardo Gutiérrez; Darlyn Amaya; Dayron Brossad; Derly Andrade Molina; Emily Sulay Saltos Montalvo; Fernanda Zurita; Gabriel Morey León; Gabriel Trueba; Juan Carlos Fernández Cadena; Juan José Guadalupe; Katheryn Sacheri Viteri; Michelle Grunauer; Monica Becerra-Wong; Nabih Dahik; Patricio Rojas-Silva; Paula Juliana Gavilanes Jarrín; Paúl Cárdenas; Rubén Armas González; Sully Márquez; Verónica Barragán
EPI_ISL_463008	Institute of Molecular Virology, University Münster	Institute of Molecular Virology, University Münster	Alexander Mellmann; Angeles Mecate Zambrano; Joachim Kühn; Linda Brunotte; Stephan Ludwig
EPI_ISL_1465438 to 1465481	Institute of Molecular and Translational Medicine / Laboratory of Experimental Medicine, Faculty of Medicine and Dentistry, Palacky University and University Hospital Olomouc	Institute of Molecular and Translational Medicine / Laboratory of Experimental Medicine, Faculty of Medicine and Dentistry, Palacky University	Barbora Blumová; Hana Jaworek; Marián Hajdúch; Rastislav Slavkovský; Tomáš Pospíšil; Tomáš Pospíšil; Vladimíra Koudeláková
EPI_ISL_508338 to 508414	Institute of Post Graduate Medical Education & Research	National Institute of Biomedical Genomics	Arindam Maitra; Aritra Biswas; Jayeeta Haldar; Monimoy Banerjee; Raja Ray; Saumitra Das
EPI_ISL_511900 to 511907	Institute of Post Graduate Medical Education & Research	National Institute of Biomedical Genomics - DBT's PAN-INDIA 1000 SARS--CoV-2 RNA Genome Sequencing Consortium	Arindam Maitra; Aritra Biswas; Jayeeta Haldar; Monimoy Banerjee; Raja Ray; Saumitra Das

EPI_ISL_1499519 to 1499543	Institute of Public Health of Dubrovnik-Neretva County	Croatian Institute of Public Health	Irena Tabain; Ivana Ferenak
EPI_ISL_1499544 to 1499548	Institute of Public Health of Istra County	Croatian Institute of Public Health	Irena Tabain; Ivana Ferenak
EPI_ISL_1499513 to 1499518	Institute of Public Health of Karlovac County	Croatian Institute of Public Health	Irena Tabain; Ivana Ferenak
EPI_ISL_1499549 to 1499562	Institute of Public Health of Koprivnica-Križevci County	Croatian Institute of Public Health	Irena Tabain; Ivana Ferenak
EPI_ISL_1499563 to 1499568	Institute of Public Health of Krapina-Zagorje County	Croatian Institute of Public Health	Irena Tabain; Ivana Ferenak
EPI_ISL_1435946, EPI_ISL_1435948, EPI_ISL_1435950, EPI_ISL_1435952, EPI_ISL_1435954, EPI_ISL_1435956, EPI_ISL_1435958, EPI_ISL_1435960, EPI_ISL_1435962, EPI_ISL_1435964			
see above	Institute of Public Health of Lika-Senj conty	Croatian Institute of Public Health	Irena Tabain; Ivana Ferenak
EPI_ISL_1499505 to 1499512	Institute of Public Health of Meimurje county	Croatian Institute of Public Health	Irena Tabain; Ivana Ferenak
EPI_ISL_1435896, EPI_ISL_1435900, EPI_ISL_1435902, EPI_ISL_1435913, EPI_ISL_1435917, EPI_ISL_1435922, EPI_ISL_1435924, EPI_ISL_1435930, EPI_ISL_1435937, EPI_ISL_1435939, EPI_ISL_1435941 to 1435942, EPI_ISL_1435944, EPI_ISL_1435995, EPI_ISL_1435997, EPI_ISL_1435999 to 1436000, EPI_ISL_1436002, EPI_ISL_1436004, EPI_ISL_1436006, EPI_ISL_1436008, EPI_ISL_1436013, EPI_ISL_1436018, EPI_ISL_1436026, EPI_ISL_1436028, EPI_ISL_1436030, EPI_ISL_1436032, EPI_ISL_1436039, EPI_ISL_1436041, EPI_ISL_1436043, EPI_ISL_1436045 to 1436046			
see above	Institute of Public Health of Varazdin county	Croatian Institute of Public Health	Irena Tabain; Ivana Ferenak
EPI_ISL_577734 to 577742, EPI_ISL_583481 to 583489, EPI_ISL_1492457 to 1492560, EPI_ISL_1547829 to 1547906	Institute of Virology, Biomedical Research Center of the Slovak Academy of Sciences, Bratislava	Faculty of Natural Sciences, Comenius University, Bratislava	Boris Klempa; Brona Brejova; Broa Brejová; Dominika Friová; Edita Staroová; Elena Tichá; Jozef Nosek; Juraj Kopacek; Juraj Kopáek; Kristina Borsova; Kristína Boršová; Lubomira Lukacikova; Martina Lickova; Martina Liková; Martina Nebohacova; Martina Neboháová; Monika Slavikova; Monika Sláviková; Sabina Fumacova Havlikova; Sabina Fumaová Havlíková; Tomas Vinar; Tomáš Vina; Viktoria Cabanova; Viktoria Hodorova; Viktória Hodorová; Viktória abanová; ubomíra Lukáiková
EPI_ISL_417877 to 417880	Institute of Virology, Biomedical Research Center of the Slovak Academy of Sciences, Bratislava; Public Health Authority of the Slovak Republic, Bratislava	Institute of Virology, Biomedical Research Center of the Slovak Academy of Sciences, Bratislava; Comenius University Science Park, Bratislava	Boris Klempa; Diana Rusáková; Edita Staroová; Elena Tichá; Jaroslav Budiš; Juraj Kopáek; Juraj Koi; Martina Liková; Miroslav Böhmer; Monika Slávikova; Sabina Fumaová Havlíková; Tomáš Szemeš; Werner Krampl
EPI_ISL_455680	Institute of pathogenic microbiology, Jiangsu Provincial Center for Disease Control and Prevention	Institute of pathogenic microbiology, Jiangsu Provincial Center for Disease Control and Prevention	Cui, L.
EPI_ISL_1420635	Instituto Estadual do Cérebro Paulo Niemayer (IECPN)	Laboratório de Imunofarmacologia	Thiago Moreno Lopes Souza
EPI_ISL_491174 to 491297	Instituto Gulbenkian de Ciência	Instituto Gulbenkian de Ciência	Cathy Paulino; Joao Sobral; João Costa; Ricardo Leite; Susana Ladeiro
EPI_ISL_426364	Instituto Nacional de Ciencias Medicas y Nutricion Salvador Zubiran	Instituto Nacional de Ciencias Medicas y Nutricion	Adnan Araiza Rodríguez; Alejandro Sánchez; Alfredo Ponce de León Garduño; Blanca Taboada; Carlos F. Arias; Carolina González Torres; Celia Boukadida; Cesar Raúl González Bonilla; Concepción Grajales Muñiz; Edgar Mendieta Condado; Eduardo Becerril Vargas; Fabiola Garcés Ayala; Fernando Ledesma Barrientos; Francisco Javier Gaytán Cervantes; Francisco Pulido; Gisela Barrera Badillo; Gloria Vázquez; Guillermo M. Ruiz-Palacios; Irma López Martínez; Joel Armando Vázquez Pérez; José Arturo Martínez Orozco; José Ernesto Ramírez González; José Esteban Muñoz Medina; Lucia Hernández Rivas; Luis Alberto García Andrade; Mario Mújica Sánchez; Pavel Isa; Pilar Ramos Cervantes; Ricardo Grande; Santiago Avila Ríos; Victor Hugo Borja Aburto; Violeta Ibarra Gonzalez
EPI_ISL_426361 to 426363, EPI_ISL_426365	Instituto Nacional de Ciencias Medicas y Nutricion Salvador Zubiran	Instituto Nacional de Ciencias Medicas y Nutricion Salvador Zubiran	Adnan Araiza Rodríguez; Alejandro Sánchez; Alfredo Ponce de León Garduño; Blanca Taboada; Carlos F. Arias; Carolina González Torres; Celia Boukadida; Cesar Raúl González Bonilla; Concepción Grajales Muñiz; Edgar Mendieta Condado; Eduardo Becerril Vargas; Fabiola Garcés Ayala; Fernando Ledesma Barrientos; Francisco Javier Gaytán Cervantes; Francisco Pulido; Gisela Barrera Badillo; Gloria Vázquez; Guillermo M. Ruiz-Palacios; Irma López Martínez; Joel Armando Vázquez Pérez; José Arturo Martínez Orozco; José Ernesto Ramírez González; José Esteban Muñoz Medina; Lucia Hernández Rivas; Luis Alberto García Andrade; Mario Mújica Sánchez; Pavel Isa; Pilar Ramos Cervantes; Ricardo Grande; Santiago Avila Ríos; Victor Hugo Borja Aburto; Violeta Ibarra Gonzalez
EPI_ISL_424345, EPI_ISL_424348, EPI_ISL_424625 to 424627	Instituto Nacional de Enfermedades Respiratorias	Instituto Nacional de Enfermedades Respiratorias	Adnan Araiza Rodríguez; Alejandro Sánchez; Alfredo Ponce de León Garduño; Blanca Taboada; Carlos F. Arias.; Carolina González Torres; Celia Boukadida; Cesar Raúl González Bonilla; Concepción Grajales Muñiz; Edgar Mendieta Condado; Eduardo Becerril Vargas; Fabiola Garcés Ayala; Fernando Ledesma Barrientos; Francisco Javier Gaytán Cervantes; Francisco Pulido; Gisela Barrera Badillo; Gloria Vázquez; Guillermo M. Ruiz-Palacios; Irma López Martínez; Joel Armando Vázquez Pérez; Jorge Salas Hernández; José Arturo Martínez Orozco; José Ernesto Ramírez González; José Esteban Muñoz Medina; Lucia Hernández Rivas; Luis Alberto García Andrade; Mario Mújica Sánchez; Pavel Isa; Pilar Ramos Cervantes; Ricardo Grande; Santiago Avila Ríos; Victor Hugo Borja Aburto; Violeta Ibarra Gonzalez
EPI_ISL_412972	Instituto Nacional de Enfermedades Respiratorias	Instituto de Diagnostico y Referencia Epidemiologicos (INDRE)	Araiza-Rodriguez Adnan; Arias Carlos; Barrera-Badillo Gisela; Boukadida Celia; Garces-Ayala Fabiola; Hernandez-Rivas Lucia; Isa Pavel; Lopez Susana; Lopez-Martinez Irma; Martinez Arturo; Mendieta-Condado Edgar; Munoz-Medina Esteban; Ramirez-Gonzalez Ernesto; Rodriguez-Maldonado Abril; Sanchez Alejandro; Taboada Blanca; Vazquez-Perez Joel; Wong-Arambula Claudia
EPI_ISL_574294	Instituto Nacional de Investigacion en Salud Pública	Instituto Nacional de Investigación en Salud Pública	Alberto Orlando Narvaez; Alfredo Bruno; Andrés Carrasco; Denisse Portugal; Doménica de Mora Coloma; Leandro Patino Patino; Manuel Gonzalez; Orson Mestanza
EPI_ISL_574292	Instituto Nacional de Investigación en Salud Pública	Instituto Nacional de Investigación en Salud Pública	Alberto Orlando Narvaez.; Alfredo Bruno Caicedo; Andrés Carrasco; Denisse Portugal; Domenica de Mora Coloma; Leandro Patino Patino; Manuel Gonzalez; Orson Mestanza
EPI_ISL_574293	Instituto Nacional de Investigación en Salud Pública	Instituto Nacional de Investigación en Salud Publica	Alberto Orlando Narvaez.; Alfredo Bruno Caicedo; Andrés Carrasco; Denisse Portugal; Doménica de Mora Coloma; Leandro Patino Patino; Manuel Gonzalez; Orson Mestanza
EPI_ISL_491944 to 491954	Instituto Nacional de Investigación en Salud Pública - INSPI	INSPI - Charité	Alberto Orlando; Alexandra Usiña; Alfredo Bruno Caicedo; Andres Moreira-Soto; Anna-Lena Sander; Denisses Portugal; Domenica de Mora Coloma; Jan Felix Drexler; Juan Carlos Zeballos; Manuel Gonzalez; Maritza Olmedo; Nina Krause; Silvia Salgado
EPI_ISL_1545272 to 1545389	Instituto Nacional de Investigação em Saúde	KRISP, KZN Research Innovation and Sequencing Platform	Afonso P; David K; Emmanuel SJ; Freitas RH; Giandhari J; Inglês L; Lutucuta S; Miranda J; Morais J; Mufinda M; Naidoo Y; Neto Z; Paulo A Carralero RR Paixão JP; Pereira A; Pillay S; Tegally H; Wilkinson E; de Oliveira T
EPI_ISL_1406722 to 1406777, EPI_ISL_1423994 to 1424001	Instituto Nacional de Medicina Genomica	Instituto Nacional de Enfermedades Respiratorias (INER), Centro de Investigación en Enfermedades Infecciosas (CIENI)	Arriaga-Canon C; Avila-Rios S; Boukadida C; Cedro-Tanda A; Herrera-Montalvo LA; Hidalgo-Miranda A; Matias-Florentino M; Mendoza-Vargas A; Reyes-Grajeda JP; Reyes-Teran G
EPI_ISL_522873 to 522878, EPI_ISL_1392922, EPI_ISL_1400795 to 1400799, EPI_ISL_1406083 to 1406142, EPI_ISL_1416873, EPI_ISL_1417442, EPI_ISL_1558802 to 1558827			
see above	Instituto Nacional de Medicina Genomica	Instituto Nacional de Medicina Genomica	Alcaraz N; Canseco Mendez JC; Cedro-Tanda A; Cisneros-Villanueva M; Garcia-Cardenas FJ; Garcia-Cárdenas FJ; Gonzalez-Barrera D; Gonzalez-Woge MA; González-Woge MA; Herrera-Montalvo LA; Herrera-Montalvo LA.; Hidalgo-Miranda A; Hurtado-Cordova E; Mendoza-Vargas A; Miranda-Ortiz H; Miranda-Ortíz H; Munguia-Garza; Munguia-Garza P; Peñaloza-Figueroa F; Ramirez-Vega O; Rangel-DeLeon D; Reyes-Grajeda JP; Rosas-Escobar P
EPI_ISL_522872, EPI_ISL_522879 to 522880, EPI_ISL_522940 to 522942, EPI_ISL_522978 to 522986, EPI_ISL_523500	Instituto Nacional de Medicina Genómica	Instituto Nacional de Medicina Genómica	Cedro-Tanda A; Cisneros-Villanueva M; Herrera-Montalvo LA; Hidalgo-Miranda A; Hurtado-Cordova E; Mendoza-Vargas A; Peñaloza-Figueroa F; Reyes-Grajeda JP
EPI_ISL_1557223	Instituto Nacional de Salud	Corporacion Corpogen Universidad de los Andes Universidad Central	Anzola; Chaib De Mares; Christian; Del Portillo; Duitama; Díaz; González; Jorge; Juan Manuel; Laura; Maryam; María Mercedes; Nelly; Patricia; Restrepo; Romero; Silvia; Zambrano
EPI_ISL_418262	Instituto Nacional de Salud	Instituto Nacional de Salud Universidad Cooperativa de Colombia Instituto Alexander von Humboldt Imperial College-London London School of Hygiene & Tropical Medicine	Astrid C. Flórez; Carlos Franco-Muñoz; Christian Julian VillabonaArenas; Diana Marcela Walteros-Acero; Diego A. Álvarez-Díaz; Erika Ospitia; Gloria Puerto; Jose A. Usme-Ciro; Juliana Barbosa; Katherine Laiton-Donato; Liz Villabona-Arenas; Luz Dary Rodriguez; Mailyn A.Gonzalez; Marcela Mercado-Reyes; Martha Lucia Ospina Martinez; Nicolas D. Franco-Sierra; Nuno Rodrigues Faria; Sergio Gomez Rangel; Sussy Echeverria; Zulma M. Cucunubá
EPI_ISL_536477 to 536565	Instituto Nacional de Salud	Laboratorio de Infecciones Respiratorias Agudas	David Tarazona; Dennis Carhuaricra; Eduardo Juscamayta Lopez; Faviola Valdivia Guerrero; Lenin Maturrano Hernandez; Nancy Rojas Serrano; Ronnie Gavilan Chavez
EPI_ISL_456116 to 456156	Instituto Nacional de Salud - Unidad de Secuenciación y Análisis Genómico	Instituto Nacional de Salud, Universidad Cooperativa de Colombia, Instituto Alexander von Humboldt, Imperial	Astrid C. Flórez; Carlos Franco-Muñoz; Christian Julian VillabonaArenas; Diana Marcela Walteros-Acero; Diego A. Álvarez-Díaz; Erika Ospitia; Gloria Puerto; Jose A. Usme-Ciro; Juliana Barbosa; Katherine Laiton-Donato; Liz Villabona-Arenas; Luz Dary Rodriguez; Mailyn A.Gonzalez; Marcela

		College-London, London School of Hygiene & Tropical Medicine	Mercado-Reyes.; Martha Lucia Ospina Martinez; Nicolas D. Franco-Sierra; Sergio Gomez-Rangel; Sussy Echeverria; Zulma M. Cucunubá
EPI_ISL_447755 to 447769, EPI_ISL_447771 to 447772, EPI_ISL_447774 to 447787, EPI_ISL_447789 to 447817	Instituto Nacional de Salud, Bogotá, Colombia	Grupo de Investigaciones Microbiológicas-UR (GIMUR), Departamento de Biología, Facultad de Ciencias Naturales, Universidad del Rosario, Bogotá, Colombia Instituto Nacional de Salud, Bogotá, Colombia Icahn School of Medicine at Mount Sinai, New York, USA	Adriana Castillo; Alberto Paniz-Mondolfi; Ana S. Gonzalez-Reiche; Angelica Rico; Aníbal A. Teherán; Carolina Florez; Carolina Hernandez; David Martínez; Emilia Mia Sordillo; Esther C. Barros; Harm van Bakel; Jesús E. Jaimes; Juan David Ramírez; Laura Vega; Lisseth Pardo; Marina Muñoz; Martha L. Ospina; Matthew M. Hernandez; Nathalia Ballesteros; Sergio Castañeda; Sergio Gomez; Viviana Simon
EPI_ISL_497736, EPI_ISL_497738, EPI_ISL_497744 to 497745, EPI_ISL_498152 to 498170, EPI_ISL_526932 to 526934, EPI_ISL_526949 to 526951, EPI_ISL_526953 to 526965, EPI_ISL_526967 to 526971, EPI_ISL_526973 to 526974			
see above	Instituto Nacional de Salud, Bogotá, Colombia	Instituto Nacional de Salud, Bogotá, Colombia	Astrid C. Flórez; Carlos Andrés Durán; Carlos Franco-Muñoz; Carolina Ferro; Christian Julian VillabonaArenas; Diana Marcela Walteros-Acero; Diego A. Álvarez-Díaz; Diego Andrés Prada; Edmilson F. de Oliveira-Filho; Felix Betzler; Franklin Prieto; Jeadran Malagón-Rojas; Jonathan Reales; Jose A. Usme-Ciro; Katherine Laiton-Donato; Liz Villabona-Arenas; Marcela Mercado-Reyes; Martha Lucia Ospina Martinez; Mauricio Pacheco-Montealegre; Nicolas D. Franco-Sierra; Sussy Echeverria; Wendy K. Jo; Zulma M. Cucunubá
EPI_ISL_1424059 to 1424063, EPI_ISL_1494947	Instituto Nacional de Salud- Dirección de Investigación en Salud Pública	Instituto Nacional de Salud- Dirección de Investigación en Salud Pública	Carlos Franco-Muñoz; Carmen Osorio; Diana Malo; Diego A. Álvarez-Díaz; Diego Andrés Prada; Gerardo Santamaría; Hector Alejandro Ruiz-Moreno; Jhonnatan Reales-González; Juan Camilo Martinez; Julian Naizaque; Katherine Laiton-Donato; Lisseth Pardo; Magdalena Wiesner; Marcela Mercado-Reyes; Marcela Mercado-Reyes.; Maria T. Herrera-Sepúlveda; Marta Lopez Blanco; Martha Lucia Ospina Martinez; Martha Lucia Ospina Martínez; Mauricio Pacheco; Paola Rojas; Sergio Gomez; Sheryll Corchuelo; Ángela Alarcon Cruz
EPI_ISL_421450 to 421452, EPI_ISL_421456, EPI_ISL_421472 to 421478, EPI_ISL_421496 to 421499, EPI_ISL_510889 to 511181, EPI_ISL_511189, EPI_ISL_511316 to 511483, EPI_ISL_511509 to 511576, EPI_ISL_511579 to 511643, EPI_ISL_511645 to 511750, EPI_ISL_1399286 to 1399332, EPI_ISL_1399402, EPI_ISL_1399406 to 1399409, EPI_ISL_1399411, EPI_ISL_1399414 to 1399421, EPI_ISL_1399425 to 1399426, EPI_ISL_1399428 to 1399435, EPI_ISL_1399437, EPI_ISL_1399439 to 1399440, EPI_ISL_1399444 to 1399452, EPI_ISL_1399454 to 1399529, EPI_ISL_1399577 to 1399583, EPI_ISL_1399589 to 1399590, EPI_ISL_1399592 to 1399596, EPI_ISL_1399617 to 1399619, EPI_ISL_1399624 to 1399629, EPI_ISL_1399634 to 1399635, EPI_ISL_1399638 to 1399643, EPI_ISL_1399645 to 1399654, EPI_ISL_1399656 to 1399658, EPI_ISL_1399660 to 1399661, EPI_ISL_1399663 to 1399669, EPI_ISL_1399671 to 1399676, EPI_ISL_1399679, EPI_ISL_1399681 to 1399742, EPI_ISL_1399877 to 1399885, EPI_ISL_1399904, EPI_ISL_1399906, EPI_ISL_1399909, EPI_ISL_1399911 to 1400015, EPI_ISL_1400154 to 1400157, EPI_ISL_1400159 to 1400160, EPI_ISL_1400167 to 1400173, EPI_ISL_1400177 to 1400180, EPI_ISL_1400183, EPI_ISL_1400186 to 1400192, EPI_ISL_1400195 to 1400212, EPI_ISL_1400214 to 1400215, EPI_ISL_1400223, EPI_ISL_1400228 to 1400234, EPI_ISL_1400236 to 1400245, EPI_ISL_1400247, EPI_ISL_1400249 to 1400263, EPI_ISL_1400265, EPI_ISL_1400267 to 1400283, EPI_ISL_1400285, EPI_ISL_1400288 to 1400292, EPI_ISL_1400294 to 1400297, EPI_ISL_1413888, EPI_ISL_1413890, EPI_ISL_1413893, EPI_ISL_1413895, EPI_ISL_1413897, EPI_ISL_1414434, EPI_ISL_1414437			
see above	Instituto Nacional de Saude (INSA)	Instituto Nacional de Saude (INSA)	Borges et al; Guiomar et al
EPI_ISL_511484 to 511508, EPI_ISL_511751 to 511771	Instituto Nacional de Saude (INSA)	Instituto Nacional de Saude (INSA) and Instituto Gulbenkian de Ciencia (IGC)	Borges et al
EPI_ISL_1399282 to 1399283, EPI_ISL_1399333 to 1399342, EPI_ISL_1399404 to 1399405, EPI_ISL_1399438, EPI_ISL_1399442 to 1399443, EPI_ISL_1399453, EPI_ISL_1399530 to 1399543, EPI_ISL_1399584 to 1399588, EPI_ISL_1399591, EPI_ISL_1399597 to 1399612, EPI_ISL_1399620 to 1399623, EPI_ISL_1399630, EPI_ISL_1399633, EPI_ISL_1399659, EPI_ISL_1399743 to 1399794, EPI_ISL_1399886 to 1399891, EPI_ISL_1399899 to 1399903, EPI_ISL_1399910, EPI_ISL_1400016 to 1400055, EPI_ISL_1400161 to 1400164, EPI_ISL_1400174, EPI_ISL_1400213, EPI_ISL_1400216 to 1400222, EPI_ISL_1400224 to 1400227, EPI_ISL_1400246, EPI_ISL_1400248, EPI_ISL_1400266, EPI_ISL_1400284, EPI_ISL_1400293			
see above	Instituto Nacional de Saude (INSA) and BioSystems & Integrative Sciences Institute (BioISI) Genomics Unit, FCUL	Instituto Nacional de Saude (INSA) and BioSystems & Integrative Sciences Institute (BioISI) Genomics Unit, FCUL	Borges et al
EPI_ISL_1399284 to 1399285, EPI_ISL_1399343 to 1399350, EPI_ISL_1399401, EPI_ISL_1399427, EPI_ISL_1399544 to 1399545, EPI_ISL_1399547, EPI_ISL_1399616, EPI_ISL_1399636 to 1399637, EPI_ISL_1399644, EPI_ISL_1399655, EPI_ISL_1399662, EPI_ISL_1399795 to 1399815, EPI_ISL_1399876, EPI_ISL_1399892, EPI_ISL_1400056 to 1400070, EPI_ISL_1400175 to 1400176			
see above	Instituto Nacional de Saude (INSA) and Institute of Biomedicine (iBiMed), Universidade de Aveiro	Instituto Nacional de Saude (INSA) and Institute of Biomedicine (iBiMed), Universidade de Aveiro	Borges et al
EPI_ISL_511182 to 511188, EPI_ISL_511190 to 511315, EPI_ISL_1399351 to 1399400, EPI_ISL_1399403, EPI_ISL_1399410, EPI_ISL_1399412 to 1399413, EPI_ISL_1399422 to 1399424, EPI_ISL_1399436, EPI_ISL_1399441, EPI_ISL_1399548, EPI_ISL_1399550 to 1399576, EPI_ISL_1399613 to 1399615, EPI_ISL_1399631 to 1399632, EPI_ISL_1399670, EPI_ISL_1399677 to 1399678, EPI_ISL_1399680, EPI_ISL_1399816 to 1399875, EPI_ISL_1399893 to 1399898, EPI_ISL_1399905, EPI_ISL_1399907, EPI_ISL_1400071 to 1400153, EPI_ISL_1400158, EPI_ISL_1400165 to 1400166, EPI_ISL_1400181 to 1400182, EPI_ISL_1400184 to 1400185, EPI_ISL_1400193 to 1400194, EPI_ISL_1400235, EPI_ISL_1400264, EPI_ISL_1400286 to 1400287, EPI_ISL_1413899, EPI_ISL_1413902, EPI_ISL_1413904, EPI_ISL_1413907, EPI_ISL_1413909, EPI_ISL_1413912, EPI_ISL_1413914, EPI_ISL_1413917, EPI_ISL_1413919, EPI_ISL_1413922, EPI_ISL_1413925, EPI_ISL_1413927, EPI_ISL_1413929, EPI_ISL_1413932, EPI_ISL_1413935, EPI_ISL_1413937, EPI_ISL_1413940, EPI_ISL_1413943			
see above	Instituto Nacional de Saude (INSA) and Instituto Gulbenkian de Ciencia (IGC)	Instituto Nacional de Saude (INSA) and Instituto Gulbenkian de Ciencia (IGC)	Borges et al
EPI_ISL_1443030 to 1443152, EPI_ISL_1444109, EPI_ISL_1444111 to 1444112, EPI_ISL_1444114 to 1444116, EPI_ISL_1444118 to 1444119, EPI_ISL_1444121 to 1444122, EPI_ISL_1444124 to 1444126, EPI_ISL_1444128 to 1444129, EPI_ISL_1444131 to 1444132, EPI_ISL_1444134 to 1444135, EPI_ISL_1444137 to 1444138, EPI_ISL_1444140 to 1444141, EPI_ISL_1444143 to 1444144, EPI_ISL_1444146 to 1444151, EPI_ISL_1444153 to 1444154, EPI_ISL_1444156 to 1444157, EPI_ISL_1444159 to 1444161, EPI_ISL_1444163 to 1444164, EPI_ISL_1444166			
see above	Instituto Nacional de Saude (INSA) and i3S - Instituto de Investigação e Inovação em Saúde	Instituto Nacional de Saude (INSA) and i3S - Instituto de Investigação e Inovação em Saúde	Borges et al
EPI_ISL_1550388	Instituto Oswaldo Cruz	Laboratorio de Virologia, Faculdade de Medicina, Universidade Federal de Mato Grosso, campus Cuiabá	Janeth Aracely Ramirez Pavon; Luciano Nakazato; María de Fátima Ferreira; Paola Cristina Resende; Renata Dezengrini Slhessarenko; Rosane Christiane Hahn; Valeria Dutra
EPI_ISL_426580	Instituto Sabin	Laboratory of Virology	Bergmann M Ribeiro; Fernando L Melo; Gustavo Barra; Ikaro A Andrade; Pedro G Mesquita; Tatsuya Nagata; Ticiane H Santa-Rita
EPI_ISL_492032 to 492048	Instituto de Biologia do Exército	Laboratório Metabolismo Macromolecular FirminoTorres de Castro, Instituto de Biofísica Carlos Chagas Filho, Universidade Federal do Rio de Janeiro	Aline Rosa Vianna de Souza; Bianca Catarina Azevedo Cabral; Caleb GM Santos; Clarissa Damaso; Elizabeth Valentin; Marcio da Costa Cipitelli; Marcos Dornelas-Ribeiro; Nádia Vaez Gonçalves da Cruz; Rodrigo Soares de Moura Neto; Rosane Silva; Tatiana LS Nogueira; Virginia Sara Grancieri do Amaral
EPI_ISL_424673	Instituto de Diagnostico y Referencia Epidemiologicos	Instituto de Diagnostico y Referencia Epidemiologicos	Adnan Araiza Rodríguez; Alejandro Sánchez; Alfredo Ponce de León Garduño; Blanca Taboada; Carlos F. Arias; Carolina González Torres; Celia Boukadida; Cesar Raúl González Bonilla; Concepción Grajales Muñiz; Edgar Mendieta Condado; Eduardo Becerril Vargas; Fabiola Garcés Ayala; Fernando Ledesma Barrientos; Francisco Javier Gaytán Cervantes; Francisco Pulido; Gisela Barrera Badillo; Gloria Vázquez; Guillermo M. Ruiz-Palacios; Irma López Martínez; Joel Armando Vázquez Pérez; José Arturo Martínez Orozco; José Ernesto Ramírez González; José Esteban Muñoz Medina; Lucia Hernández Rivas; Luis Alberto García Andrade; Mario Mújica Sánchez; Pavel Isa; Pilar Ramos Cervantes; Ricardo Grande; Santiago Avila Ríos; Victor Hugo Borja Aburto; Violeta Ibarra Gonzalez
EPI_ISL_455432, EPI_ISL_455434 to 455439, EPI_ISL_455454 to 455456, EPI_ISL_493334 to 493349, EPI_ISL_516608 to 516625, EPI_ISL_576257 to 576279	Instituto de Diagnostico y Referencia Epidemiologicos (INDRE)	Instituto de Diagnostico y Referencia Epidemiologicos (INDRE)	; Abril Rodriguez-Maldonado; Adnan Araiza-Rodriguez; Araiza-Rodriguez Adnan; Barrera-Badillo Gisela; Claudia Wong-Arambula; Dayanira Arellano-Suarez; Edgar Mendieta-Condado; Ernesto Ramirez-Gonzalez; Ernesto Ramirez-Gonzalez.; Fabiola Garces-Ayala; Garces-Ayala Fabiola; Garces-Ayala Fabiola. Ramirez-Gonzalez Ernesto; Garces-Ayala Fabiola. Taboada Ramírez Blanca. Ramirez-Gonzalez Ernesto; Garces-Ayala Fabiola. Taboada Ramirez Blanca. Ramirez-Gonzalez Ernesto; Garces-Ayala Fabiola. Taboada Ramírez Blanca. Ramirez-Gonzalez Ernesto; Gisela Barrera-Badillo; Gisela Barrera-Badillo.; Hernandez-Rivas Lucia; Irma Lopez-Martinez; Lopez-Martinez Irma; Lopez-Martinez Irma.; Lucia Hernandez-Rivas; Mendieta-Condado Edgar; Natividad Cruz-Ortiz; Ramirez-Gonzalez Ernesto; Ramirez-Gonzalez Ernesto.; Rodriguez-Maldonado Abril; Taboada Ramirez Blanca; Taboada Ramírez Blanca; Taboada Ramírez Blanca.; Taboada Ramírez Blanca. Ramirez-Gonzalez Ernesto; Tatiana Nunez-Garcia; Wong-Arambula Claudia; Wong-Arambula Claudia.
EPI_ISL_452139, EPI_ISL_452141	Instituto de Diagnostico y Referencia Epidemiologicos (INDRE)	Instituto de diagnóstico y Referencia Epidemiologicos (INDRE)	Araiza-Rodriguez Adnan; Barrera-Badillo Gisela; Garces-Ayala Fabiola; Hernandez-Rivas Lucia; Lopez-Martinez Irma; Lopez-Martinez Irma.; Mendieta-Condado Edgar; Ramirez-Gonzalez Ernesto; Rodriguez-Maldonado Abril; Wong-Arambula Claudia
EPI_ISL_424666	Instituto de Diagnóstico y Referencia Epidemiológicos	Instituto de Diagnóstico y Referencia Epidemiológicos	Adnan Araiza Rodríguez; Alejandro Sánchez; Alfredo Ponce de León Garduño; Blanca Taboada; Carlos F. Arias.; Carolina González Torres; Celia Boukadida; Cesar Raúl González Bonilla; Concepción Grajales Muñiz; Edgar Mendieta Condado; Eduardo Becerril Vargas; Fabiola Garcés Ayala; Fernando Ledesma Barrientos; Francisco Javier Gaytán Cervantes; Francisco Pulido; Gisela Barrera Badillo; Gloria Vázquez; Guillermo M. Ruiz-Palacios; Irma López Martínez; Joel Armando Vázquez Pérez; José Arturo Martínez Orozco; José Ernesto Ramírez González; José Esteban Muñoz Medina; Lucia Hernández Rivas; Luis Alberto García Andrade; Mario Mújica Sánchez; Pavel Isa; Pilar Ramos Cervantes; Ricardo Grande; Santiago Avila Ríos; Victor Hugo Borja Aburto; Violeta Ibarra Gonzalez
EPI_ISL_510064 to 510077, EPI_ISL_510461 to 510466	Instituto de Investigaciones Biomédicas de Barcelona (CSIC), Hospital Clinic i Provincial de Barcelona, Instituto de Biomedicina de Valencia (CSIC), Hospital de Sant Pau	SeqCOVID-SPAIN consortium/IBV(CSIC)	Alex Soriano; Andrea Vergara; Anna M. Planas; Israel Fernández Cadenas and SeqCOVID-SPAIN consortium; Jordi Pérez Tur; Miguel J. Martínez; Mª Angeles Marcos

EPI_ISL_1395782 to 1395785, EPI_ISL_1395802 to 1395804	Instituto de Investigaciones Biomédicas en Retrovirus y SIDA (INBIRS)	Área de Secuenciación del Laboratorio de Virología del Hospital de Niños Dr. Ricardo Gutierrez on behalf of 'Proyecto Argentino Interinstitucional de genomica de SARS-CoV-2' (PAIS Consortium)	Acuña; Alexay; D; Federico Remes Lenicov; Goya; Horacio Salomón; LE; Lusso; M; MI; Nabaes Jodar; Natale; S; Valinotto; Vanesa Seery; Viegas, M.
EPI_ISL_1557222	Instituto de Medicina Tropical de Sao Paulo	Instituto de Medicina Tropical de Sao Paulo	Camila Malta Romano; Lucy Santos Vilas Boas; Maria Cassia J Mendes-Correa
EPI_ISL_500864	Instituto de Pesquisas Biomédicas, Hospital Naval Marcílio Dias	Bioinformatics Laboratory / LNCC	Albina Luciana da Silva Freitas; Alexandra Lehmkuhl Gerber; Amilcar Tanuri; Ana Tereza Vasconcelos; Andressa Rangel de Oliveira Lima; Carolina Moreira Voloch; Claudio Alberto Mule Monteiro; Cynthia Chester Cardoso; Fernanda Conceição Silva do Amorim; Isabel de Medeiros Magalhaes de As; Luana Ferreira Martins de Toledo; Luiz Gonzaga Paula de Almeida; Marlon Daniel Lima Tonin; Mônica Gomes Monteiro da R. Santos; Shana Priscila Coutinho Barroso; Átila Duque Rossi
EPI_ISL_499083	Instituto de Virologia "Dr. J. M. Vanella", Facultad de Ciencias Medicas, Universidad Nacional de Cordoba. Laboratorio Central de la Provincia de Cordoba, Argentina. Ministerio de Salud de la provincia de Cordoba, Argentina.	Laboratorio de Virología, Hospital de Niños Ricardo Gutiérrez, CABA, Argentina.	Adrian Diaz; Brenda Konigheim; Gabriela Barbas; Gonzalo Castro; Javier Aguilar; Lorena Spinsanti; Mariana Viegas.; Mercedes Nabaes; Monica Natale; Sandra Gallego; Sebastian Blanco; Silvina Lusso; Stephanie Goya
EPI_ISL_1394086 to 1394091, EPI_ISL_1539095 to 1539096	Instytut "Pomnik-Centrum Zdrowia Dziecka" Zakad Mikrobiologii i Immunologii Klinicznej	1. National Institute of Public Health - National Institute of Hygiene; 2. Eurofins Genomics Europe Sequencing GmbH	ECDC COVID-19 WGS support team; Eurofins Genomics Europe Sequencing Team; Gierczyki Rafa; Sadkowska-Todys Magorzata; Wokowicz Tomasz; Zacharczuk Katarzyna
EPI_ISL_1494940	Investigaciones Biomédicas - Universidad de Sucre	Instituto Nacional de Salud- Dirección de Investigación en Salud Pública	Carlos Franco-Muñoz; Carmen Osorio; Diana Malo; Diego A. Álvarez-Díaz; Diego Andrés Prada; Gerardo Santamaría; Hector Alejandro Ruiz-Moreno; Jhonnatan Reales-González; Juan Camilo Martinez; Julian Naizaque; Katherine Laiton-Donato; Lisseth Pardo; Magdalena Wiesner; Marcela Mercado-Reyes; Maria T. Herrera-Sepúlveda; Marta Lopez Blanco; Martha Lucia Ospina Martinez; Paola Rojas; Sergio Gomez; Sheryll Corchuelo; Ángela Alarcon Cruz
EPI_ISL_1394063 to 1394065, EPI_ISL_1538758 to 1538759, EPI_ISL_1539031 to 1539032	Invicta	1. National Institute of Public Health - National Institute of Hygiene; 2. Eurofins Genomics Europe Sequencing GmbH	ECDC COVID-19 WGS support team; Eurofins Genomics Europe Sequencing Team; Gierczyki Rafa; Sadkowska-Todys Magorzata; Wokowicz Tomasz; Zacharczuk Katarzyna
EPI_ISL_1443284, EPI_ISL_1500195	Iroquois Memorial Hospital	Illinois Department of Public Health - Springfield Lab	Bryan Sim; Gordon McCall
EPI_ISL_419299 to 419300	Ishikawa Prefectural Institute of Public Health and Environmental Science	Pathogen Genomics Center, National Institute of Infectious Diseases	Eri Nariai; Hajime Kamiya; Kentaro Itokawa; Makoto Kuroda; Masanori Hashino; Motoi Suzuki; Rina Tanaka; Sanae Kuramoto; Tsuyoshi Sekizuka
EPI_ISL_474959 to 474994, EPI_ISL_474996 to 475025, EPI_ISL_483701, EPI_ISL_483704, EPI_ISL_483706, EPI_ISL_483708 to 483709, EPI_ISL_483711 to 483725, EPI_ISL_514267 to 514274, EPI_ISL_514276 to 514316, EPI_ISL_516884 to 516891, EPI_ISL_516893 to 516921, EPI_ISL_575332 to 575334			
see above	Israel Central Virology laboratory	Israel Central Virology laboratory	Efrat Dahan Bucris; Ella Mendelson; Michal Mandelboim; Neta Zuckerman; Oran Erster
EPI_ISL_594155 to 594162	Israel Institute for Biological Research	Israel Institute for Biological Research	Adi Beth-Din and Anat Zvi; Dana Stein; Emanuelle Mamroud; Galia Zaide; Inbar Cohen-Gihon; Libby Weiss; Ofir Israeli; Orly Laskar; Shay Weiss; Yoav Gal
EPI_ISL_474958, EPI_ISL_514275, EPI_ISL_516892	Israeli Central Virology laboratory	Israel Central Virology laboratory	Efrat Dahan Bucris; Ella Mendelson; Michal Mandelboim; Neta Zuckerman; Oran Erster
EPI_ISL_477193 to 477194, EPI_ISL_477202 to 477203	Istituto Zooprofilattico Sperimentale Puglia e Basilicata;	Beaconlab (Bioinformatics, Evolution and Comparative Genomics lab), Dept of Biosciences, University on Mila	Chiara M.; Manzari C.; Parisi A.; Pesole G.
EPI_ISL_477195 to 477201	Istituto Zooprofilattico Sperimentale Puglia e Basilicata;	Beaconlab (Bioinformatics, Evolution and Comparative Genomics lab), Dept of Biosciences, University on Milan	Chiara M.; Manzari C.; Parisi A.; Pesole G.
EPI_ISL_451961	Istituto Zooprofilattico Sperimentale Puglia e Basilicata; Dipartimento di Bioscienze, Biotecnologie e Biofarmaceutica dell'Università degli Studi di Bari "A.Moro"; Istituto di Biomembrane. Bioenergetica e Biotecnologie Molecolari del Consiglio Nazionale delle Ricerche di Bari	Beaconlab (Bioinformatics Evolution and Comparative Genomics lab), Dept of Biosciences, University of Milan	Chiara M.; Manzari C.; Parisi A.; Pesole G.
EPI_ISL_451962	Istituto Zooprofilattico Sperimentale Puglia e Basilicata; Dipartimento di Bioscienze, Biotecnologie e Biofarmaceutica dell'Università degli Studi di Bari "A.Moro"; Istituto di Biomembrane. Bioenergetica e Biotecnologie Molecolari del Consiglio Nazionale delle Ricerche di Bari	Beaconlab (Bioinformatics, Evolution and Comparative Genomics lab), Dept of Biosciences, University on Milan	Chiara M.; Manzari C.; Parisi A.; Pesole G.
EPI_ISL_468914, EPI_ISL_469016, EPI_ISL_469018 to 469023, EPI_ISL_525553 to 525574, EPI_ISL_527380	Istituto Zooprofilattico Sperimentale Puglia e Basilicata; Dipartimento di Bioscienze, Biotecnologie e Biofarmaceutica dell'Università degli Studi di Bari "A.Moro"; Istituto di Biomembrane. Bioenergetica e Biotecnologie Molecolari del Consiglio Nazionale delle Ricerche di Bari	Beaconlab (Bioinformatics, Evolution and Comparative Genomics lab), Dept of Biosciences, University on Milan	Chiara M; Chiara M.; Manzari C.; Parisi A.; Pesole G.
EPI_ISL_1394749 to 1394752	Istituto Zooprofilattico Sperimentale Umbria e Marche "Togo Rosati"	Istituto Zooprofilattico Sperimentale dell'Abruzzo e Molise "G. Caporale"	Ancora M; Biagetti M; Calistri P; Cammà C; Caporale M; Curini V; Di Domenico M; Di Lollo Valeria; Di Pasquale A; Giammarioli M; Lorusso A; Mangone I; Marcacci M; Puglia I; Rinaldi A; Savini G; Scialabba S
EPI_ISL_493332 to 493333, EPI_ISL_560407	Istituto Zooprofilattico Sperimentale del Mezzogiorno	INMI Lazzaro Spallanzani IRCCS	Antonino Di Caro; Barbara Bartolini; Cesare E.M. Gruber; Francesco Messina; Giorgia Borriello; Giovanna Fusco; Maria R. Capobianchi; Martina Rueca; Maurizio Viscardi; Sergio Brandi
EPI_ISL_1424144 to 1424233, EPI_ISL_1424235, EPI_ISL_1424266 to 1424396, EPI_ISL_1545630 to 1545631	Istituto Zooprofilattico Sperimentale del Mezzogiorno	TIGEM	Andrea Ballabio; Anna Manfredi; Antonio Grimaldi; Antonio Grimaldi Patrizia Annunziata Francesco Panariello Biancamaria Pierri Claudia Tiberio Teresa Giuliano Valentina Bouche Chiara Colantuono Maria Concetta Cuomo Denise Di Concilio Lucio Di Filippo Anna Manfredi Marcello Salvi Antonio Limone Luigi Atripaldi Pellegrino Cerino Andrea Ballabio Davide Cacchiarelli; Antonio Limone Luigi Atripaldi Pellegrino Cerino; Biancamaria Pierri Claudia Tiberio Valentina Bouche; Chiara Colantuono; Davide Cacchiarelli; Denise Di Concilio; Francesco Panariello; Lucio Di Filippo; Marcello Salvi; Maria Concetta Cuomo; Patrizia Annunziata
EPI_ISL_602304	Istituto Zooprofilattico Sperimentale del Mezzogiorno	U.O. Diagnostica Virologica Dip. Sanità Animale IZSM	Lorena Cardillo; Maurizio Viscardi; e Giovanna Fusco
EPI_ISL_1390690 to 1390715	Istituto Zooprofilattico Sperimentale del Mezzogiorno - Azienda Ospedaliera "Pugliese Ciaccio" di Catanzaro	TIGEM	Andrea Ballabio; Anna Manfredi; Antonio Grimaldi; Antonio Limone Luigi Atripaldi Pellegrino Cerino; Antonio Limone Pasquale Minchella Luigi Atripaldi Pellegrino Cerino; Biancamaria Pierri Claudia Tiberio Valentina Bouche; Chiara Colantuono; Davide Cacchiarelli; Denise Di Concilio; Francesco Panariello; Lucio Di Filippo; Marcello Salvi; Maria Concetta Cuomo; Patrizia Annunziata
EPI_ISL_1423228 to 1423245, EPI_ISL_1423257 to 1423273, EPI_ISL_1423277 to 1423325, EPI_ISL_1528617 to 1528718, EPI_ISL_1528737, EPI_ISL_1528740 to 1528742, EPI_ISL_1528744, EPI_ISL_1528760 to 1528816, EPI_ISL_1528834 to 1528870, EPI_ISL_1528878 to 1528884, EPI_ISL_1528887, EPI_ISL_1528890 to 1528891, EPI_ISL_1528904, EPI_ISL_1528906, EPI_ISL_1528910 to 1528918, EPI_ISL_1528922 to 1528923, EPI_ISL_1528925 to 1528998, EPI_ISL_1529006 to 1529028			
see above	J.W. Ruby Memorial Hospital	WVU and Marshall University Combined Genomics Core Facilities	"James Denvir; Peter Perrotta; Peter Stoilov; Ryan Percifield"; Wesley Kimble
EPI_ISL_422426 to 422427	JABER AL AHMAD AL SABAH HOSPITAL - KUWAIT CITY	Dasman Diabetes Institute	Ebaa Al-Ozairi; Fahd Al-Mulla; Qais Al-Duwairi; Rasheeba Iqbal; Sumi John
EPI_ISL_515960	JGH	Department of Neurovirology, National Institute of Mental Health and Neuroscience (NIMHANS)	Anita Desai; Chitra Pattabiraman; Harsha PK; Manjunatha Venkataswamy; Pramada Prasad; Ravi Vasanthapuram; Risha Rasheed; Shafeeq S Hameed; Vijayalakshmi Reddy
EPI_ISL_422424	Jaber Al Ahmad Al Sabah Hospital	Dasman diabetes Institute	Ebaa Al-Ozairi; Fahd Al-Mulla; Qais Al-Duwairi; Rasheeba Iqbal; Sumi John
EPI_ISL_450792 to 450799	Jamaica Ministry of Health and Wellness	Pathogen Discovery, Respiratory Viruses Branch, Division of Viral Diseases, Centers for Disease Control and Prevention	Anna Montmayeur; Anna Uehara; Bettina Bankamp; Clinton R. Paden; Haibin Wang; Jasmine Padilla; Jing Zhang; Justin Lee; Krista Queen; Rachel Marine; Suxiang Tong; Yan Li; Ying Tao; Zachary Weiner
EPI_ISL_451958	Jamil-ur-Rahman Center for Genome Research, Dr. Panjwani	Jamil-ur-Rahman Center for Genome Research, Dr. Panjwani	B.A.; I.A; Khan; Khan, S.; M.A.and Khan; Qureshi; Raza; S.A.; Shakeel, M.; Zahid, M.

	Center for Molecular Medicine and Drug Research, International Center for Chemical and Biological Sciences, University of Karachi	Center for Molecular Medicine and Drug Research, International Center for Chemical and Biological Sciences, University of Karachi	
EPI_ISL_416565 to 416634, EPI_ISL_454749	Japanese Quarantine Stations	Pathogen Genomics Center, National Institute of Infectious Diseases	Hajime Kamiya; Hideki Hasegawa; Ikuyo Takayama; Kentaro Itokawa; Makoto Kuroda; Masanori Hashino; Motoi Suzuki; Rina Tanaka; Shinji Saito; Takaji Wakita; Takuri Takahashi; Takuya Yamagishi; Tsutomu Kageyama; Tsuyoshi Sekizuka
EPI_ISL_450821 to 450822	Jarpens HC	The Public Health Agency of Sweden	Anna Risberg; Anna-Malin Linde; Gunilla Johansson; Karin Tegmark-Wisell; Maria Lind Karlberg; Mia Brytting; Olov Svartstrom; Oskar Karlsson Lindsjo; Theresa Enkirch
EPI_ISL_486396	Jayanagar General Hospital to Victoria Hospital	Department of Neurovirology, National Institute of Mental Health and Neuroscience (NIMHANS)	Anita Desai; Chitra Pattabiraman; Harsha PK; Manjunatha Venkataswamy; Ravi Vasanthapuram; Risha Rasheed; Shafeeq S Hameed; Vijayalakshmi Reddy
EPI_ISL_1423009	Jefferson Medical Center	WVU and Marshall University Combined Genomics Core Facilities	"James Denvir; Peter Perrotta; Peter Stoilov; Ryan Percifield"; Wesley Kimble
EPI_ISL_1392642 to 1392646, EPI_ISL_1416683 to 1416684, EPI_ISL_1416688 to 1416725	Jessa	Jessa	Cruys et al. on behalf of the Jessa_cmdLab
EPI_ISL_421237 to 421254, EPI_ISL_421256 to 421262, EPI_ISL_455460 to 455467	Jiangxi Province Center for Disease Control and Prevention	Jiangxi Province Center for Disease Control and Prevention	DaJin Xiao; Fang Xiao; Gang Xu; JianXiong Li; Jun Zhou; ShiWen Liu; Tian Gong; XiaoQing Liu; Xin Ran; YanNi Zhang; Ying Xiong; Yong Shi
EPI_ISL_417420	Jiangxi province Center for Disease Control and Prevention	Jiangxi province Center for Disease Control and Prevention	Li jian Xiong
EPI_ISL_434586 to 434588, EPI_ISL_434638 to 434640, EPI_ISL_434682 to 434691, EPI_ISL_438222 to 438247, EPI_ISL_457751 to 457823, EPI_ISL_511863 to 511877, EPI_ISL_1402786 to 1402931, EPI_ISL_1405466 to 1405598, EPI_ISL_1468532 to 1468655, EPI_ISL_1547907 to 1548038			
see above	Johns Hopkins Hospital Department of Pathology	Johns Hopkins Hospital Department of Pathology	Adannaya Amadi; Amanda Ernlund; C. Paul Morris; Chun Huai Luo; Craig Howser; Heba H. Mostafa; Heba Mostafa; Kristina Zudock; Mark Hopkins; Matthew Schwartz; Melanie Kirsche; Michael C. Schatz; Michael Schatz; N_di_ Trovao; Nidia Trovao; Norah Sadowski; Nídiá Trovao; Oluwaseun Falade-Nwulia; Oluwaseun Nwulia-Falade; Paul Morris; Peter M. Thielen; Shirlee Wohl; Srividya Ramakrishnan; Stuart C. Ray; Thomas Mehoke; Timothy Gilpatrick; Victoria Gniazdowski; Winston Timp; Yunfan Fan; _Amanda Ernlund; _Oluwaseun Falade-Nwulia
EPI_ISL_445242	Jokkmokks Halsocentral	The Public Health Agency of Sweden	Anna Risberg; Anna-Malin Linde; Karin Tegmark-Wisell; Maria Lind Karlberg; Markus Beland; Mia Brytting; Olov Svartstrom; Oskar Karlsson Lindsjo; Theresa Enkirch
EPI_ISL_455101, EPI_ISL_469055 to 469056	Jourcentralen	The Public Health Agency of Sweden	Anna Risberg; Anna-Malin Linde; Karin Tegmark-Wisell; Maria Lind Karlberg; Mattias Haukland; Mia Brytting; Olov Svartstrom; Oskar Karlsson Lindsjo; Petra Edquist; Reza Advani; Salvatore Ascione; Sandra Broddesson; Shamam Muradrasoli; Theresa Enkirch
EPI_ISL_1398209 to 1398258	KALEIDA CENTER FOR LABORATORY MEDICINE	Wadsworth Center, New York State Department of Health	Alexis Russel; Daryl M. Lamson; Erasmus Schneider; Erica Lasek-Nesselquist; John Kelly; Jonathan Plitnick; Kirsten St. George; Matthew Shudt; Melissa A Leisner; Navjot Singh
EPI_ISL_486397	KC General Hospital	Department of Neurovirology, National Institute of Mental Health and Neuroscience (NIMHANS)	Anita Desai; Chitra Pattabiraman; Harsha PK; Manjunatha Venkataswamy; Ravi Vasanthapuram; Risha Rasheed; Shafeeq S Hameed; Vijayalakshmi Reddy
EPI_ISL_1385814 to 1385816, EPI_ISL_1385825, EPI_ISL_1385829 to 1385832, EPI_ISL_1385839 to 1385840, EPI_ISL_1396655	KEMPEGOWDA INTERNATIONAL AIRPORT	INSACOG-KA, NIMHANS	Anita S Desai; Anson Kunjumon George; Chitra Pattabiraman; Darshan Sreenivas; Nakka Vijay Kiran Reddy; Pramada Prasad; V Ravi
EPI_ISL_457845 to 457931	KEMRI-CGMR-C	KEMRI-Wellcome Trust Research Programme/KEMRI-CGMR-C Kilifi	Githinji G. et al 2020
EPI_ISL_457932 to 457936	KEMRI-Centre for Virus Research	KEMRI-Wellcome Trust Research Programme/KEMRI-CGMR-C Kilifi	Githinji G. et al 2020
EPI_ISL_1440075, EPI_ISL_1440078, EPI_ISL_1440081, EPI_ISL_1440084, EPI_ISL_1440087, EPI_ISL_1440090, EPI_ISL_1440093, EPI_ISL_1440096, EPI_ISL_1440099, EPI_ISL_1440102, EPI_ISL_1440106, EPI_ISL_1440109, EPI_ISL_1440112, EPI_ISL_1440115, EPI_ISL_1440118 to 1440119, EPI_ISL_1440125, EPI_ISL_1440128, EPI_ISL_1440131, EPI_ISL_1440134, EPI_ISL_1440136, EPI_ISL_1440139, EPI_ISL_1440142, EPI_ISL_1440145, EPI_ISL_1440148, EPI_ISL_1440151, EPI_ISL_1440154, EPI_ISL_1440157, EPI_ISL_1440164, EPI_ISL_1440171 to 1440172, EPI_ISL_1440174, EPI_ISL_1440178, EPI_ISL_1440181, EPI_ISL_1440184, EPI_ISL_1440188, EPI_ISL_1440190, EPI_ISL_1440193, EPI_ISL_1440196, EPI_ISL_1440199, EPI_ISL_1440202, EPI_ISL_1440206 to 1440212, EPI_ISL_1440214, EPI_ISL_1440220, EPI_ISL_1440223, EPI_ISL_1440226, EPI_ISL_1440229, EPI_ISL_1440232, EPI_ISL_1440236, EPI_ISL_1440240, EPI_ISL_1440243, EPI_ISL_1440245, EPI_ISL_1440252, EPI_ISL_1440254, EPI_ISL_1440257, EPI_ISL_1440261, EPI_ISL_1440264, EPI_ISL_1440266, EPI_ISL_1440269, EPI_ISL_1440272, EPI_ISL_1440275, EPI_ISL_1440278, EPI_ISL_1440281, EPI_ISL_1440284, EPI_ISL_1440287, EPI_ISL_1440289, EPI_ISL_1440292, EPI_ISL_1440295, EPI_ISL_1440298, EPI_ISL_1440301, EPI_ISL_1440304, EPI_ISL_1440307, EPI_ISL_1440310, EPI_ISL_1440314, EPI_ISL_1440316, EPI_ISL_1440319, EPI_ISL_1440322			
see above	KEMRI-Wellcome Trust Research Programme,Kilifi	KEMRI-Wellcome Trust Research Programme,Kilifi	Githinji G.; Mburu M.W.; Mohamed K.S.; deLaurent Z.
EPI_ISL_568695 to 568872	KEMRI-Wellcome Trust Research Programme/KEMRI-CGMR-C Kilifi	KEMRI-Wellcome Trust Research Programme/KEMRI-CGMR-C Kilifi	Githinji et al 2020
EPI_ISL_1437281 to 1437282	KRH Klinikum Region Hannover: Institut für Med. Mikrobiologie und Krankenhaushygiene	Robert Koch Institute	
EPI_ISL_436684 to 436687, EPI_ISL_455629 to 455639	KRISP, KZN Research Innovation and Sequencing Platform	KRISP, KZN Research Innovation and Sequencing Platform	Chimukangara B; Deforche K; Giandhari J; Lessells R; Pillay S; Tegally H; Wilkinson E; de Oliveira T
EPI_ISL_1446938 to 1446940, EPI_ISL_1446942 to 1446943, EPI_ISL_1516313 to 1516316, EPI_ISL_1528399 to 1528403	KS Health and Environmental Laboratories	Centers for Disease Control and Prevention Division of Viral Diseases, Pathogen Discovery	Alison Laufer Halpin; Ben L. Rambo-Martin; Clinton R. Paden; Dakota Howard; Darlene Wagner; Dave Wentworth; Dhwani Batra; Jasmine Padilla; Justin Lee; Katie Dillon; Krista Queen; Kristen Knipe; Kristine Lacek; Mark Burroughs; Matthew Schmerer; Mili Sheth; Peter Cook; Sam Shepard; Sarah Nobles; Shoshona Le; Suxiang Tong; Vivien Dugan; Yvette Unoarumhi
EPI_ISL_424867, EPI_ISL_452114	KS Health and Environmental Laboratories	Pathogen Discovery, Respiratory Viruses Branch, Division of Viral Diseases, Centers for Disease Control and Prevention	Alison S. Laufer Halpin; Anna Montmayeur; Anna Uehara; Christopher A. Elkins; Clinton R. Paden; Haibin Wang; Jing Zhang; Krista Queen; Mary S. Keckler; Rachel Marine; Suxiang Tong; Yan Li; Ying Tao; Zachary Weiner
EPI_ISL_1478928 to 1478932, EPI_ISL_1500152	KSB Hospital	Illinois Department of Public Health - Springfield Lab	Bryan Sim; Gordon McCall
EPI_ISL_415154	KU Leuven, Clincal and Epidemiological Virology	KU Leuven, Clincal and Epidemiological Virology	Bert Vanmechelen; Joan Marti-Careras; Marc Van Ranst; Piet Maes.; Tony Wawina
EPI_ISL_407976, EPI_ISL_415153, EPI_ISL_415155 to 415159, EPI_ISL_416467 to 416472, EPI_ISL_416475 to 416476, EPI_ISL_417422, EPI_ISL_417424 to 417430, EPI_ISL_418270, EPI_ISL_418792 to 418798, EPI_ISL_418800, EPI_ISL_418805 to 418806, EPI_ISL_418863, EPI_ISL_418981 to 418987, EPI_ISL_418989, EPI_ISL_420314 to 420454			
see above	KU Leuven, Clinical and Epidemiological Virology	KU Leuven, Clinical and Epidemiological Virology	Annabel Rector; Bert Vanmechelen; Elke Wollants; Els Keyaerts; Joan Marti-Carerras; Joan Marti-Carreras; Lies Laenen; Marc Van Ranst; Piet Maes; Tony Wawina; and Piet Maes
EPI_ISL_458156 to 458235, EPI_ISL_462151 to 462275, EPI_ISL_464065 to 464090, EPI_ISL_476941 to 477007, EPI_ISL_522349 to 522350	KU Leuven, Rega Institute, Clinical and Epidemiological Virology	KU Leuven, Rega Institute, Clinical and Epidemiological Virology	Bert Vanmechelen; Joan Marti-Carerras; Piet Maes; Tony Wawina-Bokalanga
EPI_ISL_539300 to 539325, EPI_ISL_549006 to 549023	KWR Watercycle Research Institute	Erasmus Medical Center	Bas B. Oude Munnink; Claudia M. E. Schapendonk; David Nieuwenhuijse; Frank M. Aarestrup; Gertjan Medema; Goffe Elsinga; Leo Heijnen; Lu Lu; Marion P.G. Koopmans; Matthijs Kon; Miranda de Graaf; Ray Izquierdo-Lara; Samantha Lycett
EPI_ISL_1446335, EPI_ISL_1446338, EPI_ISL_1446344, EPI_ISL_1446347, EPI_ISL_1446350, EPI_ISL_1446352, EPI_ISL_1446355, EPI_ISL_1446358, EPI_ISL_1446361, EPI_ISL_1446364, EPI_ISL_1516353 to 1516360			
see above	KY State Public Health Lab	Centers for Disease Control and Prevention Division of Viral Diseases, Pathogen Discovery	Alison Laufer Halpin; Ben L. Rambo-Martin; Clinton R. Paden; Dakota Howard; Darlene Wagner; Dave Wentworth; Dhwani Batra; Jasmine Padilla; Justin Lee; Katie Dillon; Krista Queen; Kristen Knipe; Kristine Lacek; Mark Burroughs; Matthew Schmerer; Mili Sheth; Peter Cook; Sam Shepard; Sarah Nobles; Shoshona Le; Suxiang Tong; Vivien Dugan; Yvette Unoarumhi
EPI_ISL_495411 to 495457	Kafkas University, Faculty of Medicine, Department of Medical Microbiology	Kafkas University, Faculty of Medicine, Department of Medical Microbiology	Didem Ozgur; E. Ediz Tutuncu; Murat Karamese

EPI_ISL_1508918	Kaiser Permanente Downey Medical Center	Los Angeles County PHL	P. Hemarajata et al.
EPI_ISL_1533431 to 1533435, EPI_ISL_1533437, EPI_ISL_1533439 to 1533440, EPI_ISL_1533445, EPI_ISL_1533448, EPI_ISL_1533451, EPI_ISL_1533531, EPI_ISL_1533533, EPI_ISL_1533535, EPI_ISL_1533564 to 1533608, EPI_ISL_1559386 to 1559387			
see above	Kaiser Permanente Washington Health Research Institute	Genomics and Discovery, Respiratory Viruses Branch, Division of Viral Diseases, Centers for Disease Control and Prevention	Adam Retchless; Anna Kelleher; Anna Montmayeur; Anna Uehara; Brian Lynch; Clinton R. Paden; Haibin Wang; Han Jia Justin Ng; Jing Zhang; Justin Lee; Krista Queen; Mark Burroughs; Peter Cook; Rachel Marine; Suxiang Tong; Yan Li; Ying Tao
EPI_ISL_1508902, EPI_ISL_1508917	Kaiser Permanente West Los Angeles Medical Center	Los Angeles County PHL	P. Hemarajata et al.
EPI_ISL_1508984 to 1508986	Kaiser Regional	Santa Clara County Public Health Laboratory	Santa Clara County Public Health Department
EPI_ISL_1532141 to 1532143	Kaiser San Jose	Santa Clara County Public Health Laboratory	Santa Clara County Public Health Department
EPI_ISL_1508966 to 1508977	Kaiser Santa Clara	Santa Clara County Public Health Laboratory	Santa Clara County Public Health Department
EPI_ISL_475137 to 475138, EPI_ISL_510825 to 510826, EPI_ISL_510832 to 510833, EPI_ISL_510836 to 510837, EPI_ISL_510859, EPI_ISL_510864 to 510865	Kalmar klinisk mikrobiologi	The Public Health Agency of Sweden	Anna Risberg; Anna-Malin Linde; Karin Tegmark-Wisell; Maria Lind Karlberg; Mattias Haukland; Mia Brytting; Olov Svartstrom; Oskar Karlsson Lindsjo; Petra Edquist; Reza Advani; Sandra Broddesson; Shamam Muradrasoli
EPI_ISL_1400507, EPI_ISL_1400523, EPI_ISL_1400546 to 1400551, EPI_ISL_1400561	Kamchatka Regional Children's Infectious Diseases Hospital	WHO National Influenza Centre Russian Federation	Andrey Komissarov; Anna Ivanova; Artem Fadeev; Daria Danilenko; Dmitry Bazhenov; Dmitry Lioznov; Elena Nabieva; Georgii Bazykin; Ksenia Safina; Kseniya Komissarova; Maria Pisareva; Maria Timofeeva; Tamila Musaeva; Veronika Eder
EPI_ISL_1427573 to 1427576, EPI_ISL_1430582 to 1430583, EPI_ISL_1430740, EPI_ISL_1430749 to 1430750, EPI_ISL_1431021 to 1431030	Kanagawa Prefectural Institute of Public Health	Pathogen Genomics Center, National Institute of Infectious Diseases	; Daisuke Kobayashi; Hussein H. Aly; Ken Miyazawa; Kentaro Itokawa; Kento Fukano; Makoto Kuroda; Masanori Hashino; Minoru Nagi; Nozomu Hanaoka; Rina Tanaka; Takanobu Kato; Takashi Sakudoh; Tsuguto Fujimoto; Tsuyoshi Sekizuka
EPI_ISL_1392916 to 1392921, EPI_ISL_1396647 to 1396649, EPI_ISL_1401966 to 1401985, EPI_ISL_1412475, EPI_ISL_1412478, EPI_ISL_1412481, EPI_ISL_1412484, EPI_ISL_1412487, EPI_ISL_1412490, EPI_ISL_1412493, EPI_ISL_1412496, EPI_ISL_1412499, EPI_ISL_1412502, EPI_ISL_1412505, EPI_ISL_1412508, EPI_ISL_1412510, EPI_ISL_1412513, EPI_ISL_1412516, EPI_ISL_1412519, EPI_ISL_1412522, EPI_ISL_1412525, EPI_ISL_1412528, EPI_ISL_1412530, EPI_ISL_1443756 to 1443778, EPI_ISL_1447074 to 1447083, EPI_ISL_1447085 to 1447086, EPI_ISL_1461626 to 1461633, EPI_ISL_1461638, EPI_ISL_1465683 to 1465688, EPI_ISL_1494550 to 1494553, EPI_ISL_1500448 to 1500470, EPI_ISL_1502796 to 1502803, EPI_ISL_1510641 to 1510646			
see above	Kansas Health and Environmental Lab	Kansas Health and Environmental Lab	Ben Olsen; Jonathan Barnell; Mike Grose; and Phil Adam
EPI_ISL_454447 to 454493, EPI_ISL_454868 to 454902, EPI_ISL_455846 to 455901, EPI_ISL_469075, EPI_ISL_469077 to 469079, EPI_ISL_475086 to 475091, EPI_ISL_475095, EPI_ISL_475136, EPI_ISL_475141 to 475142, EPI_ISL_475149 to 475150, EPI_ISL_475544 to 475547, EPI_ISL_475551 to 475552, EPI_ISL_475558 to 475561, EPI_ISL_510834 to 510835, EPI_ISL_510838 to 510840, EPI_ISL_510852, EPI_ISL_510860 to 510863, EPI_ISL_560974, EPI_ISL_615086 to 615089			
see above	Karolinska Universitetslaboratoriet	The Public Health Agency of Sweden	Anna Risberg; Anna-Malin Linde; Karin Tegmark-Wisell; Maria Lind Karlberg; Mattias Haukland; Mia Brytting; Olov Svartstrom; Oskar Karlsson Lindsjo; Petra Edquist; Reza Advani; Sandra Broddesson; Shamam Muradrasoli; Shaman Muradrasoli
EPI_ISL_534259, EPI_ISL_548257 to 548258, EPI_ISL_560989 to 560990	Karolinska universitetslaboratoriet	The Public Health Agency of Sweden	Anna Risberg; Anna-Malin Linde; Karin Tegmark-Wisell; Maria Lind Karlberg; Mattias Haukland; Mia Brytting; Olov Svartstrom; Oskar Karlsson Lindsjo; Petra Edquist; Reza Advani; Sandra Broddesson
EPI_ISL_534234 to 534236, EPI_ISL_560982 to 560985	Karolinska universitetslaboratoriet SOLNA	The Public Health Agency of Sweden	Anna Risberg; Anna-Malin Linde; Karin Tegmark-Wisell; Maria Lind Karlberg; Mattias Haukland; Mia Brytting; Olov Svartstrom; Oskar Karlsson Lindsjo; Petra Edquist; Reza Advani; Sandra Broddesson
EPI_ISL_1398357	Kasih Bunda Hospital	West Java Health Laboratory; School of Life Sciences and Technology, Institut Teknologi Bandung	Azzania Fibriani; Cut Nur Cinthia Alamanda; Ema Rahmawati; Isak Solihin; Kamila Tania; Karimatu Khoirunnisa; Miftahul Faridl; Rifky Waluyajati Rachman; Rini Robiani; Ryan Bayusantika Ristandi
EPI_ISL_1464105, EPI_ISL_1464262, EPI_ISL_1464269 to 1464270	Kath. Marienkrankenhaus	Heinrich Pette Institute, Leibniz Institute for Experimental Virology	Adam Grundhoff; Alexis Robitaille; Johannes Knobloch; Martin Aepfelbacher; Nicole Fischer; Thomas Günther
EPI_ISL_1439487	Kath. St.-Johannes-Gesellschaft Dortmund gGmbH	Robert Koch Institute	
EPI_ISL_1443222 to 1443225	Katherine Shaw Bethea Hospital	Illinois Department of Public Health - Springfield Lab	Bryan Sim; Gordon McCall
EPI_ISL_442044	Kawsar Human Genetic Research Center	Kawsar Human Genetic Research Center	Fatemeh Hosseini; Maryam Abbasalipour Bashash; Mohammad Ali Khosravi; Sirous Zeinali; Solmaz Sabeghi; Yeganeh Haghdoost; Yeganeh Keshvar
EPI_ISL_469287 to 469296	Keio University Hospital	Keio University Hospital	Kenjiro Kosaki
EPI_ISL_437601, EPI_ISL_438947 to 438949, EPI_ISL_438951 to 438956, EPI_ISL_438958 to 438959, EPI_ISL_438961 to 438972	Keio University School of Medicine	Keio University School of Medicine	; Haruhiko Siomi; Kenjiro Kosaki; Toshiki Takenouchi; Yuka Iwasaki
EPI_ISL_512811 to 512821	Kenema Government Hospital, Ministry of Health and Sanitation	Kenema Government Hospital, Ministry of Health and Sanitation	Andersen, K.; Garry, R.; Goba, A.; Grant, D.; Happi, C.; Jalloh, S.; Mehta, S.; Momoh, M.; Olawoye, I.; Oluniyi, P.; Park, D.; Sandi, J.; Siddle, K.; Tomkins-Tinch, C.
EPI_ISL_1470376 to 1470394, EPI_ISL_1470396 to 1470413, EPI_ISL_1491343 to 1491360, EPI_ISL_1491368 to 1491386	Kentucky State Public Health Lab	Kentucky State Public Health Lab	Joshua Tobias; Karim George; Matthew Johnson; Rachel Zinner; Rhonda Lucas; Stephanie Lunn; Vaneet Arora; William Grooms
EPI_ISL_414510	Key Laboratory of Medical Molecular Virology (MOE/NHC/CAMS), School of Basic Medicine, Shanghai Medical College, Fudan University	Key Laboratory of Medical Molecular Virology (MOE/NHC/CAMS), School of Basic Medicine, Shanghai Medical College, Fudan University	Cai, X.; Chen, H.; Fang, F.; Feng, F.; Fu, C.; Gu, C.; Han, W.; Li, C.; Qu, D.; Song, W.; Sun, Z.; Teng, Z.; Wang, Y.; Xie; Xu, W.; Y. and Yuan, Z.; Ye, R.; Yi, Z.; Zhang, R.; Zhang, X.; Zhou, Y.; Zhu, Y.
EPI_ISL_489996 to 490012	King Fahad Medical City	King Fahad Medical City	Alghoraibi, M.; Alosaimi, B.; Enani, M.; Naeem, A.
EPI_ISL_516940 to 516986	King Georges Medical University	CSIR-National Botanical Research Institute	Amita Jain; Babita Singh; Danish Nasar Khan; Hricha Mishra; Kishan Sahu; MLB Bhatt; Mehar H.Asif; Om Prakash; Priti Prasad; SK Barik; Samir V. Sawant; Shantanu Prakash; Sumit Kr. Bag; Suruchi Shukla
EPI_ISL_458030 to 458044, EPI_ISL_471583 to 471584	King Institute of Preventive Medicine & Research	CSIR-Centre for Cellular and Molecular Biology	Archana Bharadwaj Siva; Dhiviya Vedagiri; Divya Gupta; Divya Tej Sowpati; G. Dhinakar Raj; G. Ravikumar; K Thangaraj; K.Kaveri; Karthik Bharadwaj Tallapaka; Krishnan Harinivas Harshan; Lamuk Zaveri; M. Sekar; Namami Gaur; P. Azhahianambi; P.Padmapriya; Payel Mukherjee; Priya Singh; Purushotham Vodnala; R. P. Aravindh Babu; R.Kiruba; Rakesh K Mishra; S.Magesh; S.Sivasubramanian; S.Vennila; Sakshi Shambhavi; Santosh Kumar Kuncha; Shagufta Khan; Sofia Banu; Tulasi Nagabandi; Vishal Sah
EPI_ISL_483542 to 483565, EPI_ISL_483638 to 483640	Kingdom of Bahrein Ministry of Health	Erasmus Medical Center	Amjad Ghanem Mohamed; Anne van der Linden; Bas Oude Munnink; Claudia Schapendonk; David Nieuwenhuijse; Ebrahim Shehad; Fatema; Hashmeya Al Wasti; Irina Chestakova; Marion Koopmans; Mark Pronk; Pascal Lexmond; Reina Sikkema; Richard Molenkamp; Stefan van Nieuwkoop; Theo Bestebroer; on behalf of the Dutch national COVID-19 response team.
EPI_ISL_459866 to 459869, EPI_ISL_459871 to 459875, EPI_ISL_459877 to 459892, EPI_ISL_529029 to 529030	Kingston Health Sciences Center	Queen's Genomics Lab at Ongwanada (Q-GLO)	Ayub M; Colautti R; Evans GA; Guan H; Huang D; Hudson ML; Perez-Patrigeon S; Rustom N; Sheth P; Sjaarda CP; Soares CN; Wong H
EPI_ISL_538321 to 538386	Kingston Health Sciences Centre / Queen's University	Ontario Institute for Cancer Research	Bernard Lam; Calvin Sjaarda; Ilinca Lungu; Jared T. Simpson; Katya Douchant; Lawrence E Heisler; Michael Laszloffy; Paul Krzyzanowski; Prameet M. Sheth; Richard de Borja; Robert Colautti
EPI_ISL_479990	Kitakyushu City Institute of Health and Environmental Sciences	Pathogen Genomics Center, National Institute of Infectious Diseases	Asuka Kikuchi Kentaro Itokawa; Hajime Kamiya; Katsuya Obata; Makoto Kuroda; Masanori Hashino; Motoi Suzuki; Rina Tanaka; Tsuyoshi Sekizuka
EPI_ISL_1384665 to 1384691, EPI_ISL_1396391 to 1396399, EPI_ISL_1510084, EPI_ISL_1510087, EPI_ISL_1510202, EPI_ISL_1510256 to 1510259, EPI_ISL_1510480 to 1510562			
see above	Klaipeda University Hospital	Vilnius University Hospital Santaros Klinikos, Center of Laboratory Medicine	Daniel Naumovas; Dovile Ezerskyte; Gytis Dudas; Ingrida Olendraite; Laimonas Griskevicius; Ligita Raugaite; Mindaugas Stoskus; Monika Katenaite; Rimvydas Norvilas

EPI_ISL_518820	Klinik Apotek Dein, Jakarta, Indonesia	Biosafety Level-3 Laboratory, Indonesian Institute of Sciences (LIPI)	Ade Andriani; Ahmad Fathoni; Andri Wardiana; Anggia Prasetyoputri; Anik Budhi Dharmayanthi; Ario Betha Juanssilfero; Asep M Ridwanuloh; Isa Nuryana; Puspita Lisdiyanti; Ratih Asmana Ningrum; Rifki Sadikin; Syam Budi Iryanto; Wien Kusharyoto
EPI_ISL_413021	Klinik Hirslanden Zurich	Institute of Medical Virology, University of Zurich	Alexandra Trkola; Andrea Zbinden; Christian Ruef; Fiona Steiner; Gabriela Ziltener; Jon Huder; Jürg Böni; Maryam Zaheri; Michael Huber; Patrick Redli; Riccarda Capaul; Stefan Schmutz; Verena Kufner
EPI_ISL_524479	Klinik Hirslanden Zürich	Institute of Medical Virology, University of Zurich	Alexandra Trkola; Andrea Zbinden; Fiona Steiner; Gabriela Ziltener; Jon Huder; Jürg Böni; Maryam Zaheri; Michael Huber; Patrick Redli; Riccarda Capaul; Stefan Schmutz; Verena Kufner
EPI_ISL_1438660, EPI_ISL_1438666, EPI_ISL_1441213, EPI_ISL_1441225	Klinikum Darmstadt GmbH	Robert Koch Institute	
EPI_ISL_1442531 to 1442535, EPI_ISL_1442537 to 1442542	Klinikum Ernst von Bergmann gemeinnützige GmbH - stationärer Bereich	Robert Koch Institute	
EPI_ISL_1435441, EPI_ISL_1438761, EPI_ISL_1441195, EPI_ISL_1441197	Klinikum Fulda gAG	Robert Koch Institute	
EPI_ISL_475910 to 475915, EPI_ISL_1495166 to 1495178, EPI_ISL_1495267 to 1495271, EPI_ISL_1495528 to 1495542, EPI_ISL_1495762 to 1495800	Klinikum Wels-Grieskirchen	Bergthaler laboratory, CeMM Research Center for Molecular Medicine of the Austrian Academy of Sciences	Alexander Lercher; Alexandra Popa; Andreas Bergthaler; Anna Schedl; Bekir Erguner; Benedikt Agerer; Christoph Bock; Daniela Schmid; Dorothee von Laer; Elisabeth Puchhammer-Stoeckl; Fabian Amman; Franz Allerberger; Gregor Hörmann; Guenter Weiss; Henrique Colaco; Jakob-Wendelin Genger; Jan Laine; Judith Aberle; Kinga Rigler-Hohenwarter; Lukas Endler; Maelle Le Moing; Manfred Nairz; Mark Smyth; Martin Senekowitsch; Michael Schuster; Peter Hufnagl; Petr Triska; Rainer Gattringer; Stephan Aberle; Thomas Penz; Wegene Borena
EPI_ISL_1404375 to 1404394	Klinisch Laboratorium ZNA	Klinisch Laboratorium ZNA	Verstrepen et al.
EPI_ISL_475101 to 475104, EPI_ISL_476140 to 476142, EPI_ISL_582808, EPI_ISL_615108	Klinisk Mikrobiologi	The Public Health Agency of Sweden	Anna Risberg; Anna-Malin Linde; Karin Tegmark-Wisell; Maria Lind Karlberg; Mattias Haukland; Mia Brytting; Olov Svartstrom; Oskar Karlsson Lindsjo; Petra Edquist; Reza Advani; Sandra Broddesson; Shamam Muradrasoli
EPI_ISL_615097 to 615100	Klinisk mikrobiologi Lanssjukhuset Ryhov, Jonkoping	The Public Health Agency of Sweden	Anna Risberg; Anna-Malin Linde; Karin Tegmark-Wisell; Maria Lind Karlberg; Mattias Haukland; Mia Brytting; Olov Svartstrom; Oskar Karlsson Lindsjo; Petra Edquist; Reza Advani; Sandra Broddesson
EPI_ISL_510841 to 510844, EPI_ISL_510868 to 510870, EPI_ISL_548250 to 548251, EPI_ISL_548253 to 548254, EPI_ISL_560973, EPI_ISL_615090 to 615091, EPI_ISL_615109			
see above	Klinisk mikrobiologi NAL Trollhattan	The Public Health Agency of Sweden	Anna Risberg; Anna-Malin Linde; Karin Tegmark-Wisell; Maria Lind Karlberg; Mattias Haukland; Mia Brytting; Olov Svartstrom; Oskar Karlsson Lindsjo; Petra Edquist; Reza Advani; Sandra Broddesson
EPI_ISL_429136 to 429148, EPI_ISL_429150 to 429158, EPI_ISL_429160, EPI_ISL_430860 to 430861, EPI_ISL_430863	Klinisk mikrobiologi Orebro	The Public Health Agency of Sweden	Anna Risberg; Anna-Malin Linde; Karin Tegmark-Wisell; Maria Lind Karlberg; Martin Sundqvist; Olov Svartstrom; Oskar Karlsson Lindsjo; Shaman Muradrasoli
EPI_ISL_582806 to 582807	Klinisk mikrobiologi SAS Boras	The Public Health Agency of Sweden	Anna Risberg; Anna-Malin Linde; Karin Tegmark-Wisell; Maria Lind Karlberg; Mattias Haukland; Mia Brytting; Olov Svartstrom; Oskar Karlsson Lindsjo; Petra Edquist; Reza Advani; Sandra Broddesson
EPI_ISL_475144 to 475148, EPI_ISL_475153 to 475155, EPI_ISL_475160 to 475163, EPI_ISL_510818 to 510819, EPI_ISL_510853 to 510858, EPI_ISL_582797 to 582798, EPI_ISL_582800, EPI_ISL_582835 to 582838			
see above	Klinisk mikrobiologi Vasternorrland	The Public Health Agency of Sweden	Anna Risberg; Anna-Malin Linde; Karin Tegmark-Wisell; Maria Lind Karlberg; Mattias Haukland; Mia Brytting; Olov Svartstrom; Oskar Karlsson Lindsjo; Petra Edquist; Reza Advani; Sandra Broddesson; Shamam Muradrasoli
EPI_ISL_510820 to 510824, EPI_ISL_510867, EPI_ISL_548252, EPI_ISL_560975	Klinisk mikrobiologi centralsjukhuset Karlstad	The Public Health Agency of Sweden	Anna Risberg; Anna-Malin Linde; Karin Tegmark-Wisell; Maria Lind Karlberg; Mattias Haukland; Mia Brytting; Olov Svartstrom; Oskar Karlsson Lindsjo; Petra Edquist; Reza Advani; Sandra Broddesson
EPI_ISL_429115 to 429125, EPI_ISL_430848 to 430855, EPI_ISL_450834	Klinisk mikrobiologi och vardhygien Halmstad	The Public Health Agency of Sweden	Anna Risberg; Anna-Malin Linde; Arne Kotz; Karin Tegmark-Wisell; Maria Lind Karlberg; Mia Brytting; Olov Svartstrom; Oskar Karlsson Lindsjo; Shaman Muradrasoli; Theresa Enkirch
EPI_ISL_534256, EPI_ISL_560988	Klinisk mikrobiologi, Laboratoriemedicin Gavleborg	The Public Health Agency of Sweden	Anna Risberg; Anna-Malin Linde; Karin Tegmark-Wisell; Maria Lind Karlberg; Mattias Haukland; Mia Brytting; Olov Svartstrom; Oskar Karlsson Lindsjo; Petra Edquist; Reza Advani; Sandra Broddesson
EPI_ISL_1469159 to 1469231	Klinisk mikrobiologi, Region Västerbotten	CBRN Defence and Security, Swedish Defence Research Agency	Andreas Sjödin; Anna-Lena Johansson; Annika Allard; Annika Osterman; Caroline Öhrman; David Sundell; Emelie Näslund Salomonsson; Ingrid Dacklin; Jonas Näslund; Kerstin Myrtennäs; Linda Karlsson; Malin Granberg; Mats Forsman; Stina Bäckman
EPI_ISL_424703, EPI_ISL_428148, EPI_ISL_428201, EPI_ISL_548962 to 548963, EPI_ISL_549024, EPI_ISL_549026, EPI_ISL_549176			
see above	Klinisk mikrobiologi, Region Västerbotten	Unit for Biological Agents, Department for CBRN Defence and Security, Swedish Defence Research Agency	FOI Bioinformatics team; FOI bioinformatics team
EPI_ISL_615080 to 615085	Klinisk mikrobiologi, Skanes universitetssjukhus, Lund	The Public Health Agency of Sweden	Anna Risberg; Anna-Malin Linde; Karin Tegmark-Wisell; Maria Lind Karlberg; Mattias Haukland; Mia Brytting; Olov Svartstrom; Oskar Karlsson Lindsjo; Petra Edquist; Reza Advani; Sandra Broddesson
EPI_ISL_454903, EPI_ISL_455904 to 455907	Klinisk mikrobiologi, UAS	The Public Health Agency of Sweden	Anna Risberg; Anna-Malin Linde; Karin Tegmark-Wisell; Maria Lind Karlberg; Mattias Haukland; Olov Svartstrom; Oskar Karlsson Lindsjo; Petra Edquist; Reza Advani; Shamam Muradrasoli
EPI_ISL_534245, EPI_ISL_560987	Kliniskt mikrobiologiska laboratoriet	The Public Health Agency of Sweden	Anna Risberg; Anna-Malin Linde; Karin Tegmark-Wisell; Maria Lind Karlberg; Mattias Haukland; Mia Brytting; Olov Svartstrom; Oskar Karlsson Lindsjo; Petra Edquist; Reza Advani; Sandra Broddesson
EPI_ISL_510871 to 510877, EPI_ISL_560976, EPI_ISL_582771 to 582775, EPI_ISL_594149 to 594153, EPI_ISL_615110, EPI_ISL_615117 to 615119	Klinsisk mikrobiologi Linkoping	The Public Health Agency of Sweden	Anna Risberg; Anna-Malin Linde; Karin Tegmark-Wisell; Maria Lind Karlberg; Mattias Haukland; Mia Brytting; Olov Svartstrom; Oskar Karlsson Lindsjo; Petra Edquist; Reza Advani; Sandra Broddesson
EPI_ISL_434659, EPI_ISL_450811	Knivsta VC	The Public Health Agency of Sweden	Anna Risberg; Anna-Malin Linde; Johanna Carlson; Karin Tegmark-Wisell; Maria Lind Karlberg; Mia Brytting; Olov Svartstrom; Oskar Karlsson Lindsjo; Theresa Enkirch
EPI_ISL_1534360	Knysna CDC wc WLC	NHLS/UCT	Arash Iranzadeh; Bruna Galvao; Carolyn Williamson; Deelan Doolabh; Diana Hardie; Emmanuel SJ; Innocent Mudau; Kruger Marais; Lynn Tyers; Marvin Hsiao; Stephen Korsman; Tegally H; de Oliveira T
EPI_ISL_1534385, EPI_ISL_1534388, EPI_ISL_1534403	Knysna Hospital wc KNY	NHLS/UCT	Arash Iranzadeh; Bruna Galvao; Carolyn Williamson; Deelan Doolabh; Diana Hardie; Emmanuel SJ; Innocent Mudau; Kruger Marais; Lynn Tyers; Marvin Hsiao; Stephen Korsman; Tegally H; de Oliveira T
EPI_ISL_419296, EPI_ISL_479823	Kochi Prefectural Institute of Public Health	Pathogen Genomics Center, National Institute of Infectious Diseases	Akihiko Tokaji; Hajime Kamiya; Kentaro Itokawa; Makoto Kuroda; Masanori Hashino; Motoi Suzuki; Rina Tanaka; Tsuyoshi Sekizuka
EPI_ISL_407193	Korea Centers for Disease Control & Prevention (KCDC) Center for Laboratory Control of Infectious Diseases Division of Viral Diseases	Korea Centers for Disease Control & Prevention (KCDC) Center for Laboratory Control of Infectious Diseases Division of Viral Diseases	Heui Man Kim; Hye-Joon Jo; Jeong-Min Kim; Mi-Seon Kim; Myung Guk Han; Namjoo Lee; SangHee Woo; Sehee Park; Yoon-Seok Chung
EPI_ISL_480103 to 480108, EPI_ISL_480221 to 480223	Koshigaya City Public Health Center	Pathogen Genomics Center, National Institute of Infectious Diseases	Aya Tamura; Hajime Kamiya; Kentaro Itokawa; Kyohei Sakata; Makoto Kuroda; Masanori Hashino; Motoi Suzuki; Rina Tanaka; Takumi Daimon; Tsuyoshi Sekizuka; Yoko Togawa; Yoshiko Hamada; Yuka Furui
EPI_ISL_434657	Kristianstadkliniken	The Public Health Agency of Sweden	Anna Risberg; Anna-Malin Linde; Karin Tegmark-Wisell; Maria Lind Karlberg; Mia Brytting; Mia Settergren Hammer; Olov Svartstrom; Oskar Karlsson Lindsjo; Theresa Enkirch
EPI_ISL_434651	Krokoms Halsocentral	The Public Health Agency of Sweden	Anna Risberg; Anna-Malin Linde; Karin Tegmark-Wisell; Maria Lind Karlberg; Martin Ersson; Mia Brytting; Olov Svartstrom; Oskar Karlsson Lindsjo; Theresa Enkirch
EPI_ISL_479991 to 479996	Kumamoto City Public Health Research Institute	Pathogen Genomics Center, National Institute of Infectious	Hajime Kamiya; Kaori Tashiro; Kentaro Itokawa; Makoto Kuroda; Masanori Hashino; Motoi Suzuki; Rina Tanaka; Tsuyoshi Sekizuka

		Diseases	
EPI_ISL_479824	Kumamoto PrefecturalInstituteof Public-Health and Environmental Science	Pathogen Genomics Center, National Institute of Infectious Diseases	Hajime Kamiya; Kentaro Itokawa; Makoto Kuroda; Masanori Hashino; Motoi Suzuki; Rina Tanaka; Shunsuke Yahiro; Tsuyoshi Sekizuka
EPI_ISL_515181 to 515184, EPI_ISL_515247 to 515249	Kumasi Centre for Collaborative Research in Tropical Medicine, Kumasi.	Institute of Virology, Charité - Universitätsmedizin Berlin	Augustina Sylverken; Christian Drosten; Eric Adu; Jesse Addo Asamoah; Julia Schneider; Jörn Beheim-Schwarzbach; Michael Owusu; Philip El-Duah; Richard Phillips.; Richmond Gorman; Richmond Yeboah; Sherihane Aryeetey; Victor Max Corman
EPI_ISL_539881	Kungsbacka Narakut	The Public Health Agency of Sweden	Anna Risberg; Anna-Malin Linde; Karin Tegmark-Wisell; Maria Lind Karlberg; Mattias Haukland; Mia Brytting; Olov Svartstrom; Oskar Karlsson Lindsjo; Reza Advani; Sandra Broddesson; Theresa Enkirch
EPI_ISL_434644, EPI_ISL_450809, EPI_ISL_469067, EPI_ISL_475542, EPI_ISL_475569	Kungsholmsdoktorn	The Public Health Agency of Sweden	Anna Risberg; Anna-Malin Linde; Karin Tegmark-Wisell; Linus Hammar; Maria Lind Karlberg; Mattias Haukland; Mia Brytting; Olov Svartstrom; Oskar Karlsson Lindsjo; Petra Edquist; Reza Advani; Sandra Broddesson; Shamam Muradrasoli; Theresa Enkirch
EPI_ISL_434668, EPI_ISL_445232, EPI_ISL_455102, EPI_ISL_475529 to 475532, EPI_ISL_475568	Kungsors VC	The Public Health Agency of Sweden	Anna Risberg; Anna-Malin Linde; Jessica Karlsson; Karin Tegmark-Wisell; Maria Lind Karlberg; Mattias Haukland; Mia Brytting; Olov Svartstrom; Oskar Karlsson Lindsjo; Reza Advani; Sandra Broddesson; Theresa Enkirch
EPI_ISL_1398353, EPI_ISL_1398369	Kuningan Public Health	West Java Health Laboratory; School of Life Sciences and Technology, Institut Teknologi Bandung	Azzania Fibriani; Cut Nur Cinthia Alamanda; Ema Rahmawati; Isak Solihin; Kamila Tania; Karimatu Khoirunnisa; Miftahul Faridl; Rifky Waluyajati Rachman; Rini Robiani; Ryan Bayusantika Ristandi
EPI_ISL_539805	Kwong Wah Hospital	Hong Kong Department of Health	Alan K.L. Tsang; Dominic N.C. Tsang; Edman T.K. Lam; Peter C.W. Yip; Rickjason C.W. Chan
EPI_ISL_420039 to 420040	L'Air du Temps	National Reference Center for Viruses of Respiratory Infections, Institut Pasteur, Paris	Angela Brisebarre; Etienne Simon-Lorière; Flora Donati; Marion Barbet; Maud Vanpeene; Mélanie Albert; Méline Bizard; Sylvie Behillil; Sylvie van der Werf; Vincent Enouf
EPI_ISL_508931	L'Hôpital Nord-Ouest Tarare-Grandris	CNR Virus des Infections Respiratoires - France SUD	Alexandre Gaymard; Antonin Bal; Bruno Lina; Carine Moustaud; Florence Morfin-Sherpa; Gregory Destras; Gwendolyne Burfin; Laurence Josset; Martine Valette; Maude Bouscambert-Duchamp; Raphaelle Lamy; Solenne Brun
EPI_ISL_1446591, EPI_ISL_1446593, EPI_ISL_1447013 to 1447017, EPI_ISL_1516401 to 1516423, EPI_ISL_1528475 to 1528477	LA Office of Public Health Laboratories	Centers for Disease Control and Prevention Division of Viral Diseases, Pathogen Discovery	Alison Laufer Halpin; Ben L. Rambo-Martin; Clinton R. Paden; Dakota Howard; Darlene Wagner; Dave Wentworth; Dhwani Batra; Jasmine Padilla; Justin Lee; Katie Dillon; Krista Queen; Kristen Knipe; Kristine Lacek; Mark Burroughs; Matthew Schmerer; Mili Sheth; Peter Cook; Sam Shepard; Sarah Nobles; Shoshona Le; Suxiang Tong; Vivien Dugan; Yvette Unoarumhi
EPI_ISL_1448522, EPI_ISL_1509932	LA Office of Public Health Laboratories	Genomics and Discovery, Respiratory Viruses Branch, Division of Viral Diseases, Centers for Disease Control and Prevention	Adam Retchless; Anna Kelleher; Anna Montmayeur; Anna Uehara; Brian Lynch; Clinton R. Paden; Haibin Wang; Han Jia Justin Ng; Jing Zhang; Justin Lee; Krista Queen; Mark Burroughs; Peter Cook; Rachel Marine; Suxiang Tong; Yan Li; Ying Tao
EPI_ISL_424868, EPI_ISL_452106 to 452107, EPI_ISL_535305 to 535358	LA Office of Public Health Laboratories	Pathogen Discovery, Respiratory Viruses Branch, Division of Viral Diseases, Centers for Disease Control and Prevention	Alison S. Laufer Halpin; Anna Montmayeur; Anna Uehara; Christopher A. Elkins; Clinton Paden; Clinton R. Paden; Haibin Wang; Jing Zhang; Krista Queen; Mary S. Keckler; Rachel Marine; Suxiang Tong; Yan Li; Ying Tao; Zachary Weiner
EPI_ISL_1396823	LABM BIOLAM CLINIQUE ESTREE	Department of Virology, Henri Mondor University Hospital, Assistance Publique Hôpitaux de Paris, Université Paris-Est Créteil, INSERM U955	Alexandre Soulier; Christophe Rodriguez; Elisabeth Trawinski; Guillaume Gricourt; Jean-Michel Pawlotsky; Melissa N'Debi; Slim Fourati; Vanessa Demontant
EPI_ISL_416499 to 416500, EPI_ISL_418234, EPI_ISL_418240	LABM GH nord Essonne	National Reference Center for Viruses of Respiratory Infections, Institut Pasteur, Paris	Angela Brisebarre; Christine Lambert; Etienne Simon-Lorière; Flora Donati; Marion Barbet; Maud Vanpeene; Mélanie Albert; Méline Bizard; Mélnie Albert; Sylvie Behillil; Sylvie van der Werf; Vincent Enouf
EPI_ISL_428354, EPI_ISL_428361 to 428362, EPI_ISL_428365, EPI_ISL_443260, EPI_ISL_443305, EPI_ISL_443314	LABM GH nord Essonne de Longjumeau - BP 125	National Reference Center for Viruses of Respiratory Infections, Institut Pasteur, Paris	Angela Brisebarre; Etienne Simon-Lorière; Flora Donati; Marion Barbet; Maud Vanpeene; Mélanie Albert; Méline Bizard; Sylvie Behillil; Sylvie van der Werf; Vincent Enouf
EPI_ISL_1418252 to 1418254	LABORATOIRE BOUVIER	CNR Virus des Infections Respiratoires - France SUD	Antonin Bal; Bruno Lina; Gregory Destras; Gwendolyne Burfin; Hadrien Regue; Laurence Josset; Martine Valette; Quentin Semanas
EPI_ISL_1526389 to 1526390, EPI_ISL_1526396 to 1526400	LABORATOIRE d'ANALYSES de BIOLOGIE MEDICALES	CNR Virus des Infections Respiratoires - France SUD	Antonin Bal; Bruno Lina; Gregory Destras; Gwendolyne Burfin; Hadrien Regue; Laurence Josset; Martine Valette; Quentin Semanas
EPI_ISL_1393933 to 1393941, EPI_ISL_1394255 to 1394259, EPI_ISL_1425559, EPI_ISL_1538594 to 1538597, EPI_ISL_1538828 to 1538829, EPI_ISL_1538831, EPI_ISL_1538936 to 1538937, EPI_ISL_1539078 to 1539079			
see above	LABORATORIA MEDYCZNE OPTIMED	1. National Institute of Public Health - National Institute of Hygiene; 2. Eurofins Genomics Europe Sequencing GmbH	ECDC COVID-19 WGS support team; Eurofins Genomics Europe Sequencing Team; Gierczyki Rafa; Sadkowska-Todys Magorzata; Wokowicz Tomasz; Zacharczuk Katarzyna
EPI_ISL_445271	LABORATORIO CLINICA CHILLAN	Instituto de Salud Publica de Chile	Alejandra Acevedo; Andrés E Castillo; Bárbara Parra; Carolina Tambley; Gabriel Leal; Jaime Lagos; Jorge Fernandez; Loredana Arata; Patricia Bustos; Paz Tapia; Rodrigo Fasce; Winston Andrade
EPI_ISL_445333	LABORATORIO CLINICA UNIVERSITARIA DE CONCEPCION	Instituto de Salud Publica de Chile	Alejandra Acevedo; Andrés E Castillo; Bárbara Parra; Carolina Tambley; Gabriel Leal; Jaime Lagos; Jorge Fernandez; Loredana Arata; Patricia Bustos; Paz Tapia; Rodrigo Fasce; Winston Andrade
EPI_ISL_1494945 to 1494946	LABORATORIO CLINICO IMAT SAS	Instituto Nacional de Salud- Dirección de Investigación en Salud Pública	Carlos Franco-Muñoz; Carmen Osorio; Diana Malo; Diego A. Álvarez-Díaz; Diego Andrés Prada; Gerardo Santamaría; Hector Alejandro Ruiz-Moreno; Jhonnatan Reales-González; Juan Camilo Martinez; Julian Naizaque; Katherine Laiton-Donato; Lisseth Pardo; Magdalena Wiesner; Marcela Mercado-Reyes; Maria T. Herrera-Sepúlveda; Marta Lopez Blanco; Martha Lucia Ospina Martinez; Paola Rojas; Sergio Gomez; Sheryll Corchuelo; Ángela Alarcon Cruz
EPI_ISL_1445155	LABORATORIO DE FRANCA	Instituto Butantan / Mendelics	Antonio Jorge Martins; Bibiana Santos; Claudia Renata dos Santos Barros; David Schlesinger; Debora Botequio Moretti; Dimas Tadeu Covas; Elaine Cristina Marqueze; Elaine Vieira dos Santos; Erika Freitas; Evandra Strazza Rodrigues; Flavia Aburjaile; José Salvatore Leister Patané; João Paulo Kitajima; Luiz Carlos Junior de Alcantara; Maria Carolina Elias; Marta Giovanetti; Rafael dos Santos Bezerra; Raul Machado Neto; Ricardo Haddad; Rodrigo Tocantins Calado.; Sandra Coccuzzo Sampaio; Simone Kashima; Svetoslav Nanev Slavov; Vagner Fonseca; Vincent Louis Viala
EPI_ISL_445279	LABORATORIO INMUNOLAB SPA	Instituto de Salud Publica de Chile	Alejandra Acevedo; Andrés E Castillo; Bárbara Parra; Carolina Tambley; Gabriel Leal; Jaime Lagos; Jorge Fernandez; Loredana Arata; Patricia Bustos; Paz Tapia; Rodrigo Fasce; Winston Andrade
EPI_ISL_445273 to 445274, EPI_ISL_445278	LABORATORIO TORRE MEDICA LTDA.	Instituto de Salud Publica de Chile	Alejandra Acevedo; Andrés E Castillo; Bárbara Parra; Carolina Tambley; Gabriel Leal; Jaime Lagos; Jorge Fernandez; Loredana Arata; Patricia Bustos; Paz Tapia; Rodrigo Fasce; Winston Andrade
EPI_ISL_1393903 to 1393922, EPI_ISL_1393962 to 1393983, EPI_ISL_1538436, EPI_ISL_1538456, EPI_ISL_1538471, EPI_ISL_1538479, EPI_ISL_1538486, EPI_ISL_1538499, EPI_ISL_1538512, EPI_ISL_1538582 to 1538589, EPI_ISL_1538694 to 1538706, EPI_ISL_1538854, EPI_ISL_1538872, EPI_ISL_1538920 to 1538924, EPI_ISL_1539005 to 1539012, EPI_ISL_1539108 to 1539109, EPI_ISL_1539135 to 1539136, EPI_ISL_1539199, EPI_ISL_1539213 to 1539214			
see above	LABORATORIUM BADA KLINICZNYCH WSSE w OPOLU	1. National Institute of Public Health - National Institute of Hygiene; 2. Eurofins Genomics Europe Sequencing GmbH	ECDC COVID-19 WGS support team; Eurofins Genomics Europe Sequencing Team; Gierczyki Rafa; Sadkowska-Todys Magorzata; Wokowicz Tomasz; Zacharczuk Katarzyna
EPI_ISL_1538473, EPI_ISL_1538788 to 1538789	LABORATORIUM GENETYCZNE SALVE MEDICA	1. National Institute of Public Health - National Institute of Hygiene; 2. Eurofins Genomics Europe Sequencing GmbH	ECDC COVID-19 WGS support team; Eurofins Genomics Europe Sequencing Team; Gierczyki Rafa; Sadkowska-Todys Magorzata; Wokowicz Tomasz; Zacharczuk Katarzyna
EPI_ISL_1468413 to 1468415, EPI_ISL_1468431, EPI_ISL_1493573 to 1493577	LACEN do Estado de Goias	Instituto Adolfo Lutz, Interdiciplinary Procedures Center, Strategic Laboratory	Caio Vinicius Dias Lopes; Claudia Regina Gonçalves; Claudio Tavares Sacchi; Erica Valessa Ramos Gomes; Karoline Rodrigues Campos
EPI_ISL_1493578 to 1493579, EPI_ISL_1493583 to 1493584, EPI_ISL_1493595 to 1493600, EPI_ISL_1494924, EPI_ISL_1520107 to 1520109	LACEN do Estado de Rondonia	Instituto Adolfo Lutz, Interdiciplinary Procedures Center, Strategic Laboratory	Caio Vinicius Dias Lopes; Claudia Regina Gonçalves; Claudio Tavares Sacchi; Erica Valessa Ramos Gomes; Karoline Rodrigues Campos
EPI_ISL_1468432 to 1468441	LACEN do Mato Grosso do Sul	Instituto Adolfo Lutz, Interdiciplinary Procedures Center, Strategic Laboratory	Caio Vinicius Dias Lopes; Claudia Regina Gonçalves; Claudio Tavares Sacchi; Erica Valessa Ramos Gomes; Karoline Rodrigues Campos
EPI_ISL_502779, EPI_ISL_502875	LACEN/PE	LABBE, Federal University of Pernambuco	ANTONIO CARLOS DE FREITAS; BRUNO SAMPAIO; HEIDI LACERDA ALVES DA CRUZ; MAIRA GALDINO DA ROCHA PITTA; MARCOS ANTONIO

			DE MORAIS JUNIOR; MARCOS DA SILVEIRA REGUEIRA NETO; MICHELLY CRISTINY PEREIRA; REGINALDO GONCALVES DE LIMA NETO; SERGIO DE SA LEITAO PAIVA JUNIOR; VALDIR DE QUEIROZ BALBINO.; WILSON JOSE DA SILVA JUNIOR; ZILDENE DE SOUSA SILVEIRA
EPI_ISL_1524886	LADR GmbH MVZ Dr. Kramer & Kollegen Geesthacht	Hannover Medical School, Institute of Virology	Lars Steinbrück
EPI_ISL_1418192, EPI_ISL_1418194 to 1418200	LAM AVERON LABO RODEZ	CNR Virus des Infections Respiratoires - France SUD	Antonin Bal; Bruno Lina; Gregory Destras; Gwendolyne Burfin; Hadrien Regue; Laurence Josset; Martine Valette; Quentin Semanas
EPI_ISL_1461110	LAM BAYARD-BIOPYRENEES	CNR Virus des Infections Respiratoires - France SUD	Antonin Bal; Bruno Lina; Gregory Destras; Gwendolyne Burfin; Hadrien Regue; Laurence Josset; Martine Valette; Quentin Semanas
EPI_ISL_1461052	LAM COLONNA DE CINARCA	CNR Virus des Infections Respiratoires - France SUD	Antonin Bal; Bruno Lina; Gregory Destras; Gwendolyne Burfin; Hadrien Regue; Laurence Josset; Martine Valette; Quentin Semanas
EPI_ISL_1418218, EPI_ISL_1418220 to 1418228	LAM GEN-BIO GRAVANCHES	CNR Virus des Infections Respiratoires - France SUD	Antonin Bal; Bruno Lina; Gregory Destras; Gwendolyne Burfin; Hadrien Regue; Laurence Josset; Martine Valette; Quentin Semanas
EPI_ISL_1418255	LAM VAL DE GARONNE	CNR Virus des Infections Respiratoires - France SUD	Antonin Bal; Bruno Lina; Gregory Destras; Gwendolyne Burfin; Hadrien Regue; Laurence Josset; Martine Valette; Quentin Semanas
EPI_ISL_1446194	LATE - Laboratório de Técnicas Especiais - Hospital Israelita Albert Einstein	LATE - Laboratório de Técnicas Especiais - Hospital Israelita Albert Einstein	Ana Paula Moreira Salles; Deyvid Amgarten; Fernanda de Mello Malta; João Renato Rebello Pinho; Pedro Henrique Sebe Rodrigues; Raquel Riyuzo
EPI_ISL_1403498	LBA Casteljaloux	Cerba Lab	Benazra M; Haïm-Boukobza S; Lecorche E; Olivi M; Roquebert B; Trombert-Paolantoni S; Zimmer S
EPI_ISL_1547827	LBM ALPHABIO	LBM ALPHABIO	PHILIPPE HALFON; VINCENT GARCIA
EPI_ISL_1461182 to 1461187, EPI_ISL_1461189, EPI_ISL_1526264, EPI_ISL_1526268 to 1526271, EPI_ISL_1526273 to 1526276, EPI_ISL_1526278 to 1526280, EPI_ISL_1526282 to 1526286, EPI_ISL_1526288 to 1526298, EPI_ISL_1526300 to 1526312, EPI_ISL_1526314 to 1526316, EPI_ISL_1526318 to 1526326			
see above	LBM UNIBIO VALENTIN	CNR Virus des Infections Respiratoires - France SUD	Antonin Bal; Bruno Lina; Gregory Destras; Gwendolyne Burfin; Hadrien Regue; Laurence Josset; Martine Valette; Quentin Semanas
EPI_ISL_1398204 to 1398208	LENOX HILL HOSPITAL	Wadsworth Center, New York State Department of Health	Alexis Russel; Daryl M. Lamson; Erasmus Schneider; Erica Lasek-Nesselquist; John Kelly; Jonathan Plitnick; Kirsten St. George; Matthew Shudt; Melissa A Leisner; Navjot Singh
EPI_ISL_1516777 to 1516786	LESP Aguascalientes	Instituto de Diagnostico y Referencia Epidemiologicos (INDRE)	Abril Rodriguez-Maldonado; Ariadna Medina-Benitez; Claudia Wong-Arambula; Ernesto Ramirez-Gonzalez.; Gisela Barrera-Badillo; Irma Lopez-Martinez; Joaquin Quiroz-Mercado; Lucia Hernandez-Rivas; Natividad Cruz-Ortiz; Sergio Rangel-Guerrero; Tatiana Nunez-Garcia; Vanessa Rivero-Arredondo
EPI_ISL_1399280, EPI_ISL_1424050, EPI_ISL_1504108, EPI_ISL_1532264, EPI_ISL_1558798	LESP Baja California	Instituto de Diagnostico y Referencia Epidemiologicos (INDRE)	Abril Rodriguez-Maldonado; Ariadna Medina-Benitez; Claudia Wong-Arambula; Ernesto Ramirez-Gonzalez.; Gisela Barrera-Badillo; Irma Lopez-Martinez; Joaquin Quiroz-Mercado; Lucia Hernandez-Rivas; Natividad Cruz-Ortiz; Sergio Rangel-Guerrero; Tatiana Nunez-Garcia; Vanessa Rivero-Arredondo
EPI_ISL_1504105, EPI_ISL_1532243 to 1532245, EPI_ISL_1558801	LESP Baja California Sur	Instituto de Diagnostico y Referencia Epidemiologicos (INDRE)	Abril Rodriguez-Maldonado; Ariadna Medina-Benitez; Claudia Wong-Arambula; Ernesto Ramirez-Gonzalez.; Gisela Barrera-Badillo; Irma Lopez-Martinez; Joaquin Quiroz-Mercado; Lucia Hernandez-Rivas; Natividad Cruz-Ortiz; Sergio Rangel-Guerrero; Tatiana Nunez-Garcia; Vanessa Rivero-Arredondo
EPI_ISL_1400299, EPI_ISL_1424015, EPI_ISL_1483066 to 1483067	LESP Campeche	Instituto de Diagnostico y Referencia Epidemiologicos (INDRE)	Abril Rodriguez-Maldonado; Ariadna Medina-Benitez; Claudia Wong-Arambula; Ernesto Ramirez-Gonzalez.; Gisela Barrera-Badillo; Irma Lopez-Martinez; Joaquin Quiroz-Mercado; Lucia Hernandez-Rivas; Natividad Cruz-Ortiz; Sergio Rangel-Guerrero; Tatiana Nunez-Garcia; Vanessa Rivero-Arredondo
EPI_ISL_1424013, EPI_ISL_1424020, EPI_ISL_1532246 to 1532247	LESP Chiapas	Instituto de Diagnostico y Referencia Epidemiologicos (INDRE)	Abril Rodriguez-Maldonado; Ariadna Medina-Benitez; Claudia Wong-Arambula; Ernesto Ramirez-Gonzalez.; Gisela Barrera-Badillo; Irma Lopez-Martinez; Joaquin Quiroz-Mercado; Lucia Hernandez-Rivas; Natividad Cruz-Ortiz; Sergio Rangel-Guerrero; Tatiana Nunez-Garcia; Vanessa Rivero-Arredondo
EPI_ISL_1399271, EPI_ISL_1405905, EPI_ISL_1423506 to 1423509, EPI_ISL_1423512, EPI_ISL_1483074, EPI_ISL_1494626 to 1494630, EPI_ISL_1504084 to 1504088, EPI_ISL_1516766 to 1516768, EPI_ISL_1516816, EPI_ISL_1516818 to 1516823, EPI_ISL_1558791 to 1558793			
see above	LESP Chihuahua	Instituto de Diagnostico y Referencia Epidemiologicos (INDRE)	Abril Rodriguez-Maldonado; Ariadna Medina-Benitez; Claudia Wong-Arambula; Ernesto Ramirez-Gonzalez.; Gisela Barrera-Badillo; Irma Lopez-Martinez; Joaquin Quiroz-Mercado; Lucia Hernandez-Rivas; Natividad Cruz-Ortiz; Sergio Rangel-Guerrero; Tatiana Nunez-Garcia; Vanessa Rivero-Arredondo
EPI_ISL_1399281, EPI_ISL_1423510, EPI_ISL_1424051 to 1424053, EPI_ISL_1469941, EPI_ISL_1494636 to 1494647, EPI_ISL_1504083, EPI_ISL_1516787 to 1516788, EPI_ISL_1516817, EPI_ISL_1532265, EPI_ISL_1532279			
see above	LESP Ciudad de Mexico	Instituto de Diagnostico y Referencia Epidemiologicos (INDRE)	Abril Rodriguez-Maldonado; Ariadna Medina-Benitez; Claudia Wong-Arambula; Ernesto Ramirez-Gonzalez.; Gisela Barrera-Badillo; Irma Lopez-Martinez; Joaquin Quiroz-Mercado; Lucia Hernandez-Rivas; Natividad Cruz-Ortiz; Sergio Rangel-Guerrero; Tatiana Nunez-Garcia; Vanessa Rivero-Arredondo
EPI_ISL_1399278, EPI_ISL_1424044 to 1424045, EPI_ISL_1516791 to 1516793, EPI_ISL_1532280	LESP Coahuila	Instituto de Diagnostico y Referencia Epidemiologicos (INDRE)	Abril Rodriguez-Maldonado; Ariadna Medina-Benitez; Claudia Wong-Arambula; Ernesto Ramirez-Gonzalez.; Gisela Barrera-Badillo; Irma Lopez-Martinez; Joaquin Quiroz-Mercado; Lucia Hernandez-Rivas; Natividad Cruz-Ortiz; Sergio Rangel-Guerrero; Tatiana Nunez-Garcia; Vanessa Rivero-Arredondo
EPI_ISL_1532249	LESP Estado de Mexico	Instituto de Diagnostico y Referencia Epidemiologicos (INDRE)	Abril Rodriguez-Maldonado; Ariadna Medina-Benitez; Claudia Wong-Arambula; Ernesto Ramirez-Gonzalez.; Gisela Barrera-Badillo; Irma Lopez-Martinez; Joaquin Quiroz-Mercado; Lucia Hernandez-Rivas; Natividad Cruz-Ortiz; Sergio Rangel-Guerrero; Tatiana Nunez-Garcia; Vanessa Rivero-Arredondo
EPI_ISL_1399260 to 1399261, EPI_ISL_1400304, EPI_ISL_1400314 to 1400349, EPI_ISL_1405900 to 1405904, EPI_ISL_1405906 to 1405907, EPI_ISL_1405909 to 1405910, EPI_ISL_1405912, EPI_ISL_1405914 to 1405915, EPI_ISL_1424010 to 1424012, EPI_ISL_1424034, EPI_ISL_1469942 to 1469944, EPI_ISL_1483048, EPI_ISL_1494657 to 1494669, EPI_ISL_1516794, EPI_ISL_1516796, EPI_ISL_1532238			
see above	LESP Guanajuato	Instituto de Diagnostico y Referencia Epidemiologicos (INDRE)	Abril Rodriguez-Maldonado; Ariadna Medina-Benitez; Claudia Wong-Arambula; Ernesto Ramirez-Gonzalez.; Gisela Barrera-Badillo; Irma Lopez-Martinez; Joaquin Quiroz-Mercado; Lucia Hernandez-Rivas; Natividad Cruz-Ortiz; Sergio Rangel-Guerrero; Tatiana Nunez-Garcia; Vanessa Rivero-Arredondo
EPI_ISL_1424043, EPI_ISL_1483073, EPI_ISL_1504104, EPI_ISL_1532240 to 1532242	LESP Guerrero	Instituto de Diagnostico y Referencia Epidemiologicos (INDRE)	Abril Rodriguez-Maldonado; Ariadna Medina-Benitez; Claudia Wong-Arambula; Ernesto Ramirez-Gonzalez.; Gisela Barrera-Badillo; Irma Lopez-Martinez; Joaquin Quiroz-Mercado; Lucia Hernandez-Rivas; Natividad Cruz-Ortiz; Sergio Rangel-Guerrero; Tatiana Nunez-Garcia; Vanessa Rivero-Arredondo
EPI_ISL_1424008 to 1424009, EPI_ISL_1483058, EPI_ISL_1494655 to 1494656, EPI_ISL_1516763	LESP Hidalgo	Instituto de Diagnostico y Referencia Epidemiologicos (INDRE)	Abril Rodriguez-Maldonado; Ariadna Medina-Benitez; Claudia Wong-Arambula; Ernesto Ramirez-Gonzalez.; Gisela Barrera-Badillo; Irma Lopez-Martinez; Joaquin Quiroz-Mercado; Lucia Hernandez-Rivas; Natividad Cruz-Ortiz; Sergio Rangel-Guerrero; Tatiana Nunez-Garcia; Vanessa Rivero-Arredondo
EPI_ISL_1399259, EPI_ISL_1399270, EPI_ISL_1400298, EPI_ISL_1405908, EPI_ISL_1405911, EPI_ISL_1405913, EPI_ISL_1469937, EPI_ISL_1483068 to 1483072, EPI_ISL_1504109, EPI_ISL_1516765, EPI_ISL_1516795, EPI_ISL_1532266 to 1532270, EPI_ISL_1558799 to 1558800			
see above	LESP Jalisco	Instituto de Diagnostico y Referencia Epidemiologicos (INDRE)	Abril Rodriguez-Maldonado; Ariadna Medina-Benitez; Claudia Wong-Arambula; Ernesto Ramirez-Gonzalez.; Gisela Barrera-Badillo; Irma Lopez-Martinez; Joaquin Quiroz-Mercado; Lucia Hernandez-Rivas; Natividad Cruz-Ortiz; Sergio Rangel-Guerrero; Tatiana Nunez-Garcia; Vanessa Rivero-Arredondo
EPI_ISL_1492456	LESP Jalisco	Instituto de diagnóstico y Referencia Epidemiologicos (INDRE)	Abril Rodriguez-Maldonado; Ariadna Medina-Benitez; Claudia Wong-Arambula; Ernesto Ramirez-Gonzalez.; Gisela Barrera-Badillo; Irma Lopez-Martinez; Joaquin Quiroz-Mercado; Lucia Hernandez-Rivas; Natividad Cruz-Ortiz; Sergio Rangel-Guerrero; Tatiana Nunez-Garcia; Vanessa Rivero-Arredondo
EPI_ISL_1516762, EPI_ISL_1558828 to 1558833	LESP Jalisco/UdeG	Instituto de Diagnostico y Referencia Epidemiologicos (INDRE)	Abril Rodriguez-Maldonado; Ariadna Medina-Benitez; Claudia Wong-Arambula; Ernesto Ramirez-Gonzalez.; Gisela Barrera-Badillo; Irma Lopez-Martinez; Joaquin Quiroz-Mercado; Lucia Hernandez-Rivas; Natividad Cruz-Ortiz; Sergio Rangel-Guerrero; Tatiana Nunez-Garcia; Vanessa Rivero-Arredondo
EPI_ISL_1400300 to 1400303, EPI_ISL_1400305 to 1400309, EPI_ISL_1405899, EPI_ISL_1424017, EPI_ISL_1424019, EPI_ISL_1424042, EPI_ISL_1504068 to 1504082, EPI_ISL_1516808 to 1516815			
see above	LESP Morelos	Instituto de Diagnostico y Referencia Epidemiologicos (INDRE)	Abril Rodriguez-Maldonado; Ariadna Medina-Benitez; Claudia Wong-Arambula; Ernesto Ramirez-Gonzalez.; Gisela Barrera-Badillo; Irma Lopez-Martinez; Joaquin Quiroz-Mercado; Lucia Hernandez-Rivas; Natividad Cruz-Ortiz; Sergio Rangel-Guerrero; Tatiana Nunez-Garcia; Vanessa Rivero-Arredondo
EPI_ISL_1399273 to 1399274, EPI_ISL_1483040 to 1483041	LESP Nayarit	Instituto de Diagnostico y Referencia Epidemiologicos (INDRE)	Abril Rodriguez-Maldonado; Ariadna Medina-Benitez; Claudia Wong-Arambula; Ernesto Ramirez-Gonzalez.; Gisela Barrera-Badillo; Irma Lopez-Martinez; Joaquin Quiroz-Mercado; Lucia Hernandez-Rivas; Natividad Cruz-Ortiz; Sergio Rangel-Guerrero; Tatiana Nunez-Garcia; Vanessa Rivero-Arredondo
EPI_ISL_1399262 to 1399269, EPI_ISL_1469938 to 1469939, EPI_ISL_1483032 to 1483037, EPI_ISL_1494631 to 1494635, EPI_ISL_1494648 to 1494649, EPI_ISL_1494673, EPI_ISL_1504058 to 1504067, EPI_ISL_1516769 to 1516773, EPI_ISL_1516797 to 1516807, EPI_ISL_1532271			
see above	LESP Nuevo Leon	Instituto de Diagnostico y Referencia Epidemiologicos (INDRE)	Abril Rodriguez-Maldonado; Ariadna Medina-Benitez; Claudia Wong-Arambula; Ernesto Ramirez-Gonzalez.; Gisela Barrera-Badillo; Irma Lopez-Martinez; Joaquin Quiroz-Mercado; Lucia Hernandez-Rivas; Natividad Cruz-Ortiz; Sergio Rangel-Guerrero; Tatiana Nunez-Garcia; Vanessa Rivero-Arredondo
EPI_ISL_1400310 to 1400313, EPI_ISL_1483059 to 1483065, EPI_ISL_1516764	LESP Puebla	Instituto de Diagnostico y Referencia Epidemiologicos (INDRE)	Abril Rodriguez-Maldonado; Ariadna Medina-Benitez; Claudia Wong-Arambula; Ernesto Ramirez-Gonzalez.; Gisela Barrera-Badillo; Irma Lopez-Martinez; Joaquin Quiroz-Mercado; Lucia Hernandez-Rivas; Natividad Cruz-Ortiz; Sergio Rangel-Guerrero; Tatiana Nunez-Garcia; Vanessa Rivero-Arredondo
EPI_ISL_1399272, EPI_ISL_1423513, EPI_ISL_1424016, EPI_ISL_1424021 to 1424033, EPI_ISL_1483044 to 1483047, EPI_ISL_1483049 to 1483050, EPI_ISL_1504089 to 1504103, EPI_ISL_1504110 to 1504111, EPI_ISL_1516775, EPI_ISL_1516824 to 1516826, EPI_ISL_1532236 to 1532237, EPI_ISL_1532239, EPI_ISL_1532272 to 1532278, EPI_ISL_1558794 to 1558795			
see above	LESP Queretaro	Instituto de Diagnostico y Referencia Epidemiologicos (INDRE)	Abril Rodriguez-Maldonado; Ariadna Medina-Benitez; Claudia Wong-Arambula; Ernesto Ramirez-Gonzalez.; Gisela Barrera-Badillo; Irma Lopez-Martinez; Joaquin Quiroz-Mercado; Lucia Hernandez-Rivas; Natividad Cruz-Ortiz; Sergio Rangel-Guerrero; Tatiana Nunez-Garcia; Vanessa Rivero-Arredondo

EPI_ISL_1532281	LESP Quintana Roo	Instituto de Diagnostico y Referencia Epidemiologicos (INDRE)	Abril Rodriguez-Maldonado; Ariadna Medina-Benitez; Claudia Wong-Arambula; Ernesto Ramirez-Gonzalez.; Gisela Barrera-Badillo; Irma Lopez-Martinez; Joaquin Quiroz-Mercado; Lucia Hernandez-Rivas; Natividad Cruz-Ortiz; Sergio Rangel-Guerrero; Tatiana Nunez-Garcia; Vanessa Rivero-Arredondo
EPI_ISL_1469940, EPI_ISL_1483042 to 1483043, EPI_ISL_1494670 to 1494672	LESP Sinaloa	Instituto de Diagnostico y Referencia Epidemiologicos (INDRE)	Abril Rodriguez-Maldonado; Ariadna Medina-Benitez; Claudia Wong-Arambula; Ernesto Ramirez-Gonzalez.; Gisela Barrera-Badillo; Irma Lopez-Martinez; Joaquin Quiroz-Mercado; Lucia Hernandez-Rivas; Natividad Cruz-Ortiz; Sergio Rangel-Guerrero; Tatiana Nunez-Garcia; Vanessa Rivero-Arredondo
EPI_ISL_1399275 to 1399277, EPI_ISL_1423511, EPI_ISL_1424039 to 1424041, EPI_ISL_1494650 to 1494654, EPI_ISL_1516776, EPI_ISL_1516789 to 1516790	LESP Tamaulipas	Instituto de Diagnostico y Referencia Epidemiologicos (INDRE)	Abril Rodriguez-Maldonado; Ariadna Medina-Benitez; Claudia Wong-Arambula; Ernesto Ramirez-Gonzalez.; Gisela Barrera-Badillo; Irma Lopez-Martinez; Joaquin Quiroz-Mercado; Lucia Hernandez-Rivas; Natividad Cruz-Ortiz; Sergio Rangel-Guerrero; Tatiana Nunez-Garcia; Vanessa Rivero-Arredondo
EPI_ISL_1399279, EPI_ISL_1424018, EPI_ISL_1424046 to 1424049, EPI_ISL_1483055 to 1483057	LESP Veracruz	Instituto de Diagnostico y Referencia Epidemiologicos (INDRE)	Abril Rodriguez-Maldonado; Ariadna Medina-Benitez; Claudia Wong-Arambula; Ernesto Ramirez-Gonzalez.; Gisela Barrera-Badillo; Irma Lopez-Martinez; Joaquin Quiroz-Mercado; Lucia Hernandez-Rivas; Natividad Cruz-Ortiz; Sergio Rangel-Guerrero; Tatiana Nunez-Garcia; Vanessa Rivero-Arredondo
EPI_ISL_1423514, EPI_ISL_1424007, EPI_ISL_1424014, EPI_ISL_1424035 to 1424038, EPI_ISL_1483038 to 1483039, EPI_ISL_1516774, EPI_ISL_1532248			
see above	LESP Yucatan	Instituto de Diagnostico y Referencia Epidemiologicos (INDRE)	Abril Rodriguez-Maldonado; Ariadna Medina-Benitez; Claudia Wong-Arambula; Ernesto Ramirez-Gonzalez.; Gisela Barrera-Badillo; Irma Lopez-Martinez; Joaquin Quiroz-Mercado; Lucia Hernandez-Rivas; Natividad Cruz-Ortiz; Sergio Rangel-Guerrero; Tatiana Nunez-Garcia; Vanessa Rivero-Arredondo
EPI_ISL_1504106 to 1504107, EPI_ISL_1532250 to 1532263, EPI_ISL_1558796 to 1558797	LESPZacatecas	Instituto de Diagnostico y Referencia Epidemiologicos (INDRE)	Abril Rodriguez-Maldonado; Ariadna Medina-Benitez; Claudia Wong-Arambula; Ernesto Ramirez-Gonzalez.; Gisela Barrera-Badillo; Irma Lopez-Martinez; Joaquin Quiroz-Mercado; Lucia Hernandez-Rivas; Natividad Cruz-Ortiz; Sergio Rangel-Guerrero; Tatiana Nunez-Garcia; Vanessa Rivero-Arredondo
EPI_ISL_1524913 to 1524995	LHUB-ULB	Labo Klinische Biologie, UZA	Basil Britto Xavier; Christine Lammens; Herman Goossens; Jasmine Coppens; Marie Le Mercier; Veerle Matheeussen
EPI_ISL_1416764, EPI_ISL_1416771, EPI_ISL_1416777 to 1416778, EPI_ISL_1416780, EPI_ISL_1416792, EPI_ISL_1416797, EPI_ISL_1416802, EPI_ISL_1455661, EPI_ISL_1455665 to 1455666, EPI_ISL_1498264, EPI_ISL_1498282 to 1498288, EPI_ISL_1498290 to 1498293, EPI_ISL_1498296, EPI_ISL_1498300			
see above	LHUB-ULB	UAntwerp, Laboratory of Medical Microbiology	Basil Britto Xavier; Christine Lammens; Herman Goossens; Jasmine Coppens; Marie Le Mercier; Veerle Matheeussen
EPI_ISL_498226	LIC	LIC	LIC
EPI_ISL_469017, EPI_ISL_469049, EPI_ISL_469051 to 469054, EPI_ISL_482732 to 482740	LNR National Reference Laboratory, Mohammed VI University of Health Sciences	Medical Biotechnology Laboratory, Rabat Medical and Pharmacy School, Mohammed The Vth University in Rabat	Chakib NEJJARI; Houda BENRAHMA; Idrissa DIAWARA; Idrissa Diawara; Imane SMYEJ; Jalil El ATAR; Jalil El Atar; Jalila RAHOUI; Lahcen BELYAMANI and Azeddine IBRAHIMI; Laila SBABOU; Laila SBABOU; Loubna ALLAM; M.W. CHEMAO-ELFIHRI; Meriem LAAMARTI; Mouna OUADGHIRI; Rachid EL JAOUDI; Rachid MENTAG; Rokaia LAAMRTI; Rokia LAAMARTI; Saaid AMZAZI; Souad KARTTI
EPI_ISL_1469988	LSUHS Emerging Viral Threat Laboratory	LSUHS Emerging Viral Threat Laboratory	Alexander J. Mijalis; Andrew D. Yurochko; Christopher G. Kevil; Gregory L. Ware; Jennifer L. Carroll; Jeremy P. Kamil; John A. Vanchiere; Maarten Van Diest; Rona S. Scott
EPI_ISL_435034 to 435044, EPI_ISL_435550 to 435579, EPI_ISL_451203 to 451297, EPI_ISL_455980 to 456002, EPI_ISL_574295 to 574430, EPI_ISL_578698 to 578700, EPI_ISL_578704 to 579057			
see above	LSUHS Emerging Viral Threat Laboratory	Microbial Genome Sequencing Center	Abida Siddiqa; Adam Greer; Andrew D. Yurochko; Byeong-Jae Lee; Camille F. Abshire; Chan-ki Min; Christopher G. Kevil; Daniel J. Snyder; Edna Ondari; Jason M. Bodily; Jeremy P. Kamil; John A. Vanchiere; Katarzyna Zwolinska; Maarten Van Diest; Malgorzata Bienkowska-Haba; Martin J. Sapp; Md Maksudul Alam; Michelle M. Arnold; Monica Gestal-Cartele; Paul M. Weinberger; Rona S. Scott; Vaughn S. Cooper
EPI_ISL_528637 to 528638	LVM/UFRJ	Bioinformatics Laboratory / LNCC	Amilcar Tanuri; Ana Teresa R. Vasconcelos; Bruno B. Bezerra; Diana Marianni; Elena Cobos; Fabio Limonte; Gustavo D. P. Silva; Isadora A. Correa; Luciana B. Arruda; Luciana J. Costa; Lucio A. Caldas; Luiz Almeida; Luiza Higga; M. Romário M. de Souza; Marcelo Bozza; Orlando Ferreira; Sharton V. A. Coelho; Terezinha M. Castineiras; Wanderley de Souza
EPI_ISL_528539	LVM/UFRJ	LNCC	Amilcar Tanuri; Ana Teresa R. Vasconcelos; Bruno B. Bezerra; Diana Marianni; Elena Cobos; Fabio Limonte; Gustavo M. Romário M. de Souza; Isadora A. Correa; Luciana B. Arruda; Luciana J. Costa.; Lucio A. Caldas; Luiz Almeida; Luiza Higga; Marcelo Bozza; Orlando Ferreira; Sharton V. A. Coelho; Terezinha M. Castineiras; Wanderley de Souza
EPI_ISL_443307	La Villa Papyri	National Reference Center for Viruses of Respiratory Infections, Institut Pasteur, Paris	Angela Brisebarre; Etienne Simon-Lorière; Flora Donati; Marion Barbet; Maud Vanpeene; Mélanie Albert; Méline Bizard; Sylvie Behillil; Sylvie van der Werf; Vincent Enouf
EPI_ISL_1469249, EPI_ISL_1469251	Lab Intibios Karawang	National Institute of Health Research and Development	Arie Ardiansyah Nugraha; Hana Apsari Pawestri; Hartanti Dian Ikawati; Kartika Dewi Puspa; Krisna Pangesti; Nelly Puspandari; Subangkit; Vivi Setiawaty
EPI_ISL_501614 to 501631	Lab Microbiology, Pathology Department, William Harvey Hospital	Wellcome Sanger Institute for the COVID-19 Genomics UK (COG-UK) consortium	Cordelia Langford; David K. Jackson; Dominic Kwiatkowski; Ewan Harrison; Felicity Ryan and Alex Alderton; Hannah Lowe; Ian Johnston; John Sillitoe on behalf of the Wellcome Sanger Institute COVID-19 Surveillance Team (http://www.sanger.ac.uk/covid-team); Roberto Amato; Samuel Moses; Sonia Goncalves
EPI_ISL_1404169 to 1404248, EPI_ISL_1443345 to 1443440, EPI_ISL_1532856 to 1532981, EPI_ISL_1532983 to 1533033	Lab voor klinische biologie	Lab voor klinische biologie	Bruno Verhasselt; Hannelore Hamerlinck; Marija Janevska
EPI_ISL_419259, EPI_ISL_419264 to 419266, EPI_ISL_425048, EPI_ISL_425050 to 425064, EPI_ISL_434347 to 434365, EPI_ISL_451164 to 451182, EPI_ISL_468727 to 468746, EPI_ISL_475060 to 475082, EPI_ISL_481221 to 481233			
see above	Lab voor klinische biologie	Onderzoeksgroep Virologie	Basiel Cole; Bruno Verhasselt; Hans Nauwynck; Jozefien De Clercq; Laurens Lambrechts; Linos Vandekerckhove; Marthe Pauwels; Nick Vereecke; Sebastiaan Theuns
EPI_ISL_1423274 to 1423276	LabCorp Dublin	WVU and Marshall University Combined Genomics Core Facilities	"James Denvir; Peter Perrotta; Peter Stoilov; Ryan Percifield"; Wesley Kimble
EPI_ISL_1432934, EPI_ISL_1432949, EPI_ISL_1432974, EPI_ISL_1432976, EPI_ISL_1432985, EPI_ISL_1433006, EPI_ISL_1433029, EPI_ISL_1433054, EPI_ISL_1433058, EPI_ISL_1433060, EPI_ISL_1433072, EPI_ISL_1433083 to 1433085, EPI_ISL_1433095, EPI_ISL_1433098, EPI_ISL_1433100, EPI_ISL_1433103, EPI_ISL_1433107, EPI_ISL_1433111, EPI_ISL_1433120, EPI_ISL_1433124, EPI_ISL_1433135, EPI_ISL_1433147, EPI_ISL_1433153, EPI_ISL_1433159, EPI_ISL_1433166, EPI_ISL_1433170, EPI_ISL_1433176, EPI_ISL_1433195, EPI_ISL_1433207, EPI_ISL_1433214 to 1433216, EPI_ISL_1433220, EPI_ISL_1433229, EPI_ISL_1433231, EPI_ISL_1433235, EPI_ISL_1433238 to 1433239, EPI_ISL_1433252, EPI_ISL_1433257 to 1433259, EPI_ISL_1433279, EPI_ISL_1433281, EPI_ISL_1433284, EPI_ISL_1433288, EPI_ISL_1433302, EPI_ISL_1433319, EPI_ISL_1433336, EPI_ISL_1433346, EPI_ISL_1433356, EPI_ISL_1433366, EPI_ISL_1436010, EPI_ISL_1436021, EPI_ISL_1436031, EPI_ISL_1436040, EPI_ISL_1436051, EPI_ISL_1436075 to 1436079, EPI_ISL_1436082 to 1436083, EPI_ISL_1436087, EPI_ISL_1436095, EPI_ISL_1436105, EPI_ISL_1436111, EPI_ISL_1436120, EPI_ISL_1436124, EPI_ISL_1436131 to 1436133, EPI_ISL_1436141, EPI_ISL_1436154, EPI_ISL_1436157, EPI_ISL_1436170 to 1436172, EPI_ISL_1436177, EPI_ISL_1436187, EPI_ISL_1436191, EPI_ISL_1436194, EPI_ISL_1436196, EPI_ISL_1436205 to 1436207, EPI_ISL_1436209 to 1436210, EPI_ISL_1436218 to 1436222, EPI_ISL_1436227, EPI_ISL_1436235 to 1436236, EPI_ISL_1436238 to 1436239, EPI_ISL_1436248, EPI_ISL_1436250, EPI_ISL_1436253, EPI_ISL_1436260, EPI_ISL_1436265, EPI_ISL_1436274, EPI_ISL_1436277 to 1436278, EPI_ISL_1436290, EPI_ISL_1436295, EPI_ISL_1436306, EPI_ISL_1436310, EPI_ISL_1436312, EPI_ISL_1436318, EPI_ISL_1436327 to 1436328, EPI_ISL_1436334, EPI_ISL_1436341, EPI_ISL_1436348, EPI_ISL_1436356, EPI_ISL_1436363, EPI_ISL_1436365 to 1436366, EPI_ISL_1436369, EPI_ISL_1436374 to 1436375, EPI_ISL_1436379, EPI_ISL_1436385, EPI_ISL_1436393, EPI_ISL_1438791, EPI_ISL_1439038, EPI_ISL_1439119, EPI_ISL_1439142, EPI_ISL_1439283, EPI_ISL_1439298			
see above	LabKom - Labor Augsburg MVZ GmbH	Robert Koch Institute	
EPI_ISL_1436064, EPI_ISL_1436072, EPI_ISL_1436136, EPI_ISL_1436163, EPI_ISL_1436176, EPI_ISL_1436245, EPI_ISL_1436361			
see above	LabKom - Labor Hamburg-Lübeck	Robert Koch Institute	
EPI_ISL_1436003, EPI_ISL_1436049, EPI_ISL_1436066, EPI_ISL_1436068, EPI_ISL_1436080 to 1436081, EPI_ISL_1436084 to 1436085, EPI_ISL_1436088, EPI_ISL_1436096, EPI_ISL_1436099, EPI_ISL_1436104, EPI_ISL_1436112, EPI_ISL_1436116, EPI_ISL_1436146, EPI_ISL_1436148 to 1436151, EPI_ISL_1436153, EPI_ISL_1436159, EPI_ISL_1436161, EPI_ISL_1436168, EPI_ISL_1436183, EPI_ISL_1436195, EPI_ISL_1436213, EPI_ISL_1436223 to 1436224, EPI_ISL_1436234, EPI_ISL_1436241, EPI_ISL_1436246, EPI_ISL_1436259, EPI_ISL_1436273, EPI_ISL_1436276, EPI_ISL_1436279, EPI_ISL_1436283 to 1436284, EPI_ISL_1436289, EPI_ISL_1436297, EPI_ISL_1436299, EPI_ISL_1436302, EPI_ISL_1436316 to 1436317, EPI_ISL_1436319, EPI_ISL_1436330 to 1436331, EPI_ISL_1436362			
see above	LabKom - Labor Hannover MVZ GmbH	Robert Koch Institute	
EPI_ISL_1432980, EPI_ISL_1436262, EPI_ISL_1439032, EPI_ISL_1439034, EPI_ISL_1439040, EPI_ISL_1439055, EPI_ISL_1439062 to 1439064, EPI_ISL_1439071, EPI_ISL_1439083, EPI_ISL_1439100, EPI_ISL_1439113, EPI_ISL_1439121, EPI_ISL_1439137 to 1439138, EPI_ISL_1439157, EPI_ISL_1439171, EPI_ISL_1439180, EPI_ISL_1439189, EPI_ISL_1439199 to 1439200, EPI_ISL_1439211, EPI_ISL_1439232, EPI_ISL_1439235, EPI_ISL_1439238, EPI_ISL_1439244, EPI_ISL_1439253, EPI_ISL_1439256, EPI_ISL_1439264, EPI_ISL_1439268, EPI_ISL_1439273, EPI_ISL_1439287, EPI_ISL_1439293, EPI_ISL_1439296, EPI_ISL_1439308, EPI_ISL_1439323, EPI_ISL_1439345, EPI_ISL_1439365			
see above	LabKom - Labor Mainz MVZ GmbH	Robert Koch Institute	
EPI_ISL_1436029, EPI_ISL_1436070, EPI_ISL_1436073 to 1436074, EPI_ISL_1436086, EPI_ISL_1436090, EPI_ISL_1436113, EPI_ISL_1436128, EPI_ISL_1436135, EPI_ISL_1436184, EPI_ISL_1436199 to 1436200, EPI_ISL_1436214, EPI_ISL_1436216, EPI_ISL_1436247, EPI_ISL_1436255, EPI_ISL_1436258, EPI_ISL_1436261, EPI_ISL_1436275, EPI_ISL_1436281, EPI_ISL_1436314, EPI_ISL_1436336, EPI_ISL_1436359, EPI_ISL_1436373, EPI_ISL_1436380, EPI_ISL_1436388, EPI_ISL_1438695, EPI_ISL_1438709, EPI_ISL_1438715, EPI_ISL_1438720, EPI_ISL_1438733 to 1438735, EPI_ISL_1438748, EPI_ISL_1438806,			

EPI_ISL_1438825, EPI_ISL_1438833, EPI_ISL_1438841, EPI_ISL_1438856, EPI_ISL_1438869, EPI_ISL_1438878, EPI_ISL_1438893, EPI_ISL_1438939, EPI_ISL_1438941, EPI_ISL_1438946, EPI_ISL_1438971, EPI_ISL_1439009, EPI_ISL_1439011 to 1439012, EPI_ISL_1439022, EPI_ISL_1439041, EPI_ISL_1439049, EPI_ISL_1439068, EPI_ISL_1439096 to 1439097, EPI_ISL_1439102 to 1439103, EPI_ISL_1439110, EPI_ISL_1439120, EPI_ISL_1439130, EPI_ISL_1439148, EPI_ISL_1439154 to 1439155, EPI_ISL_1439162, EPI_ISL_1439184, EPI_ISL_1439192, EPI_ISL_1439204, EPI_ISL_1439207, EPI_ISL_1439210, EPI_ISL_1439221, EPI_ISL_1439226, EPI_ISL_1439269, EPI_ISL_1439275, EPI_ISL_1439284, EPI_ISL_1439301, EPI_ISL_1439304 to 1439305, EPI_ISL_1439313, EPI_ISL_1439316 to 1439317, EPI_ISL_1439333, EPI_ISL_1439341, EPI_ISL_1439346, EPI_ISL_1439348, EPI_ISL_1439352, EPI_ISL_1439362 to 1439363, EPI_ISL_1439366

see above | LabKom - Labor an der Salzbrücke MVZ GmbH | Robert Koch Institute

EPI_ISL_1439033, EPI_ISL_1439042, EPI_ISL_1439050, EPI_ISL_1439052, EPI_ISL_1439056, EPI_ISL_1439060 to 1439061, EPI_ISL_1439066, EPI_ISL_1439072, EPI_ISL_1439074 to 1439075, EPI_ISL_1439079, EPI_ISL_1439085, EPI_ISL_1439087, EPI_ISL_1439091, EPI_ISL_1439093, EPI_ISL_1439104, EPI_ISL_1439107, EPI_ISL_1439112, EPI_ISL_1439126, EPI_ISL_1439129, EPI_ISL_1439131, EPI_ISL_1439144, EPI_ISL_1439146, EPI_ISL_1439160, EPI_ISL_1439163, EPI_ISL_1439165, EPI_ISL_1439173, EPI_ISL_1439175, EPI_ISL_1439178, EPI_ISL_1439183, EPI_ISL_1439186, EPI_ISL_1439194 to 1439195, EPI_ISL_1439203, EPI_ISL_1439216, EPI_ISL_1439222, EPI_ISL_1439225, EPI_ISL_1439229 to 1439230, EPI_ISL_1439234, EPI_ISL_1439240, EPI_ISL_1439249 to 1439250, EPI_ISL_1439254, EPI_ISL_1439265 to 1439266, EPI_ISL_1439271, EPI_ISL_1439279, EPI_ISL_1439286, EPI_ISL_1439288, EPI_ISL_1439290, EPI_ISL_1439292, EPI_ISL_1439297, EPI_ISL_1439299, EPI_ISL_1439303, EPI_ISL_1439306 to 1439307, EPI_ISL_1439310, EPI_ISL_1439312, EPI_ISL_1439324, EPI_ISL_1439328 to 1439329, EPI_ISL_1439331, EPI_ISL_1439334, EPI_ISL_1439349, EPI_ISL_1439351, EPI_ISL_1439354, EPI_ISL_1439356

see above | LabKom - MVZ Labor Bochum MLB GmbH | Robert Koch Institute

EPI_ISL_456157, EPI_ISL_456172 to 456180, EPI_ISL_456196 to 456212, EPI_ISL_456300, EPI_ISL_456379 to 456384, EPI_ISL_456388 to 456394, EPI_ISL_534335, EPI_ISL_547969, EPI_ISL_547976, EPI_ISL_547978 to 547979, EPI_ISL_547990 to 547992, EPI_ISL_548017, EPI_ISL_548021 to 548027, EPI_ISL_548040 to 548041, EPI_ISL_548049 to 548051, EPI_ISL_548068 to 548073, EPI_ISL_548077 to 548080, EPI_ISL_548083 to 548084, EPI_ISL_548106, EPI_ISL_548108 to 548111, EPI_ISL_548119 to 548120, EPI_ISL_548122, EPI_ISL_579059, EPI_ISL_579097 to 579098, EPI_ISL_579105 to 579117, EPI_ISL_579119 to 579120, EPI_ISL_579122 to 579123, EPI_ISL_579156, EPI_ISL_579403, EPI_ISL_579405, EPI_ISL_579425, EPI_ISL_582017, EPI_ISL_1469088, EPI_ISL_1469090 to 1469091, EPI_ISL_1469093 to 1469095, EPI_ISL_1469097 to 1469104, EPI_ISL_1469106 to 1469107

see above | LabPLUS | Institute of Environmental Science and Research (ESR) | Anja Werno; Antje van der Linden; Arlo Upton; Chris Mansell; David Hammer; Dragana Drinkovic; Erasmus Smit; Gary McAuliffe; Hana Sofia Andersson; Hermes Perez; James Ussher; Jill Sherwood; Jing Wang; Joep de Ligt; Josh Freeman; Julia Howard; Juliet Elvy; Lauren Jelly; Mary DeAlmeida; Matt Blakiston; Matt Storey; Matthew Rogers; Max Bloomfield; Michael Addidle; Michelle Balm; Muhammad Faisal; Nikki Freed; Olin Silander; Olivia Stroeven; Rachel Boyle; Sally Roberts; SallyAnn Harbison; Sarah Jefferies; Sharmini Muttaiyah; Susan Morpeth; Susan Taylor; Timothy Blackmore; Vani Sathyendran; Veronica Playle; Virginia Hope; Xiaoyun Ren

EPI_ISL_547970 to 547975, EPI_ISL_547977, EPI_ISL_547986 to 547989, EPI_ISL_547993 to 547995, EPI_ISL_548013 to 548016, EPI_ISL_548046, EPI_ISL_548104 to 548105, EPI_ISL_579058, EPI_ISL_579099, EPI_ISL_579421 to 579424

see above | LabTests | Institute of Environmental Science and Research (ESR) | Anja Werno; Antje van der Linden; Arlo Upton; Chris Mansell; David Hammer; Dragana Drinkovic; Erasmus Smit; Gary McAuliffe; Hana Sofia Andersson; Hermes Perez; James Ussher; Jill Sherwood; Jing Wang; Joep de Ligt; Josh Freeman; Julia Howard; Juliet Elvy; Lauren Jelly; Mary DeAlmeida; Matt Blakiston; Matt Storey; Matthew Rogers; Max Bloomfield; Michael Addidle; Michelle Balm; Muhammad Faisal; Nikki Freed; Olin Silander; Sally Roberts; Sarah Jefferies; Sharmini Muttaiyah; Susan Morpeth; Susan Taylor; Timothy Blackmore; Vani Sathyendran; Veronica Playle; Virginia Hope; Xiaoyun Ren

EPI_ISL_1469288 to 1469289, EPI_ISL_1534708 | Labkesda Karawang | National Institute of Health Research and Development | Arie Ardiansyah Nugraha; Hana Apsari Pawestri; Hartanti Dian Ikawati; Kartika Dewi Puspa; Krisna Pangesti; Nelly Puspandari; Subangkit; Vivi Setiawaty

EPI_ISL_560637 to 560642, EPI_ISL_1389165 to 1389181, EPI_ISL_1389196 to 1389253, EPI_ISL_1389257 to 1389266, EPI_ISL_1389268 to 1389274, EPI_ISL_1389276 to 1389286, EPI_ISL_1389289 to 1389306, EPI_ISL_1389312 to 1389318, EPI_ISL_1389320 to 1389343, EPI_ISL_1389345 to 1389352, EPI_ISL_1416812 to 1416822, EPI_ISL_1416833 to 1416849, EPI_ISL_1416908, EPI_ISL_1416952 to 1416965, EPI_ISL_1417015 to 1417023, EPI_ISL_1417026 to 1417051, EPI_ISL_1417071 to 1417094, EPI_ISL_1417112 to 1417215, EPI_ISL_1443880, EPI_ISL_1443890, EPI_ISL_1443896, EPI_ISL_1443903 to 1443904, EPI_ISL_1443908 to 1443910, EPI_ISL_1463448 to 1463540, EPI_ISL_1465483 to 1465534, EPI_ISL_1465537, EPI_ISL_1465541 to 1465609, EPI_ISL_1489730 to 1489736, EPI_ISL_1489739, EPI_ISL_1489835 to 1489842, EPI_ISL_1516918 to 1516919, EPI_ISL_1516942 to 1516957, EPI_ISL_1516981, EPI_ISL_1516983 to 1516984, EPI_ISL_1516986 to 1516988, EPI_ISL_1516990 to 1516991, EPI_ISL_1516993 to 1516994, EPI_ISL_1516996 to 1516997, EPI_ISL_1516999 to 1517001, EPI_ISL_1517071, EPI_ISL_1517073 to 1517074, EPI_ISL_1517076, EPI_ISL_1517102 to 1517103, EPI_ISL_1517105 to 1517106, EPI_ISL_1517108 to 1517110, EPI_ISL_1517112, EPI_ISL_1517114 to 1517115, EPI_ISL_1517117 to 1517118, EPI_ISL_1517120, EPI_ISL_1517194 to 1517197, EPI_ISL_1517199 to 1517293, EPI_ISL_1517435 to 1517463, EPI_ISL_1517468 to 1517471, EPI_ISL_1517489, EPI_ISL_1517494, EPI_ISL_1526789 to 1526792, EPI_ISL_1528193, EPI_ISL_1528202, EPI_ISL_1528211 to 1528213, EPI_ISL_1528215 to 1528217, EPI_ISL_1528221, EPI_ISL_1528223, EPI_ISL_1528226 to 1528228, EPI_ISL_1528231, EPI_ISL_1528237 to 1528238, EPI_ISL_1540446, EPI_ISL_1554904

see above | Labo Analyses Med | National Reference Center for Viruses of Respiratory Infections, Institut Pasteur, Paris | Adrien Pain; Alain Delepine; Amaury Vaysse; Angela Brisebarre; Anne Cady; BéAtrice Lesimple; Camille Capel; Catherine Coignard; Catherine Delamare; Christophe Malabat; Claire Felloni; Corinne Maufrais; CéLine Bressollette; CéLine Ramanantsoa; Damien Mornico; Dominique Descamps; Emmanuelle Permal; Etienne Simon-Lorière; Fabiana Gambaro; Fabienne Huet; Felloni Claire; Frédéric Lemoine; Gael Millot; Gentilhomme Hervé; Geoffroy Lorin De La Grandmaison; Gestin Brieuc; GréGoire Potiron; Hue; Ilka Engelmann; Jean-FrançOis Comes; Karine Michez; Le (V); Louise Lefrançois; M.-J. Blanc-Galiby; Marion Barbet; Maud Vanpeene; Mj Dufour; Méline Bizard; Nabil Gastli; OphéLie Said-Delattre; Pichard Ronan; Pierre Lechat; Pierre-Yves Leonard; Ronan Pichard; Sarah Schillinger; Selas Medilys; Sophie Chalmin; StéPhanie Guyomard-Rabenirina; Sylvie Behillil; Sylvie van der Werf; SéVerine Haouisee; Vicky Le; Victoire Baillet; Vincent Enouf

EPI_ISL_593924 to 593933 | Labo Analyses Med, Puteaux | National Reference Center for Viruses of Respiratory Infections, Institut Pasteur, Paris | Etienne Simon-Lorière; Fabiana Gambaro; Maud Vanpeene; Sylvie Behillil; Sylvie van der Werf; Vincent Enouf

EPI_ISL_593904 to 593909 | Labo Analyses Med, Sarcelles | National Reference Center for Viruses of Respiratory Infections, Institut Pasteur, Paris | Etienne Simon-Lorière; Fabiana Gambaro; Maud Vanpeene; Sylvie Behillil; Sylvie van der Werf; Vincent Enouf

EPI_ISL_420063 | Labo BM - Site de Juvisy - Hopital Général | National Reference Center for Viruses of Respiratory Infections, Institut Pasteur, Paris | Angela Brisebarre; Etienne Simon-Lorière; Flora Donati; Marion Barbet; Maud Vanpeene; Mélanie Albert; Méline Bizard; Sylvie Behillil; Sylvie van der Werf; Vincent Enouf

EPI_ISL_1443748 to 1443755 | Labo Klinische Biologie, UZA | Labo Klinische Biologie, UZA | Basil Britto Xavier; Christine Lammens; Herman Goossens; Jasmine Coppens; Marie Le Mercier; Veerle Matheeussen

EPI_ISL_1492705, EPI_ISL_1492707 | Labo Medicom | GIGA Medical Genomics | Bouchra Boujemla; Cécile Meex; Keith Durkin; Maria Artesi; Marie-Pierre Hayette; Nathalie Renotte; Pierrette Melin; Raphaël Boreux; Sébastien Bontems; Vincent Bours

EPI_ISL_1416859 to 1416872, EPI_ISL_1416874 to 1416906, EPI_ISL_1443873 to 1443879 | Labo analyses med | National Reference Center for Viruses of Respiratory Infections, Institut Pasteur, Paris | Angela Brisebarre; Anne Holstein; Camille Capel; Christophe Malabat; Corinne Maufrais; Etienne Simon-Lorière; FréDéRique Canis; Frédéric Lemoine; GréGoire Potiron; JéRome Guinard; Louise Lefrançois; Marion Barbet; Maud Vanpeene; Méline Bizard; Nathalie Coppe-Dron; Ronan Pichard; StéPhanie Guyomard-Rabenirina; Sylvie Behillil; Sylvie van der Werf; Thibault Guinoiseau; Vincent Enouf

EPI_ISL_416029 | Laboiratório Fleury | Instituto Adolfo Lutz, Interdiciplinary Procedures Center, Strategic Laboratory | Adriana Bugno; Adriano Abbud; Carlos Henrique Camargo; Claudia Regina Gonçalves; Claudio Tavares Sacchi; Daniela Bernardes Borges da Silva; Fabiana Cristina Pereira dos Santos; Maria do Carmo Sampaio Tavares Timenetsky; Simone Guadagnucci Morillo; Terezinha Maria de Paiva

EPI_ISL_1393675 to 1393688, EPI_ISL_1425555, EPI_ISL_1447228, EPI_ISL_1447250, EPI_ISL_1447265, EPI_ISL_1447271 to 1447272, EPI_ISL_1447279, EPI_ISL_1538514, EPI_ISL_1538540 to 1538543, EPI_ISL_1538687 to 1538691, EPI_ISL_1538860, EPI_ISL_1538879, EPI_ISL_1538889 to 1538890, EPI_ISL_1539002 to 1539003, EPI_ISL_1539169

see above | Labolatorium Biologi Molekularnej USK | 1. National Institute of Public Health - National Institute of Hygiene; 2. Eurofins Genomics Europe Sequencing GmbH | ECDC COVID-19 WGS support team; Eurofins Genomics Europe Sequencing Team; Gierczyki Rafa; Sadkowska-Todys Magorzata; Wokowicz Tomasz; Zacharczuk Katarzyna

EPI_ISL_1432926, EPI_ISL_1432930, EPI_ISL_1432965, EPI_ISL_1432987, EPI_ISL_1432989, EPI_ISL_1432993, EPI_ISL_1432998, EPI_ISL_1433013, EPI_ISL_1433021, EPI_ISL_1433033, EPI_ISL_1433068, EPI_ISL_1433075 to 1433076, EPI_ISL_1433079, EPI_ISL_1433093, EPI_ISL_1433099, EPI_ISL_1433105, EPI_ISL_1433110, EPI_ISL_1433113 to 1433114, EPI_ISL_1433122 to 1433123, EPI_ISL_1433130, EPI_ISL_1433154, EPI_ISL_1433158, EPI_ISL_1433167, EPI_ISL_1433175, EPI_ISL_1433191, EPI_ISL_1433193, EPI_ISL_1433204, EPI_ISL_1433208, EPI_ISL_1433233, EPI_ISL_1433241, EPI_ISL_1433266, EPI_ISL_1433268, EPI_ISL_1433272, EPI_ISL_1433285, EPI_ISL_1433332, EPI_ISL_1433344, EPI_ISL_1433368

see above | Labor 28 MVZ GmbH | Robert Koch Institute

EPI_ISL_1433378, EPI_ISL_1433380, EPI_ISL_1433382, EPI_ISL_1433384, EPI_ISL_1433386, EPI_ISL_1433388, EPI_ISL_1433390, EPI_ISL_1433392, EPI_ISL_1433394, EPI_ISL_1433396, EPI_ISL_1433398, EPI_ISL_1433400, EPI_ISL_1433402, EPI_ISL_1433404, EPI_ISL_1433406, EPI_ISL_1433408, EPI_ISL_1433410, EPI_ISL_1433412, EPI_ISL_1433414, EPI_ISL_1433416, EPI_ISL_1433418, EPI_ISL_1433420, EPI_ISL_1433422, EPI_ISL_1433426, EPI_ISL_1433428, EPI_ISL_1433430, EPI_ISL_1433432, EPI_ISL_1433434 to 1433435, EPI_ISL_1433437, EPI_ISL_1433439, EPI_ISL_1433441, EPI_ISL_1433444, EPI_ISL_1433446, EPI_ISL_1433448 to 1433449, EPI_ISL_1433451, EPI_ISL_1433453, EPI_ISL_1433466, EPI_ISL_1433468, EPI_ISL_1433470, EPI_ISL_1433472, EPI_ISL_1433474, EPI_ISL_1433476, EPI_ISL_1433478

see above | Labor Becker & Kollegen (Standort München) | Robert Koch Institute

EPI_ISL_1464114 to 1464129, EPI_ISL_1464131 to 1464171, EPI_ISL_1464173 to 1464261, EPI_ISL_1464417 to 1464582 | Labor Dr. Fenner und Kollegen | Heinrich Pette Institute, Leibniz Institute for Experimental Virology | Adam Grundhoff; Alexis Robitaille; Johannes Knobloch; Martin Aepfelbacher; Nicole Fischer; Thomas Günther

EPI_ISL_1439798, EPI_ISL_1439804 to 1439805, EPI_ISL_1439813, EPI_ISL_1439820, EPI_ISL_1439831 to 1439832, EPI_ISL_1439845, EPI_ISL_1439856 to 1439857, EPI_ISL_1439863, EPI_ISL_1439865, EPI_ISL_1442546, EPI_ISL_1442548, EPI_ISL_1442551, EPI_ISL_1442553 to 1442557, EPI_ISL_1442559 to 1442562, EPI_ISL_1442566 to 1442571

see above | Labor Dr. Heidrich & Kollegen MVZ GmbH Hamburg | Robert Koch Institute

EPI_ISL_1432936, EPI_ISL_1432961, EPI_ISL_1432967, EPI_ISL_1433025, EPI_ISL_1433081, EPI_ISL_1433125, EPI_ISL_1433127, EPI_ISL_1433142, EPI_ISL_1433162, EPI_ISL_1433200, EPI_ISL_1433228, EPI_ISL_1433265, EPI_ISL_1433292, EPI_ISL_1433364, EPI_ISL_1438691, EPI_ISL_1438700, EPI_ISL_1438716, EPI_ISL_1438722, EPI_ISL_1438759, EPI_ISL_1438762, EPI_ISL_1438854, EPI_ISL_1438859, EPI_ISL_1438867, EPI_ISL_1438905, EPI_ISL_1438917, EPI_ISL_1438925, EPI_ISL_1438931, EPI_ISL_1438938, EPI_ISL_1438947, EPI_ISL_1438954 to 1438955, EPI_ISL_1438989, EPI_ISL_1439004, EPI_ISL_1439014, EPI_ISL_1439017

see above | Labor Dr. Schumacher MVZ | Robert Koch Institute

EPI_ISL_1433741, EPI_ISL_1433764, EPI_ISL_1433780, EPI_ISL_1433844, EPI_ISL_1433908, EPI_ISL_1433950, EPI_ISL_1433965, EPI_ISL_1433971, EPI_ISL_1433990, EPI_ISL_1434003, EPI_ISL_1434011, EPI_ISL_1434015, EPI_ISL_1434074, EPI_ISL_1434125, EPI_ISL_1434130, EPI_ISL_1434170, EPI_ISL_1434185,

EPI_ISL_1434214, EPI_ISL_1434295, EPI_ISL_1434309, EPI_ISL_1434325, EPI_ISL_1434351			
see above	Labor Dr. Wisplinghoff - Köln	Robert Koch Institute	
EPI_ISL_476705, EPI_ISL_487398 to 487431	Labor Kneißler GmbH & Co. KG	Heinrich Pette Institute, Leibniz Institute for Experimental Virology	Adam Grundhoff; Manja Czech-Sioli; Matthias Ottinger; Melanie M. Brinkmann; Nicole Fischer; Thomas Günther
EPI_ISL_1435456 to 1435458, EPI_ISL_1435461 to 1435466	Labor Kneißler GmbH & Co. KG	Robert Koch Institute	
EPI_ISL_1435344, EPI_ISL_1435357, EPI_ISL_1435393 to 1435395, EPI_ISL_1435407, EPI_ISL_1435413, EPI_ISL_1435420, EPI_ISL_1435423, EPI_ISL_1439462, EPI_ISL_1439510, EPI_ISL_1439513			
see above	Labor Lübeck bzw. Laborärztliche Gemeinschaftspraxis Lübeck	Robert Koch Institute	
EPI_ISL_1433599, EPI_ISL_1433603, EPI_ISL_1433610, EPI_ISL_1433619, EPI_ISL_1433621, EPI_ISL_1433627, EPI_ISL_1433629, EPI_ISL_1433631, EPI_ISL_1433633 to 1433634, EPI_ISL_1433637, EPI_ISL_1433640, EPI_ISL_1433642, EPI_ISL_1433644, EPI_ISL_1433649, EPI_ISL_1433653, EPI_ISL_1433655, EPI_ISL_1433659, EPI_ISL_1433661 to 1433662, EPI_ISL_1433668, EPI_ISL_1433670 to 1433673, EPI_ISL_1433675, EPI_ISL_1433677 to 1433678, EPI_ISL_1434439, EPI_ISL_1434446, EPI_ISL_1434465, EPI_ISL_1434467, EPI_ISL_1434489, EPI_ISL_1434494, EPI_ISL_1434496, EPI_ISL_1434510, EPI_ISL_1434514, EPI_ISL_1434518			
see above	Labor MönchengladbachMVZ Dr. Stein + Kollegen GbR	Robert Koch Institute	
EPI_ISL_1436832, EPI_ISL_1436841, EPI_ISL_1436849, EPI_ISL_1436873, EPI_ISL_1436881, EPI_ISL_1436889, EPI_ISL_1436896, EPI_ISL_1436919, EPI_ISL_1436932, EPI_ISL_1436934, EPI_ISL_1436947, EPI_ISL_1441730 to 1441731, EPI_ISL_1441762, EPI_ISL_1441764, EPI_ISL_1441779, EPI_ISL_1441781			
see above	Labor ZOTZ|KLIMAS; MVZ Düsseldorf-Centrum	Robert Koch Institute	
EPI_ISL_1438400, EPI_ISL_1438427, EPI_ISL_1438491, EPI_ISL_1438509	Labor für medizinische Mikrobiologie Bakteriologie Mykologie Virologie & Infektionsserologie	Robert Koch Institute	
EPI_ISL_445220	Laborarory for Respiratory Viruses, "Cantacuzino" National Military-Medical Institute for Resararch and Development	Cantacuzino Institute	A.Cretu; L.Ustea; M.Lazar
EPI_ISL_1434452, EPI_ISL_1438616, EPI_ISL_1438631, EPI_ISL_1438676, EPI_ISL_1439523, EPI_ISL_1439525	Laborarztpraxis Jochen Hüter	Robert Koch Institute	
EPI_ISL_1502112, EPI_ISL_1502567, EPI_ISL_1508821, EPI_ISL_1508849, EPI_ISL_1508890, EPI_ISL_1508894, EPI_ISL_1508896 to 1508897, EPI_ISL_1508916, EPI_ISL_1508941, EPI_ISL_1508944 to 1508945, EPI_ISL_1508947, EPI_ISL_1508949, EPI_ISL_1508956 to 1508957, EPI_ISL_1508959, EPI_ISL_1508961 to 1508962, EPI_ISL_1508989 to 1508991, EPI_ISL_1509096, EPI_ISL_1509098, EPI_ISL_1509100, EPI_ISL_1509102, EPI_ISL_1509273, EPI_ISL_1509638, EPI_ISL_1524650, EPI_ISL_1524700, EPI_ISL_1524703, EPI_ISL_1524708, EPI_ISL_1524713, EPI_ISL_1524717, EPI_ISL_1524720, EPI_ISL_1524723, EPI_ISL_1548504			
see above	Laboratoire Biolim/FSS/UL	Unité Mixte Internationale TransVIHMI (UMI 233 IRD - U1175 INSERM - Université de Montpellier) IRD (Institut de recherche pour le développement)	Abla A. KONOU; Adodo SADJI; Ahidjo AYOUBA; Akoélé SILIADIN; Alassane OURO-MEDELI; Amivi EHLAN; Améyo DORKENOO; Anoumou DAGNRA; Christelle BUTEL; Déléma MABA; Eric DELAPORTE; Issaka Maman; Kokou TEGUENI; Koku AGBODEKA; Laetitia SERRANO; Martine PEETERS; Messanh DOUFFAN; Mireille PRINCE-DAVID; Mounerou SALOU; Sidonie A.M.KAGNISSODE; Sika DOSSIM; Syntiche DEVATCHAGNI Adodo SADJI; Syntyche DEVATCHAGNI; Wembo A. HALATOKO
EPI_ISL_1495980	Laboratoire Biorylis	National Reference Center for Viruses of Respiratory Infections, Institut Pasteur, Paris	Angela Brisebarre; Camille Capel; Etienne Simon-Lorière; Louise Lefrançois; Marion Barbet; Maud Vanpeene; Méline Bizard; Potiron Grégoire; Sylvie Behillil; Sylvie van der Werf; Vincent Enouf
EPI_ISL_1396891	Laboratoire LENYS	Department of Virology, Henri Mondor University Hospital, Assistance Publique Hôpitaux de Paris, Université Paris-Est Créteil, INSERM U955	Alexandre Soulier; Christophe Rodriguez; Elisabeth Trawinski; Guillaume Gricourt; Jean-Michel Pawlotsky; Melissa N'Debi; Slim Fourati; Vanessa Demontant
EPI_ISL_421734 to 421763, EPI_ISL_428939 to 428962, EPI_ISL_429706 to 429791, EPI_ISL_429793 to 429800, EPI_ISL_434487 to 434515, EPI_ISL_445053 to 445076, EPI_ISL_459893 to 459906			
see above	Laboratoire National de Sante, Microbiology, Virology	Laboratoire National de Sante, Microbiology, Epidemiology and Microbial Genomics	Anke Wienecke-Baldacchino; Ardashel Latsuzbaia; Catherine Ragimbeau; Guillaume Fournier; Jessica Tapp; Joel Mossong; Tamir Abdelrahman; Trung Nguyen Nguyen
EPI_ISL_413593	Laboratoire National de Santé	Erasmus Medical Center	Anne van der Linden; Annemiek van der Eijk; Bas Oude Munnink; Claudia Schapendonk; Corine GeurtsvanKessel; David Nieuwenhuijse; G. Fournier; Irina Chestakova; J. Mossong; Jeroen van Kampen; Jolanda Voermans; Marion Koopmans; Mark Pronk; Pascal Lexmond; Reina Sikkema; Richard Molenkamp; T. Abdelrahman; T. Nguyen; on behalf of the Dutch national COVID-19 response team.
EPI_ISL_419562 to 419607	Laboratoire National de Santé, Microbiology, Virology	Laboratoire National de Santé, Microbiology, Epidemiology and Microbial Genomics	Anke Wienecke-Baldacchino; Ardashel Latsuzbaia; Catherine Ragimbeau; Guillaume Fournier; Jessica Tapp; Joel Mossong; Tamir Abdelrahman; Trung Nguyen Nguyen
EPI_ISL_417526 to 417534	Laboratoire Nationale de Santé, Microbiology, Virology	Laboratoire Nationale de Santé, Microbiology, Epidemiology and Microbial Genomics	Anke Wienecke-Baldacchino; Ardashel Latsuzbaia; Catherine Ragimbeau; Guillaume Fournier; Jessica Tapp; Joel Mossong; Tamir Abdelrahman; Trung Nguyen Nguyen
EPI_ISL_443284 to 443288	Laboratoire de Microbiologie - Bât A - CH René Dubois	National Reference Center for Viruses of Respiratory Infections, Institut Pasteur, Paris	Angela Brisebarre; Etienne Simon-Lorière; Flora Donati; Marion Barbet; Maud Vanpeene; Mélanie Albert; Méline Bizard; Pascale Martres; Sylvie Behillil; Sylvie van der Werf; Vincent Enouf
EPI_ISL_451400	Laboratoire de Recherche et d'Analyse Médicale de la Gendarmerie Royale	Laboratoire de Recherche et d'Analyse Médicale de la Gendarmerie Royale	Amal SOUIRI; Sanaâ LEMRISS; Saâd EL KABBAJ
EPI_ISL_476024 to 476026	Laboratoire de Recherche et d'Analyses Médicales de la Gendarmerie Royale	Laboratoire de Recherche et d'Analyses Médicales de la Gendarmerie Royale	Amal SOUIRI; Amal Souiri; Ayoub Jibjibe; Mahmoud Yartaoui; Marouane El Rhouila; Mohamed Chahmi; Mohamed Labioui; Nabil Lemzaoui; Nabil Ouaariba; Nadia Kandoussi; Omar Mestoui; Samiha Sellak; Sanaâ LEMRISS; Sanaâ Lemriss; Saâd EL KABBAJ; Saâd El Kabbaj.
EPI_ISL_414600, EPI_ISL_414623	Laboratoire de Virologie Institut de Virologie - INSERM U 1109 Hôpitaux Universitaires de Strasbourg	National Reference Center for Viruses of Respiratory Infections, Institut Pasteur, Paris	Angela Brisebarre; Flora Donati Vincent Enouf; Marion Barbet; Maud Vanpeene; Méline Bizard; Mélnie Albert; Samira Fafi-Kremer; Sylvie Behillil; Sylvie van der Werf
EPI_ISL_413996 to 413997, EPI_ISL_413999, EPI_ISL_414019 to 414023	Laboratoire de Virologie, HUG	Swiss National Reference Centre for Influenza	LAUBSCHER Florian et al.
EPI_ISL_1392906	Laboratoire de la rage et de la faune sauvage, ANSES, Malzéville.	GRAM2.0, Université de Caen Normandie / Laboratoire de Virologie, CHU de Caen / National Reference Center for Respiratory Viruses France Nord, Institut Pasteur, Paris. / Laboratoire de la rage et de la faune sauvage ANSES, Malzéville.	Angela BRISEBARRE; Astrid VABRET; Camille Capel; Elodie MONCHATRE-LEROY.; Estelle LEPERCHOIS; Etienne SIMON-LORIERE; Evelyne PICARD-MEYER; Fabiana GAMBARO; Flora DONATI; Franck BOUE; Gilles SALVAT; Januel VOUTEAU; Justine HAMEL; Marine WASNIEWSKY; Maud Vanpeene; Meriadeg AR GOUILH; Mélanie ALBERT; Méline Bizard; Nicolas NESI; Sandrine CORBET; Sophie LEVALTIER; Sylvie BEHILLIL; Sylvie VAN DER WERF; Vincent ENOUF
EPI_ISL_444494 to 444516, EPI_ISL_450638 to 450653, EPI_ISL_463903 to 463969, EPI_ISL_476498 to 476513, EPI_ISL_515197 to 515246	Laboratoire de microbiologie, Hopital de Verdun	Smith Laboratory, Centre de Recherche CHU Sainte-Justine	Ivan Pavlov; Marieke Rozendaal; Martin Smith
EPI_ISL_1390770 to 1391008, EPI_ISL_1400572 to 1400794, EPI_ISL_1403093 to 1403337, EPI_ISL_1404716 to 1404877, EPI_ISL_1522382 to 1522573, EPI_ISL_1523366 to 1523524, EPI_ISL_1532652 to 1532693, EPI_ISL_1532701 to 1532730, EPI_ISL_1534160 to 1534181			
see above	Laboratoire de santé publique du Québec	Laboratoire de santé publique du Québec	Guillaume Bourque; Ioannis Ragoussis; Jesse Shapiro; Mark Lathrop and Michel Roger on behalf of the CoVSeQ research group; Mark Lathrop and Michel Roger on behalf of the CoVSeQ research group (http://covseq.ca/researchgroup); Sandrine Moreira
EPI_ISL_476822 to 476831, EPI_ISL_476833 to 476834	Laboratoire des Fièvres Hémorragiques Virales du Benin	Charité-Universitätsmedizin Berlin	Anges; Drexler; Jan Felix; Moreira-Soto Andres; Sander Anna-Lena; Yadouleton
EPI_ISL_1383642 to 1383811, EPI_ISL_1384150 to 1384159, EPI_ISL_1522576, EPI_ISL_1522580, EPI_ISL_1522584 to 1522585, EPI_ISL_1522588, EPI_ISL_1522590, EPI_ISL_1522595, EPI_ISL_1522604, EPI_ISL_1522608, EPI_ISL_1522610, EPI_ISL_1522612, EPI_ISL_1522618 to 1522619, EPI_ISL_1522641, EPI_ISL_1522643 to 1522644, EPI_ISL_1522650, EPI_ISL_1522652, EPI_ISL_1522658, EPI_ISL_1522665 to 1522666, EPI_ISL_1522668 to 1522671, EPI_ISL_1522675, EPI_ISL_1522679, EPI_ISL_1522681, EPI_ISL_1522683, EPI_ISL_1522686, EPI_ISL_1522692 to 1522693, EPI_ISL_1522695 to 1522698, EPI_ISL_1522707, EPI_ISL_1522710, EPI_ISL_1522713, EPI_ISL_1522719, EPI_ISL_1522729, EPI_ISL_1522731 to 1522732, EPI_ISL_1522735 to 1522736, EPI_ISL_1522746, EPI_ISL_1522751 to 1522752, EPI_ISL_1522757, EPI_ISL_1522759, EPI_ISL_1522762, EPI_ISL_1522766, EPI_ISL_1522768, EPI_ISL_1522774, EPI_ISL_1522780, EPI_ISL_1522782, EPI_ISL_1522795 to 1522800, EPI_ISL_1522970 to 1523026, EPI_ISL_1523092 to 1523111, EPI_ISL_1523114, EPI_ISL_1523118, EPI_ISL_1523120 to 1523121, EPI_ISL_1523129, EPI_ISL_1523138, EPI_ISL_1523140 to 1523141, EPI_ISL_1523165, EPI_ISL_1523168, EPI_ISL_1523184, EPI_ISL_1523191 to 1523192, EPI_ISL_1523210 to 1523211, EPI_ISL_1523214, EPI_ISL_1523223, EPI_ISL_1523309 to 1523344, EPI_ISL_1523363 to 1523365			

see above	Laboratoire national de sante, Microbiology, Virology	Laboratoire national de sante, Microbiology, Microbial Genomics Platform	Anke Wienecke-Baldacchino; Catherine Ragimbeau; Fatu Djabi; Jessica Tapp; Lise Pignon; Raoul Salmon; Tamir Abdelrahman; Trung Nguyen Nguyen
EPI_ISL_1384555 to 1384636, EPI_ISL_1522592, EPI_ISL_1522621, EPI_ISL_1522640, EPI_ISL_1522794, EPI_ISL_1522968 to 1522969, EPI_ISL_1523145, EPI_ISL_1523155, EPI_ISL_1523171, EPI_ISL_1523308			
see above	Laboratoires Reunis	Laboratoire national de sante, Microbiology, Microbial Genomics Platform	Anke Wienecke-Baldacchino; Bernard Weber; Catherine Ragimbeau; Fatu Djabi; Jessica Tapp; Lise Pignon; Raoul Salmon; Tamir Abdelrahman
EPI_ISL_1384160 to 1384554, EPI_ISL_1522578, EPI_ISL_1522586, EPI_ISL_1522591, EPI_ISL_1522593, EPI_ISL_1522596, EPI_ISL_1522600 to 1522601, EPI_ISL_1522603, EPI_ISL_1522609, EPI_ISL_1522611, EPI_ISL_1522614, EPI_ISL_1522616 to 1522617, EPI_ISL_1522624, EPI_ISL_1522626, EPI_ISL_1522629, EPI_ISL_1522632, EPI_ISL_1522639, EPI_ISL_1522645 to 1522646, EPI_ISL_1522649, EPI_ISL_1522656, EPI_ISL_1522659, EPI_ISL_1522663, EPI_ISL_1522673, EPI_ISL_1522680, EPI_ISL_1522684 to 1522685, EPI_ISL_1522691, EPI_ISL_1522702, EPI_ISL_1522704, EPI_ISL_1522709, EPI_ISL_1522716, EPI_ISL_1522718, EPI_ISL_1522720 to 1522722, EPI_ISL_1522727, EPI_ISL_1522734, EPI_ISL_1522738, EPI_ISL_1522745, EPI_ISL_1522749, EPI_ISL_1522755 to 1522756, EPI_ISL_1522761, EPI_ISL_1522763 to 1522765, EPI_ISL_1522767, EPI_ISL_1522771, EPI_ISL_1522776, EPI_ISL_1522778, EPI_ISL_1522791 to 1522793, EPI_ISL_1522916 to 1522967, EPI_ISL_1523071 to 1523091, EPI_ISL_1523113, EPI_ISL_1523116 to 1523117, EPI_ISL_1523119, EPI_ISL_1523123 to 1523125, EPI_ISL_1523128, EPI_ISL_1523130, EPI_ISL_1523132, EPI_ISL_1523137, EPI_ISL_1523143, EPI_ISL_1523146, EPI_ISL_1523148, EPI_ISL_1523169, EPI_ISL_1523173, EPI_ISL_1523175, EPI_ISL_1523178, EPI_ISL_1523180 to 1523183, EPI_ISL_1523199, EPI_ISL_1523201, EPI_ISL_1523203, EPI_ISL_1523208, EPI_ISL_1523221, EPI_ISL_1523290 to 1523307, EPI_ISL_1523358 to 1523362			
see above	Laboratoires d'analyses medicales - Ketterthill	Laboratoire national de sante, Microbiology, Microbial Genomics Platform	Anke Wienecke-Baldacchino; Caroline Scheiber; Catherine Ragimbeau; Fatu Djabi; Jessica Tapp; Lise Pignon; Raoul Salmon; Serge Vedy; Tamir Abdelrahman
EPI_ISL_1423696 to 1423736	Laboratori de Referència de Catalunya	SeqCOVID-SPAIN consortium/IBV(CSIC)	Aida Esperanza Ramírez Marinero; Alba Vilas Basil; Eduardo Padilla León; Mireia Canal Aranda and SeqCOVID-SPAIN consortium
EPI_ISL_1393630 to 1393639, EPI_ISL_1425554, EPI_ISL_1538433, EPI_ISL_1538496, EPI_ISL_1538547 to 1538549, EPI_ISL_1538755 to 1538756, EPI_ISL_1538892 to 1538896, EPI_ISL_1539029, EPI_ISL_1539114 to 1539115, EPI_ISL_1539161, EPI_ISL_1539179			
see above	Laboratoria Medyczne Bruss grupa ALAB sp. z o.o. Medyczne Laboratorium Diagnostyczne	1. National Institute of Public Health - National Institute of Hygiene; 2. Eurofins Genomics Europe Sequencing GmbH	ECDC COVID-19 WGS support team; Eurofins Genomics Europe Sequencing Team; Gierczyki Rafa; Sadkowska-Todys Magorzata; Wokowicz Tomasz; Zacharczuk Katarzyna
EPI_ISL_429128 to 429135, EPI_ISL_430856 to 430859, EPI_ISL_434641 to 434642, EPI_ISL_450836 to 450837	Laboratoriemedicin	The Public Health Agency of Sweden	Anna Risberg; Anna-Malin Linde; Karin Tegmark-Wisell; Maria Lind Karlberg; Mia Brytting; Olov Svartstrom; Oskar Karlsson Lindsjo; Shaman Muradrasoli; Theresa Enkirch
EPI_ISL_534248, EPI_ISL_534252 to 534255, EPI_ISL_534257 to 534258	Laboratoriemedicin Vasternorrland	The Public Health Agency of Sweden	Anna Risberg; Anna-Malin Linde; Karin Tegmark-Wisell; Maria Lind Karlberg; Mattias Haukland; Mia Brytting; Olov Svartstrom; Oskar Karlsson Lindsjo; Petra Edquist; Reza Advani; Sandra Broddesson
EPI_ISL_1394738, EPI_ISL_1394756 to 1394757, EPI_ISL_1499645 to 1499651	Laboratorio Analisi Osp. Città di Castello - Azienda USL Umbria1	Istituto Zooprofilattico Sperimentale dell'Abruzzo e Molise "G. Caporale"	Ancora M; Calistri P; Cammà C; Caporale M; Curini V; Delli Compagni E; Di Domenico M; Di Lollo Valeria; Di Pasquale A; Lorusso A; Malagigi V; Mangone I; Marcacci M; Puglia I; Rinaldi A; Savini G; Scialabba S; Tacconi P
EPI_ISL_430793 to 430794	Laboratorio Análisis Clínicos, Unidad de Servicios Diagnósticos, Swiss Medical Group	Área de Secuenciación del Laboratorio de Virología del Hospital de Niños Dr. Ricardo Gutierrez on behalf of 'Proyecto Argentino Interinstitucional de genomica de SARS-CoV-2' (PAIS Consortium)	AS; D; Goya; Guevara; LE; Lusso; MI; MS; Mistchenko; Nabaes Jodar; Natale; O; S; SM; Sanchez; Valinotto; Vicario; Viegas, M.
EPI_ISL_1406296 to 1406309, EPI_ISL_1461253 to 1461277, EPI_ISL_1494675 to 1494713	Laboratorio Aziendale di Microbiologia e Virologia, Azienda Sanitaria dell'Alto Adige	Laboratorio Aziendale di Microbiologia e Virologia, Azienda Sanitaria dell'Alto Adige	Anne Picard; Bartolomeo Mobilio Rodriguez; Chiara Cantaloni; Claudia Volpato; Elisa Masi; Elisabetta Giacobazzi; Elisabetta Pagani; Irene Bianconi; Stefanie Wieser
EPI_ISL_458085	Laboratorio Biologia Molecolare Sars Cov2 - UOC Laboratorio Analisi - Servizio Medicina di Laboratorio , Ospedale "San Francesco" - ATS- ASSL Nuoro	Laboratorio specialistico UOC Ematologia - Ospedale "San Francesco" - ATS-ASSL Nuoro	Aspproni Rosanna; Casu Gavino; Fancello Tatiana; Fiamma Maura; Floris Anna Rita; Lo Maglio Iana; Mameli Giuseppe.; Monne Maria Itria; Palmas Angelo Domenico; Piras Giovanna; Sanna Filomena; Sulis Vincenzo; Toja Alessandro
EPI_ISL_458084	Laboratorio Biologia Molecolare Sars Cov2 - UOC Laboratorio Analisi - Servizio Medicina di Laboratorio, Ospedale "San Francesco" - ATS-ASSL Nuoro	Laboratorio specialistico UOC Ematologia - Ospedale "San Francesco" - ATS-ASSL Nuoro	Aspproni Rosanna; Casu Gavino; Fancello Tatiana; Fiamma Maura; Floris Anna Rita; Lo Maglio Iana; Mameli Giuseppe.; Monne Maria Itria; Palmas Angelo Domenico; Piras Giovanna; Sanna Filomena; Sulis Vincenzo; Toja Alessandro
EPI_ISL_613706, EPI_ISL_613710, EPI_ISL_613953, EPI_ISL_613955, EPI_ISL_614396 to 614398, EPI_ISL_614889, EPI_ISL_1557219 to 1557221			
see above	Laboratorio Biologia Molecolare Sars Cov2 - UOC Laboratorio Analisi - Servizio Medicina di Laboratorio, Ospedale "San Francesco" - ATS-ASSL Nuoro	Laboratorio specialistico UOC Ematologia - Ospedale "San Francesco" - ATS-ASSL Nuoro	; Aspproni Rosanna; Casu Gavino; Fancello Tatiana; Fiamma Maura; Floris Anna Rita; Lo Maglio Iana; Malune Paolo; Mameli Giuseppe; Mameli Giuseppe.; Monne Maria Itria; Palmas Angelo Domenico; Piras Giovanna; Sanna Filomena; Sulis Vincenzo; Toja Alessandro
EPI_ISL_613560	Laboratorio Biologia Molecolare Sars Cov2 - UOC Laboratorio Analisi - Servizio Medicina di Laboratorio, Ospedale "San Francesco" - ATS-ASSL Nuoro Via Manniron i 1, 08100 Nuoro	Laboratorio specialistico UOC Ematologia - Ospedale "San Francesco" - ATS-ASSL Nuoro Nuoro	Aspproni Rosanna; Casu Gavino; Fancello Tatiana; Fiamma Maura; Floris Anna Rita; Lo Maglio Iana; Mameli Giuseppe; Monne Maria Itria; Palmas Angelo Domenico; Piras Giovanna; Sanna Filomena; Sulis Vincenzo; Toja Alessandro
EPI_ISL_547965	Laboratorio Biologia Molecolare SarsCov2 UOC LAboratorio Analisi SErvizio Medicina di Laboratorio Ospedale San Francesco ATS-ASSL Nuoro	Laboratorio Specialistico UOC Ematologia Ospedale San Francesco - ATS ASSL NUORO	Aspproni Rosanna; Casu Gavino; Fancello Tatiana; Fiamma Maura; Floris Anna Rita; Lo Maglio Iana; Mameli Giuseppe.; Monne Maria Itria; Palmas Angelo Domenico; Piras Giovanna; Sanna Filomena; Sulis Vincenzo; Toja Alessandro
EPI_ISL_1395932 to 1395935	Laboratorio Central De Redes y Programas	Grupo de Genómica y Bioinformática del Instituto de Investigación de la Cadena Láctea CONICET-INTA on behalf of 'Proyecto Argentino Interinstitucional de genomica de SARS-CoV-2' (PAIS Consortium)	AF; Amadio; Antonieta Cayré; Eberhardt; Gerardo Andino; Irazoqui; Laura Lescano; MF; Natalia Ruiz Diaz
EPI_ISL_1416332 to 1416682	Laboratorio Central de Epidemiología (LCE)	Instituto de Biotecnología de la UNAM	Alejandro Sanchez-Flores; Alfredo Herrera-Estrella; Alicia Ocaña-Mondragón; Angel Gustavo Salas-Lais; Bernardo Martínez-Miguel; Blanca Taboada; Brenda Irasema Maldonado-Meza; Carla Ivón Herrera-Najera; Carlos F. Arias; Celia Boukadida; Clara Esperanza Santacruz-Tinoco; Concepción Grajales-Muñiz; Consorcio Mexicano de Vigilancia Genómica (CoViGen-Mex). Authors (in alphabetical order): Julio Elias Alvarado-Yaah; Fernando Fontove-Herrera; Francisco Pulido; Gloria Elena Espinoza-Ayala; Gloria María Molina-Salinas; Gloria Vazquez; Hector Esteban Paz-Juárez; Hector Montoya-Fuentes; Helen Haydee Fernanda Ramírez-Plascencia; Jorge Ivan Salinal-Nevarez; José Antonio Enciso-Moreno; José Esteban Muñoz-Medina; José de Jesús Nuñez-Contreras; Juan Bautista Chale-Dzul; Luis Alberto Ochoa-Carrera; Margarita Matías-Florentino; María Guadalupe Santiago-Mauricio; María Guadalupe de Jesús Mireles-Rivera; Nelly Sélem-Mojica; Pavel Isa; Ricardo Grande; Santiago Ávila-Ríos; Víctor Hugo Borja-Aburto
EPI_ISL_1492637 to 1492681	Laboratorio Central de Salud Publica de Paraguay	Laboratorio Central de Salud Publica de Paraguay	Andrea Gómez de la Fuente; Cynthia Vázquez; Flavia Aburjaile; Juan Torales; Luiz Carlos Junior Alcantara; Marta Giovanetti; María José Ortega; María Liz Gamarra; Shirley Villalba; Talita Adelino; Vagner Fonseca
EPI_ISL_1395936 to 1395958	Laboratorio Central de Salud Pública	Grupo de Genómica y Bioinformática del Instituto de Investigación de la Cadena Láctea CONICET-INTA on behalf of 'Proyecto Argentino Interinstitucional de genomica de SARS-CoV-2' (PAIS Consortium)	AF; Amadio; Antonieta Cayré; Eberhardt; Erica Struss; Esteban Paredes; Irazoqui; Laura Lescano; MF; Natalia Andrea Ayala y María Verónica Gómez
EPI_ISL_1395959 to 1395966	Laboratorio Central de Salud Pública de la Provincia de Jujuy	Instituto de Patologia Vegetal (CIAP-INTA) on behalf of 'Proyecto Argentino Interinstitucional de genomica de SARS-CoV-2' (PAIS Consortium)	A. Miguel Alejandro Charre; Amadio; Ariel David Fridman; Claudia Mamani; Debat, HJ.; FD; Fabiana Vaca.; Fernández; Irazoqui, M.; Marquez, N.
EPI_ISL_1533990, EPI_ISL_1533992 to 1533994, EPI_ISL_1533996 to 1533997, EPI_ISL_1534002 to 1534003, EPI_ISL_1534006 to 1534010	Laboratorio Central de Saude Publica do Estado de Santa Catarina (LACEN-SC)	Laboratory of Respiratory Viruses and Measles, Oswaldo Cruz Institute, FIOCRUZ	Alice Sampaio Rocha; Ana Carolina Mendonca; Anna Carolina Paixao; Darcita Buerger Rovaris; Fernando Motta; Luciana Appolinario; Marilda Siqueira on behalf of the Fiocruz COVID-19 Genomic Surveillance Network; Paola Resende; Renata Serrano Lopes; Sandra Bianchini Fernandes
EPI_ISL_1534004	Laboratorio Central de Saude Publica do Estado de Sergipe (LACEN-SE)	Laboratory of Respiratory Viruses and Measles, Oswaldo Cruz Institute, FIOCRUZ	Alice Sampaio Rocha; Ana Carolina Mendonca; Anna Carolina Paixao; Cliomar Alves dos Santos; Fernando Motta; Luciana Appolinario; Marilda Siqueira on behalf of the Fiocruz COVID-19 Genomic Surveillance Network; Paola Resende; Renata Serrano Lopes
EPI_ISL_1465185 to 1465186, EPI_ISL_1465188 to 1465189, EPI_ISL_1465191 to 1465192, EPI_ISL_1465194 to 1465196, EPI_ISL_1465198 to 1465199, EPI_ISL_1465201 to 1465203, EPI_ISL_1465205 to 1465206, EPI_ISL_1465208 to 1465210, EPI_ISL_1465212 to 1465213, EPI_ISL_1465215 to 1465217, EPI_ISL_1465219 to 1465222, EPI_ISL_1465224 to 1465226, EPI_ISL_1465228 to 1465229, EPI_ISL_1465231 to 1465232, EPI_ISL_1465234 to 1465236, EPI_ISL_1465238 to 1465239, EPI_ISL_1465241 to 1465243, EPI_ISL_1465245 to 1465246, EPI_ISL_1465248 to 1465250, EPI_ISL_1465252 to 1465255, EPI_ISL_1465257 to 1465259,			

EPI_ISL_1465261 to 1465262, EPI_ISL_1465264 to 1465265, EPI_ISL_1465267 to 1465268, EPI_ISL_1465270 to 1465271, EPI_ISL_1465273 to 1465275, EPI_ISL_1465277 to 1465278			
see above	Laboratorio Central de Saude Publica do Estado do Maranhao (LACEN-MA)	Laboratory of Respiratory Viruses and Measles, Oswaldo Cruz Institute, FIOCRUZ	Alice Sampaio Rocha; Ana Carolina Mendonca; Anna Carolina Paixao; Fernando Motta; Lidio Gonçalves Lima Neto; Luciana Appolinario; Marilda Siqueira on behalf of the Fiocruz COVID-19 Genomic Surveillance Network; Paola Resende; Renata Serrano Lopes
EPI_ISL_1533978, EPI_ISL_1533999 to 1534001	Laboratorio Central de Saude Publica do Estado do Parana (LACEN-PR)	Laboratory of Respiratory Viruses and Measles, Oswaldo Cruz Institute, FIOCRUZ	Alice Sampaio Rocha; Ana Carolina Mendonca; Anna Carolina Paixao; Fernando Motta; Irina Nastassja Riediger; Luciana Appolinario; Maria do Carmo Debur; Marilda Siqueira on behalf of the Fiocruz COVID-19 Genomic Surveillance Network; Paola Resende; Renata Serrano Lopes
EPI_ISL_1533979, EPI_ISL_1534013	Laboratorio Central de Saude Publica do Estado do Rio Grande do Sul (LACEN-RS)	Laboratory of Respiratory Viruses and Measles, Oswaldo Cruz Institute, FIOCRUZ	Alice Sampaio Rocha; Ana Carolina Mendonca; Anna Carolina Paixao; Fernando Motta; Luciana Appolinario; Marilda Siqueira on behalf of the Fiocruz COVID-19 Genomic Surveillance Network; Paola Resende; Renata Serrano Lopes; Richard Salvato; Tatiana Schaffer Gregianini
EPI_ISL_1534012	Laboratorio Central de Saude Publica do Estado do Rio de Janeiro (LACEN-RJ)	Laboratory of Respiratory Viruses and Measles, Oswaldo Cruz Institute, FIOCRUZ	Alice Sampaio Rocha; Ana Carolina Mendonca; Andrea Cony Cavalcanti; Anna Carolina Paixao; Fernando Motta; Luciana Appolinario; Marilda Siqueira on behalf of the Fiocruz COVID-19 Genomic Surveillance Network; Paola Resende; Renata Serrano Lopes
EPI_ISL_1395840 to 1395931	Laboratorio Central de la Ciudad de Santa Fe	Grupo de Genómica y Bioinformática del Instituto de Investigación de la Cadena Láctea CONICET-INTA on behalf of 'Proyecto Argentino Interinstitucional de genomica de SARS-CoV-2' (PAIS Consortium)	AF; Amadio; C; Eberhardt; G; Irazoqui; JM; MF; Mugna; Ojeda; Pastor; Rompato; V
EPI_ISL_1395967 to 1396060	Laboratorio Central, Ministerio de Salud Cordoba	Instituto de Patologia Vegetal (CIAP-INTA) on behalf of 'Proyecto Argentino Interinstitucional de genomica de SARS-CoV-2' (PAIS Consortium)	Barbas, G.; Castro, G.; Debat, HJ.; FD; Fernández; M.B.; Marquez, N.; Pisano; Re, V.
EPI_ISL_480321	Laboratorio Clínico San José	Charité Virology-University of Costa Rica	Andres Moreira-Soto; Eugenia Corrales-Aguilar; Hugo Núñez Navas; Ignacio Postigo-Hidalgo; Jan Felix Drexler
EPI_ISL_424667	Laboratorio Estatal de Salud Publica del Estado de México	Instituto de Diagnóstico y Referencia Epidemiológicos	Adnan Araiza Rodríguez; Alejandro Sánchez; Alfredo Ponce de León Garduño; Blanca Taboada; Carlos F. Arias.; Carolina González Torres; Celia Boukadida; Cesar Raúl González Bonilla; Concepción Grajales Muñiz; Edgar Mendieta Condado; Eduardo Becerril Vargas; Fabiola Garcés Ayala; Fernando Ledesma Barrientos; Francisco Javier Gaytán Cervantes; Francisco Pulido; Gisela Barrera Badillo; Gloria Vázquez; Guillermo M. Ruiz-Palacios; Irma López Martínez; Joel Armando Vázquez Pérez; José Arturo Martínez Orozco; José Ernesto Ramírez González; José Esteban Muñoz Medina; Lucia Hernández Rivas; Luis Alberto García Andrade; Mario Mújica Sánchez; Pavel Isa; Pilar Ramos Cervantes; Ricardo Grande; Santiago Avila Ríos; Victor Hugo Borja Aburto; Violeta Ibarra Gonzalez
EPI_ISL_424672	Laboratorio Estatal de Salud Publica del Estado de Puebla	Instituto de Diagnostico y Referencia Epidemiologicos	Adnan Araiza Rodríguez; Alejandro Sánchez; Alfredo Ponce de León Garduño; Blanca Taboada; Carlos F. Arias; Carolina González Torres; Celia Boukadida; Cesar Raúl González Bonilla; Concepción Grajales Muñiz; Edgar Mendieta Condado; Eduardo Becerril Vargas; Fabiola Garcés Ayala; Fernando Ledesma Barrientos; Francisco Javier Gaytán Cervantes; Francisco Pulido; Gisela Barrera Badillo; Gloria Vázquez; Guillermo M. Ruiz-Palacios; Irma López Martínez; Joel Armando Vázquez Pérez; José Arturo Martínez Orozco; José Ernesto Ramírez González; José Esteban Muñoz Medina; Lucia Hernández Rivas; Luis Alberto García Andrade; Mario Mújica Sánchez; Pavel Isa; Pilar Ramos Cervantes; Ricardo Grande; Santiago Avila Ríos; Victor Hugo Borja Aburto; Violeta Ibarra Gonzalez
EPI_ISL_424670	Laboratorio Estatal de Salud Publica del Estado de Queretaro	Instituto de Diagnóstico y Referencia Epidemiologicos	Adnan Araiza Rodríguez; Alejandro Sánchez; Alfredo Ponce de León Garduño; Blanca Taboada; Carlos F. Arias; Carolina González Torres; Celia Boukadida; Cesar Raúl González Bonilla; Concepción Grajales Muñiz; Edgar Mendieta Condado; Eduardo Becerril Vargas; Fabiola Garcés Ayala; Fernando Ledesma Barrientos; Francisco Javier Gaytán Cervantes; Francisco Pulido; Gisela Barrera Badillo; Gloria Vázquez; Guillermo M. Ruiz-Palacios; Irma López Martínez; Joel Armando Vázquez Pérez; José Arturo Martínez Orozco; José Ernesto Ramírez González; José Esteban Muñoz Medina; Lucia Hernández Rivas; Luis Alberto García Andrade; Mario Mújica Sánchez; Pavel Isa; Pilar Ramos Cervantes; Ricardo Grande; Santiago Avila Ríos; Victor Hugo Borja Aburto; Violeta Ibarra Gonzalez
EPI_ISL_1525249	Laboratorio HUB -Azienda Ospedaliero Universitaria - AOU - Cagliari	Laboratorio SPOKE Biologia Molecolare -Azienda Ospedaliero Universitaria - AOU - Cagliari	Alessandra Scano; Ferdinando Coghe; Germano Orrù; Riccardo Cappai; Sara Fais
EPI_ISL_480328	Laboratorio LABIN	Charité Virology-University of Costa Rica	Andres Moreira-Soto; Eugenia Corrales-Aguilar; Ignacio Postigo-Hidalgo; Ignacio Soto Pacheco; Jan Felix Drexler
EPI_ISL_1385805	Laboratorio Microbiologia P.O. Cardarelli	Laboratorio Microbiologia P.O. Cardarelli	Giovanna Niro; Massimiliano Scutellà; Valentina Felice
EPI_ISL_1385803	Laboratorio Microbiologia P.O.Cardarelli	Laboratorio Microbiologia P.O. Cardarelli	Giovanna Niro; Scutellà Massimiliano; Valentina Felice
EPI_ISL_1406778	Laboratorio Microbiologia PO Cardarelli	laboratorio Microbiologia PO Cardarelli	Felice Valentina; Giovanna Niro; Massimiliano Scutellà
EPI_ISL_1533981 to 1533982, EPI_ISL_1533984 to 1533989	Laboratorio Nacional de Salud	Laboratory of Respiratory Viruses and Measles, Oswaldo Cruz Institute, FIOCRUZ	Ana Carolina Mendonca; Anna Carolina Paixao; Cesar Roberto Conde Pereira; Claudia Estrada; Fernando Motta; Luciana Appolinario; Marilda Siqueira on behalf of the Fiocruz COVID-19 Genomic Surveillance Network; Paola Resende
EPI_ISL_1396383 to 1396390	Laboratorio Salud Publica Mendoza	Nodo de Secuenciación Tierra del Fuego - Hospital Regional Ushuaia - Centro Austral De Investigaciones Científicas - Universidad Nacional De Tierra Del Fuego on behalf of 'Proyecto Argentino Interinstitucional de genomica de SARS-CoV-2' (PAIS Consortium)	Aguirre Carolina; Alberto Carena; Alejandro Ezequiel Rojas; Andrea Falaschi; Belén Ortiz; Bosio Lia; Carlos Espul; Cristian Garay; Cristina Fernanda Nardi; Fernando Gallego; Fernando Giuliani; Ivan Dario Gramundi; Luciana Martinez; Maria Belen Peralta Roca; Patricia Robledo; Santiago Guillermo Ceballos; Silvia Zerrer; Silvina Denita
EPI_ISL_468753 to 468759	Laboratorio de Biologia Molecular, Facultad de Medicina, Universidad de Atacama	Center for Mathematical Modeling and Center for Genome Regulation. Santiago, Chile	Allende ML; C Echeverria; Gaete A; González M; Maass A; Palma R; Travisany D; Urra C; Varas M
EPI_ISL_457940 to 457973, EPI_ISL_480428 to 480429, EPI_ISL_480431 to 480438	Laboratorio de Biología Molecular Asociación Española Primera en Salud	Departments of Pathology and Medicine, New York University School of Medicine	Adriana Heguy; Christian Marier; Gael Westby; Gonzalo Manrique; Maria Noel Zubillaga; Maria Victoria Elizondo; Matthew T Maurano; Paul Zappile
EPI_ISL_417034, EPI_ISL_1533609	Laboratorio de Ecologia de Doencas Transmissiveis na Amazonia, Instituto Leonidas e Maria Deane - Fiocruz Amazonia	Laboratorio de Ecologia de Doencas Transmissiveis na Amazonia, Instituto Leonidas e Maria Deane - Fiocruz Amazonia	André Corado; Debora Duarte; Felipe Naveca; Felipe Naveca on behalf of the Fiocruz COVID-19 Genomic Surveillance Network; Fernanda Nascimento; George Silva; Karina Pessoa; Luciana Gonçalves; Maria Júlia Brandão; Matilde Mejía; Michele Jesus; Sérgio Luz; Valdinete Nascimento; Victor Souza; Ágatha Costa
EPI_ISL_545955	Laboratorio de Infecciones Respiratorias Agudas. Centro Nacional de Salud Publica,Instituto Nacional de Salud	Laboratorio de Infecciones Respiratorias Agudas. Centro Nacional de Salud Publica,Instituto Nacional de Salud	Juscamayta, E.
EPI_ISL_591531 to 591534	Laboratorio de Infectologia y virologia molecular	Center for Mathematical Modeling and Center for Genome Regulation. Santiago, Chile	Allende ML; Ferres M.; Gaete A; González M; Maass A; Palma R; Travisany D; Urra C; Valiente F; Varas M
EPI_ISL_1511641, EPI_ISL_1511644	Laboratorio de Patologia Clinica - UNICAMP	Laboratorio de Estudos de Virus Emergentes	Alessandro S. Farias; Aline Vieira; André S. Vieira; Angelica Schreiber; Antonio C. G. Carlos Jr; Barbara F. N. Carvalho; Camila L. Simeoni; Daniel A. Toledo-Teixeira; Emerson S.S. França; Ester C. Sabino; Fabiana Granja; Fernando R. Spilki; Gisele A. Pedroso; Ingra M. Claro; José Luiz Proenca-Modena; Julia Forato; Kamila C. S. Krywacz; Karina Bispo-dos-Santos; Luciana S. Mofatto; Luís Felipe Bachur; Luís G. O. Cardoso; Magnun N. N. Santos; Marcelo A. Mori; Maria H. P. Pavan; Maria L. Moretti; Mariene R. Amorim; Natalia S. Brunetti; Nuno R. Faria; Patricia A. F. Leme; Pierina L. Parise; Rodrigo Angerami; Tania R. Zaccariotto; William M. Souza
EPI_ISL_1511399	Laboratorio de Patologia Clinica - UNICAMP	Laboratorio de Estudos de Virus Emergentes - UNICAMP	Alessandro S. Farias; Aline Vieira; André S. Vieira; Angelica Schreiber; Antonio C. G. Carlos Jr; Barbara F. N. Carvalho; Camila L. Simeoni; Daniel A. Toledo-Teixeira; Emerson S.S. França; Ester C. Sabino; Fabiana Granja; Fernando R. Spilki; Gisele A. Pedroso; Ingra M. Claro; José Luiz Proenca-Modena; Julia Forato; Kamila C. S. Krywacz; Karina Bispo-dos-Santos; Luciana S. Mofatto; Luís Felipe Bachur; Luís G. O. Cardoso; Magnun N. N. Santos; Marcelo A. Mori; Maria H. P. Pavan; Maria L. Moretti; Mariene R. Amorim; Natalia S. Brunetti; Nuno R. Faria; Patricia A. F. Leme; Pierina L. Parise; Rodrigo Angerami; Tania R. Zaccariotto; William M. Souza
EPI_ISL_517770, EPI_ISL_517959	Laboratorio de Referencia Nacional de Virus Respiratorio. Centro Nacional de Salud Publica. Instituto Nacional de Salud Peru	Laboratorio de Referencia Nacional de Biotecnología y Biología Molecular. Centro Nacional de Salud Publica. Instituto Nacional de Salud Peru.	Carlos Padilla Rojas; Henri Bailon Calderon; Johanna Balbuena Torres; Karolyn Vega Chozo; Marco Galarza Perez; Maribel Huaringa Nuñez; Nancy Rojas Serrano.; Omar Caceres Rey; Priscila Lope Pari
EPI_ISL_516626, EPI_ISL_516628, EPI_ISL_516646 to 516647, EPI_ISL_516653 to 516654, EPI_ISL_516685 to 516686, EPI_ISL_516721 to 516722, EPI_ISL_516987, EPI_ISL_517531, EPI_ISL_517686 to 517687, EPI_ISL_517713, EPI_ISL_517958, EPI_ISL_523810, EPI_ISL_523954, EPI_ISL_523979, EPI_ISL_523994, EPI_ISL_524471, EPI_ISL_524473, EPI_ISL_525206, EPI_ISL_527787, EPI_ISL_527789			

see above	Laboratorio de Referencia Nacional de Virus Respiratorio. Centro Nacional de Salud Publica. Instituto Nacional de Salud Peru.	Laboratorio de Referencia Nacional de Biotecnología y Biología Molecular. Centro Nacional de Salud Publica. Instituto Nacional de Salud Peru.	Carlos Padilla Rojas; Henri Bailon Calderon; Johanna Balbuena Torres; Karolyn Vega Chozo; Marco Galarza Perez; Maribel Huaringa Nuñez; Nancy Rojas Serrano.; Omar Caceres Rey; Priscila Lope Pari
EPI_ISL_1549140	Laboratorio de Referencia Nacional de Virus Respiratorio. Instituto Nacional de Salud Peru	Laboratorio de Referencia Nacional de Biotecnología y Biología Molecular. Instituto Nacional de Salud Peru	Carlos Padilla Rojas; Henri Bailon Calderon; Johanna Balbuena Torrez; Karolyn Vega Chozo; Luis Barcena; Marco Galarza Perez; Maribel Huaringa Nuñez; Nancy Rojas Serrano; Omar Caceres Rey; Priscila Lope Pari
EPI_ISL_1548074	Laboratorio de Referencia Nacional de Virus Respiratorio. Instituto Nacional de Salud Perú	Laboratorio de Referencia Nacional de Biotecnología y Biología Molecular. Instituto Nacional de Salud Perú	Carlos Padilla Rojas; Henri Bailon Calderon; Johanna Balbuena Torrez; Karolyn Vega Chozo; Luis Barcena; Marco Galarza Perez; Maribel Huaringa Nuñez; Nancy Rojas Serrano; Omar Caceres Rey; Priscila Lope Pari
EPI_ISL_489836 to 489839, EPI_ISL_489987 to 489990, EPI_ISL_490209, EPI_ISL_490315 to 490316, EPI_ISL_490975 to 490976, EPI_ISL_491427 to 491430, EPI_ISL_491433 to 491436, EPI_ISL_491458 to 491464, EPI_ISL_514226, EPI_ISL_514317, EPI_ISL_514338 to 514342, EPI_ISL_1532145 to 1532235, EPI_ISL_1534622 to 1534689			
see above	Laboratorio de Referencia Nacional de Virus Respiratorio. Instituto Nacional de Salud Perú	Laboratorio de Referencia Nacional de Biotecnología y Biología Molecular. Instituto Nacional de Salud Perú	Carlos Padilla Rojas; Henri Bailon Calderon; Johanna Balbuena Torres; Johanna Balbuena Torrez; Karolyn Vega Chozo; Karolyn Chozo Vega; Karolyn Vega Chozo; Luis Barcena; Marco Galarza Perez; Maribel Huaringa Nuñez; Nancy Rojas Serrano; Nancy Rojas Serrano.; Omar Caceres Rey; Priscila Lope Pari
EPI_ISL_491172	Laboratorio de Referencia Nacional de Virus Respiratorio. Instituto Nacional de Salud Perú	Laboratorio de Referencia Nacional de Biotecnología y Biología Molecular. Instituto Nacional de Salud Perú.	Carlos Padilla Rojas; Henri Bailon Calderon; Johanna Balbuena Torres; Karolyn Chozo Vega; Marco Galarza Perez; Maribel Huaringa Nuñez; Nancy Rojas Serrano; Omar Caceres Rey; Priscila Lope Pari
EPI_ISL_487269	Laboratorio de Referencia Nacional de Virus Respiratorio. Instituto Nacional de Salud.	Laboratorio de Referencia Nacional de Biotecnología y Biología Molecular. Instituto Nacional de Salud.	Carlos Padilla Rojas; Henri Bailon Calderon; Johanna Balbuena Torres; Karolyn Chozo Vega; Marco Galarza Perez; Maribel Huaringa Nuñez; Nancy Rojas Serrano; Omar Caceres Rey; Priscila Lope Pari
EPI_ISL_415787, EPI_ISL_514227	Laboratorio de Referencia Nacional de Virus Respiratorio. Instituto Nacional de Salud. Peru	Laboratorio de Referencia Nacional de Biotecnologia y Biologia Molecular.Instituto Nacional de Salud.Peru	Carlos Padilla Rojas; Hemri Bailon Calderon; Henri Bailon Calderon; Johanna Balbuena Torres; Johanna Balbuena Torrez; Karolyn Vega Chozo; Marco Galarza Perez; Maribel Huaringa Nuñez; Nancy Rojas Serrano; Omar Caceres Rey; Priscila Lope Pari
EPI_ISL_514264 to 514265	Laboratorio de Referencia Nacional de Virus Respiratorio. Instituto Nacional de Salud. Perú	Laboratorio de Referencia Nacional de Biotecnologia y Biologia Molecular.Instituto Nacional de Salud.Perú	Carlos Padilla Rojas; Henri Bailon Calderon; Johanna Balbuena Torres; Karolyn Vega Chozo; Marco Galarza Perez; Maribel Huaringa Nuñez; Nancy Rojas Serrano; Omar Caceres Rey; Priscila Lope Pari
EPI_ISL_491431	Laboratorio de Referencia Nacional de Virus Respiratorio. Instituto Nacional de Salud. Perú	Laboratorio de Referencia Nacional de Biotecnología y Biología Molecular. Instituto Nacional de Salud.Perú	Carlos Padilla Rojas; Henri Bailon Calderon; Johanna Balbuena Torres; Karolyn Vega Chozo; Marco Galarza Perez; Maribel Huaringa Nuñez; Nancy Rojas Serrano.; Omar Caceres Rey; Priscila Lope Pari
EPI_ISL_491432	Laboratorio de Referencia Nacional de Virus Respiratorio. Instituto Nacional de Salud.Perú	Laboratorio de Referencia Nacional de Biotecnología y Biología Molecular. Instituto Nacional de Salud.Perú	Carlos Padilla Rojas; Henri Bailon Calderon; Johanna Balbuena Torres; Karolyn Vega Chozo; Marco Galarza Perez; Maribel Huaringa Nuñez; Nancy Rojas Serrano.; Omar Caceres Rey; Priscila Lope Pari
EPI_ISL_529065 to 529079, EPI_ISL_540923 to 540992, EPI_ISL_568510 to 568555	Laboratorio de Referencia Nacional de Virus Respiratorios, Instituto Nacional de Salud Perú	Laboratorio de Genómica Microbiana, Universidad Peruana Cayetano Heredia	Alejandra Dávila-Barclay; Brenda Ayzanoa; Camila Castillo-Vilcahuaman; Guillermo Salvatierra; Janet Huancachoque; Luis González; Maribel Huaringa; Pablo Tsukayama; Pedro E. Romero; Pool Marcos
EPI_ISL_482468	Laboratorio de Referencia Nacional de Virus Respiratorios. Instituto Nacional de Salud Peru	Laboratorio de Referencia Nacional de Biotecnologia y Biologia Molecular. Instituto Nacional de Salud Peru	Carlos Padilla Rojas; Henri Bailon Calderon; Johanna Balbuena Torres; Karolyn Vega Chozo; Maribel Huaringa Nuñez; Nancy Rojas Serrano; Omar Caceres Rey; Priscila Lope Pari
EPI_ISL_1494941 to 1494943	Laboratorio de Salud Publica Arauca	Instituto Nacional de Salud- Dirección de Investigación en Salud Pública	Carlos Franco-Muñoz; Carmen Osorio; Diana Malo; Diego A. Álvarez-Díaz; Diego Andrés Prada; Gerardo Santamaría; Hector Alejandro Ruiz-Moreno; Jhonnatan Reales-González; Juan Camilo Martinez; Julian Naizaque; Katherine Laiton-Donato; Lisseth Pardo; Magdalena Wiesner; Marcela Mercado-Reyes; Maria T. Herrera-Sepúlveda; Marta Lopez Blanco; Martha Lucia Ospina Martinez; Paola Rojas; Sergio Gomez; Sheryll Corchuelo; Ángela Alarcon Cruz
EPI_ISL_1494950	Laboratorio de Salud Publica de Santander	Instituto Nacional de Salud- Dirección de Investigación en Salud Pública	Carlos Franco-Muñoz; Carmen Osorio; Diana Malo; Diego A. Álvarez-Díaz; Diego Andrés Prada; Gerardo Santamaría; Hector Alejandro Ruiz-Moreno; Jhonnatan Reales-González; Juan Camilo Martinez; Julian Naizaque; Katherine Laiton-Donato; Lisseth Pardo; Magdalena Wiesner; Marcela Mercado-Reyes; Maria T. Herrera-Sepúlveda; Marta Lopez Blanco; Martha Lucia Ospina Martinez; Paola Rojas; Sergio Gomez; Sheryll Corchuelo; Ángela Alarcon Cruz
EPI_ISL_1396061 to 1396091	Laboratorio de Salud Pública	Instituto de Patologia Vegetal (CIAP-INTA) on behalf of 'Proyecto Argentino Interinstitucional de genomica de SARS-CoV-2' (PAIS Consortium)	A. Mariana B. Salmerón; Amadio; Ana Maria Zamora; Dardo E. Costas; Debat, HJ.; FD; Fernández; Graciela Alabarse.; Gustavo Ruìz de Huidobro; Irazoqui, M.; Marquez, N.
EPI_ISL_1494959	Laboratorio de Salud Pública - Secretaría Distrital de Salud	Instituto Nacional de Salud- Dirección de Investigación en Salud Pública	Carlos Franco-Muñoz; Carmen Osorio; Diana Malo; Diego A. Álvarez-Díaz; Diego Andrés Prada; Gerardo Santamaría; Hector Alejandro Ruiz-Moreno; Jhonnatan Reales-González; Juan Camilo Martinez; Julian Naizaque; Katherine Laiton-Donato; Lisseth Pardo; Magdalena Wiesner; Marcela Mercado-Reyes; Maria T. Herrera-Sepúlveda; Marta Lopez Blanco; Martha Lucia Ospina Martinez; Paola Rojas; Sergio Gomez; Sheryll Corchuelo; Ángela Alarcon Cruz
EPI_ISL_470615 to 470650	Laboratorio de Virologia Molecular / UFRJ	Bioinformatics Laboratory / LNCC	Alexandra Gerber; Amilcar Tanuri; Ana Paula Guimarães; CADDE-group; Carolina Voloch; Ester Cerdeira Sabino; Filipe Romero; Ingra Morales Claro; Jaqueline Goes de Jesus; Laboratorio Hermes Pardini; Laboratorio Simile; Luiz Gonzaga Paula de Almeida; Mariane Talon; Nuno Rodrigues Faria; Renato Santana Aguiar e Ana Tereza Vasconcelos; Ronaldo da Silva Francisco Junior; Terezinha Marta Pereira; working group UFMG; working group UFRJ; Átila Duque Rossi
EPI_ISL_1533977, EPI_ISL_1533991, EPI_ISL_1533995, EPI_ISL_1533998	Laboratorio de Virologia Molecular / UFRJ	Laboratory of Respiratory Viruses and Measles, Oswaldo Cruz Institute, FIOCRUZ	Alice Sampaio Rocha; Amilcar Tanuri; Ana Carolina Mendonca; Anna Carolina Paixao; Carolina M Voloch; Fernando Motta; Luciana Appolinario; Marilda Siqueira on behalf of the Fiocruz COVID-19 Genomic Surveillance Network; Paola Resende; Renata Serrano Lopes
EPI_ISL_1399180, EPI_ISL_1399228 to 1399229, EPI_ISL_1399258, EPI_ISL_1406186, EPI_ISL_1406188, EPI_ISL_1406190 to 1406191, EPI_ISL_1406193, EPI_ISL_1406200, EPI_ISL_1406203, EPI_ISL_1406240, EPI_ISL_1406248, EPI_ISL_1406272 to 1406273, EPI_ISL_1406275 to 1406276, EPI_ISL_1406280, EPI_ISL_1406282, EPI_ISL_1406291, EPI_ISL_1406293, EPI_ISL_1406387 to 1406389, EPI_ISL_1406392, EPI_ISL_1406396, EPI_ISL_1406398, EPI_ISL_1406408 to 1406410, EPI_ISL_1406417 to 1406430, EPI_ISL_1406598, EPI_ISL_1406689, EPI_ISL_1472108			
see above	Laboratorio de Virología HUCA	Laboratorio de Virología HUCA	Abreu F; Alvarez-Arguelles ME; Boga JA; Castelló C; Costales I; Coto E; Gómez de Oña J; Martín-Rodríguez G; Melón S; Perez-Martínez Z; Rojo S; Sandoval M
EPI_ISL_1396246 to 1396331	Laboratorio de Virología del Hospital de Niños Dr. Ricardo Gutierrez	Biocódices SA. on behalf of 'Proyecto Argentino Interinstitucional de genomica de SARS-CoV-2' (PAIS Consortium)	A; Acevedo; Alexay; Alvarez Lopez; Barreda Frank; Berros; C; Dopazo, H.; E; G; Grandis; J; JM; Jacques; Labarta; M; ME; Medina; Mistchenko; N; O; S; Streitenberger; Thomas; Villegas; Zubrzycki J
EPI_ISL_430795, EPI_ISL_430799 to 430801, EPI_ISL_430803 to 430804, EPI_ISL_430807, EPI_ISL_430809 to 430818, EPI_ISL_1395777 to 1395781, EPI_ISL_1395786 to 1395787, EPI_ISL_1395791 to 1395797, EPI_ISL_1395805 to 1395806, EPI_ISL_1396149 to 1396245			
see above	Laboratorio de Virología del Hospital de Niños Dr. Ricardo Gutierrez	Área de Secuenciación del Laboratorio de Virología del Hospital de Niños Dr. Ricardo Gutierrez on behalf of 'Proyecto Argentino Interinstitucional de genomica de SARS-CoV-2' (PAIS Consortium)	A; AS; Acevedo; Acuña; Alexay; Alvarez Lopez; Barreda Frank; C; D; E; Echavarria; G; Goya; Grandis; Gravis; Jacques; LE; Labarta; Lusso; M; ME; MI; MS; Medina; Mistchenko; N; Nabaes Jodar; Natale; O; S; Streitenberger; Thomas; Valinotto; Viegas, M.; Villegas
EPI_ISL_1396092 to 1396123	Laboratorio de Virus Respiratorios y Neurovirosis. Hospital Señor del Milagro	Instituto de Patologia Vegetal (CIAP-INTA) on behalf of 'Proyecto Argentino Interinstitucional de genomica de SARS-CoV-2' (PAIS Consortium)	A. Dra. Raskovsky Viviana; Amadio; Debat, HJ.; Dr. Lavaque Esteban; Dra. Veronica Lesser. Tecnica: Pamela Cajal; FD; Fernanda Aguero.; Fernández; Irazoqui, M.; Marquez, N.
EPI_ISL_1396124 to 1396131, EPI_ISL_1396140 to 1396148	Laboratorio de la Dirección de Epidemiología	Área de Secuenciación del Laboratorio de Virología del Hospital de Niños Dr. Ricardo Gutierrez on behalf of 'Proyecto Argentino Interinstitucional de genomica de SARS-CoV-2' (PAIS Consortium)	AG; Acuña; Alexay; Bertone; C; D; Goya; JC; LE; Lusso; M; MI; Nabaes Jodar; Natale; Rechimont; S; Usero; Valinotto; Viegas, M.; Villasana
EPI_ISL_1395790	Laboratorio de salud pública, Facultad de Ciencias Exactas, UNLP	Área de Secuenciación del Laboratorio de Virología del Hospital de Niños Dr. Ricardo Gutierrez on behalf of 'Proyecto Argentino Interinstitucional de genomica de SARS-CoV-2' (PAIS Consortium)	Acuña; Andrés Angelletti; Andrés Cordero; Carina Tersigni; D; Goya; LE; Laura Delaplace; Lusso; M; MI; Nabaes Jodar; Natale; Rosana Isabel Toro; S; Valinotto; Victoria Nadalich; Viegas, M.; Víctoria Cabassi
EPI_ISL_1395789, EPI_ISL_1395798 to 1395801	Laboratorio del Hospital Interzonal General de Agudos "Evita"	Área de Secuenciación del Laboratorio de Virología del Hospital de Niños Dr. Ricardo Gutierrez on behalf of 'Proyecto	Acuña; Alejandra Musto; Alexay; D; Erica Luczak; Goya; Isabel Desimone; LE; Lorena Serrano; Lusso; M; MI; Nabaes Jodar; Natale; Omar Grossi; Rubén Pelagamos; S; Valinotto; Viegas, M.

		Argentino Interinstitucional de genomica de SARS-CoV-2' (PAIS Consortium)	
EPI_ISL_1396332 to 1396363	Laboratorio del Hospital Regional Ushuaia Gdor. Ernesto Campos	Nodo de Secuenciación Tierra del Fuego - Hospital Regional Ushuaia - Centro Austral De Investigaciones Científicas - Universidad Nacional De Tierra Del Fuego on behalf of 'Proyecto Argentino Interinstitucional de genomica de SARS-CoV-2' (PAIS Consortium)	Alejandro Ezequiel Rojas; Carina Andrea De Roccis; Carolina Beatriz Yulan; Cristina Fernanda Nardi; Fernando Gallego; Gabriel Alejandro Castro; Ivan Dario Gramundi; Manuel Fabian Boutureira; Santiago Guillermo Ceballos; Silvana Beatriz Cáceres
EPI_ISL_1395788, EPI_ISL_1395807	Laboratorio del Hospital Teresa Germani	Área de Secuenciación del Laboratorio de Virología del Hospital de Niños Dr. Ricardo Gutierrez on behalf of 'Proyecto Argentino Interinstitucional de genomica de SARS-CoV-2' (PAIS Consortium)	Acuña; Alexay; Anabel Luzio; D; Gisela Sauco; Goya; LE; Lusso; M; MI; Mauricio Spacaventto; Nabaes Jodar; Natale; S; Valinotto; Viegas, M.
EPI_ISL_1442994 to 1442999	Laboratorio di Genetica Medica Matera, Italy	Laboratorio di Genetica Medica Matera, Italy	Angela Mitidieri; Arianna Allegretti; Domenico Dell'Edera; Simone Francesca
EPI_ISL_1499593	Laboratorio di Microbiologia e Virologia, Università Vita-Salute San Raffaele, Milan	Laboratorio di Microbiologia e Virologia, Università Vita-Salute San Raffaele, Milan	Elena Criscuolo; Massimo Clementi; Matteo Castelli; Nicasio Mancini on behalf of SARS-CoV-2 ITALIAN RESEARCH ENTERPRISE-(SCIRE) Collaborative Group; Nicola Clementi; Roberta Antonia Diotti; Roberto Ferrarese
EPI_ISL_413489, EPI_ISL_1499504, EPI_ISL_1499572, EPI_ISL_1499594	Laboratorio di Microbiologia e Virologia, Università Vita-Salute San Raffaele, Milano	Laboratorio di Microbiologia e Virologia, Università Vita-Salute San Raffaele, Milano	C. Di Resta; E. Boeri; E. Criscuolo; Elena Criscuolo; G. Lo Raso; I. Negri; M. Castelli; M. Clementi; M. Sampaolo; Massimo Clementi; Matteo Castelli; N. Mancini & N. Clementi; Nicasio Mancini; Nicola Clementi; Nicola Clementi on behalf of SARS-CoV-2 ITALIAN RESEARCH ENTERPRISE-(SCIRE) Collaborative Group; R. Burioni; R. Ferrarese; R.A Diotti; Roberta Antonia Diotti; Roberto Ferrarese; V. Amato; V. Caputo; on behalf of SARS-CoV-2 ITALIAN RESEARCH ENTERPRISE-(SCIRE) Collaborative Group
EPI_ISL_1406780, EPI_ISL_1495144 to 1495149, EPI_ISL_1495151	Laboratorio microbiologia PO Cardarelli	Laboratorio microbiologia PO Cardarelli	Giovanna Niro; Giovanna niro; Massimiliano Scutella; Massimiliano Scutellà; Massimiliano scutella; Valentina Felice; Valentina felice; Valentna Felice; giovanna Niro
EPI_ISL_1406779, EPI_ISL_1406781	Laboratorio microbiologia PO Cardarelli	laboratorio microbiologia PO Cardarelli	Giovanna Niro; Massimiliano Scutellà; Valentina Felice
EPI_ISL_1538461, EPI_ISL_1539088 to 1539090	Laboratorium Analityczne Centrum	1. National Institute of Public Health - National Institute of Hygiene; 2. Eurofins Genomics Europe Sequencing GmbH	ECDC COVID-19 WGS support team; Eurofins Genomics Europe Sequencing Team; Gierczyki Rafa; Sadkowska-Todys Magorzata; Wokowicz Tomasz; Zacharczuk Katarzyna
EPI_ISL_1394274 to 1394280, EPI_ISL_1538513, EPI_ISL_1538604 to 1538609, EPI_ISL_1538777 to 1538779, EPI_ISL_1538950 to 1538956, EPI_ISL_1539045 to 1539046	Laboratorium Analiz Lekarskich Alab Rzeszów	1. National Institute of Public Health - National Institute of Hygiene; 2. Eurofins Genomics Europe Sequencing GmbH	ECDC COVID-19 WGS support team; Eurofins Genomics Europe Sequencing Team; Gierczyki Rafa; Sadkowska-Todys Magorzata; Wokowicz Tomasz; Zacharczuk Katarzyna
EPI_ISL_1394270 to 1394273, EPI_ISL_1538611, EPI_ISL_1538780 to 1538784, EPI_ISL_1538960 to 1538961, EPI_ISL_1539047 to 1539048, EPI_ISL_1539231 to 1539232	Laboratorium Analiz Lekarskich Alab Tarnobrzeg	1. National Institute of Public Health - National Institute of Hygiene; 2. Eurofins Genomics Europe Sequencing GmbH	ECDC COVID-19 WGS support team; Eurofins Genomics Europe Sequencing Team; Gierczyki Rafa; Sadkowska-Todys Magorzata; Wokowicz Tomasz; Zacharczuk Katarzyna
EPI_ISL_1393699 to 1393700, EPI_ISL_1393984 to 1393991, EPI_ISL_1538443, EPI_ISL_1538454, EPI_ISL_1538467, EPI_ISL_1538475, EPI_ISL_1538485, EPI_ISL_1538500, EPI_ISL_1538520, EPI_ISL_1538530 to 1538531, EPI_ISL_1538558 to 1538564, EPI_ISL_1538725 to 1538730, EPI_ISL_1538873, EPI_ISL_1538908, EPI_ISL_1539014 to 1539015, EPI_ISL_1539147, EPI_ISL_1539151, EPI_ISL_1539153			
see above	Laboratorium Badawczo-Rozwojowe Vitagenum	1. National Institute of Public Health - National Institute of Hygiene; 2. Eurofins Genomics Europe Sequencing GmbH	ECDC COVID-19 WGS support team; Eurofins Genomics Europe Sequencing Team; Gierczyki Rafa; Sadkowska-Todys Magorzata; Wokowicz Tomasz; Zacharczuk Katarzyna
EPI_ISL_1393701 to 1393708, EPI_ISL_1393993 to 1394002, EPI_ISL_1425561, EPI_ISL_1538555 to 1538557, EPI_ISL_1538731 to 1538734, EPI_ISL_1538903 to 1538907, EPI_ISL_1539016 to 1539017, EPI_ISL_1539183 to 1539184, EPI_ISL_1539216			
see above	Laboratorium Diagnostyczne Centrum Medycznego Luxmed	1. National Institute of Public Health - National Institute of Hygiene; 2. Eurofins Genomics Europe Sequencing GmbH	ECDC COVID-19 WGS support team; Eurofins Genomics Europe Sequencing Team; Gierczyki Rafa; Sadkowska-Todys Magorzata; Wokowicz Tomasz; Zacharczuk Katarzyna
EPI_ISL_1538491, EPI_ISL_1538748, EPI_ISL_1539142	Laboratorium Diagnostyczno-Analityczne	1. National Institute of Public Health - National Institute of Hygiene; 2. Eurofins Genomics Europe Sequencing GmbH	ECDC COVID-19 WGS support team; Eurofins Genomics Europe Sequencing Team; Gierczyki Rafa; Sadkowska-Todys Magorzata; Wokowicz Tomasz; Zacharczuk Katarzyna
EPI_ISL_1393871 to 1393873, EPI_ISL_1447261	Laboratorium Diagnostyczno-Analityczne Pozna	1. National Institute of Public Health - National Institute of Hygiene; 2. Eurofins Genomics Europe Sequencing GmbH	ECDC COVID-19 WGS support team; Eurofins Genomics Europe Sequencing Team; Gierczyki Rafa; Sadkowska-Todys Magorzata; Wokowicz Tomasz; Zacharczuk Katarzyna
EPI_ISL_1447202 to 1447203, EPI_ISL_1447235, EPI_ISL_1447284	Laboratorium Diagnostyki COVID-19 IGC PAN	1. National Institute of Public Health - National Institute of Hygiene; 2. Eurofins Genomics Europe Sequencing GmbH	ECDC COVID-19 WGS support team; Eurofins Genomics Europe Sequencing Team; Gierczyki Rafa; Sadkowska-Todys Magorzata; Wokowicz Tomasz; Zacharczuk Katarzyna
EPI_ISL_1538445, EPI_ISL_1538493, EPI_ISL_1538785, EPI_ISL_1539049 to 1539052	Laboratorium Diagnostyki Molekularnej,Cytogenetyki i Cytometrii	1. National Institute of Public Health - National Institute of Hygiene; 2. Eurofins Genomics Europe Sequencing GmbH	ECDC COVID-19 WGS support team; Eurofins Genomics Europe Sequencing Team; Gierczyki Rafa; Sadkowska-Todys Magorzata; Wokowicz Tomasz; Zacharczuk Katarzyna
EPI_ISL_1394176 to 1394180, EPI_ISL_1425547	Laboratorium Epidemiologii	1. National Institute of Public Health - National Institute of Hygiene; 2. Eurofins Genomics Europe Sequencing GmbH	ECDC COVID-19 WGS support team; Eurofins Genomics Europe Sequencing Team; Gierczyki Rafa; Sadkowska-Todys Magorzata; Wokowicz Tomasz; Zacharczuk Katarzyna
EPI_ISL_1538441, EPI_ISL_1538478, EPI_ISL_1538498, EPI_ISL_1538506, EPI_ISL_1538508, EPI_ISL_1538524, EPI_ISL_1538528, EPI_ISL_1538626 to 1538636, EPI_ISL_1538808 to 1538820, EPI_ISL_1538855, EPI_ISL_1538858, EPI_ISL_1538874, EPI_ISL_1538965 to 1538970, EPI_ISL_1539060 to 1539061, EPI_ISL_1539123, EPI_ISL_1539139, EPI_ISL_1539152, EPI_ISL_1539171, EPI_ISL_1539177, EPI_ISL_1539203 to 1539205, EPI_ISL_1539233 to 1539235			
see above	Laboratorium Epidemiologii WSSE w Szczecinie	1. National Institute of Public Health - National Institute of Hygiene; 2. Eurofins Genomics Europe Sequencing GmbH	ECDC COVID-19 WGS support team; Eurofins Genomics Europe Sequencing Team; Gierczyki Rafa; Sadkowska-Todys Magorzata; Wokowicz Tomasz; Zacharczuk Katarzyna
EPI_ISL_1393992, EPI_ISL_1394010 to 1394013	Laboratorium Genetyczne Alab Plus Sp. z o.o.	1. National Institute of Public Health - National Institute of Hygiene; 2. Eurofins Genomics Europe Sequencing GmbH	ECDC COVID-19 WGS support team; Eurofins Genomics Europe Sequencing Team; Gierczyki Rafa; Sadkowska-Todys Magorzata; Wokowicz Tomasz; Zacharczuk Katarzyna
EPI_ISL_1393710 to 1393716, EPI_ISL_1394003 to 1394005, EPI_ISL_1425541, EPI_ISL_1538465, EPI_ISL_1538484, EPI_ISL_1538737	Laboratorium INNGEN	1. National Institute of Public Health - National Institute of Hygiene; 2. Eurofins Genomics Europe Sequencing GmbH	ECDC COVID-19 WGS support team; Eurofins Genomics Europe Sequencing Team; Gierczyki Rafa; Sadkowska-Todys Magorzata; Wokowicz Tomasz; Zacharczuk Katarzyna
EPI_ISL_1415898	Laboratorium Intibios Karawang	National Institute of Health Research and Development	Arie Ardiansyah Nugraha; Hana Apsari Pawestri; Hartanti Dian Ikawati; Kartika Dewi Puspa; Krisna Nur Andriana Pangesti; Nelly Puspandari; Subangkit; Vivi Setiawaty
EPI_ISL_511879	Laboratorium Kesehatan Provinsi Jawa Barat	Molecular Genetics Laboratory-Faculty of Medicine-Universitas Padjadjaran; School of Life Sciences and Technology & School of Pharmacy-Institut Teknologi Bandung; Laboratorium Kesehatan Provinsi Jawa Barat	Agung Eru Wibowo; Azzania Fibriani; Catur Riani; Cut Nur Cinthia Alamanda; Ema Rahmawati; Hammam Riza; Hesti Lina Wiraswati; Husna Nugrahapraja; Lia Faridah; Marselina Irasonia Tan; Rifky Waluyajati Rachman; Ryan Bayusantika Ristandi; Savira Ekawardhani; Soni Solistia Wirawan; Tarwadi; Yunia Sribudiani
EPI_ISL_528745	Laboratorium Kesehatan Provinsi Jawa Barat	School of Life Sciences and Technology & School of Pharmacy-Institut Teknologi Bandung; Molecular Genetics Laboratory-Faculty of Medicine-Universitas Padjadjaran; Laboratorium Kesehatan Provinsi Jawa Barat	Agung Eru Wibowo; Azzania Fibriani; Catur Riani; Cut Nur Cinthia Alamanda; Ema Rahmawati; Fensi Amalina; Hammam Riza; Hesti Lina Wiraswati; Husna Nugrahapraja; Irvan Faizal; Lia Faridah; Marselina Irasonia Tan; Mas Rizky A.A. Syamsunarno; Rifky Waluyajati Rachman; Ryan Bayusantika Ristandi; Savira Ekawardhani; Sony Solistia Wirawan; Tarwadi; Tri Hanggono Achmad; Yunia Sribudiani
EPI_ISL_1394233 to 1394246, EPI_ISL_1538600 to 1538603, EPI_ISL_1538826, EPI_ISL_1538875, EPI_ISL_1538878, EPI_ISL_1538943 to 1538948, EPI_ISL_1539070 to 1539075			
see above	Laboratorium Medyczne BIOLAB	1. National Institute of Public Health - National Institute of Hygiene; 2. Eurofins Genomics Europe Sequencing GmbH	ECDC COVID-19 WGS support team; Eurofins Genomics Europe Sequencing Team; Gierczyki Rafa; Sadkowska-Todys Magorzata; Wokowicz Tomasz; Zacharczuk Katarzyna

EPI_ISL_1538827, EPI_ISL_1538877, EPI_ISL_1538949, EPI_ISL_1539076 to 1539077, EPI_ISL_1539166	Laboratorium Medyczne GEN LAB	1. National Institute of Public Health - National Institute of Hygiene; 2. Eurofins Genomics Europe Sequencing GmbH	ECDC COVID-19 WGS support team; Eurofins Genomics Europe Sequencing Team; Gierczyki Rafa; Sadkowska-Todys Magorzata; Wokowicz Tomasz; Zacharczuk Katarzyna
EPI_ISL_1393813 to 1393819, EPI_ISL_1393822 to 1393828, EPI_ISL_1393830, EPI_ISL_1393832 to 1393833, EPI_ISL_1394119 to 1394137, EPI_ISL_1425546, EPI_ISL_1538645 to 1538648, EPI_ISL_1538844 to 1538845, EPI_ISL_1538869, EPI_ISL_1538982, EPI_ISL_1539102 to 1539105, EPI_ISL_1539237			
see above	Laboratorium Medyczne LAB-MED	1. National Institute of Public Health - National Institute of Hygiene; 2. Eurofins Genomics Europe Sequencing GmbH	ECDC COVID-19 WGS support team; Eurofins Genomics Europe Sequencing Team; Gierczyki Rafa; Sadkowska-Todys Magorzata; Wokowicz Tomasz; Zacharczuk Katarzyna
EPI_ISL_1447214, EPI_ISL_1447229, EPI_ISL_1447251, EPI_ISL_1538489, EPI_ISL_1538545 to 1538546, EPI_ISL_1538891, EPI_ISL_1539167			
see above	Laboratorium Mikrobiologiczne Dolnolskie Centrum Transplantacji Komórkowych z Krajowym Bankiem Dawców Szpiku	1. National Institute of Public Health - National Institute of Hygiene; 2. Eurofins Genomics Europe Sequencing GmbH	ECDC COVID-19 WGS support team; Eurofins Genomics Europe Sequencing Team; Gierczyki Rafa; Sadkowska-Todys Magorzata; Wokowicz Tomasz; Zacharczuk Katarzyna
EPI_ISL_1394101 to 1394104, EPI_ISL_1538859, EPI_ISL_1538863, EPI_ISL_1538929 to 1538931, EPI_ISL_1539056 to 1539058	Laboratorium Mikrobiologiczne RSS w Grudzidzu	1. National Institute of Public Health - National Institute of Hygiene; 2. Eurofins Genomics Europe Sequencing GmbH	ECDC COVID-19 WGS support team; Eurofins Genomics Europe Sequencing Team; Gierczyki Rafa; Sadkowska-Todys Magorzata; Wokowicz Tomasz; Zacharczuk Katarzyna
EPI_ISL_1538457, EPI_ISL_1538463, EPI_ISL_1538566, EPI_ISL_1538912	Laboratorium Mikrobiologiczne Radomski Szpital Specjalistyczny im Dr Tytusa Chaubiskiego	1. National Institute of Public Health - National Institute of Hygiene; 2. Eurofins Genomics Europe Sequencing GmbH	ECDC COVID-19 WGS support team; Eurofins Genomics Europe Sequencing Team; Gierczyki Rafa; Sadkowska-Todys Magorzata; Wokowicz Tomasz; Zacharczuk Katarzyna
EPI_ISL_1539126	Laboratorium Mikrobiologii SZKPP Uniwersytetu Medycznego w Poznaniu	1. National Institute of Public Health - National Institute of Hygiene; 2. Eurofins Genomics Europe Sequencing GmbH	ECDC COVID-19 WGS support team; Eurofins Genomics Europe Sequencing Team; Gierczyki Rafa; Sadkowska-Todys Magorzata; Wokowicz Tomasz; Zacharczuk Katarzyna
EPI_ISL_1538683	Laboratorium Mikrobiologii Szpital Kliniczny Przemienienia Paskiego UM w Poznaniu	1. National Institute of Public Health - National Institute of Hygiene; 2. Eurofins Genomics Europe Sequencing GmbH	ECDC COVID-19 WGS support team; Eurofins Genomics Europe Sequencing Team; Gierczyki Rafa; Sadkowska-Todys Magorzata; Wokowicz Tomasz; Zacharczuk Katarzyna
EPI_ISL_1393689 to 1393698	Laboratorium badawczo-rozwojowe VITAGENUM	1. National Institute of Public Health - National Institute of Hygiene; 2. Eurofins Genomics Europe Sequencing GmbH	ECDC COVID-19 WGS support team; Eurofins Genomics Europe Sequencing Team; Gierczyki Rafa; Sadkowska-Todys Magorzata; Wokowicz Tomasz; Zacharczuk Katarzyna
EPI_ISL_1393762 to 1393771, EPI_ISL_1393773 to 1393777, EPI_ISL_1394181 to 1394201, EPI_ISL_1425548	Laboratorum Epidemiologii WSSE w Szczecinie	1. National Institute of Public Health - National Institute of Hygiene; 2. Eurofins Genomics Europe Sequencing GmbH	ECDC COVID-19 WGS support team; Eurofins Genomics Europe Sequencing Team; Gierczyki Rafa; Sadkowska-Todys Magorzata; Wokowicz Tomasz; Zacharczuk Katarzyna
EPI_ISL_1421755 to 1421778, EPI_ISL_1421780 to 1422215, EPI_ISL_1461710 to 1461815, EPI_ISL_1461817 to 1461836, EPI_ISL_1461838 to 1461994, EPI_ISL_1461996 to 1462248, EPI_ISL_1462250 to 1462499, EPI_ISL_1462501, EPI_ISL_1462504, EPI_ISL_1462507, EPI_ISL_1462511, EPI_ISL_1462514, EPI_ISL_1462517, EPI_ISL_1462520, EPI_ISL_1462522, EPI_ISL_1462529, EPI_ISL_1462532, EPI_ISL_1462535, EPI_ISL_1462538, EPI_ISL_1462541, EPI_ISL_1462544, EPI_ISL_1462547, EPI_ISL_1462550, EPI_ISL_1462554, EPI_ISL_1462557, EPI_ISL_1462560, EPI_ISL_1462562, EPI_ISL_1462565, EPI_ISL_1462568, EPI_ISL_1462571, EPI_ISL_1462574, EPI_ISL_1462577, EPI_ISL_1462580, EPI_ISL_1462583, EPI_ISL_1462586, EPI_ISL_1462590, EPI_ISL_1462592, EPI_ISL_1462595, EPI_ISL_1462598, EPI_ISL_1462602, EPI_ISL_1462605, EPI_ISL_1462608, EPI_ISL_1462611, EPI_ISL_1462614, EPI_ISL_1462616, EPI_ISL_1462619, EPI_ISL_1462622, EPI_ISL_1462625 to 1463067, EPI_ISL_1463069 to 1463150, EPI_ISL_1463152 to 1463256, EPI_ISL_1463258 to 1463444, EPI_ISL_1480977 to 1481548, EPI_ISL_1513631 to 1515761, EPI_ISL_1548117 to 1548157, EPI_ISL_1548159 to 1548426, EPI_ISL_1548428 to 1548503, EPI_ISL_1548505 to 1548644, EPI_ISL_1548646 to 1549037, EPI_ISL_1549039 to 1549133, EPI_ISL_1549141 to 1549167, EPI_ISL_1549169 to 1549213, EPI_ISL_1549215 to 1549237, EPI_ISL_1549239 to 1549477, EPI_ISL_1549479 to 1549486, EPI_ISL_1549488 to 1549569, EPI_ISL_1549571, EPI_ISL_1549574, EPI_ISL_1549577, EPI_ISL_1549580, EPI_ISL_1549583, EPI_ISL_1549585, EPI_ISL_1549589 to 1549606, EPI_ISL_1549608 to 1549715, EPI_ISL_1549717 to 1549726, EPI_ISL_1549728 to 1549755, EPI_ISL_1549757 to 1549853, EPI_ISL_1549855 to 1549902, EPI_ISL_1549905 to 1549986, EPI_ISL_1549988 to 1550054, EPI_ISL_1550056 to 1550158, EPI_ISL_1550160 to 1550273, EPI_ISL_1550275 to 1550387			
see above	Laboratory Corporation of America	Centers for Disease Control and Prevention Division of Viral Diseases, Pathogen Discovery	Adrian Paskey; Amanda Douglas; Amanda Suchanek; Andrea Throop; Ayla Burns; Ben L. Rambo-Martin; Benjamin Rambo-Martin; Bobbi Croy; Brian Krueger; Brian Norvell; Christopher Gulvick; Christos Petropoulos; Clinton R. Paden; Craig Lukasik; Dakota Howard; Darlene Wagner; Debbie Boles; Dhwani Batra; Duncan MacCannell; Eyad Almasri; Goran Stevovic; Howard Engler; Hrushikesh Deshmukh; Jake Humphrey; Jana Schroth; Jason Caravas; Joe Voshell; John Pruitt; Jonathan Meltzer; Jonathan Williams; Kara Moser; Kimberly Wagner; Lax Iyer; Lyndon Tilson; Manoj Jain; Marcia Eisenberg; Mary Ann Cristobal; Mary Williamson; Matthew Schmerer; Michael Levandoski; Mike Sapeta; Mindy Nye; Minoo Agarwal; Mohan Kolli; Nuthawin Charoensri; Oren Cohen; Peter W. Cook; Prashant Gupta; Qian Zeng; Rama Ghatti; Scott Parker; Scott Ryan; Scott Sammons; Shatavia Morrison; Stanley Letovsky; Steven Ragan; Suresh Babu Selvaraju; Susan Countryman; Susan Hicks; Suxiang Tong; Suzanne Dale; Thomas Urban; Tim Kuphal; Tricia Zwiefelhofer; Vincent Drouillon; Yvette Unoarumhi
EPI_ISL_476403 to 476405	Laboratory DASA	Instituto de Medicina Tropical da Univesidade de São Paulo	Camila Alves Maia da Silva; Darlan da Silva Candido; Erika Regina Manuli; Ester Sabino; Flavia Cristina da Silva Sales; Giula Magalhaes Ferreira; Jaqueline Goes de Jesus; Julien Theze; Mariana Severo Ramundo; Nuno Faria; Samples: José Eduardo Levi; Sequencing: Ingra Morales Claro; Thais de Moura Coletti
EPI_ISL_514425 to 514431, EPI_ISL_541649 to 541655, EPI_ISL_541657 to 541662, EPI_ISL_582530	Laboratory Diagnostic, Veterinary Specialized Institute Kraljevo	Laboratory Diagnostic, Veterinary Specialized Institute Kraljevo	Afonso, C.; Banovic Djeri, B.; Jankovic, M.; Jovanovic, T.; Knezevic, A.; Petrovic, T.; Sekler, M.; Tesovic, B.; Vidanovic, D.; Volkening, J.
EPI_ISL_445086 to 445087, EPI_ISL_482744, EPI_ISL_541656	Laboratory Diagnostic, Veterinary Specialized Institute Kraljevo	Laboratory Diagnostic, Veterinary Specialized Institute Kraljevo	Afonso, C.; Banovic Djeri, B.; C.L.; Debeljak, Z.; Dmitric, M.; J. and Alfonso; J. and Alfonso, C.; Jankovic, M.; Jovanovic, T.; Knezevic, A.; Matovic, K.; Petrovic, T.; Sekler, M.; Tesovic, B.; Vaskovic, N.; Vidanovic, D.; Volkening; Volkening, J.
EPI_ISL_476220 to 476221, EPI_ISL_476230 to 476231, EPI_ISL_476233 to 476236, EPI_ISL_476406 to 476407, EPI_ISL_476426 to 476427	Laboratory Fleury	Instituto de Medicina Tropical da Univesidade de São Paulo	Camila Alves Maia da Silva; Darlan da Silva Candido; Erika Regina Manuli; Ester Sabino; Flavia Cristina da Silva Sales; Giula Magalhaes Ferreira; Jaqueline Goes de Jesus; Julien Theze; Mariana Severo Ramundo; Nuno Faria; Samples: Celso Granato; Sequencing: Ingra Morales Claro; Thais de Moura Coletti
EPI_ISL_415741 to 415743, EPI_ISL_417518 to 417525, EPI_ISL_424969 to 424975, EPI_ISL_424978, EPI_ISL_444274 to 444278, EPI_ISL_452178 to 452179, EPI_ISL_464092 to 464094			
see above	Laboratory Medicine	Department of Laboratory Medicine, Lin-Kou Chang Gung Memorial Hospital, Taoyuan, Taiwan	Cheng-Hsun Chiu; Cheng-Ta Yang; Chi-Hsien Huang; Chung-Guei Huang; Guang-Wu Chen; Kuang-Tso Le; Kuo-Chien Tsao; Kuo-Ming Lee; Mei-Jen Hsiao; Peng-Nien Huang; Po-Wei Huang; Shin-Ru Shih; Shu-Li Yang; Shu-Min Lin; Yi-Chun Liu; Yu-Nong Gong
EPI_ISL_411915	Laboratory Medicine	Department of Laboratory Medicine, Lin-Kou Chang Gung Memorial Hospital, Taoyuan, Taiwan.	Chung-Guei Huang; Kuo-Chien Tsao; Shin-Ru Shih; Shu-Li Yang; Yhu-Chering Huang; Yi-Chun Li; Yu-Nong Gong
EPI_ISL_529208 to 529212	Laboratory Medicine, University of Washington	University of Washington, Laboratory Medicine	Greninger, A.; Jerome, K.; Roychoudhury, P.
EPI_ISL_1508895	Laboratory for HIV and opportunistic infections diagnosis The Republican Research and Practical Center for Epidemiology and Microbiology (RRPCEM)	Laboratory for HIV and opportunistic infections diagnosis The Republican Research and Practical Center for Epidemiology and Microbiology (RRPCEM)	Alena Mikhalenka; Alexander Kilchevcky; Alina Drozd; Anatoly Krasko; Elena Gasich; Kirill Bulda; Leonid Valentovich; Nastassia Kabankova; Vladimir Gorbunov; Yauhen Sysaliatsin
EPI_ISL_445243, EPI_ISL_455468 to 455477, EPI_ISL_455479	Laboratory for Respiratory Viruses, Cantacuzino National Military-Medical Institute for Research and Development	Cantacuzino Institute	A.Cretu; L.Ustea; M.Lazar; T.Durfee; Tim Durfee
EPI_ISL_471416 to 471424	Laboratory for Respiratory Viruses, National Influenza Centre, Cantacuzino National Military-Medical Institute for Research and Development	Cantacuzino Institute	Luiza Ustea; Mihaela Lazar; Nicoleta Paraschiv; Tim Durfee
EPI_ISL_437689	Laboratory for Urgent Response to Biological Threats	Institut Pasteur CIBU / ERI	A. Kwasiborski; C. Balière; C. Batéjat; J. Vanhomwegen; JC. Manuguerra; V. Caro; V. Hourdel
EPI_ISL_437690	Laboratory for Urgent Response to Biological Threats	Institut Pasteur CIBU /ERI	A. Kwasiborski; C. Balière; C. Batéjat; H. Hourdel; J. Vanhomwegen; JC. Manuguerra; V. Caro
EPI_ISL_435045 to 435048	Laboratory of Applied Genetics	RSE "National Center for Biotechnology"	Alexandr Shevtsov; Asylulan Amirgazin; Ilyas Akhmetollayev; Ruslan Kalendar; Viktoriya Lutsay; Yerlan Ramanculov
EPI_ISL_572398	Laboratory of Biology and Identification of Arboviruses	Pathogenic Microorganisms Variability Laboratory	Alexander Gintsburg; Alexey Prilipov; Alexey Shchetinin; Alina Kozlova; Andrei Siniavin; Maria Nikiforova; Muhammad Saifullin; Victor Larichev; Vladimir Gushchin
EPI_ISL_1500433 to 1500445, EPI_ISL_1547806 to 1547826	Laboratory of Clinical Microbiology, Virology and Bioemergencies, ASST Fatebenefratelli Sacco - Sacco	Laboratory of Clinical Microbiology, Virology and Bioemergencies, ASST Fatebenefratelli Sacco - Sacco	Alberto Rizzo; Alessandro Mancon; Fiorenza Bracchitta; Luca Rizzuto; Maria Rita Gismondo; Valeria Micheli

	Hospital	Hospital	
EPI_ISL_1420660	Laboratory of Clinical Virology	Greek Genome Center, Biomedical Research Foundation of the Academy of Athens (BRFAA)	Dimitrios Thanos; Emmanouil Athanasiadis; George Sourvinos; Ioannis Vatsellas; Katerina Zoi; Theodoros Loupis
EPI_ISL_1469989 to 1470000, EPI_ISL_1470070 to 1470077	Laboratory of Communicable Diseases	1. Laboratory of Communicable Diseases (Estonia); 2. Eurofins Genomics Europe Sequencing GmbH	Liidia Dotsenko et al.
EPI_ISL_477160, EPI_ISL_477163	Laboratory of Dr. John Lednicky	University of Florida	Chang-Yu Wu; John A. Lednicky; John Glenn Morris, Jr.; Kuttichantran Subramaniam; Maha A. Elbadry; Thomas B. Waltzek; and John Glenn Morris, Jr.
EPI_ISL_1527390 to 1527398	Laboratory of Extremely Dangerous Infections of Federal Budget Health Care Instute "Center of Hygiene and Epidemiology in Sakhalin region"	Group of Genomics and Postgenomic Technologies of Central Research Institute of Epidemiology	Akimkin VG; Arutiunian AE; Bulanenko VP; Byankina MA; Golubeva AG; Kaptelova VV; Kondrasheva LY; Korneenko EV; Saenko SS; Samoilov AE; Speranskaya AS; Tivanova EV; Valdokhina AV; Zagorodnov YL
EPI_ISL_1510606	Laboratory of Extremely Dangerous Infections of Federal Budget Health Care Instute "Center of Hygiene and Epidemiology in Sverdlovsk region"	Group of Genomics and Postgenomic Technologies of Central Research Institute of Epidemilology	Akimkin VG; Berlina YY; Bulanenko VP; Cherkashina AS; Durasova AL; Egorova EA; Esaulkova AY; Golubeva AG; Kaptelova VV; Kondrasheva LY; Korneenko EV; Saenko SS; Samoilov AE; Shipulina OY; Snitkovskaya TE; Solovyeva ED; Speranskaya AS; Tivanova EV; Valdokhina AV; Zotova MI
EPI_ISL_437625	Laboratory of Genomics & Bioinformatics, Institute of Immunology and Experimental Therapy, Polish Academy of Sciences Oddzial Mikrobiologii Wojewodzkiej Stacji Sanitarno-Epidemiologicznej.	Laboratory of Genomics & Bioinformatics, Institute of Immunology and Experimental Therapy, Polish Academy of Sciences	Aleksandra Herud; Dariusz Martynowski; Dorota Kujawa; Grazyna Zalewska; Joanna Sikorska; Krzysztof Jakub Pawlik; Oskar Karpinski and Lukasz Laczmanski; Paulina Zebrowska
EPI_ISL_1420724 to 1420732	Laboratory of Immunohematology, Division of Hematology	Greek Genome Center, Biomedical Research Foundation of the Academy of Athens (BRFAA)	Athanasia Mouzaki; Dimitrios Thanos; Emmanouil Athanasiadis; Ioannis Vatsellas; Katerina Zoi; Theodoros Loupis
EPI_ISL_536399	Laboratory of Immunovirology. Universidad de Antioquia	Instituto Nacional de Salud - Unidad de Secuenciación y Genómica	Carlos Franco-Muñoz; Diego Álvarez-Diaz and Marcela Mercado-Reyes; Francisco J. Díaz; Katherine Laiton-Donato; Lizdany Flórez; Wbeimar Aguilar-Jimenez
EPI_ISL_452327 to 452364	Laboratory of Infectious Diseases Center of Beijing Ditan Hospital	Laboratory of Infectious Diseases Center of Beijing Ditan Hospital	Chengjie Jie; Fengting Yu; Linghang Wang; Liting Yan; Siyuan Yang; Yunxia Tang
EPI_ISL_417445 to 417447	Laboratory of Infectious Diseases, Department of Biomedical and Clinical Sciences L. Sacco, University of Milan	Laboratory of Infectious Diseases, Department of Biomedical and Clinical Sciences L. Sacco, University of Milan	Agostino Riva; Alessia Lai; Annalisa Bergna; Arianna Gabrieli; Claudia Balotta; Dario Bernacchia; Gianguglielmo Zehender; Giuliano Rizzardini; Luca Meroni; Maciej Tarkowski; Massimo Galli; Spinello Antinori; Stefano Rusconi
EPI_ISL_418263 to 418265	Laboratory of Microbiology, Department of Medicine, National and Kapodistrian University of Athens, Greece	Laboratory of Biology, Department of Medicine, Democritus University of Thrace, Greece	Elisavet Gatzidou; Ioannis Karakasiliotis; Maria Bampali; Nikolaos Dovrolis; Nikolaos Spanakis; Stavroula Veletza
EPI_ISL_427043, EPI_ISL_434455 to 434486, EPI_ISL_437874 to 437911	Laboratory of Microbiology, Medical School, National and Kapodistrian University of Athens	Laboratory of Biology, Department of Medicine, Democritus University of Thrace	Bampali, M.; Dovrolis, N.; Froukala, E.; Gatzidou, E.; Kassela K.; N. and Karakasiliotis, I.; Spanakis; Stavropoulou, A.; Tsakris, A.; Veletza, S.
EPI_ISL_613563 to 613564, EPI_ISL_613707 to 613708	Laboratory of Molecular Biology, Blood Center of Ribeirão Preto	Laboratory of Molecular Biology, Blood Center of Ribeirão Preto, Faculty of Medicine of Ribeirão Preto, University of São Paulo	Aparecida Y Yamamoto; Diego Villa Clé; Dimas T Covas; Elaine V Santos; Evandra S Rodrigues; Glauco de Carvalho Pereira; Joilson Xavier; Luiz CJ Alcantara; Marta Giovanetti; Rodrigo T Calado; Simone Kashima; Svetoslav N Slavov; Talita Adelino; Vagner Fonseca
EPI_ISL_613709, EPI_ISL_613711, EPI_ISL_613951 to 613952, EPI_ISL_613954, EPI_ISL_613956, EPI_ISL_613961 to 613965, EPI_ISL_614011, EPI_ISL_614155 to 614156			
see above	Laboratory of Molecular Biology, Blood Center of Ribeirão Preto, Faculty of Medicine of Ribeirão Preto, University of São Paulo	Laboratory of Molecular Biology, Blood Center of Ribeirão Preto, Faculty of Medicine of Ribeirão Preto, University of São Paulo	Aparecida Y Yamamoto; Diego Villa Clé; Dimas T Covas; Elaine V Santos; Evandra S Rodrigues; Glauco de Carvalho Pereira; Joilson Xavier; Luiz CJ Alcantara; Marta Giovanetti; Rodrigo T Calado; Simone Kashima; Svetoslav N Slavov; Talita Adelino; Vagner Fonseca
EPI_ISL_428209, EPI_ISL_450746, EPI_ISL_451644 to 451647	Laboratory of Molecular Biology, Diagnostyka sp. z o.o.	Laboratory of Recombinant Vaccines	Anna Piotrowska-Mietelska; Boguslaw Szewczyk; Krystyna Bienkowska-Szewczyk; Lukasz Rabalski; Maciej Kosinski
EPI_ISL_425228 to 425229, EPI_ISL_426051, EPI_ISL_426356 to 426360	Laboratory of Molecular Genetics, 2nd Faculty of Medicine, Charles University in Prague, Prague, Czech Republic	Laboratory of Molecular Genetics, 2nd Faculty of Medicine, Charles University in Prague, Prague, Czech Republic	Katerina Polackova; Kateina Poláková; Lenka Kramna; Lenka Kramná; Ondrej Cinek; Ondej Cinek
EPI_ISL_428853, EPI_ISL_498559 to 498563	Laboratory of Molecular Virology International Center for Genetic Engineering and Biotechnology (ICGEB)	ARGO Open Lab Platform for Genome Sequencing	D'Agaro P; Dal Monego S; D'Agaro P; Licastro D; Marcello A; Rajasekharan S; Segat L
EPI_ISL_417419, EPI_ISL_417421, EPI_ISL_417423, EPI_ISL_428854	Laboratory of Molecular Virology International Center for Genetic Engineering and Biotechnology (ICGEB)	ARGO Open Lab Platform for Genome sequencing	D'Agaro P; Dal Monego S; Licastro D; Marcello A; Rajasekharan; Rajasekharan S; Segat L
EPI_ISL_417418	Laboratory of Molecular Virology International Center fro Genetic Engineering and Biotechnology (ICGEB)	ARGO Open Lab Platform for Genome sequencing	D'Agaro P; Dal Monego S; Licastro D; Marcello A; Rajasekharan S; Segat L
EPI_ISL_479616 to 479619, EPI_ISL_479790 to 479791, EPI_ISL_525495 to 525496	Laboratory of Molecular Virology of the International Centre for Genetic Engineering and Biotechnology (ICGEB)	ARGO Open Lab Platform for Genome Sequencing	Confalonieri M; Confalonieri M Marcello A; Confalonieri P; D; D'Agaro P; Dal Monego S; Licastro; Licastro D; Marcello A; Rajasekharan S; Salton F; Segat L
EPI_ISL_584048 to 584049, EPI_ISL_584051 to 584052	Laboratory of Molecular Virology, Department of Biomedical, Surgical and Dental Sciences University of Milano	Laboratory of Molecular Virology, Department of Biomedical, Surgical and Dental Sciences University of Milano	Anselmi; B. and Ferrante, P.; Basilico, N.; Bianchi, M.; Binda, S.; C. and Ferrante, P.; D'Alessandro, S.; Delbue, S.; Dolci, M.; E; Fattori, M.; Ferrante, P.; G. and Pariani; Galli; Galli, C.; Incorvaia; Modenese, A.; Parapini, S.; Pariani, E.; Primache, V.; Signorini, L.; Taramelli, D.
EPI_ISL_415658, EPI_ISL_415660 to 415661	Laboratory of Molecular Virology, Pontificia Universidad Católica de Chile	MSHS Pathogen Surveillance Program	Ajay Obla; Ana Silvia Gonzalez-Reiche; Eileen Serrano; Hala Alshammary; Harm van Bakel; Juan Soto; Matthew Hernandez; Melissa Smith; Mitchell Sullivan; Pablo Vial; Rafael A. Medina; Robert Sebra; Shwetha Sridhar Hara; Tamara Garcia; Viviana Simon; Ying-Chih Wang; Zenab Khan
EPI_ISL_1406696	Laboratory of Respiratory Viruses Teaching and Clinical Center of the Medical University of Lodz	Biobank Lab, Department of Molecular Biophysics, Faculty of Biology and Environmental Protection, University of Lodz	Borowiec Maciej.; Dastych Jaroslaw; Dród Izabela; Dziadek Jarosaw; Paradowska Edyta; Sobalska-Kwapis Marta; Strapagiel Dominik; Traczyk-Borszyska Magdalena; Tymoniuk Bogusaw
EPI_ISL_1489924 to 1489926, EPI_ISL_1495043, EPI_ISL_1495045, EPI_ISL_1495047, EPI_ISL_1495049, EPI_ISL_1495051, EPI_ISL_1495053 to 1495054			
see above	Laboratory of Respiratory Viruses Teaching and Clinical Center of the Medical University of Lodz	Biobank Lab, Department of Molecular Biophysics, Faculty of Biology and Environmental Protection, University of Lodz	Borowiec Maciej.; Dastych Jaroslaw; Dród Izabela; Dziadek Jarosaw; Paradowska Edyta; Sobalska-Kwapis Marta; Strapagiel Dominik; Traczyk-Borszyska Magdalena; Tymoniuk Bogusaw
EPI_ISL_1543934, EPI_ISL_1543937, EPI_ISL_1543985, EPI_ISL_1543987	Laboratory of Respiratory Viruses Teaching and Clinical Center of the Medical University of Lodz	Laboratory of Respiratory Viruses Teaching and Clinical Center of the Medical University of Lodz	; Borowiec Maciej; Dastych Jaroslaw; Dród Izabela; Dziadek Jarosaw; Paradowska Edyta; Sobalska-Kwapis Marta; Strapagiel Dominik; Traczyk-Borszyska Magdalena; Tymoniuk Bogusaw
EPI_ISL_427294 to 427304, EPI_ISL_456071 to 456075, EPI_ISL_456079 to 456081, EPI_ISL_456084 to 456087, EPI_ISL_456089 to 456106, EPI_ISL_467344 to 467371, EPI_ISL_541347 to 541369, EPI_ISL_1402429 to 1402431, EPI_ISL_1533980, EPI_ISL_1533983, EPI_ISL_1534005, EPI_ISL_1534011, EPI_ISL_1534014 to 1534016			
see above	Laboratory of Respiratory Viruses and Measles, Oswaldo Cruz Institute, FIOCRUZ	Laboratory of Respiratory Viruses and Measles, Oswaldo Cruz Institute, FIOCRUZ	Alex Pauvolid-Correa; Alice Sampaio Rocha; Aline Mattos; Ana Beatriz Machado Lima; Ana Carolina Mendonca; Ana Carolina Mendonça; Anna Carolina Paixao; Anna Carolina Paixão; Braulia Caetano; Cinthia Avila; Cristiana Garcia; Felipe Naveca; Fernando Motta; Jonathan Lopes; Luciana Appolinario; Maria Ogrzewalska; Marilda Siqueira on behalf of the Fiocruz COVID-19 Genomic Surveillance Network; Mia Ferreira Araujo; Milene Miranda; Paola Resende; Priscila Born; Renata Serrano Lopes; Roxana Loayza; Sunando Roy
EPI_ISL_1527382 to 1527389	Laboratory of Virology of Federal Budget Health Care Institution "Center of Hygiene and Epidemiology in Arkhangelsk region"	Group of Genomics and Postgenomic Technologies of Central Research Institute of Epidemilology	Akimkin VG; Bulanenko VP; Cherkashina AS; Golubeva AG; Kaptelova VV; Kondrasheva LY; Korneenko EV; Ponomareva YA; Saenko SS; Samoilov AE; Semyina LV; Shishko LA; Speranskaya AS; Tivanova EV; Valdokhina AV
EPI_ISL_451298 to 451305	Laboratory of Virology, INMI Lazzaro Spallanzani IRCCS	Laboratory of Virology, INMI Lazzaro Spallanzani IRCCS	Antonino Di Caro; Barbara Bartolini; Cesare E.M. Gruber; Francesco Messina; Giuseppe Ippolito; Maria R. Capobianchi; Martina Rueca
EPI_ISL_1489930, EPI_ISL_1489932	Laboratory of Virology, National center of expertise	RSE "National Center of Expertise" and RSE "National center for Biotechnology"	Abdaliyev Askar; Amirgazin Asylulan; Balykbaev Kanat; Kamalova Dinara; Ramankulov Erlan; Sharipova Saule; Shevtsov Alexandr; Tungushbayev Talgat
EPI_ISL_454575	Laboratory of virology, National Center of Expertise	Laboratory of molecular-genetic research, National Center for	; Abdaliyev Askar; Akhmetollayev Ilyas; Amirgazin Asylulan; Aushakhmetova Zabira; Kalendar Ruslan; Lutsay Viktoriya; Rakhmetova Akbota; Ramankulov

		Expertise, Kazakhstan National Center for Biotechnology, Kazakhstan	Yerlan; Shevtsov Alexandr
EPI_ISL_454584 to 454586, EPI_ISL_454589	Laboratory of virology, National Center of Expertise	Laboratory of molecular-genetic research, National Center for Expertise, Kazakhstan National Center for Biotechnology, Kazakhstan	; Abdaliyev Askar; Akhmetollayev Ilyas; Amirgazin Asylulan; Aushakhmetova Zabira; Kalendar Ruslan; Lutsay Viktoriya; Rakhmetova Akbota; Ramankulov Yerlan; Shevtsov Alexandr
EPI_ISL_454596 to 454597, EPI_ISL_454599 to 454600, EPI_ISL_454604	Laboratory of virology, National Center of Expertise	Laboratory of molecular-genetic research, National Center for Expertise, Kazakhstan National Center for Biotechnology, Kazakhstan	; Abdaliyev Askar; Akhmetollayev Ilyas; Amirgazin Asylulan; Aushakhmetova Zabira; Kalendar Ruslan; Lutsay Viktoriya; Rakhmetova Akbota; Ramankulov Yerlan; Shevtsov Alexandr
EPI_ISL_454576 to 454577, EPI_ISL_454579 to 454580, EPI_ISL_454582, EPI_ISL_454587, EPI_ISL_454590 to 454591, EPI_ISL_454593 to 454594, EPI_ISL_454598, EPI_ISL_454601, EPI_ISL_454603			
see above	Laboratory of virology, National Center of Expertise	Laboratory of molecular-genetic research, National Center of Expertise, Kazakhstan National Center for Biotechnology, Kazakhstan	; Abdaliyev Askar; Akhmetollayev Ilyas; Amirgazin Asylulan; Aushakhmetova Zabira; Kalendar Ruslan; Lutsay Viktoriya; Rakhmetova Akbota; Ramankulov Yerlan; Shevtsov Alexandr
EPI_ISL_1448017 to 1448018, EPI_ISL_1448020	Laboratory of virology, National center of expertise	RSE "National Center for Biotechnology" and RSE "National Center of Expertise"	Abdaliyev Askar; Amirgazin Asylulan; Balykbaev Kanat; Kamalova Dinara; Ramankulov Yerlan; Sharipova Saule; Shevtsov Alexandr; Tungushbayev Talgat
EPI_ISL_576119	Laboratory, The Bio Arte Limited	Laboratory, The Bio Arte Limited	Biazzo, M.; Madeddu, S.; Pinzauti, D.; Santoro, F.
EPI_ISL_576117 to 576118, EPI_ISL_576120 to 576126	Laboratory, The Bio Arte Limited	Laboratory, The Bio Arte Limited	Biazzo, M.; Madeddu, S.; Pinzauti, D.; Santoro, F.
EPI_ISL_414045, EPI_ISL_456076 to 456077, EPI_ISL_456082 to 456083, EPI_ISL_456088	Laboratório Central de Saúde Pública Noel Nutels (LACEN-RJ)	Laboratory of Respiratory Viruses and Measles, Oswaldo Cruz Institute, FIOCRUZ	Aline Mattos; Alisson Fabri; Braulia Caetano; Cristiana Garcia; Fernando Motta; Joilson Xavier; Jonathan Lopes; Luciana Appolinario; Maria Nóbrega; Maria Ogrzewalska; Marilda Siqueira on behalf of the Fiocruz COVID-19 Genomic Surveillance Network; Marilda SiqueiraFiocruz COVID-19 Genomic Surveillance Network; Milene Miranda; Paola Resende; Sunando Roy
EPI_ISL_415105, EPI_ISL_427293	Laboratório Central de Saúde Pública Professor Gonçalo Moniz (LACEN-BA)	Laboratory of Respiratory Viruses and Measles, Oswaldo Cruz Institute, FIOCRUZ	Aline Mattos; Allison Fabri; Braulia Caetano; Cristiana Garcia; Fernando Motta; Joilson Xavier; Jonathan Lopes; Luciana Appolinario; Maria Nóbrega; Maria Ogrzewalska; Marilda Siqueira Fiocruz COVID-19 Genomic Surveillance Network; Marilda Siqueira on behalf of the Fiocruz COVID-19 Genomic Surveillance Network; Milene Miranda; Paola Resende; Priscila Born; Sunando Roy
EPI_ISL_427292	Laboratório Central de Saúde Pública do Estado de Alagoas (LACEN-AL)	Laboratory of Respiratory Viruses and Measles, Oswaldo Cruz Institute, FIOCRUZ	Aline Mattos; Braulia Caetano; Cristiana Garcia; Fernando Motta; Jonathan Lopes; Luciana Appolinario; Maria Ogrzewalska; Marilda Siqueira on behalf of the Fiocruz COVID-19 Genomic Surveillance Network; Milene Miranda; Paola Resende; Priscila Born; Sunando Roy
EPI_ISL_500460 to 500486, EPI_ISL_500865 to 500875, EPI_ISL_572334 to 572396	Laboratório Central de Saúde Pública do Estado de Pernambuco (LACEN-PE)	WallauLab, Aggeu Magalhaes Institute	Alexandre Freitas da Silva; Antonio Mauro Rezende; Armando de Menezes Neto; Bruna Santos Lima Figueiredo de Sá; Caroline Targino Alves da Silva; Claudio Eduardo Cavalcanti; Constância Flávia Junqueira Ayres; Cássia Docena; Derciliano Lopes da Cruz; Duschinka Ribeiro Duarte Guedes; Elisama Helvecio; Filipe Zimmer Dezordi; Gabriel Luz Wallau on behalf of the Fiocruz COVID-19 Genomic Surveillance Network; Gonzalo Bello; Kamila Gaudêncio da Silva Sales; Larissa Krokovsky; Laís Ceschini Machado; Luciane Caroline Albuquerque Bezerra; Luydson Richardson Silva Vasconcelos; Marcelo Henrique Santos Paiva; Maria Almerice Lopes da Silva; Matheus Filgueira Bezerra; Michelle da Silva Barros; Paola Cristina Resende; Renata Pessôa Germano Mendes; Rodrigo Moraes Loyo Arcoverde; Severino Jefferson Ribeiro da Silva; Sinval Pinto Brandão Filho; Tiago Gräf; Wheverton Ricardo Correia do Nascimento
EPI_ISL_427305 to 427306, EPI_ISL_541370 to 541371	Laboratório Central de Saúde Pública do Estado de Santa Catarina (LACEN-SC)	Laboratory of Respiratory Viruses and Measles, Oswaldo Cruz Institute, FIOCRUZ	Aline Mattos; Ana Carolina Mendonça; Anna Carolina Paixão; Braulia Caetano; Cristiana Garcia; Fernando Motta; Jonathan Lopes; Luciana Appolinario; Maria Ogrzewalska; Marilda Siqueira on behalf of the Fiocruz COVID-19 Genomic Surveillance Network; Milene Miranda; Paola Resende; Priscila Born; Sandra Bianchini; Sunando Roy
EPI_ISL_541372 to 541396	Laboratório Central de Saúde Pública do Estado de Sergipe (LACEN-SE)	Laboratory of Respiratory Viruses and Measles, Oswaldo Cruz Institute, FIOCRUZ	Ana Carolina Mendonça; Anna Carolina Paixão; Clioma Santos; Fernando Motta; Jonathan Lopes; Luciana Appolinario; Marilda Siqueira on behalf of the Fiocruz COVID-19 Genomic Surveillance Network; Paola Resende
EPI_ISL_415128	Laboratório Central de Saúde Pública do Estado do Espírito Santo (LACEN-ES)	Laboratory of Respiratory Viruses and Measles, Oswaldo Cruz Institute, FIOCRUZ	Aline Mattos; Allison Fabri; Braulia Caetano; Cristiana Garcia; Fernando Motta; Joilson Xavier; Jonathan Lopes; Luciana Appolinario; Maria Nóbrega; Maria Ogrzewalska; Marilda Siqueira on behalf of the Fiocruz COVID-19 Genomic Surveillance Network; Milene Miranda; Paola Resende; Sunando Roy
EPI_ISL_541340 to 541346	Laboratório Central de Saúde Pública do Estado do Paraná (LACEN-PR)	Laboratory of Respiratory Viruses and Measles, Oswaldo Cruz Institute, FIOCRUZ	Ana Carolina Mendonça; Anna Carolina Paixão; Fernando Motta; Irina Riediger; Jonathan Lopes; Luciana Appolinario; Maria do Carmo Debur; Marilda Siqueira on behalf of the Fiocruz COVID-19 Genomic Surveillance Network; Paola Resende
EPI_ISL_417943	Laboratório Hermes Pardini	Bioinformatics Laboratory	Alexandra Gerber; Amilcar Tanuri; Ana Paula Guimarães; Cristiano Xavier Lima; Cássia Alves Gonçalves; Diana Mariani; Ester Cerdeira Sabino; Filipe Romero; Ingra Morales Claro; Isabela de Carvalho Leitão; Jaqueline Goes de Jesus; Laboratorio Hermes Pardini; Laboratorio Simile; Luciana Jesus Costa e André Felipe Santos; Luiz Gonzaga Paula de Almeida; Lídia Boullosa; Mariane Talon; Mauro Teixeira; Nuno Rodrigues Faria; Orlando Ferreira Jr.; Pinto Castiñeiras; Rafael de Mello Galliez; Renato Santana Aguiar e Ana Tereza Vasconcelos; Richard Araújo Maia; Rodrigo Brindeiro; Ronaldo da Silva Francisco Junior; Terezinha Marta Pereira; Érica Ramos dos Santos Nascimento
EPI_ISL_417928 to 417930, EPI_ISL_417934, EPI_ISL_417936, EPI_ISL_417940, EPI_ISL_417945, EPI_ISL_417949	Laboratório Hermes Pardini	Bioinformatics Laboratory - LNCC	Alexandra Gerber; Amilcar Tanuri; Ana Paula Guimarães; Cristiano Xavier Lima; Cássia Alves Gonçalves; Cássia Cristina Alves Gonçalves; Diana Mariani; Ester Cerdeira Sabino; Filipe Romero; Ingra Morales Claro; Isabela de Carvalho Leitão; Jaqueline Goes de Jesus; Laboratorio Hermes Pardini; Laboratorio Simile; Luciana Jesus Costa e André Felipe Santos; Luiz Gonzaga Paula de Almeida; Lídia Boullosa; Mariane Talon; Mauro Teixeira; Nuno Rodrigues Faria; Orlando Ferreira Jr.; Pinto Castiñeiras; Rafael de Mello Galliez; Renato Santana Aguiar e Ana Tereza Vasconcelos; Richard Araújo Maia; Rodrigo Brindeiro; Ronaldo da Silva Francisco Junior; Terezinha Marta Pereira; Érica Ramos dos Santos Nascimento
EPI_ISL_417925 to 417926	Laboratório Simili	Bioinformatics Laboratory / LNCC	Alexandra Gerber; Amilcar Tanuri; Ana Paula Guimarães; Cristiano Xavier Lima; Cássia Cristina Alves Gonçalves; Diana Mariani; Ester Cerdeira Sabino; Filipe Romero; Francisco Junior; Ingra Morales Claro; Isabela de Carvalho Leitão; Jaqueline Goes de Jesus; Laboratorio Hermes Pardini; Laboratorio Simile; Luciana Jesus Costa e André Felipe Santos; Luiz Gonzaga Paula de Almeida; Lídia Boullosa; Mariane Talon; Mauro Teixeira; Nuno Rodrigues Faria; Orlando Ferreira Jr.; Pinto Castiñeiras; Rafael de Mello Galliez; Renato Santana Aguiar e Ana Tereza Vasconcelos; Richard Araújo Maia; Rodrigo Brindeiro; Ronaldo da Silva; Ronaldo da Silva Francisco Junior; Terezinha Marta Pereira; Érica Ramos dos Santos Nascimento
EPI_ISL_1494960 to 1495042, EPI_ISL_1497548 to 1497549	Laboratório de Biologia Integrativa	Laboratório de Biologia Integrativa	Alessandro Clayton de Souza Ferreira; Aline Brito de Lima; Carolina Moreira Voloch; Daniel Costa Queiroz; Danielle Alves Gomes Zauli; Diego Menezes Bonfim; Filipe Romero Rebello Moreira; Frederico Scott Varella Malta; Joice do Prado Silva; Lucyene Miguita Luiz; Nuno Rodrigues Faria; Paula Luize Camargos Fonseca; Rafael Marques de Souza; Renan Pedra de Souza; Renato Santana Aguiar; Rennan Garcias Moreira; Victor Cavalcanti Pardini; Victor Emmanuel Viana Geddes
EPI_ISL_476152 to 476170, EPI_ISL_476337 to 476349, EPI_ISL_476387 to 476402, EPI_ISL_476408 to 476425	Laboratório de Patologia Clínica - UNICAMP	Laboratório de Estudos de Vírus Emergentes - UNICAMP	Angelica Schreiber; Camila Simeoni; Darlan da Silva Candido; Jaqueline Goes Jesus e William Marciel de Souza; José Luiz Proença-Modena; Julia Forato; Julien Theze; Luiz Gonzaga; Magnun Nueldo Nunes dos Santos; Marcilio Jorge Fumagalli; Mariene Ribeiro Amorim; Nuno Rodrigues Faria
EPI_ISL_1464626 to 1464677	Laboratório de Virologia - UNIFESP	Laboratory of Respiratory Viruses and Measles, Oswaldo Cruz Institute, FIOCRUZ	Alice Sampaio Rocha; Ana Carolina Mendonca; Anna Carolina Paixao; Fernando Motta; Luciana Appolinario; Marilda Siqueira on behalf of the Fiocruz COVID-19 Genomic Surveillance Network; Nancy Belei; Paola Resende; Renata Serrano Lopes
EPI_ISL_541397 to 541399	Laboratório de Virologia Comparada e Ambiental - LVCA - IOC	Laboratory of Respiratory Viruses and Measles, Oswaldo Cruz Institute, FIOCRUZ	Ana Carolina Mendonça; Camille Ferreira Mannarino; Fernando Motta; Luciana Appolinario; Marilda Siqueira; Marize Pereira Miagostovich on behalf of the Fiocruz COVID-19 Genomic Surveillance Network; Paola Resende; Tatiana Prado; Tulio Machado Fumian
EPI_ISL_1433518, EPI_ISL_1433526, EPI_ISL_1433563, EPI_ISL_1433567, EPI_ISL_1433569, EPI_ISL_1433576, EPI_ISL_1433594, EPI_ISL_1433736 to 1433737, EPI_ISL_1439380, EPI_ISL_1439398, EPI_ISL_1439416, EPI_ISL_1439432			
see above	Labormedizin Darmstadt	Robert Koch Institute	
EPI_ISL_1394263 to 1394269, EPI_ISL_1538481, EPI_ISL_1538502, EPI_ISL_1538610, EPI_ISL_1538957 to 1538959	Labotarorium Diagnostyki Molekularnej,Cytogenetyki i Cytometrii	1. National Institute of Public Health - National Institute of Hygiene; 2. Eurofins Genomics Europe Sequencing GmbH	ECDC COVID-19 WGS support team; Eurofins Genomics Europe Sequencing Team; Gierczyki Rafa; Sadkowska-Todys Magorzata; Wokowicz Tomasz; Zacharczuk Katarzyna
EPI_ISL_1534246	Ladismith (Nissenville) Clinic wc LAF	NHLS/UCT	Arash Iranzadeh; Bruna Galvao; Carolyn Williamson; Deelan Doolabh; Diana Hardie; Innocent Mudau; Kruger Marais; Lynn Tyers; Marvin Hsiao; Stephen Korsman

EPI_ISL_1416726	Lafene Health Center	Kansas State University Veterinary Diagnostic Laboratory	Andrea Lu; Emily Cox; Jamie Henningson; Jianfa Bai; Kasey Hogan; Lance Noll; Rachel Palinski; Tyler Doerksen
EPI_ISL_1423977 to 1423983, EPI_ISL_1499106 to 1499111, EPI_ISL_1499595 to 1499620, EPI_ISL_1500666 to 1500667, EPI_ISL_1515790 to 1515797	Lafene Health Center	Kansas State Veterinary Diagnostic Laboratory	Andreea Lu; Elizabeth Porter; Emily Cox; Jamie Henningson; Jianfa Bai; Kasey Hogan; Lance Noll; Rachel Palinski; Tyler Doerksen
EPI_ISL_434669, EPI_ISL_475515	Lakargruppen	The Public Health Agency of Sweden	Anna Risberg; Anna-Malin Linde; Boris Klanger; Karin Tegmark-Wisell; Maria Lind Karlberg; Mattias Haukland; Mia Brytting; Olov Svartstrom; Oskar Karlsson Lindsjo; Reza Advani; Sandra Broddesson; Theresa Enkirch
EPI_ISL_1441242, EPI_ISL_1441244 to 1441245, EPI_ISL_1441250 to 1441251, EPI_ISL_1441255, EPI_ISL_1441257 to 1441258, EPI_ISL_1441260 to 1441261, EPI_ISL_1441263, EPI_ISL_1441265, EPI_ISL_1441268, EPI_ISL_1441270 to 1441275, EPI_ISL_1441278, EPI_ISL_1441281, EPI_ISL_1441284, EPI_ISL_1441287, EPI_ISL_1441292, EPI_ISL_1441294 to 1441295, EPI_ISL_1441298, EPI_ISL_1441300, EPI_ISL_1441306, EPI_ISL_1441308			
see above	Landesuntersuchungsanstalt für das Gesundheits- und Veterinärwesen (LUA) Sachsen	Robert Koch Institute	
EPI_ISL_1531531 to 1531532	Landstuhl Regional Medical Center	US Air Force School of Aerospace Medicine	Amanda Javorina; Anthony Fries; Clarise Starr; Cole Anderson; Elizabeth Macias; Fritz Castillo; Jennifer Meyer; Sarah Purves; William Buggele; William Gruner
EPI_ISL_534237 to 534238, EPI_ISL_548255	Lanssjukhuset Kalmar	The Public Health Agency of Sweden	Anna Risberg; Anna-Malin Linde; Karin Tegmark-Wisell; Maria Lind Karlberg; Mattias Haukland; Mia Brytting; Olov Svartstrom; Oskar Karlsson Lindsjo; Petra Edquist; Reza Advani; Sandra Broddesson
EPI_ISL_407079	Lapland Central Hospital	Department of Virology, University of Helsinki and Helsinki University Hospital, Helsinki, Finland	Hannimari Kallio-Kokko; Olli Vapalahti; Suvi Kuivanen; Teemu Smura
EPI_ISL_437089 to 437096, EPI_ISL_486422 to 486426, EPI_ISL_486428, EPI_ISL_486430 to 486436, EPI_ISL_512647 to 512652, EPI_ISL_515185 to 515186, EPI_ISL_534204 to 534205, EPI_ISL_534209			
see above	Latvijas Infektoloijas centrs	Latvian Biomedical Research and Study Centre	Ivars Silamielis; Jeena Storoženko; Jnis Kloviš; Jnis Pjalkovskis; Kaspars Megnis; Monta Ustinova; Oksana Savicka; Tatjana Kolupajeva; Uga Dumpis; Vita Rovte; ikita Zrelovs
EPI_ISL_451489, EPI_ISL_451545, EPI_ISL_451572, EPI_ISL_451602, EPI_ISL_451609, EPI_ISL_451611, EPI_ISL_451631, EPI_ISL_451641, EPI_ISL_455041, EPI_ISL_455048, EPI_ISL_455063, EPI_ISL_498769 to 498772, EPI_ISL_513403 to 513408, EPI_ISL_526143, EPI_ISL_526202 to 526204, EPI_ISL_544957, EPI_ISL_544962, EPI_ISL_545040, EPI_ISL_591487 to 591491, EPI_ISL_593653			
see above	Laverty Pathology	NSW Health Pathology - Institute of Clinical Pathology and Medical Research; Westmead Hospital; University of Sydney	CIDM-PH et al.
EPI_ISL_421507 to 421508	Le Château de Seine-Port	National Reference Center for Viruses of Respiratory Infections, Institut Pasteur, Paris	Angela Brisebarre; Etienne Simon-Lorière; Flora Donati; Marion Barbet; Maud Vanpeene; Mélanie Albert; Méline Bizard; Sylvie Behillil; Sylvie van der Werf; Vincent Enouf
EPI_ISL_498551 to 498552, EPI_ISL_498554, EPI_ISL_498556	Lebanese American University	Lebanese American University	Abdallah, J.; Abi Habib, W.; El Shesheny, R.; Goldstein; J. and Kayali, G.; Mokhbat, J.; R.J.; Webby
EPI_ISL_447588	Lednicky Lab	Lednicky lab	Alam; C.J.; Elbadry; Gibson; J.A.; J.C.; J.G. Jr. and Lednicky; M.A.; M.M.; Morris; Stephenson; Subramaniam, K.; T.B.; Waltzek
EPI_ISL_434677	Lednicky Laboratory at Emerging Pathogens Institute	Lednicky Laboratory at Emerging Pathogens Institute	C.-Y.; Clugston; Elbadry; J.A.; J.G. Jr. and Lednicky; J.R.; M.A.; Morris; S.N.; Shankar; Wu
EPI_ISL_447900	Lednicky Laboratory at Emerging Pathogens Institute	University of Florida	Alam; C.J.; Elbadry; Gibson; J.A.; J.C.; J.G.; Lednicky; M.A. and Morris; M.M.; Stephenson
EPI_ISL_434636	Lednicky Laboratory, Emerging Pathogens Institute, University of Florida	Lednicky Laboratory at Emerging Pathogens Institute, University of Florida	Alam, M.; C.J.; Elbadry; Gibson; J.A.; J.C.; J.G. Jr. and Lednicky; M.A.; Morris; Stephenson; Subramaniam, K.; T.B.; Waltzek
EPI_ISL_434637	Lednicky Laboratory, Emerging Pathogens Institute, University of Florida.	Lednicky Laboratory, Emerging Pathogens Institute, University of Florida.	C.J.; Elbadry; Gibson; J.A.; J.C.; J.G. Jr. and Lednicky; M.A.; Morris; Stephenson; Subramaniam, K.; T.B.; Waltzek
EPI_ISL_569664 to 569688	Lee Lab	Lee Lab	Gina Faraci; Jane F. Emerson; Pamela M. Ward; Sung Yong Park; and Ha Youn Lee
EPI_ISL_538811, EPI_ISL_538818, EPI_ISL_538841, EPI_ISL_538851, EPI_ISL_538863, EPI_ISL_538866, EPI_ISL_538899, EPI_ISL_538917, EPI_ISL_538945, EPI_ISL_538987, EPI_ISL_538994, EPI_ISL_539006, EPI_ISL_539030, EPI_ISL_539070, EPI_ISL_539116 to 539117, EPI_ISL_539120, EPI_ISL_539143, EPI_ISL_539150, EPI_ISL_539152, EPI_ISL_539157, EPI_ISL_539185, EPI_ISL_539197, EPI_ISL_539213			
see above	Leeds Teaching Hospitals NHS Trust and Public Health England, National Infection Service (Leeds laboratory)	Wellcome Sanger Institute for the COVID-19 Genomics UK (COG-UK) Consortium	Antony Hale and Alex Alderton; Cordelia Langford; David K. Jackson; Dominic Kwiatkowski; Ewan Harrison; Holli Carden; Ian Johnston; John Sillitoe on behalf of the Wellcome Sanger Institute COVID-19 Surveillance Team; Katherine L. Harper; Louissa Macfarlane-Smith; Roberto Amato; Sonia Goncalves
EPI_ISL_538748 to 538810, EPI_ISL_538812 to 538817, EPI_ISL_538819 to 538840, EPI_ISL_538842 to 538850, EPI_ISL_538852 to 538862, EPI_ISL_538864 to 538865, EPI_ISL_538867 to 538898, EPI_ISL_538900 to 538916, EPI_ISL_538918 to 538944, EPI_ISL_538946 to 538986, EPI_ISL_538988 to 538993, EPI_ISL_538995 to 539005, EPI_ISL_539007 to 539029, EPI_ISL_539031 to 539069, EPI_ISL_539071 to 539115, EPI_ISL_539118 to 539119, EPI_ISL_539121 to 539142, EPI_ISL_539144 to 539149, EPI_ISL_539151, EPI_ISL_539153 to 539156, EPI_ISL_539158 to 539184, EPI_ISL_539186 to 539196, EPI_ISL_539198 to 539212, EPI_ISL_539214 to 539220			
see above	Leeds Teaching Hospitals NHS Trust and Public Health England, National Infection Service (Leeds laboratory)	Wellcome Sanger Institute for the COVID-19 Genomics UK (COG-UK) consortium	Antony Hale and Alex Alderton; Cordelia Langford; David K. Jackson; Dominic Kwiatkowski; Ewan Harrison; Holli Carden; Ian Johnston; John Sillitoe on behalf of the Wellcome Sanger Institute COVID-19 Surveillance Team; Katherine L. Harper; Louissa Macfarlane-Smith; Roberto Amato; Sonia Goncalves
EPI_ISL_1534272, EPI_ISL_1534370, EPI_ISL_1534441	Lentegeur Hospital wc LGH	NHLS/UCT	Arash Iranzadeh; Bruna Galvao; Carolyn Williamson; Deelan Doolabh; Diana Hardie; Emmanuel SJ; Innocent Mudau; Kruger Marais; Lynn Tyers; Marvin Hsiao; Stephen Korsman; Tegally H; de Oliveira T
EPI_ISL_1436850 to 1436851, EPI_ISL_1436853, EPI_ISL_1436855, EPI_ISL_1436857, EPI_ISL_1436859, EPI_ISL_1436900, EPI_ISL_1436902			
see above	Libramont	Plateforme de testing Namuroise	; Degosserie Jonathan; Denis Olivier; Mullier François; Otto Gaetan
EPI_ISL_1384702 to 1384720, EPI_ISL_1396400, EPI_ISL_1398431 to 1398455, EPI_ISL_1510082, EPI_ISL_1510093, EPI_ISL_1510095 to 1510098, EPI_ISL_1510121 to 1510159, EPI_ISL_1510196, EPI_ISL_1510204, EPI_ISL_1510207, EPI_ISL_1510210 to 1510218			
see above	Life Sciences Center, Vilnius University	Vilnius University Hospital Santaros Klinikos, Center of Laboratory Medicine	Daniel Naumovas; Dovile Ezerskyte; Gytis Dudas; Ingrida Olendraite; Laimonas Griskevicius; Ligita Raugaite; Mindaugas Stoskus; Monika Katenaite; Rimvydas Norvilas
EPI_ISL_511644	Life and Health Sciences Research Institute, University of Minho, Braga	Instituto Nacional de Saude (INSA)	Borges et al
EPI_ISL_551294, EPI_ISL_551327, EPI_ISL_551412, EPI_ISL_551453, EPI_ISL_551484, EPI_ISL_551496, EPI_ISL_551530, EPI_ISL_552492, EPI_ISL_552818, EPI_ISL_552841, EPI_ISL_552972 to 552973, EPI_ISL_553421, EPI_ISL_553568, EPI_ISL_553778, EPI_ISL_553784, EPI_ISL_553812, EPI_ISL_553846, EPI_ISL_553866, EPI_ISL_553946, EPI_ISL_554478, EPI_ISL_554484, EPI_ISL_554497, EPI_ISL_554537, EPI_ISL_554601, EPI_ISL_554667, EPI_ISL_554709, EPI_ISL_554763, EPI_ISL_554845, EPI_ISL_554868, EPI_ISL_554929, EPI_ISL_555019, EPI_ISL_555041, EPI_ISL_555127, EPI_ISL_555129, EPI_ISL_555148, EPI_ISL_555259, EPI_ISL_555303 to 555304, EPI_ISL_555391, EPI_ISL_555398, EPI_ISL_555432 to 555433, EPI_ISL_555502, EPI_ISL_555510 to 555511, EPI_ISL_555650 to 555651, EPI_ISL_555660, EPI_ISL_555794, EPI_ISL_555807, EPI_ISL_555815, EPI_ISL_555887, EPI_ISL_555947, EPI_ISL_555986, EPI_ISL_555990, EPI_ISL_556000, EPI_ISL_556141, EPI_ISL_556348, EPI_ISL_556350, EPI_ISL_556411, EPI_ISL_556428, EPI_ISL_556433, EPI_ISL_556435, EPI_ISL_556505, EPI_ISL_556510, EPI_ISL_557002, EPI_ISL_557043, EPI_ISL_557110, EPI_ISL_557142, EPI_ISL_557625, EPI_ISL_557711, EPI_ISL_557728, EPI_ISL_557730, EPI_ISL_557739, EPI_ISL_557760, EPI_ISL_557852, EPI_ISL_557896, EPI_ISL_557999, EPI_ISL_558024, EPI_ISL_558126, EPI_ISL_558185, EPI_ISL_558222, EPI_ISL_558311, EPI_ISL_558362, EPI_ISL_558387, EPI_ISL_558422, EPI_ISL_558432, EPI_ISL_558543, EPI_ISL_558605, EPI_ISL_558617, EPI_ISL_558796, EPI_ISL_558846, EPI_ISL_558872, EPI_ISL_558877, EPI_ISL_558888, EPI_ISL_558950, EPI_ISL_558994, EPI_ISL_559019, EPI_ISL_559042, EPI_ISL_559046, EPI_ISL_559120, EPI_ISL_559129, EPI_ISL_559213, EPI_ISL_559228, EPI_ISL_559261, EPI_ISL_559275, EPI_ISL_559337, EPI_ISL_559353, EPI_ISL_559447, EPI_ISL_559576, EPI_ISL_559591, EPI_ISL_559616, EPI_ISL_559644, EPI_ISL_567803, EPI_ISL_567811, EPI_ISL_575536, EPI_ISL_579951, EPI_ISL_579971, EPI_ISL_580062, EPI_ISL_580138, EPI_ISL_580187, EPI_ISL_580210, EPI_ISL_580285, EPI_ISL_580563, EPI_ISL_580686, EPI_ISL_580708, EPI_ISL_580780, EPI_ISL_580824, EPI_ISL_581423, EPI_ISL_586612, EPI_ISL_586617, EPI_ISL_586621, EPI_ISL_586709, EPI_ISL_586907, EPI_ISL_587085, EPI_ISL_587265, EPI_ISL_587977, EPI_ISL_588007, EPI_ISL_588026, EPI_ISL_589289, EPI_ISL_589302, EPI_ISL_589329, EPI_ISL_589342, EPI_ISL_589344, EPI_ISL_589376, EPI_ISL_597633, EPI_ISL_597984, EPI_ISL_598163, EPI_ISL_606948, EPI_ISL_606960, EPI_ISL_607189, EPI_ISL_607200, EPI_ISL_607894, EPI_ISL_608147, EPI_ISL_608216, EPI_ISL_608310, EPI_ISL_609209, EPI_ISL_610332, EPI_ISL_610502 to 610503, EPI_ISL_611093, EPI_ISL_1408908, EPI_ISL_1408911, EPI_ISL_1408913, EPI_ISL_1408932, EPI_ISL_1408941, EPI_ISL_1408948, EPI_ISL_1408953, EPI_ISL_1408958 to 1408959, EPI_ISL_1408961, EPI_ISL_1408966, EPI_ISL_1408973, EPI_ISL_1408988, EPI_ISL_1408990, EPI_ISL_1409004, EPI_ISL_1409006, EPI_ISL_1409011, EPI_ISL_1409016, EPI_ISL_1409031, EPI_ISL_1409075, EPI_ISL_1409082, EPI_ISL_1409092, EPI_ISL_1409104, EPI_ISL_1409114, EPI_ISL_1409124, EPI_ISL_1409146, EPI_ISL_1409151, EPI_ISL_1409164, EPI_ISL_1409180, EPI_ISL_1409187, EPI_ISL_1409189, EPI_ISL_1409200, EPI_ISL_1409208, EPI_ISL_1409211, EPI_ISL_1409216, EPI_ISL_1409218, EPI_ISL_1409223, EPI_ISL_1409228 to 1409229, EPI_ISL_1409232, EPI_ISL_1409236, EPI_ISL_1409238 to 1409239, EPI_ISL_1409241, EPI_ISL_1409243 to 1409244, EPI_ISL_1409257, EPI_ISL_1409273, EPI_ISL_1409276, EPI_ISL_1409281, EPI_ISL_1409284 to 1409286, EPI_ISL_1409288, EPI_ISL_1409293, EPI_ISL_1409299 to 1409300, EPI_ISL_1409306, EPI_ISL_1409308, EPI_ISL_1409312, EPI_ISL_1409319 to 1409320, EPI_ISL_1409323, EPI_ISL_1409328 to 1409329, EPI_ISL_1409334, EPI_ISL_1409339, EPI_ISL_1409346, EPI_ISL_1409348, EPI_ISL_1409351, EPI_ISL_1409353, EPI_ISL_1409355, EPI_ISL_1409363 to 1409365, EPI_ISL_1409374, EPI_ISL_1409376, EPI_ISL_1409381 to 1409382, EPI_ISL_1409385, EPI_ISL_1409387, EPI_ISL_1409397 to 1409398, EPI_ISL_1409400, EPI_ISL_1409402, EPI_ISL_1409405, EPI_ISL_1409408, EPI_ISL_1409412, EPI_ISL_1409415, EPI_ISL_1409418 to 1409419, EPI_ISL_1409424, EPI_ISL_1409426, EPI_ISL_1409431, EPI_ISL_1409434 to 1409436, EPI_ISL_1409438, EPI_ISL_1409440, EPI_ISL_1409445, EPI_ISL_1409451, EPI_ISL_1409455, EPI_ISL_1409459, EPI_ISL_1409463 to 1409464, EPI_ISL_1409466 to 1409467, EPI_ISL_1409473, EPI_ISL_1409476, EPI_ISL_1409480, EPI_ISL_1409486 to 1409490, EPI_ISL_1409503 to 1409505, EPI_ISL_1409507, EPI_ISL_1409511 to 1409513, EPI_ISL_1409515, EPI_ISL_1409521, EPI_ISL_1409528, EPI_ISL_1409534, EPI_ISL_1409538, EPI_ISL_1409542, EPI_ISL_1409548, EPI_ISL_1409554 to 1409556, EPI_ISL_1409565, EPI_ISL_1409570, EPI_ISL_1409573, EPI_ISL_1409575, EPI_ISL_1409578, EPI_ISL_1409581, EPI_ISL_1409588, EPI_ISL_1409595, EPI_ISL_1409603, EPI_ISL_1409613 to 1409614, EPI_ISL_1409621, EPI_ISL_1409626 to 1409628, EPI_ISL_1409631, EPI_ISL_1409635 to 1409636, EPI_ISL_1409644 to 1409645, EPI_ISL_1409648, EPI_ISL_1409650, EPI_ISL_1409653, EPI_ISL_1409658, EPI_ISL_1409667, EPI_ISL_1409676, EPI_ISL_1409678, EPI_ISL_1409681, EPI_ISL_1409688, EPI_ISL_1409695, EPI_ISL_1409703 to 1409704, EPI_ISL_1409706, EPI_ISL_1409714, EPI_ISL_1409719 to 1409720, EPI_ISL_1409725 to 1409726, EPI_ISL_1409729, EPI_ISL_1409740, EPI_ISL_1409750, EPI_ISL_1409755 to 1409756, EPI_ISL_1409759, EPI_ISL_1409762, EPI_ISL_1409764 to 1409765, EPI_ISL_1409768 to 1409770, EPI_ISL_1409775 to 1409777, EPI_ISL_1409779 to 1409782, EPI_ISL_1409784 to 1409793, EPI_ISL_1409795 to 1409808, EPI_ISL_1409810 to 1409812,			

EPI_ISL_1409814 to 1409816, EPI_ISL_1409818 to 1409835, EPI_ISL_1409837 to 1409842, EPI_ISL_1409844 to 1409857, EPI_ISL_1409860, EPI_ISL_1409862, EPI_ISL_1409864 to 1409867, EPI_ISL_1409869, EPI_ISL_1409871 to 1409872, EPI_ISL_1409874, EPI_ISL_1409876 to 1409882, EPI_ISL_1409884 to 1409885, EPI_ISL_1409887 to 1409888, EPI_ISL_1409890, EPI_ISL_1409892 to 1409893, EPI_ISL_1409895 to 1409905, EPI_ISL_1409908 to 1409911, EPI_ISL_1409914 to 1409917, EPI_ISL_1409919 to 1409925, EPI_ISL_1409927 to 1409931, EPI_ISL_1409933 to 1409934, EPI_ISL_1409936 to 1409937, EPI_ISL_1409939 to 1409950, EPI_ISL_1409952 to 1409959, EPI_ISL_1409962 to 1409966, EPI_ISL_1409968 to 1409969, EPI_ISL_1409971 to 1409975, EPI_ISL_1409977 to 1409980, EPI_ISL_1409982 to 1409986, EPI_ISL_1409988 to 1409992, EPI_ISL_1409994 to 1409995, EPI_ISL_1409997 to 1410000, EPI_ISL_1410003 to 1410005, EPI_ISL_1410007 to 1410008, EPI_ISL_1410010 to 1410012, EPI_ISL_1410014 to 1410031, EPI_ISL_1410033 to 1410036, EPI_ISL_1410038 to 1410045, EPI_ISL_1410049 to 1410050, EPI_ISL_1410052, EPI_ISL_1410054 to 1410055, EPI_ISL_1410057 to 1410066, EPI_ISL_1410068 to 1410070, EPI_ISL_1410072, EPI_ISL_1410074 to 1410075, EPI_ISL_1410077 to 1410078, EPI_ISL_1410288, EPI_ISL_1410294, EPI_ISL_1410296, EPI_ISL_1410301 to 1410303, EPI_ISL_1410305, EPI_ISL_1410307 to 1410313, EPI_ISL_1410317, EPI_ISL_1410319, EPI_ISL_1410321, EPI_ISL_1410329, EPI_ISL_1410331 to 1410332, EPI_ISL_1410335 to 1410337, EPI_ISL_1410339 to 1410340, EPI_ISL_1410343 to 1410344, EPI_ISL_1410346, EPI_ISL_1410348, EPI_ISL_1410350 to 1410354, EPI_ISL_1410359, EPI_ISL_1410362 to 1410363, EPI_ISL_1410365, EPI_ISL_1410367, EPI_ISL_1410369 to 1410372, EPI_ISL_1410376 to 1410382, EPI_ISL_1410386, EPI_ISL_1410388 to 1410389, EPI_ISL_1410392 to 1410393, EPI_ISL_1410398 to 1410399, EPI_ISL_1410401 to 1410402, EPI_ISL_1410404 to 1410405, EPI_ISL_1410407 to 1410408, EPI_ISL_1410411, EPI_ISL_1410414, EPI_ISL_1410416, EPI_ISL_1410418 to 1410420, EPI_ISL_1410423, EPI_ISL_1410429 to 1410431, EPI_ISL_1410436, EPI_ISL_1410438 to 1410440, EPI_ISL_1410442 to 1410447, EPI_ISL_1410449, EPI_ISL_1410452, EPI_ISL_1410454 to 1410458, EPI_ISL_1410463, EPI_ISL_1410466, EPI_ISL_1410468, EPI_ISL_1410470, EPI_ISL_1410472, EPI_ISL_1410474, EPI_ISL_1410478 to 1410479, EPI_ISL_1410481, EPI_ISL_1410497 to 1410498, EPI_ISL_1410500, EPI_ISL_1410503 to 1410506, EPI_ISL_1410510, EPI_ISL_1410512 to 1410513, EPI_ISL_1410520, EPI_ISL_1410523 to 1410524, EPI_ISL_1410527, EPI_ISL_1410530 to 1410533, EPI_ISL_1410541 to 1410542, EPI_ISL_1410549 to 1410550, EPI_ISL_1410554, EPI_ISL_1410557, EPI_ISL_1410559 to 1410560, EPI_ISL_1410563, EPI_ISL_1410565, EPI_ISL_1410570, EPI_ISL_1410573, EPI_ISL_1410575, EPI_ISL_1410580, EPI_ISL_1410588, EPI_ISL_1410591, EPI_ISL_1410593 to 1410594, EPI_ISL_1410596 to 1410597, EPI_ISL_1410602, EPI_ISL_1410607, EPI_ISL_1410612, EPI_ISL_1410615, EPI_ISL_1410620, EPI_ISL_1410622, EPI_ISL_1410625, EPI_ISL_1410627 to 1410628, EPI_ISL_1410630, EPI_ISL_1410632, EPI_ISL_1410639, EPI_ISL_1410641 to 1410642, EPI_ISL_1410644, EPI_ISL_1410646, EPI_ISL_1410649, EPI_ISL_1410651 to 1410652, EPI_ISL_1410659, EPI_ISL_1410662, EPI_ISL_1410664 to 1410665, EPI_ISL_1410667 to 1410668, EPI_ISL_1410670, EPI_ISL_1410672, EPI_ISL_1410678, EPI_ISL_1410680 to 1410681, EPI_ISL_1410683, EPI_ISL_1410688 to 1410689, EPI_ISL_1410691, EPI_ISL_1410698, EPI_ISL_1410704, EPI_ISL_1410706, EPI_ISL_1410708, EPI_ISL_1410710, EPI_ISL_1410713 to 1410714, EPI_ISL_1410718, EPI_ISL_1410724, EPI_ISL_1410727, EPI_ISL_1410729, EPI_ISL_1410732, EPI_ISL_1410739 to 1410740, EPI_ISL_1410742, EPI_ISL_1410748, EPI_ISL_1410752, EPI_ISL_1410754, EPI_ISL_1410757, EPI_ISL_1410762 to 1410763, EPI_ISL_1410765 to 1410766, EPI_ISL_1410768, EPI_ISL_1410770 to 1410771, EPI_ISL_1410775, EPI_ISL_1410777, EPI_ISL_1410780 to 1410781, EPI_ISL_1410783, EPI_ISL_1410786, EPI_ISL_1410789, EPI_ISL_1410793, EPI_ISL_1410796, EPI_ISL_1410798, EPI_ISL_1410802 to 1410806, EPI_ISL_1410808 to 1410809, EPI_ISL_1410815 to 1410818, EPI_ISL_1410821, EPI_ISL_1410823 to 1410826, EPI_ISL_1410828, EPI_ISL_1410830 to 1410832, EPI_ISL_1410834, EPI_ISL_1410836 to 1410838, EPI_ISL_1410846, EPI_ISL_1410848, EPI_ISL_1410851 to 1410852, EPI_ISL_1410855, EPI_ISL_1410857, EPI_ISL_1410859 to 1410860, EPI_ISL_1410862 to 1410863, EPI_ISL_1410866, EPI_ISL_1410868 to 1410872, EPI_ISL_1410874 to 1410877, EPI_ISL_1410883, EPI_ISL_1410885 to 1410889, EPI_ISL_1410891, EPI_ISL_1410893 to 1410894, EPI_ISL_1410896 to 1410897, EPI_ISL_1410899, EPI_ISL_1410901 to 1410902, EPI_ISL_1410904, EPI_ISL_1410907, EPI_ISL_1410910 to 1410911, EPI_ISL_1410913, EPI_ISL_1410915, EPI_ISL_1410918 to 1410919, EPI_ISL_1410924, EPI_ISL_1410928, EPI_ISL_1410930 to 1410931, EPI_ISL_1410935, EPI_ISL_1410938, EPI_ISL_1410940, EPI_ISL_1410942, EPI_ISL_1410944, EPI_ISL_1410946 to 1410947, EPI_ISL_1410949, EPI_ISL_1410951, EPI_ISL_1410953 to 1410954, EPI_ISL_1410957 to 1410958, EPI_ISL_1410961 to 1410963, EPI_ISL_1410971 to 1410974, EPI_ISL_1410977, EPI_ISL_1410981, EPI_ISL_1410983, EPI_ISL_1410985 to 1410988, EPI_ISL_1410990, EPI_ISL_1410994 to 1410995, EPI_ISL_1410997, EPI_ISL_1410999 to 1411002, EPI_ISL_1411010, EPI_ISL_1411015, EPI_ISL_1411019, EPI_ISL_1411022, EPI_ISL_1411024, EPI_ISL_1411028 to 1411030, EPI_ISL_1411032, EPI_ISL_1411035 to 1411039, EPI_ISL_1411041, EPI_ISL_1411044 to 1411045, EPI_ISL_1411051 to 1411054, EPI_ISL_1411056, EPI_ISL_1411058, EPI_ISL_1411060 to 1411062, EPI_ISL_1411067, EPI_ISL_1411069 to 1411073, EPI_ISL_1411075, EPI_ISL_1411080, EPI_ISL_1411085 to 1411086, EPI_ISL_1411089, EPI_ISL_1411091, EPI_ISL_1411094, EPI_ISL_1411100, EPI_ISL_1411457 to 1411458, EPI_ISL_1411462, EPI_ISL_1411465, EPI_ISL_1411468, EPI_ISL_1411470, EPI_ISL_1411473, EPI_ISL_1411476 to 1411477, EPI_ISL_1411482, EPI_ISL_1411492, EPI_ISL_1411497 to 1411498, EPI_ISL_1411501, EPI_ISL_1411503, EPI_ISL_1411508, EPI_ISL_1411556, EPI_ISL_1411558, EPI_ISL_1411567, EPI_ISL_1411569, EPI_ISL_1411579, EPI_ISL_1411581 to 1411582, EPI_ISL_1411587, EPI_ISL_1411589, EPI_ISL_1411592, EPI_ISL_1411600, EPI_ISL_1411618, EPI_ISL_1411625, EPI_ISL_1411651 to 1411652, EPI_ISL_1411656 to 1411658, EPI_ISL_1411665, EPI_ISL_1411671, EPI_ISL_1411675, EPI_ISL_1411680, EPI_ISL_1411682 to 1411684, EPI_ISL_1411687, EPI_ISL_1411689, EPI_ISL_1411691, EPI_ISL_1411694, EPI_ISL_1411697 to 1411698, EPI_ISL_1411701, EPI_ISL_1411705, EPI_ISL_1411708 to 1411711, EPI_ISL_1411727, EPI_ISL_1411729, EPI_ISL_1411731, EPI_ISL_1411735, EPI_ISL_1411740 to 1411741, EPI_ISL_1411753, EPI_ISL_1411758, EPI_ISL_1411765 to 1411766, EPI_ISL_1411774 to 1411775, EPI_ISL_1411779, EPI_ISL_1411781, EPI_ISL_1411788, EPI_ISL_1411791, EPI_ISL_1411794, EPI_ISL_1411797, EPI_ISL_1411804, EPI_ISL_1411806, EPI_ISL_1411809 to 1411811, EPI_ISL_1411816, EPI_ISL_1411819 to 1411820, EPI_ISL_1411826 to 1411827, EPI_ISL_1411840, EPI_ISL_1411848 to 1411849, EPI_ISL_1411852 to 1411860, EPI_ISL_1411862 to 1411870, EPI_ISL_1411874 to 1411875, EPI_ISL_1411877 to 1411878, EPI_ISL_1411880, EPI_ISL_1411882, EPI_ISL_1411884, EPI_ISL_1411886, EPI_ISL_1411888 to 1411889, EPI_ISL_1411893, EPI_ISL_1411895 to 1411908, EPI_ISL_1411910 to 1411911, EPI_ISL_1411913, EPI_ISL_1411916, EPI_ISL_1411919 to 1411926, EPI_ISL_1411928 to 1411931, EPI_ISL_1411933 to 1411939, EPI_ISL_1411941 to 1411945, EPI_ISL_1411947 to 1411951, EPI_ISL_1411953 to 1411956, EPI_ISL_1411959 to 1411970, EPI_ISL_1411973 to 1411988, EPI_ISL_1411990 to 1411996, EPI_ISL_1411998, EPI_ISL_1412000 to 1412004, EPI_ISL_1412006, EPI_ISL_1412008 to 1412010, EPI_ISL_1412012 to 1412015, EPI_ISL_1412017 to 1412021, EPI_ISL_1412024 to 1412025, EPI_ISL_1412027 to 1412030, EPI_ISL_1412032 to 1412034, EPI_ISL_1412036, EPI_ISL_1412038, EPI_ISL_1412041 to 1412042, EPI_ISL_1412046, EPI_ISL_1412048, EPI_ISL_1412050, EPI_ISL_1412053 to 1412054, EPI_ISL_1412056 to 1412058, EPI_ISL_1412060 to 1412069, EPI_ISL_1412072 to 1412074, EPI_ISL_1412079 to 1412085, EPI_ISL_1412087 to 1412090, EPI_ISL_1412094 to 1412097, EPI_ISL_1412100 to 1412101, EPI_ISL_1412103 to 1412111, EPI_ISL_1412113, EPI_ISL_1412115 to 1412116, EPI_ISL_1412118 to 1412122, EPI_ISL_1412125 to 1412128, EPI_ISL_1412131 to 1412134, EPI_ISL_1412137 to 1412138, EPI_ISL_1412141, EPI_ISL_1412143, EPI_ISL_1412145 to 1412146, EPI_ISL_1412148 to 1412151, EPI_ISL_1412153 to 1412156, EPI_ISL_1412158 to 1412160, EPI_ISL_1412162, EPI_ISL_1412164 to 1412168, EPI_ISL_1412171 to 1412172, EPI_ISL_1412174 to 1412175, EPI_ISL_1412177 to 1412180, EPI_ISL_1412182, EPI_ISL_1412184 to 1412185, EPI_ISL_1412187 to 1412205, EPI_ISL_1412207 to 1412211, EPI_ISL_1412213 to 1412223, EPI_ISL_1412225 to 1412228, EPI_ISL_1412230 to 1412234, EPI_ISL_1412236 to 1412238, EPI_ISL_1412242 to 1412259, EPI_ISL_1412262 to 1412263, EPI_ISL_1412266 to 1412267, EPI_ISL_1412269, EPI_ISL_1412271 to 1412280, EPI_ISL_1412282 to 1412287, EPI_ISL_1412289 to 1412297, EPI_ISL_1412299 to 1412300, EPI_ISL_1412302 to 1412305, EPI_ISL_1412307, EPI_ISL_1412309 to 1412310, EPI_ISL_1412312 to 1412316, EPI_ISL_1412318 to 1412319, EPI_ISL_1412322 to 1412324, EPI_ISL_1412326 to 1412327, EPI_ISL_1412329, EPI_ISL_1412334 to 1412336, EPI_ISL_1412341 to 1412344, EPI_ISL_1412347 to 1412353, EPI_ISL_1412355, EPI_ISL_1412357, EPI_ISL_1412359 to 1412360, EPI_ISL_1412362 to 1412364, EPI_ISL_1412366 to 1412367, EPI_ISL_1412370 to 1412371, EPI_ISL_1412373, EPI_ISL_1412375, EPI_ISL_1412377 to 1412382, EPI_ISL_1412384 to 1412385, EPI_ISL_1412387, EPI_ISL_1412389 to 1412391, EPI_ISL_1412393, EPI_ISL_1412395 to 1412398, EPI_ISL_1412400 to 1412403, EPI_ISL_1412406 to 1412409, EPI_ISL_1412412 to 1412413, EPI_ISL_1412415, EPI_ISL_1412418 to 1412419, EPI_ISL_1412421 to 1412430, EPI_ISL_1412432 to 1412434, EPI_ISL_1412436, EPI_ISL_1412438, EPI_ISL_1412441 to 1412448, EPI_ISL_1412451 to 1412453, EPI_ISL_1412455 to 1412461, EPI_ISL_1412465, EPI_ISL_1412467, EPI_ISL_1412469 to 1412474, EPI_ISL_1412477, EPI_ISL_1412479 to 1412480, EPI_ISL_1412482 to 1412483, EPI_ISL_1412485, EPI_ISL_1412488 to 1412489, EPI_ISL_1412491 to 1412492, EPI_ISL_1412494, EPI_ISL_1412497 to 1412498, EPI_ISL_1412500 to 1412501, EPI_ISL_1412503 to 1412504, EPI_ISL_1412506 to 1412507, EPI_ISL_1412511 to 1412512, EPI_ISL_1412514 to 1412515, EPI_ISL_1412517 to 1412518, EPI_ISL_1412520 to 1412521, EPI_ISL_1412523 to 1412524, EPI_ISL_1412526, EPI_ISL_1412529, EPI_ISL_1412531 to 1412538, EPI_ISL_1412540 to 1412544, EPI_ISL_1412546, EPI_ISL_1412548 to 1412549, EPI_ISL_1412551 to 1412563, EPI_ISL_1412565 to 1412567, EPI_ISL_1412574, EPI_ISL_1412577, EPI_ISL_1412579, EPI_ISL_1412588, EPI_ISL_1412590 to 1412593, EPI_ISL_1412596, EPI_ISL_1412599, EPI_ISL_1412601, EPI_ISL_1412603 to 1412604, EPI_ISL_1412606 to 1412607, EPI_ISL_1412610 to 1412611, EPI_ISL_1412613, EPI_ISL_1412618 to 1412620, EPI_ISL_1412623, EPI_ISL_1412627, EPI_ISL_1412631, EPI_ISL_1412633 to 1412636, EPI_ISL_1412638 to 1412639, EPI_ISL_1412642, EPI_ISL_1412645, EPI_ISL_1412649, EPI_ISL_1412651 to 1412653, EPI_ISL_1412655 to 1412656, EPI_ISL_1412660 to 1412661, EPI_ISL_1412665, EPI_ISL_1412667 to 1412671, EPI_ISL_1412677, EPI_ISL_1412679 to 1412681, EPI_ISL_1412684 to 1412687, EPI_ISL_1412689, EPI_ISL_1412695 to 1412697, EPI_ISL_1412699 to 1412700, EPI_ISL_1412707, EPI_ISL_1412709 to 1412710, EPI_ISL_1412714, EPI_ISL_1412716, EPI_ISL_1412718, EPI_ISL_1412721, EPI_ISL_1412726, EPI_ISL_1412728, EPI_ISL_1412730 to 1412733, EPI_ISL_1412737 to 1412738, EPI_ISL_1412742, EPI_ISL_1412746, EPI_ISL_1412749 to 1412750, EPI_ISL_1412752 to 1412753, EPI_ISL_1412756, EPI_ISL_1412758 to 1412761, EPI_ISL_1412766, EPI_ISL_1412769 to 1412770, EPI_ISL_1412772 to 1412774, EPI_ISL_1412778, EPI_ISL_1412780, EPI_ISL_1412784 to 1412785, EPI_ISL_1412788 to 1412789, EPI_ISL_1412791, EPI_ISL_1412795 to 1412796, EPI_ISL_1412799 to 1412801, EPI_ISL_1412803, EPI_ISL_1412808, EPI_ISL_1412810, EPI_ISL_1412814 to 1412815, EPI_ISL_1412818 to 1412819, EPI_ISL_1412821 to 1412822, EPI_ISL_1412824 to 1412826, EPI_ISL_1412829, EPI_ISL_1412832 to 1412833, EPI_ISL_1412835 to 1412836, EPI_ISL_1412838 to 1412845, EPI_ISL_1412849 to 1412851, EPI_ISL_1412853 to 1412856, EPI_ISL_1412860, EPI_ISL_1412862, EPI_ISL_1412864, EPI_ISL_1412869 to 1412874, EPI_ISL_1412878, EPI_ISL_1412880, EPI_ISL_1412882, EPI_ISL_1412884, EPI_ISL_1412886 to 1412888, EPI_ISL_1412890, EPI_ISL_1412892, EPI_ISL_1412895 to 1412897, EPI_ISL_1412900, EPI_ISL_1412904 to 1412908, EPI_ISL_1412910 to 1412913, EPI_ISL_1412915, EPI_ISL_1412917, EPI_ISL_1412919, EPI_ISL_1412923, EPI_ISL_1412925 to 1412926, EPI_ISL_1413061, EPI_ISL_1413064, EPI_ISL_1413066 to 1413067, EPI_ISL_1413073 to 1413074, EPI_ISL_1413078 to 1413079, EPI_ISL_1413082, EPI_ISL_1413084, EPI_ISL_1413086, EPI_ISL_1413090 to 1413091, EPI_ISL_1413093, EPI_ISL_1413096, EPI_ISL_1413099, EPI_ISL_1413104, EPI_ISL_1413107, EPI_ISL_1413111, EPI_ISL_1413114 to 1413120, EPI_ISL_1413123, EPI_ISL_1413125, EPI_ISL_1413127 to 1413129, EPI_ISL_1413131 to 1413132, EPI_ISL_1413134, EPI_ISL_1413138, EPI_ISL_1413140, EPI_ISL_1413142, EPI_ISL_1413145 to 1413146, EPI_ISL_1413149, EPI_ISL_1413155 to 1413156, EPI_ISL_1413164, EPI_ISL_1413167, EPI_ISL_1413169, EPI_ISL_1413174, EPI_ISL_1413178, EPI_ISL_1413182 to 1413183, EPI_ISL_1413185 to 1413186, EPI_ISL_1413189, EPI_ISL_1413195, EPI_ISL_1413197, EPI_ISL_1413203, EPI_ISL_1413206, EPI_ISL_1413208, EPI_ISL_1413210, EPI_ISL_1413213 to 1413214, EPI_ISL_1413216, EPI_ISL_1413222 to 1413223, EPI_ISL_1413225, EPI_ISL_1413227, EPI_ISL_1413231, EPI_ISL_1413234, EPI_ISL_1413236, EPI_ISL_1413239 to 1413240, EPI_ISL_1413245, EPI_ISL_1413248, EPI_ISL_1413250, EPI_ISL_1413253, EPI_ISL_1413255, EPI_ISL_1413258, EPI_ISL_1413261, EPI_ISL_1413265, EPI_ISL_1413268, EPI_ISL_1413270, EPI_ISL_1413273, EPI_ISL_1413278, EPI_ISL_1413281, EPI_ISL_1413284, EPI_ISL_1413286, EPI_ISL_1413289, EPI_ISL_1413292, EPI_ISL_1413297, EPI_ISL_1413300 to 1413301, EPI_ISL_1413305 to 1413306, EPI_ISL_1413308, EPI_ISL_1413313, EPI_ISL_1413315, EPI_ISL_1413317, EPI_ISL_1413321, EPI_ISL_1413323, EPI_ISL_1413327, EPI_ISL_1413329, EPI_ISL_1413332, EPI_ISL_1413337, EPI_ISL_1413343, EPI_ISL_1413345, EPI_ISL_1413348 to 1413349, EPI_ISL_1413351, EPI_ISL_1413353, EPI_ISL_1413356 to 1413357, EPI_ISL_1413359 to 1413360, EPI_ISL_1413365, EPI_ISL_1413370 to 1413371, EPI_ISL_1413373, EPI_ISL_1413375 to 1413376, EPI_ISL_1413381, EPI_ISL_1413383 to 1413384, EPI_ISL_1413387, EPI_ISL_1413389, EPI_ISL_1413395, EPI_ISL_1413398, EPI_ISL_1413402, EPI_ISL_1413409, EPI_ISL_1413412, EPI_ISL_1413415, EPI_ISL_1413417, EPI_ISL_1413419 to 1413420, EPI_ISL_1413428, EPI_ISL_1413438, EPI_ISL_1413443 to 1413444, EPI_ISL_1413446, EPI_ISL_1413457, EPI_ISL_1413459, EPI_ISL_1413461 to 1413462, EPI_ISL_1413464, EPI_ISL_1413466, EPI_ISL_1413472, EPI_ISL_1413474, EPI_ISL_1413480 to 1413481, EPI_ISL_1413484, EPI_ISL_1413487, EPI_ISL_1413492, EPI_ISL_1413495, EPI_ISL_1413500, EPI_ISL_1413502 to 1413503, EPI_ISL_1413505, EPI_ISL_1413507, EPI_ISL_1413510, EPI_ISL_1413517 to 1413518, EPI_ISL_1413521, EPI_ISL_1413526, EPI_ISL_1413539, EPI_ISL_1413543, EPI_ISL_1413545, EPI_ISL_1413548, EPI_ISL_1413550, EPI_ISL_1413552, EPI_ISL_1413555, EPI_ISL_1413558, EPI_ISL_1413560, EPI_ISL_1413562, EPI_ISL_1413565, EPI_ISL_1413567, EPI_ISL_1413570, EPI_ISL_1413573, EPI_ISL_1413577 to 1413578, EPI_ISL_1413581, EPI_ISL_1413584, EPI_ISL_1413586, EPI_ISL_1413595, EPI_ISL_1413604, EPI_ISL_1413606, EPI_ISL_1413609, EPI_ISL_1413617, EPI_ISL_1413629, EPI_ISL_1413640, EPI_ISL_1413668, EPI_ISL_1413673, EPI_ISL_1413677, EPI_ISL_1413679, EPI_ISL_1413691 to 1413692, EPI_ISL_1413694, EPI_ISL_1413716 to 1413717, EPI_ISL_1413738, EPI_ISL_1413740 to 1413741, EPI_ISL_1413747, EPI_ISL_1413752, EPI_ISL_1413756, EPI_ISL_1413770, EPI_ISL_1413800 to 1413801, EPI_ISL_1413806, EPI_ISL_1413811, EPI_ISL_1413817, EPI_ISL_1413825, EPI_ISL_1413834, EPI_ISL_1413841, EPI_ISL_1413843, EPI_ISL_1413849, EPI_ISL_1413858, EPI_ISL_1413867, EPI_ISL_1413869, EPI_ISL_1413871, EPI_ISL_1413873 to 1413874, EPI_ISL_1413883, EPI_ISL_1413889, EPI_ISL_1413891, EPI_ISL_1413894, EPI_ISL_1413896, EPI_ISL_1413903, EPI_ISL_1413905, EPI_ISL_1413913, EPI_ISL_1413920 to 1413921, EPI_ISL_1413924, EPI_ISL_1413931, EPI_ISL_1413933 to 1413934, EPI_ISL_1413944, EPI_ISL_1413947, EPI_ISL_1413958 to 1413959, EPI_ISL_1413967, EPI_ISL_1413969, EPI_ISL_1413971, EPI_ISL_1413978 to 1413980, EPI_ISL_1413983, EPI_ISL_1413985, EPI_ISL_1413990, EPI_ISL_1413993, EPI_ISL_1413997, EPI_ISL_1413999 to 1414001, EPI_ISL_1414004, EPI_ISL_1414006 to 1414007, EPI_ISL_1414014, EPI_ISL_1414018, EPI_ISL_1414025, EPI_ISL_1414032, EPI_ISL_1414035, EPI_ISL_1414043, EPI_ISL_1414048, EPI_ISL_1414050, EPI_ISL_1414054, EPI_ISL_1414057, EPI_ISL_1414062 to 1414433, EPI_ISL_1414435 to 1414436, EPI_ISL_1414438 to 1415060, EPI_ISL_1415429 to 1415815, EPI_ISL_1415817, EPI_ISL_1415819 to 1415821, EPI_ISL_1415825 to 1415826, EPI_ISL_1415829 to 1415833, EPI_ISL_1415836 to 1415837, EPI_ISL_1415841 to 1415842, EPI_ISL_1415851, EPI_ISL_1415853, EPI_ISL_1415855 to 1415856, EPI_ISL_1415860, EPI_ISL_1415862, EPI_ISL_1415875 to 1415877, EPI_ISL_1415879 to 1415883, EPI_ISL_1415886, EPI_ISL_1415891, EPI_ISL_1415893 to 1415894, EPI_ISL_1415901, EPI_ISL_1415904, EPI_ISL_1415910 to 1415911, EPI_ISL_1415914 to 1415918, EPI_ISL_1415924, EPI_ISL_1415926, EPI_ISL_1415928 to 1415929, EPI_ISL_1415931 to 1415932, EPI_ISL_1415934, EPI_ISL_1415937 to 1415938, EPI_ISL_1415941 to 1415942, EPI_ISL_1415945, EPI_ISL_1415947 to 1415949, EPI_ISL_1415954, EPI_ISL_1415958, EPI_ISL_1415960, EPI_ISL_1415963 to 1415964, EPI_ISL_1415966, EPI_ISL_1415968 to 1415969, EPI_ISL_1415972, EPI_ISL_1415978 to 1415979, EPI_ISL_1415985, EPI_ISL_1415988, EPI_ISL_1415991, EPI_ISL_1415997, EPI_ISL_1416002 to 1416004, EPI_ISL_1416007, EPI_ISL_1416011 to 1416012, EPI_ISL_1416015 to 1416016, EPI_ISL_1416019, EPI_ISL_1416021, EPI_ISL_1416029 to 1416030, EPI_ISL_1416032 to 1416036, EPI_ISL_1416039, EPI_ISL_1416041, EPI_ISL_1416044 to 1416045, EPI_ISL_1416047, EPI_ISL_1416052, EPI_ISL_1416055, EPI_ISL_1416057 to 1416058, EPI_ISL_1416063, EPI_ISL_1416066, EPI_ISL_1416072 to 1416073, EPI_ISL_1416076 to 1416078, EPI_ISL_1416080 to 1416084, EPI_ISL_1416087 to 1416091, EPI_ISL_1416095 to 1416096, EPI_ISL_1416103, EPI_ISL_1416106 to 1416108, EPI_ISL_1416111, EPI_ISL_1416115, EPI_ISL_1416118 to 1416119, EPI_ISL_1416122, EPI_ISL_1416124 to 1416125, EPI_ISL_1416172 to 1416173, EPI_ISL_1416175 to 1416179, EPI_ISL_1449029 to 1449349, EPI_ISL_1449352, EPI_ISL_1449357 to 1449358, EPI_ISL_1449360, EPI_ISL_1449370 to 1449371, EPI_ISL_1449373, EPI_ISL_1449375, EPI_ISL_1449380, EPI_ISL_1449382, EPI_ISL_1449384, EPI_ISL_1449386, EPI_ISL_1449392 to 1449394, EPI_ISL_1449399, EPI_ISL_1449401 to 1449402, EPI_ISL_1449408, EPI_ISL_1449411, EPI_ISL_1449413 to 1449415, EPI_ISL_1449417, EPI_ISL_1449419, EPI_ISL_1449421 to 1449424, EPI_ISL_1449426, EPI_ISL_1449432, EPI_ISL_1449438, EPI_ISL_1449442, EPI_ISL_1449447, EPI_ISL_1449451, EPI_ISL_1449453, EPI_ISL_1449455 to 1449456, EPI_ISL_1449458, EPI_ISL_1449460, EPI_ISL_1449463 to 1449467, EPI_ISL_1449469 to 1449470, EPI_ISL_1449473, EPI_ISL_1449475, EPI_ISL_1449479 to 1449480, EPI_ISL_1449487 to 1449488, EPI_ISL_1449490, EPI_ISL_1449495 to 1449497, EPI_ISL_1449499 to 1449500, EPI_ISL_1449502, EPI_ISL_1449504 to 1449505, EPI_ISL_1449507, EPI_ISL_1449510, EPI_ISL_1449512 to 1449514, EPI_ISL_1449519, EPI_ISL_1449525 to 1449526, EPI_ISL_1449531, EPI_ISL_1449534, EPI_ISL_1449539, EPI_ISL_1449549, EPI_ISL_1449552, EPI_ISL_1449555, EPI_ISL_1449565, EPI_ISL_1449568 to 1449569, EPI_ISL_1449574, EPI_ISL_1449577 to 1449580, EPI_ISL_1449584, EPI_ISL_1449587, EPI_ISL_1449589, EPI_ISL_1449596, EPI_ISL_1449599, EPI_ISL_1449610, EPI_ISL_1449612 to 1449613, EPI_ISL_1449615, EPI_ISL_1449622, EPI_ISL_1449625, EPI_ISL_1449629, EPI_ISL_1449633, EPI_ISL_1449639, EPI_ISL_1449642, EPI_ISL_1449651, EPI_ISL_1449654 to 1449661, EPI_ISL_1449663 to 1449667, EPI_ISL_1449669 to 1449671, EPI_ISL_1449675 to 1449680, EPI_ISL_1449683 to 1449689, EPI_ISL_1449691, EPI_ISL_1449694, EPI_ISL_1449696, EPI_ISL_1449699 to 1449701, EPI_ISL_1449703 to 1449704, EPI_ISL_1449706 to 1449708, EPI_ISL_1449710 to 1449714, EPI_ISL_1449717, EPI_ISL_1449720, EPI_ISL_1449723 to 1449724, EPI_ISL_1449726 to 1449729, EPI_ISL_1449731 to 1449732, EPI_ISL_1449734 to 1449736, EPI_ISL_1449739, EPI_ISL_1449742 to 1449745, EPI_ISL_1449747 to 1449748, EPI_ISL_1449752 to 1449756, EPI_ISL_1449758 to 1449765, EPI_ISL_1449767 to 1449768, EPI_ISL_1449770 to 1449777, EPI_ISL_1449780, EPI_ISL_1449783 to 1449787, EPI_ISL_1449789 to 1449794, EPI_ISL_1449796 to 1449799, EPI_ISL_1449803 to 1449804, EPI_ISL_1449806 to 1449808, EPI_ISL_1449810 to 1449813, EPI_ISL_1449815 to 1449816, EPI_ISL_1449818 to 1449823, EPI_ISL_1449825, EPI_ISL_1449827 to 1449838, EPI_ISL_1449840 to 1449843, EPI_ISL_1449845, EPI_ISL_1449848, EPI_ISL_1449851 to 1449856, EPI_ISL_1449858 to 1449861, EPI_ISL_1449864 to 1449865, EPI_ISL_1449867 to 1449869, EPI_ISL_1449871 to 1449875, EPI_ISL_1449877 to 1449879, EPI_ISL_1449881 to 1449882, EPI_ISL_1449884 to 1449885, EPI_ISL_1449888, EPI_ISL_1449890 to 1449900, EPI_ISL_1449902 to 1449905, EPI_ISL_1449907, EPI_ISL_1449910 to 1449912, EPI_ISL_1449914 to 1449922, EPI_ISL_1449925 to 1449929, EPI_ISL_1449931 to 1449933, EPI_ISL_1449935 to 1449936, EPI_ISL_1449938 to 1449939, EPI_ISL_1449943, EPI_ISL_1449945 to 1449950, EPI_ISL_1449952 to 1449955, EPI_ISL_1449957 to 1449961, EPI_ISL_1449963 to 1449967, EPI_ISL_1449969, EPI_ISL_1449971 to 1449972, EPI_ISL_1449976 to 1449980, EPI_ISL_1449983 to 1449985, EPI_ISL_1449987 to 1449988, EPI_ISL_1449990, EPI_ISL_1449992, EPI_ISL_1449994 to 1449996, EPI_ISL_1449998, EPI_ISL_1450000, EPI_ISL_1450002 to 1450003, EPI_ISL_1450005, EPI_ISL_1450007, EPI_ISL_1450009 to 1450014, EPI_ISL_1450017, EPI_ISL_1450021, EPI_ISL_1450023, EPI_ISL_1450027, EPI_ISL_1450030, EPI_ISL_1450032, EPI_ISL_1450035 to 1450041, EPI_ISL_1450043, EPI_ISL_1450046, EPI_ISL_1450048 to 1450050, EPI_ISL_1450052, EPI_ISL_1450057, EPI_ISL_1450062, EPI_ISL_1450064, EPI_ISL_1450067 to 1450070, EPI_ISL_1450072, EPI_ISL_1450074, EPI_ISL_1450076 to 1450079, EPI_ISL_1450081 to 1450082, EPI_ISL_1450084, EPI_ISL_1450089 to 1450094, EPI_ISL_1450096 to 1450098, EPI_ISL_1450102 to 1450104, EPI_ISL_1450107 to 1450108, EPI_ISL_1450111 to 1450112, EPI_ISL_1450114, EPI_ISL_1450116 to 1450121, EPI_ISL_1450123, EPI_ISL_1450126 to 1450127, EPI_ISL_1450129 to 1450135, EPI_ISL_1450137, EPI_ISL_1450141 to 1450143, EPI_ISL_1450146 to 1450150, EPI_ISL_1450152, EPI_ISL_1450155 to 1450156, EPI_ISL_1450164 to 1450165, EPI_ISL_1450167, EPI_ISL_1450169 to 1450170, EPI_ISL_1450172, EPI_ISL_1450177 to 1450178, EPI_ISL_1450181 to 1450183, EPI_ISL_1450187 to 1450189, EPI_ISL_1450193, EPI_ISL_1450197 to 1450198, EPI_ISL_1450200 to 1450201, EPI_ISL_1450203 to 1450206, EPI_ISL_1450208 to 1450209, EPI_ISL_1450212 to 1450213, EPI_ISL_1450216 to 1450219, EPI_ISL_1450221 to 1450222, EPI_ISL_1450224, EPI_ISL_1450226, EPI_ISL_1450228 to 1450231, EPI_ISL_1450237 to 1450241, EPI_ISL_1450243 to 1450244, EPI_ISL_1450247 to 1450250, EPI_ISL_1450253, EPI_ISL_1450256 to 1450258, EPI_ISL_1450268, EPI_ISL_1450270 to 1450272, EPI_ISL_1450276 to 1450283, EPI_ISL_1450286 to 1450287, EPI_ISL_1450293 to 1450294, EPI_ISL_1450300 to 1450301, EPI_ISL_1450305, EPI_ISL_1450307 to 1450309, EPI_ISL_1450311, EPI_ISL_1450318 to 1450320, EPI_ISL_1450322 to 1450324, EPI_ISL_1450326, EPI_ISL_1450330, EPI_ISL_1450332, EPI_ISL_1450336, EPI_ISL_1450338, EPI_ISL_1450345 to 1450346, EPI_ISL_1450348, EPI_ISL_1450350, EPI_ISL_1450352, EPI_ISL_1450354, EPI_ISL_1450356, EPI_ISL_1450358, EPI_ISL_1450361, EPI_ISL_1450363, EPI_ISL_1450367 to 1450368, EPI_ISL_1450371, EPI_ISL_1450374, EPI_ISL_1450376 to 1450377, EPI_ISL_1450379 to 1450382, EPI_ISL_1450384, EPI_ISL_1450388 to 1450393, EPI_ISL_1450395, EPI_ISL_1450398 to 1450399, EPI_ISL_1450401 to 1450402, EPI_ISL_1450404 to 1450406, EPI_ISL_1450409 to 1450410, EPI_ISL_1450412, EPI_ISL_1450415 to 1450417, EPI_ISL_1450419 to 1450422, EPI_ISL_1450424, EPI_ISL_1450428 to 1450432, EPI_ISL_1450434 to 1450436, EPI_ISL_1450440, EPI_ISL_1450442, EPI_ISL_1450444 to 1450446, EPI_ISL_1450449, EPI_ISL_1450454 to 1450457, EPI_ISL_1450459, EPI_ISL_1450461 to 1450462, EPI_ISL_1450465, EPI_ISL_1450467, EPI_ISL_1450470 to 1450471, EPI_ISL_1450473 to 1450475, EPI_ISL_1450479 to 1450481, EPI_ISL_1450483 to 1450484, EPI_ISL_1450486, EPI_ISL_1450489 to 1450492, EPI_ISL_1450495, EPI_ISL_1450499, EPI_ISL_1450501, EPI_ISL_1450505 to 1450507, EPI_ISL_1450509 to 1450510, EPI_ISL_1450513 to 1450514, EPI_ISL_1450518, EPI_ISL_1450521, EPI_ISL_1450523, EPI_ISL_1450525, EPI_ISL_1450527 to 1450528, EPI_ISL_1450531, EPI_ISL_1450533 to 1450537, EPI_ISL_1450542, EPI_ISL_1450544 to 1450549, EPI_ISL_1450551, EPI_ISL_1450554 to 1450557, EPI_ISL_1450560, EPI_ISL_1450562, EPI_ISL_1450566, EPI_ISL_1450568 to 1450569, EPI_ISL_1450574 to 1450581, EPI_ISL_1450583, EPI_ISL_1450585 to 1450586, EPI_ISL_1450588 to 1450589, EPI_ISL_1450591 to 1450593, EPI_ISL_1450595, EPI_ISL_1450597, EPI_ISL_1450600 to 1450603, EPI_ISL_1451483 to 1451484, EPI_ISL_1451486, EPI_ISL_1451492,

EPI_ISL_1451501, EPI_ISL_1451504, EPI_ISL_1451507, EPI_ISL_1451510, EPI_ISL_1451514, EPI_ISL_1451516 to 1451517, EPI_ISL_1451522, EPI_ISL_1451524, EPI_ISL_1451526 to 1451527, EPI_ISL_1451531, EPI_ISL_1451535, EPI_ISL_1451544 to 1451546, EPI_ISL_1451548 to 1451549, EPI_ISL_1451552 to 1451553, EPI_ISL_1451560, EPI_ISL_1451562, EPI_ISL_1451564, EPI_ISL_1451566, EPI_ISL_1451569 to 1451570, EPI_ISL_1451575, EPI_ISL_1451588, EPI_ISL_1451591, EPI_ISL_1451595 to 1451597, EPI_ISL_1451606, EPI_ISL_1451611, EPI_ISL_1451618, EPI_ISL_1451621, EPI_ISL_1451623, EPI_ISL_1451625 to 1451626, EPI_ISL_1451636, EPI_ISL_1451638 to 1451639, EPI_ISL_1451645 to 1451647, EPI_ISL_1451655 to 1451656, EPI_ISL_1451662, EPI_ISL_1451669, EPI_ISL_1451672 to 1451674, EPI_ISL_1451680, EPI_ISL_1451686, EPI_ISL_1451688 to 1451689, EPI_ISL_1451691 to 1451692, EPI_ISL_1451695, EPI_ISL_1451698, EPI_ISL_1451702, EPI_ISL_1451709 to 1451711, EPI_ISL_1451715, EPI_ISL_1451720, EPI_ISL_1451724, EPI_ISL_1451728, EPI_ISL_1451730 to 1451731, EPI_ISL_1451734 to 1451735, EPI_ISL_1451737, EPI_ISL_1451739 to 1451742, EPI_ISL_1451744 to 1451746, EPI_ISL_1451751, EPI_ISL_1451753, EPI_ISL_1451762, EPI_ISL_1451767, EPI_ISL_1451773, EPI_ISL_1451775 to 1451776, EPI_ISL_1451784, EPI_ISL_1451788, EPI_ISL_1451792, EPI_ISL_1451794 to 1451795, EPI_ISL_1451797 to 1451798, EPI_ISL_1451800 to 1451801, EPI_ISL_1451803 to 1451804, EPI_ISL_1451807 to 1451810, EPI_ISL_1451813 to 1451814, EPI_ISL_1451816 to 1451817, EPI_ISL_1451819 to 1451828, EPI_ISL_1451830 to 1451831, EPI_ISL_1451833, EPI_ISL_1451835 to 1451836, EPI_ISL_1451839, EPI_ISL_1451843 to 1451844, EPI_ISL_1451846 to 1451849, EPI_ISL_1451851, EPI_ISL_1451853 to 1451857, EPI_ISL_1451860 to 1451861, EPI_ISL_1451863 to 1451868, EPI_ISL_1451871, EPI_ISL_1451873 to 1451874, EPI_ISL_1451877 to 1451878, EPI_ISL_1451880, EPI_ISL_1451884 to 1451885, EPI_ISL_1451889 to 1451891, EPI_ISL_1451893 to 1451895, EPI_ISL_1451897 to 1451899, EPI_ISL_1451901 to 1451902, EPI_ISL_1451905 to 1451906, EPI_ISL_1451908, EPI_ISL_1451910 to 1451913, EPI_ISL_1451915 to 1451916, EPI_ISL_1451920 to 1451921, EPI_ISL_1451923 to 1451929, EPI_ISL_1451933 to 1451934, EPI_ISL_1451938 to 1451939, EPI_ISL_1451941, EPI_ISL_1451943 to 1451955, EPI_ISL_1451959 to 1451965, EPI_ISL_1451968, EPI_ISL_1451971 to 1451974, EPI_ISL_1451976, EPI_ISL_1451978, EPI_ISL_1451981 to 1451986, EPI_ISL_1451989 to 1451991, EPI_ISL_1451993 to 1451997, EPI_ISL_1452000 to 1452001, EPI_ISL_1452004, EPI_ISL_1452006 to 1452007, EPI_ISL_1452009, EPI_ISL_1452012 to 1452013, EPI_ISL_1452015, EPI_ISL_1452018, EPI_ISL_1452020 to 1452029, EPI_ISL_1452031, EPI_ISL_1452033 to 1452034, EPI_ISL_1452037 to 1452038, EPI_ISL_1452042, EPI_ISL_1452044 to 1452049, EPI_ISL_1452055, EPI_ISL_1452057 to 1452059, EPI_ISL_1452064 to 1452072, EPI_ISL_1452074, EPI_ISL_1452077, EPI_ISL_1452086, EPI_ISL_1452090 to 1452093, EPI_ISL_1452095, EPI_ISL_1452104 to 1452105, EPI_ISL_1452107, EPI_ISL_1452109, EPI_ISL_1452113 to 1452115, EPI_ISL_1452119, EPI_ISL_1452122 to 1452123, EPI_ISL_1452126, EPI_ISL_1452128, EPI_ISL_1452131, EPI_ISL_1452135, EPI_ISL_1452138, EPI_ISL_1452141 to 1452144, EPI_ISL_1452146 to 1452147, EPI_ISL_1452158, EPI_ISL_1452160, EPI_ISL_1452162 to 1452163, EPI_ISL_1452169, EPI_ISL_1452172, EPI_ISL_1452174, EPI_ISL_1452177 to 1452178, EPI_ISL_1452187, EPI_ISL_1452189, EPI_ISL_1452191, EPI_ISL_1452194, EPI_ISL_1452198, EPI_ISL_1452204 to 1452205, EPI_ISL_1452208, EPI_ISL_1452210, EPI_ISL_1452212, EPI_ISL_1452215 to 1452216, EPI_ISL_1452220, EPI_ISL_1452228 to 1452229, EPI_ISL_1452233, EPI_ISL_1452238 to 1452240, EPI_ISL_1452242, EPI_ISL_1452245 to 1452246, EPI_ISL_1452252 to 1452253, EPI_ISL_1452256 to 1452258, EPI_ISL_1452260, EPI_ISL_1452278, EPI_ISL_1452280, EPI_ISL_1452284, EPI_ISL_1452289, EPI_ISL_1452295, EPI_ISL_1452299, EPI_ISL_1452302, EPI_ISL_1452306, EPI_ISL_1452308 to 1452309, EPI_ISL_1452315, EPI_ISL_1452317 to 1452318, EPI_ISL_1452320, EPI_ISL_1452322, EPI_ISL_1452325 to 1452326, EPI_ISL_1452344, EPI_ISL_1452626, EPI_ISL_1452631 to 1452632, EPI_ISL_1452638, EPI_ISL_1452644, EPI_ISL_1452647, EPI_ISL_1452649 to 1452650, EPI_ISL_1452665, EPI_ISL_1452674, EPI_ISL_1452680, EPI_ISL_1452682, EPI_ISL_1452684 to 1452685, EPI_ISL_1452692 to 1452694, EPI_ISL_1452697, EPI_ISL_1452699, EPI_ISL_1452702 to 1452704, EPI_ISL_1452707 to 1452708, EPI_ISL_1452710 to 1452712, EPI_ISL_1452715 to 1452716, EPI_ISL_1452718, EPI_ISL_1452720, EPI_ISL_1452722, EPI_ISL_1452728 to 1452730, EPI_ISL_1452738, EPI_ISL_1452740, EPI_ISL_1452743, EPI_ISL_1452745, EPI_ISL_1452750 to 1452751, EPI_ISL_1452753, EPI_ISL_1452755, EPI_ISL_1452758, EPI_ISL_1452761, EPI_ISL_1452764, EPI_ISL_1452767 to 1452768, EPI_ISL_1452772, EPI_ISL_1452777 to 1452778, EPI_ISL_1452780 to 1452781, EPI_ISL_1452783, EPI_ISL_1452786 to 1452787, EPI_ISL_1452790, EPI_ISL_1452792, EPI_ISL_1452794 to 1452795, EPI_ISL_1452798, EPI_ISL_1452800, EPI_ISL_1452808, EPI_ISL_1452812, EPI_ISL_1452815, EPI_ISL_1452817, EPI_ISL_1452819, EPI_ISL_1452826, EPI_ISL_1452831, EPI_ISL_1452833, EPI_ISL_1452835 to 1452836, EPI_ISL_1452839, EPI_ISL_1452842, EPI_ISL_1452851, EPI_ISL_1452854, EPI_ISL_1452856 to 1452857, EPI_ISL_1452860, EPI_ISL_1452862, EPI_ISL_1452866, EPI_ISL_1452870 to 1452874, EPI_ISL_1452877, EPI_ISL_1452882, EPI_ISL_1452887, EPI_ISL_1452890 to 1452893, EPI_ISL_1452899, EPI_ISL_1452901, EPI_ISL_1452904 to 1452905, EPI_ISL_1452910 to 1452911, EPI_ISL_1452914, EPI_ISL_1452917, EPI_ISL_1452920, EPI_ISL_1452922 to 1452923, EPI_ISL_1452925, EPI_ISL_1452929, EPI_ISL_1452932, EPI_ISL_1452935, EPI_ISL_1452945, EPI_ISL_1452949 to 1452951, EPI_ISL_1452954, EPI_ISL_1452957, EPI_ISL_1452959, EPI_ISL_1452961, EPI_ISL_1452963, EPI_ISL_1452967, EPI_ISL_1452976, EPI_ISL_1452982, EPI_ISL_1452986 to 1452987, EPI_ISL_1452992, EPI_ISL_1452994, EPI_ISL_1453001, EPI_ISL_1453004, EPI_ISL_1453007, EPI_ISL_1453009 to 1453010, EPI_ISL_1453017 to 1453018, EPI_ISL_1453025 to 1453026, EPI_ISL_1453029, EPI_ISL_1453040, EPI_ISL_1453046, EPI_ISL_1453048, EPI_ISL_1453050 to 1453053, EPI_ISL_1453055, EPI_ISL_1453061 to 1453063, EPI_ISL_1453069 to 1453071, EPI_ISL_1453073, EPI_ISL_1453079, EPI_ISL_1453104, EPI_ISL_1453107 to 1453108, EPI_ISL_1453111, EPI_ISL_1453113, EPI_ISL_1453115 to 1453116, EPI_ISL_1453124, EPI_ISL_1453128, EPI_ISL_1453137, EPI_ISL_1453142, EPI_ISL_1453147 to 1453148, EPI_ISL_1453152, EPI_ISL_1453158, EPI_ISL_1453160, EPI_ISL_1453162, EPI_ISL_1453166, EPI_ISL_1453178, EPI_ISL_1453182 to 1453185, EPI_ISL_1453189 to 1453191, EPI_ISL_1453193 to 1453195, EPI_ISL_1453198, EPI_ISL_1453200 to 1453203, EPI_ISL_1453205, EPI_ISL_1453207 to 1453209, EPI_ISL_1453211, EPI_ISL_1453216 to 1453218, EPI_ISL_1453223 to 1453227, EPI_ISL_1453229 to 1453233, EPI_ISL_1453235 to 1453240, EPI_ISL_1453242 to 1453245, EPI_ISL_1453247 to 1453248, EPI_ISL_1453251 to 1453253, EPI_ISL_1453256 to 1453257, EPI_ISL_1453259, EPI_ISL_1453261 to 1453264, EPI_ISL_1453269 to 1453270, EPI_ISL_1453272, EPI_ISL_1453274 to 1453275, EPI_ISL_1453277, EPI_ISL_1453282, EPI_ISL_1453284 to 1453290, EPI_ISL_1453293, EPI_ISL_1453295 to 1453296, EPI_ISL_1453299, EPI_ISL_1453301 to 1453302, EPI_ISL_1453304 to 1453310, EPI_ISL_1453312, EPI_ISL_1453318, EPI_ISL_1453321 to 1453322, EPI_ISL_1453324, EPI_ISL_1453326, EPI_ISL_1453328, EPI_ISL_1453330, EPI_ISL_1453332 to 1453334, EPI_ISL_1453336, EPI_ISL_1453339 to 1453340, EPI_ISL_1453342 to 1453345, EPI_ISL_1453348 to 1453350, EPI_ISL_1453352 to 1453354, EPI_ISL_1453356 to 1453358, EPI_ISL_1453363, EPI_ISL_1453365 to 1453368, EPI_ISL_1453372, EPI_ISL_1453374, EPI_ISL_1453376 to 1453377, EPI_ISL_1453379 to 1453391, EPI_ISL_1453397, EPI_ISL_1453399, EPI_ISL_1453401 to 1453403, EPI_ISL_1453406, EPI_ISL_1453408, EPI_ISL_1453410, EPI_ISL_1453412, EPI_ISL_1453414, EPI_ISL_1453416 to 1453419, EPI_ISL_1453421 to 1453423, EPI_ISL_1453425, EPI_ISL_1453428 to 1453432, EPI_ISL_1453435 to 1453436, EPI_ISL_1453438 to 1453439, EPI_ISL_1453441, EPI_ISL_1453443, EPI_ISL_1453446, EPI_ISL_1453450 to 1453452, EPI_ISL_1453454 to 1453458, EPI_ISL_1453460 to 1453462, EPI_ISL_1453464 to 1453469, EPI_ISL_1453473, EPI_ISL_1453478, EPI_ISL_1453487, EPI_ISL_1453490 to 1453497, EPI_ISL_1453499, EPI_ISL_1453501 to 1453503, EPI_ISL_1453505, EPI_ISL_1453508 to 1453510, EPI_ISL_1453512, EPI_ISL_1453514 to 1453517, EPI_ISL_1453520, EPI_ISL_1453522 to 1453525, EPI_ISL_1453527 to 1453533, EPI_ISL_1453535, EPI_ISL_1453538 to 1453539, EPI_ISL_1453541 to 1453547, EPI_ISL_1453549, EPI_ISL_1453551 to 1453554, EPI_ISL_1453556 to 1453558, EPI_ISL_1453560 to 1453563, EPI_ISL_1453565 to 1453566, EPI_ISL_1453568, EPI_ISL_1453570, EPI_ISL_1453574 to 1453576, EPI_ISL_1453578, EPI_ISL_1453580 to 1453581, EPI_ISL_1453585, EPI_ISL_1453587 to 1453594, EPI_ISL_1453596 to 1453602, EPI_ISL_1453605 to 1453614, EPI_ISL_1453616 to 1453625, EPI_ISL_1453627 to 1453643, EPI_ISL_1453645, EPI_ISL_1453647, EPI_ISL_1453649 to 1453654, EPI_ISL_1453656 to 1453658, EPI_ISL_1453661 to 1453664, EPI_ISL_1453666 to 1453676, EPI_ISL_1453679 to 1453691, EPI_ISL_1453693 to 1453698, EPI_ISL_1453701, EPI_ISL_1453703 to 1453704, EPI_ISL_1453707, EPI_ISL_1453709 to 1453710, EPI_ISL_1453713, EPI_ISL_1453715 to 1453717, EPI_ISL_1453719 to 1453735, EPI_ISL_1453737 to 1453748, EPI_ISL_1453750 to 1453752, EPI_ISL_1453754 to 1453763, EPI_ISL_1453765 to 1453771, EPI_ISL_1453773 to 1453776, EPI_ISL_1453778, EPI_ISL_1453780, EPI_ISL_1453784 to 1453786, EPI_ISL_1453788 to 1453790, EPI_ISL_1453792, EPI_ISL_1453795, EPI_ISL_1453797 to 1453798, EPI_ISL_1453800 to 1453801, EPI_ISL_1453805 to 1453807, EPI_ISL_1453809 to 1453813, EPI_ISL_1453815 to 1453817, EPI_ISL_1453820, EPI_ISL_1453822 to 1453826, EPI_ISL_1453828 to 1453829, EPI_ISL_1453831, EPI_ISL_1453835 to 1453837, EPI_ISL_1453839 to 1453845, EPI_ISL_1453848 to 1453849, EPI_ISL_1453851 to 1453852, EPI_ISL_1453857, EPI_ISL_1453859 to 1453862, EPI_ISL_1453864, EPI_ISL_1453867, EPI_ISL_1453873 to 1453874, EPI_ISL_1453876 to 1453878, EPI_ISL_1453881 to 1453883, EPI_ISL_1453886, EPI_ISL_1453889, EPI_ISL_1453891 to 1453892, EPI_ISL_1453894 to 1453896, EPI_ISL_1453899 to 1453905, EPI_ISL_1453907, EPI_ISL_1453911 to 1453912, EPI_ISL_1453915, EPI_ISL_1453917 to 1453920, EPI_ISL_1453922 to 1453923, EPI_ISL_1453925, EPI_ISL_1453930, EPI_ISL_1453932 to 1453939, EPI_ISL_1453943 to 1453946, EPI_ISL_1453949 to 1453950, EPI_ISL_1453953 to 1453956, EPI_ISL_1453959 to 1453964, EPI_ISL_1453967, EPI_ISL_1453969, EPI_ISL_1453971 to 1453974, EPI_ISL_1453976 to 1453977, EPI_ISL_1453979 to 1453987, EPI_ISL_1453989 to 1453992, EPI_ISL_1453996, EPI_ISL_1453998 to 1453999, EPI_ISL_1454001, EPI_ISL_1454003, EPI_ISL_1454006, EPI_ISL_1454009 to 1454010, EPI_ISL_1454012, EPI_ISL_1454014 to 1454016, EPI_ISL_1454018 to 1454019, EPI_ISL_1454024, EPI_ISL_1454026 to 1454027, EPI_ISL_1454029 to 1454032, EPI_ISL_1454036 to 1454037, EPI_ISL_1454039 to 1454041, EPI_ISL_1454043, EPI_ISL_1454046, EPI_ISL_1454050, EPI_ISL_1454053 to 1454055, EPI_ISL_1454059, EPI_ISL_1454062 to 1454064, EPI_ISL_1454066, EPI_ISL_1454068, EPI_ISL_1454070 to 1454073, EPI_ISL_1454077 to 1454078, EPI_ISL_1454081 to 1454082, EPI_ISL_1454084, EPI_ISL_1454088, EPI_ISL_1454090 to 1454094, EPI_ISL_1454098 to 1454099, EPI_ISL_1454103, EPI_ISL_1454106, EPI_ISL_1454108 to 1454114, EPI_ISL_1454116, EPI_ISL_1454118 to 1454122, EPI_ISL_1454124, EPI_ISL_1454126 to 1454131, EPI_ISL_1454138 to 1454139, EPI_ISL_1454142 to 1454143, EPI_ISL_1454146, EPI_ISL_1454148, EPI_ISL_1454150 to 1454151, EPI_ISL_1454153 to 1454156, EPI_ISL_1454326 to 1454327, EPI_ISL_1454329 to 1454346, EPI_ISL_1454350 to 1454354, EPI_ISL_1454356, EPI_ISL_1454358 to 1454363, EPI_ISL_1454365 to 1454367, EPI_ISL_1454369 to 1454374, EPI_ISL_1454376 to 1454378, EPI_ISL_1454380 to 1454382, EPI_ISL_1454384 to 1454390, EPI_ISL_1454392 to 1454397, EPI_ISL_1454400 to 1454408, EPI_ISL_1454410, EPI_ISL_1454412 to 1454417, EPI_ISL_1454419 to 1454428, EPI_ISL_1454430 to 1454434, EPI_ISL_1454436 to 1454439, EPI_ISL_1454441, EPI_ISL_1454443 to 1454445, EPI_ISL_1454447, EPI_ISL_1454449 to 1454453, EPI_ISL_1454455 to 1454466, EPI_ISL_1454469 to 1454470, EPI_ISL_1454472 to 1454474, EPI_ISL_1454476 to 1454478, EPI_ISL_1454480 to 1454484, EPI_ISL_1454486 to 1454493, EPI_ISL_1454495 to 1454496, EPI_ISL_1454498 to 1454501, EPI_ISL_1454503, EPI_ISL_1454505 to 1454506, EPI_ISL_1454508 to 1454520, EPI_ISL_1454522, EPI_ISL_1454524, EPI_ISL_1454527 to 1454529, EPI_ISL_1454531 to 1454535, EPI_ISL_1454537 to 1454545, EPI_ISL_1454547 to 1454550, EPI_ISL_1454552, EPI_ISL_1454554 to 1454557, EPI_ISL_1454559 to 1454571, EPI_ISL_1454573 to 1454574, EPI_ISL_1454576 to 1454577, EPI_ISL_1454579 to 1454581, EPI_ISL_1454583, EPI_ISL_1454585 to 1454593, EPI_ISL_1454595 to 1454601, EPI_ISL_1454603 to 1454605, EPI_ISL_1454607 to 1454608, EPI_ISL_1454610 to 1454611, EPI_ISL_1454613 to 1454617, EPI_ISL_1454621 to 1454624, EPI_ISL_1454627, EPI_ISL_1454629 to 1454637, EPI_ISL_1454642 to 1454643, EPI_ISL_1454645, EPI_ISL_1454647 to 1454648, EPI_ISL_1454650, EPI_ISL_1454652 to 1454653, EPI_ISL_1454655 to 1454657, EPI_ISL_1454659 to 1454664, EPI_ISL_1454666 to 1454670, EPI_ISL_1454672 to 1454674, EPI_ISL_1454676 to 1454679, EPI_ISL_1454682, EPI_ISL_1454687, EPI_ISL_1454691, EPI_ISL_1454696, EPI_ISL_1454698 to 1454700, EPI_ISL_1454703 to 1454706, EPI_ISL_1454708 to 1454709, EPI_ISL_1454712 to 1454714, EPI_ISL_1454716 to 1454717, EPI_ISL_1454725 to 1454729, EPI_ISL_1454734 to 1454735, EPI_ISL_1454737 to 1454739, EPI_ISL_1454741, EPI_ISL_1454743 to 1454744, EPI_ISL_1454746 to 1454747, EPI_ISL_1454749 to 1454750, EPI_ISL_1454754 to 1454756, EPI_ISL_1454762, EPI_ISL_1454765 to 1454768, EPI_ISL_1454770, EPI_ISL_1454772, EPI_ISL_1454774 to 1454775, EPI_ISL_1454777, EPI_ISL_1454779 to 1454796, EPI_ISL_1454798, EPI_ISL_1454800 to 1454801, EPI_ISL_1454803, EPI_ISL_1454806 to 1454810, EPI_ISL_1454812, EPI_ISL_1454814 to 1454816, EPI_ISL_1454818, EPI_ISL_1454820 to 1454826, EPI_ISL_1454828 to 1454832, EPI_ISL_1454834 to 1454841, EPI_ISL_1454845, EPI_ISL_1454847 to 1454848, EPI_ISL_1454851, EPI_ISL_1454853, EPI_ISL_1454857 to 1454860, EPI_ISL_1454862, EPI_ISL_1454868 to 1454871, EPI_ISL_1454873, EPI_ISL_1454878 to 1454879, EPI_ISL_1454881, EPI_ISL_1454883 to 1454887, EPI_ISL_1454892, EPI_ISL_1454894 to 1454895, EPI_ISL_1454898 to 1454899, EPI_ISL_1454901 to 1454902, EPI_ISL_1454905 to 1454906, EPI_ISL_1454908 to 1454909, EPI_ISL_1454912 to 1454914, EPI_ISL_1454916, EPI_ISL_1454918 to 1454924, EPI_ISL_1454930 to 1454931, EPI_ISL_1454934, EPI_ISL_1454936 to 1454937, EPI_ISL_1454940 to 1454941, EPI_ISL_1454944, EPI_ISL_1454946, EPI_ISL_1454950 to 1454952, EPI_ISL_1454954, EPI_ISL_1454960, EPI_ISL_1454963, EPI_ISL_1454969 to 1454973, EPI_ISL_1454975 to 1454988, EPI_ISL_1454990 to 1454992, EPI_ISL_1455005, EPI_ISL_1455008 to 1455020, EPI_ISL_1455022 to 1455023, EPI_ISL_1455028 to 1455029, EPI_ISL_1455031 to 1455032, EPI_ISL_1455035, EPI_ISL_1455038, EPI_ISL_1455040, EPI_ISL_1455046 to 1455047, EPI_ISL_1455050 to 1455051, EPI_ISL_1455053, EPI_ISL_1455055 to 1455056, EPI_ISL_1455060, EPI_ISL_1455067, EPI_ISL_1455070, EPI_ISL_1455072 to 1455073, EPI_ISL_1455075, EPI_ISL_1455079 to 1455081, EPI_ISL_1455084 to 1455086, EPI_ISL_1455090, EPI_ISL_1455092, EPI_ISL_1455094 to 1455095, EPI_ISL_1455099 to 1455105, EPI_ISL_1455109, EPI_ISL_1455111 to 1455115, EPI_ISL_1455117, EPI_ISL_1455119 to 1455120, EPI_ISL_1455122, EPI_ISL_1455124, EPI_ISL_1455126 to 1455129, EPI_ISL_1455132, EPI_ISL_1455134 to 1455139, EPI_ISL_1455142, EPI_ISL_1455144, EPI_ISL_1455147 to 1455148, EPI_ISL_1455150 to 1455153, EPI_ISL_1455158, EPI_ISL_1455161 to 1455163, EPI_ISL_1455165, EPI_ISL_1455167 to 1455168, EPI_ISL_1455170, EPI_ISL_1455173 to 1455175, EPI_ISL_1455177, EPI_ISL_1455180, EPI_ISL_1455183, EPI_ISL_1455186 to 1455187, EPI_ISL_1455189 to 1455191, EPI_ISL_1455197, EPI_ISL_1455201 to 1455202, EPI_ISL_1455204 to 1455205, EPI_ISL_1455207, EPI_ISL_1455209, EPI_ISL_1455214 to 1455216, EPI_ISL_1455219, EPI_ISL_1455222 to 1455226, EPI_ISL_1455228, EPI_ISL_1455231, EPI_ISL_1455236, EPI_ISL_1455239, EPI_ISL_1455245, EPI_ISL_1455252 to 1455254, EPI_ISL_1455259 to 1455261, EPI_ISL_1455264 to 1455265, EPI_ISL_1455269, EPI_ISL_1455271, EPI_ISL_1455279, EPI_ISL_1455285, EPI_ISL_1455290, EPI_ISL_1455292, EPI_ISL_1455296 to 1455297, EPI_ISL_1455303 to 1455304, EPI_ISL_1455307 to 1455310, EPI_ISL_1455323, EPI_ISL_1455328, EPI_ISL_1455330, EPI_ISL_1455336, EPI_ISL_1455338, EPI_ISL_1455347 to 1455348, EPI_ISL_1455353, EPI_ISL_1455360, EPI_ISL_1455363, EPI_ISL_1455366, EPI_ISL_1455377, EPI_ISL_1455392 to 1455393, EPI_ISL_1455406, EPI_ISL_1455408, EPI_ISL_1455412, EPI_ISL_1455414 to 1455415, EPI_ISL_1455420, EPI_ISL_1455426, EPI_ISL_1455430, EPI_ISL_1455436, EPI_ISL_1455443, EPI_ISL_1455448, EPI_ISL_1455463, EPI_ISL_1455465 to 1455466, EPI_ISL_1455468, EPI_ISL_1455473, EPI_ISL_1455475, EPI_ISL_1455477 to 1455479, EPI_ISL_1455485, EPI_ISL_1455487 to 1455488, EPI_ISL_1455492 to 1455514, EPI_ISL_1455518 to 1455519, EPI_ISL_1455521 to 1455531, EPI_ISL_1455533, EPI_ISL_1455535, EPI_ISL_1455538 to 1455540, EPI_ISL_1455542 to 1455553, EPI_ISL_1455556 to 1455558, EPI_ISL_1455561 to 1455565, EPI_ISL_1455567, EPI_ISL_1455573, EPI_ISL_1455575 to 1455576, EPI_ISL_1455578 to 1455579, EPI_ISL_1455613, EPI_ISL_1455615, EPI_ISL_1455617, EPI_ISL_1455622, EPI_ISL_1455625, EPI_ISL_1455627, EPI_ISL_1455631 to 1455632, EPI_ISL_1455648, EPI_ISL_1466161, EPI_ISL_1466163 to 1466172, EPI_ISL_1466174 to 1466183, EPI_ISL_1466185 to 1466193, EPI_ISL_1466197 to 1466228, EPI_ISL_1466230 to 1466234, EPI_ISL_1466236 to 1466238, EPI_ISL_1466240 to 1466241, EPI_ISL_1466243 to 1466245, EPI_ISL_1466248 to 1466251, EPI_ISL_1466253, EPI_ISL_1466255 to 1466259, EPI_ISL_1466261 to 1466269, EPI_ISL_1466272 to 1466280, EPI_ISL_1466282, EPI_ISL_1466285 to 1466286, EPI_ISL_1466288 to 1466295, EPI_ISL_1466297 to 1466305, EPI_ISL_1466307 to 1466308, EPI_ISL_1466310 to 1466313, EPI_ISL_1466315, EPI_ISL_1466317 to 1466321, EPI_ISL_1466323 to 1466324, EPI_ISL_1466327 to 1466333, EPI_ISL_1466336 to 1466337, EPI_ISL_1466339 to 1466343, EPI_ISL_1466345 to 1466361, EPI_ISL_1466363 to 1466373, EPI_ISL_1466376 to 1466380, EPI_ISL_1466382 to 1466397, EPI_ISL_1466399 to 1466408, EPI_ISL_1466410 to 1466416, EPI_ISL_1466418 to 1466419, EPI_ISL_1466421 to 1466425, EPI_ISL_1466427, EPI_ISL_1466429 to 1466436, EPI_ISL_1466438 to 1466439, EPI_ISL_1466441 to 1466443, EPI_ISL_1466445, EPI_ISL_1466447, EPI_ISL_1466450 to 1466456, EPI_ISL_1466458 to 1466462, EPI_ISL_1466464 to 1466468, EPI_ISL_1466470, EPI_ISL_1466472 to 1466473, EPI_ISL_1466475 to 1466477, EPI_ISL_1466479, EPI_ISL_1466481, EPI_ISL_1466483, EPI_ISL_1466485 to 1466486, EPI_ISL_1466488 to 1466490, EPI_ISL_1466493 to 1466494, EPI_ISL_1466497, EPI_ISL_1466500, EPI_ISL_1466503 to 1466504, EPI_ISL_1466507, EPI_ISL_1466510, EPI_ISL_1466512, EPI_ISL_1466514 to 1466515, EPI_ISL_1466518 to 1466520, EPI_ISL_1466522 to 1466523, EPI_ISL_1466525 to 1466526, EPI_ISL_1466529 to 1466531, EPI_ISL_1466534 to 1466535, EPI_ISL_1466537, EPI_ISL_1466539 to 1466541, EPI_ISL_1466544 to 1466549, EPI_ISL_1466552, EPI_ISL_1466554 to 1466555, EPI_ISL_1466557 to 1466558, EPI_ISL_1466562 to 1466563, EPI_ISL_1466566 to 1466568, EPI_ISL_1466570 to 1466576, EPI_ISL_1466578 to 1466579, EPI_ISL_1466582 to 1466583, EPI_ISL_1466585 to 1466586, EPI_ISL_1466588, EPI_ISL_1466590 to 1466592, EPI_ISL_1466602 to 1466603, EPI_ISL_1466605, EPI_ISL_1466607 to 1466609, EPI_ISL_1466611, EPI_ISL_1466613 to 1466615, EPI_ISL_1466618 to 1466619, EPI_ISL_1466623 to 1466625, EPI_ISL_1466628 to 1466629, EPI_ISL_1466631 to 1466633, EPI_ISL_1466635 to 1466640, EPI_ISL_1466642 to 1466643, EPI_ISL_1466646 to 1466647, EPI_ISL_1466651, EPI_ISL_1466657, EPI_ISL_1466662, EPI_ISL_1466664, EPI_ISL_1466666, EPI_ISL_1466668 to 1466672, EPI_ISL_1466676 to 1466677, EPI_ISL_1466681 to 1466682, EPI_ISL_1466684, EPI_ISL_1466688 to 1466690, EPI_ISL_1466692, EPI_ISL_1466699 to 1466700, EPI_ISL_1466703 to 1466706, EPI_ISL_1466712 to 1466721, EPI_ISL_1466723 to 1466724, EPI_ISL_1466726 to 1466727, EPI_ISL_1466729 to 1466732, EPI_ISL_1466734 to 1466735, EPI_ISL_1466740, EPI_ISL_1466743, EPI_ISL_1466923 to 1466933, EPI_ISL_1466935 to 1466945, EPI_ISL_1466947 to 1466948, EPI_ISL_1466950, EPI_ISL_1466952 to 1466954, EPI_ISL_1466959 to 1466961, EPI_ISL_1466963 to 1466974, EPI_ISL_1466976 to 1466982, EPI_ISL_1466984 to 1466986, EPI_ISL_1466988 to 1466989, EPI_ISL_1466994 to 1466996, EPI_ISL_1466998 to 1466999, EPI_ISL_1467001 to 1467008, EPI_ISL_1467010 to 1467016, EPI_ISL_1467020 to 1467025, EPI_ISL_1467027 to 1467028, EPI_ISL_1467030 to 1467033, EPI_ISL_1467035 to 1467037, EPI_ISL_1467039 to 1467040, EPI_ISL_1467042 to 1467043, EPI_ISL_1467045, EPI_ISL_1467047 to 1467048, EPI_ISL_1467050, EPI_ISL_1467052 to 1467054, EPI_ISL_1467056 to 1467058, EPI_ISL_1467060 to 1467066, EPI_ISL_1467069 to 1467074, EPI_ISL_1467076, EPI_ISL_1467078 to 1467079, EPI_ISL_1467081 to 1467087, EPI_ISL_1467089 to 1467097, EPI_ISL_1467099 to 1467112, EPI_ISL_1467114, EPI_ISL_1467116 to 1467121, EPI_ISL_1467123 to 1467127, EPI_ISL_1467129 to 1467130, EPI_ISL_1467132 to 1467133, EPI_ISL_1467135 to 1467137, EPI_ISL_1467141 to 1467146, EPI_ISL_1467148 to 1467166, EPI_ISL_1467168 to 1467169, EPI_ISL_1467172 to 1467179, EPI_ISL_1467181, EPI_ISL_1467183 to 1467186, EPI_ISL_1467189, EPI_ISL_1467191 to 1467205, EPI_ISL_1467207, EPI_ISL_1467209 to 1467228, EPI_ISL_1467232, EPI_ISL_1467234 to 1467235, EPI_ISL_1467237 to 1467238, EPI_ISL_1467240 to 1467251, EPI_ISL_1467254 to 1467257, EPI_ISL_1467262, EPI_ISL_1467265, EPI_ISL_1467268, EPI_ISL_1467270 to 1467273, EPI_ISL_1467278, EPI_ISL_1467281, EPI_ISL_1467285 to 1467286, EPI_ISL_1467289, EPI_ISL_1467294, EPI_ISL_1467299, EPI_ISL_1467302, EPI_ISL_1467308 to 1467309, EPI_ISL_1467312, EPI_ISL_1467315, EPI_ISL_1467318, EPI_ISL_1467322 to 1467323, EPI_ISL_1467329, EPI_ISL_1467331 to 1467332, EPI_ISL_1467334, EPI_ISL_1467350, EPI_ISL_1467355, EPI_ISL_1467366, EPI_ISL_1467369, EPI_ISL_1467373, EPI_ISL_1467379 to 1467380, EPI_ISL_1467383, EPI_ISL_1467386, EPI_ISL_1467388, EPI_ISL_1467397, EPI_ISL_1467401, EPI_ISL_1467406, EPI_ISL_1467408 to 1467409, EPI_ISL_1467412, EPI_ISL_1467417, EPI_ISL_1467428 to 1467431, EPI_ISL_1467434, EPI_ISL_1467437, EPI_ISL_1467444, EPI_ISL_1467451, EPI_ISL_1467463, EPI_ISL_1467467 to 1467468, EPI_ISL_1467470 to 1467471, EPI_ISL_1467475, EPI_ISL_1467479, EPI_ISL_1467481, EPI_ISL_1467486 to 1467487, EPI_ISL_1467491, EPI_ISL_1467493, EPI_ISL_1467499, EPI_ISL_1467510, EPI_ISL_1467517, EPI_ISL_1467525, EPI_ISL_1467531, EPI_ISL_1467534 to 1467535, EPI_ISL_1467538, EPI_ISL_1467545 to 1467546, EPI_ISL_1467549, EPI_ISL_1467553 to 1467554, EPI_ISL_1467563, EPI_ISL_1467565, EPI_ISL_1467567 to 1467568, EPI_ISL_1467576, EPI_ISL_1467804 to 1467834, EPI_ISL_1467839, EPI_ISL_1467842, EPI_ISL_1467846, EPI_ISL_1467848 to 1467851, EPI_ISL_1467855 to 1467856, EPI_ISL_1467861, EPI_ISL_1467863 to 1467867, EPI_ISL_1467869 to 1467873, EPI_ISL_1467878, EPI_ISL_1467882 to 1467884, EPI_ISL_1467890 to 1467891, EPI_ISL_1467893 to 1467896, EPI_ISL_1467901 to 1467904, EPI_ISL_1467906 to 1467907, EPI_ISL_1467909 to 1467912, EPI_ISL_1467915 to 1467916, EPI_ISL_1467920, EPI_ISL_1467922 to 1467923, EPI_ISL_1467931, EPI_ISL_1467934 to 1467936, EPI_ISL_1467938, EPI_ISL_1467940, EPI_ISL_1467942, EPI_ISL_1467944 to 1467945, EPI_ISL_1467950 to 1467951, EPI_ISL_1467954, EPI_ISL_1467958 to 1467962, EPI_ISL_1467964, EPI_ISL_1467971, EPI_ISL_1467974, EPI_ISL_1467976 to 1467977, EPI_ISL_1467982 to 1467985, EPI_ISL_1467988 to 1467990, EPI_ISL_1467992, EPI_ISL_1467997 to 1467998, EPI_ISL_1468003 to 1468005, EPI_ISL_1468011, EPI_ISL_1468013, EPI_ISL_1468015, EPI_ISL_1468018, EPI_ISL_1468020 to 1468021, EPI_ISL_1468023 to 1468024, EPI_ISL_1468027, EPI_ISL_1468030, EPI_ISL_1468033 to 1468036, EPI_ISL_1468038 to 1468040, EPI_ISL_1468042, EPI_ISL_1468044, EPI_ISL_1468046 to 1468047, EPI_ISL_1468050, EPI_ISL_1468054, EPI_ISL_1468057 to 1468058, EPI_ISL_1468061, EPI_ISL_1468064, EPI_ISL_1468067 to 1468068, EPI_ISL_1468070, EPI_ISL_1468072, EPI_ISL_1468078 to 1468079, EPI_ISL_1468082 to 1468083, EPI_ISL_1468086, EPI_ISL_1468090, EPI_ISL_1468092 to 1468093, EPI_ISL_1468101, EPI_ISL_1468107 to 1468108, EPI_ISL_1468111, EPI_ISL_1468117, EPI_ISL_1468119, EPI_ISL_1468124, EPI_ISL_1468127 to 1468129, EPI_ISL_1468132 to 1468133, EPI_ISL_1468135, EPI_ISL_1468138, EPI_ISL_1468140 to 1468143, EPI_ISL_1468145, EPI_ISL_1468148 to 1468149, EPI_ISL_1468151 to 1468155, EPI_ISL_1468158, EPI_ISL_1468160 to 1468164, EPI_ISL_1468167 to 1468169, EPI_ISL_1468171, EPI_ISL_1468175, EPI_ISL_1468177, EPI_ISL_1468179 to 1468180, EPI_ISL_1468182 to 1468184, EPI_ISL_1468186 to 1468192, EPI_ISL_1468194 to 1468195, EPI_ISL_1468197 to 1468200, EPI_ISL_1468202 to 1468206, EPI_ISL_1468209, EPI_ISL_1468211 to 1468212, EPI_ISL_1468214, EPI_ISL_1468216 to 1468217, EPI_ISL_1468219, EPI_ISL_1468222 to 1468231, EPI_ISL_1468233, EPI_ISL_1468238 to 1468240, EPI_ISL_1468242 to 1468245, EPI_ISL_1468247, EPI_ISL_1468250 to 1468255, EPI_ISL_1468257 to 1468260, EPI_ISL_1468262 to 1468265, EPI_ISL_1468272 to 1468274, EPI_ISL_1468276, EPI_ISL_1468279 to 1468281, EPI_ISL_1468283 to 1468286,

EPI_ISL_1468288 to 1468291, EPI_ISL_1468293 to 1468295, EPI_ISL_1468298 to 1468302, EPI_ISL_1468307 to 1468308, EPI_ISL_1468310 to 1468312, EPI_ISL_1468315 to 1468317, EPI_ISL_1468320, EPI_ISL_1468322 to 1468324, EPI_ISL_1468326 to 1468328, EPI_ISL_1468330, EPI_ISL_1468332 to 1468333, EPI_ISL_1468335, EPI_ISL_1468337 to 1468338, EPI_ISL_1468340, EPI_ISL_1468343, EPI_ISL_1468346 to 1468351, EPI_ISL_1468356 to 1468357, EPI_ISL_1468359 to 1468363, EPI_ISL_1468365, EPI_ISL_1468368 to 1468369, EPI_ISL_1468372 to 1468373, EPI_ISL_1468375, EPI_ISL_1468377 to 1468381, EPI_ISL_1468383, EPI_ISL_1468385, EPI_ISL_1468387, EPI_ISL_1468389 to 1468393, EPI_ISL_1468395 to 1468400, EPI_ISL_1468403, EPI_ISL_1472409, EPI_ISL_1472412 to 1472413, EPI_ISL_1472416 to 1472417, EPI_ISL_1472419 to 1472420, EPI_ISL_1472423 to 1472425, EPI_ISL_1472429 to 1472430, EPI_ISL_1472432 to 1472433, EPI_ISL_1472437 to 1472440, EPI_ISL_1472444 to 1472445, EPI_ISL_1472447, EPI_ISL_1472449 to 1472450, EPI_ISL_1472457, EPI_ISL_1472459, EPI_ISL_1472461, EPI_ISL_1472466 to 1472467, EPI_ISL_1472469 to 1472473, EPI_ISL_1472475 to 1472477, EPI_ISL_1472479 to 1472480, EPI_ISL_1472482, EPI_ISL_1472484, EPI_ISL_1472486 to 1472491, EPI_ISL_1472493, EPI_ISL_1472495, EPI_ISL_1472497 to 1472501, EPI_ISL_1472503 to 1472507, EPI_ISL_1472509 to 1472510, EPI_ISL_1472514 to 1472525, EPI_ISL_1472529, EPI_ISL_1472531, EPI_ISL_1472534, EPI_ISL_1472537 to 1472546, EPI_ISL_1472548, EPI_ISL_1472550, EPI_ISL_1472553, EPI_ISL_1472555, EPI_ISL_1472559, EPI_ISL_1472561, EPI_ISL_1472563 to 1472567, EPI_ISL_1472570 to 1472571, EPI_ISL_1472573 to 1472577, EPI_ISL_1472581, EPI_ISL_1472585 to 1472592, EPI_ISL_1472595, EPI_ISL_1472598 to 1472599, EPI_ISL_1472601 to 1472603, EPI_ISL_1472607, EPI_ISL_1472609 to 1472610, EPI_ISL_1472614 to 1472616, EPI_ISL_1472618, EPI_ISL_1472620, EPI_ISL_1472623 to 1472624, EPI_ISL_1472630, EPI_ISL_1472632, EPI_ISL_1472635 to 1472637, EPI_ISL_1472639 to 1472642, EPI_ISL_1472645, EPI_ISL_1472648 to 1472649, EPI_ISL_1472651, EPI_ISL_1472654, EPI_ISL_1472659, EPI_ISL_1472666, EPI_ISL_1472671, EPI_ISL_1472673 to 1472675, EPI_ISL_1472678 to 1472679, EPI_ISL_1472682 to 1472683, EPI_ISL_1472685, EPI_ISL_1472687 to 1472691, EPI_ISL_1472693 to 1472695, EPI_ISL_1472699 to 1472700, EPI_ISL_1472706 to 1472707, EPI_ISL_1472715, EPI_ISL_1472727, EPI_ISL_1472742, EPI_ISL_1472756, EPI_ISL_1472766, EPI_ISL_1472769, EPI_ISL_1472777 to 1472778, EPI_ISL_1472780, EPI_ISL_1472786, EPI_ISL_1472797, EPI_ISL_1472809, EPI_ISL_1472819, EPI_ISL_1472830, EPI_ISL_1472840, EPI_ISL_1472867, EPI_ISL_1472869 to 1472870, EPI_ISL_1472876, EPI_ISL_1472880, EPI_ISL_1472898, EPI_ISL_1472911, EPI_ISL_1472925, EPI_ISL_1472929, EPI_ISL_1472931 to 1472932, EPI_ISL_1472943 to 1472944, EPI_ISL_1473803 to 1473804, EPI_ISL_1473807 to 1473809, EPI_ISL_1473812 to 1474155, EPI_ISL_1474158 to 1474160, EPI_ISL_1483803, EPI_ISL_1483809, EPI_ISL_1483812, EPI_ISL_1483815 to 1483816, EPI_ISL_1483840, EPI_ISL_1483846, EPI_ISL_1483848, EPI_ISL_1483850, EPI_ISL_1483863 to 1483865, EPI_ISL_1483869, EPI_ISL_1483879, EPI_ISL_1483893, EPI_ISL_1483908 to 1483909, EPI_ISL_1483913 to 1483914, EPI_ISL_1483921, EPI_ISL_1483924, EPI_ISL_1483940, EPI_ISL_1483942 to 1483944, EPI_ISL_1483947 to 1483948, EPI_ISL_1483950, EPI_ISL_1483952, EPI_ISL_1483956, EPI_ISL_1483958, EPI_ISL_1483961, EPI_ISL_1483966 to 1483967, EPI_ISL_1483969, EPI_ISL_1483973, EPI_ISL_1483975 to 1483976, EPI_ISL_1483978, EPI_ISL_1483980, EPI_ISL_1483982, EPI_ISL_1483985, EPI_ISL_1483990 to 1483994, EPI_ISL_1483996, EPI_ISL_1483998, EPI_ISL_1484002, EPI_ISL_1484007, EPI_ISL_1484012, EPI_ISL_1484017, EPI_ISL_1484022, EPI_ISL_1484026, EPI_ISL_1484030, EPI_ISL_1484033, EPI_ISL_1484037, EPI_ISL_1484039, EPI_ISL_1484052, EPI_ISL_1484055, EPI_ISL_1484061, EPI_ISL_1484066 to 1484067, EPI_ISL_1484072, EPI_ISL_1484077, EPI_ISL_1484090 to 1484091, EPI_ISL_1484094 to 1484095, EPI_ISL_1484097 to 1484098, EPI_ISL_1484100, EPI_ISL_1484106, EPI_ISL_1484112 to 1484115, EPI_ISL_1484119, EPI_ISL_1484122, EPI_ISL_1484124 to 1484125, EPI_ISL_1484127, EPI_ISL_1484129 to 1484130, EPI_ISL_1484134 to 1484135, EPI_ISL_1484149, EPI_ISL_1484155, EPI_ISL_1484160, EPI_ISL_1484165, EPI_ISL_1484169, EPI_ISL_1484172, EPI_ISL_1484180, EPI_ISL_1484186 to 1484188, EPI_ISL_1484190 to 1484191, EPI_ISL_1484193 to 1484195, EPI_ISL_1484198 to 1484199, EPI_ISL_1484204, EPI_ISL_1484207, EPI_ISL_1484213 to 1484219, EPI_ISL_1484221 to 1484222, EPI_ISL_1484227 to 1484229, EPI_ISL_1484246, EPI_ISL_1484252, EPI_ISL_1484254, EPI_ISL_1484256 to 1484257, EPI_ISL_1484264 to 1484265, EPI_ISL_1484269 to 1484271, EPI_ISL_1484275, EPI_ISL_1484283, EPI_ISL_1484289, EPI_ISL_1484297, EPI_ISL_1484299, EPI_ISL_1484302, EPI_ISL_1484308, EPI_ISL_1484311, EPI_ISL_1484315, EPI_ISL_1484321, EPI_ISL_1484326, EPI_ISL_1484329, EPI_ISL_1484334, EPI_ISL_1484348, EPI_ISL_1484351, EPI_ISL_1484355, EPI_ISL_1484359, EPI_ISL_1484363 to 1484364, EPI_ISL_1484383, EPI_ISL_1484390, EPI_ISL_1484396, EPI_ISL_1484399, EPI_ISL_1484402, EPI_ISL_1484405, EPI_ISL_1484407 to 1484408, EPI_ISL_1484419 to 1484420, EPI_ISL_1484423, EPI_ISL_1484425, EPI_ISL_1484429, EPI_ISL_1484432, EPI_ISL_1484437, EPI_ISL_1484439, EPI_ISL_1484442, EPI_ISL_1484456, EPI_ISL_1484461, EPI_ISL_1484463 to 1484464, EPI_ISL_1484468 to 1484470, EPI_ISL_1484475, EPI_ISL_1484489, EPI_ISL_1484492, EPI_ISL_1484495, EPI_ISL_1484506 to 1484508, EPI_ISL_1484512, EPI_ISL_1484514, EPI_ISL_1484521, EPI_ISL_1484524, EPI_ISL_1484526, EPI_ISL_1484529 to 1484530, EPI_ISL_1484536, EPI_ISL_1484541, EPI_ISL_1484543, EPI_ISL_1484563, EPI_ISL_1484565, EPI_ISL_1484569, EPI_ISL_1484577, EPI_ISL_1484580 to 1484581, EPI_ISL_1484584 to 1484585, EPI_ISL_1484589, EPI_ISL_1484605, EPI_ISL_1484608, EPI_ISL_1484610, EPI_ISL_1484612 to 1484613, EPI_ISL_1484616, EPI_ISL_1484618, EPI_ISL_1484620, EPI_ISL_1484622 to 1484626, EPI_ISL_1484628, EPI_ISL_1484630, EPI_ISL_1484632, EPI_ISL_1484635 to 1484636, EPI_ISL_1484638 to 1484640, EPI_ISL_1484642 to 1484644, EPI_ISL_1484646 to 1484647, EPI_ISL_1484652 to 1484653, EPI_ISL_1484655, EPI_ISL_1484657, EPI_ISL_1484660 to 1484664, EPI_ISL_1484666, EPI_ISL_1484668 to 1484674, EPI_ISL_1484676 to 1484679, EPI_ISL_1484681 to 1484684, EPI_ISL_1484686, EPI_ISL_1484688 to 1484690, EPI_ISL_1484692 to 1484697, EPI_ISL_1484699 to 1484705, EPI_ISL_1484707, EPI_ISL_1484709, EPI_ISL_1484711, EPI_ISL_1484713 to 1484716, EPI_ISL_1484721 to 1484727, EPI_ISL_1484729 to 1484732, EPI_ISL_1484734 to 1484738, EPI_ISL_1484740 to 1484746, EPI_ISL_1484748 to 1484753, EPI_ISL_1484755 to 1484758, EPI_ISL_1484760 to 1484761, EPI_ISL_1484763, EPI_ISL_1484766 to 1484770, EPI_ISL_1484774 to 1484780, EPI_ISL_1484782 to 1484787, EPI_ISL_1484790 to 1484793, EPI_ISL_1484796, EPI_ISL_1484798 to 1484799, EPI_ISL_1484801 to 1484802, EPI_ISL_1484804 to 1484806, EPI_ISL_1484808 to 1484812, EPI_ISL_1484814, EPI_ISL_1484816 to 1484817, EPI_ISL_1484819 to 1484826, EPI_ISL_1484828 to 1484829, EPI_ISL_1484831 to 1484833, EPI_ISL_1484835, EPI_ISL_1484837 to 1484841, EPI_ISL_1484843 to 1484853, EPI_ISL_1484855 to 1484856, EPI_ISL_1484858 to 1484862, EPI_ISL_1484864 to 1484866, EPI_ISL_1484868 to 1484871, EPI_ISL_1484873 to 1484882, EPI_ISL_1484884 to 1484890, EPI_ISL_1484892 to 1484893, EPI_ISL_1484897 to 1484901, EPI_ISL_1484903 to 1484910, EPI_ISL_1484912, EPI_ISL_1484914 to 1484918, EPI_ISL_1484920 to 1484921, EPI_ISL_1484924, EPI_ISL_1484926, EPI_ISL_1484928 to 1484934, EPI_ISL_1484936 to 1484937, EPI_ISL_1484939 to 1484941, EPI_ISL_1484943 to 1484947, EPI_ISL_1484949 to 1484951, EPI_ISL_1484954, EPI_ISL_1484956, EPI_ISL_1484959, EPI_ISL_1484962 to 1484972, EPI_ISL_1484974 to 1484983, EPI_ISL_1484987 to 1484990, EPI_ISL_1484992 to 1484997, EPI_ISL_1484999 to 1485006, EPI_ISL_1485008, EPI_ISL_1485010 to 1485017, EPI_ISL_1485020 to 1485025, EPI_ISL_1485027 to 1485041, EPI_ISL_1485043, EPI_ISL_1485045 to 1485047, EPI_ISL_1485049, EPI_ISL_1485051 to 1485055, EPI_ISL_1485057, EPI_ISL_1485059 to 1485066, EPI_ISL_1485068 to 1485075, EPI_ISL_1485077 to 1485078, EPI_ISL_1485081, EPI_ISL_1485083, EPI_ISL_1485085 to 1485092, EPI_ISL_1485094 to 1485095, EPI_ISL_1485097, EPI_ISL_1485099 to 1485100, EPI_ISL_1485102 to 1485105, EPI_ISL_1485108 to 1485111, EPI_ISL_1485113 to 1485120, EPI_ISL_1485122 to 1485125, EPI_ISL_1485127 to 1485129, EPI_ISL_1485131, EPI_ISL_1485135 to 1485137, EPI_ISL_1485139 to 1485140, EPI_ISL_1485142 to 1485144, EPI_ISL_1485146, EPI_ISL_1485148 to 1485165, EPI_ISL_1485167 to 1485174, EPI_ISL_1485176 to 1485181, EPI_ISL_1485185 to 1485190, EPI_ISL_1485192 to 1485194, EPI_ISL_1485197 to 1485198, EPI_ISL_1485200 to 1485203, EPI_ISL_1485205 to 1485210, EPI_ISL_1485212, EPI_ISL_1485215, EPI_ISL_1485217, EPI_ISL_1485219 to 1485223, EPI_ISL_1485225, EPI_ISL_1485229, EPI_ISL_1485231 to 1485233, EPI_ISL_1485235 to 1485236, EPI_ISL_1485239, EPI_ISL_1485242 to 1485248, EPI_ISL_1485250 to 1485251, EPI_ISL_1485253 to 1485259, EPI_ISL_1485261, EPI_ISL_1485265, EPI_ISL_1485267 to 1485269, EPI_ISL_1485271 to 1485272, EPI_ISL_1485274 to 1485275, EPI_ISL_1485277 to 1485289, EPI_ISL_1485291, EPI_ISL_1485293 to 1485297, EPI_ISL_1485299, EPI_ISL_1485302, EPI_ISL_1485304 to 1485306, EPI_ISL_1485308 to 1485312, EPI_ISL_1485314, EPI_ISL_1485316 to 1485320, EPI_ISL_1485322 to 1485324, EPI_ISL_1485330 to 1485331, EPI_ISL_1485337 to 1485342, EPI_ISL_1485345 to 1485346, EPI_ISL_1485349 to 1485350, EPI_ISL_1485352 to 1485354, EPI_ISL_1485356 to 1485360, EPI_ISL_1485362, EPI_ISL_1485364 to 1485366, EPI_ISL_1485368 to 1485370, EPI_ISL_1485373 to 1485374, EPI_ISL_1485376 to 1485377, EPI_ISL_1485380, EPI_ISL_1485383 to 1485384, EPI_ISL_1485386 to 1485387, EPI_ISL_1485389 to 1485394, EPI_ISL_1485396 to 1485397, EPI_ISL_1485400 to 1485405, EPI_ISL_1485407 to 1485411, EPI_ISL_1485413, EPI_ISL_1485415 to 1485417, EPI_ISL_1485420 to 1485421, EPI_ISL_1485427, EPI_ISL_1485430 to 1485432, EPI_ISL_1485434 to 1485437, EPI_ISL_1485439 to 1485446, EPI_ISL_1485448 to 1485454, EPI_ISL_1485459, EPI_ISL_1485462 to 1485466, EPI_ISL_1485468 to 1485469, EPI_ISL_1485471, EPI_ISL_1485473 to 1485474, EPI_ISL_1485476 to 1485480, EPI_ISL_1485483 to 1485486, EPI_ISL_1485488 to 1485492, EPI_ISL_1485494 to 1485495, EPI_ISL_1485497, EPI_ISL_1485499 to 1485500, EPI_ISL_1485502 to 1485503, EPI_ISL_1485505 to 1485507, EPI_ISL_1485509 to 1485523, EPI_ISL_1485525 to 1485529, EPI_ISL_1485532 to 1485535, EPI_ISL_1485537 to 1485543, EPI_ISL_1485545 to 1485555, EPI_ISL_1485557 to 1485561, EPI_ISL_1485564 to 1485573, EPI_ISL_1485575 to 1485577, EPI_ISL_1485579 to 1485583, EPI_ISL_1485585 to 1485590, EPI_ISL_1485592, EPI_ISL_1485595 to 1485598, EPI_ISL_1485604 to 1485605, EPI_ISL_1485607 to 1485608, EPI_ISL_1485610 to 1485612, EPI_ISL_1485615 to 1485622, EPI_ISL_1485624, EPI_ISL_1485626 to 1485627, EPI_ISL_1485629, EPI_ISL_1485631 to 1485634, EPI_ISL_1485636 to 1485640, EPI_ISL_1485643 to 1485645, EPI_ISL_1485647 to 1485648, EPI_ISL_1485650, EPI_ISL_1485652, EPI_ISL_1485654 to 1485657, EPI_ISL_1485660 to 1485669, EPI_ISL_1485671 to 1485678, EPI_ISL_1485680, EPI_ISL_1485682 to 1485683, EPI_ISL_1485685 to 1485693, EPI_ISL_1485696 to 1485698, EPI_ISL_1485700, EPI_ISL_1485702, EPI_ISL_1485704 to 1485710, EPI_ISL_1485715 to 1485722, EPI_ISL_1485724 to 1485726, EPI_ISL_1485729 to 1485730, EPI_ISL_1485732, EPI_ISL_1485734 to 1485739, EPI_ISL_1485741 to 1485748, EPI_ISL_1485750, EPI_ISL_1485752, EPI_ISL_1485754, EPI_ISL_1485757, EPI_ISL_1485760, EPI_ISL_1485762, EPI_ISL_1485765 to 1485770, EPI_ISL_1485772 to 1485773, EPI_ISL_1485775 to 1485788, EPI_ISL_1485791, EPI_ISL_1485795 to 1485801, EPI_ISL_1485803 to 1485809, EPI_ISL_1485811 to 1485812, EPI_ISL_1485814, EPI_ISL_1485816 to 1485831, EPI_ISL_1485833 to 1485834, EPI_ISL_1485836 to 1485855, EPI_ISL_1485857 to 1485862, EPI_ISL_1485864 to 1485865, EPI_ISL_1485867 to 1485870, EPI_ISL_1485873 to 1485875, EPI_ISL_1485877 to 1485878, EPI_ISL_1485880 to 1485891, EPI_ISL_1485893 to 1485897, EPI_ISL_1485899 to 1485909, EPI_ISL_1485911 to 1485935, EPI_ISL_1485937 to 1485946, EPI_ISL_1485948 to 1485961, EPI_ISL_1485963 to 1485965, EPI_ISL_1485967 to 1485972, EPI_ISL_1485974 to 1485984, EPI_ISL_1485986 to 1485991, EPI_ISL_1485993 to 1485998, EPI_ISL_1486000 to 1486011, EPI_ISL_1486013 to 1486023, EPI_ISL_1486025 to 1486027, EPI_ISL_1486029, EPI_ISL_1486031 to 1486044, EPI_ISL_1486046 to 1486053, EPI_ISL_1486055 to 1486057, EPI_ISL_1486061 to 1486064, EPI_ISL_1486066, EPI_ISL_1486068 to 1486070, EPI_ISL_1486072 to 1486074, EPI_ISL_1486076 to 1486078, EPI_ISL_1486080 to 1486086, EPI_ISL_1486088 to 1486090, EPI_ISL_1486093 to 1486096, EPI_ISL_1486098 to 1486106, EPI_ISL_1486108 to 1486112, EPI_ISL_1486114 to 1486116, EPI_ISL_1486119, EPI_ISL_1486121 to 1486123, EPI_ISL_1486126, EPI_ISL_1486128 to 1486131, EPI_ISL_1486133 to 1486134, EPI_ISL_1486136 to 1486138, EPI_ISL_1486143, EPI_ISL_1486145 to 1486148, EPI_ISL_1486150 to 1486151, EPI_ISL_1486154 to 1486155, EPI_ISL_1486157 to 1486158, EPI_ISL_1486161 to 1486165, EPI_ISL_1486167, EPI_ISL_1486171, EPI_ISL_1486174, EPI_ISL_1486177 to 1486178, EPI_ISL_1486180, EPI_ISL_1486183 to 1486188, EPI_ISL_1486193 to 1486197, EPI_ISL_1486200 to 1486203, EPI_ISL_1486207 to 1486210, EPI_ISL_1486212, EPI_ISL_1486214 to 1486217, EPI_ISL_1486219 to 1486221, EPI_ISL_1486223 to 1486225, EPI_ISL_1486227 to 1486228, EPI_ISL_1486230 to 1486232, EPI_ISL_1486235 to 1486236, EPI_ISL_1486239 to 1486241, EPI_ISL_1486243 to 1486244, EPI_ISL_1486247, EPI_ISL_1486249, EPI_ISL_1486251 to 1486252, EPI_ISL_1486255 to 1486258, EPI_ISL_1486260 to 1486261, EPI_ISL_1486264 to 1486269, EPI_ISL_1486273 to 1486274, EPI_ISL_1486278, EPI_ISL_1486280 to 1486284, EPI_ISL_1486286 to 1486287, EPI_ISL_1486289 to 1486290, EPI_ISL_1486294 to 1486296, EPI_ISL_1486302 to 1486304, EPI_ISL_1486308 to 1486310, EPI_ISL_1486312, EPI_ISL_1486314 to 1486316, EPI_ISL_1486318, EPI_ISL_1486320 to 1486325, EPI_ISL_1486328, EPI_ISL_1486331 to 1486333, EPI_ISL_1486335 to 1486340, EPI_ISL_1486342 to 1486343, EPI_ISL_1486345 to 1486347, EPI_ISL_1486349, EPI_ISL_1486352 to 1486358, EPI_ISL_1486360, EPI_ISL_1486362 to 1486366, EPI_ISL_1486368, EPI_ISL_1486370 to 1486371, EPI_ISL_1486373 to 1486374, EPI_ISL_1486377 to 1486378, EPI_ISL_1486380, EPI_ISL_1486382, EPI_ISL_1486384, EPI_ISL_1486386 to 1486387, EPI_ISL_1486389 to 1486390, EPI_ISL_1486393 to 1486398, EPI_ISL_1486401 to 1486402, EPI_ISL_1486405, EPI_ISL_1486409, EPI_ISL_1486411 to 1486412, EPI_ISL_1486415, EPI_ISL_1486418, EPI_ISL_1486421, EPI_ISL_1486424 to 1486428, EPI_ISL_1486430 to 1486431, EPI_ISL_1486434, EPI_ISL_1486436, EPI_ISL_1486439 to 1486443, EPI_ISL_1486448 to 1486449, EPI_ISL_1486451 to 1486452, EPI_ISL_1486455, EPI_ISL_1486458 to 1486459, EPI_ISL_1486465, EPI_ISL_1486470, EPI_ISL_1486472, EPI_ISL_1486474 to 1486477, EPI_ISL_1486481, EPI_ISL_1486483 to 1486484, EPI_ISL_1486490, EPI_ISL_1486493 to 1486494, EPI_ISL_1486497, EPI_ISL_1486505, EPI_ISL_1486507, EPI_ISL_1486513, EPI_ISL_1486515, EPI_ISL_1486520 to 1486521, EPI_ISL_1486526, EPI_ISL_1486529 to 1486530, EPI_ISL_1486533, EPI_ISL_1486538, EPI_ISL_1486542, EPI_ISL_1486554, EPI_ISL_1486556 to 1486557, EPI_ISL_1486559 to 1486560, EPI_ISL_1486566, EPI_ISL_1486570, EPI_ISL_1486579, EPI_ISL_1486581 to 1486582, EPI_ISL_1486588, EPI_ISL_1486591 to 1486592, EPI_ISL_1486594, EPI_ISL_1486597 to 1486598, EPI_ISL_1486600 to 1486601, EPI_ISL_1486603, EPI_ISL_1486611, EPI_ISL_1486617 to 1486618, EPI_ISL_1486620 to 1486621, EPI_ISL_1486623, EPI_ISL_1486629 to 1486630, EPI_ISL_1486633, EPI_ISL_1486635 to 1486636, EPI_ISL_1486638 to 1486639, EPI_ISL_1486646, EPI_ISL_1486650, EPI_ISL_1486654, EPI_ISL_1486663 to 1486664, EPI_ISL_1486666 to 1486667, EPI_ISL_1486670, EPI_ISL_1486672, EPI_ISL_1486679, EPI_ISL_1486683, EPI_ISL_1486688 to 1486690, EPI_ISL_1486692, EPI_ISL_1486697, EPI_ISL_1486700 to 1486701, EPI_ISL_1486708, EPI_ISL_1486711, EPI_ISL_1486715, EPI_ISL_1486717, EPI_ISL_1486720 to 1486721, EPI_ISL_1486723, EPI_ISL_1486725, EPI_ISL_1486728 to 1486729, EPI_ISL_1486734 to 1486735, EPI_ISL_1486738, EPI_ISL_1486742, EPI_ISL_1486750, EPI_ISL_1486752, EPI_ISL_1486754, EPI_ISL_1486759, EPI_ISL_1486761 to 1486762, EPI_ISL_1486765, EPI_ISL_1486780, EPI_ISL_1486786, EPI_ISL_1486790, EPI_ISL_1486792, EPI_ISL_1486794 to 1486795, EPI_ISL_1486797, EPI_ISL_1486804, EPI_ISL_1486807 to 1486811, EPI_ISL_1486813 to 1486814, EPI_ISL_1486816 to 1486817, EPI_ISL_1486819 to 1486824, EPI_ISL_1486826 to 1486831, EPI_ISL_1486833 to 1486835, EPI_ISL_1486837 to 1486838, EPI_ISL_1486841 to 1486856, EPI_ISL_1486858 to 1486865, EPI_ISL_1486867, EPI_ISL_1486869 to 1486871, EPI_ISL_1486874 to 1486875, EPI_ISL_1486877 to 1486879, EPI_ISL_1486881 to 1486883, EPI_ISL_1486886 to 1486889, EPI_ISL_1486892 to 1486895, EPI_ISL_1486897 to 1486898, EPI_ISL_1486900 to 1486912, EPI_ISL_1486914 to 1486915, EPI_ISL_1486917 to 1486918, EPI_ISL_1486920 to 1486924, EPI_ISL_1486927 to 1486932, EPI_ISL_1486934 to 1486936, EPI_ISL_1486938, EPI_ISL_1486940 to 1486943, EPI_ISL_1486945 to 1486950, EPI_ISL_1486954 to 1486955, EPI_ISL_1486957 to 1486958, EPI_ISL_1486961 to 1486963, EPI_ISL_1486966 to 1486967, EPI_ISL_1486969 to 1486976, EPI_ISL_1486978 to 1486981, EPI_ISL_1486983 to 1486986, EPI_ISL_1486988, EPI_ISL_1486990 to 1486992, EPI_ISL_1486995, EPI_ISL_1486998, EPI_ISL_1487002, EPI_ISL_1487004, EPI_ISL_1487006 to 1487012, EPI_ISL_1487014 to 1487015, EPI_ISL_1487018 to 1487021, EPI_ISL_1487023, EPI_ISL_1487025 to 1487031, EPI_ISL_1487033, EPI_ISL_1487035, EPI_ISL_1487038 to 1487041, EPI_ISL_1487043, EPI_ISL_1487046 to 1487051, EPI_ISL_1487053 to 1487055, EPI_ISL_1487057 to 1487059, EPI_ISL_1487061, EPI_ISL_1487064 to 1487067, EPI_ISL_1487069, EPI_ISL_1487071, EPI_ISL_1487073, EPI_ISL_1487075 to 1487076, EPI_ISL_1487078 to 1487082, EPI_ISL_1487084 to 1487085, EPI_ISL_1487087 to 1487088, EPI_ISL_1487091 to 1487093, EPI_ISL_1487097, EPI_ISL_1487099, EPI_ISL_1487101 to 1487102, EPI_ISL_1487104 to 1487105, EPI_ISL_1487108 to 1487113, EPI_ISL_1487115 to 1487116, EPI_ISL_1487119 to 1487122, EPI_ISL_1487128, EPI_ISL_1487130, EPI_ISL_1487133 to 1487134, EPI_ISL_1487137 to 1487142, EPI_ISL_1487144 to 1487145, EPI_ISL_1487147 to 1487148, EPI_ISL_1487150, EPI_ISL_1487152 to 1487158, EPI_ISL_1487378 to 1487387, EPI_ISL_1487389 to 1487392, EPI_ISL_1487395, EPI_ISL_1487397 to 1487398, EPI_ISL_1487400 to 1487401, EPI_ISL_1487403 to 1487404, EPI_ISL_1487406 to 1487414, EPI_ISL_1487417 to 1487422, EPI_ISL_1487425 to 1487432, EPI_ISL_1487436, EPI_ISL_1487438 to 1487439, EPI_ISL_1487442 to 1487445, EPI_ISL_1487447 to 1487451, EPI_ISL_1487454 to 1487456, EPI_ISL_1487459 to 1487461, EPI_ISL_1487463, EPI_ISL_1487465 to 1487467, EPI_ISL_1487469 to 1487473, EPI_ISL_1487476, EPI_ISL_1487478, EPI_ISL_1487481 to 1487484, EPI_ISL_1487486 to 1487489, EPI_ISL_1487492 to 1487495, EPI_ISL_1487498, EPI_ISL_1487500, EPI_ISL_1487503 to 1487506, EPI_ISL_1487508 to 1487510, EPI_ISL_1487512 to 1487517, EPI_ISL_1487519 to 1487520, EPI_ISL_1487523 to 1487524, EPI_ISL_1487527 to 1487529, EPI_ISL_1487532 to 1487536, EPI_ISL_1487538, EPI_ISL_1487540, EPI_ISL_1487542 to 1487545, EPI_ISL_1487548, EPI_ISL_1487553 to 1487561, EPI_ISL_1487563 to 1487565, EPI_ISL_1487567 to 1487571, EPI_ISL_1487574 to 1487576, EPI_ISL_1487578 to 1487580, EPI_ISL_1487582 to 1487584, EPI_ISL_1487586 to 1487588, EPI_ISL_1487590 to 1487591, EPI_ISL_1487593 to 1487594, EPI_ISL_1487596, EPI_ISL_1487598 to 1487602, EPI_ISL_1487604 to 1487606, EPI_ISL_1487608, EPI_ISL_1487610 to 1487612, EPI_ISL_1487615, EPI_ISL_1487618 to 1487620, EPI_ISL_1487622 to 1487623, EPI_ISL_1487626 to 1487628, EPI_ISL_1487630 to 1487634, EPI_ISL_1487636, EPI_ISL_1487638 to 1487639, EPI_ISL_1487642 to 1487647, EPI_ISL_1487649 to 1487652, EPI_ISL_1487654, EPI_ISL_1487656, EPI_ISL_1487659, EPI_ISL_1487661 to 1487669, EPI_ISL_1487671 to 1487695, EPI_ISL_1487697, EPI_ISL_1487699, EPI_ISL_1487702 to 1487703, EPI_ISL_1487705 to 1487707, EPI_ISL_1487709 to 1487711, EPI_ISL_1487713 to 1487715, EPI_ISL_1487717 to 1487718, EPI_ISL_1487720 to 1487724, EPI_ISL_1487873 to 1487880, EPI_ISL_1487882 to 1487898, EPI_ISL_1487900, EPI_ISL_1487902, EPI_ISL_1487904, EPI_ISL_1487906 to 1487920, EPI_ISL_1487922 to 1487960, EPI_ISL_1487962 to 1487966, EPI_ISL_1487968 to 1487969, EPI_ISL_1487971 to 1487996, EPI_ISL_1487998, EPI_ISL_1488000 to 1488055, EPI_ISL_1488057 to 1488127, EPI_ISL_1488129 to 1488131, EPI_ISL_1488133 to 1488145, EPI_ISL_1488147 to 1488152, EPI_ISL_1488154 to 1488164, EPI_ISL_1488166 to 1488167, EPI_ISL_1488169 to 1488201, EPI_ISL_1488203 to 1488222, EPI_ISL_1488224 to 1488225, EPI_ISL_1488228, EPI_ISL_1488231 to 1488244, EPI_ISL_1488247 to 1488248, EPI_ISL_1488250, EPI_ISL_1488256 to 1488258, EPI_ISL_1488260, EPI_ISL_1488262 to 1488263, EPI_ISL_1488265, EPI_ISL_1488272, EPI_ISL_1488274 to 1488279, EPI_ISL_1488281 to 1488282, EPI_ISL_1488287 to 1488288, EPI_ISL_1488290, EPI_ISL_1488293, EPI_ISL_1488295, EPI_ISL_1488299, EPI_ISL_1488301 to 1488303, EPI_ISL_1488305 to 1488306, EPI_ISL_1488309 to 1488312, EPI_ISL_1488315, EPI_ISL_1488318 to 1488319, EPI_ISL_1488321, EPI_ISL_1488324, EPI_ISL_1488327, EPI_ISL_1488330 to 1488332, EPI_ISL_1488334, EPI_ISL_1488336, EPI_ISL_1488339 to 1488340, EPI_ISL_1488342, EPI_ISL_1488345, EPI_ISL_1488349 to 1488352, EPI_ISL_1488356 to 1488358, EPI_ISL_1488360, EPI_ISL_1488363, EPI_ISL_1488365, EPI_ISL_1488368, EPI_ISL_1488370 to 1488375, EPI_ISL_1488377 to 1488381, EPI_ISL_1488383, EPI_ISL_1488385 to 1488386, EPI_ISL_1488396, EPI_ISL_1488401 to 1488404, EPI_ISL_1488407 to 1488409, EPI_ISL_1488411 to 1488415, EPI_ISL_1488417, EPI_ISL_1488419, EPI_ISL_1488421, EPI_ISL_1488425, EPI_ISL_1488429 to 1488432, EPI_ISL_1488434, EPI_ISL_1488436 to 1488437, EPI_ISL_1488439 to 1488443, EPI_ISL_1488445, EPI_ISL_1488447 to 1488449, EPI_ISL_1488452 to 1488454, EPI_ISL_1488456 to 1488457, EPI_ISL_1488460 to 1488462, EPI_ISL_1488464 to 1488471, EPI_ISL_1488473 to 1488475, EPI_ISL_1488478, EPI_ISL_1488480, EPI_ISL_1488482, EPI_ISL_1488484 to 1488488, EPI_ISL_1488490 to 1488493, EPI_ISL_1488497, EPI_ISL_1488499, EPI_ISL_1488501, EPI_ISL_1488503 to 1488509, EPI_ISL_1488511 to 1488512, EPI_ISL_1488514 to 1488515, EPI_ISL_1488517 to 1488520, EPI_ISL_1488522, EPI_ISL_1488524, EPI_ISL_1488526 to 1488528, EPI_ISL_1488530 to 1488534, EPI_ISL_1488536 to 1488540, EPI_ISL_1488542 to 1488543, EPI_ISL_1488545 to 1488549, EPI_ISL_1488551, EPI_ISL_1488553 to 1488554, EPI_ISL_1488556 to 1488557, EPI_ISL_1488559 to 1488564, EPI_ISL_1488566 to 1488574, EPI_ISL_1488577 to 1488578, EPI_ISL_1488580, EPI_ISL_1488582 to 1488584, EPI_ISL_1488586 to 1488593, EPI_ISL_1488595 to 1488597, EPI_ISL_1488599, EPI_ISL_1488601 to 1488604, EPI_ISL_1488606 to 1488607, EPI_ISL_1488609 to 1488612, EPI_ISL_1488616, EPI_ISL_1488618 to 1488626, EPI_ISL_1488628 to 1488629, EPI_ISL_1488631, EPI_ISL_1488634 to 1488639, EPI_ISL_1488642 to 1488646, EPI_ISL_1488648 to 1488650, EPI_ISL_1488652 to 1488653, EPI_ISL_1488655 to 1488658, EPI_ISL_1488660 to 1488673, EPI_ISL_1488676 to 1488677, EPI_ISL_1488679, EPI_ISL_1488681 to 1488683, EPI_ISL_1488685 to 1488694, EPI_ISL_1488696 to 1488703, EPI_ISL_1488705 to 1488713, EPI_ISL_1488715 to 1488718, EPI_ISL_1488720 to 1488721, EPI_ISL_1488724 to 1488727, EPI_ISL_1488729 to 1488732, EPI_ISL_1488734 to 1488736, EPI_ISL_1488738 to 1488739, EPI_ISL_1488741 to 1488750, EPI_ISL_1488753 to 1488755, EPI_ISL_1488757 to 1488761, EPI_ISL_1488765 to 1488769, EPI_ISL_1488771 to 1488773, EPI_ISL_1488775 to 1488776, EPI_ISL_1488778 to 1488788, EPI_ISL_1488790 to 1488794, EPI_ISL_1488796 to 1488797, EPI_ISL_1488799 to 1488801, EPI_ISL_1488803 to 1488805, EPI_ISL_1488807 to 1488808, EPI_ISL_1488811 to 1488812, EPI_ISL_1488816 to 1488820, EPI_ISL_1488822, EPI_ISL_1488824 to 1488826, EPI_ISL_1488828 to 1488829, EPI_ISL_1488831 to 1488833, EPI_ISL_1488837 to 1488838, EPI_ISL_1488840, EPI_ISL_1488842 to 1488857, EPI_ISL_1488860, EPI_ISL_1488863 to 1488865, EPI_ISL_1488869 to 1488870, EPI_ISL_1488873, EPI_ISL_1488875, EPI_ISL_1488879, EPI_ISL_1488884, EPI_ISL_1488888, EPI_ISL_1488891, EPI_ISL_1488896, EPI_ISL_1488899, EPI_ISL_1488904, EPI_ISL_1488906, EPI_ISL_1488910 to 1488912, EPI_ISL_1488917, EPI_ISL_1488920, EPI_ISL_1488922, EPI_ISL_1488924 to 1488925, EPI_ISL_1488931, EPI_ISL_1488937, EPI_ISL_1488939, EPI_ISL_1488942 to 1488943, EPI_ISL_1488945 to 1488947, EPI_ISL_1488949, EPI_ISL_1488951 to 1488952, EPI_ISL_1488962, EPI_ISL_1488966, EPI_ISL_1488968, EPI_ISL_1488970, EPI_ISL_1488972 to 1488973, EPI_ISL_1488975, EPI_ISL_1488978, EPI_ISL_1488983, EPI_ISL_1488993, EPI_ISL_1489001, EPI_ISL_1489003 to 1489004, EPI_ISL_1489009 to 1489010, EPI_ISL_1489012, EPI_ISL_1489014, EPI_ISL_1489016 to 1489017, EPI_ISL_1489020 to 1489021, EPI_ISL_1489025, EPI_ISL_1489028, EPI_ISL_1489033, EPI_ISL_1489035 to 1489036, EPI_ISL_1489041 to 1489042, EPI_ISL_1489046, EPI_ISL_1489048, EPI_ISL_1489050, EPI_ISL_1489054 to 1489055, EPI_ISL_1489058, EPI_ISL_1489063, EPI_ISL_1489072, EPI_ISL_1489075, EPI_ISL_1489078, EPI_ISL_1489087, EPI_ISL_1489089, EPI_ISL_1489093 to 1489094, EPI_ISL_1489097, EPI_ISL_1489100 to 1489101, EPI_ISL_1489104, EPI_ISL_1489108 to 1489109, EPI_ISL_1489111, EPI_ISL_1489113, EPI_ISL_1489115 to 1489117, EPI_ISL_1489122, EPI_ISL_1489126, EPI_ISL_1489138,

EPI_ISL_1489140 to 1489142, EPI_ISL_1489151, EPI_ISL_1489153, EPI_ISL_1489155, EPI_ISL_1489180, EPI_ISL_1489190, EPI_ISL_1489195, EPI_ISL_1489215, EPI_ISL_1489227, EPI_ISL_1489240, EPI_ISL_1489246, EPI_ISL_1489249, EPI_ISL_1489252, EPI_ISL_1489271, EPI_ISL_1489273, EPI_ISL_1489299 to 1489301, EPI_ISL_1489308, EPI_ISL_1489312, EPI_ISL_1489329, EPI_ISL_1489331, EPI_ISL_1489340, EPI_ISL_1489355, EPI_ISL_1489365, EPI_ISL_1489381, EPI_ISL_1489383, EPI_ISL_1489386, EPI_ISL_1489398, EPI_ISL_1489404, EPI_ISL_1489408, EPI_ISL_1489425, EPI_ISL_1489428, EPI_ISL_1489435, EPI_ISL_1489457 to 1489461, EPI_ISL_1489463 to 1489471, EPI_ISL_1489473 to 1489476, EPI_ISL_1489480 to 1489492, EPI_ISL_1489526, EPI_ISL_1489528, EPI_ISL_1504112, EPI_ISL_1504117, EPI_ISL_1504122, EPI_ISL_1504126 to 1504127, EPI_ISL_1504129, EPI_ISL_1504137, EPI_ISL_1504140, EPI_ISL_1504143, EPI_ISL_1504145 to 1504146, EPI_ISL_1504151, EPI_ISL_1504153 to 1504155, EPI_ISL_1504158, EPI_ISL_1504165, EPI_ISL_1504168 to 1504169, EPI_ISL_1504175, EPI_ISL_1504185 to 1504186, EPI_ISL_1504188, EPI_ISL_1504203 to 1504207, EPI_ISL_1504213, EPI_ISL_1504216, EPI_ISL_1504218 to 1504219, EPI_ISL_1504223, EPI_ISL_1504227 to 1504229, EPI_ISL_1504231, EPI_ISL_1504233 to 1504234, EPI_ISL_1504237, EPI_ISL_1504245, EPI_ISL_1504247, EPI_ISL_1504250, EPI_ISL_1504254, EPI_ISL_1504256, EPI_ISL_1504260 to 1504263, EPI_ISL_1504271 to 1504275, EPI_ISL_1504285, EPI_ISL_1504290, EPI_ISL_1504292, EPI_ISL_1504295 to 1504296, EPI_ISL_1504301, EPI_ISL_1504311, EPI_ISL_1504317 to 1504318, EPI_ISL_1504324, EPI_ISL_1504328, EPI_ISL_1504332, EPI_ISL_1504336, EPI_ISL_1504339, EPI_ISL_1504341 to 1504342, EPI_ISL_1504344 to 1504345, EPI_ISL_1504349 to 1504351, EPI_ISL_1504353, EPI_ISL_1504357, EPI_ISL_1504359 to 1504360, EPI_ISL_1504363, EPI_ISL_1504367, EPI_ISL_1504370 to 1504372, EPI_ISL_1504376 to 1504377, EPI_ISL_1504384 to 1504385, EPI_ISL_1504387, EPI_ISL_1504395 to 1504396, EPI_ISL_1504405 to 1504406, EPI_ISL_1504408, EPI_ISL_1504411 to 1504415, EPI_ISL_1504423, EPI_ISL_1504431, EPI_ISL_1504433 to 1504436, EPI_ISL_1504441, EPI_ISL_1504444 to 1504445, EPI_ISL_1504450, EPI_ISL_1504455 to 1504459, EPI_ISL_1504462, EPI_ISL_1504465, EPI_ISL_1504468, EPI_ISL_1504470, EPI_ISL_1504480, EPI_ISL_1504483, EPI_ISL_1504488, EPI_ISL_1504494, EPI_ISL_1504497 to 1504501, EPI_ISL_1504503, EPI_ISL_1504508, EPI_ISL_1504518, EPI_ISL_1504520, EPI_ISL_1504522, EPI_ISL_1504524, EPI_ISL_1504527, EPI_ISL_1504529, EPI_ISL_1504531, EPI_ISL_1504536, EPI_ISL_1504538 to 1504539, EPI_ISL_1504543, EPI_ISL_1504547, EPI_ISL_1504549, EPI_ISL_1504552 to 1504553, EPI_ISL_1504560, EPI_ISL_1504562, EPI_ISL_1504565 to 1504566, EPI_ISL_1504568, EPI_ISL_1504571 to 1504572, EPI_ISL_1504574, EPI_ISL_1504576 to 1504580, EPI_ISL_1504582, EPI_ISL_1504595 to 1504596, EPI_ISL_1504603, EPI_ISL_1504605, EPI_ISL_1504609, EPI_ISL_1504612, EPI_ISL_1504616 to 1504617, EPI_ISL_1504621 to 1504622, EPI_ISL_1504626, EPI_ISL_1504630 to 1504631, EPI_ISL_1504634, EPI_ISL_1504639, EPI_ISL_1504642, EPI_ISL_1504645, EPI_ISL_1504650 to 1504654, EPI_ISL_1504656, EPI_ISL_1504660 to 1504661, EPI_ISL_1504665, EPI_ISL_1504721, EPI_ISL_1504761, EPI_ISL_1504766, EPI_ISL_1504775, EPI_ISL_1504788 to 1504789, EPI_ISL_1504792, EPI_ISL_1504813, EPI_ISL_1504822, EPI_ISL_1504838, EPI_ISL_1504840, EPI_ISL_1504844, EPI_ISL_1504847, EPI_ISL_1504849, EPI_ISL_1504858 to 1504859, EPI_ISL_1504883, EPI_ISL_1504902 to 1504903, EPI_ISL_1504918, EPI_ISL_1504921 to 1504922, EPI_ISL_1504951, EPI_ISL_1504965, EPI_ISL_1504977, EPI_ISL_1504990, EPI_ISL_1504992, EPI_ISL_1504999, EPI_ISL_1505008, EPI_ISL_1505057, EPI_ISL_1505066, EPI_ISL_1505074, EPI_ISL_1505087, EPI_ISL_1505091, EPI_ISL_1505094, EPI_ISL_1505122, EPI_ISL_1505125, EPI_ISL_1505142, EPI_ISL_1505153, EPI_ISL_1505168, EPI_ISL_1505175 to 1505180, EPI_ISL_1505183, EPI_ISL_1505185 to 1505186, EPI_ISL_1505188 to 1505189, EPI_ISL_1505193, EPI_ISL_1505195 to 1505198, EPI_ISL_1505200, EPI_ISL_1505203 to 1505205, EPI_ISL_1505209, EPI_ISL_1505212 to 1505214, EPI_ISL_1505216 to 1505217, EPI_ISL_1505222, EPI_ISL_1505224, EPI_ISL_1505228, EPI_ISL_1505231 to 1505232, EPI_ISL_1505234, EPI_ISL_1505236 to 1505237, EPI_ISL_1505239, EPI_ISL_1505242, EPI_ISL_1505244 to 1505246, EPI_ISL_1505250, EPI_ISL_1505253 to 1505257, EPI_ISL_1505259, EPI_ISL_1505263 to 1505264, EPI_ISL_1505266, EPI_ISL_1505268 to 1505269, EPI_ISL_1505271 to 1505272, EPI_ISL_1505278 to 1505281, EPI_ISL_1505283 to 1505284, EPI_ISL_1505286, EPI_ISL_1505290, EPI_ISL_1505292, EPI_ISL_1505296 to 1505297, EPI_ISL_1505299 to 1505300, EPI_ISL_1505303, EPI_ISL_1505305, EPI_ISL_1505308 to 1505310, EPI_ISL_1505312 to 1505313, EPI_ISL_1505316 to 1505319, EPI_ISL_1505321 to 1505322, EPI_ISL_1505324, EPI_ISL_1505326, EPI_ISL_1505328 to 1505335, EPI_ISL_1505338 to 1505339, EPI_ISL_1505343 to 1505349, EPI_ISL_1505354 to 1505355, EPI_ISL_1505357 to 1505359, EPI_ISL_1505364, EPI_ISL_1505366, EPI_ISL_1505368 to 1505369, EPI_ISL_1505373 to 1505379, EPI_ISL_1505381 to 1505382, EPI_ISL_1505388 to 1505390, EPI_ISL_1505394 to 1505395, EPI_ISL_1505397 to 1505398, EPI_ISL_1505400 to 1505401, EPI_ISL_1505403 to 1505404, EPI_ISL_1505407 to 1505415, EPI_ISL_1505417, EPI_ISL_1505419, EPI_ISL_1505422 to 1505425, EPI_ISL_1505429 to 1505433, EPI_ISL_1505435, EPI_ISL_1505437 to 1505438, EPI_ISL_1505441, EPI_ISL_1505443 to 1505445, EPI_ISL_1505447, EPI_ISL_1505449 to 1505451, EPI_ISL_1505455, EPI_ISL_1505461 to 1505466, EPI_ISL_1505468 to 1505469, EPI_ISL_1505473 to 1505488, EPI_ISL_1505490, EPI_ISL_1505494 to 1505495, EPI_ISL_1505498 to 1505508, EPI_ISL_1505510 to 1505519, EPI_ISL_1505521 to 1505524, EPI_ISL_1505526 to 1505530, EPI_ISL_1505532 to 1505533, EPI_ISL_1505536 to 1505538, EPI_ISL_1505540, EPI_ISL_1505542, EPI_ISL_1505544, EPI_ISL_1505546 to 1505547, EPI_ISL_1505549, EPI_ISL_1505552 to 1505562, EPI_ISL_1505564 to 1505568, EPI_ISL_1505570 to 1505577, EPI_ISL_1505579, EPI_ISL_1505581 to 1505582, EPI_ISL_1505584 to 1505588, EPI_ISL_1505590, EPI_ISL_1505592 to 1505593, EPI_ISL_1505596 to 1505598, EPI_ISL_1505601 to 1505607, EPI_ISL_1505609, EPI_ISL_1505611, EPI_ISL_1505613, EPI_ISL_1505615 to 1505620, EPI_ISL_1505622 to 1505624, EPI_ISL_1505626 to 1505630, EPI_ISL_1505632 to 1505636, EPI_ISL_1505638 to 1505642, EPI_ISL_1505644 to 1505649, EPI_ISL_1505651 to 1505652, EPI_ISL_1505654, EPI_ISL_1505659 to 1505676, EPI_ISL_1505678 to 1505680, EPI_ISL_1505682 to 1505692, EPI_ISL_1505696 to 1505697, EPI_ISL_1505699 to 1505705, EPI_ISL_1505707 to 1505711, EPI_ISL_1505713 to 1505715, EPI_ISL_1505717, EPI_ISL_1505719 to 1505722, EPI_ISL_1505724 to 1505726, EPI_ISL_1505729 to 1505734, EPI_ISL_1505736 to 1505738, EPI_ISL_1505740 to 1505741, EPI_ISL_1505744 to 1505749, EPI_ISL_1505751 to 1505755, EPI_ISL_1505757 to 1505766, EPI_ISL_1505769, EPI_ISL_1505772 to 1505785, EPI_ISL_1505787, EPI_ISL_1505789 to 1505792, EPI_ISL_1505795 to 1505796, EPI_ISL_1505798 to 1505799, EPI_ISL_1505801 to 1505803, EPI_ISL_1505806, EPI_ISL_1505809 to 1505810, EPI_ISL_1505813, EPI_ISL_1505815 to 1505818, EPI_ISL_1505820, EPI_ISL_1505823 to 1505825, EPI_ISL_1505828 to 1505830, EPI_ISL_1505832 to 1505833, EPI_ISL_1505835 to 1505838, EPI_ISL_1505840, EPI_ISL_1505844, EPI_ISL_1505846 to 1505849, EPI_ISL_1505852 to 1505855, EPI_ISL_1505860, EPI_ISL_1505863, EPI_ISL_1505867 to 1505871, EPI_ISL_1505874 to 1505876, EPI_ISL_1505878 to 1505881, EPI_ISL_1505883, EPI_ISL_1505887 to 1505888, EPI_ISL_1505890, EPI_ISL_1505892 to 1505893, EPI_ISL_1505895 to 1505897, EPI_ISL_1505900 to 1505901, EPI_ISL_1505908, EPI_ISL_1505910, EPI_ISL_1505912 to 1505913, EPI_ISL_1505915, EPI_ISL_1505917 to 1505919, EPI_ISL_1505921 to 1505922, EPI_ISL_1505924 to 1505928, EPI_ISL_1505933 to 1505935, EPI_ISL_1505937 to 1505939, EPI_ISL_1505941 to 1505943, EPI_ISL_1505945, EPI_ISL_1505949, EPI_ISL_1505951 to 1505954, EPI_ISL_1505956, EPI_ISL_1505958 to 1505959, EPI_ISL_1505964 to 1505965, EPI_ISL_1505969 to 1505971, EPI_ISL_1505974 to 1505975, EPI_ISL_1505979 to 1505980, EPI_ISL_1505986 to 1505987, EPI_ISL_1505989 to 1505991, EPI_ISL_1505993 to 1505994, EPI_ISL_1505996, EPI_ISL_1506000 to 1506001, EPI_ISL_1506004 to 1506005, EPI_ISL_1506007 to 1506010, EPI_ISL_1506014, EPI_ISL_1506016 to 1506020, EPI_ISL_1506023, EPI_ISL_1506025, EPI_ISL_1506027, EPI_ISL_1506029 to 1506030, EPI_ISL_1506033 to 1506036, EPI_ISL_1506040, EPI_ISL_1506042 to 1506044, EPI_ISL_1506047, EPI_ISL_1506049, EPI_ISL_1506051 to 1506055, EPI_ISL_1506057 to 1506059, EPI_ISL_1506062 to 1506063, EPI_ISL_1506067, EPI_ISL_1506069, EPI_ISL_1506071, EPI_ISL_1506074 to 1506076, EPI_ISL_1506079 to 1506080, EPI_ISL_1506086, EPI_ISL_1506088, EPI_ISL_1506090 to 1506091, EPI_ISL_1506093, EPI_ISL_1506097, EPI_ISL_1506099 to 1506100, EPI_ISL_1506102 to 1506103, EPI_ISL_1506144, EPI_ISL_1506163, EPI_ISL_1506192, EPI_ISL_1506266, EPI_ISL_1506277 to 1506278, EPI_ISL_1506292, EPI_ISL_1506305, EPI_ISL_1506353, EPI_ISL_1506407, EPI_ISL_1506424, EPI_ISL_1506426 to 1506427, EPI_ISL_1506434, EPI_ISL_1506440, EPI_ISL_1506461, EPI_ISL_1506480, EPI_ISL_1506532 to 1506536, EPI_ISL_1506549, EPI_ISL_1506562, EPI_ISL_1506564, EPI_ISL_1506571 to 1506574, EPI_ISL_1506668, EPI_ISL_1507041 to 1507044, EPI_ISL_1507196 to 1507197, EPI_ISL_1507211, EPI_ISL_1507213, EPI_ISL_1507232, EPI_ISL_1507519, EPI_ISL_1507573 to 1507580, EPI_ISL_1507707 to 1507709, EPI_ISL_1507766, EPI_ISL_1507794, EPI_ISL_1507796, EPI_ISL_1507799 to 1507800, EPI_ISL_1507802, EPI_ISL_1507805 to 1507807, EPI_ISL_1507809, EPI_ISL_1507813 to 1507818, EPI_ISL_1507852, EPI_ISL_1507950, EPI_ISL_1507988, EPI_ISL_1508008, EPI_ISL_1508438, EPI_ISL_1508491, EPI_ISL_1517496, EPI_ISL_1517498, EPI_ISL_1517500 to 1517503, EPI_ISL_1517505, EPI_ISL_1517508 to 1517509, EPI_ISL_1517512, EPI_ISL_1517516 to 1517518, EPI_ISL_1517521, EPI_ISL_1517523, EPI_ISL_1517527 to 1517530, EPI_ISL_1517532, EPI_ISL_1517537 to 1517541, EPI_ISL_1517544, EPI_ISL_1517546, EPI_ISL_1517548, EPI_ISL_1517550, EPI_ISL_1517553, EPI_ISL_1517555 to 1517558, EPI_ISL_1517560, EPI_ISL_1517563, EPI_ISL_1517570, EPI_ISL_1517573, EPI_ISL_1517576 to 1517577, EPI_ISL_1517579 to 1517580, EPI_ISL_1517584, EPI_ISL_1517592, EPI_ISL_1517603 to 1517604, EPI_ISL_1517607, EPI_ISL_1517615, EPI_ISL_1517623, EPI_ISL_1517630, EPI_ISL_1517635, EPI_ISL_1517640, EPI_ISL_1517645, EPI_ISL_1517647, EPI_ISL_1517649, EPI_ISL_1517654, EPI_ISL_1517666, EPI_ISL_1517668, EPI_ISL_1517671, EPI_ISL_1517675 to 1517679, EPI_ISL_1517681, EPI_ISL_1517685, EPI_ISL_1517688, EPI_ISL_1517697 to 1517698, EPI_ISL_1517700, EPI_ISL_1517702, EPI_ISL_1517706, EPI_ISL_1517708, EPI_ISL_1517711, EPI_ISL_1517717 to 1517719, EPI_ISL_1517723, EPI_ISL_1517725, EPI_ISL_1517729, EPI_ISL_1517731, EPI_ISL_1517734 to 1517736, EPI_ISL_1517740, EPI_ISL_1517743, EPI_ISL_1517746, EPI_ISL_1517748, EPI_ISL_1517751, EPI_ISL_1517753 to 1517764, EPI_ISL_1517766, EPI_ISL_1517768 to 1517771, EPI_ISL_1517773 to 1517776, EPI_ISL_1517779 to 1517786, EPI_ISL_1517790, EPI_ISL_1517792 to 1517793, EPI_ISL_1517795 to 1517806, EPI_ISL_1517808 to 1517810, EPI_ISL_1517812 to 1517813, EPI_ISL_1517815 to 1517843, EPI_ISL_1517845 to 1517863, EPI_ISL_1517865 to 1517870, EPI_ISL_1517872 to 1517874, EPI_ISL_1517876 to 1517888, EPI_ISL_1517890, EPI_ISL_1517892 to 1517893, EPI_ISL_1517895 to 1517897, EPI_ISL_1517899, EPI_ISL_1517903 to 1517912, EPI_ISL_1517914 to 1517917, EPI_ISL_1517919, EPI_ISL_1517922 to 1517924, EPI_ISL_1517926 to 1517929, EPI_ISL_1517931, EPI_ISL_1517933 to 1517935, EPI_ISL_1517937, EPI_ISL_1517939 to 1517948, EPI_ISL_1517950, EPI_ISL_1517954 to 1517955, EPI_ISL_1517957, EPI_ISL_1517959 to 1517965, EPI_ISL_1517967 to 1517974, EPI_ISL_1517977 to 1517979, EPI_ISL_1517981 to 1517985, EPI_ISL_1517987 to 1517998, EPI_ISL_1518000 to 1518005, EPI_ISL_1518009 to 1518011, EPI_ISL_1518013, EPI_ISL_1518015 to 1518023, EPI_ISL_1518025 to 1518026, EPI_ISL_1518028 to 1518030, EPI_ISL_1518033 to 1518042, EPI_ISL_1518045, EPI_ISL_1518047 to 1518053, EPI_ISL_1518055 to 1518056, EPI_ISL_1518058 to 1518065, EPI_ISL_1518068 to 1518070, EPI_ISL_1518072, EPI_ISL_1518074 to 1518077, EPI_ISL_1518079 to 1518081, EPI_ISL_1518086, EPI_ISL_1518089 to 1518091, EPI_ISL_1518095 to 1518096, EPI_ISL_1518098 to 1518100, EPI_ISL_1518102, EPI_ISL_1518104 to 1518110, EPI_ISL_1518113, EPI_ISL_1518115 to 1518117, EPI_ISL_1518119 to 1518120, EPI_ISL_1518122 to 1518129, EPI_ISL_1518131 to 1518132, EPI_ISL_1518134, EPI_ISL_1518137, EPI_ISL_1518139, EPI_ISL_1518141 to 1518144, EPI_ISL_1518146 to 1518148, EPI_ISL_1518150 to 1518151, EPI_ISL_1518153 to 1518156, EPI_ISL_1518158 to 1518159, EPI_ISL_1518163, EPI_ISL_1518165 to 1518166, EPI_ISL_1518168 to 1518171, EPI_ISL_1518177, EPI_ISL_1518180 to 1518181, EPI_ISL_1518184, EPI_ISL_1518187 to 1518188, EPI_ISL_1518190 to 1518193, EPI_ISL_1518197, EPI_ISL_1518199 to 1518202, EPI_ISL_1518204, EPI_ISL_1518206, EPI_ISL_1518208, EPI_ISL_1518210 to 1518213, EPI_ISL_1518218 to 1518220, EPI_ISL_1518224 to 1518227, EPI_ISL_1518229, EPI_ISL_1518233 to 1518235, EPI_ISL_1518237 to 1518238, EPI_ISL_1518240, EPI_ISL_1518242 to 1518245, EPI_ISL_1518247 to 1518248, EPI_ISL_1518250, EPI_ISL_1518252 to 1518253, EPI_ISL_1518258 to 1518259, EPI_ISL_1518261 to 1518262, EPI_ISL_1518264 to 1518265, EPI_ISL_1518267 to 1518272, EPI_ISL_1518274 to 1518276, EPI_ISL_1518279, EPI_ISL_1518282 to 1518287, EPI_ISL_1518290, EPI_ISL_1518292 to 1518293, EPI_ISL_1518295 to 1518296, EPI_ISL_1518298 to 1518302, EPI_ISL_1518304 to 1518307, EPI_ISL_1518309 to 1518310, EPI_ISL_1518313, EPI_ISL_1518315, EPI_ISL_1518317, EPI_ISL_1518320 to 1518321, EPI_ISL_1518323 to 1518327, EPI_ISL_1518329, EPI_ISL_1518333, EPI_ISL_1518335, EPI_ISL_1518337 to 1518341, EPI_ISL_1518345 to 1518347, EPI_ISL_1518351 to 1518354, EPI_ISL_1518356 to 1518357, EPI_ISL_1518359 to 1518362, EPI_ISL_1518364 to 1518368, EPI_ISL_1518370 to 1518372, EPI_ISL_1518375 to 1518381, EPI_ISL_1518383 to 1518384, EPI_ISL_1518386 to 1518387, EPI_ISL_1518389 to 1518391, EPI_ISL_1518395, EPI_ISL_1518397 to 1518398, EPI_ISL_1518400 to 1518404, EPI_ISL_1518407 to 1518409, EPI_ISL_1518412, EPI_ISL_1518415 to 1518417, EPI_ISL_1518422, EPI_ISL_1518424, EPI_ISL_1518427 to 1518430, EPI_ISL_1518433, EPI_ISL_1518435 to 1518438, EPI_ISL_1518440 to 1518443, EPI_ISL_1518445 to 1518447, EPI_ISL_1518450, EPI_ISL_1518452 to 1518454, EPI_ISL_1518457, EPI_ISL_1518460, EPI_ISL_1518463 to 1518464, EPI_ISL_1518466 to 1518475, EPI_ISL_1518477, EPI_ISL_1518479 to 1518480, EPI_ISL_1518482, EPI_ISL_1518485, EPI_ISL_1518487, EPI_ISL_1518490 to 1518492, EPI_ISL_1518494 to 1518498, EPI_ISL_1518500, EPI_ISL_1518502, EPI_ISL_1518504 to 1518506, EPI_ISL_1518508 to 1518510, EPI_ISL_1518512, EPI_ISL_1518514, EPI_ISL_1518516 to 1518518, EPI_ISL_1518520, EPI_ISL_1518522 to 1518524, EPI_ISL_1518526 to 1518527, EPI_ISL_1518529, EPI_ISL_1518533, EPI_ISL_1518540 to 1518541, EPI_ISL_1518543 to 1518547, EPI_ISL_1518550 to 1518552, EPI_ISL_1518555, EPI_ISL_1518560 to 1518561, EPI_ISL_1518566 to 1518575, EPI_ISL_1518577 to 1518578, EPI_ISL_1518580 to 1518581, EPI_ISL_1518583, EPI_ISL_1518585 to 1518588, EPI_ISL_1518591 to 1518592, EPI_ISL_1518595 to 1518597, EPI_ISL_1518599 to 1518603, EPI_ISL_1518605 to 1518606, EPI_ISL_1518609, EPI_ISL_1518611, EPI_ISL_1518614 to 1518619, EPI_ISL_1518624 to 1518625, EPI_ISL_1518627, EPI_ISL_1518629 to 1518635, EPI_ISL_1518637, EPI_ISL_1518640, EPI_ISL_1518642 to 1518644, EPI_ISL_1518648, EPI_ISL_1518651 to 1518652, EPI_ISL_1518655 to 1518656, EPI_ISL_1518659 to 1518664, EPI_ISL_1518668 to 1518671, EPI_ISL_1518673, EPI_ISL_1518675 to 1518678, EPI_ISL_1518680, EPI_ISL_1518683 to 1518686, EPI_ISL_1518688 to 1518689, EPI_ISL_1518692 to 1518694, EPI_ISL_1518697 to 1518700, EPI_ISL_1518702 to 1518712, EPI_ISL_1518714, EPI_ISL_1518716 to 1518717, EPI_ISL_1518719 to 1518724, EPI_ISL_1518726, EPI_ISL_1518728 to 1518729, EPI_ISL_1518731 to 1518735, EPI_ISL_1518737 to 1518745, EPI_ISL_1518747 to 1518752, EPI_ISL_1518754 to 1518756, EPI_ISL_1518758, EPI_ISL_1518760 to 1518761, EPI_ISL_1518764 to 1518770, EPI_ISL_1518772 to 1518776, EPI_ISL_1518778 to 1518785, EPI_ISL_1518787 to 1518790, EPI_ISL_1518794 to 1518802, EPI_ISL_1518804 to 1518809, EPI_ISL_1518812 to 1518816, EPI_ISL_1518820, EPI_ISL_1518823 to 1518829, EPI_ISL_1518831 to 1518835, EPI_ISL_1518837, EPI_ISL_1518839 to 1518847, EPI_ISL_1518849 to 1518852, EPI_ISL_1518854 to 1518860, EPI_ISL_1518863 to 1518864, EPI_ISL_1518866 to 1518872, EPI_ISL_1518874 to 1518876, EPI_ISL_1518878 to 1518880, EPI_ISL_1518882 to 1518887, EPI_ISL_1518890 to 1518900, EPI_ISL_1518902, EPI_ISL_1518904 to 1518906, EPI_ISL_1518908 to 1518912, EPI_ISL_1518914 to 1518915, EPI_ISL_1518917 to 1518919, EPI_ISL_1518922 to 1518926, EPI_ISL_1518928 to 1518934, EPI_ISL_1518936 to 1518942, EPI_ISL_1518944 to 1518945, EPI_ISL_1518947 to 1518956, EPI_ISL_1518958, EPI_ISL_1518960 to 1518968, EPI_ISL_1518970 to 1518977, EPI_ISL_1518979 to 1518983, EPI_ISL_1518985 to 1518987, EPI_ISL_1518990 to 1518994, EPI_ISL_1519269, EPI_ISL_1519307 to 1519308, EPI_ISL_1519311, EPI_ISL_1519314 to 1519315, EPI_ISL_1519317, EPI_ISL_1519319 to 1519321, EPI_ISL_1519324 to 1519325, EPI_ISL_1519327, EPI_ISL_1519329, EPI_ISL_1519331 to 1519335, EPI_ISL_1519337, EPI_ISL_1519339 to 1519344, EPI_ISL_1519346 to 1519347, EPI_ISL_1519349 to 1519351, EPI_ISL_1519389 to 1519390, EPI_ISL_1519392, EPI_ISL_1519394 to 1519395, EPI_ISL_1519397, EPI_ISL_1519399 to 1519400, EPI_ISL_1519403 to 1519404, EPI_ISL_1519406, EPI_ISL_1519408 to 1519409, EPI_ISL_1519411 to 1519412, EPI_ISL_1519414 to 1519415, EPI_ISL_1519417 to 1519418, EPI_ISL_1519423, EPI_ISL_1519425 to 1519428, EPI_ISL_1519431 to 1519433, EPI_ISL_1519435 to 1519439, EPI_ISL_1519441 to 1519447, EPI_ISL_1519449 to 1519450, EPI_ISL_1519452 to 1519454, EPI_ISL_1519456 to 1519458, EPI_ISL_1519460 to 1519468, EPI_ISL_1519470 to 1519472, EPI_ISL_1519474, EPI_ISL_1519476 to 1519478, EPI_ISL_1519480, EPI_ISL_1519482 to 1519483, EPI_ISL_1519485, EPI_ISL_1519490 to 1519492, EPI_ISL_1519494, EPI_ISL_1519496 to 1519498, EPI_ISL_1519501 to 1519504, EPI_ISL_1519506 to 1519509, EPI_ISL_1519512 to 1519513, EPI_ISL_1519515, EPI_ISL_1519517 to 1519522, EPI_ISL_1519524 to 1519528, EPI_ISL_1519530, EPI_ISL_1519533 to 1519535, EPI_ISL_1519537, EPI_ISL_1519539 to 1519542, EPI_ISL_1519544 to 1519546, EPI_ISL_1519548 to 1519551, EPI_ISL_1519555 to 1519556, EPI_ISL_1519558 to 1519567, EPI_ISL_1519569 to 1519571, EPI_ISL_1519573 to 1519574, EPI_ISL_1519576, EPI_ISL_1519578 to 1519585, EPI_ISL_1519587, EPI_ISL_1519590 to 1519592, EPI_ISL_1519595 to 1519596, EPI_ISL_1519599 to 1519600, EPI_ISL_1519603 to 1519606, EPI_ISL_1519608, EPI_ISL_1519610 to 1519615, EPI_ISL_1519617 to 1519626, EPI_ISL_1519628 to 1519638, EPI_ISL_1519640 to 1519644, EPI_ISL_1519646 to 1519649, EPI_ISL_1519918 to 1519919, EPI_ISL_1519921 to 1519925, EPI_ISL_1534749 to 1534750, EPI_ISL_1534752 to 1534756, EPI_ISL_1534759 to 1534760, EPI_ISL_1534763 to 1534773, EPI_ISL_1534780, EPI_ISL_1534782, EPI_ISL_1534784 to 1534785, EPI_ISL_1534787 to 1534788, EPI_ISL_1534790, EPI_ISL_1534792 to 1534794, EPI_ISL_1534796 to 1534800, EPI_ISL_1534802, EPI_ISL_1534805, EPI_ISL_1534807 to 1534817, EPI_ISL_1534819, EPI_ISL_1534822, EPI_ISL_1534824 to 1534833, EPI_ISL_1534835, EPI_ISL_1534838, EPI_ISL_1534840 to 1534841, EPI_ISL_1534844, EPI_ISL_1534846 to 1534847, EPI_ISL_1534849, EPI_ISL_1534851, EPI_ISL_1534853, EPI_ISL_1534855 to 1534857, EPI_ISL_1534859 to 1534862, EPI_ISL_1534864 to 1534867, EPI_ISL_1534869 to 1534871, EPI_ISL_1534873, EPI_ISL_1534875 to 1534878, EPI_ISL_1534881 to 1534885, EPI_ISL_1534887 to 1534890, EPI_ISL_1534892 to 1534893, EPI_ISL_1534896 to 1534910, EPI_ISL_1534912 to 1534913, EPI_ISL_1534915, EPI_ISL_1534919 to 1534920, EPI_ISL_1534922 to 1534924, EPI_ISL_1534926, EPI_ISL_1534929, EPI_ISL_1534931 to 1534937, EPI_ISL_1534939 to 1534941, EPI_ISL_1534944, EPI_ISL_1534946 to 1534948, EPI_ISL_1534951 to 1534952, EPI_ISL_1534954 to 1534956, EPI_ISL_1534959, EPI_ISL_1534962, EPI_ISL_1534964, EPI_ISL_1534966, EPI_ISL_1534968 to 1534973, EPI_ISL_1534979 to 1534980, EPI_ISL_1534982, EPI_ISL_1534985 to 1534986, EPI_ISL_1534988 to 1534989, EPI_ISL_1534991, EPI_ISL_1534994 to 1534995, EPI_ISL_1534997 to 1535000, EPI_ISL_1535002, EPI_ISL_1535004 to 1535005, EPI_ISL_1535007, EPI_ISL_1535009 to 1535012, EPI_ISL_1535014, EPI_ISL_1535019, EPI_ISL_1535021, EPI_ISL_1535023 to 1535024, EPI_ISL_1535027 to 1535032, EPI_ISL_1535034, EPI_ISL_1535036 to 1535038, EPI_ISL_1535043 to 1535045, EPI_ISL_1535048, EPI_ISL_1535050, EPI_ISL_1535053 to 1535055, EPI_ISL_1535059, EPI_ISL_1535061 to 1535062, EPI_ISL_1535065, EPI_ISL_1535067, EPI_ISL_1535071, EPI_ISL_1535074, EPI_ISL_1535077 to 1535078, EPI_ISL_1535080, EPI_ISL_1535082, EPI_ISL_1535084 to 1535085, EPI_ISL_1535090 to 1535092, EPI_ISL_1535095 to 1535096, EPI_ISL_1535098 to 1535099, EPI_ISL_1535105, EPI_ISL_1535107, EPI_ISL_1535110, EPI_ISL_1535113, EPI_ISL_1535116 to 1535120, EPI_ISL_1535125 to 1535126, EPI_ISL_1535128 to 1535130, EPI_ISL_1535132 to 1535133, EPI_ISL_1535139 to 1535140, EPI_ISL_1535143 to 1535144, EPI_ISL_1535146, EPI_ISL_1535151, EPI_ISL_1535153 to 1535154, EPI_ISL_1535156, EPI_ISL_1535159, EPI_ISL_1535161 to 1535162, EPI_ISL_1535164, EPI_ISL_1535167, EPI_ISL_1535169, EPI_ISL_1535174, EPI_ISL_1535177, EPI_ISL_1535180 to 1535182, EPI_ISL_1535186, EPI_ISL_1535190 to 1535191, EPI_ISL_1535194 to 1535195, EPI_ISL_1535198, EPI_ISL_1535203 to 1535204, EPI_ISL_1535206 to 1535207, EPI_ISL_1535210, EPI_ISL_1535215 to 1535216, EPI_ISL_1535218 to 1535219, EPI_ISL_1535222 to 1535223, EPI_ISL_1535227, EPI_ISL_1535229, EPI_ISL_1535231, EPI_ISL_1535234, EPI_ISL_1535238, EPI_ISL_1535241 to 1535244, EPI_ISL_1535246, EPI_ISL_1535248, EPI_ISL_1535250 to 1535255, EPI_ISL_1535262 to 1535266, EPI_ISL_1535271, EPI_ISL_1535274 to 1535276, EPI_ISL_1535279 to 1535283, EPI_ISL_1535285, EPI_ISL_1535287 to 1535288, EPI_ISL_1535291 to 1535292, EPI_ISL_1535295 to 1535297, EPI_ISL_1535302 to 1535303, EPI_ISL_1535306, EPI_ISL_1535310, EPI_ISL_1535312 to 1535315, EPI_ISL_1535318 to 1535319, EPI_ISL_1535322, EPI_ISL_1535325 to 1535327, EPI_ISL_1535331, EPI_ISL_1535333, EPI_ISL_1535602, EPI_ISL_1535604, EPI_ISL_1535606, EPI_ISL_1535608, EPI_ISL_1535613, EPI_ISL_1535615, EPI_ISL_1535617 to 1535618, EPI_ISL_1535621, EPI_ISL_1535624 to 1535626, EPI_ISL_1535630 to 1535631, EPI_ISL_1535636 to 1535639, EPI_ISL_1535641, EPI_ISL_1535643, EPI_ISL_1535645 to 1535648, EPI_ISL_1535651 to 1535654, EPI_ISL_1535657 to 1535660, EPI_ISL_1535662, EPI_ISL_1535668 to 1535669, EPI_ISL_1535671, EPI_ISL_1535674 to 1535675, EPI_ISL_1535678, EPI_ISL_1535680 to 1535681, EPI_ISL_1535683, EPI_ISL_1535686, EPI_ISL_1535688 to 1535691, EPI_ISL_1535693, EPI_ISL_1535695 to 1535697, EPI_ISL_1535702, EPI_ISL_1535704 to 1535706, EPI_ISL_1535712 to 1535713, EPI_ISL_1535717 to 1535719, EPI_ISL_1535723, EPI_ISL_1535727 to 1535729, EPI_ISL_1535732, EPI_ISL_1535734 to 1535735, EPI_ISL_1535737, EPI_ISL_1535740 to 1535741, EPI_ISL_1535746 to 1535749, EPI_ISL_1535752, EPI_ISL_1535755, EPI_ISL_1535760, EPI_ISL_1535762 to 1535763, EPI_ISL_1535765, EPI_ISL_1535767, EPI_ISL_1535770 to 1535771, EPI_ISL_1535775 to 1535778, EPI_ISL_1535781, EPI_ISL_1535784 to 1535785, EPI_ISL_1535787, EPI_ISL_1535790 to 1535791, EPI_ISL_1535793 to 1535794, EPI_ISL_1535796 to 1535800, EPI_ISL_1535805, EPI_ISL_1535808 to 1535810, EPI_ISL_1535812 to 1535813, EPI_ISL_1535819, EPI_ISL_1535823, EPI_ISL_1535831, EPI_ISL_1535834 to 1535837, EPI_ISL_1535839 to 1535840, EPI_ISL_1535842 to 1535843, EPI_ISL_1535845, EPI_ISL_1535847 to 1535848, EPI_ISL_1535853 to 1535855, EPI_ISL_1535858 to 1535859, EPI_ISL_1535863 to 1535865, EPI_ISL_1535873, EPI_ISL_1535876 to 1535877, EPI_ISL_1535882 to 1535885, EPI_ISL_1535889 to 1535891, EPI_ISL_1535893 to 1535896, EPI_ISL_1535899 to 1535902, EPI_ISL_1535904 to 1535906, EPI_ISL_1535908, EPI_ISL_1535913 to 1535915, EPI_ISL_1535917 to 1535918, EPI_ISL_1535922 to 1535923, EPI_ISL_1535925 to 1535933, EPI_ISL_1535935 to 1535936, EPI_ISL_1535940, EPI_ISL_1535942 to 1535943, EPI_ISL_1535945 to 1535949, EPI_ISL_1535952 to 1535956, EPI_ISL_1535958 to 1535962, EPI_ISL_1535964, EPI_ISL_1535967 to 1535968, EPI_ISL_1535970 to 1535974, EPI_ISL_1535977 to 1535979, EPI_ISL_1535981, EPI_ISL_1535986, EPI_ISL_1535988, EPI_ISL_1535990, EPI_ISL_1535993, EPI_ISL_1535995 to 1535998, EPI_ISL_1536000, EPI_ISL_1536002 to 1536004, EPI_ISL_1536007, EPI_ISL_1536009 to 1536012, EPI_ISL_1536014 to 1536016, EPI_ISL_1536019 to 1536020, EPI_ISL_1536022 to 1536023, EPI_ISL_1536026 to 1536027, EPI_ISL_1536031 to 1536033, EPI_ISL_1536035, EPI_ISL_1536038 to 1536040, EPI_ISL_1536044 to 1536047, EPI_ISL_1536050 to 1536061, EPI_ISL_1536063 to 1536064, EPI_ISL_1536066 to 1536067, EPI_ISL_1536069 to 1536070, EPI_ISL_1536072 to 1536073, EPI_ISL_1536075 to 1536083, EPI_ISL_1536085 to 1536087, EPI_ISL_1536089 to 1536091, EPI_ISL_1536094, EPI_ISL_1536097 to 1536107, EPI_ISL_1536109 to 1536110, EPI_ISL_1536112 to 1536114, EPI_ISL_1536117 to 1536119, EPI_ISL_1536121 to 1536124, EPI_ISL_1536127, EPI_ISL_1536129 to 1536130, EPI_ISL_1536133 to 1536136, EPI_ISL_1536139 to 1536143, EPI_ISL_1536145 to 1536148, EPI_ISL_1536150, EPI_ISL_1536153 to 1536155,

EPI_ISL_1536157 to 1536158, EPI_ISL_1536162 to 1536170, EPI_ISL_1536173 to 1536174, EPI_ISL_1536176 to 1536178, EPI_ISL_1536180 to 1536193, EPI_ISL_1536195 to 1536201, EPI_ISL_1536203 to 1536213, EPI_ISL_1536215 to 1536216, EPI_ISL_1536218 to 1536227, EPI_ISL_1536229, EPI_ISL_1536231 to 1536232, EPI_ISL_1536234 to 1536236, EPI_ISL_1536238 to 1536252, EPI_ISL_1536254 to 1536263, EPI_ISL_1536265 to 1536266, EPI_ISL_1536268 to 1536275, EPI_ISL_1536277 to 1536286, EPI_ISL_1536288 to 1536295, EPI_ISL_1536297 to 1536301, EPI_ISL_1536303 to 1536309, EPI_ISL_1536311, EPI_ISL_1536313 to 1536319, EPI_ISL_1536321, EPI_ISL_1536323 to 1536328, EPI_ISL_1536330 to 1536342, EPI_ISL_1536344 to 1536347, EPI_ISL_1536349, EPI_ISL_1536352 to 1536356, EPI_ISL_1536358 to 1536361, EPI_ISL_1536363 to 1536369, EPI_ISL_1536371 to 1536372, EPI_ISL_1536374 to 1536379, EPI_ISL_1536381 to 1536394, EPI_ISL_1536397 to 1536401, EPI_ISL_1536403, EPI_ISL_1536405 to 1536408, EPI_ISL_1536410, EPI_ISL_1536412 to 1536420, EPI_ISL_1536424, EPI_ISL_1536426 to 1536428, EPI_ISL_1536430 to 1536432, EPI_ISL_1536434 to 1536441, EPI_ISL_1536443 to 1536445, EPI_ISL_1536447 to 1536452, EPI_ISL_1536454 to 1536457, EPI_ISL_1536459 to 1536466, EPI_ISL_1536468 to 1536485, EPI_ISL_1536487 to 1536502, EPI_ISL_1536504 to 1536507, EPI_ISL_1536509 to 1536519, EPI_ISL_1536522 to 1536524, EPI_ISL_1536526, EPI_ISL_1536528 to 1536532, EPI_ISL_1536534, EPI_ISL_1536538, EPI_ISL_1536541, EPI_ISL_1536545 to 1536551, EPI_ISL_1536553, EPI_ISL_1536555 to 1536556, EPI_ISL_1536567, EPI_ISL_1536571 to 1536572, EPI_ISL_1536575 to 1536577, EPI_ISL_1536583, EPI_ISL_1536585 to 1536586, EPI_ISL_1536596, EPI_ISL_1536602, EPI_ISL_1536606, EPI_ISL_1536618 to 1536619, EPI_ISL_1536625, EPI_ISL_1536629 to 1536630, EPI_ISL_1536635, EPI_ISL_1536637, EPI_ISL_1536639 to 1536641, EPI_ISL_1536643 to 1536644, EPI_ISL_1536647, EPI_ISL_1536651 to 1536653, EPI_ISL_1536656 to 1536657, EPI_ISL_1536667, EPI_ISL_1536673, EPI_ISL_1536675, EPI_ISL_1536678, EPI_ISL_1536686 to 1536687, EPI_ISL_1536695 to 1536696, EPI_ISL_1536699, EPI_ISL_1536706, EPI_ISL_1536709, EPI_ISL_1536718, EPI_ISL_1536720 to 1536721, EPI_ISL_1536724, EPI_ISL_1536727 to 1536728, EPI_ISL_1536731, EPI_ISL_1536735, EPI_ISL_1536738, EPI_ISL_1536741, EPI_ISL_1536751, EPI_ISL_1536757 to 1536759, EPI_ISL_1536762, EPI_ISL_1536764 to 1536765, EPI_ISL_1536769, EPI_ISL_1536771, EPI_ISL_1536774, EPI_ISL_1536777, EPI_ISL_1536781, EPI_ISL_1536785 to 1536787, EPI_ISL_1536791, EPI_ISL_1536797 to 1536798, EPI_ISL_1536803, EPI_ISL_1536812, EPI_ISL_1536815, EPI_ISL_1536820, EPI_ISL_1536830, EPI_ISL_1536834, EPI_ISL_1536836, EPI_ISL_1536840, EPI_ISL_1536843, EPI_ISL_1536846, EPI_ISL_1536848, EPI_ISL_1536850, EPI_ISL_1536853, EPI_ISL_1536856, EPI_ISL_1536864 to 1536868, EPI_ISL_1536871, EPI_ISL_1536875, EPI_ISL_1536877, EPI_ISL_1536883, EPI_ISL_1536889 to 1536890, EPI_ISL_1536895 to 1536896, EPI_ISL_1536899 to 1536901, EPI_ISL_1536907 to 1536908, EPI_ISL_1536910, EPI_ISL_1536913, EPI_ISL_1536915 to 1536916, EPI_ISL_1536922, EPI_ISL_1536924, EPI_ISL_1536927 to 1536928, EPI_ISL_1536932, EPI_ISL_1536935 to 1536936, EPI_ISL_1536940 to 1536941, EPI_ISL_1536943, EPI_ISL_1536946, EPI_ISL_1536949 to 1536950, EPI_ISL_1536952, EPI_ISL_1536955, EPI_ISL_1536958, EPI_ISL_1536960 to 1536961, EPI_ISL_1536965, EPI_ISL_1536970, EPI_ISL_1536980, EPI_ISL_1536982 to 1536983, EPI_ISL_1536988 to 1536989, EPI_ISL_1536992, EPI_ISL_1536999, EPI_ISL_1537006, EPI_ISL_1537010, EPI_ISL_1537016, EPI_ISL_1537018, EPI_ISL_1537021, EPI_ISL_1537023, EPI_ISL_1537027, EPI_ISL_1537033, EPI_ISL_1537035, EPI_ISL_1537038 to 1537040, EPI_ISL_1537042, EPI_ISL_1537044, EPI_ISL_1537047, EPI_ISL_1537051 to 1537052, EPI_ISL_1537059, EPI_ISL_1537061 to 1537062, EPI_ISL_1537065 to 1537067, EPI_ISL_1537070, EPI_ISL_1537072, EPI_ISL_1537075, EPI_ISL_1537078 to 1537079, EPI_ISL_1537088, EPI_ISL_1537094, EPI_ISL_1537099, EPI_ISL_1537104, EPI_ISL_1537113, EPI_ISL_1537115, EPI_ISL_1537121, EPI_ISL_1537127 to 1537128, EPI_ISL_1537132 to 1537133, EPI_ISL_1537137, EPI_ISL_1537152, EPI_ISL_1537193, EPI_ISL_1537226, EPI_ISL_1537236, EPI_ISL_1537240, EPI_ISL_1537315, EPI_ISL_1537318 to 1537320, EPI_ISL_1537322 to 1537323, EPI_ISL_1537327 to 1537328, EPI_ISL_1537620 to 1537621, EPI_ISL_1537632, EPI_ISL_1537642, EPI_ISL_1537651, EPI_ISL_1537655, EPI_ISL_1537661, EPI_ISL_1537663, EPI_ISL_1537665, EPI_ISL_1537667, EPI_ISL_1537669 to 1537670, EPI_ISL_1537673, EPI_ISL_1537687, EPI_ISL_1537689 to 1537690, EPI_ISL_1537693, EPI_ISL_1537695, EPI_ISL_1537702, EPI_ISL_1537711, EPI_ISL_1537715, EPI_ISL_1537719, EPI_ISL_1537726, EPI_ISL_1537728, EPI_ISL_1537758 to 1537760, EPI_ISL_1537790, EPI_ISL_1537793, EPI_ISL_1537804, EPI_ISL_1537808, EPI_ISL_1537813, EPI_ISL_1537820, EPI_ISL_1537822, EPI_ISL_1537826, EPI_ISL_1537840, EPI_ISL_1538102, EPI_ISL_1538153, EPI_ISL_1538273, EPI_ISL_1538277 to 1538278, EPI_ISL_1538283, EPI_ISL_1538288, EPI_ISL_1538294, EPI_ISL_1538297, EPI_ISL_1538300 to 1538301, EPI_ISL_1538304, EPI_ISL_1538397, EPI_ISL_1544864, EPI_ISL_1544934, EPI_ISL_1544939

see above	Lighthouse Lab in Alderley Park	Wellcome Sanger Institute for the COVID-19 Genomics UK (COG-UK) Consortium	Cordelia Langford; David K. Jackson; Dominic Kwiatkowski; Ewan Harrison; Ian Johnston; Jacquelyn Wynn; Jeffrey Barrett; John Sillitoe on behalf of the Wellcome Sanger Institute COVID-19 Surveillance Team; John Sillitoe on behalf of the Wellcome Sanger Institute COVID-19 Surveillance Team (http://www.sanger.ac.uk/covid-team); Mairead Hyland; Roberto Amato; Sonia Goncalves; The Lighthouse Lab in Alderley Park and Alex Alderton

EPI_ISL_551285 to 551293, EPI_ISL_551295 to 551326, EPI_ISL_551328 to 551411, EPI_ISL_551413 to 551452, EPI_ISL_551454 to 551483, EPI_ISL_551485 to 551495, EPI_ISL_551497 to 551529, EPI_ISL_551531 to 551576, EPI_ISL_551735 to 551736, EPI_ISL_551740, EPI_ISL_551742 to 551743, EPI_ISL_551749, EPI_ISL_551751, EPI_ISL_551755 to 551756, EPI_ISL_551759 to 551764, EPI_ISL_551769, EPI_ISL_551772, EPI_ISL_551776, EPI_ISL_551779 to 551780, EPI_ISL_551784 to 551785, EPI_ISL_551789, EPI_ISL_551795 to 551796, EPI_ISL_551798 to 551799, EPI_ISL_551801, EPI_ISL_551803 to 551804, EPI_ISL_551806 to 551808, EPI_ISL_551810 to 551812, EPI_ISL_551815, EPI_ISL_551818 to 551819, EPI_ISL_551823 to 551827, EPI_ISL_551832, EPI_ISL_551834, EPI_ISL_552446, EPI_ISL_552449, EPI_ISL_552463 to 552464, EPI_ISL_552466, EPI_ISL_552468, EPI_ISL_552473, EPI_ISL_552476, EPI_ISL_552479, EPI_ISL_552481, EPI_ISL_552486, EPI_ISL_552488, EPI_ISL_552506 to 552508, EPI_ISL_552512, EPI_ISL_552515 to 552516, EPI_ISL_552523, EPI_ISL_552526 to 552527, EPI_ISL_552530, EPI_ISL_552533 to 552534, EPI_ISL_552539 to 552540, EPI_ISL_552547, EPI_ISL_552556, EPI_ISL_552562, EPI_ISL_552565 to 552566, EPI_ISL_552572, EPI_ISL_552574, EPI_ISL_552576 to 552577, EPI_ISL_552579 to 552580, EPI_ISL_552583, EPI_ISL_552587, EPI_ISL_552590 to 552591, EPI_ISL_552595 to 552596, EPI_ISL_552598, EPI_ISL_552607 to 552608, EPI_ISL_552614, EPI_ISL_552617 to 552618, EPI_ISL_552620, EPI_ISL_552622, EPI_ISL_552629, EPI_ISL_552639, EPI_ISL_552642, EPI_ISL_552644, EPI_ISL_552646, EPI_ISL_552655, EPI_ISL_552659, EPI_ISL_552665 to 552666, EPI_ISL_552669, EPI_ISL_552671, EPI_ISL_552679, EPI_ISL_552681, EPI_ISL_552686, EPI_ISL_552689, EPI_ISL_552699, EPI_ISL_552703, EPI_ISL_552706 to 552707, EPI_ISL_552712 to 552713, EPI_ISL_552717, EPI_ISL_552719 to 552724, EPI_ISL_552726, EPI_ISL_552728 to 552732, EPI_ISL_552735, EPI_ISL_552741 to 552742, EPI_ISL_552745 to 552746, EPI_ISL_552748 to 552750, EPI_ISL_552760 to 552761, EPI_ISL_552768, EPI_ISL_552770 to 552771, EPI_ISL_552773 to 552777, EPI_ISL_552779 to 552780, EPI_ISL_552782 to 552783, EPI_ISL_552785, EPI_ISL_552792 to 552794, EPI_ISL_552796 to 552798, EPI_ISL_552800 to 552801, EPI_ISL_552803 to 552808, EPI_ISL_552812 to 552813, EPI_ISL_552815 to 552817, EPI_ISL_552819, EPI_ISL_552821 to 552822, EPI_ISL_552824 to 552840, EPI_ISL_552843 to 552851, EPI_ISL_552853 to 552866, EPI_ISL_552868 to 552873, EPI_ISL_552876 to 552878, EPI_ISL_552881 to 552904, EPI_ISL_552906 to 552909, EPI_ISL_552911 to 552915, EPI_ISL_552917 to 552935, EPI_ISL_552937, EPI_ISL_552940 to 552946, EPI_ISL_552951 to 552952, EPI_ISL_552954 to 552960, EPI_ISL_552962, EPI_ISL_552964 to 552966, EPI_ISL_552968 to 552971, EPI_ISL_552974, EPI_ISL_552976 to 552978, EPI_ISL_552980 to 552986, EPI_ISL_552988 to 552989, EPI_ISL_552991 to 552994, EPI_ISL_552996, EPI_ISL_552998 to 553006, EPI_ISL_553269, EPI_ISL_553271 to 553274, EPI_ISL_553277 to 553294, EPI_ISL_553296 to 553322, EPI_ISL_553324 to 553420, EPI_ISL_553422 to 553567, EPI_ISL_553569 to 553731, EPI_ISL_553733 to 553777, EPI_ISL_553779 to 553783, EPI_ISL_553785 to 553811, EPI_ISL_553813 to 553845, EPI_ISL_553847 to 553865, EPI_ISL_553867 to 553870, EPI_ISL_553872 to 553878, EPI_ISL_553881 to 553884, EPI_ISL_553886 to 553888, EPI_ISL_553890 to 553892, EPI_ISL_553895, EPI_ISL_553899 to 553904, EPI_ISL_553906, EPI_ISL_553908 to 553928, EPI_ISL_553930 to 553933, EPI_ISL_553935 to 553938, EPI_ISL_553940 to 553945, EPI_ISL_553947 to 553951, EPI_ISL_553953, EPI_ISL_553958 to 553964, EPI_ISL_553966 to 553971, EPI_ISL_553973 to 553982, EPI_ISL_553984, EPI_ISL_553986, EPI_ISL_553988, EPI_ISL_553990 to 553992, EPI_ISL_553995 to 554002, EPI_ISL_554004, EPI_ISL_554006 to 554008, EPI_ISL_554010 to 554014, EPI_ISL_554016 to 554023, EPI_ISL_554027, EPI_ISL_554029 to 554041, EPI_ISL_554043 to 554047, EPI_ISL_554049 to 554053, EPI_ISL_554055 to 554056, EPI_ISL_554059 to 554064, EPI_ISL_554066 to 554079, EPI_ISL_554081 to 554096, EPI_ISL_554098, EPI_ISL_554102 to 554117, EPI_ISL_554120, EPI_ISL_554122, EPI_ISL_554126, EPI_ISL_554129 to 554131, EPI_ISL_554136, EPI_ISL_554140, EPI_ISL_554149, EPI_ISL_554151, EPI_ISL_554155 to 554158, EPI_ISL_554164 to 554165, EPI_ISL_554170 to 554172, EPI_ISL_554174 to 554176, EPI_ISL_554183, EPI_ISL_554186 to 554190, EPI_ISL_554192 to 554194, EPI_ISL_554196 to 554199, EPI_ISL_554202 to 554203, EPI_ISL_554206 to 554207, EPI_ISL_554209, EPI_ISL_554212, EPI_ISL_554215, EPI_ISL_554219, EPI_ISL_554222, EPI_ISL_554225, EPI_ISL_554227, EPI_ISL_554229 to 554230, EPI_ISL_554233, EPI_ISL_554235, EPI_ISL_554238, EPI_ISL_554241, EPI_ISL_554250, EPI_ISL_554252, EPI_ISL_554257, EPI_ISL_554260, EPI_ISL_554263, EPI_ISL_554265 to 554266, EPI_ISL_554268, EPI_ISL_554270, EPI_ISL_554275, EPI_ISL_554278 to 554280, EPI_ISL_554282 to 554283, EPI_ISL_554288, EPI_ISL_554292, EPI_ISL_554296, EPI_ISL_554298 to 554299, EPI_ISL_554301, EPI_ISL_554305, EPI_ISL_554309, EPI_ISL_554318, EPI_ISL_554320 to 554477, EPI_ISL_554479 to 554483, EPI_ISL_554485 to 554496, EPI_ISL_554498 to 554536, EPI_ISL_554538 to 554600, EPI_ISL_554602 to 554618, EPI_ISL_554620, EPI_ISL_554623 to 554626, EPI_ISL_554630 to 554632, EPI_ISL_554640, EPI_ISL_554642, EPI_ISL_554646, EPI_ISL_554651, EPI_ISL_554653 to 554654, EPI_ISL_554656 to 554657, EPI_ISL_554661, EPI_ISL_554663 to 554664, EPI_ISL_554666, EPI_ISL_554677, EPI_ISL_554680 to 554681, EPI_ISL_554686, EPI_ISL_554688 to 554689, EPI_ISL_554692, EPI_ISL_554694, EPI_ISL_554696 to 554697, EPI_ISL_554703, EPI_ISL_554708, EPI_ISL_554710, EPI_ISL_554716, EPI_ISL_554725 to 554727, EPI_ISL_554730, EPI_ISL_554732, EPI_ISL_554735, EPI_ISL_554741, EPI_ISL_554743, EPI_ISL_554754 to 554757, EPI_ISL_554759, EPI_ISL_554761, EPI_ISL_554767, EPI_ISL_554771 to 554772, EPI_ISL_554776 to 554780, EPI_ISL_554782, EPI_ISL_554792, EPI_ISL_554794 to 554795, EPI_ISL_554797, EPI_ISL_554799, EPI_ISL_554802, EPI_ISL_554804, EPI_ISL_554807 to 554813, EPI_ISL_554815 to 554816, EPI_ISL_554820, EPI_ISL_554827, EPI_ISL_554830, EPI_ISL_554832 to 554834, EPI_ISL_554836 to 554837, EPI_ISL_554841, EPI_ISL_554846 to 554848, EPI_ISL_554850, EPI_ISL_554853, EPI_ISL_554856, EPI_ISL_554861 to 554867, EPI_ISL_554869 to 554928, EPI_ISL_554930 to 555018, EPI_ISL_555020 to 555040, EPI_ISL_555042 to 555126, EPI_ISL_555128, EPI_ISL_555130 to 555147, EPI_ISL_555149 to 555258, EPI_ISL_555260 to 555302, EPI_ISL_555305 to 555390, EPI_ISL_555392 to 555397, EPI_ISL_555399 to 555431, EPI_ISL_555434 to 555501, EPI_ISL_555503 to 555509, EPI_ISL_555512 to 555557, EPI_ISL_555559 to 555615, EPI_ISL_555617 to 555649, EPI_ISL_555652 to 555659, EPI_ISL_555661 to 555793, EPI_ISL_555795 to 555806, EPI_ISL_555808 to 555814, EPI_ISL_555816 to 555842, EPI_ISL_555845 to 555863, EPI_ISL_555865 to 555867, EPI_ISL_555869 to 555874, EPI_ISL_555876 to 555886, EPI_ISL_555888 to 555909, EPI_ISL_555911 to 555938, EPI_ISL_555940 to 555944, EPI_ISL_555946, EPI_ISL_555949 to 555954, EPI_ISL_555956 to 555972, EPI_ISL_555974 to 555975, EPI_ISL_555977 to 555985, EPI_ISL_555987 to 555989, EPI_ISL_555991 to 555992, EPI_ISL_555994 to 555999, EPI_ISL_556001 to 556011, EPI_ISL_556013 to 556020, EPI_ISL_556023 to 556025, EPI_ISL_556027 to 556038, EPI_ISL_556040, EPI_ISL_556042 to 556046, EPI_ISL_556048 to 556057, EPI_ISL_556059 to 556062, EPI_ISL_556064 to 556065, EPI_ISL_556067 to 556068, EPI_ISL_556070 to 556074, EPI_ISL_556078, EPI_ISL_556080 to 556082, EPI_ISL_556084 to 556086, EPI_ISL_556088 to 556089, EPI_ISL_556096, EPI_ISL_556099 to 556101, EPI_ISL_556103, EPI_ISL_556105 to 556108, EPI_ISL_556110 to 556111, EPI_ISL_556113 to 556114, EPI_ISL_556117 to 556122, EPI_ISL_556124 to 556125, EPI_ISL_556127 to 556132, EPI_ISL_556135 to 556137, EPI_ISL_556140, EPI_ISL_556142 to 556145, EPI_ISL_556147, EPI_ISL_556149, EPI_ISL_556151 to 556157, EPI_ISL_556159 to 556162, EPI_ISL_556167 to 556169, EPI_ISL_556171, EPI_ISL_556173 to 556175, EPI_ISL_556177 to 556182, EPI_ISL_556184 to 556185, EPI_ISL_556188 to 556194, EPI_ISL_556196, EPI_ISL_556200, EPI_ISL_556202 to 556203, EPI_ISL_556205 to 556206, EPI_ISL_556209 to 556210, EPI_ISL_556212, EPI_ISL_556215 to 556217, EPI_ISL_556219 to 556222, EPI_ISL_556224 to 556231, EPI_ISL_556234, EPI_ISL_556236, EPI_ISL_556238 to 556241, EPI_ISL_556243, EPI_ISL_556245 to 556247, EPI_ISL_556249 to 556250, EPI_ISL_556252, EPI_ISL_556254 to 556258, EPI_ISL_556260, EPI_ISL_556262, EPI_ISL_556264 to 556265, EPI_ISL_556268, EPI_ISL_556270 to 556274, EPI_ISL_556277 to 556281, EPI_ISL_556283 to 556284, EPI_ISL_556286, EPI_ISL_556288, EPI_ISL_556290, EPI_ISL_556292, EPI_ISL_556295 to 556297, EPI_ISL_556299, EPI_ISL_556301 to 556303, EPI_ISL_556306 to 556308, EPI_ISL_556310 to 556316, EPI_ISL_556319 to 556320, EPI_ISL_556322 to 556323, EPI_ISL_556325 to 556337, EPI_ISL_556339 to 556343, EPI_ISL_556345 to 556347, EPI_ISL_556352 to 556356, EPI_ISL_556358 to 556410, EPI_ISL_556412 to 556424, EPI_ISL_556426 to 556427, EPI_ISL_556429 to 556432, EPI_ISL_556436 to 556445, EPI_ISL_556447 to 556458, EPI_ISL_556460 to 556470, EPI_ISL_556472 to 556481, EPI_ISL_556483 to 556487, EPI_ISL_556489 to 556493, EPI_ISL_556495 to 556499, EPI_ISL_556501 to 556504, EPI_ISL_556506 to 556507, EPI_ISL_556511 to 556515, EPI_ISL_556576 to 556638, EPI_ISL_556985 to 557001, EPI_ISL_557003 to 557042, EPI_ISL_557044 to 557109, EPI_ISL_557111 to 557141, EPI_ISL_557143 to 557165, EPI_ISL_557351, EPI_ISL_557354, EPI_ISL_557356, EPI_ISL_557359, EPI_ISL_557361, EPI_ISL_557363, EPI_ISL_557366, EPI_ISL_557371, EPI_ISL_557373 to 557374, EPI_ISL_557378, EPI_ISL_557380 to 557381, EPI_ISL_557383, EPI_ISL_557388, EPI_ISL_557390, EPI_ISL_557398 to 557399, EPI_ISL_557404 to 557406, EPI_ISL_557408, EPI_ISL_557411 to 557412, EPI_ISL_557414 to 557415, EPI_ISL_557417, EPI_ISL_557419, EPI_ISL_557423, EPI_ISL_557425, EPI_ISL_557428, EPI_ISL_557430, EPI_ISL_557433 to 557434, EPI_ISL_557436, EPI_ISL_557439 to 557440, EPI_ISL_557446 to 557447, EPI_ISL_557449, EPI_ISL_557452 to 557454, EPI_ISL_557466, EPI_ISL_557468 to 557469, EPI_ISL_557471, EPI_ISL_557474, EPI_ISL_557476, EPI_ISL_557481 to 557482, EPI_ISL_557484 to 557485, EPI_ISL_557487 to 557488, EPI_ISL_557491, EPI_ISL_557493, EPI_ISL_557497, EPI_ISL_557499 to 557500, EPI_ISL_557507, EPI_ISL_557511, EPI_ISL_557518, EPI_ISL_557522 to 557523, EPI_ISL_557526 to 557528, EPI_ISL_557530 to 557531, EPI_ISL_557533, EPI_ISL_557537, EPI_ISL_557539, EPI_ISL_557541, EPI_ISL_557543 to 557544, EPI_ISL_557551, EPI_ISL_557553 to 557624, EPI_ISL_557626 to 557700, EPI_ISL_557702 to 557710, EPI_ISL_557712 to 557727, EPI_ISL_557729, EPI_ISL_557731 to 557738, EPI_ISL_557743 to 557744, EPI_ISL_557746 to 557747, EPI_ISL_557749, EPI_ISL_557751 to 557752, EPI_ISL_557754 to 557756, EPI_ISL_557759, EPI_ISL_557761 to 557763, EPI_ISL_557767, EPI_ISL_557769 to 557770, EPI_ISL_557772 to 557774, EPI_ISL_557776 to 557777, EPI_ISL_557779 to 557780, EPI_ISL_557783, EPI_ISL_557785, EPI_ISL_557787 to 557788, EPI_ISL_557790, EPI_ISL_557792, EPI_ISL_557794, EPI_ISL_557797 to 557799, EPI_ISL_557801, EPI_ISL_557803 to 557806, EPI_ISL_557809 to 557810, EPI_ISL_557813 to 557815, EPI_ISL_557818 to 557820, EPI_ISL_557825 to 557826, EPI_ISL_557831, EPI_ISL_557833 to 557837, EPI_ISL_557839, EPI_ISL_557841 to 557842, EPI_ISL_557844, EPI_ISL_557846, EPI_ISL_557850 to 557851, EPI_ISL_557855 to 557857, EPI_ISL_557859 to 557864, EPI_ISL_557871 to 557875, EPI_ISL_557881 to 557883, EPI_ISL_557886 to 557888, EPI_ISL_557891 to 557892, EPI_ISL_557897, EPI_ISL_557899 to 557904, EPI_ISL_557906, EPI_ISL_557910, EPI_ISL_557912 to 557914, EPI_ISL_557916, EPI_ISL_557918, EPI_ISL_557920, EPI_ISL_557922 to 557924, EPI_ISL_557928 to 557932, EPI_ISL_557934 to 557936, EPI_ISL_557939, EPI_ISL_557943, EPI_ISL_557945 to 557946, EPI_ISL_557950 to 557951, EPI_ISL_557955, EPI_ISL_557957, EPI_ISL_557960 to 557962, EPI_ISL_557966, EPI_ISL_557968 to 557969, EPI_ISL_557971, EPI_ISL_557973, EPI_ISL_557975, EPI_ISL_557978, EPI_ISL_557980 to 557983, EPI_ISL_557985 to 557988, EPI_ISL_557990 to 557998, EPI_ISL_558000, EPI_ISL_558002 to 558020, EPI_ISL_558022 to 558023, EPI_ISL_558025, EPI_ISL_558027, EPI_ISL_558029, EPI_ISL_558031 to 558036, EPI_ISL_558038 to 558040, EPI_ISL_558042 to 558057, EPI_ISL_558059 to 558067, EPI_ISL_558069 to 558088, EPI_ISL_558090 to 558095, EPI_ISL_558097 to 558102, EPI_ISL_558104 to 558105, EPI_ISL_558107 to 558125, EPI_ISL_558128 to 558154, EPI_ISL_558156 to 558184, EPI_ISL_558186 to 558194, EPI_ISL_558196 to 558221, EPI_ISL_558223 to 558233, EPI_ISL_558235 to 558236, EPI_ISL_558238, EPI_ISL_558240, EPI_ISL_558244, EPI_ISL_558256, EPI_ISL_558259 to 558260, EPI_ISL_558263, EPI_ISL_558268 to 558270, EPI_ISL_558274, EPI_ISL_558276 to 558277, EPI_ISL_558288 to 558289, EPI_ISL_558291, EPI_ISL_558307, EPI_ISL_558312, EPI_ISL_558316, EPI_ISL_558321, EPI_ISL_558324, EPI_ISL_558329, EPI_ISL_558332, EPI_ISL_558335, EPI_ISL_558338, EPI_ISL_558343, EPI_ISL_558345, EPI_ISL_558348, EPI_ISL_558350, EPI_ISL_558354 to 558355, EPI_ISL_558364, EPI_ISL_558368, EPI_ISL_558379, EPI_ISL_558389, EPI_ISL_558394 to 558396, EPI_ISL_558404 to 558421, EPI_ISL_558423 to 558431, EPI_ISL_558433 to 558542, EPI_ISL_558544 to 558604, EPI_ISL_558606 to 558616, EPI_ISL_558618 to 558637, EPI_ISL_558639 to 558730, EPI_ISL_558732 to 558795, EPI_ISL_558797 to 558845, EPI_ISL_558847 to 558871, EPI_ISL_558873 to 558876, EPI_ISL_558878 to 558887, EPI_ISL_558889 to 558902, EPI_ISL_558904 to 558913, EPI_ISL_558915 to 558919, EPI_ISL_558922, EPI_ISL_558924, EPI_ISL_558927, EPI_ISL_558929 to 558931, EPI_ISL_558933, EPI_ISL_558935 to 558936, EPI_ISL_558938 to 558948, EPI_ISL_558952 to 558958, EPI_ISL_558960 to 558964, EPI_ISL_558966 to 558969, EPI_ISL_558971 to 558972, EPI_ISL_558974, EPI_ISL_558976 to 558979, EPI_ISL_558982 to 558986, EPI_ISL_558988 to 558992, EPI_ISL_558995 to 558999, EPI_ISL_559001 to 559004, EPI_ISL_559006 to 559008, EPI_ISL_559010, EPI_ISL_559012 to 559016, EPI_ISL_559018, EPI_ISL_559020 to 559021, EPI_ISL_559023 to 559025, EPI_ISL_559027, EPI_ISL_559029 to 559041, EPI_ISL_559044 to 559045, EPI_ISL_559048, EPI_ISL_559050 to 559056, EPI_ISL_559058 to 559060, EPI_ISL_559062, EPI_ISL_559064 to 559070, EPI_ISL_559072 to 559079, EPI_ISL_559082, EPI_ISL_559085 to 559087, EPI_ISL_559089 to 559099, EPI_ISL_559101 to 559103, EPI_ISL_559105, EPI_ISL_559107 to 559116, EPI_ISL_559118, EPI_ISL_559121 to 559124, EPI_ISL_559127 to 559128, EPI_ISL_559130, EPI_ISL_559134 to 559135, EPI_ISL_559137 to 559146, EPI_ISL_559148, EPI_ISL_559150 to 559153, EPI_ISL_559155 to 559156, EPI_ISL_559159 to 559162, EPI_ISL_559165, EPI_ISL_559168 to 559170, EPI_ISL_559172 to 559173, EPI_ISL_559175 to 559180, EPI_ISL_559182 to 559186, EPI_ISL_559188 to 559193, EPI_ISL_559196 to 559200, EPI_ISL_559202 to 559203, EPI_ISL_559205 to 559206, EPI_ISL_559208 to 559212, EPI_ISL_559214, EPI_ISL_559216 to 559217, EPI_ISL_559219 to 559222, EPI_ISL_559224 to 559227, EPI_ISL_559229 to 559231, EPI_ISL_559233 to 559234, EPI_ISL_559237 to 559240, EPI_ISL_559242 to 559243, EPI_ISL_559245, EPI_ISL_559248 to 559252, EPI_ISL_559255, EPI_ISL_559257 to 559258, EPI_ISL_559260, EPI_ISL_559262, EPI_ISL_559265 to 559274, EPI_ISL_559276, EPI_ISL_559279 to 559282, EPI_ISL_559285 to 559286, EPI_ISL_559288 to 559289, EPI_ISL_559291 to 559295, EPI_ISL_559297 to 559299, EPI_ISL_559301 to 559311, EPI_ISL_559313, EPI_ISL_559315 to 559318, EPI_ISL_559321 to 559325, EPI_ISL_559327 to 559330, EPI_ISL_559332 to 559334, EPI_ISL_559336, EPI_ISL_559338 to 559351, EPI_ISL_559356 to 559367, EPI_ISL_559369 to 559446, EPI_ISL_559448 to 559575, EPI_ISL_559577 to 559590, EPI_ISL_559592 to 559615, EPI_ISL_559617 to 559643, EPI_ISL_559645 to 559661, EPI_ISL_566110 to 566420, EPI_ISL_567533 to 567802, EPI_ISL_567804 to 567810, EPI_ISL_567812 to 568222, EPI_ISL_568467 to 568468, EPI_ISL_568474 to 568475, EPI_ISL_568477, EPI_ISL_575399 to 575535, EPI_ISL_575537 to 575760, EPI_ISL_575982 to 575983, EPI_ISL_579919 to 579950, EPI_ISL_579952 to 579970, EPI_ISL_579972 to 580061, EPI_ISL_580063 to 580137, EPI_ISL_580139 to 580186, EPI_ISL_580188 to 580209, EPI_ISL_580211 to 580281, EPI_ISL_580284, EPI_ISL_580288 to 580298, EPI_ISL_580302 to 580304, EPI_ISL_580306 to 580312, EPI_ISL_580314 to 580321, EPI_ISL_580323 to 580324, EPI_ISL_580326, EPI_ISL_580328 to 580331, EPI_ISL_580334 to 580337, EPI_ISL_580339, EPI_ISL_580342 to 580352, EPI_ISL_580355 to 580361, EPI_ISL_580363, EPI_ISL_580365 to 580366, EPI_ISL_580370, EPI_ISL_580372, EPI_ISL_580374 to 580384, EPI_ISL_580386 to 580390, EPI_ISL_580392, EPI_ISL_580395, EPI_ISL_580397 to 580399, EPI_ISL_580401 to 580413, EPI_ISL_580415 to 580417, EPI_ISL_580419 to 580420, EPI_ISL_580422 to 580434, EPI_ISL_580436 to 580443, EPI_ISL_580445 to 580448, EPI_ISL_580450 to 580452, EPI_ISL_580454 to 580458, EPI_ISL_580460 to 580464, EPI_ISL_580466, EPI_ISL_580468 to 580470, EPI_ISL_580472 to 580477, EPI_ISL_580482 to 580484, EPI_ISL_580487 to 580494, EPI_ISL_580496 to 580498, EPI_ISL_580500 to 580501, EPI_ISL_580503 to 580507, EPI_ISL_580510, EPI_ISL_580512 to 580514, EPI_ISL_580516 to 580531, EPI_ISL_580534 to 580535, EPI_ISL_580537 to 580549, EPI_ISL_580551 to 580556, EPI_ISL_580558, EPI_ISL_580560 to 580562, EPI_ISL_580566, EPI_ISL_580568, EPI_ISL_580570 to 580572, EPI_ISL_580574 to 580575, EPI_ISL_580578, EPI_ISL_580580 to 580581, EPI_ISL_580583 to 580585, EPI_ISL_580588 to 580592, EPI_ISL_580595 to 580602, EPI_ISL_580604 to 580606, EPI_ISL_580608 to 580609, EPI_ISL_580611 to 580617, EPI_ISL_580619 to 580620, EPI_ISL_580622 to 580624, EPI_ISL_580627 to 580630, EPI_ISL_580633, EPI_ISL_580635 to 580636, EPI_ISL_580640, EPI_ISL_580645, EPI_ISL_580648, EPI_ISL_580650 to 580651, EPI_ISL_580655, EPI_ISL_580660, EPI_ISL_580663, EPI_ISL_580674 to 580675, EPI_ISL_580677 to 580682, EPI_ISL_580689 to 580692, EPI_ISL_580695 to 580697, EPI_ISL_580699, EPI_ISL_580706, EPI_ISL_580710, EPI_ISL_580720, EPI_ISL_580722 to 580723, EPI_ISL_580730, EPI_ISL_580734, EPI_ISL_580737 to 580738, EPI_ISL_580740, EPI_ISL_580754, EPI_ISL_580763 to 580764, EPI_ISL_580769, EPI_ISL_580771, EPI_ISL_580784, EPI_ISL_580787 to 580789, EPI_ISL_580791, EPI_ISL_580821 to 580823, EPI_ISL_580825, EPI_ISL_580828, EPI_ISL_580833, EPI_ISL_580835, EPI_ISL_580849, EPI_ISL_580851, EPI_ISL_580853, EPI_ISL_581367, EPI_ISL_581378 to 581380, EPI_ISL_581391, EPI_ISL_581404, EPI_ISL_581409, EPI_ISL_581411 to 581414, EPI_ISL_581416 to 581417, EPI_ISL_581424 to 581427, EPI_ISL_581429 to 581433, EPI_ISL_581435 to 581436, EPI_ISL_581442, EPI_ISL_581444 to 581446, EPI_ISL_581448, EPI_ISL_586572 to 586611, EPI_ISL_586613 to 586616, EPI_ISL_586618 to 586620, EPI_ISL_586622 to 586708, EPI_ISL_586710 to 586906, EPI_ISL_586908 to 587084, EPI_ISL_587086 to 587264, EPI_ISL_587266 to 587302, EPI_ISL_587711 to 587976, EPI_ISL_587978 to 588006, EPI_ISL_588008 to 588025, EPI_ISL_588027 to 588059, EPI_ISL_589267 to 589288, EPI_ISL_589290 to 589296, EPI_ISL_589298 to 589301, EPI_ISL_589303, EPI_ISL_589305 to 589328, EPI_ISL_589330 to 589333, EPI_ISL_589336 to 589341, EPI_ISL_589343, EPI_ISL_589345 to 589354, EPI_ISL_589356 to

589375, EPI_ISL_589378 to 589406, EPI_ISL_589408 to 589416, EPI_ISL_589418 to 589422, EPI_ISL_589424 to 589429, EPI_ISL_589431 to 589439, EPI_ISL_589441 to 589492, EPI_ISL_589494 to 589518, EPI_ISL_589522 to 589525, EPI_ISL_589527 to 589566, EPI_ISL_589568 to 589571, EPI_ISL_589762,
EPI_ISL_589765, EPI_ISL_589778 to 589786, EPI_ISL_589802, EPI_ISL_590181, EPI_ISL_590186, EPI_ISL_590193 to 590194, EPI_ISL_590198, EPI_ISL_590200, EPI_ISL_590206, EPI_ISL_590210, EPI_ISL_590213 to 590215, EPI_ISL_590220, EPI_ISL_590224 to 590226, EPI_ISL_590228, EPI_ISL_590234 to 590235,
EPI_ISL_590239, EPI_ISL_590246, EPI_ISL_590250, EPI_ISL_590255, EPI_ISL_590257, EPI_ISL_590259 to 590261, EPI_ISL_590263, EPI_ISL_590265, EPI_ISL_590272, EPI_ISL_590274 to 590277, EPI_ISL_590282 to 590284, EPI_ISL_590286, EPI_ISL_590288, EPI_ISL_590295 to 590296, EPI_ISL_590300,
EPI_ISL_590306 to 590307, EPI_ISL_590309, EPI_ISL_590314, EPI_ISL_590319, EPI_ISL_590321, EPI_ISL_590327 to 590329, EPI_ISL_590335, EPI_ISL_590337, EPI_ISL_590346 to 590347, EPI_ISL_590351 to 590352, EPI_ISL_590355, EPI_ISL_590357, EPI_ISL_590359, EPI_ISL_590361, EPI_ISL_590364,
EPI_ISL_590366 to 590367, EPI_ISL_590369, EPI_ISL_590373, EPI_ISL_590379 to 590380, EPI_ISL_590383, EPI_ISL_590386, EPI_ISL_590388, EPI_ISL_590390, EPI_ISL_590392, EPI_ISL_590397 to 590399, EPI_ISL_590403, EPI_ISL_590405, EPI_ISL_590419, EPI_ISL_590422 to 590423, EPI_ISL_590438,
EPI_ISL_590441 to 590442, EPI_ISL_590446, EPI_ISL_590448, EPI_ISL_590452 to 590453, EPI_ISL_590462, EPI_ISL_597523 to 597632, EPI_ISL_597634 to 597983, EPI_ISL_597985 to 598162, EPI_ISL_598164 to 598248, EPI_ISL_598824 to 598830, EPI_ISL_600521, EPI_ISL_600523, EPI_ISL_600525 to 600526,
EPI_ISL_600528 to 600529, EPI_ISL_600531 to 600532, EPI_ISL_600534, EPI_ISL_600536 to 600537, EPI_ISL_602155, EPI_ISL_602158 to 602161, EPI_ISL_605932 to 606295, EPI_ISL_606924 to 606947, EPI_ISL_606949 to 606959, EPI_ISL_606961 to 607188, EPI_ISL_607190 to 607199, EPI_ISL_607201 to 607290,
EPI_ISL_607623 to 607893, EPI_ISL_607895 to 608146, EPI_ISL_608148 to 608215, EPI_ISL_608217 to 608309, EPI_ISL_608311 to 608346, EPI_ISL_608350 to 608352, EPI_ISL_608355 to 608356, EPI_ISL_608358, EPI_ISL_608361 to 608362, EPI_ISL_608364 to 608367, EPI_ISL_608369, EPI_ISL_608371 to 608372,
EPI_ISL_608374 to 608380, EPI_ISL_608383 to 608387, EPI_ISL_608390, EPI_ISL_608399, EPI_ISL_608401, EPI_ISL_608403, EPI_ISL_608405 to 608406, EPI_ISL_608409, EPI_ISL_608413 to 608415, EPI_ISL_608418 to 608422, EPI_ISL_608425, EPI_ISL_608431, EPI_ISL_608434 to 608435, EPI_ISL_608438,
EPI_ISL_608440 to 608441, EPI_ISL_608443 to 608445, EPI_ISL_608448, EPI_ISL_608452 to 608454, EPI_ISL_608457, EPI_ISL_608460 to 608462, EPI_ISL_608465, EPI_ISL_608468 to 608470, EPI_ISL_608473 to 608474, EPI_ISL_608479, EPI_ISL_608481, EPI_ISL_608483, EPI_ISL_608487 to 608489, EPI_ISL_608491,
EPI_ISL_608496, EPI_ISL_608499 to 608500, EPI_ISL_608502 to 608503, EPI_ISL_608505, EPI_ISL_608511 to 608513, EPI_ISL_608516 to 608520, EPI_ISL_608526, EPI_ISL_608528, EPI_ISL_608533 to 608534, EPI_ISL_608539, EPI_ISL_608541 to 608543, EPI_ISL_608548 to 608549, EPI_ISL_608551, EPI_ISL_608553 to
608556, EPI_ISL_608562 to 608564, EPI_ISL_608567, EPI_ISL_608569, EPI_ISL_608574 to 608575, EPI_ISL_608579 to 608584, EPI_ISL_608586 to 608587, EPI_ISL_608589 to 608595, EPI_ISL_608599 to 608601, EPI_ISL_608603, EPI_ISL_608605 to 608606, EPI_ISL_608609 to 608610, EPI_ISL_608614, EPI_ISL_608617,
EPI_ISL_608621 to 608623, EPI_ISL_608625, EPI_ISL_608630, EPI_ISL_608634 to 608635, EPI_ISL_608639 to 608640, EPI_ISL_608646 to 608649, EPI_ISL_608652, EPI_ISL_608655, EPI_ISL_608657 to 608659, EPI_ISL_608661 to 608663, EPI_ISL_608665, EPI_ISL_608667 to 608670, EPI_ISL_608672, EPI_ISL_608674 to
608677, EPI_ISL_608680, EPI_ISL_608684 to 608687, EPI_ISL_608690 to 608692, EPI_ISL_608694, EPI_ISL_608847, EPI_ISL_608849, EPI_ISL_608851, EPI_ISL_608853, EPI_ISL_608855 to 608856, EPI_ISL_608858, EPI_ISL_608860, EPI_ISL_608862 to 608866, EPI_ISL_608869, EPI_ISL_608872, EPI_ISL_608874,
EPI_ISL_608877 to 608878, EPI_ISL_608880, EPI_ISL_608883 to 608891, EPI_ISL_608895 to 608900, EPI_ISL_608903 to 608904, EPI_ISL_608909, EPI_ISL_608911, EPI_ISL_608913 to 608917, EPI_ISL_608919 to 608922, EPI_ISL_608925 to 608926, EPI_ISL_608928 to 608934, EPI_ISL_608938, EPI_ISL_608941 to 608942,
EPI_ISL_608944 to 608946, EPI_ISL_608948, EPI_ISL_608950, EPI_ISL_608952, EPI_ISL_608955 to 608957, EPI_ISL_608961 to 608964, EPI_ISL_608966, EPI_ISL_608970 to 608973, EPI_ISL_608976, EPI_ISL_608979 to 608981, EPI_ISL_608983 to 608985, EPI_ISL_608988 to 608989, EPI_ISL_608991 to 608995,
EPI_ISL_608998 to 609000, EPI_ISL_609002, EPI_ISL_609004, EPI_ISL_609007, EPI_ISL_609009 to 609013, EPI_ISL_609016, EPI_ISL_609019 to 609020, EPI_ISL_609022, EPI_ISL_609028, EPI_ISL_609031 to 609032, EPI_ISL_609034 to 609035, EPI_ISL_609039 to 609041, EPI_ISL_609043, EPI_ISL_609047,
EPI_ISL_609051 to 609053, EPI_ISL_609055 to 609057, EPI_ISL_609060 to 609061, EPI_ISL_609064 to 609065, EPI_ISL_609068 to 609070, EPI_ISL_609073 to 609075, EPI_ISL_609078 to 609080, EPI_ISL_609083, EPI_ISL_609085, EPI_ISL_609087, EPI_ISL_609089 to 609090, EPI_ISL_609092 to 609096, EPI_ISL_609098,
EPI_ISL_609102 to 609107, EPI_ISL_609109, EPI_ISL_609112, EPI_ISL_609120, EPI_ISL_609124 to 609125, EPI_ISL_609127, EPI_ISL_609130, EPI_ISL_609132 to 609135, EPI_ISL_609141, EPI_ISL_609144, EPI_ISL_609146 to 609148, EPI_ISL_609150, EPI_ISL_609152 to 609154, EPI_ISL_609157, EPI_ISL_609207 to
609208, EPI_ISL_609210 to 609218, EPI_ISL_610286 to 610331, EPI_ISL_610333 to 610501, EPI_ISL_610504 to 610628, EPI_ISL_610922 to 611092, EPI_ISL_611094 to 611286

see above	Lighthouse Lab in Alderley Park	Wellcome Sanger Institute for the COVID-19 Genomics UK (COG-UK) consortium	Cordelia Langford; David K. Jackson; Dominic Kwiatkowski; Ewan Harrison; Ian Johnston; Jacquelyn Wynn; John Sillitoe on behalf of the Wellcome Sanger Institute COVID-19 Surveillance Team; John Sillitoe on behalf of the Wellcome Sanger Institute COVID-19 Surveillance Team (http://www.sanger.ac.uk/covid-team); Mairead Hyland; Roberto Amato; Sonia Goncalves; The Lighthouse Lab in Alderley Park and Alex Alderton

EPI_ISL_551850, EPI_ISL_556698, EPI_ISL_556770, EPI_ISL_556887, EPI_ISL_556940, EPI_ISL_566455, EPI_ISL_566474, EPI_ISL_580332, EPI_ISL_580594, EPI_ISL_596969, EPI_ISL_597074, EPI_ISL_597099, EPI_ISL_597189, EPI_ISL_608347, EPI_ISL_608455, EPI_ISL_608681, EPI_ISL_608907, EPI_ISL_609121,
EPI_ISL_609378, EPI_ISL_609393, EPI_ISL_609450, EPI_ISL_609472, EPI_ISL_609527, EPI_ISL_609534, EPI_ISL_609600, EPI_ISL_609746, EPI_ISL_610702, EPI_ISL_610779, EPI_ISL_610843, EPI_ISL_1390313 to 1390319, EPI_ISL_1409196 to 1409197, EPI_ISL_1409213, EPI_ISL_1409226, EPI_ISL_1409249 to
1409250, EPI_ISL_1409266, EPI_ISL_1409274, EPI_ISL_1409292, EPI_ISL_1409307, EPI_ISL_1409317, EPI_ISL_1409337, EPI_ISL_1409341, EPI_ISL_1409349, EPI_ISL_1409356, EPI_ISL_1409378, EPI_ISL_1409380, EPI_ISL_1409384, EPI_ISL_1409389 to 1409390, EPI_ISL_1409404, EPI_ISL_1409417,
EPI_ISL_1409450, EPI_ISL_1409452, EPI_ISL_1409465, EPI_ISL_1409470, EPI_ISL_1409472, EPI_ISL_1409474, EPI_ISL_1409495 to 1409497, EPI_ISL_1409499 to 1409502, EPI_ISL_1409506, EPI_ISL_1409508 to 1409510, EPI_ISL_1409514, EPI_ISL_1409517, EPI_ISL_1409519 to 1409520, EPI_ISL_1409522 to 1409523,
EPI_ISL_1409526, EPI_ISL_1409533, EPI_ISL_1409535, EPI_ISL_1409537, EPI_ISL_1409541, EPI_ISL_1409545 to 1409546, EPI_ISL_1409549, EPI_ISL_1409552, EPI_ISL_1409559 to 1409564, EPI_ISL_1409566 to 1409569, EPI_ISL_1409571, EPI_ISL_1409580, EPI_ISL_1409582, EPI_ISL_1409584 to 1409587,
EPI_ISL_1409590 to 1409592, EPI_ISL_1409598, EPI_ISL_1409601 to 1409602, EPI_ISL_1409605 to 1409606, EPI_ISL_1409608, EPI_ISL_1409610, EPI_ISL_1409617 to 1409619, EPI_ISL_1409622 to 1409623, EPI_ISL_1409630, EPI_ISL_1409641, EPI_ISL_1409643, EPI_ISL_1409646 to 1409647, EPI_ISL_1409649,
EPI_ISL_1409655 to 1409657, EPI_ISL_1409659 to 1409661, EPI_ISL_1409666, EPI_ISL_1409668, EPI_ISL_1409671 to 1409674, EPI_ISL_1409677, EPI_ISL_1409679, EPI_ISL_1409682 to 1409684, EPI_ISL_1409686, EPI_ISL_1409690, EPI_ISL_1409693 to 1409694, EPI_ISL_1409696 to 1409699, EPI_ISL_1409701 to
1409702, EPI_ISL_1409705, EPI_ISL_1409709 to 1409710, EPI_ISL_1409712 to 1409713, EPI_ISL_1409715 to 1409716, EPI_ISL_1409718, EPI_ISL_1409721 to 1409722, EPI_ISL_1409728, EPI_ISL_1409730 to 1409732, EPI_ISL_1409734 to 1409735, EPI_ISL_1409739, EPI_ISL_1409741 to 1409742, EPI_ISL_1409746 to
1409749, EPI_ISL_1409752, EPI_ISL_1409760 to 1409761, EPI_ISL_1409763, EPI_ISL_1409766 to 1409767, EPI_ISL_1409772 to 1409773, EPI_ISL_1410079 to 1410287, EPI_ISL_1411119, EPI_ISL_1411135, EPI_ISL_1411154, EPI_ISL_1411174, EPI_ISL_1411207, EPI_ISL_1411220, EPI_ISL_1411267, EPI_ISL_1411273,
EPI_ISL_1411289, EPI_ISL_1411573, EPI_ISL_1411850, EPI_ISL_1412224, EPI_ISL_1412261, EPI_ISL_1412268, EPI_ISL_1412298, EPI_ISL_1412306, EPI_ISL_1412308, EPI_ISL_1412311, EPI_ISL_1412320 to 1412321, EPI_ISL_1412328, EPI_ISL_1412331 to 1412333, EPI_ISL_1412337, EPI_ISL_1412345,
EPI_ISL_1412361, EPI_ISL_1412368 to 1412369, EPI_ISL_1412386, EPI_ISL_1412394, EPI_ISL_1412404 to 1412405, EPI_ISL_1412411, EPI_ISL_1412417, EPI_ISL_1412420, EPI_ISL_1412435, EPI_ISL_1412439 to 1412440, EPI_ISL_1412468, EPI_ISL_1412539, EPI_ISL_1412564, EPI_ISL_1412927, EPI_ISL_1412931,
EPI_ISL_1412933, EPI_ISL_1412937, EPI_ISL_1412939, EPI_ISL_1412942, EPI_ISL_1412944 to 1412945, EPI_ISL_1412948 to 1412949, EPI_ISL_1412953, EPI_ISL_1412955 to 1412956, EPI_ISL_1412958, EPI_ISL_1412960, EPI_ISL_1412962, EPI_ISL_1412964 to 1412967, EPI_ISL_1412969 to 1412971, EPI_ISL_1412978
to 1412981, EPI_ISL_1412986 to 1412989, EPI_ISL_1412994 to 1412995, EPI_ISL_1412997 to 1412998, EPI_ISL_1413002, EPI_ISL_1413005 to 1413009, EPI_ISL_1413013 to 1413019, EPI_ISL_1413025 to 1413029, EPI_ISL_1413032 to 1413035, EPI_ISL_1413038 to 1413039, EPI_ISL_1413043 to 1413044, EPI_ISL_1413046
to 1413047, EPI_ISL_1413051, EPI_ISL_1413053 to 1413057, EPI_ISL_1413059, EPI_ISL_1415847, EPI_ISL_1415890, EPI_ISL_1415895, EPI_ISL_1415962, EPI_ISL_1415993, EPI_ISL_1416008, EPI_ISL_1416046, EPI_ISL_1416053, EPI_ISL_1416056, EPI_ISL_1416112, EPI_ISL_1416131, EPI_ISL_1416133,
EPI_ISL_1416181 to 1416186, EPI_ISL_1451152 to 1451153, EPI_ISL_1451158, EPI_ISL_1451160, EPI_ISL_1451163 to 1451167, EPI_ISL_1451169, EPI_ISL_1451173, EPI_ISL_1451175 to 1451176, EPI_ISL_1451178, EPI_ISL_1451180 to 1451182, EPI_ISL_1451191 to 1451195, EPI_ISL_1451198 to 1451201,
EPI_ISL_1451203, EPI_ISL_1451206, EPI_ISL_1451208, EPI_ISL_1451210, EPI_ISL_1451214 to 1451216, EPI_ISL_1451221 to 1451222, EPI_ISL_1451224 to 1451228, EPI_ISL_1451231 to 1451233, EPI_ISL_1451237 to 1451240, EPI_ISL_1451243 to 1451244, EPI_ISL_1451249, EPI_ISL_1451252, EPI_ISL_1451254 to
1451255, EPI_ISL_1451260 to 1451261, EPI_ISL_1451263 to 1451264, EPI_ISL_1451266 to 1451270, EPI_ISL_1451274, EPI_ISL_1451276, EPI_ISL_1451279 to 1451281, EPI_ISL_1451284, EPI_ISL_1451286 to 1451288, EPI_ISL_1451290 to 1451291, EPI_ISL_1451294 to 1451296, EPI_ISL_1451300, EPI_ISL_1451304,
EPI_ISL_1451308 to 1451311, EPI_ISL_1451314 to 1451317, EPI_ISL_1451320, EPI_ISL_1451322, EPI_ISL_1451326 to 1451327, EPI_ISL_1451329, EPI_ISL_1451332, EPI_ISL_1451334, EPI_ISL_1451339, EPI_ISL_1451342 to 1451347, EPI_ISL_1451349 to 1451351, EPI_ISL_1451354, EPI_ISL_1451356 to 1451358,
EPI_ISL_1451360, EPI_ISL_1451362, EPI_ISL_1451364, EPI_ISL_1451367 to 1451371, EPI_ISL_1451373, EPI_ISL_1451376, EPI_ISL_1451380, EPI_ISL_1451382 to 1451384, EPI_ISL_1451386 to 1451388, EPI_ISL_1451390, EPI_ISL_1451393 to 1451395, EPI_ISL_1451399, EPI_ISL_1451402, EPI_ISL_1451406,
EPI_ISL_1451408 to 1451409, EPI_ISL_1451411 to 1451413, EPI_ISL_1451415 to 1451417, EPI_ISL_1451420, EPI_ISL_1451424 to 1451425, EPI_ISL_1451428, EPI_ISL_1451433, EPI_ISL_1451435 to 1451437, EPI_ISL_1451440, EPI_ISL_1451442 to 1451444, EPI_ISL_1451446, EPI_ISL_1451450 to 1451452,
EPI_ISL_1451454, EPI_ISL_1451457, EPI_ISL_1451459 to 1451461, EPI_ISL_1451463, EPI_ISL_1451465 to 1451466, EPI_ISL_1451471, EPI_ISL_1451508, EPI_ISL_1451537, EPI_ISL_1451541, EPI_ISL_1451555 to 1451556, EPI_ISL_1451576, EPI_ISL_1451579, EPI_ISL_1451581, EPI_ISL_1451600, EPI_ISL_1451613,
EPI_ISL_1451620, EPI_ISL_1451628, EPI_ISL_1451641, EPI_ISL_1451653, EPI_ISL_1451657, EPI_ISL_1451663, EPI_ISL_1451670, EPI_ISL_1451682, EPI_ISL_1451685, EPI_ISL_1451707 to 1451708, EPI_ISL_1451719, EPI_ISL_1451752, EPI_ISL_1451778 to 1451779, EPI_ISL_1453489, EPI_ISL_1454157,
EPI_ISL_1454159 to 1454164, EPI_ISL_1454166, EPI_ISL_1454168, EPI_ISL_1454170 to 1454171, EPI_ISL_1454173, EPI_ISL_1454175 to 1454178, EPI_ISL_1454181 to 1454183, EPI_ISL_1454185 to 1454186, EPI_ISL_1454188 to 1454192, EPI_ISL_1454194 to 1454198, EPI_ISL_1454200 to 1454202, EPI_ISL_1454204 to
1454210, EPI_ISL_1454212, EPI_ISL_1454218, EPI_ISL_1454220 to 1454223, EPI_ISL_1454225, EPI_ISL_1454227, EPI_ISL_1454230, EPI_ISL_1454234, EPI_ISL_1454236, EPI_ISL_1454238, EPI_ISL_1454241 to 1454243, EPI_ISL_1454248 to 1454259, EPI_ISL_1454263, EPI_ISL_1454267, EPI_ISL_1454269 to 1454275,
EPI_ISL_1454277, EPI_ISL_1454280 to 1454283, EPI_ISL_1454288 to 1454289, EPI_ISL_1454292 to 1454295, EPI_ISL_1454300, EPI_ISL_1454303, EPI_ISL_1454306 to 1454307, EPI_ISL_1454309, EPI_ISL_1454311, EPI_ISL_1454315 to 1454321, EPI_ISL_1454323 to 1454325, EPI_ISL_1454328, EPI_ISL_1454347 to
1454349, EPI_ISL_1454355, EPI_ISL_1454357, EPI_ISL_1454364, EPI_ISL_1454368, EPI_ISL_1454375, EPI_ISL_1454379, EPI_ISL_1454383, EPI_ISL_1454391, EPI_ISL_1454398 to 1454399, EPI_ISL_1454409, EPI_ISL_1454411, EPI_ISL_1454418, EPI_ISL_1454429, EPI_ISL_1454435, EPI_ISL_1454440,
EPI_ISL_1454442, EPI_ISL_1454446, EPI_ISL_1454448, EPI_ISL_1454454, EPI_ISL_1454467 to 1454468, EPI_ISL_1454471, EPI_ISL_1454475, EPI_ISL_1454479, EPI_ISL_1454485, EPI_ISL_1454494, EPI_ISL_1454497, EPI_ISL_1454502, EPI_ISL_1454504, EPI_ISL_1454507, EPI_ISL_1454521, EPI_ISL_1454523,
EPI_ISL_1454525 to 1454526, EPI_ISL_1454530, EPI_ISL_1454536, EPI_ISL_1454546, EPI_ISL_1454551, EPI_ISL_1454553, EPI_ISL_1454558, EPI_ISL_1454572, EPI_ISL_1454575, EPI_ISL_1454578, EPI_ISL_1454582, EPI_ISL_1454584, EPI_ISL_1454594, EPI_ISL_1454602, EPI_ISL_1454606, EPI_ISL_1454609,
EPI_ISL_1454612, EPI_ISL_1454618 to 1454620, EPI_ISL_1454625 to 1454626, EPI_ISL_1454628, EPI_ISL_1454993 to 1455002, EPI_ISL_1455241, EPI_ISL_1455247, EPI_ISL_1455256, EPI_ISL_1455272, EPI_ISL_1455289, EPI_ISL_1455300, EPI_ISL_1455305 to 1455306, EPI_ISL_1455318, EPI_ISL_1455320,
EPI_ISL_1455327, EPI_ISL_1455341, EPI_ISL_1455356, EPI_ISL_1455358, EPI_ISL_1455384, EPI_ISL_1455399, EPI_ISL_1455422, EPI_ISL_1455428, EPI_ISL_1455431, EPI_ISL_1455433 to 1455434, EPI_ISL_1455438, EPI_ISL_1455515 to 1455517, EPI_ISL_1455637 to 1455647, EPI_ISL_1465904 to 1465907,
EPI_ISL_1465913, EPI_ISL_1465915, EPI_ISL_1465925 to 1465927, EPI_ISL_1465929 to 1465930, EPI_ISL_1465936, EPI_ISL_1465941, EPI_ISL_1465944, EPI_ISL_1465949, EPI_ISL_1465952, EPI_ISL_1465957, EPI_ISL_1465967, EPI_ISL_1465970 to 1465971, EPI_ISL_1465973 to 1465975, EPI_ISL_1465982,
EPI_ISL_1465992, EPI_ISL_1466006, EPI_ISL_1466009 to 1466011, EPI_ISL_1466013, EPI_ISL_1466016, EPI_ISL_1466019 to 1466020, EPI_ISL_1466023 to 1466024, EPI_ISL_1466029, EPI_ISL_1466034, EPI_ISL_1466039, EPI_ISL_1466044, EPI_ISL_1466046, EPI_ISL_1466054 to 1466055, EPI_ISL_1466067,
EPI_ISL_1466076, EPI_ISL_1466079, EPI_ISL_1466083 to 1466084, EPI_ISL_1466088, EPI_ISL_1466100 to 1466102, EPI_ISL_1466109, EPI_ISL_1466111 to 1466113, EPI_ISL_1466115, EPI_ISL_1466117, EPI_ISL_1466121, EPI_ISL_1466125, EPI_ISL_1466129, EPI_ISL_1466133, EPI_ISL_1466141, EPI_ISL_1466143,
EPI_ISL_1466148, EPI_ISL_1466158, EPI_ISL_1467581 to 1467802, EPI_ISL_1468404, EPI_ISL_1473447 to 1473453, EPI_ISL_1473455 to 1473513, EPI_ISL_1473515 to 1473558, EPI_ISL_1473560 to 1473585, EPI_ISL_1473587 to 1473596, EPI_ISL_1473598 to 1473600, EPI_ISL_1473602 to 1473604, EPI_ISL_1473606 to
1473609, EPI_ISL_1473611, EPI_ISL_1473616 to 1473621, EPI_ISL_1473623, EPI_ISL_1473625 to 1473626, EPI_ISL_1473628, EPI_ISL_1473630 to 1473632, EPI_ISL_1473634, EPI_ISL_1473636 to 1473637, EPI_ISL_1473639 to 1473646, EPI_ISL_1473648, EPI_ISL_1473651, EPI_ISL_1473653, EPI_ISL_1473655 to
1473658, EPI_ISL_1473660 to 1473664, EPI_ISL_1473666 to 1473668, EPI_ISL_1473671, EPI_ISL_1473673, EPI_ISL_1473675, EPI_ISL_1473677 to 1473681, EPI_ISL_1473683, EPI_ISL_1473685 to 1473686, EPI_ISL_1473688 to 1473689, EPI_ISL_1473693 to 1473694, EPI_ISL_1473697 to 1473698, EPI_ISL_1473700,
EPI_ISL_1473703 to 1473704, EPI_ISL_1473706, EPI_ISL_1473708, EPI_ISL_1473711, EPI_ISL_1473713 to 1473719, EPI_ISL_1473721, EPI_ISL_1473723 to 1473726, EPI_ISL_1473728, EPI_ISL_1473733 to 1473734, EPI_ISL_1473737, EPI_ISL_1473739 to 1473743, EPI_ISL_1473745 to 1473748, EPI_ISL_1473750,
EPI_ISL_1473752 to 1473754, EPI_ISL_1473756 to 1473761, EPI_ISL_1473763 to 1473771, EPI_ISL_1473773 to 1473776, EPI_ISL_1473778 to 1473779, EPI_ISL_1473781 to 1473783, EPI_ISL_1473785 to 1473788, EPI_ISL_1473790 to 1473792, EPI_ISL_1473794 to 1473795, EPI_ISL_1473797, EPI_ISL_1473799,
EPI_ISL_1474162 to 1474165, EPI_ISL_1483806 to 1483807, EPI_ISL_1483810 to 1483811, EPI_ISL_1483813 to 1483814, EPI_ISL_1483817 to 1483819, EPI_ISL_1483821 to 1483824, EPI_ISL_1483826, EPI_ISL_1483828 to 1483829, EPI_ISL_1483832, EPI_ISL_1483834, EPI_ISL_1483836 to 1483837, EPI_ISL_1483842 to
1483843, EPI_ISL_1483845, EPI_ISL_1483847, EPI_ISL_1483849, EPI_ISL_1483851 to 1483852, EPI_ISL_1483854, EPI_ISL_1483856 to 1483857, EPI_ISL_1483859, EPI_ISL_1483866 to 1483867, EPI_ISL_1483873 to 1483874, EPI_ISL_1483876, EPI_ISL_1483878, EPI_ISL_1483881, EPI_ISL_1483885, EPI_ISL_1483888,
EPI_ISL_1483890 to 1483891, EPI_ISL_1483894, EPI_ISL_1483898 to 1483901, EPI_ISL_1483904, EPI_ISL_1483906, EPI_ISL_1483911, EPI_ISL_1483917 to 1483918, EPI_ISL_1483920, EPI_ISL_1483926, EPI_ISL_1483929, EPI_ISL_1483935 to 1483936, EPI_ISL_1483938, EPI_ISL_1483954, EPI_ISL_1483957,
EPI_ISL_1483959, EPI_ISL_1483963 to 1483965, EPI_ISL_1483968, EPI_ISL_1483970, EPI_ISL_1483972, EPI_ISL_1483974, EPI_ISL_1483983 to 1483984, EPI_ISL_1483986 to 1483987, EPI_ISL_1483995, EPI_ISL_1483999, EPI_ISL_1484003, EPI_ISL_1484011, EPI_ISL_1484013, EPI_ISL_1484015, EPI_ISL_1484020,
EPI_ISL_1484024 to 1484025, EPI_ISL_1484029, EPI_ISL_1484031, EPI_ISL_1484034, EPI_ISL_1484036, EPI_ISL_1484042, EPI_ISL_1484045, EPI_ISL_1484047, EPI_ISL_1484050, EPI_ISL_1484053 to 1484054, EPI_ISL_1484057 to 1484059, EPI_ISL_1484063, EPI_ISL_1484065, EPI_ISL_1484068 to 1484070,
EPI_ISL_1484074 to 1484076, EPI_ISL_1484080 to 1484083, EPI_ISL_1484087, EPI_ISL_1484089, EPI_ISL_1484317 to 1484318, EPI_ISL_1484320, EPI_ISL_1484323, EPI_ISL_1484325, EPI_ISL_1484328, EPI_ISL_1484331 to 1484332, EPI_ISL_1484335, EPI_ISL_1484337 to 1484338, EPI_ISL_1484340 to 1484341,
EPI_ISL_1484343 to 1484344, EPI_ISL_1484346, EPI_ISL_1484349, EPI_ISL_1484352, EPI_ISL_1484354, EPI_ISL_1484356, EPI_ISL_1484358, EPI_ISL_1484361, EPI_ISL_1484366 to 1484367, EPI_ISL_1484369 to 1484370, EPI_ISL_1484372 to 1484373, EPI_ISL_1484375 to 1484377, EPI_ISL_1484379 to 1484380,
EPI_ISL_1484382, EPI_ISL_1484385 to 1484386, EPI_ISL_1484388, EPI_ISL_1484391, EPI_ISL_1484393 to 1484394, EPI_ISL_1484397, EPI_ISL_1484400, EPI_ISL_1484404, EPI_ISL_1484410 to 1484411, EPI_ISL_1484413 to 1484414, EPI_ISL_1484416 to 1484417, EPI_ISL_1484422, EPI_ISL_1484426, EPI_ISL_1484428,
EPI_ISL_1484431, EPI_ISL_1484434, EPI_ISL_1484436, EPI_ISL_1484440, EPI_ISL_1484443, EPI_ISL_1484445 to 1484446, EPI_ISL_1484448 to 1484449, EPI_ISL_1484451 to 1484455, EPI_ISL_1484457 to 1484460, EPI_ISL_1484462, EPI_ISL_1484465 to 1484467, EPI_ISL_1484471 to 1484474, EPI_ISL_1484476 to
1484488, EPI_ISL_1484490 to 1484491, EPI_ISL_1484493 to 1484494, EPI_ISL_1484496 to 1484505, EPI_ISL_1484509 to 1484511, EPI_ISL_1484513, EPI_ISL_1484515 to 1484520, EPI_ISL_1484522 to 1484523, EPI_ISL_1484525, EPI_ISL_1484527 to 1484528, EPI_ISL_1484531 to 1484535, EPI_ISL_1484537 to 1484540,
EPI_ISL_1484542, EPI_ISL_1484544 to 1484562, EPI_ISL_1484564, EPI_ISL_1484566 to 1484568, EPI_ISL_1484570 to 1484576, EPI_ISL_1484578 to 1484579, EPI_ISL_1484582 to 1484583, EPI_ISL_1484586 to 1484588, EPI_ISL_1484590 to 1484604, EPI_ISL_1484606 to 1484607, EPI_ISL_1484609, EPI_ISL_1487163,
EPI_ISL_1487176, EPI_ISL_1487181, EPI_ISL_1487206, EPI_ISL_1487208, EPI_ISL_1487210, EPI_ISL_1487255, EPI_ISL_1487270, EPI_ISL_1487288, EPI_ISL_1487291, EPI_ISL_1487306, EPI_ISL_1487309 to 1487310, EPI_ISL_1487316, EPI_ISL_1487318, EPI_ISL_1487320, EPI_ISL_1487355, EPI_ISL_1487376,
EPI_ISL_1487394, EPI_ISL_1487399, EPI_ISL_1487402, EPI_ISL_1487416, EPI_ISL_1487423 to 1487424, EPI_ISL_1487435, EPI_ISL_1487441, EPI_ISL_1487446, EPI_ISL_1487453, EPI_ISL_1487457, EPI_ISL_1487464, EPI_ISL_1487468, EPI_ISL_1487475, EPI_ISL_1487479 to 1487480, EPI_ISL_1487485,
EPI_ISL_1487490, EPI_ISL_1487496, EPI_ISL_1487507, EPI_ISL_1487525 to 1487526, EPI_ISL_1487531, EPI_ISL_1487539, EPI_ISL_1487547, EPI_ISL_1487550, EPI_ISL_1487552, EPI_ISL_1487562, EPI_ISL_1487572, EPI_ISL_1487581, EPI_ISL_1487585, EPI_ISL_1487592, EPI_ISL_1487597, EPI_ISL_1487607,
EPI_ISL_1487616, EPI_ISL_1487621, EPI_ISL_1487625, EPI_ISL_1487629, EPI_ISL_1487635, EPI_ISL_1487637, EPI_ISL_1487640 to 1487641, EPI_ISL_1487660, EPI_ISL_1487696, EPI_ISL_1487698, EPI_ISL_1487700 to 1487701, EPI_ISL_1487708, EPI_ISL_1487712, EPI_ISL_1487726, EPI_ISL_1487744,
EPI_ISL_1487775, EPI_ISL_1487791, EPI_ISL_1487813, EPI_ISL_1487831 to 1487832, EPI_ISL_1487852 to 1487872, EPI_ISL_1488521, EPI_ISL_1488529, EPI_ISL_1488535, EPI_ISL_1488541, EPI_ISL_1488550, EPI_ISL_1488555, EPI_ISL_1488565, EPI_ISL_1488581, EPI_ISL_1488585, EPI_ISL_1488594,
EPI_ISL_1488598, EPI_ISL_1488613 to 1488614, EPI_ISL_1488632, EPI_ISL_1488640, EPI_ISL_1488647, EPI_ISL_1488684, EPI_ISL_1488695, EPI_ISL_1488714, EPI_ISL_1488719, EPI_ISL_1488722, EPI_ISL_1488728, EPI_ISL_1488737, EPI_ISL_1488751 to 1488752, EPI_ISL_1488756, EPI_ISL_1488764,
EPI_ISL_1488770, EPI_ISL_1488810, EPI_ISL_1488823, EPI_ISL_1488827, EPI_ISL_1488834 to 1488835, EPI_ISL_1488858, EPI_ISL_1489163, EPI_ISL_1489167, EPI_ISL_1489173 to 1489174, EPI_ISL_1489181, EPI_ISL_1489184, EPI_ISL_1489191, EPI_ISL_1489194, EPI_ISL_1489197, EPI_ISL_1489199,
EPI_ISL_1489202, EPI_ISL_1489204, EPI_ISL_1489220, EPI_ISL_1489231 to 1489233, EPI_ISL_1489237, EPI_ISL_1489248, EPI_ISL_1489251, EPI_ISL_1489256 to 1489257, EPI_ISL_1489264, EPI_ISL_1489272, EPI_ISL_1489275, EPI_ISL_1489288, EPI_ISL_1489295, EPI_ISL_1489307, EPI_ISL_1489311,
EPI_ISL_1489313, EPI_ISL_1489323, EPI_ISL_1489325, EPI_ISL_1489330, EPI_ISL_1489334, EPI_ISL_1489338, EPI_ISL_1489343 to 1489344, EPI_ISL_1489346, EPI_ISL_1489356, EPI_ISL_1489359 to 1489360, EPI_ISL_1489369, EPI_ISL_1489378 to 1489379, EPI_ISL_1489382, EPI_ISL_1489388, EPI_ISL_1489391 to
1489392, EPI_ISL_1489394, EPI_ISL_1489401, EPI_ISL_1489403, EPI_ISL_1489407, EPI_ISL_1489413, EPI_ISL_1489421 to 1489422, EPI_ISL_1489430, EPI_ISL_1489436, EPI_ISL_1489439 to 1489440, EPI_ISL_1489443, EPI_ISL_1489451, EPI_ISL_1489455, EPI_ISL_1489495 to 1489525, EPI_ISL_1504403,
EPI_ISL_1504409, EPI_ISL_1504419, EPI_ISL_1504421, EPI_ISL_1504425, EPI_ISL_1504427 to 1504428, EPI_ISL_1504437, EPI_ISL_1504440, EPI_ISL_1504447, EPI_ISL_1504451, EPI_ISL_1504453, EPI_ISL_1504472 to 1504473, EPI_ISL_1504476 to 1504477, EPI_ISL_1504481, EPI_ISL_1504484, EPI_ISL_1504486,
EPI_ISL_1504492, EPI_ISL_1504496, EPI_ISL_1504506, EPI_ISL_1504513 to 1504515, EPI_ISL_1504517, EPI_ISL_1504525 to 1504526, EPI_ISL_1504530, EPI_ISL_1504534, EPI_ISL_1504540, EPI_ISL_1504548, EPI_ISL_1504551, EPI_ISL_1504555 to 1504556, EPI_ISL_1504561, EPI_ISL_1504569, EPI_ISL_1504583 to
1504584, EPI_ISL_1504587 to 1504588, EPI_ISL_1504590 to 1504591, EPI_ISL_1504598, EPI_ISL_1504607, EPI_ISL_1504610, EPI_ISL_1504614 to 1504615, EPI_ISL_1504620, EPI_ISL_1504625, EPI_ISL_1504629, EPI_ISL_1504636 to 1504637, EPI_ISL_1504643 to 1504644, EPI_ISL_1504662 to 1504663,
EPI_ISL_1504668 to 1504675, EPI_ISL_1504678 to 1504679, EPI_ISL_1504688 to 1504689, EPI_ISL_1504691 to 1504693, EPI_ISL_1504695, EPI_ISL_1504699, EPI_ISL_1504701 to 1504702, EPI_ISL_1504704 to 1504705, EPI_ISL_1504708, EPI_ISL_1504713, EPI_ISL_1504715, EPI_ISL_1504717 to 1504720,

EPI_ISL_1504722, EPI_ISL_1504725 to 1504726, EPI_ISL_1504733, EPI_ISL_1504735, EPI_ISL_1504737 to 1504739, EPI_ISL_1504746, EPI_ISL_1504748, EPI_ISL_1504755, EPI_ISL_1504757, EPI_ISL_1504759 to 1504760, EPI_ISL_1504774, EPI_ISL_1504777, EPI_ISL_1504779 to 1504782, EPI_ISL_1504785, EPI_ISL_1504787, EPI_ISL_1504790, EPI_ISL_1504793 to 1504794, EPI_ISL_1504796 to 1504800, EPI_ISL_1504802 to 1504803, EPI_ISL_1504809 to 1504812, EPI_ISL_1504816, EPI_ISL_1504818, EPI_ISL_1504821, EPI_ISL_1504823, EPI_ISL_1504825, EPI_ISL_1504828, EPI_ISL_1504830, EPI_ISL_1504832 to 1504833, EPI_ISL_1504837, EPI_ISL_1504839, EPI_ISL_1504841 to 1504843, EPI_ISL_1504845, EPI_ISL_1504848, EPI_ISL_1504850, EPI_ISL_1504856 to 1504857, EPI_ISL_1504860, EPI_ISL_1504862, EPI_ISL_1504865 to 1504867, EPI_ISL_1504869 to 1504870, EPI_ISL_1504872 to 1504874, EPI_ISL_1504876, EPI_ISL_1504881, EPI_ISL_1504884 to 1504885, EPI_ISL_1504887, EPI_ISL_1504891 to 1504892, EPI_ISL_1504896 to 1504899, EPI_ISL_1504901, EPI_ISL_1504904, EPI_ISL_1504907 to 1504909, EPI_ISL_1504917, EPI_ISL_1504920, EPI_ISL_1504923, EPI_ISL_1504933, EPI_ISL_1504939, EPI_ISL_1504941, EPI_ISL_1504943, EPI_ISL_1504948, EPI_ISL_1504955, EPI_ISL_1504957, EPI_ISL_1504959, EPI_ISL_1504961, EPI_ISL_1504964, EPI_ISL_1504966, EPI_ISL_1504968, EPI_ISL_1504979 to 1504981, EPI_ISL_1504987 to 1504988, EPI_ISL_1504991, EPI_ISL_1504996, EPI_ISL_1505004, EPI_ISL_1505012 to 1505014, EPI_ISL_1505017 to 1505018, EPI_ISL_1505025, EPI_ISL_1505028, EPI_ISL_1505034, EPI_ISL_1505036 to 1505038, EPI_ISL_1505043, EPI_ISL_1505045 to 1505046, EPI_ISL_1505049 to 1505050, EPI_ISL_1505061, EPI_ISL_1505064, EPI_ISL_1505070, EPI_ISL_1505077, EPI_ISL_1505080, EPI_ISL_1505082, EPI_ISL_1505084, EPI_ISL_1505090, EPI_ISL_1505095 to 1505096, EPI_ISL_1505098, EPI_ISL_1505103, EPI_ISL_1505108, EPI_ISL_1505110 to 1505111, EPI_ISL_1505116, EPI_ISL_1505124, EPI_ISL_1505126, EPI_ISL_1505129, EPI_ISL_1505136, EPI_ISL_1505138 to 1505140, EPI_ISL_1505144, EPI_ISL_1505147, EPI_ISL_1505150, EPI_ISL_1505158 to 1505159, EPI_ISL_1505164 to 1505165, EPI_ISL_1505170, EPI_ISL_1505173, EPI_ISL_1506105 to 1506113, EPI_ISL_1506128 to 1506133, EPI_ISL_1506139, EPI_ISL_1506145, EPI_ISL_1506157 to 1506159, EPI_ISL_1506165, EPI_ISL_1506172, EPI_ISL_1506175, EPI_ISL_1506514, EPI_ISL_1506520, EPI_ISL_1506528 to 1506531, EPI_ISL_1507597, EPI_ISL_1507942, EPI_ISL_1508560 to 1508561, EPI_ISL_1508648, EPI_ISL_1517765, EPI_ISL_1517767, EPI_ISL_1517772, EPI_ISL_1517777 to 1517778, EPI_ISL_1517787 to 1517789, EPI_ISL_1517791, EPI_ISL_1517794, EPI_ISL_1517807, EPI_ISL_1517811, EPI_ISL_1517814, EPI_ISL_1517844, EPI_ISL_1517864, EPI_ISL_1517871, EPI_ISL_1517875, EPI_ISL_1517889, EPI_ISL_1517891, EPI_ISL_1517894, EPI_ISL_1517898, EPI_ISL_1517900 to 1517902, EPI_ISL_1517913, EPI_ISL_1517918, EPI_ISL_1517920 to 1517921, EPI_ISL_1517925, EPI_ISL_1517930, EPI_ISL_1517932, EPI_ISL_1517936, EPI_ISL_1517938, EPI_ISL_1517949, EPI_ISL_1517951 to 1517953, EPI_ISL_1517956, EPI_ISL_1517958, EPI_ISL_1517966, EPI_ISL_1517975 to 1517976, EPI_ISL_1517980, EPI_ISL_1517986, EPI_ISL_1517999, EPI_ISL_1518006 to 1518008, EPI_ISL_1518012, EPI_ISL_1518014, EPI_ISL_1518024, EPI_ISL_1518027, EPI_ISL_1518031 to 1518032, EPI_ISL_1518043 to 1518044, EPI_ISL_1518046, EPI_ISL_1518054, EPI_ISL_1518057, EPI_ISL_1519271, EPI_ISL_1519274, EPI_ISL_1519276 to 1519278, EPI_ISL_1519280, EPI_ISL_1519285, EPI_ISL_1519288 to 1519294, EPI_ISL_1519297 to 1519301, EPI_ISL_1519303 to 1519306, EPI_ISL_1519929 to 1519931, EPI_ISL_1519968, EPI_ISL_1534757 to 1534758, EPI_ISL_1534762, EPI_ISL_1534775, EPI_ISL_1534777 to 1534779, EPI_ISL_1534781, EPI_ISL_1534783, EPI_ISL_1534786, EPI_ISL_1534789, EPI_ISL_1534791, EPI_ISL_1534795, EPI_ISL_1534803 to 1534804, EPI_ISL_1534818, EPI_ISL_1534820 to 1534821, EPI_ISL_1534823, EPI_ISL_1534834, EPI_ISL_1534836 to 1534837, EPI_ISL_1534839, EPI_ISL_1534843, EPI_ISL_1534850, EPI_ISL_1534854, EPI_ISL_1534858, EPI_ISL_1534863, EPI_ISL_1534874, EPI_ISL_1534879, EPI_ISL_1534886, EPI_ISL_1534895, EPI_ISL_1534914, EPI_ISL_1534917 to 1534918, EPI_ISL_1534921, EPI_ISL_1534927 to 1534928, EPI_ISL_1534930, EPI_ISL_1534938, EPI_ISL_1534943, EPI_ISL_1534945, EPI_ISL_1534949, EPI_ISL_1534957 to 1534958, EPI_ISL_1534961, EPI_ISL_1534963, EPI_ISL_1534965, EPI_ISL_1534967, EPI_ISL_1534975 to 1534976, EPI_ISL_1534978, EPI_ISL_1534981, EPI_ISL_1534983 to 1534984, EPI_ISL_1534992 to 1534993, EPI_ISL_1534996, EPI_ISL_1535001, EPI_ISL_1535003, EPI_ISL_1535006, EPI_ISL_1535008, EPI_ISL_1535016, EPI_ISL_1535033, EPI_ISL_1535035, EPI_ISL_1535042, EPI_ISL_1535070, EPI_ISL_1535100, EPI_ISL_1535157, EPI_ISL_1535178, EPI_ISL_1535258, EPI_ISL_1535267, EPI_ISL_1535272, EPI_ISL_1535293, EPI_ISL_1535301, EPI_ISL_1535329 to 1535330, EPI_ISL_1535332, EPI_ISL_1537316 to 1537317, EPI_ISL_1537321, EPI_ISL_1537324 to 1537326, EPI_ISL_1538020, EPI_ISL_1538029, EPI_ISL_1538031, EPI_ISL_1538036, EPI_ISL_1538039, EPI_ISL_1538043, EPI_ISL_1538053, EPI_ISL_1538057, EPI_ISL_1538063, EPI_ISL_1538067, EPI_ISL_1538069, EPI_ISL_1538071 to 1538073, EPI_ISL_1538078, EPI_ISL_1538080, EPI_ISL_1538084, EPI_ISL_1538088, EPI_ISL_1538090 to 1538091, EPI_ISL_1538093, EPI_ISL_1544778, EPI_ISL_1544783, EPI_ISL_1544792

see above	Lighthouse Lab in Cambridge	Wellcome Sanger Institute for the COVID-19 Genomics UK (COG-UK) Consortium	Cordelia Langford; David K. Jackson; Dominic Kwiatkowski; Ewan Harrison; Ian Johnston; Jeffrey Barrett; John Sillitoe on behalf of the Wellcome Sanger Institute COVID-19 Surveillance Team; John Sillitoe on behalf of the Wellcome Sanger Institute COVID-19 Surveillance Team (http://www.sanger.ac.uk/covid-team); Rob Howes; Roberto Amato; Sonia Goncalves; The Lighthouse Lab in Cambridge and Alex Alderton

EPI_ISL_551835 to 551849, EPI_ISL_551851 to 551921, EPI_ISL_551925, EPI_ISL_551932, EPI_ISL_551951, EPI_ISL_551953, EPI_ISL_551967, EPI_ISL_551980, EPI_ISL_551983, EPI_ISL_551990, EPI_ISL_551994, EPI_ISL_552001, EPI_ISL_552005, EPI_ISL_552008 to 552010, EPI_ISL_552017, EPI_ISL_552051, EPI_ISL_552065, EPI_ISL_552073, EPI_ISL_552091, EPI_ISL_552094, EPI_ISL_552098, EPI_ISL_552105, EPI_ISL_552124, EPI_ISL_552151, EPI_ISL_552166, EPI_ISL_552170, EPI_ISL_552173, EPI_ISL_552180, EPI_ISL_552185, EPI_ISL_555843 to 555844, EPI_ISL_555864, EPI_ISL_555868, EPI_ISL_555875, EPI_ISL_555910, EPI_ISL_555939, EPI_ISL_555945, EPI_ISL_555948, EPI_ISL_555955, EPI_ISL_555973, EPI_ISL_555976, EPI_ISL_555993, EPI_ISL_556012, EPI_ISL_556021 to 556022, EPI_ISL_556026, EPI_ISL_556039, EPI_ISL_556041, EPI_ISL_556047, EPI_ISL_556058, EPI_ISL_556063, EPI_ISL_556066, EPI_ISL_556069, EPI_ISL_556305, EPI_ISL_556309, EPI_ISL_556317 to 556318, EPI_ISL_556321, EPI_ISL_556324, EPI_ISL_556338, EPI_ISL_556344, EPI_ISL_556349, EPI_ISL_556351, EPI_ISL_556357, EPI_ISL_556425, EPI_ISL_556434, EPI_ISL_556446, EPI_ISL_556459, EPI_ISL_556471, EPI_ISL_556482, EPI_ISL_556488, EPI_ISL_556494, EPI_ISL_556500, EPI_ISL_556508 to 556509, EPI_ISL_556516 to 556575, EPI_ISL_556639 to 556697, EPI_ISL_556699 to 556769, EPI_ISL_556771 to 556886, EPI_ISL_556888 to 556939, EPI_ISL_556941 to 556984, EPI_ISL_566423, EPI_ISL_566428 to 566429, EPI_ISL_566444 to 566445, EPI_ISL_566453, EPI_ISL_566456 to 566457, EPI_ISL_566467, EPI_ISL_566473, EPI_ISL_566492 to 566493, EPI_ISL_566495, EPI_ISL_566500, EPI_ISL_566508, EPI_ISL_566518 to 566519, EPI_ISL_566524, EPI_ISL_566528, EPI_ISL_566534, EPI_ISL_566537, EPI_ISL_566543 to 566544, EPI_ISL_566567, EPI_ISL_566576, EPI_ISL_566586, EPI_ISL_566589, EPI_ISL_566610 to 566611, EPI_ISL_566625, EPI_ISL_566631, EPI_ISL_566638, EPI_ISL_566659, EPI_ISL_566661 to 566662, EPI_ISL_566666, EPI_ISL_566672, EPI_ISL_566674, EPI_ISL_568385, EPI_ISL_568436, EPI_ISL_568453 to 568454, EPI_ISL_568458, EPI_ISL_568461, EPI_ISL_568472 to 568473, EPI_ISL_580282 to 580283, EPI_ISL_580286 to 580287, EPI_ISL_580299 to 580301, EPI_ISL_580305, EPI_ISL_580313, EPI_ISL_580322, EPI_ISL_580325, EPI_ISL_580327, EPI_ISL_580333, EPI_ISL_580338, EPI_ISL_580340 to 580341, EPI_ISL_580353 to 580354, EPI_ISL_580362, EPI_ISL_580364, EPI_ISL_580367 to 580369, EPI_ISL_580371, EPI_ISL_580373, EPI_ISL_580385, EPI_ISL_580391, EPI_ISL_580393 to 580394, EPI_ISL_580396, EPI_ISL_580400, EPI_ISL_580414, EPI_ISL_580418, EPI_ISL_580421, EPI_ISL_580435, EPI_ISL_580444, EPI_ISL_580449, EPI_ISL_580453, EPI_ISL_580459, EPI_ISL_580465, EPI_ISL_580467, EPI_ISL_580471, EPI_ISL_580478 to 580481, EPI_ISL_580485 to 580486, EPI_ISL_580495, EPI_ISL_580499, EPI_ISL_580502, EPI_ISL_580508 to 580509, EPI_ISL_580511, EPI_ISL_580515, EPI_ISL_580532 to 580533, EPI_ISL_580536, EPI_ISL_580550, EPI_ISL_580557, EPI_ISL_580559, EPI_ISL_580564 to 580565, EPI_ISL_580567, EPI_ISL_580569, EPI_ISL_580573, EPI_ISL_580576 to 580577, EPI_ISL_580579, EPI_ISL_580582, EPI_ISL_580586 to 580587, EPI_ISL_580593, EPI_ISL_580603, EPI_ISL_580607, EPI_ISL_580610, EPI_ISL_580618, EPI_ISL_580621, EPI_ISL_580625 to 580626, EPI_ISL_581394, EPI_ISL_581396, EPI_ISL_590173, EPI_ISL_596928 to 596968, EPI_ISL_596970 to 597073, EPI_ISL_597075 to 597098, EPI_ISL_597100 to 597188, EPI_ISL_597190 to 597233, EPI_ISL_597237 to 597239, EPI_ISL_597241, EPI_ISL_597243, EPI_ISL_597246 to 597248, EPI_ISL_597250 to 597251, EPI_ISL_597253 to 597259, EPI_ISL_597262, EPI_ISL_597264 to 597266, EPI_ISL_597269 to 597271, EPI_ISL_597273 to 597274, EPI_ISL_597279, EPI_ISL_597282 to 597287, EPI_ISL_597289, EPI_ISL_597293 to 597295, EPI_ISL_597297, EPI_ISL_597300 to 597301, EPI_ISL_597304 to 597310, EPI_ISL_597316 to 597317, EPI_ISL_597319 to 597321, EPI_ISL_597323 to 597325, EPI_ISL_597327, EPI_ISL_597331, EPI_ISL_597335, EPI_ISL_597337, EPI_ISL_597340 to 597346, EPI_ISL_597349 to 597350, EPI_ISL_597354, EPI_ISL_597358, EPI_ISL_597361, EPI_ISL_597365 to 597368, EPI_ISL_597371 to 597375, EPI_ISL_597377 to 597378, EPI_ISL_597380 to 597381, EPI_ISL_597388, EPI_ISL_597390, EPI_ISL_597392, EPI_ISL_597396 to 597399, EPI_ISL_597401 to 597403, EPI_ISL_597405, EPI_ISL_597412, EPI_ISL_597414, EPI_ISL_597418, EPI_ISL_597424 to 597428, EPI_ISL_597432, EPI_ISL_597435, EPI_ISL_597437 to 597438, EPI_ISL_597441, EPI_ISL_597445 to 597446, EPI_ISL_597449 to 597451, EPI_ISL_597453, EPI_ISL_597458 to 597462, EPI_ISL_597464, EPI_ISL_597466 to 597470, EPI_ISL_597472 to 597473, EPI_ISL_597476 to 597480, EPI_ISL_597483, EPI_ISL_597487 to 597490, EPI_ISL_597494 to 597495, EPI_ISL_597497, EPI_ISL_597502, EPI_ISL_597504 to 597505, EPI_ISL_597507 to 597508, EPI_ISL_597510, EPI_ISL_597512, EPI_ISL_597517, EPI_ISL_597519 to 597520, EPI_ISL_601493, EPI_ISL_601495, EPI_ISL_601499, EPI_ISL_601501, EPI_ISL_601506, EPI_ISL_601515, EPI_ISL_601522, EPI_ISL_601530, EPI_ISL_601541, EPI_ISL_601547, EPI_ISL_601549 to 601550, EPI_ISL_601555, EPI_ISL_601567, EPI_ISL_601569, EPI_ISL_601571, EPI_ISL_601578 to 601580, EPI_ISL_601585, EPI_ISL_601588 to 601591, EPI_ISL_601594, EPI_ISL_601604, EPI_ISL_601606, EPI_ISL_601611, EPI_ISL_601615, EPI_ISL_601617 to 601619, EPI_ISL_601622, EPI_ISL_601624, EPI_ISL_601627 to 601628, EPI_ISL_601634, EPI_ISL_601639, EPI_ISL_601641, EPI_ISL_601648, EPI_ISL_601650, EPI_ISL_601657, EPI_ISL_601663 to 601664, EPI_ISL_601666, EPI_ISL_601668, EPI_ISL_601673, EPI_ISL_601682, EPI_ISL_601685, EPI_ISL_601690 to 601691, EPI_ISL_601700, EPI_ISL_601704, EPI_ISL_601709, EPI_ISL_601715, EPI_ISL_601728, EPI_ISL_601730, EPI_ISL_601733, EPI_ISL_601740, EPI_ISL_601747 to 601748, EPI_ISL_601764 to 601766, EPI_ISL_601768, EPI_ISL_601771, EPI_ISL_601778, EPI_ISL_601783, EPI_ISL_601785, EPI_ISL_602154, EPI_ISL_608348 to 608349, EPI_ISL_608353 to 608354, EPI_ISL_608357, EPI_ISL_608359 to 608360, EPI_ISL_608363, EPI_ISL_608368, EPI_ISL_608370, EPI_ISL_608373, EPI_ISL_608381 to 608382, EPI_ISL_608388 to 608389, EPI_ISL_608391 to 608398, EPI_ISL_608400, EPI_ISL_608402, EPI_ISL_608404, EPI_ISL_608407 to 608408, EPI_ISL_608410 to 608412, EPI_ISL_608416 to 608417, EPI_ISL_608423 to 608424, EPI_ISL_608426 to 608430, EPI_ISL_608432 to 608433, EPI_ISL_608436 to 608437, EPI_ISL_608439, EPI_ISL_608442, EPI_ISL_608446 to 608447, EPI_ISL_608449 to 608451, EPI_ISL_608456, EPI_ISL_608458 to 608459, EPI_ISL_608463 to 608464, EPI_ISL_608466 to 608467, EPI_ISL_608471 to 608472, EPI_ISL_608475 to 608478, EPI_ISL_608480, EPI_ISL_608482, EPI_ISL_608484 to 608486, EPI_ISL_608490, EPI_ISL_608492 to 608495, EPI_ISL_608497 to 608498, EPI_ISL_608501, EPI_ISL_608504, EPI_ISL_608506 to 608510, EPI_ISL_608514 to 608515, EPI_ISL_608521 to 608525, EPI_ISL_608527, EPI_ISL_608529 to 608532, EPI_ISL_608535 to 608538, EPI_ISL_608540, EPI_ISL_608544 to 608547, EPI_ISL_608550, EPI_ISL_608552, EPI_ISL_608557 to 608561, EPI_ISL_608565 to 608566, EPI_ISL_608568, EPI_ISL_608570 to 608573, EPI_ISL_608576 to 608578, EPI_ISL_608585, EPI_ISL_608588, EPI_ISL_608596 to 608598, EPI_ISL_608602, EPI_ISL_608604, EPI_ISL_608607 to 608608, EPI_ISL_608611 to 608613, EPI_ISL_608615 to 608616, EPI_ISL_608618 to 608620, EPI_ISL_608624, EPI_ISL_608626 to 608629, EPI_ISL_608631 to 608633, EPI_ISL_608636 to 608638, EPI_ISL_608641 to 608645, EPI_ISL_608650 to 608651, EPI_ISL_608653 to 608654, EPI_ISL_608656, EPI_ISL_608660, EPI_ISL_608664, EPI_ISL_608666, EPI_ISL_608671, EPI_ISL_608673, EPI_ISL_608678 to 608679, EPI_ISL_608682 to 608683, EPI_ISL_608688 to 608689, EPI_ISL_608693, EPI_ISL_608695 to 608696, EPI_ISL_608845, EPI_ISL_608852, EPI_ISL_608867 to 608868, EPI_ISL_608873, EPI_ISL_608875 to 608876, EPI_ISL_608879, EPI_ISL_608881, EPI_ISL_608892, EPI_ISL_608902, EPI_ISL_608906, EPI_ISL_608908, EPI_ISL_608910, EPI_ISL_608912, EPI_ISL_608918, EPI_ISL_608923 to 608924, EPI_ISL_608927, EPI_ISL_608935, EPI_ISL_608937, EPI_ISL_608939, EPI_ISL_608943, EPI_ISL_608947, EPI_ISL_608949, EPI_ISL_608953, EPI_ISL_608958 to 608960, EPI_ISL_608965, EPI_ISL_608967 to 608969, EPI_ISL_608974, EPI_ISL_608982, EPI_ISL_608987, EPI_ISL_608990, EPI_ISL_608996 to 608997, EPI_ISL_609003, EPI_ISL_609005, EPI_ISL_609014, EPI_ISL_609018, EPI_ISL_609023, EPI_ISL_609025, EPI_ISL_609033, EPI_ISL_609037, EPI_ISL_609045, EPI_ISL_609048, EPI_ISL_609050, EPI_ISL_609054, EPI_ISL_609067, EPI_ISL_609072, EPI_ISL_609077, EPI_ISL_609081 to 609082, EPI_ISL_609086, EPI_ISL_609088, EPI_ISL_609091, EPI_ISL_609097, EPI_ISL_609099 to 609101, EPI_ISL_609111, EPI_ISL_609113 to 609114, EPI_ISL_609118 to 609119, EPI_ISL_609122 to 609123, EPI_ISL_609129, EPI_ISL_609131, EPI_ISL_609142 to 609143, EPI_ISL_609145, EPI_ISL_609156, EPI_ISL_609158 to 609177, EPI_ISL_609180 to 609181, EPI_ISL_609183, EPI_ISL_609188, EPI_ISL_609193, EPI_ISL_609195 to 609196, EPI_ISL_609200 to 609201, EPI_ISL_609203 to 609205, EPI_ISL_609308 to 609377, EPI_ISL_609379 to 609392, EPI_ISL_609394 to 609449, EPI_ISL_609451 to 609471, EPI_ISL_609473 to 609526, EPI_ISL_609528 to 609533, EPI_ISL_609535 to 609599, EPI_ISL_609601 to 609745, EPI_ISL_609747 to 609800, EPI_ISL_610629 to 610701, EPI_ISL_610703 to 610778, EPI_ISL_610780 to 610842, EPI_ISL_610844 to 610921

see above	Lighthouse Lab in Cambridge	Wellcome Sanger Institute for the COVID-19 Genomics UK (COG-UK) consortium	Cordelia Langford; David K. Jackson; Dominic Kwiatkowski; Ewan Harrison; Ian Johnston; John Sillitoe on behalf of the Wellcome Sanger Institute COVID-19 Surveillance Team; John Sillitoe on behalf of the Wellcome Sanger Institute COVID-19 Surveillance Team (http://www.sanger.ac.uk/covid-team); Rob Howes; Roberto Amato; Sonia Goncalves; The Lighthouse Lab in Cambridge and Alex Alderton

EPI_ISL_530482, EPI_ISL_530899, EPI_ISL_530963, EPI_ISL_531211, EPI_ISL_531688, EPI_ISL_531872, EPI_ISL_532012, EPI_ISL_532294, EPI_ISL_532511, EPI_ISL_532583, EPI_ISL_532851, EPI_ISL_533086, EPI_ISL_533403, EPI_ISL_536889, EPI_ISL_537017, EPI_ISL_539906, EPI_ISL_540030, EPI_ISL_540278, EPI_ISL_540281, EPI_ISL_540351, EPI_ISL_549772, EPI_ISL_549784, EPI_ISL_567024, EPI_ISL_567059, EPI_ISL_567184, EPI_ISL_567202, EPI_ISL_567231, EPI_ISL_568301, EPI_ISL_568315, EPI_ISL_568317, EPI_ISL_568344, EPI_ISL_568392, EPI_ISL_568398, EPI_ISL_576050, EPI_ISL_576064, EPI_ISL_576088, EPI_ISL_579794, EPI_ISL_579835, EPI_ISL_579843, EPI_ISL_580909, EPI_ISL_581045, EPI_ISL_581359, EPI_ISL_587303, EPI_ISL_587386, EPI_ISL_587477, EPI_ISL_588075, EPI_ISL_588125, EPI_ISL_588128, EPI_ISL_588175, EPI_ISL_588496, EPI_ISL_588541, EPI_ISL_588572, EPI_ISL_588674, EPI_ISL_588717, EPI_ISL_588844 to 588845, EPI_ISL_588927, EPI_ISL_588940, EPI_ISL_588962, EPI_ISL_589248, EPI_ISL_589936, EPI_ISL_590046, EPI_ISL_590497, EPI_ISL_598970, EPI_ISL_599010, EPI_ISL_599077, EPI_ISL_599166, EPI_ISL_599183, EPI_ISL_599194, EPI_ISL_599201, EPI_ISL_599270, EPI_ISL_599314, EPI_ISL_599590, EPI_ISL_599627, EPI_ISL_599816, EPI_ISL_600139, EPI_ISL_600221, EPI_ISL_600643, EPI_ISL_600824, EPI_ISL_600904, EPI_ISL_601022, EPI_ISL_601150, EPI_ISL_601192, EPI_ISL_601214, EPI_ISL_601262, EPI_ISL_601366, EPI_ISL_601797, EPI_ISL_601834, EPI_ISL_601950, EPI_ISL_602006, EPI_ISL_602015, EPI_ISL_602077, EPI_ISL_602083, EPI_ISL_602090 to 602091, EPI_ISL_602102, EPI_ISL_602106, EPI_ISL_602109, EPI_ISL_602122, EPI_ISL_609187, EPI_ISL_609192, EPI_ISL_611304, EPI_ISL_611378, EPI_ISL_1389622 to 1390312, EPI_ISL_1390320 to 1390376, EPI_ISL_1408909, EPI_ISL_1408915, EPI_ISL_1408918, EPI_ISL_1408921 to 1408922, EPI_ISL_1408928 to 1408929, EPI_ISL_1408934, EPI_ISL_1408937, EPI_ISL_1408939 to 1408940, EPI_ISL_1408944 to 1408947, EPI_ISL_1408950, EPI_ISL_1408954, EPI_ISL_1408962 to 1408963, EPI_ISL_1408965, EPI_ISL_1408968 to 1408969, EPI_ISL_1408972, EPI_ISL_1408974, EPI_ISL_1408978, EPI_ISL_1408980 to 1408983, EPI_ISL_1408986, EPI_ISL_1408992, EPI_ISL_1408996 to 1408997, EPI_ISL_1409000, EPI_ISL_1409002, EPI_ISL_1409005, EPI_ISL_1409008 to 1409010, EPI_ISL_1409012 to 1409015, EPI_ISL_1409017 to 1409018, EPI_ISL_1409020 to 1409021, EPI_ISL_1409024, EPI_ISL_1409027, EPI_ISL_1409029, EPI_ISL_1409032, EPI_ISL_1409034, EPI_ISL_1409037 to 1409040, EPI_ISL_1409043 to 1409044, EPI_ISL_1409047 to 1409051, EPI_ISL_1409055, EPI_ISL_1409063, EPI_ISL_1409066, EPI_ISL_1409068 to 1409069, EPI_ISL_1409071, EPI_ISL_1409073 to 1409074, EPI_ISL_1409076, EPI_ISL_1409078, EPI_ISL_1409080 to 1409081, EPI_ISL_1409085, EPI_ISL_1409087 to 1409088, EPI_ISL_1409090 to 1409091, EPI_ISL_1409095 to 1409097, EPI_ISL_1409099 to 1409100, EPI_ISL_1409103, EPI_ISL_1409109 to 1409110, EPI_ISL_1409113, EPI_ISL_1409115 to 1409118, EPI_ISL_1409123, EPI_ISL_1409129 to 1409131, EPI_ISL_1409136 to 1409138, EPI_ISL_1409142 to 1409145, EPI_ISL_1409148, EPI_ISL_1409155 to 1409157, EPI_ISL_1409159 to 1409161, EPI_ISL_1409163, EPI_ISL_1409165, EPI_ISL_1409170, EPI_ISL_1409172 to 1409174, EPI_ISL_1409178, EPI_ISL_1409181 to 1409182, EPI_ISL_1409184, EPI_ISL_1409198 to 1409199, EPI_ISL_1409204 to 1409205, EPI_ISL_1409210, EPI_ISL_1409214, EPI_ISL_1409220 to 1409221, EPI_ISL_1409225, EPI_ISL_1409231, EPI_ISL_1409233 to 1409234, EPI_ISL_1409237, EPI_ISL_1409247, EPI_ISL_1409256, EPI_ISL_1409261, EPI_ISL_1409263 to 1409264, EPI_ISL_1409270, EPI_ISL_1409277, EPI_ISL_1409280, EPI_ISL_1409309, EPI_ISL_1409314, EPI_ISL_1409318, EPI_ISL_1409324, EPI_ISL_1409327, EPI_ISL_1409331 to 1409332, EPI_ISL_1409336, EPI_ISL_1409342, EPI_ISL_1409344 to 1409345, EPI_ISL_1409347, EPI_ISL_1409352, EPI_ISL_1409354, EPI_ISL_1409357 to 1409358, EPI_ISL_1409361, EPI_ISL_1409366, EPI_ISL_1409370 to 1409371, EPI_ISL_1409377, EPI_ISL_1409379, EPI_ISL_1409393, EPI_ISL_1409396, EPI_ISL_1409399, EPI_ISL_1409403, EPI_ISL_1409406 to 1409407, EPI_ISL_1409414, EPI_ISL_1409421, EPI_ISL_1409429, EPI_ISL_1409441, EPI_ISL_1409444, EPI_ISL_1409457 to 1409458, EPI_ISL_1409461, EPI_ISL_1409468 to 1409469, EPI_ISL_1409471, EPI_ISL_1409477, EPI_ISL_1409484, EPI_ISL_1409494, EPI_ISL_1409516, EPI_ISL_1409518, EPI_ISL_1409524, EPI_ISL_1409529, EPI_ISL_1409531 to 1409532, EPI_ISL_1409539, EPI_ISL_1409547, EPI_ISL_1409550 to 1409551, EPI_ISL_1409557, EPI_ISL_1409572, EPI_ISL_1409577, EPI_ISL_1409579, EPI_ISL_1409583, EPI_ISL_1409593 to 1409594, EPI_ISL_1409596 to 1409597, EPI_ISL_1409604, EPI_ISL_1409607, EPI_ISL_1409609, EPI_ISL_1409611 to 1409612, EPI_ISL_1409615, EPI_ISL_1409624, EPI_ISL_1409632, EPI_ISL_1409634, EPI_ISL_1409638, EPI_ISL_1409642, EPI_ISL_1409652, EPI_ISL_1409654, EPI_ISL_1409663 to 1409665, EPI_ISL_1409669 to 1409670, EPI_ISL_1409685, EPI_ISL_1409689, EPI_ISL_1409691 to 1409692, EPI_ISL_1409717, EPI_ISL_1409723, EPI_ISL_1409727, EPI_ISL_1409733, EPI_ISL_1409736, EPI_ISL_1409738, EPI_ISL_1409754, EPI_ISL_1409758, EPI_ISL_1409771, EPI_ISL_1409774, EPI_ISL_1409778, EPI_ISL_1409783, EPI_ISL_1409794, EPI_ISL_1409809, EPI_ISL_1409813, EPI_ISL_1409817, EPI_ISL_1409836, EPI_ISL_1409843, EPI_ISL_1409858 to 1409859, EPI_ISL_1409861, EPI_ISL_1409863, EPI_ISL_1409868, EPI_ISL_1409870, EPI_ISL_1409873, EPI_ISL_1409875, EPI_ISL_1409883, EPI_ISL_1409886, EPI_ISL_1409889, EPI_ISL_1409891, EPI_ISL_1409894, EPI_ISL_1409906 to 1409907, EPI_ISL_1409912 to 1409913, EPI_ISL_1409918, EPI_ISL_1409926, EPI_ISL_1409932, EPI_ISL_1409935, EPI_ISL_1409938, EPI_ISL_1409951, EPI_ISL_1409960 to 1409961, EPI_ISL_1409967, EPI_ISL_1409970, EPI_ISL_1409976, EPI_ISL_1409981, EPI_ISL_1409987, EPI_ISL_1409993, EPI_ISL_1409996, EPI_ISL_1410001 to 1410002, EPI_ISL_1410006, EPI_ISL_1410009, EPI_ISL_1410013, EPI_ISL_1410032, EPI_ISL_1410037, EPI_ISL_1410046 to 1410048, EPI_ISL_1410051, EPI_ISL_1410053, EPI_ISL_1410056, EPI_ISL_1410067, EPI_ISL_1410071, EPI_ISL_1410073, EPI_ISL_1410076, EPI_ISL_1410289 to 1410293, EPI_ISL_1410295, EPI_ISL_1410297 to 1410300, EPI_ISL_1410304, EPI_ISL_1410306, EPI_ISL_1410314 to 1410316, EPI_ISL_1410318, EPI_ISL_1410320, EPI_ISL_1410322 to 1410328, EPI_ISL_1410330, EPI_ISL_1410333 to 1410334, EPI_ISL_1410338, EPI_ISL_1410341 to 1410342, EPI_ISL_1410345, EPI_ISL_1410347, EPI_ISL_1410349, EPI_ISL_1410355 to 1410358, EPI_ISL_1410360 to 1410361, EPI_ISL_1410364, EPI_ISL_1410366, EPI_ISL_1410368, EPI_ISL_1410373 to 1410375, EPI_ISL_1410383 to 1410385, EPI_ISL_1410387, EPI_ISL_1410390 to 1410391, EPI_ISL_1410394 to 1410397, EPI_ISL_1410400, EPI_ISL_1410403, EPI_ISL_1410406, EPI_ISL_1410409 to 1410410, EPI_ISL_1410412 to 1410413, EPI_ISL_1410415, EPI_ISL_1410417, EPI_ISL_1410421 to 1410422, EPI_ISL_1410424 to 1410428, EPI_ISL_1410432 to 1410435, EPI_ISL_1410437, EPI_ISL_1410441, EPI_ISL_1410448, EPI_ISL_1410450 to 1410451, EPI_ISL_1410453, EPI_ISL_1410459 to 1410462, EPI_ISL_1410464 to 1410465, EPI_ISL_1410467, EPI_ISL_1410469, EPI_ISL_1410471, EPI_ISL_1410473, EPI_ISL_1410475 to 1410477, EPI_ISL_1410480, EPI_ISL_1410482 to 1410496, EPI_ISL_1410499, EPI_ISL_1410501 to 1410502, EPI_ISL_1410507 to 1410509, EPI_ISL_1410511, EPI_ISL_1410514 to 1410519, EPI_ISL_1410521 to 1410522, EPI_ISL_1410525 to 1410526, EPI_ISL_1410528 to 1410529, EPI_ISL_1410534 to 1410537, EPI_ISL_1410539, EPI_ISL_1410544, EPI_ISL_1410546 to 1410547, EPI_ISL_1410552, EPI_ISL_1410555, EPI_ISL_1410562, EPI_ISL_1410567 to 1410568, EPI_ISL_1410572, EPI_ISL_1410576, EPI_ISL_1410578, EPI_ISL_1410581, EPI_ISL_1410583 to 1410584, EPI_ISL_1410586, EPI_ISL_1410589, EPI_ISL_1410599, EPI_ISL_1410601, EPI_ISL_1410604, EPI_ISL_1410606, EPI_ISL_1410609 to 1410610, EPI_ISL_1410614, EPI_ISL_1410617, EPI_ISL_1410619, EPI_ISL_1410623, EPI_ISL_1410633, EPI_ISL_1410635 to 1410636, EPI_ISL_1410638, EPI_ISL_1410647, EPI_ISL_1410654, EPI_ISL_1410656 to 1410657, EPI_ISL_1410661, EPI_ISL_1410673, EPI_ISL_1410675 to 1410676, EPI_ISL_1410684, EPI_ISL_1410686, EPI_ISL_1410692, EPI_ISL_1410694 to 1410695, EPI_ISL_1410697, EPI_ISL_1410700, EPI_ISL_1410702 to 1410703, EPI_ISL_1410711, EPI_ISL_1410716,

EPI_ISL_1410719, EPI_ISL_1410721 to 1410722, EPI_ISL_1410726, EPI_ISL_1410730, EPI_ISL_1410734 to 1410735, EPI_ISL_1410737, EPI_ISL_1410744 to 1410745, EPI_ISL_1410747, EPI_ISL_1410750, EPI_ISL_1410755, EPI_ISL_1410759 to 1410760, EPI_ISL_1410773, EPI_ISL_1410778, EPI_ISL_1410784, EPI_ISL_1410787 to 1410788, EPI_ISL_1410790 to 1410792, EPI_ISL_1410794 to 1410795, EPI_ISL_1410797, EPI_ISL_1410799 to 1410801, EPI_ISL_1410807, EPI_ISL_1410810 to 1410814, EPI_ISL_1410819 to 1410820, EPI_ISL_1410822, EPI_ISL_1410827, EPI_ISL_1410829, EPI_ISL_1410833, EPI_ISL_1410835, EPI_ISL_1410839 to 1410845, EPI_ISL_1410847, EPI_ISL_1410849 to 1410850, EPI_ISL_1410853 to 1410854, EPI_ISL_1410856, EPI_ISL_1410858, EPI_ISL_1410861, EPI_ISL_1410864 to 1410865, EPI_ISL_1410867, EPI_ISL_1410873, EPI_ISL_1410878 to 1410882, EPI_ISL_1410884, EPI_ISL_1410890, EPI_ISL_1410892, EPI_ISL_1410895, EPI_ISL_1410898, EPI_ISL_1410900, EPI_ISL_1410903, EPI_ISL_1410905 to 1410906, EPI_ISL_1410908 to 1410909, EPI_ISL_1410912, EPI_ISL_1410914, EPI_ISL_1410916 to 1410917, EPI_ISL_1410920 to 1410923, EPI_ISL_1410925 to 1410927, EPI_ISL_1410929, EPI_ISL_1410932 to 1410934, EPI_ISL_1410936 to 1410937, EPI_ISL_1410939, EPI_ISL_1410941, EPI_ISL_1410943, EPI_ISL_1410945, EPI_ISL_1410948, EPI_ISL_1410950, EPI_ISL_1410952, EPI_ISL_1410955 to 1410956, EPI_ISL_1410959 to 1410960, EPI_ISL_1410964 to 1410970, EPI_ISL_1410975 to 1410976, EPI_ISL_1410978 to 1410980, EPI_ISL_1410982, EPI_ISL_1410984, EPI_ISL_1410989, EPI_ISL_1410991 to 1410993, EPI_ISL_1410996, EPI_ISL_1410998, EPI_ISL_1411003, EPI_ISL_1411008 to 1411009, EPI_ISL_1411011 to 1411014, EPI_ISL_1411016 to 1411018, EPI_ISL_1411020 to 1411021, EPI_ISL_1411023, EPI_ISL_1411025 to 1411027, EPI_ISL_1411031, EPI_ISL_1411033 to 1411034, EPI_ISL_1411040, EPI_ISL_1411042 to 1411043, EPI_ISL_1411046 to 1411050, EPI_ISL_1411055, EPI_ISL_1411057, EPI_ISL_1411059, EPI_ISL_1411063 to 1411066, EPI_ISL_1411068, EPI_ISL_1411074, EPI_ISL_1411076 to 1411079, EPI_ISL_1411081 to 1411084, EPI_ISL_1411087 to 1411088, EPI_ISL_1411090, EPI_ISL_1411092 to 1411093, EPI_ISL_1411095 to 1411099, EPI_ISL_1411103 to 1411104, EPI_ISL_1411106 to 1411111, EPI_ISL_1411117, EPI_ISL_1411120 to 1411121, EPI_ISL_1411126, EPI_ISL_1411129 to 1411130, EPI_ISL_1411132, EPI_ISL_1411134, EPI_ISL_1411137, EPI_ISL_1411139 to 1411141, EPI_ISL_1411143 to 1411146, EPI_ISL_1411148 to 1411149, EPI_ISL_1411151 to 1411152, EPI_ISL_1411155, EPI_ISL_1411157, EPI_ISL_1411160, EPI_ISL_1411162, EPI_ISL_1411164 to 1411167, EPI_ISL_1411169, EPI_ISL_1411173, EPI_ISL_1411175 to 1411178, EPI_ISL_1411181 to 1411182, EPI_ISL_1411186 to 1411188, EPI_ISL_1411191 to 1411193, EPI_ISL_1411198 to 1411203, EPI_ISL_1411206, EPI_ISL_1411208, EPI_ISL_1411210, EPI_ISL_1411212, EPI_ISL_1411214 to 1411217, EPI_ISL_1411221 to 1411222, EPI_ISL_1411224, EPI_ISL_1411226 to 1411228, EPI_ISL_1411235, EPI_ISL_1411241 to 1411242, EPI_ISL_1411247 to 1411249, EPI_ISL_1411251 to 1411255, EPI_ISL_1411257, EPI_ISL_1411259, EPI_ISL_1411262 to 1411265, EPI_ISL_1411269 to 1411271, EPI_ISL_1411275 to 1411277, EPI_ISL_1411281, EPI_ISL_1411284, EPI_ISL_1411288, EPI_ISL_1411290 to 1411294, EPI_ISL_1411298, EPI_ISL_1411300 to 1411301, EPI_ISL_1411305, EPI_ISL_1411307, EPI_ISL_1411312, EPI_ISL_1411314, EPI_ISL_1411318, EPI_ISL_1411321, EPI_ISL_1411323 to 1411324, EPI_ISL_1411326 to 1411327, EPI_ISL_1411330 to 1411333, EPI_ISL_1411336, EPI_ISL_1411338, EPI_ISL_1411340 to 1411344, EPI_ISL_1411347, EPI_ISL_1411349 to 1411351, EPI_ISL_1411353 to 1411355, EPI_ISL_1411359, EPI_ISL_1411361, EPI_ISL_1411363 to 1411364, EPI_ISL_1411366 to 1411368, EPI_ISL_1411373, EPI_ISL_1411376, EPI_ISL_1411381, EPI_ISL_1411383, EPI_ISL_1411388, EPI_ISL_1411390, EPI_ISL_1411392, EPI_ISL_1411396 to 1411399, EPI_ISL_1411403, EPI_ISL_1411407 to 1411408, EPI_ISL_1411410, EPI_ISL_1411414, EPI_ISL_1411417 to 1411418, EPI_ISL_1411420 to 1411421, EPI_ISL_1411427, EPI_ISL_1411431, EPI_ISL_1411433 to 1411435, EPI_ISL_1411439, EPI_ISL_1411442 to 1411444, EPI_ISL_1411447, EPI_ISL_1411449 to 1411451, EPI_ISL_1411453, EPI_ISL_1411460 to 1411461, EPI_ISL_1411463, EPI_ISL_1411466, EPI_ISL_1411469, EPI_ISL_1411474 to 1411475, EPI_ISL_1411478 to 1411479, EPI_ISL_1411484, EPI_ISL_1411486, EPI_ISL_1411494, EPI_ISL_1411499, EPI_ISL_1411502, EPI_ISL_1411507, EPI_ISL_1411563, EPI_ISL_1411572, EPI_ISL_1411577, EPI_ISL_1411596, EPI_ISL_1411603, EPI_ISL_1411607 to 1411608, EPI_ISL_1411621, EPI_ISL_1411630, EPI_ISL_1411635, EPI_ISL_1411638 to 1411641, EPI_ISL_1411643, EPI_ISL_1411646 to 1411647, EPI_ISL_1411649, EPI_ISL_1411661, EPI_ISL_1411666, EPI_ISL_1411668, EPI_ISL_1411672, EPI_ISL_1411677, EPI_ISL_1411679, EPI_ISL_1411681, EPI_ISL_1411685 to 1411686, EPI_ISL_1411690, EPI_ISL_1411693, EPI_ISL_1411696, EPI_ISL_1411706, EPI_ISL_1411713 to 1411715, EPI_ISL_1411718 to 1411719, EPI_ISL_1411721 to 1411723, EPI_ISL_1411736, EPI_ISL_1411738 to 1411739, EPI_ISL_1411743 to 1411744, EPI_ISL_1411750 to 1411752, EPI_ISL_1411761, EPI_ISL_1411768, EPI_ISL_1411772, EPI_ISL_1411776, EPI_ISL_1411778, EPI_ISL_1411784 to 1411785, EPI_ISL_1411795 to 1411796, EPI_ISL_1411798 to 1411799, EPI_ISL_1411803, EPI_ISL_1411807, EPI_ISL_1411812, EPI_ISL_1411814 to 1411815, EPI_ISL_1411817, EPI_ISL_1411829, EPI_ISL_1411833 to 1411834, EPI_ISL_1411836 to 1411838, EPI_ISL_1411841 to 1411842, EPI_ISL_1411861, EPI_ISL_1411871 to 1411873, EPI_ISL_1411876, EPI_ISL_1411879, EPI_ISL_1411881, EPI_ISL_1411883, EPI_ISL_1411885, EPI_ISL_1411887, EPI_ISL_1411890 to 1411892, EPI_ISL_1411894, EPI_ISL_1411909, EPI_ISL_1411912, EPI_ISL_1411914 to 1411915, EPI_ISL_1411917 to 1411918, EPI_ISL_1411927, EPI_ISL_1411932, EPI_ISL_1411940, EPI_ISL_1411946, EPI_ISL_1411952, EPI_ISL_1411957 to 1411958, EPI_ISL_1411971 to 1411972, EPI_ISL_1411989, EPI_ISL_1411997, EPI_ISL_1411999, EPI_ISL_1412005, EPI_ISL_1412007, EPI_ISL_1412011, EPI_ISL_1412016, EPI_ISL_1412022 to 1412023, EPI_ISL_1412026, EPI_ISL_1412031, EPI_ISL_1412035, EPI_ISL_1412037, EPI_ISL_1412039 to 1412040, EPI_ISL_1412043 to 1412045, EPI_ISL_1412047, EPI_ISL_1412049, EPI_ISL_1412051 to 1412052, EPI_ISL_1412055, EPI_ISL_1412059, EPI_ISL_1412070 to 1412071, EPI_ISL_1412075 to 1412078, EPI_ISL_1412086, EPI_ISL_1412091 to 1412093, EPI_ISL_1412098 to 1412099, EPI_ISL_1412102, EPI_ISL_1412112, EPI_ISL_1412114, EPI_ISL_1412117, EPI_ISL_1412123 to 1412124, EPI_ISL_1412129 to 1412130, EPI_ISL_1412135 to 1412136, EPI_ISL_1412139 to 1412140, EPI_ISL_1412142, EPI_ISL_1412144, EPI_ISL_1412147, EPI_ISL_1412152, EPI_ISL_1412157, EPI_ISL_1412161, EPI_ISL_1412163, EPI_ISL_1412169 to 1412170, EPI_ISL_1412173, EPI_ISL_1412176, EPI_ISL_1412181, EPI_ISL_1412183, EPI_ISL_1412186, EPI_ISL_1412235, EPI_ISL_1412260, EPI_ISL_1412264, EPI_ISL_1412288, EPI_ISL_1412325, EPI_ISL_1412330, EPI_ISL_1412356, EPI_ISL_1412358, EPI_ISL_1412372, EPI_ISL_1412376, EPI_ISL_1412383, EPI_ISL_1412399, EPI_ISL_1412414, EPI_ISL_1412416, EPI_ISL_1412431, EPI_ISL_1412449, EPI_ISL_1412463, EPI_ISL_1412466, EPI_ISL_1412476, EPI_ISL_1412495, EPI_ISL_1412509, EPI_ISL_1412545, EPI_ISL_1412550, EPI_ISL_1412568 to 1412573, EPI_ISL_1412575 to 1412576, EPI_ISL_1412578, EPI_ISL_1412580 to 1412587, EPI_ISL_1412589, EPI_ISL_1412594 to 1412595, EPI_ISL_1412597 to 1412598, EPI_ISL_1412600, EPI_ISL_1412602, EPI_ISL_1412605, EPI_ISL_1412608 to 1412609, EPI_ISL_1412612, EPI_ISL_1412614 to 1412617, EPI_ISL_1412621 to 1412622, EPI_ISL_1412624 to 1412626, EPI_ISL_1412628 to 1412630, EPI_ISL_1412632, EPI_ISL_1412637, EPI_ISL_1412640 to 1412641, EPI_ISL_1412643 to 1412644, EPI_ISL_1412646 to 1412648, EPI_ISL_1412650, EPI_ISL_1412654, EPI_ISL_1412657 to 1412659, EPI_ISL_1412662 to 1412664, EPI_ISL_1412666, EPI_ISL_1412672 to 1412676, EPI_ISL_1412678, EPI_ISL_1412682 to 1412683, EPI_ISL_1412688, EPI_ISL_1412690 to 1412694, EPI_ISL_1412698, EPI_ISL_1412701 to 1412706, EPI_ISL_1412708, EPI_ISL_1412711 to 1412713, EPI_ISL_1412715, EPI_ISL_1412717, EPI_ISL_1412719 to 1412720, EPI_ISL_1412722 to 1412725, EPI_ISL_1412727, EPI_ISL_1412729, EPI_ISL_1412734 to 1412736, EPI_ISL_1412739 to 1412741, EPI_ISL_1412743 to 1412745, EPI_ISL_1412747 to 1412748, EPI_ISL_1412751, EPI_ISL_1412754 to 1412755, EPI_ISL_1412757, EPI_ISL_1412762 to 1412765, EPI_ISL_1412767 to 1412768, EPI_ISL_1412771, EPI_ISL_1412775 to 1412777, EPI_ISL_1412779, EPI_ISL_1412781 to 1412783, EPI_ISL_1412786 to 1412787, EPI_ISL_1412790, EPI_ISL_1412792 to 1412794, EPI_ISL_1412797 to 1412798, EPI_ISL_1412802, EPI_ISL_1412804 to 1412807, EPI_ISL_1412809, EPI_ISL_1412811 to 1412813, EPI_ISL_1412816 to 1412817, EPI_ISL_1412820, EPI_ISL_1412823, EPI_ISL_1412827 to 1412828, EPI_ISL_1412830 to 1412831, EPI_ISL_1412834, EPI_ISL_1412837, EPI_ISL_1412846 to 1412848, EPI_ISL_1412852, EPI_ISL_1412857 to 1412859, EPI_ISL_1412861, EPI_ISL_1412863, EPI_ISL_1412865 to 1412868, EPI_ISL_1412875 to 1412877, EPI_ISL_1412879, EPI_ISL_1412881, EPI_ISL_1412883, EPI_ISL_1412885, EPI_ISL_1412889, EPI_ISL_1412891, EPI_ISL_1412893 to 1412894, EPI_ISL_1412898 to 1412899, EPI_ISL_1412901 to 1412903, EPI_ISL_1412909, EPI_ISL_1412914, EPI_ISL_1412916, EPI_ISL_1412918, EPI_ISL_1412920 to 1412922, EPI_ISL_1412924, EPI_ISL_1413062 to 1413063, EPI_ISL_1413065, EPI_ISL_1413068 to 1413072, EPI_ISL_1413075 to 1413077, EPI_ISL_1413080 to 1413081, EPI_ISL_1413083, EPI_ISL_1413085, EPI_ISL_1413087 to 1413089, EPI_ISL_1413092, EPI_ISL_1413094 to 1413095, EPI_ISL_1413097 to 1413098, EPI_ISL_1413100 to 1413103, EPI_ISL_1413105 to 1413106, EPI_ISL_1413108 to 1413110, EPI_ISL_1413112 to 1413113, EPI_ISL_1413121 to 1413122, EPI_ISL_1413124, EPI_ISL_1413126, EPI_ISL_1413130, EPI_ISL_1413135, EPI_ISL_1413137, EPI_ISL_1413143, EPI_ISL_1413148, EPI_ISL_1413151, EPI_ISL_1413153, EPI_ISL_1413158 to 1413159, EPI_ISL_1413161 to 1413162, EPI_ISL_1413166, EPI_ISL_1413170, EPI_ISL_1413172, EPI_ISL_1413175, EPI_ISL_1413177, EPI_ISL_1413180, EPI_ISL_1413188, EPI_ISL_1413190, EPI_ISL_1413192, EPI_ISL_1413194, EPI_ISL_1413198, EPI_ISL_1413200 to 1413201, EPI_ISL_1413205, EPI_ISL_1413211, EPI_ISL_1413217, EPI_ISL_1413219 to 1413220, EPI_ISL_1413228, EPI_ISL_1413230, EPI_ISL_1413233, EPI_ISL_1413237, EPI_ISL_1413242, EPI_ISL_1413244, EPI_ISL_1413247, EPI_ISL_1413252, EPI_ISL_1413256, EPI_ISL_1413259, EPI_ISL_1413262, EPI_ISL_1413264, EPI_ISL_1413267, EPI_ISL_1413271, EPI_ISL_1413275 to 1413276, EPI_ISL_1413279, EPI_ISL_1413283, EPI_ISL_1413288, EPI_ISL_1413291, EPI_ISL_1413294 to 1413295, EPI_ISL_1413298, EPI_ISL_1413303, EPI_ISL_1413310, EPI_ISL_1413312, EPI_ISL_1413318, EPI_ISL_1413320, EPI_ISL_1413324, EPI_ISL_1413326, EPI_ISL_1413331, EPI_ISL_1413334 to 1413335, EPI_ISL_1413338, EPI_ISL_1413340, EPI_ISL_1413342, EPI_ISL_1413346, EPI_ISL_1413354, EPI_ISL_1413362, EPI_ISL_1413364, EPI_ISL_1413367 to 1413368, EPI_ISL_1413378 to 1413379, EPI_ISL_1413386, EPI_ISL_1413391 to 1413392, EPI_ISL_1413394, EPI_ISL_1413397, EPI_ISL_1413400, EPI_ISL_1413403, EPI_ISL_1413405 to 1413406, EPI_ISL_1413408, EPI_ISL_1413411, EPI_ISL_1413414, EPI_ISL_1413422 to 1413423, EPI_ISL_1413425, EPI_ISL_1413430, EPI_ISL_1413432 to 1413433, EPI_ISL_1413435 to 1413436, EPI_ISL_1413440 to 1413441, EPI_ISL_1413448 to 1413449, EPI_ISL_1413451, EPI_ISL_1413453 to 1413454, EPI_ISL_1413456, EPI_ISL_1413467, EPI_ISL_1413469 to 1413470, EPI_ISL_1413475, EPI_ISL_1413477 to 1413478, EPI_ISL_1413483, EPI_ISL_1413486, EPI_ISL_1413489, EPI_ISL_1413491, EPI_ISL_1413494, EPI_ISL_1413497, EPI_ISL_1413499, EPI_ISL_1413508, EPI_ISL_1413512 to 1413513, EPI_ISL_1413515, EPI_ISL_1413520, EPI_ISL_1413523 to 1413524, EPI_ISL_1413527, EPI_ISL_1413529, EPI_ISL_1413531 to 1413532, EPI_ISL_1413534 to 1413535, EPI_ISL_1413537, EPI_ISL_1413540 to 1413541, EPI_ISL_1413546, EPI_ISL_1413553, EPI_ISL_1413557, EPI_ISL_1413564, EPI_ISL_1413569, EPI_ISL_1413572, EPI_ISL_1413575, EPI_ISL_1413580, EPI_ISL_1413583, EPI_ISL_1413588, EPI_ISL_1413590 to 1413591, EPI_ISL_1413593, EPI_ISL_1413596, EPI_ISL_1413598 to 1413599, EPI_ISL_1413601, EPI_ISL_1413603, EPI_ISL_1413608, EPI_ISL_1413611 to 1413612, EPI_ISL_1413614, EPI_ISL_1413616, EPI_ISL_1413619 to 1413620, EPI_ISL_1413622, EPI_ISL_1413624 to 1413625, EPI_ISL_1413627, EPI_ISL_1413630, EPI_ISL_1413632 to 1413633, EPI_ISL_1413635 to 1413636, EPI_ISL_1413638, EPI_ISL_1413641, EPI_ISL_1413643 to 1413644, EPI_ISL_1413646, EPI_ISL_1413648 to 1413650, EPI_ISL_1413652, EPI_ISL_1413654 to 1413655, EPI_ISL_1413657, EPI_ISL_1413659 to 1413660, EPI_ISL_1413662 to 1413663, EPI_ISL_1413665 to 1413666, EPI_ISL_1413670 to 1413671, EPI_ISL_1413674, EPI_ISL_1413676, EPI_ISL_1413681, EPI_ISL_1413683 to 1413684, EPI_ISL_1413686, EPI_ISL_1413688 to 1413689, EPI_ISL_1413695, EPI_ISL_1413697 to 1413698, EPI_ISL_1413700 to 1413701, EPI_ISL_1413703, EPI_ISL_1413705 to 1413706, EPI_ISL_1413708 to 1413709, EPI_ISL_1413711, EPI_ISL_1413713 to 1413714, EPI_ISL_1413719, EPI_ISL_1413721 to 1413722, EPI_ISL_1413724 to 1413725, EPI_ISL_1413727, EPI_ISL_1413729 to 1413730, EPI_ISL_1413732, EPI_ISL_1413734 to 1413735, EPI_ISL_1413737, EPI_ISL_1413743 to 1413744, EPI_ISL_1413746, EPI_ISL_1413749, EPI_ISL_1413751, EPI_ISL_1413754, EPI_ISL_1413757, EPI_ISL_1413759 to 1413760, EPI_ISL_1413762, EPI_ISL_1413764 to 1413765, EPI_ISL_1413767 to 1413768, EPI_ISL_1413772 to 1413773, EPI_ISL_1413775, EPI_ISL_1413777 to 1413778, EPI_ISL_1413780, EPI_ISL_1413782 to 1413783, EPI_ISL_1413785 to 1413786, EPI_ISL_1413788, EPI_ISL_1413790 to 1413791, EPI_ISL_1413793, EPI_ISL_1413795 to 1413796, EPI_ISL_1413798, EPI_ISL_1413802, EPI_ISL_1413804, EPI_ISL_1413808 to 1413809, EPI_ISL_1413812, EPI_ISL_1413814, EPI_ISL_1413816, EPI_ISL_1413819 to 1413820, EPI_ISL_1413822, EPI_ISL_1413824, EPI_ISL_1413827 to 1413828, EPI_ISL_1413830, EPI_ISL_1413832 to 1413833, EPI_ISL_1413836, EPI_ISL_1413838 to 1413839, EPI_ISL_1413844, EPI_ISL_1413846 to 1413847, EPI_ISL_1413850, EPI_ISL_1413852, EPI_ISL_1413854 to 1413855, EPI_ISL_1413857, EPI_ISL_1413860, EPI_ISL_1413862 to 1413863, EPI_ISL_1413865 to 1413866, EPI_ISL_1413868, EPI_ISL_1413870, EPI_ISL_1413872, EPI_ISL_1413875 to 1413882, EPI_ISL_1413884 to 1413887, EPI_ISL_1413892, EPI_ISL_1413898, EPI_ISL_1413900 to 1413901, EPI_ISL_1413906, EPI_ISL_1413908, EPI_ISL_1413910 to 1413911, EPI_ISL_1413915 to 1413916, EPI_ISL_1413918, EPI_ISL_1413923, EPI_ISL_1413926, EPI_ISL_1413928, EPI_ISL_1413930, EPI_ISL_1413936, EPI_ISL_1413938 to 1413939, EPI_ISL_1413941 to 1413942, EPI_ISL_1413945 to 1413946, EPI_ISL_1413948 to 1413957, EPI_ISL_1413960 to 1413966, EPI_ISL_1413968, EPI_ISL_1413970, EPI_ISL_1413972 to 1413977, EPI_ISL_1413981 to 1413982, EPI_ISL_1413984, EPI_ISL_1413986 to 1413989, EPI_ISL_1413991 to 1413992, EPI_ISL_1413994 to 1413996, EPI_ISL_1413998, EPI_ISL_1414002 to 1414003, EPI_ISL_1414005, EPI_ISL_1414008 to 1414013, EPI_ISL_1414015 to 1414017, EPI_ISL_1414019 to 1414024, EPI_ISL_1414026 to 1414031, EPI_ISL_1414033 to 1414034, EPI_ISL_1414036 to 1414042, EPI_ISL_1414044 to 1414047, EPI_ISL_1414049, EPI_ISL_1414051 to 1414053, EPI_ISL_1414055 to 1414056, EPI_ISL_1414058 to 1414061, EPI_ISL_1415816, EPI_ISL_1415818, EPI_ISL_1415827, EPI_ISL_1415834, EPI_ISL_1415845, EPI_ISL_1415852, EPI_ISL_1415854, EPI_ISL_1415863 to 1415865, EPI_ISL_1415870, EPI_ISL_1415874, EPI_ISL_1415878, EPI_ISL_1415884 to 1415885, EPI_ISL_1415888, EPI_ISL_1415892, EPI_ISL_1415905, EPI_ISL_1415907 to 1415908, EPI_ISL_1415921, EPI_ISL_1415925, EPI_ISL_1415930, EPI_ISL_1415935, EPI_ISL_1415940, EPI_ISL_1415943, EPI_ISL_1415946, EPI_ISL_1415957, EPI_ISL_1415970, EPI_ISL_1415984, EPI_ISL_1415986 to 1415987, EPI_ISL_1415995, EPI_ISL_1415999, EPI_ISL_1416001, EPI_ISL_1416013 to 1416014, EPI_ISL_1416018, EPI_ISL_1416024 to 1416025, EPI_ISL_1416042 to 1416043, EPI_ISL_1416050, EPI_ISL_1416071, EPI_ISL_1416074 to 1416075, EPI_ISL_1416086, EPI_ISL_1416094, EPI_ISL_1416098, EPI_ISL_1416102, EPI_ISL_1416109 to 1416110, EPI_ISL_1416117, EPI_ISL_1416123, EPI_ISL_1416174, EPI_ISL_1416180, EPI_ISL_1416187 to 1416190, EPI_ISL_1448789 to 1448790, EPI_ISL_1448792 to 1448796, EPI_ISL_1448800, EPI_ISL_1448802 to 1448803, EPI_ISL_1448805 to 1448806, EPI_ISL_1448808, EPI_ISL_1448810 to 1448817, EPI_ISL_1448819 to 1448828, EPI_ISL_1448830 to 1448837, EPI_ISL_1448839, EPI_ISL_1448841 to 1448842, EPI_ISL_1448844 to 1448846, EPI_ISL_1448848 to 1448853, EPI_ISL_1448855 to 1448856, EPI_ISL_1448858, EPI_ISL_1448861 to 1448862, EPI_ISL_1448864 to 1448867, EPI_ISL_1448869 to 1448873, EPI_ISL_1448875, EPI_ISL_1448877 to 1448895, EPI_ISL_1448897 to 1448898, EPI_ISL_1448900 to 1448904, EPI_ISL_1448906 to 1448907, EPI_ISL_1448910 to 1448916, EPI_ISL_1448918, EPI_ISL_1448921 to 1448926, EPI_ISL_1448928 to 1448932, EPI_ISL_1448934 to 1448938, EPI_ISL_1448940 to 1448946, EPI_ISL_1448950, EPI_ISL_1448953, EPI_ISL_1448955 to 1448964, EPI_ISL_1448966 to 1448967, EPI_ISL_1448969, EPI_ISL_1448973 to 1448975, EPI_ISL_1448978, EPI_ISL_1448980 to 1448983, EPI_ISL_1448985 to 1448988, EPI_ISL_1448991 to 1448992, EPI_ISL_1448995 to 1449004, EPI_ISL_1449006 to 1449014, EPI_ISL_1449016, EPI_ISL_1449020, EPI_ISL_1449022, EPI_ISL_1449027 to 1449028, EPI_ISL_1449350, EPI_ISL_1449353 to 1449356, EPI_ISL_1449359, EPI_ISL_1449361, EPI_ISL_1449363, EPI_ISL_1449366 to 1449369, EPI_ISL_1449372, EPI_ISL_1449377 to 1449379, EPI_ISL_1449388 to 1449390, EPI_ISL_1449395 to 1449398, EPI_ISL_1449400, EPI_ISL_1449404 to 1449406, EPI_ISL_1449409 to 1449410, EPI_ISL_1449416, EPI_ISL_1449418, EPI_ISL_1449420, EPI_ISL_1449425, EPI_ISL_1449429 to 1449430, EPI_ISL_1449433 to 1449435, EPI_ISL_1449437, EPI_ISL_1449441, EPI_ISL_1449443 to 1449446, EPI_ISL_1449448, EPI_ISL_1449461 to 1449462, EPI_ISL_1449468, EPI_ISL_1449471 to 1449472, EPI_ISL_1449474, EPI_ISL_1449478, EPI_ISL_1449482 to 1449484, EPI_ISL_1449486, EPI_ISL_1449492 to 1449493, EPI_ISL_1449498, EPI_ISL_1449501, EPI_ISL_1449503, EPI_ISL_1449509, EPI_ISL_1449516 to 1449518, EPI_ISL_1449520, EPI_ISL_1449524, EPI_ISL_1449527 to 1449529, EPI_ISL_1449533, EPI_ISL_1449535, EPI_ISL_1449537, EPI_ISL_1449541 to 1449542, EPI_ISL_1449546, EPI_ISL_1449548, EPI_ISL_1449550 to 1449551, EPI_ISL_1449553 to 1449554, EPI_ISL_1449556, EPI_ISL_1449558, EPI_ISL_1449560 to 1449564, EPI_ISL_1449571 to 1449572, EPI_ISL_1449575 to 1449576, EPI_ISL_1449581 to 1449583, EPI_ISL_1449585 to 1449586, EPI_ISL_1449588, EPI_ISL_1449591 to 1449595, EPI_ISL_1449597 to 1449598, EPI_ISL_1449600 to 1449604, EPI_ISL_1449606 to 1449609, EPI_ISL_1449611, EPI_ISL_1449614, EPI_ISL_1449616 to 1449617, EPI_ISL_1449619, EPI_ISL_1449623 to 1449624, EPI_ISL_1449626, EPI_ISL_1449628, EPI_ISL_1449631 to 1449632, EPI_ISL_1449635 to 1449636, EPI_ISL_1449638, EPI_ISL_1449644 to 1449646, EPI_ISL_1449648 to 1449650, EPI_ISL_1449662, EPI_ISL_1449682, EPI_ISL_1449690, EPI_ISL_1449692 to 1449693, EPI_ISL_1449695, EPI_ISL_1449697 to 1449698, EPI_ISL_1449702, EPI_ISL_1449705, EPI_ISL_1449709, EPI_ISL_1449716, EPI_ISL_1449721 to 1449722, EPI_ISL_1449725, EPI_ISL_1449730, EPI_ISL_1449733, EPI_ISL_1449737, EPI_ISL_1449741, EPI_ISL_1449749 to 1449751, EPI_ISL_1449769, EPI_ISL_1449778 to 1449779, EPI_ISL_1449782, EPI_ISL_1449788, EPI_ISL_1449795, EPI_ISL_1449800 to 1449802, EPI_ISL_1449809, EPI_ISL_1449814, EPI_ISL_1449817, EPI_ISL_1449826, EPI_ISL_1449839, EPI_ISL_1449847, EPI_ISL_1449850, EPI_ISL_1449857, EPI_ISL_1449863, EPI_ISL_1449870, EPI_ISL_1449876, EPI_ISL_1449887, EPI_ISL_1449908, EPI_ISL_1449913, EPI_ISL_1449923 to 1449924, EPI_ISL_1449930, EPI_ISL_1449934, EPI_ISL_1449940, EPI_ISL_1449944, EPI_ISL_1450303 to 1450304, EPI_ISL_1450310, EPI_ISL_1450314 to 1450315, EPI_ISL_1450321, EPI_ISL_1450327 to 1450328, EPI_ISL_1450331, EPI_ISL_1450333 to 1450334, EPI_ISL_1450337, EPI_ISL_1450340, EPI_ISL_1450342 to 1450344, EPI_ISL_1450349, EPI_ISL_1450353, EPI_ISL_1450362, EPI_ISL_1450366, EPI_ISL_1450370, EPI_ISL_1450372 to 1450373, EPI_ISL_1450378, EPI_ISL_1450385 to 1450387, EPI_ISL_1450394, EPI_ISL_1450397, EPI_ISL_1450403, EPI_ISL_1450408, EPI_ISL_1450411, EPI_ISL_1450414, EPI_ISL_1450418, EPI_ISL_1450433, EPI_ISL_1450438, EPI_ISL_1450441, EPI_ISL_1450450 to 1450451, EPI_ISL_1450453, EPI_ISL_1450463 to 1450464, EPI_ISL_1450466, EPI_ISL_1450469, EPI_ISL_1450472, EPI_ISL_1450476 to 1450477, EPI_ISL_1450482, EPI_ISL_1450485, EPI_ISL_1450487 to 1450488, EPI_ISL_1450500, EPI_ISL_1450503, EPI_ISL_1450508, EPI_ISL_1450519, EPI_ISL_1450522, EPI_ISL_1450524, EPI_ISL_1450526, EPI_ISL_1450529, EPI_ISL_1450538, EPI_ISL_1450552 to 1450553, EPI_ISL_1450558 to 1450559, EPI_ISL_1450565, EPI_ISL_1450567, EPI_ISL_1450570, EPI_ISL_1450582, EPI_ISL_1450584, EPI_ISL_1450590, EPI_ISL_1450596, EPI_ISL_1450598 to 1450599, EPI_ISL_1450604 to 1450606, EPI_ISL_1450895 to 1450899, EPI_ISL_1450901, EPI_ISL_1450904, EPI_ISL_1450906, EPI_ISL_1450910, EPI_ISL_1450915, EPI_ISL_1450918 to 1450920, EPI_ISL_1450922, EPI_ISL_1450927, EPI_ISL_1450930, EPI_ISL_1450932, EPI_ISL_1450934 to 1450937, EPI_ISL_1450941, EPI_ISL_1450945, EPI_ISL_1450947, EPI_ISL_1450949 to 1450950, EPI_ISL_1450954, EPI_ISL_1450956, EPI_ISL_1450958, EPI_ISL_1450965, EPI_ISL_1450967, EPI_ISL_1450969 to 1450970, EPI_ISL_1450973 to 1450974, EPI_ISL_1450978, EPI_ISL_1450981 to 1450982, EPI_ISL_1450989, EPI_ISL_1450991, EPI_ISL_1450993, EPI_ISL_1450997, EPI_ISL_1451001, EPI_ISL_1451009 to 1451012, EPI_ISL_1451014 to 1451016, EPI_ISL_1451024 to 1451025, EPI_ISL_1451028 to 1451030, EPI_ISL_1451034 to 1451035, EPI_ISL_1451039, EPI_ISL_1451042 to 1451045, EPI_ISL_1451048, EPI_ISL_1451052, EPI_ISL_1451056, EPI_ISL_1451058 to 1451059, EPI_ISL_1451064, EPI_ISL_1451067 to 1451068, EPI_ISL_1451071 to 1451072, EPI_ISL_1451076, EPI_ISL_1451081, EPI_ISL_1451089 to 1451090, EPI_ISL_1451095, EPI_ISL_1451097, EPI_ISL_1451102 to 1451103, EPI_ISL_1451105 to 1451107, EPI_ISL_1451114, EPI_ISL_1451116 to 1451120, EPI_ISL_1451122, EPI_ISL_1451126, EPI_ISL_1451129 to 1451131, EPI_ISL_1451147, EPI_ISL_1451149, EPI_ISL_1451154, EPI_ISL_1451157, EPI_ISL_1451159, EPI_ISL_1451162, EPI_ISL_1451170, EPI_ISL_1451174, EPI_ISL_1451179, EPI_ISL_1451183, EPI_ISL_1451185, EPI_ISL_1451187 to 1451189, EPI_ISL_1451196 to 1451197, EPI_ISL_1451211 to 1451212, EPI_ISL_1451220, EPI_ISL_1451235, EPI_ISL_1451241, EPI_ISL_1451246, EPI_ISL_1451251, EPI_ISL_1451271 to 1451272, EPI_ISL_1451275, EPI_ISL_1451277, EPI_ISL_1451285, EPI_ISL_1451293, EPI_ISL_1451301 to 1451303, EPI_ISL_1451313, EPI_ISL_1451321, EPI_ISL_1451323 to 1451325, EPI_ISL_1451333, EPI_ISL_1451335, EPI_ISL_1451353, EPI_ISL_1451355, EPI_ISL_1451359, EPI_ISL_1451363, EPI_ISL_1451366, EPI_ISL_1451372, EPI_ISL_1451375, EPI_ISL_1451378, EPI_ISL_1451385, EPI_ISL_1451389, EPI_ISL_1451392, EPI_ISL_1451397 to 1451398, EPI_ISL_1451400, EPI_ISL_1451404, EPI_ISL_1451407, EPI_ISL_1451410, EPI_ISL_1451418, EPI_ISL_1451421, EPI_ISL_1451423, EPI_ISL_1451429, EPI_ISL_1451434, EPI_ISL_1451439, EPI_ISL_1451441, EPI_ISL_1451445, EPI_ISL_1451447 to 1451449, EPI_ISL_1451453, EPI_ISL_1451472, EPI_ISL_1451475, EPI_ISL_1451481, EPI_ISL_1451487, EPI_ISL_1451496 to 1451497, EPI_ISL_1451502 to 1451503, EPI_ISL_1451505, EPI_ISL_1451509, EPI_ISL_1451519 to 1451520, EPI_ISL_1451533 to 1451534, EPI_ISL_1451538 to 1451540, EPI_ISL_1451547, EPI_ISL_1451561, EPI_ISL_1451567, EPI_ISL_1451571 to 1451573, EPI_ISL_1451577, EPI_ISL_1451580, EPI_ISL_1451585, EPI_ISL_1451590, EPI_ISL_1451599, EPI_ISL_1451601 to 1451602, EPI_ISL_1451607, EPI_ISL_1451609, EPI_ISL_1451615, EPI_ISL_1451633 to 1451634, EPI_ISL_1451640, EPI_ISL_1451648, EPI_ISL_1451651, EPI_ISL_1451654, EPI_ISL_1451666 to 1451668, EPI_ISL_1451671, EPI_ISL_1451676 to 1451678, EPI_ISL_1451683 to 1451684, EPI_ISL_1451687, EPI_ISL_1451690, EPI_ISL_1451693 to 1451694, EPI_ISL_1451697, EPI_ISL_1451706, EPI_ISL_1451713, EPI_ISL_1451717, EPI_ISL_1451729, EPI_ISL_1451732, EPI_ISL_1451736, EPI_ISL_1451747, EPI_ISL_1451750, EPI_ISL_1451754, EPI_ISL_1451756 to 1451757, EPI_ISL_1451760 to 1451761, EPI_ISL_1451766, EPI_ISL_1451769, EPI_ISL_1451785 to 1451787, EPI_ISL_1451789 to 1451791, EPI_ISL_1451793, EPI_ISL_1451796, EPI_ISL_1451799, EPI_ISL_1451802, EPI_ISL_1451805 to 1451806, EPI_ISL_1451811 to 1451812, EPI_ISL_1451815, EPI_ISL_1451818, EPI_ISL_1451829, EPI_ISL_1451832, EPI_ISL_1451834, EPI_ISL_1451837 to 1451838, EPI_ISL_1451840 to 1451842, EPI_ISL_1451845, EPI_ISL_1451850, EPI_ISL_1451852, EPI_ISL_1451858 to 1451859, EPI_ISL_1451862, EPI_ISL_1451869 to 1451870, EPI_ISL_1451872, EPI_ISL_1451875 to 1451876, EPI_ISL_1451879, EPI_ISL_1451881 to 1451883, EPI_ISL_1451886 to 1451888, EPI_ISL_1451892, EPI_ISL_1451896, EPI_ISL_1451900, EPI_ISL_1451903 to 1451904, EPI_ISL_1451907, EPI_ISL_1451909, EPI_ISL_1451914, EPI_ISL_1451917 to 1451919, EPI_ISL_1451922, EPI_ISL_1451930 to 1451932, EPI_ISL_1451935 to 1451937, EPI_ISL_1451940, EPI_ISL_1451942, EPI_ISL_1451956 to 1451958, EPI_ISL_1451966 to 1451967, EPI_ISL_1451969 to 1451970, EPI_ISL_1451975, EPI_ISL_1451977, EPI_ISL_1451979 to 1451980, EPI_ISL_1451987 to 1451988, EPI_ISL_1451992, EPI_ISL_1451998 to 1451999, EPI_ISL_1452002 to 1452003, EPI_ISL_1452005, EPI_ISL_1452008, EPI_ISL_1452010 to 1452011, EPI_ISL_1452014, EPI_ISL_1452016 to 1452017, EPI_ISL_1452019, EPI_ISL_1452030,

EPI_ISL_1452032, EPI_ISL_1452035 to 1452036, EPI_ISL_1452039 to 1452041, EPI_ISL_1452043, EPI_ISL_1452050 to 1452054, EPI_ISL_1452056, EPI_ISL_1452060 to 1452063, EPI_ISL_1452073, EPI_ISL_1452076, EPI_ISL_1452078, EPI_ISL_1452080, EPI_ISL_1452082 to 1452083, EPI_ISL_1452088 to 1452089,
EPI_ISL_1452094, EPI_ISL_1452098, EPI_ISL_1452101, EPI_ISL_1452108, EPI_ISL_1452110, EPI_ISL_1452112, EPI_ISL_1452117, EPI_ISL_1452121, EPI_ISL_1452124, EPI_ISL_1452127, EPI_ISL_1452129, EPI_ISL_1452132, EPI_ISL_1452136, EPI_ISL_1452139, EPI_ISL_1452148, EPI_ISL_1452151 to 1452153,
EPI_ISL_1452156, EPI_ISL_1452165, EPI_ISL_1452167, EPI_ISL_1452170 to 1452171, EPI_ISL_1452179, EPI_ISL_1452181, EPI_ISL_1452183, EPI_ISL_1452190, EPI_ISL_1452201, EPI_ISL_1452203, EPI_ISL_1452209, EPI_ISL_1452211, EPI_ISL_1452214, EPI_ISL_1452217, EPI_ISL_1452219, EPI_ISL_1452221,
EPI_ISL_1452227, EPI_ISL_1452230, EPI_ISL_1452234 to 1452235, EPI_ISL_1452237, EPI_ISL_1452241, EPI_ISL_1452243 to 1452244, EPI_ISL_1452247 to 1452249, EPI_ISL_1452251, EPI_ISL_1452255, EPI_ISL_1452261, EPI_ISL_1452263 to 1452265, EPI_ISL_1452267 to 1452268, EPI_ISL_1452270 to 1452271,
EPI_ISL_1452274 to 1452276, EPI_ISL_1452283, EPI_ISL_1452286 to 1452287, EPI_ISL_1452290 to 1452292, EPI_ISL_1452294, EPI_ISL_1452296, EPI_ISL_1452300 to 1452301, EPI_ISL_1452303, EPI_ISL_1452305, EPI_ISL_1452307, EPI_ISL_1452310, EPI_ISL_1452312, EPI_ISL_1452314, EPI_ISL_1452316,
EPI_ISL_1452319, EPI_ISL_1452323 to 1452324, EPI_ISL_1452327 to 1452333, EPI_ISL_1452335 to 1452337, EPI_ISL_1452339, EPI_ISL_1452341, EPI_ISL_1452343, EPI_ISL_1452345 to 1452346, EPI_ISL_1452351 to 1452355, EPI_ISL_1452359 to 1452360, EPI_ISL_1452362 to 1452368, EPI_ISL_1452370 to 1452374,
EPI_ISL_1452376 to 1452382, EPI_ISL_1452385 to 1452391, EPI_ISL_1452394 to 1452395, EPI_ISL_1452398, EPI_ISL_1452400, EPI_ISL_1452402 to 1452404, EPI_ISL_1452407, EPI_ISL_1452409, EPI_ISL_1452412 to 1452414, EPI_ISL_1452417 to 1452430, EPI_ISL_1452432, EPI_ISL_1452434 to 1452435,
EPI_ISL_1452437 to 1452440, EPI_ISL_1452442, EPI_ISL_1452444, EPI_ISL_1452446, EPI_ISL_1452448, EPI_ISL_1452450 to 1452453, EPI_ISL_1452455 to 1452456, EPI_ISL_1452459 to 1452464, EPI_ISL_1452470, EPI_ISL_1452473 to 1452474, EPI_ISL_1452476 to 1452478, EPI_ISL_1452480 to 1452481,
EPI_ISL_1452483, EPI_ISL_1452485, EPI_ISL_1452487, EPI_ISL_1452490, EPI_ISL_1452492, EPI_ISL_1452494 to 1452498, EPI_ISL_1452500, EPI_ISL_1452502 to 1452503, EPI_ISL_1452505 to 1452508, EPI_ISL_1452510 to 1452512, EPI_ISL_1452514 to 1452515, EPI_ISL_1452518 to 1452520, EPI_ISL_1452522 to
1452523, EPI_ISL_1452526 to 1452536, EPI_ISL_1452538 to 1452539, EPI_ISL_1452541, EPI_ISL_1452543, EPI_ISL_1452546, EPI_ISL_1452548 to 1452550, EPI_ISL_1452552, EPI_ISL_1452554, EPI_ISL_1452556 to 1452565, EPI_ISL_1452567 to 1452569, EPI_ISL_1452571, EPI_ISL_1452573 to 1452575,
EPI_ISL_1452577, EPI_ISL_1452582 to 1452590, EPI_ISL_1452592 to 1452593, EPI_ISL_1452595, EPI_ISL_1452597 to 1452600, EPI_ISL_1452602 to 1452603, EPI_ISL_1452606, EPI_ISL_1452608 to 1452609, EPI_ISL_1452612 to 1452616, EPI_ISL_1452618 to 1452624, EPI_ISL_1452627 to 1452630, EPI_ISL_1452633 to
1452637, EPI_ISL_1452639 to 1452643, EPI_ISL_1452645 to 1452646, EPI_ISL_1452648, EPI_ISL_1452651 to 1452664, EPI_ISL_1452666 to 1452673, EPI_ISL_1452675 to 1452679, EPI_ISL_1452681, EPI_ISL_1452683, EPI_ISL_1452686 to 1452691, EPI_ISL_1452695 to 1452696, EPI_ISL_1452698, EPI_ISL_1452700 to
1452701, EPI_ISL_1452705 to 1452706, EPI_ISL_1452709, EPI_ISL_1452713 to 1452714, EPI_ISL_1452717, EPI_ISL_1452719, EPI_ISL_1452721, EPI_ISL_1452723 to 1452727, EPI_ISL_1452731 to 1452737, EPI_ISL_1452739, EPI_ISL_1452741 to 1452742, EPI_ISL_1452744, EPI_ISL_1452746 to 1452749,
EPI_ISL_1452752, EPI_ISL_1452754, EPI_ISL_1452756 to 1452757, EPI_ISL_1452759 to 1452760, EPI_ISL_1452762 to 1452763, EPI_ISL_1452765 to 1452766, EPI_ISL_1452769 to 1452771, EPI_ISL_1452773 to 1452776, EPI_ISL_1452779, EPI_ISL_1452782, EPI_ISL_1452784 to 1452785, EPI_ISL_1452788 to 1452789,
EPI_ISL_1452791, EPI_ISL_1452793, EPI_ISL_1452796 to 1452797, EPI_ISL_1452799, EPI_ISL_1452801 to 1452807, EPI_ISL_1452809 to 1452811, EPI_ISL_1452813 to 1452814, EPI_ISL_1452816, EPI_ISL_1452818, EPI_ISL_1452820 to 1452825, EPI_ISL_1452827 to 1452830, EPI_ISL_1452832, EPI_ISL_1452834,
EPI_ISL_1452837 to 1452838, EPI_ISL_1452840 to 1452841, EPI_ISL_1452843 to 1452850, EPI_ISL_1452852 to 1452853, EPI_ISL_1452855, EPI_ISL_1452858 to 1452859, EPI_ISL_1452861, EPI_ISL_1452863 to 1452865, EPI_ISL_1452867 to 1452869, EPI_ISL_1452875 to 1452876, EPI_ISL_1452878 to 1452881,
EPI_ISL_1452883 to 1452886, EPI_ISL_1452889, EPI_ISL_1452896, EPI_ISL_1452900, EPI_ISL_1452906, EPI_ISL_1452908 to 1452909, EPI_ISL_1452912 to 1452913, EPI_ISL_1452915 to 1452916, EPI_ISL_1452919, EPI_ISL_1452921, EPI_ISL_1452924, EPI_ISL_1452926, EPI_ISL_1452928, EPI_ISL_1452930,
EPI_ISL_1452936, EPI_ISL_1452938 to 1452939, EPI_ISL_1452943, EPI_ISL_1452946, EPI_ISL_1452955, EPI_ISL_1452960, EPI_ISL_1452962, EPI_ISL_1452965, EPI_ISL_1452968 to 1452971, EPI_ISL_1452973 to 1452975, EPI_ISL_1452977 to 1452978, EPI_ISL_1452981, EPI_ISL_1452983, EPI_ISL_1452985,
EPI_ISL_1452991, EPI_ISL_1452993, EPI_ISL_1452996, EPI_ISL_1452999 to 1453000, EPI_ISL_1453002 to 1453003, EPI_ISL_1453005, EPI_ISL_1453008, EPI_ISL_1453011 to 1453013, EPI_ISL_1453015 to 1453016, EPI_ISL_1453021 to 1453023, EPI_ISL_1453027, EPI_ISL_1453030 to 1453032, EPI_ISL_1453034 to
1453039, EPI_ISL_1453042 to 1453043, EPI_ISL_1453047, EPI_ISL_1453056 to 1453058, EPI_ISL_1453060, EPI_ISL_1453065 to 1453066, EPI_ISL_1453068, EPI_ISL_1453072, EPI_ISL_1453075 to 1453076, EPI_ISL_1453078, EPI_ISL_1453080 to 1453089, EPI_ISL_1453092 to 1453095, EPI_ISL_1453097 to 1453098,
EPI_ISL_1453100 to 1453101, EPI_ISL_1453103, EPI_ISL_1453105, EPI_ISL_1453109 to 1453110, EPI_ISL_1453114, EPI_ISL_1453117 to 1453118, EPI_ISL_1453120, EPI_ISL_1453122 to 1453123, EPI_ISL_1453125 to 1453127, EPI_ISL_1453130, EPI_ISL_1453132, EPI_ISL_1453135 to 1453136, EPI_ISL_1453138 to
1453139, EPI_ISL_1453143 to 1453144, EPI_ISL_1453146, EPI_ISL_1453153 to 1453157, EPI_ISL_1453159, EPI_ISL_1453163, EPI_ISL_1453165, EPI_ISL_1453167, EPI_ISL_1453169, EPI_ISL_1453174 to 1453175, EPI_ISL_1453179, EPI_ISL_1453181, EPI_ISL_1453186 to 1453188, EPI_ISL_1453192, EPI_ISL_1453196 to
1453197, EPI_ISL_1453199, EPI_ISL_1453204, EPI_ISL_1453206, EPI_ISL_1453210, EPI_ISL_1453212 to 1453215, EPI_ISL_1453219 to 1453222, EPI_ISL_1453228, EPI_ISL_1453234, EPI_ISL_1453241, EPI_ISL_1453246, EPI_ISL_1453249 to 1453250, EPI_ISL_1453254 to 1453255, EPI_ISL_1453258, EPI_ISL_1453260,
EPI_ISL_1453265 to 1453268, EPI_ISL_1453271, EPI_ISL_1453273, EPI_ISL_1453276, EPI_ISL_1453278 to 1453281, EPI_ISL_1453283, EPI_ISL_1453291 to 1453292, EPI_ISL_1453294, EPI_ISL_1453297 to 1453298, EPI_ISL_1453300, EPI_ISL_1453303, EPI_ISL_1453311, EPI_ISL_1453313 to 1453317, EPI_ISL_1453319
to 1453320, EPI_ISL_1453323, EPI_ISL_1453325, EPI_ISL_1453327, EPI_ISL_1453329, EPI_ISL_1453331, EPI_ISL_1453335, EPI_ISL_1453337 to 1453338, EPI_ISL_1453341, EPI_ISL_1453346 to 1453347, EPI_ISL_1453351, EPI_ISL_1453355, EPI_ISL_1453359 to 1453362, EPI_ISL_1453364, EPI_ISL_1453369 to 1453371,
EPI_ISL_1453373, EPI_ISL_1453375, EPI_ISL_1453378, EPI_ISL_1453392 to 1453396, EPI_ISL_1453398, EPI_ISL_1453400, EPI_ISL_1453404 to 1453405, EPI_ISL_1453407, EPI_ISL_1453409, EPI_ISL_1453411, EPI_ISL_1453413, EPI_ISL_1453415, EPI_ISL_1453420, EPI_ISL_1453424, EPI_ISL_1453426 to 1453427,
EPI_ISL_1453433 to 1453434, EPI_ISL_1453437, EPI_ISL_1453440, EPI_ISL_1453442, EPI_ISL_1453444 to 1453445, EPI_ISL_1453447 to 1453449, EPI_ISL_1453453, EPI_ISL_1453459, EPI_ISL_1453463, EPI_ISL_1453470 to 1453471, EPI_ISL_1453475 to 1453476, EPI_ISL_1453479, EPI_ISL_1453481 to 1453482,
EPI_ISL_1453484 to 1453485, EPI_ISL_1453850, EPI_ISL_1453853 to 1453856, EPI_ISL_1453858, EPI_ISL_1453863, EPI_ISL_1453865 to 1453866, EPI_ISL_1453868 to 1453872, EPI_ISL_1453875, EPI_ISL_1453879 to 1453880, EPI_ISL_1453884 to 1453885, EPI_ISL_1453887 to 1453888, EPI_ISL_1453890,
EPI_ISL_1453893, EPI_ISL_1453897 to 1453898, EPI_ISL_1453906, EPI_ISL_1453908 to 1453910, EPI_ISL_1453913 to 1453914, EPI_ISL_1453916, EPI_ISL_1453921, EPI_ISL_1453924, EPI_ISL_1453926 to 1453929, EPI_ISL_1453931, EPI_ISL_1453940 to 1453942, EPI_ISL_1453947 to 1453948, EPI_ISL_1453951 to
1453952, EPI_ISL_1453957 to 1453958, EPI_ISL_1453965 to 1453966, EPI_ISL_1453968, EPI_ISL_1453970, EPI_ISL_1453975, EPI_ISL_1453978, EPI_ISL_1453988, EPI_ISL_1453993 to 1453995, EPI_ISL_1453997, EPI_ISL_1454000, EPI_ISL_1454002, EPI_ISL_1454004 to 1454005, EPI_ISL_1454007 to 1454008,
EPI_ISL_1454011, EPI_ISL_1454013, EPI_ISL_1454017, EPI_ISL_1454020 to 1454023, EPI_ISL_1454025, EPI_ISL_1454028, EPI_ISL_1454033 to 1454035, EPI_ISL_1454038, EPI_ISL_1454042, EPI_ISL_1454044 to 1454045, EPI_ISL_1454047 to 1454049, EPI_ISL_1454051 to 1454052, EPI_ISL_1454056 to 1454058,
EPI_ISL_1454060 to 1454061, EPI_ISL_1454065, EPI_ISL_1454067, EPI_ISL_1454069, EPI_ISL_1454074 to 1454076, EPI_ISL_1454079 to 1454080, EPI_ISL_1454083, EPI_ISL_1454085 to 1454087, EPI_ISL_1454089, EPI_ISL_1454095 to 1454097, EPI_ISL_1454100 to 1454102, EPI_ISL_1454104 to 1454105,
EPI_ISL_1454107, EPI_ISL_1454115, EPI_ISL_1454117, EPI_ISL_1454123, EPI_ISL_1454125, EPI_ISL_1454132 to 1454137, EPI_ISL_1454140 to 1454141, EPI_ISL_1454144 to 1454145, EPI_ISL_1454147, EPI_ISL_1454149, EPI_ISL_1454152, EPI_ISL_1454158, EPI_ISL_1454165, EPI_ISL_1454167, EPI_ISL_1454169,
EPI_ISL_1454172, EPI_ISL_1454174, EPI_ISL_1454179 to 1454180, EPI_ISL_1454184, EPI_ISL_1454187, EPI_ISL_1454193, EPI_ISL_1454199, EPI_ISL_1454203, EPI_ISL_1454211, EPI_ISL_1454213 to 1454217, EPI_ISL_1454219, EPI_ISL_1454224, EPI_ISL_1454226, EPI_ISL_1454228 to 1454229, EPI_ISL_1454231 to
1454233, EPI_ISL_1454235, EPI_ISL_1454237, EPI_ISL_1454239 to 1454240, EPI_ISL_1454244 to 1454247, EPI_ISL_1454260 to 1454262, EPI_ISL_1454264 to 1454266, EPI_ISL_1454268, EPI_ISL_1454276, EPI_ISL_1454278 to 1454279, EPI_ISL_1454284 to 1454287, EPI_ISL_1454290 to 1454291, EPI_ISL_1454296 to
1454299, EPI_ISL_1454301 to 1454302, EPI_ISL_1454304 to 1454305, EPI_ISL_1454308, EPI_ISL_1454310, EPI_ISL_1454312 to 1454314, EPI_ISL_1454322, EPI_ISL_1454638 to 1454641, EPI_ISL_1454644, EPI_ISL_1454646, EPI_ISL_1454649, EPI_ISL_1454675, EPI_ISL_1454683 to 1454686, EPI_ISL_1454695,
EPI_ISL_1454701 to 1454702, EPI_ISL_1454719, EPI_ISL_1454722, EPI_ISL_1454730, EPI_ISL_1454732, EPI_ISL_1454745, EPI_ISL_1454752, EPI_ISL_1454757 to 1454758, EPI_ISL_1454761, EPI_ISL_1454763, EPI_ISL_1454773, EPI_ISL_1454778, EPI_ISL_1454797, EPI_ISL_1454799, EPI_ISL_1454827,
EPI_ISL_1454844, EPI_ISL_1454846, EPI_ISL_1454852, EPI_ISL_1454854, EPI_ISL_1454856, EPI_ISL_1454865, EPI_ISL_1454867, EPI_ISL_1454875, EPI_ISL_1454877, EPI_ISL_1454880, EPI_ISL_1454890 to 1454891, EPI_ISL_1454896, EPI_ISL_1454900, EPI_ISL_1454903, EPI_ISL_1454915, EPI_ISL_1454925 to
1454928, EPI_ISL_1454939, EPI_ISL_1454943, EPI_ISL_1454948 to 1454949, EPI_ISL_1454953, EPI_ISL_1454956 to 1454958, EPI_ISL_1454965, EPI_ISL_1454967 to 1454968, EPI_ISL_1454974, EPI_ISL_1454989, EPI_ISL_1455003 to 1455004, EPI_ISL_1455006 to 1455007, EPI_ISL_1455021, EPI_ISL_1455024 to
1455027, EPI_ISL_1455030, EPI_ISL_1455033 to 1455034, EPI_ISL_1455036 to 1455037, EPI_ISL_1455039, EPI_ISL_1455041 to 1455045, EPI_ISL_1455048 to 1455049, EPI_ISL_1455052, EPI_ISL_1455054, EPI_ISL_1455057 to 1455059, EPI_ISL_1455061 to 1455066, EPI_ISL_1455068 to 1455069, EPI_ISL_1455071,
EPI_ISL_1455074, EPI_ISL_1455076 to 1455078, EPI_ISL_1455082 to 1455083, EPI_ISL_1455087 to 1455089, EPI_ISL_1455091, EPI_ISL_1455093, EPI_ISL_1455096 to 1455098, EPI_ISL_1455106 to 1455108, EPI_ISL_1455110, EPI_ISL_1455116, EPI_ISL_1455118, EPI_ISL_1455121, EPI_ISL_1455123, EPI_ISL_1455125,
EPI_ISL_1455130 to 1455131, EPI_ISL_1455133, EPI_ISL_1455140 to 1455141, EPI_ISL_1455143, EPI_ISL_1455145 to 1455146, EPI_ISL_1455149, EPI_ISL_1455154 to 1455157, EPI_ISL_1455159 to 1455160, EPI_ISL_1455164, EPI_ISL_1455166, EPI_ISL_1455169, EPI_ISL_1455171 to 1455172, EPI_ISL_1455176,
EPI_ISL_1455178 to 1455179, EPI_ISL_1455181 to 1455182, EPI_ISL_1455184 to 1455185, EPI_ISL_1455188, EPI_ISL_1455192 to 1455196, EPI_ISL_1455198 to 1455200, EPI_ISL_1455203, EPI_ISL_1455206, EPI_ISL_1455208, EPI_ISL_1455210 to 1455213, EPI_ISL_1455217 to 1455218, EPI_ISL_1455220 to 1455221,
EPI_ISL_1455227, EPI_ISL_1455229 to 1455230, EPI_ISL_1455232 to 1455235, EPI_ISL_1455237, EPI_ISL_1455243, EPI_ISL_1455249, EPI_ISL_1455251, EPI_ISL_1455255, EPI_ISL_1455262 to 1455263, EPI_ISL_1455273, EPI_ISL_1455282 to 1455283, EPI_ISL_1455286 to 1455288, EPI_ISL_1455293, EPI_ISL_1455299,
EPI_ISL_1455312, EPI_ISL_1455314, EPI_ISL_1455319, EPI_ISL_1455321, EPI_ISL_1455325, EPI_ISL_1455332, EPI_ISL_1455334 to 1455335, EPI_ISL_1455343, EPI_ISL_1455346, EPI_ISL_1455351 to 1455352, EPI_ISL_1455354, EPI_ISL_1455357, EPI_ISL_1455359, EPI_ISL_1455367 to 1455368, EPI_ISL_1455370,
EPI_ISL_1455372, EPI_ISL_1455381, EPI_ISL_1455385, EPI_ISL_1455389, EPI_ISL_1455398, EPI_ISL_1455402 to 1455404, EPI_ISL_1455407, EPI_ISL_1455409, EPI_ISL_1455413, EPI_ISL_1455447, EPI_ISL_1455449, EPI_ISL_1455451 to 1455452, EPI_ISL_1455454, EPI_ISL_1455458, EPI_ISL_1455464,
EPI_ISL_1455472, EPI_ISL_1455474, EPI_ISL_1455480 to 1455481, EPI_ISL_1455520, EPI_ISL_1455532, EPI_ISL_1455534, EPI_ISL_1455536 to 1455537, EPI_ISL_1455541, EPI_ISL_1455566, EPI_ISL_1455572, EPI_ISL_1455574, EPI_ISL_1455577, EPI_ISL_1455580 to 1455592, EPI_ISL_1455594 to 1455596,
EPI_ISL_1455599, EPI_ISL_1455603 to 1455605, EPI_ISL_1455608 to 1455611, EPI_ISL_1455616, EPI_ISL_1455618, EPI_ISL_1455620, EPI_ISL_1455623, EPI_ISL_1465897 to 1465903, EPI_ISL_1465908 to 1465912, EPI_ISL_1465914, EPI_ISL_1465916 to 1465924, EPI_ISL_1465928, EPI_ISL_1465931 to 1465935,
EPI_ISL_1465937 to 1465940, EPI_ISL_1465942 to 1465943, EPI_ISL_1465945 to 1465948, EPI_ISL_1465950 to 1465951, EPI_ISL_1465953 to 1465956, EPI_ISL_1465958 to 1465966, EPI_ISL_1465968 to 1465969, EPI_ISL_1465972, EPI_ISL_1465976 to 1465981, EPI_ISL_1465983 to 1465991, EPI_ISL_1465993 to 1466005,
EPI_ISL_1466007 to 1466008, EPI_ISL_1466012, EPI_ISL_1466014 to 1466015, EPI_ISL_1466017 to 1466018, EPI_ISL_1466021 to 1466022, EPI_ISL_1466025 to 1466028, EPI_ISL_1466030 to 1466033, EPI_ISL_1466035 to 1466038, EPI_ISL_1466040 to 1466043, EPI_ISL_1466045, EPI_ISL_1466047 to 1466053,
EPI_ISL_1466056 to 1466066, EPI_ISL_1466068 to 1466075, EPI_ISL_1466077 to 1466078, EPI_ISL_1466080 to 1466082, EPI_ISL_1466085 to 1466087, EPI_ISL_1466089 to 1466099, EPI_ISL_1466103 to 1466108, EPI_ISL_1466110, EPI_ISL_1466114, EPI_ISL_1466116, EPI_ISL_1466118 to 1466120, EPI_ISL_1466122 to
1466124, EPI_ISL_1466126 to 1466128, EPI_ISL_1466130 to 1466132, EPI_ISL_1466134 to 1466140, EPI_ISL_1466142, EPI_ISL_1466144 to 1466147, EPI_ISL_1466149 to 1466157, EPI_ISL_1466159 to 1466160, EPI_ISL_1466162, EPI_ISL_1466173, EPI_ISL_1466184, EPI_ISL_1466194 to 1466196, EPI_ISL_1466229,
EPI_ISL_1466235, EPI_ISL_1466239, EPI_ISL_1466242, EPI_ISL_1466246 to 1466247, EPI_ISL_1466252, EPI_ISL_1466254, EPI_ISL_1466260, EPI_ISL_1466270 to 1466271, EPI_ISL_1466281, EPI_ISL_1466283 to 1466284, EPI_ISL_1466287, EPI_ISL_1466296, EPI_ISL_1466306, EPI_ISL_1466309, EPI_ISL_1466314,
EPI_ISL_1466316, EPI_ISL_1466322, EPI_ISL_1466325 to 1466326, EPI_ISL_1466334 to 1466335, EPI_ISL_1466338, EPI_ISL_1466344, EPI_ISL_1466362, EPI_ISL_1466374 to 1466375, EPI_ISL_1466381, EPI_ISL_1466398, EPI_ISL_1466409, EPI_ISL_1466417, EPI_ISL_1466420, EPI_ISL_1466426, EPI_ISL_1466428,
EPI_ISL_1466437, EPI_ISL_1466440, EPI_ISL_1466449, EPI_ISL_1466471, EPI_ISL_1466480, EPI_ISL_1466498, EPI_ISL_1466502, EPI_ISL_1466506, EPI_ISL_1466524, EPI_ISL_1466527, EPI_ISL_1466532 to 1466533, EPI_ISL_1466538, EPI_ISL_1466542 to 1466543, EPI_ISL_1466553, EPI_ISL_1466556,
EPI_ISL_1466559, EPI_ISL_1466561, EPI_ISL_1466564 to 1466565, EPI_ISL_1466580, EPI_ISL_1466587, EPI_ISL_1466589, EPI_ISL_1466596 to 1466597, EPI_ISL_1466600 to 1466601, EPI_ISL_1466604, EPI_ISL_1466612, EPI_ISL_1466620, EPI_ISL_1466622, EPI_ISL_1466627, EPI_ISL_1466644 to 1466645,
EPI_ISL_1466648, EPI_ISL_1466650, EPI_ISL_1466654, EPI_ISL_1466658 to 1466659, EPI_ISL_1466661, EPI_ISL_1466665, EPI_ISL_1466667, EPI_ISL_1466673 to 1466674, EPI_ISL_1466679, EPI_ISL_1466687, EPI_ISL_1466694, EPI_ISL_1466701 to 1466702, EPI_ISL_1466707, EPI_ISL_1466710, EPI_ISL_1466728,
EPI_ISL_1466736, EPI_ISL_1466744 to 1466745, EPI_ISL_1466747 to 1466751, EPI_ISL_1466753 to 1466763, EPI_ISL_1466765 to 1466767, EPI_ISL_1466769 to 1466770, EPI_ISL_1466772 to 1466774, EPI_ISL_1466776 to 1466778, EPI_ISL_1466780 to 1466782, EPI_ISL_1466784, EPI_ISL_1466790 to 1466792,
EPI_ISL_1466796 to 1466797, EPI_ISL_1466799 to 1466804, EPI_ISL_1466806 to 1466808, EPI_ISL_1466810 to 1466811, EPI_ISL_1466814 to 1466816, EPI_ISL_1466818 to 1466819, EPI_ISL_1466821, EPI_ISL_1466823, EPI_ISL_1466825 to 1466828, EPI_ISL_1466830, EPI_ISL_1466833 to 1466837, EPI_ISL_1466839,
EPI_ISL_1466841 to 1466843, EPI_ISL_1466845, EPI_ISL_1466848 to 1466849, EPI_ISL_1466851, EPI_ISL_1466853, EPI_ISL_1466855, EPI_ISL_1466857 to 1466865, EPI_ISL_1466867, EPI_ISL_1466869, EPI_ISL_1466871, EPI_ISL_1466873 to 1466877, EPI_ISL_1466879 to 1466880, EPI_ISL_1466882, EPI_ISL_1466885,
EPI_ISL_1466887, EPI_ISL_1466891, EPI_ISL_1466893, EPI_ISL_1466895, EPI_ISL_1466898 to 1466905, EPI_ISL_1466907 to 1466909, EPI_ISL_1466911 to 1466912, EPI_ISL_1466914 to 1466915, EPI_ISL_1466918 to 1466919, EPI_ISL_1466921, EPI_ISL_1467279, EPI_ISL_1467291, EPI_ISL_1467360, EPI_ISL_1467374,
EPI_ISL_1467404, EPI_ISL_1467421, EPI_ISL_1467423, EPI_ISL_1467425, EPI_ISL_1467441, EPI_ISL_1467474, EPI_ISL_1467483, EPI_ISL_1467516, EPI_ISL_1467536, EPI_ISL_1467544, EPI_ISL_1467552, EPI_ISL_1467579, EPI_ISL_1468134, EPI_ISL_1468136 to 1468137, EPI_ISL_1468139, EPI_ISL_1468144,
EPI_ISL_1468146 to 1468147, EPI_ISL_1468150, EPI_ISL_1468156 to 1468157, EPI_ISL_1468159, EPI_ISL_1468165 to 1468166, EPI_ISL_1468170, EPI_ISL_1468172 to 1468174, EPI_ISL_1468176, EPI_ISL_1468178, EPI_ISL_1468181, EPI_ISL_1468185, EPI_ISL_1468193, EPI_ISL_1468196, EPI_ISL_1468201,
EPI_ISL_1468207 to 1468208, EPI_ISL_1468210, EPI_ISL_1468213, EPI_ISL_1468215, EPI_ISL_1468218, EPI_ISL_1468220 to 1468221, EPI_ISL_1468232, EPI_ISL_1468234 to 1468237, EPI_ISL_1468241, EPI_ISL_1468246, EPI_ISL_1468248 to 1468249, EPI_ISL_1468256, EPI_ISL_1468261, EPI_ISL_1468266 to 1468271,
EPI_ISL_1468275, EPI_ISL_1468277 to 1468278, EPI_ISL_1468282, EPI_ISL_1468287, EPI_ISL_1468292, EPI_ISL_1468296 to 1468297, EPI_ISL_1468303 to 1468306, EPI_ISL_1468309, EPI_ISL_1468313 to 1468314, EPI_ISL_1468318 to 1468319, EPI_ISL_1468321, EPI_ISL_1468325, EPI_ISL_1468329, EPI_ISL_1468331,
EPI_ISL_1468334, EPI_ISL_1468336, EPI_ISL_1468339, EPI_ISL_1468341 to 1468342, EPI_ISL_1468344 to 1468345, EPI_ISL_1468352 to 1468355, EPI_ISL_1468358, EPI_ISL_1468364, EPI_ISL_1468366 to 1468367, EPI_ISL_1468370 to 1468371, EPI_ISL_1468374, EPI_ISL_1468376, EPI_ISL_1468382, EPI_ISL_1468384,
EPI_ISL_1468386, EPI_ISL_1468388, EPI_ISL_1468394, EPI_ISL_1468401 to 1468402, EPI_ISL_1472414, EPI_ISL_1472421, EPI_ISL_1472434, EPI_ISL_1472441, EPI_ISL_1472443, EPI_ISL_1472454, EPI_ISL_1472460, EPI_ISL_1472463, EPI_ISL_1472468, EPI_ISL_1472478, EPI_ISL_1472481, EPI_ISL_1472485,
EPI_ISL_1472526 to 1472527, EPI_ISL_1472530, EPI_ISL_1472533, EPI_ISL_1472535, EPI_ISL_1472549, EPI_ISL_1472556 to 1472558, EPI_ISL_1472568, EPI_ISL_1472579, EPI_ISL_1472583, EPI_ISL_1472597, EPI_ISL_1472604 to 1472606, EPI_ISL_1472612, EPI_ISL_1472617, EPI_ISL_1472619, EPI_ISL_1472625,
EPI_ISL_1472629, EPI_ISL_1472631, EPI_ISL_1472633, EPI_ISL_1472643 to 1472644, EPI_ISL_1472647, EPI_ISL_1472652 to 1472653, EPI_ISL_1472656, EPI_ISL_1472661 to 1472662, EPI_ISL_1472664 to 1472665, EPI_ISL_1472668, EPI_ISL_1472676 to 1472677, EPI_ISL_1472697 to 1472698, EPI_ISL_1472703 to
1472704, EPI_ISL_1472708 to 1472710, EPI_ISL_1472712 to 1472714, EPI_ISL_1472718, EPI_ISL_1472720 to 1472721, EPI_ISL_1472723 to 1472726, EPI_ISL_1472728, EPI_ISL_1472730, EPI_ISL_1472732 to 1472733, EPI_ISL_1472735 to 1472740, EPI_ISL_1472743 to 1472746, EPI_ISL_1472748 to 1472752,
EPI_ISL_1472755, EPI_ISL_1472758 to 1472763, EPI_ISL_1472768, EPI_ISL_1472770 to 1472771, EPI_ISL_1472773 to 1472776, EPI_ISL_1472779, EPI_ISL_1472781, EPI_ISL_1472783 to 1472784, EPI_ISL_1472787 to 1472788, EPI_ISL_1472790 to 1472793, EPI_ISL_1472795 to 1472796, EPI_ISL_1472798 to 1472799,
EPI_ISL_1472803 to 1472806, EPI_ISL_1472808, EPI_ISL_1472810 to 1472813, EPI_ISL_1472816 to 1472818, EPI_ISL_1472820 to 1472823, EPI_ISL_1472826, EPI_ISL_1472828 to 1472829, EPI_ISL_1472831, EPI_ISL_1472833 to 1472836, EPI_ISL_1472838, EPI_ISL_1472842, EPI_ISL_1472844 to 1472850,
EPI_ISL_1472852 to 1472853, EPI_ISL_1472855 to 1472859, EPI_ISL_1472861 to 1472862, EPI_ISL_1472864 to 1472865, EPI_ISL_1472868, EPI_ISL_1472871 to 1472875, EPI_ISL_1472877 to 1472879, EPI_ISL_1472882 to 1472884, EPI_ISL_1472886 to 1472892, EPI_ISL_1472894 to 1472897, EPI_ISL_1472899,
EPI_ISL_1472902 to 1472906, EPI_ISL_1472908 to 1472909, EPI_ISL_1472912 to 1472919, EPI_ISL_1472921 to 1472924, EPI_ISL_1472926, EPI_ISL_1472930, EPI_ISL_1472933, EPI_ISL_1472936, EPI_ISL_1472938 to 1472939, EPI_ISL_1472942, EPI_ISL_1472945 to 1473446, EPI_ISL_1473800 to 1473802,
EPI_ISL_1473805 to 1473806, EPI_ISL_1473811, EPI_ISL_1474157, EPI_ISL_1483804 to 1483805, EPI_ISL_1483808, EPI_ISL_1483820, EPI_ISL_1483825, EPI_ISL_1483835, EPI_ISL_1483838, EPI_ISL_1483841, EPI_ISL_1483844, EPI_ISL_1483853, EPI_ISL_1483855, EPI_ISL_1483861 to 1483862, EPI_ISL_1483870 to
1483872, EPI_ISL_1483875, EPI_ISL_1483877, EPI_ISL_1483887, EPI_ISL_1483889, EPI_ISL_1483892, EPI_ISL_1483896 to 1483897, EPI_ISL_1483902, EPI_ISL_1483910, EPI_ISL_1483916, EPI_ISL_1483919, EPI_ISL_1483922, EPI_ISL_1483925, EPI_ISL_1483928, EPI_ISL_1483930, EPI_ISL_1483933,
EPI_ISL_1483937, EPI_ISL_1483939, EPI_ISL_1483941, EPI_ISL_1483946, EPI_ISL_1483951, EPI_ISL_1483955, EPI_ISL_1483977, EPI_ISL_1483988 to 1483989, EPI_ISL_1484000, EPI_ISL_1484005, EPI_ISL_1484008 to 1484009, EPI_ISL_1484018, EPI_ISL_1484021, EPI_ISL_1484027 to 1484028, EPI_ISL_1484035,
EPI_ISL_1484040, EPI_ISL_1484046, EPI_ISL_1484049, EPI_ISL_1484051, EPI_ISL_1484056, EPI_ISL_1484060, EPI_ISL_1484062, EPI_ISL_1484064, EPI_ISL_1484078, EPI_ISL_1484085, EPI_ISL_1484088, EPI_ISL_1484092 to 1484093, EPI_ISL_1484096, EPI_ISL_1484099, EPI_ISL_1484101 to 1484102,
EPI_ISL_1484104 to 1484105, EPI_ISL_1484107 to 1484111, EPI_ISL_1484116 to 1484117, EPI_ISL_1484120 to 1484121, EPI_ISL_1484123, EPI_ISL_1484128, EPI_ISL_1484132, EPI_ISL_1484136, EPI_ISL_1484138 to 1484141, EPI_ISL_1484143 to 1484148, EPI_ISL_1484150, EPI_ISL_1484152 to 1484154,
EPI_ISL_1484156 to 1484159, EPI_ISL_1484161 to 1484164, EPI_ISL_1484166 to 1484168, EPI_ISL_1484171, EPI_ISL_1484174, EPI_ISL_1484176 to 1484179, EPI_ISL_1484181 to 1484185, EPI_ISL_1484189, EPI_ISL_1484192, EPI_ISL_1484196 to 1484197, EPI_ISL_1484200 to 1484201, EPI_ISL_1484205 to 1484206,
EPI_ISL_1484208 to 1484209, EPI_ISL_1484211 to 1484212, EPI_ISL_1484220, EPI_ISL_1484223 to 1484226, EPI_ISL_1484230 to 1484245, EPI_ISL_1484247 to 1484250, EPI_ISL_1484253, EPI_ISL_1484255, EPI_ISL_1484259, EPI_ISL_1484262 to 1484263, EPI_ISL_1484266 to 1484268, EPI_ISL_1484272 to 1484274,
EPI_ISL_1484276 to 1484281, EPI_ISL_1484284 to 1484288, EPI_ISL_1484290 to 1484292, EPI_ISL_1484294 to 1484296, EPI_ISL_1484298, EPI_ISL_1484300 to 1484301, EPI_ISL_1484303 to 1484307, EPI_ISL_1484309 to 1484310, EPI_ISL_1484312 to 1484313, EPI_ISL_1484611, EPI_ISL_1484614 to 1484615,
EPI_ISL_1484617, EPI_ISL_1484619, EPI_ISL_1484621, EPI_ISL_1484627, EPI_ISL_1484629, EPI_ISL_1484631, EPI_ISL_1484633 to 1484634, EPI_ISL_1484637, EPI_ISL_1484641, EPI_ISL_1484645, EPI_ISL_1484648 to 1484651, EPI_ISL_1484654, EPI_ISL_1484656, EPI_ISL_1484658 to 1484659, EPI_ISL_1484665,
EPI_ISL_1484667, EPI_ISL_1484675, EPI_ISL_1484680, EPI_ISL_1484685, EPI_ISL_1484687, EPI_ISL_1484691, EPI_ISL_1484698, EPI_ISL_1484706, EPI_ISL_1484708, EPI_ISL_1484710, EPI_ISL_1484712, EPI_ISL_1484717 to 1484720, EPI_ISL_1484728, EPI_ISL_1484733, EPI_ISL_1484739, EPI_ISL_1484747,
EPI_ISL_1484754, EPI_ISL_1484759, EPI_ISL_1484762, EPI_ISL_1484764 to 1484765, EPI_ISL_1484771 to 1484773, EPI_ISL_1484781, EPI_ISL_1484788 to 1484789, EPI_ISL_1484794 to 1484795, EPI_ISL_1484797, EPI_ISL_1484800, EPI_ISL_1484803, EPI_ISL_1484807, EPI_ISL_1484813, EPI_ISL_1484815,
EPI_ISL_1484818, EPI_ISL_1484827, EPI_ISL_1484830, EPI_ISL_1484834, EPI_ISL_1484836, EPI_ISL_1485182 to 1485184, EPI_ISL_1485191, EPI_ISL_1485195 to 1485196, EPI_ISL_1485199, EPI_ISL_1485204, EPI_ISL_1485211, EPI_ISL_1485213 to 1485214, EPI_ISL_1485216, EPI_ISL_1485218, EPI_ISL_1485224,
EPI_ISL_1485226 to 1485228, EPI_ISL_1485230, EPI_ISL_1485234, EPI_ISL_1485237 to 1485238, EPI_ISL_1485240 to 1485241, EPI_ISL_1485249, EPI_ISL_1485252, EPI_ISL_1485260, EPI_ISL_1485262 to 1485264, EPI_ISL_1485266, EPI_ISL_1485270, EPI_ISL_1485273, EPI_ISL_1485276, EPI_ISL_1485290,

EPI_ISL_1485292, EPI_ISL_1485298, EPI_ISL_1485300 to 1485301, EPI_ISL_1485303, EPI_ISL_1485307, EPI_ISL_1485313, EPI_ISL_1485315, EPI_ISL_1485321, EPI_ISL_1485325 to 1485329, EPI_ISL_1485332 to 1485336, EPI_ISL_1485343 to 1485344, EPI_ISL_1485347 to 1485348, EPI_ISL_1485351, EPI_ISL_1485355, EPI_ISL_1485361, EPI_ISL_1485363, EPI_ISL_1485367, EPI_ISL_1485371 to 1485372, EPI_ISL_1485375, EPI_ISL_1485378 to 1485379, EPI_ISL_1485381 to 1485382, EPI_ISL_1485385, EPI_ISL_1485388, EPI_ISL_1485395, EPI_ISL_1485398 to 1485399, EPI_ISL_1485406, EPI_ISL_1485412, EPI_ISL_1485414, EPI_ISL_1485418 to 1485419, EPI_ISL_1485422 to 1485426, EPI_ISL_1485428 to 1485429, EPI_ISL_1485433, EPI_ISL_1485438, EPI_ISL_1485447, EPI_ISL_1485455 to 1485458, EPI_ISL_1485460 to 1485461, EPI_ISL_1485467, EPI_ISL_1485470, EPI_ISL_1485472, EPI_ISL_1485475, EPI_ISL_1485810, EPI_ISL_1485813, EPI_ISL_1485815, EPI_ISL_1485832, EPI_ISL_1485835, EPI_ISL_1485856, EPI_ISL_1485863, EPI_ISL_1485866, EPI_ISL_1485871 to 1485872, EPI_ISL_1485876, EPI_ISL_1485879, EPI_ISL_1485892, EPI_ISL_1485898, EPI_ISL_1485910, EPI_ISL_1485936, EPI_ISL_1485947, EPI_ISL_1485962, EPI_ISL_1485966, EPI_ISL_1485973, EPI_ISL_1485985, EPI_ISL_1485992, EPI_ISL_1485999, EPI_ISL_1486012, EPI_ISL_1486024, EPI_ISL_1486028, EPI_ISL_1486030, EPI_ISL_1486045, EPI_ISL_1486054, EPI_ISL_1486058 to 1486060, EPI_ISL_1486065, EPI_ISL_1486067, EPI_ISL_1486071, EPI_ISL_1486075, EPI_ISL_1486079, EPI_ISL_1486087, EPI_ISL_1486091 to 1486092, EPI_ISL_1486097, EPI_ISL_1486107, EPI_ISL_1486118, EPI_ISL_1486124 to 1486125, EPI_ISL_1486127, EPI_ISL_1486140, EPI_ISL_1486142, EPI_ISL_1486149, EPI_ISL_1486152 to 1486153, EPI_ISL_1486160, EPI_ISL_1486168, EPI_ISL_1486172, EPI_ISL_1486175 to 1486176, EPI_ISL_1486181 to 1486182, EPI_ISL_1486190 to 1486191, EPI_ISL_1486198 to 1486199, EPI_ISL_1486204, EPI_ISL_1486211, EPI_ISL_1486213, EPI_ISL_1486218, EPI_ISL_1486229, EPI_ISL_1486234, EPI_ISL_1486237 to 1486238, EPI_ISL_1486245 to 1486246, EPI_ISL_1486248, EPI_ISL_1486250, EPI_ISL_1486253, EPI_ISL_1486263, EPI_ISL_1486272, EPI_ISL_1486275, EPI_ISL_1486277, EPI_ISL_1486279, EPI_ISL_1486285, EPI_ISL_1486291 to 1486292, EPI_ISL_1486297, EPI_ISL_1486299 to 1486300, EPI_ISL_1486305, EPI_ISL_1486311, EPI_ISL_1486317, EPI_ISL_1486319, EPI_ISL_1486326, EPI_ISL_1486329 to 1486330, EPI_ISL_1486334, EPI_ISL_1486350, EPI_ISL_1486361, EPI_ISL_1486372, EPI_ISL_1486375, EPI_ISL_1486379, EPI_ISL_1486388, EPI_ISL_1486391 to 1486392, EPI_ISL_1487159 to 1487162, EPI_ISL_1487164 to 1487175, EPI_ISL_1487177 to 1487180, EPI_ISL_1487182 to 1487205, EPI_ISL_1487207, EPI_ISL_1487209, EPI_ISL_1487211 to 1487254, EPI_ISL_1487256 to 1487269, EPI_ISL_1487271 to 1487287, EPI_ISL_1487289 to 1487290, EPI_ISL_1487292 to 1487305, EPI_ISL_1487307 to 1487308, EPI_ISL_1487311 to 1487315, EPI_ISL_1487317, EPI_ISL_1487319, EPI_ISL_1487321 to 1487354, EPI_ISL_1487356 to 1487375, EPI_ISL_1487377, EPI_ISL_1487725, EPI_ISL_1487727 to 1487743, EPI_ISL_1487745 to 1487774, EPI_ISL_1487776 to 1487790, EPI_ISL_1487792 to 1487812, EPI_ISL_1487814 to 1487830, EPI_ISL_1487833 to 1487851, EPI_ISL_1488229, EPI_ISL_1488245 to 1488246, EPI_ISL_1488249, EPI_ISL_1488251 to 1488255, EPI_ISL_1488264, EPI_ISL_1488266 to 1488269, EPI_ISL_1488271, EPI_ISL_1488273, EPI_ISL_1488280, EPI_ISL_1488283 to 1488285, EPI_ISL_1488289, EPI_ISL_1488291, EPI_ISL_1488296 to 1488297, EPI_ISL_1488300, EPI_ISL_1488304, EPI_ISL_1488307 to 1488308, EPI_ISL_1488313 to 1488314, EPI_ISL_1488316 to 1488317, EPI_ISL_1488320, EPI_ISL_1488322 to 1488323, EPI_ISL_1488325 to 1488326, EPI_ISL_1488328 to 1488329, EPI_ISL_1488335, EPI_ISL_1488341, EPI_ISL_1488344, EPI_ISL_1488347 to 1488348, EPI_ISL_1488353, EPI_ISL_1488355, EPI_ISL_1488359, EPI_ISL_1488362, EPI_ISL_1488367, EPI_ISL_1488369, EPI_ISL_1488382, EPI_ISL_1488384, EPI_ISL_1488389 to 1488395, EPI_ISL_1488398 to 1488400, EPI_ISL_1488405, EPI_ISL_1488410, EPI_ISL_1488416, EPI_ISL_1488418, EPI_ISL_1488420, EPI_ISL_1488424, EPI_ISL_1488426, EPI_ISL_1488444, EPI_ISL_1488446, EPI_ISL_1488455, EPI_ISL_1488458 to 1488459, EPI_ISL_1488463, EPI_ISL_1488472, EPI_ISL_1488479, EPI_ISL_1488481, EPI_ISL_1488483, EPI_ISL_1488489, EPI_ISL_1488494 to 1488496, EPI_ISL_1488498, EPI_ISL_1488500, EPI_ISL_1488502, EPI_ISL_1488510, EPI_ISL_1488513, EPI_ISL_1488516, EPI_ISL_1488866 to 1488868, EPI_ISL_1488871 to 1488872, EPI_ISL_1488874, EPI_ISL_1488876 to 1488878, EPI_ISL_1488880 to 1488883, EPI_ISL_1488885 to 1488887, EPI_ISL_1488889 to 1488890, EPI_ISL_1488892 to 1488895, EPI_ISL_1488897 to 1488898, EPI_ISL_1488900 to 1488903, EPI_ISL_1488905, EPI_ISL_1488907 to 1488909, EPI_ISL_1488913 to 1488916, EPI_ISL_1488918 to 1488919, EPI_ISL_1488921, EPI_ISL_1488923, EPI_ISL_1488926 to 1488930, EPI_ISL_1488932 to 1488936, EPI_ISL_1488938, EPI_ISL_1488940 to 1488941, EPI_ISL_1488944, EPI_ISL_1488948, EPI_ISL_1488950, EPI_ISL_1488953 to 1488961, EPI_ISL_1488963 to 1488965, EPI_ISL_1488967, EPI_ISL_1488969, EPI_ISL_1488971, EPI_ISL_1488974, EPI_ISL_1488976 to 1488977, EPI_ISL_1488979 to 1488982, EPI_ISL_1488984 to 1488992, EPI_ISL_1488994 to 1489000, EPI_ISL_1489002, EPI_ISL_1489005 to 1489008, EPI_ISL_1489011, EPI_ISL_1489013, EPI_ISL_1489015, EPI_ISL_1489018 to 1489019, EPI_ISL_1489022 to 1489024, EPI_ISL_1489026 to 1489027, EPI_ISL_1489029 to 1489032, EPI_ISL_1489034, EPI_ISL_1489037 to 1489040, EPI_ISL_1489043 to 1489045, EPI_ISL_1489047, EPI_ISL_1489049, EPI_ISL_1489051 to 1489053, EPI_ISL_1489056 to 1489057, EPI_ISL_1489059 to 1489062, EPI_ISL_1489064 to 1489071, EPI_ISL_1489073 to 1489074, EPI_ISL_1489076 to 1489077, EPI_ISL_1489079 to 1489086, EPI_ISL_1489088, EPI_ISL_1489090 to 1489092, EPI_ISL_1489095 to 1489096, EPI_ISL_1489098 to 1489099, EPI_ISL_1489102 to 1489103, EPI_ISL_1489105 to 1489107, EPI_ISL_1489110, EPI_ISL_1489112, EPI_ISL_1489114, EPI_ISL_1489118 to 1489121, EPI_ISL_1489123 to 1489125, EPI_ISL_1489127 to 1489137, EPI_ISL_1489139, EPI_ISL_1489143 to 1489150, EPI_ISL_1489152, EPI_ISL_1489154, EPI_ISL_1489156 to 1489160, EPI_ISL_1489162, EPI_ISL_1489164, EPI_ISL_1489166, EPI_ISL_1489168 to 1489170, EPI_ISL_1489172, EPI_ISL_1489176 to 1489179, EPI_ISL_1489182, EPI_ISL_1489186, EPI_ISL_1489188, EPI_ISL_1489193, EPI_ISL_1489196, EPI_ISL_1489198, EPI_ISL_1489201, EPI_ISL_1489203, EPI_ISL_1489209 to 1489211, EPI_ISL_1489213, EPI_ISL_1489216, EPI_ISL_1489218, EPI_ISL_1489224, EPI_ISL_1489229 to 1489230, EPI_ISL_1489234 to 1489235, EPI_ISL_1489238, EPI_ISL_1489241, EPI_ISL_1489247, EPI_ISL_1489250, EPI_ISL_1489254 to 1489255, EPI_ISL_1489258 to 1489260, EPI_ISL_1489265 to 1489266, EPI_ISL_1489268 to 1489270, EPI_ISL_1489276 to 1489277, EPI_ISL_1489279, EPI_ISL_1489281 to 1489283, EPI_ISL_1489286 to 1489287, EPI_ISL_1489289 to 1489292, EPI_ISL_1489294, EPI_ISL_1489297 to 1489298, EPI_ISL_1489302 to 1489303, EPI_ISL_1489306, EPI_ISL_1489309 to 1489310, EPI_ISL_1489314 to 1489316, EPI_ISL_1489320, EPI_ISL_1489322, EPI_ISL_1489324, EPI_ISL_1489326 to 1489328, EPI_ISL_1489333, EPI_ISL_1489335, EPI_ISL_1489337, EPI_ISL_1489339, EPI_ISL_1489341 to 1489342, EPI_ISL_1489345, EPI_ISL_1489347 to 1489349, EPI_ISL_1489351, EPI_ISL_1489354, EPI_ISL_1489357, EPI_ISL_1489361, EPI_ISL_1489363 to 1489364, EPI_ISL_1489366 to 1489367, EPI_ISL_1489370 to 1489374, EPI_ISL_1489377, EPI_ISL_1489380, EPI_ISL_1489384 to 1489385, EPI_ISL_1489387, EPI_ISL_1489390, EPI_ISL_1489397, EPI_ISL_1489399, EPI_ISL_1489402, EPI_ISL_1489405 to 1489406, EPI_ISL_1489409 to 1489412, EPI_ISL_1489414 to 1489415, EPI_ISL_1489417 to 1489419, EPI_ISL_1489427, EPI_ISL_1489429, EPI_ISL_1489431 to 1489434, EPI_ISL_1489442, EPI_ISL_1489444, EPI_ISL_1489447, EPI_ISL_1489449, EPI_ISL_1489453, EPI_ISL_1489456, EPI_ISL_1489477 to 1489479, EPI_ISL_1504113 to 1504114, EPI_ISL_1504118, EPI_ISL_1504120 to 1504121, EPI_ISL_1504123 to 1504124, EPI_ISL_1504130 to 1504131, EPI_ISL_1504134 to 1504136, EPI_ISL_1504139, EPI_ISL_1504142, EPI_ISL_1504144, EPI_ISL_1504148, EPI_ISL_1504150, EPI_ISL_1504152, EPI_ISL_1504156 to 1504157, EPI_ISL_1504159 to 1504161, EPI_ISL_1504164, EPI_ISL_1504170 to 1504173, EPI_ISL_1504176, EPI_ISL_1504178 to 1504179, EPI_ISL_1504182, EPI_ISL_1504187, EPI_ISL_1504189 to 1504194, EPI_ISL_1504197 to 1504199, EPI_ISL_1504201 to 1504202, EPI_ISL_1504210 to 1504212, EPI_ISL_1504214 to 1504215, EPI_ISL_1504217, EPI_ISL_1504220 to 1504221, EPI_ISL_1504224, EPI_ISL_1504230, EPI_ISL_1504232, EPI_ISL_1504236, EPI_ISL_1504238, EPI_ISL_1504241 to 1504242, EPI_ISL_1504246, EPI_ISL_1504251 to 1504253, EPI_ISL_1504255, EPI_ISL_1504257 to 1504259, EPI_ISL_1504264, EPI_ISL_1504266 to 1504270, EPI_ISL_1504277 to 1504279, EPI_ISL_1504281 to 1504283, EPI_ISL_1504286 to 1504287, EPI_ISL_1504293, EPI_ISL_1504299 to 1504300, EPI_ISL_1504302, EPI_ISL_1504304 to 1504305, EPI_ISL_1504307 to 1504310, EPI_ISL_1504312, EPI_ISL_1504314 to 1504315, EPI_ISL_1504319, EPI_ISL_1504326, EPI_ISL_1504330 to 1504331, EPI_ISL_1504334, EPI_ISL_1504337 to 1504338, EPI_ISL_1504343, EPI_ISL_1504346, EPI_ISL_1504354, EPI_ISL_1504356, EPI_ISL_1504358, EPI_ISL_1504362, EPI_ISL_1504364, EPI_ISL_1504366, EPI_ISL_1504378 to 1504379, EPI_ISL_1504382 to 1504383, EPI_ISL_1504389 to 1504391, EPI_ISL_1504393, EPI_ISL_1504397 to 1504402, EPI_ISL_1504404, EPI_ISL_1504407, EPI_ISL_1504410, EPI_ISL_1504416 to 1504418, EPI_ISL_1504420, EPI_ISL_1504422, EPI_ISL_1504424, EPI_ISL_1504426, EPI_ISL_1504429 to 1504430, EPI_ISL_1504432, EPI_ISL_1504438 to 1504439, EPI_ISL_1504442 to 1504443, EPI_ISL_1504446, EPI_ISL_1504448 to 1504449, EPI_ISL_1504452, EPI_ISL_1504454, EPI_ISL_1504460 to 1504461, EPI_ISL_1504463 to 1504464, EPI_ISL_1504466 to 1504467, EPI_ISL_1504469, EPI_ISL_1504471, EPI_ISL_1504474 to 1504475, EPI_ISL_1504478 to 1504479, EPI_ISL_1504482, EPI_ISL_1504485, EPI_ISL_1504487, EPI_ISL_1504489 to 1504490, EPI_ISL_1504493, EPI_ISL_1504495, EPI_ISL_1504502, EPI_ISL_1504504 to 1504505, EPI_ISL_1504507, EPI_ISL_1504509 to 1504512, EPI_ISL_1504516, EPI_ISL_1504519, EPI_ISL_1504521, EPI_ISL_1504523, EPI_ISL_1504528, EPI_ISL_1504532 to 1504533, EPI_ISL_1504535, EPI_ISL_1504537, EPI_ISL_1504541 to 1504542, EPI_ISL_1504544 to 1504546, EPI_ISL_1504550, EPI_ISL_1504554, EPI_ISL_1504557 to 1504559, EPI_ISL_1504563 to 1504564, EPI_ISL_1504567, EPI_ISL_1504570, EPI_ISL_1504573, EPI_ISL_1504575, EPI_ISL_1504581, EPI_ISL_1504585 to 1504586, EPI_ISL_1504589, EPI_ISL_1504592 to 1504594, EPI_ISL_1504597, EPI_ISL_1504599 to 1504602, EPI_ISL_1504604, EPI_ISL_1504606, EPI_ISL_1504608, EPI_ISL_1504611, EPI_ISL_1504613, EPI_ISL_1504618 to 1504619, EPI_ISL_1504623 to 1504624, EPI_ISL_1504627 to 1504628, EPI_ISL_1504632 to 1504633, EPI_ISL_1504635, EPI_ISL_1504638, EPI_ISL_1504640 to 1504641, EPI_ISL_1504646 to 1504649, EPI_ISL_1504655, EPI_ISL_1504657 to 1504659, EPI_ISL_1504664, EPI_ISL_1504666, EPI_ISL_1504676 to 1504677, EPI_ISL_1504680 to 1504681, EPI_ISL_1504686 to 1504687, EPI_ISL_1504697, EPI_ISL_1504700, EPI_ISL_1504709, EPI_ISL_1504711, EPI_ISL_1504714, EPI_ISL_1504727, EPI_ISL_1504729, EPI_ISL_1504731, EPI_ISL_1504736, EPI_ISL_1504740 to 1504741, EPI_ISL_1504747, EPI_ISL_1504752, EPI_ISL_1504754, EPI_ISL_1504762 to 1504765, EPI_ISL_1504770, EPI_ISL_1504778, EPI_ISL_1504783, EPI_ISL_1504786, EPI_ISL_1504801, EPI_ISL_1504807, EPI_ISL_1504815, EPI_ISL_1504819, EPI_ISL_1504824, EPI_ISL_1504836, EPI_ISL_1504852 to 1504855, EPI_ISL_1504863 to 1504864, EPI_ISL_1504877, EPI_ISL_1504880, EPI_ISL_1504886, EPI_ISL_1504889 to 1504890, EPI_ISL_1504900, EPI_ISL_1504906, EPI_ISL_1504910, EPI_ISL_1504912, EPI_ISL_1504914, EPI_ISL_1504916, EPI_ISL_1504919, EPI_ISL_1504931, EPI_ISL_1504934 to 1504935, EPI_ISL_1504946 to 1504947, EPI_ISL_1504950, EPI_ISL_1504952, EPI_ISL_1504958, EPI_ISL_1504963, EPI_ISL_1504971 to 1504972, EPI_ISL_1504975 to 1504976, EPI_ISL_1504982 to 1504983, EPI_ISL_1504986, EPI_ISL_1505001, EPI_ISL_1505005 to 1505006, EPI_ISL_1505011, EPI_ISL_1505015, EPI_ISL_1505021 to 1505022, EPI_ISL_1505024, EPI_ISL_1505027, EPI_ISL_1505029, EPI_ISL_1505033, EPI_ISL_1505039 to 1505040, EPI_ISL_1505044, EPI_ISL_1505048, EPI_ISL_1505054, EPI_ISL_1505056, EPI_ISL_1505058, EPI_ISL_1505060, EPI_ISL_1505062, EPI_ISL_1505065, EPI_ISL_1505068 to 1505069, EPI_ISL_1505072 to 1505073, EPI_ISL_1505076, EPI_ISL_1505083, EPI_ISL_1505086, EPI_ISL_1505089, EPI_ISL_1505093, EPI_ISL_1505104 to 1505106, EPI_ISL_1505115, EPI_ISL_1505119 to 1505120, EPI_ISL_1505128, EPI_ISL_1505130, EPI_ISL_1505132, EPI_ISL_1505148, EPI_ISL_1505160, EPI_ISL_1505167, EPI_ISL_1505172, EPI_ISL_1505174, EPI_ISL_1505181, EPI_ISL_1505184, EPI_ISL_1505187, EPI_ISL_1505190 to 1505191, EPI_ISL_1505201, EPI_ISL_1505206 to 1505208, EPI_ISL_1505211, EPI_ISL_1505215, EPI_ISL_1505218, EPI_ISL_1505220 to 1505221, EPI_ISL_1505225, EPI_ISL_1505227, EPI_ISL_1505229 to 1505230, EPI_ISL_1505233, EPI_ISL_1505240, EPI_ISL_1505243, EPI_ISL_1505247 to 1505248, EPI_ISL_1505258, EPI_ISL_1505260 to 1505261, EPI_ISL_1505265, EPI_ISL_1505267, EPI_ISL_1505270, EPI_ISL_1505273, EPI_ISL_1505277, EPI_ISL_1505289, EPI_ISL_1505291, EPI_ISL_1505293 to 1505295, EPI_ISL_1505301, EPI_ISL_1505304, EPI_ISL_1505306 to 1505307, EPI_ISL_1505311, EPI_ISL_1505314, EPI_ISL_1505320, EPI_ISL_1505323, EPI_ISL_1505325, EPI_ISL_1505350, EPI_ISL_1505356, EPI_ISL_1505361 to 1505363, EPI_ISL_1505367, EPI_ISL_1505370 to 1505371, EPI_ISL_1505383, EPI_ISL_1505385, EPI_ISL_1505392 to 1505393, EPI_ISL_1505399, EPI_ISL_1505405, EPI_ISL_1505420 to 1505421, EPI_ISL_1505434, EPI_ISL_1505436, EPI_ISL_1505442, EPI_ISL_1505446, EPI_ISL_1505448, EPI_ISL_1505452 to 1505454, EPI_ISL_1505457 to 1505458, EPI_ISL_1505807, EPI_ISL_1505821 to 1505822, EPI_ISL_1505834, EPI_ISL_1505839, EPI_ISL_1505843, EPI_ISL_1505877, EPI_ISL_1505885 to 1505886, EPI_ISL_1505903, EPI_ISL_1505920, EPI_ISL_1505929, EPI_ISL_1505931, EPI_ISL_1505936, EPI_ISL_1505972, EPI_ISL_1505976, EPI_ISL_1505999, EPI_ISL_1506012, EPI_ISL_1506022, EPI_ISL_1506037 to 1506038, EPI_ISL_1506048, EPI_ISL_1506056, EPI_ISL_1506072, EPI_ISL_1506083, EPI_ISL_1506134 to 1506138, EPI_ISL_1506140 to 1506143, EPI_ISL_1506146 to 1506156, EPI_ISL_1506160 to 1506162, EPI_ISL_1506164, EPI_ISL_1506166 to 1506171, EPI_ISL_1506173 to 1506174, EPI_ISL_1506176 to 1506191, EPI_ISL_1506193 to 1506265, EPI_ISL_1506267 to 1506276, EPI_ISL_1506279 to 1506291, EPI_ISL_1506293 to 1506304, EPI_ISL_1506306 to 1506352, EPI_ISL_1506354 to 1506406, EPI_ISL_1506408 to 1506423, EPI_ISL_1506428 to 1506433, EPI_ISL_1506435 to 1506439, EPI_ISL_1506441 to 1506460, EPI_ISL_1506462 to 1506479, EPI_ISL_1506481 to 1506513, EPI_ISL_1506515, EPI_ISL_1506517 to 1506519, EPI_ISL_1506521 to 1506527, EPI_ISL_1506537 to 1506548, EPI_ISL_1506550 to 1506561, EPI_ISL_1506563, EPI_ISL_1506565 to 1506570, EPI_ISL_1506575 to 1506667, EPI_ISL_1506669 to 1507040, EPI_ISL_1507045 to 1507195, EPI_ISL_1507198 to 1507210, EPI_ISL_1507212, EPI_ISL_1507214 to 1507231, EPI_ISL_1507233 to 1507482, EPI_ISL_1507484 to 1507518, EPI_ISL_1507520 to 1507572, EPI_ISL_1507581 to 1507596, EPI_ISL_1507598 to 1507706, EPI_ISL_1507710 to 1507765, EPI_ISL_1507767 to 1507793, EPI_ISL_1507795, EPI_ISL_1507797 to 1507798, EPI_ISL_1507801, EPI_ISL_1507803 to 1507804, EPI_ISL_1507808, EPI_ISL_1507810 to 1507812, EPI_ISL_1507819 to 1507851, EPI_ISL_1507853 to 1507876, EPI_ISL_1507878 to 1507941, EPI_ISL_1507943 to 1507949, EPI_ISL_1507951 to 1507987, EPI_ISL_1507989 to 1508007, EPI_ISL_1508009 to 1508437, EPI_ISL_1508439 to 1508490, EPI_ISL_1508492 to 1508559, EPI_ISL_1508562 to 1508647, EPI_ISL_1508649 to 1508710, EPI_ISL_1508712 to 1508819, EPI_ISL_1517497, EPI_ISL_1517499, EPI_ISL_1517504, EPI_ISL_1517506 to 1517507, EPI_ISL_1517510 to 1517511, EPI_ISL_1517513 to 1517515, EPI_ISL_1517519 to 1517520, EPI_ISL_1517522, EPI_ISL_1517524 to 1517526, EPI_ISL_1517531, EPI_ISL_1517533 to 1517536, EPI_ISL_1517542 to 1517543, EPI_ISL_1517545, EPI_ISL_1517547, EPI_ISL_1517549, EPI_ISL_1517551 to 1517552, EPI_ISL_1517554, EPI_ISL_1517559, EPI_ISL_1517561 to 1517562, EPI_ISL_1517564 to 1517569, EPI_ISL_1517571 to 1517572, EPI_ISL_1517574 to 1517575, EPI_ISL_1517578, EPI_ISL_1517581 to 1517583, EPI_ISL_1517585 to 1517591, EPI_ISL_1517593 to 1517602, EPI_ISL_1517605 to 1517606, EPI_ISL_1517608 to 1517614, EPI_ISL_1517616 to 1517622, EPI_ISL_1517624 to 1517629, EPI_ISL_1517631 to 1517634, EPI_ISL_1517636 to 1517639, EPI_ISL_1517641 to 1517644, EPI_ISL_1517646, EPI_ISL_1517648, EPI_ISL_1517650 to 1517653, EPI_ISL_1517655 to 1517665, EPI_ISL_1517667, EPI_ISL_1517669 to 1517670, EPI_ISL_1517672 to 1517674, EPI_ISL_1517680, EPI_ISL_1517682 to 1517684, EPI_ISL_1517686 to 1517687, EPI_ISL_1517689 to 1517696, EPI_ISL_1517699, EPI_ISL_1517701, EPI_ISL_1517703 to 1517705, EPI_ISL_1517707, EPI_ISL_1517709 to 1517710, EPI_ISL_1517712 to 1517716, EPI_ISL_1517720 to 1517722, EPI_ISL_1517724, EPI_ISL_1517726 to 1517728, EPI_ISL_1517730, EPI_ISL_1517732 to 1517733, EPI_ISL_1517737 to 1517739, EPI_ISL_1517741 to 1517742, EPI_ISL_1517744 to 1517745, EPI_ISL_1517747, EPI_ISL_1517749 to 1517750, EPI_ISL_1517752, EPI_ISL_1518393 to 1518394, EPI_ISL_1518396, EPI_ISL_1518399, EPI_ISL_1518405 to 1518406, EPI_ISL_1518410 to 1518411, EPI_ISL_1518413 to 1518414, EPI_ISL_1518418 to 1518421, EPI_ISL_1518423, EPI_ISL_1518425 to 1518426, EPI_ISL_1518431 to 1518432, EPI_ISL_1518434, EPI_ISL_1518439, EPI_ISL_1518444, EPI_ISL_1518448 to 1518449, EPI_ISL_1518451, EPI_ISL_1518455 to 1518456, EPI_ISL_1518458 to 1518459, EPI_ISL_1518461 to 1518462, EPI_ISL_1518465, EPI_ISL_1518476, EPI_ISL_1518478, EPI_ISL_1518481, EPI_ISL_1518483 to 1518484, EPI_ISL_1518486, EPI_ISL_1518488 to 1518489, EPI_ISL_1518493, EPI_ISL_1518499, EPI_ISL_1518501, EPI_ISL_1518503, EPI_ISL_1518507, EPI_ISL_1518511, EPI_ISL_1518513, EPI_ISL_1518515, EPI_ISL_1518519, EPI_ISL_1518521, EPI_ISL_1518525, EPI_ISL_1518528, EPI_ISL_1518530 to 1518532, EPI_ISL_1518534 to 1518539, EPI_ISL_1518542, EPI_ISL_1518548 to 1518549, EPI_ISL_1518553 to 1518554, EPI_ISL_1518556 to 1518559, EPI_ISL_1518562 to 1518565, EPI_ISL_1518576, EPI_ISL_1518579, EPI_ISL_1518582, EPI_ISL_1518584, EPI_ISL_1518589 to 1518590, EPI_ISL_1518593 to 1518594, EPI_ISL_1518598, EPI_ISL_1518604, EPI_ISL_1518607 to 1518608, EPI_ISL_1518610, EPI_ISL_1518612 to 1518613, EPI_ISL_1518620 to 1518623, EPI_ISL_1518626, EPI_ISL_1518628, EPI_ISL_1518636, EPI_ISL_1518638 to 1518639, EPI_ISL_1518641, EPI_ISL_1518645 to 1518647, EPI_ISL_1518649 to 1518650, EPI_ISL_1518653 to 1518654, EPI_ISL_1518657 to 1518658, EPI_ISL_1518665 to 1518667, EPI_ISL_1518672, EPI_ISL_1518674, EPI_ISL_1518995, EPI_ISL_1518998, EPI_ISL_1519001 to 1519003, EPI_ISL_1519006 to 1519008, EPI_ISL_1519016, EPI_ISL_1519019, EPI_ISL_1519021, EPI_ISL_1519029 to 1519032, EPI_ISL_1519036, EPI_ISL_1519039, EPI_ISL_1519046, EPI_ISL_1519048, EPI_ISL_1519050, EPI_ISL_1519056, EPI_ISL_1519058 to 1519060, EPI_ISL_1519062, EPI_ISL_1519064 to 1519065, EPI_ISL_1519067, EPI_ISL_1519069, EPI_ISL_1519072 to 1519075, EPI_ISL_1519077 to 1519078, EPI_ISL_1519086 to 1519087, EPI_ISL_1519089, EPI_ISL_1519092, EPI_ISL_1519095 to 1519096, EPI_ISL_1519100 to 1519101, EPI_ISL_1519104, EPI_ISL_1519107, EPI_ISL_1519109, EPI_ISL_1519112 to 1519114, EPI_ISL_1519116, EPI_ISL_1519120, EPI_ISL_1519125, EPI_ISL_1519127, EPI_ISL_1519133, EPI_ISL_1519138 to 1519139, EPI_ISL_1519145 to 1519147, EPI_ISL_1519150, EPI_ISL_1519154 to 1519157, EPI_ISL_1519162, EPI_ISL_1519169, EPI_ISL_1519175, EPI_ISL_1519178, EPI_ISL_1519185, EPI_ISL_1519189 to 1519190, EPI_ISL_1519192 to 1519193, EPI_ISL_1519196 to 1519197, EPI_ISL_1519200, EPI_ISL_1519202, EPI_ISL_1519204, EPI_ISL_1519208, EPI_ISL_1519212, EPI_ISL_1519215 to 1519216, EPI_ISL_1519218, EPI_ISL_1519222, EPI_ISL_1519224 to 1519226, EPI_ISL_1519228, EPI_ISL_1519230, EPI_ISL_1519233, EPI_ISL_1519237, EPI_ISL_1519240, EPI_ISL_1519243 to 1519244, EPI_ISL_1519247, EPI_ISL_1519249 to 1519250, EPI_ISL_1519252 to 1519255, EPI_ISL_1519259 to 1519261, EPI_ISL_1519263, EPI_ISL_1519266 to 1519268, EPI_ISL_1519309 to 1519310, EPI_ISL_1519312 to 1519313, EPI_ISL_1519318, EPI_ISL_1519322 to 1519323, EPI_ISL_1519326, EPI_ISL_1519330, EPI_ISL_1519336, EPI_ISL_1519338, EPI_ISL_1519348, EPI_ISL_1519387, EPI_ISL_1519420 to 1519422, EPI_ISL_1519429 to 1519430, EPI_ISL_1519434, EPI_ISL_1519448, EPI_ISL_1519451, EPI_ISL_1519455, EPI_ISL_1519473, EPI_ISL_1519479, EPI_ISL_1519484, EPI_ISL_1519486, EPI_ISL_1519488 to 1519489, EPI_ISL_1519495, EPI_ISL_1519500, EPI_ISL_1519511, EPI_ISL_1519514, EPI_ISL_1519531 to 1519532, EPI_ISL_1519538, EPI_ISL_1519552 to 1519554, EPI_ISL_1519557, EPI_ISL_1519568, EPI_ISL_1519572, EPI_ISL_1519575, EPI_ISL_1519577, EPI_ISL_1519586, EPI_ISL_1519588 to 1519589, EPI_ISL_1519593 to 1519594, EPI_ISL_1519597 to 1519598, EPI_ISL_1519601 to 1519602, EPI_ISL_1519607, EPI_ISL_1519609, EPI_ISL_1519616, EPI_ISL_1519627, EPI_ISL_1519639, EPI_ISL_1519645, EPI_ISL_1519650 to 1519909, EPI_ISL_1534751, EPI_ISL_1534761, EPI_ISL_1534774, EPI_ISL_1534776, EPI_ISL_1534801, EPI_ISL_1534806, EPI_ISL_1534842, EPI_ISL_1534845, EPI_ISL_1534848, EPI_ISL_1534852, EPI_ISL_1534868, EPI_ISL_1534872, EPI_ISL_1534880, EPI_ISL_1534891, EPI_ISL_1534894, EPI_ISL_1534911, EPI_ISL_1534916, EPI_ISL_1534925, EPI_ISL_1534942, EPI_ISL_1534950, EPI_ISL_1534953, EPI_ISL_1534960, EPI_ISL_1534974, EPI_ISL_1534977, EPI_ISL_1534987, EPI_ISL_1534990, EPI_ISL_1535337, EPI_ISL_1535357, EPI_ISL_1535360, EPI_ISL_1535364, EPI_ISL_1535366, EPI_ISL_1535377 to 1535378, EPI_ISL_1535380 to 1535381, EPI_ISL_1535385, EPI_ISL_1535387 to 1535388, EPI_ISL_1535392, EPI_ISL_1535399, EPI_ISL_1535403 to 1535404, EPI_ISL_1535406, EPI_ISL_1535409 to 1535410, EPI_ISL_1535413, EPI_ISL_1535416, EPI_ISL_1535418 to 1535419, EPI_ISL_1535422 to 1535423, EPI_ISL_1535428 to 1535430, EPI_ISL_1535432, EPI_ISL_1535435, EPI_ISL_1535437, EPI_ISL_1535446, EPI_ISL_1535448, EPI_ISL_1535457, EPI_ISL_1535460, EPI_ISL_1535462, EPI_ISL_1535465 to 1535467, EPI_ISL_1535470 to 1535471, EPI_ISL_1535473 to 1535475, EPI_ISL_1535481, EPI_ISL_1535483, EPI_ISL_1535485, EPI_ISL_1535490, EPI_ISL_1535492, EPI_ISL_1535494, EPI_ISL_1535498, EPI_ISL_1535501 to 1535502, EPI_ISL_1535504, EPI_ISL_1535517 to 1535519, EPI_ISL_1535522 to 1535523, EPI_ISL_1535528 to 1535530, EPI_ISL_1535537, EPI_ISL_1535540 to 1535542, EPI_ISL_1535544, EPI_ISL_1535546, EPI_ISL_1535549, EPI_ISL_1535553, EPI_ISL_1535555 to 1535556, EPI_ISL_1535559 to 1535560, EPI_ISL_1535564, EPI_ISL_1535568, EPI_ISL_1535573, EPI_ISL_1535578, EPI_ISL_1535580, EPI_ISL_1535583, EPI_ISL_1535585, EPI_ISL_1535590, EPI_ISL_1535594, EPI_ISL_1535596, EPI_ISL_1535599, EPI_ISL_1535610 to 1535611, EPI_ISL_1535614, EPI_ISL_1535616, EPI_ISL_1535622, EPI_ISL_1535634, EPI_ISL_1535649, EPI_ISL_1535655 to 1535656, EPI_ISL_1535661, EPI_ISL_1535667, EPI_ISL_1535672 to 1535673, EPI_ISL_1535677, EPI_ISL_1535679, EPI_ISL_1535682, EPI_ISL_1535685, EPI_ISL_1535687, EPI_ISL_1535692, EPI_ISL_1535698, EPI_ISL_1535708, EPI_ISL_1535714, EPI_ISL_1535716, EPI_ISL_1535722, EPI_ISL_1535725, EPI_ISL_1535730, EPI_ISL_1535736, EPI_ISL_1535742, EPI_ISL_1535744, EPI_ISL_1535751, EPI_ISL_1535754, EPI_ISL_1535764, EPI_ISL_1535766, EPI_ISL_1535773 to 1535774, EPI_ISL_1535782 to 1535783, EPI_ISL_1535802 to 1535803, EPI_ISL_1535807, EPI_ISL_1535814, EPI_ISL_1535816, EPI_ISL_1535824, EPI_ISL_1535828 to 1535829, EPI_ISL_1535838, EPI_ISL_1535841, EPI_ISL_1535846, EPI_ISL_1535851 to 1535852, EPI_ISL_1535857, EPI_ISL_1535861, EPI_ISL_1535867, EPI_ISL_1535870 to 1535871, EPI_ISL_1535874 to 1535875, EPI_ISL_1535879, EPI_ISL_1535886, EPI_ISL_1535892, EPI_ISL_1535897, EPI_ISL_1535903, EPI_ISL_1535907, EPI_ISL_1535910, EPI_ISL_1535912, EPI_ISL_1535916, EPI_ISL_1535919 to 1535921, EPI_ISL_1535924, EPI_ISL_1535934, EPI_ISL_1535937, EPI_ISL_1535939, EPI_ISL_1535944, EPI_ISL_1535950 to 1535951, EPI_ISL_1535963, EPI_ISL_1535966, EPI_ISL_1535969, EPI_ISL_1535975 to 1535976, EPI_ISL_1535982 to 1535985, EPI_ISL_1535991 to 1535992, EPI_ISL_1535994, EPI_ISL_1535999, EPI_ISL_1536001, EPI_ISL_1536005 to 1536006, EPI_ISL_1536008, EPI_ISL_1536024 to 1536025, EPI_ISL_1536028 to 1536030, EPI_ISL_1536036, EPI_ISL_1536041 to 1536043, EPI_ISL_1536049, EPI_ISL_1536062, EPI_ISL_1536068, EPI_ISL_1536071, EPI_ISL_1536108, EPI_ISL_1536111, EPI_ISL_1536116, EPI_ISL_1536120, EPI_ISL_1536125 to 1536126, EPI_ISL_1536131 to 1536132, EPI_ISL_1536137 to 1536138, EPI_ISL_1536144, EPI_ISL_1536149, EPI_ISL_1536151 to 1536152, EPI_ISL_1536156, EPI_ISL_1536160 to 1536161, EPI_ISL_1536171 to 1536172, EPI_ISL_1536175, EPI_ISL_1536179, EPI_ISL_1536539, EPI_ISL_1536542 to 1536544, EPI_ISL_1536558 to 1536560, EPI_ISL_1536568, EPI_ISL_1536570, EPI_ISL_1536574, EPI_ISL_1536580 to 1536581, EPI_ISL_1536584, EPI_ISL_1536588, EPI_ISL_1536590, EPI_ISL_1536597, EPI_ISL_1536599 to 1536600, EPI_ISL_1536603 to 1536605, EPI_ISL_1536607, EPI_ISL_1536609, EPI_ISL_1536613 to 1536614, EPI_ISL_1536617, EPI_ISL_1536626, EPI_ISL_1536628, EPI_ISL_1536631 to 1536632, EPI_ISL_1536634, EPI_ISL_1536638, EPI_ISL_1536646, EPI_ISL_1536654 to 1536655, EPI_ISL_1536660, EPI_ISL_1536665, EPI_ISL_1536671, EPI_ISL_1536680, EPI_ISL_1536683, EPI_ISL_1536685, EPI_ISL_1536689 to 1536690, EPI_ISL_1536693 to 1536694,

EPI_ISL_1536698, EPI_ISL_1536701 to 1536703, EPI_ISL_1536708, EPI_ISL_1536710, EPI_ISL_1536714 to 1536716, EPI_ISL_1536719, EPI_ISL_1536725, EPI_ISL_1536729, EPI_ISL_1536733, EPI_ISL_1536739, EPI_ISL_1536742 to 1536743, EPI_ISL_1536745, EPI_ISL_1536747, EPI_ISL_1536753 to 1536754, EPI_ISL_1536756, EPI_ISL_1536760, EPI_ISL_1536766, EPI_ISL_1536768, EPI_ISL_1536770, EPI_ISL_1536773, EPI_ISL_1536775, EPI_ISL_1536778, EPI_ISL_1536780, EPI_ISL_1536782 to 1536783, EPI_ISL_1536788 to 1536790, EPI_ISL_1536792 to 1536796, EPI_ISL_1536799 to 1536800, EPI_ISL_1536802, EPI_ISL_1536804, EPI_ISL_1536806, EPI_ISL_1536809 to 1536811, EPI_ISL_1536813, EPI_ISL_1536816 to 1536817, EPI_ISL_1536821, EPI_ISL_1536823 to 1536824, EPI_ISL_1536827, EPI_ISL_1536829, EPI_ISL_1536831, EPI_ISL_1536835, EPI_ISL_1536837, EPI_ISL_1536841 to 1536842, EPI_ISL_1536844 to 1536845, EPI_ISL_1536847, EPI_ISL_1536849, EPI_ISL_1536851, EPI_ISL_1536854 to 1536855, EPI_ISL_1536857 to 1536859, EPI_ISL_1536861, EPI_ISL_1536869 to 1536870, EPI_ISL_1536872 to 1536874, EPI_ISL_1536878 to 1536882, EPI_ISL_1536886, EPI_ISL_1536888, EPI_ISL_1536891, EPI_ISL_1536893, EPI_ISL_1536897, EPI_ISL_1536903, EPI_ISL_1536905 to 1536906, EPI_ISL_1536911 to 1536912, EPI_ISL_1536914, EPI_ISL_1536917 to 1536919, EPI_ISL_1536923, EPI_ISL_1536925 to 1536926, EPI_ISL_1536930, EPI_ISL_1536933, EPI_ISL_1536937 to 1536938, EPI_ISL_1536942, EPI_ISL_1536944 to 1536945, EPI_ISL_1536947 to 1536948, EPI_ISL_1536951, EPI_ISL_1536953 to 1536954, EPI_ISL_1536956, EPI_ISL_1536959, EPI_ISL_1536962 to 1536964, EPI_ISL_1536966 to 1536969, EPI_ISL_1536972 to 1536976, EPI_ISL_1536978 to 1536979, EPI_ISL_1536981, EPI_ISL_1536984 to 1536985, EPI_ISL_1536987, EPI_ISL_1536990 to 1536991, EPI_ISL_1536993 to 1536995, EPI_ISL_1536998, EPI_ISL_1537001 to 1537004, EPI_ISL_1537007 to 1537009, EPI_ISL_1537013, EPI_ISL_1537015, EPI_ISL_1537017, EPI_ISL_1537020, EPI_ISL_1537022, EPI_ISL_1537024, EPI_ISL_1537026, EPI_ISL_1537029 to 1537030, EPI_ISL_1537032, EPI_ISL_1537034, EPI_ISL_1537036, EPI_ISL_1537043, EPI_ISL_1537045 to 1537046, EPI_ISL_1537050, EPI_ISL_1537053 to 1537055, EPI_ISL_1537057 to 1537058, EPI_ISL_1537060, EPI_ISL_1537063 to 1537064, EPI_ISL_1537068 to 1537069, EPI_ISL_1537073 to 1537074, EPI_ISL_1537076, EPI_ISL_1537080 to 1537083, EPI_ISL_1537085 to 1537087, EPI_ISL_1537089, EPI_ISL_1537095 to 1537098, EPI_ISL_1537100, EPI_ISL_1537102 to 1537103, EPI_ISL_1537107, EPI_ISL_1537110 to 1537112, EPI_ISL_1537116 to 1537120, EPI_ISL_1537123, EPI_ISL_1537125, EPI_ISL_1537129 to 1537130, EPI_ISL_1537141 to 1537151, EPI_ISL_1537153 to 1537192, EPI_ISL_1537194 to 1537225, EPI_ISL_1537227 to 1537235, EPI_ISL_1537237 to 1537239, EPI_ISL_1537241 to 1537314, EPI_ISL_1537329, EPI_ISL_1537333, EPI_ISL_1537336 to 1537337, EPI_ISL_1537341, EPI_ISL_1537343 to 1537344, EPI_ISL_1537346, EPI_ISL_1537349 to 1537350, EPI_ISL_1537352 to 1537354, EPI_ISL_1537356 to 1537358, EPI_ISL_1537362, EPI_ISL_1537366, EPI_ISL_1537368, EPI_ISL_1537371, EPI_ISL_1537379, EPI_ISL_1537381, EPI_ISL_1537383 to 1537384, EPI_ISL_1537389, EPI_ISL_1537391 to 1537393, EPI_ISL_1537396 to 1537398, EPI_ISL_1537400 to 1537402, EPI_ISL_1537408, EPI_ISL_1537413, EPI_ISL_1537416 to 1537417, EPI_ISL_1537423 to 1537425, EPI_ISL_1537427, EPI_ISL_1537433, EPI_ISL_1537436, EPI_ISL_1537438, EPI_ISL_1537441 to 1537443, EPI_ISL_1537445, EPI_ISL_1537447, EPI_ISL_1537449 to 1537452, EPI_ISL_1537455, EPI_ISL_1537457 to 1537462, EPI_ISL_1537464 to 1537465, EPI_ISL_1537468, EPI_ISL_1537470 to 1537471, EPI_ISL_1537473 to 1537474, EPI_ISL_1537477, EPI_ISL_1537479, EPI_ISL_1537482, EPI_ISL_1537487, EPI_ISL_1537489 to 1537494, EPI_ISL_1537497 to 1537501, EPI_ISL_1537504 to 1537507, EPI_ISL_1537509 to 1537510, EPI_ISL_1537514 to 1537516, EPI_ISL_1537518, EPI_ISL_1537522, EPI_ISL_1537524 to 1537525, EPI_ISL_1537527, EPI_ISL_1537529 to 1537531, EPI_ISL_1537533, EPI_ISL_1537535, EPI_ISL_1537537 to 1537538, EPI_ISL_1537542, EPI_ISL_1537546, EPI_ISL_1537548, EPI_ISL_1537550, EPI_ISL_1537552, EPI_ISL_1537554 to 1537556, EPI_ISL_1537559, EPI_ISL_1537561, EPI_ISL_1537565 to 1537570, EPI_ISL_1537575, EPI_ISL_1537578 to 1537581, EPI_ISL_1537585, EPI_ISL_1537587, EPI_ISL_1537592 to 1537594, EPI_ISL_1537596, EPI_ISL_1537599, EPI_ISL_1537605 to 1537607, EPI_ISL_1537614, EPI_ISL_1537617, EPI_ISL_1537622, EPI_ISL_1537625, EPI_ISL_1537631, EPI_ISL_1537636, EPI_ISL_1537639 to 1537641, EPI_ISL_1537643 to 1537644, EPI_ISL_1537646, EPI_ISL_1537648 to 1537650, EPI_ISL_1537652, EPI_ISL_1537656 to 1537657, EPI_ISL_1537660, EPI_ISL_1537662, EPI_ISL_1537668, EPI_ISL_1537672, EPI_ISL_1537675 to 1537678, EPI_ISL_1537680 to 1537681, EPI_ISL_1537683 to 1537685, EPI_ISL_1537688, EPI_ISL_1537691, EPI_ISL_1537696 to 1537699, EPI_ISL_1537703, EPI_ISL_1537706 to 1537707, EPI_ISL_1537712 to 1537713, EPI_ISL_1537716 to 1537717, EPI_ISL_1537721, EPI_ISL_1537725, EPI_ISL_1537727, EPI_ISL_1537730 to 1537731, EPI_ISL_1537733 to 1537735, EPI_ISL_1537737, EPI_ISL_1537739 to 1537741, EPI_ISL_1537743 to 1537746, EPI_ISL_1537750 to 1537752, EPI_ISL_1537755 to 1537757, EPI_ISL_1537761 to 1537762, EPI_ISL_1537764 to 1537765, EPI_ISL_1537769, EPI_ISL_1537771, EPI_ISL_1537773, EPI_ISL_1537777, EPI_ISL_1537779 to 1537784, EPI_ISL_1537787, EPI_ISL_1537792, EPI_ISL_1537795 to 1537797, EPI_ISL_1537799, EPI_ISL_1537803, EPI_ISL_1537805, EPI_ISL_1537809, EPI_ISL_1537811 to 1537812, EPI_ISL_1537815, EPI_ISL_1537817 to 1537818, EPI_ISL_1537821, EPI_ISL_1537824 to 1537825, EPI_ISL_1537831 to 1537833, EPI_ISL_1537835 to 1537836, EPI_ISL_1537839, EPI_ISL_1537841, EPI_ISL_1537844 to 1538019, EPI_ISL_1538021 to 1538028, EPI_ISL_1538030, EPI_ISL_1538032 to 1538035, EPI_ISL_1538037 to 1538038, EPI_ISL_1538040 to 1538042, EPI_ISL_1538044 to 1538048, EPI_ISL_1538050 to 1538052, EPI_ISL_1538054 to 1538056, EPI_ISL_1538058 to 1538062, EPI_ISL_1538064 to 1538066, EPI_ISL_1538068, EPI_ISL_1538070, EPI_ISL_1538074 to 1538077, EPI_ISL_1538079, EPI_ISL_1538081 to 1538083, EPI_ISL_1538085 to 1538087, EPI_ISL_1538089, EPI_ISL_1538092, EPI_ISL_1538094 to 1538101, EPI_ISL_1538103 to 1538152, EPI_ISL_1538154 to 1538272, EPI_ISL_1538274 to 1538276, EPI_ISL_1538279 to 1538282, EPI_ISL_1538284 to 1538287, EPI_ISL_1538289 to 1538293, EPI_ISL_1538295 to 1538296, EPI_ISL_1538298 to 1538299, EPI_ISL_1538302 to 1538303, EPI_ISL_1538305 to 1538372, EPI_ISL_1538374 to 1538396, EPI_ISL_1538398 to 1538401, EPI_ISL_1538403, EPI_ISL_1544197, EPI_ISL_1544201, EPI_ISL_1544204 to 1544205, EPI_ISL_1544207 to 1544209, EPI_ISL_1544211, EPI_ISL_1544214 to 1544217, EPI_ISL_1544219, EPI_ISL_1544221, EPI_ISL_1544225 to 1544226, EPI_ISL_1544228, EPI_ISL_1544231 to 1544232, EPI_ISL_1544234 to 1544236, EPI_ISL_1544242 to 1544243, EPI_ISL_1544246, EPI_ISL_1544249 to 1544250, EPI_ISL_1544253 to 1544254, EPI_ISL_1544262 to 1544263, EPI_ISL_1544270, EPI_ISL_1544273, EPI_ISL_1544276 to 1544277, EPI_ISL_1544280, EPI_ISL_1544282, EPI_ISL_1544285 to 1544286, EPI_ISL_1544290, EPI_ISL_1544297, EPI_ISL_1544300, EPI_ISL_1544302, EPI_ISL_1544304 to 1544306, EPI_ISL_1544310 to 1544311, EPI_ISL_1544313, EPI_ISL_1544315, EPI_ISL_1544320 to 1544326, EPI_ISL_1544328, EPI_ISL_1544332 to 1544333, EPI_ISL_1544336 to 1544338, EPI_ISL_1544340 to 1544341, EPI_ISL_1544343, EPI_ISL_1544346 to 1544347, EPI_ISL_1544351 to 1544352, EPI_ISL_1544357, EPI_ISL_1544359 to 1544365, EPI_ISL_1544371 to 1544372, EPI_ISL_1544375 to 1544378, EPI_ISL_1544380 to 1544381, EPI_ISL_1544387, EPI_ISL_1544389, EPI_ISL_1544394 to 1544395, EPI_ISL_1544398 to 1544401, EPI_ISL_1544403 to 1544404, EPI_ISL_1544406, EPI_ISL_1544410 to 1544413, EPI_ISL_1544417 to 1544418, EPI_ISL_1544420, EPI_ISL_1544423, EPI_ISL_1544431, EPI_ISL_1544434 to 1544436, EPI_ISL_1544438, EPI_ISL_1544440 to 1544441, EPI_ISL_1544443 to 1544444, EPI_ISL_1544446 to 1544449, EPI_ISL_1544454, EPI_ISL_1544456, EPI_ISL_1544458 to 1544459, EPI_ISL_1544461, EPI_ISL_1544463, EPI_ISL_1544467 to 1544470, EPI_ISL_1544475, EPI_ISL_1544477 to 1544478, EPI_ISL_1544484, EPI_ISL_1544486, EPI_ISL_1544489, EPI_ISL_1544491 to 1544492, EPI_ISL_1544494 to 1544777, EPI_ISL_1544779 to 1544782, EPI_ISL_1544784 to 1544791, EPI_ISL_1544793 to 1544863, EPI_ISL_1544865 to 1544933, EPI_ISL_1544935 to 1544938, EPI_ISL_1544940 to 1545101

see above	Lighthouse Lab in Glasgow	Wellcome Sanger Institute for the COVID-19 Genomics UK (COG-UK) Consortium	Anna Dominiczak and Alex Alderton; Carol Clugston; Cordelia Langford; David Gray; David K. Jackson; Dominic Kwiatkowski; Ewan Harrison; Harper VanSteenhouse; Ian Johnston; Jeffrey Barrett; John Sillitoe on behalf of the Wellcome Sanger Institute COVID-19 Surveillance Team; John Sillitoe on behalf of the Wellcome Sanger Institute COVID-19 Surveillance Team (http://www.sanger.ac.uk/covid-team); Roberto Amato; Sonia Goncalves; Yumi Kasai

EPI_ISL_530351 to 530360, EPI_ISL_530362 to 530365, EPI_ISL_530367 to 530369, EPI_ISL_530371 to 530376, EPI_ISL_530378 to 530385, EPI_ISL_530387, EPI_ISL_530389 to 530390, EPI_ISL_530392 to 530397, EPI_ISL_530399 to 530409, EPI_ISL_530411 to 530428, EPI_ISL_530430 to 530434, EPI_ISL_530436 to 530439, EPI_ISL_530441 to 530467, EPI_ISL_530469 to 530479, EPI_ISL_530481, EPI_ISL_530483 to 530487, EPI_ISL_530490 to 530522, EPI_ISL_530524 to 530527, EPI_ISL_530529 to 530532, EPI_ISL_530534, EPI_ISL_530536 to 530541, EPI_ISL_530543 to 530552, EPI_ISL_530554 to 530561, EPI_ISL_530563 to 530595, EPI_ISL_530597 to 530641, EPI_ISL_530643 to 530658, EPI_ISL_530660 to 530672, EPI_ISL_530674 to 530681, EPI_ISL_530683 to 530688, EPI_ISL_530690 to 530707, EPI_ISL_530709 to 530721, EPI_ISL_530723 to 530739, EPI_ISL_530741 to 530742, EPI_ISL_530744 to 530745, EPI_ISL_530747 to 530753, EPI_ISL_530755 to 530763, EPI_ISL_530765, EPI_ISL_530769 to 530825, EPI_ISL_530827 to 530835, EPI_ISL_530837 to 530844, EPI_ISL_530846 to 530861, EPI_ISL_530863 to 530870, EPI_ISL_530872, EPI_ISL_530874 to 530898, EPI_ISL_530900 to 530903, EPI_ISL_530905, EPI_ISL_530907 to 530922, EPI_ISL_530924 to 530928, EPI_ISL_530930 to 530952, EPI_ISL_530954 to 530958, EPI_ISL_530960, EPI_ISL_530964 to 530972, EPI_ISL_530974 to 530975, EPI_ISL_530977 to 530978, EPI_ISL_530980 to 530982, EPI_ISL_530984, EPI_ISL_530987 to 530989, EPI_ISL_530991 to 530995, EPI_ISL_530998 to 531001, EPI_ISL_531003, EPI_ISL_531005, EPI_ISL_531007 to 531016, EPI_ISL_531018 to 531019, EPI_ISL_531021 to 531031, EPI_ISL_531034 to 531037, EPI_ISL_531039, EPI_ISL_531041 to 531044, EPI_ISL_531046 to 531047, EPI_ISL_531050 to 531051, EPI_ISL_531053, EPI_ISL_531055, EPI_ISL_531058 to 531064, EPI_ISL_531066 to 531078, EPI_ISL_531080 to 531082, EPI_ISL_531084 to 531086, EPI_ISL_531088 to 531090, EPI_ISL_531092 to 531095, EPI_ISL_531097 to 531098, EPI_ISL_531101 to 531102, EPI_ISL_531105 to 531113, EPI_ISL_531115, EPI_ISL_531117, EPI_ISL_531119 to 531122, EPI_ISL_531124 to 531125, EPI_ISL_531127 to 531129, EPI_ISL_531132 to 531141, EPI_ISL_531143 to 531146, EPI_ISL_531148 to 531153, EPI_ISL_531158, EPI_ISL_531160 to 531165, EPI_ISL_531167 to 531173, EPI_ISL_531176 to 531187, EPI_ISL_531190 to 531197, EPI_ISL_531199 to 531206, EPI_ISL_531208 to 531210, EPI_ISL_531213, EPI_ISL_531215 to 531222, EPI_ISL_531224 to 531248, EPI_ISL_531250 to 531265, EPI_ISL_531267 to 531320, EPI_ISL_531322 to 531329, EPI_ISL_531331 to 531364, EPI_ISL_531366 to 531373, EPI_ISL_531375 to 531381, EPI_ISL_531383 to 531411, EPI_ISL_531413 to 531415, EPI_ISL_531417 to 531419, EPI_ISL_531421, EPI_ISL_531423 to 531431, EPI_ISL_531433 to 531441, EPI_ISL_531443 to 531463, EPI_ISL_531465 to 531469, EPI_ISL_531471 to 531472, EPI_ISL_531474 to 531475, EPI_ISL_531478 to 531479, EPI_ISL_531481 to 531482, EPI_ISL_531486 to 531491, EPI_ISL_531493 to 531494, EPI_ISL_531496 to 531499, EPI_ISL_531501 to 531503, EPI_ISL_531505 to 531523, EPI_ISL_531525 to 531545, EPI_ISL_531547, EPI_ISL_531549 to 531556, EPI_ISL_531558 to 531562, EPI_ISL_531566 to 531568, EPI_ISL_531571 to 531574, EPI_ISL_531576, EPI_ISL_531578 to 531582, EPI_ISL_531584 to 531589, EPI_ISL_531591 to 531594, EPI_ISL_531596 to 531597, EPI_ISL_531600 to 531606, EPI_ISL_531608, EPI_ISL_531610 to 531616, EPI_ISL_531619 to 531624, EPI_ISL_531626 to 531630, EPI_ISL_531632 to 531633, EPI_ISL_531635 to 531637, EPI_ISL_531640 to 531648, EPI_ISL_531650 to 531659, EPI_ISL_531661 to 531667, EPI_ISL_531669 to 531671, EPI_ISL_531673 to 531676, EPI_ISL_531679 to 531686, EPI_ISL_531692 to 531701, EPI_ISL_531704 to 531713, EPI_ISL_531715 to 531726, EPI_ISL_531728 to 531729, EPI_ISL_531731, EPI_ISL_531733 to 531742, EPI_ISL_531744 to 531752, EPI_ISL_531756 to 531757, EPI_ISL_531759 to 531767, EPI_ISL_531769 to 531778, EPI_ISL_531780 to 531787, EPI_ISL_531789 to 531791, EPI_ISL_531793 to 531800, EPI_ISL_531803 to 531804, EPI_ISL_531806 to 531813, EPI_ISL_531816 to 531820, EPI_ISL_531822 to 531823, EPI_ISL_531825 to 531831, EPI_ISL_531833 to 531841, EPI_ISL_531843 to 531850, EPI_ISL_531852 to 531855, EPI_ISL_531859 to 531861, EPI_ISL_531864, EPI_ISL_531866 to 531871, EPI_ISL_531874 to 531876, EPI_ISL_531878 to 531879, EPI_ISL_531882 to 531883, EPI_ISL_531885 to 531886, EPI_ISL_531888, EPI_ISL_531891, EPI_ISL_531893, EPI_ISL_531895 to 531904, EPI_ISL_531906 to 531911, EPI_ISL_531914 to 531917, EPI_ISL_531920 to 531924, EPI_ISL_531926 to 531928, EPI_ISL_531930 to 531931, EPI_ISL_531934, EPI_ISL_531937 to 531942, EPI_ISL_531944, EPI_ISL_531946 to 531950, EPI_ISL_531952 to 531953, EPI_ISL_531955 to 531962, EPI_ISL_531964 to 531972, EPI_ISL_531974 to 531977, EPI_ISL_531980 to 531981, EPI_ISL_531983, EPI_ISL_531985 to 531986, EPI_ISL_531988 to 531989, EPI_ISL_531991 to 531994, EPI_ISL_531996 to 532002, EPI_ISL_532006, EPI_ISL_532008 to 532011, EPI_ISL_532013 to 532017, EPI_ISL_532019 to 532023, EPI_ISL_532025 to 532031, EPI_ISL_532033 to 532037, EPI_ISL_532039 to 532048, EPI_ISL_532050 to 532052, EPI_ISL_532054, EPI_ISL_532056, EPI_ISL_532058 to 532061, EPI_ISL_532063 to 532069, EPI_ISL_532072 to 532075, EPI_ISL_532079, EPI_ISL_532082 to 532084, EPI_ISL_532086 to 532092, EPI_ISL_532095 to 532097, EPI_ISL_532099 to 532101, EPI_ISL_532103 to 532104, EPI_ISL_532106 to 532117, EPI_ISL_532119 to 532122, EPI_ISL_532124, EPI_ISL_532126 to 532128, EPI_ISL_532132 to 532137, EPI_ISL_532139 to 532152, EPI_ISL_532154 to 532167, EPI_ISL_532169 to 532177, EPI_ISL_532179 to 532185, EPI_ISL_532187 to 532191, EPI_ISL_532193, EPI_ISL_532198 to 532199, EPI_ISL_532201, EPI_ISL_532204 to 532209, EPI_ISL_532211 to 532213, EPI_ISL_532215 to 532218, EPI_ISL_532220, EPI_ISL_532222 to 532234, EPI_ISL_532236 to 532240, EPI_ISL_532242 to 532243, EPI_ISL_532245 to 532248, EPI_ISL_532250 to 532257, EPI_ISL_532259 to 532262, EPI_ISL_532264 to 532270, EPI_ISL_532273 to 532274, EPI_ISL_532277, EPI_ISL_532279 to 532282, EPI_ISL_532284 to 532287, EPI_ISL_532289 to 532292, EPI_ISL_532295 to 532300, EPI_ISL_532302 to 532310, EPI_ISL_532312 to 532315, EPI_ISL_532318 to 532320, EPI_ISL_532322 to 532326, EPI_ISL_532329 to 532333, EPI_ISL_532336 to 532337, EPI_ISL_532339, EPI_ISL_532341, EPI_ISL_532343 to 532346, EPI_ISL_532348, EPI_ISL_532350, EPI_ISL_532352, EPI_ISL_532354, EPI_ISL_532357 to 532362, EPI_ISL_532365 to 532378, EPI_ISL_532380 to 532383, EPI_ISL_532385 to 532387, EPI_ISL_532389, EPI_ISL_532391 to 532393, EPI_ISL_532395 to 532398, EPI_ISL_532400 to 532401, EPI_ISL_532403, EPI_ISL_532405 to 532413, EPI_ISL_532415 to 532429, EPI_ISL_532433 to 532437, EPI_ISL_532439, EPI_ISL_532441 to 532443, EPI_ISL_532445, EPI_ISL_532447 to 532451, EPI_ISL_532453, EPI_ISL_532455, EPI_ISL_532457 to 532462, EPI_ISL_532465 to 532468, EPI_ISL_532470 to 532471, EPI_ISL_532474 to 532479, EPI_ISL_532481 to 532483, EPI_ISL_532485 to 532489, EPI_ISL_532491, EPI_ISL_532494 to 532505, EPI_ISL_532507 to 532509, EPI_ISL_532513, EPI_ISL_532516 to 532517, EPI_ISL_532519 to 532522, EPI_ISL_532524 to 532530, EPI_ISL_532532 to 532537, EPI_ISL_532539, EPI_ISL_532541 to 532562, EPI_ISL_532565 to 532571, EPI_ISL_532573 to 532581, EPI_ISL_532584 to 532586, EPI_ISL_532588 to 532593, EPI_ISL_532595 to 532597, EPI_ISL_532599 to 532619, EPI_ISL_532622 to 532624, EPI_ISL_532627 to 532632, EPI_ISL_532634 to 532638, EPI_ISL_532640 to 532647, EPI_ISL_532649 to 532658, EPI_ISL_532660 to 532669, EPI_ISL_532671 to 532682, EPI_ISL_532684 to 532688, EPI_ISL_532690 to 532696, EPI_ISL_532698 to 532704, EPI_ISL_532706 to 532714, EPI_ISL_532716 to 532732, EPI_ISL_532734 to 532737, EPI_ISL_532739 to 532743, EPI_ISL_532745, EPI_ISL_532747 to 532749, EPI_ISL_532751 to 532752, EPI_ISL_532754, EPI_ISL_532756 to 532764, EPI_ISL_532766, EPI_ISL_532768 to 532783, EPI_ISL_532785, EPI_ISL_532787 to 532790, EPI_ISL_532792 to 532810, EPI_ISL_532812 to 532834, EPI_ISL_532836 to 532837, EPI_ISL_532839 to 532843, EPI_ISL_532845 to 532850, EPI_ISL_532852 to 532857, EPI_ISL_532859 to 532863, EPI_ISL_532866 to 532873, EPI_ISL_532877, EPI_ISL_532879 to 532884, EPI_ISL_532887 to 532890, EPI_ISL_532892 to 532893, EPI_ISL_532895, EPI_ISL_532897 to 532904, EPI_ISL_532907 to 532914, EPI_ISL_532916 to 532925, EPI_ISL_532927 to 532931, EPI_ISL_532934 to 532947, EPI_ISL_532949 to 532961, EPI_ISL_532963 to 532977, EPI_ISL_532979 to 532982, EPI_ISL_532984 to 532987, EPI_ISL_532989, EPI_ISL_532991, EPI_ISL_532993, EPI_ISL_532995 to 532999, EPI_ISL_533001 to 533015, EPI_ISL_533017 to 533026, EPI_ISL_533028 to 533071, EPI_ISL_533073 to 533085, EPI_ISL_533087 to 533227, EPI_ISL_533229, EPI_ISL_533233 to 533235, EPI_ISL_533238 to 533240, EPI_ISL_533242 to 533251, EPI_ISL_533253 to 533255, EPI_ISL_533257 to 533261, EPI_ISL_533263 to 533269, EPI_ISL_533272 to 533282, EPI_ISL_533284 to 533288, EPI_ISL_533290 to 533297, EPI_ISL_533299 to 533310, EPI_ISL_533312 to 533342, EPI_ISL_533344 to 533345, EPI_ISL_533347 to 533349, EPI_ISL_533352 to 533354, EPI_ISL_533356 to 533357, EPI_ISL_533359 to 533364, EPI_ISL_533366 to 533375, EPI_ISL_533377 to 533393, EPI_ISL_533395, EPI_ISL_533397 to 533402, EPI_ISL_533404 to 533405, EPI_ISL_533407 to 533418, EPI_ISL_533420, EPI_ISL_533422 to 533434, EPI_ISL_536819 to 536888, EPI_ISL_536890 to 537016, EPI_ISL_537018 to 537205, EPI_ISL_539897 to 539905, EPI_ISL_539907 to 540029, EPI_ISL_540031 to 540277, EPI_ISL_540279 to 540280, EPI_ISL_540282 to 540350, EPI_ISL_540352 to 540420, EPI_ISL_541784 to 541846, EPI_ISL_549547 to 549771, EPI_ISL_549773 to 549783, EPI_ISL_549785 to 549807, EPI_ISL_553732, EPI_ISL_566998 to 567023, EPI_ISL_567025 to 567058, EPI_ISL_567060 to 567183, EPI_ISL_567185 to 567201, EPI_ISL_567203 to 567230, EPI_ISL_567232 to 567532, EPI_ISL_568223 to 568288, EPI_ISL_568291 to 568300, EPI_ISL_568302 to 568314, EPI_ISL_568316, EPI_ISL_568318 to 568343, EPI_ISL_568345 to 568363, EPI_ISL_568366 to 568384, EPI_ISL_568388 to 568391, EPI_ISL_568393 to 568394, EPI_ISL_568396 to 568397, EPI_ISL_568399 to 568435, EPI_ISL_568437 to 568452, EPI_ISL_568455 to 568457, EPI_ISL_568459 to 568460, EPI_ISL_568462 to 568465, EPI_ISL_575338 to 575366, EPI_ISL_575368 to 575398, EPI_ISL_575995 to 576049, EPI_ISL_576051 to 576063, EPI_ISL_576065 to 576069, EPI_ISL_576071 to 576087, EPI_ISL_576089 to 576093, EPI_ISL_579759 to 579793, EPI_ISL_579795 to 579834, EPI_ISL_579836 to 579842, EPI_ISL_579844 to 579918, EPI_ISL_580858 to 580860, EPI_ISL_580862 to 580863, EPI_ISL_580865 to 580872, EPI_ISL_580874, EPI_ISL_580876, EPI_ISL_580878 to 580881, EPI_ISL_580883 to 580884, EPI_ISL_580886 to 580891, EPI_ISL_580893 to 580895, EPI_ISL_580899, EPI_ISL_580901, EPI_ISL_580905 to 580908, EPI_ISL_580911 to 580912, EPI_ISL_580914, EPI_ISL_580916, EPI_ISL_580919 to 580920, EPI_ISL_580922 to 580928, EPI_ISL_580930 to 580934, EPI_ISL_580936 to 580939, EPI_ISL_580941 to 580947, EPI_ISL_580949, EPI_ISL_580952 to 580958, EPI_ISL_580960, EPI_ISL_580962 to 580975, EPI_ISL_580977 to 580978, EPI_ISL_580981 to 580989, EPI_ISL_580992, EPI_ISL_580994, EPI_ISL_580997, EPI_ISL_580999, EPI_ISL_581001 to 581003, EPI_ISL_581005 to 581007, EPI_ISL_581010 to 581017, EPI_ISL_581019 to 581022, EPI_ISL_581024, EPI_ISL_581027, EPI_ISL_581030 to 581031, EPI_ISL_581033 to 581034, EPI_ISL_581036 to 581037, EPI_ISL_581039, EPI_ISL_581042 to 581044, EPI_ISL_581047 to 581053, EPI_ISL_581055, EPI_ISL_581057, EPI_ISL_581059 to 581063, EPI_ISL_581066, EPI_ISL_581068, EPI_ISL_581070 to 581073, EPI_ISL_581075, EPI_ISL_581077, EPI_ISL_581079 to 581083, EPI_ISL_581085, EPI_ISL_581087 to 581092, EPI_ISL_581094 to 581096, EPI_ISL_581098, EPI_ISL_581100 to 581102, EPI_ISL_581104 to 581105, EPI_ISL_581108 to 581110, EPI_ISL_581112, EPI_ISL_581127, EPI_ISL_581132 to 581133, EPI_ISL_581141, EPI_ISL_581146, EPI_ISL_581154, EPI_ISL_581166, EPI_ISL_581174, EPI_ISL_581182 to 581183, EPI_ISL_581189, EPI_ISL_581193 to 581194, EPI_ISL_581196, EPI_ISL_581214, EPI_ISL_581217, EPI_ISL_581223, EPI_ISL_581226, EPI_ISL_581229 to 581230, EPI_ISL_581245, EPI_ISL_581247, EPI_ISL_581261 to 581262, EPI_ISL_581267, EPI_ISL_581285, EPI_ISL_581291, EPI_ISL_581310, EPI_ISL_581323 to 581324, EPI_ISL_581329, EPI_ISL_581334, EPI_ISL_581337, EPI_ISL_581341, EPI_ISL_581344, EPI_ISL_581346, EPI_ISL_581350, EPI_ISL_581354, EPI_ISL_581358, EPI_ISL_581377, EPI_ISL_581381, EPI_ISL_581384, EPI_ISL_581387, EPI_ISL_581392, EPI_ISL_581395, EPI_ISL_581402 to 581403, EPI_ISL_581405 to 581408, EPI_ISL_581410, EPI_ISL_581418 to 581420, EPI_ISL_581437, EPI_ISL_581439, EPI_ISL_581441, EPI_ISL_581443, EPI_ISL_587304 to 587385, EPI_ISL_587387 to 587476, EPI_ISL_587478 to 587710, EPI_ISL_588060 to 588074, EPI_ISL_588076 to 588124, EPI_ISL_588126 to 588127, EPI_ISL_588129 to 588174, EPI_ISL_588176 to 588495, EPI_ISL_588497 to 588540, EPI_ISL_588542 to 588571, EPI_ISL_588573 to 588673, EPI_ISL_588675 to 588716, EPI_ISL_588718 to 588843, EPI_ISL_588846 to 588926, EPI_ISL_588928 to 588939, EPI_ISL_588941 to 588961, EPI_ISL_588963 to 589016, EPI_ISL_589020, EPI_ISL_589022 to 589023, EPI_ISL_589025, EPI_ISL_589027, EPI_ISL_589031 to 589033, EPI_ISL_589035 to 589038, EPI_ISL_589042, EPI_ISL_589044 to 589049, EPI_ISL_589052 to 589056, EPI_ISL_589058 to 589062, EPI_ISL_589064 to 589066, EPI_ISL_589068 to 589069, EPI_ISL_589071 to 589073, EPI_ISL_589076, EPI_ISL_589078, EPI_ISL_589082, EPI_ISL_589084 to 589085, EPI_ISL_589087 to 589089, EPI_ISL_589092 to 589094, EPI_ISL_589096, EPI_ISL_589100, EPI_ISL_589102, EPI_ISL_589105 to 589107, EPI_ISL_589112 to 589113, EPI_ISL_589116, EPI_ISL_589119 to 589120, EPI_ISL_589124 to 589126, EPI_ISL_589128 to 589129, EPI_ISL_589131 to 589132, EPI_ISL_589134, EPI_ISL_589136, EPI_ISL_589138 to 589146, EPI_ISL_589148 to 589152, EPI_ISL_589154 to 589156, EPI_ISL_589158 to 589159, EPI_ISL_589162 to 589165, EPI_ISL_589175, EPI_ISL_589177 to 589178, EPI_ISL_589180 to 589181, EPI_ISL_589186, EPI_ISL_589188 to 589194, EPI_ISL_589197, EPI_ISL_589201 to 589204, EPI_ISL_589206 to 589210, EPI_ISL_589213 to 589220, EPI_ISL_589222 to 589223, EPI_ISL_589228, EPI_ISL_589230, EPI_ISL_589232, EPI_ISL_589234, EPI_ISL_589236, EPI_ISL_589238 to 589241, EPI_ISL_589244 to 589246, EPI_ISL_589249 to 589250, EPI_ISL_589252, EPI_ISL_589254 to 589255, EPI_ISL_589257 to 589258, EPI_ISL_589263, EPI_ISL_589774 to 589777, EPI_ISL_589788, EPI_ISL_589790, EPI_ISL_589793, EPI_ISL_589803 to 589935, EPI_ISL_589937 to 590045, EPI_ISL_590047 to 590171, EPI_ISL_590176 to 590180, EPI_ISL_590182 to 590185, EPI_ISL_590187 to 590192, EPI_ISL_590195 to 590197, EPI_ISL_590199, EPI_ISL_590201 to 590205, EPI_ISL_590207 to 590209, EPI_ISL_590211 to 590212, EPI_ISL_590216 to 590219, EPI_ISL_590221 to 590223, EPI_ISL_590227, EPI_ISL_590229 to 590233, EPI_ISL_590236 to 590238, EPI_ISL_590240 to 590245, EPI_ISL_590247 to 590249, EPI_ISL_590251 to 590254, EPI_ISL_590256, EPI_ISL_590258, EPI_ISL_590262, EPI_ISL_590264, EPI_ISL_590266 to 590271, EPI_ISL_590273, EPI_ISL_590278 to 590281, EPI_ISL_590285, EPI_ISL_590287, EPI_ISL_590289 to 590294, EPI_ISL_590297 to 590299, EPI_ISL_590301 to 590305, EPI_ISL_590308, EPI_ISL_590310 to 590313, EPI_ISL_590315 to 590318, EPI_ISL_590320, EPI_ISL_590322 to 590326, EPI_ISL_590330 to 590334, EPI_ISL_590336, EPI_ISL_590338 to 590345, EPI_ISL_590348 to 590350, EPI_ISL_590353 to 590354, EPI_ISL_590356, EPI_ISL_590358, EPI_ISL_590360, EPI_ISL_590362 to 590363, EPI_ISL_590365, EPI_ISL_590368, EPI_ISL_590370 to 590372, EPI_ISL_590374 to 590378, EPI_ISL_590381 to 590382, EPI_ISL_590384 to 590385, EPI_ISL_590387, EPI_ISL_590389, EPI_ISL_590391, EPI_ISL_590393 to 590396, EPI_ISL_590400 to 590402, EPI_ISL_590404,

EPI_ISL_590406 to 590418, EPI_ISL_590420 to 590421, EPI_ISL_590424 to 590437, EPI_ISL_590439 to 590440, EPI_ISL_590443 to 590445, EPI_ISL_590447, EPI_ISL_590449 to 590451, EPI_ISL_590454 to 590461, EPI_ISL_590463 to 590496, EPI_ISL_590498 to 590688, EPI_ISL_597234 to 597236, EPI_ISL_597240, EPI_ISL_597242, EPI_ISL_597244 to 597245, EPI_ISL_597249, EPI_ISL_597252, EPI_ISL_597260 to 597261, EPI_ISL_597263, EPI_ISL_597267 to 597268, EPI_ISL_597272, EPI_ISL_597275 to 597278, EPI_ISL_597280 to 597281, EPI_ISL_597288, EPI_ISL_597290 to 597292, EPI_ISL_597296, EPI_ISL_597298 to 597299, EPI_ISL_597302 to 597303, EPI_ISL_597311 to 597315, EPI_ISL_597318, EPI_ISL_597322, EPI_ISL_597326, EPI_ISL_597328 to 597330, EPI_ISL_597332 to 597334, EPI_ISL_597336, EPI_ISL_597338 to 597339, EPI_ISL_597347 to 597348, EPI_ISL_597351 to 597353, EPI_ISL_597355 to 597357, EPI_ISL_597359 to 597360, EPI_ISL_597362 to 597364, EPI_ISL_597369 to 597370, EPI_ISL_597376, EPI_ISL_597379, EPI_ISL_597382 to 597387, EPI_ISL_597389, EPI_ISL_597391, EPI_ISL_597393 to 597395, EPI_ISL_597400, EPI_ISL_597404, EPI_ISL_597406 to 597411, EPI_ISL_597413, EPI_ISL_597415 to 597417, EPI_ISL_597419 to 597423, EPI_ISL_597429 to 597431, EPI_ISL_597433 to 597434, EPI_ISL_597436, EPI_ISL_597439 to 597440, EPI_ISL_597442 to 597444, EPI_ISL_597447 to 597448, EPI_ISL_597452, EPI_ISL_597454 to 597457, EPI_ISL_597463, EPI_ISL_597465, EPI_ISL_597471, EPI_ISL_597474 to 597475, EPI_ISL_597481 to 597482, EPI_ISL_597484 to 597486, EPI_ISL_597491 to 597493, EPI_ISL_597496, EPI_ISL_597498 to 597501, EPI_ISL_597503, EPI_ISL_597506, EPI_ISL_597509, EPI_ISL_597511, EPI_ISL_597513 to 597516, EPI_ISL_597518, EPI_ISL_597521 to 597522, EPI_ISL_598831 to 598969, EPI_ISL_598971 to 599009, EPI_ISL_599011 to 599076, EPI_ISL_599078 to 599165, EPI_ISL_599167 to 599182, EPI_ISL_599184 to 599193, EPI_ISL_599195 to 599200, EPI_ISL_599202 to 599269, EPI_ISL_599271 to 599313, EPI_ISL_599315 to 599589, EPI_ISL_599591 to 599626, EPI_ISL_599628 to 599815, EPI_ISL_599817, EPI_ISL_600128 to 600138, EPI_ISL_600140 to 600220, EPI_ISL_600222 to 600427, EPI_ISL_600447 to 600448, EPI_ISL_600450, EPI_ISL_600452 to 600453, EPI_ISL_600455, EPI_ISL_600457 to 600458, EPI_ISL_600460, EPI_ISL_600462, EPI_ISL_600464 to 600465, EPI_ISL_600467, EPI_ISL_600469 to 600470, EPI_ISL_600472, EPI_ISL_600474 to 600475, EPI_ISL_600477, EPI_ISL_600479, EPI_ISL_600481 to 600483, EPI_ISL_600485 to 600486, EPI_ISL_600488, EPI_ISL_600490 to 600491, EPI_ISL_600493 to 600494, EPI_ISL_600496 to 600497, EPI_ISL_600499, EPI_ISL_600501, EPI_ISL_600503 to 600504, EPI_ISL_600506, EPI_ISL_600508 to 600509, EPI_ISL_600511, EPI_ISL_600513 to 600514, EPI_ISL_600516, EPI_ISL_600518, EPI_ISL_600520, EPI_ISL_600539 to 600540, EPI_ISL_600542, EPI_ISL_600544 to 600545, EPI_ISL_600547 to 600548, EPI_ISL_600550 to 600551, EPI_ISL_600553, EPI_ISL_600555 to 600556, EPI_ISL_600558, EPI_ISL_600560 to 600561, EPI_ISL_600563, EPI_ISL_600565 to 600566, EPI_ISL_600568 to 600569, EPI_ISL_600571 to 600642, EPI_ISL_600644 to 600823, EPI_ISL_600825 to 600891, EPI_ISL_600893 to 600900, EPI_ISL_600902 to 600903, EPI_ISL_600905 to 600911, EPI_ISL_600914, EPI_ISL_600916 to 600922, EPI_ISL_600924, EPI_ISL_600932 to 601021, EPI_ISL_601023 to 601149, EPI_ISL_601151 to 601191, EPI_ISL_601193 to 601213, EPI_ISL_601215 to 601223, EPI_ISL_601225 to 601233, EPI_ISL_601236 to 601238, EPI_ISL_601241, EPI_ISL_601244 to 601246, EPI_ISL_601250 to 601255, EPI_ISL_601257, EPI_ISL_601259 to 601261, EPI_ISL_601265 to 601266, EPI_ISL_601269, EPI_ISL_601271, EPI_ISL_601274, EPI_ISL_601276, EPI_ISL_601279, EPI_ISL_601284 to 601286, EPI_ISL_601288, EPI_ISL_601295 to 601296, EPI_ISL_601300, EPI_ISL_601308 to 601309, EPI_ISL_601315, EPI_ISL_601318 to 601319, EPI_ISL_601323, EPI_ISL_601328, EPI_ISL_601331 to 601336, EPI_ISL_601339, EPI_ISL_601341 to 601342, EPI_ISL_601344, EPI_ISL_601347 to 601348, EPI_ISL_601351 to 601353, EPI_ISL_601355 to 601358, EPI_ISL_601361 to 601362, EPI_ISL_601364, EPI_ISL_601372, EPI_ISL_601374, EPI_ISL_601377, EPI_ISL_601380, EPI_ISL_601384, EPI_ISL_601386 to 601387, EPI_ISL_601389, EPI_ISL_601391, EPI_ISL_601393, EPI_ISL_601400 to 601403, EPI_ISL_601405, EPI_ISL_601407 to 601408, EPI_ISL_601411, EPI_ISL_601413 to 601414, EPI_ISL_601416, EPI_ISL_601421, EPI_ISL_601423 to 601426, EPI_ISL_601429 to 601430, EPI_ISL_601432 to 601433, EPI_ISL_601435 to 601436, EPI_ISL_601445 to 601446, EPI_ISL_601453 to 601454, EPI_ISL_601459 to 601462, EPI_ISL_601466 to 601467, EPI_ISL_601469, EPI_ISL_601471, EPI_ISL_601477, EPI_ISL_601479, EPI_ISL_601481 to 601483, EPI_ISL_601486, EPI_ISL_601488 to 601489, EPI_ISL_601787 to 601796, EPI_ISL_601798 to 601833, EPI_ISL_601835 to 601949, EPI_ISL_601951 to 602005, EPI_ISL_602007 to 602014, EPI_ISL_602016 to 602026, EPI_ISL_602030 to 602076, EPI_ISL_602078 to 602082, EPI_ISL_602084 to 602089, EPI_ISL_602092 to 602101, EPI_ISL_602103 to 602105, EPI_ISL_602107 to 602108, EPI_ISL_602110 to 602121, EPI_ISL_602123 to 602144, EPI_ISL_602156 to 602157, EPI_ISL_609178 to 609179, EPI_ISL_609182, EPI_ISL_609184 to 609186, EPI_ISL_609189 to 609191, EPI_ISL_609194, EPI_ISL_609197 to 609199, EPI_ISL_609202, EPI_ISL_609206, EPI_ISL_611287 to 611303, EPI_ISL_611305 to 611377, EPI_ISL_611379 to 611484			
see above	Lighthouse Lab in Glasgow	Wellcome Sanger Institute for the COVID-19 Genomics UK (COG-UK) consortium	Anna Dominiczak and Alex Alderton; Carol Clugston; Cordelia Langford; David Gray; David K. Jackson; Dominic Kwiatkowski; Ewan Harrison; Harper VanSteenhouse; Ian Johnston; John Sillitoe; John Sillitoe on behalf of the Wellcome Sanger Institute COVID-19 Surveillance Team; John Sillitoe on behalf of the Wellcome Sanger Institute COVID-19 Surveillance Team (http://www.sanger.ac.uk/covid-team); Roberto Amato; Sonia Goncalves; Yumi Kasai
EPI_ISL_540823 to 540870, EPI_ISL_559947 to 559970, EPI_ISL_573769, EPI_ISL_585297 to 585421	Lighthouse Lab in Glasgow / MRC-University of Glasgow Centre for Virus Research	COVID-19 Genomics UK (COG-UK) Consortium	Alasdair MacLean; Ana da Silva Filipe; Anna Dominiczak; Antonia Ho; Carol Clugston; Daniel Mair; David Gray; David L Robertson; Elihu Aranday-Cortes; Emma Thomson; Harper VanSteenhouse; James Shepherd; Jenna Nichols; Joseph Hughes; Kathy Li; Kathy Smollett; Kyriaki Nomikou; Lily Tong; Marc Niebel; Matthew Holden; Natasha Jesudason; Natasha Johnson; Patawee Asamaphan; Rajiv Shah; Richard Orton; Rory Gunson; Sarah McDonald; Sharif Shaaban; Sreenu Vattipally; Stephen Carmichael; Yumi Kasai
EPI_ISL_549829, EPI_ISL_549861, EPI_ISL_550069, EPI_ISL_550485, EPI_ISL_550849, EPI_ISL_550983, EPI_ISL_551001, EPI_ISL_551007, EPI_ISL_551013, EPI_ISL_551092, EPI_ISL_551218, EPI_ISL_552106, EPI_ISL_552157, EPI_ISL_552240, EPI_ISL_552306, EPI_ISL_552458, EPI_ISL_552880, EPI_ISL_553084, EPI_ISL_554195, EPI_ISL_554213, EPI_ISL_554273, EPI_ISL_554281, EPI_ISL_554662, EPI_ISL_554798, EPI_ISL_556207, EPI_ISL_557186, EPI_ISL_557189, EPI_ISL_557213, EPI_ISL_557238, EPI_ISL_557355, EPI_ISL_557379, EPI_ISL_557504, EPI_ISL_557840, EPI_ISL_557843, EPI_ISL_557885, EPI_ISL_557952, EPI_ISL_558237, EPI_ISL_558261, EPI_ISL_558283, EPI_ISL_558328, EPI_ISL_558921, EPI_ISL_559017, EPI_ISL_559264, EPI_ISL_559287, EPI_ISL_559670, EPI_ISL_559706, EPI_ISL_559724, EPI_ISL_559733 to 559734, EPI_ISL_559740, EPI_ISL_566951, EPI_ISL_566986, EPI_ISL_580840, EPI_ISL_581149, EPI_ISL_581366, EPI_ISL_589050, EPI_ISL_589130, EPI_ISL_589184, EPI_ISL_589262, EPI_ISL_589334, EPI_ISL_589567, EPI_ISL_598249, EPI_ISL_598305, EPI_ISL_598330 to 598331, EPI_ISL_598361, EPI_ISL_598408, EPI_ISL_598433, EPI_ISL_598436, EPI_ISL_598606, EPI_ISL_598638, EPI_ISL_598667, EPI_ISL_598821, EPI_ISL_599845, EPI_ISL_599888, EPI_ISL_599948, EPI_ISL_599960, EPI_ISL_600056, EPI_ISL_601290, EPI_ISL_601399, EPI_ISL_601652, EPI_ISL_601667, EPI_ISL_606311, EPI_ISL_606314, EPI_ISL_606353, EPI_ISL_606402, EPI_ISL_606415, EPI_ISL_606462, EPI_ISL_606516, EPI_ISL_606524, EPI_ISL_606588, EPI_ISL_606608, EPI_ISL_606654, EPI_ISL_606797, EPI_ISL_607448, EPI_ISL_607461, EPI_ISL_607516, EPI_ISL_607550, EPI_ISL_607572, EPI_ISL_607579, EPI_ISL_607587, EPI_ISL_608748, EPI_ISL_608834, EPI_ISL_609230, EPI_ISL_609267, EPI_ISL_609302, EPI_ISL_1408910, EPI_ISL_1408912, EPI_ISL_1408914, EPI_ISL_1408916 to 1408917, EPI_ISL_1408919 to 1408920, EPI_ISL_1408923 to 1408927, EPI_ISL_1408930 to 1408931, EPI_ISL_1408933, EPI_ISL_1408935 to 1408936, EPI_ISL_1408938, EPI_ISL_1408942 to 1408943, EPI_ISL_1408949, EPI_ISL_1408951 to 1408952, EPI_ISL_1408955 to 1408957, EPI_ISL_1408960, EPI_ISL_1408964, EPI_ISL_1408967, EPI_ISL_1408970 to 1408971, EPI_ISL_1408975 to 1408977, EPI_ISL_1408979, EPI_ISL_1408984 to 1408985, EPI_ISL_1408987, EPI_ISL_1408989, EPI_ISL_1408991, EPI_ISL_1408993 to 1408995, EPI_ISL_1408998 to 1408999, EPI_ISL_1409001, EPI_ISL_1409003, EPI_ISL_1409007, EPI_ISL_1409019, EPI_ISL_1409022 to 1409023, EPI_ISL_1409025 to 1409026, EPI_ISL_1409028, EPI_ISL_1409030, EPI_ISL_1409033, EPI_ISL_1409035 to 1409036, EPI_ISL_1409041 to 1409042, EPI_ISL_1409045 to 1409046, EPI_ISL_1409052 to 1409054, EPI_ISL_1409056 to 1409062, EPI_ISL_1409064 to 1409065, EPI_ISL_1409067, EPI_ISL_1409070, EPI_ISL_1409072, EPI_ISL_1409077, EPI_ISL_1409079, EPI_ISL_1409083 to 1409084, EPI_ISL_1409086, EPI_ISL_1409089, EPI_ISL_1409093 to 1409094, EPI_ISL_1409098, EPI_ISL_1409101 to 1409102, EPI_ISL_1409105 to 1409108, EPI_ISL_1409111 to 1409112, EPI_ISL_1409119 to 1409122, EPI_ISL_1409125 to 1409128, EPI_ISL_1409132 to 1409135, EPI_ISL_1409139 to 1409141, EPI_ISL_1409147, EPI_ISL_1409149 to 1409150, EPI_ISL_1409152 to 1409154, EPI_ISL_1409158, EPI_ISL_1409162, EPI_ISL_1409166 to 1409169, EPI_ISL_1409171, EPI_ISL_1409175 to 1409177, EPI_ISL_1409179, EPI_ISL_1409183, EPI_ISL_1409186, EPI_ISL_1409190 to 1409195, EPI_ISL_1409202 to 1409203, EPI_ISL_1409206 to 1409207, EPI_ISL_1409215, EPI_ISL_1409219, EPI_ISL_1409222, EPI_ISL_1409227, EPI_ISL_1409245 to 1409246, EPI_ISL_1409248, EPI_ISL_1409251, EPI_ISL_1409253 to 1409254, EPI_ISL_1409260, EPI_ISL_1409262, EPI_ISL_1409265, EPI_ISL_1409267 to 1409269, EPI_ISL_1409272, EPI_ISL_1409275, EPI_ISL_1409279, EPI_ISL_1409282, EPI_ISL_1409287, EPI_ISL_1409289 to 1409291, EPI_ISL_1409295 to 1409296, EPI_ISL_1409298, EPI_ISL_1409301 to 1409303, EPI_ISL_1409305, EPI_ISL_1409310, EPI_ISL_1409313, EPI_ISL_1409315, EPI_ISL_1409322, EPI_ISL_1409325 to 1409326, EPI_ISL_1409335, EPI_ISL_1409350, EPI_ISL_1409360, EPI_ISL_1409368 to 1409369, EPI_ISL_1409372, EPI_ISL_1409375, EPI_ISL_1409383, EPI_ISL_1409391, EPI_ISL_1409395, EPI_ISL_1409409 to 1409410, EPI_ISL_1409413, EPI_ISL_1409422 to 1409423, EPI_ISL_1409425, EPI_ISL_1409427, EPI_ISL_1409439, EPI_ISL_1409442, EPI_ISL_1409446 to 1409447, EPI_ISL_1409453 to 1409454, EPI_ISL_1409460, EPI_ISL_1409478 to 1409479, EPI_ISL_1409481 to 1409482, EPI_ISL_1409491 to 1409493, EPI_ISL_1411454, EPI_ISL_1411471 to 1411472, EPI_ISL_1411481, EPI_ISL_1411488, EPI_ISL_1411491, EPI_ISL_1411504, EPI_ISL_1411506, EPI_ISL_1411560 to 1411561, EPI_ISL_1411565, EPI_ISL_1411570, EPI_ISL_1411584, EPI_ISL_1411597, EPI_ISL_1411599, EPI_ISL_1411602, EPI_ISL_1411610, EPI_ISL_1411612, EPI_ISL_1411623, EPI_ISL_1411627, EPI_ISL_1411632, EPI_ISL_1411645, EPI_ISL_1411648, EPI_ISL_1411650, EPI_ISL_1411653 to 1411654, EPI_ISL_1411659 to 1411660, EPI_ISL_1411662, EPI_ISL_1411667, EPI_ISL_1411669 to 1411670, EPI_ISL_1411695, EPI_ISL_1411700, EPI_ISL_1411703, EPI_ISL_1411717, EPI_ISL_1411725, EPI_ISL_1411732, EPI_ISL_1411737, EPI_ISL_1411746, EPI_ISL_1411754 to 1411755, EPI_ISL_1411757, EPI_ISL_1411759, EPI_ISL_1411762, EPI_ISL_1411764, EPI_ISL_1411769, EPI_ISL_1411771, EPI_ISL_1411777, EPI_ISL_1411790, EPI_ISL_1411793, EPI_ISL_1411800 to 1411801, EPI_ISL_1411805, EPI_ISL_1411818, EPI_ISL_1411821, EPI_ISL_1411825, EPI_ISL_1411832, EPI_ISL_1411835, EPI_ISL_1411844, EPI_ISL_1415822 to 1415824, EPI_ISL_1415828, EPI_ISL_1415835, EPI_ISL_1415838 to 1415840, EPI_ISL_1415843 to 1415844, EPI_ISL_1415846, EPI_ISL_1415848 to 1415850, EPI_ISL_1415857 to 1415859, EPI_ISL_1415861, EPI_ISL_1415866 to 1415869, EPI_ISL_1415871 to 1415873, EPI_ISL_1415887, EPI_ISL_1415889, EPI_ISL_1415896 to 1415897, EPI_ISL_1415899 to 1415900, EPI_ISL_1415902 to 1415903, EPI_ISL_1415906, EPI_ISL_1415909, EPI_ISL_1415912 to 1415913, EPI_ISL_1415919 to 1415920, EPI_ISL_1415922 to 1415923, EPI_ISL_1415927, EPI_ISL_1415933, EPI_ISL_1415936, EPI_ISL_1415939, EPI_ISL_1415944, EPI_ISL_1415950 to 1415953, EPI_ISL_1415955 to 1415956, EPI_ISL_1415959, EPI_ISL_1415961, EPI_ISL_1415965, EPI_ISL_1415967, EPI_ISL_1415971, EPI_ISL_1415973 to 1415977, EPI_ISL_1415980 to 1415983, EPI_ISL_1415989 to 1415990, EPI_ISL_1415992, EPI_ISL_1415994, EPI_ISL_1415996, EPI_ISL_1415998, EPI_ISL_1416000, EPI_ISL_1416005 to 1416006, EPI_ISL_1416009 to 1416010, EPI_ISL_1416017, EPI_ISL_1416020, EPI_ISL_1416022 to 1416023, EPI_ISL_1416026 to 1416028, EPI_ISL_1416031, EPI_ISL_1416037 to 1416038, EPI_ISL_1416040, EPI_ISL_1416048 to 1416049, EPI_ISL_1416051, EPI_ISL_1416054, EPI_ISL_1416059 to 1416062, EPI_ISL_1416064 to 1416065, EPI_ISL_1416067 to 1416070, EPI_ISL_1416079, EPI_ISL_1416085, EPI_ISL_1416092 to 1416093, EPI_ISL_1416097, EPI_ISL_1416099 to 1416101, EPI_ISL_1416104 to 1416105, EPI_ISL_1416113 to 1416114, EPI_ISL_1416116, EPI_ISL_1416120 to 1416121, EPI_ISL_1448788, EPI_ISL_1448791, EPI_ISL_1448797 to 1448799, EPI_ISL_1448801, EPI_ISL_1448804, EPI_ISL_1448807, EPI_ISL_1448809, EPI_ISL_1448818, EPI_ISL_1448829, EPI_ISL_1448838, EPI_ISL_1448840, EPI_ISL_1448843, EPI_ISL_1448847, EPI_ISL_1448854, EPI_ISL_1448857, EPI_ISL_1448859 to 1448860, EPI_ISL_1448863, EPI_ISL_1448868, EPI_ISL_1448874, EPI_ISL_1448876, EPI_ISL_1448896, EPI_ISL_1448899, EPI_ISL_1448905, EPI_ISL_1448908 to 1448909, EPI_ISL_1448917, EPI_ISL_1448919 to 1448920, EPI_ISL_1448927, EPI_ISL_1448933, EPI_ISL_1448939, EPI_ISL_1448947 to 1448949, EPI_ISL_1448951 to 1448952, EPI_ISL_1448954, EPI_ISL_1448965, EPI_ISL_1448968, EPI_ISL_1448970 to 1448972, EPI_ISL_1448976 to 1448977, EPI_ISL_1448979, EPI_ISL_1448984, EPI_ISL_1448989 to 1448990, EPI_ISL_1448993 to 1448994, EPI_ISL_1449005, EPI_ISL_1449015, EPI_ISL_1449017 to 1449019, EPI_ISL_1449021, EPI_ISL_1449023 to 1449026, EPI_ISL_1449351, EPI_ISL_1449362, EPI_ISL_1449364 to 1449365, EPI_ISL_1449374, EPI_ISL_1449376, EPI_ISL_1449381, EPI_ISL_1449383, EPI_ISL_1449385, EPI_ISL_1449387, EPI_ISL_1449391, EPI_ISL_1449403, EPI_ISL_1449407, EPI_ISL_1449412, EPI_ISL_1449427 to 1449428, EPI_ISL_1449431, EPI_ISL_1449436, EPI_ISL_1449439 to 1449440, EPI_ISL_1449449 to 1449450, EPI_ISL_1449452, EPI_ISL_1449454, EPI_ISL_1449457, EPI_ISL_1449459, EPI_ISL_1449476 to 1449477, EPI_ISL_1449481, EPI_ISL_1449485, EPI_ISL_1449489, EPI_ISL_1449491, EPI_ISL_1449494, EPI_ISL_1449506, EPI_ISL_1449508, EPI_ISL_1449511, EPI_ISL_1449515, EPI_ISL_1449521 to 1449523, EPI_ISL_1449530, EPI_ISL_1449532, EPI_ISL_1449536, EPI_ISL_1449538, EPI_ISL_1449540, EPI_ISL_1449543 to 1449545, EPI_ISL_1449547, EPI_ISL_1449557, EPI_ISL_1449559, EPI_ISL_1449566 to 1449567, EPI_ISL_1449570, EPI_ISL_1449573, EPI_ISL_1449590, EPI_ISL_1449605, EPI_ISL_1449618, EPI_ISL_1449620 to 1449621, EPI_ISL_1449627, EPI_ISL_1449630, EPI_ISL_1449634, EPI_ISL_1449637, EPI_ISL_1449640 to 1449641, EPI_ISL_1449643, EPI_ISL_1449647, EPI_ISL_1449652 to 1449653, EPI_ISL_1449668, EPI_ISL_1449672 to 1449674, EPI_ISL_1449681, EPI_ISL_1449715, EPI_ISL_1449718 to 1449719, EPI_ISL_1449738, EPI_ISL_1449740, EPI_ISL_1449746, EPI_ISL_1449757, EPI_ISL_1449766, EPI_ISL_1449781, EPI_ISL_1449805, EPI_ISL_1449824, EPI_ISL_1449844, EPI_ISL_1449846, EPI_ISL_1449849, EPI_ISL_1449862, EPI_ISL_1449866, EPI_ISL_1449880, EPI_ISL_1449883, EPI_ISL_1449886, EPI_ISL_1449889, EPI_ISL_1449901, EPI_ISL_1449906, EPI_ISL_1449909, EPI_ISL_1449937, EPI_ISL_1449941 to 1449942, EPI_ISL_1449951, EPI_ISL_1449956, EPI_ISL_1449962, EPI_ISL_1449968, EPI_ISL_1449970, EPI_ISL_1449973 to 1449975, EPI_ISL_1449981 to 1449982, EPI_ISL_1449986, EPI_ISL_1449989, EPI_ISL_1449991, EPI_ISL_1449993, EPI_ISL_1449997, EPI_ISL_1449999, EPI_ISL_1450001, EPI_ISL_1450004, EPI_ISL_1450006, EPI_ISL_1450008, EPI_ISL_1450015 to 1450016, EPI_ISL_1450018 to 1450020, EPI_ISL_1450022, EPI_ISL_1450024 to 1450026, EPI_ISL_1450028 to 1450029, EPI_ISL_1450031, EPI_ISL_1450033 to 1450034, EPI_ISL_1450042, EPI_ISL_1450044 to 1450045, EPI_ISL_1450047, EPI_ISL_1450051, EPI_ISL_1450053 to 1450056, EPI_ISL_1450058 to 1450061, EPI_ISL_1450063, EPI_ISL_1450065 to 1450066, EPI_ISL_1450071, EPI_ISL_1450073, EPI_ISL_1450075, EPI_ISL_1450080, EPI_ISL_1450083, EPI_ISL_1450085 to 1450088, EPI_ISL_1450095, EPI_ISL_1450099 to 1450101, EPI_ISL_1450105 to 1450106, EPI_ISL_1450109 to 1450110, EPI_ISL_1450113, EPI_ISL_1450115, EPI_ISL_1450122, EPI_ISL_1450124 to 1450125, EPI_ISL_1450128, EPI_ISL_1450136, EPI_ISL_1450138 to 1450140, EPI_ISL_1450144 to 1450145, EPI_ISL_1450151, EPI_ISL_1450153 to 1450154, EPI_ISL_1450157 to 1450163, EPI_ISL_1450166, EPI_ISL_1450168, EPI_ISL_1450171, EPI_ISL_1450173 to 1450176, EPI_ISL_1450179 to 1450180, EPI_ISL_1450184 to 1450186, EPI_ISL_1450190 to 1450192, EPI_ISL_1450194 to 1450196, EPI_ISL_1450199, EPI_ISL_1450202, EPI_ISL_1450207, EPI_ISL_1450210 to 1450211, EPI_ISL_1450214 to 1450215, EPI_ISL_1450220, EPI_ISL_1450223, EPI_ISL_1450225, EPI_ISL_1450227, EPI_ISL_1450232 to 1450236, EPI_ISL_1450242, EPI_ISL_1450245 to 1450246, EPI_ISL_1450251 to 1450252, EPI_ISL_1450254 to 1450255, EPI_ISL_1450259 to 1450267, EPI_ISL_1450269, EPI_ISL_1450273 to 1450275, EPI_ISL_1450284 to 1450285, EPI_ISL_1450288 to 1450292, EPI_ISL_1450295 to 1450299, EPI_ISL_1450302, EPI_ISL_1450306, EPI_ISL_1450312 to 1450313, EPI_ISL_1450316 to 1450317, EPI_ISL_1450325, EPI_ISL_1450329, EPI_ISL_1450335, EPI_ISL_1450339, EPI_ISL_1450341, EPI_ISL_1450347, EPI_ISL_1450351, EPI_ISL_1450355, EPI_ISL_1450357, EPI_ISL_1450359 to 1450360, EPI_ISL_1450364 to 1450365, EPI_ISL_1450369, EPI_ISL_1450375, EPI_ISL_1450383, EPI_ISL_1450396, EPI_ISL_1450400, EPI_ISL_1450407, EPI_ISL_1450413, EPI_ISL_1450423, EPI_ISL_1450425 to 1450427, EPI_ISL_1450437, EPI_ISL_1450439, EPI_ISL_1450443, EPI_ISL_1450447 to 1450448, EPI_ISL_1450452, EPI_ISL_1450458, EPI_ISL_1450460, EPI_ISL_1450468, EPI_ISL_1450478, EPI_ISL_1450493 to 1450494, EPI_ISL_1450496 to 1450498, EPI_ISL_1450502, EPI_ISL_1450504, EPI_ISL_1450511 to 1450512, EPI_ISL_1450515 to 1450517, EPI_ISL_1450520, EPI_ISL_1450530, EPI_ISL_1450532, EPI_ISL_1450539 to 1450541, EPI_ISL_1450543, EPI_ISL_1450550, EPI_ISL_1450561, EPI_ISL_1450563 to 1450564, EPI_ISL_1450571 to 1450573, EPI_ISL_1450587, EPI_ISL_1450594, EPI_ISL_1450890 to 1450894, EPI_ISL_1450900, EPI_ISL_1450902 to 1450903, EPI_ISL_1450905, EPI_ISL_1450907 to 1450909, EPI_ISL_1450911 to 1450914, EPI_ISL_1450916 to 1450917, EPI_ISL_1450921, EPI_ISL_1450923 to 1450926, EPI_ISL_1450928 to 1450929, EPI_ISL_1450931, EPI_ISL_1450933, EPI_ISL_1450938 to 1450940, EPI_ISL_1450942 to 1450944, EPI_ISL_1450946, EPI_ISL_1450948, EPI_ISL_1450951 to 1450953, EPI_ISL_1450955, EPI_ISL_1450957, EPI_ISL_1450959 to 1450964, EPI_ISL_1450966, EPI_ISL_1450968, EPI_ISL_1450971 to 1450972, EPI_ISL_1450975 to 1450977, EPI_ISL_1450979 to 1450980, EPI_ISL_1450983 to 1450988, EPI_ISL_1450990, EPI_ISL_1450992, EPI_ISL_1450994 to 1450996, EPI_ISL_1450998 to 1451000, EPI_ISL_1451002 to 1451008, EPI_ISL_1451013, EPI_ISL_1451017 to 1451023, EPI_ISL_1451026 to 1451027, EPI_ISL_1451031 to 1451033, EPI_ISL_1451036 to 1451038, EPI_ISL_1451040 to 1451041, EPI_ISL_1451046 to 1451047, EPI_ISL_1451049 to 1451051, EPI_ISL_1451053 to 1451055, EPI_ISL_1451057, EPI_ISL_1451060 to 1451063, EPI_ISL_1451065 to 1451066, EPI_ISL_1451069 to 1451070, EPI_ISL_1451073 to 1451075, EPI_ISL_1451077 to 1451080, EPI_ISL_1451082 to 1451088, EPI_ISL_1451091 to 1451094, EPI_ISL_1451096, EPI_ISL_1451098 to 1451101, EPI_ISL_1451104, EPI_ISL_1451108 to 1451113, EPI_ISL_1451115, EPI_ISL_1451121, EPI_ISL_1451123 to 1451125, EPI_ISL_1451127 to 1451128, EPI_ISL_1451132 to 1451146, EPI_ISL_1451148, EPI_ISL_1451150, EPI_ISL_1451480, EPI_ISL_1451495, EPI_ISL_1451525, EPI_ISL_1451558 to 1451559, EPI_ISL_1451586, EPI_ISL_1451637, EPI_ISL_1451649, EPI_ISL_1451675, EPI_ISL_1451681, EPI_ISL_1451718, EPI_ISL_1451726, EPI_ISL_1451765, EPI_ISL_1451770, EPI_ISL_1451772, EPI_ISL_1451777, EPI_ISL_1452075, EPI_ISL_1452079, EPI_ISL_1452081, EPI_ISL_1452084 to 1452085, EPI_ISL_1452087, EPI_ISL_1452096 to 1452097, EPI_ISL_1452099 to 1452100, EPI_ISL_1452102 to 1452103, EPI_ISL_1452106, EPI_ISL_1452111, EPI_ISL_1452116, EPI_ISL_1452118, EPI_ISL_1452120, EPI_ISL_1452125, EPI_ISL_1452130, EPI_ISL_1452133 to 1452134, EPI_ISL_1452137, EPI_ISL_1452140, EPI_ISL_1452145, EPI_ISL_1452149 to 1452150, EPI_ISL_1452154 to 1452155, EPI_ISL_1452157, EPI_ISL_1452159, EPI_ISL_1452161, EPI_ISL_1452164, EPI_ISL_1452166, EPI_ISL_1452168, EPI_ISL_1452173, EPI_ISL_1452175 to 1452176, EPI_ISL_1452180, EPI_ISL_1452182, EPI_ISL_1452184 to 1452186, EPI_ISL_1452188, EPI_ISL_1452192 to 1452193, EPI_ISL_1452195 to 1452197, EPI_ISL_1452199 to 1452200, EPI_ISL_1452202, EPI_ISL_1452206 to 1452207, EPI_ISL_1452213, EPI_ISL_1452218, EPI_ISL_1452222 to 1452226, EPI_ISL_1452231 to 1452232, EPI_ISL_1452236, EPI_ISL_1452250, EPI_ISL_1452254, EPI_ISL_1452259, EPI_ISL_1452262, EPI_ISL_1452266, EPI_ISL_1452269, EPI_ISL_1452272 to 1452273, EPI_ISL_1452277, EPI_ISL_1452279, EPI_ISL_1452281 to 1452282, EPI_ISL_1452285, EPI_ISL_1452288, EPI_ISL_1452293, EPI_ISL_1452297 to 1452298, EPI_ISL_1452304, EPI_ISL_1452311, EPI_ISL_1452313, EPI_ISL_1452321, EPI_ISL_1452334, EPI_ISL_1452338, EPI_ISL_1452340, EPI_ISL_1452342, EPI_ISL_1452347 to 1452350, EPI_ISL_1452356 to 1452358, EPI_ISL_1452361, EPI_ISL_1452369, EPI_ISL_1452375, EPI_ISL_1452383 to 1452384, EPI_ISL_1452392 to 1452393, EPI_ISL_1452396 to 1452397, EPI_ISL_1452399, EPI_ISL_1452401, EPI_ISL_1452405 to 1452406, EPI_ISL_1452408, EPI_ISL_1452410 to 1452411, EPI_ISL_1452415 to 1452416, EPI_ISL_1452431, EPI_ISL_1452433, EPI_ISL_1452436, EPI_ISL_1452441, EPI_ISL_1452443, EPI_ISL_1452445, EPI_ISL_1452447, EPI_ISL_1452449, EPI_ISL_1452454, EPI_ISL_1452457 to 1452458, EPI_ISL_1452465 to 1452469, EPI_ISL_1452471 to 1452472, EPI_ISL_1452475, EPI_ISL_1452479, EPI_ISL_1452482, EPI_ISL_1452484, EPI_ISL_1452486, EPI_ISL_1452488 to 1452489, EPI_ISL_1452491, EPI_ISL_1452493, EPI_ISL_1452499, EPI_ISL_1452501, EPI_ISL_1452504, EPI_ISL_1452509, EPI_ISL_1452513, EPI_ISL_1452516 to 1452517, EPI_ISL_1452521, EPI_ISL_1452524 to 1452525, EPI_ISL_1452537, EPI_ISL_1452540, EPI_ISL_1452542, EPI_ISL_1452544 to 1452545, EPI_ISL_1452547, EPI_ISL_1452551, EPI_ISL_1452553, EPI_ISL_1452555, EPI_ISL_1452566, EPI_ISL_1452570, EPI_ISL_1452572, EPI_ISL_1452576, EPI_ISL_1452578 to 1452581, EPI_ISL_1452591, EPI_ISL_1452594, EPI_ISL_1452596, EPI_ISL_1452601, EPI_ISL_1452604 to 1452605, EPI_ISL_1452607, EPI_ISL_1452610 to 1452611, EPI_ISL_1452617, EPI_ISL_1452625, EPI_ISL_1452888, EPI_ISL_1452894 to 1452895, EPI_ISL_1452897 to 1452898, EPI_ISL_1452902 to 1452903, EPI_ISL_1452907, EPI_ISL_1452918, EPI_ISL_1452927, EPI_ISL_1452931, EPI_ISL_1452933 to 1452934, EPI_ISL_1452937, EPI_ISL_1452940 to 1452942, EPI_ISL_1452944, EPI_ISL_1452947 to 1452948, EPI_ISL_1452952 to 1452953, EPI_ISL_1452956, EPI_ISL_1452958, EPI_ISL_1452964, EPI_ISL_1452966, EPI_ISL_1452972, EPI_ISL_1452979 to 1452980, EPI_ISL_1452984, EPI_ISL_1452988 to 1452990, EPI_ISL_1452995, EPI_ISL_1452997 to 1452998, EPI_ISL_1453006, EPI_ISL_1453014, EPI_ISL_1453019 to 1453020, EPI_ISL_1453024, EPI_ISL_1453028, EPI_ISL_1453033, EPI_ISL_1453041, EPI_ISL_1453044 to 1453045, EPI_ISL_1453049, EPI_ISL_1453054, EPI_ISL_1453059, EPI_ISL_1453064, EPI_ISL_1453067, EPI_ISL_1453074, EPI_ISL_1453077, EPI_ISL_1453090 to 1453091, EPI_ISL_1453096, EPI_ISL_1453099, EPI_ISL_1453102, EPI_ISL_1453106, EPI_ISL_1453112, EPI_ISL_1453119, EPI_ISL_1453121, EPI_ISL_1453129, EPI_ISL_1453131, EPI_ISL_1453133 to 1453134, EPI_ISL_1453140 to 1453141, EPI_ISL_1453145, EPI_ISL_1453149 to 1453151, EPI_ISL_1453161, EPI_ISL_1453164, EPI_ISL_1453168, EPI_ISL_1453170 to 1453173, EPI_ISL_1453176 to 1453177, EPI_ISL_1453180, EPI_ISL_1453472,			

EPI_ISL_1453474, EPI_ISL_1453477, EPI_ISL_1453480, EPI_ISL_1453483, EPI_ISL_1453486, EPI_ISL_1453488, EPI_ISL_1453498, EPI_ISL_1453500, EPI_ISL_1453504, EPI_ISL_1453506 to 1453507, EPI_ISL_1453511, EPI_ISL_1453513, EPI_ISL_1453518 to 1453519, EPI_ISL_1453521, EPI_ISL_1453526, EPI_ISL_1453534, EPI_ISL_1453536 to 1453537, EPI_ISL_1453540, EPI_ISL_1453548, EPI_ISL_1453550, EPI_ISL_1453555, EPI_ISL_1453559, EPI_ISL_1453564, EPI_ISL_1453567, EPI_ISL_1453569, EPI_ISL_1453571 to 1453573, EPI_ISL_1453577, EPI_ISL_1453579, EPI_ISL_1453582 to 1453584, EPI_ISL_1453586, EPI_ISL_1453595, EPI_ISL_1453603 to 1453604, EPI_ISL_1453615, EPI_ISL_1453626, EPI_ISL_1453644, EPI_ISL_1453646, EPI_ISL_1453648, EPI_ISL_1453655, EPI_ISL_1453659 to 1453660, EPI_ISL_1453665, EPI_ISL_1453677 to 1453678, EPI_ISL_1453692, EPI_ISL_1453699 to 1453700, EPI_ISL_1453702, EPI_ISL_1453705 to 1453706, EPI_ISL_1453708, EPI_ISL_1453711 to 1453712, EPI_ISL_1453714, EPI_ISL_1453718, EPI_ISL_1453736, EPI_ISL_1453749, EPI_ISL_1453753, EPI_ISL_1453764, EPI_ISL_1453772, EPI_ISL_1453777, EPI_ISL_1453779, EPI_ISL_1453781 to 1453783, EPI_ISL_1453787, EPI_ISL_1453791, EPI_ISL_1453793 to 1453794, EPI_ISL_1453796, EPI_ISL_1453799, EPI_ISL_1453802 to 1453804, EPI_ISL_1453808, EPI_ISL_1453814, EPI_ISL_1453818 to 1453819, EPI_ISL_1453821, EPI_ISL_1453827, EPI_ISL_1453830, EPI_ISL_1453832 to 1453834, EPI_ISL_1453838, EPI_ISL_1453846 to 1453847, EPI_ISL_1454651, EPI_ISL_1454654, EPI_ISL_1454658, EPI_ISL_1454665, EPI_ISL_1454671, EPI_ISL_1454680 to 1454681, EPI_ISL_1454689 to 1454690, EPI_ISL_1454692 to 1454694, EPI_ISL_1454697, EPI_ISL_1454707, EPI_ISL_1454710 to 1454711, EPI_ISL_1454715, EPI_ISL_1454718, EPI_ISL_1454720 to 1454721, EPI_ISL_1454723 to 1454724, EPI_ISL_1454731, EPI_ISL_1454733, EPI_ISL_1454736, EPI_ISL_1454740, EPI_ISL_1454742, EPI_ISL_1454748, EPI_ISL_1454751, EPI_ISL_1454753, EPI_ISL_1454759 to 1454760, EPI_ISL_1454764, EPI_ISL_1454769, EPI_ISL_1454771, EPI_ISL_1454776, EPI_ISL_1454802, EPI_ISL_1454804 to 1454805, EPI_ISL_1454811, EPI_ISL_1454813, EPI_ISL_1454817, EPI_ISL_1454819, EPI_ISL_1454833, EPI_ISL_1454842 to 1454843, EPI_ISL_1454849 to 1454850, EPI_ISL_1454855, EPI_ISL_1454861, EPI_ISL_1454863 to 1454864, EPI_ISL_1454866, EPI_ISL_1454872, EPI_ISL_1454874, EPI_ISL_1454876, EPI_ISL_1454882, EPI_ISL_1454888 to 1454889, EPI_ISL_1454893, EPI_ISL_1454897, EPI_ISL_1454904, EPI_ISL_1454907, EPI_ISL_1454910 to 1454911, EPI_ISL_1454917, EPI_ISL_1454929, EPI_ISL_1454932 to 1454933, EPI_ISL_1454935, EPI_ISL_1454938, EPI_ISL_1454942, EPI_ISL_1454945, EPI_ISL_1454947, EPI_ISL_1454966, EPI_ISL_1455238, EPI_ISL_1455240, EPI_ISL_1455242, EPI_ISL_1455244, EPI_ISL_1455246, EPI_ISL_1455248, EPI_ISL_1455250, EPI_ISL_1455257 to 1455258, EPI_ISL_1455266 to 1455268, EPI_ISL_1455270, EPI_ISL_1455274 to 1455278, EPI_ISL_1455280 to 1455281, EPI_ISL_1455284, EPI_ISL_1455291, EPI_ISL_1455294 to 1455295, EPI_ISL_1455298, EPI_ISL_1455301 to 1455302, EPI_ISL_1455311, EPI_ISL_1455313, EPI_ISL_1455315 to 1455317, EPI_ISL_1455322, EPI_ISL_1455324, EPI_ISL_1455326, EPI_ISL_1455329, EPI_ISL_1455331, EPI_ISL_1455333, EPI_ISL_1455337, EPI_ISL_1455339 to 1455340, EPI_ISL_1455342, EPI_ISL_1455344 to 1455345, EPI_ISL_1455349 to 1455350, EPI_ISL_1455355, EPI_ISL_1455361 to 1455362, EPI_ISL_1455364 to 1455365, EPI_ISL_1455369, EPI_ISL_1455371, EPI_ISL_1455373 to 1455376, EPI_ISL_1455378 to 1455380, EPI_ISL_1455382 to 1455383, EPI_ISL_1455386 to 1455388, EPI_ISL_1455390 to 1455391, EPI_ISL_1455394 to 1455397, EPI_ISL_1455400 to 1455401, EPI_ISL_1455405, EPI_ISL_1455410 to 1455411, EPI_ISL_1455416 to 1455419, EPI_ISL_1455421, EPI_ISL_1455423 to 1455425, EPI_ISL_1455427, EPI_ISL_1455429, EPI_ISL_1455432, EPI_ISL_1455435, EPI_ISL_1455437, EPI_ISL_1455439 to 1455442, EPI_ISL_1455444 to 1455446, EPI_ISL_1455450, EPI_ISL_1455453, EPI_ISL_1455455 to 1455457, EPI_ISL_1455459 to 1455462, EPI_ISL_1455467, EPI_ISL_1455469 to 1455471, EPI_ISL_1455476, EPI_ISL_1455482 to 1455484, EPI_ISL_1455486, EPI_ISL_1455489 to 1455491, EPI_ISL_1455554 to 1455555, EPI_ISL_1455559 to 1455560, EPI_ISL_1455568 to 1455571, EPI_ISL_1455593, EPI_ISL_1455597 to 1455598, EPI_ISL_1455600 to 1455602, EPI_ISL_1455606 to 1455607, EPI_ISL_1455612, EPI_ISL_1455614, EPI_ISL_1455619, EPI_ISL_1455621, EPI_ISL_1455624, EPI_ISL_1455626, EPI_ISL_1455628 to 1455630, EPI_ISL_1466444, EPI_ISL_1466446, EPI_ISL_1466448, EPI_ISL_1466457, EPI_ISL_1466463, EPI_ISL_1466469, EPI_ISL_1466474, EPI_ISL_1466478, EPI_ISL_1466482, EPI_ISL_1466484, EPI_ISL_1466487, EPI_ISL_1466491 to 1466492, EPI_ISL_1466495 to 1466496, EPI_ISL_1466499, EPI_ISL_1466501, EPI_ISL_1466505, EPI_ISL_1466508 to 1466509, EPI_ISL_1466511, EPI_ISL_1466513, EPI_ISL_1466516 to 1466517, EPI_ISL_1466521, EPI_ISL_1466528, EPI_ISL_1466536, EPI_ISL_1466550 to 1466551, EPI_ISL_1466560, EPI_ISL_1466569, EPI_ISL_1466577, EPI_ISL_1466581, EPI_ISL_1466584, EPI_ISL_1466593 to 1466595, EPI_ISL_1466598 to 1466599, EPI_ISL_1466606, EPI_ISL_1466610, EPI_ISL_1466616 to 1466617, EPI_ISL_1466621, EPI_ISL_1466626, EPI_ISL_1466630, EPI_ISL_1466634, EPI_ISL_1466641, EPI_ISL_1466649, EPI_ISL_1466652 to 1466653, EPI_ISL_1466655 to 1466656, EPI_ISL_1466660, EPI_ISL_1466663, EPI_ISL_1466675, EPI_ISL_1466678, EPI_ISL_1466680, EPI_ISL_1466683, EPI_ISL_1466685 to 1466686, EPI_ISL_1466691, EPI_ISL_1466693, EPI_ISL_1466695 to 1466698, EPI_ISL_1466708 to 1466709, EPI_ISL_1466711, EPI_ISL_1466722, EPI_ISL_1466725, EPI_ISL_1466733, EPI_ISL_1466737 to 1466739, EPI_ISL_1466741 to 1466742, EPI_ISL_1466746, EPI_ISL_1466752, EPI_ISL_1466764, EPI_ISL_1466768, EPI_ISL_1466771, EPI_ISL_1466775, EPI_ISL_1466779, EPI_ISL_1466783, EPI_ISL_1466785 to 1466789, EPI_ISL_1466793 to 1466795, EPI_ISL_1466798, EPI_ISL_1466805, EPI_ISL_1466809, EPI_ISL_1466812 to 1466813, EPI_ISL_1466817, EPI_ISL_1466820, EPI_ISL_1466822, EPI_ISL_1466824, EPI_ISL_1466829, EPI_ISL_1466831 to 1466832, EPI_ISL_1466838, EPI_ISL_1466840, EPI_ISL_1466844, EPI_ISL_1466846 to 1466847, EPI_ISL_1466850, EPI_ISL_1466852, EPI_ISL_1466854, EPI_ISL_1466856, EPI_ISL_1466866, EPI_ISL_1466868, EPI_ISL_1466870, EPI_ISL_1466872, EPI_ISL_1466878, EPI_ISL_1466881, EPI_ISL_1466883 to 1466884, EPI_ISL_1466886, EPI_ISL_1466888 to 1466890, EPI_ISL_1466892, EPI_ISL_1466894, EPI_ISL_1466896 to 1466897, EPI_ISL_1466906, EPI_ISL_1466910, EPI_ISL_1466913, EPI_ISL_1466916 to 1466917, EPI_ISL_1466920, EPI_ISL_1466922, EPI_ISL_1467259, EPI_ISL_1467266, EPI_ISL_1467274, EPI_ISL_1467277, EPI_ISL_1467280, EPI_ISL_1467284, EPI_ISL_1467297 to 1467298, EPI_ISL_1467301, EPI_ISL_1467306 to 1467307, EPI_ISL_1467310, EPI_ISL_1467314, EPI_ISL_1467316, EPI_ISL_1467320, EPI_ISL_1467324, EPI_ISL_1467328, EPI_ISL_1467333, EPI_ISL_1467338 to 1467339, EPI_ISL_1467344 to 1467347, EPI_ISL_1467353, EPI_ISL_1467357, EPI_ISL_1467359, EPI_ISL_1467361, EPI_ISL_1467368, EPI_ISL_1467375 to 1467376, EPI_ISL_1467378, EPI_ISL_1467381 to 1467382, EPI_ISL_1467384, EPI_ISL_1467391, EPI_ISL_1467393 to 1467394, EPI_ISL_1467396, EPI_ISL_1467398, EPI_ISL_1467400, EPI_ISL_1467415 to 1467416, EPI_ISL_1467419 to 1467420, EPI_ISL_1467422, EPI_ISL_1467432, EPI_ISL_1467436, EPI_ISL_1467438 to 1467439, EPI_ISL_1467445 to 1467446, EPI_ISL_1467448, EPI_ISL_1467452 to 1467453, EPI_ISL_1467457, EPI_ISL_1467464 to 1467465, EPI_ISL_1467469, EPI_ISL_1467472, EPI_ISL_1467477, EPI_ISL_1467484, EPI_ISL_1467492, EPI_ISL_1467495 to 1467497, EPI_ISL_1467503 to 1467504, EPI_ISL_1467506, EPI_ISL_1467509, EPI_ISL_1467511, EPI_ISL_1467513, EPI_ISL_1467515, EPI_ISL_1467518, EPI_ISL_1467520, EPI_ISL_1467522 to 1467524, EPI_ISL_1467528 to 1467529, EPI_ISL_1467533, EPI_ISL_1467537, EPI_ISL_1467539 to 1467541, EPI_ISL_1467550, EPI_ISL_1467559, EPI_ISL_1467564, EPI_ISL_1467573 to 1467574, EPI_ISL_1467577 to 1467578, EPI_ISL_1467803, EPI_ISL_1467835 to 1467838, EPI_ISL_1467840 to 1467841, EPI_ISL_1467843 to 1467845, EPI_ISL_1467847, EPI_ISL_1467852 to 1467854, EPI_ISL_1467857 to 1467860, EPI_ISL_1467862, EPI_ISL_1467868, EPI_ISL_1467874 to 1467877, EPI_ISL_1467879 to 1467881, EPI_ISL_1467885 to 1467889, EPI_ISL_1467892, EPI_ISL_1467897 to 1467900, EPI_ISL_1467905, EPI_ISL_1467908, EPI_ISL_1467913 to 1467914, EPI_ISL_1467917 to 1467919, EPI_ISL_1467921, EPI_ISL_1467924 to 1467930, EPI_ISL_1467932 to 1467933, EPI_ISL_1467937, EPI_ISL_1467939, EPI_ISL_1467941, EPI_ISL_1467943, EPI_ISL_1467946 to 1467949, EPI_ISL_1467952 to 1467953, EPI_ISL_1467955 to 1467957, EPI_ISL_1467963, EPI_ISL_1467965 to 1467970, EPI_ISL_1467972 to 1467973, EPI_ISL_1467975, EPI_ISL_1467978 to 1467981, EPI_ISL_1467986 to 1467987, EPI_ISL_1467991, EPI_ISL_1467993 to 1467996, EPI_ISL_1467999 to 1468002, EPI_ISL_1468006 to 1468010, EPI_ISL_1468012, EPI_ISL_1468014, EPI_ISL_1468016 to 1468017, EPI_ISL_1468019, EPI_ISL_1468022, EPI_ISL_1468025 to 1468026, EPI_ISL_1468028 to 1468029, EPI_ISL_1468031 to 1468032, EPI_ISL_1468037, EPI_ISL_1468041, EPI_ISL_1468043, EPI_ISL_1468045, EPI_ISL_1468048 to 1468049, EPI_ISL_1468051 to 1468053, EPI_ISL_1468055 to 1468056, EPI_ISL_1468059 to 1468060, EPI_ISL_1468062 to 1468063, EPI_ISL_1468065 to 1468066, EPI_ISL_1468069, EPI_ISL_1468071, EPI_ISL_1468073 to 1468077, EPI_ISL_1468080 to 1468081, EPI_ISL_1468084 to 1468085, EPI_ISL_1468087 to 1468089, EPI_ISL_1468091, EPI_ISL_1468094 to 1468100, EPI_ISL_1468102 to 1468106, EPI_ISL_1468109 to 1468110, EPI_ISL_1468112 to 1468116, EPI_ISL_1468118, EPI_ISL_1468120 to 1468123, EPI_ISL_1468125 to 1468126, EPI_ISL_1468130 to 1468131, EPI_ISL_1472408, EPI_ISL_1472410 to 1472411, EPI_ISL_1472415, EPI_ISL_1472418, EPI_ISL_1472422, EPI_ISL_1472426 to 1472428, EPI_ISL_1472431, EPI_ISL_1472435 to 1472436, EPI_ISL_1472442, EPI_ISL_1472446, EPI_ISL_1472448, EPI_ISL_1472451 to 1472453, EPI_ISL_1472455 to 1472456, EPI_ISL_1472458, EPI_ISL_1472462, EPI_ISL_1472464 to 1472465, EPI_ISL_1472474, EPI_ISL_1472483, EPI_ISL_1472492, EPI_ISL_1472494, EPI_ISL_1472496, EPI_ISL_1472502, EPI_ISL_1472508, EPI_ISL_1472511 to 1472513, EPI_ISL_1472528, EPI_ISL_1472532, EPI_ISL_1472536, EPI_ISL_1472547, EPI_ISL_1472551 to 1472552, EPI_ISL_1472554, EPI_ISL_1472560, EPI_ISL_1472562, EPI_ISL_1472569, EPI_ISL_1472572, EPI_ISL_1472578, EPI_ISL_1472580, EPI_ISL_1472582, EPI_ISL_1472584, EPI_ISL_1472593 to 1472594, EPI_ISL_1472596, EPI_ISL_1472600, EPI_ISL_1472608, EPI_ISL_1472611, EPI_ISL_1472613, EPI_ISL_1472621 to 1472622, EPI_ISL_1472626 to 1472628, EPI_ISL_1472634, EPI_ISL_1472638, EPI_ISL_1472646, EPI_ISL_1472655, EPI_ISL_1472657 to 1472658, EPI_ISL_1472660, EPI_ISL_1472663, EPI_ISL_1472667, EPI_ISL_1472669 to 1472670, EPI_ISL_1472672, EPI_ISL_1472680 to 1472681, EPI_ISL_1472684, EPI_ISL_1472686, EPI_ISL_1472692, EPI_ISL_1472696, EPI_ISL_1473514, EPI_ISL_1473559, EPI_ISL_1473810, EPI_ISL_1474156, EPI_ISL_1474161, EPI_ISL_1484103, EPI_ISL_1484118, EPI_ISL_1484126, EPI_ISL_1484131, EPI_ISL_1484133, EPI_ISL_1484137, EPI_ISL_1484142, EPI_ISL_1484151, EPI_ISL_1484170, EPI_ISL_1484173, EPI_ISL_1484175, EPI_ISL_1484202 to 1484203, EPI_ISL_1484210, EPI_ISL_1484251, EPI_ISL_1484258, EPI_ISL_1484260 to 1484261, EPI_ISL_1484282, EPI_ISL_1484293, EPI_ISL_1484842, EPI_ISL_1484854, EPI_ISL_1484857, EPI_ISL_1484863, EPI_ISL_1484867, EPI_ISL_1484872, EPI_ISL_1484883, EPI_ISL_1484891, EPI_ISL_1484894 to 1484896, EPI_ISL_1484902, EPI_ISL_1484911, EPI_ISL_1484913, EPI_ISL_1484919, EPI_ISL_1484922 to 1484923, EPI_ISL_1484925, EPI_ISL_1484927, EPI_ISL_1484935, EPI_ISL_1484938, EPI_ISL_1484942, EPI_ISL_1484948, EPI_ISL_1484952 to 1484953, EPI_ISL_1484955, EPI_ISL_1484957 to 1484958, EPI_ISL_1484960 to 1484961, EPI_ISL_1484973, EPI_ISL_1484984 to 1484986, EPI_ISL_1484991, EPI_ISL_1484998, EPI_ISL_1485007, EPI_ISL_1485009, EPI_ISL_1485018 to 1485019, EPI_ISL_1485026, EPI_ISL_1485042, EPI_ISL_1485044, EPI_ISL_1485048, EPI_ISL_1485050, EPI_ISL_1485056, EPI_ISL_1485058, EPI_ISL_1485067, EPI_ISL_1485076, EPI_ISL_1485079 to 1485080, EPI_ISL_1485082, EPI_ISL_1485084, EPI_ISL_1485093, EPI_ISL_1485096, EPI_ISL_1485098, EPI_ISL_1485101, EPI_ISL_1485106 to 1485107, EPI_ISL_1485112, EPI_ISL_1485121, EPI_ISL_1485126, EPI_ISL_1485130, EPI_ISL_1485132 to 1485134, EPI_ISL_1485138, EPI_ISL_1485141, EPI_ISL_1485145, EPI_ISL_1485147, EPI_ISL_1485166, EPI_ISL_1485175, EPI_ISL_1485501, EPI_ISL_1485524, EPI_ISL_1485544, EPI_ISL_1485563, EPI_ISL_1485574, EPI_ISL_1485584, EPI_ISL_1485599, EPI_ISL_1485603, EPI_ISL_1485606, EPI_ISL_1485701, EPI_ISL_1485728, EPI_ISL_1485733, EPI_ISL_1485740, EPI_ISL_1485756, EPI_ISL_1485761, EPI_ISL_1485763, EPI_ISL_1486113, EPI_ISL_1486117, EPI_ISL_1486120, EPI_ISL_1486132, EPI_ISL_1486135, EPI_ISL_1486139, EPI_ISL_1486141, EPI_ISL_1486144, EPI_ISL_1486156, EPI_ISL_1486159, EPI_ISL_1486166, EPI_ISL_1486169 to 1486170, EPI_ISL_1486173, EPI_ISL_1486179, EPI_ISL_1486189, EPI_ISL_1486192, EPI_ISL_1486205 to 1486206, EPI_ISL_1486222, EPI_ISL_1486226, EPI_ISL_1486233, EPI_ISL_1486242, EPI_ISL_1486254, EPI_ISL_1486259, EPI_ISL_1486262, EPI_ISL_1486270 to 1486271, EPI_ISL_1486276, EPI_ISL_1486288, EPI_ISL_1486293, EPI_ISL_1486298, EPI_ISL_1486301, EPI_ISL_1486306 to 1486307, EPI_ISL_1486313, EPI_ISL_1486327, EPI_ISL_1486341, EPI_ISL_1486344, EPI_ISL_1486348, EPI_ISL_1486351, EPI_ISL_1486359, EPI_ISL_1486367, EPI_ISL_1486369, EPI_ISL_1486376, EPI_ISL_1486381, EPI_ISL_1486383, EPI_ISL_1486385, EPI_ISL_1486400, EPI_ISL_1486403 to 1486404, EPI_ISL_1486406 to 1486408, EPI_ISL_1486410, EPI_ISL_1486413 to 1486414, EPI_ISL_1486416 to 1486417, EPI_ISL_1486419 to 1486420, EPI_ISL_1486423, EPI_ISL_1486432 to 1486433, EPI_ISL_1486435, EPI_ISL_1486444, EPI_ISL_1486446 to 1486447, EPI_ISL_1486450, EPI_ISL_1486453 to 1486454, EPI_ISL_1486456 to 1486457, EPI_ISL_1486460 to 1486464, EPI_ISL_1486466 to 1486469, EPI_ISL_1486471, EPI_ISL_1486473, EPI_ISL_1486479 to 1486480, EPI_ISL_1486482, EPI_ISL_1486485 to 1486489, EPI_ISL_1486491 to 1486492, EPI_ISL_1486495 to 1486496, EPI_ISL_1486498, EPI_ISL_1486500, EPI_ISL_1486502 to 1486504, EPI_ISL_1486506, EPI_ISL_1486509 to 1486512, EPI_ISL_1486516, EPI_ISL_1486518, EPI_ISL_1486523 to 1486524, EPI_ISL_1486527, EPI_ISL_1486532, EPI_ISL_1486535 to 1486536, EPI_ISL_1486539, EPI_ISL_1486541, EPI_ISL_1486544, EPI_ISL_1486547, EPI_ISL_1486551, EPI_ISL_1486553, EPI_ISL_1486561, EPI_ISL_1486563 to 1486564, EPI_ISL_1486567, EPI_ISL_1486569, EPI_ISL_1486572, EPI_ISL_1486576, EPI_ISL_1486578, EPI_ISL_1486584 to 1486586, EPI_ISL_1486589, EPI_ISL_1486595, EPI_ISL_1486604, EPI_ISL_1486606 to 1486607, EPI_ISL_1486609, EPI_ISL_1486612, EPI_ISL_1486614 to 1486615, EPI_ISL_1486624, EPI_ISL_1486626, EPI_ISL_1486632, EPI_ISL_1486641, EPI_ISL_1486644 to 1486645, EPI_ISL_1486648, EPI_ISL_1486651, EPI_ISL_1486653, EPI_ISL_1486656 to 1486657, EPI_ISL_1486659, EPI_ISL_1486661, EPI_ISL_1486669, EPI_ISL_1486673, EPI_ISL_1486675 to 1486677, EPI_ISL_1486682, EPI_ISL_1486685 to 1486686, EPI_ISL_1486694 to 1486695, EPI_ISL_1486698, EPI_ISL_1486702, EPI_ISL_1486704 to 1486705, EPI_ISL_1486712, EPI_ISL_1486714, EPI_ISL_1486718, EPI_ISL_1486726, EPI_ISL_1486731, EPI_ISL_1486737, EPI_ISL_1486740 to 1486741, EPI_ISL_1486745, EPI_ISL_1486747, EPI_ISL_1486749, EPI_ISL_1486753, EPI_ISL_1486756 to 1486757, EPI_ISL_1486763, EPI_ISL_1486768, EPI_ISL_1486771 to 1486772, EPI_ISL_1486774 to 1486775, EPI_ISL_1486777 to 1486778, EPI_ISL_1486783 to 1486784, EPI_ISL_1486789, EPI_ISL_1486798 to 1486803, EPI_ISL_1486806, EPI_ISL_1486812, EPI_ISL_1486815, EPI_ISL_1486818, EPI_ISL_1486825, EPI_ISL_1486832, EPI_ISL_1486836, EPI_ISL_1486839 to 1486840, EPI_ISL_1486857, EPI_ISL_1486866, EPI_ISL_1486868, EPI_ISL_1486872 to 1486873, EPI_ISL_1486876, EPI_ISL_1486880, EPI_ISL_1486884 to 1486885, EPI_ISL_1486890 to 1486891, EPI_ISL_1486896, EPI_ISL_1486899, EPI_ISL_1486913, EPI_ISL_1486916, EPI_ISL_1486919, EPI_ISL_1486925 to 1486926, EPI_ISL_1486933, EPI_ISL_1486937, EPI_ISL_1486939, EPI_ISL_1486944, EPI_ISL_1486951 to 1486953, EPI_ISL_1486956, EPI_ISL_1486959 to 1486960, EPI_ISL_1486964 to 1486965, EPI_ISL_1486968, EPI_ISL_1486977, EPI_ISL_1486982, EPI_ISL_1486987, EPI_ISL_1486989, EPI_ISL_1486993 to 1486994, EPI_ISL_1486996 to 1486997, EPI_ISL_1486999 to 1487001, EPI_ISL_1487003, EPI_ISL_1487005, EPI_ISL_1487013, EPI_ISL_1487016 to 1487017, EPI_ISL_1487022, EPI_ISL_1487024, EPI_ISL_1487032, EPI_ISL_1487034, EPI_ISL_1487036 to 1487037, EPI_ISL_1487042, EPI_ISL_1487044 to 1487045, EPI_ISL_1487052, EPI_ISL_1487056, EPI_ISL_1487060, EPI_ISL_1487062 to 1487063, EPI_ISL_1487068, EPI_ISL_1487070, EPI_ISL_1487072, EPI_ISL_1487074, EPI_ISL_1487077, EPI_ISL_1487083, EPI_ISL_1487086, EPI_ISL_1487089 to 1487090, EPI_ISL_1487094 to 1487096, EPI_ISL_1487098, EPI_ISL_1487100, EPI_ISL_1487103, EPI_ISL_1487106 to 1487107, EPI_ISL_1487114, EPI_ISL_1487117 to 1487118, EPI_ISL_1487123 to 1487127, EPI_ISL_1487129, EPI_ISL_1487131 to 1487132, EPI_ISL_1487135 to 1487136, EPI_ISL_1487143, EPI_ISL_1487146, EPI_ISL_1487149, EPI_ISL_1487151, EPI_ISL_1487388, EPI_ISL_1487393, EPI_ISL_1487396, EPI_ISL_1487405, EPI_ISL_1487415, EPI_ISL_1487433 to 1487434, EPI_ISL_1487437, EPI_ISL_1487440, EPI_ISL_1487452, EPI_ISL_1487458, EPI_ISL_1487462, EPI_ISL_1487474, EPI_ISL_1487477, EPI_ISL_1487491, EPI_ISL_1487497, EPI_ISL_1487499, EPI_ISL_1487501 to 1487502, EPI_ISL_1487511, EPI_ISL_1487518, EPI_ISL_1487521 to 1487522, EPI_ISL_1487530, EPI_ISL_1487537, EPI_ISL_1487541, EPI_ISL_1487546, EPI_ISL_1487549, EPI_ISL_1487551, EPI_ISL_1487566, EPI_ISL_1487573, EPI_ISL_1487577, EPI_ISL_1487589, EPI_ISL_1487595, EPI_ISL_1487603, EPI_ISL_1487609, EPI_ISL_1487613 to 1487614, EPI_ISL_1487617, EPI_ISL_1487624, EPI_ISL_1487648, EPI_ISL_1487653, EPI_ISL_1487655, EPI_ISL_1487657 to 1487658, EPI_ISL_1487670, EPI_ISL_1487704, EPI_ISL_1487716, EPI_ISL_1487719, EPI_ISL_1488227, EPI_ISL_1488230, EPI_ISL_1488259, EPI_ISL_1488261, EPI_ISL_1488270, EPI_ISL_1488286, EPI_ISL_1488292, EPI_ISL_1488294, EPI_ISL_1488298, EPI_ISL_1488333, EPI_ISL_1488337 to 1488338, EPI_ISL_1488343, EPI_ISL_1488346, EPI_ISL_1488354, EPI_ISL_1488361, EPI_ISL_1488364, EPI_ISL_1488366, EPI_ISL_1488376, EPI_ISL_1488387 to 1488388, EPI_ISL_1488397, EPI_ISL_1488406, EPI_ISL_1488422 to 1488423, EPI_ISL_1488427 to 1488428, EPI_ISL_1488433, EPI_ISL_1488435, EPI_ISL_1488438, EPI_ISL_1488450 to 1488451, EPI_ISL_1488476 to 1488477, EPI_ISL_1489462, EPI_ISL_1489472, EPI_ISL_1504115 to 1504116, EPI_ISL_1504119, EPI_ISL_1504125, EPI_ISL_1504128, EPI_ISL_1504132 to 1504133, EPI_ISL_1504138, EPI_ISL_1504141, EPI_ISL_1504147, EPI_ISL_1504149, EPI_ISL_1504162 to 1504163, EPI_ISL_1504166 to 1504167, EPI_ISL_1504174, EPI_ISL_1504177, EPI_ISL_1504180 to 1504181, EPI_ISL_1504183 to 1504184, EPI_ISL_1504195 to 1504196, EPI_ISL_1504200, EPI_ISL_1504208 to 1504209, EPI_ISL_1504222, EPI_ISL_1504225 to 1504226, EPI_ISL_1504235, EPI_ISL_1504239 to 1504240, EPI_ISL_1504243 to 1504244, EPI_ISL_1504248 to 1504249, EPI_ISL_1504265, EPI_ISL_1504276, EPI_ISL_1504280, EPI_ISL_1504284, EPI_ISL_1504288 to 1504289, EPI_ISL_1504291, EPI_ISL_1504294, EPI_ISL_1504297 to 1504298, EPI_ISL_1504303, EPI_ISL_1504306, EPI_ISL_1504313, EPI_ISL_1504316, EPI_ISL_1504320 to 1504323, EPI_ISL_1504325, EPI_ISL_1504327, EPI_ISL_1504329, EPI_ISL_1504333, EPI_ISL_1504335, EPI_ISL_1504340, EPI_ISL_1504347 to 1504348, EPI_ISL_1504352, EPI_ISL_1504355, EPI_ISL_1504361, EPI_ISL_1504365, EPI_ISL_1504368 to 1504369, EPI_ISL_1504373 to 1504375, EPI_ISL_1504380 to 1504381, EPI_ISL_1504386, EPI_ISL_1504388, EPI_ISL_1504392, EPI_ISL_1504394, EPI_ISL_1504911, EPI_ISL_1504915, EPI_ISL_1504924 to 1504929, EPI_ISL_1504932, EPI_ISL_1504936 to 1504938, EPI_ISL_1504942, EPI_ISL_1504945, EPI_ISL_1504949, EPI_ISL_1504956, EPI_ISL_1504962, EPI_ISL_1504967, EPI_ISL_1504969 to 1504970, EPI_ISL_1504973, EPI_ISL_1504984, EPI_ISL_1504989, EPI_ISL_1504993 to 1504995, EPI_ISL_1504997 to 1504998, EPI_ISL_1505000, EPI_ISL_1505002 to 1505003, EPI_ISL_1505007, EPI_ISL_1505009 to 1505010, EPI_ISL_1505016, EPI_ISL_1505019 to 1505020, EPI_ISL_1505026, EPI_ISL_1505030 to 1505032, EPI_ISL_1505035, EPI_ISL_1505041 to 1505042, EPI_ISL_1505047, EPI_ISL_1505052 to 1505053, EPI_ISL_1505055, EPI_ISL_1505063, EPI_ISL_1505067, EPI_ISL_1505071, EPI_ISL_1505079, EPI_ISL_1505081, EPI_ISL_1505088, EPI_ISL_1505099 to 1505100, EPI_ISL_1505102, EPI_ISL_1505109, EPI_ISL_1505112, EPI_ISL_1505114, EPI_ISL_1505118, EPI_ISL_1505121, EPI_ISL_1505123, EPI_ISL_1505127, EPI_ISL_1505131, EPI_ISL_1505133 to 1505134, EPI_ISL_1505137, EPI_ISL_1505141, EPI_ISL_1505143, EPI_ISL_1505149, EPI_ISL_1505151, EPI_ISL_1505154 to 1505157, EPI_ISL_1505161 to 1505163, EPI_ISL_1505166, EPI_ISL_1505169, EPI_ISL_1505171, EPI_ISL_1505182, EPI_ISL_1505192, EPI_ISL_1505194, EPI_ISL_1505199, EPI_ISL_1505202, EPI_ISL_1505210, EPI_ISL_1505219, EPI_ISL_1505223, EPI_ISL_1505226, EPI_ISL_1505235, EPI_ISL_1505238, EPI_ISL_1505241, EPI_ISL_1505249, EPI_ISL_1505251 to 1505252, EPI_ISL_1505262, EPI_ISL_1505274 to 1505276, EPI_ISL_1505282, EPI_ISL_1505285, EPI_ISL_1505287 to 1505288, EPI_ISL_1505298, EPI_ISL_1505302, EPI_ISL_1505315, EPI_ISL_1505327, EPI_ISL_1505336 to 1505337, EPI_ISL_1505340 to 1505342, EPI_ISL_1505351 to 1505353, EPI_ISL_1505360, EPI_ISL_1505365, EPI_ISL_1505372, EPI_ISL_1505380, EPI_ISL_1505384, EPI_ISL_1505386 to 1505387, EPI_ISL_1505391, EPI_ISL_1505396, EPI_ISL_1505402, EPI_ISL_1505406, EPI_ISL_1505416, EPI_ISL_1505418, EPI_ISL_1505426 to 1505428, EPI_ISL_1505439 to 1505440, EPI_ISL_1505456, EPI_ISL_1505797, EPI_ISL_1505800, EPI_ISL_1505804 to 1505805, EPI_ISL_1505819, EPI_ISL_1505826, EPI_ISL_1505831, EPI_ISL_1505841 to 1505842, EPI_ISL_1505850 to 1505851, EPI_ISL_1505857, EPI_ISL_1505861, EPI_ISL_1505864, EPI_ISL_1505872, EPI_ISL_1505882, EPI_ISL_1505884, EPI_ISL_1505891, EPI_ISL_1505894, EPI_ISL_1505898, EPI_ISL_1505902, EPI_ISL_1505904 to 1505906, EPI_ISL_1505909, EPI_ISL_1505911, EPI_ISL_1505914, EPI_ISL_1505916, EPI_ISL_1505923, EPI_ISL_1505930, EPI_ISL_1505932, EPI_ISL_1505946 to 1505948, EPI_ISL_1505955, EPI_ISL_1505957, EPI_ISL_1505962, EPI_ISL_1505966 to 1505968, EPI_ISL_1505978, EPI_ISL_1505981 to 1505985, EPI_ISL_1505988, EPI_ISL_1505992, EPI_ISL_1505997 to 1505998, EPI_ISL_1506011, EPI_ISL_1506013, EPI_ISL_1506021, EPI_ISL_1506024, EPI_ISL_1506028, EPI_ISL_1506031 to 1506032, EPI_ISL_1506061, EPI_ISL_1506064, EPI_ISL_1506068, EPI_ISL_1506073, EPI_ISL_1506077, EPI_ISL_1506081 to 1506082, EPI_ISL_1506084, EPI_ISL_1506087, EPI_ISL_1506095 to 1506096, EPI_ISL_1506098, EPI_ISL_1506101, EPI_ISL_1518066 to 1518067, EPI_ISL_1518071, EPI_ISL_1518083 to 1518085, EPI_ISL_1518087 to 1518088, EPI_ISL_1518093 to 1518094, EPI_ISL_1518101, EPI_ISL_1518103, EPI_ISL_1518111, EPI_ISL_1518118, EPI_ISL_1518121, EPI_ISL_1518133, EPI_ISL_1518135 to 1518136, EPI_ISL_1518138, EPI_ISL_1518140, EPI_ISL_1518145, EPI_ISL_1518149, EPI_ISL_1518157, EPI_ISL_1518160 to 1518162, EPI_ISL_1518164, EPI_ISL_1518167, EPI_ISL_1518172 to 1518176, EPI_ISL_1518178 to 1518179, EPI_ISL_1518182, EPI_ISL_1518185 to 1518186, EPI_ISL_1518189, EPI_ISL_1518194, EPI_ISL_1518198, EPI_ISL_1518203, EPI_ISL_1518205, EPI_ISL_1518207, EPI_ISL_1518214, EPI_ISL_1518221 to 1518223, EPI_ISL_1518228, EPI_ISL_1518230 to 1518231, EPI_ISL_1518236, EPI_ISL_1518249, EPI_ISL_1518251, EPI_ISL_1518254 to 1518255, EPI_ISL_1518257, EPI_ISL_1518260, EPI_ISL_1518263, EPI_ISL_1518266, EPI_ISL_1518273, EPI_ISL_1518277 to 1518278, EPI_ISL_1518281, EPI_ISL_1518289, EPI_ISL_1518291, EPI_ISL_1518294, EPI_ISL_1518297, EPI_ISL_1518303, EPI_ISL_1518308, EPI_ISL_1518311 to 1518312, EPI_ISL_1518314, EPI_ISL_1518316, EPI_ISL_1518318, EPI_ISL_1518322, EPI_ISL_1518328, EPI_ISL_1518330, EPI_ISL_1518332, EPI_ISL_1518334, EPI_ISL_1518336, EPI_ISL_1518343 to 1518344, EPI_ISL_1518349, EPI_ISL_1518355, EPI_ISL_1518358, EPI_ISL_1518363, EPI_ISL_1518369, EPI_ISL_1518374, EPI_ISL_1518382, EPI_ISL_1518385, EPI_ISL_1518388, EPI_ISL_1518392, EPI_ISL_1518687, EPI_ISL_1518690 to 1518691, EPI_ISL_1518695 to 1518696, EPI_ISL_1518701, EPI_ISL_1518713, EPI_ISL_1518715, EPI_ISL_1518718, EPI_ISL_1518725, EPI_ISL_1518727, EPI_ISL_1518730, EPI_ISL_1518736, EPI_ISL_1518746, EPI_ISL_1518753, EPI_ISL_1518757, EPI_ISL_1518759, EPI_ISL_1518762 to 1518763, EPI_ISL_1518771, EPI_ISL_1518777, EPI_ISL_1518786, EPI_ISL_1518791 to 1518793, EPI_ISL_1518803, EPI_ISL_1518810 to 1518811, EPI_ISL_1518817 to 1518819, EPI_ISL_1518821 to 1518822, EPI_ISL_1518830, EPI_ISL_1518836, EPI_ISL_1518838, EPI_ISL_1518848, EPI_ISL_1518853, EPI_ISL_1518861 to 1518862, EPI_ISL_1518865, EPI_ISL_1518873, EPI_ISL_1518877, EPI_ISL_1518881, EPI_ISL_1518888 to 1518889, EPI_ISL_1518901, EPI_ISL_1518903, EPI_ISL_1518907, EPI_ISL_1518913, EPI_ISL_1518916, EPI_ISL_1518920 to 1518921, EPI_ISL_1518927, EPI_ISL_1518935, EPI_ISL_1518943, EPI_ISL_1518946, EPI_ISL_1518957, EPI_ISL_1518959, EPI_ISL_1518969, EPI_ISL_1518978, EPI_ISL_1518984, EPI_ISL_1518988 to 1518989, EPI_ISL_1518996 to 1518997, EPI_ISL_1518999 to 1519000, EPI_ISL_1519004 to 1519005, EPI_ISL_1519009 to 1519015, EPI_ISL_1519017 to 1519018, EPI_ISL_1519020, EPI_ISL_1519022 to 1519028, EPI_ISL_1519033 to 1519035, EPI_ISL_1519037 to 1519038,

EPI_ISL_1519040 to 1519045, EPI_ISL_1519047, EPI_ISL_1519049, EPI_ISL_1519051 to 1519055, EPI_ISL_1519057, EPI_ISL_1519061, EPI_ISL_1519063, EPI_ISL_1519066, EPI_ISL_1519068, EPI_ISL_1519070 to 1519071, EPI_ISL_1519076, EPI_ISL_1519079 to 1519085, EPI_ISL_1519088, EPI_ISL_1519090 to 1519091, EPI_ISL_1519093 to 1519094, EPI_ISL_1519097 to 1519099, EPI_ISL_1519102 to 1519103, EPI_ISL_1519105 to 1519106, EPI_ISL_1519108, EPI_ISL_1519110 to 1519111, EPI_ISL_1519115, EPI_ISL_1519117 to 1519119, EPI_ISL_1519121 to 1519124, EPI_ISL_1519126, EPI_ISL_1519128 to 1519132, EPI_ISL_1519134 to 1519137, EPI_ISL_1519140 to 1519144, EPI_ISL_1519148 to 1519149, EPI_ISL_1519151 to 1519153, EPI_ISL_1519158 to 1519161, EPI_ISL_1519163 to 1519168, EPI_ISL_1519170 to 1519174, EPI_ISL_1519176 to 1519177, EPI_ISL_1519179 to 1519184, EPI_ISL_1519186 to 1519188, EPI_ISL_1519191, EPI_ISL_1519194 to 1519195, EPI_ISL_1519198 to 1519199, EPI_ISL_1519201, EPI_ISL_1519203, EPI_ISL_1519205 to 1519207, EPI_ISL_1519209 to 1519211, EPI_ISL_1519213 to 1519214, EPI_ISL_1519217, EPI_ISL_1519219 to 1519221, EPI_ISL_1519223, EPI_ISL_1519229, EPI_ISL_1519231 to 1519232, EPI_ISL_1519234 to 1519236, EPI_ISL_1519238 to 1519239, EPI_ISL_1519241 to 1519242, EPI_ISL_1519245, EPI_ISL_1519248, EPI_ISL_1519251, EPI_ISL_1519256 to 1519258, EPI_ISL_1519262, EPI_ISL_1519265, EPI_ISL_1519295, EPI_ISL_1535013, EPI_ISL_1535015, EPI_ISL_1535017 to 1535018, EPI_ISL_1535020, EPI_ISL_1535022, EPI_ISL_1535025 to 1535026, EPI_ISL_1535039 to 1535041, EPI_ISL_1535046 to 1535047, EPI_ISL_1535049, EPI_ISL_1535051 to 1535052, EPI_ISL_1535056 to 1535058, EPI_ISL_1535060, EPI_ISL_1535063 to 1535064, EPI_ISL_1535066, EPI_ISL_1535068 to 1535069, EPI_ISL_1535072 to 1535073, EPI_ISL_1535075 to 1535076, EPI_ISL_1535079, EPI_ISL_1535081, EPI_ISL_1535083, EPI_ISL_1535086 to 1535089, EPI_ISL_1535093 to 1535094, EPI_ISL_1535097, EPI_ISL_1535101 to 1535104, EPI_ISL_1535106, EPI_ISL_1535108 to 1535109, EPI_ISL_1535111 to 1535112, EPI_ISL_1535114 to 1535115, EPI_ISL_1535121 to 1535124, EPI_ISL_1535127, EPI_ISL_1535131, EPI_ISL_1535134 to 1535138, EPI_ISL_1535141 to 1535142, EPI_ISL_1535145, EPI_ISL_1535147 to 1535150, EPI_ISL_1535152, EPI_ISL_1535155, EPI_ISL_1535158, EPI_ISL_1535160, EPI_ISL_1535163, EPI_ISL_1535165 to 1535166, EPI_ISL_1535168, EPI_ISL_1535170 to 1535173, EPI_ISL_1535175 to 1535176, EPI_ISL_1535179, EPI_ISL_1535183 to 1535185, EPI_ISL_1535187 to 1535189, EPI_ISL_1535192 to 1535193, EPI_ISL_1535196 to 1535197, EPI_ISL_1535199 to 1535202, EPI_ISL_1535205, EPI_ISL_1535208 to 1535209, EPI_ISL_1535211 to 1535214, EPI_ISL_1535217, EPI_ISL_1535220 to 1535221, EPI_ISL_1535224 to 1535226, EPI_ISL_1535228, EPI_ISL_1535230, EPI_ISL_1535232 to 1535233, EPI_ISL_1535235 to 1535237, EPI_ISL_1535239 to 1535240, EPI_ISL_1535245, EPI_ISL_1535247, EPI_ISL_1535249, EPI_ISL_1535256 to 1535257, EPI_ISL_1535259 to 1535261, EPI_ISL_1535268 to 1535270, EPI_ISL_1535273, EPI_ISL_1535277 to 1535278, EPI_ISL_1535284, EPI_ISL_1535286, EPI_ISL_1535289 to 1535290, EPI_ISL_1535294, EPI_ISL_1535298 to 1535300, EPI_ISL_1535304 to 1535305, EPI_ISL_1535307 to 1535309, EPI_ISL_1535311, EPI_ISL_1535316 to 1535317, EPI_ISL_1535320 to 1535321, EPI_ISL_1535323 to 1535324, EPI_ISL_1535328, EPI_ISL_1535334 to 1535336, EPI_ISL_1535338 to 1535356, EPI_ISL_1535358 to 1535359, EPI_ISL_1535361 to 1535363, EPI_ISL_1535365, EPI_ISL_1535367 to 1535376, EPI_ISL_1535379, EPI_ISL_1535382 to 1535384, EPI_ISL_1535386, EPI_ISL_1535389 to 1535391, EPI_ISL_1535393 to 1535398, EPI_ISL_1535400 to 1535402, EPI_ISL_1535405, EPI_ISL_1535407 to 1535408, EPI_ISL_1535411 to 1535412, EPI_ISL_1535414 to 1535415, EPI_ISL_1535417, EPI_ISL_1535420 to 1535421, EPI_ISL_1535424 to 1535427, EPI_ISL_1535431, EPI_ISL_1535433 to 1535434, EPI_ISL_1535436, EPI_ISL_1535438 to 1535445, EPI_ISL_1535447, EPI_ISL_1535449 to 1535456, EPI_ISL_1535458 to 1535459, EPI_ISL_1535461, EPI_ISL_1535463 to 1535464, EPI_ISL_1535468 to 1535469, EPI_ISL_1535472, EPI_ISL_1535476 to 1535480, EPI_ISL_1535482, EPI_ISL_1535484, EPI_ISL_1535486 to 1535489, EPI_ISL_1535491, EPI_ISL_1535493, EPI_ISL_1535495 to 1535497, EPI_ISL_1535499 to 1535500, EPI_ISL_1535503, EPI_ISL_1535505 to 1535516, EPI_ISL_1535520 to 1535521, EPI_ISL_1535524 to 1535527, EPI_ISL_1535531 to 1535536, EPI_ISL_1535538 to 1535539, EPI_ISL_1535543, EPI_ISL_1535545, EPI_ISL_1535547 to 1535548, EPI_ISL_1535550 to 1535552, EPI_ISL_1535554, EPI_ISL_1535557 to 1535558, EPI_ISL_1535561 to 1535563, EPI_ISL_1535565 to 1535567, EPI_ISL_1535569 to 1535572, EPI_ISL_1535574 to 1535577, EPI_ISL_1535579, EPI_ISL_1535581 to 1535582, EPI_ISL_1535584, EPI_ISL_1535586 to 1535589, EPI_ISL_1535591 to 1535593, EPI_ISL_1535595, EPI_ISL_1535597 to 1535598, EPI_ISL_1535600 to 1535601, EPI_ISL_1535603, EPI_ISL_1535605, EPI_ISL_1535607, EPI_ISL_1535609, EPI_ISL_1535619 to 1535620, EPI_ISL_1535623, EPI_ISL_1535629, EPI_ISL_1535635, EPI_ISL_1535640, EPI_ISL_1535644, EPI_ISL_1535650, EPI_ISL_1535663, EPI_ISL_1535665, EPI_ISL_1535670, EPI_ISL_1535676, EPI_ISL_1535694, EPI_ISL_1535699 to 1535700, EPI_ISL_1535703, EPI_ISL_1535709, EPI_ISL_1535711, EPI_ISL_1535715, EPI_ISL_1535721, EPI_ISL_1535726, EPI_ISL_1535731, EPI_ISL_1535733, EPI_ISL_1535738, EPI_ISL_1535743, EPI_ISL_1535745, EPI_ISL_1535750, EPI_ISL_1535756 to 1535757, EPI_ISL_1535768, EPI_ISL_1535779 to 1535780, EPI_ISL_1535786, EPI_ISL_1535795, EPI_ISL_1535801, EPI_ISL_1535811, EPI_ISL_1535815, EPI_ISL_1535817 to 1535818, EPI_ISL_1535822, EPI_ISL_1535827, EPI_ISL_1535830, EPI_ISL_1535832 to 1535833, EPI_ISL_1535844, EPI_ISL_1535849 to 1535850, EPI_ISL_1535860, EPI_ISL_1535866, EPI_ISL_1535869, EPI_ISL_1535880, EPI_ISL_1535887 to 1535888, EPI_ISL_1535909, EPI_ISL_1535911, EPI_ISL_1535938, EPI_ISL_1535941, EPI_ISL_1535957, EPI_ISL_1535965, EPI_ISL_1535980, EPI_ISL_1535987, EPI_ISL_1535989, EPI_ISL_1536013, EPI_ISL_1536017 to 1536018, EPI_ISL_1536021, EPI_ISL_1536034, EPI_ISL_1536037, EPI_ISL_1536048, EPI_ISL_1536065, EPI_ISL_1536074, EPI_ISL_1536084, EPI_ISL_1536088, EPI_ISL_1536092 to 1536093, EPI_ISL_1536095 to 1536096, EPI_ISL_1536115, EPI_ISL_1536128, EPI_ISL_1536159, EPI_ISL_1536202, EPI_ISL_1536214, EPI_ISL_1536228, EPI_ISL_1536230, EPI_ISL_1536233, EPI_ISL_1536253, EPI_ISL_1536264, EPI_ISL_1536276, EPI_ISL_1536296, EPI_ISL_1536302, EPI_ISL_1536310, EPI_ISL_1536312, EPI_ISL_1536320, EPI_ISL_1536329, EPI_ISL_1536343, EPI_ISL_1536348, EPI_ISL_1536350, EPI_ISL_1536357, EPI_ISL_1536380, EPI_ISL_1536396, EPI_ISL_1536402, EPI_ISL_1536404, EPI_ISL_1536409, EPI_ISL_1536411, EPI_ISL_1536423, EPI_ISL_1536425, EPI_ISL_1536429, EPI_ISL_1536442, EPI_ISL_1536446, EPI_ISL_1536453, EPI_ISL_1536467, EPI_ISL_1536503, EPI_ISL_1536508, EPI_ISL_1536521, EPI_ISL_1536527, EPI_ISL_1536535 to 1536536, EPI_ISL_1536540, EPI_ISL_1536552, EPI_ISL_1536554, EPI_ISL_1536557, EPI_ISL_1536561 to 1536566, EPI_ISL_1536569, EPI_ISL_1536573, EPI_ISL_1536578 to 1536579, EPI_ISL_1536582, EPI_ISL_1536587, EPI_ISL_1536589, EPI_ISL_1536591 to 1536595, EPI_ISL_1536598, EPI_ISL_1536601, EPI_ISL_1536608, EPI_ISL_1536610 to 1536612, EPI_ISL_1536615 to 1536616, EPI_ISL_1536620 to 1536624, EPI_ISL_1536627, EPI_ISL_1536633, EPI_ISL_1536636, EPI_ISL_1536642, EPI_ISL_1536645, EPI_ISL_1536648 to 1536650, EPI_ISL_1536658 to 1536659, EPI_ISL_1536661 to 1536664, EPI_ISL_1536666, EPI_ISL_1536668 to 1536670, EPI_ISL_1536672, EPI_ISL_1536674, EPI_ISL_1536676 to 1536677, EPI_ISL_1536679, EPI_ISL_1536681 to 1536682, EPI_ISL_1536684, EPI_ISL_1536688, EPI_ISL_1536691 to 1536692, EPI_ISL_1536697, EPI_ISL_1536700, EPI_ISL_1536704 to 1536705, EPI_ISL_1536707, EPI_ISL_1536711 to 1536713, EPI_ISL_1536717, EPI_ISL_1536722 to 1536723, EPI_ISL_1536726, EPI_ISL_1536730, EPI_ISL_1536732, EPI_ISL_1536734, EPI_ISL_1536736 to 1536737, EPI_ISL_1536740, EPI_ISL_1536744, EPI_ISL_1536746, EPI_ISL_1536748 to 1536750, EPI_ISL_1536752, EPI_ISL_1536755, EPI_ISL_1536761, EPI_ISL_1536763, EPI_ISL_1536767, EPI_ISL_1536772, EPI_ISL_1536776, EPI_ISL_1536779, EPI_ISL_1536784, EPI_ISL_1536801, EPI_ISL_1536805, EPI_ISL_1536807 to 1536808, EPI_ISL_1536814, EPI_ISL_1536818 to 1536819, EPI_ISL_1536822, EPI_ISL_1536825 to 1536826, EPI_ISL_1536828, EPI_ISL_1536832 to 1536833, EPI_ISL_1536838 to 1536839, EPI_ISL_1536852, EPI_ISL_1536860, EPI_ISL_1536876, EPI_ISL_1536884 to 1536885, EPI_ISL_1536887, EPI_ISL_1536892, EPI_ISL_1536894, EPI_ISL_1536902, EPI_ISL_1536909, EPI_ISL_1536921, EPI_ISL_1536929, EPI_ISL_1536931, EPI_ISL_1536939, EPI_ISL_1536957, EPI_ISL_1536971, EPI_ISL_1536977, EPI_ISL_1536986, EPI_ISL_1537000, EPI_ISL_1537011 to 1537012, EPI_ISL_1537014, EPI_ISL_1537019, EPI_ISL_1537028, EPI_ISL_1537037, EPI_ISL_1537041, EPI_ISL_1537048, EPI_ISL_1537056, EPI_ISL_1537077, EPI_ISL_1537084, EPI_ISL_1537090, EPI_ISL_1537093, EPI_ISL_1537105, EPI_ISL_1537108 to 1537109, EPI_ISL_1537114, EPI_ISL_1537330 to 1537332, EPI_ISL_1537334 to 1537335, EPI_ISL_1537338 to 1537340, EPI_ISL_1537342, EPI_ISL_1537345, EPI_ISL_1537347 to 1537348, EPI_ISL_1537351, EPI_ISL_1537355, EPI_ISL_1537359 to 1537361, EPI_ISL_1537363 to 1537365, EPI_ISL_1537367, EPI_ISL_1537369 to 1537370, EPI_ISL_1537372 to 1537378, EPI_ISL_1537380, EPI_ISL_1537382, EPI_ISL_1537385 to 1537388, EPI_ISL_1537390, EPI_ISL_1537394 to 1537395, EPI_ISL_1537399, EPI_ISL_1537403 to 1537407, EPI_ISL_1537409 to 1537412, EPI_ISL_1537414 to 1537415, EPI_ISL_1537418 to 1537422, EPI_ISL_1537426, EPI_ISL_1537428 to 1537432, EPI_ISL_1537434 to 1537435, EPI_ISL_1537437, EPI_ISL_1537439 to 1537440, EPI_ISL_1537444, EPI_ISL_1537446, EPI_ISL_1537448, EPI_ISL_1537453 to 1537454, EPI_ISL_1537456, EPI_ISL_1537463, EPI_ISL_1537466 to 1537467, EPI_ISL_1537469, EPI_ISL_1537472, EPI_ISL_1537475 to 1537476, EPI_ISL_1537478, EPI_ISL_1537480 to 1537481, EPI_ISL_1537483 to 1537486, EPI_ISL_1537488, EPI_ISL_1537495 to 1537496, EPI_ISL_1537502 to 1537503, EPI_ISL_1537508, EPI_ISL_1537511 to 1537513, EPI_ISL_1537517, EPI_ISL_1537519 to 1537521, EPI_ISL_1537523, EPI_ISL_1537526, EPI_ISL_1537528, EPI_ISL_1537532, EPI_ISL_1537534, EPI_ISL_1537536, EPI_ISL_1537539 to 1537541, EPI_ISL_1537543 to 1537545, EPI_ISL_1537547, EPI_ISL_1537549, EPI_ISL_1537551, EPI_ISL_1537553, EPI_ISL_1537557 to 1537558, EPI_ISL_1537560, EPI_ISL_1537562 to 1537564, EPI_ISL_1537571 to 1537574, EPI_ISL_1537576 to 1537577, EPI_ISL_1537582 to 1537584, EPI_ISL_1537586, EPI_ISL_1537588 to 1537591, EPI_ISL_1537595, EPI_ISL_1537597 to 1537598, EPI_ISL_1537600 to 1537604, EPI_ISL_1537608 to 1537613, EPI_ISL_1537615 to 1537616, EPI_ISL_1537618 to 1537619, EPI_ISL_1537623 to 1537624, EPI_ISL_1537626 to 1537630, EPI_ISL_1537633 to 1537635, EPI_ISL_1537637 to 1537638, EPI_ISL_1537645, EPI_ISL_1537647, EPI_ISL_1537653 to 1537654, EPI_ISL_1537658 to 1537659, EPI_ISL_1537664, EPI_ISL_1537666, EPI_ISL_1537671, EPI_ISL_1537674, EPI_ISL_1537679, EPI_ISL_1537682, EPI_ISL_1537686, EPI_ISL_1537692, EPI_ISL_1537694, EPI_ISL_1537700 to 1537701, EPI_ISL_1537704 to 1537705, EPI_ISL_1537708 to 1537710, EPI_ISL_1537714, EPI_ISL_1537718, EPI_ISL_1537720, EPI_ISL_1537722 to 1537724, EPI_ISL_1537729, EPI_ISL_1537732, EPI_ISL_1537736, EPI_ISL_1537738, EPI_ISL_1537742, EPI_ISL_1537747 to 1537749, EPI_ISL_1537753 to 1537754, EPI_ISL_1537763, EPI_ISL_1537766 to 1537768, EPI_ISL_1537770, EPI_ISL_1537772, EPI_ISL_1537774 to 1537776, EPI_ISL_1537778, EPI_ISL_1537785 to 1537786, EPI_ISL_1537788 to 1537789, EPI_ISL_1537791, EPI_ISL_1537794, EPI_ISL_1537798, EPI_ISL_1537800 to 1537802, EPI_ISL_1537806 to 1537807, EPI_ISL_1537810, EPI_ISL_1537814, EPI_ISL_1537816, EPI_ISL_1537819, EPI_ISL_1537823, EPI_ISL_1537827 to 1537830, EPI_ISL_1537834, EPI_ISL_1537837 to 1537838, EPI_ISL_1537842 to 1537843, EPI_ISL_1538373, EPI_ISL_1538402, EPI_ISL_1544198 to 1544200, EPI_ISL_1544202, EPI_ISL_1544206, EPI_ISL_1544212 to 1544213, EPI_ISL_1544220, EPI_ISL_1544230, EPI_ISL_1544233, EPI_ISL_1544237 to 1544238, EPI_ISL_1544241, EPI_ISL_1544244 to 1544245, EPI_ISL_1544247, EPI_ISL_1544251 to 1544252, EPI_ISL_1544255 to 1544258, EPI_ISL_1544265 to 1544266, EPI_ISL_1544268 to 1544269, EPI_ISL_1544271 to 1544272, EPI_ISL_1544275, EPI_ISL_1544278 to 1544279, EPI_ISL_1544283 to 1544284, EPI_ISL_1544287, EPI_ISL_1544289, EPI_ISL_1544291 to 1544292, EPI_ISL_1544298, EPI_ISL_1544301, EPI_ISL_1544303, EPI_ISL_1544307, EPI_ISL_1544312, EPI_ISL_1544316, EPI_ISL_1544318, EPI_ISL_1544327, EPI_ISL_1544329 to 1544330, EPI_ISL_1544334 to 1544335, EPI_ISL_1544342, EPI_ISL_1544344 to 1544345, EPI_ISL_1544348, EPI_ISL_1544350, EPI_ISL_1544353, EPI_ISL_1544355 to 1544356, EPI_ISL_1544358, EPI_ISL_1544366 to 1544369, EPI_ISL_1544373 to 1544374, EPI_ISL_1544379, EPI_ISL_1544383 to 1544384, EPI_ISL_1544386, EPI_ISL_1544391 to 1544393, EPI_ISL_1544402, EPI_ISL_1544405, EPI_ISL_1544408, EPI_ISL_1544421 to 1544422, EPI_ISL_1544427 to 1544430, EPI_ISL_1544439, EPI_ISL_1544442, EPI_ISL_1544445, EPI_ISL_1544450 to 1544452, EPI_ISL_1544455, EPI_ISL_1544457, EPI_ISL_1544460, EPI_ISL_1544471 to 1544472, EPI_ISL_1544479, EPI_ISL_1544481 to 1544483, EPI_ISL_1544488

see above	Lighthouse Lab in Milton Keynes	Wellcome Sanger Institute for the COVID-19 Genomics UK (COG-UK) Consortium	Cordelia Langford; David K. Jackson; Dominic Kwiatkowski; Ewan Harrison; Ian Johnston; Jeffrey Barrett; John Sillitoe on behalf of the Wellcome Sanger Institute COVID-19 Surveillance Team; John Sillitoe on behalf of the Wellcome Sanger Institute COVID-19 Surveillance Team (http://www.sanger.ac.uk/covid-team); Roberto Amato; Sonia Goncalves; The Lighthouse Lab in Alderley Park and Alex Alderton; The Lighthouse Lab in Milton Keynes and Alex Alderton

EPI_ISL_549808 to 549828, EPI_ISL_549830 to 549860, EPI_ISL_549862 to 550068, EPI_ISL_550070 to 550484, EPI_ISL_550486 to 550848, EPI_ISL_550850 to 550982, EPI_ISL_550984 to 551000, EPI_ISL_551002 to 551006, EPI_ISL_551008 to 551012, EPI_ISL_551014 to 551091, EPI_ISL_551093 to 551217, EPI_ISL_551219 to 551284, EPI_ISL_551577 to 551734, EPI_ISL_551737 to 551739, EPI_ISL_551741, EPI_ISL_551744 to 551748, EPI_ISL_551750, EPI_ISL_551752 to 551754, EPI_ISL_551757 to 551758, EPI_ISL_551765 to 551768, EPI_ISL_551770 to 551771, EPI_ISL_551773 to 551775, EPI_ISL_551777 to 551778, EPI_ISL_551781 to 551783, EPI_ISL_551786 to 551788, EPI_ISL_551790 to 551794, EPI_ISL_551797, EPI_ISL_551800, EPI_ISL_551802, EPI_ISL_551805, EPI_ISL_551809, EPI_ISL_551813 to 551814, EPI_ISL_551816 to 551817, EPI_ISL_551820 to 551822, EPI_ISL_551828 to 551831, EPI_ISL_551833, EPI_ISL_551922 to 551924, EPI_ISL_551926 to 551931, EPI_ISL_551933 to 551950, EPI_ISL_551952, EPI_ISL_551954 to 551966, EPI_ISL_551968 to 551979, EPI_ISL_551981 to 551982, EPI_ISL_551984 to 551989, EPI_ISL_551991 to 551993, EPI_ISL_551995 to 552000, EPI_ISL_552002 to 552004, EPI_ISL_552006 to 552007, EPI_ISL_552011 to 552016, EPI_ISL_552018 to 552050, EPI_ISL_552052 to 552064, EPI_ISL_552066 to 552072, EPI_ISL_552074 to 552090, EPI_ISL_552092 to 552093, EPI_ISL_552095 to 552097, EPI_ISL_552099 to 552104, EPI_ISL_552107 to 552123, EPI_ISL_552125 to 552150, EPI_ISL_552152 to 552156, EPI_ISL_552158 to 552165, EPI_ISL_552167 to 552169, EPI_ISL_552171 to 552172, EPI_ISL_552174 to 552179, EPI_ISL_552181 to 552184, EPI_ISL_552186 to 552239, EPI_ISL_552241 to 552305, EPI_ISL_552307 to 552445, EPI_ISL_552447 to 552448, EPI_ISL_552450 to 552457, EPI_ISL_552459 to 552462, EPI_ISL_552465, EPI_ISL_552467, EPI_ISL_552469 to 552472, EPI_ISL_552474 to 552475, EPI_ISL_552477 to 552478, EPI_ISL_552480, EPI_ISL_552482 to 552485, EPI_ISL_552487, EPI_ISL_552489 to 552491, EPI_ISL_552493 to 552505, EPI_ISL_552509 to 552511, EPI_ISL_552513 to 552514, EPI_ISL_552517 to 552522, EPI_ISL_552524 to 552525, EPI_ISL_552528 to 552529, EPI_ISL_552531 to 552532, EPI_ISL_552535 to 552538, EPI_ISL_552541 to 552546, EPI_ISL_552548 to 552555, EPI_ISL_552557 to 552561, EPI_ISL_552563 to 552564, EPI_ISL_552567 to 552571, EPI_ISL_552573, EPI_ISL_552575, EPI_ISL_552578, EPI_ISL_552581 to 552582, EPI_ISL_552584 to 552586, EPI_ISL_552588 to 552589, EPI_ISL_552592 to 552594, EPI_ISL_552597, EPI_ISL_552599 to 552606, EPI_ISL_552609 to 552613, EPI_ISL_552615 to 552616, EPI_ISL_552619, EPI_ISL_552621, EPI_ISL_552623 to 552628, EPI_ISL_552630 to 552638, EPI_ISL_552640 to 552641, EPI_ISL_552643, EPI_ISL_552645, EPI_ISL_552647 to 552654, EPI_ISL_552656 to 552658, EPI_ISL_552660 to 552664, EPI_ISL_552667 to 552668, EPI_ISL_552670, EPI_ISL_552672 to 552678, EPI_ISL_552680, EPI_ISL_552682 to 552685, EPI_ISL_552687 to 552688, EPI_ISL_552690 to 552698, EPI_ISL_552700 to 552702, EPI_ISL_552704 to 552705, EPI_ISL_552708 to 552711, EPI_ISL_552714 to 552716, EPI_ISL_552718, EPI_ISL_552725, EPI_ISL_552727, EPI_ISL_552733 to 552734, EPI_ISL_552736 to 552740, EPI_ISL_552743 to 552744, EPI_ISL_552747, EPI_ISL_552751 to 552759, EPI_ISL_552762 to 552767, EPI_ISL_552769, EPI_ISL_552772, EPI_ISL_552778, EPI_ISL_552781, EPI_ISL_552784, EPI_ISL_552786 to 552791, EPI_ISL_552795, EPI_ISL_552799, EPI_ISL_552802, EPI_ISL_552809 to 552811, EPI_ISL_552814, EPI_ISL_552820, EPI_ISL_552823, EPI_ISL_552842, EPI_ISL_552852, EPI_ISL_552867, EPI_ISL_552874 to 552875, EPI_ISL_552879, EPI_ISL_552905, EPI_ISL_552910, EPI_ISL_552916, EPI_ISL_552936, EPI_ISL_552938 to 552939, EPI_ISL_552947 to 552950, EPI_ISL_552953, EPI_ISL_552961, EPI_ISL_552963, EPI_ISL_552967, EPI_ISL_552975, EPI_ISL_552979, EPI_ISL_552987, EPI_ISL_552990, EPI_ISL_552995, EPI_ISL_552997, EPI_ISL_553007 to 553083, EPI_ISL_553085 to 553268, EPI_ISL_553270, EPI_ISL_553275 to 553276, EPI_ISL_553295, EPI_ISL_553323, EPI_ISL_553871, EPI_ISL_553879 to 553880, EPI_ISL_553885, EPI_ISL_553889, EPI_ISL_553893 to 553894, EPI_ISL_553896 to 553898, EPI_ISL_553905, EPI_ISL_553907, EPI_ISL_553929, EPI_ISL_553934, EPI_ISL_553939, EPI_ISL_553952, EPI_ISL_553954 to 553957, EPI_ISL_553965, EPI_ISL_553972, EPI_ISL_553983, EPI_ISL_553985, EPI_ISL_553987, EPI_ISL_553989, EPI_ISL_553993 to 553994, EPI_ISL_554003, EPI_ISL_554005, EPI_ISL_554009, EPI_ISL_554015, EPI_ISL_554024 to 554026, EPI_ISL_554028, EPI_ISL_554042, EPI_ISL_554048, EPI_ISL_554054, EPI_ISL_554057 to 554058, EPI_ISL_554065, EPI_ISL_554080, EPI_ISL_554097, EPI_ISL_554099 to 554101, EPI_ISL_554118 to 554119, EPI_ISL_554121, EPI_ISL_554123 to 554125, EPI_ISL_554127 to 554128, EPI_ISL_554132 to 554135, EPI_ISL_554137 to 554139, EPI_ISL_554141 to 554148, EPI_ISL_554150, EPI_ISL_554152 to 554154, EPI_ISL_554159 to 554163, EPI_ISL_554166 to 554169, EPI_ISL_554173, EPI_ISL_554177 to 554182, EPI_ISL_554184 to 554185, EPI_ISL_554191, EPI_ISL_554200 to 554201, EPI_ISL_554204 to 554205, EPI_ISL_554208, EPI_ISL_554210 to 554211, EPI_ISL_554214, EPI_ISL_554216 to 554218, EPI_ISL_554220 to 554221, EPI_ISL_554223 to 554224, EPI_ISL_554226, EPI_ISL_554228, EPI_ISL_554231 to 554232, EPI_ISL_554234, EPI_ISL_554236 to 554237, EPI_ISL_554239 to 554240, EPI_ISL_554242 to 554249, EPI_ISL_554251, EPI_ISL_554253 to 554256, EPI_ISL_554258 to 554259, EPI_ISL_554261 to 554262, EPI_ISL_554264, EPI_ISL_554267, EPI_ISL_554269, EPI_ISL_554271 to 554272, EPI_ISL_554274, EPI_ISL_554276 to 554277, EPI_ISL_554284 to 554287, EPI_ISL_554289 to 554291, EPI_ISL_554293 to 554295, EPI_ISL_554297, EPI_ISL_554300, EPI_ISL_554302 to 554304, EPI_ISL_554306 to 554308, EPI_ISL_554310 to 554317, EPI_ISL_554319, EPI_ISL_554619, EPI_ISL_554621 to 554622, EPI_ISL_554627 to 554629, EPI_ISL_554633 to 554639, EPI_ISL_554641, EPI_ISL_554643 to 554645, EPI_ISL_554647 to 554650, EPI_ISL_554652, EPI_ISL_554655, EPI_ISL_554658 to 554660, EPI_ISL_554665, EPI_ISL_554668 to 554676, EPI_ISL_554678 to 554679, EPI_ISL_554682 to 554685, EPI_ISL_554687, EPI_ISL_554690 to 554691, EPI_ISL_554693, EPI_ISL_554695, EPI_ISL_554698 to 554702, EPI_ISL_554704 to 554707, EPI_ISL_554711 to 554715, EPI_ISL_554717 to 554724, EPI_ISL_554728 to 554729, EPI_ISL_554731, EPI_ISL_554733 to 554734, EPI_ISL_554736 to 554740, EPI_ISL_554742, EPI_ISL_554744 to 554753, EPI_ISL_554758, EPI_ISL_554760, EPI_ISL_554762, EPI_ISL_554764 to 554766, EPI_ISL_554768 to 554770, EPI_ISL_554773 to 554775, EPI_ISL_554781, EPI_ISL_554783 to 554791, EPI_ISL_554793, EPI_ISL_554796, EPI_ISL_554800 to 554801, EPI_ISL_554803, EPI_ISL_554805 to 554806, EPI_ISL_554814, EPI_ISL_554817 to 554819, EPI_ISL_554821 to 554826, EPI_ISL_554828 to 554829, EPI_ISL_554831, EPI_ISL_554835, EPI_ISL_554838 to 554840, EPI_ISL_554842 to 554844, EPI_ISL_554849, EPI_ISL_554851 to 554852, EPI_ISL_554854 to 554855, EPI_ISL_554857 to 554860, EPI_ISL_555558, EPI_ISL_555616, EPI_ISL_556075 to 556077, EPI_ISL_556079, EPI_ISL_556083, EPI_ISL_556087, EPI_ISL_556090 to 556095, EPI_ISL_556097 to 556098, EPI_ISL_556102, EPI_ISL_556104, EPI_ISL_556109, EPI_ISL_556112, EPI_ISL_556115 to 556116, EPI_ISL_556123, EPI_ISL_556126, EPI_ISL_556133 to 556134, EPI_ISL_556138 to 556139, EPI_ISL_556146, EPI_ISL_556148, EPI_ISL_556150, EPI_ISL_556158, EPI_ISL_556163 to 556166, EPI_ISL_556170, EPI_ISL_556172, EPI_ISL_556176, EPI_ISL_556183, EPI_ISL_556186 to 556187, EPI_ISL_556195, EPI_ISL_556197 to 556199, EPI_ISL_556201, EPI_ISL_556204, EPI_ISL_556208, EPI_ISL_556211, EPI_ISL_556213 to 556214, EPI_ISL_556218, EPI_ISL_556223, EPI_ISL_556232 to 556233, EPI_ISL_556235, EPI_ISL_556237, EPI_ISL_556242, EPI_ISL_556244, EPI_ISL_556248, EPI_ISL_556251, EPI_ISL_556253, EPI_ISL_556259, EPI_ISL_556261, EPI_ISL_556263, EPI_ISL_556266 to 556267, EPI_ISL_556269, EPI_ISL_556275 to 556276, EPI_ISL_556282, EPI_ISL_556285, EPI_ISL_556287, EPI_ISL_556289, EPI_ISL_556291, EPI_ISL_556293 to 556294, EPI_ISL_556298, EPI_ISL_556300, EPI_ISL_556304, EPI_ISL_557166 to 557185, EPI_ISL_557187 to 557188, EPI_ISL_557190 to 557212, EPI_ISL_557214 to 557237, EPI_ISL_557239 to 557350, EPI_ISL_557352 to 557353, EPI_ISL_557357 to 557358, EPI_ISL_557360, EPI_ISL_557362, EPI_ISL_557364 to 557365, EPI_ISL_557367 to 557370, EPI_ISL_557372, EPI_ISL_557375 to 557377, EPI_ISL_557382, EPI_ISL_557384 to 557387, EPI_ISL_557389, EPI_ISL_557391 to 557397, EPI_ISL_557400 to 557403, EPI_ISL_557407, EPI_ISL_557409 to 557410, EPI_ISL_557413, EPI_ISL_557416, EPI_ISL_557418, EPI_ISL_557420 to 557422, EPI_ISL_557424, EPI_ISL_557426 to 557427, EPI_ISL_557429, EPI_ISL_557431 to 557432, EPI_ISL_557435, EPI_ISL_557437 to 557438, EPI_ISL_557441 to 557445, EPI_ISL_557448, EPI_ISL_557450 to 557451, EPI_ISL_557455 to 557465, EPI_ISL_557467, EPI_ISL_557470, EPI_ISL_557472 to 557473, EPI_ISL_557475, EPI_ISL_557477 to 557480, EPI_ISL_557483, EPI_ISL_557486, EPI_ISL_557489 to 557490, EPI_ISL_557492, EPI_ISL_557494 to 557496, EPI_ISL_557498, EPI_ISL_557501 to 557503, EPI_ISL_557505 to 557506, EPI_ISL_557508 to 557510, EPI_ISL_557512 to 557517, EPI_ISL_557519 to 557521, EPI_ISL_557524 to 557525, EPI_ISL_557529, EPI_ISL_557532, EPI_ISL_557534 to 557536, EPI_ISL_557538, EPI_ISL_557540, EPI_ISL_557542, EPI_ISL_557545 to 557550, EPI_ISL_557552, EPI_ISL_557701, EPI_ISL_557740 to 557742, EPI_ISL_557745, EPI_ISL_557748, EPI_ISL_557750, EPI_ISL_557753, EPI_ISL_557757 to 557758, EPI_ISL_557764 to 557766, EPI_ISL_557768, EPI_ISL_557771, EPI_ISL_557775, EPI_ISL_557778, EPI_ISL_557781 to 557782, EPI_ISL_557784, EPI_ISL_557786, EPI_ISL_557789, EPI_ISL_557791, EPI_ISL_557793, EPI_ISL_557795 to 557796, EPI_ISL_557800, EPI_ISL_557802, EPI_ISL_557807 to 557808, EPI_ISL_557811 to 557812, EPI_ISL_557816 to 557817, EPI_ISL_557821 to 557824, EPI_ISL_557827 to 557830, EPI_ISL_557832, EPI_ISL_557838, EPI_ISL_557845, EPI_ISL_557847 to 557849, EPI_ISL_557853 to 557854, EPI_ISL_557858, EPI_ISL_557865 to 557870, EPI_ISL_557876 to 557880, EPI_ISL_557884, EPI_ISL_557889 to 557890, EPI_ISL_557893 to 557895, EPI_ISL_557898, EPI_ISL_557905, EPI_ISL_557907 to 557909, EPI_ISL_557911, EPI_ISL_557915, EPI_ISL_557917, EPI_ISL_557919, EPI_ISL_557921, EPI_ISL_557925 to 557927, EPI_ISL_557933, EPI_ISL_557937 to 557938, EPI_ISL_557940 to 557942, EPI_ISL_557944, EPI_ISL_557947 to 557949, EPI_ISL_557953 to 557954, EPI_ISL_557956, EPI_ISL_557958 to 557959, EPI_ISL_557963 to 557965, EPI_ISL_557967, EPI_ISL_557970, EPI_ISL_557972, EPI_ISL_557974, EPI_ISL_557976 to 557977, EPI_ISL_557979, EPI_ISL_557984, EPI_ISL_557989, EPI_ISL_558001, EPI_ISL_558021, EPI_ISL_558026, EPI_ISL_558028, EPI_ISL_558030, EPI_ISL_558037, EPI_ISL_558041, EPI_ISL_558058, EPI_ISL_558068, EPI_ISL_558089, EPI_ISL_558096, EPI_ISL_558103, EPI_ISL_558106, EPI_ISL_558127, EPI_ISL_558155, EPI_ISL_558195, EPI_ISL_558234, EPI_ISL_558239, EPI_ISL_558241 to 558243, EPI_ISL_558245 to 558255, EPI_ISL_558257 to 558258,

EPI_ISL_558262, EPI_ISL_558264 to 558267, EPI_ISL_558271 to 558273, EPI_ISL_558275, EPI_ISL_558278 to 558282, EPI_ISL_558284 to 558287, EPI_ISL_558290, EPI_ISL_558292 to 558306, EPI_ISL_558308 to 558310, EPI_ISL_558313 to 558315, EPI_ISL_558317 to 558320, EPI_ISL_558322 to 558323, EPI_ISL_558325 to 558327, EPI_ISL_558330 to 558331, EPI_ISL_558333 to 558334, EPI_ISL_558336 to 558337, EPI_ISL_558339 to 558342, EPI_ISL_558344, EPI_ISL_558346 to 558347, EPI_ISL_558349, EPI_ISL_558351 to 558353, EPI_ISL_558356 to 558361, EPI_ISL_558363, EPI_ISL_558365 to 558367, EPI_ISL_558369 to 558378, EPI_ISL_558380 to 558386, EPI_ISL_558388, EPI_ISL_558390 to 558393, EPI_ISL_558397 to 558403, EPI_ISL_558638, EPI_ISL_558731, EPI_ISL_558903, EPI_ISL_558914, EPI_ISL_558920, EPI_ISL_558923, EPI_ISL_558925 to 558926, EPI_ISL_558928, EPI_ISL_558932, EPI_ISL_558934, EPI_ISL_558937, EPI_ISL_558949, EPI_ISL_558951, EPI_ISL_558959, EPI_ISL_558965, EPI_ISL_558970, EPI_ISL_558973, EPI_ISL_558975, EPI_ISL_558980 to 558981, EPI_ISL_558987, EPI_ISL_558993, EPI_ISL_559000, EPI_ISL_559005, EPI_ISL_559009, EPI_ISL_559011, EPI_ISL_559022, EPI_ISL_559026, EPI_ISL_559028, EPI_ISL_559043, EPI_ISL_559047, EPI_ISL_559049, EPI_ISL_559057, EPI_ISL_559061, EPI_ISL_559063, EPI_ISL_559071, EPI_ISL_559080 to 559081, EPI_ISL_559083 to 559084, EPI_ISL_559088, EPI_ISL_559100, EPI_ISL_559104, EPI_ISL_559106, EPI_ISL_559117, EPI_ISL_559119, EPI_ISL_559125 to 559126, EPI_ISL_559131 to 559133, EPI_ISL_559136, EPI_ISL_559147, EPI_ISL_559149, EPI_ISL_559154, EPI_ISL_559157 to 559158, EPI_ISL_559163 to 559164, EPI_ISL_559166 to 559167, EPI_ISL_559171, EPI_ISL_559174, EPI_ISL_559181, EPI_ISL_559187, EPI_ISL_559194 to 559195, EPI_ISL_559201, EPI_ISL_559204, EPI_ISL_559207, EPI_ISL_559215, EPI_ISL_559218, EPI_ISL_559223, EPI_ISL_559232, EPI_ISL_559235 to 559236, EPI_ISL_559241, EPI_ISL_559244, EPI_ISL_559246 to 559247, EPI_ISL_559253 to 559254, EPI_ISL_559256, EPI_ISL_559259, EPI_ISL_559263, EPI_ISL_559277 to 559278, EPI_ISL_559283 to 559284, EPI_ISL_559290, EPI_ISL_559296, EPI_ISL_559300, EPI_ISL_559312, EPI_ISL_559314, EPI_ISL_559319 to 559320, EPI_ISL_559326, EPI_ISL_559331, EPI_ISL_559335, EPI_ISL_559352, EPI_ISL_559354 to 559355, EPI_ISL_559368, EPI_ISL_559662 to 559669, EPI_ISL_559671 to 559705, EPI_ISL_559707 to 559723, EPI_ISL_559725 to 559732, EPI_ISL_559735 to 559739, EPI_ISL_559741 to 559752, EPI_ISL_566421 to 566422, EPI_ISL_566424 to 566427, EPI_ISL_566430 to 566443, EPI_ISL_566446 to 566452, EPI_ISL_566454, EPI_ISL_566458 to 566466, EPI_ISL_566468 to 566472, EPI_ISL_566475 to 566491, EPI_ISL_566494, EPI_ISL_566496 to 566499, EPI_ISL_566501 to 566507, EPI_ISL_566509 to 566517, EPI_ISL_566520 to 566523, EPI_ISL_566525 to 566527, EPI_ISL_566529 to 566533, EPI_ISL_566535 to 566536, EPI_ISL_566538 to 566542, EPI_ISL_566545 to 566566, EPI_ISL_566568 to 566575, EPI_ISL_566577 to 566585, EPI_ISL_566587 to 566588, EPI_ISL_566590 to 566609, EPI_ISL_566612 to 566624, EPI_ISL_566626 to 566630, EPI_ISL_566632 to 566637, EPI_ISL_566639 to 566658, EPI_ISL_566660, EPI_ISL_566663 to 566665, EPI_ISL_566667 to 566671, EPI_ISL_566673, EPI_ISL_566675 to 566950, EPI_ISL_566952 to 566985, EPI_ISL_566987 to 566997, EPI_ISL_568289 to 568290, EPI_ISL_568364 to 568365, EPI_ISL_568386 to 568387, EPI_ISL_568395, EPI_ISL_568466, EPI_ISL_568469 to 568471, EPI_ISL_568476, EPI_ISL_575367, EPI_ISL_575761 to 575981, EPI_ISL_575984 to 575994, EPI_ISL_576094, EPI_ISL_580631 to 580632, EPI_ISL_580634, EPI_ISL_580637 to 580639, EPI_ISL_580641 to 580644, EPI_ISL_580646 to 580647, EPI_ISL_580649, EPI_ISL_580652 to 580654, EPI_ISL_580656 to 580659, EPI_ISL_580661 to 580662, EPI_ISL_580664 to 580673, EPI_ISL_580676, EPI_ISL_580683 to 580685, EPI_ISL_580687 to 580688, EPI_ISL_580693 to 580694, EPI_ISL_580698, EPI_ISL_580700 to 580705, EPI_ISL_580707, EPI_ISL_580709, EPI_ISL_580711 to 580719, EPI_ISL_580721, EPI_ISL_580724 to 580729, EPI_ISL_580731 to 580733, EPI_ISL_580735 to 580736, EPI_ISL_580739, EPI_ISL_580741 to 580753, EPI_ISL_580755 to 580762, EPI_ISL_580765 to 580768, EPI_ISL_580770, EPI_ISL_580772 to 580779, EPI_ISL_580781 to 580783, EPI_ISL_580785 to 580786, EPI_ISL_580790, EPI_ISL_580792 to 580820, EPI_ISL_580826 to 580827, EPI_ISL_580829 to 580832, EPI_ISL_580834, EPI_ISL_580836 to 580839, EPI_ISL_580841 to 580848, EPI_ISL_580850, EPI_ISL_580852, EPI_ISL_580854 to 580857, EPI_ISL_580861, EPI_ISL_580864, EPI_ISL_580873, EPI_ISL_580875, EPI_ISL_580877, EPI_ISL_580882, EPI_ISL_580885, EPI_ISL_580892, EPI_ISL_580896 to 580898, EPI_ISL_580900, EPI_ISL_580902 to 580904, EPI_ISL_580910, EPI_ISL_580913, EPI_ISL_580915, EPI_ISL_580917 to 580918, EPI_ISL_580921, EPI_ISL_580929, EPI_ISL_580935, EPI_ISL_580940, EPI_ISL_580948, EPI_ISL_580950 to 580951, EPI_ISL_580959, EPI_ISL_580961, EPI_ISL_580976, EPI_ISL_580979 to 580980, EPI_ISL_580990 to 580991, EPI_ISL_580993, EPI_ISL_580995 to 580996, EPI_ISL_580998, EPI_ISL_581000, EPI_ISL_581004, EPI_ISL_581008 to 581009, EPI_ISL_581018, EPI_ISL_581023, EPI_ISL_581025 to 581026, EPI_ISL_581028 to 581029, EPI_ISL_581032, EPI_ISL_581035, EPI_ISL_581038, EPI_ISL_581040 to 581041, EPI_ISL_581046, EPI_ISL_581054, EPI_ISL_581056, EPI_ISL_581058, EPI_ISL_581064 to 581065, EPI_ISL_581067, EPI_ISL_581069, EPI_ISL_581074, EPI_ISL_581076, EPI_ISL_581078, EPI_ISL_581084, EPI_ISL_581086, EPI_ISL_581093, EPI_ISL_581097, EPI_ISL_581099, EPI_ISL_581103, EPI_ISL_581106 to 581107, EPI_ISL_581111, EPI_ISL_581113 to 581126, EPI_ISL_581128 to 581131, EPI_ISL_581134 to 581140, EPI_ISL_581142 to 581145, EPI_ISL_581147 to 581148, EPI_ISL_581150 to 581153, EPI_ISL_581155 to 581165, EPI_ISL_581167 to 581173, EPI_ISL_581175 to 581181, EPI_ISL_581184 to 581188, EPI_ISL_581190 to 581192, EPI_ISL_581195, EPI_ISL_581197 to 581213, EPI_ISL_581215 to 581216, EPI_ISL_581218 to 581222, EPI_ISL_581224 to 581225, EPI_ISL_581227 to 581228, EPI_ISL_581231 to 581244, EPI_ISL_581246, EPI_ISL_581248 to 581260, EPI_ISL_581263 to 581266, EPI_ISL_581268 to 581284, EPI_ISL_581286 to 581290, EPI_ISL_581292 to 581309, EPI_ISL_581311 to 581322, EPI_ISL_581325 to 581328, EPI_ISL_581330 to 581333, EPI_ISL_581335 to 581336, EPI_ISL_581338 to 581340, EPI_ISL_581342 to 581343, EPI_ISL_581345, EPI_ISL_581347 to 581349, EPI_ISL_581351 to 581353, EPI_ISL_581355 to 581357, EPI_ISL_581360 to 581365, EPI_ISL_581368 to 581376, EPI_ISL_581382 to 581383, EPI_ISL_581385 to 581386, EPI_ISL_581388 to 581390, EPI_ISL_581393, EPI_ISL_581397 to 581401, EPI_ISL_581415, EPI_ISL_581421 to 581422, EPI_ISL_581428, EPI_ISL_581434, EPI_ISL_581438, EPI_ISL_581440, EPI_ISL_581447, EPI_ISL_589017 to 589019, EPI_ISL_589021, EPI_ISL_589024, EPI_ISL_589026, EPI_ISL_589028 to 589030, EPI_ISL_589034, EPI_ISL_589039 to 589041, EPI_ISL_589043, EPI_ISL_589051, EPI_ISL_589057, EPI_ISL_589063, EPI_ISL_589067, EPI_ISL_589070, EPI_ISL_589074 to 589075, EPI_ISL_589077, EPI_ISL_589079 to 589081, EPI_ISL_589083, EPI_ISL_589086, EPI_ISL_589090 to 589091, EPI_ISL_589095, EPI_ISL_589097 to 589099, EPI_ISL_589101, EPI_ISL_589103 to 589104, EPI_ISL_589108 to 589111, EPI_ISL_589114 to 589115, EPI_ISL_589117 to 589118, EPI_ISL_589121 to 589123, EPI_ISL_589127, EPI_ISL_589133, EPI_ISL_589135, EPI_ISL_589137, EPI_ISL_589147, EPI_ISL_589153, EPI_ISL_589157, EPI_ISL_589160 to 589161, EPI_ISL_589166 to 589174, EPI_ISL_589176, EPI_ISL_589179, EPI_ISL_589182 to 589183, EPI_ISL_589185, EPI_ISL_589187, EPI_ISL_589195 to 589196, EPI_ISL_589198 to 589200, EPI_ISL_589205, EPI_ISL_589211 to 589212, EPI_ISL_589221, EPI_ISL_589224 to 589227, EPI_ISL_589229, EPI_ISL_589231, EPI_ISL_589233, EPI_ISL_589235, EPI_ISL_589237, EPI_ISL_589242 to 589243, EPI_ISL_589247, EPI_ISL_589251, EPI_ISL_589253, EPI_ISL_589256, EPI_ISL_589259 to 589261, EPI_ISL_589264 to 589266, EPI_ISL_589297, EPI_ISL_589304, EPI_ISL_589335, EPI_ISL_589355, EPI_ISL_589377, EPI_ISL_589407, EPI_ISL_589417, EPI_ISL_589423, EPI_ISL_589430, EPI_ISL_589440, EPI_ISL_589493, EPI_ISL_589519 to 589521, EPI_ISL_589526, EPI_ISL_589572 to 589761, EPI_ISL_589763 to 589764, EPI_ISL_589766 to 589773, EPI_ISL_589787, EPI_ISL_589789, EPI_ISL_589791 to 589792, EPI_ISL_589794 to 589801, EPI_ISL_590172, EPI_ISL_590174 to 590175, EPI_ISL_598250 to 598304, EPI_ISL_598306 to 598329, EPI_ISL_598332 to 598360, EPI_ISL_598362 to 598407, EPI_ISL_598409 to 598432, EPI_ISL_598434 to 598435, EPI_ISL_598437 to 598605, EPI_ISL_598607 to 598637, EPI_ISL_598639 to 598666, EPI_ISL_598668 to 598820, EPI_ISL_598822 to 598823, EPI_ISL_599818 to 599844, EPI_ISL_599846 to 599887, EPI_ISL_599889 to 599947, EPI_ISL_599949 to 599959, EPI_ISL_599961 to 600055, EPI_ISL_600057 to 600127, EPI_ISL_600892, EPI_ISL_600901, EPI_ISL_600912 to 600913, EPI_ISL_600915, EPI_ISL_600923, EPI_ISL_600925 to 600931, EPI_ISL_601224, EPI_ISL_601234 to 601235, EPI_ISL_601239 to 601240, EPI_ISL_601242 to 601243, EPI_ISL_601247 to 601249, EPI_ISL_601256, EPI_ISL_601258, EPI_ISL_601263 to 601264, EPI_ISL_601267 to 601268, EPI_ISL_601270, EPI_ISL_601272 to 601273, EPI_ISL_601275, EPI_ISL_601277 to 601278, EPI_ISL_601280 to 601283, EPI_ISL_601287, EPI_ISL_601289, EPI_ISL_601291 to 601294, EPI_ISL_601297 to 601299, EPI_ISL_601301 to 601307, EPI_ISL_601310 to 601314, EPI_ISL_601316 to 601317, EPI_ISL_601320 to 601322, EPI_ISL_601324 to 601327, EPI_ISL_601329 to 601330, EPI_ISL_601337 to 601338, EPI_ISL_601340, EPI_ISL_601343, EPI_ISL_601345 to 601346, EPI_ISL_601349 to 601350, EPI_ISL_601354, EPI_ISL_601359 to 601360, EPI_ISL_601363, EPI_ISL_601365, EPI_ISL_601367 to 601371, EPI_ISL_601373, EPI_ISL_601375 to 601376, EPI_ISL_601378 to 601379, EPI_ISL_601381 to 601383, EPI_ISL_601385, EPI_ISL_601388, EPI_ISL_601390, EPI_ISL_601392, EPI_ISL_601394 to 601398, EPI_ISL_601404, EPI_ISL_601406, EPI_ISL_601409 to 601410, EPI_ISL_601412, EPI_ISL_601415, EPI_ISL_601417 to 601420, EPI_ISL_601422, EPI_ISL_601427 to 601428, EPI_ISL_601431, EPI_ISL_601434, EPI_ISL_601437 to 601444, EPI_ISL_601447 to 601452, EPI_ISL_601455 to 601458, EPI_ISL_601463 to 601465, EPI_ISL_601468, EPI_ISL_601470, EPI_ISL_601472 to 601476, EPI_ISL_601478, EPI_ISL_601480, EPI_ISL_601484 to 601485, EPI_ISL_601487, EPI_ISL_601490 to 601492, EPI_ISL_601494, EPI_ISL_601496 to 601498, EPI_ISL_601500, EPI_ISL_601502 to 601505, EPI_ISL_601507 to 601514, EPI_ISL_601516 to 601521, EPI_ISL_601523 to 601529, EPI_ISL_601531 to 601540, EPI_ISL_601542 to 601546, EPI_ISL_601548, EPI_ISL_601551 to 601554, EPI_ISL_601556 to 601566, EPI_ISL_601568, EPI_ISL_601570, EPI_ISL_601572 to 601577, EPI_ISL_601581 to 601584, EPI_ISL_601586 to 601587, EPI_ISL_601592 to 601593, EPI_ISL_601595 to 601603, EPI_ISL_601605, EPI_ISL_601607 to 601610, EPI_ISL_601612 to 601614, EPI_ISL_601616, EPI_ISL_601620 to 601621, EPI_ISL_601623, EPI_ISL_601625 to 601626, EPI_ISL_601629 to 601633, EPI_ISL_601635 to 601638, EPI_ISL_601640, EPI_ISL_601642 to 601647, EPI_ISL_601649, EPI_ISL_601651, EPI_ISL_601653 to 601656, EPI_ISL_601658 to 601662, EPI_ISL_601665, EPI_ISL_601669 to 601672, EPI_ISL_601674 to 601681, EPI_ISL_601683 to 601684, EPI_ISL_601686 to 601689, EPI_ISL_601692 to 601699, EPI_ISL_601701 to 601703, EPI_ISL_601705 to 601708, EPI_ISL_601710 to 601714, EPI_ISL_601716 to 601727, EPI_ISL_601729, EPI_ISL_601731 to 601732, EPI_ISL_601734 to 601739, EPI_ISL_601741 to 601746, EPI_ISL_601749 to 601763, EPI_ISL_601767, EPI_ISL_601769 to 601770, EPI_ISL_601772 to 601777, EPI_ISL_601779 to 601782, EPI_ISL_601784, EPI_ISL_601786, EPI_ISL_602027 to 602029, EPI_ISL_602145 to 602153, EPI_ISL_606296 to 606310, EPI_ISL_606312 to 606313, EPI_ISL_606315 to 606352, EPI_ISL_606354 to 606401, EPI_ISL_606403 to 606414, EPI_ISL_606416 to 606461, EPI_ISL_606463 to 606515, EPI_ISL_606517 to 606523, EPI_ISL_606525 to 606587, EPI_ISL_606589 to 606607, EPI_ISL_606609 to 606653, EPI_ISL_606655 to 606796, EPI_ISL_606798 to 606923, EPI_ISL_607291 to 607447, EPI_ISL_607449 to 607460, EPI_ISL_607462 to 607515, EPI_ISL_607517 to 607549, EPI_ISL_607551 to 607571, EPI_ISL_607573 to 607578, EPI_ISL_607580 to 607586, EPI_ISL_607588 to 607622, EPI_ISL_608697 to 608747, EPI_ISL_608749 to 608833, EPI_ISL_608835 to 608844, EPI_ISL_608846, EPI_ISL_608848, EPI_ISL_608850, EPI_ISL_608854, EPI_ISL_608857, EPI_ISL_608859, EPI_ISL_608861, EPI_ISL_608870 to 608871, EPI_ISL_608882, EPI_ISL_608893 to 608894, EPI_ISL_608901, EPI_ISL_608905, EPI_ISL_608936, EPI_ISL_608940, EPI_ISL_608951, EPI_ISL_608954, EPI_ISL_608975, EPI_ISL_608977 to 608978, EPI_ISL_608986, EPI_ISL_609001, EPI_ISL_609006, EPI_ISL_609008, EPI_ISL_609015, EPI_ISL_609017, EPI_ISL_609021, EPI_ISL_609024, EPI_ISL_609026 to 609027, EPI_ISL_609029 to 609030, EPI_ISL_609036, EPI_ISL_609038, EPI_ISL_609042, EPI_ISL_609044, EPI_ISL_609046, EPI_ISL_609049, EPI_ISL_609058 to 609059, EPI_ISL_609062 to 609063, EPI_ISL_609066, EPI_ISL_609071, EPI_ISL_609076, EPI_ISL_609084, EPI_ISL_609108, EPI_ISL_609110, EPI_ISL_609115 to 609117, EPI_ISL_609126, EPI_ISL_609128, EPI_ISL_609136 to 609140, EPI_ISL_609149, EPI_ISL_609151, EPI_ISL_609155, EPI_ISL_609219 to 609229, EPI_ISL_609231 to 609266, EPI_ISL_609268 to 609301, EPI_ISL_609303 to 609307			
see above	Lighthouse Lab in Milton Keynes	Wellcome Sanger Institute for the COVID-19 Genomics UK (COG-UK) consortium	Cordelia Langford; David K. Jackson; Dominic Kwiatkowski; Ewan Harrison; Ian Johnston; John Sillitoe on behalf of the Wellcome Sanger Institute COVID-19 Surveillance Team; John Sillitoe on behalf of the Wellcome Sanger Institute COVID-19 Surveillance Team (http://www.sanger.ac.uk/covid-team); Roberto Amato; Sonia Goncalves; The Lighthouse Lab in Alderley Park and Alex Alderton; The Lighthouse Lab in Milton Keynes and Alex Alderton
EPI_ISL_1440951 to 1440959, EPI_ISL_1440961 to 1440966	Limbach - Ganzimmun Diagnostics AG Mainz	Robert Koch Institute	
EPI_ISL_1435303, EPI_ISL_1435308, EPI_ISL_1435313, EPI_ISL_1435315 to 1435316, EPI_ISL_1435321, EPI_ISL_1435324 to 1435325, EPI_ISL_1435327, EPI_ISL_1435332, EPI_ISL_1438635, EPI_ISL_1438672, EPI_ISL_1441196, EPI_ISL_1441206, EPI_ISL_1441214, EPI_ISL_1441218, EPI_ISL_1441220			
see above	Limbach - MVZ Gemeinschaftslabor Suhl	Robert Koch Institute	
EPI_ISL_1436395 to 1436433, EPI_ISL_1436435 to 1436471, EPI_ISL_1436473 to 1436494, EPI_ISL_1436496 to 1436501, EPI_ISL_1436503 to 1436528, EPI_ISL_1436530 to 1436579	Limbach - MVZ Humangenetik Ulm	Robert Koch Institute	
EPI_ISL_1433597, EPI_ISL_1433608, EPI_ISL_1433612, EPI_ISL_1433614, EPI_ISL_1433617, EPI_ISL_1433625, EPI_ISL_1433638, EPI_ISL_1433646, EPI_ISL_1433651, EPI_ISL_1433657, EPI_ISL_1438605, EPI_ISL_1438624, EPI_ISL_1438639, EPI_ISL_1438664, EPI_ISL_1438669, EPI_ISL_1439520, EPI_ISL_1439522, EPI_ISL_1439524, EPI_ISL_1441649, EPI_ISL_1441651, EPI_ISL_1441653 to 1441655, EPI_ISL_1441657 to 1441658, EPI_ISL_1441661, EPI_ISL_1441663 to 1441665, EPI_ISL_1441667 to 1441668, EPI_ISL_1441673 to 1441674, EPI_ISL_1441676 to 1441677, EPI_ISL_1441679 to 1441681			
see above	Limbach - MVZ Labor Eveld & Kollegen Essen	Robert Koch Institute	
EPI_ISL_1441650	Limbach - MVZ Labor Ludwigsburg	Robert Koch Institute	
EPI_ISL_484354 to 484373, EPI_ISL_484392 to 484406, EPI_ISL_490563 to 490580, EPI_ISL_493549 to 493600, EPI_ISL_493603 to 493605, EPI_ISL_493610 to 493613, EPI_ISL_526431 to 526432, EPI_ISL_549352, EPI_ISL_549356, EPI_ISL_549362, EPI_ISL_549365, EPI_ISL_549371 to 549372, EPI_ISL_549375, EPI_ISL_549381, EPI_ISL_549387, EPI_ISL_549517 to 549522, EPI_ISL_572507 to 572508, EPI_ISL_572510, EPI_ISL_572527, EPI_ISL_572705, EPI_ISL_572938, EPI_ISL_572943 to 572944, EPI_ISL_573437 to 573449, EPI_ISL_1387196 to 1387211, EPI_ISL_1475495 to 1475542			
see above	Lincolnshire Hospitals and DeepSeq Nottingham	COVID-19 Genomics UK (COG-UK) Consortium	Christopher Moore; Fei Sang; Joeseph Chappell; Johnny Debebe; Jonathan Ball; Matthew Carlisle; Matthew Loose; Nadine Holmes; Nichola Duckworth; Patrick McClure; Sarah Walsh; Tim Sloan; Victoria Wright
EPI_ISL_541847 to 541877	Lithuanian University of Health Sciences Hospital, Department of Laboratory Medicine	Lithuanian University of Health Sciences, Laboratory of Molecular Cardiology	Arnoldas Pautienius; Astra Vitkauskiene; Dovydas Gecys; Kamile Tamusauskaite; Laura Pareckaite; Lukas Zemaitis; Vaiva Lesauskaite
EPI_ISL_536398, EPI_ISL_603082 to 603122	Lithuanian University of Health Sciences Hospital, Department of Laboratory Medicine	Lithuanian University of Health Sciences, Molecular cardiology lab.	Arnoldas Pautienius; Astra Vitkauskiene; Dovydas Gecys; Ingrida Olendrait; Kamile Tamusauskaite; Laura Pareckaite; Lukas Zemaitis; Vaiva Lesauskaite
EPI_ISL_439672 to 439862, EPI_ISL_440852 to 440950, EPI_ISL_449330 to 449475, EPI_ISL_449488 to 449625, EPI_ISL_453195 to 453459, EPI_ISL_457259 to 457269, EPI_ISL_471993 to 472135, EPI_ISL_478372 to 478403, EPI_ISL_484254 to 484263, EPI_ISL_490331 to 490420, EPI_ISL_499460 to 499471, EPI_ISL_499473, EPI_ISL_499475 to 499477, EPI_ISL_499480 to 499481, EPI_ISL_499483 to 499484, EPI_ISL_499486 to 499489, EPI_ISL_499491 to 499492, EPI_ISL_499496 to 499501, EPI_ISL_499509, EPI_ISL_499511 to 499514, EPI_ISL_499516 to 499545, EPI_ISL_499557, EPI_ISL_499564 to 499567, EPI_ISL_499569 to 499571, EPI_ISL_499573 to 499586, EPI_ISL_499589 to 499590, EPI_ISL_499593 to 499594, EPI_ISL_499600, EPI_ISL_499604 to 499605, EPI_ISL_499607 to 499608, EPI_ISL_499611 to 499622, EPI_ISL_499624 to 499625, EPI_ISL_499627 to 499630, EPI_ISL_499632 to 499776, EPI_ISL_499811 to 499827, EPI_ISL_499831 to 499838, EPI_ISL_499840 to 499850, EPI_ISL_499854, EPI_ISL_499856 to 499857, EPI_ISL_499859 to 499860, EPI_ISL_499862 to 499868, EPI_ISL_499870 to 499893, EPI_ISL_499896, EPI_ISL_499898 to 499904, EPI_ISL_499906 to 499907, EPI_ISL_499909, EPI_ISL_499911, EPI_ISL_499914 to 499932, EPI_ISL_499934 to 499946, EPI_ISL_499948 to 499949, EPI_ISL_499951 to 499965, EPI_ISL_499967, EPI_ISL_499984 to 500156, EPI_ISL_512342 to 512347, EPI_ISL_517001 to 517509, EPI_ISL_526367 to 526394, EPI_ISL_529254, EPI_ISL_529258 to 529260, EPI_ISL_529263, EPI_ISL_529270 to 529271, EPI_ISL_529274, EPI_ISL_529280, EPI_ISL_529284, EPI_ISL_529287, EPI_ISL_529293, EPI_ISL_529295 to 529296, EPI_ISL_529302, EPI_ISL_529304 to 529305, EPI_ISL_529466, EPI_ISL_529484, EPI_ISL_529490, EPI_ISL_529498, EPI_ISL_529509 to 529510, EPI_ISL_529513, EPI_ISL_529516, EPI_ISL_534732 to 534755, EPI_ISL_540584 to 540639, EPI_ISL_549354, EPI_ISL_549358, EPI_ISL_572402, EPI_ISL_572404, EPI_ISL_572426, EPI_ISL_572429, EPI_ISL_572431, EPI_ISL_572436, EPI_ISL_572443, EPI_ISL_572445, EPI_ISL_572451, EPI_ISL_572466, EPI_ISL_572473, EPI_ISL_572478 to 572479, EPI_ISL_572500, EPI_ISL_572502, EPI_ISL_572506, EPI_ISL_572511, EPI_ISL_572552, EPI_ISL_572554, EPI_ISL_572557, EPI_ISL_572562, EPI_ISL_572578, EPI_ISL_572584, EPI_ISL_572588, EPI_ISL_572601, EPI_ISL_572604, EPI_ISL_572650, EPI_ISL_572669, EPI_ISL_572677, EPI_ISL_572679, EPI_ISL_572682, EPI_ISL_572687 to 572688, EPI_ISL_572691, EPI_ISL_572695 to 572696, EPI_ISL_572710, EPI_ISL_572733 to 572734, EPI_ISL_572736 to 572740, EPI_ISL_572748, EPI_ISL_572750, EPI_ISL_572761, EPI_ISL_572769, EPI_ISL_572771, EPI_ISL_572773, EPI_ISL_572813, EPI_ISL_572815, EPI_ISL_572817, EPI_ISL_572822, EPI_ISL_572824, EPI_ISL_572827, EPI_ISL_572843, EPI_ISL_572904 to 572908, EPI_ISL_573236 to 573246, EPI_ISL_576961 to 576983, EPI_ISL_584591 to 584621, EPI_ISL_594849 to 594860, EPI_ISL_595799 to 595801, EPI_ISL_595822, EPI_ISL_611514 to 611517, EPI_ISL_611521 to 611522, EPI_ISL_611542 to 611543, EPI_ISL_611565, EPI_ISL_611567 to 611571, EPI_ISL_611575, EPI_ISL_611579 to 611580, EPI_ISL_611600, EPI_ISL_611603, EPI_ISL_611613, EPI_ISL_611621, EPI_ISL_611627, EPI_ISL_611629 to 611630, EPI_ISL_611632, EPI_ISL_611634 to 611635, EPI_ISL_611642, EPI_ISL_611653 to 611654, EPI_ISL_611658, EPI_ISL_611664, EPI_ISL_611667, EPI_ISL_611672, EPI_ISL_611675, EPI_ISL_611694 to 611695, EPI_ISL_611721, EPI_ISL_611723 to 611724, EPI_ISL_611727 to 611731, EPI_ISL_611734, EPI_ISL_611736 to 611737, EPI_ISL_611739, EPI_ISL_611741, EPI_ISL_611743, EPI_ISL_611750, EPI_ISL_611764 to 611765, EPI_ISL_611784 to			

611786, EPI_ISL_611790, EPI_ISL_611796, EPI_ISL_611811, EPI_ISL_611815, EPI_ISL_611821, EPI_ISL_611827, EPI_ISL_611845, EPI_ISL_611851, EPI_ISL_611853, EPI_ISL_611857, EPI_ISL_611885, EPI_ISL_611889, EPI_ISL_611891 to 611893, EPI_ISL_611895, EPI_ISL_611897 to 611899, EPI_ISL_611908, EPI_ISL_611910 to 611911, EPI_ISL_611914 to 611915, EPI_ISL_611918, EPI_ISL_611921, EPI_ISL_611929, EPI_ISL_611974, EPI_ISL_611977 to 611978, EPI_ISL_611981, EPI_ISL_611984 to 611985, EPI_ISL_611991, EPI_ISL_611995, EPI_ISL_611999, EPI_ISL_612001, EPI_ISL_612003, EPI_ISL_612009 to 612010, EPI_ISL_612016 to 612017, EPI_ISL_612019, EPI_ISL_612021, EPI_ISL_612024, EPI_ISL_612035 to 612036, EPI_ISL_612039, EPI_ISL_612041 to 612044, EPI_ISL_612046, EPI_ISL_612051, EPI_ISL_612059 to 612061, EPI_ISL_612064 to 612066, EPI_ISL_612070 to 612072, EPI_ISL_612075, EPI_ISL_612080, EPI_ISL_612088, EPI_ISL_612101, EPI_ISL_612108, EPI_ISL_612111 to 612112, EPI_ISL_612114 to 612115, EPI_ISL_612190 to 612191, EPI_ISL_612401 to 612449, EPI_ISL_1386729, EPI_ISL_1386732 to 1386734, EPI_ISL_1386736 to 1386745, EPI_ISL_1386759 to 1386786, EPI_ISL_1386789 to 1386801, EPI_ISL_1386803 to 1386812, EPI_ISL_1474654 to 1474668, EPI_ISL_1474670 to 1474684, EPI_ISL_1474686 to 1474689, EPI_ISL_1474691 to 1474692, EPI_ISL_1474694 to 1474699, EPI_ISL_1474701 to 1474714, EPI_ISL_1474716 to 1474733, EPI_ISL_1474735 to 1474738, EPI_ISL_1474740 to 1474744, EPI_ISL_1474747 to 1474748, EPI_ISL_1474750 to 1474799, EPI_ISL_1475751 to 1475758, EPI_ISL_1545984 to 1545996, EPI_ISL_1545998 to 1546004, EPI_ISL_1546006 to 1546011, EPI_ISL_1546013 to 1546043, EPI_ISL_1546045, EPI_ISL_1546047, EPI_ISL_1546049 to 1546050, EPI_ISL_1546052, EPI_ISL_1546054, EPI_ISL_1546056, EPI_ISL_1546058 to 1546059, EPI_ISL_1546061, EPI_ISL_1546063, EPI_ISL_1546065, EPI_ISL_1546067, EPI_ISL_1546069, EPI_ISL_1546071 to 1546072, EPI_ISL_1546076, EPI_ISL_1546078, EPI_ISL_1546080 to 1546081, EPI_ISL_1546083, EPI_ISL_1546085, EPI_ISL_1546092 to 1546093, EPI_ISL_1546095, EPI_ISL_1546097, EPI_ISL_1546099, EPI_ISL_1546101, EPI_ISL_1546103, EPI_ISL_1546105, EPI_ISL_1546107 to 1546108, EPI_ISL_1546110, EPI_ISL_1546112, EPI_ISL_1546115, EPI_ISL_1546118, EPI_ISL_1546120, EPI_ISL_1546122, EPI_ISL_1546124, EPI_ISL_1546129, EPI_ISL_1546131, EPI_ISL_1546133, EPI_ISL_1546135, EPI_ISL_1546137, EPI_ISL_1546139 to 1546228			
see above	Liverpool Clinical Laboratories	COVID-19 Genomics UK (COG-UK) Consortium	A Alrezaihi; Alessandro Gerada; Alistair Darby; Angela Cowell; Anita Lucaci; Anu Chawla; Cassie Olateju; Catherine Hartley; Charlotte Nelson; Ecaterina Vamos; Elaine O'Toole; Elaine O'ÄôToole; Eleanor G Bentley; Ghada T Shawli; Hermione Webster; Isabel García-Dorival; Isabel Garca-Dorival; James Johnson; James P Stewart; Jenifer Manson; Joanne Watts; Jones Benjamin; Jordan J Clark; Julian Hiscox; L Luu; Lucille Rainbow; M Almsaud; Margaret Hughes; Mark Whitehead; Matthew Gemmell; Miren Iturriza-Gomara; Muhannad Alruwaili; N.P Randle; Neil Swainston; PKF Gilmore; Parul Sharma; Rebekah Penrice-Randal; Rebekah Penrice-Randal¬†; Richard Eccles; Richard Gregory; Sam Haldenby; Steve Paterson; Stuart D Armstrong; Trevor Ian Robinson; Ximeng Han
EPI_ISL_1443202, EPI_ISL_1443246, EPI_ISL_1443291 to 1443292	Logan County Health Department	Illinois Department of Public Health - Springfield Lab	Bryan Sim; Gordon McCall
EPI_ISL_1500206 to 1500207	Logan County Health Dept	Illinois Department of Public Health - Springfield Lab	Bryan Sim; Gordon McCall
EPI_ISL_1497543, EPI_ISL_1503992, EPI_ISL_1503995, EPI_ISL_1504027, EPI_ISL_1508909	Los Angeles County PHL	Los Angeles County PHL	P. Hemarajata et al.
EPI_ISL_436044	Louisiana Office of Public Health Laboratories	Pathogen Discovery, Respiratory Viruses Branch, Division of Viral Diseases, Centers for Disease Control and Prevention	Anna Uehara; Bettina Bankamp; Clinton R. Paden; Haibin Wang; Jing Zhang; Krista Queen; Suxiang Tong; Yan Li; Ying Tao; Zachary Weiner
EPI_ISL_1495626	Ludwig Boltzmann Institut für Experimentelle und Klinische Traumatologie, Lorenz Böhler UKH	Bergthaler laboratory, CeMM Research Center for Molecular Medicine of the Austrian Academy of Sciences	Andreas Bergthaler; Anna Schedl; Bekir Erguner; Benedikt Agerer; Christoph Bock; Fabian Amman; Jan Laine; Lukas Endler; Maelle Le Moing; Martin Senekowitsch; Michael Schuster; Petr Triska; Thomas Penz
EPI_ISL_434658	Lundens VC	The Public Health Agency of Sweden	Anna Risberg; Anna-Malin Linde; Karin Tegmark-Wisell; Maria Lind Karlberg; Marita Dagner; Mia Brytting; Olov Svartstrom; Oskar Karlsson Lindsjo; Theresa Enkirch
EPI_ISL_430844	Lung Center of the Philippines	Research Institute for Tropical Medicine	Bautista; Brunker, K.; C.S.; C.T.; D.L.; Demetria; E.S.; F.G.M.; I.A.P.; Manalo; Medado; Mercado; O.J.T.; Onza; Polotan
EPI_ISL_1501694 to 1501717, EPI_ISL_1501720, EPI_ISL_1501723, EPI_ISL_1501726, EPI_ISL_1501729, EPI_ISL_1501732, EPI_ISL_1501735, EPI_ISL_1501738, EPI_ISL_1501741, EPI_ISL_1501744, EPI_ISL_1501747, EPI_ISL_1501749, EPI_ISL_1501753, EPI_ISL_1501756, EPI_ISL_1501758, EPI_ISL_1501761, EPI_ISL_1501764, EPI_ISL_1501767, EPI_ISL_1501770, EPI_ISL_1501773, EPI_ISL_1501776, EPI_ISL_1501779, EPI_ISL_1501782, EPI_ISL_1501785, EPI_ISL_1501788, EPI_ISL_1501791 to 1502051			
see above	Lurie Children's Hospital of Chicago	Northwestern University - Ozer Lab	Egon A. Ozer; Judd F. Hultquist; Lacy M. Simons; Larry K. Kociolek; Michael G. Ison; Ramon Lorenzo-Redondo; Taylor J. Dean; William J. Muller; Xiaotian; Zheng
EPI_ISL_470790 to 470800, EPI_ISL_477277 to 477290, EPI_ISL_481241, EPI_ISL_509686, EPI_ISL_514629 to 514635, EPI_ISL_515267 to 515268, EPI_ISL_530163 to 530168, EPI_ISL_534217, EPI_ISL_560798 to 560805, EPI_ISL_1396411, EPI_ISL_1402725, EPI_ISL_1402741 to 1402742, EPI_ISL_1402744 to 1402781, EPI_ISL_1403667 to 1403668, EPI_ISL_1423534 to 1423592, EPI_ISL_1445979, EPI_ISL_1445981, EPI_ISL_1445984 to 1445986, EPI_ISL_1445991, EPI_ISL_1445995, EPI_ISL_1445998, EPI_ISL_1446009 to 1446010, EPI_ISL_1446012, EPI_ISL_1446023 to 1446024, EPI_ISL_1446027, EPI_ISL_1446061 to 1446062, EPI_ISL_1446064 to 1446065, EPI_ISL_1446067 to 1446068, EPI_ISL_1446070, EPI_ISL_1446072 to 1446074, EPI_ISL_1446076, EPI_ISL_1446078 to 1446080, EPI_ISL_1479016 to 1479017, EPI_ISL_1479137 to 1479138, EPI_ISL_1499689 to 1499722, EPI_ISL_1527464, EPI_ISL_1527467, EPI_ISL_1527469, EPI_ISL_1527471, EPI_ISL_1527492 to 1527496, EPI_ISL_1527505, EPI_ISL_1527507, EPI_ISL_1527509, EPI_ISL_1527523 to 1527527, EPI_ISL_1531644 to 1531733			
see above	M Health Fairview	Minnesota Department of Health, Public Health Laboratory	Alexandra Lorentz; Jacob Garfin; Kelly Pung; Matt Plumb; and Xiong Wang
EPI_ISL_443183, EPI_ISL_443253 to 443257, EPI_ISL_445077	M Health Fairview	University of Minnesota Genomics Center	Andrew Nelson; Benjamin Auch; Daryl M. Gohl; Jerry Daniel; John Garbe; Patrick Grady; Ray Watson; Sophia Yohe; and Kenneth B. Beckman
EPI_ISL_514637, EPI_ISL_514643, EPI_ISL_514650, EPI_ISL_514652, EPI_ISL_514657	M Health Fairview St. Joseph's Hospital	Minnesota Department of Health, Public Health Laboratory	Jacob Garfin; Matt Plumb; and Xiong Wang
EPI_ISL_451149 to 451153, EPI_ISL_451666	M.P Shah Government Medocal college Jamnagar	Gujarat Biotechnology Research Centre	; A M Kadri; Afzal Ansari; Akanksha Verma; Amit Kanani; Ankit Hinsu; Apurvasinh Puvar; Bhavesh Modi; Binita Aring; Chaitanya Joshi; Dinesh Kumar; Fenil Patel; Gaurishankar Shrimali; Janvi Raval; Komal Patel; Labdhi Pandya; Madhvi Joshi; Maharshi Pandya; Monika Gandhi; Neha Rajpara; Nidhi Patel; Nitin Savaliya; Pinal Trivedi; Pragya Sharma; Pritesh Sabara; Priti Pandita; R D Dixit; Raghawendra Kumar; Ramesh Pandit; Snehal Bagatharia; Tejas Shah; Zarna Patel; Zuber Saiyed
EPI_ISL_1446992 to 1446998	MA State Public Health Laboratory	Centers for Disease Control and Prevention Division of Viral Diseases, Pathogen Discovery	Alison Laufer Halpin; Ben L. Rambo-Martin; Clinton R. Paden; Dakota Howard; Darlene Wagner; Dave Wentworth; Dhwani Batra; Jasmine Padilla; Justin Lee; Katie Dillon; Krista Queen; Kristen Knipe; Kristine Lacek; Mark Burroughs; Matthew Schmerer; Mili Sheth; Peter Cook; Sam Shepard; Sarah Nobles; Shoshona Le; Suxiang Tong; Vivien Dugan; Yvette Unoarumhi
EPI_ISL_424843 to 424847, EPI_ISL_424908 to 424920	MA State Public Health Laboratory	Pathogen Discovery, Respiratory Viruses Branch, Division of Viral Diseases, Centers for Disease Control and Prevention	Alison S. Laufer Halpin; Anna Uehara; Christopher A. Elkins; Clinton R. Paden; Haibin Wang; Jing Zhang; Krista Queen; Mary S. Keckler; Rachel Marine; Suxiang Tong; Yan Li; Ying Tao
EPI_ISL_1446630 to 1446631, EPI_ISL_1446859 to 1446860, EPI_ISL_1446862, EPI_ISL_1447052, EPI_ISL_1516322 to 1516342, EPI_ISL_1528451 to 1528459	MD DOH Laboratories Administration	Centers for Disease Control and Prevention Division of Viral Diseases, Pathogen Discovery	Alison Laufer Halpin; Ben L. Rambo-Martin; Clinton R. Paden; Dakota Howard; Darlene Wagner; Dave Wentworth; Dhwani Batra; Jasmine Padilla; Justin Lee; Katie Dillon; Krista Queen; Kristen Knipe; Kristine Lacek; Mark Burroughs; Matthew Schmerer; Mili Sheth; Peter Cook; Sam Shepard; Sarah Nobles; Shoshona Le; Suxiang Tong; Vivien Dugan; Yvette Unoarumhi
EPI_ISL_424869, EPI_ISL_426425, EPI_ISL_447843	MD DOH Laboratories Administration	Pathogen Discovery, Respiratory Viruses Branch, Division of Viral Diseases, Centers for Disease Control and Prevention	Alison S. Laufer Halpin; Anna Uehara; Christopher A. Elkins; Clinton R. Paden; Haibin Wang; Jasmine Padilla; Jing Zhang; Justin Lee; Krista Queen; Mary S. Keckler; Rachel Marine; Suxiang Tong; Yan Li; Ying Tao
EPI_ISL_1502804 to 1502809, EPI_ISL_1506117 to 1506119	MD Laboratories	Los Angeles County PHL	P. Hemarajata et al.
EPI_ISL_524873 to 524903, EPI_ISL_534699 to 534716, EPI_ISL_591022 to 591036, EPI_ISL_602258 to 602277, EPI_ISL_1493168 to 1493257, EPI_ISL_1493436 to 1493439	MD PHL	MD PHL	Maryland Department of Health Laboratories Administration
EPI_ISL_1434444, EPI_ISL_1434450, EPI_ISL_1434481, EPI_ISL_1434516, EPI_ISL_1435292, EPI_ISL_1435294 to 1435295, EPI_ISL_1435297, EPI_ISL_1435299, EPI_ISL_1435301 to 1435302, EPI_ISL_1435304 to 1435305, EPI_ISL_1435307, EPI_ISL_1435309, EPI_ISL_1435311 to 1435312, EPI_ISL_1435314, EPI_ISL_1435317 to 1435319, EPI_ISL_1435322 to 1435323, EPI_ISL_1435326, EPI_ISL_1435329 to 1435331, EPI_ISL_1435428 to 1435439, EPI_ISL_1435442 to 1435455, EPI_ISL_1435978, EPI_ISL_1435980, EPI_ISL_1435982, EPI_ISL_1435984, EPI_ISL_1435986, EPI_ISL_1435988, EPI_ISL_1435990, EPI_ISL_1435993 to 1435994, EPI_ISL_1435996, EPI_ISL_1435998, EPI_ISL_1436001, EPI_ISL_1438598, EPI_ISL_1438606, EPI_ISL_1438609, EPI_ISL_1438611, EPI_ISL_1438614, EPI_ISL_1438620, EPI_ISL_1438634, EPI_ISL_1438644, EPI_ISL_1438652, EPI_ISL_1438658, EPI_ISL_1438662, EPI_ISL_1438667, EPI_ISL_1438671, EPI_ISL_1439521, EPI_ISL_1441158 to 1441194, EPI_ISL_1441198 to 1441199, EPI_ISL_1441201, EPI_ISL_1441203 to 1441205, EPI_ISL_1441208 to 1441212, EPI_ISL_1441215 to 1441217, EPI_ISL_1441222 to 1441224, EPI_ISL_1441226, EPI_ISL_1441228 to 1441230, EPI_ISL_1441660, EPI_ISL_1441666, EPI_ISL_1441669, EPI_ISL_1441671, EPI_ISL_1441682 to 1441686, EPI_ISL_1441688 to 1441708, EPI_ISL_1441710 to 1441716			
see above	MDI Limbach Berlin GmbH	Robert Koch Institute	
EPI_ISL_594134 to 594147, EPI_ISL_603040 to 603048	MDU-PHL, The Peter Doherty Institute for Infection and Immunity	MDU-PHL, The Peter Doherty Institute for Infection and Immunity	Caly, L.; Druce, J.; M.B.; M.L.; N.L.; Sait; Sait, M.; Schultz; Seemann, T.; Sherry; Sherry, N.
EPI_ISL_1446594, EPI_ISL_1446598 to 1446600, EPI_ISL_1446803 to 1446806, EPI_ISL_1516256 to 1516261, EPI_ISL_1528363 to 1528364	ME Health and Environmental Testing Laboratory	Centers for Disease Control and Prevention Division of Viral Diseases, Pathogen Discovery	Alison Laufer Halpin; Ben L. Rambo-Martin; Clinton R. Paden; Dakota Howard; Darlene Wagner; Dave Wentworth; Dhwani Batra; Jasmine Padilla; Justin Lee; Katie Dillon; Krista Queen; Kristen Knipe; Kristine Lacek; Mark Burroughs; Matthew Schmerer; Mili Sheth; Peter Cook; Sam Shepard; Sarah Nobles; Shoshona Le; Suxiang Tong; Vivien Dugan; Yvette Unoarumhi

EPI_ISL_1408894, EPI_ISL_1509942	ME Health and Environmental Testing Laboratory	Genomics and Discovery, Respiratory Viruses Branch, Division of Viral Diseases, Centers for Disease Control and Prevention	Adam Retchless; Anna Kelleher; Anna Montmayeur; Anna Uehara; Brian Lynch; Clinton R. Paden; Haibin Wang; Han Jia Justin Ng; Jing Zhang; Justin Lee; Krista Queen; Mark Burroughs; Peter Cook; Rachel Marine; Suxiang Tong; Yan Li; Ying Tao
EPI_ISL_1403510, EPI_ISL_1403530 to 1403531	MEDICO COMPETENTE P.O. SULMONA - CASTEL DI SANGRO SULMONA(L'AQUILA)	Istituto Zooprofilattico Sperimentale dell'Abruzzo e Molise "G. Caporale"	Ancora M; Calistri P; Cammà C; Curini V; Delli Compagni E; Di Domenico M; Di Pasquale A; Lorusso A; Mangone I; Marcacci M; Puglia I; Rinaldi A; Savini G; Scialabba S
EPI_ISL_1464106 to 1464108, EPI_ISL_1464263 to 1464268, EPI_ISL_1464586 to 1464587, EPI_ISL_1464590	MEDILYS Laborgesellschaft mbH	Heinrich Pette Institute, Leibniz Institute for Experimental Virology	Adam Grundhoff; Alexis Robitaille; Johannes Knobloch; Martin Aepfelbacher; Nicole Fischer; Thomas Günther
EPI_ISL_1461077, EPI_ISL_1461079, EPI_ISL_1461081, EPI_ISL_1461083 to 1461084, EPI_ISL_1461086, EPI_ISL_1461088, EPI_ISL_1461090, EPI_ISL_1461093, EPI_ISL_1461095, EPI_ISL_1461097, EPI_ISL_1461099, EPI_ISL_1461102, EPI_ISL_1461104, EPI_ISL_1461106			
see above	MEDIPOLE CHALLES LES EAUX	CNR Virus des Infections Respiratoires - France SUD	Antonin Bal; Bruno Lina; Gregory Destras; Gwendolyne Burfin; Hadrien Regue; Laurence Josset; Martine Valette; Quentin Semanas
EPI_ISL_445311, EPI_ISL_445328	MEGASALUD S.A.	Instituto de Salud Publica de Chile	Alejandra Acevedo; Andrés E Castillo; Bárbara Parra; Carolina Tambley; Gabriel Leal; Jaime Lagos; Jorge Fernandez; Loredana Arata; Patricia Bustos; Paz Tapia; Rodrigo Fasce; Winston Andrade
EPI_ISL_445263, EPI_ISL_445358	MEGASALUD SPA.	Instituto de Salud Publica de Chile	Alejandra Acevedo; Andrés E Castillo; Bárbara Parra; Carolina Tambley; Gabriel Leal; Jaime Lagos; Jorge Fernandez; Loredana Arata; Patricia Bustos; Paz Tapia; Rodrigo Fasce; Winston Andrade
EPI_ISL_1395710 to 1395725, EPI_ISL_1397383 to 1397388, EPI_ISL_1398040 to 1398041, EPI_ISL_1398043	MEMORIAL SLOAN KETTERING CANCER CENTER	Wadsworth Center, New York State Department of Health	Alexis Russel; Daryl M. Lamson; Erasmus Schneider; Erica Lasek-Nesselquist; John Kelly; Jonathan Plitnick; Kirsten St. George; Matthew Shudt; Melissa A Leisner; Navjot Singh
EPI_ISL_568877 to 569608	MEPHI, Aix Marseille University	MEPHI, Aix Marseille University	Anthony LEVASSEUR
EPI_ISL_413568	MHC Drente	Erasmus Medical Center	Anne van der Linden; Annemiek van der Eijk; Aura Timen; Bas Oude Munnink; Claudia Schapendonk; Corien Swaan; Corine GeurtsvanKessel; David Nieuwenhuijse; Irina Chestakova; Jeroen van Kampen; Jolanda Voermans; Madelief Mollers; Manon Haverkate; Marion Koopmans; Mark Pronk; Mart Stein; Pascal Lexmond; Reina Sikkema; Richard Molenkamp; Sandra Kengne Kamga Mobou; on behalf of the Dutch national COVID-19 response team.
EPI_ISL_413591	MHC Flevoland	Erasmus Medical Center	Anne van der Linden; Annemiek van der Eijk; Aura Timen; Bas Oude Munnink; Claudia Schapendonk; Corien Swaan; Corine GeurtsvanKessel; David Nieuwenhuijse; Irina Chestakova; Jeroen van Kampen; Jolanda Voermans; Madelief Mollers; Manon Haverkate; Marion Koopmans; Mark Pronk; Mart Stein; Pascal Lexmond; Reina Sikkema; Richard Molenkamp; Sandra Kengne Kamga Mobou; on behalf of the Dutch national COVID-19 response team.
EPI_ISL_413566	MHC Gooi & Vechtstreek	Erasmus Medical Center	Anne van der Linden; Annemiek van der Eijk; Aura Timen; Bas Oude Munnink; Claudia Schapendonk; Corien Swaan; Corine GeurtsvanKessel; David Nieuwenhuijse; Irina Chestakova; Jeroen van Kampen; Jolanda Voermans; Madelief Mollers; Manon Haverkate; Marion Koopmans; Mark Pronk; Mart Stein; Pascal Lexmond; Reina Sikkema; Richard Molenkamp; Sandra Kengne Kamga Mobou; on behalf of the Dutch national COVID-19 response team.
EPI_ISL_413579	MHC Haaglanden	Erasmus Medical Center	Anne van der Linden; Annemiek van der Eijk; Aura Timen; Bas Oude Munnink; Claudia Schapendonk; Corien Swaan; Corine GeurtsvanKessel; David Nieuwenhuijse; Irina Chestakova; Jeroen van Kampen; Jolanda Voermans; Madelief Mollers; Manon Haverkate; Marion Koopmans; Mark Pronk; Mart Stein; Pascal Lexmond; Reina Sikkema; Richard Molenkamp; Sandra Kengne Kamga Mobou; on behalf of the Dutch national COVID-19 response team.
EPI_ISL_413590	MHC Utrecht	Erasmus Medical Center	Anne van der Linden; Annemiek van der Eijk; Aura Timen; Bas Oude Munnink; Claudia Schapendonk; Corien Swaan; Corine GeurtsvanKessel; David Nieuwenhuijse; Irina Chestakova; Jeroen van Kampen; Jolanda Voermans; Madelief Mollers; Manon Haverkate; Marion Koopmans; Mark Pronk; Mart Stein; Pascal Lexmond; Reina Sikkema; Richard Molenkamp; Sandra Kengne Kamga Mobou; on behalf of the Dutch national COVID-19 response team.
EPI_ISL_413564	MHC West-Brabant	Erasmus Medical Center	Anne van der Linden; Annemiek van der Eijk; Aura Timen; Bas Oude Munnink; Claudia Schapendonk; Corien Swaan; Corine GeurtsvanKessel; David Nieuwenhuijse; Irina Chestakova; Jeroen van Kampen; Jolanda Voermans; Madelief Mollers; Manon Haverkate; Marion Koopmans; Mark Pronk; Mart Stein; Pascal Lexmond; Reina Sikkema; Richard Molenkamp; Sandra Kengne Kamga Mobou; on behalf of the Dutch national COVID-19 response team.
EPI_ISL_1446678 to 1446680, EPI_ISL_1446946 to 1446969, EPI_ISL_1447030 to 1447044, EPI_ISL_1516362 to 1516363, EPI_ISL_1528308 to 1528313	MI - Michigan Department of Health and Human Services - Bureau of Laboratories	Centers for Disease Control and Prevention Division of Viral Diseases, Pathogen Discovery	Alison Laufer Halpin; Ben L. Rambo-Martin; Clinton R. Paden; Dakota Howard; Darlene Wagner; Dave Wentworth; Dhwani Batra; Jasmine Padilla; Justin Lee; Katie Dillon; Krista Queen; Kristen Knipe; Kristine Lacek; Mark Burroughs; Matthew Schmerer; Mili Sheth; Peter Cook; Sam Shepard; Sarah Nobles; Shoshona Le; Suxiang Tong; Vivien Dugan; Yvette Unoarumhi
EPI_ISL_486394, EPI_ISL_486398 to 486399, EPI_ISL_515968 to 515973	MIMS	Department of Neurovirology, National Institute of Mental Health and Neuroscience (NIMHANS)	Anita Desai; Chitra Pattabiraman; Harsha PK; Manjunatha Venkataswamy; Pramada Prasad; Ravi Vasanthapuram; Risha Rasheed; Shafeeq S Hameed; Vijayalakshmi Reddy
EPI_ISL_1524887 to 1524888, EPI_ISL_1524896 to 1524905	MKK Mühlenkreiskliniken, Johannes Wesling Klinikum Minden	Hannover Medical School, Institute of Virology	Lars Steinbrück
EPI_ISL_1538846 to 1538848, EPI_ISL_1538983, EPI_ISL_1539116, EPI_ISL_1539206	MLD Szpital w. Elbiety	1. National Institute of Public Health - National Institute of Hygiene; 2. Eurofins Genomics Europe Sequencing GmbH	ECDC COVID-19 WGS support team; Eurofins Genomics Europe Sequencing Team; Gierczyki Rafa; Sadkowska-Todys Magorzata; Wokowicz Tomasz; Zacharczuk Katarzyna
EPI_ISL_452108, EPI_ISL_452113, EPI_ISL_452133, EPI_ISL_594463	MN Department of Health	Pathogen Discovery, Respiratory Viruses Branch, Division of Viral Diseases, Centers for Disease Control and Prevention	Alison S. Laufer Halpin; Anna Montmayeur; Anna Uehara; Christopher A. Elkins; Clinton Paden; Clinton R. Paden; Haibin Wang; Jing Zhang; Julu Bhatnagar; Krista Queen; Mary S. Keckler; Rachel Marine; Suxiang Tong; Yan Li; Ying Tao; Zachary Weiner
EPI_ISL_1516302 to 1516304	MN PHL Division, Minnesota Department of Health	Centers for Disease Control and Prevention Division of Viral Diseases, Pathogen Discovery	Alison Laufer Halpin; Ben L. Rambo-Martin; Clinton R. Paden; Dakota Howard; Darlene Wagner; Dave Wentworth; Dhwani Batra; Jasmine Padilla; Justin Lee; Katie Dillon; Krista Queen; Kristen Knipe; Kristine Lacek; Mark Burroughs; Matthew Schmerer; Mili Sheth; Peter Cook; Sam Shepard; Sarah Nobles; Shoshona Le; Suxiang Tong; Vivien Dugan; Yvette Unoarumhi
EPI_ISL_426426 to 426427, EPI_ISL_527667 to 527736	MN PHL Division, Minnesota Department of Health	Pathogen Discovery, Respiratory Viruses Branch, Division of Viral Diseases, Centers for Disease Control and Prevention	Alison S. Laufer Halpin; Anna Montmayeur; Anna Uehara; Brian Lynch; Christopher A. Elkins; Clinton R. Paden; Haibin Wang; Jing Zhang; Krista Queen; Mary S. Keckler; Rachel Marine; Suxiang Tong; Yan Li; Ying Tao
EPI_ISL_1446299, EPI_ISL_1446313, EPI_ISL_1446787, EPI_ISL_1446790, EPI_ISL_1446923 to 1446927, EPI_ISL_1516400, EPI_ISL_1528503 to 1528505			
see above	MO State Public Health Laboratory	Centers for Disease Control and Prevention Division of Viral Diseases, Pathogen Discovery	Alison Laufer Halpin; Ben L. Rambo-Martin; Clinton R. Paden; Dakota Howard; Darlene Wagner; Dave Wentworth; Dhwani Batra; Jasmine Padilla; Justin Lee; Katie Dillon; Krista Queen; Kristen Knipe; Kristine Lacek; Mark Burroughs; Matthew Schmerer; Mili Sheth; Peter Cook; Sam Shepard; Sarah Nobles; Shoshona Le; Suxiang Tong; Vivien Dugan; Yvette Unoarumhi
EPI_ISL_424870	MO State Public Health Laboratory	Pathogen Discovery, Respiratory Viruses Branch, Division of Viral Diseases, Centers for Disease Control and Prevention	Alison S. Laufer Halpin; Anna Uehara; Christopher A. Elkins; Clinton R. Paden; Haibin Wang; Jing Zhang; Krista Queen; Mary S. Keckler; Rachel Marine; Suxiang Tong; Yan Li; Ying Tao
EPI_ISL_1397618 to 1397622, EPI_ISL_1397788 to 1397809, EPI_ISL_1397870 to 1397885, EPI_ISL_1398034 to 1398038, EPI_ISL_1398048 to 1398067, EPI_ISL_1398120 to 1398122, EPI_ISL_1398132 to 1398136, EPI_ISL_1398331, EPI_ISL_1398333 to 1398339, EPI_ISL_1462551, EPI_ISL_1465668 to 1465672, EPI_ISL_1465701 to 1465713, EPI_ISL_1465794 to 1465801			
see above	MONTEFIORE MEDICAL CENTER LABORATORIES	Wadsworth Center, New York State Department of Health	Alexis Russel; Alexis Russell; Catharine Prussing; Daryl M. Lamson; Erasmus Schneider; Erica Lasek-Nesselquist; John Kelly; Jonathan Plitnick; Kirsten St. George; Matthew Shudt; Melissa A Leisner; Navjot Singh
EPI_ISL_1465802, EPI_ISL_1465849 to 1465852, EPI_ISL_1482336 to 1482345	MONTEFIORE NEW ROCHELLE HOSPITAL LABORATORY	Wadsworth Center, New York State Department of Health	Alexis Russell; Catharine Prussing; Daryl M. Lamson; Erasmus Schneider; Erica Lasek-Nesselquist; John Kelly; Jonathan Plitnick; Kirsten St. George; Matthew Shudt; Melissa A Leisner; Navjot Singh
EPI_ISL_1469313 to 1469432	MRC/UVRI & LSHTM Uganda Research Unit	Where sequence data have been generated and submitted to GISAID	Dan Lule Bugembe; Isaac Sseeewanyana; Matthew Cotten; My V.T. Phan; Patrick Semanda; Pontiano Kaleebu; Susan Nabadda
EPI_ISL_428856	MRCG at LSHTM Genomics Lab	MRCG at LSHTM Genomics lab	Sesay et al
EPI_ISL_428857, EPI_ISL_471158 to 471171, EPI_ISL_560992 to 561204, EPI_ISL_561207, EPI_ISL_561209 to 561230, EPI_ISL_561234 to 561238, EPI_ISL_561240 to 561241, EPI_ISL_561249, EPI_ISL_561252 to 561253, EPI_ISL_561255 to 561256, EPI_ISL_561258 to 561269, EPI_ISL_561272, EPI_ISL_561274, EPI_ISL_561276, EPI_ISL_561281, EPI_ISL_561283, EPI_ISL_561285, EPI_ISL_561288 to 561295, EPI_ISL_561297, EPI_ISL_561302, EPI_ISL_561304, EPI_ISL_561309 to 561310, EPI_ISL_561312, EPI_ISL_561315 to 561316, EPI_ISL_561319, EPI_ISL_561322, EPI_ISL_561325, EPI_ISL_561328			
see above	MRCG at LSHTM Genomics lab	MRCG at LSHTM Genomics lab	Abdoulie Kanteh; Abdul Karim sesay; Bakary Sanyang; Jarra Manneh; Mariama Kujabi; Sesay et al

EPI_ISL_428855	MRCG at LSHTM Geomics lab	MRCG at LSHTM Genomics lab	Sesay et al
EPI_ISL_1446290, EPI_ISL_1446293, EPI_ISL_1446296, EPI_ISL_1446302, EPI_ISL_1446304, EPI_ISL_1446307, EPI_ISL_1446310, EPI_ISL_1446315, EPI_ISL_1446318, EPI_ISL_1446321, EPI_ISL_1446766 to 1446772, EPI_ISL_1446932 to 1446936, EPI_ISL_1516368 to 1516372, EPI_ISL_1528411 to 1528413			
see above	MS Public Health Laboratory	Centers for Disease Control and Prevention Division of Viral Diseases, Pathogen Discovery	Alison Laufer Halpin; Ben L. Rambo-Martin; Clinton R. Paden; Dakota Howard; Darlene Wagner; Dave Wentworth; Dhwani Batra; Jasmine Padilla; Justin Lee; Katie Dillon; Krista Queen; Kristen Knipe; Kristine Lacek; Mark Burroughs; Matthew Schmerer; Mili Sheth; Peter Cook; Sam Shepard; Sarah Nobles; Shoshona Le; Suxiang Tong; Vivien Dugan; Yvette Unoarumhi
EPI_ISL_414476, EPI_ISL_415151, EPI_ISL_421348 to 421435, EPI_ISL_421593 to 421635, EPI_ISL_422488 to 422562, EPI_ISL_450004 to 450170	MSHS Clinical Microbiology Laboratories	MSHS Pathogen Surveillance Program	Adolfo Garcia-Sarstre; Adolfo Garcia-Sastre; Ajay Obla; Alberto Paniz-mondolfi; Ana S. Gonzalez-Reiche; Ana Silvia Gonzalez-Reiche; Ana Sylvia Gonzalez-Reiche; Bremy Albuquerque; Bremy Alburquerque; Emilia Sordillo; Florian Krammer; Gopi Patel; Harm van Bakel; Jayeeta Dutta; Jose Polanco; Juan Soto; Judith Aberg; Lisa Miorin; Matthew Hernandez; Melissa Gitman; Melissa Smith; Mitchell Sullivan; Nancy Francoeur; Randy Albrecht; Robert Sebra; Shelcie Fabre; Shwetha Sridhar Hara; Viviana Simon; Wen-chun Liu; Ying-Chih Wang; Zenab Khan
EPI_ISL_1446447, EPI_ISL_1446450, EPI_ISL_1446453, EPI_ISL_1446476, EPI_ISL_1446480, EPI_ISL_1446482, EPI_ISL_1446486, EPI_ISL_1446684 to 1446691, EPI_ISL_1446857, EPI_ISL_1516305 to 1516312, EPI_ISL_1528294 to 1528302			
see above	MT Public Health Laboratory	Centers for Disease Control and Prevention Division of Viral Diseases, Pathogen Discovery	Alison Laufer Halpin; Ben L. Rambo-Martin; Clinton R. Paden; Dakota Howard; Darlene Wagner; Dave Wentworth; Dhwani Batra; Jasmine Padilla; Justin Lee; Katie Dillon; Krista Queen; Kristen Knipe; Kristine Lacek; Mark Burroughs; Matthew Schmerer; Mili Sheth; Peter Cook; Sam Shepard; Sarah Nobles; Shoshona Le; Suxiang Tong; Vivien Dugan; Yvette Unoarumhi
EPI_ISL_1408887, EPI_ISL_1408893, EPI_ISL_1408897, EPI_ISL_1509926, EPI_ISL_1509946	MT Public Health Laboratory	Genomics and Discovery, Respiratory Viruses Branch, Division of Viral Diseases, Centers for Disease Control and Prevention	Adam Retchless; Anna Kelleher; Anna Montmayeur; Anna Uehara; Brian Lynch; Clinton R. Paden; Haibin Wang; Han Jia Justin Ng; Jing Zhang; Justin Lee; Krista Queen; Mark Burroughs; Peter Cook; Rachel Marine; Suxiang Tong; Yan Li; Ying Tao
EPI_ISL_1482648 to 1482973	MUSC Molecular Pathology Laboratory	MUSC Molecular Pathology Laboratory	Dariusz Pytel; Frederick S. Nolte; Jaclyn Dunne; Julie W. Hirschhorn; Kristen Maurer; W. Bailey Glen Jr
EPI_ISL_445309, EPI_ISL_445355	MUTUAL DE SEGURIDAD C.CH.C.	Instituto de Salud Publica de Chile	Alejandra Acevedo; Andrés E Castillo; Bárbara Parra; Carolina Tambley; Gabriel Leal; Jaime Lagos; Jorge Fernandez; Loredana Arata; Patricia Bustos; Paz Tapia; Rodrigo Fasce; Winston Andrade
EPI_ISL_1435341 to 1435343, EPI_ISL_1435346 to 1435353, EPI_ISL_1435355 to 1435356, EPI_ISL_1435358 to 1435361, EPI_ISL_1435363 to 1435378, EPI_ISL_1435380 to 1435381, EPI_ISL_1435383, EPI_ISL_1435386 to 1435387, EPI_ISL_1435389 to 1435390, EPI_ISL_1435396 to 1435399, EPI_ISL_1435401 to 1435405, EPI_ISL_1435408 to 1435412, EPI_ISL_1435414 to 1435419, EPI_ISL_1435421 to 1435422, EPI_ISL_1435424 to 1435427, EPI_ISL_1439442 to 1439443, EPI_ISL_1439447, EPI_ISL_1439450, EPI_ISL_1439456, EPI_ISL_1439469, EPI_ISL_1439474, EPI_ISL_1439478, EPI_ISL_1439480 to 1439481, EPI_ISL_1439488, EPI_ISL_1439491 to 1439495, EPI_ISL_1439497 to 1439500, EPI_ISL_1439502 to 1439503, EPI_ISL_1439505 to 1439509, EPI_ISL_1439512, EPI_ISL_1439514			
see above	MVZ Dr. Eberhard & Partner Dortmund	Robert Koch Institute	
EPI_ISL_1434506, EPI_ISL_1435320, EPI_ISL_1435328, EPI_ISL_1441202	MVZ Labor Dortmund	Robert Koch Institute	
EPI_ISL_1442509	MVZ Labor Dr. Fenner und Kollegen (Standort Hamburg)	Robert Koch Institute	
EPI_ISL_1433601, EPI_ISL_1433676, EPI_ISL_1434469, EPI_ISL_1438584, EPI_ISL_1438590, EPI_ISL_1438594 to 1438596, EPI_ISL_1438599 to 1438600, EPI_ISL_1438602, EPI_ISL_1438607, EPI_ISL_1438610, EPI_ISL_1438617, EPI_ISL_1438625 to 1438626, EPI_ISL_1438629 to 1438630, EPI_ISL_1438637, EPI_ISL_1438641, EPI_ISL_1438646 to 1438647, EPI_ISL_1438653, EPI_ISL_1438656, EPI_ISL_1438663, EPI_ISL_1438665, EPI_ISL_1438670, EPI_ISL_1438674 to 1438675, EPI_ISL_1438677 to 1438678, EPI_ISL_1438681 to 1438685, EPI_ISL_1441227, EPI_ISL_1441648, EPI_ISL_1441652			
see above	MVZ Labor Dr. Limbach & Kollegen GbR	Robert Koch Institute	
EPI_ISL_1433607, EPI_ISL_1433623, EPI_ISL_1433665, EPI_ISL_1433669, EPI_ISL_1441656, EPI_ISL_1441659, EPI_ISL_1441662, EPI_ISL_1441672			
see above	MVZ Labor Dr. Reising-Ackermann und Kollegen	Robert Koch Institute	
EPI_ISL_1434520 to 1434521, EPI_ISL_1434524, EPI_ISL_1434526, EPI_ISL_1434528, EPI_ISL_1434530, EPI_ISL_1434532, EPI_ISL_1434534, EPI_ISL_1434536, EPI_ISL_1434538, EPI_ISL_1434540, EPI_ISL_1434542, EPI_ISL_1434544, EPI_ISL_1434546, EPI_ISL_1434548, EPI_ISL_1434550, EPI_ISL_1434552, EPI_ISL_1434554, EPI_ISL_1434556, EPI_ISL_1434559, EPI_ISL_1434561, EPI_ISL_1434563, EPI_ISL_1434567 to 1434568, EPI_ISL_1434570, EPI_ISL_1434572, EPI_ISL_1434574, EPI_ISL_1434576, EPI_ISL_1434578, EPI_ISL_1434581 to 1434582, EPI_ISL_1434584, EPI_ISL_1434586, EPI_ISL_1434588, EPI_ISL_1434590, EPI_ISL_1434592, EPI_ISL_1434594, EPI_ISL_1434596, EPI_ISL_1434598, EPI_ISL_1434600 to 1434601, EPI_ISL_1434603, EPI_ISL_1434605, EPI_ISL_1434607, EPI_ISL_1434609, EPI_ISL_1434611, EPI_ISL_1434613, EPI_ISL_1439438, EPI_ISL_1439625 to 1439646, EPI_ISL_1439648 to 1439670, EPI_ISL_1440873 to 1440878, EPI_ISL_1440880 to 1440884, EPI_ISL_1440886 to 1440899, EPI_ISL_1440901 to 1440909, EPI_ISL_1440911 to 1440934, EPI_ISL_1440936 to 1440950, EPI_ISL_1442896 to 1442912, EPI_ISL_1442914 to 1442925, EPI_ISL_1442927 to 1442941			
see above	MVZ Labor Krone GbR	Robert Koch Institute	
EPI_ISL_1434454, EPI_ISL_1434463, EPI_ISL_1434471, EPI_ISL_1434479, EPI_ISL_1434485, EPI_ISL_1435293, EPI_ISL_1435296, EPI_ISL_1435298, EPI_ISL_1435306, EPI_ISL_1435310, EPI_ISL_1441219			
see above	MVZ Medizinisches Labor Hannover GmbH	Robert Koch Institute	
EPI_ISL_1433501, EPI_ISL_1433503, EPI_ISL_1433524, EPI_ISL_1433535, EPI_ISL_1433543, EPI_ISL_1433559, EPI_ISL_1433565			
see above	MVZ für Laboratoriumsmedizin und Mikrobiologie Koblenz-Mittelrhein (Labor Koblenz)	Robert Koch Institute	
EPI_ISL_1435354, EPI_ISL_1439452	MVZ für Laboratoriumsmedizin und Mikrobiologie Würzburg	Robert Koch Institute	
EPI_ISL_1443226 to 1443232, EPI_ISL_1443288, EPI_ISL_1478849, EPI_ISL_1478895 to 1478896, EPI_ISL_1478905, EPI_ISL_1478926, EPI_ISL_1553186, EPI_ISL_1553261 to 1553263			
see above	Macon County Jail	Illinois Department of Public Health - Springfield Lab	Bryan Sim; Gordon McCall
EPI_ISL_508423 to 508440	Mahatma Gandhi Institute of Medical Sciences	National Institute of Biomedical Genomics	Arindam Maitra; Deepashri Maraskolhe; Rahul Narang; Saumitra Das; Vijayshri Deotale
EPI_ISL_511923 to 511929, EPI_ISL_522437	Mahatma Gandhi Institute of Medical Sciences	National Institute of Biomedical Genomics - DBT's PAN-INDIA 1000 SARS--CoV-2 RNA Genome Sequencing Consortium	Arindam Maitra; Deepashri Maraskolhe; Rahul Narang; Saumitra Das; Vijayshri Deotale
EPI_ISL_614294	Maimonides Medical Center	New York City Public Health Laboratory	Jade Wang; et al.
EPI_ISL_1502087 to 1502111	Maine HETL	Health and Environmental Testing Laboratory	Barter, M.; Dewey, H.; H. and Tewhey, R.; Lynch, R.; Martha, J.; Matluk, N.; Munger
EPI_ISL_513414 to 513510	Maine HETL	Tewhey Lab, The Jackson Laboratory	Barter, M.; Dewey, H.; H. and Tewhey, R.; Lynch, R.; Matluk, N.; Munger
EPI_ISL_1495983	Maine Health and Environmental Testing Laboratory	Maine Health and Environmental Testing Laboratory	Barter, M.; Dewey, H.; H. and Tewhey, R.; Lynch, R.; Martha, J.; Matluk, N.; Munger
EPI_ISL_1523643 to 1523793	Maine Health and Environmental Testing Laboratory	Tewhey Lab, The Jackson Laboratory	Barter, M.; Dewey, H.; H. and Tewhey, R.; Iosue, F.; Lynch, R.; Matluk, N.; Munger
EPI_ISL_428348	Maison de Santé du Val d'Ormois	National Reference Center for Viruses of Respiratory Infections, Institut Pasteur, Paris	Angela Brisebarre; Etienne Simon-Lorière; Flora Donati; Marion Barbet; Maud Vanpeene; Mélanie Albert; Méline Bizard; Sylvie Behillil; Sylvie van der Werf; Vincent Enouf
EPI_ISL_430838	Makati Medical Center	Research Institute for Tropical Medicine	Bautista; Brunker, K.; C.S.; C.T.; D.L.; Demetria; E.S.; F.G.M.; I.A.P.; Manalo; Medado; Mercado; O.J.T.; Onza; Polotan
EPI_ISL_528738 to 528744, EPI_ISL_568873 to 568875, EPI_ISL_582124, EPI_ISL_1435802, EPI_ISL_1435804, EPI_ISL_1435806, EPI_ISL_1435808, EPI_ISL_1435810, EPI_ISL_1435812, EPI_ISL_1435814, EPI_ISL_1435816 to 1435817, EPI_ISL_1435820			
see above	Malaysia Genome Institute	Malaysia Genome Institute	Azrin Ahmad; Enizza Kasim; Gan Han Ming; Irni Suhayu Sapian; Mohd Faizal Abu Bakar; Mohd Noor Mat Isa; Nor Azfa Johari; Nor Azfa Johari.; Nurhezreen Md Iqbal; Shahrul Hisham Zainal Ariffin; Shamsidar Sopie; Siti Noraini Othman; Tan Ju Lin; Yusuf Muhammad Noor
EPI_ISL_1385833 to 1385835	Mandya Institute of Medical Sciences	INSACOG-KA, NIMHANS	Anita S Desai; Anson Kunjumon George; Chitra Pattabiraman; Darshan Sreenivas; Nakka Vijay Kiran Reddy; Pramada Prasad; V Ravi
EPI_ISL_1478853, EPI_ISL_1553233	Maple Street Clinic	Illinois Department of Public Health - Springfield Lab	Bryan Sim; Gordon McCall
EPI_ISL_1547801	Mapmygenome	CSIR-Centre for Cellular and Molecular Biology - INSACOG	Amareshwar Vodapalli; Ara Sreenivas; B Himasri; Divya Tej Sowpati; Karthik Bharadwaj Tallapaka; Krishna Khairnar; Lamuk Zaveri; Onkar Kulkarni; Rakesh K Mishra; Sharath Chandra Thota; Shreekant Verma; Sofia Banu; Viswagithe S L
EPI_ISL_1439433, EPI_ISL_1439435, EPI_ISL_1439437, EPI_ISL_1439439, EPI_ISL_1439441, EPI_ISL_1439444, EPI_ISL_1439446, EPI_ISL_1439448 to 1439449, EPI_ISL_1439451, EPI_ISL_1439455, EPI_ISL_1439470, EPI_ISL_1439472, EPI_ISL_1439482, EPI_ISL_1439484, EPI_ISL_1528160 to 1528169, EPI_ISL_1557224 to 1557246, EPI_ISL_1559612 to 1559614			
see above	Marche en Famenne	Plateforme de testing Namuroise	; Céline Maschietto; Degosserie Jonathan; Denis Olivier; Mullier François; Otto Gaetan
EPI_ISL_583198	Marin County Public Health Department	Chan-Zuckerberg Biohub	CZB Cliahub Consortium

EPI_ISL_1455670	Marius Nasta Institute of Pneumology	"Stefan S. Nicolau"Institute of Virology	Adriana Plesa; Alina Nastasie; Ana Iulia Neagu; Anca Botezatu; Camelia Sultana; Carmen Cristina Diaconu; Coralia Bleotu; Cristina Mambet; Denisa Dragu; Gabriela Anton; Ioana Pitica; Iulia Virginia Iancu; Laura Grecu; Laura Necula; Lilia Matei; Marinela Bostan; Mihaela Economescu; Mirela Mihaila; Saviana Nedeianu; Simona Ruta; Tudor Constantinescu
EPI_ISL_1533441 to 1533442, EPI_ISL_1533532, EPI_ISL_1533534	Marshfield Clinic Research Institute	Genomics and Discovery, Respiratory Viruses Branch, Division of Viral Diseases, Centers for Disease Control and Prevention	Adam Retchless; Anna Kelleher; Anna Montmayeur; Anna Uehara; Brian Lynch; Clinton R. Paden; Haibin Wang; Han Jia Justin Ng; Jing Zhang; Justin Lee; Krista Queen; Mark Burroughs; Peter Cook; Rachel Marine; Suxiang Tong; Yan Li; Ying Tao
EPI_ISL_481265 to 481282, EPI_ISL_486912 to 486917, EPI_ISL_509444 to 509491, EPI_ISL_522824 to 522854, EPI_ISL_522869 to 522871	Maryland Department of Health	Maryland Department of Health	Keller, E.; Maryland Department of Health Laboratories Administration
EPI_ISL_454421 to 454435	Maryland Department of Health Laboratories Administration	Maryland Department of Health Laboratories Administration	MDH Laboratories Administration
EPI_ISL_525118, EPI_ISL_529167 to 529168	Maryland Department of Health Laboratory	Maryland Department of Health Laboratory	Eric Keller; Maryland Department of Health Laboratories Administration
EPI_ISL_1400802 to 1400941, EPI_ISL_1444777 to 1444908, EPI_ISL_1448029 to 1448042, EPI_ISL_1469292 to 1469312, EPI_ISL_1500246 to 1500425, EPI_ISL_1516754 to 1516759	Maryland Genomics, Institute for Genome Sciences, University of Maryland School of Medicine	Maryland Genomics, Institute for Genome Sciences, University of Maryland School of Medicine	Aditya; Claire M; Fraser; Holly; Humphrys; Jacques; Kranthi; Lisa D; Luke J; Mehta; Mike; Ott; Ravel; Roussey; Sadzewicz; Sandra; Tallon; Vavikolanu
EPI_ISL_538265 to 538274, EPI_ISL_560806 to 560832, EPI_ISL_1482980 to 1483025	Maryland Public Health Laboratory	Maryland Public Health Laboratory	Maryland Department of Health Laboratories Administration
EPI_ISL_1443268, EPI_ISL_1478850 to 1478851, EPI_ISL_1478890, EPI_ISL_1478900, EPI_ISL_1478921, EPI_ISL_1500172 to 1500175, EPI_ISL_1500218, EPI_ISL_1500229 to 1500231, EPI_ISL_1500245, EPI_ISL_1553246 to 1553247			
see above	Mason District Hospital	Illinois Department of Public Health - Springfield Lab	Bryan Sim; Gordon McCall
EPI_ISL_409067	Massachusetts Department of Public Health	Pathogen Discovery, Respiratory Viruses Branch, Division of Viral Diseases, Centers for Dieases Control and Prevention	Anna Uehara; Brett L. Whitaker; Brian Lynch; Clinton R. Paden; Janna' R. Murray; Jing Zhang; Krista Queen; Lijuan Wang; Senthil Kumar K. Sakthivel; Shifaq Kamili; Stephen Lindstrom; Susan I. Gerber; Suxiang Tong; Xiaoyan Lu; Yan Li; Ying Tao
EPI_ISL_460097 to 460472, EPI_ISL_1413133, EPI_ISL_1413136, EPI_ISL_1413139	Massachusetts General Hospital	Infectious Disease Program, Broad Institute of Harvard and MIT	A.E.; Adams, G.; Anahtar, M.; B.L.; B.W.; Bauer, M.; Birren; Branda, J.; Carter, A.; Cerrato, F.; Chaluvadi, S.; Chapman; Cusick, C.; D.J.; DeRuff, K.; E. and Sabeti; Flowers, K.; Gallagher, G.; Gladden-Young, A.; Gnirke, A.; Harris, J.; J.E.; K.J.; LaRocque, R.; Lagerborg, K.; Lemieux; Lin; Loreth, C.; MacInnis; Neumann, A.; Normandin, E.; P.C.; Park; Pierce, V.; Reilly, S.; Rosenberg; Rosenberg, E.; Rudy, M.; Ryan, E.; S.B.; Sabeti; Shaw, B.; Siddle; Slater, D.; Smole, S.; Tomkins-Tinch, C.; Turbett, S.
EPI_ISL_1404617 to 1404623, EPI_ISL_1406697 to 1406704, EPI_ISL_1503460 to 1503791, EPI_ISL_1527091 to 1527236, EPI_ISL_1528093 to 1528159	Massachusetts State Public Health Laboratory	Massachusetts State Public Health Laboratory	Andrew Lang; Glen Gallagher; Sandra Smole; Timelia Fink
EPI_ISL_418799, EPI_ISL_418801, EPI_ISL_420878 to 420879	Mater Pathology	Public Health Virology Laboratory	Alyssa Pyke; Amanda De Jong; Andrew Van Den Hurk; Bixing Huang; Carmel Taylor; David Warrilow; Doris Genge; Elisabeth Gamez; Glen Hewitson; Ian Maxwell Mackay; Inga Sultana; Jamie McMahon; Jean Barcelon; Judy Northill; Mitchell Finger; Natalie Simpson; Neelima Nair; Peter Burtonclay; Peter Moore; Sarah Wheatley; Sean Moody; Sonja Hall-Mendelin; Timothy Gardam; and Frederick Moore
EPI_ISL_508415 to 508422	Maulana Azad Medical College	National Institute of Biomedical Genomics	Arindam Maitra; Oves Siddiqui; Saumitra Das; Sonal Saxena; Vikas Manchanda
EPI_ISL_420898 to 420909, EPI_ISL_420911 to 420912	Max von Pettenkofer Institute, Virology, National Reference Center for Retroviruses, LMU Munich	Laboratory for Functional Genome Analysis, Dept. Genomics, Gene Center of the LMU Munich	Alexander Graf; Ashok Varadharajan; Helmut Blum; Max Muenchhoff; Oliver Keppler; Stefan Krebs
EPI_ISL_437204 to 437297, EPI_ISL_437359, EPI_ISL_451936 to 451947, EPI_ISL_452103 to 452104, EPI_ISL_466874 to 466925, EPI_ISL_548949 to 548961, EPI_ISL_549025			
see above	Max von Pettenkofer Institute, Virology, National Reference Center for Retroviruses, LMU München	Laboratory for Functional Genome Analysis, Dept. Genomics, Gene Center of the LMU Munich	Alexander Graf; Helmut Blum; Max Muenchhoff; Oliver Keppler; Stefan Krebs
EPI_ISL_477272 to 477276, EPI_ISL_477291 to 477312, EPI_ISL_481242, EPI_ISL_482967 to 482987, EPI_ISL_495597 to 495609, EPI_ISL_496918, EPI_ISL_507957 to 507961, EPI_ISL_514619 to 514628, EPI_ISL_514636, EPI_ISL_514638 to 514640, EPI_ISL_514644, EPI_ISL_514653 to 514656, EPI_ISL_515263 to 515266, EPI_ISL_518856 to 518900, EPI_ISL_521153, EPI_ISL_527630 to 527631, EPI_ISL_539842 to 539849, EPI_ISL_560792 to 560797, EPI_ISL_603010 to 603011, EPI_ISL_1396409 to 1396410, EPI_ISL_1446028, EPI_ISL_1446048 to 1446049, EPI_ISL_1446051 to 1446052, EPI_ISL_1446054 to 1446055, EPI_ISL_1479042 to 1479096, EPI_ISL_1482393, EPI_ISL_1527465, EPI_ISL_1527468, EPI_ISL_1527470, EPI_ISL_1527472, EPI_ISL_1527474, EPI_ISL_1527476 to 1527487, EPI_ISL_1527498 to 1527502, EPI_ISL_1527510 to 1527521			
see above	Mayo Clinic & Mayo Clinic Laboratories	Minnesota Department of Health, Public Health Laboratory	Alexandra Lorentz; Jacob Garfin; Kelly Pung; Matt Plumb; and Xiong Wang
EPI_ISL_490978, EPI_ISL_490988, EPI_ISL_490997 to 491000, EPI_ISL_491004	Mayo Clinic Laboratories	UW Virology Lab	Alexander Greninger; Amin Addetia; Hong Xie; Keith R Jerome; Lasata Shrestha; Meei-Li Huang; Pavitra Roychoudhury; Truong Nguyen; Victoria M Rachleff
EPI_ISL_500503 to 500538, EPI_ISL_501072 to 501082, EPI_ISL_501165	Mayo Clinic Laboratories	University of Washington Virology Lab	Alexander Greninger; Amin Addetia; Hong Xie; Keith R Jerome; Lasata Shrestha; Meei-Li Huang; Pavitra Roychoudhury; Truong Nguyen; Victoria M Rachleff
EPI_ISL_569620	McCrossan Boys Ranch	South Dakota Public Health Laboratory	Jacob Garfin; Matt Plumb; Xiong Wang; and Chris Carlson
EPI_ISL_1443237, EPI_ISL_1443269 to 1443272	McDonough District Hospital	Illinois Department of Public Health - Springfield Lab	Bryan Sim; Gordon McCall
EPI_ISL_1500228	McFarland Mental H.C.	Illinois Department of Public Health - Springfield Lab	Bryan Sim; Gordon McCall
EPI_ISL_1443273	McHenry Co Coroners Office	Illinois Department of Public Health - Springfield Lab	Bryan Sim; Gordon McCall
EPI_ISL_1478859 to 1478863, EPI_ISL_1478877 to 1478880, EPI_ISL_1478897 to 1478899, EPI_ISL_1478902, EPI_ISL_1478912, EPI_ISL_1478919 to 1478920, EPI_ISL_1500170, EPI_ISL_1500177 to 1500178, EPI_ISL_1500183 to 1500194, EPI_ISL_1500201 to 1500203, EPI_ISL_1500216, EPI_ISL_1500220 to 1500227, EPI_ISL_1500243 to 1500244, EPI_ISL_1553228 to 1553231, EPI_ISL_1553235 to 1553243, EPI_ISL_1553250			
see above	Mcdonough District Hospital	Illinois Department of Public Health - Springfield Lab	Bryan Sim; Gordon McCall
EPI_ISL_1439758, EPI_ISL_1439763, EPI_ISL_1439769	Med. Labor Prof. Schenk Dr. Ansorge & Kollegen	Robert Koch Institute	
EPI_ISL_456377 to 456378	MedLab Central Ltd	Institute of Environmental Science and Research (ESR)	Anja Werno; Antje van der Linden; Arlo Upton; Chris Mansell; David Hammer; Dragana Drinkovic; Erasmus Smit; Gary McAuliffe; Hana Sofia Andersson; James Ussher; Jill Sherwood; Joep de Ligt; Josh Freeman; Julia Howard; Juliet Elvy; Lauren Jelly; Mary DeAlmeida; Matt Blakiston; Matt Storey; Matthew Rogers; Max Bloomfield; Michael Addidle; Michelle Balm; Sally Roberts; Sarah Jefferies; Sharmini Muttaiyah; Susan Morpeth; Susan Taylor; Timothy Blackmore; Vani Sathyendran; Veronica Playle; Virginia Hope; Xiaoyun Ren
EPI_ISL_482759 to 482775, EPI_ISL_483035 to 483038	Medical Ain Shams Research Institute (MASRI), Ain Shams University	Medical Ain Shams Research Institute (MASRI), Ain Shams University	Ahmad Moustafa; Ashraf Omar; Aya Mohamed; Ghada Ismael; Hala Hafez; Hesham Elghazaly; Mahmoud Elmeitini; Osama Mansour; Reham Mamdouh; Sara Elnakeep; Sara Hassan Agwa; Shaimaa Moustafa
EPI_ISL_476832	Medical Biology Department, Kocaeli University	Medical Genetics Department, Kocaeli University	Akhan S; Akpinar G; Canturk NZ.; Cine N; Eren-Keskin S; Ilgazli A; Karadenizli A; Kasap M; Savli H; Sayan M; Sunnetci-Akkoyunlu D
EPI_ISL_495535 to 495542, EPI_ISL_515800 to 515893	Medical Disagnostics Services (MDS)	KRISP, KZN Research Innovation and Sequencing Platform	Chimukangara B; ChimukangaraB; Giandhari J; Khan S; Lessells R; Mdlalose K; Pillay S; Tegally H; Wilkinson E; York D; de Oliveira T
EPI_ISL_1507483, EPI_ISL_1508711, EPI_ISL_1508825 to 1508826, EPI_ISL_1508829 to 1508832, EPI_ISL_1508835 to 1508836, EPI_ISL_1508838, EPI_ISL_1508840, EPI_ISL_1508843, EPI_ISL_1508845 to 1508848, EPI_ISL_1508850 to 1508852			
see above	Medical Genetics Laboratory, Regional Centre of Medical Genetics, Emergency County Hospital Craiova	Medical Genetics Laboratory, Regional Centre of Medical Genetics, Emergency County Hospital Craiova	Adina Dragos; Ana-Maria Buga; Anca-Lelia (Riza) Costache; Andrei Pirvu; Elena Plesea; Ioana Streata; Mihai Cucu; Mihai Ioana; Monica Cara; Razvan Plesea; Stefania Dorobantu
EPI_ISL_447838 to 447839, EPI_ISL_481216, EPI_ISL_493381 to 493383, EPI_ISL_549039 to 549041, EPI_ISL_549059, EPI_ISL_549070, EPI_ISL_549081, EPI_ISL_549085 to 549086, EPI_ISL_549164, EPI_ISL_549166, EPI_ISL_590879 to 590880, EPI_ISL_590893, EPI_ISL_590929, EPI_ISL_590932 to 590933, EPI_ISL_590945, EPI_ISL_1385964, EPI_ISL_1385967, EPI_ISL_1385970, EPI_ISL_1385973, EPI_ISL_1385978, EPI_ISL_1385986, EPI_ISL_1385989, EPI_ISL_1402951 to 1402955, EPI_ISL_1490195 to 1490196, EPI_ISL_1490199, EPI_ISL_1490204, EPI_ISL_1490220 to 1490221, EPI_ISL_1490264, EPI_ISL_1490273, EPI_ISL_1490277, EPI_ISL_1490283, EPI_ISL_1490290, EPI_ISL_1490302, EPI_ISL_1490305 to 1490306, EPI_ISL_1490308 to 1490309, EPI_ISL_1490313 to 1490314, EPI_ISL_1490324, EPI_ISL_1490327, EPI_ISL_1490329, EPI_ISL_1490355, EPI_ISL_1490358 to 1490364, EPI_ISL_1490368, EPI_ISL_1490378,			

EPI_ISL_1490393, EPI_ISL_1490395, EPI_ISL_1490397, EPI_ISL_1490419, EPI_ISL_1490432, EPI_ISL_1490435, EPI_ISL_1490438, EPI_ISL_1490440, EPI_ISL_1490442, EPI_ISL_1490445, EPI_ISL_1490447, EPI_ISL_1490450, EPI_ISL_1490454, EPI_ISL_1490494 to 1490497, EPI_ISL_1490499, EPI_ISL_1490520 to 1490522, EPI_ISL_1490555, EPI_ISL_1490572 to 1490573, EPI_ISL_1490604, EPI_ISL_1490609, EPI_ISL_1490611, EPI_ISL_1490613, EPI_ISL_1490615, EPI_ISL_1490617, EPI_ISL_1490621, EPI_ISL_1490630, EPI_ISL_1490636, EPI_ISL_1490638 to 1490644, EPI_ISL_1490646, EPI_ISL_1490649, EPI_ISL_1547544 to 1547545, EPI_ISL_1547547 to 1547548, EPI_ISL_1547551 to 1547552, EPI_ISL_1547609, EPI_ISL_1547613			
see above	Medical Microbiology Unit, Department for Laboratory Medicine, Drammen Hospital, Vestre Viken Health Trust,	Norwegian Institute of Public Health, Department of Virology	Atiya R Ali; Debech Nadia; Engebretsen Serina Beate; Garcia Llorente Ignacio; Hilde Elshaug; Hilde Synnøve Vollan; Hilde Vollan; Jon Bråte; Kamilla Heddeland Instefjord; Karoline Bragstad; Kathrine Stene-Johansen; Marie Paulsen Madsen; Olav Hungnes; Pedersen Benedikte Nevjen; Rasmus Riis Kopperud
EPI_ISL_482745 to 482746	Medical Microbiology, Leiden University Medical Center	Medical Microbiology, Leiden University Medical Center	Dalebout; E.J.; J.C.; J.C. and Sidorov, I.; J.J. and Sidorov, I.; N.S.; Ogando; Snijder; T.J.; Zevenhoven; de Vries
EPI_ISL_1523640 to 1523642	Medical Research Center, Faculty of Medicine, Syarif Hidayatullah State Islamic University Jakarta	Medical Research Center, Faculty of Medicine, Syarif Hidayatullah State Islamic University Jakarta	Chris Adhiyanto; Endah Wulandari; Erike A Suwarsono; Fika Ekayanti; Flori R Sari; Hari Hendarto; Laifa Hendarmin; Zeti Harriyati
EPI_ISL_591522 to 591530	Medicina Norte U Chile - Servicio Medico Legal	Center for Mathematical Modeling and Center for Genome Regulation. Santiago, Chile	Allende ML; Ferres M.; Gaete A; Gaggero A; González M; Maass A; Palma R; Travisany D; Urra C; Valiente F; Varas M
EPI_ISL_1394763, EPI_ISL_1394767, EPI_ISL_1394807, EPI_ISL_1394812, EPI_ISL_1394817, EPI_ISL_1394824, EPI_ISL_1394827 to 1394828, EPI_ISL_1394831 to 1394832, EPI_ISL_1394834, EPI_ISL_1394836, EPI_ISL_1394838 to 1394839, EPI_ISL_1540556, EPI_ISL_1540558, EPI_ISL_1540576 to 1540579, EPI_ISL_1540583, EPI_ISL_1540589 to 1540611, EPI_ISL_1540706			
see above	Medicina practica laboratorija	Lithuanian University of Health Sciences Hospital, Department of Genetics and Molecular Medicine	Astra Vitkauskiene; Darius Cereskevicius; Inga Nasvytiene; Mantas Sarauskas; Marius Sukys; Rasa Ugenskiene; Renaldas Jurkevicius; Rima Vainoriene; Zivile Zemeckiene
EPI_ISL_1465482	Medicine sentinel network Province	National Reference Center for Viruses of Respiratory Infections, Institut Pasteur, Paris	Angela Brisebarre; Camille Capel; Christophe Malabat; Corinne Maufrais; Damien Mornico; Etienne Simon-Lorière; Frédéric Lemoine; Louise Lefrançois; Marion Barbet; Maud Vanpeene; Méline Bizard; Nicolas Le Duff; Sylvie Behillil; Sylvie van der Werf; Vincent Enouf
EPI_ISL_435281	Medistra Hospital Jakarta	Eijkman Institute for Molecular Biology, Ministry of Research and Technology/National Agency for Research and Innovation	Amin Soebandrio; David H Muljono; Edison Johar; Frilasita A Yudhaputri; Hidayat Trimarsanto; Khin Saw Myint; Safarina G Malik
EPI_ISL_1439797, EPI_ISL_1439799 to 1439800, EPI_ISL_1439803, EPI_ISL_1439809 to 1439810, EPI_ISL_1439819, EPI_ISL_1439821 to 1439822, EPI_ISL_1439824 to 1439828, EPI_ISL_1439830, EPI_ISL_1439834 to 1439836, EPI_ISL_1439839, EPI_ISL_1439841, EPI_ISL_1439847, EPI_ISL_1439850, EPI_ISL_1439858, EPI_ISL_1439861, EPI_ISL_1439867, EPI_ISL_1439869, EPI_ISL_1442543			
see above	Medizinisch-Diagnostisches Labor Kempten allgäulab	Robert Koch Institute	
EPI_ISL_581452 to 581454, EPI_ISL_581456 to 581461, EPI_ISL_581463 to 581471, EPI_ISL_581473 to 581485	Medizinische Klinik Innere Medizin I, Universitätsklinikum Tübingen	NGS Competence Center Tübingen, Institut für Medizinische Mikrobiologie und Hygiene, Universitätsklinikum Tübingen	Angel Angelov
EPI_ISL_1436580 to 1436619	Medizinische Laboratorien Düsseldorf	Robert Koch Institute	
EPI_ISL_1434437, EPI_ISL_1434441, EPI_ISL_1434456, EPI_ISL_1434458, EPI_ISL_1434461, EPI_ISL_1434473, EPI_ISL_1434475, EPI_ISL_1434477, EPI_ISL_1434483, EPI_ISL_1434487, EPI_ISL_1434492, EPI_ISL_1434498, EPI_ISL_1434500, EPI_ISL_1434502, EPI_ISL_1434504, EPI_ISL_1434508, EPI_ISL_1434512, EPI_ISL_1438586 to 1438589, EPI_ISL_1438592 to 1438593, EPI_ISL_1438597, EPI_ISL_1438601, EPI_ISL_1438603, EPI_ISL_1438608, EPI_ISL_1438615, EPI_ISL_1438619, EPI_ISL_1438621 to 1438623, EPI_ISL_1438627 to 1438628, EPI_ISL_1438632 to 1438633, EPI_ISL_1438636, EPI_ISL_1438638, EPI_ISL_1438640, EPI_ISL_1438643, EPI_ISL_1438645, EPI_ISL_1438648, EPI_ISL_1438651, EPI_ISL_1438654, EPI_ISL_1438657, EPI_ISL_1438659, EPI_ISL_1438661, EPI_ISL_1438668, EPI_ISL_1438679, EPI_ISL_1441234, EPI_ISL_1441236, EPI_ISL_1441238, EPI_ISL_1441246, EPI_ISL_1441249, EPI_ISL_1441252 to 1441253, EPI_ISL_1441256, EPI_ISL_1441259, EPI_ISL_1441279, EPI_ISL_1441282, EPI_ISL_1441285 to 1441286, EPI_ISL_1441288 to 1441289, EPI_ISL_1441296, EPI_ISL_1441301, EPI_ISL_1441303, EPI_ISL_1441309			
see above	Medizinisches Labor Ostsachsen MVZ GbRNebenbetriebsstätte Dresden	Robert Koch Institute	
EPI_ISL_1439445, EPI_ISL_1439464, EPI_ISL_1439468, EPI_ISL_1439471, EPI_ISL_1439475, EPI_ISL_1439483, EPI_ISL_1439490, EPI_ISL_1439496, EPI_ISL_1439501, EPI_ISL_1439504, EPI_ISL_1439511, EPI_ISL_1439515 to 1439519			
see above	Medizinisches Labor Wahl Lüdenscheid	Robert Koch Institute	
EPI_ISL_1435384, EPI_ISL_1439440	Medizinisches Versorgungszentrum für Labormedizin und Mikrobiologie Ruhr GmbH - mvzlm RUHR GmbH	Robert Koch Institute	
EPI_ISL_1434843, EPI_ISL_1434853, EPI_ISL_1434861, EPI_ISL_1435255, EPI_ISL_1435275, EPI_ISL_1435277, EPI_ISL_1438298 to 1438299, EPI_ISL_1438316, EPI_ISL_1438344, EPI_ISL_1438348, EPI_ISL_1438352, EPI_ISL_1438371, EPI_ISL_1438401, EPI_ISL_1438502, EPI_ISL_1438537, EPI_ISL_1441316, EPI_ISL_1441332, EPI_ISL_1441349, EPI_ISL_1441357, EPI_ISL_1441364 to 1441365, EPI_ISL_1441380, EPI_ISL_1441384, EPI_ISL_1441387, EPI_ISL_1441389, EPI_ISL_1441397, EPI_ISL_1441399, EPI_ISL_1441401, EPI_ISL_1441414, EPI_ISL_1441417, EPI_ISL_1441422, EPI_ISL_1441432, EPI_ISL_1441488, EPI_ISL_1441513, EPI_ISL_1441517, EPI_ISL_1441519, EPI_ISL_1441525, EPI_ISL_1441530, EPI_ISL_1441544, EPI_ISL_1441555, EPI_ISL_1441570, EPI_ISL_1441581, EPI_ISL_1441585, EPI_ISL_1441608, EPI_ISL_1441645 to 1441647			
see above	Medizinisches Zentrallabor Altenburg GmbH & Co.KG	Robert Koch Institute	
EPI_ISL_451534, EPI_ISL_451553 to 451555, EPI_ISL_451558, EPI_ISL_451567, EPI_ISL_451583, EPI_ISL_451597, EPI_ISL_451610, EPI_ISL_513341, EPI_ISL_526192, EPI_ISL_591492, EPI_ISL_593654			
see above	Medlab Pathology	NSW Health Pathology - Institute of Clinical Pathology and Medical Research; Westmead Hospital; University of Sydney	CIDM-PH et al.
EPI_ISL_536411, EPI_ISL_536793	Medtimes Molecular Laboratory	Medtimes Molecular Laboratory	Eric Chan; Isaac Chow; Jacqueline Tam; Winsome Wong
EPI_ISL_1393820 to 1393821, EPI_ISL_1393829, EPI_ISL_1393831	Medyczne Laboratorium Diagnostyczne American Heart of Poland	1. National Institute of Public Health - National Institute of Hygiene; 2. Eurofins Genomics Europe Sequencing GmbH	ECDC COVID-19 WGS support team; Eurofins Genomics Europe Sequencing Team; Gierczyki Rafa; Sadkowska-Todys Magorzata; Wokowicz Tomasz; Zacharczuk Katarzyna
EPI_ISL_1394138 to 1394140	Medyczne Laboratorium Diagnostyczne, szpital w. Elbiety w Katowicach	1. National Institute of Public Health - National Institute of Hygiene; 2. Eurofins Genomics Europe Sequencing GmbH	ECDC COVID-19 WGS support team; Eurofins Genomics Europe Sequencing Team; Gierczyki Rafa; Sadkowska-Todys Magorzata; Wokowicz Tomasz; Zacharczuk Katarzyna
EPI_ISL_1538468, EPI_ISL_1539093	Medyczne Laboratorium Diagnostyka	1. National Institute of Public Health - National Institute of Hygiene; 2. Eurofins Genomics Europe Sequencing GmbH	ECDC COVID-19 WGS support team; Eurofins Genomics Europe Sequencing Team; Gierczyki Rafa; Sadkowska-Todys Magorzata; Wokowicz Tomasz; Zacharczuk Katarzyna
EPI_ISL_583427 to 583428, EPI_ISL_583430 to 583433, EPI_ISL_583437 to 583440, EPI_ISL_583443 to 583451, EPI_ISL_583455 to 583457, EPI_ISL_583460 to 583462, EPI_ISL_583465 to 583466, EPI_ISL_583468 to 583470, EPI_ISL_583472, EPI_ISL_583474 to 583476, EPI_ISL_583478 to 583480			
see above	Memorial Sloan Kettering Cancer Center	van Bakel Laboratory, Genetics and Genomics Sciences, Icahn School of Medicine at Mount Sinai	Adolfo García-Sastre; Adriana van de Guchte; Ajay Obla; Ana S. Gonzalez-Reiche; Genovefa Papanicolaou; Gunjan Shah; Harm van Bakel; Jayeeta Dutta; Judith Aberg; Kent Sepkowitz; Miguel-Angel Perales; Ngolela Esther Babady; Sadaf Aslam; Teresa Aydillo; Tobias Hohl; Zenab Khan; and Mini Kamboj
EPI_ISL_1520158	Mercy Hospital and Medical Center	Illinois Department of Public Health - Chicago Lab	Ira Heimler; Vineet K. Dhiman
EPI_ISL_436800 to 436890, EPI_ISL_437097 to 437186, EPI_ISL_447057 to 447118, EPI_ISL_447163 to 447229, EPI_ISL_450570 to 450637, EPI_ISL_452262 to 452326, EPI_ISL_459992 to 460043, EPI_ISL_460554 to 460602, EPI_ISL_462636 to 462736, EPI_ISL_471676 to 471910, EPI_ISL_476027 to 476066, EPI_ISL_507415 to 507933, EPI_ISL_516194 to 516412, EPI_ISL_529169, EPI_ISL_529804 to 529907, EPI_ISL_565833 to 565907, EPI_ISL_565926 to 566035, EPI_ISL_576198 to 576221, EPI_ISL_577547 to 577596, EPI_ISL_583506 to 583534, EPI_ISL_594190 to 594218, EPI_ISL_614158 to 614246, EPI_ISL_1461278 to 1461289, EPI_ISL_1461322, EPI_ISL_1461378 to 1461433, EPI_ISL_1461435 to 1461625, EPI_ISL_1493062 to 1493064, EPI_ISL_1493070 to 1493102, EPI_ISL_1527661 to 1527941, EPI_ISL_1527954 to 1528092			
see above	Michigan Department of Health and Human Services, Bureau of Laboratories	Michigan Department of Health and Human Services, Bureau of Laboratories	Blankenship; Blankenship HM; H.M.; Riner D; Riner, D.; Soehnlen MK; Soehnlen, M.
EPI_ISL_454353	Microbial Genome Sequencing Center; Microbial Genomic Epidemiology Laboratory	Microbial Genomic Epidemiology Laboratory, University of Pittsburgh	Chinelo Ezeonwuku; Dan Snyder; Jane W. Marsh; Kady D. Waggle; Lee H. Harrison; Marissa P. Griffith; Mustapha M. Mustapha; Stephanie L. Mitchell; Vatsala R. Srinivasa; Vaughn S. Cooper
EPI_ISL_426583	Microbial Genomics Laboratory, Institut Pasteur Monteivdeo	Microbial Genomics Laboratory, Institut Pasteur Montevideo, Uruguay	Cecilia Salazar; Florencia Díaz-Viraqué; Gonzalo Moratorio; Gregorio Iraola; Marianoel Pereira; Pilar Moreno
EPI_ISL_426479 to 426480, EPI_ISL_429257	Microbial Genomics Laboratory, Institut Pasteur Montevideo	Microbial Genomics Laboratory, Institut Pasteur Montevideo	Cecilia Salazar; Florencia Díaz-Viraqué; Gonzalo Moratorio; Gregorio Iraola; Marianoel Pereira; Pilar Moreno
EPI_ISL_426476 to 426478	Microbial Genomics Laboratory, Institut Pasteur Montevideo	Microbial Genomics Laboratory, Institut Pasteur Montevideo, Uruguay	Cecilia Salazar; Florencia Díaz-Viraqué; Gonzalo Moratorio; Gregorio Iraola; Marianoel Pereira; Pilar Moreno
EPI_ISL_426481 to 426482	Microbial Genomics Laboratory, Institut Pasteur Montevideo, Uruguay	Microbial Genomics Laboratory, Institut Pasteur Montevideo	Cecilia Salazar; Florencia Díaz-Viraqué; Gonzalo Moratorio; Gregorio Iraola; Marianoel Pereira; Pilar Moreno
EPI_ISL_426584	Microbial Genomics Laboratory, Institut Pasteur Montevideo,	Microbial Genomics Laboratory, Institut Pasteur Montevideo,	Cecilia Salazar; Florencia Díaz-Viraqué; Gonzalo Moratorio; Gregorio Iraola; Marianoel Pereira; Pilar Moreno

	Uruguay	Uruguay	
EPI_ISL_480331 to 480348	Microbial Genomics Laboratory, Institut Pasteur de Montevideo	Microbial Genomics Laboratory, Institut Pasteur de Montevideo	Cecilia Salazar; Gonzalo Moratorio; Gregorio Iraola; Ignacio Ferrés; Marianoel Pereira; Pilar Moreno
EPI_ISL_540430	Microbial Genomics Laboratory, Institut Pasteur de Montevideo, Montevideo, Uruguay	Microbial Genomics Laboratory, Institut Pasteur de Montevideo, Montevideo, Uruguay	Cecilia Salazar; Gonzalo Moratorio; Gregorio Iraola; Marianoel Pereira; Pilar Moreno
EPI_ISL_1524394 to 1524397, EPI_ISL_1524399, EPI_ISL_1524455 to 1524456, EPI_ISL_1524679 to 1524687, EPI_ISL_1524689 to 1524691	Microbiologia e Virologia	Istituto Zooprofilattico Sperimentale delle Venezie	Adelaide Milani; Alessia Schivo; Alice Fusaro; Ambra Pastori; Annalisa Salviato; Antonia Ricci; Calogero Terregino; Edoardo Giussani; Elisa Palumbo; Erika Giorgia Quaranta; Isabella Monne; Luca Tassoni
EPI_ISL_1524469, EPI_ISL_1524569 to 1524582	Microbiologia e Virologia	Istituto di Genomica Applicata; Istituto Zooprofilattico Sperimentale delle Venezie	Alessia Schivo; Alice Fusaro; Ambra Pastori; Annalisa Salviato; Antonia Ricci; Calogero Terregino; Davide Scaglione; Edoardo Giussani; Eleonora Paparelli; Erika Giorgia Quaranta; Francesca Bruno; Gabriele Magris; Irena Jurman; Isabella Monne; Luca Tassoni; Maria Varotto; Michele Morgante; Silvia Ormelli; Valeria D'Amico; Vera Vendramin
EPI_ISL_480558 to 480562, EPI_ISL_480587, EPI_ISL_480612 to 480618, EPI_ISL_480620, EPI_ISL_480687, EPI_ISL_480745 to 480763, EPI_ISL_480767 to 480774, EPI_ISL_480776 to 480777, EPI_ISL_518063 to 518080, EPI_ISL_518093 to 518097, EPI_ISL_518231 to 518237, EPI_ISL_518241 to 518243, EPI_ISL_518250 to 518283, EPI_ISL_518285 to 518290, EPI_ISL_518292 to 518337, EPI_ISL_518339 to 518380, EPI_ISL_518383 to 518387, EPI_ISL_518402 to 518423, EPI_ISL_518472 to 518505, EPI_ISL_518507 to 518557, EPI_ISL_518559 to 518586, EPI_ISL_518588 to 518590, EPI_ISL_518592 to 518598, EPI_ISL_518600 to 518611, EPI_ISL_518613 to 518614, EPI_ISL_518619, EPI_ISL_518621 to 518624, EPI_ISL_518626 to 518650, EPI_ISL_518652 to 518701, EPI_ISL_518703 to 518708, EPI_ISL_518711 to 518722, EPI_ISL_518725 to 518733, EPI_ISL_518735 to 518736, EPI_ISL_518745 to 518796, EPI_ISL_518901 to 518902, EPI_ISL_518904, EPI_ISL_518906 to 518915, EPI_ISL_518917 to 518931, EPI_ISL_518933, EPI_ISL_518939 to 518973, EPI_ISL_518975, EPI_ISL_518977 to 518980, EPI_ISL_519052, EPI_ISL_519054 to 519057, EPI_ISL_519059 to 519102, EPI_ISL_519104 to 519166, EPI_ISL_519168 to 519171, EPI_ISL_519173 to 519223, EPI_ISL_519226, EPI_ISL_519228, EPI_ISL_519230 to 519236, EPI_ISL_519238 to 519255, EPI_ISL_519327 to 519330, EPI_ISL_519333 to 519369, EPI_ISL_519371 to 519384, EPI_ISL_519386 to 519426, EPI_ISL_519428 to 519430, EPI_ISL_519432 to 519443, EPI_ISL_519445 to 519446, EPI_ISL_519449 to 519451, EPI_ISL_519453 to 519496, EPI_ISL_519498, EPI_ISL_519501, EPI_ISL_519503 to 519509, EPI_ISL_519511 to 519518, EPI_ISL_519522 to 519543, EPI_ISL_519547 to 519559, EPI_ISL_519561 to 519569, EPI_ISL_519571 to 519605, EPI_ISL_519607 to 519643, EPI_ISL_519645 to 519767, EPI_ISL_519770 to 519771, EPI_ISL_519773, EPI_ISL_519780 to 519795, EPI_ISL_519797 to 519808, EPI_ISL_519810 to 519813, EPI_ISL_519815 to 519894, EPI_ISL_519896 to 519911, EPI_ISL_519913 to 519947, EPI_ISL_519951 to 519958, EPI_ISL_519963 to 519980, EPI_ISL_519983 to 519990, EPI_ISL_519992 to 520005, EPI_ISL_520007 to 520031, EPI_ISL_520041 to 520055, EPI_ISL_520057 to 520059, EPI_ISL_520061 to 520063, EPI_ISL_520066 to 520070, EPI_ISL_520072 to 520075, EPI_ISL_520077, EPI_ISL_520079 to 520083, EPI_ISL_520085 to 520087, EPI_ISL_520089 to 520119, EPI_ISL_520121 to 520180, EPI_ISL_520215 to 520231, EPI_ISL_520233 to 520248, EPI_ISL_520392 to 520561, EPI_ISL_520563 to 520603, EPI_ISL_520606 to 520621, EPI_ISL_520623 to 520643, EPI_ISL_520645 to 520662, EPI_ISL_520765, EPI_ISL_520767 to 520771, EPI_ISL_520773 to 520774, EPI_ISL_520776 to 520788, EPI_ISL_520844 to 520863, EPI_ISL_520929 to 521086, EPI_ISL_521089 to 521101, EPI_ISL_521154, EPI_ISL_521156 to 521168, EPI_ISL_521170 to 521223, EPI_ISL_521226 to 521244, EPI_ISL_521247 to 521248, EPI_ISL_521251 to 521264, EPI_ISL_521266 to 521269, EPI_ISL_521278 to 521314, EPI_ISL_521450 to 521461, EPI_ISL_521562 to 521583, EPI_ISL_521585 to 521816, EPI_ISL_521818 to 521820, EPI_ISL_521822 to 521839, EPI_ISL_521887 to 521888, EPI_ISL_521906 to 521910, EPI_ISL_521923, EPI_ISL_521926 to 521935, EPI_ISL_521939 to 521944, EPI_ISL_521972 to 521974, EPI_ISL_521989 to 522054, EPI_ISL_522111 to 522118, EPI_ISL_522188 to 522193, EPI_ISL_522195 to 522232, EPI_ISL_522243 to 522253, EPI_ISL_561378 to 561379, EPI_ISL_561381 to 561387, EPI_ISL_561389 to 561391, EPI_ISL_561393 to 561396, EPI_ISL_561398 to 561402, EPI_ISL_561405 to 561406, EPI_ISL_561408, EPI_ISL_561410 to 561413, EPI_ISL_561415, EPI_ISL_561417 to 561419, EPI_ISL_561422 to 561424, EPI_ISL_561427, EPI_ISL_561429 to 561432, EPI_ISL_561435 to 561439, EPI_ISL_561441 to 561449, EPI_ISL_561451 to 561452, EPI_ISL_561454 to 561457, EPI_ISL_561459 to 561461, EPI_ISL_561463 to 561464, EPI_ISL_561466 to 561467, EPI_ISL_561469, EPI_ISL_561471 to 561490, EPI_ISL_561492 to 561497, EPI_ISL_561499 to 561507, EPI_ISL_561509 to 561513, EPI_ISL_561516, EPI_ISL_561518, EPI_ISL_561520 to 561522, EPI_ISL_561525 to 561531, EPI_ISL_561534, EPI_ISL_561536 to 561540, EPI_ISL_561542 to 561543, EPI_ISL_561545 to 561556, EPI_ISL_561558, EPI_ISL_561560 to 561564, EPI_ISL_561566 to 561577, EPI_ISL_561579 to 561581, EPI_ISL_561583, EPI_ISL_561585, EPI_ISL_561587 to 561589, EPI_ISL_561592 to 561611, EPI_ISL_561615 to 561620, EPI_ISL_561623 to 561624, EPI_ISL_561626 to 561628, EPI_ISL_561631 to 561650, EPI_ISL_561654, EPI_ISL_561658 to 561660, EPI_ISL_561662 to 561670, EPI_ISL_561672, EPI_ISL_561675 to 561678, EPI_ISL_561680 to 561681, EPI_ISL_561683, EPI_ISL_561685, EPI_ISL_561687 to 561689, EPI_ISL_561691 to 561696, EPI_ISL_561698 to 561706, EPI_ISL_561708 to 561710, EPI_ISL_561712 to 561714, EPI_ISL_561716 to 561720, EPI_ISL_561722 to 561725, EPI_ISL_561727, EPI_ISL_561729 to 561740, EPI_ISL_561743 to 561749, EPI_ISL_561751, EPI_ISL_561753 to 561754, EPI_ISL_561756 to 561763, EPI_ISL_561767 to 561776, EPI_ISL_561779 to 561786, EPI_ISL_561789 to 561790, EPI_ISL_561792, EPI_ISL_561794 to 561798, EPI_ISL_561801 to 561802, EPI_ISL_561804 to 561807, EPI_ISL_561809 to 561815, EPI_ISL_561817 to 561824, EPI_ISL_561826 to 561839, EPI_ISL_561841 to 561842, EPI_ISL_561845 to 561851, EPI_ISL_561853 to 561855, EPI_ISL_561857, EPI_ISL_561859 to 561862, EPI_ISL_561864 to 561865, EPI_ISL_561867 to 561878, EPI_ISL_561880 to 561881, EPI_ISL_561883 to 561892, EPI_ISL_561895 to 561906, EPI_ISL_561908 to 561909, EPI_ISL_561911 to 561916, EPI_ISL_561919 to 561927, EPI_ISL_561929 to 561934, EPI_ISL_561937 to 561945, EPI_ISL_561947 to 561956, EPI_ISL_561958 to 561974, EPI_ISL_561976 to 561985, EPI_ISL_561989 to 561990, EPI_ISL_561992 to 561993, EPI_ISL_561995 to 562010, EPI_ISL_562013 to 562015, EPI_ISL_562017 to 562020, EPI_ISL_562022, EPI_ISL_562024 to 562032, EPI_ISL_562034 to 562047, EPI_ISL_562049 to 562054, EPI_ISL_562058 to 562065, EPI_ISL_562071, EPI_ISL_562073 to 562075, EPI_ISL_562077 to 562079, EPI_ISL_562082 to 562084, EPI_ISL_562086 to 562103, EPI_ISL_562107 to 562108, EPI_ISL_562111 to 562114, EPI_ISL_562116 to 562122, EPI_ISL_562124 to 562135, EPI_ISL_562137, EPI_ISL_562139 to 562144, EPI_ISL_562146 to 562151, EPI_ISL_562153 to 562154, EPI_ISL_562156, EPI_ISL_562159 to 562162, EPI_ISL_562165, EPI_ISL_562167 to 562174, EPI_ISL_562176 to 562178, EPI_ISL_562180 to 562181, EPI_ISL_562183 to 562188, EPI_ISL_562190 to 562193, EPI_ISL_562195 to 562196, EPI_ISL_562198 to 562199, EPI_ISL_562201, EPI_ISL_562203 to 562206, EPI_ISL_562210, EPI_ISL_562212, EPI_ISL_562214 to 562215, EPI_ISL_562218 to 562223, EPI_ISL_562226 to 562227, EPI_ISL_562230, EPI_ISL_562232 to 562236, EPI_ISL_562238, EPI_ISL_562240 to 562241, EPI_ISL_562243, EPI_ISL_562246 to 562249, EPI_ISL_562251 to 562257, EPI_ISL_562260, EPI_ISL_562262 to 562263, EPI_ISL_562265 to 562266, EPI_ISL_562268, EPI_ISL_562270 to 562271, EPI_ISL_562276 to 562279, EPI_ISL_562281, EPI_ISL_562283 to 562285, EPI_ISL_562287, EPI_ISL_562291 to 562293, EPI_ISL_562295, EPI_ISL_562298 to 562303, EPI_ISL_562305 to 562307, EPI_ISL_562309 to 562316, EPI_ISL_562318 to 562321, EPI_ISL_562323 to 562325, EPI_ISL_562327, EPI_ISL_562329 to 562330, EPI_ISL_562332, EPI_ISL_562334, EPI_ISL_562336 to 562341, EPI_ISL_562343 to 562347, EPI_ISL_562349, EPI_ISL_562352 to 562353, EPI_ISL_562355, EPI_ISL_562358 to 562362, EPI_ISL_562364 to 562365, EPI_ISL_562367 to 562374, EPI_ISL_562378, EPI_ISL_562380, EPI_ISL_562382 to 562384, EPI_ISL_562386 to 562388, EPI_ISL_562392, EPI_ISL_562394 to 562395, EPI_ISL_562397 to 562399, EPI_ISL_562402 to 562405, EPI_ISL_562407 to 562409, EPI_ISL_562411, EPI_ISL_562416 to 562429, EPI_ISL_562432 to 562434, EPI_ISL_562436, EPI_ISL_562438 to 562439, EPI_ISL_562443 to 562444, EPI_ISL_562448 to 562459, EPI_ISL_562461 to 562462, EPI_ISL_562464 to 562465, EPI_ISL_562467 to 562473, EPI_ISL_562475 to 562481, EPI_ISL_562485 to 562487, EPI_ISL_562489 to 562501, EPI_ISL_562504, EPI_ISL_562506 to 562508, EPI_ISL_562510 to 562512, EPI_ISL_562515 to 562516, EPI_ISL_562519 to 562523, EPI_ISL_562525, EPI_ISL_562528 to 562530, EPI_ISL_562532 to 562534, EPI_ISL_562537, EPI_ISL_562541, EPI_ISL_562543 to 562544, EPI_ISL_562547, EPI_ISL_562549 to 562551, EPI_ISL_562553 to 562556, EPI_ISL_562560 to 562562, EPI_ISL_562564 to 562582, EPI_ISL_562584, EPI_ISL_562590 to 562592, EPI_ISL_562594, EPI_ISL_562596 to 562627, EPI_ISL_562629, EPI_ISL_562631 to 562639, EPI_ISL_562641 to 562644, EPI_ISL_562647 to 562657, EPI_ISL_562660 to 562668, EPI_ISL_562670, EPI_ISL_562672 to 562695, EPI_ISL_562698 to 562699, EPI_ISL_562701 to 562704, EPI_ISL_562706 to 562753, EPI_ISL_562755 to 562790, EPI_ISL_562793 to 562802, EPI_ISL_562810 to 562837, EPI_ISL_562846 to 562905, EPI_ISL_562907 to 562909, EPI_ISL_562914 to 562923, EPI_ISL_562925 to 562928, EPI_ISL_562930 to 562935, EPI_ISL_562937 to 562945, EPI_ISL_562947 to 562950, EPI_ISL_562952 to 562958, EPI_ISL_562960, EPI_ISL_562962 to 562999, EPI_ISL_563001 to 563005, EPI_ISL_563007 to 563009, EPI_ISL_563011, EPI_ISL_563013 to 563016, EPI_ISL_563018 to 563022, EPI_ISL_563024 to 563040, EPI_ISL_563042 to 563051, EPI_ISL_563053 to 563065, EPI_ISL_563067 to 563072, EPI_ISL_563074 to 563111, EPI_ISL_563113, EPI_ISL_563117 to 563130, EPI_ISL_563134 to 563135, EPI_ISL_563145 to 563168, EPI_ISL_563170 to 563172, EPI_ISL_563174 to 563185, EPI_ISL_563187 to 563188, EPI_ISL_563190 to 563195, EPI_ISL_563198 to 563200, EPI_ISL_563202, EPI_ISL_563204 to 563206, EPI_ISL_563208 to 563216, EPI_ISL_563218, EPI_ISL_563221 to 563222, EPI_ISL_563224, EPI_ISL_563226 to 563241, EPI_ISL_563243, EPI_ISL_563245 to 563365, EPI_ISL_563367 to 563406, EPI_ISL_563424 to 563496, EPI_ISL_563500 to 563550, EPI_ISL_563552 to 563608, EPI_ISL_563610 to 563720, EPI_ISL_563724 to 563789, EPI_ISL_563791, EPI_ISL_563793 to 563807, EPI_ISL_563809 to 563810, EPI_ISL_563812 to 563816, EPI_ISL_563818 to 563819, EPI_ISL_563821 to 563831, EPI_ISL_563833 to 563839, EPI_ISL_563841 to 563865, EPI_ISL_563867 to 563879, EPI_ISL_563881 to 563924, EPI_ISL_563926 to 563937, EPI_ISL_563939 to 563972, EPI_ISL_563976, EPI_ISL_563979 to 563986, EPI_ISL_563989 to 563993, EPI_ISL_563995 to 563996, EPI_ISL_564050 to 564054, EPI_ISL_564111 to 564144, EPI_ISL_564146 to 564179, EPI_ISL_564255 to 564269, EPI_ISL_564271 to 564317, EPI_ISL_564351, EPI_ISL_564398 to 564403, EPI_ISL_564405 to 564454, EPI_ISL_564471 to 564490, EPI_ISL_564494 to 564495, EPI_ISL_564497, EPI_ISL_564503 to 564520, EPI_ISL_564522 to 564543, EPI_ISL_564545, EPI_ISL_564565 to 564904, EPI_ISL_564906 to 565003, EPI_ISL_565008 to 565033, EPI_ISL_565035 to 565039, EPI_ISL_565042 to 565109, EPI_ISL_565111 to 565135, EPI_ISL_565223 to 565242, EPI_ISL_565244 to 565263, EPI_ISL_565265 to 565276, EPI_ISL_565280 to 565306, EPI_ISL_565309, EPI_ISL_565315 to 565381, EPI_ISL_565383 to 565384, EPI_ISL_565386 to 565393, EPI_ISL_565397 to 565398, EPI_ISL_565400, EPI_ISL_565405 to 565408, EPI_ISL_565410 to 565414, EPI_ISL_565416 to 565458, EPI_ISL_565460, EPI_ISL_565462, EPI_ISL_565468, EPI_ISL_565470 to 565478, EPI_ISL_565484, EPI_ISL_565492 to 565508, EPI_ISL_565510 to 565565, EPI_ISL_565567 to 565622, EPI_ISL_565625 to 565729, EPI_ISL_565743, EPI_ISL_565745 to 565829, EPI_ISL_591550 to 591557, EPI_ISL_591559 to 591560, EPI_ISL_591563 to 591564, EPI_ISL_591566 to 591570, EPI_ISL_591572, EPI_ISL_591575 to 591577, EPI_ISL_591579, EPI_ISL_591581 to 591583, EPI_ISL_591586, EPI_ISL_591588 to 591589, EPI_ISL_591593 to 591594, EPI_ISL_591596 to 591598, EPI_ISL_591600 to 591602, EPI_ISL_591604 to 591608, EPI_ISL_591610 to 591612, EPI_ISL_591614, EPI_ISL_591616 to 591618, EPI_ISL_591620, EPI_ISL_591622, EPI_ISL_591624 to 591625, EPI_ISL_591627 to 591637, EPI_ISL_591639, EPI_ISL_591641 to 591642, EPI_ISL_591644, EPI_ISL_591646 to 591651, EPI_ISL_591655, EPI_ISL_591660 to 591665, EPI_ISL_591668, EPI_ISL_591671, EPI_ISL_591673 to 591678, EPI_ISL_591680, EPI_ISL_591682 to 591683, EPI_ISL_591685 to 591687, EPI_ISL_591689 to 591697, EPI_ISL_591699, EPI_ISL_591701 to 591702, EPI_ISL_591705, EPI_ISL_591709 to 591714, EPI_ISL_591716 to 591720, EPI_ISL_591722, EPI_ISL_591724, EPI_ISL_591727 to 591728, EPI_ISL_591730 to 591731, EPI_ISL_591733, EPI_ISL_591735 to 591736, EPI_ISL_591738, EPI_ISL_591741 to 591742, EPI_ISL_591745, EPI_ISL_591747 to 591753, EPI_ISL_591756 to 591757, EPI_ISL_591759, EPI_ISL_591762, EPI_ISL_591764 to 591766, EPI_ISL_591768, EPI_ISL_591776 to 591777, EPI_ISL_591780 to 591781, EPI_ISL_591784 to 591785, EPI_ISL_591787 to 591788, EPI_ISL_591793 to 591798, EPI_ISL_591801 to 591806, EPI_ISL_591808 to 591821, EPI_ISL_591823, EPI_ISL_591826 to 591848, EPI_ISL_591853, EPI_ISL_591905 to 591906, EPI_ISL_591910 to 591911, EPI_ISL_591913 to 591916, EPI_ISL_591918, EPI_ISL_591920 to 591924, EPI_ISL_591926 to 591927, EPI_ISL_591929 to 591931, EPI_ISL_591933 to 591939, EPI_ISL_591961 to 591967, EPI_ISL_591969 to 591981, EPI_ISL_591983 to 592012, EPI_ISL_592014 to 592027, EPI_ISL_592029 to 592035, EPI_ISL_592037 to 592049, EPI_ISL_592051 to 592068, EPI_ISL_592070 to 592103, EPI_ISL_592106 to 592135, EPI_ISL_592196 to 592282, EPI_ISL_592284 to 592379, EPI_ISL_592381 to 592382, EPI_ISL_592384 to 592391, EPI_ISL_592393 to 592414, EPI_ISL_592416 to 592484, EPI_ISL_592487, EPI_ISL_592510, EPI_ISL_592517 to 592530, EPI_ISL_592536, EPI_ISL_592538 to 592547, EPI_ISL_592549 to 592563, EPI_ISL_592565 to 592567, EPI_ISL_592569 to 592576, EPI_ISL_592579 to 592589, EPI_ISL_592591 to 592596, EPI_ISL_592598 to 592599, EPI_ISL_592601 to 592609, EPI_ISL_592611 to 592615, EPI_ISL_592617 to 592628, EPI_ISL_592630 to 592648, EPI_ISL_592650 to 592659, EPI_ISL_592662 to 592667, EPI_ISL_592669 to 592686, EPI_ISL_592688 to 592715, EPI_ISL_592717 to 592719, EPI_ISL_592721 to 592729, EPI_ISL_592731 to 592752, EPI_ISL_592754 to 592769, EPI_ISL_592772 to 592780, EPI_ISL_592784 to 592789, EPI_ISL_592791 to 592794, EPI_ISL_592796 to 592803, EPI_ISL_592805 to 592813, EPI_ISL_592815 to 592819, EPI_ISL_592822 to 592823, EPI_ISL_592825 to 592827, EPI_ISL_592829 to 592835, EPI_ISL_592838 to 592857, EPI_ISL_592861, EPI_ISL_592866 to 592877, EPI_ISL_592882 to 592887, EPI_ISL_592889 to 592902, EPI_ISL_592904 to 592906, EPI_ISL_592908 to 592957, EPI_ISL_592959 to 592968, EPI_ISL_592986 to 593002, EPI_ISL_593008 to 593032, EPI_ISL_593034, EPI_ISL_593036 to 593047, EPI_ISL_593049 to 593124, EPI_ISL_593129 to 593214, EPI_ISL_593217 to 593255, EPI_ISL_593257 to 593293, EPI_ISL_593295 to 593328, EPI_ISL_593330 to 593410, EPI_ISL_593413 to 593416, EPI_ISL_593418 to 593421, EPI_ISL_593427 to 593429, EPI_ISL_593431 to 593432, EPI_ISL_593434 to 593435, EPI_ISL_593437 to 593441, EPI_ISL_593443 to 593448, EPI_ISL_593450 to 593475			
see above	Microbiological Diagnostic Unit - Public Health Laboratory (MDU-PHL)	MDU-PHL	M. B.; Sait, M.; Schultz; Schultz M.; Schultz M. B.; Seemann T.; Seemann, T.; Sherry, N.
EPI_ISL_518651, EPI_ISL_519895, EPI_ISL_520604 to 520605, EPI_ISL_520644	Microbiological Diagnostic Unit - Public Health Laboratory (MDU-PHL)	Microbiological Diagnostic Unit - Public Health Laboratory (MDU-PHL)	Sait, M.; Schultz M.; Seemann T.; Sherry, N.
EPI_ISL_419714 to 419732, EPI_ISL_419823 to 419825, EPI_ISL_419827 to 419830, EPI_ISL_419980 to 419981, EPI_ISL_419999 to 420003, EPI_ISL_420006 to 420017, EPI_ISL_426905, EPI_ISL_426912 to 426922, EPI_ISL_427054 to 427078, EPI_ISL_427133, EPI_ISL_427148 to 427149, EPI_ISL_430470 to 430472, EPI_ISL_430639 to 430687, EPI_ISL_430715 to 430717			
see above	Microbiological Diagnostic Unit Public Health Laboratory	Microbiological Diagnostic Unit Public Health Laboratory	Sait, M.; Schultz M.; Seemann T.; Sherry, N.
EPI_ISL_456478 to 456479, EPI_ISL_456594 to 456595	Microbiological Diagnostic Unit Public Health Laboratory	Microbiological Diagnostic Unit Public Health Laboratory, The Peter Doherty Institute for Infection and Immunity	Sait, M.; Schultz M.; Seemann T.; Sherry, N.
EPI_ISL_447899	Microbiology	Microbiology	Ahmed; Hasanuzzaman, M.; Islam; M. and Saha; M.S.; M.S.I.; Malaker, R.; Rahman, H.; S.K.; Saha, S.; Sajib; Z.B.
EPI_ISL_513925	Microbiology & Bioinformatics and Biostatistics, Kohat University of Science and Technology (Pakistan) & Shanghai Jiao Tong University (China)	Microbiology & Bioinformatics and Biostatistics, Kohat University of Science and Technology (Pakistan) & Shanghai Jiao Tong University (China)	A.S.; Ali, S.; D.Q.; Khan; M.T.; Muhammad; N. and Wei; T.A.
EPI_ISL_528788 to 528808, EPI_ISL_541753, EPI_ISL_541756, EPI_ISL_541767, EPI_ISL_541773 to 541774	Microbiology Department, Barking Havering and Redbridge University Hospitals NHS trust	Wellcome Sanger Institute for the COVID-19 Genomics UK (COG-UK) consortium	Amy Ash; Cherian Koshy and Alex Alderton; Cordelia Langford; David K. Jackson; Dominic Kwiatkowski; Ewan Harrison; Fatima Ali; Ian Johnston; John Sillitoe on behalf of the Wellcome Sanger Institute COVID-19 Surveillance Team; John Sillitoe on behalf of the Wellcome Sanger Institute COVID-19 Surveillance Team (http://www.sanger.ac.uk/covid-team); Roberto Amato; Sonia Goncalves
EPI_ISL_482069, EPI_ISL_482071 to 482074, EPI_ISL_482076, EPI_ISL_482078 to 482081, EPI_ISL_482083 to 482090, EPI_ISL_482092 to 482095, EPI_ISL_482098 to 482100, EPI_ISL_482103 to 482104, EPI_ISL_482106 to 482114, EPI_ISL_482116 to 482119, EPI_ISL_488837 to 488839, EPI_ISL_488841 to 488844, EPI_ISL_488846, EPI_ISL_488848 to 488852, EPI_ISL_488857 to 488860, EPI_ISL_488862, EPI_ISL_488865 to 488871, EPI_ISL_488873			

see above	Microbiology Department, Hereford County Hospital	Wellcome Sanger Institute for the COVID-19 Genomics UK (COG-UK) consortium	Alison Johnson; Cordelia Langford; David K. Jackson; Dominic Kwiatkowski; Ewan Harrison; Fenella Halstead; Ian Johnston; Jane Thomas; John Sillitoe on behalf of the Wellcome Sanger Institute COVID-19 Surveillance Team (http://www.sanger.ac.uk/covid-team); Roberto Amato; Samantha Lamb and Alex Alderton; Sonia Goncalves; Venkat Sivaprakasam; Wendy Hogsden
EPI_ISL_1385995 to 1386006, EPI_ISL_1386008, EPI_ISL_1386010, EPI_ISL_1386012, EPI_ISL_1386015, EPI_ISL_1386017, EPI_ISL_1386019, EPI_ISL_1386022, EPI_ISL_1386024, EPI_ISL_1386026, EPI_ISL_1386029, EPI_ISL_1386031, EPI_ISL_1386034 to 1386060, EPI_ISL_1386062 to 1386065, EPI_ISL_1386067 to 1386076, EPI_ISL_1386078 to 1386083, EPI_ISL_1386086 to 1386087, EPI_ISL_1386089, EPI_ISL_1386091 to 1386093, EPI_ISL_1386095, EPI_ISL_1386097 to 1386105, EPI_ISL_1405968 to 1405983, EPI_ISL_1405985 to 1406004, EPI_ISL_1406006 to 1406018, EPI_ISL_1406020 to 1406022, EPI_ISL_1406024 to 1406039, EPI_ISL_1406041 to 1406044, EPI_ISL_1406046 to 1406060, EPI_ISL_1406062 to 1406064, EPI_ISL_1486514, EPI_ISL_1486517, EPI_ISL_1486519, EPI_ISL_1486522, EPI_ISL_1486525, EPI_ISL_1486528, EPI_ISL_1486531, EPI_ISL_1486534, EPI_ISL_1486537, EPI_ISL_1486540, EPI_ISL_1486543, EPI_ISL_1486546, EPI_ISL_1486549, EPI_ISL_1486552, EPI_ISL_1486555, EPI_ISL_1486558, EPI_ISL_1486562, EPI_ISL_1486565, EPI_ISL_1486568, EPI_ISL_1486571, EPI_ISL_1486574, EPI_ISL_1486577, EPI_ISL_1486580, EPI_ISL_1486583, EPI_ISL_1486587, EPI_ISL_1486590, EPI_ISL_1486593, EPI_ISL_1486596, EPI_ISL_1486599, EPI_ISL_1486602, EPI_ISL_1486605, EPI_ISL_1486608, EPI_ISL_1486610, EPI_ISL_1486613, EPI_ISL_1486616, EPI_ISL_1486619, EPI_ISL_1486622, EPI_ISL_1486625, EPI_ISL_1486628, EPI_ISL_1486631, EPI_ISL_1486634, EPI_ISL_1486637, EPI_ISL_1486640, EPI_ISL_1486643, EPI_ISL_1486647, EPI_ISL_1486649, EPI_ISL_1486652, EPI_ISL_1486655, EPI_ISL_1486658, EPI_ISL_1486662, EPI_ISL_1486665, EPI_ISL_1486668, EPI_ISL_1486671, EPI_ISL_1486674, EPI_ISL_1486678, EPI_ISL_1486681, EPI_ISL_1486684, EPI_ISL_1486687, EPI_ISL_1486691, EPI_ISL_1486693, EPI_ISL_1486696, EPI_ISL_1486699, EPI_ISL_1486703, EPI_ISL_1486706, EPI_ISL_1486709, EPI_ISL_1486713, EPI_ISL_1486716, EPI_ISL_1486719, EPI_ISL_1486722, EPI_ISL_1486724, EPI_ISL_1486727, EPI_ISL_1486730, EPI_ISL_1486733, EPI_ISL_1486736, EPI_ISL_1486739, EPI_ISL_1486743, EPI_ISL_1486746, EPI_ISL_1486748, EPI_ISL_1486751, EPI_ISL_1486755, EPI_ISL_1486758, EPI_ISL_1486760, EPI_ISL_1486764, EPI_ISL_1486767, EPI_ISL_1486770, EPI_ISL_1486773, EPI_ISL_1486776, EPI_ISL_1486779, EPI_ISL_1486782, EPI_ISL_1486785, EPI_ISL_1486787, EPI_ISL_1486791, EPI_ISL_1486793, EPI_ISL_1503828 to 1503855, EPI_ISL_1503857, EPI_ISL_1503859 to 1503881, EPI_ISL_1503883 to 1503902, EPI_ISL_1503904 to 1503913, EPI_ISL_1503916 to 1503920, EPI_ISL_1547706 to 1547748, EPI_ISL_1547750 to 1547798			
see above	Microbiology Department, Laboratori Clínic Metropolitana Nord. Hospital Universitari Germans Trias i Pujol.	Can Ruti SARS-CoV-2 Sequencing Hub (HUGTiP/IrsiCaixa/IGTP)	Alba Sánchez; Anna Not; Antoni E Bordoy; Bonaventura Clotet; Cristina Casañ; Cristina Esteban; Francesc Catala-Moll; Gemma Clara; Ignacio Blanco; Marc Noguera-Julian; Maria Casadellà; Mariona Parera; Mercedes Guerrero; Montserrat Giménez; Pere-Joan Cardona; Pilar Armengol; Roger Paredes; Verónica Saludes; and Elisa Martró on behalf of the Can Ruti SARS-CoV-2 Sequencing Hub.
EPI_ISL_1495974 to 1495979, EPI_ISL_1498305 to 1498312, EPI_ISL_1502113 to 1502128, EPI_ISL_1502199 to 1502210, EPI_ISL_1509952 to 1509959	Microbiology Department, University Hospital Donostia	Microbiology Department, University Hospital Donostia	Cilla G.; Gomez M; Marimon JM; Montes M; Piñeiro L; Sorarrain A
EPI_ISL_1478754, EPI_ISL_1478757 to 1478843, EPI_ISL_1483319 to 1483320, EPI_ISL_1532792 to 1532799	Microbiology Department. Complexo Hospitalario Universitario de Vigo	Microbiology Department. Complexo Hospitalario Universitario de Vigo	Alfaia N; Alfaya N; Alonso I; Alvarez M; Cabrera JJ; Carballo R; Cores O; Cortizo S; Martinez L; Mediero G; Perez S; Potel C; Regueiro B; Rey S; Vasallo FJ; del-Campo V
EPI_ISL_462991	Microbiology Division	Microbiology Division	Flores, H.
EPI_ISL_529080 to 529108	Microbiology Division, SC DHEC	Microbiology Division, SC DHEC	Flores, H.
EPI_ISL_447281 to 447310	Microbiology Division, Barzilai University Medical Center	Stern Lab	Stern Lab
EPI_ISL_510537	Microbiology Division, SC DHEC	Microbiology Division, SC DHEC	Flores, H.
EPI_ISL_538390 to 538434, EPI_ISL_539879, EPI_ISL_541663 to 541679, EPI_ISL_574543 to 574575	Microbiology Division, South Carolina Department of Health and Environmental Control	Microbiology Division, South Carolina Department of Health and Environmental Control	Flores, H.
EPI_ISL_468331 to 468344	Microbiology Service, University Hospital of A Coruna-Biomedical Research Institute	Genomes & Disease, Center for Research in Molecular Medicine and Chronic Diseases, University of Santiago de Compostela	Ana Pequeno-Valtierra; Cristina Gomez; Daniel Garcia-Souto; G Bou; Javier Temes; Jorge Arca; Jorge Rodriguez-Castro; Jose M. C. Tubio; Juan A. Vallejo; Kelly Conde; M Poza; Martin Santamarina; Soraya Rumbo
EPI_ISL_455312 to 455313	Microbiology Unit, Department of Pathology & Laboratory Medicine, IIUM Medical Centre	SEA Microbiome Unit, Faculty of Industrial Sciences & Technology, Universiti Malaysia Pahang	Ahmad Hafiz Bin Zulkifly; Ahmad Hafiz Zulkifly; Ahmad Mahfuz Gazali; Hajar Fauzan Ahmad; IIUM Medical Centre Covid19 Taskforce; Mohd Fazli Farida Asras; Mohd Nazli Bin Kamarulzaman; Muhammad Adam Lee Abdullah; Norhidayah Binti Kamarudin; Norhidayah Kamarudin; UMP Covid19 Team
EPI_ISL_418267, EPI_ISL_418269	Microbiology and Immunology department, Pasteur institute in Ho Chi Minh city	Microbiology and Immunology department, Pasteur institute in Ho Chi Minh city	A.H.; C.Q.; Cao; D.T.; Dao; H.Q.; H.T.; H.T.T.; Hoang; Huynh; K.C.; L.T.; L.T. and Nguyen; L.T.K.; Le; Luong; M.H.; N.P.H.; N.T.; Nguyen; Pham; Phan; Phung; Q.C.; Q.D.; S.N.; T.M.; T.N.A.; T.T.; T.T.N.; T.V.; T.V. and Phan; Tran; Tran, T.; Vo; Vu
EPI_ISL_509424 to 509429	Microbiology and Immunology, University of South Alabama	Microbiology and Immunology, University of South Alabama	Borchert; Fouty, B.; G.M.; Houserova, D.; J.O.; R.A.; R.R.; Rayner; Roberts; Wood
EPI_ISL_468067	Microbiology and Immunology, The Peter Doherty Institute for Infection and Immunity	Microbiology and Immunology, The Peter Doherty Institute for Infection and Immunity	Antonia da Costa, E.; Barreto, I.; Caly, L.; Canisia, D.; Dakh, F.; Dolores de Jesus da Costa, M.; Douglas; Druce, J.; Francis, J.; Freeman, K.; Jayanti Pereira Tilman, A.; M.B.; Marr, I.; Meumann, E.; N. and Baird, R.; Sait, M.; Salles de Sousa, A.; Schultz; Seemann, T.; Sherry, N.; Soares da Silva, E.; Wapling, J.; Ximenes, J.
EPI_ISL_1416330 to 1416331, EPI_ISL_1417439 to 1417441	Microbiology and Virology Laboratory, "Policlinico Riuniti, Azienda Ospedaliero Universitaria, Foggia"	Microbiology and Virology Laboratory, "Policlinico Riuniti, Azienda Ospedaliero Universitaria, Foggia"	Annamaria D'Aprile; Daniela Pisanelli; Donatella Cedola; Fabio Arena; Loris Micelli; Maria Pia Patrizio; Maria Rosaria Lipsi; Maurizio Margaglione and Rosella De Nittis.; Settimia Altamura; Tiziana Rollo; Valeria Delli Carri
EPI_ISL_447258 to 447280	Microbiology laboratory, Assuta Ashdod University-Affiliated Hospital	Stern Lab	Stern Lab
EPI_ISL_613459 to 613462	Microbiology, Koc University	Microbiology, Koc University	Can, F.; Dogan, O.; Kuskucu; M.A.; Nurtop, E.; Ozer, B.
EPI_ISL_513585 to 513588	Microbiology, Pathology, Kettering General Hospital	Wellcome Sanger Institute for the COVID-19 Genomics UK (COG-UK) consortium	Anita Kenyon; Cordelia Langford; David K. Jackson; Dominic Kwiatkowski; Ewan Harrison; Ian Johnston; John Sillitoe on behalf of the Wellcome Sanger Institute COVID-19 Surveillance Team (http://www.sanger.ac.uk/covid-team); Roberto Amato; Sahar El-dirdiri; Sonia Goncalves; Thomas Davis and Alex Alderton
EPI_ISL_434571	Microbiology, The University of Hong Kong	Microbiology, The University of Hong Kong	Chan; J.F.W. and Yuen; K.-Y.
EPI_ISL_475717 to 475721	Microbiology, University Hospital Donostia	Microbiology, University Hospital Donostia	Cilla, G.; J.M.; Marimon; Montes, M.; Pineiro, L.
EPI_ISL_429874, EPI_ISL_486646 to 486665	Microbiology, Virology and Biemergency Laboratory-ASST FBF Sacco	Microbiology, Virology and Biemergency Laboratory-ASST FBF Sacco	Comandatore F; Mancon A; Micheli V; Rimoldi SG; Romeri F; Stefani F
EPI_ISL_456213 to 456214, EPI_ISL_456255 to 456258, EPI_ISL_456376, EPI_ISL_456385 to 456387, EPI_ISL_456403, EPI_ISL_548001 to 548012, EPI_ISL_548031 to 548039, EPI_ISL_548052 to 548056, EPI_ISL_548074, EPI_ISL_548081 to 548082, EPI_ISL_548094 to 548097, EPI_ISL_548103, EPI_ISL_548112 to 548115, EPI_ISL_548121, EPI_ISL_548123 to 548125, EPI_ISL_548127, EPI_ISL_548130, EPI_ISL_548132 to 548133, EPI_ISL_548144 to 548146, EPI_ISL_579100 to 579104, EPI_ISL_579129 to 579132, EPI_ISL_579228 to 579232, EPI_ISL_579301 to 579314, EPI_ISL_579404, EPI_ISL_1469089, EPI_ISL_1469092, EPI_ISL_1469096, EPI_ISL_1469105			
see above	Middlemore Hospital	Institute of Environmental Science and Research (ESR)	Anja Werno; Antje van der Linden; Arlo Upton; Chris Mansell; David Hammer; Dragana Drinkovic; Erasmus Smit; Gary McAuliffe; Hana Sofia Andersson; Hermes Perez; James Ussher; Jill Sherwood; Jing Wang; Joep de Ligt; Josh Freeman; Julia Howard; Juliet Elvy; Lauren Jelly; Mary DeAlmeida; Matt Blakiston; Matt Storey; Matthew Rogers; Max Bloomfield; Michael Addidle; Michelle Balm; Muhammad Faisal; Nikki Freed; Olin Silander; Olivia Stroeven; Rachel Boyle; Sally Roberts; SallyAnn Harbison; Sarah Jefferies; Sharmini Muttaiyah; Susan Morpeth; Susan Taylor; Timothy Blackmore; Vani Sathyendran; Veronica Playle; Virginia Hope; Xiaoyun Ren
EPI_ISL_417413, EPI_ISL_428712 to 428723, EPI_ISL_429861 to 429873, EPI_ISL_437304 to 437335, EPI_ISL_1386007, EPI_ISL_1386009, EPI_ISL_1386011, EPI_ISL_1386013 to 1386014, EPI_ISL_1386016, EPI_ISL_1386018, EPI_ISL_1386020 to 1386021, EPI_ISL_1386023, EPI_ISL_1386025, EPI_ISL_1386027 to 1386028, EPI_ISL_1386030, EPI_ISL_1386032 to 1386033, EPI_ISL_1403686 to 1403779, EPI_ISL_1533611 to 1533688, EPI_ISL_1534462 to 1534527			
see above	Ministry of Health Turkey	Ministry of Health Turkey	Aye Baak Alta; Fatma Bayrakdar; Gulay Korukluoglu; Gülay Korukluolu; Selçuk Klç; Suleyman Yalcin; Süleyman Yalcin; Süleyman Yalçn; Tülin Demir; Yasemin Cosgun; Yasemin Cogun
EPI_ISL_427404 to 427412, EPI_ISL_427414 to 427420	Ministry of Public Health (MoPH)	Biomedical Research Center (BRC)	Abdullatif Al-Khal; Ajaeb D. M. H. Al-Nabet; Asmaa A. Al-Thani.; Einas A. E. Al-Kuwari; Fatiha M. Benslimane; Hadi M. Yassine; Hamad E. Al-Romaihi; Heba A. Al-Khatib; Mohammed Al-Thani; Muna A. S. Al-Maslamani; Nourah B. M. Younes; Peter V. Coyle; Salih Al-Marri; Sonia Boughattas
EPI_ISL_414588 to 414590, EPI_ISL_417189, EPI_ISL_417191 to 417192, EPI_ISL_417194, EPI_ISL_417196, EPI_ISL_417198, EPI_ISL_417469 to 417480, EPI_ISL_417492 to 417503, EPI_ISL_419388, EPI_ISL_419390 to 419397, EPI_ISL_421683 to 421692, EPI_ISL_427273 to 427287, EPI_ISL_437360 to 437386, EPI_ISL_450748 to 450780, EPI_ISL_460045 to 460078, EPI_ISL_462845 to 462911, EPI_ISL_470747 to 470789, EPI_ISL_476706 to 476766, EPI_ISL_482946 to 482966, EPI_ISL_482988 to 483017, EPI_ISL_484692, EPI_ISL_495610 to 495628, EPI_ISL_496916 to 496917, EPI_ISL_496919 to 496930, EPI_ISL_507934 to 507956, EPI_ISL_507973 to 507983, EPI_ISL_514616 to 514618, EPI_ISL_514658 to 514672, EPI_ISL_515262, EPI_ISL_515269, EPI_ISL_518855, EPI_ISL_527573 to 527629, EPI_ISL_530172 to 530224, EPI_ISL_539824 to 539841, EPI_ISL_560743 to 560791, EPI_ISL_576228 to 576256, EPI_ISL_590753 to 590822, EPI_ISL_590837, EPI_ISL_602956 to 602999, EPI_ISL_603006 to 603009, EPI_ISL_603015, EPI_ISL_603018 to 603020, EPI_ISL_1402708 to 1402724, EPI_ISL_1402726 to 1402740, EPI_ISL_1423516 to 1423533, EPI_ISL_1445988, EPI_ISL_1445994, EPI_ISL_1445997, EPI_ISL_1446003 to 1446004, EPI_ISL_1446006, EPI_ISL_1446014, EPI_ISL_1446017, EPI_ISL_1446020 to 1446021, EPI_ISL_1446030 to 1446031, EPI_ISL_1446033 to 1446034, EPI_ISL_1446036 to 1446037, EPI_ISL_1446039 to 1446040, EPI_ISL_1446042 to 1446043, EPI_ISL_1446045 to 1446046, EPI_ISL_1479018 to 1479041, EPI_ISL_1479134 to 1479136, EPI_ISL_1479140 to 1479159, EPI_ISL_1499652 to 1499685, EPI_ISL_1531573 to 1531633			
see above	Minnesota Department of Health, Public Health Laboratory	Minnesota Department of Health, Public Health Laboratory	Alexandra Lorentz; Jacob Garfin; Jacob Garfin and Xiong Wang; Jake Garfin and Xiong Wang; Matt Plumb; Xiong Wang; and Xiong Wang

EPI_ISL_1392807 to 1392888, EPI_ISL_1489843 to 1489923	Missouri State Public Health Laboratory	Missouri State Public Health Laboratory	Ashley New; Joshua Barry; Matthew Sinn
EPI_ISL_1534321, EPI_ISL_1534426 to 1534427, EPI_ISL_1534435, EPI_ISL_1534447	Mitchells Plain Hospital wc MPH	NHLS/UCT	Arash Iranzadeh; Bruna Galvao; Carolyn Williamson; Deelan Doolabh; Diana Hardie; Emmanuel SJ; Innocent Mudau; Kruger Marais; Lynn Tyers; Marvin Hsiao; Stephen Korsman; Tegally H; de Oliveira T
EPI_ISL_458079	Mitra Keluarga Hospital Kenjeran	Institute of Tropical Disease, Universitas Airlangga	Aldise M Nastri; Anastasia W Jefuna; Gatot Soegiarto; Jezzy R Dewantari; Kazufumi Shimizu; Krisnoadi Rahardjo; Laksmi Wulandari; Maria I Lusida; Mitsuhiro Nishimura; Resti Yudhawati; Retno A Setyoningrum; Rima R Prasetya; Soetjipto; Yasuko Mori; Yohko K Shimizu
EPI_ISL_437192	Mitra Keluarga Kelapa Gading Hospital	Eijkman Institute for Molecular Biology, Ministry of Research and Technology/National Agency for Research and Innovation	Amin Soebandrio; David H Muljono; Edison Johar; Frilasita A Yudhaputri; Hidayat Trimarsanto; Khin Saw Myint; Safarina G Malik
EPI_ISL_435119	Mohammed Bin Rashid University of Medicine and Health Sciences	Al Jalila Children's Hospital	Abdulmajeed Alkhaja; Abiola Catherine Senok; Ahmad Abou Tayoun; Alawi Alsheikh-Ali; Divinlal Harilal; Hamda Khansaheb; Hanan Al Suwaidi; Mohammed Uddin; Norbert Nowotny; Qutayba Hamid; Rabih Halwani; Rifat Hamoudi; Rupa Murthy Varghese; Sathishkumar Ramaswamy; Tom Loney; Zulfa Omar Deesi
EPI_ISL_435120 to 435143, EPI_ISL_463740, EPI_ISL_469276 to 469281, EPI_ISL_520663 to 520744, EPI_ISL_523952 to 523953	Mohammed Bin Rashid University of Medicine and Health Sciences	Al Jalila Genomics Center	Abdulmajeed Alkhaja; Abiola Catherine Senok; Ahmad Abou Tayoun; Alawi Alsheikh-Ali; Divinlal Harilal; Hamda Khansaheb; Hanan Al Suwaidi; Mohammed Uddin; Norbert Nowotny; Qutayba Hamid; Rabih Halwani; Rifat Hamoudi; Rupa Murthy Varghese; Sathishkumar Ramaswamy; Tom Loney; Zulfa Omar Deesi
EPI_ISL_419313	Molecular Biology and Biotechnology Lab II	Molecular Biology and Biotechnology Lab II	Aimen Malik; Azka Noureen; Kumail Ali Rizvi; Muhammad Javaid Asad; Sana Shamshad; Tayyaba Zainab
EPI_ISL_421573 to 421576	Molecular Diagnostic Services	KRISP, KZN Research Innovation and Sequencing Platform	Chimukangara B; Deforche K; Giandhari J; Lessells R; Ngcapu S; Pillay S; Samsunder N; Tegally H; Wilkinson E; de Oliveira T
EPI_ISL_421572	Molecular Diagnostic Services and FLowpath	KRISP, KZN Research Innovation and Sequencing Platform	Chimukangara B; Deforche K; Giandhari J; Lessells R; Ngcapu S; Pillay S; Samsunder N; Tegally H; Wilkinson E; de Oliveira T
EPI_ISL_467431, EPI_ISL_467444 to 467448, EPI_ISL_467475 to 467491, EPI_ISL_467494 to 467506, EPI_ISL_482702 to 482709, EPI_ISL_482714 to 482723, EPI_ISL_482851 to 482872, EPI_ISL_487329 to 487341			
see above	Molecular Diagnostics Services (MDS)	KRISP, KZN Research Innovation and Sequencing Platform	Chimukangara B; Giandhari J; Khan S; Lessells R; Mdlalose K; Pillay S; Tegally H; Wilkinson E; York D; de Oliveira T
EPI_ISL_462995 to 463000	Molecular Genetics	Molecular Genetics	Coto, E.; Gomez, J.
EPI_ISL_444455, EPI_ISL_444519	Molecular Infectious Disease	Molecular Infectious Disease	Anderson; B.P.; Gerasimova, A.; Kagan; R.M. and Owen, R.; Rosenthal; S.H.
EPI_ISL_430018, EPI_ISL_430063, EPI_ISL_451957, EPI_ISL_498193, EPI_ISL_578701 to 578702	Molecular Pathology Division, Department of Pathology, Hong Kong Sanatorium & Hospital	Molecular Pathology Division, Department of Pathology, Hong Kong Sanatorium & Hospital	Chun Hang AU; Dona N. HO; Edmond S.K. MA; Ho Yin LAM; Jonpaul S.T. ZEE; Simon Y.M. LAM; Tsun Leung CHAN; Wai Sing CHAN
EPI_ISL_451306 to 451309, EPI_ISL_460079 to 460095	Molecular Virology Unit, Fondazione IRCCS Policlinico San Matteo , Pavia	Laboratory of Virology, INMI Lazzaro Spallanzani IRCCS	Antonino Di Caro; Antonio Piralla; Barbara Bartolini; Cesare E.M. Gruber; Fausto Baldanti; Maria R. Capobianchi; Martina Rueca
EPI_ISL_1403609 to 1403611	Molecular Virology Unit, Microbiology and Virology Depertment, Fondazione IRCCS Policlinico San Matteo, Pavia	Molecular Virology Unit, Microbiology and Virology Depertment, Fondazione IRCCS Policlinico San Matteo, Pavia	Antonio Piralla; Fausto Baldanti; Federica Giardina; Gherard Batisti Biffignandi; Guglielmo Ferrari; Stefania Paolucci; Stefano Gaiarsa
EPI_ISL_510535	Molecular Virology, Instituto Carlos Chagas / Fiocruz Paraná	Universidade Federal do Parana (UFPR)	Duarte dos Santos, C.; Raboni, S.; Suzukawa, A.; Tscha, M.; Zanluca, C.
EPI_ISL_460096, EPI_ISL_460604 to 460605, EPI_ISL_462149 to 462150, EPI_ISL_467774 to 467775, EPI_ISL_470539, EPI_ISL_479620 to 479624, EPI_ISL_486815 to 486829, EPI_ISL_534337 to 534349, EPI_ISL_539299, EPI_ISL_610227 to 610244, EPI_ISL_1510607 to 1510619, EPI_ISL_1527364 to 1527381			
see above	Molecular diagnostic laboratory of Federal Budget Institution of Science "Central Research Institute of Epidemiology" of The Federal Service on Customers' Rights Protection and Human Well-being Surveillance	Group of Genomics and Postgenomic Technologies of Central Research Institute of Epidemilology	Akimkin VG; Berlina YY; Bukharina A.Y.; Bukharina AY; Bulanenko VP; Cherkashina AS; Golubeva AG; Kaptelova VV; Kondrasheva LY; Korneenko EV; Saenko SS; Samoilov AE; Shipulina OY; Sizova TV; Solovyeva ED; Speranskaya AS; Tivanova EV; Valdokhina AV; Zotova MI
EPI_ISL_614347 to 614395	Molecular diagnostic unit for viral haemorrhagic fevers and emerging viruses, Bouaké CHU Laboratory	Project group Epidemology of Highly Pathogenic Microorganisms, Robert Koch-Institute	Adjaratou Traoré; Bamba Fatoumata Touré; Chantal Akoua-Koffi; Coulibaly Mbegnan; Diané Bamourou; Essia Belarbi; Etilé Anoh; Fabian Leendertz; Grit Schubert; Kra Ouffoué; Monemo Pacome; Safiatou Karidioula; Soundélé Maïté
EPI_ISL_1529004	Mon Health Medical Center	WVU and Marshall University Combined Genomics Core Facilities	"James Denvir; Peter Perrotta; Peter Stoilov; Ryan Percifield"; Wesley Kimble
EPI_ISL_406844	Monash Medical Centre	Collaboration between the University of Melbourne at The Peter Doherty Institute for Infection and Immunity, and the Victorian Infectious Disease Reference Laboratory	Caly, L.; Druce; G; J. and Taiaroa; Schultz, M.; Seemann, T.
EPI_ISL_1416327 to 1416328, EPI_ISL_1420823 to 1420825, EPI_ISL_1420828, EPI_ISL_1490470, EPI_ISL_1490650 to 1490651, EPI_ISL_1490654, EPI_ISL_1490659, EPI_ISL_1490662 to 1490665, EPI_ISL_1490668, EPI_ISL_1490670, EPI_ISL_1490710, EPI_ISL_1490712 to 1490713, EPI_ISL_1490715, EPI_ISL_1548039, EPI_ISL_1548042 to 1548043, EPI_ISL_1548073, EPI_ISL_1548075 to 1548077, EPI_ISL_1548111 to 1548116, EPI_ISL_1549139, EPI_ISL_1549168, EPI_ISL_1549478, EPI_ISL_1549727, EPI_ISL_1549987, EPI_ISL_1550159, EPI_ISL_1551694, EPI_ISL_1551952, EPI_ISL_1552084			
see above	Montana Public Health Laboratory	Montana Public Health Laboratory	Carrie Biskupiak; Deborah Gibson; Joy Ritter; Michelle Mozer
EPI_ISL_467945	Montefiore Medical Center, Dept. of Pathology, Clinical Virology	Albert Einstein College of Medicine, Dept. of Microbiology & Immunology, Chandran lab	Amanda Mengotto; Amy S. Fox; Ariel S. Wirchnianski; Catalina Florez; D. Yitzchak Goldstein; Denise Haslwanter; Duncan Kimmel; Ethan Laudermilch; J. Maximilian Fels; Johanna P. Daily; John M. Greally; Karin A. Skalina; Kartik Chandran; Libusha Kelly; M. Eugenia Dieterle; Michael B. Prystowsky; Robert H. Bortz III; Rohit K. Jangra; Ryan Forster; Saad Khan; Shahina B. Maqbool; Wendy A. Szymczak
EPI_ISL_426883 to 426897, EPI_ISL_1391277 to 1391362, EPI_ISL_1391364 to 1391370	Motol University Hospital	Institute of Applied Biotechnologies a.s.	Adam Novotný; Jan Geryk; Kateina Kvapilová; Martin Kašný; Milan Macek; Ondej Brzo; Pavel Devínek; Petr Brož; Petr Klempt; Petr Kvapil
EPI_ISL_1534245, EPI_ISL_1534267	Mowbray Maternity Hospital wc MMH	NHLS/UCT	Arash Iranzadeh; Bruna Galvao; Carolyn Williamson; Deelan Doolabh; Diana Hardie; Innocent Mudau; Kruger Marais; Lynn Tyers; Marvin Hsiao; Stephen Korsman
EPI_ISL_1509262 to 1509269	Murphy Medical Association	Grubaugh Lab - Yale School of Public Health	Anderson Brito; Annie Watkins; Caleb Neal; Chaney Kalinich; Chantal Vogels; Eva Laszlo; Isabel Ott; Jessica Rothman; Joseph Fauver; Mallery Breban; Mary Petrone; Nathan Grubaugh; Steven Murphy; Tara Alpert
EPI_ISL_490205 to 490208	München Klinik Schwabing	MGZ Medical Genetics Center	Dieter A. Wolf; Elke Holinski-Feder
EPI_ISL_510810 to 510816	NA	The Public Health Agency of Sweden	Anna Risberg; Anna-Malin Linde; Karin Tegmark-Wisell; Maria Lind Karlberg; Mattias Haukland; Mia Brytting; Olov Svartstrom; Oskar Karlsson Lindsjo; Petra Edquist; Reza Advani; Sandra Broddesson
EPI_ISL_1528919, EPI_ISL_1528924, EPI_ISL_1529000 to 1529002	NA	WVU and Marshall University Combined Genomics Core Facilities	"James Denvir; Peter Perrotta; Peter Stoilov; Ryan Percifield"; Wesley Kimble
EPI_ISL_1446489, EPI_ISL_1446491, EPI_ISL_1446494, EPI_ISL_1446497, EPI_ISL_1446499, EPI_ISL_1446502, EPI_ISL_1446505, EPI_ISL_1446508, EPI_ISL_1446511, EPI_ISL_1446513, EPI_ISL_1446541, EPI_ISL_1446547 to 1446571, EPI_ISL_1446741 to 1446742, EPI_ISL_1446913 to 1446922, EPI_ISL_1447012, EPI_ISL_1516388 to 1516399, EPI_ISL_1528508 to 1528516			
see above	NC State Laboratory of Public Health	Centers for Disease Control and Prevention Division of Viral Diseases, Pathogen Discovery	Alison Laufer Halpin; Ben L. Rambo-Martin; Clinton R. Paden; Dakota Howard; Darlene Wagner; Dave Wentworth; Dhwani Batra; Jasmine Padilla; Justin Lee; Katie Dillon; Krista Queen; Kristen Knipe; Kristine Lacek; Mark Burroughs; Matthew Schmerer; Mili Sheth; Peter Cook; Sam Shepard; Sarah Nobles; Shoshona Le; Suxiang Tong; Vivien Dugan; Yvette Unoarumhi
EPI_ISL_424871 to 424873, EPI_ISL_426428, EPI_ISL_452124, EPI_ISL_452126, EPI_ISL_540441	NC State Laboratory of Public Health	Pathogen Discovery, Respiratory Viruses Branch, Division of Viral Diseases, Centers for Disease Control and Prevention	Alison S. Laufer Halpin; Anna Montmayeur; Anna Uehara; Christopher A. Elkins; Clinton R. Paden; Haibin Wang; Jing Zhang; Krista Queen; Mary S. Keckler; Rachel Marine; Suxiang Tong; Yan Li; Ying Tao; Zachary Weiner
EPI_ISL_528683 to 528684	NCDC Institute of Genomics and Integrative Biology	NCDC Institute of Genomics and Integrative Biology	Ajit Shewale; Akshay Kanakan; Anurag Agrawal; Azka Khan; Bharathram Uppili; Mahesh S. Dhar; Mohammed Faruq; Partha Rakshit; Pooja Sharma; RadhaKrishnan VS; Rajesh Pandey; Robin Marwal; Sandhya Kabra; Simmi Tiwari; Sujeet Singh; Tushar Nale; Vivekanand A
EPI_ISL_581505 to 581507, EPI_ISL_614157	NCDC/IGIB	NCDC/IGIB	Ajit Shewale; Akshay Kanakan; Anurag Agrawal; Azka Khan; Bharathram Uppili; Mahesh S. Dhar; Mohammed Faruq; Nishu Tyagi; Partha Rakshit; Pooja Sharma; RadhaKrishnan VS; Rajesh Pandey; Robin Marwal; Sandhya Kabra; Simmi Tiwari; Sujeet Singh; Tushar Nale; Vivekanand A

EPI_ISL_1498121, EPI_ISL_1498918, EPI_ISL_1499112	NCSLPH	NCSLPH	Chase K; Glover W; Greene S; Miller MC
EPI_ISL_1446723 to 1446724, EPI_ISL_1446746 to 1446758, EPI_ISL_1446985	ND Dept. of Health Laboratory Services-Microbiology	Centers for Disease Control and Prevention Division of Viral Diseases, Pathogen Discovery	Alison Laufer Halpin; Ben L. Rambo-Martin; Clinton R. Paden; Dakota Howard; Darlene Wagner; Dave Wentworth; Dhwani Batra; Jasmine Padilla; Justin Lee; Katie Dillon; Krista Queen; Kristen Knipe; Kristine Lacek; Mark Burroughs; Matthew Schmerer; Mili Sheth; Peter Cook; Sam Shepard; Sarah Nobles; Shoshona Le; Suxiang Tong; Vivien Dugan; Yvette Unoarumhi
EPI_ISL_1446825 to 1446839, EPI_ISL_1446937, EPI_ISL_1528286 to 1528290	NE Public Health Laboratory	Centers for Disease Control and Prevention Division of Viral Diseases, Pathogen Discovery	Alison Laufer Halpin; Ben L. Rambo-Martin; Clinton R. Paden; Dakota Howard; Darlene Wagner; Dave Wentworth; Dhwani Batra; Jasmine Padilla; Justin Lee; Katie Dillon; Krista Queen; Kristen Knipe; Kristine Lacek; Mark Burroughs; Matthew Schmerer; Mili Sheth; Peter Cook; Sam Shepard; Sarah Nobles; Shoshona Le; Suxiang Tong; Vivien Dugan; Yvette Unoarumhi
EPI_ISL_1448512, EPI_ISL_1448519, EPI_ISL_1478202	NE Public Health Laboratory	Genomics and Discovery, Respiratory Viruses Branch, Division of Viral Diseases, Centers for Disease Control and Prevention	Anna Kelleher; Anna Uehara; Brian Lynch; Clinton R. Paden; Haibin Wang; Han Jia Justin Ng; Jing Zhang; Krista Queen; Peter Cook; Suxiang Tong; Yan Li; Ying Tao
EPI_ISL_424874 to 424875	NE Public Health Laboratory	Pathogen Discovery, Respiratory Viruses Branch, Division of Viral Diseases, Centers for Disease Control and Prevention	Alison S. Laufer Halpin; Anna Uehara; Christopher A. Elkins; Clinton R. Paden; Haibin Wang; Jing Zhang; Krista Queen; Mary S. Keckler; Rachel Marine; Suxiang Tong; Yan Li; Ying Tao
EPI_ISL_605882 to 605923	NGS Lab, DNA SOLUTION LTD.	NGS Lab, DNA SOLUTION LTD.	Chowdhury, M.; H.U.; Haider; Hasan; Hosen; K.N.; Khaleque, A.; Khan; Khan, M.; M.B.; M.F.A.; M.H.; M.I.; M.N.I.; Polol; Rabbi; Rahman, M.; Razu; Sufian, A.
EPI_ISL_420791 to 420792	NH Department of Health and Human Services Public Health Labs	Pathogen Discovery, Respiratory Viruses Branch, Division of Viral Diseases, Centers for Disease Control and Prevention	Alison S. Laufer Halpin; Anne Uehara; Christopher A. Elkins; Clinton R. Paden; Haibin Wang; Jasmine Padilla; Jing Zhang; Justin Lee; Krista Queen; Mary S. Keckler; Rachel Marine; Suxiang Tong; Yan Li; Ying Tao
EPI_ISL_1447059, EPI_ISL_1528493 to 1528496	NH Dept. of Health and Human Services Public Health Labs	Centers for Disease Control and Prevention Division of Viral Diseases, Pathogen Discovery	Alison Laufer Halpin; Ben L. Rambo-Martin; Clinton R. Paden; Dakota Howard; Darlene Wagner; Dave Wentworth; Dhwani Batra; Jasmine Padilla; Justin Lee; Katie Dillon; Krista Queen; Kristen Knipe; Kristine Lacek; Mark Burroughs; Matthew Schmerer; Mili Sheth; Peter Cook; Sam Shepard; Sarah Nobles; Shoshona Le; Suxiang Tong; Vivien Dugan; Yvette Unoarumhi
EPI_ISL_1408892, EPI_ISL_1509930	NH Dept. of Health and Human Services Public Health Labs	Genomics and Discovery, Respiratory Viruses Branch, Division of Viral Diseases, Centers for Disease Control and Prevention	Adam Retchless; Anna Kelleher; Anna Montmayeur; Anna Uehara; Brian Lynch; Clinton R. Paden; Haibin Wang; Han Jia Justin Ng; Jing Zhang; Justin Lee; Krista Queen; Mark Burroughs; Peter Cook; Rachel Marine; Suxiang Tong; Yan Li; Ying Tao
EPI_ISL_424876 to 424877	NH Dept. of Health and Human Services Public Health Labs	Pathogen Discovery, Respiratory Viruses Branch, Division of Viral Diseases, Centers for Disease Control and Prevention	Alison S. Laufer Halpin; Anna Uehara; Christopher A. Elkins; Clinton R. Paden; Haibin Wang; Jing Zhang; Krista Queen; Mary S. Keckler; Rachel Marine; Suxiang Tong; Yan Li; Ying Tao
EPI_ISL_411950, EPI_ISL_411952 to 411953	NHC Key laboratory of Enteric Pathogenic Microbiology, Institute of Pathogenic Microbiology	Jiangsu Provincial Center for Disease Control & Prevention	Baoli Zhu; Bin Wu; Fengcai Zhu; Kangchen Zhao; Lunbiao Cui; Ming Wu; Tao Wu; Xiaojuan Zhu; Yin Chen; Yiyue Ge
EPI_ISL_467436 to 467443, EPI_ISL_467492 to 467493, EPI_ISL_467507 to 467515, EPI_ISL_467517 to 467524, EPI_ISL_482710 to 482713, EPI_ISL_482724 to 482731, EPI_ISL_482848 to 482850, EPI_ISL_487277 to 487328, EPI_ISL_487348, EPI_ISL_495516 to 495534, EPI_ISL_495543 to 495562, EPI_ISL_498054 to 498126, EPI_ISL_509223 to 509371, EPI_ISL_515568 to 515799, EPI_ISL_518033 to 518053, EPI_ISL_529720 to 529803, EPI_ISL_535390 to 535488, EPI_ISL_535496 to 535572, EPI_ISL_602632 to 602930, EPI_ISL_605785 to 605790			
see above	NHLS-IALCH	KRISP, KZN Research Innovation and Sequencing Platform	Chimukangara B; Giandhari J; Khan S; Lessells R; Mdlalose K; Pillay S; Tegally H; Wilkinson E; York D; de Oliveira T
EPI_ISL_459108, EPI_ISL_489581, EPI_ISL_534395, EPI_ISL_534634	NHSGGC West of Scotland Specialist Virology Centre / MRC-University of Glasgow Centre for Virus Research	Wellcome Sanger Institute for the COVID-19 Genomics UK (COG-UK) Consortium	Alasdair MacLean; Alice Broos; Ana da Silva Filipe; Antonia Ho; Cordelia Langford; Daniel Mair; David K. Jackson; David L Robertson; Dominic Kwiatkowski; Elihu Aranday-Cortes; Emma Thomson and Alex Alderton; Ewan Harrison; Ian Johnston; James Shepherd; Jenna Nichols; John Sillitoe on behalf of the Wellcome Sanger Institute COVID-19 Surveillance Team; Joseph Hughes; Kathy Li; Kathy Smollett; Kirstyn Brunker; Kyriaki Nomikou; Lily Tong; Marc Niebel; Natasha Jesudason; Natasha Johnson; Patawee Asamaphan; Rajiv Shah; Richard Orton; Roberto Amato; Rory Gunson; Sarah McDonald; Sonia Goncalves; Sreenu Vattipally; Stephen Carmichael; Yasmin Parr
EPI_ISL_459095 to 459107, EPI_ISL_459109 to 459165, EPI_ISL_459505 to 459724, EPI_ISL_469930 to 470012, EPI_ISL_489380, EPI_ISL_489391 to 489393, EPI_ISL_489396, EPI_ISL_489401, EPI_ISL_489407 to 489408, EPI_ISL_489413, EPI_ISL_489418, EPI_ISL_489429, EPI_ISL_489442, EPI_ISL_489449, EPI_ISL_489455, EPI_ISL_489470, EPI_ISL_489480, EPI_ISL_489489, EPI_ISL_489523, EPI_ISL_489530, EPI_ISL_489536 to 489537, EPI_ISL_489543, EPI_ISL_489547, EPI_ISL_489567, EPI_ISL_489578 to 489580, EPI_ISL_489582 to 489706, EPI_ISL_501633 to 501635, EPI_ISL_524494, EPI_ISL_524496, EPI_ISL_524499 to 524500, EPI_ISL_524504, EPI_ISL_524507, EPI_ISL_524510 to 524514, EPI_ISL_524516 to 524520, EPI_ISL_524523 to 524524, EPI_ISL_524526 to 524527, EPI_ISL_524532 to 524536, EPI_ISL_524539 to 524540, EPI_ISL_524542 to 524543, EPI_ISL_524545, EPI_ISL_524547, EPI_ISL_524549 to 524550, EPI_ISL_524552, EPI_ISL_524554 to 524557, EPI_ISL_524559, EPI_ISL_524561 to 524563, EPI_ISL_524565 to 524567, EPI_ISL_524569, EPI_ISL_524571 to 524572, EPI_ISL_530361, EPI_ISL_530366, EPI_ISL_530370, EPI_ISL_530377, EPI_ISL_530386, EPI_ISL_530388, EPI_ISL_530391, EPI_ISL_530398, EPI_ISL_530410, EPI_ISL_530429, EPI_ISL_530435, EPI_ISL_530440, EPI_ISL_530468, EPI_ISL_530480, EPI_ISL_530488 to 530489, EPI_ISL_530523, EPI_ISL_530528, EPI_ISL_530533, EPI_ISL_530535, EPI_ISL_530542, EPI_ISL_530553, EPI_ISL_530562, EPI_ISL_530596, EPI_ISL_530642, EPI_ISL_530659, EPI_ISL_530673, EPI_ISL_530682, EPI_ISL_530689, EPI_ISL_530708, EPI_ISL_530722, EPI_ISL_530740, EPI_ISL_530743, EPI_ISL_530746, EPI_ISL_530754, EPI_ISL_530764, EPI_ISL_530766 to 530768, EPI_ISL_530826, EPI_ISL_530836, EPI_ISL_530845, EPI_ISL_530862, EPI_ISL_530871, EPI_ISL_530873, EPI_ISL_530904, EPI_ISL_530906, EPI_ISL_530923, EPI_ISL_530929, EPI_ISL_530953, EPI_ISL_530959, EPI_ISL_530961 to 530962, EPI_ISL_530973, EPI_ISL_530976, EPI_ISL_530979, EPI_ISL_530983, EPI_ISL_530985 to 530986, EPI_ISL_530990, EPI_ISL_530996 to 530997, EPI_ISL_531002, EPI_ISL_531004, EPI_ISL_531006, EPI_ISL_531017, EPI_ISL_531020, EPI_ISL_531032 to 531033, EPI_ISL_531038, EPI_ISL_531040, EPI_ISL_531045, EPI_ISL_531048 to 531049, EPI_ISL_531052, EPI_ISL_531054, EPI_ISL_531056 to 531057, EPI_ISL_531065, EPI_ISL_531079, EPI_ISL_531083, EPI_ISL_531087, EPI_ISL_531091, EPI_ISL_531096, EPI_ISL_531099 to 531100, EPI_ISL_531103 to 531104, EPI_ISL_531114, EPI_ISL_531116, EPI_ISL_531118, EPI_ISL_531123, EPI_ISL_531126, EPI_ISL_531130 to 531131, EPI_ISL_531142, EPI_ISL_531147, EPI_ISL_531154 to 531157, EPI_ISL_531159, EPI_ISL_531166, EPI_ISL_531174 to 531175, EPI_ISL_531188 to 531189, EPI_ISL_531198, EPI_ISL_531207, EPI_ISL_531212, EPI_ISL_531214, EPI_ISL_531223, EPI_ISL_531249, EPI_ISL_531266, EPI_ISL_531321, EPI_ISL_531330, EPI_ISL_531365, EPI_ISL_531374, EPI_ISL_531382, EPI_ISL_531412, EPI_ISL_531416, EPI_ISL_531420, EPI_ISL_531422, EPI_ISL_531432, EPI_ISL_531442, EPI_ISL_531464, EPI_ISL_531470, EPI_ISL_531473, EPI_ISL_531476 to 531477, EPI_ISL_531480, EPI_ISL_531483 to 531485, EPI_ISL_531492, EPI_ISL_531495, EPI_ISL_531500, EPI_ISL_531504, EPI_ISL_531524, EPI_ISL_531546, EPI_ISL_531548, EPI_ISL_531557, EPI_ISL_531563 to 531565, EPI_ISL_531569 to 531570, EPI_ISL_531575, EPI_ISL_531577, EPI_ISL_531583, EPI_ISL_531590, EPI_ISL_531595, EPI_ISL_531598 to 531599, EPI_ISL_531607, EPI_ISL_531609, EPI_ISL_531617 to 531618, EPI_ISL_531625, EPI_ISL_531631, EPI_ISL_531634, EPI_ISL_531638 to 531639, EPI_ISL_531649, EPI_ISL_531660, EPI_ISL_531668, EPI_ISL_531672, EPI_ISL_531677 to 531678, EPI_ISL_531687, EPI_ISL_531689 to 531691, EPI_ISL_531702 to 531703, EPI_ISL_531714, EPI_ISL_531727, EPI_ISL_531730, EPI_ISL_531732, EPI_ISL_531743, EPI_ISL_531753 to 531755, EPI_ISL_531758, EPI_ISL_531768, EPI_ISL_531779, EPI_ISL_531788, EPI_ISL_531792, EPI_ISL_531801 to 531802, EPI_ISL_531805, EPI_ISL_531814 to 531815, EPI_ISL_531821, EPI_ISL_531824, EPI_ISL_531832, EPI_ISL_531842, EPI_ISL_531851, EPI_ISL_531856 to 531858, EPI_ISL_531862 to 531863, EPI_ISL_531865, EPI_ISL_531873, EPI_ISL_531877, EPI_ISL_531880 to 531881, EPI_ISL_531884, EPI_ISL_531887, EPI_ISL_531889 to 531890, EPI_ISL_531892, EPI_ISL_531894, EPI_ISL_531905, EPI_ISL_531912 to 531913, EPI_ISL_531918 to 531919, EPI_ISL_531925, EPI_ISL_531929, EPI_ISL_531932 to 531933, EPI_ISL_531935 to 531936, EPI_ISL_531943, EPI_ISL_531945, EPI_ISL_531951, EPI_ISL_531954, EPI_ISL_531963, EPI_ISL_531973, EPI_ISL_531978 to 531979, EPI_ISL_531982, EPI_ISL_531984, EPI_ISL_531987, EPI_ISL_531990, EPI_ISL_531995, EPI_ISL_532003 to 532005, EPI_ISL_532007, EPI_ISL_532018, EPI_ISL_532024, EPI_ISL_532032, EPI_ISL_532038, EPI_ISL_532049, EPI_ISL_532053, EPI_ISL_532055, EPI_ISL_532057, EPI_ISL_532062, EPI_ISL_532070 to 532071, EPI_ISL_532076 to 532078, EPI_ISL_532080 to 532081, EPI_ISL_532085, EPI_ISL_532093 to 532094, EPI_ISL_532098, EPI_ISL_532102, EPI_ISL_532105, EPI_ISL_532118, EPI_ISL_532123, EPI_ISL_532125, EPI_ISL_532129 to 532131, EPI_ISL_532138, EPI_ISL_532153, EPI_ISL_532168, EPI_ISL_532178, EPI_ISL_532186, EPI_ISL_532192, EPI_ISL_532194 to 532197, EPI_ISL_532200, EPI_ISL_532202 to 532203, EPI_ISL_532210, EPI_ISL_532214, EPI_ISL_532219, EPI_ISL_532221, EPI_ISL_532235, EPI_ISL_532241, EPI_ISL_532244, EPI_ISL_532249, EPI_ISL_532258, EPI_ISL_532263, EPI_ISL_532271 to 532272, EPI_ISL_532275 to 532276, EPI_ISL_532278, EPI_ISL_532283, EPI_ISL_532288, EPI_ISL_532293, EPI_ISL_532301, EPI_ISL_532311, EPI_ISL_532316 to 532317, EPI_ISL_532321, EPI_ISL_532327 to 532328, EPI_ISL_532334 to 532335, EPI_ISL_532338, EPI_ISL_532340, EPI_ISL_532342, EPI_ISL_532347, EPI_ISL_532349, EPI_ISL_532351, EPI_ISL_532353, EPI_ISL_532355 to 532356, EPI_ISL_532363 to 532364, EPI_ISL_532379, EPI_ISL_532384, EPI_ISL_532388, EPI_ISL_532390, EPI_ISL_532394, EPI_ISL_532399, EPI_ISL_532402, EPI_ISL_532404, EPI_ISL_532414, EPI_ISL_532430 to 532432, EPI_ISL_532438, EPI_ISL_532440, EPI_ISL_532444, EPI_ISL_532446, EPI_ISL_532452, EPI_ISL_532454, EPI_ISL_532456, EPI_ISL_532463 to 532464, EPI_ISL_532469, EPI_ISL_532472 to 532473, EPI_ISL_532480, EPI_ISL_532484, EPI_ISL_532490, EPI_ISL_532492 to 532493, EPI_ISL_532506, EPI_ISL_532510, EPI_ISL_532512, EPI_ISL_532514 to 532515, EPI_ISL_532518, EPI_ISL_532523, EPI_ISL_532531, EPI_ISL_532538, EPI_ISL_532540, EPI_ISL_532563 to 532564, EPI_ISL_532572, EPI_ISL_532582, EPI_ISL_532587, EPI_ISL_532594, EPI_ISL_532598, EPI_ISL_532620 to 532621, EPI_ISL_532625 to 532626, EPI_ISL_532633, EPI_ISL_532639, EPI_ISL_532648, EPI_ISL_532670, EPI_ISL_532697, EPI_ISL_532715, EPI_ISL_532733, EPI_ISL_532738, EPI_ISL_532744, EPI_ISL_532746, EPI_ISL_532750, EPI_ISL_532753, EPI_ISL_532755, EPI_ISL_532765, EPI_ISL_532767, EPI_ISL_532784, EPI_ISL_532786, EPI_ISL_532791, EPI_ISL_532811, EPI_ISL_532835, EPI_ISL_532838, EPI_ISL_532844, EPI_ISL_532858, EPI_ISL_532864 to 532865, EPI_ISL_532874 to 532876, EPI_ISL_532878, EPI_ISL_532885 to 532886, EPI_ISL_532891, EPI_ISL_532894, EPI_ISL_532896, EPI_ISL_532905 to 532906, EPI_ISL_532915, EPI_ISL_532932 to 532933, EPI_ISL_532948, EPI_ISL_532962, EPI_ISL_532988, EPI_ISL_533000, EPI_ISL_533016, EPI_ISL_533027, EPI_ISL_533072, EPI_ISL_533228, EPI_ISL_533230 to 533232, EPI_ISL_533236 to 533237, EPI_ISL_533241, EPI_ISL_533252, EPI_ISL_533256, EPI_ISL_533262, EPI_ISL_533270 to 533271, EPI_ISL_533283, EPI_ISL_533289, EPI_ISL_533298, EPI_ISL_533311, EPI_ISL_533343, EPI_ISL_533346, EPI_ISL_533350 to 533351, EPI_ISL_533355, EPI_ISL_533358, EPI_ISL_533365, EPI_ISL_533376, EPI_ISL_533394, EPI_ISL_533396, EPI_ISL_533406, EPI_ISL_533419, EPI_ISL_533421, EPI_ISL_534365 to 534394, EPI_ISL_534396 to 534633, EPI_ISL_534635 to 534690			
see above	NHSGGC West of Scotland Specialist Virology Centre / MRC-University of Glasgow Centre for Virus Research	Wellcome Sanger Institute for the COVID-19 Genomics UK (COG-UK) consortium	Alasdair MacLean; Alice Broos; Ana da Silva Filipe; Antonia Ho; Cordelia Langford; Daniel Mair; David K. Jackson; David L Robertson; Dominic Kwiatkowski; Elihu Aranday-Cortes; Emma Thomson and Alex Alderton; Ewan Harrison; Ian Johnston; James Shepherd; Jenna Nichols; John Sillitoe; John Sillitoe on behalf of the Wellcome Sanger Institute COVID-19 Surveillance Team (http://www.sanger.ac.uk/covid-team); Joseph Hughes; Kathy Li; Kathy Smollett; Kirstyn Brunker; Kyriaki Nomikou; Lily Tong; Marc Niebel; Natasha Jesudason; Natasha Johnson; Patawee Asamaphan; Rajiv Shah; Richard Orton; Roberto Amato; Rory Gunson; Sarah McDonald; Sonia Goncalves; Sreenu Vattipally; Stephen Carmichael; Yasmin Parr
EPI_ISL_418241 to 418242, EPI_ISL_420037	NIC Viral Respiratory Unit - Institut Pasteur of Algeria	National Reference Center for Viruses of Respiratory Infections, Institut Pasteur, Paris	Angela Brisebarre; Etienne Simon-Lorière; Fawzi Derrar; Flora Donati; Marion Barbet; Maud Vanpeene; Mélanie Albert; Méline Bizard; Sylvie Behillil; Sylvie van der Werf; Vincent Enouf
EPI_ISL_1538519, EPI_ISL_1538849 to 1538853	NIO, Zakad Biochemii i Analityki Klinicznej	1. National Institute of Public Health - National Institute of Hygiene; 2. Eurofins Genomics Europe Sequencing GmbH	ECDC COVID-19 WGS support team; Eurofins Genomics Europe Sequencing Team; Gierczyki Rafa; Sadkowska-Todys Magorzata; Wokowicz Tomasz; Zacharczuk Katarzyna
EPI_ISL_452192 to 452217, EPI_ISL_454521 to 454570, EPI_ISL_479493 to 479573, EPI_ISL_479657 to 479661, EPI_ISL_479776, EPI_ISL_577639 to 577713, EPI_ISL_577716 to 577717, EPI_ISL_577720 to 577731, EPI_ISL_582241			
see above	NIV Influenza	NIV Influenza	Potdar V
EPI_ISL_450321 to 450325	NIV Pune	CSIR-Centre for Cellular and Molecular Biology	A. Awhale; A. Gondhalikar; A. Jagtap; Archana Bharadwaj Siva; B. Minhas S. Bharadwaj; Deepak Kumar; Devi Prasad Vijayashankara; Dhiviya Vedagiri; Disha Nanda; Divya Das; Divya Gupta; Dr ML Choudhary; Dr Priya Abraham; Dr V A Potdar; G. Aditya Kumar; Gangumala Srinivas Reddy; H Kaushal Y. Gurav; H. Kengle; Jotin Gogoi; K. Patel; Karthik Bharadwaj Tallapaka; Koushick Sivakumar; Krishnan Harinivas Harshan; Lamuk Zaveri; M. Kakade; Manish; Manish Bhattacharjee; N Srivastava; Namami Gaur; P Shinde; P. Malsane; Payel Mukherjee; Pooja Ramesh Gupta; Priya Singh; Purushotham

			Vodnala; R. Gunjikar; Rajan Kumar Jha; Rakesh K Mishra; Ravi Prasad Mukku; Renu Sudhakar; S. Bhorekar; S. Digraskar; S. Hundekar; S. Jadhav; S. Salve; S. Tomar; Sakshi Shambhavi; Santosh Kumar Kuncha; Shagufta Khan; Shraddha Vijay Lahoti; Sofia Banu; Somesh Gorde; Sujoy Deb; Swati Bayyana; Tulasi Nagabandi; U. Saha; V Malik; V. Awtade; V. Vipat; Vishal Sah; Yogesh Balakartik; Zeba Rizvi
EPI_ISL_1446412, EPI_ISL_1446415, EPI_ISL_1446436, EPI_ISL_1446444, EPI_ISL_1446712 to 1446713, EPI_ISL_1447027 to 1447028, EPI_ISL_1516172 to 1516187, EPI_ISL_1528326 to 1528338			
see above	NJ Public Health and Environmental Laboratories	Centers for Disease Control and Prevention Division of Viral Diseases, Pathogen Discovery	Alison Laufer Halpin; Ben L. Rambo-Martin; Clinton R. Paden; Dakota Howard; Darlene Wagner; Dave Wentworth; Dhwani Batra; Jasmine Padilla; Justin Lee; Katie Dillon; Krista Queen; Kristen Knipe; Kristine Lacek; Mark Burroughs; Matthew Schmerer; Mili Sheth; Peter Cook; Sam Shepard; Sarah Nobles; Shoshona Le; Suxiang Tong; Vivien Dugan; Yvette Unoarumhi
EPI_ISL_424878, EPI_ISL_424901, EPI_ISL_452116 to 452117	NJ Public Health and Environmental Laboratories	Pathogen Discovery, Respiratory Viruses Branch, Division of Viral Diseases, Centers for Disease Control and Prevention	Alison S. Laufer Halpin; Anna Montmayeur; Anna Uehara; Christopher A. Elkins; Clinton R. Paden; Haibin Wang; Jing Zhang; Krista Queen; Mary S. Keckler; Rachel Marine; Suxiang Tong; Yan Li; Ying Tao; Zachary Weiner
EPI_ISL_1493440 to 1493493, EPI_ISL_1559333 to 1559368	NJDOH, Public Health and Environmental Laboratories	NJ_PHEL	Byeong Jeong; Dana Woell; Jacquelyn Deverell; Lindsey Bodnar; Matthew Scarnati; Mohammad M Ali; Shiv K. Verma
EPI_ISL_1404997 to 1405049, EPI_ISL_1489657 to 1489716	NJDOH, Public Health and Environmental Laboratories	New Jersey Public Health and Environmental Laboratories (NJ PHEL)	Byeong Jeong; Dana Woell; Lindsey Bodnar; Shiv K. Verma
EPI_ISL_1530877 to 1530945	NJDOH, Public Health and Environmental Laboratories	New Jersey Public Health and Environmental Laboratories (NJPHEL)	Byeong Jeong; Dana Woell; Jacquelyn Deverell; Lindsey Bodnar; Matthew Scarnati; Mohammad M Ali; Shiv K. Verma
EPI_ISL_452109, EPI_ISL_594462	NM Department of Health	Pathogen Discovery, Respiratory Viruses Branch, Division of Viral Diseases, Centers for Disease Control and Prevention	Alison S. Laufer Halpin; Anna Montmayeur; Anna Uehara; Christopher A. Elkins; Clinton Paden; Clinton R. Paden; Haibin Wang; Jing Zhang; Julu Bhatnagar; Krista Queen; Mary S. Keckler; Rachel Marine; Suxiang Tong; Yan Li; Ying Tao; Zachary Weiner
EPI_ISL_1516236 to 1516244	NM Dept. Health, Scientific Laboratory Division	Centers for Disease Control and Prevention Division of Viral Diseases, Pathogen Discovery	Alison Laufer Halpin; Ben L. Rambo-Martin; Clinton R. Paden; Dakota Howard; Darlene Wagner; Dave Wentworth; Dhwani Batra; Jasmine Padilla; Justin Lee; Katie Dillon; Krista Queen; Kristen Knipe; Kristine Lacek; Mark Burroughs; Matthew Schmerer; Mili Sheth; Peter Cook; Sam Shepard; Sarah Nobles; Shoshona Le; Suxiang Tong; Vivien Dugan; Yvette Unoarumhi
EPI_ISL_422382, EPI_ISL_422384, EPI_ISL_422387, EPI_ISL_422390, EPI_ISL_422394, EPI_ISL_422397 to 422406	NMIMR, Department of Virology	WACCBIP, University of Ghana	Abraham Kwabena Anang; Augustina Arjaquah; Augustina Arjarquah; Bright Adu; Collins M. Misita; Collins M. Morang'a; Dominic S. Y. Amuzu; Erasmus Kotey; Evelyn Bonney; Fred Tei-Maya; George B. Kyei; Gordon A. Awandare; Ivy Asante; Joyce M. Ngoi; Kofi Bonney; Linda Boatemaa; Miriam Eshun; Peter Quashie; Selassie Kumordjie; Vanessa Magnussen; William Ampofo
EPI_ISL_1540614 to 1540615, EPI_ISL_1540617 to 1540634, EPI_ISL_1540636 to 1540701, EPI_ISL_1540703 to 1540705	NMVRVI	Lithuanian University of Health Sciences Hospital, Department of Genetics and Molecular Medicine	Astra Vitkauskiene; Darius Cereskevicius; Inga Nasvytiene; Mantas Sarauskas; Marius Sukys; Rasa Ugenskiene; Renaldas Jurkevicius; Rima Vainoriene; Zivile Zemeckiene
EPI_ISL_1395750, EPI_ISL_1395753 to 1395754, EPI_ISL_1395756 to 1395758, EPI_ISL_1395760, EPI_ISL_1395763, EPI_ISL_1397393, EPI_ISL_1397395, EPI_ISL_1397403, EPI_ISL_1397405, EPI_ISL_1397407, EPI_ISL_1397409, EPI_ISL_1397650 to 1397693, EPI_ISL_1482346 to 1482353			
see above	NORTH SHORE UNIVERSITY HOSPITAL	Wadsworth Center, New York State Department of Health	Alexis Russel; Alexis Russell; Catharine Prussing; Daryl M. Lamson; Erasmus Schneider; Erica Lasek-Nesselquist; John Kelly; Jonathan Plitnick; Kirsten St. George; Matthew Shudt; Melissa A Leisner; Navjot Singh
EPI_ISL_1395707, EPI_ISL_1397829 to 1397830, EPI_ISL_1397907 to 1397911, EPI_ISL_1398042, EPI_ISL_1398068 to 1398119, EPI_ISL_1462556, EPI_ISL_1462558 to 1462559, EPI_ISL_1462561, EPI_ISL_1462563 to 1462564, EPI_ISL_1462566 to 1462567, EPI_ISL_1462569 to 1462570, EPI_ISL_1462576, EPI_ISL_1462578 to 1462579, EPI_ISL_1462581 to 1462582, EPI_ISL_1462584 to 1462585, EPI_ISL_1462587 to 1462589, EPI_ISL_1462591, EPI_ISL_1462593 to 1462594, EPI_ISL_1462596 to 1462597, EPI_ISL_1462599 to 1462601, EPI_ISL_1462603 to 1462604, EPI_ISL_1462606 to 1462607, EPI_ISL_1462609 to 1462610, EPI_ISL_1462612 to 1462613, EPI_ISL_1462615, EPI_ISL_1462617 to 1462618, EPI_ISL_1462620 to 1462621, EPI_ISL_1462623 to 1462624, EPI_ISL_1465678 to 1465682, EPI_ISL_1465755 to 1465759, EPI_ISL_1465761 to 1465766, EPI_ISL_1465835 to 1465837, EPI_ISL_1482556 to 1482572, EPI_ISL_1482580 to 1482616			
see above	NORTHWELL HEALTH LABORATORIES	Wadsworth Center, New York State Department of Health	Alexis Russel; Alexis Russell; Catharine Prussing; Daryl M. Lamson; Erasmus Schneider; Erica Lasek-Nesselquist; John Kelly; Jonathan Plitnick; Kirsten St. George; Matthew Shudt; Melissa A Leisner; Navjot Singh
EPI_ISL_1434241	NOVELAB VILLEFRANCHE	CNR Virus des Infections Respiratoires - France SUD	Antonin Bal; Bruno Lina; Gregory Destras; Gwendolyne Burfin; Hadrien Regue; Laurence Josset; Martine Valette; Quentin Semanas
EPI_ISL_416742 to 416743	NRL for Influenza, Centrum Epidemiology and Microbiology of National Institute of Public Health, Czech Republic	Charite Universitaetsmedizin Berlin, Institute of Virology	Akexander Nagy; Barbara Muehlemann; Christian Drosten; Dusan Trnka; Helena Jirincova; Jaromira Vecerova; Jorn Beheim-Schwarzbach; Julia Schneider; Ludmila Novakova; Talitha Veith; Terry Jones; Victor M Corman
EPI_ISL_514440 to 514442, EPI_ISL_514580, EPI_ISL_514613 to 514615	NSTU COVID-19 Diagnostic Center	NSU Genome Research Institute (NGRI), North South University	Abdus Sadique; Aura Rahman; Dr. Muhammad Maqsud Hossain; Hasan Mahmud Reza; Jahidul Alam; Md Aminul Islam; Prof; Prof. Abdul Khaleque; Prof. Firoz Ahmed; Prof. Kazi Nadim Hasan; Prof. Md. Didar-Ul-Alam; Tahrima Huq; Tamanna Afroze
EPI_ISL_514434	NSTU COVID-19 Diagnostic Center,	NSU Genome Research Institute (NGRI), North South University	Abdus Sadique; Aura Rahman; Dr. Muhammad Maqsud Hossain; Hasan Mahmud Reza; Jahidul Alam; Md Aminul Islam; Prof; Prof. Abdul Khaleque; Prof. Firoz Ahmed; Prof. Kazi Nadim Hasan; Prof. Md. Didar-Ul-Alam; Tahrima Huq
EPI_ISL_458712, EPI_ISL_492457, EPI_ISL_492467, EPI_ISL_492929, EPI_ISL_492931	NU-OMICS DNA Sequencing research facility, Northumbria University	Wellcome Sanger Institute for the COVID-19 Genomics UK (COG-UK) Consortium	Andrew Nelson; Brendan Payne; Chris Duncan; Clive Graham; Cordelia Langford; Darren Smith and Alex Alderton; David K. Jackson; Debra Padgett; Dominic Kwiatkowski; Edward Barton; Emma Swindells; Ewan Harrison; Garren Scott; Gary Black; Gary Eltringham; Greg Young; Ian Johnston; Jane Greenaway; Jennifer Collins; John Allan; John Sillitoe on behalf of the Wellcome Sanger Institute COVID-19 Surveillance Team; Joshua Loh; Lynn Dover; Matthew Bashton; Paul Baker; Roberto Amato; Sarah Essex; Sheia Waugh; Shirelle Burton-Fanning; Sonia Goncalves; Steve Ligget; Wen Yew; Yusri Taha
EPI_ISL_458578 to 458711, EPI_ISL_458713 to 458718, EPI_ISL_469302 to 469344, EPI_ISL_469805, EPI_ISL_469915, EPI_ISL_488003 to 488186, EPI_ISL_488456 to 488836, EPI_ISL_489064 to 489119, EPI_ISL_489121 to 489155, EPI_ISL_492207 to 492210, EPI_ISL_492450 to 492456, EPI_ISL_492458 to 492466, EPI_ISL_492468 to 492494, EPI_ISL_492916 to 492928, EPI_ISL_492930, EPI_ISL_492932 to 492977			
see above	NU-OMICS DNA Sequencing research facility, Northumbria University	Wellcome Sanger Institute for the COVID-19 Genomics UK (COG-UK) consortium	Andrew Nelson; Brendan Payne; Chris Duncan; Clive Graham; Cordelia Langford; Darren Smith and Alex Alderton; David K. Jackson; Debra Padgett; Dominic Kwiatkowski; Edward Barton; Emma Swindells; Ewan Harrison; Garren Scott; Gary Black; Gary Eltringham; Greg Young; Ian Johnston; Jane Greenaway; Jennifer Collins; John Allan; John Sillitoe on behalf of the Wellcome Sanger Institute COVID-19 Surveillance Team (http://www.sanger.ac.uk/covid-team); Joshua Loh; Lynn Dover; Matthew Bashton; Paul Baker; Roberto Amato; Sarah Essex; Sheia Waugh; Shirelle Burton-Fanning; Sonia Goncalves; Steve Ligget; Wen Yew; Yusri Taha
EPI_ISL_1446743, EPI_ISL_1528420 to 1528423	NV State Public Health Laboratory	Centers for Disease Control and Prevention Division of Viral Diseases, Pathogen Discovery	Alison Laufer Halpin; Ben L. Rambo-Martin; Clinton R. Paden; Dakota Howard; Darlene Wagner; Dave Wentworth; Dhwani Batra; Jasmine Padilla; Justin Lee; Katie Dillon; Krista Queen; Kristen Knipe; Kristine Lacek; Mark Burroughs; Matthew Schmerer; Mili Sheth; Peter Cook; Sam Shepard; Sarah Nobles; Shoshona Le; Suxiang Tong; Vivien Dugan; Yvette Unoarumhi
EPI_ISL_426429	NV State Public Health Laboratory	Pathogen Discovery, Respiratory Viruses Branch, Division of Viral Diseases, Centers for Disease Control and Prevention	Alison S. Laufer Halpin; Anna Uehara; Christopher A. Elkins; Clinton R. Paden; Haibin Wang; Jing Zhang; Krista Queen; Mary S. Keckler; Rachel Marine; Suxiang Tong; Yan Li; Ying Tao
EPI_ISL_424879	NV-Southern Nevada Public Health Laboratory	Pathogen Discovery, Respiratory Viruses Branch, Division of Viral Diseases, Centers for Disease Control and Prevention	Alison S. Laufer Halpin; Anna Uehara; Christopher A. Elkins; Clinton R. Paden; Haibin Wang; Jing Zhang; Krista Queen; Mary S. Keckler; Rachel Marine; Suxiang Tong; Yan Li; Ying Tao
EPI_ISL_1446974 to 1446975, EPI_ISL_1447060 to 1447062, EPI_ISL_1516205 to 1516212	NYC Department of Health and Mental Hygiene	Centers for Disease Control and Prevention Division of Viral Diseases, Pathogen Discovery	Alison Laufer Halpin; Ben L. Rambo-Martin; Clinton R. Paden; Dakota Howard; Darlene Wagner; Dave Wentworth; Dhwani Batra; Jasmine Padilla; Justin Lee; Katie Dillon; Krista Queen; Kristen Knipe; Kristine Lacek; Mark Burroughs; Matthew Schmerer; Mili Sheth; Peter Cook; Sam Shepard; Sarah Nobles; Shoshona Le; Suxiang Tong; Vivien Dugan; Yvette Unoarumhi
EPI_ISL_420793, EPI_ISL_424906, EPI_ISL_436047 to 436082, EPI_ISL_480784 to 480785	NYC Department of Health and Mental Hygiene	Pathogen Discovery, Respiratory Viruses Branch, Division of Viral Diseases, Centers for Disease Control and Prevention	Alison S. Laufer Halpin; Anna Uehara; Anne Uehara; Bettina Bankamp; Christine Mahl; Christopher A. Elkins; Christy Harrison; Clinton R. Paden; Haibin Wang; Jasmine Padilla; Jennifer Rakeman; Jing Zhang; Justin Lee; Krista Queen; Mary S. Keckler; Rachel Marine; Sally Slavinski; Suxiang Tong; Yan Li; Ying Tao; Zachary Weiner
EPI_ISL_1395706, EPI_ISL_1395708 to 1395709, EPI_ISL_1397623 to 1397628, EPI_ISL_1398284 to 1398311, EPI_ISL_1398318 to 1398330, EPI_ISL_1398340 to 1398352, EPI_ISL_1461319 to 1461321, EPI_ISL_1461323 to 1461355, EPI_ISL_1465714 to 1465722, EPI_ISL_1465805 to 1465834, EPI_ISL_1482287 to 1482295			
see above	NYC Pandemic Response Lab	Wadsworth Center, New York State Department of Health	Alexis Russel; Alexis Russell; Catharine Prussing; Daryl M. Lamson; Erasmus Schneider; Erica Lasek-Nesselquist; John Kelly; Jonathan Plitnick; Kirsten St. George; Matthew Shudt; Melissa A Leisner; Navjot Singh
EPI_ISL_594457	NYS Department of Health	Pathogen Discovery, Respiratory Viruses Branch, Division of Viral Diseases, Centers for Disease Control and Prevention	Anna Uehara; Clinton Paden; Haibin Wang; Jing Zhang; Julu Bhatnagar; Krista Queen; Suxiang Tong; Yan Li; Ying Tao
EPI_ISL_416830 to 416832, EPI_ISL_418190 to 418205, EPI_ISL_418254, EPI_ISL_418968 to	NYU Langone Health	Department of Pathology and Medicine, New York University School of Medicine	Adriana Heguy; Amy Rapkiewicz; Antonio Serrano; Christian Marier; Dacia Dimartino; Emily Huang; Gael Westby; George Jour; Guomiao Shen; Jared Pinnell; John Cadley; John Chen; Margaret Black; Maria Aguero-Rosenfeld; Marie Samanovic-Golden; Mark J. Mulligan; Mark Mulligan; Matija Snuderl;

418980			Matt Maurano; Matthew T. Maurano; Megan Hogan; Nick Vulpescu; Paolo Cotzia; Paul Zappile; Peter Meyn; Sitharam Ramaswami; Xiaojun Feng; Yutong Zhang
EPI_ISL_414639, EPI_ISL_419696 to 419705, EPI_ISL_420296 to 420302, EPI_ISL_420307 to 420309, EPI_ISL_420570 to 420582, EPI_ISL_420584 to 420591, EPI_ISL_421577 to 421592, EPI_ISL_421704 to 421733, EPI_ISL_424929 to 424968, EPI_ISL_426617 to 426626, EPI_ISL_427469 to 427521, EPI_ISL_427524 to 427525, EPI_ISL_427627 to 427642, EPI_ISL_428757 to 428805, EPI_ISL_430319 to 430434, EPI_ISL_435475 to 435549, EPI_ISL_435721 to 435722, EPI_ISL_436733 to 436743, EPI_ISL_444613 to 444792, EPI_ISL_450393 to 450402, EPI_ISL_451401 to 451479, EPI_ISL_451665, EPI_ISL_456003 to 456070, EPI_ISL_456107 to 456115, EPI_ISL_458017 to 458022, EPI_ISL_460603, EPI_ISL_467377 to 467422, EPI_ISL_1423376 to 1423503, EPI_ISL_1532731 to 1532785			
see above	NYU Langone Health	Departments of Pathology and Medicine, New York University School of Medicine	Adriana Heguy; Alireza Khodadadi-Jamayran; Amy Rapkiewicz; Andre M. Ribeiro-dos-Santos; Andrew Lytle; Antonio Serrano; Brendan Belovarac; Christian Marier; Dacia Dimartino; Emily Guzman; Emily Huang; Gael Westby; George Jour; Guiqing Wang; Guomiao Shen; Iman Osman; Jared Pinnell; John Cadley; John Chen; Lawrence H. Lin; Ludovic Boytard; Margaret Black; Maria Aguero-Rosenfeld; Marie Samanovic-Golden; Mark J. Mulligan; Matija Snuderl; Matthew T. Maurano; Megan Hogan; Nick Vulpescu; Paolo Cotzia; Paul Zappile; Peter Meyn; Raquel Ordonez Ciriza; Raven Luther; Sitharam Ramaswami; Tatyana Gindin; Theodore Vougiouklakis; Vanessa Raabe; Xiaojun Feng; Yutong Zhang
EPI_ISL_1394175, EPI_ISL_1394202, EPI_ISL_1538637 to 1538638, EPI_ISL_1538821 to 1538823, EPI_ISL_1539062 to 1539064	NZOZ Meditest Diagnostyka Medyczna	1. National Institute of Public Health - National Institute of Hygiene; 2. Eurofins Genomics Europe Sequencing GmbH	ECDC COVID-19 WGS support team; Eurofins Genomics Europe Sequencing Team; Gierczyki Rafa; Sadkowska-Todys Magorzata; Wokowicz Tomasz; Zacharczuk Katarzyna
EPI_ISL_1394068 to 1394075	NZOZ Medyczne Laboratorium Diagnostyczne	1. National Institute of Public Health - National Institute of Hygiene; 2. Eurofins Genomics Europe Sequencing GmbH	ECDC COVID-19 WGS support team; Eurofins Genomics Europe Sequencing Team; Gierczyki Rafa; Sadkowska-Todys Magorzata; Wokowicz Tomasz; Zacharczuk Katarzyna
EPI_ISL_1538565, EPI_ISL_1538567	NZOZ Medyczne Laboratorium Diagnostyczne;	1. National Institute of Public Health - National Institute of Hygiene; 2. Eurofins Genomics Europe Sequencing GmbH	ECDC COVID-19 WGS support team; Eurofins Genomics Europe Sequencing Team; Gierczyki Rafa; Sadkowska-Todys Magorzata; Wokowicz Tomasz; Zacharczuk Katarzyna
EPI_ISL_479997 to 480001	Nagano Environmental Conservation Research Institute	Pathogen Genomics Center, National Institute of Infectious Diseases	Hajime Kamiya; Kentaro Itokawa; Makoto Kuroda; Masanori Hashino; Motoi Suzuki; Naoko Shimodaira; Rina Tanaka; Tsuyoshi Sekizuka
EPI_ISL_480002 to 480003	Nagasaki Prefectural Institute for Environmental Research and Public Health	Pathogen Genomics Center, National Institute of Infectious Diseases	Fumiaki Matsumoto; Hajime Kamiya; Kentaro Itokawa; Makoto Kuroda; Masanori Hashino; Motoi Suzuki; Rina Tanaka; Tsuyoshi Sekizuka
EPI_ISL_480042 to 480064	Nagoya City Public Health Research Institute	Pathogen Genomics Center, National Institute of Infectious Diseases	Hajime Kamiya; Kentaro Itokawa; Makoto Kuroda; Masanori Hashino; Motoi Suzuki; Rina Tanaka; Shinichiro Shibata; Takuya Miki; Tsuyoshi Sekizuka
EPI_ISL_434660, EPI_ISL_434674, EPI_ISL_445229, EPI_ISL_445236, EPI_ISL_450817, EPI_ISL_450823, EPI_ISL_450825, EPI_ISL_452229 to 452230, EPI_ISL_452235, EPI_ISL_452239, EPI_ISL_475565			
see above	Narhalsan Backa vardcentral	The Public Health Agency of Sweden	Anna Risberg; Anna-Malin Linde; Karin Tegmark-Wisell; Maria Lind Karlberg; Mats Olsson; Mattias Haukland; Mia Brytting; Olov Svartstrom; Oskar Karlsson Lindsjo; Reza Advani; Sandra Broddesson; Theresa Enkirch
EPI_ISL_539885, EPI_ISL_582840	Narhalsan Fjallbacka VC	The Public Health Agency of Sweden	Anna Risberg; Anna-Malin Linde; Karin Tegmark-Wisell; Maria Lind Karlberg; Mattias Haukland; Mia Brytting; Olov Svartstrom; Oskar Karlsson Lindsjo; Petra Edquist; Reza Advani; Sandra Broddesson; Theresa Enkirch
EPI_ISL_455107	Narhalsan Mellerud	The Public Health Agency of Sweden	Anna Risberg; Anna-Malin Linde; Karin Tegmark-Wisell; Maria Lind Karlberg; Maria Nykvist; Mia Brytting; Olov Svartstrom; Oskar Karlsson Lindsjo; Theresa Enkirch
EPI_ISL_434670, EPI_ISL_445237, EPI_ISL_450815, EPI_ISL_450820	Narhalsan Molnlycke, Barn och ungdomsmedicin	The Public Health Agency of Sweden	Anna Risberg; Anna-Malin Linde; Karin Tegmark-Wisell; Maria Lind Karlberg; Mats Reimer; Mia Brytting; Olov Svartstrom; Oskar Karlsson Lindsjo; Theresa Enkirch
EPI_ISL_475537	Narhalsan Oden VC	The Public Health Agency of Sweden	Anna Risberg; Anna-Malin Linde; Karin Tegmark-Wisell; Maria Lind Karlberg; Mattias Haukland; Mia Brytting; Olov Svartstrom; Oskar Karlsson Lindsjo; Reza Advani; Sandra Broddesson
EPI_ISL_445224, EPI_ISL_450830, EPI_ISL_469069, EPI_ISL_539882	Narhalsan Olskroken VC	The Public Health Agency of Sweden	Anna Risberg; Anna-Malin Linde; Karin Tegmark-Wisell; Mahin Ghoroghi; Maria Lind Karlberg; Mattias Haukland; Mia Brytting; Olov Svartstrom; Oskar Karlsson Lindsjo; Petra Edquist; Reza Advani; Sandra Broddesson; Shamam Muradrasoli; Theresa Enkirch
EPI_ISL_434671, EPI_ISL_450829, EPI_ISL_452238, EPI_ISL_469058, EPI_ISL_469060 to 469061, EPI_ISL_475524, EPI_ISL_475539			
see above	Narhalsan Sjobo vardcentral	The Public Health Agency of Sweden	Anna Risberg; Anna-Malin Linde; Karin Tegmark-Wisell; Lovisa Hjerten; Maria Lind Karlberg; Mattias Haukland; Mia Brytting; Olov Svartstrom; Oskar Karlsson Lindsjo; Petra Edquist; Reza Advani; Sandra Broddesson; Shamam Muradrasoli; Theresa Enkirch
EPI_ISL_1393840, EPI_ISL_1394080 to 1394082	Narodowy Instytut Onkologii im. Marii Skodowskiej-Curie Pastwowy Instytut Badawczy	1. National Institute of Public Health - National Institute of Hygiene; 2. Eurofins Genomics Europe Sequencing GmbH	ECDC COVID-19 WGS support team; Eurofins Genomics Europe Sequencing Team; Gierczyki Rafa; Sadkowska-Todys Magorzata; Wokowicz Tomasz; Zacharczuk Katarzyna
EPI_ISL_523960, EPI_ISL_523962, EPI_ISL_523964, EPI_ISL_523966, EPI_ISL_523968	National Agency for Public Health, Republic of Moldova	Charite Universitatsmedizin Berlin, Institute of Virology	Ala Halacu; Barbara Muhlemann; Barbara Mühlemann; Christian Drosten; Elizabeta Jancheska; Golubinka Bosevska; Joern Beheim-Schwarzbach; Jorn Beheim-Schwarzbach; Julia Schneider; Maja Kuzmanovska; Mariana Apostol; Talitha Veith; Terry Jones; Victor M Corman
EPI_ISL_454572	National Center of Expertise	National Center for Expertise, Kazakhstan National Center for Biotechnology, Kazakhstan	; Abdaliyev Askar; Akhmetollayev Ilyas; Amirgazin Asylulan; Aushakhmetova Zabira; Kalendar Ruslan; Lutsay Viktoriya; Rakhmetova Akbota; Ramankulov Yerlan; Shevtsov Alexandr
EPI_ISL_454571	National Center of Expertise	National Center for Expertise, National Center for Biotechnology, Kazakhstan	; Abdaliyev Askar; Akhmetollayev Ilyas; Amirgazin Asylulan; Aushakhmetova Zabira; Kalendar Ruslan; Lutsay Viktoriya; Rakhmetova Akbota; Ramankulov Yerlan; Shevtsov Alexandr
EPI_ISL_1401099 to 1401464, EPI_ISL_1406690 to 1406695, EPI_ISL_1509759 to 1509887	National Center of Infectious and Parasitic Diseases	National Center of Infectious and Parasitic Diseases	Alexiev; Alexiev et al; Dimitrova; Dobrinov; Donchev; Grigorova I.; Grigorova L.; Hristova; Ivanov; Kantardjiev; Korsun; Philipova; Stoitsova; Stoykov; Trifonova
EPI_ISL_496524 to 496526, EPI_ISL_496531 to 496532, EPI_ISL_496535 to 496536, EPI_ISL_496555 to 496587, EPI_ISL_497873 to 497879, EPI_ISL_1384818	National Centre For Cell Science	National Centre For Cell Science	Ajay Pillai; Arvind Sahu; DBT's PAN-INDIA 1000 SARS-CoV2 RNA genome sequencing consortium; Dhiraj Paul; Girdhari Lal; Janesh Kumar; Kavita Bala Anand; Kunal Jani; Maharashtra COVID-19 Study Group; Manoj Kumar Bhat; Mitali Inamdar; Mohak P Gujare; Murlidhar Tambe; Radha Chauhan; Rajesh Karyakarte; Rajiv Mohan Gupta; Santosh Karade; Shelinder Pal Singh Shergill; Shivang P. Bhanushali; Sonal Manik Chavan; Sourav Sen; Suvarna Joshi; Vasudevan Seshadri; Yogesh S Shouche; Yogesh Shouche
EPI_ISL_1384813 to 1384817, EPI_ISL_1384819 to 1384858, EPI_ISL_1384860 to 1384862, EPI_ISL_1384864 to 1384884, EPI_ISL_1415086 to 1415128, EPI_ISL_1415132 to 1415133, EPI_ISL_1415137 to 1415156, EPI_ISL_1415158 to 1415292, EPI_ISL_1415294 to 1415391, EPI_ISL_1511198 to 1511202, EPI_ISL_1533729 to 1533801, EPI_ISL_1540451 to 1540457, EPI_ISL_1543988 to 1544074			
see above	National Centre For Cell Science	National Centre For Cell Science - INSACOG	Ajay Pillai; Dhiraj Paul; INSACOG Consortium team; Manoj Kumar Bhat; Mitali Inamdar; Mohak P Gujare; Shivang P. Bhanushali; Sonal Manik Chavan; Yogesh Shouche; Yogesh Shouche.
EPI_ISL_1384859, EPI_ISL_1384863	National Centre For Cell Science	National Institute of Virology, Pune	Ajay Pillai; Dhiraj Paul; Manoj Kumar Bhat; Mitali Inamdar; Mohak P Gujare; Shivang P. Bhanushali; Sonal Manik Chavan; Yogesh Shouche
EPI_ISL_436413 to 436463, EPI_ISL_482491 to 482574, EPI_ISL_482587 to 482671	National Centre for Disease control (NCDC)	NCDC/CSIR-IGIB	Aarti Tewari; Anurag Agrawal*; Bharathram Uppili; Bibhash Nandi; Debasis Dash; Dhirendra Kumar; Hema Gogia; Hemlata Lall; Himanshu Vashisht; Mahesh S Dhar; Manju Bala; Meena Datta; Mitali Mukerji; Mohammed Faruq; Nidhi Saini; Nishu Tyagi; Partha Rakshit*; Pooja Sharma; Poonam Gupta; Pramod Kumar#; Prateek Singh; Preeti Madan; Priyanka Singh; Radhakrishanan VS; Rajesh Pandey#; Robin Marwal; Sandhya Kabra; Saruchi Wadhwa; Satyabrata Bag; Simrita Singh; Sujeet Singh; Uma Sharma; Varun Jaiswal; Vivekanand A
EPI_ISL_435060 to 435112	National Centre for Disease control (NCDC), CSIR-Institute of Genomics and Integrative Biology (CSIR-IGIB)	NCDC/CSIR-IGIB	Aarti Tewari; Anurag Agrawal; Bharathram Uppili; Bibhash Nandi; Debasis Dash; Dhirendra Kumar; Hema Gogia; Hemlata Lall; Himanshu Vashisht; Mahesh Dhar; Manju Bala; Meena Datta; Mitali Mukerji; Mohammed Faruq; Nidhi Saini; Nishu Tyagi; Partha Rakshit; Pooja Sharma; Poonam Gupta; Pramod Kumar; Prateek Singh; Preeti Madan; Priyanka Singh; Rajesh Pandey; Sandhya Kabra; Saruchi Wadhwa; Satyabrata Bag; Simrita Singh; Sujeet Singh; Uma Sharma; Varun Jaiswal; Vivekanand A
EPI_ISL_407988, EPI_ISL_410535, EPI_ISL_414378 to 414380, EPI_ISL_420099 to 420111	National Centre for Infectious Diseases	Programme in Emerging Infectious Diseases, Duke-NUS Medical School	Barnaby E Young; Danielle E Anderson; David CB Lye; Gavin JD Smith; Jayanthi Jayakumar; Linfa Wang; Louisa Sun; Martin Linster; Yan Zhuang; Yee Sin Leo; Yvonne CF Su
EPI_ISL_1384722 to 1384773, EPI_ISL_1396401 to 1396403, EPI_ISL_1398489 to 1398523	National Food and Veterinary Risk Assessment Institute	Vilnius University Hospital Santaros Klinikos, Center of Laboratory Medicine	Daniel Naumovas; Dovile Ezerskyte; Gytis Dudas; Ingrida Olendraite; Laimonas Griskevicius; Ligita Raugaite; Mindaugas Stoskus; Monika Katenaite; Rimvydas Norvilas

EPI_ISL_466839 to 466872, EPI_ISL_528427 to 528429, EPI_ISL_528540 to 528636, EPI_ISL_528880	National Genomics Core-Center for DNA Fingerprinting and Diagnostics	National Genomics Core- Center for DNA Fingerprinting and Diagnostics (NGC-CDFD)- DBT's PAN-INDIA-1000 Genome consortium	ASHWIN DALAL; Abhijeeth Singh Thakur; Ajay Kumar Chaudhary; Akash Chinchole; Akruti Shah; Amrita Bhattacherjee; Arjita Jaiswal; Arpita Singh; Ashwin Dalal; Asodu Sandeep Sarma; B. Krishna Murthy; Bala Pratyusha; Bathula Siddardha; Bharath Kumar; Bhavani Sontam; Binod Bihari Pradhan; Brahmaji Sontyana; C Bala Maddeleti; C. Arun Kumar; Ch Venkateshwar Goud; Chandra Shekhar Singh; Chandra Shekhar V; Chendra Shekar P; Chilakala Gangi Reddy; Chinthakindi KrishnaPrasad; DEBASHIS MITRA; DIVYA VASHISHT; Debashis Mitra; Debashish Mitra; Devanshi Gupta; Devinder Singh Negi; Dhanraj Adey; Divya Vashisht; Edurugatla Dinesh; G Shashikanth; Guru Raja; Heena Shah; Hilal Ahmad Reshi; J. Mallikarjun; Jamal Md Nurul Jain; Jayashree Ladke; Jenige Aravindh Kumar; K. Viswakalyan; K.Manohar; Kaisar Ahmad Lone; Kandali Sreethi Sreenivasulu Reddy; Kausika Kumar Malik; Lavanya Banda; M Sri Lalitha; MURALI DHARAN BASHYAM; Madhumohan Rao; Mohammad Mudassir; Mugdha Singh; Murali Dharan Bashyam; Muthulakshmi; N. Sudheer; Nalini Raghunathan; Nanci Rani K; Neeraj Kumar; Nimmala Naresh; Niteen Pathak; Parveen Kumar; Pradipta Hore; R HARINARAYANAN; R Harinarayanan; R Lakshmi Vaishna; R. Angalena; R. Manorama; RASHNA BHANDARI; Rahul Baroi; Rajeshree Sanyal; Rajitha Ponnala; Raju Kumar; Rashna Bhandari; Reelina Basu; Romila Moirangthem; S Vasantha Rani; SPR Prasad; Sandip Patra; Sanjana Sarkar; Sayantan Goswami; Seyed Khaja Ali; Shaffiqu T S; Shaik Nasar Vali; Shalini Aricthota; Shivangi Wagh; Shivani Yadav; Shruti Dasgupta; Shubhra Ganguli; Sobhan Babu; Srinivas G; Suchitra Upreti; Sunke Vijayakumar; Sunu Joseph; Surya Chodisetty; Surya Vamshi; Swathi Chodisetty; T. Navaneetha; Usha Rani Dutta; V Naga Sailaja; Vani Singh; Vinay Donipadi; Vineesha Oddi; Yogesh Patidar
EPI_ISL_1407095 to 1407253	National HIV Reference Laboratory, Ministry of Health, Public Health Institute of Malawi	KRISP, KZN Research Innovation and Sequencing Platform	Auld A; Chilima B; Chiwaula M; Emmanuel SJ; Giandhari J; Kaba M; Kampira E; Kasambara W; Kim L; Lessells R; Maida A; Mvula B; Mwangomba W; Naidoo Y; Panja L; Pillay S; Tegally H; Wadonda N; Wilkinson E; de Oliveira T
EPI_ISL_560385, EPI_ISL_560387 to 560390	National Health Laboratory	Botswana Institute for Technology Research and Innovation	Dineo Emang Tshiamo; Dineo Emang Tshiamo. Gape Nyepetsi; Gape Nyepetsi; Kefentse Arnold Tumedi; Madisa Mine; Maitshwarelo Ignatius Matsheka; Thongbotho Mphoyakgosi
EPI_ISL_560386	National Health Laboratory	Botswana Institute for Technology Research and innovation	Dineo Emang Tshiamo. Gape Nyepetsi; Kefentse Arnold Tumedi; Madisa Mine; Maitshwarelo Ignatius Matsheka; Thongbotho Mphoyakgosi
EPI_ISL_464112 to 464133, EPI_ISL_464135 to 464158	National Health Laboratory Service (NHLS), Tygerberg	Division of Medical Virology, Stellenbosch University and National Health Laboratory Service (NHLS)	Bronwyn Kleinhans; Eduan Wilkindon; Gert van Zyl; Houriiyah Tegally; Kayla Delaney; Susan Engelbrecht; Tulio de Oliveira; Wolfgang Preiser
EPI_ISL_464134	National Health Laboratory Service (NHLS), Tygerberg	Stellenbosch University and NHLS	Bronwyn Kleinhans; Eduan Wilkindon; Gert van Zyl; Houriiyah Tegally; Kayla Delaney; Susan Engelbrecht; Tulio de Oliveira; Wolfgang Preiser
EPI_ISL_1550912 to 1550962	National Health Laboratory Service, South Africa	KRISP, KZN Research Innovation and Sequencing Platform	Emmanuel SJ; Giandhari J; Khan S; Lessells R; Mdlalose K; Naidoo Y; Pillay S; Ramphal U; Tegally H; Wilkinson E; York D; de Oliveira T
EPI_ISL_480601 to 480602	National Health Laboratory, Timor-Leste	MDU-PHL	Antonia da Costa, E.; Baird, R.; Barreto, I.; Caly, L.; Canisia, D.; Dakh, F.; Dolores de Jesus da Costa, M.; Douglas, N.; Francis, J.; Freeman, K.; Jayanti Pereira Tilman, A.; Marr, I.; Sait, M.; Salles de Sousa, A.; Schultz, M.; Seemann, T.; Sherry, N.; Soares da Silva, E.; Wapling, J.; Ximenes, J.
EPI_ISL_456597 to 456612	National Health Laboratory, Timor-Leste	Microbiological Diagnostic Unit Public Health Laboratory, The Peter Doherty Institute for Infection and Immunity	Antonia da Costa, E.; Baird, R.; Barreto, I.; Caly, L.; Canisia, D.; Dakh, F.; Dolores de Jesus da Costa, M.; Douglas, N.; Francis, J.; Freeman, K.; Jayanti Pereira Tilman, A.; Marr, I.; Sait, M.; Salles de Sousa, A.; Schultz, M.; Seemann, T.; Sherry, N.; Soares da Silva, E.; Wapling, J.; Ximenes, J.
EPI_ISL_435303, EPI_ISL_435305, EPI_ISL_435308, EPI_ISL_435310 to 435317, EPI_ISL_455694 to 455718, EPI_ISL_511891 to 511898	National Hospital of Tropical Diseases	Oxford University Clinical Research Unit, Hanoi, Vietnam	H. Rogier van Doorn; H.Rogier van Doorn; Le Nguyen Minh Hoa; Nguyen Thi Hong Thuong; Nguyen Thi Ngoc Diep; Nguyen Thi Tam; Nguyen Thu Trang; Pham Ngoc Thach; Van Dinh Trang; Vu Thi Ngoc Bich; on behalf of the OUCRU COVID-19 research group
EPI_ISL_416028, EPI_ISL_416031 to 416032, EPI_ISL_416035 to 416036, EPI_ISL_515525	National Influenza Center - Instituto Adolfo Lutz	Instituto Adolfo Lutz, Interdiciplinary Procedures Center, Strategic Laboratory	Adriana Bugno; Adriano Abbud; Carlos Henrique Camargo; Claudia Regina Gonçalves; Claudio Tavares Sacchi; Daniela Bernardes Borges da Silva; Erica Valessa Ramos Gomes; Fabiana Cristina Pereira dos Santos; Maria do Carmo Sampaio Tavares Timenetsky; Simone Guadagnucci Morillo; Terezinha Maria de Paiva
EPI_ISL_408668	National Influenza Center - National Institute of Hygiene and Epidemiology (NIHE)	National Influenza Center - National Institute of Hygiene and Epidemiology (NIHE)	; Hoang Vu Mai Phuong; Le Thi Quynh Mai; Le Thi Thanh; Nguyen Le Khanh Hang; Nguyen Phuong Anh; Nguyen Vu Son; Pham Thi Hien; Tran Thu Huong; Ung Thi Hong Trang; Vuong Duc Cuong
EPI_ISL_486887 to 486889	National Influenza Center, Bahrain	National Influenza Center, Bahrain	AlWasti, H.; Altaif, Z.; Shehab, F.; Zaed, A.
EPI_ISL_420543, EPI_ISL_420545, EPI_ISL_420547, EPI_ISL_420549, EPI_ISL_420551, EPI_ISL_420553, EPI_ISL_420555, EPI_ISL_421662 to 421672, EPI_ISL_424361 to 424365, EPI_ISL_426179			
see above	National Influenza Center, Indian Council of Medical Research - National Institute of Virology	Indian Council of Medical Research-National Institute of Virology, Microbial Containment Complex	Amita Jain; Anita M. Shete; Anita Shete-Aich; Atanu Basu; Bharati Malhotra; Bharti Malhotra; Deepika Chaudhary; Dimpal A. Nyayanit; Gajanan Sapkal; Gururaj Deshpande; Hitesh Dighe; Lalit Dar; M Choudhary; Manohar. L. Chaudhary; Padinjaremattathil Thankappan Ullas; Pragya D. Yadav; Pragya D. Yadav. Savita Patil; Pranita Gawande; Prasad Sarkale; Priya Abraham; Sarah Cherian; Savita Patil; Sreelekshmy Mohandas; Triparna Majumdar; Varsha Potdar
EPI_ISL_416427 to 416431	National Influenza Center, National Institute of Hygiene and Epidemiology (NIHE)	National Influenza Center, National Institute of Hygiene and Epidemiology (NIHE)	Dang Duc Anh; Futoshi Hasebe; Hoang Vu Mai Phuong; Kouichi Morita; Le Quynh Mai; Le Thi Thanh; Meng Ling Moi; Nguyen Le Khanh Hang; Nguyen Phuong Anh; Nguyen Vu Son; Pham Hong Quynh Anh; Pham Thi Hien; Taichiro Takemura; Takeshi Nabeshima; Tran Thu Huong; Ung Thi Hong Trang; Vuong Duc Cuong
EPI_ISL_467778 to 467781	National Influenza Centre Romania	Charite Universitatsmedizin Berlin, Institute of Virology	Barbara Muehlemann; Christian Drosten; Jorn Beheim-Schwarzbach; Julia Schneider; L. Ustea; M. Lazar; N. Paraschiv; Talitha Veith; Terry Jones; Victor M Corman
EPI_ISL_476835, EPI_ISL_476837 to 476839, EPI_ISL_476841, EPI_ISL_476843, EPI_ISL_476845, EPI_ISL_476847, EPI_ISL_476851			
see above	National Influenza Centre for Northern Greece	National Influenza Centre for Northern Greece	Maria Christoforidi
EPI_ISL_476836	National Influenza Centre for Nothern Greece	National Influenza Centre for Nothern Greece	Maria Christoforidi
EPI_ISL_490201	National Influenza Centre of INSPI-Ecuador	INSPI - Charité	Alberto Orlando; Alexandra Usiña; Alfredo Bruno Caicedo; Andres Moreira-Soto; Anna-Lena Sander; Denisses Portugal; Domenica de Mora Coloma; Jan Felix Drexler; Juan Carlos Zeballos; Manuel Gonzalez; Maritza Olmedo; Nina Krause; Silvia Salgado
EPI_ISL_410301	National Influenza Centre, National Public Health Laboratory, Kathmandu, Nepal	The University of Hong Kong	Alfonso J. Rodriguez-Morales; Anup Bastola; Basu Dev Pandey; Bibek Kumar Lal; Daniel Chu; Haogao Gu; Leo Poon; Malik Peiris; Ranjit Sah; Runa Jha
EPI_ISL_450171 to 450172	National Influenza centre, Sri Lanka	The University of Hong Kong	Daniel KW Chu; Jude Jayamaha; Malik Peiris
EPI_ISL_1469238, EPI_ISL_1469241 to 1469245, EPI_ISL_1469252, EPI_ISL_1469255, EPI_ISL_1469259 to 1469270, EPI_ISL_1469276 to 1469278, EPI_ISL_1469290 to 1469291, EPI_ISL_1534706 to 1534707			
see above	National Institute Health Research and Development	National Institute of Health Research and Development	Agustiningsih; Arie Ardiansyah Nugraha; Fauzul Muna; Hana Apsari Pawestri; Hartanti Dian Ikawati; Herna; Holy Arif Wibowo; Irene Lorinda Indalao; Kartika Dewi Puspa; Kindi Adam; Krisna Nur Andriana Pangesti; Krisna Pangesti; Natalie Laurencia Kipuw; Nelly Puspandari; Ni Ketut Susilarini; Nike Susanti; Nur Ika Hariastuti; Nurika Hariastuti; Reni Herman; Ririn Ramadhany; Subangkit; Tati Febriyanti; Triyani Soekarso; Ully Alfi Nikmah; Vivi Setiawaty; Vivi Setiawaty.; Yuni Rukminiati
EPI_ISL_596386	National Institute for Allergy and Infectious Diseases Integrated Research Facility - Frederick (NIAID IRF- Frederick), National Institutes of Health (NIH)	National Institute for Allergy and Infectious Diseases Integrated Research Facility - Frederick (NIAID IRF-Frederick), National Institutes of Health (NIH)	B. and Palacios, G.; Beitzel; J.R.; Kocher, G.; Kugelman
EPI_ISL_417186, EPI_ISL_430297, EPI_ISL_435058 to 435059, EPI_ISL_450296 to 450301, EPI_ISL_450495, EPI_ISL_470878 to 470880, EPI_ISL_490255 to 490314, EPI_ISL_504186 to 504244, EPI_ISL_514355 to 514424, EPI_ISL_515133 to 515180			
see above	National Institute for Communicable Diseases of the National Health Laboratory Service	National Institute for Communicable Diseases of the National Health Laboratory Service	A; Allam M; Bhiman JN; Ismail A; Khumalo Z; Kwenda S; Mnyameni F; Mohale T; Mtshali P; Subramoney K; van Heusden P; von Gottberg
EPI_ISL_1404878 to 1404913	National Institute for Food and Veterinary Risk Assessment	Lithuanian University of Health Sciences	Arnoldas Pautienius; Dovydas Gecys; Gediminas Alzbutas; Kamile Tamusauskaite; Lukas Zemaitis; Vaiva Lesauskaite
EPI_ISL_469254 to 469255	National Institute for Viral Disease Control and Prevention, China CDC	Institute of Viral Disease Control and Prevention, China CDC	Chun Huang; Dayan Wang; George F. Gao; George Fu Gao; Guizhen Wu; Jun Han; Li Zhao; Lijuan Chen; Peihua NiuBaoying Huang; Roujian Lu; Shiwen Wang; Weifeng Shi; Wenbo Xu; Wenjie Tan; Wenling Wang; William J. Liu; Xiang ZhaoLijuan Chen; Yanhai Wang; Yao MengZhixiao ChenYuchao Wu; Yong Zhang; Yubai Bi
EPI_ISL_408480 to 408486, EPI_ISL_408488	National Institute for Viral Disease Control and Prevention, China CDC	National Institute for Viral Disease Control & Prevention, CCDC	Baoying Huang; Baoying HuangLi ZhaoFei YeWenbo XuGeorge F. GaoGuizhen Wu; Chongshan Li; Fei Ye; George F. Gao; Guizhen Wu; Hengqin Wang; Huiping Yang; Jianan Xu; Jianxiong Li; Kangchen Zhao; Li Zhao; Peihua Niu; Quanyi Wang; Roujian Lu; Sheng Ye; Shenjiao Wang; Ti Liu; Wenbo Xu;

			Wenjie Tan; Wenjie TanXiaoqing FuXiang ZhaoWenling Wang Peihua NiuRoujian Lu; Wenjie TanZhen Teng; Wenling Wang; Xiang Zhao; Xiang ZhaoWenling Wang Peihua NiuRoujian Lu; Yang Pan; Yanhong SunBaoying HuangLi ZhaoFei YeWenbo XuGeorge F. GaoGuizhen Wu; Yong Shi; Zhaoguo Wang
EPI_ISL_408487	National Institute for Viral Disease Control and Prevention, China CDC	National Institute for Viral Disease Control & Prevention, China CDC	Baoying Huang; Fei Ye; George F. Gao; Guizhen Wu; Jin Xu; Li Zhao; Peihua Niu; Roujian Lu; Wenbo Xu; Wenjie Tan; Wenling Wang; Xiang Zhao; Xueyong Huang
EPI_ISL_469256, EPI_ISL_498691 to 498694, EPI_ISL_575329 to 575330, EPI_ISL_591270 to 591280	National Institute for Viral Disease Control and Prevention, China CDC	National Institute for Viral Disease Control and Prevention, China CDC	Cao Chen; Chun Huang; Dayan Wang; Dong Xia; Geogia Fu Gao; George F. Gao; George F.Gao; Guizhen Wu; Haijun Du; Hong Wang; Huilai Ma; Ji Wang; Jingdong Song; Juan Song; Jun Han; Kang Xiao; LingLing Mao; Qinqin Song; Rongbao Gao; Ruqin Gao; Shiwen Wang; Shuai Pang; Weifeng Shi; WenQing YaoWenbo Xu; Wenbo Xu; William J. Liu; Xiang Zhao; Xiang ZhaoLijuan Chen; Xuancheng Lu; Yang Song; Yanhai Wang; Yao Meng; Yao MengZhixiao ChenYuchao Wu; Yong Zhang; Yong ZhangBo ZhijianJianqun Zhang; Yuan He; Yuchao Wu; Zhaoguo Wang; ZhiQiang Xia; Zhixiao Chen
EPI_ISL_458133	National Institute of Biotechnology	Bioinformatics Division, National Institute of Biotechnology	Arittra Bhattacharjee; Asif Rashid; Chaman Ara Keya; Keshob Chandra Das; Md. Hadisur Rahman; Md. Moniruzzaman; Md. Nazrul Islam; Md. Ruhul Amin; Md. Salim Khan; Md. Salimullah; Mohammad Uzzal Hossain
EPI_ISL_447904	National Institute of Biotechnology	National Institute of Biotechnology	Arittra Bhattacharjee; Asif Rashid; Chaman Ara Keya; Irfan Ahmed; Keshob Chandra Das; Md. Hadisur Rahman; Md. Moniruzzaman; Md. Nazrul Islam; Md. Ruhul Amin; Md. Salimullah; Mohammad Uzzal Hossain; Tahia Anan Rahman
EPI_ISL_538504 to 538505, EPI_ISL_538508 to 538509, EPI_ISL_574433, EPI_ISL_576386 to 576387, EPI_ISL_1391009 to 1391018, EPI_ISL_1411159, EPI_ISL_1413427, EPI_ISL_1415427			
see above	National Institute of Health Research and Development	National Institute of Health Research and Development	A; AA; Adam; Agustiningsih; Arie Ardiansyah Nugraha; F; Fauzul Muna; Febriyani; Febriyanti; HA; HD; Hana Apsari Pawestri; Hariastuti; Hartanti Dian Ikawati; Herman; Herna; Holy Arif Wibowo; IL; Ikawati; Indalau; Irene Lorinda Indalao; J; K; KD; KNA; Kartika Dewi Puspa; Kindi Adam; Kipuw; Krisna Nur Andriana Pangesti; Kurniawati; M; Muna; Mursinah; N; NI; NK; NL; Natalie Laurencia Kipuw; Nelly Puspandari; Ni Ketut Susilarini; Nike Susanti; Nikmah; Nugraha; Nurhadi; Nurika Hariastuti; Paisal; Pangesti; Pawestri; Puspa; Puspandari; Puspandary; R; Ramadhany; Reni Herman; Ririn Ramadhany; Rizki; Setiawaty; Setiawaty, V.; Soekarso; Subangkit; Susanti; Susilarini; T; Tati Febriyanti; Triyani Soekarso; UA; Ully Alfi Nikmah; V; Vivi Setiawaty.; Wibowo; Yuni Rukminiati
EPI_ISL_450197, EPI_ISL_455584, EPI_ISL_455589 to 455593, EPI_ISL_455624	National Institute of Health. Department of medical Sciences, Ministry of Public Health, Thailand	National Institute of Health. Department of medical Sciences, Ministry of Public Health, Thailand	Chittaganpitch; Malinee; Okada; Parnmen; Phuygun; Pilailuk; Siripaporn; Sittiporn; Sunthareeya; Thanadachakul; Thanutsapa; Waicharoen; Warawan; Wongboot
EPI_ISL_479756 to 479758	National Institute of Hygiene and Epidemiology (NIHE)	National Key Laboratory of Gene Technology, Institute of Biotechnology (IBT)	Chu Hoang Ha; Dang Duc Anh; Futoshi Hasebe; Ho Thi Thuong; Hoang Ha; Hoang Vu Mai Phuong; Le Quynh Mai; Le Thi Thanh; Le Tung Lam; Nguyen Hong Trang; Nguyen Le Khanh Hang; Nguyen Phuong Anh; Nguyen Vu Son; Pham Thi Hien; Taichiro Takemura; Tran Huyen Linh; Tran Thu Huong; Truong Nam Hai; Ung Thi Hong Trang; Vuong Duc Cuong
EPI_ISL_1447363 to 1447487	National Institute of Infectious Diseases (NIID)	National Institute of Infectious Diseases (NIID)	Hideka Miura; Kentaro Itokawa; Kiyoko Okamoto; Kumiko Araki; Makoto Kuroda; Masanori Hashino; Rina Tanaka; Seiichiro Fujisaki; Shinichiro Hirai; Tsuyoshi Sekizuka
EPI_ISL_1418308 to 1418309, EPI_ISL_1418314 to 1418315, EPI_ISL_1418317 to 1418322, EPI_ISL_1418325 to 1418330	National Institute of Infectious Diseases-Prof. Dr. Matei Bals Molecular Diagnostics Laboratory	National Institute of Infectious Diseases-Prof. Dr. Matei Bals Molecular Diagnostics Laboratory	Corina Casangiu; Dan Otelea; Ionelia Nicolae; Leontina Banica; Marius Surleac; Ovidiu Vlaicu; Simona Paraschiv
EPI_ISL_480414 to 480421, EPI_ISL_480424 to 480427, EPI_ISL_480439 to 480444, EPI_ISL_1492682, EPI_ISL_1493059, EPI_ISL_1493066, EPI_ISL_1493069, EPI_ISL_1493103, EPI_ISL_1493106, EPI_ISL_1493108			
see above	National Institute of Laboratory Medicine and Referral Center	Bangladesh Council of Scientific and Industrial Research	A. K. M. Shamsuzzaman; Abu Sayeed Mohammad Mahmud; Asish Kumar Ghosh; Barna Goswami; Eshrar Osman; Iffat Jahan; Mahmuda Yeasmin; Md. Ahasan Habib; Md. Maruf Ahmed Molla; Md. Murshed Hasan Sarkar; Md. Saddam Hossain; Md. Salim Khan; Mohammad Samir Uzzaman; Salek Ahmed Sajib; Shahina Akter; Shahjahan Siddike; Sheikh Md. Selim Al Din; Tanjina Akhter Banu; Tanjina Akhter Banu; Tasnim Nafisa; Utpal Chandra Ray
EPI_ISL_1538405	National Institute of Laboratory Medicine and Referral Center	Dr. Qudrat-I-Khuda Road, Dhaka-1205, Bangladesh	A. K. M. Shamsuzzaman; Abu Sayeed Mohammad Mahmud; Arifa Akram; Asish Kumar Ghosh; Barna Goswami; Eshrar Osman; Iffat Jahan; Mahmuda Yeasmin; Md. Ahasan Habib; Md. Maruf Ahmed Molla; Md. Murshed Hasan Sarkar; Md. Saddam Hossain; Md. Salim Khan; Mohammad Mohi Uddin; Mohammad Samir Uzzaman; Shahina Akter; Tanjina Akhter Banu; Tasnim Nafisa
EPI_ISL_455420, EPI_ISL_455458 to 455459, EPI_ISL_462090 to 462098, EPI_ISL_464159 to 464166, EPI_ISL_465163 to 465164, EPI_ISL_466626 to 466630, EPI_ISL_466636 to 466639, EPI_ISL_466644 to 466645, EPI_ISL_466649 to 466650, EPI_ISL_466686 to 466694, EPI_ISL_469285 to 469286, EPI_ISL_469297 to 469300, EPI_ISL_475083 to 475084, EPI_ISL_475165 to 475173, EPI_ISL_475238, EPI_ISL_475754 to 475761, EPI_ISL_480445 to 480450, EPI_ISL_482485 to 482489, EPI_ISL_482700 to 482701, EPI_ISL_483622 to 483624, EPI_ISL_483626 to 483636, EPI_ISL_483641 to 483647, EPI_ISL_483686 to 483687, EPI_ISL_483689 to 483695, EPI_ISL_483699 to 483700, EPI_ISL_483703, EPI_ISL_483705, EPI_ISL_483707, EPI_ISL_483710, EPI_ISL_487362 to 487364, EPI_ISL_487366 to 487368, EPI_ISL_487371 to 487373, EPI_ISL_487375 to 487376, EPI_ISL_487378, EPI_ISL_487380, EPI_ISL_487382 to 487386, EPI_ISL_487392 to 487396, EPI_ISL_490109 to 490111, EPI_ISL_490113 to 490114, EPI_ISL_490144, EPI_ISL_490164 to 490165, EPI_ISL_490167 to 490168, EPI_ISL_498227 to 498228, EPI_ISL_498253 to 498254, EPI_ISL_498267 to 498268, EPI_ISL_498272 to 498274, EPI_ISL_498417 to 498419, EPI_ISL_498466 to 498467, EPI_ISL_498549 to 498550, EPI_ISL_498783 to 498797, EPI_ISL_498800 to 498806, EPI_ISL_498808 to 498809, EPI_ISL_498811, EPI_ISL_498814 to 498818, EPI_ISL_498830, EPI_ISL_498892, EPI_ISL_498932 to 498933, EPI_ISL_503958, EPI_ISL_504040, EPI_ISL_504137, EPI_ISL_504176 to 504184, EPI_ISL_514129 to 514130, EPI_ISL_514228 to 514252, EPI_ISL_603221 to 603225, EPI_ISL_603238 to 603247, EPI_ISL_603249 to 603250, EPI_ISL_1508828, EPI_ISL_1508833, EPI_ISL_1508841, EPI_ISL_1508940, EPI_ISL_1508942 to 1508943, EPI_ISL_1508946, EPI_ISL_1508950, EPI_ISL_1508952, EPI_ISL_1508954 to 1508955, EPI_ISL_1508999 to 1509001, EPI_ISL_1509272, EPI_ISL_1509298 to 1509299, EPI_ISL_1509637, EPI_ISL_1509924, EPI_ISL_1520103, EPI_ISL_1520105, EPI_ISL_1524771, EPI_ISL_1524775 to 1524776, EPI_ISL_1524802, EPI_ISL_1524872, EPI_ISL_1531533 to 1531534, EPI_ISL_1531553 to 1531572, EPI_ISL_1531735, EPI_ISL_1531743 to 1531744, EPI_ISL_1531776, EPI_ISL_1532065, EPI_ISL_1532855, EPI_ISL_1532982, EPI_ISL_1533423, EPI_ISL_1533426 to 1533427, EPI_ISL_1533429 to 1533430, EPI_ISL_1533728, EPI_ISL_1533802, EPI_ISL_1533852, EPI_ISL_1534610 to 1534621, EPI_ISL_1538406 to 1538411			
see above	National Institute of Laboratory Medicine and Referral Center	Genomic Research Lab, BCSIR	; A. K. M. Shamsuzzaman; Abu Sayeed Mohammad Mahmud; Arifa Akram; Asish Kumar Ghos; Asish Kumar Ghosh; Barna Goswami; Bayzid Bin Monir; Eshrar Osman; Iffat Jahan; Mahmuda Yeasmin; MahmudaYeasmin; Md. Ahasan Habib; Md. Ahashan Habib; Md. Maruf Ahmed Molla; Md. Masum Hossain Arif; Md. Murshed Hasan Sarkar; Md. Murshed Hasan Sarker; Md. Saddam Hossain; Md. Salim Khan; Mohammad Mohi Uddin; Mohammad Samir Uzzaman; Monira Parveen; Salek Ahmed Sajib; Shahina Akhter; Shahina Akter; Shahjahan Siddike; Sheikh Md. Selim Al Din; Tanjina Akhter Banu; Tanjina Akhter Banu; Tarannum Taznin; Tasnim Nafisa; Utpal Chandra Ray; Uzzaman
EPI_ISL_1508891 to 1508893, EPI_ISL_1508915	National Institute of Laboratory Medicine and Referral Center	Genomic Research Lab. BCSIR	A. K. M. Shamsuzzaman; Abu Sayeed Mohammad Mahmud; Arifa Akram; Asish Kumar Ghosh; Barna Goswami; Eshrar Osman; Iffat Jahan; Mahmuda Yeasmin; Md. Ahasan Habib; Md. Maruf Ahmed Molla; Md. Murshed Hasan Sarkar; Md. Saddam Hossain; Md. Salim Khan; Mohammad Samir Uzzaman; Shahina Akter; Tanjina Akhter Banu; Tasnim Nafisa
EPI_ISL_1492070 to 1492071, EPI_ISL_1492073 to 1492074, EPI_ISL_1492076 to 1492077, EPI_ISL_1492079, EPI_ISL_1492081 to 1492082, EPI_ISL_1492084 to 1492085, EPI_ISL_1492087, EPI_ISL_1492089 to 1492090, EPI_ISL_1492092 to 1492093, EPI_ISL_1492095, EPI_ISL_1492097, EPI_ISL_1492099 to 1492100, EPI_ISL_1492102 to 1492103, EPI_ISL_1492105, EPI_ISL_1492107 to 1492108, EPI_ISL_1492110 to 1492111, EPI_ISL_1492113 to 1492114, EPI_ISL_1492116 to 1492117, EPI_ISL_1492119, EPI_ISL_1492121, EPI_ISL_1492123 to 1492124, EPI_ISL_1492126 to 1492127, EPI_ISL_1492129 to 1492130, EPI_ISL_1492132 to 1492133, EPI_ISL_1492135, EPI_ISL_1492137 to 1492138, EPI_ISL_1492140 to 1492141, EPI_ISL_1492143 to 1492144, EPI_ISL_1492146 to 1492443, EPI_ISL_1498826 to 1498915			
see above	National Institute of Public Health	National Institute of Public Health	Alexander Nagy; Dusan Trnka; Helena Jirincova; Jaromira Vecerova; Timotej Suri
EPI_ISL_1510278 to 1510419	National Institute of Public Health	State Veterinary Institute Prague	A; D; H; J; Jirincova; Nagy; Suri; T; Trnka; Vecerova
EPI_ISL_539776, EPI_ISL_539778 to 539782	National Institute of Public Health (Czech Republic)	State Veterinary Institute Prague	A; D; H; Jirincova; L; Nagy; Novakova; Trnka; Vecerova, J.
EPI_ISL_1447198 to 1447199, EPI_ISL_1447215 to 1447227, EPI_ISL_1447230 to 1447234, EPI_ISL_1447236, EPI_ISL_1447254 to 1447256, EPI_ISL_1447258 to 1447259, EPI_ISL_1447269 to 1447270, EPI_ISL_1447282 to 1447283, EPI_ISL_1538664			
see above	National Institute of Public Health - National Institute of Hygiene	1. National Institute of Public Health - National Institute of Hygiene; 2. Eurofins Genomics Europe Sequencing GmbH	ECDC COVID-19 WGS support team; Eurofins Genomics Europe Sequencing Team; Gierczyki Rafa; Sadkowska-Todys Magorzata; Wokowicz Tomasz; Zacharczuk Katarzyna
EPI_ISL_541681 to 541751	National Institute of Virology, NIV Influenza	National Institute of Virology, NIV Influenza	Potdar V
EPI_ISL_434554 to 434558	National Institutes of Health, University of the Philippines Manila	Philippine Genome Center	Alessandra C. Sanchez; Benedict A. Maralit; Bernard Demot; Carlo M. Lapid; Christina Tan; El King D. Morado; Eva Maria Cutiongco-de la Paz; Francis A. Tablizo; Haifa L.Gaza; Jan Michael C. Yap; Jarvin E. Nipales; Jo-Hannah S. Llames; John Mark Velasco; Joshua Gregor A. Dizon; Joy Ann Petronio-Santos; Julius Aaron Mejia; Kris P. Punayan; Kristianne Arielle D. Gabriel; Maribell Dollete; Marissa M. Alejandria; Raul V. Destura; Shana F. Genavia; Shebna Rose D. Fabilloren; Shiela Mae M. Araiza; Sonia Salamat; and Cynthia P. Saloma
EPI_ISL_431833	National Institutes of Health, University of the Philippines Manila	Philippine Genome Center, University of the Philippines System	Alessandra C. Sanchez; Benedict A. Maralit; Bernard Demot; Carlo M. Lapid; Christina Tan; El King D. Morado; Eva Maria Cutiongco-de la Paz; Francis A. Tablizo; Haifa L.Gaza; Jan Michael C. Yap; Jarvin E. Nipales; Jo-Hannah S. Llames; John Mark Velasco; Joshua Gregor A. Dizon; Joy Ann Petronio-Santos; Julius Aaron Mejia; Kris P. Punayan; Kristianne Arielle D. Gabriel; Maribell Dollete; Marissa M. Alejandria; Raul V. Destura; Shana F. Genavia; Shebna Rose D. Fabilloren; Shiela Mae M. Araiza; Sonia Salamat; and Cynthia P. Saloma
EPI_ISL_449799	National Laboratory for Health, Environment and Food	National Laboratory for Health, Environment and Food	Blazun Vosner H.; Duh D.; Hedzet S.; Janezic S.; Mahnic A.; Rupnik M.; Zavrsnik J.
EPI_ISL_1490940, EPI_ISL_1491000, EPI_ISL_1491005 to 1491019, EPI_ISL_1491021 to 1491023, EPI_ISL_1491027, EPI_ISL_1491138 to 1491177, EPI_ISL_1491188 to 1491189, EPI_ISL_1491196 to 1491197, EPI_ISL_1491264, EPI_ISL_1491279 to 1491286, EPI_ISL_1491288, EPI_ISL_1491291 to 1491301, EPI_ISL_1491329, EPI_ISL_1491341			

see above	National Laboratory for Health, Environment and Food, OMM, Celje	CISLD (Clinical Institute of Special Laboratory Diagnostics), University Children's Hospital, University Medical Center Ljubljana	Ana Grom; Barbara Jenko Bizjan; Jernej Kova; Katarina Kozmos; Marko Pokorn; Maruša Debeljak; Robert Šket; Tadej Battelino; Tine Tesovnik
EPI_ISL_1490923, EPI_ISL_1490936 to 1490938, EPI_ISL_1490941, EPI_ISL_1490951, EPI_ISL_1490964 to 1490977, EPI_ISL_1491003 to 1491004, EPI_ISL_1491020, EPI_ISL_1491024, EPI_ISL_1491038 to 1491039, EPI_ISL_1491099 to 1491105, EPI_ISL_1491136, EPI_ISL_1491185 to 1491187, EPI_ISL_1491192 to 1491195, EPI_ISL_1491200, EPI_ISL_1491208 to 1491262, EPI_ISL_1491276 to 1491278, EPI_ISL_1491287, EPI_ISL_1491290, EPI_ISL_1491303 to 1491308, EPI_ISL_1491310 to 1491312			
see above	National Laboratory for Health, Environment and Food, OMM, Koper	CISLD (Clinical Institute of Special Laboratory Diagnostics), University Children's Hospital, University Medical Center Ljubljana	Ana Grom; Barbara Jenko Bizjan; Jernej Kova; Katarina Kozmos; Marko Pokorn; Maruša Debeljak; Robert Šket; Tadej Battelino; Tine Tesovnik
EPI_ISL_1490944 to 1490950, EPI_ISL_1490952, EPI_ISL_1491033 to 1491035, EPI_ISL_1491040 to 1491048, EPI_ISL_1491106, EPI_ISL_1491183 to 1491184, EPI_ISL_1491190, EPI_ISL_1491201 to 1491206, EPI_ISL_1491263, EPI_ISL_1491302, EPI_ISL_1491315 to 1491316, EPI_ISL_1491319, EPI_ISL_1491328			
see above	National Laboratory for Health, Environment and Food, OMM, Kranj	CISLD (Clinical Institute of Special Laboratory Diagnostics), University Children's Hospital, University Medical Center Ljubljana	Ana Grom; Barbara Jenko Bizjan; Jernej Kova; Katarina Kozmos; Marko Pokorn; Maruša Debeljak; Robert Šket; Tadej Battelino; Tine Tesovnik
EPI_ISL_1490920 to 1490922, EPI_ISL_1490924 to 1490935, EPI_ISL_1490939, EPI_ISL_1490943, EPI_ISL_1490978 to 1490999, EPI_ISL_1491025 to 1491026, EPI_ISL_1491028 to 1491032, EPI_ISL_1491037, EPI_ISL_1491081 to 1491098, EPI_ISL_1491107 to 1491133, EPI_ISL_1491137, EPI_ISL_1491178 to 1491182, EPI_ISL_1491191, EPI_ISL_1491198 to 1491199, EPI_ISL_1491265 to 1491274, EPI_ISL_1491309, EPI_ISL_1491313, EPI_ISL_1491317 to 1491318, EPI_ISL_1491320 to 1491327, EPI_ISL_1491330 to 1491334, EPI_ISL_1491336 to 1491339			
see above	National Laboratory for Health, Environment and Food, OMM, Maribor	CISLD (Clinical Institute of Special Laboratory Diagnostics), University Children's Hospital, University Medical Center Ljubljana	Ana Grom; Barbara Jenko Bizjan; Jernej Kova; Katarina Kozmos; Marko Pokorn; Maruša Debeljak; Robert Šket; Tadej Battelino; Tine Tesovnik
EPI_ISL_1490919, EPI_ISL_1490942, EPI_ISL_1490953 to 1490963, EPI_ISL_1491001 to 1491002, EPI_ISL_1491036, EPI_ISL_1491049 to 1491053, EPI_ISL_1491055 to 1491080, EPI_ISL_1491134 to 1491135, EPI_ISL_1491207, EPI_ISL_1491275, EPI_ISL_1491289, EPI_ISL_1491314, EPI_ISL_1491335, EPI_ISL_1491340			
see above	National Laboratory for Health, Environment and Food, OMM, Novo mesto	CISLD (Clinical Institute of Special Laboratory Diagnostics), University Children's Hospital, University Medical Center Ljubljana	Ana Grom; Barbara Jenko Bizjan; Jernej Kova; Katarina Kozmos; Marko Pokorn; Maruša Debeljak; Robert Šket; Tadej Battelino; Tine Tesovnik
EPI_ISL_512597 to 512637, EPI_ISL_512639 to 512644, EPI_ISL_512810	National Laboratory for Influenza/Virology reference laboratory, Public Health Center of the Ministry of Health of Ukraine	Respiratory Virus Unit, Microbiology Services Colindale, Public Health England	Dr. Iryna Demchyshyna; PHE Covid Sequencing Team
EPI_ISL_483637	National Laboratory of Virology, Szentágothai Research Centre	National Laboratory of Virology, Szentágothai Research Centre	Balázs Somogyi; Endre Gábor Tóth; Ferenc Jakab; Gábor Kemenesi
EPI_ISL_1392640 to 1392641	National Medical Research Center for Obstetrics, Gynecology and Perinatology named after Academician V.I.Kulakov of the Ministry of Healthcare of Russian Federation	National Medical Research Center for Obstetrics, Gynecology and Perinatology named after Academician V.I.Kulakov of the Ministry of Healthcare of Russian Federation	Andrey Donnikov; Andrey Goltsov; Denis Rebrikov; Dmitry Abramov; Dmitry Korostin; Dmitry Trofimov; Gennady Sukhikh; Jekaterina Shubina; Tatiana Priputnevich; Yegor Botsmanov
EPI_ISL_1386250 to 1386252, EPI_ISL_1386441, EPI_ISL_1386701, EPI_ISL_1386926, EPI_ISL_1387178, EPI_ISL_1387353, EPI_ISL_1387637, EPI_ISL_1387988, EPI_ISL_1388503, EPI_ISL_1388979, EPI_ISL_1389156 to 1389159, EPI_ISL_1524382, EPI_ISL_1524384, EPI_ISL_1524647, EPI_ISL_1524698 to 1524699, EPI_ISL_1524701 to 1524702, EPI_ISL_1524704 to 1524707, EPI_ISL_1524711 to 1524712, EPI_ISL_1524714 to 1524716, EPI_ISL_1524718 to 1524719, EPI_ISL_1524721 to 1524722, EPI_ISL_1524724 to 1524726, EPI_ISL_1524753 to 1524756, EPI_ISL_1524772 to 1524774			
see above	National Platform bis UMONS/Jolimont	National Platform bis UMONS/Jolimont	François Dufrasne; Gautier Detry; Guillaume Bayon-Vicente; Ruddy Wattiez
EPI_ISL_1447292 to 1447320	National Public Health Center, COVID Laboratory	National Public Health Center, National Biosafety Laboratory	Bernadett Pályi; Dániel Déri; Judit Henczkó; Norbert Solymosi; Nóra Magyar; Zoltán Kis
EPI_ISL_457833 to 457838, EPI_ISL_457842 to 457844	National Public Health Laboratory	KEMRI-Wellcome Trust Research Programme/KEMRI-CGMR-C Kilifi	Githinji G. et al 2020
EPI_ISL_416829, EPI_ISL_416866, EPI_ISL_416884 to 416886, EPI_ISL_416907	National Public Health Laboratory	Malaysia Genome Institute	Azrin Ahmad; Enizza Kasim; Hani Mat Hussin; Irni Suhayu Sapian; Mohd Faizal Abu Bakar; Mohd Noor Mat Isa; Noorliza Mohamad Noordin; Nor Azfa Johari; Norazimah Tajudin; Nurhezreen Md Iqbal; Rehan Shuhada Abu Bakar; Selvanesan Sengol; Shahrul Hisham Zainal Ariffin; Shamsidar Sopie; Siti Noraini Othman; W Afiza W Mohd Arifin; Yu Kie Chem; Yusuf Muhammad Noor
EPI_ISL_410719	National Public Health Laboratory	National Public Health Laboratory	Cui L; Lin RTP; Mak TM; Octavia S
EPI_ISL_410716	National Public Health Laboratory, National Centre for Infectious Diseases	National Centre for Infectious Diseases, National Centre for Infectious Diseases	Cui L; Lin RTP; Mak TM; Octavia S
EPI_ISL_410713 to 410715, EPI_ISL_418992 to 419001, EPI_ISL_422428 to 422435, EPI_ISL_428822 to 428850, EPI_ISL_435678 to 435700, EPI_ISL_443188 to 443249, EPI_ISL_462276 to 462433, EPI_ISL_469080 to 469115, EPI_ISL_469117 to 469155, EPI_ISL_469274, EPI_ISL_475937 to 475999, EPI_ISL_479574 to 479603, EPI_ISL_483576 to 483621, EPI_ISL_490049 to 490079, EPI_ISL_493390 to 493425, EPI_ISL_498564 to 498618, EPI_ISL_512822 to 512842, EPI_ISL_516807 to 516828, EPI_ISL_527359 to 527379, EPI_ISL_536412 to 536454, EPI_ISL_548972 to 549005, EPI_ISL_574493 to 574542, EPI_ISL_596456 to 596497, EPI_ISL_605817 to 605824, EPI_ISL_1442942 to 1442955, EPI_ISL_1476987 to 1477042, EPI_ISL_1489717 to 1489728, EPI_ISL_1519355 to 1519358, EPI_ISL_1519360 to 1519366, EPI_ISL_1519368 to 1519375, EPI_ISL_1519377 to 1519384, EPI_ISL_1519386, EPI_ISL_1519388, EPI_ISL_1519391, EPI_ISL_1519393, EPI_ISL_1519396, EPI_ISL_1519398, EPI_ISL_1519401, EPI_ISL_1519405, EPI_ISL_1519407, EPI_ISL_1519410, EPI_ISL_1519413, EPI_ISL_1519416, EPI_ISL_1519419, EPI_ISL_1524777 to 1524801, EPI_ISL_1543939 to 1543982, EPI_ISL_1545779			
see above	National Public Health Laboratory, National Centre for Infectious Diseases	National Public Health Laboratory, National Centre for Infectious Diseases	Chavatte JM; Chavatte Jean-Marc; Cui L; Cui Lin; Grace Jie Yin Ngan; Lin Cui; Lin RTP; Lin Raymond Tzer Pin; Mak TM; Mak Tze Minn; Octavia S; Octavia Sophie; Raymond Tzer Pin Lin; Royce Ang; Sophie Octavia; Tze Minn Mak; Zhenyang Zhou; Zhou Z
EPI_ISL_1497286 to 1497524, EPI_ISL_1503144 to 1503230	National Public Health Organization	National Public Health Organization	Kyriaki Tryfinopoulou et al
EPI_ISL_1498123 to 1498125	National Reference Laboratory	National Reference Laboratory	Enatha Mukantwari; Jeanne d'Arc Umuringa; Umuringa Jeanne d'Arc; Yvan Butera
EPI_ISL_480224, EPI_ISL_480297 to 480310	National Reference Laboratory "Influenza and acute respiratory diseases"	NRL-HIV	Ivailo Alexiev; Ivan Ivanov; Ivva Philipova
EPI_ISL_507008	National Veterinary Institute	National Veterinary Institute	Siamak Zohari
EPI_ISL_435674 to 435677, EPI_ISL_443187	National Virology Reference Laboratory	National Public Health Laboratory, National Centre for Infectious Diseases	Chavatte Jean-Marc; Cui Lin; Lin Raymond Tzer Pin; Mak Tze Minn; Octavia Sophie; Taib Surita; Zaini Zainun
EPI_ISL_605058 to 605080	National Virus Reference Laboratory	Irish Coronavirus Sequencing Consortium - Helixworks	Conor Crosbie; Nimesh Pinnamaneni; Sachin Chalapati
EPI_ISL_500573 to 500595, EPI_ISL_501252 to 501274, EPI_ISL_512087 to 512106, EPI_ISL_525352 to 525418, EPI_ISL_528440 to 528484, EPI_ISL_578202 to 578253, EPI_ISL_578256 to 578334, EPI_ISL_596889 to 596927, EPI_ISL_1396894 to 1397382, EPI_ISL_1398569 to 1398923, EPI_ISL_1398985 to 1399160, EPI_ISL_1402432 to 1402434, EPI_ISL_1402782 to 1402785, EPI_ISL_1416727 to 1416760, EPI_ISL_1483429 to 1483572, EPI_ISL_1491525 to 1491530, EPI_ISL_1499723 to 1499726, EPI_ISL_1499728 to 1500085, EPI_ISL_1501269 to 1501307, EPI_ISL_1502211 to 1502434, EPI_ISL_1510758 to 1510935, EPI_ISL_1511121 to 1511123, EPI_ISL_1511272 to 1511398, EPI_ISL_1552243 to 1552244, EPI_ISL_1552246 to 1552248, EPI_ISL_1552250 to 1552251, EPI_ISL_1552253, EPI_ISL_1552256, EPI_ISL_1552258, EPI_ISL_1552261, EPI_ISL_1552263, EPI_ISL_1552266, EPI_ISL_1552268, EPI_ISL_1552271, EPI_ISL_1552274, EPI_ISL_1552276, EPI_ISL_1552279, EPI_ISL_1552281, EPI_ISL_1552284, EPI_ISL_1552287, EPI_ISL_1552289, EPI_ISL_1552292, EPI_ISL_1552294, EPI_ISL_1552297, EPI_ISL_1552300, EPI_ISL_1552303, EPI_ISL_1552306, EPI_ISL_1552309, EPI_ISL_1552312, EPI_ISL_1552315 to 1552320, EPI_ISL_1552322 to 1552323, EPI_ISL_1552325 to 1552326, EPI_ISL_1552328 to 1552329, EPI_ISL_1552331 to 1552332, EPI_ISL_1552335, EPI_ISL_1552338, EPI_ISL_1552340, EPI_ISL_1552343, EPI_ISL_1552345, EPI_ISL_1552348, EPI_ISL_1552352, EPI_ISL_1552354, EPI_ISL_1552357, EPI_ISL_1552360, EPI_ISL_1552363, EPI_ISL_1552366, EPI_ISL_1552369, EPI_ISL_1552372, EPI_ISL_1552374, EPI_ISL_1552377, EPI_ISL_1552380 to 1552381, EPI_ISL_1552383 to 1552384, EPI_ISL_1552386 to 1552387, EPI_ISL_1552389, EPI_ISL_1552391, EPI_ISL_1552393, EPI_ISL_1552396, EPI_ISL_1552399, EPI_ISL_1552401, EPI_ISL_1552404, EPI_ISL_1552407, EPI_ISL_1552410, EPI_ISL_1552413, EPI_ISL_1552416, EPI_ISL_1552419, EPI_ISL_1552422, EPI_ISL_1552425, EPI_ISL_1552427, EPI_ISL_1552430, EPI_ISL_1552433, EPI_ISL_1552435, EPI_ISL_1552438, EPI_ISL_1552441, EPI_ISL_1552444, EPI_ISL_1552447 to 1552448, EPI_ISL_1552450 to 1552451, EPI_ISL_1552453 to 1552454, EPI_ISL_1552456 to 1552457, EPI_ISL_1552459, EPI_ISL_1552461 to 1552462, EPI_ISL_1552464 to 1552465, EPI_ISL_1552467 to 1552468, EPI_ISL_1552470 to 1552472, EPI_ISL_1552474 to 1552475, EPI_ISL_1552477 to 1552478, EPI_ISL_1552480 to 1552481, EPI_ISL_1552483, EPI_ISL_1552485 to 1552486, EPI_ISL_1552488 to 1552489, EPI_ISL_1552491 to 1552492, EPI_ISL_1552494 to 1552495, EPI_ISL_1552497 to 1552498, EPI_ISL_1552500 to 1552501, EPI_ISL_1552503 to 1552504, EPI_ISL_1552506 to 1552507, EPI_ISL_1552509 to 1552510, EPI_ISL_1552512 to 1552513, EPI_ISL_1552515 to 1552516, EPI_ISL_1552518 to 1552519, EPI_ISL_1552521 to 1552522, EPI_ISL_1552524 to 1552525, EPI_ISL_1552527 to 1552528, EPI_ISL_1552530 to 1552531, EPI_ISL_1552533 to 1552534, EPI_ISL_1552536 to 1552538, EPI_ISL_1552540 to 1552541, EPI_ISL_1552543, EPI_ISL_1552545 to 1552546, EPI_ISL_1552548 to 1552549, EPI_ISL_1552551 to 1552552, EPI_ISL_1552554 to 1552555, EPI_ISL_1552557 to 1552558, EPI_ISL_1552560 to 1552561, EPI_ISL_1552563 to 1552564, EPI_ISL_1552566 to 1552567, EPI_ISL_1552569 to 1552570, EPI_ISL_1552572 to 1552573, EPI_ISL_1552575 to 1552576, EPI_ISL_1552578 to 1552579, EPI_ISL_1552581 to 1552582, EPI_ISL_1552584 to 1552585, EPI_ISL_1552587 to 1552588, EPI_ISL_1552590 to 1552591, EPI_ISL_1552593, EPI_ISL_1552595 to 1552596, EPI_ISL_1552598 to 1552599, EPI_ISL_1552601, EPI_ISL_1552603 to 1552604, EPI_ISL_1552606 to 1552607, EPI_ISL_1552609 to 1552610, EPI_ISL_1552612 to 1552613, EPI_ISL_1552615 to 1552616, EPI_ISL_1552618 to 1552619, EPI_ISL_1552621 to 1552622, EPI_ISL_1552624 to 1552625, EPI_ISL_1552627 to 1552628, EPI_ISL_1552630 to 1552631, EPI_ISL_1552633 to 1552634, EPI_ISL_1552636 to 1552638, EPI_ISL_1552640 to 1552641, EPI_ISL_1552643 to 1552644, EPI_ISL_1552646 to 1552647, EPI_ISL_1552649 to 1552650, EPI_ISL_1552652 to 1552653, EPI_ISL_1552655 to 1552656, EPI_ISL_1552658 to 1552659, EPI_ISL_1552661 to 1552662, EPI_ISL_1552664, EPI_ISL_1552666 to 1552667, EPI_ISL_1552669 to 1552670, EPI_ISL_1552672 to 1552673, EPI_ISL_1552675 to 1552676, EPI_ISL_1552678 to 1552679, EPI_ISL_1552681 to 1552682, EPI_ISL_1552684			
see above	National Virus Reference Laboratory	National Virus Reference Laboratory	Aditi Chaturvedi; Calum Walsh; Charlene Bennet; Charlene Bennett; Cillian F De Gascun; Daniel Hare; Fiona Crispie; Gabriel Gonzalez; Guerrino Macori; Jonathan Dean; Matthew McCabe; Michael Carr; Paul Cotter; Seamus Fanning; Suzie Coughlan; Zoe Yandle
EPI_ISL_493149 to 493190	National Virus Resource Center, Chinese Academy of	Computational Virology Group, Center for Bacteria and	Di Liu; Hongping Wei; Jianjun Chen; Jin Xiong; Yi Huang; Yi Yan

	Sciences, Wuhan 430071, China	Viruses Resources and Bioinformation, Wuhan Institute of Virology, Chinese Academy of SciencesWuhan 430071, China	
EPI_ISL_444994 to 445000	Naval Health Research Center	Naval Medical Research Center Biological Defense Research Directorate	Adrian Paskey; Chris Myers; Dessiree Pena-Gomez; Ewell Hollis; Kimberly Bishop-Lilly; Kyle Long; Logan Voegtly; Melinda Balansay-Ames; Nathaniel Christy; Regina Cer; Roger Pan
EPI_ISL_491907 to 491931, EPI_ISL_491942 to 491943	Naval Infectious Diseases Diagnostic Laboratory	Naval Medical Research Center Biological Defense Research Directorate	Adrian Paskey; Dessiree Pena-Gomez; Francisco Malgon Bautista; Hua Wei Chen; Kimberly Bishop-Lilly; Kyle Long; Lindsay Glang; Logan Voegtly; Mark Simons; Megan Schilling; Regina Cer; Victor Sugiharto
EPI_ISL_514128	Navy and Marine Corps Public Health Center	Centers for Disease Control and Prevention	Anna Montmayeur; Anna Uehara; Clinton R. Paden; Haibin Wang; Jing Zhang; Krista Queen; Rachel Marine; Suxiang Tong; Yan Li; Ying Tao
EPI_ISL_514062 to 514092	Navy and Marine Corps Public Health Center	Pathogen Discovery, Respiratory Viruses Branch, Division of Viral Diseases, Centers for Disease Control and Prevention	Anna Montmayeur; Anna Uehara; Clinton R. Paden; Haibin Wang; Jing Zhang; Krista Queen; Rachel Marine; Suxiang Tong; Yan Li; Ying Tao
EPI_ISL_1400461 to 1400471, EPI_ISL_1444673 to 1444674, EPI_ISL_1444676 to 1444677, EPI_ISL_1444679 to 1444680, EPI_ISL_1444682 to 1444683, EPI_ISL_1444685 to 1444686, EPI_ISL_1444688, EPI_ISL_1503792 to 1503802			
see above	Nebraska Public Health Laboratory	NPHL COVID-19 Response Team	NPHL COVID-19 Response Team
EPI_ISL_428202 to 428206, EPI_ISL_466652 to 466683, EPI_ISL_467298, EPI_ISL_475174 to 475237	Nebraska Public Health Laboratory	UNMC COVID-19 Response Team	UNMC COVID-19 Response Team
EPI_ISL_514673 to 514674, EPI_ISL_515293 to 515462, EPI_ISL_1399162 to 1399179, EPI_ISL_1404589 to 1404613, EPI_ISL_1422992 to 1423007, EPI_ISL_1469493 to 1469548, EPI_ISL_1483242 to 1483278, EPI_ISL_1493146 to 1493164, EPI_ISL_1501675 to 1501683, EPI_ISL_1511631 to 1511639			
see above	Nevada State Public Health Laboratory	Nevada State Public Health Laboratory	Andrew Gorzalski; Chris Laverdure; Cyprian Rosetto; Cyprian Rossetto; David Jackson; Heather Kerwin; Joel R. Sevinsky; Mark Pandori; Natalie Crawford; Paul Hartley; Richard Tillet; Richard Tillett; Stephanie Van Hooser; Subhash C. Verma; and Mark Pandori
EPI_ISL_1534438	New Horizon Clinic wc NZC	NHLS/UCT	Arash Iranzadeh; Bruna Galvao; Carolyn Williamson; Deelan Doolabh; Diana Hardie; Emmanuel SJ; Innocent Mudau; Kruger Marais; Lynn Tyers; Marvin Hsiao; Stephen Korsman; Tegally H; de Oliveira T
EPI_ISL_510878 to 510888, EPI_ISL_535263 to 535303, EPI_ISL_542012 to 542097, EPI_ISL_569625 to 569663, EPI_ISL_574260 to 574291, EPI_ISL_602162 to 602207, EPI_ISL_1400942 to 1400963, EPI_ISL_1400967 to 1400981, EPI_ISL_1400984, EPI_ISL_1493389 to 1493435, EPI_ISL_1530948 to 1530989			
see above	New Mexico Department of Health Scientific Laboratory	New Mexico Department of Health Scientific Laboratory	Anastacia Griego-Fisher; D'Eldra Malone; D'eldra Malone; Ellie Johnson; Jennifer Benoit
EPI_ISL_467525 to 467664, EPI_ISL_485872, EPI_ISL_507984 to 508121, EPI_ISL_610250, EPI_ISL_610253, EPI_ISL_610268 to 610281	New Mexico Department of Health Scientific Laboratory Division	Center for Global Health, University of New Mexico Health Sciences Center	Darrell Dinwiddie; Daryl Domman; Joseph Hicks; Kurt Schwalm; Michael Edwards; Twila Kunde
EPI_ISL_596447 to 596448, EPI_ISL_602208 to 602209, EPI_ISL_602278 to 602281	New Mexico Department of Health Scientific Laboratory Division	Los Alamos National Laboratory Bioscience Division	Alina Deshpande; Cheryl Gleasner; Chien-Chi Lo; Joseph Hicks; Kim McMurry; Michael Edwards; Migun Shakya; Patrick Chain; Twila Kunde
EPI_ISL_1397953 to 1397957	New South Wales Health Pathology Royal Prince Alfred Hospital	Microbiology RPAH	Au, J.; Bull, R.; Deveson, I.; Foster, C.; Rawlinson, W.; Ruiz Silva, M.; Van Hal, S.
EPI_ISL_1394642, EPI_ISL_1394644 to 1394664, EPI_ISL_1397460 to 1397541	New York Presbyterian Hospital	Wadsworth Center, New York State Department of Health	Alexis Russel; Daryl M. Lamson; Erasmus Schneider; Erica Lasek-Nesselquist; John Kelly; Jonathan Plitnick; Kirsten St. George; Matthew Shudt; Melissa A Leisner; Navjot Singh
EPI_ISL_427526 to 427618	NewYork-Presbyterian & Mason Lab	Mason Lab	Alon Shaiber; Arryn Craney; Benjamin Young; Cem Meydan; Chandrima Bhattacharya; Christopher E. Mason; Christopher Mozsary; Craig D. Westover; Daniel J. Butler; David Danko; Dmitry Meleshko; Dong Xu; Ebrahim Afshinnekoo; Fritz J. Sedlazeck; Hanna Rennert; Iman Hajirasouliha; Jenny Xiang; Joel Rosiene; John Sipley; Jonathan Foox; Justyna Gawrys; Krista Ryon; Lars F. Westblade; Lin Cong; Marcin Imielinski; Maria Sierra; Massimo Loda; Matthew MacKay; Melissa Cushing; Mirella Salvatore; Nikolay A. Ivanov; Phyllis Ruggiero; Priya Velu; Shawn Levy
EPI_ISL_516934 to 516936, EPI_ISL_516938	Nicolae Testemitanu State University of Medicine and Pharmacy	International Centre for Genetic Engineering and Biotechnology (ICGEB) and ARGO Open Lab Platform for Genome Sequencing	Dal Monego S; Licastro D; Marcello A; Rajasekharan S; Ulinici M
EPI_ISL_1436808 to 1436816, EPI_ISL_1436818 to 1436828	Niedersächsisches Landesgesundheitsamt (NLGA)	Robert Koch Institute	
EPI_ISL_455422, EPI_ISL_455426	Nigeria Centre for Disease Control	African Centre of Excellence for Genomics of Infectious Diseases (ACEGID), Redeemer's University, Ede, Osun State, Nigeria	Ajogbasile F.V.; Folarin O.A.; Happi C.T.; Ihekweazu C.; Kayode A.; Oguzie J.; Olawoye I.; Olumade T.; Oluniyi P.E.; Uwanibe J.
EPI_ISL_455362, EPI_ISL_455412 to 455415, EPI_ISL_455418 to 455419, EPI_ISL_455423 to 455425, EPI_ISL_455427, EPI_ISL_455429 to 455431, EPI_ISL_487087, EPI_ISL_487089 to 487092, EPI_ISL_487095 to 487096, EPI_ISL_487098 to 487099, EPI_ISL_487101 to 487112, EPI_ISL_527872 to 527915			
see above	Nigeria Centre for Disease Control (NCDC)	African Centre of Excellence for Genomics of Infectious Diseases (ACEGID), Redeemer's University, Ede, Osun State, Nigeria	Ajogbasile F.V.; Folarin O.A.; Happi C.T.; Ihekweazu C.; Kayode A.; Oguzie J.; Olawoye I.; Olumade T.; Oluniyi P.E.; Oluniyi P.E. et al; Uwanibe J.
EPI_ISL_487113	Nigeria Centre for Disease Control (NCDC)	Redeemer's University, ACEGID	Ajogbasile F.V.; Folarin O.A.; Happi C.T.; Ihekweazu C.; Kayode A.; Oguzie J.; Olawoye I.; Olumade T.; Oluniyi P.E.; Uwanibe J.
EPI_ISL_479913 to 479924	Niigata City Public Health Research Institute	Pathogen Genomics Center, National Institute of Infectious Diseases	Hajime Kamiya; Kentaro Itokawa; Makoto Kuroda; Masanori Hashino; Motoi Suzuki; Rina Tanaka; Tsuyoshi Sekizuka; Yurie Takahashi
EPI_ISL_479869, EPI_ISL_479902, EPI_ISL_480226, EPI_ISL_1426573 to 1426574, EPI_ISL_1427585 to 1427586, EPI_ISL_1428574 to 1428587, EPI_ISL_1428594 to 1428599, EPI_ISL_1428604 to 1428609, EPI_ISL_1429633, EPI_ISL_1429716 to 1429721, EPI_ISL_1430685, EPI_ISL_1430705 to 1430714, EPI_ISL_1430849 to 1430854, EPI_ISL_1431521 to 1431523, EPI_ISL_1431724, EPI_ISL_1431730			
see above	Niigata Prefectural Institute of Public Health and Environmental Sciences	Pathogen Genomics Center, National Institute of Infectious Diseases	; Chang-Kweng Lim; Chikako Shimokawa; Daisuke Kobayashi; Eri Nakayama; Eunsil Park; Hajime Kamiya; Hussein H. Aly; Ken Miyazawa; Kentaro Itokawa; Kento Fukano; Makoto Kuroda; Masanori Hashino; Minoru Nagi; Motohiko Ogawa; Motoi Suzuki; Nozomu Hanaoka; Reiko Arai; Rina Tanaka; Shigeru Tanjima; Takahiro Maeki; Takanobu Kato; Takashi Sakudoh; Tsuguto Fujimoto; Tsuyoshi Sekizuka; Yasutaka Hoshino; Yoshihiro Kaku
EPI_ISL_420151	Nordland Hospital - Bodo, Laboratory Department, Molecular Biology Unit	Norwegian Institute of Public Health, Department of Virology	Hilde Elshaug; Kamilla Heddeland Instefjord; Karoline Bragstad; Kathrine Stene-Johansen; Olav Hungnes
EPI_ISL_1490276, EPI_ISL_1490304, EPI_ISL_1490312, EPI_ISL_1490316, EPI_ISL_1490326, EPI_ISL_1490356, EPI_ISL_1490367, EPI_ISL_1490369, EPI_ISL_1490372, EPI_ISL_1490391, EPI_ISL_1490409, EPI_ISL_1490412, EPI_ISL_1490433, EPI_ISL_1490436, EPI_ISL_1490444, EPI_ISL_1490449, EPI_ISL_1490452, EPI_ISL_1490458, EPI_ISL_1490462 to 1490463, EPI_ISL_1490581, EPI_ISL_1490584 to 1490585, EPI_ISL_1490589, EPI_ISL_1490618, EPI_ISL_1490635, EPI_ISL_1490647, EPI_ISL_1547603, EPI_ISL_1547605, EPI_ISL_1547615, EPI_ISL_1547619 to 1547620, EPI_ISL_1547622			
see above	Nordland Hospital - Bodo, Laboratory Department, Molecular Biology Unit	Norwegian Institute of Public Health, Department of Virology	Atiya R Ali; Debech Nadia; Engebretsen Serina Beate; Garcia Llorente Ignacio; Hilde Elshaug; Hilde Vollan; Jon Bråte; Kamilla Heddeland Instefjord; Karoline Bragstad; Kathrine Stene-Johansen; Marie Paulsen Madsen; Olav Hungnes; Pedersen Benedikte Nevjen; Rasmus Riis Kopperud
EPI_ISL_534239 to 534243	Norra Alvsborgs lanssjukhus	The Public Health Agency of Sweden	Anna Risberg; Anna-Malin Linde; Karin Tegmark-Wisell; Maria Lind Karlberg; Mattias Haukland; Mia Brytting; Olov Svartstrom; Oskar Karlsson Lindsjo; Petra Edquist; Reza Advani; Sandra Broddesson
EPI_ISL_1470059 to 1470069, EPI_ISL_1470122 to 1470136	North Estonia Medical Centre laboratory	1. Laboratory of Communicable Diseases (Estonia); 2. Eurofins Genomics Europe Sequencing GmbH	Liidia Dotsenko et al.
EPI_ISL_456294, EPI_ISL_456323, EPI_ISL_456395 to 456397, EPI_ISL_548018 to 548020, EPI_ISL_548028 to 548030, EPI_ISL_548042 to 548044, EPI_ISL_548047 to 548048, EPI_ISL_548057 to 548063, EPI_ISL_548065 to 548067, EPI_ISL_548075 to 548076, EPI_ISL_579093 to 579096, EPI_ISL_579406 to 579407			
see above	North Shore Hospital	Institute of Environmental Science and Research (ESR)	Anja Werno; Antje van der Linden; Arlo Upton; Chris Mansell; David Hammer; Dragana Drinkovic; Erasmus Smit; Gary McAuliffe; Hana Sofia Andersson; Hermes Perez; James Ussher; Jill Sherwood; Jing Wang; Joep de Ligt; Josh Freeman; Julia Howard; Juliet Elvy; Lauren Jelly; Mary DeAlmeida; Matt Blakiston; Matt Storey; Matthew Rogers; Max Bloomfield; Michael Addidle; Michelle Balm; Muhammad Faisal; Nikki Freed; Olin Silander; Sally Roberts; Sarah Jefferies; Sharmini Muttaiyah; Susan Morpeth; Susan Taylor; Timothy Blackmore; Vani Sathyendran; Veronica Playle; Virginia Hope; Xiaoyun Ren
EPI_ISL_482138	North West London Pathology, Imperial College Healthcare NHS Trust	Wellcome Sanger Institute for the COVID-19 Genomics UK (COG-UK) Consortium	Aileen Rowan; Alison Holmes; Anjna Badhan; Carolina Herrera and Alex Alderton; Cordelia Langford; David K. Jackson; David Muir; Dominic Kwiatkowski; Ewan Harrison; Frankie Bolt; Graham Taylor; Ian Johnston; James Price; John Sillitoe on behalf of the Wellcome Sanger Institute COVID-19 Surveillance

			Team; Ling Li; Paul Randell; Roberto Amato; Sonia Goncalves
EPI_ISL_482124, EPI_ISL_482131, EPI_ISL_516831 to 516883, EPI_ISL_524619 to 524712	North West London Pathology, Imperial College Healthcare NHS Trust	Wellcome Sanger Institute for the COVID-19 Genomics UK (COG-UK) consortium	Aileen Rowan; Alison Holmes; Anjna Badhan; Carolina Herrera and Alex Alderton; Cordelia Langford; David K. Jackson; David Muir; Dominic Kwiatkowski; Ewan Harrison; Frankie Bolt; Graham Taylor; Ian Johnston; James Price; John Sillitoe on behalf of the Wellcome Sanger Institute COVID-19 Surveillance Team (http://www.sanger.ac.uk/covid-team); Ling Li; Paul Randell; Roberto Amato; Sonia Goncalves
EPI_ISL_453486 to 453542, EPI_ISL_453544, EPI_ISL_457303 to 457325, EPI_ISL_461791 to 461804, EPI_ISL_472155 to 472160, EPI_ISL_472162 to 472238, EPI_ISL_472240 to 472290, EPI_ISL_478473 to 478498, EPI_ISL_478500 to 478511, EPI_ISL_478513, EPI_ISL_478516 to 478518, EPI_ISL_478520 to 478622, EPI_ISL_478624 to 478668, EPI_ISL_484264 to 484327, EPI_ISL_490440 to 490495, EPI_ISL_493452 to 493540, EPI_ISL_499472, EPI_ISL_499474, EPI_ISL_499478, EPI_ISL_499482, EPI_ISL_499485, EPI_ISL_499493 to 499494, EPI_ISL_499502 to 499508, EPI_ISL_499510, EPI_ISL_499515, EPI_ISL_499546 to 499556, EPI_ISL_499558 to 499561, EPI_ISL_499563, EPI_ISL_499568, EPI_ISL_499572, EPI_ISL_499587, EPI_ISL_499595 to 499598, EPI_ISL_499602, EPI_ISL_499606, EPI_ISL_499609 to 499610, EPI_ISL_499626, EPI_ISL_499777 to 499807, EPI_ISL_507110 to 507129, EPI_ISL_512348 to 512365, EPI_ISL_572409 to 572410, EPI_ISL_572412, EPI_ISL_572424, EPI_ISL_572438 to 572439, EPI_ISL_572450, EPI_ISL_572452, EPI_ISL_572464, EPI_ISL_572480, EPI_ISL_572488 to 572489, EPI_ISL_572491, EPI_ISL_572513, EPI_ISL_572515, EPI_ISL_572517, EPI_ISL_572525, EPI_ISL_572530, EPI_ISL_572532, EPI_ISL_572568, EPI_ISL_572570 to 572572, EPI_ISL_572579, EPI_ISL_572581, EPI_ISL_572594 to 572597, EPI_ISL_572644, EPI_ISL_572648, EPI_ISL_572663 to 572664, EPI_ISL_572700 to 572704, EPI_ISL_572712 to 572713, EPI_ISL_572729, EPI_ISL_572744, EPI_ISL_572747, EPI_ISL_572751, EPI_ISL_572753, EPI_ISL_572783, EPI_ISL_572795, EPI_ISL_572798, EPI_ISL_572802, EPI_ISL_572840, EPI_ISL_572845, EPI_ISL_572909 to 572911, EPI_ISL_572942, EPI_ISL_572950, EPI_ISL_573266 to 573413, EPI_ISL_573760 to 573761, EPI_ISL_573799, EPI_ISL_573871 to 573872, EPI_ISL_576986 to 577039, EPI_ISL_584681 to 584715, EPI_ISL_584847 to 584882, EPI_ISL_585504 to 585532, EPI_ISL_594987 to 595045, EPI_ISL_611524, EPI_ISL_611572, EPI_ISL_611574, EPI_ISL_611576 to 611577, EPI_ISL_611591, EPI_ISL_611643 to 611649, EPI_ISL_611657, EPI_ISL_611668, EPI_ISL_611746 to 611747, EPI_ISL_611752, EPI_ISL_611756 to 611760, EPI_ISL_611766 to 611770, EPI_ISL_611839, EPI_ISL_611846, EPI_ISL_611864, EPI_ISL_611868, EPI_ISL_611917, EPI_ISL_611930, EPI_ISL_611941 to 611953, EPI_ISL_611980, EPI_ISL_612008, EPI_ISL_612026 to 612027, EPI_ISL_612030, EPI_ISL_612054, EPI_ISL_612085, EPI_ISL_612090, EPI_ISL_612097, EPI_ISL_612100, EPI_ISL_612104, EPI_ISL_612110, EPI_ISL_612134, EPI_ISL_612523 to 612545, EPI_ISL_612549 to 612570, EPI_ISL_1387050 to 1387052, EPI_ISL_1387054, EPI_ISL_1387062 to 1387075, EPI_ISL_1387077 to 1387085, EPI_ISL_1387088, EPI_ISL_1387090 to 1387091, EPI_ISL_1387094 to 1387100, EPI_ISL_1387108, EPI_ISL_1387113 to 1387132, EPI_ISL_1475189, EPI_ISL_1475191 to 1475193, EPI_ISL_1475196, EPI_ISL_1475204, EPI_ISL_1475206 to 1475224, EPI_ISL_1475232 to 1475233, EPI_ISL_1475239, EPI_ISL_1475258 to 1475259, EPI_ISL_1475306 to 1475386, EPI_ISL_1475388 to 1475485			
see above	Northumbria University / South Tees Hospitals NHS Foundation Trust / North Cumbria Integrated Care NHS Foundation Trust / North Tees and Hartlepool NHS Foundation Trust / Newcastle Hospitals NHS Foundation Trust	COVID-19 Genomics UK (COG-UK) Consortium	Andrew Nelson; Brendan Payne; Clive Graham; Darren L Smith; Debra Padgett; Edward Barton; Emma Swindells; Garren Scott; Gary Black; Gary Eltringham; Giles S Holt; Greg R Young; Jane Greenaway; Jennifer Collins; John Allan; Joshua Loh; Lynn Dover; Matthew Bashton; Mohammad A Tariq; Paul Baker; Sarah Essex; Steve Liggett; Wen C Yew; Yusri Taha
EPI_ISL_453543	Northumbria University / South Tees Hospitals NHS Foundation Trust / North Cumbria Integrated Care NHS Foundation Trust / North Tees and Hartlepool NHS Foundation Trust / Newcastle Hospitals NHS Foundation Trust	Northumbria University	Andrew Nelson; Brendan Payne; Clive Graham; Darren L Smith; Debra Padgett; Edward Barton; Emma Swindells; Garren Scott; Gary Black; Gary Eltringham; Giles S Holt; Greg R Young; Jane Greenaway; Jennifer Collins; John Allan; Joshua Loh; Lynn Dover; Matthew Bashton; Mohammad A Tariq; Paul Baker; Sarah Essex; Steve Liggett; Wen C Yew; Yusri Taha
EPI_ISL_1520167	Northwest Community Hospital	Illinois Department of Public Health - Chicago Lab	Ira Heimler; Vineet K. Dhiman
EPI_ISL_1520143, EPI_ISL_1520165	Northwestern Medicine	Illinois Department of Public Health - Chicago Lab	Ira Heimler; Vineet K. Dhiman
EPI_ISL_1501684 to 1501693, EPI_ISL_1502052 to 1502074	Northwestern Memorial Hospital	Northwestern University - Ozer Lab	Chad J. Achenbach; Chao Qi; Egon A. Ozer; Judd F. Hultquist; Lacy M. Simons; Lawrence J. Jennings; Michael G. Ison; Ramon Lorenzo-Redondo; Taylor J. Dean
EPI_ISL_444520 to 444609	Northwestern Memorial Hospital	Ozer Lab	Alan R. Hauser; Chad J. Achenbach; Chao Qi; Egon A. Ozer; Hannah H. Nam; Judd F. Hultquist; Lacy M. Simons; Lawrence J. Jennings; Michael G. Ison; Ramon Lorenzo-Redondo; Scott C. Roberts
EPI_ISL_1490255, EPI_ISL_1490259, EPI_ISL_1490269 to 1490270, EPI_ISL_1490275, EPI_ISL_1490279 to 1490280, EPI_ISL_1490286, EPI_ISL_1490310, EPI_ISL_1490317 to 1490319, EPI_ISL_1490328, EPI_ISL_1490371, EPI_ISL_1490375, EPI_ISL_1490434, EPI_ISL_1490437, EPI_ISL_1490439, EPI_ISL_1490451, EPI_ISL_1490478 to 1490479, EPI_ISL_1490576 to 1490579, EPI_ISL_1490637, EPI_ISL_1547549 to 1547550, EPI_ISL_1547652, EPI_ISL_1547655 to 1547656			
see above	Norwegian Institute of Public Health, Department of Virology	Norwegian Institute of Public Health, Department of Virology	Atiya R Ali; Debech Nadia; Engebretsen Serina Beate; Garcia Llorente Ignacio; Hilde Elshaug; Hilde Vollan; Jon Bråte; Kamilla Heddeland Instefjord; Karoline Bragstad; Kathrine Stene-Johansen; Marie Paulsen Madsen; Olav Hungnes; Pedersen Benedikte Nevjen; Rasmus Riis Kopperud
EPI_ISL_534326	Notre Dame Intermedica Saude AS	Instituto Adolfo Lutz, Interdiciplinary Procedures Center, Strategic Laboratory	Claudia Regina Gonçalves; Claudio Tavares Sacchi; Erica Valessa Ramos Gomes
EPI_ISL_512846	O.I.J. MORGUE JUDICIAL	Inciensa, Instituto Costarricense de Investigación y Enseñanza en Nutrición y Salud	Adriana Godinez & Melany Calderon; Claudio Soto-Garita; Estela Cordero; Francisco Duarte; Hebleen Porras
EPI_ISL_1447091, EPI_ISL_1447100, EPI_ISL_1447104, EPI_ISL_1447111, EPI_ISL_1447114, EPI_ISL_1447119, EPI_ISL_1447138, EPI_ISL_1447177 to 1447178, EPI_ISL_1447183			
see above	OCME Office Of Chief Medical Examiner	New York City Public Health Laboratory	Jade Wang; et al.
EPI_ISL_1446621 to 1446629, EPI_ISL_1446858, EPI_ISL_1516373 to 1516376, EPI_ISL_1528497 to 1528502	OH Department of Health Laboratory	Centers for Disease Control and Prevention Division of Viral Diseases, Pathogen Discovery	Alison Laufer Halpin; Ben L. Rambo-Martin; Clinton R. Paden; Dakota Howard; Darlene Wagner; Dave Wentworth; Dhwani Batra; Jasmine Padilla; Justin Lee; Katie Dillon; Krista Queen; Kristen Knipe; Kristine Lacek; Mark Burroughs; Matthew Schmerer; Mili Sheth; Peter Cook; Sam Shepard; Sarah Nobles; Shoshona Le; Suxiang Tong; Vivien Dugan; Yvette Unoarumhi
EPI_ISL_424880, EPI_ISL_426430 to 426431	OH Department of Health Laboratory	Pathogen Discovery, Respiratory Viruses Branch, Division of Viral Diseases, Centers for Disease Control and Prevention	Alison S. Laufer Halpin; Anna Uehara; Christopher A. Elkins; Clinton R. Paden; Haibin Wang; Jing Zhang; Krista Queen; Mary S. Keckler; Rachel Marine; Suxiang Tong; Yan Li; Ying Tao
EPI_ISL_525803, EPI_ISL_525808 to 525824, EPI_ISL_525826, EPI_ISL_525828 to 525832, EPI_ISL_525834 to 525840, EPI_ISL_525842 to 525846, EPI_ISL_525848, EPI_ISL_525850, EPI_ISL_525852 to 525855, EPI_ISL_525859, EPI_ISL_525861, EPI_ISL_525863 to 525864, EPI_ISL_525866 to 525867, EPI_ISL_525869, EPI_ISL_525871 to 525873, EPI_ISL_525875, EPI_ISL_525877, EPI_ISL_525879, EPI_ISL_525882, EPI_ISL_525884, EPI_ISL_525886 to 525890, EPI_ISL_526087 to 526090, EPI_ISL_526092 to 526093, EPI_ISL_526097			
see above	OHSU Lab Services Molecular Microbiology Lab	Ginkgo Bioworks Clinical Laboratory	Alec J. Hirsch; Alex Plocik; Andrew C. Adey; Becky Schilling; Benjamin N. Bimber; Birgitte Simen; Brendan L. O'Connell; Brian J. O'Roak; Daniel N. Streblow; Guang Fan; James McGann; Jim Griffin; Keith Robison; Malaika Mckenzie-Bennett; Michelle Spencer; Rebecca Littlefield; Ruth V. Nichols; William B. Messer
EPI_ISL_437520 to 437535, EPI_ISL_468080 to 468129, EPI_ISL_509059 to 509214, EPI_ISL_525802, EPI_ISL_525804 to 525807, EPI_ISL_525825, EPI_ISL_525827, EPI_ISL_525833, EPI_ISL_525841, EPI_ISL_525847, EPI_ISL_525849, EPI_ISL_525851, EPI_ISL_525856 to 525858, EPI_ISL_525860, EPI_ISL_525862, EPI_ISL_525865, EPI_ISL_525868, EPI_ISL_525870, EPI_ISL_525874, EPI_ISL_525876, EPI_ISL_525878, EPI_ISL_525880 to 525881, EPI_ISL_525883, EPI_ISL_525885, EPI_ISL_525891 to 526086, EPI_ISL_526091, EPI_ISL_526094 to 526096, EPI_ISL_526098 to 526114, EPI_ISL_526551 to 526552, EPI_ISL_1541210 to 1541552, EPI_ISL_1541554 to 1542072			
see above	OHSU Lab Services Molecular Microbiology Lab	Oregon SARS-CoV-2 Genome Sequencing Center	Alec J. Hirsch; Andrew C. Adey; Benjamin N. Bimber; Brendan L. O'Connell; Brian J. O'Roak; Daniel N. Streblow; Donna Hansel; Guang Fan; Kayla Carter; Ruth V. Nichols; Sally B. Grindstaff; Sally Grindstaff; Sonia Acharya; William B. Messer; Xuan Qin
EPI_ISL_1516296 to 1516301	OK Public Health Laboratory, Oklahoma State DOH	Centers for Disease Control and Prevention Division of Viral Diseases, Pathogen Discovery	Alison Laufer Halpin; Ben L. Rambo-Martin; Clinton R. Paden; Dakota Howard; Darlene Wagner; Dave Wentworth; Dhwani Batra; Jasmine Padilla; Justin Lee; Katie Dillon; Krista Queen; Kristen Knipe; Kristine Lacek; Mark Burroughs; Matthew Schmerer; Mili Sheth; Peter Cook; Sam Shepard; Sarah Nobles; Shoshona Le; Suxiang Tong; Vivien Dugan; Yvette Unoarumhi
EPI_ISL_1424057 to 1424058	OLIMPUS MAGDALENA	Instituto Nacional de Salud- Dirección de Investigación en Salud Pública	Carlos Franco-Muñoz; Diego A. Álvarez-Díaz; Diego Andrés Prada; Gerardo Santamaría; Hector Alejandro Ruiz-Moreno; Jhonnatan Reales-González; Julian Naizaque; Katherine Laiton-Donato; Magdalena Wiesner; Marcela Mercado-Reyes.; Maria T. Herrera-Sepúlveda; Martha Lucia Ospina Martínez; Sheryll Corchuelo
EPI_ISL_1396406, EPI_ISL_1549134 to 1549138	OLVZ Aalst	OLVZ Aalst	Astrid Holderbeke
EPI_ISL_457705	OMAN-NIC	Department of Microbiology and Immunology- SQUH	Abdulla Balkhair; Ahlam Al-Amri; Aisha Al-Amri; Aisha Al-Busaidi; Amina Al Jardani; Fahad Zadjali; Fatma BaAlawi; Hamida AL Barwani; Hanan Al-kindi; Intisar Al-Shukri; Khulood Al-Mammary; Mohammed Al-Tobi; Samiha Al Kharusi; Samira Al-Maruqi; Zeyana AL-Dahmani
EPI_ISL_1446324, EPI_ISL_1446327, EPI_ISL_1446330, EPI_ISL_1446332, EPI_ISL_1446341, EPI_ISL_1446759 to 1446765, EPI_ISL_1447029, EPI_ISL_1516198 to 1516204, EPI_ISL_1528321 to 1528325			
see above	OR State PHL-Virology/Immunology Section	Centers for Disease Control and Prevention Division of Viral Diseases, Pathogen Discovery	Alison Laufer Halpin; Ben L. Rambo-Martin; Clinton R. Paden; Dakota Howard; Darlene Wagner; Dave Wentworth; Dhwani Batra; Jasmine Padilla; Justin Lee; Katie Dillon; Krista Queen; Kristen Knipe; Kristine Lacek; Mark Burroughs; Matthew Schmerer; Mili Sheth; Peter Cook; Sam Shepard; Sarah Nobles; Shoshona Le; Suxiang Tong; Vivien Dugan; Yvette Unoarumhi
EPI_ISL_419558	OR State PHL-Virology/Immunology Section	Pathogen Discovery, Respiratory Viruses Branch, Division of Viral Diseases, Centers for Disease Control and Prevention	Anna Uehara; Clinton R. Paden; Haibin Wang; Jasmine Padilla; Jing Zhang; Justin Lee; Krista Queen; Suxiang Tong; Yan Li; Ying Tao
EPI_ISL_534316	OS Mun Santana Lauro Ribas Braga	Instituto Adolfo Lutz, Interdiciplinary Procedures Center, Strategic Laboratory	Claudia Regina Gonçalves; Claudio Tavares Sacchi; Erica Valessa Ramos Gomes
EPI_ISL_1443199 to 1443201, EPI_ISL_1443203 to 1443206, EPI_ISL_1443208 to 1443213	OSF St Francis Medical Center	Illinois Department of Public Health - Springfield Lab	Bryan Sim; Gordon McCall
EPI_ISL_1553188 to 1553212, EPI_ISL_1553214	OSF St. Francis Medical Center	Illinois Department of Public Health - Springfield Lab	Bryan Sim; Gordon McCall

to 1553216, EPI_ISL_1553251			
EPI_ISL_1500153 to 1500169, EPI_ISL_1500171	OSF St. Francis Medical Center - OSF System Laboratory	Illinois Department of Public Health - Springfield Lab	Bryan Sim; Gordon McCall
EPI_ISL_1502472, EPI_ISL_1502522, EPI_ISL_1502524 to 1502525	OSPEDALE "F. RENZETTI" LANCIANO - MEDICINA INTERNA LANCIANO(CHIETI)	Istituto Zooprofilattico Sperimentale dell'Abruzzo e Molise "G. Caporale"	Ancora M; Calistri P; Cammà C; Caporale M; Curini V; Delli Compagni E; Di Domenico M; Di Lollo Valeria; Di Pasquale A; Lorusso A; Mangone I; Marcacci M; Puglia I; Rinaldi A; Savini G; Scialabba S
EPI_ISL_1403552	OSPEDALE CIVILE GIULIANOVA - MEDICINA INTERNA GIULIANOVA(TERAMO)	Istituto Zooprofilattico Sperimentale dell'Abruzzo e Molise "G. Caporale"	Ancora M; Calistri P; Cammà C; Curini V; Delli Compagni E; Di Domenico M; Di Pasquale A; Lorusso A; Mangone I; Marcacci M; Puglia I; Rinaldi A; Savini G; Scialabba S
EPI_ISL_1392650 to 1392651, EPI_ISL_1403508, EPI_ISL_1502449, EPI_ISL_1502517	OSPEDALE CIVILE GIULIANOVA - PRONTO SOCCORSO GIULIANOVA(TERAMO)	Istituto Zooprofilattico Sperimentale dell'Abruzzo e Molise "G. Caporale"	Ancora M; Calistri P; Cammà C; Caporale M; Curini V; Delli Compagni E; Di Domenico M; Di Lollo Valeria; Di Pasquale A; Lorusso A; Mangone I; Marcacci M; Puglia I; Rinaldi A; Savini G; Scialabba S
EPI_ISL_1403548, EPI_ISL_1403555, EPI_ISL_1415421	OSPEDALE CIVILE SANT'OMERO - PRONTO SOCCORSO SANT'OMERO(TERAMO)	Istituto Zooprofilattico Sperimentale dell'Abruzzo e Molise "G. Caporale"	Ancora M; Calistri P; Cammà C; Caporale M; Curini V; Delli Compagni E; Di Domenico M; Di Lollo Valeria; Di Pasquale A; Lorusso A; Mangone I; Marcacci M; Puglia I; Rinaldi A; Savini G; Scialabba S
EPI_ISL_1391395	OSPEDALE S.S. ANNUNZIATA CHIETI	Istituto Zooprofilattico Sperimentale dell'Abruzzo e Molise "G. Caporale"	Ancora M; Calistri P; Cammà C; Caporale M; Curini V; Di Domenico M; Di Lollo Valeria; Di Pasquale A; Lorusso A; Mangone I; Marcacci M; Puglia I; Rinaldi A; Savini G; Scialabba S
EPI_ISL_1502540 to 1502566	OSPEDALE SAN SALVATORE - MEDICINA DI LABORATORIO L'AQUILA(L'AQUILA)	Istituto Zooprofilattico Sperimentale dell'Abruzzo e Molise "G. Caporale"	Ancora M; Calistri P; Cammà C; Caporale M; Curini V; Delli Compagni E; Di Domenico M; Di Lollo Valeria; Di Pasquale A; Lorusso A; Mangone I; Marcacci M; Puglia I; Rinaldi A; Savini G; Scialabba S
EPI_ISL_430010	OSU Wexner Medical Center	James Molecular Lab - OSUWMC	Dan Jones; Erica Vincent; Huolin Tu; Matt Avenarius; Matt Hunt; Preeti Pancholi
EPI_ISL_498171 to 498192	OUCRU	OUCRU	Dinh Nguyen Huy Man; Guy Thwaites; Lam Anh Nguyet; Lam Minh Yen; Le Manh Hung; Le Nguyen Truc Nhu; Nghiem My Ngoc; Ngo Ngoc Quang Minh; Nguyen Tan Binh; Nguyen Thanh Dung; Nguyen Thanh Phong; Nguyen Thanh Truong; Nguyen Thi Han Ny; Nguyen Thi Thu Hong; Nguyen Tri Dung; Nguyen Van Vinh Chau; Tang Chi Thuong; Tran Chanh Xuan; Tran Nguyen Hoang Tu; Tran Tan Thanh; Tran Tinh Hien; Vo Thanh Lam; and Le Van Tan; for OUCRU COVID-19 research group*
EPI_ISL_450738 to 450745	OUCRU/HTD	OUCRU/HTD	Dinh Nguyen Huy Man; Guy Thwaites; Lam Anh Nguyet; Lam Minh Yen; Le Manh Hung; Le Nguyen Truc Nhu; Nghiem My Ngoc; Ngo Ngoc Quang Minh; Nguyen Tan Binh; Nguyen Thanh Dung; Nguyen Thanh Phong; Nguyen Thanh Truong; Nguyen Thi Han Ny; Nguyen Thi Thu Hong; Nguyen Tri Dung; Nguyen Van Vinh Chau; Tang Chi Thuong; Tran Chanh Xuan; Tran Nguyen Hoang Tu; Tran Tan Thanh; Tran Tinh Hien; Vo Thanh Lam; and Le Van Tan; for OUCRU COVID-19 research group*
EPI_ISL_1441264, EPI_ISL_1441266, EPI_ISL_1441277	Oberlausitz Kliniken gGmBH	Robert Koch Institute	
EPI_ISL_1534271, EPI_ISL_1534279	Ocean View CDC wc OCE	NHLS/UCT	Arash Iranzadeh; Bruna Galvao; Carolyn Williamson; Deelan Doolabh; Diana Hardie; Innocent Mudau; Kruger Marais; Lynn Tyers; Marvin Hsiao; Stephen Korsman
EPI_ISL_426627 to 426628	Ochsner Health	BioInfoExperts, LLC	Amy Feehan; David Moraga Amador; David Nolan; Julia-Garcia-Diaz; Luke Caruso; Lydia Von Borstel; Rebecca Rose; Sissy Cross; Susanna Lamers; Tong Yang; Wayra Navia; Xiao Hui Zhou
EPI_ISL_436939 to 436961, EPI_ISL_455609 to 455623, EPI_ISL_463277 to 463300, EPI_ISL_468701 to 468717	Ochsner Health	Bioinfoexperts, LLC	Amy Feehan; David J. Nolan; David Moraga Amador; Julia-Garcia-Diaz; Luke Caruso; Lydia Von Borstel; Rebecca Rose; Sissy Cross; Susanna L. Lamers; Tong Yang; Wayra Navia; Xiao Hui Zhou
EPI_ISL_1447195, EPI_ISL_1447253, EPI_ISL_1447257	Odzia Mikrobiologii WSSE Wrocaw	1. National Institute of Public Health - National Institute of Hygiene; 2. Eurofins Genomics Europe Sequencing GmbH	ECDC COVID-19 WGS support team; Eurofins Genomics Europe Sequencing Team; Gierczyki Rafa; Sadkowska-Todys Magorzata; Wokowicz Tomasz; Zacharczuk Katarzyna
EPI_ISL_1491575	Office of Diseases Prevention and Control Region 4 Saraburi	COVID-19 Network Investigations (CONI) Alliance	Anek Mungaomklang; Angkana Huang; Anthony R. Jones; Arporn Wangwiwatsin; Bhakbhoom Panthan; Chonticha Klungtong; Duangkamon Loesbanluechai; Ekawat Pasomsub; Elizabeth Batty; Insee Sensorn; Janjira Thaipadungpanit; Jutikul Kaewmalakul; Khajohn Joonlasak; Kingkan Rakmanee; Krittikorn Kumpornsin; Namfon Kotanan; Nathamon Runnachot; Pakjira Rimdusit; Payon Pengyo; Praima Moonmuang; Sataporn Hatsadichart; Sirinapa Singthong; Siriporn Lakesukthom; Siriwan Yaemnimnual; Stefan Fernandez; Suttiruk Changchawai; Thanat Chookajorn; Theerarat Kochakarn; Treewat Watthanachockchai; Wasun Chantratita; Wonvimol Lemprasert; Wudtichai Manasatienkij
EPI_ISL_1553183	Ogle County Correctional Center	Illinois Department of Public Health - Springfield Lab	Bryan Sim; Gordon McCall
EPI_ISL_1386661, EPI_ISL_1389160 to 1389162, EPI_ISL_1389275, EPI_ISL_1389397, EPI_ISL_1389399, EPI_ISL_1390768, EPI_ISL_1463151, EPI_ISL_1463445 to 1463447, EPI_ISL_1463541 to 1463543, EPI_ISL_1468405 to 1468410, EPI_ISL_1533424 to 1533425, EPI_ISL_1533428, EPI_ISL_1545442 to 1545444			
see above	Ohio Department of Health Laboratory	Ohio Department of Health Laboratory	Allison Black; Brent Lee; Caitlin McDonnell; Eric Brandt; Erica Leasure; Glen McGillivary; Heather Blankenship; Holmes; Jade Mowery; Jennifer; Kelsey Florek; Keoni Omura; Kirtana Ramadugu; Quanta Brown; Stephanie Mccracken; Tyler Payne; and Tammy Bannerman
EPI_ISL_1391363	Ohio State University Wexner Medical Center	Polaris Molecular Laboratory	Chappell D; Jones D; Leber A; Snyder P; Tu H; Wang H
EPI_ISL_479979 to 479984, EPI_ISL_480109 to 480119	Oita Prefectural Institute of Public Health and Environmental Science	Pathogen Genomics Center, National Institute of Infectious Diseases	Hajime Kamiya; Kentaro Itokawa; Makoto Kuroda; Mari Sasaki; Masanori Hashino; Motoi Suzuki; Rina Tanaka; Tsuyoshi Sekizuka
EPI_ISL_535361 to 535364	Oklahoma Animal Disease Diagnostic Laboratory	Oklahoma Animal Disease Diagnostic Laboratory	Akhilesh Ramachandran; Anil Kaul; Girish Patil; Jeremiah Saliki; Jerry Malayer; John C Ritchey; Sai Narayanan; Sunil More; Teluguakula Narasaraju
EPI_ISL_576127	Oklahoma Animal Disease Diagnostic Laboratory, Oklahoma State University	Oklahoma Animal Disease Diagnostic Laboratory, Oklahoma State University	J.C.; J.R.; J.T.; Kaul, A.; Malayer; More, S.; Narasaraju, T.; Narayanan, S.; Patil, G.; Ramachandran, A.; Ritchey; Saliki
EPI_ISL_487231	Oklahoma State University Diagnostic Laboratory-Oklahoma Animal Disease Diagnostic Laboratory	Oklahoma Animal Disease Diagnostic Laboratory	Akhilesh Ramachandran; Anil Kaul; Girish Patil; Jerry Saliki; John Ritchey; Sai Narayanan; Sunil More; Teluguakula Narasaraju
EPI_ISL_455111	Olof Norrby	The Public Health Agency of Sweden	Anna Risberg; Anna-Malin Linde; Bla Kustens halsocentral; Karin Tegmark-Wisell; Maria Lind Karlberg; Mia Brytting; Olov Svartstrom; Oskar Karlsson Lindsjo; Theresa Enkirch
EPI_ISL_458137	Oman National Influenza Centre	Department of Microbiology and Immunology	Abdulla Balkhair; Ahlam Al-Amri; Aisha Al-Amri; Aisha Al-Busaidi; Amina Al Jardani; Fahad Zadjali; Fatma BaAlawi; Hamida AL Barwani; Hanan Al-kindi; Intisar Al-Shukri; Khulood Al-Mammary; Mohammed Al-Tobi; Samiha Al Kharusi; Samira Al-Maruqi; Zeyana AL-Dahmani
EPI_ISL_458116 to 458128	Oman National Influenza Centre	Department of Microbiology and Immunology-SQUH	Abdulla Balkhair; Ahlam Al-Amri; Aisha Al-Amri; Aisha Al-Busaidi; Amina Al Jardani; Fahad Zadjali; Fatma BaAlawi; Hamida AL Barwani; Hanan Al-kindi; Intisar Al-Shukri; Khulood Al-Mammary; Mohammed Al-Tobi; Samiha Al Kharusi; Samira Al-Maruqi; Zeyana AL-Dahmani
EPI_ISL_457703, EPI_ISL_457707	Oman-NIC	Department of Microbiology and Immunology- SQUH	Abdulla Balkhair; Ahlam Al-Amri; Aisha Al-Amri; Aisha Al-Busaidi; Amina Al Jardani; Fahad Zadjali; Fatma BaAlawi; Hamida AL Barwani; Hanan Al-kindi; Intisar Al-Shukri; Khulood Al-Mammary; Mohammed Al-Tobi; Samiha Al Kharusi; Samira Al-Maruqi; Zeyana AL-Dahmani
EPI_ISL_457981 to 457984, EPI_ISL_491968 to 492026	Oman-NIC	Department of Microbiology and Immunology-SQUH	Abdulla Balkhair; Ahlam Al-Amri; Aisha Al-Amri; Aisha Al-Busaidi; Amina Al Jardani; Fahad Zadjali; Fatma BaAlawi; Hamida AL Barwani; Hanan Al-kindi; Intisar Al-Shukri; Khulood Al-Mammary; Mohammed Al-Tobi; Samiha Al Kharusi; Samira Al-Maruqi; Zeyana AL-Dahmani
EPI_ISL_457702	Oman-NIC	Microbiology laboratory- Sultan Qaboos University Hospital	Abdulla Balkhair; Ahlam Al-Amri; Aisha Al-Amri; Aisha Al-Busaidi; Amina Al Jardani; Fahad Zadjali; Fatma BaAlawi; Hamida AL Barwani; Hanan Al-kindi; Intisar Al-Shukri; Khulood Al-Mammary; Mohammed Al-Tobi; Samiha Al Kharusi; Samira Al-Maruqi; Zeyana AL-Dahmani
EPI_ISL_457701, EPI_ISL_457704, EPI_ISL_457706, EPI_ISL_457937 to 457939, EPI_ISL_457974 to 457980, EPI_ISL_457985 to 457998	Oman-NIC	Oman-NIC	Abdulla Balkhair; Ahlam Al-Amri; Aisha Al-Amri; Aisha Al-Busaidi; Amina Al Jardani; Fahad Zadjali; Fatma BaAlawi; Hamida AL Barwani; Hanan Al-kindi; Intisar Al-Shukri; Khulood Al-Mammary; Mohammed Al-Tobi; Samiha Al Kharusi; Samira Al-Maruqi; Zeyana AL-Dahmani
EPI_ISL_491116, EPI_ISL_491121 to 491171, EPI_ISL_525423 to 525429, EPI_ISL_525466, EPI_ISL_1517192, EPI_ISL_1532282 to 1532305	Oman-National Influenza Center	Biotechnology & OMICs Laboratory	Abdul Latif Khan; Adil Al-Wahaibi; Adil Khan; Ahlam Al-Amri; Ahmed Al Harrasi; Ahmed Al Harrasi.; Ahmed Al-Harrasi; Ahmed Al-Rawahi; Ahmed N Al-Rawahi; Aisha Al-Amri; Aisha Al-Busaidi; Amal Al-Maani; Amina Al-Jardani; Amina Al-Jardani.; Bilal Hussain; Fahad Al-Balushi; Hanan Al-Kindi; Intisar Al-Shukri; Laila Al-Balushi; Sajjad Asaf; Samiha Al-Kharusi; Samira Al-Mahruqi; Samiya Al-Zadjali; Saqib Bilal; Seif Al-Abri; Seif Al-Abri.

EPI_ISL_518821 to 518854	Oman-National Influenza Center	Biotechnology & OMICs Laboratory, Natural & Medical Sciences Research Center, University of Nizwa	Abdul Latif Khan; Adil Al-Wahaibi; Adil Khan; Ahlam Al-Amri; Ahmed Al-Harrasi; Ahmed Al-Rawahi; Aisha Al-Amri; Aisha Al-Busaidi; Amina Al-Jardani; Hanan Al-Kindi; Intisar Al-Shukri; Sajjad Asaf; Samiha Al-Kharusi; Samira Al-Mahruqi; Seif Al-Abri; Seif Al-Abri.
EPI_ISL_492065	Oman-National Influenza Center	Department of Microbiology and Immunology-SQUH Department of Microbiology and Immunology, Sultan Qaboos University Hopsital, P.O 35, Postal code 123	Abdulla Balkhair; Amina Al Jardani; Azza Al-Rashdi; Fahad Zadjali; Fatma BaAlawi; Hamida AL Barwani; Hanan Al-kindi; Intisar Al-Shukri; Khulood Al-Mammary; Samiha Al Kharusi; Samira Al-Maruqi; Zeyana AL-Dahmani
EPI_ISL_1443666 to 1443682	Omics Sciences Laboratory	Omics Sciences Laboratory	Darlyn Amaya; Derly Andrade Molina; Emily Sulay Saltos Montalvo; Gabriel Morey León; Juan Carlos Fernández Cadena; Katheryn Sacheri Viteri; Paula Juliana Gavilanes Jarrín; Rubén Armas González
EPI_ISL_569735 to 569857	Omsk Research Institute of Natural Focal Infections	WHO National Influenza Centre Russian Federation	Aleksei Vasilenko; Andrey Komissarov; Artem Fadeev; Daria Nashatyreva; Ekaterina Gradoboeva; Ekaterina Savkina; Elena Poleshchuk; Valery Yakimenko
EPI_ISL_434661 to 434662, EPI_ISL_434664, EPI_ISL_434673, EPI_ISL_475528, EPI_ISL_475533 to 475535, EPI_ISL_475538, EPI_ISL_539883 to 539884			
see above	Omtanken Grimmered	The Public Health Agency of Sweden	Anna Risberg; Anna-Malin Linde; Bernd Sengpiel; Karin Tegmark-Wisell; Maria Lind Karlberg; Mattias Haukland; Mia Brytting; Olov Svartstrom; Oskar Karlsson Lindsjo; Reza Advani; Sandra Broddesson; Theresa Enkirch
EPI_ISL_468592 to 468614, EPI_ISL_513707 to 513772, EPI_ISL_582975 to 583045, EPI_ISL_1477443, EPI_ISL_1477495, EPI_ISL_1477498, EPI_ISL_1477572, EPI_ISL_1477582, EPI_ISL_1477611, EPI_ISL_1477663, EPI_ISL_1477986, EPI_ISL_1478024, EPI_ISL_1478030, EPI_ISL_1478106, EPI_ISL_1478110, EPI_ISL_1478175, EPI_ISL_1529158, EPI_ISL_1529336			
see above	Orange County Public Health Lab	Chan-Zuckerberg Biohub	CZB Cliahub Consortium
EPI_ISL_486120 to 486279, EPI_ISL_513789 to 513839, EPI_ISL_548270 to 548276, EPI_ISL_548278, EPI_ISL_548283, EPI_ISL_548285, EPI_ISL_548287, EPI_ISL_548289, EPI_ISL_548291, EPI_ISL_548293, EPI_ISL_548296 to 548298, EPI_ISL_548300 to 548301, EPI_ISL_548305 to 548306, EPI_ISL_548308 to 548309, EPI_ISL_548312 to 548313, EPI_ISL_548315, EPI_ISL_548317 to 548318, EPI_ISL_548323, EPI_ISL_548326, EPI_ISL_548328, EPI_ISL_548332, EPI_ISL_548334, EPI_ISL_548373, EPI_ISL_548375, EPI_ISL_548377 to 548378, EPI_ISL_548381, EPI_ISL_548383, EPI_ISL_548386 to 548387, EPI_ISL_548389, EPI_ISL_548393, EPI_ISL_548400, EPI_ISL_548404 to 548405, EPI_ISL_548408, EPI_ISL_548411 to 548413, EPI_ISL_548416, EPI_ISL_548418, EPI_ISL_548424, EPI_ISL_548431, EPI_ISL_548438 to 548441, EPI_ISL_548444 to 548445, EPI_ISL_548449, EPI_ISL_548454, EPI_ISL_548456, EPI_ISL_548460, EPI_ISL_548462 to 548465, EPI_ISL_548467, EPI_ISL_548469, EPI_ISL_548484 to 548485, EPI_ISL_548488, EPI_ISL_548580 to 548581, EPI_ISL_548585 to 548587, EPI_ISL_548589 to 548592, EPI_ISL_548594, EPI_ISL_548601, EPI_ISL_548608 to 548609, EPI_ISL_548611, EPI_ISL_548613 to 548615, EPI_ISL_548620, EPI_ISL_548623, EPI_ISL_548625, EPI_ISL_548627 to 548628, EPI_ISL_548634 to 548635, EPI_ISL_548637 to 548638, EPI_ISL_548640 to 548641, EPI_ISL_548646, EPI_ISL_548648, EPI_ISL_548651, EPI_ISL_548653 to 548654, EPI_ISL_548660, EPI_ISL_548662, EPI_ISL_548665 to 548668, EPI_ISL_548674 to 548675, EPI_ISL_548677			
see above	Orange County Public Health Laboratory	Chan-Zuckerberg Biohub	CZB Cliahub Consortium
EPI_ISL_1492583 to 1492619, EPI_ISL_1497618 to 1497678, EPI_ISL_1547502 to 1547505	Orebro University Hospital	Orebro University Hospital	Sundqvist M et al
EPI_ISL_455840 to 455841, EPI_ISL_475128 to 475135, EPI_ISL_475139, EPI_ISL_510817, EPI_ISL_560972, EPI_ISL_582785 to 582789, EPI_ISL_582791 to 582793, EPI_ISL_582841, EPI_ISL_615079, EPI_ISL_615111 to 615114, EPI_ISL_615120			
see above	Orebro klinisk mikrobiologi	The Public Health Agency of Sweden	Anna Risberg; Anna-Malin Linde; Karin Tegmark-Wisell; Maria Lind Karlberg; Mattias Haukland; Mia Brytting; Olov Svartstrom; Oskar Karlsson Lindsjo; Petra Edquist; Reza Advani; Sandra Broddesson; Shamam Muradrasoli
EPI_ISL_1527630 to 1527633	Oregon Department of Agriculture	Diagnostic Virology Laboratory, National Veterinary Services Laboratories, USDA1920 Dayton Avenue, Ames, IA 50010, USA	Emily R. Love; Kerrie M. Franzen; Mary L. Killian; Mia Torchetti; Suelee Robbe-Austerman; Tod P. Stuber
EPI_ISL_1393629, EPI_ISL_1394338, EPI_ISL_1402541, EPI_ISL_1402707, EPI_ISL_1403049 to 1403050, EPI_ISL_1403386, EPI_ISL_1403666, EPI_ISL_1403669, EPI_ISL_1403781 to 1403783, EPI_ISL_1403809, EPI_ISL_1403827, EPI_ISL_1404130, EPI_ISL_1404143, EPI_ISL_1424131 to 1424142, EPI_ISL_1482647, EPI_ISL_1482974 to 1482978, EPI_ISL_1493060 to 1493061, EPI_ISL_1493067 to 1493068, EPI_ISL_1493124, EPI_ISL_1493145, EPI_ISL_1493165 to 1493167, EPI_ISL_1493258 to 1493268, EPI_ISL_1493363 to 1493365, EPI_ISL_1511640, EPI_ISL_1511642, EPI_ISL_1511645, EPI_ISL_1511697, EPI_ISL_1511920, EPI_ISL_1512183, EPI_ISL_1512497, EPI_ISL_1512745, EPI_ISL_1523525 to 1523543, EPI_ISL_1531535 to 1531552			
see above	Oregon State Public Health Laboratory	Oregon State Public Health Laboratory	Eugene Yeboah; G. Libuit; Joel R. Sevinksy; John Fontana; John Fontana and Shane Sevey; Karim Morey; Kevin; LaDonna Grenz; Laura Tsaknadidis; Laura Tsaknaridis; Rafia Razzaque; Shane Sevey; Vanda Makris
EPI_ISL_420794	Oregon State Public Health- Virology section	Pathogen Discovery, Respiratory Viruses Branch, Division of Viral Diseases, Centers for Disease Control and Prevention	Alison S. Laufer Halpin; Anne Uehara; Christopher A. Elkins; Clinton R. Paden; Haibin Wang; Jasmine Padilla; Jing Zhang; Justin Lee; Krista Queen; Mary S. Keckler; Rachel Marine; Suxiang Tong; Yan Li; Ying Tao
EPI_ISL_1394858 to 1394869, EPI_ISL_1404650 to 1404679, EPI_ISL_1405940 to 1405945, EPI_ISL_1416256 to 1416311, EPI_ISL_1423984 to 1423993, EPI_ISL_1494925 to 1494939, EPI_ISL_1501718 to 1501719, EPI_ISL_1501721 to 1501722, EPI_ISL_1501724 to 1501725, EPI_ISL_1501727 to 1501728, EPI_ISL_1501730 to 1501731, EPI_ISL_1501733 to 1501734, EPI_ISL_1501736 to 1501737, EPI_ISL_1501739 to 1501740, EPI_ISL_1501742 to 1501743, EPI_ISL_1501745 to 1501746, EPI_ISL_1501748, EPI_ISL_1501750 to 1501752, EPI_ISL_1501754 to 1501755, EPI_ISL_1501757, EPI_ISL_1501759 to 1501760, EPI_ISL_1501762 to 1501763, EPI_ISL_1501765 to 1501766, EPI_ISL_1501768 to 1501769, EPI_ISL_1501771 to 1501772, EPI_ISL_1501774 to 1501775, EPI_ISL_1501777 to 1501778, EPI_ISL_1501780 to 1501781, EPI_ISL_1501783 to 1501784, EPI_ISL_1501786 to 1501787, EPI_ISL_1501789 to 1501790, EPI_ISL_1504042 to 1504057			
see above	Oregon State University TRACE Project	OSU Center for Genome Research and Biocomputing	Oregon State University TRACE Project
EPI_ISL_475511	Orestadsklinikens VC	The Public Health Agency of Sweden	Anna Risberg; Anna-Malin Linde; Karin Tegmark-Wisell; Maria Lind Karlberg; Mattias Haukland; Mia Brytting; Olov Svartstrom; Oskar Karlsson Lindsjo; Reza Advani; Sandra Broddesson
EPI_ISL_1387237 to 1387241, EPI_ISL_1387243 to 1387259, EPI_ISL_1387261 to 1387275, EPI_ISL_1387277, EPI_ISL_1387279 to 1387282, EPI_ISL_1387284 to 1387296, EPI_ISL_1387298 to 1387299, EPI_ISL_1387301 to 1387304, EPI_ISL_1387306 to 1387311, EPI_ISL_1387313 to 1387314, EPI_ISL_1387316 to 1387318, EPI_ISL_1387320, EPI_ISL_1387322 to 1387325, EPI_ISL_1387327 to 1387329, EPI_ISL_1387332 to 1387340, EPI_ISL_1387342 to 1387349, EPI_ISL_1387351 to 1387352, EPI_ISL_1387354 to 1387361, EPI_ISL_1387363 to 1387364, EPI_ISL_1387367 to 1387370, EPI_ISL_1387373 to 1387376, EPI_ISL_1387378 to 1387383, EPI_ISL_1387385 to 1387386, EPI_ISL_1387388, EPI_ISL_1387390, EPI_ISL_1387393 to 1387398, EPI_ISL_1387401 to 1387402, EPI_ISL_1387405 to 1387407, EPI_ISL_1387409, EPI_ISL_1387411, EPI_ISL_1387413 to 1387414, EPI_ISL_1387416, EPI_ISL_1387418 to 1387422, EPI_ISL_1387425, EPI_ISL_1387427 to 1387429, EPI_ISL_1387432, EPI_ISL_1387436 to 1387437, EPI_ISL_1387439 to 1387440, EPI_ISL_1387443 to 1387446, EPI_ISL_1387448 to 1387449, EPI_ISL_1387452 to 1387455, EPI_ISL_1387461, EPI_ISL_1387463 to 1387468, EPI_ISL_1387470 to 1387472, EPI_ISL_1387475 to 1387476, EPI_ISL_1387478 to 1387482, EPI_ISL_1387484 to 1387487, EPI_ISL_1387489, EPI_ISL_1387491, EPI_ISL_1387494 to 1387495, EPI_ISL_1387497 to 1387510, EPI_ISL_1387512 to 1387513, EPI_ISL_1387515 to 1387523, EPI_ISL_1387525 to 1387537, EPI_ISL_1387539 to 1387541, EPI_ISL_1387543 to 1387545, EPI_ISL_1387548 to 1387551, EPI_ISL_1387553, EPI_ISL_1387555 to 1387580, EPI_ISL_1387582 to 1387586, EPI_ISL_1387588 to 1387598, EPI_ISL_1387600 to 1387603, EPI_ISL_1387605 to 1387611, EPI_ISL_1387613 to 1387617, EPI_ISL_1387619 to 1387626, EPI_ISL_1387628 to 1387630, EPI_ISL_1387632 to 1387634, EPI_ISL_1387640 to 1387644, EPI_ISL_1387646 to 1387648, EPI_ISL_1387650 to 1387654, EPI_ISL_1387657 to 1387658, EPI_ISL_1387660 to 1387669, EPI_ISL_1387671, EPI_ISL_1387673 to 1387674, EPI_ISL_1387676, EPI_ISL_1387678 to 1387683, EPI_ISL_1387686 to 1387688, EPI_ISL_1387690 to 1387693, EPI_ISL_1387695 to 1387705, EPI_ISL_1387707 to 1387708, EPI_ISL_1387710 to 1387715, EPI_ISL_1387721, EPI_ISL_1387723, EPI_ISL_1387725, EPI_ISL_1387730 to 1387732, EPI_ISL_1387735 to 1387736, EPI_ISL_1387739, EPI_ISL_1387743, EPI_ISL_1387745 to 1387751, EPI_ISL_1387754, EPI_ISL_1387756 to 1387758, EPI_ISL_1387760 to 1387762, EPI_ISL_1387765 to 1387770, EPI_ISL_1387772 to 1387773, EPI_ISL_1387780, EPI_ISL_1387782 to 1387785, EPI_ISL_1387788, EPI_ISL_1387791 to 1387792, EPI_ISL_1387795 to 1387799, EPI_ISL_1387801, EPI_ISL_1387804 to 1387822, EPI_ISL_1387824, EPI_ISL_1387826 to 1387836, EPI_ISL_1387838 to 1387839, EPI_ISL_1387841, EPI_ISL_1387843, EPI_ISL_1387845 to 1387847, EPI_ISL_1387849 to 1387853, EPI_ISL_1387855, EPI_ISL_1387857 to 1387863, EPI_ISL_1387866, EPI_ISL_1387872 to 1387877, EPI_ISL_1387881 to 1387883, EPI_ISL_1387889, EPI_ISL_1387893, EPI_ISL_1387898 to 1387903, EPI_ISL_1387905, EPI_ISL_1387907 to 1387908, EPI_ISL_1387914, EPI_ISL_1387916 to 1387919, EPI_ISL_1387921, EPI_ISL_1387923, EPI_ISL_1387926 to 1387931, EPI_ISL_1387934, EPI_ISL_1387938, EPI_ISL_1387942, EPI_ISL_1387945, EPI_ISL_1387947, EPI_ISL_1387950, EPI_ISL_1387954 to 1387965, EPI_ISL_1387967, EPI_ISL_1387970 to 1387975, EPI_ISL_1387979, EPI_ISL_1387981 to 1387984, EPI_ISL_1387989, EPI_ISL_1387991 to 1388003, EPI_ISL_1388005 to 1388011, EPI_ISL_1388013 to 1388015, EPI_ISL_1388017 to 1388020, EPI_ISL_1388023 to 1388024, EPI_ISL_1388029 to 1388031, EPI_ISL_1388033 to 1388037, EPI_ISL_1388040 to 1388042, EPI_ISL_1388044 to 1388049, EPI_ISL_1388052 to 1388053, EPI_ISL_1388056, EPI_ISL_1388059 to 1388062, EPI_ISL_1388064 to 1388065, EPI_ISL_1388068, EPI_ISL_1388071 to 1388072, EPI_ISL_1388074, EPI_ISL_1388076 to 1388082, EPI_ISL_1388084 to 1388086, EPI_ISL_1388089 to 1388090, EPI_ISL_1388092 to 1388093, EPI_ISL_1388095 to 1388104, EPI_ISL_1388111, EPI_ISL_1388113, EPI_ISL_1388115, EPI_ISL_1388120, EPI_ISL_1388123, EPI_ISL_1388125, EPI_ISL_1388127, EPI_ISL_1388129, EPI_ISL_1388135, EPI_ISL_1388138, EPI_ISL_1388140, EPI_ISL_1388142, EPI_ISL_1388148, EPI_ISL_1388151, EPI_ISL_1388153, EPI_ISL_1388155, EPI_ISL_1388157, EPI_ISL_1388159, EPI_ISL_1388161, EPI_ISL_1388163, EPI_ISL_1388165, EPI_ISL_1388169, EPI_ISL_1388172 to 1388173, EPI_ISL_1388176, EPI_ISL_1388178, EPI_ISL_1388180, EPI_ISL_1388186, EPI_ISL_1388190, EPI_ISL_1388192, EPI_ISL_1388196, EPI_ISL_1388205, EPI_ISL_1388207, EPI_ISL_1388209, EPI_ISL_1388211, EPI_ISL_1388213, EPI_ISL_1388215, EPI_ISL_1388217, EPI_ISL_1388219, EPI_ISL_1388221, EPI_ISL_1388223, EPI_ISL_1388225, EPI_ISL_1388231, EPI_ISL_1388236 to 1388237, EPI_ISL_1388241, EPI_ISL_1388243, EPI_ISL_1388246, EPI_ISL_1388248, EPI_ISL_1388250, EPI_ISL_1388254, EPI_ISL_1388256, EPI_ISL_1388260, EPI_ISL_1388266, EPI_ISL_1388268, EPI_ISL_1388274, EPI_ISL_1388282, EPI_ISL_1388284, EPI_ISL_1388286, EPI_ISL_1388288, EPI_ISL_1388294, EPI_ISL_1388296, EPI_ISL_1388302 to 1388303, EPI_ISL_1388307, EPI_ISL_1388309, EPI_ISL_1388311, EPI_ISL_1388313, EPI_ISL_1388315, EPI_ISL_1388327, EPI_ISL_1388331, EPI_ISL_1388335, EPI_ISL_1388337, EPI_ISL_1388343, EPI_ISL_1388345, EPI_ISL_1388347, EPI_ISL_1388353, EPI_ISL_1388357, EPI_ISL_1388359, EPI_ISL_1388361, EPI_ISL_1388363, EPI_ISL_1388369, EPI_ISL_1388371, EPI_ISL_1388374, EPI_ISL_1388378, EPI_ISL_1388381, EPI_ISL_1388383, EPI_ISL_1388387, EPI_ISL_1388391, EPI_ISL_1388395, EPI_ISL_1388399, EPI_ISL_1388411, EPI_ISL_1388415, EPI_ISL_1388418, EPI_ISL_1388424, EPI_ISL_1388426, EPI_ISL_1388428, EPI_ISL_1388430 to 1388431, EPI_ISL_1388433, EPI_ISL_1388435, EPI_ISL_1388439, EPI_ISL_1388441, EPI_ISL_1388443, EPI_ISL_1388445 to 1388446, EPI_ISL_1388448, EPI_ISL_1388450, EPI_ISL_1388452, EPI_ISL_1388454, EPI_ISL_1388456, EPI_ISL_1388458 to 1388459, EPI_ISL_1388461, EPI_ISL_1388463, EPI_ISL_1388465, EPI_ISL_1388467 to 1388468, EPI_ISL_1388470, EPI_ISL_1388475, EPI_ISL_1388477, EPI_ISL_1388479, EPI_ISL_1388485, EPI_ISL_1388487, EPI_ISL_1388489, EPI_ISL_1388491, EPI_ISL_1388493, EPI_ISL_1388495 to 1388496, EPI_ISL_1388498, EPI_ISL_1388500, EPI_ISL_1388502, EPI_ISL_1388505 to 1388506, EPI_ISL_1388508, EPI_ISL_1388510, EPI_ISL_1388513, EPI_ISL_1388515, EPI_ISL_1388519, EPI_ISL_1388521, EPI_ISL_1388523, EPI_ISL_1388525, EPI_ISL_1388527, EPI_ISL_1388531, EPI_ISL_1388533, EPI_ISL_1388535, EPI_ISL_1388537, EPI_ISL_1388539, EPI_ISL_1388541, EPI_ISL_1388545, EPI_ISL_1388549, EPI_ISL_1388551, EPI_ISL_1388553, EPI_ISL_1388555, EPI_ISL_1388557, EPI_ISL_1388559, EPI_ISL_1388563, EPI_ISL_1388565, EPI_ISL_1388567, EPI_ISL_1388569, EPI_ISL_1388573, EPI_ISL_1388577, EPI_ISL_1388581 to 1388582, EPI_ISL_1388584, EPI_ISL_1388589, EPI_ISL_1388591, EPI_ISL_1388593 to 1388594, EPI_ISL_1388596, EPI_ISL_1388599, EPI_ISL_1388601 to 1388602, EPI_ISL_1388605, EPI_ISL_1388607, EPI_ISL_1388609, EPI_ISL_1388611, EPI_ISL_1388613, EPI_ISL_1388615, EPI_ISL_1388617, EPI_ISL_1388619, EPI_ISL_1388621, EPI_ISL_1388623, EPI_ISL_1388625, EPI_ISL_1388627 to 1388628, EPI_ISL_1388630, EPI_ISL_1388632, EPI_ISL_1388634, EPI_ISL_1388636, EPI_ISL_1388638, EPI_ISL_1388640 to 1388641, EPI_ISL_1475759 to 1475760, EPI_ISL_1475762, EPI_ISL_1475767 to 1475768, EPI_ISL_1475772 to 1475773, EPI_ISL_1475776, EPI_ISL_1475779 to 1475784, EPI_ISL_1475786 to 1475789, EPI_ISL_1475791 to 1475796, EPI_ISL_1475798 to 1475802, EPI_ISL_1475804 to 1475805, EPI_ISL_1475808 to 1475819, EPI_ISL_1475821 to 1475825, EPI_ISL_1475827 to 1475828, EPI_ISL_1475830, EPI_ISL_1475832 to 1475833, EPI_ISL_1475836 to 1475838, EPI_ISL_1475840 to 1475841, EPI_ISL_1475843 to 1475844, EPI_ISL_1475847, EPI_ISL_1475850, EPI_ISL_1475852 to 1475855, EPI_ISL_1475857 to 1475859, EPI_ISL_1475861, EPI_ISL_1475863, EPI_ISL_1475865 to 1475866, EPI_ISL_1475868 to 1475872, EPI_ISL_1475874, EPI_ISL_1475876 to 1475890, EPI_ISL_1475894 to 1475897, EPI_ISL_1475901 to 1475904, EPI_ISL_1475907 to 1475919, EPI_ISL_1475921 to 1475926, EPI_ISL_1475928, EPI_ISL_1475930 to 1475935, EPI_ISL_1475937 to 1475938, EPI_ISL_1475942, EPI_ISL_1475947 to 1475951, EPI_ISL_1475953 to 1475954, EPI_ISL_1475956, EPI_ISL_1475960 to 1475961, EPI_ISL_1475963 to 1475965, EPI_ISL_1475967 to 1475968, EPI_ISL_1475970 to 1475973, EPI_ISL_1475977 to 1475980, EPI_ISL_1475982 to 1475983, EPI_ISL_1475985 to 1475991, EPI_ISL_1475993 to 1475997, EPI_ISL_1476000 to 1476002, EPI_ISL_1476004 to 1476008, EPI_ISL_1476010, EPI_ISL_1476012 to 1476016, EPI_ISL_1476018 to 1476020, EPI_ISL_1476023 to 1476030, EPI_ISL_1476033 to 1476039, EPI_ISL_1476041 to 1476044, EPI_ISL_1476046, EPI_ISL_1476049 to 1476050, EPI_ISL_1476052 to 1476055, EPI_ISL_1476057 to 1476062, EPI_ISL_1476065 to 1476066, EPI_ISL_1476070, EPI_ISL_1476072 to 1476074, EPI_ISL_1476078 to 1476079, EPI_ISL_1476085, EPI_ISL_1476095, EPI_ISL_1476098 to 1476099, EPI_ISL_1476101 to 1476106, EPI_ISL_1476108 to 1476109, EPI_ISL_1476111 to 1476114, EPI_ISL_1476116 to 1476131, EPI_ISL_1476133 to 1476135, EPI_ISL_1476137 to 1476138, EPI_ISL_1476140 to 1476143, EPI_ISL_1476149, EPI_ISL_1476151 to 1476157, EPI_ISL_1476160, EPI_ISL_1476165 to 1476166, EPI_ISL_1476168, EPI_ISL_1476171, EPI_ISL_1476173 to 1476174, EPI_ISL_1476176 to 1476179, EPI_ISL_1476182, EPI_ISL_1476184 to 1476185, EPI_ISL_1476189 to 1476190, EPI_ISL_1476192 to 1476193, EPI_ISL_1476195 to 1476197, EPI_ISL_1476199, EPI_ISL_1476201, EPI_ISL_1476204, EPI_ISL_1476206 to 1476208, EPI_ISL_1476215, EPI_ISL_1476219, EPI_ISL_1476222 to 1476225, EPI_ISL_1476227 to 1476228, EPI_ISL_1476231 to 1476233, EPI_ISL_1476235 to 1476282, EPI_ISL_1476284 to 1476290, EPI_ISL_1476292 to 1476295, EPI_ISL_1476299 to 1476302, EPI_ISL_1476304 to 1476308, EPI_ISL_1476311 to 1476314, EPI_ISL_1476316, EPI_ISL_1476319, EPI_ISL_1476323 to 1476325, EPI_ISL_1476327 to 1476330, EPI_ISL_1476332 to 1476337, EPI_ISL_1476340 to 1476342, EPI_ISL_1476344 to 1476363, EPI_ISL_1476365 to 1476366, EPI_ISL_1476370, EPI_ISL_1476372 to			

1476373, EPI_ISL_1476375 to 1476376, EPI_ISL_1476378 to 1476379, EPI_ISL_1476381 to 1476389, EPI_ISL_1476391 to 1476392, EPI_ISL_1476395, EPI_ISL_1476397 to 1476398, EPI_ISL_1476400 to 1476407, EPI_ISL_1476411, EPI_ISL_1476414 to 1476416, EPI_ISL_1476418 to 1476419, EPI_ISL_1476421 to 1476422, EPI_ISL_1476424, EPI_ISL_1476426, EPI_ISL_1476430 to 1476432, EPI_ISL_1476435 to 1476436, EPI_ISL_1476438 to 1476441, EPI_ISL_1476443, EPI_ISL_1476446, EPI_ISL_1476451 to 1476458, EPI_ISL_1476460 to 1476461, EPI_ISL_1476464 to 1476467, EPI_ISL_1476469, EPI_ISL_1476471 to 1476472, EPI_ISL_1476474, EPI_ISL_1476476 to 1476477, EPI_ISL_1476480 to 1476488, EPI_ISL_1476490 to 1476494, EPI_ISL_1476496, EPI_ISL_1476498 to 1476516, EPI_ISL_1476518 to 1476527, EPI_ISL_1476529 to 1476548, EPI_ISL_1546251, EPI_ISL_1546253 to 1546255, EPI_ISL_1546257 to 1546268, EPI_ISL_1546270, EPI_ISL_1546273 to 1546277, EPI_ISL_1546279 to 1546286, EPI_ISL_1546289 to 1546291, EPI_ISL_1546293, EPI_ISL_1546295 to 1546329, EPI_ISL_1546331 to 1546336, EPI_ISL_1546339 to 1546342, EPI_ISL_1546344 to 1546345, EPI_ISL_1546347 to 1546356, EPI_ISL_1546358 to 1546365, EPI_ISL_1546370 to 1546371, EPI_ISL_1546373 to 1546385, EPI_ISL_1546387 to 1546388, EPI_ISL_1546390, EPI_ISL_1546392, EPI_ISL_1546394 to 1546396, EPI_ISL_1546398 to 1546403, EPI_ISL_1546405, EPI_ISL_1546407 to 1546415, EPI_ISL_1546417, EPI_ISL_1546419, EPI_ISL_1546421 to 1546423, EPI_ISL_1546426, EPI_ISL_1546428, EPI_ISL_1546430 to 1546461, EPI_ISL_1546463 to 1546470, EPI_ISL_1546472 to 1546478, EPI_ISL_1546480, EPI_ISL_1546482 to 1546484, EPI_ISL_1546486 to 1546489, EPI_ISL_1546491 to 1546499, EPI_ISL_1546501 to 1546503, EPI_ISL_1546505, EPI_ISL_1546507 to 1546508, EPI_ISL_1546511 to 1546512, EPI_ISL_1546514 to 1546518, EPI_ISL_1546520, EPI_ISL_1546523 to 1546527, EPI_ISL_1546530, EPI_ISL_1546533 to 1546539, EPI_ISL_1546541, EPI_ISL_1546544 to 1546547, EPI_ISL_1546549, EPI_ISL_1546553, EPI_ISL_1546557 to 1546562, EPI_ISL_1546564 to 1546574, EPI_ISL_1546576 to 1546583, EPI_ISL_1546585, EPI_ISL_1546587 to 1546591, EPI_ISL_1546593 to 1546623, EPI_ISL_1546625 to 1546629, EPI_ISL_1546631 to 1546633, EPI_ISL_1546635, EPI_ISL_1546637 to 1546642, EPI_ISL_1546644 to 1546645, EPI_ISL_1546648 to 1546649, EPI_ISL_1546651 to 1546657, EPI_ISL_1546660 to 1546661, EPI_ISL_1546663 to 1546666, EPI_ISL_1546668 to 1546672, EPI_ISL_1546674 to 1546681, EPI_ISL_1546683 to 1546684, EPI_ISL_1546686 to 1546693, EPI_ISL_1546695 to 1546713, EPI_ISL_1546715 to 1546728, EPI_ISL_1546730 to 1546742, EPI_ISL_1546744 to 1546745, EPI_ISL_1546748 to 1546749, EPI_ISL_1546751 to 1546755, EPI_ISL_1546758, EPI_ISL_1546760, EPI_ISL_1546762 to 1546764, EPI_ISL_1546768 to 1546773, EPI_ISL_1546775 to 1546780, EPI_ISL_1546782 to 1546791, EPI_ISL_1546793 to 1546805, EPI_ISL_1546808 to 1546814, EPI_ISL_1546816 to 1546818, EPI_ISL_1546820 to 1546825, EPI_ISL_1546828 to 1546829, EPI_ISL_1546834, EPI_ISL_1546840, EPI_ISL_1546842 to 1546844, EPI_ISL_1546846 to 1546861, EPI_ISL_1546864 to 1547227			
see above	Originating lab: Wales Specialist Virology Centre Sequencing lab: Pathogen Genomics Unit	Public Health Wales Microbiology Cardiff Wales Specialist Virology Centre	Alec Birchley; Alexander Adams; Amy Gaskin; Angela Marchbank; Bree Gatica-Wilcox; Catherine Moore; Jason Coombes; Joanne Watkins; Joel Southgate; Johnathan Evans; Laura Gifford; Lauren Gilbert; Lee Graham; Malorie Perry; Matthew Bull; Nicole Pacchiarini; Sally Corden; Sara Kumziene-Summerhayes; Sara Rey; Sarah Taylor; Simon Cottrell; Sophie Jones; Tom Connor
EPI_ISL_455100, EPI_ISL_475519	Orsa VC	The Public Health Agency of Sweden	Anna Risberg; Anna-Karin Lundqvist; Anna-Malin Linde; Karin Tegmark-Wisell; Maria Lind Karlberg; Mattias Haukland; Mia Brytting; Olov Svartstrom; Oskar Karlsson Lindsjo; Reza Advani; Sandra Broddesson; Theresa Enkirch
EPI_ISL_1427154 to 1427165, EPI_ISL_1427607 to 1427609, EPI_ISL_1427729 to 1427732, EPI_ISL_1427784 to 1427799, EPI_ISL_1428610, EPI_ISL_1429579 to 1429582, EPI_ISL_1429584 to 1429587, EPI_ISL_1430552 to 1430554, EPI_ISL_1430570 to 1430571, EPI_ISL_1430579, EPI_ISL_1430581, EPI_ISL_1430585 to 1430624, EPI_ISL_1430626, EPI_ISL_1430635 to 1430638, EPI_ISL_1430653 to 1430655, EPI_ISL_1430668 to 1430671, EPI_ISL_1430681, EPI_ISL_1430700, EPI_ISL_1430721, EPI_ISL_1430734, EPI_ISL_1430824, EPI_ISL_1431390 to 1431443, EPI_ISL_1431577, EPI_ISL_1431719, EPI_ISL_1432329, EPI_ISL_1432331, EPI_ISL_1432333, EPI_ISL_1432335, EPI_ISL_1432337, EPI_ISL_1432339 to 1432340			
see above	Osaka Institute of Public Health, Morinomiya Center	Pathogen Genomics Center, National Institute of Infectious Diseases	; Chang-Kweng Lim; Chikako Shimokawa; Daisuke Kobayashi; Eri Nakayama; Eunsil Park; Hussein H. Aly; Ken Miyazawa; Kentaro Itokawa; Kousho Wakae; Makoto Kuroda; Masanori Hashino; Minoru Nagi; Motohiko Ogawa; Nozomu Hanaoka; Rina Tanaka; Shigeru Tanjima; Takahiro Maeki; Takanobu Kato; Takashi Sakudoh; Tsuguto Fujimoto; Tsuyoshi Sekizuka; Yasutaka Hoshino; Yoshihiro Kaku
EPI_ISL_417483	Oslo University Hospital, Department of Medical Microbiology	Norwegian Institute of Public Health	Hilde Elshaug; Kamilla Heddeland Instefjord; Karoline Bragstad; Kathrine Stene-Johansen; Olav Hungnes
EPI_ISL_417484, EPI_ISL_417488 to 417490, EPI_ISL_420135, EPI_ISL_420150, EPI_ISL_471175, EPI_ISL_471177, EPI_ISL_481214 to 481215, EPI_ISL_481217 to 481218, EPI_ISL_493351 to 493355, EPI_ISL_493385 to 493389, EPI_ISL_500800, EPI_ISL_549029 to 549035, EPI_ISL_590878, EPI_ISL_590900, EPI_ISL_590908 to 590910, EPI_ISL_590913 to 590914, EPI_ISL_590916, EPI_ISL_590918, EPI_ISL_590923, EPI_ISL_590927 to 590928, EPI_ISL_590930, EPI_ISL_590934 to 590935, EPI_ISL_590978 to 590980, EPI_ISL_591011, EPI_ISL_591020, EPI_ISL_1490260, EPI_ISL_1547627 to 1547632			
see above	Oslo University Hospital, Department of Medical Microbiology	Norwegian Institute of Public Health, Department of Virology	Atiya R Ali; Debech Nadia; Engebretsen Serina Beate; Garcia Llorente Ignacio; Hilde Elshaug; Hilde Synnøve Vollan; Hilde Vollan; Jon Bråte; Kamilla Heddeland Instefjord; Karoline Bragstad; Kathrine Stene-Johansen; Marie Paulsen Madsen; Olav Hungnes; Pedersen Benedikte Nevjen; Rasmus Riis Kopperud
EPI_ISL_458066 to 458069, EPI_ISL_495274 to 495297	Osmania Medical College	CSIR-Centre for Cellular and Molecular Biology	Archana Bharadwaj Siva; Dhiviya Vedagiri; Divya Gupta; Divya Tej Sowpati; Karthik Bharadwaj Tallapaka; Krishnan Harinivas Harshan; Lamuk Zaveri; M Soujanya Reddy; Mahboob Khan; Namami Gaur; Nikhil Hajirnis; Onkar Kulkarni; Payel Mukherjee; Pratheusa Maccha; Priya Singh; Purushotham Vodnala; Rakesh K Mishra; Sakshi Shambhavi; Santosh Kumar Kuncha; Shagufta Khan; Shashikala Reddy; Sofia Banu; Tulasi Nagabandi; Vishal Sah
EPI_ISL_447902	Osmania University	Osmania University	Amit; An and Kumar, K.; Bosinger; Kalyani, P.; Nagamani, K.; Pankaj Singh, D.; Radhakrishna, M.; Raja Rao, M.; Ravikumar, P.; S.E. and Rama, A.; Sunitha, P.; Thrilok Chander, B.; U.A.
EPI_ISL_528922, EPI_ISL_528924, EPI_ISL_528926 to 528928, EPI_ISL_529018, EPI_ISL_529026	Ospedale "Giuseppe Mazzini"-Teramo	Istitituto Zooprofilattico Sperimentale dell'Abruzzo e Molise "G.Caporale"	Ancora M; Cammà C; Curini V; Di Domenico M; Di Pasquale A; Lorusso A; Mangone I; Marcacci M; Puglia I; Rinaldi A; Savini G.
EPI_ISL_529014 to 529015, EPI_ISL_529022	Ospedale "Ss. Annunziata"	Istitituto Zooprofilattico Sperimentale dell'Abruzzo e Molise "G.Caporale"	Ancora M; Cammà C; Curini V; Di Domenico M; Di Pasquale A; Lorusso A; Mangone I; Marcacci M; Puglia I; Rinaldi A; Savini G.
EPI_ISL_1391073 to 1391076	Ospedale Casa Sollievo della Sofferenza	Istituto Zooprofilattico Sperimentale della Puglia e della Basilicata	Bianco A.; Capozzi L.; Del Sambro L.; Difato L.; Giannico A; Labonia M.; Parisi A.; Ridolfi D.; Simone D.
EPI_ISL_420564	Ospedale Civile Castel Di Sangro	Istituto Zooprofilattico Sperimentale dell'Abruzzo e Molise "G.Caporale"	Ancora M; Cammà C; Curini V; Di Domenico M; Di Pasquale A; Lorusso A; Mangone I; Marcacci M; Puglia I; Rinaldi A; Savini G
EPI_ISL_418260 to 418261, EPI_ISL_420563, EPI_ISL_429228, EPI_ISL_429230 to 429235	Ospedale Civile Giuseppe Mazzini	Istituto Zooprofilattico Sperimentale dell'Abruzzo e Molise "G. Caporale"	Ancora M; Camma C; Cammà C; Curini V; Di Domenico M; Di Pasquale A; Lorusso A; Mangone I; Marcacci M; Puglia I; Rinaldi A; Savini G; Savini G.
EPI_ISL_420565, EPI_ISL_420568 to 420569, EPI_ISL_420583, EPI_ISL_420592, EPI_ISL_435145, EPI_ISL_436723	Ospedale Civile Giuseppe Mazzini	Istituto Zooprofilattico Sperimentale dell'Abruzzo e Molise "G.Caporale"	Ancora M; Cammà C; Curini V; Di Domenico M; Di Pasquale A; Lorusso A; Mangone I; Marcacci M; Puglia I; Rinaldi A; Savini G
EPI_ISL_418257	Ospedale Civile Giuseppe Mazzini, Teramo	Istituto Zooprofilattico Sperimentale dell'Abruzzo e Molise "G.Caporale"	Ancora M; Cammà C; Curini V; Di Domenico M; Di Pasquale A; Lorusso A; Mangone I; Marcacci M; Puglia I; Rinaldi A; Savini G.
EPI_ISL_528990	Ospedale Civile Maria SS. dello Splendore	Istitituto Zooprofilattico Sperimentale dell'Abruzzo e Molise "G.Caporale"	Ancora M; Cammà C; Curini V; Di Domenico M; Di Pasquale A; Lorusso A; Mangone I; Marcacci M; Puglia I; Rinaldi A; Savini G.
EPI_ISL_429236	Ospedale Civile S. Liberatore di Atri	Istituto Zooprofilattico Sperimentale dell'Abruzzo e Molise "G. Caporale"	Ancora M; Camma C; Curini V; Di Domenico M; Di Pasquale A; Lorusso A; Mangone I; Marcacci M; Puglia I; Rinaldi A; Savini G
EPI_ISL_436719 to 436722, EPI_ISL_436724, EPI_ISL_436731 to 436732	Ospedale Civile S. Liberatore di Atri	Istituto Zooprofilattico Sperimentale dell'Abruzzo e Molise "G.Caporale"	Ancora M; Cammà C; Curini V; Di Domenico M; Di Pasquale A; Lorusso A; Mangone I; Marcacci M; Puglia I; Rinaldi A; Savini G
EPI_ISL_528919, EPI_ISL_528923, EPI_ISL_528929, EPI_ISL_528993, EPI_ISL_529007, EPI_ISL_529009, EPI_ISL_529020 to 529021			
see above	Ospedale Civile S. Liberatore-Atri	Istitituto Zooprofilattico Sperimentale dell'Abruzzo e Molise "G.Caporale"	Ancora M; Cammà C; Curini V; Di Domenico M; Di Pasquale A; Lorusso A; Mangone I; Marcacci M; Puglia I; Rinaldi A; Savini G.
EPI_ISL_1464621, EPI_ISL_1492581	Ospedale Cristo Re	INMI Lazzaro Spallanzani IRCCS	A Di Caro; B Bartolini; CEM Gruber; E Giombini; F Messina; F Santini; G Bonfiglio; M Rueca; MR Capobianchi; O Butera
EPI_ISL_1492564	Ospedale F.Spaziani	INMI Lazzaro Spallanzani IRCCS	A Di Caro; B Bartolini; C Gargiulo; CEM Gruber; E Giombini; F Messina; F Santini; G Bonfiglio; G Brocco; M Rueca; MR Capobianchi; O Butera; R Pulselli
EPI_ISL_1391020 to 1391023, EPI_ISL_1391029 to 1391037	Ospedale Generale Regionale F. Miulli	Istituto Zooprofilattico Sperimentale della Puglia e della Basilicata	Bianco A.; Capozzi L.; Ceci E.; Del Sambro L.; Parisi A.; Simone D.
EPI_ISL_1524409 to 1524411, EPI_ISL_1524620 to 1524623	Ospedale Magalini	Istituto Zooprofilattico Sperimentale delle Venezie	Adelaide Milani; Alessia Schivo; Alice Fusaro; Ambra Pastori; Annalisa Salviato; Antonia Ricci; Calogero Terregino; Edoardo Giussani; Elisa Palumbo; Erika Giorgia Quaranta; Isabella Monne; Luca Tassoni
EPI_ISL_1524466, EPI_ISL_1524539 to 1524547	Ospedale Magalini	Istituto di Genomica Applicata; Istituto Zooprofilattico Sperimentale delle Venezie	Alessia Schivo; Alice Fusaro; Ambra Pastori; Annalisa Salviato; Antonia Ricci; Calogero Terregino; Davide Scaglione; Edoardo Giussani; Eleonora Paparelli; Erika Giorgia Quaranta; Francesca Bruno; Gabriele Magris; Irena Jurman; Isabella Monne; Luca Tassoni; Maria Varotto; Michele Morgante; Silvia Ormelli; Valeria D'Amico; Vera Vendramin
EPI_ISL_1524625 to 1524630	Ospedale Mater Salutis	Istituto Zooprofilattico Sperimentale delle Venezie	Adelaide Milani; Alessia Schivo; Alice Fusaro; Ambra Pastori; Annalisa Salviato; Antonia Ricci; Calogero Terregino; Edoardo Giussani; Elisa Palumbo; Erika Giorgia Quaranta; Isabella Monne; Luca Tassoni
EPI_ISL_1524467 to 1524468, EPI_ISL_1524561 to 1524568	Ospedale Mater Salutis	Istituto di Genomica Applicata; Istituto Zooprofilattico Sperimentale delle Venezie	Alessia Schivo; Alice Fusaro; Ambra Pastori; Annalisa Salviato; Antonia Ricci; Calogero Terregino; Davide Scaglione; Edoardo Giussani; Eleonora Paparelli; Erika Giorgia Quaranta; Francesca Bruno; Gabriele Magris; Irena Jurman; Isabella Monne; Luca Tassoni; Maria Varotto; Michele Morgante; Silvia Ormelli; Valeria D'Amico; Vera Vendramin
EPI_ISL_429229	Ospedale Regionale San Salvatore	Istituto Zooprofilattico Sperimentale dell'Abruzzo e Molise "G.	Ancora M; Camma C; Curini V; Di Domenico M; Di Pasquale A; Lorusso A; Mangone I; Marcacci M; Puglia I; Rinaldi A; Savini G

		Caporale"	
EPI_ISL_420566 to 420567, EPI_ISL_436718	Ospedale Regionale San Salvatore	Istituto Zooprofilattico Sperimentale dell'Abruzzo e Molise "G.Caporale"	Ancora M; Cammà C; Curini V; Di Domenico M; Di Pasquale A; Lorusso A; Mangone I; Marcacci M; Puglia I; Rinaldi A; Savini G
EPI_ISL_528925	Ospedale Regionale San Salvatore-L'Aquila	Istitituto Zooprofilattico Sperimentale dell'Abruzzo e Molise "G.Caporale"	Ancora M; Cammà C; Curini V; Di Domenico M; Di Pasquale A; Lorusso A; Mangone I; Marcacci M; Puglia I; Rinaldi A; Savini G.
EPI_ISL_1464622, EPI_ISL_1492579 to 1492580, EPI_ISL_1495999	Ospedale S.M. Goretti	INMI Lazzaro Spallanzani IRCCS	A Di Caro; A Lucci; B Bartolini; CEM Gruber; E Giombini; F Messina; F Santini; G Bonfiglio; L Corso; L Di Biase; M Rueca; MR Capobianchi; O Butera; S Pignalosa
EPI_ISL_435148, EPI_ISL_435150 to 435151	Ospedale SS Annunziata	Istituto Zooprofilattico Sperimentale dell'Abruzzo e Molise "G.Caporale"	Ancora M; Cammà C; Curini V; Di Domenico M; Di Pasquale A; Lorusso A; Mangone I; Marcacci M; Puglia I; Rinaldi A; Savini G
EPI_ISL_528991 to 528992, EPI_ISL_529016	Ospedale SS Annunziata-Sulmona	Istitituto Zooprofilattico Sperimentale dell'Abruzzo e Molise "G.Caporale"	Ancora M; Cammà C; Curini V; Di Domenico M; Di Pasquale A; Lorusso A; Mangone I; Marcacci M; Puglia I; Rinaldi A; Savini G.
EPI_ISL_1524406 to 1524408, EPI_ISL_1524624	Ospedale San Bonifacio	Istituto Zooprofilattico Sperimentale delle Venezie	Adelaide Milani; Alessia Schivo; Alice Fusaro; Ambra Pastori; Annalisa Salviato; Antonia Ricci; Calogero Terregino; Edoardo Giussani; Elisa Palumbo; Erika Giorgia Quaranta; Isabella Monne; Luca Tassoni
EPI_ISL_1464623	Ospedale San Camillo De Lellis	INMI Lazzaro Spallanzani IRCCS	A De Luca; A Di Caro; B Bartolini; CEM Gruber; E Giombini; F Messina; F Santini; G Bonfiglio; L Casertano; M Rueca; MR Capobianchi; O Butera; S Venarubea
EPI_ISL_1524734	Ospedale San Camillo De Lellis di Rieti	INMI Lazzaro Spallanzani IRCCS	A De Luca; A Di Caro; B Bartolini; CEM Gruber; E Giombini; F Messina; F Santini; G Bonfiglio; L Casertano; M Rueca; MR Capobianchi; O Butera; S Venarubea
EPI_ISL_1492567, EPI_ISL_1492571, EPI_ISL_1492582, EPI_ISL_1524742	Ospedale San Filippo Neri	INMI Lazzaro Spallanzani IRCCS	A Di Caro; A Tamburro; B Bartolini; CEM Gruber; E Giombini; F Messina; F Santini; G Bonfiglio; M Meledandri; M Rueca; ML Schiavone; MR Capobianchi; O Butera
EPI_ISL_1492577, EPI_ISL_1524735 to 1524736, EPI_ISL_1524740 to 1524741	Ospedale San Giovanni Evangelista	INMI Lazzaro Spallanzani IRCCS	A Di Caro; B Bartolini; CEM Gruber; D Cerini; D Di Fusco; E Giombini; F Messina; F Santini; G Bonfiglio; M Rueca; MR Capobianchi; O Butera
EPI_ISL_1391065 to 1391072	Ospedale Santa Caterina Novella	Istituto Zooprofilattico Sperimentale della Puglia e della Basilicata	Bianco A.; Bruno A. R.; Capozzi L.; Del Sambro L.; Difato L.; Parisi A.; Simone D.
EPI_ISL_1391077 to 1391097	Ospedale Santissima Annunziata	Istituto Zooprofilattico Sperimentale della Puglia e della Basilicata	Bianco A.; Capozzi L.; Del Sambro L.; Difato L.; Giannico A; Parisi A.; Ridolfi D.; Simone D.; Tampoia M.
EPI_ISL_1391058 to 1391064	Ospedale Vito Fazzi	Istituto Zooprofilattico Sperimentale della Puglia e della Basilicata	Bianco A.; Capozzi L.; Del Sambro L.; Difato L.; Lobreglio G.; Parisi A.; Simone D.
EPI_ISL_1492569	Ospedale dei Castelli	INMI Lazzaro Spallanzani IRCCS	A Di Caro; B Bartolini; CEM Gruber; E Giombini; F Messina; F Santini; G Bonfiglio; M Rueca; MR Capobianchi; O Butera
EPI_ISL_1464624 to 1464625, EPI_ISL_1492565, EPI_ISL_1524729 to 1524733	Ospedale di Genzano - ASL RM 6	INMI Lazzaro Spallanzani IRCCS	A Di Caro; B Bartolini; CEM Gruber; E Conti; E Giombini; F Messina; F Santini; G Bonfiglio; G Tramini; M Rueca; MR Capobianchi; O Butera
EPI_ISL_1391038 to 1391052, EPI_ISL_1497610	Ospedale di Venere	Istituto Zooprofilattico Sperimentale della Puglia e della Basilicata	Bianco A.; Capozzi L.; Del Sambro L.; Giannico A.; Iacobellis M.; Parisi A.; Ridolfi D.; Simone D.
EPI_ISL_418256	Ospedale "San Liberatore" di Atri	Istituto Zooprofilattico Sperimentale dell'Abruzzo e Molise "G. Caporale"	Ancora M; Cammà C; Curini V; Di Domenico M; Di Pasquale A; Lorusso A; Mangone I; Marcacci M; Puglia I; Rinaldi A; Savini G.
EPI_ISL_475124, EPI_ISL_476145 to 476147, EPI_ISL_548248	Ostersund klinisk mikrobiologi	The Public Health Agency of Sweden	Anna Risberg; Anna-Malin Linde; Karin Tegmark-Wisell; Maria Lind Karlberg; Mattias Haukland; Mia Brytting; Olov Svartstrom; Oskar Karlsson Lindsjo; Petra Edquist; Reza Advani; Sandra Broddesson; Shamam Muradrasoli
EPI_ISL_449786, EPI_ISL_471174, EPI_ISL_481207, EPI_ISL_481209 to 481213, EPI_ISL_493350, EPI_ISL_549042 to 549047, EPI_ISL_549092 to 549111, EPI_ISL_549113 to 549118, EPI_ISL_549126 to 549131, EPI_ISL_549133 to 549142, EPI_ISL_549144 to 549153, EPI_ISL_549155 to 549163, EPI_ISL_590894 to 590897, EPI_ISL_590921 to 590922, EPI_ISL_590931, EPI_ISL_590948, EPI_ISL_590986 to 591006, EPI_ISL_591009, EPI_ISL_591015 to 591017, EPI_ISL_1402932 to 1402936, EPI_ISL_1402938 to 1402940, EPI_ISL_1402944, EPI_ISL_1402947, EPI_ISL_1402949 to 1402950, EPI_ISL_1402956 to 1402967, EPI_ISL_1402969 to 1402970, EPI_ISL_1402972 to 1402973, EPI_ISL_1402975 to 1402976, EPI_ISL_1490192, EPI_ISL_1490194, EPI_ISL_1490266 to 1490267, EPI_ISL_1490381, EPI_ISL_1490383 to 1490384, EPI_ISL_1490386 to 1490390, EPI_ISL_1490408, EPI_ISL_1490410 to 1490411, EPI_ISL_1490414, EPI_ISL_1490416 to 1490418, EPI_ISL_1490492 to 1490493, EPI_ISL_1490498, EPI_ISL_1490500 to 1490501, EPI_ISL_1490517 to 1490519, EPI_ISL_1490523, EPI_ISL_1490556, EPI_ISL_1490570 to 1490571, EPI_ISL_1490580, EPI_ISL_1490587, EPI_ISL_1490595, EPI_ISL_1490612, EPI_ISL_1490614, EPI_ISL_1547555 to 1547560, EPI_ISL_1547562, EPI_ISL_1547565, EPI_ISL_1547570, EPI_ISL_1547573, EPI_ISL_1547593 to 1547595, EPI_ISL_1547634, EPI_ISL_1547646 to 1547648, EPI_ISL_1547665 to 1547666, EPI_ISL_1547668, EPI_ISL_1547671, EPI_ISL_1547673, EPI_ISL_1547675 to 1547676, EPI_ISL_1547679 to 1547682, EPI_ISL_1547684 to 1547687, EPI_ISL_1547689, EPI_ISL_1547692, EPI_ISL_1547705			
see above	Ostfold Hospital Trust - Kalnes, Centre for Laboratory Medicine, Section for gene technology and infection serology	Norwegian Institute of Public Health, Department of Virology	Atiya R Ali; Debech Nadia; Engebretsen Serina Beate; Garcia Llorente Ignacio; Hilde Elshaug; Hilde Synnøve Vollan; Hilde Vollan; Jon Bråte; Kamilla Heddeland Instefjord; Karoline Bragstad; Kathrine Stene-Johansen; Marie Paulsen Madsen; Olav Hungnes; Pedersen Benedikte Nevjen; Rasmus Riis Kopperud
EPI_ISL_420148	Ostfold Hospital Trust -Kalnes Centre for Laboratory Medicine Section for gene technology and infection serology	Norwegian Institute of Public Health, Department of Virology	Hilde Elshaug; Kamilla Heddeland Instefjord; Karoline Bragstad; Kathrine Stene-Johansen; Olav Hungnes
EPI_ISL_1534277, EPI_ISL_1534293, EPI_ISL_1534344, EPI_ISL_1534378, EPI_ISL_1534456	Oudtshoorn Hospital wc OUD	NHLS/UCT	Arash Iranzadeh; Bruna Galvao; Carolyn Williamson; Deelan Doolabh; Diana Hardie; Emmanuel SJ; Innocent Mudau; Kruger Marais; Lynn Tyers; Marvin Hsiao; Stephen Korsman; Tegally H; de Oliveira T
EPI_ISL_1416966 to 1416991, EPI_ISL_1443891 to 1443894	Outre mer	National Reference Center for Viruses of Respiratory Infections, Institut Pasteur, Paris	Angela Brisebarre; Anne Holstein; Camille Capel; Christophe Malabat; Claire Felloni; Corinne Maufrais; CéLine Ramanantsoa; Esther Gyde; Etienne Simon-Lorière; Frédéric Lemoine; GréGoire Potiron; JéRome Guinard; Louise Lefrançois; Marion Barbet; Maud Vanpeene; Méline Bizard; StéPhanie Guyomard-Rabenirina; Sylvie Behillil; Sylvie van der Werf; Vincent Enouf
EPI_ISL_448449 to 448547, EPI_ISL_448549, EPI_ISL_448551, EPI_ISL_448553, EPI_ISL_448555, EPI_ISL_448557, EPI_ISL_448559, EPI_ISL_448561, EPI_ISL_448564 to 448565, EPI_ISL_448774 to 448825, EPI_ISL_478729, EPI_ISL_478731 to 478732, EPI_ISL_478734 to 478736, EPI_ISL_478738 to 478744, EPI_ISL_478746 to 478749, EPI_ISL_478751 to 478754, EPI_ISL_478756 to 478759, EPI_ISL_478761, EPI_ISL_478763 to 478764, EPI_ISL_478766 to 478777, EPI_ISL_478779 to 478780, EPI_ISL_478782 to 478787, EPI_ISL_478789 to 478792, EPI_ISL_478794 to 478795, EPI_ISL_478797 to 478799, EPI_ISL_478801 to 478803, EPI_ISL_478806 to 478816, EPI_ISL_478819 to 478820, EPI_ISL_478822 to 478824, EPI_ISL_478826 to 478830, EPI_ISL_478832, EPI_ISL_478834 to 478845, EPI_ISL_478847 to 478848, EPI_ISL_478850 to 478852, EPI_ISL_478854 to 478861, EPI_ISL_478863 to 478882, EPI_ISL_478884, EPI_ISL_478886 to 478891, EPI_ISL_478893 to 478895, EPI_ISL_478897 to 478898, EPI_ISL_478900 to 478901, EPI_ISL_478903 to 478909, EPI_ISL_478911 to 478912, EPI_ISL_478914 to 478920, EPI_ISL_478922 to 478924, EPI_ISL_478926 to 478933, EPI_ISL_478935 to 478940, EPI_ISL_478942 to 478943, EPI_ISL_478945 to 478946, EPI_ISL_478948 to 478954, EPI_ISL_478956 to 478957, EPI_ISL_478959 to 478960, EPI_ISL_478962 to 478964, EPI_ISL_478967 to 478969, EPI_ISL_478971 to 478972, EPI_ISL_478974 to 478977, EPI_ISL_478979 to 478980, EPI_ISL_478982 to 478984, EPI_ISL_478986 to 478987, EPI_ISL_478989, EPI_ISL_478991 to 478992, EPI_ISL_478994 to 478995, EPI_ISL_478997 to 478999, EPI_ISL_479001, EPI_ISL_479003 to 479004, EPI_ISL_479006, EPI_ISL_479008, EPI_ISL_479010, EPI_ISL_479012, EPI_ISL_479014, EPI_ISL_479017, EPI_ISL_479020, EPI_ISL_479022, EPI_ISL_479026 to 479031, EPI_ISL_479033 to 479038, EPI_ISL_479041 to 479054, EPI_ISL_479056 to 479062, EPI_ISL_479064, EPI_ISL_479066 to 479069, EPI_ISL_479071 to 479076, EPI_ISL_479078 to 479080, EPI_ISL_479082 to 479087, EPI_ISL_479089 to 479091, EPI_ISL_479093, EPI_ISL_479095 to 479097, EPI_ISL_479099 to 479104, EPI_ISL_479108 to 479114, EPI_ISL_479116 to 479154, EPI_ISL_479156 to 479167, EPI_ISL_479169 to 479174, EPI_ISL_534763 to 535023, EPI_ISL_549329 to 549333, EPI_ISL_549335, EPI_ISL_549337 to 549340, EPI_ISL_549342, EPI_ISL_549345 to 549348, EPI_ISL_549396 to 549459, EPI_ISL_549523 to 549546, EPI_ISL_559753 to 559897, EPI_ISL_559984 to 560053, EPI_ISL_560069 to 560082, EPI_ISL_560305 to 560306, EPI_ISL_572714, EPI_ISL_572723 to 572724, EPI_ISL_572726 to 572728, EPI_ISL_572846, EPI_ISL_572849 to 572850, EPI_ISL_572857 to 572861, EPI_ISL_572869 to 572870, EPI_ISL_572873, EPI_ISL_572891 to 572897, EPI_ISL_572902 to 572903, EPI_ISL_572917 to 572926, EPI_ISL_572933 to 572937, EPI_ISL_572947, EPI_ISL_572952 to 572953, EPI_ISL_572958, EPI_ISL_572960, EPI_ISL_572962 to 573084, EPI_ISL_573154 to 573235, EPI_ISL_573566 to 573651, EPI_ISL_573759, EPI_ISL_573811 to 573855, EPI_ISL_573866 to 573869, EPI_ISL_574257, EPI_ISL_576569 to 576876, EPI_ISL_576920 to 576960, EPI_ISL_577068 to 577095, EPI_ISL_577383 to 577384, EPI_ISL_577401 to 577416, EPI_ISL_584680, EPI_ISL_594467 to 594483, EPI_ISL_594617 to 594644, EPI_ISL_594889 to 594922, EPI_ISL_595326 to 595328, EPI_ISL_595617 to 595798, EPI_ISL_1387213, EPI_ISL_1387216 to 1387219, EPI_ISL_1387221, EPI_ISL_1387225 to 1387227, EPI_ISL_1387229 to 1387236, EPI_ISL_1475543, EPI_ISL_1475547 to 1475550, EPI_ISL_1475552, EPI_ISL_1475555, EPI_ISL_1475557 to 1475560, EPI_ISL_1475562 to 1475563, EPI_ISL_1475567 to 1475568, EPI_ISL_1475570, EPI_ISL_1475572, EPI_ISL_1475574, EPI_ISL_1475576 to 1475577, EPI_ISL_1475580 to 1475581, EPI_ISL_1475583 to 1475587, EPI_ISL_1475589 to 1475591, EPI_ISL_1475593 to 1475595, EPI_ISL_1475601 to 1475607, EPI_ISL_1475609, EPI_ISL_1475611 to 1475613, EPI_ISL_1475615 to 1475621, EPI_ISL_1475623 to 1475633, EPI_ISL_1475635, EPI_ISL_1475638 to 1475642, EPI_ISL_1475644, EPI_ISL_1475647 to 1475649, EPI_ISL_1475651 to 1475658, EPI_ISL_1475661 to 1475662, EPI_ISL_1475664 to 1475679, EPI_ISL_1475683 to 1475684, EPI_ISL_1475686, EPI_ISL_1475688 to 1475689, EPI_ISL_1475692 to 1475694, EPI_ISL_1475697 to 1475701, EPI_ISL_1475703 to 1475704, EPI_ISL_1475706, EPI_ISL_1475708 to 1475712, EPI_ISL_1475716, EPI_ISL_1475718 to 1475721, EPI_ISL_1475723 to 1475731, EPI_ISL_1475733 to 1475734, EPI_ISL_1475737 to 1475738, EPI_ISL_1475740 to 1475744, EPI_ISL_1475746 to 1475747, EPI_ISL_1475749 to 1475750			
see above	Oxford Viromics, NDM, University of Oxford; Oxford University Hospitals; Basingstoke and North Hampshire Hospital	COVID-19 Genomics UK (COG-UK) Consortium	Alex Mobbs; Amy Trebes; Anita Justice; Catrin Moore; Christophe Fraser; David Bonsall; David Buck; Emma Wise; George Macintyre; Jessica Lynch; John Todd; Mariateresa de Cesare; Matilde Mori; Monique Andersson; Nathan Moore; Nick Cortes; Robert Shaw; Stephen Kidd; Tanya Golubchik; Timothy Peto
EPI_ISL_528425 to 528426	P. D. Hinduja Hospital and Medical Research Centre	Institute of Genomics and Integrative Biology - Council of Scientific and Industrial Research	Akshay Kanakan; Amol Borse; Janani Srinivasa Vasudevan; Jasmina Savak; Jayanthi Shastri; Mahesh Sangar; Manish Pathak; Mayuresh Vishwanathan; Nirjhar Chatterjee; Rajesh Pandey; Ranjeet Maurya; Sachee Agrawal; Shruthi Sachidanandan; Shrutika Pophale; Shweta Kawankar; Subrat Thanapati;

			Suresh Poojari; Swapneil Parikh; Utkarsha Yelve; Vasil Nachan; Vivekanand A
EPI_ISL_1516276 to 1516295	PA Department of Health, Bureau of Laboratories	Centers for Disease Control and Prevention Division of Viral Diseases, Pathogen Discovery	Alison Laufer Halpin; Ben L. Rambo-Martin; Clinton R. Paden; Dakota Howard; Darlene Wagner; Dave Wentworth; Dhwani Batra; Jasmine Padilla; Justin Lee; Katie Dillon; Krista Queen; Kristen Knipe; Kristine Lacek; Mark Burroughs; Matthew Schmerer; Mili Sheth; Peter Cook; Sam Shepard; Sarah Nobles; Shoshona Le; Suxiang Tong; Vivien Dugan; Yvette Unoarumhi
EPI_ISL_424881 to 424886, EPI_ISL_426432 to 426434, EPI_ISL_447844, EPI_ISL_450802 to 450803, EPI_ISL_576170 to 576171	PA Department of Health, Bureau of Laboratories	Pathogen Discovery, Respiratory Viruses Branch, Division of Viral Diseases, Centers for Disease Control and Prevention	Alison S. Laufer Halpin; Anna Montmayeur; Anna Uehara; Bettina Bankamp; Brian Lynch; Christopher A. Elkins; Clinton R. Paden; Haibin Wang; Jasmine Padilla; Jing Zhang; Justin Lee; Krista Queen; Mary S. Keckler; Peter Cook; Rachel Marine; Suxiang Tong; Yan Li; Ying Tao; Zachary Weiner
EPI_ISL_458491, EPI_ISL_458786, EPI_ISL_458800, EPI_ISL_458872, EPI_ISL_459039, EPI_ISL_482028, EPI_ISL_487569, EPI_ISL_487593, EPI_ISL_487609, EPI_ISL_488342, EPI_ISL_488409, EPI_ISL_488411, EPI_ISL_492514, EPI_ISL_492594, EPI_ISL_492596, EPI_ISL_539895			
see above	PHE South West Regional Laboratory, National Infection Service	Wellcome Sanger Institute for the COVID-19 Genomics UK (COG-UK) Consortium	Barry Vipond; Cordelia Langford; David K. Jackson; Dominic Kwiatkowski; Dr Peter Muir; Ewan Harrison; Hannah Pymont; Ian Johnston; John Sillitoe on behalf of the Wellcome Sanger Institute COVID-19 Surveillance Team; Rich Hopes; Roberto Amato; Sonia Goncalves; Stephanie Hutchings; and Alex Alderton
EPI_ISL_439953, EPI_ISL_439957, EPI_ISL_439959 to 439960, EPI_ISL_439962, EPI_ISL_439964 to 439967, EPI_ISL_439969, EPI_ISL_439971 to 439974, EPI_ISL_439976, EPI_ISL_439978 to 439979, EPI_ISL_439981 to 439986, EPI_ISL_439988, EPI_ISL_439992, EPI_ISL_439998, EPI_ISL_440000 to 440002, EPI_ISL_440005 to 440006, EPI_ISL_440008, EPI_ISL_440013, EPI_ISL_440016, EPI_ISL_440018, EPI_ISL_440020 to 440023, EPI_ISL_440026 to 440029, EPI_ISL_440031 to 440033, EPI_ISL_440035 to 440039, EPI_ISL_440042 to 440043, EPI_ISL_440050 to 440052, EPI_ISL_440054, EPI_ISL_440056, EPI_ISL_440058, EPI_ISL_440063, EPI_ISL_440065, EPI_ISL_440068, EPI_ISL_440070 to 440071, EPI_ISL_440081, EPI_ISL_440084, EPI_ISL_440087, EPI_ISL_440091 to 440095, EPI_ISL_440098, EPI_ISL_440100 to 440103, EPI_ISL_440105 to 440106, EPI_ISL_440113 to 440116, EPI_ISL_440118 to 440119, EPI_ISL_440123 to 440124, EPI_ISL_440126 to 440128, EPI_ISL_440130 to 440131, EPI_ISL_440134 to 440137, EPI_ISL_440139 to 440142, EPI_ISL_440145, EPI_ISL_440148 to 440149, EPI_ISL_440151, EPI_ISL_440153 to 440154, EPI_ISL_440156 to 440158, EPI_ISL_440160, EPI_ISL_440162, EPI_ISL_440165, EPI_ISL_440167 to 440168, EPI_ISL_440172, EPI_ISL_440174 to 440175, EPI_ISL_440178, EPI_ISL_440187, EPI_ISL_440189 to 440192, EPI_ISL_440194, EPI_ISL_440196, EPI_ISL_440198 to 440201, EPI_ISL_440204 to 440206, EPI_ISL_440210, EPI_ISL_440212, EPI_ISL_440214 to 440216, EPI_ISL_440218 to 440222, EPI_ISL_440224 to 440225, EPI_ISL_440227 to 440229, EPI_ISL_440232 to 440233, EPI_ISL_440235 to 440236, EPI_ISL_440238, EPI_ISL_440241 to 440243, EPI_ISL_440245, EPI_ISL_440248 to 440253, EPI_ISL_440255 to 440256, EPI_ISL_440262, EPI_ISL_440266, EPI_ISL_440269, EPI_ISL_440272 to 440277, EPI_ISL_440286, EPI_ISL_440291 to 440292, EPI_ISL_440294, EPI_ISL_440298 to 440299, EPI_ISL_440623 to 440809, EPI_ISL_442624 to 442810, EPI_ISL_443686 to 444021, EPI_ISL_458299 to 458490, EPI_ISL_458492 to 458515, EPI_ISL_458719 to 458785, EPI_ISL_458787 to 458799, EPI_ISL_458801 to 458871, EPI_ISL_458873 to 459038, EPI_ISL_459040 to 459094, EPI_ISL_459725 to 459727, EPI_ISL_467693 to 467719, EPI_ISL_469345 to 469804, EPI_ISL_469806 to 469843, EPI_ISL_469914, EPI_ISL_469916 to 469917, EPI_ISL_470356 to 470357, EPI_ISL_470529 to 470530, EPI_ISL_481763 to 482027, EPI_ISL_482029 to 482031, EPI_ISL_487524, EPI_ISL_487526 to 487528, EPI_ISL_487530 to 487533, EPI_ISL_487535 to 487536, EPI_ISL_487538 to 487539, EPI_ISL_487541 to 487545, EPI_ISL_487547 to 487549, EPI_ISL_487551 to 487555, EPI_ISL_487560 to 487561, EPI_ISL_487563, EPI_ISL_487565 to 487567, EPI_ISL_487570 to 487571, EPI_ISL_487573 to 487577, EPI_ISL_487579 to 487580, EPI_ISL_487583, EPI_ISL_487586 to 487588, EPI_ISL_487590 to 487592, EPI_ISL_487594, EPI_ISL_487596, EPI_ISL_487598 to 487599, EPI_ISL_487601 to 487605, EPI_ISL_487608, EPI_ISL_487610 to 487613, EPI_ISL_487617 to 487620, EPI_ISL_487622 to 487624, EPI_ISL_487629, EPI_ISL_487634, EPI_ISL_488187 to 488341, EPI_ISL_488343 to 488408, EPI_ISL_488410, EPI_ISL_488412 to 488455, EPI_ISL_488877 to 488878, EPI_ISL_489120, EPI_ISL_492185 to 492188, EPI_ISL_492195, EPI_ISL_492197, EPI_ISL_492202 to 492203, EPI_ISL_492211 to 492449, EPI_ISL_492505 to 492513, EPI_ISL_492515 to 492593, EPI_ISL_492595, EPI_ISL_492597 to 492745, EPI_ISL_492764 to 492835, EPI_ISL_495097 to 495121, EPI_ISL_501555 to 501556, EPI_ISL_501558 to 501561, EPI_ISL_501563 to 501599, EPI_ISL_501601, EPI_ISL_501603 to 501612, EPI_ISL_511955 to 511958, EPI_ISL_511960 to 511969, EPI_ISL_511971 to 511981, EPI_ISL_513584, EPI_ISL_524486 to 524492, EPI_ISL_539894, EPI_ISL_539896			
see above	PHE South West Regional Laboratory, National Infection Service	Wellcome Sanger Institute for the COVID-19 Genomics UK (COG-UK) consortium	Alex Alderton; Barry Vipond; Cordelia Langford; David K. Jackson; Dominic Kwiatkowski; Dr Peter Muir; Ewan Harrison; Hannah Pymont; Ian Johnston; John Sillitoe on behalf of the Wellcome Sanger Institute COVID-19 Surveillance Team; John Sillitoe on behalf of the Wellcome Sanger Institute COVID-19 Surveillance Team (http://www.sanger.ac.uk/covid-team); Rich Hopes; Roberto Amato; Sonia Goncalves; Stephanie Hutchings; and Alex Alderton
EPI_ISL_1396519 to 1396521, EPI_ISL_1396524 to 1396527, EPI_ISL_1396530, EPI_ISL_1404914, EPI_ISL_1404920, EPI_ISL_1424004 to 1424006, EPI_ISL_1465879 to 1465886, EPI_ISL_1465889 to 1465891, EPI_ISL_1465893 to 1465896, EPI_ISL_1516885 to 1516887			
see above	PHV-FSS	PHV-FSS	Son Nguyen
EPI_ISL_1469258	PKM TEMPURAN KARAWANG	National Institute of Health Research and Development	Agustiningsih; Arie Ardiansyah Nugraha; Fauzul Muna; Hana Apsari Pawestri; Hartanti Dian Ikawati; Herna; Holy Arif Wibowo; Irene Lorinda Indalao; Kartika Dewi Puspa; Kindi Adam; Krisna Nur Andriana Pangesti; Natalie Laurencia Kipuw; Nelly Puspandari; Ni Ketut Susilarini; Nike Susanti; Nurika Hariastuti; Reni Herman; Ririn Ramadhany; Subangkit; Tati Febriyanti; Triyani Soekarso; Ully Alfi Nikmah; Vivi Setiawaty.; Yuni Rukminiati
EPI_ISL_1445090	POLICLINICA COVID 19 ITAPETININGA	Instituto Butantan / Mendelics	Antonio Jorge Martins; Bibiana Santos; Claudia Renata dos Santos Barros; David Schlesinger; Debora Botequio Moretti; Dimas Tadeu Covas; Elaine Cristina Marqueze; Elaine Vieira dos Santos; Erika Freitas; Evandra Strazza Rodrigues; Flavia Aburjaile; José Salvatore Leister Patané; João Paulo Kitajima; Luiz Carlos Junior de Alcantara; Maria Carolina Elias; Marta Giovanetti; Rafael dos Santos Bezerra; Raul Machado Neto; Ricardo Haddad; Rodrigo Tocantins Calado.; Sandra Coccuzzo Sampaio; Simone Kashima; Svetoslav Nanev Slavov; Vagner Fonseca; Vincent Louis Viala
EPI_ISL_445252	PONTIFICIA U. CATOLICA FAC. MEDICINA	Instituto de Salud Publica de Chile	Alejandra Acevedo; Andrés E Castillo; Bárbara Parra; Carolina Tambley; Gabriel Leal; Jaime Lagos; Jorge Fernandez; Loredana Arata; Patricia Bustos; Paz Tapia; Rodrigo Fasce; Winston Andrade
EPI_ISL_445356	PONTIFICIA U. CATOLICA SERV. LABORATORIO	Instituto de Salud Publica de Chile	Alejandra Acevedo; Andrés E Castillo; Bárbara Parra; Carolina Tambley; Gabriel Leal; Jaime Lagos; Jorge Fernandez; Loredana Arata; Patricia Bustos; Paz Tapia; Rodrigo Fasce; Winston Andrade
EPI_ISL_445265	PONTIFICIA UNIVERSIDAD CATOLICA DE CHILE	Instituto de Salud Publica de Chile	Alejandra Acevedo; Andrés E Castillo; Bárbara Parra; Carolina Tambley; Gabriel Leal; Jaime Lagos; Jorge Fernandez; Loredana Arata; Patricia Bustos; Paz Tapia; Rodrigo Fasce; Winston Andrade
EPI_ISL_1445224	PPA FENELON GUEDES PEREIRA	Instituto Butantan / Mendelics	Antonio Jorge Martins; Bibiana Santos; Claudia Renata dos Santos Barros; David Schlesinger; Debora Botequio Moretti; Dimas Tadeu Covas; Elaine Cristina Marqueze; Elaine Vieira dos Santos; Erika Freitas; Evandra Strazza Rodrigues; Flavia Aburjaile; José Salvatore Leister Patané; João Paulo Kitajima; Luiz Carlos Junior de Alcantara; Maria Carolina Elias; Marta Giovanetti; Rafael dos Santos Bezerra; Raul Machado Neto; Ricardo Haddad; Rodrigo Tocantins Calado.; Sandra Coccuzzo Sampaio; Simone Kashima; Svetoslav Nanev Slavov; Vagner Fonseca; Vincent Louis Viala
EPI_ISL_1445207	PPA MARCIO VALDEVINO BATISTA	Instituto Butantan / Mendelics	Antonio Jorge Martins; Bibiana Santos; Claudia Renata dos Santos Barros; David Schlesinger; Debora Botequio Moretti; Dimas Tadeu Covas; Elaine Cristina Marqueze; Elaine Vieira dos Santos; Erika Freitas; Evandra Strazza Rodrigues; Flavia Aburjaile; José Salvatore Leister Patané; João Paulo Kitajima; Luiz Carlos Junior de Alcantara; Maria Carolina Elias; Marta Giovanetti; Rafael dos Santos Bezerra; Raul Machado Neto; Ricardo Haddad; Rodrigo Tocantins Calado.; Sandra Coccuzzo Sampaio; Simone Kashima; Svetoslav Nanev Slavov; Vagner Fonseca; Vincent Louis Viala
EPI_ISL_447845	PR - Biological and Chemical Emergencies Lab Office of Public Health Preparedness and Response	Pathogen Discovery, Respiratory Viruses Branch, Division of Viral Diseases, Centers for Disease Control and Prevention	Alison S. Laufer Halpin; Anna Uehara; Christopher A. Elkins; Clinton R. Paden; Haibin Wang; Jasmine Padilla; Jing Zhang; Justin Lee; Krista Queen; Mary S. Keckler; Suxiang Tong; Yan Li; Ying Tao
EPI_ISL_1516262 to 1516263, EPI_ISL_1528460 to 1528461	PR Public Health Lab	Centers for Disease Control and Prevention Division of Viral Diseases, Pathogen Discovery	Alison Laufer Halpin; Ben L. Rambo-Martin; Clinton R. Paden; Dakota Howard; Darlene Wagner; Dave Wentworth; Dhwani Batra; Jasmine Padilla; Justin Lee; Katie Dillon; Krista Queen; Kristen Knipe; Kristine Lacek; Mark Burroughs; Matthew Schmerer; Mili Sheth; Peter Cook; Sam Shepard; Sarah Nobles; Shoshona Le; Suxiang Tong; Vivien Dugan; Yvette Unoarumhi
EPI_ISL_1483748	PRESCRIPTIONS EXTERIEURES	CHU Clermont-Ferrand, service de virologie	Bisseux Maxime; Combes Patricia; Henquell Cécile; Mirand Audrey
EPI_ISL_1403500 to 1403501, EPI_ISL_1403585 to 1403591, EPI_ISL_1403593 to 1403600, EPI_ISL_1420831, EPI_ISL_1502523, EPI_ISL_1502526 to 1502527	PRESIDIO TERRITORIALE CASOLI CASOLI(CHIETI)	Istituto Zooprofilattico Sperimentale dell'Abruzzo e Molise "G. Caporale"	Ancora M; Calistri P; Cammà C; Caporale M; Curini V; Delli Compagni E; Di Domenico M; Di Lollo Valeria; Di Pasquale A; Lorusso A; Mangone I; Marcacci M; Puglia I; Rinaldi A; Savini G; Scialabba S
EPI_ISL_1445130 to 1445132	PRONTO ATENDIMENTO DA PONTE DE SAO JOAO	Instituto Butantan / Mendelics	Antonio Jorge Martins; Bibiana Santos; Claudia Renata dos Santos Barros; David Schlesinger; Debora Botequio Moretti; Dimas Tadeu Covas; Elaine Cristina Marqueze; Elaine Vieira dos Santos; Erika Freitas; Evandra Strazza Rodrigues; Flavia Aburjaile; José Salvatore Leister Patané; João Paulo Kitajima; Luiz Carlos Junior de Alcantara; Maria Carolina Elias; Marta Giovanetti; Rafael dos Santos Bezerra; Raul Machado Neto; Ricardo Haddad; Rodrigo Tocantins Calado.; Sandra Coccuzzo Sampaio; Simone Kashima; Svetoslav Nanev Slavov; Vagner Fonseca; Vincent Louis Viala
EPI_ISL_1445185, EPI_ISL_1445187, EPI_ISL_1445193 to 1445194	PRONTO ATENDIMENTO SAO JOSE	Instituto Butantan / Mendelics	Antonio Jorge Martins; Bibiana Santos; Claudia Renata dos Santos Barros; David Schlesinger; Debora Botequio Moretti; Dimas Tadeu Covas; Elaine Cristina Marqueze; Elaine Vieira dos Santos; Erika Freitas; Evandra Strazza Rodrigues; Flavia Aburjaile; José Salvatore Leister Patané; João Paulo Kitajima; Luiz Carlos Junior de Alcantara; Maria Carolina Elias; Marta Giovanetti; Rafael dos Santos Bezerra; Raul Machado Neto; Ricardo Haddad; Rodrigo Tocantins Calado.; Sandra Coccuzzo Sampaio; Simone Kashima; Svetoslav Nanev Slavov; Vagner Fonseca; Vincent Louis Viala
EPI_ISL_1445096 to 1445100, EPI_ISL_1445102 to 1445108, EPI_ISL_1445111 to 1445112, EPI_ISL_1445186, EPI_ISL_1445188 to 1445192	PRONTO ATENDIMENTO VILA PADRE ANCHIETA	Instituto Butantan / Mendelics	Antonio Jorge Martins; Bibiana Santos; Claudia Renata dos Santos Barros; David Schlesinger; Debora Botequio Moretti; Dimas Tadeu Covas; Elaine Cristina Marqueze; Elaine Vieira dos Santos; Erika Freitas; Evandra Strazza Rodrigues; Flavia Aburjaile; José Salvatore Leister Patané; João Paulo Kitajima; Luiz Carlos Junior de Alcantara; Maria Carolina Elias; Marta Giovanetti; Rafael dos Santos Bezerra; Raul Machado Neto; Ricardo Haddad; Rodrigo Tocantins Calado.; Sandra Coccuzzo Sampaio; Simone Kashima; Svetoslav Nanev Slavov; Vagner Fonseca; Vincent Louis Viala
EPI_ISL_1445225	PRONTO SOCORRO DR ANTONIO FLAVIO FRANCA	Instituto Butantan / Mendelics	Antonio Jorge Martins; Bibiana Santos; Claudia Renata dos Santos Barros; David Schlesinger; Debora Botequio Moretti; Dimas Tadeu Covas; Elaine Cristina Marqueze; Elaine Vieira dos Santos; Erika Freitas; Evandra Strazza Rodrigues; Flavia Aburjaile; José Salvatore Leister Patané; João Paulo Kitajima; Luiz Carlos Junior de Alcantara; Maria Carolina Elias; Marta Giovanetti; Rafael dos Santos Bezerra; Raul Machado Neto; Ricardo Haddad; Rodrigo Tocantins Calado.; Sandra Coccuzzo Sampaio; Simone Kashima; Svetoslav Nanev Slavov; Vagner Fonseca; Vincent Louis Viala

EPI_ISL_1445174	PRONTO SOCORRO MUNICIPAL DE TATUI	Instituto Butantan / Mendelics	Antonio Jorge Martins; Bibiana Santos; Claudia Renata dos Santos Barros; David Schlesinger; Debora Botequio Moretti; Dimas Tadeu Covas; Elaine Cristina Marqueze; Elaine Vieira dos Santos; Erika Freitas; Evandra Strazza Rodrigues; Flavia Aburjaile; José Salvatore Leister Patané; João Paulo Kitajima; Luiz Carlos Junior de Alcantara; Maria Carolina Elias; Marta Giovanetti; Rafael dos Santos Bezerra; Raul Machado Neto; Ricardo Haddad; Rodrigo Tocantins Calado.; Sandra Coccuzzo Sampaio; Simone Kashima; Svetoslav Nanev Slavov; Vagner Fonseca; Vincent Louis Viala
EPI_ISL_534322	PS Mun Julio Tupy	Instituto Adolfo Lutz, Interdiciplinary Procedures Center, Strategic Laboratory	Claudia Regina Gonçalves; Claudio Tavares Sacchi; Erica Valessa Ramos Gomes
EPI_ISL_1533706	PS Mun Santana Lauro Ribas Braga	Instituto Adolfo Lutz, Interdiciplinary Procedures Center, Strategic Laboratory	Caio Vinicius Dias Lopes; Claudia Regina Gonçalves; Claudio Tavares Sacchi; Erica Valessa Ramos Gomes; Karoline Rodrigues Campos; Leonardo Jose Tadeu de Araujo
EPI_ISL_523973	PS Municipal Dona Maria Antonieta Ferreira de Barros	Instituto Adolfo Lutz, Interdiciplinary Procedures Center, Strategic Laboratory	Claudia Regina Gonçalves; Claudio Tavares Sacchi; Erica Valessa Ramos Gomes
EPI_ISL_547570	PS Municipal Dr Augusto Gomes de Mattos	Instituto Adolfo Lutz, Interdiciplinary Procedures Center, Strategic Laboratory	Claudia Regina Gonçalves; Claudio Tavares Sacchi; Erica Valessa Ramos Gomes; Karoline Rodrigues Campos
EPI_ISL_515523 to 515524, EPI_ISL_515566, EPI_ISL_524466, EPI_ISL_527866	PS Municipal Dr Lauro Ribas Braga	Instituto Adolfo Lutz, Interdiciplinary Procedures Center, Strategic Laboratory	01246-1301; 355 - Brazil; Av. Dr. Arnaldo; Cerqueira Cesar; Claudia Regina Gonçalves; Claudio Tavares Sacchi; Erica Valessa Ramos Gomes; São Paulo - SP
EPI_ISL_524465	PS Municipal Dr. Caetano Virgílio Neto	Instituto Adolfo Lutz, Interdiciplinary Procedures Center, Strategic Laboratory	Claudia Regina Gonçalves; Claudio Tavares Sacchi; Erica Valessa Ramos Gomes
EPI_ISL_534321	PS e Maternidade Nair Fonseca Leitao Arantes	Instituto Adolfo Lutz, Interdiciplinary Procedures Center, Strategic Laboratory	Claudia Regina Gonçalves; Claudio Tavares Sacchi; Erica Valessa Ramos Gomes
EPI_ISL_1534367, EPI_ISL_1534425, EPI_ISL_1534432, EPI_ISL_1534434, EPI_ISL_1534445, EPI_ISL_1534458	Pacaltsdorp Clinic wc PAC	NHLS/UCT	Arash Iranzadeh; Bruna Galvao; Carolyn Williamson; Deelan Doolabh; Diana Hardie; Emmanuel SJ; Innocent Mudau; Kruger Marais; Lynn Tyers; Marvin Hsiao; Stephen Korsman; Tegally H; de Oliveira T
EPI_ISL_596500 to 596538, EPI_ISL_596542 to 596565, EPI_ISL_596567 to 596568	Palestinian Ministry of Health	Molecular Genetics Lab	Damien Richard; Dana Najjar; Francois Balloux; Hisham Darwish; Husam Sallam; Issa Shtayeh; Lucy van Dorp; Mahmoud Ruzayqat; Nouar Qutob; Osama Najjar; Zaidoun Salah
EPI_ISL_1443215, EPI_ISL_1500211, EPI_ISL_1553248	Pana Community Hospital	Illinois Department of Public Health - Springfield Lab	Bryan Sim; Gordon McCall
EPI_ISL_1384885 to 1385783, EPI_ISL_1470741 to 1472076, EPI_ISL_1542074 to 1542917, EPI_ISL_1542919 to 1543920	Pandemic Response Lab - NYC	Pandemic Response Lab, R&D	Cybill del Castillo; Dylan Law; Haiping Hao; Henry Lee; Jon Laurent; Katharine Nelson; Melissa Hopkins; Michael Hammerling; Pradeep Bugga; Shinyoung Clair Kang; Sol Rey; William Ward
EPI_ISL_450785 to 450791, EPI_ISL_455015 to 455016, EPI_ISL_461481 to 461482, EPI_ISL_483829 to 483849	Pandit Deendayal Upadhyay Government Medical College, Rajkot	Gujarat Biotechnology Research Centre	A M Kadri; Afzal Ansari; Amit Kanani; Anjali Rajwar; Ankit Hinsu; Apurvasinh Puvar; Armi Chaudhari; Arti Trivedi; Bhavesh Modi; Bhavya Jindal; Camellia Chakraborty; Chaitanya Joshi; Dinesh Kumar; Gauravi Dhruv; Gaurishankar Shrimali; Harsh Bakshi; Janvi Raval; Komal Patel; Labdhi Pandya; Madhvi Joshi; Maharshi Pandya; Manish Pattani; Monika Gandhi; Neelam Nathani; Neha Rajpara; Nidhi Patel; Nikha Trivedi; Nitin Savaliya; Pinal Trivedi; Pooja P Doshi; Prakash Modi; Pritesh Sabara; R D Dixit; Raghawendra Kumar; Ramesh Pandit; Sejul Antala; Sharmistha Majumdar; Siddhant Kumar; Snehal Bagatharia; Tejas Shah; Umang Mishra; Zarna Patel; Zuber Saiyed
EPI_ISL_455605	Panyananthaphikkhu Chonprathan Medical Center	National Institute of Health. Department of medical Sciences, Ministry of Public Health, Thailand	Chittaganpitch; Malinee; Okada; Parnmen; Phuygun; Pilailuk; Siripaporn; Sittiporn; Sunthareeya; Thanadachakul; Thanutsapa; Waicharoen; Warawan; Wongboot
EPI_ISL_434701	Panyanunthaphikkhu Chonprathan Medical Center (PCMC)	National Institute of Health. Department of medical Sciences, Ministry of Public Health, Thailand	Chittaganpitch; Malinee; Okada; Parnmen; Phuygun; Pilailuk; Siripaporn; Sittiporn; Sunthareeya; Thanadachakul; Thanutsapa; Waicharoen; Warawan; Wongboot
EPI_ISL_421502 to 421503	Parc des Dames	National Reference Center for Viruses of Respiratory Infections, Institut Pasteur, Paris	Angela Brisebarre; Etienne Simon-Lorière; Flora Donati; Marion Barbet; Maud Vanpeene; Mélanie Albert; Méline Bizard; Sylvie Behillil; Sylvie van der Werf; Vincent Enouf
EPI_ISL_430845	Pasig City General Hospital	Research Institute for Tropical Medicine	Bautista; Brunker, K.; C.S.; C.T.; D.L.; Demetria; E.S.; F.G.M.; I.A.P.; Manalo; Medado; Mercado; O.J.T.; Onza; Polotan
EPI_ISL_1478907, EPI_ISL_1500217	Passavant Area Hospital	Illinois Department of Public Health - Springfield Lab	Bryan Sim; Gordon McCall
EPI_ISL_448222	Pasteur Insitute Ho Chi Minh City	National Key Laboratory of Gene Technology, Institute of Biotechnology, Vietnam Academy of Science and Technology	Cao Minh Thang; Chu Hoang Ha; Ho Thi Thuong; Hoang Ha; Huynh Thi Kim Loan; Le Tung Lam; Luong Chan Quang; Luu Thuy Tien; Nguyen Hong Trang; Nguyen Thi Ngoc Thao; Nguyen Trung Hieu; Nguyen Vu Thuong; Pham Duy Quang; Phan Trong Lan; Tran Huyen Linh; Truong Nam Hai
EPI_ISL_442523	Pasteur Institute of Iran	Kawsar Human Genetic Research Company	Elmira Khateri; Maraym Firoozi; Maryam Abbasalipour Bashash; Mohammad Ali Khosravi; Razieh Zeinali and Fahimeh Hoseini; Sanaz Mostafavi Jabbari; Sirous Zeinali; Sormeh Pourtavakoli
EPI_ISL_463749	Pasteur Institute of Iran	Rapid Response Team	Afsaneh Zokaei; Ahmad Ghasemi; Ahmad Mahmoudi; Akram Abouie Mehrizi; Ali Maleki; Ali Torabi; Amir Hesam Nemati; Arash Arashkia; Azam Amirian; Azita Eshratkhah mohammadnejad; Elmira Vadaye kheiri; Farideh niknam oskouei; Fatemeh Fotouhi-Chahooki; Hadiseh Shokouhi Targhi; Hajarossadat Ghaderi; Hesam Karimi; Kayhan Azadmanesh; Kazem Baesi; Mahboobeh Rafigh; Mahmood Barati; Mahsa Ghalejoogh; Mahsa Tavakolirad; Mehdi Fazlalipour; Mehdi Rohani; Mehri Habibi; Mina Agharezaei; Mohamad Mahdi Mortazavipour; Mohammad Hassan Pouriayevali; Mohammad Reza Asadi Karam; Mohammad Sadegh Shams Nosrati; Mostafa Salehi-Vaziri; Neda Afzali; Parastoo Yekta Sanati; Saber Esmaeili; Sahar Khakifirouz; Sanam Azad-Mazjiri; Seyed Dawood Mousavi Nasab; Seyedeh Atefe Hosseini; Seyedeh Sahar Bathaeian; Seyedeh Zahra Moravej; Tahereh Mohammadi; Tahmineh Jalali; Zabihollah Shoja; Zahra Ahmadi; Zahra Fereydouni; Zahra Nejatipour
EPI_ISL_456163, EPI_ISL_456171, EPI_ISL_456194 to 456195, EPI_ISL_456215, EPI_ISL_456221 to 456224, EPI_ISL_456301, EPI_ISL_582018 to 582021			
see above	PathLab Bay of Plenty	Institute of Environmental Science and Research (ESR)	Anja Werno; Antje van der Linden; Arlo Upton; Chris Mansell; David Hammer; Dragana Drinkovic; Erasmus Smit; Gary McAuliffe; Hana Sofia Andersson; Hermes Perez; James Ussher; Jill Sherwood; Jing Wang; Joep de Ligt; Josh Freeman; Julia Howard; Juliet Elvy; Lauren Jelly; Mary DeAlmeida; Matt Blakiston; Matt Storey; Matthew Rogers; Max Bloomfield; Michael Addidle; Michelle Balm; Muhammad Faisal; Nikki Freed; Olin Silander; Sally Roberts; Sarah Jefferies; Sharmini Muttaiyah; Susan Morpeth; Susan Taylor; Timothy Blackmore; Vani Sathyendran; Veronica Playle; Virginia Hope; Xiaoyun Ren
EPI_ISL_420456, EPI_ISL_470830 to 470875	PathWest Laboratory Medicine WA	PathWest Laboratory Medicine WA	Avram Levy; Chisha Sikazwe; David Smith and David Speers; Jurissa Lang
EPI_ISL_512712 to 512765, EPI_ISL_594166 to 594179, EPI_ISL_596677 to 596888, EPI_ISL_605825 to 605877, EPI_ISL_605879 to 605881, EPI_ISL_1384811 to 1384812, EPI_ISL_1416321 to 1416326, EPI_ISL_1448426 to 1448430, EPI_ISL_1508992 to 1508997			
see above	PathWest Laboratory Medicine WA	PathWest Laboratory Medicine WA Microbial Surveillance Unit	PathWest Laboratory Medicine WA Microbial Surveillance Unit
EPI_ISL_582022 to 582023	Pathlab Lakes	Institute of Environmental Science and Research (ESR)	Anja Werno; Antje van der Linden; Arlo Upton; Chris Mansell; David Hammer; Dragana Drinkovic; Erasmus Smit; Gary McAuliffe; Hana Sofia Andersson; Hermes Perez; James Ussher; Jill Sherwood; Jing Wang; Joep de Ligt; Josh Freeman; Julia Howard; Juliet Elvy; Lauren Jelly; Mary DeAlmeida; Matt Blakiston; Matt Storey; Matthew Rogers; Max Bloomfield; Michael Addidle; Michelle Balm; Muhammad Faisal; Nikki Freed; Olin Silander; Sally Roberts; Sarah Jefferies; Sharmini Muttaiyah; Susan Morpeth; Susan Taylor; Timothy Blackmore; Vani Sathyendran; Veronica Playle; Virginia Hope; Xiaoyun Ren
EPI_ISL_1520168	Pathnostics Laboratory	Illinois Department of Public Health - Chicago Lab	Ira Heimler; Vineet K. Dhiman
EPI_ISL_1483210, EPI_ISL_1483213, EPI_ISL_1483221, EPI_ISL_1483230	Pathogen Discovery, Respiratory Viruses Branch, Division of Viral Diseases, Centers for Disease Control and Prevention	Pathogen Discovery, Respiratory Viruses Branch, Division of Viral Diseases, Centers for Disease Control and Prevention	Anna Kelleher; Anna Uehara; Brian Lynch; Clinton R. Paden; Haibin Wang; Han Jia Justin Ng; Jing Zhang; Krista Queen; Peter Cook; Suxiang Tong; Yan Li; Ying Tao
EPI_ISL_480208, EPI_ISL_591347 to 591470, EPI_ISL_591535 to 591539, EPI_ISL_1425572, EPI_ISL_1425601 to 1425604, EPI_ISL_1425614, EPI_ISL_1425616, EPI_ISL_1425620 to 1425695, EPI_ISL_1425697 to 1425698, EPI_ISL_1425700, EPI_ISL_1425703, EPI_ISL_1425706 to 1425711, EPI_ISL_1425713, EPI_ISL_1425717 to 1425718, EPI_ISL_1425721 to 1425724, EPI_ISL_1425740 to 1425747, EPI_ISL_1425777 to 1425858, EPI_ISL_1425861 to 1425878, EPI_ISL_1425882 to 1425918, EPI_ISL_1425938 to 1425956, EPI_ISL_1425958 to 1425974, EPI_ISL_1425976 to 1426045, EPI_ISL_1426048 to 1426141, EPI_ISL_1426158 to 1426265, EPI_ISL_1426371 to 1426567, EPI_ISL_1426589, EPI_ISL_1426594 to 1426631, EPI_ISL_1426633 to 1426635, EPI_ISL_1426638 to 1426642, EPI_ISL_1426645 to 1426647, EPI_ISL_1426659 to 1426660, EPI_ISL_1426662 to 1426991, EPI_ISL_1427105 to 1427153, EPI_ISL_1427166 to 1427339, EPI_ISL_1427506 to 1427533, EPI_ISL_1427553 to 1427567, EPI_ISL_1427587 to 1427601, EPI_ISL_1427611 to 1427653, EPI_ISL_1427656 to 1427671, EPI_ISL_1427673 to 1427682, EPI_ISL_1427687 to 1427688, EPI_ISL_1427694 to 1427715, EPI_ISL_1427733 to 1427783, EPI_ISL_1427830 to 1428173, EPI_ISL_1428175, EPI_ISL_1428177 to 1428182, EPI_ISL_1428184 to 1428187, EPI_ISL_1428189 to 1428233, EPI_ISL_1428236 to 1428250, EPI_ISL_1428260 to 1428287, EPI_ISL_1428314 to 1428568, EPI_ISL_1428570, EPI_ISL_1428611 to 1428631, EPI_ISL_1428636 to 1428639, EPI_ISL_1428641 to 1428643, EPI_ISL_1428645 to 1429340, EPI_ISL_1429342 to 1429555, EPI_ISL_1429557 to 1429571, EPI_ISL_1429574 to 1429578, EPI_ISL_1429590 to 1429591, EPI_ISL_1429593 to 1429632, EPI_ISL_1429634 to 1429638, EPI_ISL_1429640 to 1429715, EPI_ISL_1429722 to 1429757, EPI_ISL_1429759 to 1429763, EPI_ISL_1429765 to 1429788, EPI_ISL_1429790 to 1429915,			

EPI_ISL_1429917 to 1430056, EPI_ISL_1430058 to 1430156, EPI_ISL_1430158 to 1430551, EPI_ISL_1430555 to 1430560, EPI_ISL_1430576 to 1430577, EPI_ISL_1430672 to 1430680, EPI_ISL_1430683 to 1430684, EPI_ISL_1430686, EPI_ISL_1430699, EPI_ISL_1430701 to 1430704, EPI_ISL_1430715 to 1430720, EPI_ISL_1430722 to 1430733, EPI_ISL_1430735, EPI_ISL_1430738 to 1430739, EPI_ISL_1430741 to 1430748, EPI_ISL_1430751 to 1430754, EPI_ISL_1430773 to 1430823, EPI_ISL_1430829 to 1430848, EPI_ISL_1430855 to 1430867, EPI_ISL_1430875 to 1431020, EPI_ISL_1431031 to 1431128, EPI_ISL_1431171 to 1431389, EPI_ISL_1431444 to 1431478, EPI_ISL_1431480 to 1431520, EPI_ISL_1431524 to 1431576, EPI_ISL_1431579 to 1431610, EPI_ISL_1431612 to 1431614, EPI_ISL_1431616 to 1431641, EPI_ISL_1431709 to 1431718, EPI_ISL_1431720 to 1431723, EPI_ISL_1431725, EPI_ISL_1431727 to 1431729, EPI_ISL_1431731 to 1431735, EPI_ISL_1431737 to 1431738, EPI_ISL_1431741 to 1431744, EPI_ISL_1431765 to 1431801, EPI_ISL_1431806 to 1431823, EPI_ISL_1431829 to 1432004, EPI_ISL_1432006 to 1432025, EPI_ISL_1432027, EPI_ISL_1432085, EPI_ISL_1432087 to 1432088, EPI_ISL_1432090, EPI_ISL_1432092, EPI_ISL_1432094, EPI_ISL_1432096, EPI_ISL_1432099, EPI_ISL_1432101 to 1432102, EPI_ISL_1432105 to 1432106, EPI_ISL_1432108, EPI_ISL_1432111 to 1432112, EPI_ISL_1432115, EPI_ISL_1432117, EPI_ISL_1432119, EPI_ISL_1432121, EPI_ISL_1432124, EPI_ISL_1432126 to 1432127, EPI_ISL_1432129, EPI_ISL_1432131, EPI_ISL_1432133, EPI_ISL_1432135, EPI_ISL_1432137, EPI_ISL_1432139, EPI_ISL_1432141 to 1432142, EPI_ISL_1432144, EPI_ISL_1432146, EPI_ISL_1432148, EPI_ISL_1432150, EPI_ISL_1432152, EPI_ISL_1432154, EPI_ISL_1432156, EPI_ISL_1432158, EPI_ISL_1432160, EPI_ISL_1432162, EPI_ISL_1432164 to 1432165, EPI_ISL_1432167, EPI_ISL_1432169, EPI_ISL_1432171 to 1432172, EPI_ISL_1432174, EPI_ISL_1432176, EPI_ISL_1432178, EPI_ISL_1432180, EPI_ISL_1432182, EPI_ISL_1432184 to 1432185, EPI_ISL_1432187, EPI_ISL_1432189, EPI_ISL_1432191, EPI_ISL_1432193, EPI_ISL_1432195, EPI_ISL_1432197 to 1432198, EPI_ISL_1432200, EPI_ISL_1432202, EPI_ISL_1432204, EPI_ISL_1432206, EPI_ISL_1432208, EPI_ISL_1432210, EPI_ISL_1432212, EPI_ISL_1432214, EPI_ISL_1432216, EPI_ISL_1432218, EPI_ISL_1432220, EPI_ISL_1432222, EPI_ISL_1432224, EPI_ISL_1432226, EPI_ISL_1432228, EPI_ISL_1432230, EPI_ISL_1432232 to 1432233, EPI_ISL_1432235, EPI_ISL_1432237 to 1432238, EPI_ISL_1432240, EPI_ISL_1432242 to 1432243, EPI_ISL_1432245, EPI_ISL_1432247, EPI_ISL_1432249, EPI_ISL_1432251, EPI_ISL_1432253 to 1432254, EPI_ISL_1432256, EPI_ISL_1432258, EPI_ISL_1432261 to 1432262, EPI_ISL_1432264, EPI_ISL_1432266, EPI_ISL_1432268, EPI_ISL_1432270, EPI_ISL_1432272 to 1432273, EPI_ISL_1432276 to 1432277, EPI_ISL_1432279, EPI_ISL_1432282 to 1432283, EPI_ISL_1432285, EPI_ISL_1432287, EPI_ISL_1432289, EPI_ISL_1432291, EPI_ISL_1432293, EPI_ISL_1432295, EPI_ISL_1432297, EPI_ISL_1432299, EPI_ISL_1432301, EPI_ISL_1432303 to 1432304, EPI_ISL_1432306, EPI_ISL_1432308, EPI_ISL_1432310, EPI_ISL_1432313 to 1432314, EPI_ISL_1432316, EPI_ISL_1432318, EPI_ISL_1432320, EPI_ISL_1432322, EPI_ISL_1432324, EPI_ISL_1432326 to 1432327, EPI_ISL_1432342, EPI_ISL_1432344, EPI_ISL_1432346, EPI_ISL_1432348, EPI_ISL_1432350, EPI_ISL_1432352, EPI_ISL_1432354, EPI_ISL_1432356 to 1432357, EPI_ISL_1432359, EPI_ISL_1432361, EPI_ISL_1432363 to 1432364, EPI_ISL_1432366, EPI_ISL_1432368, EPI_ISL_1432370, EPI_ISL_1432372, EPI_ISL_1432374, EPI_ISL_1432376 to 1432377, EPI_ISL_1432379, EPI_ISL_1432381, EPI_ISL_1432383, EPI_ISL_1432385, EPI_ISL_1432387, EPI_ISL_1432389, EPI_ISL_1432391, EPI_ISL_1432393 to 1432394, EPI_ISL_1432396, EPI_ISL_1432398, EPI_ISL_1432401, EPI_ISL_1432403 to 1432404, EPI_ISL_1432406, EPI_ISL_1432408, EPI_ISL_1432410, EPI_ISL_1432412, EPI_ISL_1432414, EPI_ISL_1432416, EPI_ISL_1432418, EPI_ISL_1432420, EPI_ISL_1432422, EPI_ISL_1432424 to 1432425, EPI_ISL_1432427, EPI_ISL_1432429, EPI_ISL_1432431, EPI_ISL_1432433, EPI_ISL_1432435, EPI_ISL_1432437, EPI_ISL_1432439, EPI_ISL_1432441 to 1432442, EPI_ISL_1432444, EPI_ISL_1432446, EPI_ISL_1432448, EPI_ISL_1432450, EPI_ISL_1432452, EPI_ISL_1432454, EPI_ISL_1432456, EPI_ISL_1432458 to 1432459, EPI_ISL_1432461, EPI_ISL_1432463, EPI_ISL_1432465, EPI_ISL_1432467, EPI_ISL_1432469, EPI_ISL_1432471 to 1432472, EPI_ISL_1432474, EPI_ISL_1432476, EPI_ISL_1432478, EPI_ISL_1432480, EPI_ISL_1432482 to 1432486, EPI_ISL_1432488, EPI_ISL_1432490, EPI_ISL_1432492 to 1432493, EPI_ISL_1432495, EPI_ISL_1432497, EPI_ISL_1432499, EPI_ISL_1432501 to 1432502, EPI_ISL_1432504, EPI_ISL_1432506 to 1432507, EPI_ISL_1432509, EPI_ISL_1432511, EPI_ISL_1432513, EPI_ISL_1432515, EPI_ISL_1432517 to 1432518, EPI_ISL_1432520, EPI_ISL_1432522, EPI_ISL_1432524, EPI_ISL_1432526 to 1432527, EPI_ISL_1432529, EPI_ISL_1432531, EPI_ISL_1432533, EPI_ISL_1432535 to 1432536, EPI_ISL_1432538, EPI_ISL_1432540, EPI_ISL_1432542, EPI_ISL_1432544, EPI_ISL_1432546, EPI_ISL_1432548, EPI_ISL_1432550, EPI_ISL_1432552 to 1432553, EPI_ISL_1432555, EPI_ISL_1432557, EPI_ISL_1432559, EPI_ISL_1432561, EPI_ISL_1432563, EPI_ISL_1432565, EPI_ISL_1432567 to 1432568, EPI_ISL_1432570, EPI_ISL_1432572, EPI_ISL_1432574, EPI_ISL_1432576, EPI_ISL_1432578, EPI_ISL_1432580, EPI_ISL_1432582, EPI_ISL_1432584 to 1432585, EPI_ISL_1432587, EPI_ISL_1432589, EPI_ISL_1432591, EPI_ISL_1432593, EPI_ISL_1432595, EPI_ISL_1432597 to 1432598, EPI_ISL_1432600, EPI_ISL_1432602, EPI_ISL_1432604, EPI_ISL_1432606, EPI_ISL_1432608, EPI_ISL_1432610, EPI_ISL_1432612, EPI_ISL_1432614 to 1432615, EPI_ISL_1432617, EPI_ISL_1432619, EPI_ISL_1432621, EPI_ISL_1432623, EPI_ISL_1432625, EPI_ISL_1432627, EPI_ISL_1432629, EPI_ISL_1432631 to 1432632, EPI_ISL_1432634, EPI_ISL_1432636, EPI_ISL_1432638, EPI_ISL_1432640, EPI_ISL_1432642, EPI_ISL_1432644, EPI_ISL_1432646, EPI_ISL_1432648 to 1432649, EPI_ISL_1432651, EPI_ISL_1432653, EPI_ISL_1432655, EPI_ISL_1432657, EPI_ISL_1432659 to 1432660, EPI_ISL_1432662, EPI_ISL_1432664, EPI_ISL_1432666, EPI_ISL_1432668, EPI_ISL_1432670 to 1432671, EPI_ISL_1432673, EPI_ISL_1432675, EPI_ISL_1432677, EPI_ISL_1432680 to 1432681, EPI_ISL_1432683, EPI_ISL_1432685, EPI_ISL_1432687, EPI_ISL_1432689, EPI_ISL_1432691, EPI_ISL_1432693, EPI_ISL_1432695, EPI_ISL_1432697, EPI_ISL_1432699, EPI_ISL_1432701, EPI_ISL_1432703 to 1432704, EPI_ISL_1432707, EPI_ISL_1432709 to 1432710, EPI_ISL_1432712, EPI_ISL_1432714, EPI_ISL_1432716, EPI_ISL_1432718 to 1432719, EPI_ISL_1432721, EPI_ISL_1432723, EPI_ISL_1432725, EPI_ISL_1432727 to 1432728, EPI_ISL_1432730, EPI_ISL_1432732, EPI_ISL_1432734, EPI_ISL_1432736, EPI_ISL_1432738 to 1432739, EPI_ISL_1432741, EPI_ISL_1432743, EPI_ISL_1432745, EPI_ISL_1432747, EPI_ISL_1432749 to 1432750, EPI_ISL_1432752, EPI_ISL_1432754, EPI_ISL_1432756, EPI_ISL_1432758 to 1432759, EPI_ISL_1432761, EPI_ISL_1432763, EPI_ISL_1432765, EPI_ISL_1432767, EPI_ISL_1432769, EPI_ISL_1432771, EPI_ISL_1432773, EPI_ISL_1432775 to 1432776, EPI_ISL_1432778, EPI_ISL_1432780, EPI_ISL_1432782, EPI_ISL_1432784, EPI_ISL_1432786, EPI_ISL_1432788 to 1432789, EPI_ISL_1432791, EPI_ISL_1432793, EPI_ISL_1432795, EPI_ISL_1432797 to 1432798, EPI_ISL_1432801 to 1432802, EPI_ISL_1432804, EPI_ISL_1432806, EPI_ISL_1432808, EPI_ISL_1432810, EPI_ISL_1432812 to 1432813, EPI_ISL_1432815, EPI_ISL_1432817, EPI_ISL_1432819, EPI_ISL_1432821, EPI_ISL_1432823, EPI_ISL_1432825 to 1432826, EPI_ISL_1432828, EPI_ISL_1432830, EPI_ISL_1432832, EPI_ISL_1432834 to 1432835, EPI_ISL_1432837, EPI_ISL_1432839, EPI_ISL_1432841 to 1432842, EPI_ISL_1432845 to 1432846, EPI_ISL_1432848, EPI_ISL_1432850, EPI_ISL_1432852, EPI_ISL_1432854, EPI_ISL_1432856, EPI_ISL_1432858, EPI_ISL_1432860, EPI_ISL_1432862 to 1432863, EPI_ISL_1432865, EPI_ISL_1432867 to 1432868, EPI_ISL_1432870, EPI_ISL_1432872, EPI_ISL_1432874, EPI_ISL_1432876 to 1432877, EPI_ISL_1432879, EPI_ISL_1432881, EPI_ISL_1432883, EPI_ISL_1432885, EPI_ISL_1432887 to 1432888, EPI_ISL_1432890, EPI_ISL_1432892, EPI_ISL_1432894, EPI_ISL_1432896 to 1432897, EPI_ISL_1432899, EPI_ISL_1432901, EPI_ISL_1432903 to 1432904, EPI_ISL_1432906, EPI_ISL_1432908, EPI_ISL_1432910, EPI_ISL_1432912, EPI_ISL_1432914, EPI_ISL_1432916 to 1432917, EPI_ISL_1432919, EPI_ISL_1432921, EPI_ISL_1432923, EPI_ISL_1432925, EPI_ISL_1432927, EPI_ISL_1432929, EPI_ISL_1432944, EPI_ISL_1432946, EPI_ISL_1432948, EPI_ISL_1432950 to 1432951, EPI_ISL_1432953, EPI_ISL_1432955, EPI_ISL_1432957, EPI_ISL_1432959 to 1432960, EPI_ISL_1432962, EPI_ISL_1432964, EPI_ISL_1432966, EPI_ISL_1432968 to 1432969, EPI_ISL_1432971, EPI_ISL_1432973, EPI_ISL_1432975, EPI_ISL_1432977, EPI_ISL_1432979, EPI_ISL_1432981, EPI_ISL_1432983 to 1432984, EPI_ISL_1432986, EPI_ISL_1432988, EPI_ISL_1432990, EPI_ISL_1432992, EPI_ISL_1432994, EPI_ISL_1432996 to 1432997, EPI_ISL_1432999, EPI_ISL_1433001, EPI_ISL_1433003, EPI_ISL_1433005, EPI_ISL_1433007 to 1433008, EPI_ISL_1433011 to 1433012, EPI_ISL_1433014, EPI_ISL_1433016, EPI_ISL_1433018, EPI_ISL_1433020, EPI_ISL_1433022, EPI_ISL_1433024, EPI_ISL_1433026, EPI_ISL_1433028, EPI_ISL_1433030, EPI_ISL_1433032, EPI_ISL_1433034, EPI_ISL_1433036, EPI_ISL_1433038, EPI_ISL_1433040, EPI_ISL_1433042 to 1433043, EPI_ISL_1433045, EPI_ISL_1433047, EPI_ISL_1433049, EPI_ISL_1433051, EPI_ISL_1433053, EPI_ISL_1433055 to 1433056, EPI_ISL_1433293, EPI_ISL_1433295, EPI_ISL_1433297, EPI_ISL_1433307, EPI_ISL_1433310, EPI_ISL_1433312, EPI_ISL_1433314, EPI_ISL_1433316, EPI_ISL_1433318, EPI_ISL_1433320, EPI_ISL_1433322, EPI_ISL_1433324, EPI_ISL_1433326, EPI_ISL_1433328, EPI_ISL_1433330 to 1433331, EPI_ISL_1433333, EPI_ISL_1433335, EPI_ISL_1433337, EPI_ISL_1433339, EPI_ISL_1433341, EPI_ISL_1433343, EPI_ISL_1433345, EPI_ISL_1433348, EPI_ISL_1433350, EPI_ISL_1433352 to 1433353, EPI_ISL_1433355, EPI_ISL_1433357, EPI_ISL_1433359, EPI_ISL_1433361, EPI_ISL_1433363, EPI_ISL_1433365, EPI_ISL_1433367, EPI_ISL_1433369, EPI_ISL_1433371, EPI_ISL_1433373, EPI_ISL_1433391, EPI_ISL_1433393, EPI_ISL_1433395, EPI_ISL_1433397, EPI_ISL_1433399, EPI_ISL_1433401, EPI_ISL_1433403, EPI_ISL_1433405, EPI_ISL_1433407, EPI_ISL_1433409, EPI_ISL_1433411, EPI_ISL_1433413, EPI_ISL_1433415, EPI_ISL_1433417, EPI_ISL_1433419, EPI_ISL_1433421, EPI_ISL_1433423, EPI_ISL_1433425, EPI_ISL_1433427, EPI_ISL_1433429, EPI_ISL_1433431, EPI_ISL_1433433, EPI_ISL_1433436, EPI_ISL_1433438, EPI_ISL_1433440, EPI_ISL_1433442 to 1433443, EPI_ISL_1433445, EPI_ISL_1433447, EPI_ISL_1433450, EPI_ISL_1433452, EPI_ISL_1433454 to 1433465, EPI_ISL_1433467, EPI_ISL_1433469, EPI_ISL_1433471, EPI_ISL_1433473, EPI_ISL_1433475, EPI_ISL_1433477, EPI_ISL_1433479, EPI_ISL_1433481, EPI_ISL_1433483, EPI_ISL_1433485, EPI_ISL_1433487, EPI_ISL_1433489, EPI_ISL_1433492, EPI_ISL_1433494, EPI_ISL_1433496, EPI_ISL_1433498, EPI_ISL_1433500, EPI_ISL_1433502, EPI_ISL_1433504, EPI_ISL_1433506, EPI_ISL_1433508, EPI_ISL_1433510, EPI_ISL_1433512, EPI_ISL_1433514, EPI_ISL_1433516, EPI_ISL_1433519, EPI_ISL_1433521, EPI_ISL_1433523, EPI_ISL_1433525, EPI_ISL_1433527, EPI_ISL_1433529, EPI_ISL_1433531 to 1433532, EPI_ISL_1433534, EPI_ISL_1433536, EPI_ISL_1433538, EPI_ISL_1433540, EPI_ISL_1433542, EPI_ISL_1433544			
see above	Pathogen Genomics Center, National Institute of Infectious Diseases	Pathogen Genomics Center, National Institute of Infectious Diseases	Hajime Kamiya; Kentaro Itokawa; Makoto Kuroda; Masanori Hashino; Motoi Suzuki; Rina Tanaka; Ryohei Nomoto; Tsuyoshi Sekizuka
EPI_ISL_437459 to 437479, EPI_ISL_437481 to 437497, EPI_ISL_437691 to 437762, EPI_ISL_512874 to 513264	Pathogen Genomics Lab King Abdullah University of Science and Technology(KAUST)	Pathogen Genomics Lab King Abdullah University of Science and Technology(KAUST)	Abdulaziz Alahmadi; Afrah Alsomali; Ahmad Bakur Mahmoud; Amanda; Amanda Ooi; Amit Kumar Subudhi; Amit Subudhi; Anwar Hashem; Arnab Pain; Asim Khogeer; Fadwa Alofi; Fathia Ben Rached; Fathia Ben-Rached; Fathia Rached; Jumana Taha; Kahled Algithami; Khaled Algithami; Luke; Luke Esau; Naif Almontashiri; Nashwa Al-khotani; Raeece Naeem; Rahul P Salunke; Rahul Salunke; Sara Mfarrej; Sharif Hala
EPI_ISL_470897 to 470899	Pathogenic Microorganisms Variability Laboratory	Pathogenic Microorganisms Variability Laboratory	Alexander Gintsburg; Alexey Shchetinin; Andrey Botikov; Elena Shidlovskaya; Maria Nikiforova; Nadezhda Kuznetsova; Vladimir Gushchin
EPI_ISL_566077 to 566083, EPI_ISL_596318 to 596352, EPI_ISL_1400496, EPI_ISL_1400499, EPI_ISL_1400508, EPI_ISL_1400517, EPI_ISL_1400569 to 1400570, EPI_ISL_1491584 to 1491587, EPI_ISL_1491589 to 1491592, EPI_ISL_1491594 to 1491596, EPI_ISL_1491603, EPI_ISL_1491605 to 1491607, EPI_ISL_1491614, EPI_ISL_1491617, EPI_ISL_1491628 to 1491670			
see above	Pathogenic Microorganisms Variability Laboratory	WHO National Influenza Centre Russian Federation	Alexander Gintsburg; Alexey Masharsky; Alexey Shchetinin; Andrey Komissarov; Andrey Pochtovyy; Anna Ignatieva; Anna Ivanova; Artem Fadeev; Artem Tkachuk; Daria Danilenko; Dmitry Bazhenov; Dmitry Lioznov; Ekaterina Milashenko; Elena Burtseva; Elena Nabieva; Elena Shidlovskaya; Elena Vokalova; Elizaveta Divisenko; Evgeniya Mukasheva; Evgeny Usachev; Evgeny Usachev; Georgii Bazykin; Kirill Krasnoslobotsev; Ksenia Safina; Kseniya Komissarova; Ludmila Kolobukhina; Maria Baturova; Maria Nikiforova; Maxim Rubalsky; Nadezhda Kuznetsova; Oleg Rubalsky; Olga Burgasova; Sergey Alkhovsky; Svetlana Smetanina; Svetlana Trushakova; Tatyana Vishnevskaya; Valeria Bacalin; Vladimir Gushchin
EPI_ISL_583619 to 583629	Pathologie-Labor Dr. Obrist- Dr. Brunhuber	Bergthaler laboratory, CeMM Research Center for Molecular Medicine of the Austrian Academy of Sciences	Adi Steinrigl; Alexander Lercher; Alexandra Popa; Andreas Bergthaler; Benedikt Agerer; Christian Paar; Christoph Bock; Daniela Schmid; Dorothee von Laer; Elisabeth Puchhammer-Stoeckl; Franz Allerberger; Gernot Walder; Gregor Hörmann; Guenter Weiss; Gunther Vogl; Henrique Colaco; Jakob-Wendelin Genger; Jan Laine; Judith Aberle; Kinga Rigler-Hohenwarter; Lukas Endler; Manfred Nairz; Mark Smyth; Martin Senekowitsch; Michael Schuster; Peter Hufnagl; Peter Obrist; Rainer Gattringer; Sabine Sussitz-Rack; Stephan Aberle; Thomas Penz; Wegene Borena
EPI_ISL_421636	Pathology North	Public Health Virology Laboratory	Alyssa Pyke; Amanda De Jong; Andrew Van Den Hurk; Bixing Huang; Carmel Taylor; David Warrilow; Doris Genge; Elisabeth Gamez; Glen Hewitson; Ian Maxwell Mackay; Inga Sultana; Jamie McMahon; Jean Barcelon; Judy Northill; Mitchell Finger; Natalie Simpson; Neelima Nair; Peter Burtonclay; Peter Moore; Sarah Wheatley; Sean Moody; Sonja Hall-Mendelin; Timothy Gardam; and Frederick Moore
EPI_ISL_513335, EPI_ISL_513360, EPI_ISL_513368, EPI_ISL_526152, EPI_ISL_593655 to 593658	Pathology North - Hunter - NSW Health Pathology	NSW Health Pathology - Institute of Clinical Pathology and Medical Research; Westmead Hospital; University of Sydney	CIDM-PH et al.
EPI_ISL_451487 to 451488, EPI_ISL_451552, EPI_ISL_451604, EPI_ISL_513386 to 513388	Pathology North - NSW Health Pathology	NSW Health Pathology - Institute of Clinical Pathology and Medical Research; Westmead Hospital; University of Sydney	CIDM-PH et al.
EPI_ISL_478673 to 478674, EPI_ISL_513375 to 513376, EPI_ISL_526128, EPI_ISL_526130, EPI_ISL_526132 to 526133, EPI_ISL_526154 to 526155, EPI_ISL_526170, EPI_ISL_544969, EPI_ISL_544976, EPI_ISL_544982, EPI_ISL_544991, EPI_ISL_545008, EPI_ISL_591493, EPI_ISL_593659			
see above	Pathology North - Royal North Shore Hospital - NSW Health Pathology	NSW Health Pathology - Institute of Clinical Pathology and Medical Research; Westmead Hospital; University of Sydney	CIDM-PH et al.
EPI_ISL_451587, EPI_ISL_451589, EPI_ISL_451600, EPI_ISL_490043	Pathology North Hunter- NSW Health Pathology	NSW Health Pathology - Institute of Clinical Pathology and Medical Research; Westmead Hospital; University of Sydney	CIDM-PH et al.
EPI_ISL_407894, EPI_ISL_407896, EPI_ISL_410717 to 410718, EPI_ISL_414414, EPI_ISL_417031, EPI_ISL_418802 to 418804, EPI_ISL_418807 to 418808, EPI_ISL_444611, EPI_ISL_444793			
see above	Pathology Queensland	Public Health Virology Laboratory	Alyssa Pyke; Amanda De Jong; Andrew Van Den Hurk; Ben Huang; Bixing Huang; Carmel Taylor; David Warrilow; Doris Genge; Elisabeth Gamez; Glen Hewitson; Ian Maxwell Mackay; Inga Sultana; Jamie McMahon; Jean Barcelon; Judy Northill; Mitchell Finger; Natalie Simpson; Neelima Nair; Peter Burtonclay; Peter Moore; Sarah Wheatley; Sean Moody; Sonja Hall-Mendelin; Timothy Gardam; and Frederick Moore; and Frederick Moore.
EPI_ISL_447595	Pathology Queensland, Sunshine Coast University Hospital	Public Health Virology Laboratory	Alyssa Pyke; Amanda De Jong; Andrew Van Den Hurk; Bixing Huang; Carmel Taylor; David Warrilow; Doris Genge; Elisabeth Gamez; Glen Hewitson; Ian Maxwell Mackay; Inga Sultana; Jamie McMahon; Jean Barcelon; Judy Northill; Mitchell Finger; Natalie Simpson; Neelima Nair; Peter Burtonclay; Peter Moore; Sarah Wheatley; Sean Moody; Sonja Hall-Mendelin; Timothy Gardam; and Frederick Moore
EPI_ISL_451530 to 451531, EPI_ISL_451533, EPI_ISL_451536, EPI_ISL_451542 to 451544, EPI_ISL_451549, EPI_ISL_451559 to 451565, EPI_ISL_451578, EPI_ISL_451581, EPI_ISL_451603, EPI_ISL_451608, EPI_ISL_451642 to 451643, EPI_ISL_455037, EPI_ISL_455071, EPI_ISL_455094			
see above	Pathology Sydney South West - NSW Health Pathology	NSW Health Pathology - Institute of Clinical Pathology and Medical Research; Westmead Hospital; University of Sydney	CIDM-PH et al.

EPI_ISL_451490 to 451516, EPI_ISL_451522 to 451529, EPI_ISL_451532, EPI_ISL_451535, EPI_ISL_451537 to 451539, EPI_ISL_451541, EPI_ISL_451546 to 451547, EPI_ISL_451550 to 451551, EPI_ISL_451556 to 451557, EPI_ISL_451566, EPI_ISL_451568 to 451571, EPI_ISL_451573, EPI_ISL_451575 to 451577, EPI_ISL_451579 to 451580, EPI_ISL_451582, EPI_ISL_451584 to 451586, EPI_ISL_451606 to 451607, EPI_ISL_451613 to 451630, EPI_ISL_451637 to 451640, EPI_ISL_455028 to 455035, EPI_ISL_455038 to 455040, EPI_ISL_455044 to 455047, EPI_ISL_455049, EPI_ISL_455055 to 455062, EPI_ISL_455064, EPI_ISL_455066 to 455067, EPI_ISL_455069 to 455070, EPI_ISL_455095, EPI_ISL_490036, EPI_ISL_490038 to 490039, EPI_ISL_490044, EPI_ISL_490046, EPI_ISL_498748 to 498750, EPI_ISL_498761 to 498762, EPI_ISL_498766 to 498767, EPI_ISL_498776 to 498777, EPI_ISL_498779 to 498780, EPI_ISL_513321 to 513322, EPI_ISL_513324 to 513326, EPI_ISL_513332, EPI_ISL_513338 to 513339, EPI_ISL_513342, EPI_ISL_513345 to 513346, EPI_ISL_513350 to 513351, EPI_ISL_513358 to 513359, EPI_ISL_513369 to 513371, EPI_ISL_513378, EPI_ISL_513380, EPI_ISL_513390 to 513395, EPI_ISL_526115 to 526118, EPI_ISL_526138 to 526141, EPI_ISL_526150 to 526151, EPI_ISL_526156, EPI_ISL_526161 to 526165, EPI_ISL_526199, EPI_ISL_526689, EPI_ISL_544955, EPI_ISL_544986, EPI_ISL_544989 to 544990, EPI_ISL_545006, EPI_ISL_545029, EPI_ISL_545032 to 545033, EPI_ISL_545038, EPI_ISL_545046 to 545047, EPI_ISL_545050, EPI_ISL_591494 to 591501, EPI_ISL_593660 to 593682, EPI_ISL_593770			
see above	Pathology West - NSW Health Pathology	NSW Health Pathology - Institute of Clinical Pathology and Medical Research; Westmead Hospital; University of Sydney	CIDM-PH et al.
EPI_ISL_1468422 to 1468423	Penitenciaria Compacta de Avanhandava	Instituto Adolfo Lutz, Interdiciplinary Procedures Center, Strategic Laboratory	Caio Vinicius Dias Lopes; Claudia Regina Gonçalves; Claudio Tavares Sacchi; Erica Valessa Ramos Gomes; Karoline Rodrigues Campos
EPI_ISL_1500196	Peoria County Jail	Illinois Department of Public Health - Springfield Lab	Bryan Sim; Gordon McCall
EPI_ISL_455585, EPI_ISL_455608	Phramongkutklao Hospital	National Institute of Health. Department of medical Sciences, Ministry of Public Health, Thailand	Chittaganpitch; Malinee; Okada; Parnmen; Phuygun; Pilailuk; Siripaporn; Sittiporn; Sunthareeya; Thanadachakul; Thanutsapa; Waicharoen; Warawan; Wongboot
EPI_ISL_468066	Physiology, Istanbul Medeniyet University	Physiology, Istanbul Medeniyet University	B. and Kara, A.; Caykara, B.; Cevher Keskin; H.H.; Pence; Pence, S.; Tekin, S.; Yiyit, N.
EPI_ISL_514750	Pirogov Russian National Research Medical University	Pirogov Russian National Research Medical University, Research and Development	Blagodatskikh; K.A.
EPI_ISL_443308	Plaisance	National Reference Center for Viruses of Respiratory Infections, Institut Pasteur, Paris	Angela Brisebarre; Etienne Simon-Lorière; Flora Donati; Marion Barbet; Maud Vanpeene; Mélanie Albert; Méline Bizard; Sylvie Behillil; Sylvie van der Werf; Vincent Enouf
EPI_ISL_1437047, EPI_ISL_1437053, EPI_ISL_1437057, EPI_ISL_1437059, EPI_ISL_1437061, EPI_ISL_1437063, EPI_ISL_1437065, EPI_ISL_1437070, EPI_ISL_1437075, EPI_ISL_1437077, EPI_ISL_1437079, EPI_ISL_1437082, EPI_ISL_1437084, EPI_ISL_1437686, EPI_ISL_1437688, EPI_ISL_1437701, EPI_ISL_1437703 to 1437704, EPI_ISL_1437707, EPI_ISL_1437709, EPI_ISL_1437711, EPI_ISL_1437722, EPI_ISL_1437725, EPI_ISL_1437727, EPI_ISL_1437737, EPI_ISL_1438443, EPI_ISL_1438445, EPI_ISL_1438449, EPI_ISL_1438454, EPI_ISL_1438456, EPI_ISL_1438458, EPI_ISL_1438460, EPI_ISL_1438462, EPI_ISL_1438464, EPI_ISL_1438466, EPI_ISL_1439453, EPI_ISL_1439457, EPI_ISL_1439459, EPI_ISL_1439461, EPI_ISL_1439463, EPI_ISL_1439465, EPI_ISL_1439467, EPI_ISL_1439473, EPI_ISL_1439476 to 1439477, EPI_ISL_1439479, EPI_ISL_1439486, EPI_ISL_1527634 to 1527660, EPI_ISL_1528170 to 1528180, EPI_ISL_1548078 to 1548110, EPI_ISL_1557247 to 1557253, EPI_ISL_1559615 to 1559625			
see above	Plateforme de testing Namuroise	Plateforme de testing Namuroise	; Céline Maschietto; Degosserie Jonathan; Denis Olivier; Mullier François; Otto Gaetan
EPI_ISL_1443699 to 1443747, EPI_ISL_1498313 to 1498342, EPI_ISL_1498346 to 1498372, EPI_ISL_1498374 to 1498377, EPI_ISL_1549570, EPI_ISL_1549572 to 1549573, EPI_ISL_1549575 to 1549576, EPI_ISL_1549578 to 1549579, EPI_ISL_1549581 to 1549582, EPI_ISL_1549584, EPI_ISL_1549586 to 1549588			
see above	Platform BIS UZA/UAntwerpen	Labo Klinische Biologie, UZA	Basil Britto Xavier; Christine Lammens; Herman Goossens; Jasmine Coppens; Marie Le Mercier; Veerle Matheeussen
EPI_ISL_1416761, EPI_ISL_1416765 to 1416768, EPI_ISL_1416772 to 1416775, EPI_ISL_1416779, EPI_ISL_1416782 to 1416791, EPI_ISL_1416793 to 1416794, EPI_ISL_1416798 to 1416799, EPI_ISL_1416801, EPI_ISL_1416803, EPI_ISL_1416805 to 1416810, EPI_ISL_1455659, EPI_ISL_1455662, EPI_ISL_1455664, EPI_ISL_1455667 to 1455668, EPI_ISL_1498122, EPI_ISL_1498265 to 1498269, EPI_ISL_1498271 to 1498281, EPI_ISL_1498289, EPI_ISL_1498295, EPI_ISL_1498297 to 1498299, EPI_ISL_1498303, EPI_ISL_1558615 to 1558714			
see above	Platform BIS UZA/UAntwerpen	UAntwerp, Laboratory of Medical Microbiology	Basil Britto Xavier; Christine Lammens; Herman Goossens; Jasmine Coppens; Marie Le Mercier; Veerle Matheeussen
EPI_ISL_1469433 to 1469436, EPI_ISL_1469438 to 1469449	Platform BIS UZA/UAntwerpen	UZA, Clinical Biology	Basil Britto Xavier; Christine Lammens; Herman Goossens; Jasmine Coppens; Marie Le Mercier; Veerle Matheeussen
EPI_ISL_522547 to 522548	Platforme CYROI	UMR PIMIT Université de La Réunion	Camille Lebarbenchon; David Wilkinson; Patrick Mavingui
EPI_ISL_1534424	Plettenberg Bay Clinic wc PLC	NHLS/UCT	Arash Iranzadeh; Bruna Galvao; Carolyn Williamson; Deelan Doolabh; Diana Hardie; Emmanuel SJ; Innocent Mudau; Kruger Marais; Lynn Tyers; Marvin Hsiao; Stephen Korsman; Tegally H; de Oliveira T
EPI_ISL_1393717 to 1393719, EPI_ISL_1394281 to 1394282	Podkarpackie Centrum Genetyczne OncoGenLab Pracownia Diagnostyki Molekularnej	1. National Institute of Public Health - National Institute of Hygiene; 2. Eurofins Genomics Europe Sequencing GmbH	ECDC COVID-19 WGS support team; Eurofins Genomics Europe Sequencing Team; Gierczyki Rafa; Sadkowska-Todys Magorzata; Wokowicz Tomasz; Zacharczuk Katarzyna
EPI_ISL_536263 to 536264	Point de Service de Témiscaming-et-Kipawa	Laboratoire de santé publique du Québec	Guillaume Bourque; Ioannis Ragoussis; Jesse Shapiro; Mark Lathrop and Michel Roger on behalf of the CoVSeQ research group; Sandrine Moreira
EPI_ISL_539850 to 539851	Pok Oi Hospital	Hong Kong Department of Health	Alan K.L. Tsang; Dominic N.C. Tsang; Edman T.K. Lam; Peter C.W. Yip; Rickjason C.W. Chan
EPI_ISL_1533719	Policlinica Munic da Est Turistica de Holambra	Instituto Adolfo Lutz, Interdiciplinary Procedures Center, Strategic Laboratory	Caio Vinicius Dias Lopes; Claudia Regina Gonçalves; Claudio Tavares Sacchi; Erica Valessa Ramos Gomes; Karoline Rodrigues Campos; Leonardo Jose Tadeu de Araujo
EPI_ISL_1492568	Policlinico Casilino	INMI Lazzaro Spallanzani IRCCS	A Di Caro; B Bartolini; CEM Gruber; E Giombini; F Messina; F Santini; G Bonfiglio; M Rueca; MR Capobianchi; O Butera
EPI_ISL_1492575	Polo Sanitario San Feliciano	INMI Lazzaro Spallanzani IRCCS	A Di Caro; B Bartolini; CEM Gruber; E Giombini; F Messina; F Santini; G Bonfiglio; M Rueca; MR Capobianchi; O Butera
EPI_ISL_1497525, EPI_ISL_1506115, EPI_ISL_1508898 to 1508901	Pomona Valley Hospital Medical Center	Los Angeles County PHL	P. Hemarajata et al.
EPI_ISL_1469812	Posto de Saúde Cambará do Sul	Epiclin	Ana Paula Muterle; Carolina Comerlato; Eliana Márcia Da Ros Wendland; Fernando Hayashi Sant'Anna; Janira Prichula; Juliana Comerlato
EPI_ISL_1394078 to 1394079, EPI_ISL_1394083 to 1394085, EPI_ISL_1538497, EPI_ISL_1538509, EPI_ISL_1538568 to 1538574, EPI_ISL_1538839, EPI_ISL_1538913 to 1538914, EPI_ISL_1539092, EPI_ISL_1539137			
see above	Powiatowa Stacja Sanitarno-Epidemiologiczna w Radomiu	1. National Institute of Public Health - National Institute of Hygiene; 2. Eurofins Genomics Europe Sequencing GmbH	ECDC COVID-19 WGS support team; Eurofins Genomics Europe Sequencing Team; Gierczyki Rafa; Sadkowska-Todys Magorzata; Wokowicz Tomasz; Zacharczuk Katarzyna
EPI_ISL_1538749 to 1538750, EPI_ISL_1539150	Pracownia Biologii Molekularnej Laboratorium Centralne SPZOZ w Krotoszynie	1. National Institute of Public Health - National Institute of Hygiene; 2. Eurofins Genomics Europe Sequencing GmbH	ECDC COVID-19 WGS support team; Eurofins Genomics Europe Sequencing Team; Gierczyki Rafa; Sadkowska-Todys Magorzata; Wokowicz Tomasz; Zacharczuk Katarzyna
EPI_ISL_1447237, EPI_ISL_1447268	Pracownia Diagnostyki Molekularnej WSSK	1. National Institute of Public Health - National Institute of Hygiene; 2. Eurofins Genomics Europe Sequencing GmbH	ECDC COVID-19 WGS support team; Eurofins Genomics Europe Sequencing Team; Gierczyki Rafa; Sadkowska-Todys Magorzata; Wokowicz Tomasz; Zacharczuk Katarzyna
EPI_ISL_1394105, EPI_ISL_1538865, EPI_ISL_1538926, EPI_ISL_1539200	Pracownia Genetyki Klinicznej i Patologii Molekularnej 10 WSK	1. National Institute of Public Health - National Institute of Hygiene; 2. Eurofins Genomics Europe Sequencing GmbH	ECDC COVID-19 WGS support team; Eurofins Genomics Europe Sequencing Team; Gierczyki Rafa; Sadkowska-Todys Magorzata; Wokowicz Tomasz; Zacharczuk Katarzyna
EPI_ISL_1393669 to 1393672, EPI_ISL_1538435, EPI_ISL_1538534, EPI_ISL_1538686, EPI_ISL_1538880 to 1538881, EPI_ISL_1538995 to 1538997, EPI_ISL_1539178			
see above	Pracownia Mikrobiologiczna Wojewódzki Szpital Specjalistyczny	1. National Institute of Public Health - National Institute of Hygiene; 2. Eurofins Genomics Europe Sequencing GmbH	ECDC COVID-19 WGS support team; Eurofins Genomics Europe Sequencing Team; Gierczyki Rafa; Sadkowska-Todys Magorzata; Wokowicz Tomasz; Zacharczuk Katarzyna
EPI_ISL_430841, EPI_ISL_434698 to 434699, EPI_ISL_434702 to 434706, EPI_ISL_455606 to 455607	Praram 9 Hospital	National Institute of Health. Department of medical Sciences, Ministry of Public Health, Thailand	Chittaganpitch; Malinee; Okada; Parnmen; Phuygun; Pilailuk; Siripaporn; Sittiporn; Sunthareeya; Thanadachakul; Thanutsapa; Waicharoen; Warawan; Wongboot
EPI_ISL_1436971, EPI_ISL_1436993, EPI_ISL_1437017, EPI_ISL_1437089, EPI_ISL_1437279, EPI_ISL_1438311, EPI_ISL_1441366, EPI_ISL_1442846			
see above	Praxisgemeinschaft für Laboratoriumsmedizin Labor Blumenstraße; Praxis Dr. E. Wietschel & Dr. F. Wietschel	Robert Koch Institute	
EPI_ISL_1436976, EPI_ISL_1436987, EPI_ISL_1436989, EPI_ISL_1438479, EPI_ISL_1438546, EPI_ISL_1441486, EPI_ISL_1441631, EPI_ISL_1441636, EPI_ISL_1442831, EPI_ISL_1442833			
see above	Praxisgemeinschaft für Laboratoriumsmedizin Labor Blumenstraße; Praxis Dr. Sel und Dr. Späte	Robert Koch Institute	
EPI_ISL_418255	Presidio Ospedaliero "S. Spirito" - PESCARA	Istituto Zooprofilattico Sperimentale dell'Abruzzo e Molise "G. Caporale"	Cammà C; Di Pasquale A; Lorusso A; Mangone I; Marcacci M; Monaco F; Puglia I; Rinaldi A; Savini G
EPI_ISL_529013	Presidio Ospedaliero "S.Filippo e Nicola"-Avezzano	Istitituto Zooprofilattico Sperimentale dell'Abruzzo e Molise	Ancora M; Cammà C; Curini V; Di Domenico M; Di Pasquale A; Lorusso A; Mangone I; Marcacci M; Puglia I; Rinaldi A; Savini G.

		"G.Caporale"	
EPI_ISL_528920 to 528921	Presidio Ospedaliero "Santo Spirito"-Pescara	Istituto Zooprofilattico Sperimentale dell'Abruzzo e Molise "G.Caporale"	Ancora M; Cammà C; Curini V; Di Domenico M; Di Pasquale A; Lorusso A; Mangone I; Marcacci M; Puglia I; Rinaldi A; Savini G.
EPI_ISL_429226 to 429227	Presidio Ospedaliero Santo Spirito	Istituto Zooprofilattico Sperimentale dell'Abruzzo e Molise "G. Caporale"	Ancora M; Camma C; Curini V; Di Domenico M; Di Pasquale A; Lorusso A; Mangone I; Marcacci M; Puglia I; Rinaldi A; Savini G
EPI_ISL_1391053 to 1391057	Presidio di Brindisi Di Summa - Perrino	Istituto Zooprofilattico Sperimentale della Puglia e della Basilicata	Bianco A.; Capozzi L.; Del Sambro L.; Difato L.; Giannico A.; Parisi A.; Ridolfi D.; Santoro A.; Simone D.
EPI_ISL_418258 to 418259	Presidio ospedaliero "Santo Spirito"	Istituto Zooprofilattico Sperimentale dell'Abruzzo e Molise "G. Caporale"	Ancora M; Cammà C; Curini V; Di Domenico M; Di Pasquale A; Lorusso A; Mangone I; Marcacci M; Puglia I; Rinaldi A; Savini G.
EPI_ISL_1528999	Preston Taylor Community Health Center	WVU and Marshall University Combined Genomics Core Facilities	"James Denvir; Peter Perrotta; Peter Stoilov; Ryan Percifield"; Wesley Kimble
EPI_ISL_436097	Prince Charles Hospital	Public Health Virology Laboratory, Forensics and Scientific Services, Queensland Health	Alyssa Pyke; Amanda De Jong; Andrew Van Den Hurk; Bixing Huang; Carmel Taylor; David Warrilow; Doris Genge; Elisabeth Gamez; Glen Hewitson; Ian Maxwell Mackay; Inga Sultana and Frederick Moore; Jamie McMahon; Jean Barcelon; Judy Northill; Lisa Leckie; Mitchell Finger; Natalie Simpson; Neelima Nair; Peter Burtonclay; Peter Moore; Sarah Wheatley; Sean Moody; Sonja Hall-Mendelin
EPI_ISL_417064, EPI_ISL_539813	Prince of Wales Hospital	Hong Kong Department of Health	Alan K.L. Tsang; Dominic N.C. Tsang; Edman T.K. Lam; Peter C.W. Yip; Rickjason C.W. Chan
EPI_ISL_539806 to 539808	Princess Margaret Hospital	Hong Kong Department of Health	Alan K.L. Tsang; Dominic N.C. Tsang; Edman T.K. Lam; Peter C.W. Yip; Rickjason C.W. Chan
EPI_ISL_1402423 to 1402428, EPI_ISL_1439578, EPI_ISL_1439580, EPI_ISL_1439584, EPI_ISL_1439589, EPI_ISL_1439593, EPI_ISL_1477044 to 1477046			
see above	Private clinic of Biogen Med, Tashkent, Uzbekistan	Center of Genomics and bioinformatics, Bioinformatics laboratory	Abdurakhmon N Yusupov; Ibrokhim Y Abdurakhmonov.; Mirzakamol S Ayubov; Mukhammadjon H Mirzakhmedov; Shukhrat E Shermatov; Zabardast T Buriev
EPI_ISL_1435362, EPI_ISL_1435379, EPI_ISL_1435382, EPI_ISL_1435388, EPI_ISL_1435400, EPI_ISL_1439454, EPI_ISL_1439458, EPI_ISL_1439460, EPI_ISL_1439485, EPI_ISL_1439489			
see above	Procomcure Biotech Germany GmbH	Robert Koch Institute	
EPI_ISL_477204, EPI_ISL_514432	Prof. Massimo Zollo CEINGE TASK-FORCE COVID19 - Regione Campania	Prof. Massimo Zollo CEINGE TASK-FORCE COVID19 - Regione Campania	; 2; 2* 1 CEINGE Biotecnologie Avanzate; 8* and Massimo Zollo1; Angelo Boccia; Angelo Boccia2; Azienda Sanitaria Ospedali dei Colli; Claudia Tiberio; Claudia Tiberio4; Dae young Kong; Dae young Kong8; Fatemeh asadzadeh; Fatemeh asadzadeh1; Giorgia Borriello; Giorgia Borriello3; Giovanna Fusco; Giovanna Fusco3; Giovanni Paolella; Giovanni Paolella1; Giuseppe Castaldo; Giuseppe Castaldo1; Hong Yeoul Kim 7; Hong Yeoul Kim and Massimo Zollo; Indust; Italia 2 Dipartimento di Medicina Molecolare e Biotecnologie Mediche DMMBM University of Naples Federico II; Italia 3 Istituto Zooprofilattico Sperimentale del Mezzogiorno; Italia 4 -U.O.C. di Patologia Clinica Ospedale D. Cotugno; Italia 6 Department of Microbiology; Italy. 5 Università La Sapienza di Roma; Jae Ho Jung; Jae Ho Jung6; Jae Myun Lee; Jae Myun Lee5; Korea 8 Haim bio co.; Korea 7 Department of Surgery; Kyong Seop Yun; Kyong Seop Yun7; Laura Marrone; Laura Marrone1; Lorenzo Chiariotti; Lorenzo Chiariotti1; Ltd; Luigi Atripaldi; Luigi Atripaldi4; Marika Comegna; Marika Comegna1; Martina Bianchi; Martina Bianchi4; Maurizio Viscardi; Maurizio Viscardi3; Naples; Rino Cerino; Rino Cerino3; Roberto Siciliano; Roberto Siciliano1; Seoul; Sergio Brandi; Sergio Brandi3; Stefano Pascarella; Stefano Pascarella4; Veronica Ferrucci; Veronica Ferrucci1; Yonsei University College of Medicine
EPI_ISL_513513 to 513583	Programa de Oncovirologia, Instituto Nacional de Câncer	Programa de Oncovirologia, Instituto Nacional de Câncer	Andreia C. de Melo; Brunna M. Alves; Claudia Cicala; James Arthos; João P.B. Viola; Juliana D. Siqueira; Livia R. Goes; Marcelo A. Soares
EPI_ISL_591519 to 591521	Programa de Virología, Facultad de Medicina UC	Center for Mathematical Modeling and Center for Genome Regulation. Santiago, Chile	Allende ML; Ferres M.; Gaete A; Gaggero A; González M; Maass A; Palma R; Travisany D; Urra C; Valiente F; Varas M
EPI_ISL_574479 to 574492	Programme in Emerging Infectious Diseases, Duke-NUS Medical School	National Public Health Laboratory, National Centre for Infectious Diseases	Adrian Eng Zheng Kang; Danielle E Anderson; Lin Cui; Raymond Tzer Pin Lin; Sophie Octavia; Tze Minn Mak; Zhenyang Zhou
EPI_ISL_1469564, EPI_ISL_1469602, EPI_ISL_1469615, EPI_ISL_1469621, EPI_ISL_1469657, EPI_ISL_1469712, EPI_ISL_1469825, EPI_ISL_1479129			
see above	Pronto Atendimento Campo Bom	Epiclin	Ana Paula Muterle; Carolina Comerlato; Eliana Márcia Da Ros Wendland; Fernando Hayashi Sant'Anna; Janira Prichula; Juliana Comerlato
EPI_ISL_1469721	Pronto Atendimento Cruzeiro do Sul	Epiclin	Ana Paula Muterle; Carolina Comerlato; Eliana Márcia Da Ros Wendland; Fernando Hayashi Sant'Anna; Janira Prichula; Juliana Comerlato
EPI_ISL_527858	Pronto Atendimento Sancta Maggiore Jardim Paulista	Instituto Adolfo Lutz, Interdiciplinary Procedures Center, Strategic Laboratory	Claudia Regina Gonçalves; Claudio Tavares Sacchi; Erica Valessa Ramos Gomes
EPI_ISL_1533703	Pronto Atendimento Sao Jose	Instituto Adolfo Lutz, Interdiciplinary Procedures Center, Strategic Laboratory	Caio Vinicius Dias Lopes; Claudia Regina Gonçalves; Claudio Tavares Sacchi; Erica Valessa Ramos Gomes; Karoline Rodrigues Campos; Leonardo Jose Tadeu de Araujo
EPI_ISL_1533714	Pronto Socorro Dr Osmar Mesquita	Instituto Adolfo Lutz, Interdiciplinary Procedures Center, Strategic Laboratory	Caio Vinicius Dias Lopes; Claudia Regina Gonçalves; Claudio Tavares Sacchi; Erica Valessa Ramos Gomes; Karoline Rodrigues Campos; Leonardo Jose Tadeu de Araujo
EPI_ISL_603021	Pronto Socorro Dr. Conrado Cesarino Nuvolini	Instituto Adolfo Lutz, Interdiciplinary Procedures Center, Strategic Laboratory	Claudia Regina Gonçalves; Claudio Tavares Sacchi; Erica Valessa Ramos Gomes; Karoline Rodrigues Campos
EPI_ISL_471556	Pronto Socorro Jose Ibrahin	Instituto Adolfo Lutz, Interdiciplinary Procedures Center, Strategic Laboratory	Claudia Regina Gonçalves; Claudio Tavares Sacchi; Erica Valessa Ramos Gomes
EPI_ISL_527867	Pronto Socorro Municipal - Balneario São José	Instituto Adolfo Lutz, Interdiciplinary Procedures Center, Strategic Laboratory	Claudia Regina Gonçalves; Claudio Tavares Sacchi; Erica Valessa Ramos Gomes
EPI_ISL_523961	Pronto Socorro Municipal 21 de Junho	Instituto Adolfo Lutz, Interdiciplinary Procedures Center, Strategic Laboratory	Claudia Regina Gonçalves; Claudio Tavares Sacchi; Erica Valessa Ramos Gomes
EPI_ISL_515529	Pronto Socorro Municipal Julio Tupy	Instituto Adolfo Lutz, Interdiciplinary Procedures Center, Strategic Laboratory	Claudia Regina Gonçalves; Claudio Tavares Sacchi; Erica Valessa Ramos Gomes
EPI_ISL_515554, EPI_ISL_523958 to 523959	Pronto Socorro Municipal de Perus	Instituto Adolfo Lutz, Interdiciplinary Procedures Center, Strategic Laboratory	Claudia Regina Gonçalves; Claudio Tavares Sacchi; Erica Valessa Ramos Gomes
EPI_ISL_491475	Protacio Hospital	Research Institute for Tropical Medicine	Catalino Demetria; Criselda Bautista; Daria Manalo; Edelwisa Mercado; Francisco Gerardo Polotan; Inez Andrea Medado; Kirstyn Brunker; Ma. Angelica Tujan; Othoniel Jan Onza
EPI_ISL_582134	Protzer Lab	Protzer Lab	Alexander Karollus; Andrea Theumer; Dieter Hoffmann; Eva Schulte; Julien Gagneur; Max von Kleist; Oliver Drechsel; Ulrike Protzer
EPI_ISL_404895	Providence Regional Medical Center	Division of Viral Diseases, Centers for Disease Control and Prevention	C.R.; Gerber; Li, Y.; Lindstrom, S.; Lu, X.; Paden; Queen, K.; S.I.; Tao, Y.; Tong, S.; Zhang, J.
EPI_ISL_482292 to 482467, EPI_ISL_486830 to 486831	Providence St. Joseph Health Molecular Genomics Laboratory	Providence St. Joseph Health Molecular Genomics Laboratory	Alexa K Dowdell; Brian D Piening; Carlo B Bifulco; Fred L Robinson; Mary Campbell
EPI_ISL_538513	Provincial Health Laboratory Bekasi West Java	National Institute of Health Research and Development	AA; HA; HD; Ikawati; KD; KNA; Nugraha; Paisal; Pangesti; Pawestri; Puspa; Setiawaty, V.; Soekarso; Subangkit; T
EPI_ISL_1401567 to 1401722, EPI_ISL_1520181 to 1520329, EPI_ISL_1520331 to 1520365, EPI_ISL_1524015 to 1524329, EPI_ISL_1525000 to 1525001, EPI_ISL_1526607 to 1526777, EPI_ISL_1543921 to 1543923			
see above	Public Health Authority of the Slovak Republic	Laboratory of Genomics and Bioinformatics, Comenius University Science Park	Anna Giová; Diana Rusáková; Jaroslav Budiš; Miroslav Böhmer; Tatiana Sedláková; Tomáš Szemes
EPI_ISL_572329	Public Health Authority of the Slovak Republic, Bratislava	Faculty of Natural Sciences, Comenius University in Bratislava	Boris Klempa; Broa Brejová; Dominika Friová; Edita Staroová; Elena Tichá; Jozef Nosek; Juraj Kopáek; Kristína Boršová; Martina Liková; Martina Neboháová; Monika Sláviková; Sabina Fumaová Havlíková; Tomáš Vina; Viktória Hodorová; Viktória abanová; ubomíra Lukáiková

EPI_ISL_429815, EPI_ISL_469224 to 469240, EPI_ISL_482479	Public Health Laboratory	National Microbiology Laboratory	Adel Malek; Anna Majer; Elsie Grudeski; Gary Van Domselaar; George Zahariadis; Grace Seo; Jennifer Tanner; Kristyn Burak; Laura Gilbert; Matthew Gilmour; Morag Graham; Natalie Knox; Nathalie Bastien; Philip Mabon; Rhiannon Huzarewich; Robert Needle; Russell Mandes; Shari Tyson; Timothy Booth; Yan Li; Yang Yu
EPI_ISL_416521	Public Health Laboratory	Public Health Laboratory, Saudi CDC	A; Albarrag
EPI_ISL_613463 to 613540	Public Health Laboratory - Infectious Disease Lab, Minnesota Department of Health Infectious Disease Laboratory Submission Group	Minnesota Department of Health, Public Health Laboratory	Garfin, J.; Lorentz, A.; Plumb, M.; Wang, X.
EPI_ISL_1483026	Public Health Laboratory - Infectious Disease Lab, Minnesota Department of Health Infectious Disease Laboratory Submission Group	Public Health Laboratory - Infectious Disease Lab, Minnesota Department of Health Infectious Disease Laboratory Submission Group	Garfin, J.; Lorentz, A.; M. and Wang, X.; Plumb
EPI_ISL_416522	Public Health Laboratory, Saudi CDC	Public Health Laboratory, Saudi CDC	A; Albarrag
EPI_ISL_425177	Public Health Ontario	Public Health Agency of Canada - National Microbiology Laboratory	Amrit S. Boese; Anders Leung; Darwyn Kobasa; Jonathan Audet; Joshua Quick; Morag Graham; Natalie Knox; Nikesh Tailor; Shari Tyson
EPI_ISL_413015	Public Health Ontario Laboratory	National Microbiology Laboratory	Adrian Zetner; Anna Majer; Darryl Falzarano; Erika Landry; Gerdts Volker; Grace Seo; Guillaume Poliquin; Jocelyne Lew; Jonathan Gubbay; Matthew Gilmour; Morag Graham; Natalie Knox; Nathalie Bastien; Philip Mabon; Rob Kozak; Samira Mubareka; Shari Tyson; Stephanie Booth; Timothy Booth; Tom Graefenhan; Yan Li
EPI_ISL_576131 to 576144, EPI_ISL_593597 to 593645	Public Health Virology Laboratory, Forensic and Scientific Services (PHV-FSS)	Public Health Virology Laboratory, Forensic and Scientific Services (PHV-FSS)	Son Nguyen et al
EPI_ISL_1396522 to 1396523, EPI_ISL_1396528 to 1396529, EPI_ISL_1396531 to 1396533, EPI_ISL_1404915 to 1404919, EPI_ISL_1404921 to 1404922, EPI_ISL_1424002 to 1424003, EPI_ISL_1465887 to 1465888, EPI_ISL_1465892, EPI_ISL_1483027 to 1483031, EPI_ISL_1516882 to 1516884, EPI_ISL_1516888 to 1516889			
see above	Public Health Virology-Forensic and Scientific Services (PHV-FSS)	Public Health Virology-Forensic and Scientific Services (PHV-FSS)	Son Nguyen
EPI_ISL_512785 to 512809, EPI_ISL_513310	Public Health, United States Air Force School of Aerospace Medicine	Public Health, United States Air Force School of Aerospace Medicine	A.C.; A.K.; A.W.; B.C.; C.R.; Chapleau; Connors; E.A.; Fries; J.R.; Javorina; Lambert; Macias; Meyer; Purves; R.R.; R.R. and Starr; S.M.; Starr
EPI_ISL_513311 to 513313	Public Health, United States Air Force School of Aerospace Medicine	Public Health, United States Air Force School of Aerospace Medicine	A.C.; A.K.; A.W.; B.C.; C.R.; Chapleau; Connors; E.A.; Fries; J.R.; Javorina; Lambert; Macias; Meyer; Purves; R.R. and Starr; S.M.
EPI_ISL_1495179 to 1495266	Public Health Authority of the Slovak Republic	Bergthaler laboratory, CeMM Research Center for Molecular Medicine of the Austrian Academy of Sciences	Andreas Bergthaler; Anna Schedl; Bekir Erguner; Benedikt Agerer; Christoph Bock; Fabian Amman; Jan Laine; Lukas Endler; Maelle Le Moing; Martin Senekowitsch; Michael Schuster; Petr Triska; Thomas Penz
EPI_ISL_434541 to 434553	Puerto Rico Department of Health	Centers for Disease Control and Prevention, Dengue Branch	Betzabel Flores; Chaney Kalinich; Fabiola Cruz; Gilberto A. Santiago; Glenda Gonzalez; Jessica I. Falcon; Jorge L. Munoz-Jordan; Joseph Fauver; Keyla Charriez; Nathan Grubaugh
EPI_ISL_1398936	Purwakarta Public Health	West Java Health Laboratory	Azzania Fibriani; Cut Nur Cinthia Alamanda; Ema Rahmawati; Isak Solihin; Kamila Tania; Karimatu Khoirunnisa; Miftahul Faridl; Rifky Waluyajati Rachman; Rini Robiani; Ryan Bayusantika Ristandi
EPI_ISL_437189	Pusat Pertamina Hospital	Eijkman Institute for Molecular Biology, Ministry of Research and Technology/National Agency for Research and Innovation	Amin Soebandrio; David H Muljono; Edison Johar; Frilasita A Yudhaputri; Hidayat Trimarsanto; Khin Saw Myint; Safarina G Malik
EPI_ISL_610155	Puskesmas Cangkrep	Genetics Working Group (Pokja Genetik) Faculty of Medicine, Public Health and Nursing Universitas Gadjah Mada (FK-KMK UGM); Disease Investigation Center Wates Ministry of Agriculture Indonesia; Department of Microbiology FK-KMK UGM; Laboratorium Diagnostik Yayasan Tahija World Mosquito Program (WMP) Yogyakarta Center for Tropical Medicine FK-KMK UGM; Integrated Research Center FK-KMK UGM; Department of Computer Science and Electronics FMIPA UGM; Balai Besar Teknik Kesehatan Lingkungan dan Pengendalian Penyakit (BBTKLPP) Yogyakarta	Afiahayati; Dwi AA Nugrahaningsih; Edwin W. Daniwijaya; Eggi Arguni; Endah Supriyati; Fadil Fahri; Gunadi; Havid Setyawan; Hendra Wibawa; Indaryati; Irene; Kristy Iskandar; Ludhang P. Rizki; Marcellus; Mohamad S. Hakim; Nawang Sukestiningsih; Nungki Anggorowati; Ririn Enggy Yuliyanti; Siswanto; Titik Nuryastuti; Tri Wibawa
EPI_ISL_574610	Puskesmas Tambora	Eijkman Institute for Molecular Biology, Ministry of Research and Technology/National Agency for Research and Innovation	Amin Soebandrio; David H Muljono; Edison Johar; Frilasita A Yudhaputri; Herawati Sudoyo; Hidayat Trimarsanto; Iskandar A Adnan; Khin Saw Myint; Safarina G Malik; Willy Agustine
EPI_ISL_579515 to 579758	QEII Health Sciences Centre	National Microbiology Laboratory (NML)	Anna Majer; Anneliese Landgraff; CanCOGeN's metadata curation team; Darian Hole; Elsie Grudeski; Gary Van Domselaar; Grace Seo; Jason LeBlanc; Jennifer Tanner; Morag Graham; Natalie Knox; Nathalie Bastien; Philip Mabon; Public Health Agency of Canada's CanCOGeN team; Rhiannon Huzarewich; Russell Mandes; Shari Tyson; Timothy Booth; Todd Hatchette; Yan Li
EPI_ISL_1423015 to 1423020, EPI_ISL_1423126, EPI_ISL_1423128 to 1423130, EPI_ISL_1423211 to 1423212, EPI_ISL_1528719 to 1528736, EPI_ISL_1528738 to 1528739, EPI_ISL_1528743, EPI_ISL_1528756 to 1528759			
see above	QLabs	WVU and Marshall University Combined Genomics Core Facilities	"James Denvir; Peter Perrotta; Peter Stoilov; Ryan Percifield"; Wesley Kimble
EPI_ISL_444612	QML Pathology	Public Health Virology Laboratory	Alyssa Pyke; Amanda De Jong; Andrew Van Den Hurk; Bixing Huang; Carmel Taylor; David Warrilow; Doris Genge; Elisabeth Gamez; Glen Hewitson; Ian Maxwell Mackay; Inga Sultana; Jamie McMahon; Jean Barcelon; Judy Northill; Mitchell Finger; Natalie Simpson; Neelima Nair; Peter Burtonclay; Peter Moore; Sarah Wheatley; Sean Moody; Sonja Hall-Mendelin; Timothy Gardam; and Frederick Moore
EPI_ISL_613457	QUARANTINE CAMP	Hong Kong Department of Health	Alan K.L. Tsang; Dominic N.C. Tsang; Edman T.K. Lam; Peter C.W. Yip; Rickjason C.W. Chan
EPI_ISL_518819	Qadr Hospital, Tangerang, Banten	Biosafety Level-3 Laboratory, Indonesian Institute of Sciences (LIPI)	Ade Andriani; Ahmad Fathoni; Andri Wardiana; Anggia Prasetyoputri; Anik Budhi Dharmayanthi; Ario Betha Juanssilfero; Asep M Ridwanuloh; Isa Nuryana; Puspita Lisdiyanti; Ratih Asmana Ningrum; Rifki Sadikin; Syam Budi Iryanto; Wien Kusharyoto
EPI_ISL_448223 to 448240, EPI_ISL_448242 to 448256, EPI_ISL_448258 to 448307, EPI_ISL_448309 to 448388, EPI_ISL_448978 to 449136, EPI_ISL_449138 to 449175, EPI_ISL_453545 to 453613, EPI_ISL_457326 to 457572, EPI_ISL_461805 to 461893, EPI_ISL_472291 to 472369, EPI_ISL_472371 to 472383, EPI_ISL_478719 to 478720, EPI_ISL_480329 to 480330, EPI_ISL_484328 to 484338, EPI_ISL_490496 to 490559, EPI_ISL_493541 to 493546, EPI_ISL_499479, EPI_ISL_499490, EPI_ISL_499495, EPI_ISL_499562, EPI_ISL_499588, EPI_ISL_499591 to 499592, EPI_ISL_499599, EPI_ISL_499601, EPI_ISL_499603, EPI_ISL_499623, EPI_ISL_499631, EPI_ISL_507130, EPI_ISL_512366 to 512371, EPI_ISL_512373 to 512378, EPI_ISL_529238, EPI_ISL_529240 to 529250, EPI_ISL_529267, EPI_ISL_529273, EPI_ISL_529281 to 529282, EPI_ISL_529286, EPI_ISL_529290, EPI_ISL_529292, EPI_ISL_529294, EPI_ISL_529378 to 529381, EPI_ISL_529388 to 529389, EPI_ISL_529413, EPI_ISL_529415 to 529417, EPI_ISL_529422, EPI_ISL_529437, EPI_ISL_529451 to 529452, EPI_ISL_529457 to 529460, EPI_ISL_529469, EPI_ISL_529476 to 529477, EPI_ISL_529487 to 529489, EPI_ISL_529492, EPI_ISL_529506 to 529507, EPI_ISL_529523, EPI_ISL_529526, EPI_ISL_529557 to 529589, EPI_ISL_540691 to 540698, EPI_ISL_549336, EPI_ISL_549344, EPI_ISL_549349 to 549351, EPI_ISL_549353, EPI_ISL_549355, EPI_ISL_549359, EPI_ISL_549367 to 549370, EPI_ISL_549373, EPI_ISL_549377 to 549379, EPI_ISL_549382 to 549384, EPI_ISL_549477 to 549490, EPI_ISL_572399 to 572401, EPI_ISL_572403, EPI_ISL_572406, EPI_ISL_572419, EPI_ISL_572423, EPI_ISL_572430, EPI_ISL_572437, EPI_ISL_572440 to 572441, EPI_ISL_572447 to 572449, EPI_ISL_572453 to 572454, EPI_ISL_572456, EPI_ISL_572459, EPI_ISL_572462 to 572463, EPI_ISL_572485 to 572486, EPI_ISL_572492 to 572493, EPI_ISL_572495, EPI_ISL_572497 to 572499, EPI_ISL_572503 to 572505, EPI_ISL_572520, EPI_ISL_572526, EPI_ISL_572533, EPI_ISL_572546, EPI_ISL_572548 to 572551, EPI_ISL_572553, EPI_ISL_572556, EPI_ISL_572563, EPI_ISL_572569, EPI_ISL_572583, EPI_ISL_572591, EPI_ISL_572600, EPI_ISL_572602, EPI_ISL_572607, EPI_ISL_572615, EPI_ISL_572646, EPI_ISL_572649, EPI_ISL_572651, EPI_ISL_572653, EPI_ISL_572655 to 572656, EPI_ISL_572658, EPI_ISL_572660, EPI_ISL_572665 to 572666, EPI_ISL_572670 to 572671, EPI_ISL_572673, EPI_ISL_572711, EPI_ISL_572717 to 572720, EPI_ISL_572730, EPI_ISL_572732, EPI_ISL_572735, EPI_ISL_572742, EPI_ISL_572745, EPI_ISL_572749, EPI_ISL_572755, EPI_ISL_572758 to 572759, EPI_ISL_572764, EPI_ISL_572768, EPI_ISL_572775, EPI_ISL_572780, EPI_ISL_572785 to 572787, EPI_ISL_572789, EPI_ISL_572791, EPI_ISL_572793, EPI_ISL_572797, EPI_ISL_572804, EPI_ISL_572806, EPI_ISL_572811 to 572812, EPI_ISL_572814, EPI_ISL_572819, EPI_ISL_572823, EPI_ISL_572825 to 572826, EPI_ISL_572831, EPI_ISL_572835, EPI_ISL_572842, EPI_ISL_572844, EPI_ISL_572847, EPI_ISL_572898 to 572901, EPI_ISL_572939 to 572941, EPI_ISL_572954, EPI_ISL_573085 to 573131, EPI_ISL_573414 to 573417, EPI_ISL_577096 to 577109, EPI_ISL_584165 to 584166, EPI_ISL_584716 to 584749, EPI_ISL_584906 to 585008, EPI_ISL_585260, EPI_ISL_585583 to 585619, EPI_ISL_586240 to 586242, EPI_ISL_595115 to 595208, EPI_ISL_595210 to 595231, EPI_ISL_595837 to 596190, EPI_ISL_1386253 to 1386408, EPI_ISL_1386410 to 1386411, EPI_ISL_1386413, EPI_ISL_1386416 to 1386419, EPI_ISL_1386424, EPI_ISL_1386426 to 1386427, EPI_ISL_1386433 to 1386434, EPI_ISL_1386439 to 1386440, EPI_ISL_1386450 to 1386451, EPI_ISL_1386453, EPI_ISL_1386455 to 1386456, EPI_ISL_1386458, EPI_ISL_1386460 to 1386461, EPI_ISL_1386464, EPI_ISL_1386468, EPI_ISL_1386472 to 1386473, EPI_ISL_1386934 to 1387049, EPI_ISL_1387133 to 1387156, EPI_ISL_1474166 to 1474379, EPI_ISL_1474893 to 1474957, EPI_ISL_1476686 to 1476690			
see above	Quadram Institute Bioscience	COVID-19 Genomics UK (COG-UK) Consortium	Alexander J Trotter; Alison E. Mather; Alp Aydin; Ana P. Tedim; Anastasia Kolyva; Andrew Bell; Andrew J. Page; Claire Stuart; Dave J. Baker; Gemma L. Kay; John Wain; Justin O'Grady; Leonardo de Oliveira Martins; Lizzie Meadows; Maria Diaz; Mark Webber; Muhammed Yasir; Nabil-Fareed Alikhan; Ngozi Elumogo; Nicholas M. Thomson; Rachael Stanley; Rachel Gilroy; Reenesh Prakash; Samir Dervisevic; Samuel Bloomfield; Steven Rudder; Thanh Le-Viet
EPI_ISL_487432 to 487436	Queen Astrid Military Hospital	Institute of Tropical Medicine	Colin Anthony; Philippe Selhorst

EPI_ISL_539815	Queen Elizabeth Hospital	Hong Kong Department of Health	Alan K.L. Tsang; Dominic N.C. Tsang; Edman T.K. Lam; Peter C.W. Yip; Rickjason C.W. Chan
EPI_ISL_418814, EPI_ISL_429812 to 429814, EPI_ISL_482469 to 482470, EPI_ISL_482476 to 482477	Queen Elizabeth II Health Science Centre	National Microbiology Laboratory	Anna Majer; Elsie Grudeski; Gary Van Domselaar; Grace Seo; Jason LeBlanc; Jennifer Tanner; Kristyn Burak; Matthew Gilmour; Morag Graham; Natalie Knox; Nathalie Bastien; Philip Mabon; Rhiannon Huzarewich; Russell Mandes; Shari Tyson; Timothy Booth; Todd Hatchette; Yan Li
EPI_ISL_539803, EPI_ISL_539816 to 539817, EPI_ISL_539820	Queen Mary Hospital	Hong Kong Department of Health	Alan K.L. Tsang; Dominic N.C. Tsang; Edman T.K. Lam; Peter C.W. Yip; Rickjason C.W. Chan
EPI_ISL_425462 to 425549, EPI_ISL_425570 to 425577, EPI_ISL_425579 to 425646, EPI_ISL_432668 to 432709, EPI_ISL_432900 to 433067, EPI_ISL_441437 to 441546, EPI_ISL_441847 to 441897, EPI_ISL_448389 to 448448, EPI_ISL_453614 to 453664, EPI_ISL_457573 to 457580, EPI_ISL_461894 to 461969, EPI_ISL_472384 to 472431, EPI_ISL_478721 to 478728, EPI_ISL_484339 to 484353, EPI_ISL_484374 to 484375, EPI_ISL_484390 to 484391, EPI_ISL_490560 to 490562, EPI_ISL_493547 to 493548, EPI_ISL_493601 to 493602, EPI_ISL_493606 to 493609, EPI_ISL_499266 to 499269, EPI_ISL_499808 to 499809, EPI_ISL_512379 to 512383, EPI_ISL_514452 to 514454, EPI_ISL_526337 to 526366, EPI_ISL_526395 to 526430, EPI_ISL_529264, EPI_ISL_529266, EPI_ISL_529276, EPI_ISL_529279, EPI_ISL_529288, EPI_ISL_529306 to 529311, EPI_ISL_529383, EPI_ISL_529385, EPI_ISL_529387, EPI_ISL_529410 to 529412, EPI_ISL_529414, EPI_ISL_529423, EPI_ISL_529436, EPI_ISL_529450, EPI_ISL_529454 to 529456, EPI_ISL_529463 to 529464, EPI_ISL_529467, EPI_ISL_529479, EPI_ISL_529483, EPI_ISL_529486, EPI_ISL_529504, EPI_ISL_529519, EPI_ISL_529528 to 529556, EPI_ISL_529667, EPI_ISL_534756 to 534762, EPI_ISL_535183, EPI_ISL_540640 to 540690, EPI_ISL_540699 to 540718, EPI_ISL_540894 to 540896, EPI_ISL_549334, EPI_ISL_549357, EPI_ISL_549361, EPI_ISL_549376, EPI_ISL_549380, EPI_ISL_549385, EPI_ISL_549491 to 549516, EPI_ISL_572444, EPI_ISL_572460, EPI_ISL_572475 to 572476, EPI_ISL_572509, EPI_ISL_572582, EPI_ISL_572598, EPI_ISL_572603, EPI_ISL_572706, EPI_ISL_572799, EPI_ISL_572833, EPI_ISL_572872, EPI_ISL_573418 to 573436, EPI_ISL_573450 to 573477, EPI_ISL_577110 to 577192, EPI_ISL_584381 to 584439, EPI_ISL_584750 to 584808, EPI_ISL_585627 to 585629, EPI_ISL_594923 to 594986, EPI_ISL_595046 to 595099, EPI_ISL_595232 to 595325, EPI_ISL_611546, EPI_ISL_611582, EPI_ISL_611604, EPI_ISL_611659, EPI_ISL_611662, EPI_ISL_611670, EPI_ISL_611676 to 611677, EPI_ISL_611680, EPI_ISL_611753, EPI_ISL_611791, EPI_ISL_611810, EPI_ISL_611812 to 611813, EPI_ISL_611831, EPI_ISL_611833, EPI_ISL_611840, EPI_ISL_611919, EPI_ISL_611954 to 611956, EPI_ISL_611963, EPI_ISL_612032, EPI_ISL_612077, EPI_ISL_612107, EPI_ISL_612158 to 612159, EPI_ISL_612181 to 612183, EPI_ISL_612192, EPI_ISL_612197, EPI_ISL_612571 to 612606, EPI_ISL_613307 to 613355, EPI_ISL_1386933, EPI_ISL_1387157 to 1387177, EPI_ISL_1387179 to 1387195, EPI_ISL_1475486 to 1475494, EPI_ISL_1546239 to 1546250			
see above	Queens Medical Centre, Clinical Microbiology Department / DeepSeq Nottingham	COVID-19 Genomics UK (COG-UK) Consortium	Christopher Moore; Emily Park; Fei Sang; Gemma Clark; Hannah Howson-Wells; Jack Hill; Jayasree Dey; Joeseph Chappell; Johnny Debebe; Jonathan Ball; Joseph Chappell; Manjinder Khakh; Matthew Carlisle; Matthew Loose; Michelle M Lister; Nadine Holmes; Nichola Duckworth; Patrick McClure; Sarah Walsh; Theocharis Tsoleridis; Tim Sloan; Timothy Byaruhanga; Vicki M Fleming; Victoria Wright; Wendy Smith
EPI_ISL_530225 to 530283	Queensland Health Forensic and Scientific Services, Public Health Virology	Public Health Virology Laboratory, Forensic and Scientific Services, Queensland Health	Son Nguyen et al
EPI_ISL_1424680, EPI_ISL_1424758, EPI_ISL_1424782, EPI_ISL_1424786, EPI_ISL_1424816, EPI_ISL_1424835 to 1424844	Queensland Medical Laboratories	Melbourne Diagnostic Unit Public Health Laboratory (MDU-PHL)	N.L.; Palou, T.; Seemann, T.; Sherry; Vaccher, S.
EPI_ISL_1424519 to 1424635	Queensland Medical Laboratories	Victorian Infectious Diseases Reference Laboratory (VIDRL) and the Melbourne Diagnostic Unit Public Health Laboratory (MDU-PHL)	N.L.; Palou, T.; Seemann, T.; Sherry; Vaccher, S.
EPI_ISL_1520144 to 1520145	Quest Diagnostics	Illinois Department of Public Health - Chicago Lab	Ira Heimler; Vineet K. Dhiman
EPI_ISL_490210 to 490223	Quest Diagnostics	Q Squared Solutions - QRTP facility	Victor J Weigman
EPI_ISL_454691 to 454693, EPI_ISL_467811 to 467927, EPI_ISL_468560 to 468590, EPI_ISL_494504 to 494552, EPI_ISL_498695 to 498747, EPI_ISL_500459, EPI_ISL_571016 to 572185, EPI_ISL_603759 to 605057			
see above	Quest Diagnostics	Quest Diagnostics	Anderson; Anderson, B.; B.P.; D.F.; Gerasimova, A.; Grover, D.; Hua, M.; K.E.; Kagan; Lacbawan, F.; Liu Y.; Livingston; Owen; Owen, R.; R. AND Lacbawan, F.; R.M.; R.M. and Owen, R.; Rosenthal; S.H.; Shalhout
EPI_ISL_1391417 to 1391420, EPI_ISL_1391422 to 1391620, EPI_ISL_1391622 to 1391698, EPI_ISL_1392237, EPI_ISL_1392239 to 1392240, EPI_ISL_1392242, EPI_ISL_1392244, EPI_ISL_1392246, EPI_ISL_1392248, EPI_ISL_1392250, EPI_ISL_1392252, EPI_ISL_1392254 to 1392255, EPI_ISL_1392258, EPI_ISL_1392260, EPI_ISL_1392262, EPI_ISL_1392264, EPI_ISL_1392266, EPI_ISL_1392268, EPI_ISL_1392270, EPI_ISL_1392272, EPI_ISL_1392274, EPI_ISL_1392276, EPI_ISL_1392278, EPI_ISL_1392280, EPI_ISL_1392282, EPI_ISL_1392284, EPI_ISL_1392286, EPI_ISL_1392288, EPI_ISL_1392290, EPI_ISL_1392292, EPI_ISL_1392294, EPI_ISL_1392296, EPI_ISL_1392298, EPI_ISL_1392300, EPI_ISL_1392302, EPI_ISL_1392304 to 1392305, EPI_ISL_1392307, EPI_ISL_1392309, EPI_ISL_1392311, EPI_ISL_1392313, EPI_ISL_1392315, EPI_ISL_1392317, EPI_ISL_1392319, EPI_ISL_1392321, EPI_ISL_1392323, EPI_ISL_1392325, EPI_ISL_1392327, EPI_ISL_1392329, EPI_ISL_1392331, EPI_ISL_1392333, EPI_ISL_1392335 to 1392336, EPI_ISL_1392338, EPI_ISL_1392340, EPI_ISL_1392343, EPI_ISL_1392345, EPI_ISL_1392347, EPI_ISL_1392349, EPI_ISL_1392351, EPI_ISL_1392353, EPI_ISL_1392355, EPI_ISL_1392357, EPI_ISL_1392359, EPI_ISL_1392361, EPI_ISL_1392363, EPI_ISL_1392365, EPI_ISL_1392367 to 1392368, EPI_ISL_1392370, EPI_ISL_1392372, EPI_ISL_1392374, EPI_ISL_1392376, EPI_ISL_1392378, EPI_ISL_1392380, EPI_ISL_1392382, EPI_ISL_1392384, EPI_ISL_1392386, EPI_ISL_1392388, EPI_ISL_1392390, EPI_ISL_1392392, EPI_ISL_1392394, EPI_ISL_1392396, EPI_ISL_1392398, EPI_ISL_1392400, EPI_ISL_1392402, EPI_ISL_1392404, EPI_ISL_1392406, EPI_ISL_1392408 to 1392409, EPI_ISL_1392412, EPI_ISL_1392414, EPI_ISL_1392416 to 1392417, EPI_ISL_1392420, EPI_ISL_1392422, EPI_ISL_1392424, EPI_ISL_1392426, EPI_ISL_1392428, EPI_ISL_1392430 to 1392431, EPI_ISL_1392434, EPI_ISL_1392436, EPI_ISL_1422327 to 1422561, EPI_ISL_1422564, EPI_ISL_1422567, EPI_ISL_1422570, EPI_ISL_1422573, EPI_ISL_1422576, EPI_ISL_1422579, EPI_ISL_1422582, EPI_ISL_1422585, EPI_ISL_1422588, EPI_ISL_1422591 to 1422991, EPI_ISL_1444347 to 1444672, EPI_ISL_1444675, EPI_ISL_1444678, EPI_ISL_1444681, EPI_ISL_1444684, EPI_ISL_1444687, EPI_ISL_1444689 to 1444731, EPI_ISL_1460730 to 1460785, EPI_ISL_1479683 to 1479801, EPI_ISL_1479870 to 1480006, EPI_ISL_1480521 to 1480976, EPI_ISL_1491950 to 1492069, EPI_ISL_1492072, EPI_ISL_1492075, EPI_ISL_1492078, EPI_ISL_1492080, EPI_ISL_1492083, EPI_ISL_1492086, EPI_ISL_1492088, EPI_ISL_1492091, EPI_ISL_1492094, EPI_ISL_1492096, EPI_ISL_1492098, EPI_ISL_1492101, EPI_ISL_1492104, EPI_ISL_1492106, EPI_ISL_1492109, EPI_ISL_1492112, EPI_ISL_1492115, EPI_ISL_1492118, EPI_ISL_1492120, EPI_ISL_1492122, EPI_ISL_1492125, EPI_ISL_1492128, EPI_ISL_1492131, EPI_ISL_1492134, EPI_ISL_1492136, EPI_ISL_1492139, EPI_ISL_1492142, EPI_ISL_1492145, EPI_ISL_1493601 to 1493721, EPI_ISL_1493939 to 1494031, EPI_ISL_1511646 to 1511696, EPI_ISL_1511698 to 1511859, EPI_ISL_1525904 to 1526070, EPI_ISL_1551643 to 1551693, EPI_ISL_1551695 to 1551915, EPI_ISL_1551917 to 1551951, EPI_ISL_1551953 to 1552083, EPI_ISL_1552085 to 1552242, EPI_ISL_1552245, EPI_ISL_1552249, EPI_ISL_1552252, EPI_ISL_1552254 to 1552255, EPI_ISL_1552257, EPI_ISL_1552259 to 1552260, EPI_ISL_1552262, EPI_ISL_1552264 to 1552265, EPI_ISL_1552267, EPI_ISL_1552269 to 1552270, EPI_ISL_1552272 to 1552273, EPI_ISL_1552275, EPI_ISL_1552277 to 1552278, EPI_ISL_1552280, EPI_ISL_1552282 to 1552283, EPI_ISL_1552285 to 1552286, EPI_ISL_1552288, EPI_ISL_1552290 to 1552291, EPI_ISL_1552293, EPI_ISL_1552295 to 1552296, EPI_ISL_1552298 to 1552299, EPI_ISL_1552301, EPI_ISL_1552304 to 1552305, EPI_ISL_1552307 to 1552308, EPI_ISL_1552310 to 1552311, EPI_ISL_1552313 to 1552314, EPI_ISL_1552321, EPI_ISL_1552324, EPI_ISL_1552327, EPI_ISL_1552330, EPI_ISL_1552333 to 1552334, EPI_ISL_1552336 to 1552337, EPI_ISL_1552339, EPI_ISL_1552341 to 1552342, EPI_ISL_1552344, EPI_ISL_1552346 to 1552347, EPI_ISL_1552349 to 1552351, EPI_ISL_1552353, EPI_ISL_1552355 to 1552356, EPI_ISL_1552358 to 1552359, EPI_ISL_1552361 to 1552362, EPI_ISL_1552364 to 1552365, EPI_ISL_1552367 to 1552368, EPI_ISL_1552370 to 1552371, EPI_ISL_1552373, EPI_ISL_1552375 to 1552376, EPI_ISL_1552378 to 1552379, EPI_ISL_1552382, EPI_ISL_1552385, EPI_ISL_1552388, EPI_ISL_1552390, EPI_ISL_1552392, EPI_ISL_1552394 to 1552395, EPI_ISL_1552397 to 1552398, EPI_ISL_1552400, EPI_ISL_1552402 to 1552403, EPI_ISL_1552405 to 1552406, EPI_ISL_1552408 to 1552409, EPI_ISL_1552411 to 1552412, EPI_ISL_1552414 to 1552415, EPI_ISL_1552417 to 1552418, EPI_ISL_1552420 to 1552421, EPI_ISL_1552423 to 1552424, EPI_ISL_1552426, EPI_ISL_1552428 to 1552429, EPI_ISL_1552431 to 1552432, EPI_ISL_1552434, EPI_ISL_1552436 to 1552437, EPI_ISL_1552439 to 1552440, EPI_ISL_1552442 to 1552443, EPI_ISL_1552445 to 1552446, EPI_ISL_1552449, EPI_ISL_1552452, EPI_ISL_1552455, EPI_ISL_1552458, EPI_ISL_1552460, EPI_ISL_1552463, EPI_ISL_1552466, EPI_ISL_1552469, EPI_ISL_1552473, EPI_ISL_1552476, EPI_ISL_1552479, EPI_ISL_1552482, EPI_ISL_1552484, EPI_ISL_1552487, EPI_ISL_1552490, EPI_ISL_1552493, EPI_ISL_1552496, EPI_ISL_1552499, EPI_ISL_1552502, EPI_ISL_1552505, EPI_ISL_1552508, EPI_ISL_1552511, EPI_ISL_1552514, EPI_ISL_1552517, EPI_ISL_1552520, EPI_ISL_1552523, EPI_ISL_1552526, EPI_ISL_1552529, EPI_ISL_1552532, EPI_ISL_1552535, EPI_ISL_1552539, EPI_ISL_1552542, EPI_ISL_1552544, EPI_ISL_1552547, EPI_ISL_1552550, EPI_ISL_1552553, EPI_ISL_1552556, EPI_ISL_1552559, EPI_ISL_1552562, EPI_ISL_1552565, EPI_ISL_1552568, EPI_ISL_1552571, EPI_ISL_1552574, EPI_ISL_1552577, EPI_ISL_1552580, EPI_ISL_1552583, EPI_ISL_1552586, EPI_ISL_1552589, EPI_ISL_1552592, EPI_ISL_1552594, EPI_ISL_1552597, EPI_ISL_1552600, EPI_ISL_1552602, EPI_ISL_1552605, EPI_ISL_1552608, EPI_ISL_1552611, EPI_ISL_1552614, EPI_ISL_1552617, EPI_ISL_1552620, EPI_ISL_1552623, EPI_ISL_1552626, EPI_ISL_1552629, EPI_ISL_1552632, EPI_ISL_1552635, EPI_ISL_1552639, EPI_ISL_1552642, EPI_ISL_1552645, EPI_ISL_1552648, EPI_ISL_1552651, EPI_ISL_1552654, EPI_ISL_1552657, EPI_ISL_1552660, EPI_ISL_1552663, EPI_ISL_1552665, EPI_ISL_1552668, EPI_ISL_1552671, EPI_ISL_1552674, EPI_ISL_1552677, EPI_ISL_1552680, EPI_ISL_1552683, EPI_ISL_1552685 to 1552812, EPI_ISL_1552814 to 1552923, EPI_ISL_1552925 to 1553037, EPI_ISL_1553039 to 1553052, EPI_ISL_1553054 to 1553172, EPI_ISL_1559411 to 1559434, EPI_ISL_1559436 to 1559451, EPI_ISL_1559453 to 1559516, EPI_ISL_1559518 to 1559529, EPI_ISL_1559531 to 1559540, EPI_ISL_1559542 to 1559543, EPI_ISL_1559545 to 1559548, EPI_ISL_1559550 to 1559551, EPI_ISL_1559553 to 1559556, EPI_ISL_1559558 to 1559587			
see above	Quest Diagnostics Incorporated	Centers for Disease Control and Prevention Division of Viral Diseases, Pathogen Discovery	A. Gerasimova; A. Perez; Adrian Paskey; B. Anderson; Ben L. Rambo-Martin; Benjamin Rambo-Martin; Christopher Gulvick; Clinton R. Paden; Dakota Howard; Darlene Wagner; Dhwani Batra; Duncan MacCannell; F. Lacbawan; I. A. Shlyakhter; Jason Caravas; K.E. Livingston; Kara Moser; L.E. Bernstein; M. Hua; Matthew Schmerer; P. Tanpaiboon; Peter W. Cook; R. M. Kagan; R. Owen; R. V. Rolando; S. H. Rosenthal; Scott Sammons; Shatavia Morrison; Suxiang Tong; Y. Liu; Yvette Unoarumhi
EPI_ISL_569615	Quick Care Huron	South Dakota Public Health Laboratory	Jacob Garfin; Matt Plumb; Xiong Wang; and Chris Carlson
EPI_ISL_569621, EPI_ISL_569623	Quick Care Watertown	South Dakota Public Health Laboratory	Jacob Garfin; Matt Plumb; Xiong Wang; and Chris Carlson
EPI_ISL_415641 to 415644, EPI_ISL_416477 to 416482	R. G. Lugar Center for Public Health Research, National Center for Disease Control and Public Health (NCDC) of Georgia.	R. G. Lugar Center for Public Health Research, National Center for Disease Control and Public Health (NCDC) of Georgia.	Adam Kotorashvili; Amiran Gamkrelidze.; Ana Papkiauri; Ann Machablishvili; Anna Kasradze; Davit Tsaguria; Ekaterine Khmaladze; Ekaterine Zangaladze; Ekaterine Zhghenti; Giorgi Tomashvili; Gvantsa Brachveli; Gvantsa Chanturia; Irma Burjanadze; Ketevan Sidamonidze; Khatuna Zakhashvili; Lela Sabadze; Lela Urushadze; Magda Dgebuadze; Maia Alkhazashvili; Mari Gavashelidze; Mariam Zakalashvili; Marine Murtskhvaladze; Meri Pantsulaia; Nato Kotaria; Nino Berishvili; Paata Imnadze; Roena Sukhiashvili; Tamar Jashiashvili; Tata Imnadze; Tea Tevdoradze
EPI_ISL_1393640 to 1393642, EPI_ISL_1538757, EPI_ISL_1539030	RCKiK	1. National Institute of Public Health - National Institute of Hygiene; 2. Eurofins Genomics Europe Sequencing GmbH	ECDC COVID-19 WGS support team; Eurofins Genomics Europe Sequencing Team; Gierczyki Rafa; Sadkowska-Todys Magorzata; Wokowicz Tomasz; Zacharczuk Katarzyna
EPI_ISL_455308 to 455311, EPI_ISL_455478	REGIONAL VRDL,ICMR-RMRC BBSR	Immunogenomics group, Institute of Life Sciences, Bhubaneswar	Ajay Parida; Arup Ghosh; Atimukta Jha; COVID-19 team of ILS & RMRC; DBT's PAN-INDIA 1000 SARS-CoV2 RNA genome sequencing consortium; Debdutta Bhattacharya; Ghulam Hussain Syed; Jaya Singh Khastri; Jyotirmayee Turuk; Manasi Priyadarshini; Orissa COVID-19 study group; Punit Prasad; Rajeeb Swain; Rupesh Dash; Sanghamitra Pati; Shanti Senapati; Shuchi Smita; Soma Chattopadhyay; Sunil Raghav; Swati Madhulika; Tushar K. Beuria; Viplov K. Biswas
EPI_ISL_455749 to 455788	REGIONAL VRDL,ICMR-RMRC BBSR	Immunogenomics lab, Institute of Life Sciences, Bhubaneswar	Ajay Parida; Arup Ghosh; Atimukta Jha; COVID-19 team of ILS & RMRC; DBT's PAN-INDIA 1000 SARS-CoV2 RNA genome sequencing consortium; Debdutta Bhattacharya; Ghulam Hussain Syed; Jaya Singh Khastri; Jyotirmayee Turuk; Manasi Priyadarshini; Orissa COVID-19 study group; Punit Prasad; Rajeeb Swain; Rupesh Dash; Sanghamitra Pati; Shanti Senapati; Shuchi Smita; Soma Chattopadhyay; Sunil Raghav; Swati Madhulika; Tushar K. Beuria; Viplov K. Biswas
EPI_ISL_1446572 to 1446575, EPI_ISL_1516377 to 1516387, EPI_ISL_1528462 to 1528474	RI State Health Laboratories	Centers for Disease Control and Prevention Division of Viral Diseases, Pathogen Discovery	Alison Laufer Halpin; Ben L. Rambo-Martin; Clinton R. Paden; Dakota Howard; Darlene Wagner; Dave Wentworth; Dhwani Batra; Jasmine Padilla; Justin Lee; Katie Dillon; Krista Queen; Kristen Knipe; Kristine Lacek; Mark Burroughs; Matthew Schmerer; Mili Sheth; Peter Cook; Sam Shepard; Sarah Nobles; Shoshona Le; Suxiang Tong; Vivien Dugan; Yvette Unoarumhi

EPI_ISL_419553, EPI_ISL_424887, EPI_ISL_426435, EPI_ISL_535359 to 535360	RI State Health Laboratories	Pathogen Discovery, Respiratory Viruses Branch, Division of Viral Diseases, Centers for Disease Control and Prevention	Alison S. Laufer Halpin; Anna Uehara; Christopher A. Elkins; Clinton Paden; Clinton R. Paden; Haibin Wang; Jasmine Padilla; Jing Zhang; Justin Lee; Krista Queen; Mary S. Keckler; Rachel Marine; Suxiang Tong; Yan Li; Ying Tao
EPI_ISL_420795	RI State Health Laboratory	Pathogen Discovery, Respiratory Viruses Branch, Division of Viral Diseases, Centers for Disease Control and Prevention	Alison S. Laufer Halpin; Anne Uehara; Christopher A. Elkins; Clinton R. Paden; Haibin Wang; Jasmine Padilla; Jing Zhang; Justin Lee; Krista Queen; Mary S. Keckler; Rachel Marine; Suxiang Tong; Yan Li; Ying Tao
EPI_ISL_413569	RIVM	Erasmus Medical Center	Anne van der Linden; Annemiek van der Eijk; Aura Timen; Bas Oude Munnink; Claudia Schapendonk; Corien Swaan; Corine GeurtsvanKessel; David Nieuwenhuijse; Irina Chestakova; Jeroen van Kampen; Jolanda Voermans; Madelief Mollers; Manon Haverkate; Marion Koopmans; Mark Pronk; Mart Stein; Pascal Lexmond; Reina Sikkema; Richard Molenkamp; Sandra Kengne Kamga Mobou; on behalf of the Dutch national COVID-19 response team.
EPI_ISL_568693, EPI_ISL_574603	RS Freeport Tembagapura	Eijkman Institute for Molecular Biology, Ministry of Research and Technology/National Agency for Research and Innovation	Amin Soebandrio; David H Muljono; Edison Johar; Frilasita A Yudhaputri; Herawati Sudoyo; Hidayat Trimarsanto; Iskandar A Adnan; Khin Saw Myint; Safarina G Malik; Willy Agustine
EPI_ISL_574613, EPI_ISL_574615	RS Harapan Bunda	Eijkman Institute for Molecular Biology, Ministry of Research and Technology/National Agency for Research and Innovation	Amin Soebandrio; David H Muljono; Edison Johar; Frilasita A Yudhaputri; Herawati Sudoyo; Hidayat Trimarsanto; Iskandar A Adnan; Khin Saw Myint; Safarina G Malik; Willy Agustine
EPI_ISL_574612	RS Hermina Mekarsari	Eijkman Institute for Molecular Biology, Ministry of Research and Technology/National Agency for Research and Innovation	Amin Soebandrio; David H Muljono; Edison Johar; Frilasita A Yudhaputri; Herawati Sudoyo; Hidayat Trimarsanto; Iskandar A Adnan; Khin Saw Myint; Safarina G Malik; Willy Agustine
EPI_ISL_574607	RS Husada	Eijkman Institute for Molecular Biology, Ministry of Research and Technology/National Agency for Research and Innovation	Amin Soebandrio; David H Muljono; Edison Johar; Frilasita A Yudhaputri; Herawati Sudoyo; Hidayat Trimarsanto; Iskandar A Adnan; Khin Saw Myint; Safarina G Malik; Willy Agustine
EPI_ISL_1469247, EPI_ISL_1469256 to 1469257, EPI_ISL_1469285 to 1469286	RS Khusus Paru Karawang	National Institute of Health Research and Development	Arie Ardiansyah Nugraha; Hana Apsari Pawestri; Hartanti Dian Ikawati; Kartika Dewi Puspa; Krisna Pangesti; Nelly Puspandari; Subangkit; Vivi Setiawaty
EPI_ISL_568692, EPI_ISL_574614, EPI_ISL_574616 to 574618	RS Kramat 128	Eijkman Institute for Molecular Biology, Ministry of Research and Technology/National Agency for Research and Innovation	Amin Soebandrio; David H Muljono; Edison Johar; Frilasita A Yudhaputri; Herawati Sudoyo; Hidayat Trimarsanto; Iskandar A Adnan; Khin Saw Myint; Safarina G Malik; Willy Agustine
EPI_ISL_538499	RS Lavallete Malang East Java	National Institute of Health Research and Development	AA; HA; HD; Ikawati; KD; KNA; Nugraha; Paisal; Pangesti; Pawestri; Puspa; Setiawaty, V.; Soekarso; Subangkit; T
EPI_ISL_1469254	RS Lira Medika Karawang	National Institute of Health Research and Development	Arie Ardiansyah Nugraha; Hana Apsari Pawestri; Hartanti Dian Ikawati; Kartika Dewi Puspa; Krisna Pangesti; Nelly Puspandari; Subangkit; Vivi Setiawaty
EPI_ISL_1469248, EPI_ISL_1469253, EPI_ISL_1469287	RS Mandaya Karawang	National Institute of Health Research and Development	Arie Ardiansyah Nugraha; Hana Apsari Pawestri; Hartanti Dian Ikawati; Kartika Dewi Puspa; Krisna Pangesti; Nelly Puspandari; Subangkit; Vivi Setiawaty
EPI_ISL_574608	RS Mitra Keluarga Kelapa Gading	Eijkman Institute for Molecular Biology, Ministry of Research and Technology/National Agency for Research and Innovation	Amin Soebandrio; David H Muljono; Edison Johar; Frilasita A Yudhaputri; Herawati Sudoyo; Hidayat Trimarsanto; Iskandar A Adnan; Khin Saw Myint; Safarina G Malik; Willy Agustine
EPI_ISL_610158	RS Panti Rini	Genetics Working Group (Pokja Genetik) Faculty of Medicine, Public Health and Nursing Universitas Gadjah Mada (FK-KMK UGM); Disease Investigation Center Wates Ministry of Agriculture Indonesia; Department of Microbiology FK-KMK UGM; Laboratorium Diagnostik Yayasan Tahija World Mosquito Program (WMP) Yogyakarta Center for Tropical Medicine FK-KMK UGM; Integrated Research Center FK-KMK UGM; Department of Computer Science and Electronics FMIPA UGM	Afiahayati; Dwi AA Nugrahaningsih; Dwiki Afandy; Dyah Ayu Puspitarani; Edwin W. Daniwijaya; Eggi Arguni; Endah Supriyati; Gunadi; Hendra Wibawa; Kristy Iskandar; Ludhang P. Rizki; Marcellus; Mohamad S. Hakim; Nungki Anggorowati; Siswanto; Titik Nuryastuti; Tri Wibawa; V. Noegroho Isti Donodjati; Wawan Triharjanto
EPI_ISL_568688	RS Pelni	Eijkman Institute for Molecular Biology, Ministry of Research and Technology/National Agency for Research and Innovation	Amin Soebandrio; David H Muljono; Edison Johar; Frilasita A Yudhaputri; Herawati Sudoyo; Hidayat Trimarsanto; Iskandar A Adnan; Khin Saw Myint; Safarina G Malik; Willy Agustine
EPI_ISL_435282 to 435283, EPI_ISL_437190 to 437191	RS Pondok Indah Hospital - Pondok Indah	Eijkman Institute for Molecular Biology, Ministry of Research and Technology/National Agency for Research and Innovation	Amin Soebandrio; David H Muljono; Edison Johar; Frilasita A Yudhaputri; Hidayat Trimarsanto; Khin Saw Myint; Safarina G Malik
EPI_ISL_568689	RS Pondok Indah Puri Indah	Eijkman Institute for Molecular Biology, Ministry of Research and Technology/National Agency for Research and Innovation	Amin Soebandrio; David H Muljono; Edison Johar; Frilasita A Yudhaputri; Herawati Sudoyo; Hidayat Trimarsanto; Iskandar A Adnan; Khin Saw Myint; Safarina G Malik; Willy Agustine
EPI_ISL_574611	RS Premier Bintaro	Eijkman Institute for Molecular Biology, Ministry of Research and Technology/National Agency for Research and Innovation	Amin Soebandrio; David H Muljono; Edison Johar; Frilasita A Yudhaputri; Herawati Sudoyo; Hidayat Trimarsanto; Iskandar A Adnan; Khin Saw Myint; Safarina G Malik; Willy Agustine
EPI_ISL_538500 to 538501	RS Prima Husada Cipta Medan North Sumatra	National Institute of Health Research and Development	AA; HA; HD; Ikawati; KD; KNA; Nugraha; Paisal; Pangesti; Pawestri; Puspa; Setiawaty, V.; Soekarso; Subangkit; T
EPI_ISL_574606	RS Siloam Purwakarta	Eijkman Institute for Molecular Biology, Ministry of Research and Technology/National Agency for Research and Innovation	Amin Soebandrio; David H Muljono; Edison Johar; Frilasita A Yudhaputri; Herawati Sudoyo; Hidayat Trimarsanto; Iskandar A Adnan; Khin Saw Myint; Safarina G Malik; Willy Agustine
EPI_ISL_1392663 to 1392665, EPI_ISL_1502460, EPI_ISL_1502498	RSA GIULIANOVA GIULIANOVA(TERAMO)	Istituto Zooprofilattico Sperimentale dell'Abruzzo e Molise "G. Caporale"	Ancora M; Calistri P; Cammà C; Caporale M; Curini V; Delli Compagni E; Di Domenico M; Di Lollo Valeria; Di Pasquale A; Lorusso A; Mangone I; Marcacci M; Puglia I; Rinaldi A; Savini G; Scialabba S
EPI_ISL_529017	RSA San Raffaele Sulmona	Istitituto Zooprofilattico Sperimentale dell'Abruzzo e Molise "G.Caporale"	Ancora M; Cammà C; Curini V; Di Domenico M; Di Pasquale A; Lorusso A; Mangone I; Marcacci M; Puglia I; Rinaldi A; Savini G.
EPI_ISL_576145	RSA Universitas Gadjah Mada	Genetics Working Group (Pokja Genetik) Faculty of Medicine, Public Health and Nursing Universitas Gadjah Mada (FK-KMK UGM); Disease Investigation Center Wates Ministry of Agriculture Indonesia; Department of Microbiology FK-KMK UGM; Laboratorium Diagnostik Yayasan Tahija World Mosquito Program (WMP) Yogyakarta Center for Tropical Medicine FK-KMK UGM; Integrated Research Center FK-KMK UGM; Department of Computer Science and Electronics FMIPA UGM	Afiahayati; Dwi AA Nugrahaningsih; Edwin W. Daniwijaya; Eggi Arguni; Endah Supriyati; Fadil Fahri; Gunadi; Hendra Wibawa; Kristy Iskandar; Ludhang P. Rizki; Marcellus; Mohamad S. Hakim; Nungki Anggorowati; Siswanto; Titik Nuryastuti; Tri Wibawa; William Widitjiarso
EPI_ISL_529019	RSA/RP Villa San Giovanni - Gruppo Edos	Istitituto Zooprofilattico Sperimentale dell'Abruzzo e Molise "G.Caporale"	Ancora M; Cammà C; Curini V; Di Domenico M; Di Pasquale A; Lorusso A; Mangone I; Marcacci M; Puglia I; Rinaldi A; Savini G.
EPI_ISL_436725	RSA/RP Villa San Giovanni - Gruppo Edos	Istituto Zooprofilattico Sperimentale dell'Abruzzo e Molise "G.Caporale"	Ancora M; Cammà C; Curini V; Di Domenico M; Di Pasquale A; Lorusso A; Mangone I; Marcacci M; Puglia I; Rinaldi A; Savini G
EPI_ISL_529138	RSAL Dr. Ramelan Surabaya	Institute of Tropical Disease, Universitas Airlangga	Aldise M Nastri; Gatot Soegiarto; Jezzy R Dewantari; Kazufumi Shimizu; Krisnoadi Rahardjo; Laksmi Wulandari; Maria I Lusida; Mitsuhiro Nishimura; Radito Soesanto; Resti Yudhawati; Retno A Setyoningrum; Rima R Prasetya; Soetjipto; Yasuko Mori; Yohko K Shimizu
EPI_ISL_454497 to 454520	RSE "National Center for Biotechnology"	RSE "National Center for Biotechnology"	Akbota Rakhmetova; Alexandr Shevtsov; Askar Abdaliyev; Asylulan Amirgazin; Ilyas Akhmetollayev; Ruslan Kalendar; Viktoriya Lutsay; Yerlan Ramankulov; Zabira Aushakhmetova
EPI_ISL_574609	RSJPD Harapan Kita	Eijkman Institute for Molecular Biology, Ministry of Research and Technology/National Agency for Research and Innovation	Amin Soebandrio; David H Muljono; Edison Johar; Frilasita A Yudhaputri; Herawati Sudoyo; Hidayat Trimarsanto; Iskandar A Adnan; Khin Saw Myint; Safarina G Malik; Willy Agustine
EPI_ISL_458081, EPI_ISL_529964	RSUD Bangil Pasuruan	Institute of Tropical Disease, Universitas Airlangga	Aldise M Nastri; Arma Roosalina; Gatot Soegiarto; Jezzy R Dewantari; Kazufumi Shimizu; Krisnoadi Rahardjo; Laksmi Wulandari; Maria I Lusida; Mitsuhiro Nishimura; Resti Yudhawati; Retno A Setyoningrum; Rima R Prasetya; Soetjipto; Yasuko Mori; Yohko K Shimizu
EPI_ISL_576383	RSUD Budi Rahayu Kota Magelang	Genetics Working Group (Pokja Genetik) Faculty of Medicine, Public Health and Nursing Universitas Gadjah Mada (FK-KMK UGM); Disease Investigation Center Wates Ministry of	Aditya Rifqi Fauzi; Afiahayati; Ari Meliyanti; Audric Kenny Tedja; Dwi AA Nugrahaningsih; Edwin W. Daniwijaya; Eggi Arguni; Endah Supriyati; Gunadi; Havid Setyawan; Hendra Wibawa; Indaryati; Irene; Kristy Iskandar; Ludhang P. Rizki; Marcellus; Merliana Sari Situmeang; Mohamad S. Hakim; Nungki Anggorowati; Siswanto; Titik Nuryastuti; Tri Wibawa

		Agriculture Indonesia; Department of Microbiology FK-KMK UGM; Laboratorium Diagnostik Yayasan Tahija World Mosquito Program (WMP) Yogyakarta Center for Tropical Medicine FK-KMK UGM; Integrated Research Center FK-KMK UGM; Department of Computer Science and Electronics FMIPA UGM; Balai Besar Teknik Kesehatan Lingkungan dan Pengendalian Penyakit (BBTKLPP) Yogyakarta	
EPI_ISL_437188, EPI_ISL_529965, EPI_ISL_560991	RSUD Dr. Soetomo	Institute of Tropical Disease, Universitas Airlangga	Aldise M Nastri; Gatot Soegiarto; Jezzy R Dewantari; Joni Wahyuhadi; Kazufumi Shimizu; Krisnoadi Rahardjo; Laksmi Wulandari; Maria I Lusida; Mitsuhiro Nishimura; Resti Y Meliana; Resti Yudhawati; Retno A Setyoningrum; Rima R Prasetya; Soetjipto; Yasuko Mori; Yohko K Shimizu
EPI_ISL_610161 to 610162	RSUD Dr. Tjitrowardojo	Genetics Working Group (Pokja Genetik) Faculty of Medicine, Public Health and Nursing Universitas Gadjah Mada (FK-KMK UGM); Disease Investigation Center Wates Ministry of Agriculture Indonesia; Department of Microbiology FK-KMK UGM; Laboratorium Diagnostik Yayasan Tahija World Mosquito Program (WMP) Yogyakarta Center for Tropical Medicine FK-KMK UGM; Integrated Research Center FK-KMK UGM; Department of Computer Science and Electronics FMIPA UGM; Balai Besar Teknik Kesehatan Lingkungan dan Pengendalian Penyakit (BBTKLPP) Yogyakarta	Afiahayati; Alvin Santoso Kalim; Desyifa Mursalin; Dwi AA Nugrahaningsih; Edwin W. Daniwijaya; Eggi Arguni; Endah Supriyati; Gunadi; Havid Setyawan; Hendra Wibawa; Indaryati; Irene; Kemala Athollah; Kristy Iskandar; Ludhang P. Rizki; Marcellus; Mohamad S. Hakim; Nungki Anggorowati; Siswanto; Susan Simanjaya; Titik Nuryastuti; Tri Wibawa; Wuryanto
EPI_ISL_516829	RSUD Nyi Ageng Serang	Genetics Working Group (Pokja Genetik) Faculty of Medicine, Public Health and Nursing Universitas Gadjah Mada (FK-KMK UGM), Disease Investigation Center Wates Ministry of Agriculture Indonesia, Department of Microbiology FK-KMK UGM, Laboratorium Diagnostik Yayasan Tahija World Mosquito Program (WMP) Yogyakarta Center for Tropical Medicine FK-KMK UGM, Integrated Research Center FK-KMK UGM, Department of Computer Science and Electronics FMIPA UGM	. Marcellus; . Siswanto; Afiahayati; Beby Dewi Sartika; Dwi AA Nugrahaningsih; Dyah Ayu Puspitarani; Edwin W. Daniwijaya; Eggi Arguni; Endah Supriyati; Gunadi; Hendra Wibawa; Ludhang P. Rizki; Mohamad S. Hakim; Titik Nuryastuti; Tri Wibawa
EPI_ISL_568686	RSUD Prof. DR. Margono Soekarjo	Eijkman Institute for Molecular Biology, Ministry of Research and Technology/National Agency for Research and Innovation	Amin Soebandrio; David H Muljono; Edison Johar; Frilasita A Yudhaputri; Herawati Sudoyo; Hidayat Trimarsanto; Iskandar A Adnan; Khin Saw Myint; Safarina G Malik; Willy Agustine
EPI_ISL_529966 to 529967	RSUD Sidoarjo	Institute of Tropical Disease, Universitas Airlangga	Aldise M Nastri; Atok Irawan; Gatot Soegiarto; Jezzy R Dewantari; Kazufumi Shimizu; Krisnoadi Rahardjo; Laksmi Wulandari; Maria I Lusida; Mitsuhiro Nishimura; Resti Yudhawati; Retno A Setyoningrum; Rima R Prasetya; Soetjipto; Yasuko Mori; Yohko K Shimizu
EPI_ISL_538498	RSUD Soediono Madiun East Java	National Institute of Health Research and Development	AA; HA; HD; Ikawati; KD; KNA; Nugraha; Paisal; Pangesti; Pawestri; Puspa; Setiawaty, V.; Soekarso; Subangkit; T
EPI_ISL_538502	RSUD Sultan Imanudin Pangkalan Bun Center Kalimantan	National Institute of Health Research and Development	AA; HA; HD; Ikawati; KD; KNA; Nugraha; Paisal; Pangesti; Pawestri; Puspa; Setiawaty, V.; Soekarso; Subangkit; T
EPI_ISL_1469279 to 1469283	RSUD Surakarta	National Institute of Health Research and Development	Arie Ardiansyah Nugraha; Hana Apsari Pawestri; Hartanti Dian Ikawati; Kartika Dewi Puspa; Krisna Pangesti; Nelly Puspandari; Subangkit; Vivi Setiawaty
EPI_ISL_538510	RSUD Ulin Banjarmasin South Kalimantan	National Institute of Health Research and Development	AA; HA; HD; Ikawati; KD; KNA; M; Nugraha; Paisal; Pangesti; Pasaribu; Pawestri; Puspa; Setiawaty, V.; Soekarso; Subangkit; T
EPI_ISL_538511	RSUD Wahidin Sudirohusodo Mojokerto East Java	National Institute of Health Research and Development	AA; HA; HD; Ikawati; KD; KNA; Nugraha; Paisal; Pangesti; Pawestri; Puspa; Setiawaty, V.; Soekarso; Subangkit; T
EPI_ISL_575331	RSUD Wates	Genetics Working Group (Pokja Genetik) Faculty of Medicine, Public Health and Nursing Universitas Gadjah Mada (FK-KMK UGM); Disease Investigation Center Wates Ministry of Agriculture Indonesia; Department of Microbiology FK-KMK UGM; Laboratorium Diagnostik Yayasan Tahija World Mosquito Program (WMP) Yogyakarta Center for Tropical Medicine FK-KMK UGM; Integrated Research Center FK-KMK UGM; Department of Computer Science and Electronics FMIPA UGM	. Marcellus; Afiahayati; Alvin S. Kalim; Bastianto Kusumajaya; Dwi AA Nugrahaningsih; Edwin W. Daniwijaya; Eggi Arguni; Endah Supriyati; Gunadi; Hendra Wibawa; Kristy Iskandar; Ludhang P. Rizki; Mohamad S. Hakim; Nungki Anggorowati; Siswanto; Susan Simanjaya; Titik Nuryastuti; Tri Wibawa; Zumrati Ahmad
EPI_ISL_576115	RSUP Dr Sardjito	Genetics Working Group (Pokja Genetik) Faculty of Medicine, Public Health and Nursing Universitas Gadjah Mada (FK-KMK UGM); Disease Investigation Center Wates Ministry of Agriculture Indonesia; Department of Microbiology FK-KMK UGM; Laboratorium Diagnostik Yayasan Tahija World Mosquito Program (WMP) Yogyakarta Center for Tropical Medicine FK-KMK UGM; Integrated Research Center FK-KMK UGM; Department of Computer Science and Electronics FMIPA UGM	. Marcellus; Afiahayati; Dwi AA Nugrahaningsih; Edwin W. Daniwijaya; Eggi Arguni; Endah Supriyati; Gunadi; Hendra Wibawa; Ika Trisnawati; Ira Puspitawati; Kristy Iskandar; Ludhang P. Rizki; Maria Patricia Inggriani; Mohamad S. Hakim; Nungki Anggorowati; Nur Imma Fatimah Harahap; Osman Sianipar; Riat El Khair; Siswanto; Titik Nuryastuti; Tri Wibawa; Umi Solekhah Intansari; Untung Wirawan; Yunika Puspadewi; Elizabeth Henny Herningtiyas
EPI_ISL_576113 to 576114, EPI_ISL_576116, EPI_ISL_576128, EPI_ISL_576130	RSUP Dr. Sardjito	Genetics Working Group (Pokja Genetik) Faculty of Medicine, Public Health and Nursing Universitas Gadjah Mada (FK-KMK UGM); Disease Investigation Center Wates Ministry of Agriculture Indonesia; Department of Microbiology FK-KMK UGM; Laboratorium Diagnostik Yayasan Tahija World Mosquito Program (WMP) Yogyakarta Center for Tropical Medicine FK-KMK UGM; Integrated Research Center FK-KMK UGM; Department of Computer Science and Electronics FMIPA UGM	. Marcellus; Aditya Rifqi Fauzi; Afiahayati; Alvin S. Kalim; Alvin Santoso Kalim; Amalia Setyati; Audric Kenny Tedja; Bambang Sigit Riyanto; Cahya Dewi Satria; Desyifa Mursalin; Dwi AA Nugrahaningsih; Dwikisworo Setyowireni; Dyah Ayu Puspitarani; Edwin W. Daniwijaya; Eggi Arguni; Eko Budiono; Elisabeth Siti Herini; Endah Supriyati; Fadil Fahri; Gunadi; Hendra Wibawa; Heni Retnowulan; Ida Safitri Laksanawati; Ika Trisnawati; Ira Puspitawati; Kemala Athollah; Kristy Iskandar; Ludhang P. Rizki; Marcellus; Mohamad S. Hakim; Munawar Gani; Nungki Anggorowati; Nur Imma Fatimah Harahap; Nur Rahmi Ananda; Osman Sianipar; Riat El Khair; Rina Triasih; Ririn Enggy Yuliyanti; Satria Maulana; Siswanto; Sumardi; Susan Simanjaya; Titik Nuryastuti; Titis Widowati; Tri Wibawa; Umi Solekhah Intansari; William Widitjiarso; Yunika Puspadewi; Elizabeth Henny Herningtiyas
EPI_ISL_467376, EPI_ISL_568687, EPI_ISL_568690 to 568691	RSUP Fatmawati	Eijkman Institute for Molecular Biology, Ministry of Research and Technology/National Agency for Research and Innovation	Amin Soebandrio; David H Muljono; Edison Johar; Frilasita A Yudhaputri; Herawati Sudoyo; Hidayat Trimarsanto; Iskandar A Adnan; Khin Saw Myint; Safarina G Malik; Willy Agustine
EPI_ISL_467375, EPI_ISL_568694, EPI_ISL_574604	RSUP Prof. Dr. R. Kandou Manado	Eijkman Institute for Molecular Biology, Ministry of Research and Technology/National Agency for Research and Innovation	Amin Soebandrio; David H Muljono; Edison Johar; Frilasita A Yudhaputri; Herawati Sudoyo; Hidayat Trimarsanto; Iskandar A Adnan; Khin Saw Myint; Safarina G Malik; Willy Agustine
EPI_ISL_525492	RSUP dr. SOERADJI TIRTONEGORO	Genetics Working Group (Pokja Genetik) Faculty of Medicine, Public Health and Nursing Universitas Gadjah Mada (FK-KMK UGM), Disease Investigation Center Wates Ministry of Agriculture Indonesia, Department of Microbiology FK-KMK UGM, Laboratorium Diagnostik Yayasan Tahija World Mosquito Program (WMP) Yogyakarta Center for Tropical Medicine FK-KMK UGM, Integrated Research Center FK-KMK UGM, Department of Computer Science and Electronics FMIPA UGM	. Marcellus; . Siswanto; Afiahayati; Alvin S. Kalim; Dwi AA Nugrahaningsih; Dwiki Afandy; Edwin W. Daniwijaya; Eggi Arguni; Endah Supriyati; Gunadi; Hendra Wibawa; Indah Juliana; Kurniyanto; Ludhang P. Rizki; Mohamad S. Hakim; Titik Nuryastuti; Tri Wibawa
EPI_ISL_574605	RSUP dr. Sardjito	Eijkman Institute for Molecular Biology, Ministry of Research and Technology/National Agency for Research and Innovation	Amin Soebandrio; David H Muljono; Edison Johar; Frilasita A Yudhaputri; Herawati Sudoyo; Hidayat Trimarsanto; Iskandar A Adnan; Khin Saw Myint; Safarina G Malik; Willy Agustine

EPI_ISL_429990, EPI_ISL_435473 to 435474, EPI_ISL_445118, EPI_ISL_450190, EPI_ISL_467976, EPI_ISL_467981, EPI_ISL_467984, EPI_ISL_494570

see above	Rady's Childrens Hospital	Andersen lab at Scripps Research	Denise Malicki; Michelle Vanderpool; SEARCH Alliance San Diego; SEARCH Alliance San Diego with Christina Clarke; Teresa Mueller
EPI_ISL_450508 to 450517, EPI_ISL_454420	Rafik Hariri University Hospital	Rafik Hariri University Hospital	Rita Feghali
EPI_ISL_1491573	Ramathibodi Chakri Naruebodindra Hospital	COVID-19 Network Investigations (CONI) Alliance	Angkana Huang; Anthony R. Jones; Arporn Wangwiwatsin; Bhakbhoom Panthan; Chonticha Klungtong; Duangkamon Loesbanluechai; Ekawat Pasomsub; Elizabeth Batty; Insee Sensorn; Janjira Thaipadungpanit; Khajohn Joonlasak; Kingkan Rakmanee; Krittikorn Kumpornsin; Namfon Kotanan; Stefan Fernandez; Thanat Chookajorn; Theerarat Kochakarn; Treewat Watthanachockchai; Wasun Chantratita; Wudtichai Manasatienkij

EPI_ISL_423039 to 423043, EPI_ISL_429164 to 429184, EPI_ISL_445380, EPI_ISL_446996 to 447029, EPI_ISL_450723, EPI_ISL_455908 to 455948, EPI_ISL_498262 to 498266, EPI_ISL_512847 to 512872, EPI_ISL_568580 to 568586, EPI_ISL_1491555 to 1491560

see above	Ramathibodi Hospital	COVID-19 Network Investigations (CONI) Alliance	Angkana Huang; Anthony R. Jones; Arporn Wangwiwatsin; Bhakbhoom Panthan; Chonticha Klungtong; Duangkamon Loesbanluechai; Ekawat Pasomsub; Elizabeth Batty; Insee Sensorn; Janjira Thaipadungpanit; Khajohn Joonlasak; Khajohn Joonsalak; Kingkan Rakmanee; Krittikorn Kumpornsin; Namfon Kotanan; Poramate Jiaranai; Stefan Fernandez; Thanat Chookajorn; Theerarat Kochakarn; Treewat Watthanachockchai; Wasun Chantratita; Wudtichai Manasatienkij
EPI_ISL_434700, EPI_ISL_455604	Ramkhamhaeng Hospital	National Institute of Health. Department of medical Sciences, Ministry of Public Health, Thailand	Chittaganpitch; Malinee; Okada; Parnmen; Phuygun; Pilailuk; Siripaporn; Sittiporn; Sunthareeya; Thanadachakul; Thanutsapa; Waicharoen; Warawan; Wongboot

EPI_ISL_1389400 to 1389621, EPI_ISL_1409185, EPI_ISL_1409188, EPI_ISL_1409201, EPI_ISL_1409209, EPI_ISL_1409212, EPI_ISL_1409217, EPI_ISL_1409224, EPI_ISL_1409230, EPI_ISL_1409235, EPI_ISL_1409240, EPI_ISL_1409242, EPI_ISL_1409252, EPI_ISL_1409255, EPI_ISL_1409258 to 1409259, EPI_ISL_1409271, EPI_ISL_1409278, EPI_ISL_1409283, EPI_ISL_1409294, EPI_ISL_1409297, EPI_ISL_1409304, EPI_ISL_1409311, EPI_ISL_1409316, EPI_ISL_1409321, EPI_ISL_1409330, EPI_ISL_1409333, EPI_ISL_1409338, EPI_ISL_1409340, EPI_ISL_1409343, EPI_ISL_1409359, EPI_ISL_1409362, EPI_ISL_1409367, EPI_ISL_1409373, EPI_ISL_1409386, EPI_ISL_1409388, EPI_ISL_1409392, EPI_ISL_1409394, EPI_ISL_1409401, EPI_ISL_1409411, EPI_ISL_1409416, EPI_ISL_1409420, EPI_ISL_1409428, EPI_ISL_1409430, EPI_ISL_1409432 to 1409433, EPI_ISL_1409437, EPI_ISL_1409443, EPI_ISL_1409448 to 1409449, EPI_ISL_1409456, EPI_ISL_1409462, EPI_ISL_1409475, EPI_ISL_1409483, EPI_ISL_1409485, EPI_ISL_1409498, EPI_ISL_1409525, EPI_ISL_1409527, EPI_ISL_1409530, EPI_ISL_1409536, EPI_ISL_1409540, EPI_ISL_1409543 to 1409544, EPI_ISL_1409553, EPI_ISL_1409558, EPI_ISL_1409574, EPI_ISL_1409576, EPI_ISL_1409589, EPI_ISL_1409599 to 1409600, EPI_ISL_1409616, EPI_ISL_1409620, EPI_ISL_1409625, EPI_ISL_1409629, EPI_ISL_1409633, EPI_ISL_1409637, EPI_ISL_1409639 to 1409640, EPI_ISL_1409651, EPI_ISL_1409662, EPI_ISL_1409675, EPI_ISL_1409680, EPI_ISL_1409687, EPI_ISL_1409700, EPI_ISL_1409707, EPI_ISL_1409711, EPI_ISL_1409724, EPI_ISL_1409737, EPI_ISL_1409743 to 1409745, EPI_ISL_1409751, EPI_ISL_1409753, EPI_ISL_1409757, EPI_ISL_1411101 to 1411102, EPI_ISL_1411105, EPI_ISL_1411112 to 1411116, EPI_ISL_1411118, EPI_ISL_1411122 to 1411125, EPI_ISL_1411127 to 1411128, EPI_ISL_1411131, EPI_ISL_1411133, EPI_ISL_1411136, EPI_ISL_1411138, EPI_ISL_1411142, EPI_ISL_1411147, EPI_ISL_1411150, EPI_ISL_1411153, EPI_ISL_1411156, EPI_ISL_1411158, EPI_ISL_1411161, EPI_ISL_1411163, EPI_ISL_1411168, EPI_ISL_1411170 to 1411172, EPI_ISL_1411179 to 1411180, EPI_ISL_1411183 to 1411185, EPI_ISL_1411189 to 1411190, EPI_ISL_1411194 to 1411197, EPI_ISL_1411204 to 1411205, EPI_ISL_1411209, EPI_ISL_1411211, EPI_ISL_1411213, EPI_ISL_1411218 to 1411219, EPI_ISL_1411223, EPI_ISL_1411225, EPI_ISL_1411229 to 1411234, EPI_ISL_1411236 to 1411240, EPI_ISL_1411243 to 1411246, EPI_ISL_1411250, EPI_ISL_1411256, EPI_ISL_1411258, EPI_ISL_1411260 to 1411261, EPI_ISL_1411266, EPI_ISL_1411268, EPI_ISL_1411272, EPI_ISL_1411274, EPI_ISL_1411278 to 1411280, EPI_ISL_1411282 to 1411283, EPI_ISL_1411285 to 1411287, EPI_ISL_1411295 to 1411297, EPI_ISL_1411299, EPI_ISL_1411302 to 1411304, EPI_ISL_1411306, EPI_ISL_1411308 to 1411311, EPI_ISL_1411313, EPI_ISL_1411315 to 1411317, EPI_ISL_1411319 to 1411320, EPI_ISL_1411322, EPI_ISL_1411325, EPI_ISL_1411328 to 1411329, EPI_ISL_1411334 to 1411335, EPI_ISL_1411337, EPI_ISL_1411339, EPI_ISL_1411345 to 1411346, EPI_ISL_1411348, EPI_ISL_1411352, EPI_ISL_1411356 to 1411358, EPI_ISL_1411360, EPI_ISL_1411362, EPI_ISL_1411365, EPI_ISL_1411369 to 1411372, EPI_ISL_1411374 to 1411375, EPI_ISL_1411377 to 1411380, EPI_ISL_1411382, EPI_ISL_1411384 to 1411387, EPI_ISL_1411389, EPI_ISL_1411391, EPI_ISL_1411393 to 1411395, EPI_ISL_1411400 to 1411402, EPI_ISL_1411404 to 1411406, EPI_ISL_1411409, EPI_ISL_1411411 to 1411413, EPI_ISL_1411415 to 1411416, EPI_ISL_1411419, EPI_ISL_1411422 to 1411426, EPI_ISL_1411428 to 1411430, EPI_ISL_1411432, EPI_ISL_1411436 to 1411438, EPI_ISL_1411440 to 1411441, EPI_ISL_1411445 to 1411446, EPI_ISL_1411448, EPI_ISL_1411452, EPI_ISL_1411455 to 1411456, EPI_ISL_1411459, EPI_ISL_1411464, EPI_ISL_1411467, EPI_ISL_1411480, EPI_ISL_1411483, EPI_ISL_1411485, EPI_ISL_1411487, EPI_ISL_1411489 to 1411490, EPI_ISL_1411493, EPI_ISL_1411495 to 1411496, EPI_ISL_1411500, EPI_ISL_1411505, EPI_ISL_1411555, EPI_ISL_1411575, EPI_ISL_1411586, EPI_ISL_1411591, EPI_ISL_1411594, EPI_ISL_1411605, EPI_ISL_1411613, EPI_ISL_1411615, EPI_ISL_1411617, EPI_ISL_1411620, EPI_ISL_1411628, EPI_ISL_1411634, EPI_ISL_1411637, EPI_ISL_1411642, EPI_ISL_1411644, EPI_ISL_1411655, EPI_ISL_1411663 to 1411664, EPI_ISL_1411673 to 1411674, EPI_ISL_1411676, EPI_ISL_1411678, EPI_ISL_1411688, EPI_ISL_1411692, EPI_ISL_1411699, EPI_ISL_1411702, EPI_ISL_1411704, EPI_ISL_1411707, EPI_ISL_1411712, EPI_ISL_1411716, EPI_ISL_1411720, EPI_ISL_1411724, EPI_ISL_1411726, EPI_ISL_1411728, EPI_ISL_1411730, EPI_ISL_1411733 to 1411734, EPI_ISL_1411742, EPI_ISL_1411745, EPI_ISL_1411747 to 1411749, EPI_ISL_1411756, EPI_ISL_1411760, EPI_ISL_1411763, EPI_ISL_1411767, EPI_ISL_1411770, EPI_ISL_1411773, EPI_ISL_1411780, EPI_ISL_1411782 to 1411783, EPI_ISL_1411786 to 1411787, EPI_ISL_1411789, EPI_ISL_1411792, EPI_ISL_1411802, EPI_ISL_1411808, EPI_ISL_1411813, EPI_ISL_1411822 to 1411824, EPI_ISL_1411828, EPI_ISL_1411830 to 1411831, EPI_ISL_1411839, EPI_ISL_1411843, EPI_ISL_1411845 to 1411847, EPI_ISL_1411851, EPI_ISL_1412206, EPI_ISL_1412212, EPI_ISL_1412229, EPI_ISL_1412239 to 1412241, EPI_ISL_1412265, EPI_ISL_1412270, EPI_ISL_1412281, EPI_ISL_1412301, EPI_ISL_1412317, EPI_ISL_1412338 to 1412340, EPI_ISL_1412346, EPI_ISL_1412354, EPI_ISL_1412365, EPI_ISL_1412374, EPI_ISL_1412388, EPI_ISL_1412392, EPI_ISL_1412410, EPI_ISL_1412437, EPI_ISL_1412450, EPI_ISL_1412454, EPI_ISL_1412462, EPI_ISL_1412464, EPI_ISL_1412486, EPI_ISL_1412527, EPI_ISL_1412547, EPI_ISL_1412928, EPI_ISL_1412930, EPI_ISL_1412934, EPI_ISL_1412936, EPI_ISL_1412938, EPI_ISL_1412940 to 1412941, EPI_ISL_1412943, EPI_ISL_1412946 to 1412947, EPI_ISL_1412950 to 1412952, EPI_ISL_1412954, EPI_ISL_1412957, EPI_ISL_1412959, EPI_ISL_1412961, EPI_ISL_1412963, EPI_ISL_1412968, EPI_ISL_1412972 to 1412977, EPI_ISL_1412982 to 1412985, EPI_ISL_1412990 to 1412993, EPI_ISL_1412996, EPI_ISL_1412999 to 1413001, EPI_ISL_1413003 to 1413004, EPI_ISL_1413010 to 1413012, EPI_ISL_1413020 to 1413024, EPI_ISL_1413030 to 1413031, EPI_ISL_1413036 to 1413037, EPI_ISL_1413040 to 1413042, EPI_ISL_1413045, EPI_ISL_1413048 to 1413050, EPI_ISL_1413052, EPI_ISL_1413058, EPI_ISL_1413060, EPI_ISL_1416126 to 1416130, EPI_ISL_1416132, EPI_ISL_1416134 to 1416164, EPI_ISL_1416166 to 1416171, EPI_ISL_1450607 to 1450889, EPI_ISL_1451151, EPI_ISL_1451155 to 1451156, EPI_ISL_1451161, EPI_ISL_1451168, EPI_ISL_1451171 to 1451172, EPI_ISL_1451177, EPI_ISL_1451184, EPI_ISL_1451186, EPI_ISL_1451190, EPI_ISL_1451202, EPI_ISL_1451204 to 1451205, EPI_ISL_1451207, EPI_ISL_1451209, EPI_ISL_1451213, EPI_ISL_1451217 to 1451219, EPI_ISL_1451223, EPI_ISL_1451229 to 1451230, EPI_ISL_1451234, EPI_ISL_1451236, EPI_ISL_1451242, EPI_ISL_1451245, EPI_ISL_1451247 to 1451248, EPI_ISL_1451250, EPI_ISL_1451253, EPI_ISL_1451256 to 1451259, EPI_ISL_1451262, EPI_ISL_1451265, EPI_ISL_1451273, EPI_ISL_1451278, EPI_ISL_1451282 to 1451283, EPI_ISL_1451289, EPI_ISL_1451292, EPI_ISL_1451297 to 1451299, EPI_ISL_1451305 to 1451307, EPI_ISL_1451312, EPI_ISL_1451318 to 1451319, EPI_ISL_1451328, EPI_ISL_1451330 to 1451331, EPI_ISL_1451336 to 1451338, EPI_ISL_1451340 to 1451341, EPI_ISL_1451348, EPI_ISL_1451352, EPI_ISL_1451361, EPI_ISL_1451365, EPI_ISL_1451374, EPI_ISL_1451377, EPI_ISL_1451379, EPI_ISL_1451381, EPI_ISL_1451391, EPI_ISL_1451396, EPI_ISL_1451401, EPI_ISL_1451403, EPI_ISL_1451405, EPI_ISL_1451414, EPI_ISL_1451419, EPI_ISL_1451422, EPI_ISL_1451426 to 1451427, EPI_ISL_1451430 to 1451432, EPI_ISL_1451438, EPI_ISL_1451455 to 1451456, EPI_ISL_1451458, EPI_ISL_1451462, EPI_ISL_1451464, EPI_ISL_1451468 to 1451469, EPI_ISL_1451474, EPI_ISL_1451477 to 1451478, EPI_ISL_1451489 to 1451490, EPI_ISL_1451493, EPI_ISL_1451498 to 1451500, EPI_ISL_1451506, EPI_ISL_1451511 to 1451513, EPI_ISL_1451515, EPI_ISL_1451518, EPI_ISL_1451521, EPI_ISL_1451523, EPI_ISL_1451528 to 1451530, EPI_ISL_1451532, EPI_ISL_1451536, EPI_ISL_1451542 to 1451543, EPI_ISL_1451550 to 1451551, EPI_ISL_1451554, EPI_ISL_1451557, EPI_ISL_1451563, EPI_ISL_1451565, EPI_ISL_1451568, EPI_ISL_1451574, EPI_ISL_1451578, EPI_ISL_1451582 to 1451584, EPI_ISL_1451587, EPI_ISL_1451589, EPI_ISL_1451592 to 1451594, EPI_ISL_1451598, EPI_ISL_1451603 to 1451605, EPI_ISL_1451608, EPI_ISL_1451610, EPI_ISL_1451612, EPI_ISL_1451614, EPI_ISL_1451616 to 1451617, EPI_ISL_1451619, EPI_ISL_1451622, EPI_ISL_1451624, EPI_ISL_1451627, EPI_ISL_1451629 to 1451632, EPI_ISL_1451635, EPI_ISL_1451642 to 1451644, EPI_ISL_1451650, EPI_ISL_1451652, EPI_ISL_1451658 to 1451661, EPI_ISL_1451664 to 1451665, EPI_ISL_1451679, EPI_ISL_1451696, EPI_ISL_1451699 to 1451701, EPI_ISL_1451703 to 1451705, EPI_ISL_1451712, EPI_ISL_1451714, EPI_ISL_1451716, EPI_ISL_1451721 to 1451723, EPI_ISL_1451725, EPI_ISL_1451727, EPI_ISL_1451733, EPI_ISL_1451738, EPI_ISL_1451743, EPI_ISL_1451748 to 1451749, EPI_ISL_1451755, EPI_ISL_1451758 to 1451759, EPI_ISL_1451763 to 1451764, EPI_ISL_1451768, EPI_ISL_1451771, EPI_ISL_1451774, EPI_ISL_1451780 to 1451783, EPI_ISL_1454955, EPI_ISL_1454959, EPI_ISL_1454961 to 1454962, EPI_ISL_1454964, EPI_ISL_1455633 to 1455636, EPI_ISL_1466934, EPI_ISL_1466946, EPI_ISL_1466949, EPI_ISL_1466951, EPI_ISL_1466955 to 1466958, EPI_ISL_1466962, EPI_ISL_1466975, EPI_ISL_1466983, EPI_ISL_1466987, EPI_ISL_1466990 to 1466993, EPI_ISL_1466997, EPI_ISL_1467000, EPI_ISL_1467009, EPI_ISL_1467017 to 1467019, EPI_ISL_1467026, EPI_ISL_1467029, EPI_ISL_1467034, EPI_ISL_1467038, EPI_ISL_1467041, EPI_ISL_1467044, EPI_ISL_1467046, EPI_ISL_1467049, EPI_ISL_1467051, EPI_ISL_1467055, EPI_ISL_1467059, EPI_ISL_1467067 to 1467068, EPI_ISL_1467075, EPI_ISL_1467077, EPI_ISL_1467080, EPI_ISL_1467088, EPI_ISL_1467098, EPI_ISL_1467113, EPI_ISL_1467115, EPI_ISL_1467122, EPI_ISL_1467128, EPI_ISL_1467131, EPI_ISL_1467134, EPI_ISL_1467138 to 1467140, EPI_ISL_1467147, EPI_ISL_1467167, EPI_ISL_1467170 to 1467171, EPI_ISL_1467180, EPI_ISL_1467182, EPI_ISL_1467187 to 1467188, EPI_ISL_1467190, EPI_ISL_1467206, EPI_ISL_1467208, EPI_ISL_1467229 to 1467231, EPI_ISL_1467233, EPI_ISL_1467236, EPI_ISL_1467239, EPI_ISL_1467252 to 1467253, EPI_ISL_1467258, EPI_ISL_1467260 to 1467261, EPI_ISL_1467263 to 1467264, EPI_ISL_1467267, EPI_ISL_1467269, EPI_ISL_1467275 to 1467276, EPI_ISL_1467282 to 1467283, EPI_ISL_1467287 to 1467288, EPI_ISL_1467290, EPI_ISL_1467292 to 1467293, EPI_ISL_1467295 to 1467296, EPI_ISL_1467300, EPI_ISL_1467303 to 1467305, EPI_ISL_1467311, EPI_ISL_1467313, EPI_ISL_1467317, EPI_ISL_1467319, EPI_ISL_1467321, EPI_ISL_1467325 to 1467327, EPI_ISL_1467330, EPI_ISL_1467335 to 1467337, EPI_ISL_1467340 to 1467343, EPI_ISL_1467348 to 1467349, EPI_ISL_1467351 to 1467352, EPI_ISL_1467354, EPI_ISL_1467356, EPI_ISL_1467358, EPI_ISL_1467362 to 1467365, EPI_ISL_1467367, EPI_ISL_1467370 to 1467372, EPI_ISL_1467377, EPI_ISL_1467385, EPI_ISL_1467387, EPI_ISL_1467389 to 1467390, EPI_ISL_1467392, EPI_ISL_1467395, EPI_ISL_1467399, EPI_ISL_1467402 to 1467403, EPI_ISL_1467405, EPI_ISL_1467407, EPI_ISL_1467410 to 1467411, EPI_ISL_1467413 to 1467414, EPI_ISL_1467418, EPI_ISL_1467424, EPI_ISL_1467426 to 1467427, EPI_ISL_1467433, EPI_ISL_1467435, EPI_ISL_1467440, EPI_ISL_1467442 to 1467443, EPI_ISL_1467447, EPI_ISL_1467449 to 1467450, EPI_ISL_1467454 to 1467456, EPI_ISL_1467458 to 1467462, EPI_ISL_1467466, EPI_ISL_1467473, EPI_ISL_1467476, EPI_ISL_1467478, EPI_ISL_1467480, EPI_ISL_1467482, EPI_ISL_1467485, EPI_ISL_1467488 to 1467490, EPI_ISL_1467494, EPI_ISL_1467498, EPI_ISL_1467500 to 1467502, EPI_ISL_1467505, EPI_ISL_1467507 to 1467508, EPI_ISL_1467512, EPI_ISL_1467514, EPI_ISL_1467519, EPI_ISL_1467521, EPI_ISL_1467526 to 1467527, EPI_ISL_1467530, EPI_ISL_1467532, EPI_ISL_1467542 to 1467543, EPI_ISL_1467547 to 1467548, EPI_ISL_1467551, EPI_ISL_1467555 to 1467558, EPI_ISL_1467560 to 1467562, EPI_ISL_1467566, EPI_ISL_1467569 to 1467572, EPI_ISL_1467575, EPI_ISL_1467580, EPI_ISL_1472701 to 1472702, EPI_ISL_1472705, EPI_ISL_1472711, EPI_ISL_1472716 to 1472717, EPI_ISL_1472719, EPI_ISL_1472722, EPI_ISL_1472729, EPI_ISL_1472731, EPI_ISL_1472734, EPI_ISL_1472741, EPI_ISL_1472747, EPI_ISL_1472753 to 1472754, EPI_ISL_1472757, EPI_ISL_1472764 to 1472765, EPI_ISL_1472767, EPI_ISL_1472772, EPI_ISL_1472782, EPI_ISL_1472785, EPI_ISL_1472789, EPI_ISL_1472794, EPI_ISL_1472800 to 1472802, EPI_ISL_1472807, EPI_ISL_1472814 to 1472815, EPI_ISL_1472824 to 1472825, EPI_ISL_1472827, EPI_ISL_1472832, EPI_ISL_1472837, EPI_ISL_1472839, EPI_ISL_1472841, EPI_ISL_1472843, EPI_ISL_1472851, EPI_ISL_1472854, EPI_ISL_1472860, EPI_ISL_1472863, EPI_ISL_1472866, EPI_ISL_1472881, EPI_ISL_1472885, EPI_ISL_1472893, EPI_ISL_1472900 to 1472901, EPI_ISL_1472907, EPI_ISL_1472910, EPI_ISL_1472920, EPI_ISL_1472927 to 1472928, EPI_ISL_1472934 to 1472935, EPI_ISL_1472937, EPI_ISL_1472940 to 1472941, EPI_ISL_1473454, EPI_ISL_1473586, EPI_ISL_1473597, EPI_ISL_1473601, EPI_ISL_1473605, EPI_ISL_1473610, EPI_ISL_1473612 to 1473615, EPI_ISL_1473622, EPI_ISL_1473624, EPI_ISL_1473627, EPI_ISL_1473629, EPI_ISL_1473633, EPI_ISL_1473635, EPI_ISL_1473638, EPI_ISL_1473647, EPI_ISL_1473649 to 1473650, EPI_ISL_1473652, EPI_ISL_1473654, EPI_ISL_1473659, EPI_ISL_1473665, EPI_ISL_1473669 to 1473670, EPI_ISL_1473672, EPI_ISL_1473674, EPI_ISL_1473676, EPI_ISL_1473682, EPI_ISL_1473684, EPI_ISL_1473687, EPI_ISL_1473690 to 1473692, EPI_ISL_1473695 to 1473696, EPI_ISL_1473699, EPI_ISL_1473701 to 1473702, EPI_ISL_1473705, EPI_ISL_1473707, EPI_ISL_1473709 to 1473710, EPI_ISL_1473712, EPI_ISL_1473720, EPI_ISL_1473722, EPI_ISL_1473727, EPI_ISL_1473729 to 1473732, EPI_ISL_1473735 to 1473736, EPI_ISL_1473738, EPI_ISL_1473744, EPI_ISL_1473749, EPI_ISL_1473751, EPI_ISL_1473755, EPI_ISL_1473762, EPI_ISL_1473772, EPI_ISL_1473777, EPI_ISL_1473780, EPI_ISL_1473784, EPI_ISL_1473789, EPI_ISL_1473793, EPI_ISL_1473796, EPI_ISL_1473798, EPI_ISL_1483827, EPI_ISL_1483830 to 1483831, EPI_ISL_1483833, EPI_ISL_1483839, EPI_ISL_1483858, EPI_ISL_1483860, EPI_ISL_1483868, EPI_ISL_1483880, EPI_ISL_1483882 to 1483884, EPI_ISL_1483886, EPI_ISL_1483895, EPI_ISL_1483903, EPI_ISL_1483905, EPI_ISL_1483907, EPI_ISL_1483912, EPI_ISL_1483915, EPI_ISL_1483923, EPI_ISL_1483927, EPI_ISL_1483931 to 1483932, EPI_ISL_1483934, EPI_ISL_1483945, EPI_ISL_1483949, EPI_ISL_1483953, EPI_ISL_1483960, EPI_ISL_1483962, EPI_ISL_1483971, EPI_ISL_1483979, EPI_ISL_1483981, EPI_ISL_1483997, EPI_ISL_1484001, EPI_ISL_1484004, EPI_ISL_1484006, EPI_ISL_1484010, EPI_ISL_1484014, EPI_ISL_1484016, EPI_ISL_1484019, EPI_ISL_1484023, EPI_ISL_1484032, EPI_ISL_1484038, EPI_ISL_1484041, EPI_ISL_1484043 to 1484044, EPI_ISL_1484048, EPI_ISL_1484071, EPI_ISL_1484073, EPI_ISL_1484079, EPI_ISL_1484084, EPI_ISL_1484086, EPI_ISL_1485481 to 1485482, EPI_ISL_1485487, EPI_ISL_1485493, EPI_ISL_1485496, EPI_ISL_1485498, EPI_ISL_1485504, EPI_ISL_1485508, EPI_ISL_1485530 to 1485531, EPI_ISL_1485536, EPI_ISL_1485556, EPI_ISL_1485562, EPI_ISL_1485578, EPI_ISL_1485591, EPI_ISL_1485593 to 1485594, EPI_ISL_1485600 to 1485602, EPI_ISL_1485609, EPI_ISL_1485613 to 1485614, EPI_ISL_1485623, EPI_ISL_1485625, EPI_ISL_1485628, EPI_ISL_1485630, EPI_ISL_1485635, EPI_ISL_1485641 to 1485642, EPI_ISL_1485646, EPI_ISL_1485649, EPI_ISL_1485651, EPI_ISL_1485653, EPI_ISL_1485658 to 1485659, EPI_ISL_1485670, EPI_ISL_1485679, EPI_ISL_1485681, EPI_ISL_1485684, EPI_ISL_1485694 to 1485695, EPI_ISL_1485699, EPI_ISL_1485703, EPI_ISL_1485711 to 1485714, EPI_ISL_1485723, EPI_ISL_1485727, EPI_ISL_1485731, EPI_ISL_1485749, EPI_ISL_1485751, EPI_ISL_1485753, EPI_ISL_1485755, EPI_ISL_1485758 to 1485759, EPI_ISL_1485764, EPI_ISL_1485771, EPI_ISL_1485774, EPI_ISL_1485789 to 1485790, EPI_ISL_1485792 to 1485794, EPI_ISL_1485802, EPI_ISL_1486399, EPI_ISL_1486422, EPI_ISL_1486429, EPI_ISL_1486437 to 1486438, EPI_ISL_1486445, EPI_ISL_1486478, EPI_ISL_1486499, EPI_ISL_1486501, EPI_ISL_1486508, EPI_ISL_1486545, EPI_ISL_1486548, EPI_ISL_1486550, EPI_ISL_1486573, EPI_ISL_1486575, EPI_ISL_1486627, EPI_ISL_1486642, EPI_ISL_1486660, EPI_ISL_1486680, EPI_ISL_1486707, EPI_ISL_1486710, EPI_ISL_1486732, EPI_ISL_1486744, EPI_ISL_1486766, EPI_ISL_1486769, EPI_ISL_1486781, EPI_ISL_1486788, EPI_ISL_1486796, EPI_ISL_1486805, EPI_ISL_1487881, EPI_ISL_1487899, EPI_ISL_1487901, EPI_ISL_1487903, EPI_ISL_1487905, EPI_ISL_1487921, EPI_ISL_1487961, EPI_ISL_1487967, EPI_ISL_1487970, EPI_ISL_1487997, EPI_ISL_1487999, EPI_ISL_1488056, EPI_ISL_1488128, EPI_ISL_1488132, EPI_ISL_1488146, EPI_ISL_1488153, EPI_ISL_1488165, EPI_ISL_1488168, EPI_ISL_1488202, EPI_ISL_1488223, EPI_ISL_1488226, EPI_ISL_1488523, EPI_ISL_1488525, EPI_ISL_1488544, EPI_ISL_1488552, EPI_ISL_1488558, EPI_ISL_1488575 to 1488576, EPI_ISL_1488579, EPI_ISL_1488600, EPI_ISL_1488605, EPI_ISL_1488608, EPI_ISL_1488615, EPI_ISL_1488617, EPI_ISL_1488627, EPI_ISL_1488630, EPI_ISL_1488633, EPI_ISL_1488641, EPI_ISL_1488651, EPI_ISL_1488654, EPI_ISL_1488659, EPI_ISL_1488674 to 1488675, EPI_ISL_1488678, EPI_ISL_1488680, EPI_ISL_1488704, EPI_ISL_1488723, EPI_ISL_1488733, EPI_ISL_1488740, EPI_ISL_1488762 to 1488763, EPI_ISL_1488774, EPI_ISL_1488777, EPI_ISL_1488789, EPI_ISL_1488795, EPI_ISL_1488798, EPI_ISL_1488802, EPI_ISL_1488806, EPI_ISL_1488809, EPI_ISL_1488813 to 1488815, EPI_ISL_1488821, EPI_ISL_1488830, EPI_ISL_1488836, EPI_ISL_1488839, EPI_ISL_1488841, EPI_ISL_1488859, EPI_ISL_1488861 to 1488862, EPI_ISL_1489161, EPI_ISL_1489165, EPI_ISL_1489171, EPI_ISL_1489175, EPI_ISL_1489183, EPI_ISL_1489185, EPI_ISL_1489187, EPI_ISL_1489189, EPI_ISL_1489192, EPI_ISL_1489200, EPI_ISL_1489205 to 1489208, EPI_ISL_1489212, EPI_ISL_1489214, EPI_ISL_1489217, EPI_ISL_1489219, EPI_ISL_1489221 to 1489223, EPI_ISL_1489225 to 1489226, EPI_ISL_1489228, EPI_ISL_1489236, EPI_ISL_1489239, EPI_ISL_1489242 to 1489245, EPI_ISL_1489253, EPI_ISL_1489261 to 1489263, EPI_ISL_1489267, EPI_ISL_1489274, EPI_ISL_1489278, EPI_ISL_1489280, EPI_ISL_1489284 to 1489285, EPI_ISL_1489293, EPI_ISL_1489296, EPI_ISL_1489304 to 1489305, EPI_ISL_1489317 to 1489319, EPI_ISL_1489321, EPI_ISL_1489332, EPI_ISL_1489336, EPI_ISL_1489350, EPI_ISL_1489352 to 1489353, EPI_ISL_1489358, EPI_ISL_1489362, EPI_ISL_1489368, EPI_ISL_1489375 to 1489376, EPI_ISL_1489389, EPI_ISL_1489393, EPI_ISL_1489395 to 1489396, EPI_ISL_1489400, EPI_ISL_1489416, EPI_ISL_1489420, EPI_ISL_1489423 to 1489424, EPI_ISL_1489426, EPI_ISL_1489437 to 1489438, EPI_ISL_1489441, EPI_ISL_1489445 to 1489446, EPI_ISL_1489448, EPI_ISL_1489450, EPI_ISL_1489452, EPI_ISL_1489454, EPI_ISL_1489493 to 1489494, EPI_ISL_1504667, EPI_ISL_1504682 to 1504685, EPI_ISL_1504690, EPI_ISL_1504694, EPI_ISL_1504696, EPI_ISL_1504698, EPI_ISL_1504703, EPI_ISL_1504706 to 1504707, EPI_ISL_1504710, EPI_ISL_1504712, EPI_ISL_1504716, EPI_ISL_1504723 to 1504724, EPI_ISL_1504728, EPI_ISL_1504730, EPI_ISL_1504732, EPI_ISL_1504734, EPI_ISL_1504742 to 1504745, EPI_ISL_1504749 to 1504751, EPI_ISL_1504753, EPI_ISL_1504756, EPI_ISL_1504758, EPI_ISL_1504767 to 1504769, EPI_ISL_1504771 to 1504773, EPI_ISL_1504776, EPI_ISL_1504784, EPI_ISL_1504791, EPI_ISL_1504795, EPI_ISL_1504804 to 1504806, EPI_ISL_1504808, EPI_ISL_1504814, EPI_ISL_1504817, EPI_ISL_1504820, EPI_ISL_1504826 to 1504827, EPI_ISL_1504829, EPI_ISL_1504831, EPI_ISL_1504834 to 1504835, EPI_ISL_1504846, EPI_ISL_1504851, EPI_ISL_1504861, EPI_ISL_1504868, EPI_ISL_1504871, EPI_ISL_1504875, EPI_ISL_1504878 to 1504879, EPI_ISL_1504882, EPI_ISL_1504888, EPI_ISL_1504893 to 1504895, EPI_ISL_1504905, EPI_ISL_1504913, EPI_ISL_1504930, EPI_ISL_1504940, EPI_ISL_1504944, EPI_ISL_1504953 to 1504954, EPI_ISL_1504960, EPI_ISL_1504974, EPI_ISL_1504978, EPI_ISL_1504985, EPI_ISL_1505023, EPI_ISL_1505051, EPI_ISL_1505059, EPI_ISL_1505075, EPI_ISL_1505078, EPI_ISL_1505085, EPI_ISL_1505092, EPI_ISL_1505097, EPI_ISL_1505101, EPI_ISL_1505107, EPI_ISL_1505113, EPI_ISL_1505117, EPI_ISL_1505135, EPI_ISL_1505145 to 1505146, EPI_ISL_1505152, EPI_ISL_1505459 to 1505460, EPI_ISL_1505467, EPI_ISL_1505470 to 1505472, EPI_ISL_1505489, EPI_ISL_1505491 to 1505493, EPI_ISL_1505496 to 1505497, EPI_ISL_1505509, EPI_ISL_1505520, EPI_ISL_1505525, EPI_ISL_1505531, EPI_ISL_1505534 to 1505535, EPI_ISL_1505539, EPI_ISL_1505541, EPI_ISL_1505543, EPI_ISL_1505545, EPI_ISL_1505548, EPI_ISL_1505550 to 1505551, EPI_ISL_1505563, EPI_ISL_1505569, EPI_ISL_1505578, EPI_ISL_1505580, EPI_ISL_1505583, EPI_ISL_1505589, EPI_ISL_1505591, EPI_ISL_1505594 to 1505595, EPI_ISL_1505599 to 1505600, EPI_ISL_1505608, EPI_ISL_1505610, EPI_ISL_1505612, EPI_ISL_1505614, EPI_ISL_1505621, EPI_ISL_1505625, EPI_ISL_1505631, EPI_ISL_1505637, EPI_ISL_1505643, EPI_ISL_1505650, EPI_ISL_1505653, EPI_ISL_1505655 to 1505658, EPI_ISL_1505677, EPI_ISL_1505681, EPI_ISL_1505693 to 1505695, EPI_ISL_1505698, EPI_ISL_1505706,

EPI_ISL_1505712, EPI_ISL_1505716, EPI_ISL_1505718, EPI_ISL_1505723, EPI_ISL_1505727 to 1505728, EPI_ISL_1505735, EPI_ISL_1505739, EPI_ISL_1505742 to 1505743, EPI_ISL_1505750, EPI_ISL_1505756, EPI_ISL_1505767 to 1505768, EPI_ISL_1505770 to 1505771, EPI_ISL_1505786, EPI_ISL_1505788, EPI_ISL_1505793 to 1505794, EPI_ISL_1505808, EPI_ISL_1505811 to 1505812, EPI_ISL_1505814, EPI_ISL_1505827, EPI_ISL_1505845, EPI_ISL_1505856, EPI_ISL_1505858 to 1505859, EPI_ISL_1505862, EPI_ISL_1505866, EPI_ISL_1505873, EPI_ISL_1505889, EPI_ISL_1505899, EPI_ISL_1505907, EPI_ISL_1505940, EPI_ISL_1505944, EPI_ISL_1505950, EPI_ISL_1505960 to 1505961, EPI_ISL_1505963, EPI_ISL_1505973, EPI_ISL_1505977, EPI_ISL_1505995, EPI_ISL_1506002 to 1506003, EPI_ISL_1506006, EPI_ISL_1506015, EPI_ISL_1506026, EPI_ISL_1506039, EPI_ISL_1506041, EPI_ISL_1506045 to 1506046, EPI_ISL_1506050, EPI_ISL_1506060, EPI_ISL_1506065 to 1506066, EPI_ISL_1506070, EPI_ISL_1506078, EPI_ISL_1506085, EPI_ISL_1506089, EPI_ISL_1506092, EPI_ISL_1506094, EPI_ISL_1506104, EPI_ISL_1506425, EPI_ISL_1518073, EPI_ISL_1518078, EPI_ISL_1518082, EPI_ISL_1518092, EPI_ISL_1518097, EPI_ISL_1518112, EPI_ISL_1518114, EPI_ISL_1518130, EPI_ISL_1518152, EPI_ISL_1518183, EPI_ISL_1518195 to 1518196, EPI_ISL_1518209, EPI_ISL_1518215 to 1518217, EPI_ISL_1518232, EPI_ISL_1518239, EPI_ISL_1518241, EPI_ISL_1518246, EPI_ISL_1518256, EPI_ISL_1518280, EPI_ISL_1518288, EPI_ISL_1518319, EPI_ISL_1518331, EPI_ISL_1518342, EPI_ISL_1518348, EPI_ISL_1518350, EPI_ISL_1518373, EPI_ISL_1518679, EPI_ISL_1518681 to 1518682, EPI_ISL_1519270, EPI_ISL_1519272 to 1519273, EPI_ISL_1519275, EPI_ISL_1519279, EPI_ISL_1519281 to 1519282, EPI_ISL_1519284, EPI_ISL_1519286 to 1519287, EPI_ISL_1519302, EPI_ISL_1519910 to 1519917, EPI_ISL_1519920, EPI_ISL_1519926 to 1519928, EPI_ISL_1519932 to 1519967, EPI_ISL_1519969 to 1520097, EPI_ISL_1535612, EPI_ISL_1535627 to 1535628, EPI_ISL_1535632 to 1535633, EPI_ISL_1535642, EPI_ISL_1535664, EPI_ISL_1535666, EPI_ISL_1535684, EPI_ISL_1535701, EPI_ISL_1535707, EPI_ISL_1535710, EPI_ISL_1535720, EPI_ISL_1535724, EPI_ISL_1535739, EPI_ISL_1535753, EPI_ISL_1535758 to 1535759, EPI_ISL_1535761, EPI_ISL_1535769, EPI_ISL_1535772, EPI_ISL_1535788 to 1535789, EPI_ISL_1535792, EPI_ISL_1535804, EPI_ISL_1535806, EPI_ISL_1535820 to 1535821, EPI_ISL_1535825 to 1535826, EPI_ISL_1535856, EPI_ISL_1535862, EPI_ISL_1535868, EPI_ISL_1535872, EPI_ISL_1535878, EPI_ISL_1535881, EPI_ISL_1535898, EPI_ISL_1536194, EPI_ISL_1536217, EPI_ISL_1536237, EPI_ISL_1536267, EPI_ISL_1536287, EPI_ISL_1536322, EPI_ISL_1536351, EPI_ISL_1536362, EPI_ISL_1536370, EPI_ISL_1536373, EPI_ISL_1536395, EPI_ISL_1536421 to 1536422, EPI_ISL_1536433, EPI_ISL_1536458, EPI_ISL_1536486, EPI_ISL_1536520, EPI_ISL_1536525, EPI_ISL_1536533, EPI_ISL_1536537, EPI_ISL_1536862 to 1536863, EPI_ISL_1536898, EPI_ISL_1536904, EPI_ISL_1536920, EPI_ISL_1536934, EPI_ISL_1536996 to 1536997, EPI_ISL_1537005, EPI_ISL_1537025, EPI_ISL_1537031, EPI_ISL_1537049, EPI_ISL_1537071, EPI_ISL_1537091 to 1537092, EPI_ISL_1537101, EPI_ISL_1537106, EPI_ISL_1537122, EPI_ISL_1537124, EPI_ISL_1537126, EPI_ISL_1537131, EPI_ISL_1537134 to 1537136, EPI_ISL_1537138 to 1537140, EPI_ISL_1538049, EPI_ISL_1538404, EPI_ISL_1544203, EPI_ISL_1544210, EPI_ISL_1544218, EPI_ISL_1544222 to 1544224, EPI_ISL_1544227, EPI_ISL_1544229, EPI_ISL_1544239 to 1544240, EPI_ISL_1544248, EPI_ISL_1544259 to 1544261, EPI_ISL_1544264, EPI_ISL_1544267, EPI_ISL_1544274, EPI_ISL_1544281, EPI_ISL_1544288, EPI_ISL_1544293 to 1544296, EPI_ISL_1544299, EPI_ISL_1544308 to 1544309, EPI_ISL_1544314, EPI_ISL_1544317, EPI_ISL_1544319, EPI_ISL_1544331, EPI_ISL_1544339, EPI_ISL_1544349, EPI_ISL_1544354, EPI_ISL_1544370, EPI_ISL_1544382, EPI_ISL_1544385, EPI_ISL_1544388, EPI_ISL_1544390, EPI_ISL_1544396 to 1544397, EPI_ISL_1544407, EPI_ISL_1544409, EPI_ISL_1544414 to 1544416, EPI_ISL_1544419, EPI_ISL_1544424 to 1544426, EPI_ISL_1544432 to 1544433, EPI_ISL_1544437, EPI_ISL_1544453, EPI_ISL_1544462, EPI_ISL_1544464 to 1544466, EPI_ISL_1544473 to 1544474, EPI_ISL_1544476, EPI_ISL_1544480, EPI_ISL_1544485, EPI_ISL_1544487, EPI_ISL_1544490, EPI_ISL_1544493			
see above	Randox Laboratories	Wellcome Sanger Institute for the COVID-19 Genomics UK (COG-UK) Consortium	Cordelia Langford; David K. Jackson; Dominic Kwiatkowski; Ewan Harrison; Ian Johnston; Jeffrey Barrett; John Sillitoe on behalf of the Wellcome Sanger Institute COVID-19 Surveillance Team; Randox Laboratories and Alex Alderton; Roberto Amato; Sonia Goncalves
EPI_ISL_1534342 to 1534343, EPI_ISL_1534443	Red Cross Children's Hospital wc RXH	NHLS/UCT	Arash Iranzadeh; Bruna Galvao; Carolyn Williamson; Deelan Doolabh; Diana Hardie; Emmanuel SJ; Innocent Mudau; Kruger Marais; Lynn Tyers; Marvin Hsiao; Stephen Korsman; Tegally H; de Oliveira T
EPI_ISL_1478885, EPI_ISL_1500179, EPI_ISL_1553173 to 1553182	Reditus Labs	Illinois Department of Public Health - Springfield Lab	Bryan Sim; Gordon McCall
EPI_ISL_1415392 to 1415419	Reference Laboratory of the Ministry of Health	Laboratory of Respiratory Viruses and Measles, Oswaldo Cruz Institute, FIOCRUZ	Alice Sampaio Rocha; Ana Carolina Mendonca; Anna Carolina Paixao; Fernando Motta; Indira Martins; Jessica Edwards; Luciana Appolinario; Marilda Siqueira on behalf of the Fiocruz COVID-19 Genomic Surveillance Network; Paola Resende; Renata Serrano Lopes
EPI_ISL_414949	Regional Virus Laboratory, Belfast	Public Health Wales Microbiology Cardiff	Catherine Moore; Conall McCaughey; Joanne Watkins; Sally Corden; Tanya Curran; Tom Connor
EPI_ISL_441355 to 441436, EPI_ISL_448918 to 448977, EPI_ISL_453465 to 453485, EPI_ISL_461773 to 461790, EPI_ISL_472136 to 472154, EPI_ISL_585092 to 585259, EPI_ISL_611531, EPI_ISL_611628, EPI_ISL_611807 to 611808, EPI_ISL_612546 to 612548, EPI_ISL_1474958 to 1474972, EPI_ISL_1474974 to 1474982, EPI_ISL_1474984 to 1474998, EPI_ISL_1475000 to 1475006, EPI_ISL_1475010 to 1475018, EPI_ISL_1475021 to 1475033, EPI_ISL_1475035 to 1475040, EPI_ISL_1475042 to 1475046, EPI_ISL_1475048 to 1475052, EPI_ISL_1475054 to 1475079, EPI_ISL_1475081 to 1475084, EPI_ISL_1475086 to 1475090, EPI_ISL_1475092 to 1475101, EPI_ISL_1475103 to 1475105, EPI_ISL_1475107 to 1475112, EPI_ISL_1475115 to 1475117, EPI_ISL_1475119 to 1475120, EPI_ISL_1475122 to 1475125, EPI_ISL_1475127 to 1475132, EPI_ISL_1475134 to 1475135, EPI_ISL_1475137 to 1475138, EPI_ISL_1475140 to 1475142, EPI_ISL_1475145 to 1475149, EPI_ISL_1475151, EPI_ISL_1475153 to 1475159, EPI_ISL_1475162, EPI_ISL_1475164 to 1475166, EPI_ISL_1475169, EPI_ISL_1475171 to 1475172, EPI_ISL_1475174, EPI_ISL_1475176 to 1475177, EPI_ISL_1475179 to 1475181, EPI_ISL_1475183 to 1475188			
see above	Regional Virus Laboratory, Belfast Health and Social Care Trust	COVID-19 Genomics UK (COG-UK) Consortium	Alison Watt; Ciara Cox; Conall McCaughey; David Simpson; Derek Fairley; James McKenna; Mairead Connor; Susan Feeney; Tanya Curran; Zoltan Molnar
EPI_ISL_459358, EPI_ISL_459372, EPI_ISL_489213, EPI_ISL_489358	Regional Virus Laboratory, Belfast Health and Social Care Trust	Wellcome Sanger Institute for the COVID-19 Genomics UK (COG-UK) Consortium	Alison Watt; Ciara Cox; Conall McCaughey; Cordelia Langford; David K. Jackson; David Simpson; Derek Fairley; Dominic Kwiatkowski; Ewan Harrison; Ian Johnston; James McKenna; John Sillitoe on behalf of the Wellcome Sanger Institute COVID-19 Surveillance Team; Mairead Connor; Roberto Amato; Sonia Goncalves; Susan Feeney; Tanya Curran; Zoltan Molnar; and Alex Alderton
EPI_ISL_441659, EPI_ISL_441662 to 441663, EPI_ISL_441670, EPI_ISL_441675 to 441676, EPI_ISL_441685, EPI_ISL_441690 to 441691, EPI_ISL_441693 to 441694, EPI_ISL_441696 to 441699, EPI_ISL_441704, EPI_ISL_441708, EPI_ISL_441712, EPI_ISL_441714, EPI_ISL_441716 to 441717, EPI_ISL_441719, EPI_ISL_441730, EPI_ISL_441733, EPI_ISL_441737, EPI_ISL_441747, EPI_ISL_441751, EPI_ISL_441753, EPI_ISL_441761, EPI_ISL_441765 to 441766, EPI_ISL_441770 to 441771, EPI_ISL_441775 to 441776, EPI_ISL_441781, EPI_ISL_459324 to 459357, EPI_ISL_459359 to 459371, EPI_ISL_459373 to 459409, EPI_ISL_469844 to 469890, EPI_ISL_470013 to 470089, EPI_ISL_470534, EPI_ISL_482032 to 482056, EPI_ISL_482070, EPI_ISL_482075, EPI_ISL_482077, EPI_ISL_482082, EPI_ISL_482091, EPI_ISL_482096 to 482097, EPI_ISL_482101 to 482102, EPI_ISL_482105, EPI_ISL_482115, EPI_ISL_482132 to 482137, EPI_ISL_482139 to 482159, EPI_ISL_488874 to 488876, EPI_ISL_489156 to 489212, EPI_ISL_489214 to 489357, EPI_ISL_489359 to 489379, EPI_ISL_500954 to 501071			
see above	Regional Virus Laboratory, Belfast Health and Social Care Trust	Wellcome Sanger Institute for the COVID-19 Genomics UK (COG-UK) consortium	Alex Alderton; Alison Watt; Ciara Cox; Conall McCaughey; Cordelia Langford; David K. Jackson; David Simpson; Derek Fairley; Dominic Kwiatkowski; Ewan Harrison; Ian Johnston; James McKenna; John Sillitoe on behalf of the Wellcome Sanger Institute COVID-19 Surveillance Team (http://www.sanger.ac.uk/covid-team); Mairead Connor; Roberto Amato; Sonia Goncalves; Susan Feeney; Tanya Curran; Zoltan Molnar; and Alex Alderton
EPI_ISL_1393739 to 1393759, EPI_ISL_1394014 to 1394040, EPI_ISL_1425542, EPI_ISL_1425556 to 1425557, EPI_ISL_1425562, EPI_ISL_1538439, EPI_ISL_1538458, EPI_ISL_1538505, EPI_ISL_1538507, EPI_ISL_1538576 to 1538579, EPI_ISL_1538738 to 1538747, EPI_ISL_1538876, EPI_ISL_1538916 to 1538918, EPI_ISL_1539019 to 1539024, EPI_ISL_1539148, EPI_ISL_1539157, EPI_ISL_1539159, EPI_ISL_1539168, EPI_ISL_1539170, EPI_ISL_1539175, EPI_ISL_1539186 to 1539197, EPI_ISL_1539218 to 1539226			
see above	Regionalne Centrum Krwiodawstwa i Krwiolecznictwa w Biaymstoku Pracownia Diagnostyki Molekularnej wirusa SARS-CoV-2	1. National Institute of Public Health - National Institute of Hygiene; 2. Eurofins Genomics Europe Sequencing GmbH	ECDC COVID-19 WGS support team; Eurofins Genomics Europe Sequencing Team; Gierczyki Rafa; Sadkowska-Todys Magorzata; Wokowicz Tomasz; Zacharczuk Katarzyna
EPI_ISL_486645	Renmin Hospital of Wuhan University Hubei General Hospital	State Key Laboratory of Agriculture Microbiology, Huazhong Agric	Zhong Zou
EPI_ISL_1384802 to 1384810, EPI_ISL_1398524 to 1398531, EPI_ISL_1510160, EPI_ISL_1510219 to 1510227	Republican Panevezys Hospital	Vilnius University Hospital Santaros Klinikos, Center of Laboratory Medicine	Daniel Naumovas; Dovile Ezerskyte; Gytis Dudas; Ingrida Olendraite; Laimonas Griskevicius; Ligita Raugaite; Mindaugas Stoskus; Monika Katenaite; Rimvydas Norvilas
EPI_ISL_1510086, EPI_ISL_1510228 to 1510229	Republican Siauliai Hospital	Vilnius University Hospital Santaros Klinikos, Center of Laboratory Medicine	Daniel Naumovas; Dovile Ezerskyte; Gytis Dudas; Ingrida Olendraite; Laimonas Griskevicius; Ligita Raugaite; Mindaugas Stoskus; Monika Katenaite; Rimvydas Norvilas
EPI_ISL_430839, EPI_ISL_491467 to 491468, EPI_ISL_491474	Research Institute for Tropical Medicine	Research Institute for Tropical Medicine	Bautista; Brunker, K.; C.S.; C.T.; Catalino Demetria; Criselda Bautista; D.L.; Daria Manalo; Demetria; E.S.; Edelwisa Mercado; F.G.M.; Francisco Gerardo Polotan; I.A.P.; Inez Andrea Medado; Kirstyn Brunker; Ma. Angelica Tujan; Manalo; Medado; Mercado; O.J.T.; Onza; Othoniel Jan Onza; Polotan
EPI_ISL_451934	Research Unit, University Hospital for Infectious Diseases "Dr. Fran Mihaljevi"	Cicin Sain lab, Helmholtz Centre for Infection Research	Ivan-Christian Kurolt; Kathrin Eschke; Zeeshan Chaudhry; Željka Maak Šafranko
EPI_ISL_454417 to 454418	Research and Experiment Center, Meizhou People Hospital	Research and Experiment Center, Meizhou People Hospital	Guo, X.; L. and Yu, Z.; Zeng
EPI_ISL_467299	Research and Medical Analysis Laboratory of Gendarmerie Royale	Research and Medical Analysis Laboratory of Gendarmerie Royale	Sanaâ LEMRISS Amal SOUIRI Hicham EL OSSMANI Saâd EL Kabbaj
EPI_ISL_407071, EPI_ISL_407073, EPI_ISL_412116, EPI_ISL_414005 to 414013, EPI_ISL_414040 to 414044, EPI_ISL_414522 to 414526, EPI_ISL_415129 to 415150, EPI_ISL_417213 to 417315, EPI_ISL_418667 to 418770, EPI_ISL_420457 to 420530, EPI_ISL_420542, EPI_ISL_420631 to 420777, EPI_ISL_420910, EPI_ISL_421768 to 421949, EPI_ISL_421951 to 422012, EPI_ISL_422436, EPI_ISL_423044 to 424165, EPI_ISL_425023 to 425024, EPI_ISL_452249 to 452258, EPI_ISL_459958 to 459961, EPI_ISL_464168 to 465162, EPI_ISL_465165 to 465678, EPI_ISL_465704 to 466625, EPI_ISL_471510 to 471527, EPI_ISL_485604 to 485611, EPI_ISL_490224 to 490254, EPI_ISL_491709 to 491735, EPI_ISL_493002 to 493003, EPI_ISL_500707 to 500715, EPI_ISL_500719, EPI_ISL_500951 to 500953, EPI_ISL_514343 to 514353, EPI_ISL_523951, EPI_ISL_526537 to 526550, EPI_ISL_526754 to 526773, EPI_ISL_527257 to 527258, EPI_ISL_527329 to 527358, EPI_ISL_528388 to 528418, EPI_ISL_528430 to 528439, EPI_ISL_528930 to 528933, EPI_ISL_528950, EPI_ISL_529027 to 529028, EPI_ISL_534717 to 534730, EPI_ISL_539880, EPI_ISL_566045 to 566076, EPI_ISL_574697 to 574704, EPI_ISL_583968 to 583993, EPI_ISL_584061, EPI_ISL_584066, EPI_ISL_593804 to 593805, EPI_ISL_593810, EPI_ISL_593824 to 593825, EPI_ISL_593829, EPI_ISL_593831, EPI_ISL_593834, EPI_ISL_593837, EPI_ISL_593843, EPI_ISL_593846, EPI_ISL_593848, EPI_ISL_593850, EPI_ISL_593854, EPI_ISL_593998, EPI_ISL_603188 to 603215, EPI_ISL_609827 to 609909, EPI_ISL_613565, EPI_ISL_613568 to 613569, EPI_ISL_613571 to 613635, EPI_ISL_613957			
see above	Respiratory Virus Unit, Microbiology Services Colindale, Public Health England	Respiratory Virus Unit, Microbiology Services Colindale, Public Health England	Angie Lackenby; Joanna Ellis; Jonathan Hubb; Kirstin Edwards; Leena Bhaw; Maria Zambon; Maria Zambon.; Monica Galiano; Omolola Akinbami; PHE Covid Sequencing Team; Richard Myers; Shahjahan Miah; Steven Platt; Tiina Talts; Tina Talts
EPI_ISL_1394774, EPI_ISL_1394779, EPI_ISL_1394781, EPI_ISL_1394783, EPI_ISL_1394785, EPI_ISL_1394794, EPI_ISL_1394802, EPI_ISL_1394805, EPI_ISL_1394809, EPI_ISL_1394813, EPI_ISL_1394816, EPI_ISL_1394822, EPI_ISL_1394825, EPI_ISL_1394833			
see above	Respublikine Siaulie ligonine	Lithuanian University of Health Sciences Hospital, Department of Genetics and Molecular Medicine	Astra Vitkauskiene; Darius Cereskevicius; Inga Nasvytiene; Mantas Sarauskas; Marius Sukys; Rasa Ugenskiene; Renaldas Jurkevicius; Rima Vainoriene; Zivile Zemeckiene
EPI_ISL_1540540, EPI_ISL_1540547, EPI_ISL_1540552 to 1540554, EPI_ISL_1540561, EPI_ISL_1540573 to 1540574	Respublikine Siauliu ligonine	Lithuanian University of Health Sciences Hospital, Department of Genetics and Molecular Medicine	Astra Vitkauskiene; Darius Cereskevicius; Inga Nasvytiene; Mantas Sarauskas; Marius Sukys; Rasa Ugenskiene; Renaldas Jurkevicius; Rima Vainoriene; Zivile Zemeckiene
EPI_ISL_1534375	Retreat CHC wc RHC	NHLS/UCT	Arash Iranzadeh; Bruna Galvao; Carolyn Williamson; Deelan Doolabh; Diana Hardie; Emmanuel SJ; Innocent Mudau; Kruger Marais; Lynn Tyers; Marvin Hsiao; Stephen Korsman; Tegally H; de Oliveira T

EPI_ISL_1407334 to 1407457, EPI_ISL_1413514, EPI_ISL_1413516, EPI_ISL_1413519, EPI_ISL_1413522, EPI_ISL_1413525, EPI_ISL_1413528, EPI_ISL_1413530, EPI_ISL_1413533, EPI_ISL_1413536, EPI_ISL_1413538, EPI_ISL_1413542, EPI_ISL_1413544, EPI_ISL_1413547, EPI_ISL_1413549, EPI_ISL_1413551, EPI_ISL_1413554, EPI_ISL_1413556, EPI_ISL_1413559, EPI_ISL_1413561, EPI_ISL_1413563, EPI_ISL_1413566, EPI_ISL_1413568, EPI_ISL_1413571, EPI_ISL_1413574, EPI_ISL_1413576, EPI_ISL_1413579, EPI_ISL_1413582, EPI_ISL_1413585, EPI_ISL_1413587, EPI_ISL_1413589, EPI_ISL_1413592, EPI_ISL_1413594, EPI_ISL_1413597, EPI_ISL_1413600, EPI_ISL_1413602, EPI_ISL_1413605, EPI_ISL_1413607, EPI_ISL_1413610, EPI_ISL_1413613, EPI_ISL_1413615, EPI_ISL_1413618, EPI_ISL_1413621, EPI_ISL_1413623, EPI_ISL_1413626, EPI_ISL_1413628, EPI_ISL_1413631, EPI_ISL_1413634, EPI_ISL_1413637, EPI_ISL_1413639, EPI_ISL_1413642, EPI_ISL_1413645, EPI_ISL_1413647, EPI_ISL_1413651, EPI_ISL_1413653, EPI_ISL_1413656, EPI_ISL_1413658, EPI_ISL_1413661, EPI_ISL_1413664, EPI_ISL_1413667, EPI_ISL_1413669, EPI_ISL_1413672, EPI_ISL_1413675, EPI_ISL_1413678, EPI_ISL_1413680, EPI_ISL_1413682, EPI_ISL_1413685, EPI_ISL_1413687, EPI_ISL_1413690, EPI_ISL_1413693, EPI_ISL_1413696, EPI_ISL_1413699, EPI_ISL_1413702, EPI_ISL_1413704, EPI_ISL_1413707, EPI_ISL_1413710, EPI_ISL_1413712, EPI_ISL_1413715, EPI_ISL_1413718, EPI_ISL_1413720, EPI_ISL_1413723, EPI_ISL_1413726, EPI_ISL_1413728, EPI_ISL_1413731, EPI_ISL_1413733, EPI_ISL_1413736, EPI_ISL_1413739, EPI_ISL_1413742, EPI_ISL_1413745, EPI_ISL_1413748, EPI_ISL_1413750, EPI_ISL_1413753, EPI_ISL_1413755, EPI_ISL_1413758, EPI_ISL_1413761, EPI_ISL_1516598 to 1516664			
see above	Rhode Island Department of Health	Infectious Disease Program, Broad Institute of Harvard and MIT	Adams, G.; Azevedo, K.; B.L.; B.W.; Bauer, M.; Birren; Carter, A.; Chaluvadi, S.; D.J.; DeRuff, K.; Gladden-Young, A.; Huard, R.; J.E.; K.J.; King, E.; Lagerborg, K.; Lemieux; Loreth, C.; Miller, A.; Normandin, E.; P.C.; Park; Pearlman, L.; Reilly, S.; Rudy, M.; Sabeti; Siddle; Tomkins-Tinch, C.; and MacInnis
EPI_ISL_485003 to 485387, EPI_ISL_485873 to 485906	River Road Testing Lab	Ginkgo Bioworks Clinical Laboratory	Alex Plocik; Becky Schilling; Birgitte Simen; E. Handly Mayton; Hollis R. O'Neil; James McGann; Jim Griffin; Keith Robison; Luan V. Dinh; Malaika Mckenzie-Bennett; Michelle Spencer; Rebecca C. Christofferson; Rebecca Littlefield; Stephania A. Cormier; Thaya Stoufflet
EPI_ISL_1534332 to 1534333, EPI_ISL_1534422	Riversdale Clinic wc RAV	NHLS/UCT	Arash Iranzadeh; Bruna Galvao; Carolyn Williamson; Deelan Doolabh; Diana Hardie; Emmanuel SJ; Innocent Mudau; Kruger Marais; Lynn Tyers; Marvin Hsiao; Stephen Korsman; Tegally H; de Oliveira T
EPI_ISL_1520146 to 1520154	Riverside Medical Center	Illinois Department of Public Health - Chicago Lab	Ira Heimler; Vineet K. Dhiman
EPI_ISL_430456	Rizal Medical Center	Research Institute for Tropical Medicine	Bautista; Brunker, K.; C.S.; C.T.; D.L.; Demetria; E.S.; F.G.M.; I.A.P.; Manalo; Medado; Mercado; O.J.T.; Onza; Polotan
EPI_ISL_434517 to 434532, EPI_ISL_435445 to 435472, EPI_ISL_437540 to 437548, EPI_ISL_445119 to 445163, EPI_ISL_450173 to 450185, EPI_ISL_450231	Robert Garry lab	Andersen lab at Scripps Research	Allison Smither; Antoinette Bell; Arnaud Drouin; Dahlene Fusco; Gilberto Sabino-Santos; Kaylynn Genemaras; Lilia Melnik; Patricia Snarski; Robert Garry with SEARCH Alliance San Diego
EPI_ISL_481253, EPI_ISL_481256, EPI_ISL_481262, EPI_ISL_481264	Robert Koch Institute, National Reference center for Influenza, Berlin, Germany	Robert Koch Institute, Bioinformatics MF1, Berlin, Germany	Andrea Thuermer; Marianne Wedde; Max v. Kleist; Oliver Drechsel; Ralf Duerrwald; Rene Kmiecinski; Stephan Fuchs; Thorsten Wolff
EPI_ISL_483139 to 483157	Robert Koch Institute, ZBS1 Highly Pathogenic Viruses, Berlin, Germany	Robert Koch Institute, Bioinformatics MF1, Berlin, Germany	Andrea Thuermer; Andreas Nitsche; Janine Michel; Max v. Kleist; Oliver Drechsel; Rene Kmiecinski; Stephan Fuchs
EPI_ISL_1439128	Robert-Bosch-Krankenhaus	Robert Koch Institute	
EPI_ISL_1447143	Rockefeller University Hospital	New York City Public Health Laboratory	Jade Wang; et al.
EPI_ISL_417032	Rockhampton Base Hospital	Public Health Virology Laboratory	Alyssa Pyke; Amanda De Jong; Andrew Van Den Hurk; Bixing Huang; Carmel Taylor; David Warrilow; Doris Genge; Elisabeth Gamez; Glen Hewitson; Ian Maxwell Mackay; Inga Sultana; Jamie McMahon; Jean Barcelon; Judy Northill; Mitchell Finger; Natalie Simpson; Neelima Nair; Peter Burtonclay; Peter Moore; Sarah Wheatley; Sean Moody; Sonja Hall-Mendelin; Timothy Gardam; and Frederick Moore
EPI_ISL_514131 to 514138	Rondônia Central Public Health Laboratory (LACEN/RO), vinculated to State Health Secretariat of Rondônia (SESAU/RO)	Molecular Virology Laboratory of Oswaldo Cruz Foundation of Rondônia	Adriana Cristina Salvador Maia; Alcione de Oliveira dos Santos; Alice Paula Di Sabatino Guimarães; Aline Linhares Ferreira de Melo Mendonça; Caio Henrique Nemeth Santos; Camila Flávia Gomes Azzi; Celina Aparecida Bertoni Lugtenburg; Cicileia Correia da Silva; Felipe Gomes Naveca; Felipe Souza Nogueira-Lima; Fernando Rodrigues Máximo; Jansen Fernandes de Medeiros; Juan Miguel Vilallobos-Salcedo and Deusilene Souza Vieira; Juan Miguel Vilallobos-Salcedo and Deusilene Souza Vieira.; Juan Miguel Vilallobos-Salcedo and Deusilene Souza Vieira1; Juliana Loca Furtado; Luan Felipo Botelho-Souza; Suelen Cavalcante; Tárcio Peixoto Roca; Rita de Cássia Pontello Rampazzo
EPI_ISL_1388132, EPI_ISL_1388170 to 1388171, EPI_ISL_1388174 to 1388175, EPI_ISL_1388181, EPI_ISL_1388183, EPI_ISL_1388187, EPI_ISL_1388259, EPI_ISL_1388263, EPI_ISL_1388289, EPI_ISL_1388291, EPI_ISL_1388308, EPI_ISL_1388336, EPI_ISL_1388338, EPI_ISL_1388346, EPI_ISL_1388351, EPI_ISL_1388358, EPI_ISL_1388360, EPI_ISL_1388366, EPI_ISL_1388375, EPI_ISL_1388377, EPI_ISL_1388380, EPI_ISL_1388382, EPI_ISL_1388460, EPI_ISL_1388471, EPI_ISL_1388473 to 1388474, EPI_ISL_1388492, EPI_ISL_1388532, EPI_ISL_1388552, EPI_ISL_1388562, EPI_ISL_1388564, EPI_ISL_1388576, EPI_ISL_1388633, EPI_ISL_1388645, EPI_ISL_1388716, EPI_ISL_1388740, EPI_ISL_1388815, EPI_ISL_1388817, EPI_ISL_1389019 to 1389023, EPI_ISL_1389031 to 1389036, EPI_ISL_1389049, EPI_ISL_1389061, EPI_ISL_1389063, EPI_ISL_1389078, EPI_ISL_1389136			
see above	Rothen Medizinische Laboratorien AG	University Hospital Basel, Clinical Bacteriology	Adrian Egli; Alfredo Mari; Hans Hirsch; Helena MB Seth-Smith; Ingrid Steffen; Julia Bielicki; Karoline Leuzinger; Madlen Stange; Manuel Battegay; Tim Roloff
EPI_ISL_436098	Royal Brisbane and Women's Hospital	Public Health Virology Laboratory, Forensic and Scientific Services, Queensland Health	Alyssa Pyke; Amanda De Jong; Andrew Van Den Hurk; Bixing Huang; Carmel Taylor; David Warrilow; Doris Genge; Elisabeth Gamez; Glen Hewitson; Ian Maxwell Mackay; Inga Sultana and Frederick Moore; Jamie McMahon; Jean Barcelon; Judy Northill; Lisa Leckie; Mitchell Finger; Natalie Simpson; Neelima Nair; Peter Burtonclay; Peter Moore; Sarah Wheatley; Sean Moody; Sonja Hall-Mendelin
EPI_ISL_521860 to 521862	Royal Darwin Hospital Pathology	MDU-PHL	Caly L.; Druce J.; Meumann, E.; Sait, M.; Schultz M.; Seemann T.; Sherry, N.
EPI_ISL_492862	Royal Free Hospital / Health Services Laboratories	Wellcome Sanger Institute for the COVID-19 Genomics UK (COG-UK) Consortium	Cate Goodlad; Cordelia Langford; David K. Jackson; Dianne Irish; Dominic Kwiatkowski; Ewan Harrison; Ian Johnston; Jenny Cross; John Sillitoe on behalf of the Wellcome Sanger Institute COVID-19 Surveillance Team; Judith Heaney and Alex Alderton; Roberto Amato; Sonia Goncalves; Tabitha Mahungu; Tanzina Haque
EPI_ISL_492837, EPI_ISL_492840, EPI_ISL_492843 to 492847, EPI_ISL_492850 to 492852, EPI_ISL_492856, EPI_ISL_492863 to 492865, EPI_ISL_492868 to 492869, EPI_ISL_492872, EPI_ISL_492874, EPI_ISL_492876 to 492877, EPI_ISL_492879 to 492880, EPI_ISL_492882 to 492883, EPI_ISL_492885, EPI_ISL_492887 to 492888, EPI_ISL_492890, EPI_ISL_492894, EPI_ISL_492901 to 492902, EPI_ISL_492905 to 492906, EPI_ISL_492908, EPI_ISL_492911, EPI_ISL_492914			
see above	Royal Free Hospital / Health Services Laboratories	Wellcome Sanger Institute for the COVID-19 Genomics UK (COG-UK) consortium	Cate Goodlad; Cordelia Langford; David K. Jackson; Dianne Irish; Dominic Kwiatkowski; Ewan Harrison; Ian Johnston; Jenny Cross; John Sillitoe on behalf of the Wellcome Sanger Institute COVID-19 Surveillance Team (http://www.sanger.ac.uk/covid-team); Judith Heaney and Alex Alderton; Roberto Amato; Sonia Goncalves; Tabitha Mahungu; Tanzina Haque
EPI_ISL_577607	Royal Hobart Hospital	Royal Hobart Hospital	Cooley L.; van Haeften R.
EPI_ISL_522556 to 522646, EPI_ISL_522648 to 522684, EPI_ISL_522686 to 522727, EPI_ISL_522729 to 522769	Royal Hobart Hospital Microbiology Department	MDU-PHL	Cooley L.; M.B.; Sait M.; Schultz; Seemann T.; Sherry N.; van Haeften R.
EPI_ISL_516800	Rumah Sakit Akademik Universitas Gadjah Mada	Genetics Working Group (Pokja Genetik) Faculty of Medicine, Public Health and Nursing Universitas Gadjah Mada (FK-KMK UGM); Disease Investigation Center Wates Ministry of Agriculture Indonesia; Department of Microbiology FK-KMK UGM; Laboratorium Diagnostik Yayasan Tahija World Mosquito Program (WMP) Yogyakarta Center for Tropical Medicine FK-KMK UGM; Integrated Research center FK-KMK UGM; Department of Computer Science and Electronics FMIPA UGM	. Marcellus; Afiahayati; Alvin S. Kalim; Desyifa Mursalin; Dwi AA Nugrahaningsih; Edwin W. Daniwijaya; Eggi Arguni; Endah Supriyati; Gunadi; Hendra Wibawa; Ludhang P. Rizki; Mohamad S. Hakim; Siswanto; Titik Nuryastuti; Tri Wibawa
EPI_ISL_516806	Rumah Sakit PKU Gamping	Genetics Working Group (Pokja Genetik) Faculty of Medicine, Public Health and Nursing Universitas Gadjah Mada (FK-KMK UGM); Disease Investigation Center Wates Ministry of Agriculture Indonesia; Department of Microbiology FK-KMK UGM; Laboratorium Diagnostik Yayasan Tahija World Mosquito Program (WMP) Yogyakarta Center for Tropical Medicine FK-KMK UGM; Integrated Research center FK-KMK UGM; Department of Computer Science and Electronics FMIPA UGM	. Afiahayati; . Marcellus; . Siswanto; Ardorisye Saptaty Fornia; Dwi AA Nugrahaningsih; Edwin W. Daniwijaya; Eggi Arguni; Endah Supriyati; Gunadi; Hendra Wibawa; Kemala Athollah; Ludhang P. Rizki; Mohamad S. Hakim; Titik Nuryastuti; Tri Wibawa
EPI_ISL_491298	Rural Health Unit - Calauan, Laguna	Research Institute for Tropical Medicine	Bautista; Brunker, K.; C.S.; C.T.; D.L.; Demetria; E.S.; F.G.M.; I.A.P.; M.A.A.; Manalo; Medado; Mercado; O.J.T.; Onza; Polotan; Tujan
EPI_ISL_421275, EPI_ISL_454732, EPI_ISL_470896	Russian State Collection of Viruses	Pathogenic Microorganisms Variability Laboratory	Alexander Gintsburg; Alexey Mazus; Alexey Shchetinin; Andrey Botikov; Daria Grousova; Denis Logunov; Denis Protsenko; Ekaterina Aksenova; Elena Shidlovskaya; Inna Dolzhikova; Maria Nikiforova; Marina Kunda; Nadezhda Kuznetsova; Natalia Ryzhova; Olga Voronina; Vladimir Gushchin

EPI_ISL_420051	Résidence Eleusis	National Reference Center for Viruses of Respiratory Infections, Institut Pasteur, Paris	Angela Brisebarre; Etienne Simon-Lorière; Flora Donati; Marion Barbet; Maud Vanpeene; Mélanie Albert; Méline Bizard; Sylvie Behillil; Sylvie van der Werf; Vincent Enouf
EPI_ISL_443304	Résidence Esterel	National Reference Center for Viruses of Respiratory Infections, Institut Pasteur, Paris	Angela Brisebarre; Etienne Simon-Lorière; Flora Donati; Marion Barbet; Maud Vanpeene; Mélanie Albert; Méline Bizard; Sylvie Behillil; Sylvie van der Werf; Vincent Enouf
EPI_ISL_443303	Résidence Les Marines	National Reference Center for Viruses of Respiratory Infections, Institut Pasteur, Paris	Angela Brisebarre; Etienne Simon-Lorière; Flora Donati; Marion Barbet; Maud Vanpeene; Mélanie Albert; Méline Bizard; Sylvie Behillil; Sylvie van der Werf; Vincent Enouf
EPI_ISL_443258 to 443259	Résidence Ornano	National Reference Center for Viruses of Respiratory Infections, Institut Pasteur, Paris	Angela Brisebarre; Etienne Simon-Lorière; Flora Donati; Marion Barbet; Maud Vanpeene; Mélanie Albert; Méline Bizard; Sylvie Behillil; Sylvie van der Werf; Vincent Enouf
EPI_ISL_420046 to 420047	Résidence Villa Caroline	National Reference Center for Viruses of Respiratory Infections, Institut Pasteur, Paris	Angela Brisebarre; Etienne Simon-Lorière; Flora Donati; Marion Barbet; Maud Vanpeene; Mélanie Albert; Méline Bizard; Sylvie Behillil; Sylvie van der Werf; Vincent Enouf
EPI_ISL_420054	Résidence de maintenon	National Reference Center for Viruses of Respiratory Infections, Institut Pasteur, Paris	Angela Brisebarre; Etienne Simon-Lorière; Flora Donati; Marion Barbet; Maud Vanpeene; Mélanie Albert; Méline Bizard; Sylvie Behillil; Sylvie van der Werf; Vincent Enouf
EPI_ISL_420052	Résidence les Marines	National Reference Center for Viruses of Respiratory Infections, Institut Pasteur, Paris	Angela Brisebarre; Etienne Simon-Lorière; Flora Donati; Marion Barbet; Maud Vanpeene; Mélanie Albert; Méline Bizard; Sylvie Behillil; Sylvie van der Werf; Vincent Enouf
EPI_ISL_450193 to 450195, EPI_ISL_451077 to 451148, EPI_ISL_467985 to 468043, EPI_ISL_483066 to 483138, EPI_ISL_492109 to 492180, EPI_ISL_508122 to 508145, EPI_ISL_508605 to 508608, EPI_ISL_510542 to 510544, EPI_ISL_513911 to 513913, EPI_ISL_516550 to 516551, EPI_ISL_602577 to 602581, EPI_ISL_603123 to 603128			
see above	SA Pathology	SA Pathology	Bastian; CK; Chuan Kok Lim; G; Geoff Higgins; Higgins; I; Ivan Bastian; Julien Soubrier; Karin Kassahn; LEX; Leong; Lex Leong; Lim; Luke Walters; M; Mark Turra; Song Gao; Turra
EPI_ISL_455595 to 455603	SA Pathology	VPRL	Beard, MR.; C.K.; Coldbeck-Shackley, R.; Kirby, E.; L.E.X.; Leong; Lim; Llamas, B.; Merrett, J.; Shue, B.; Van Der Hoek, K.
EPI_ISL_1445153 to 1445154, EPI_ISL_1445156	SAE SERVICO DE ATENDIMENTO ESPECIALIZADO	Instituto Butantan / Mendelics	Antonio Jorge Martins; Bibiana Santos; Claudia Renata dos Santos Barros; David Schlesinger; Debora Botequio Moretti; Dimas Tadeu Covas; Elaine Cristina Marqueze; Elaine Vieira dos Santos; Erika Freitas; Evandra Strazza Rodrigues; Flavia Aburjaile; José Salvatore Leister Patané; João Paulo Kitajima; Luiz Carlos Junior de Alcantara; Maria Carolina Elias; Marta Giovanetti; Rafael dos Santos Bezerra; Raul Machado Neto; Ricardo Haddad; Rodrigo Tocantins Calado.; Sandra Coccuzzo Sampaio; Simone Kashima; Svetoslav Nanev Slavov; Vagner Fonseca; Vincent Louis Viala
EPI_ISL_1468428	SAE Servico de Atendimento Especializado	Instituto Adolfo Lutz, Interdiciplinary Procedures Center, Strategic Laboratory	Caio Vinicius Dias Lopes; Claudia Regina Gonçalves; Claudio Tavares Sacchi; Erica Valessa Ramos Gomes; Karoline Rodrigues Campos
EPI_ISL_1393790 to 1393792	SALVE MEDICA	1. National Institute of Public Health - National Institute of Hygiene; 2. Eurofins Genomics Europe Sequencing GmbH	ECDC COVID-19 WGS support team; Eurofins Genomics Europe Sequencing Team; Gierczyki Rafa; Sadkowska-Todys Magorzata; Wokowicz Tomasz; Zacharczuk Katarzyna
EPI_ISL_1398269	SARATOGA HOSPITAL LABORATORY	Wadsworth Center, New York State Department of Health	Alexis Russel; Daryl M. Lamson; Erasmus Schneider; Erica Lasek-Nesselquist; John Kelly; Jonathan Plitnick; Kirsten St. George; Matthew Shudt; Melissa A Leisner; Navjot Singh
EPI_ISL_1425573 to 1425578, EPI_ISL_1425586, EPI_ISL_1425588 to 1425600, EPI_ISL_1425605 to 1425613, EPI_ISL_1425615, EPI_ISL_1425617 to 1425619, EPI_ISL_1425699, EPI_ISL_1425704 to 1425705, EPI_ISL_1425712, EPI_ISL_1425714 to 1425716, EPI_ISL_1425719 to 1425720, EPI_ISL_1425725 to 1425734, EPI_ISL_1425749 to 1425776, EPI_ISL_1426046 to 1426047, EPI_ISL_1426266 to 1426291, EPI_ISL_1426570 to 1426572, EPI_ISL_1426575 to 1426579, EPI_ISL_1426587 to 1426588, EPI_ISL_1426590, EPI_ISL_1426648 to 1426649, EPI_ISL_1426992 to 1426995, EPI_ISL_1427570 to 1427572, EPI_ISL_1427577 to 1427584, EPI_ISL_1427602 to 1427606, EPI_ISL_1427610, EPI_ISL_1427654, EPI_ISL_1427683 to 1427686, EPI_ISL_1427693, EPI_ISL_1427800 to 1427829, EPI_ISL_1428571 to 1428573, EPI_ISL_1428588 to 1428589, EPI_ISL_1428591 to 1428593, EPI_ISL_1428600, EPI_ISL_1428602 to 1428603, EPI_ISL_1428632 to 1428635, EPI_ISL_1428640, EPI_ISL_1429583, EPI_ISL_1429589, EPI_ISL_1429592, EPI_ISL_1430578, EPI_ISL_1430580, EPI_ISL_1430584, EPI_ISL_1430625, EPI_ISL_1430627 to 1430634, EPI_ISL_1430639 to 1430652, EPI_ISL_1430656 to 1430667, EPI_ISL_1430697, EPI_ISL_1430736 to 1430737, EPI_ISL_1430755 to 1430762, EPI_ISL_1430825 to 1430828, EPI_ISL_1430868 to 1430874, EPI_ISL_1431129 to 1431135, EPI_ISL_1431479, EPI_ISL_1431578, EPI_ISL_1433299, EPI_ISL_1433301, EPI_ISL_1433303, EPI_ISL_1433306			
see above	SARS-CoV-2 testing team, National Institute of Infectious Diseases	Pathogen Genomics Center, National Institute of Infectious Diseases	; Chang-Kweng Lim; Chikako Shimokawa; Daisuke Kobayashi; Eri Nakayama; Eunsil Park; Hazuka Yoshida; Hidemasa Izumiya; Hideyuki Takahashi; Hiromizu Takahashi; Hiroshi Ishii; Hussein H. Aly; Ken Miyazawa; Kentaro Itokawa; Kento Fukano; Koichi Ishikawa; Kousho Wakae; Lucky Ronald Runtuwene; Makoto Kuroda; Masanori Hashino; Masatomo Morita; Midori Nakamura-Hoshi; Minoru Nagi; Motohiko Ogawa; Naomi Nojiri; Nobuo Koizumi; Nozomu Hanaoka; Rina Tanaka; Sayuri Seki; Shigeru Kusagawa; Shigeru Tanjima; Sunao Iyoda; Tadashi Kikuchi; Takahiro Maeki; Takanobu Kato; Takashi Sakudoh; Tsuguto Fujimoto; Tsuyoshi Sekizuka; Yasutaka Hoshino; Yasuyuki Morishima; Yoshihiro Kaku
EPI_ISL_1445080, EPI_ISL_1445085, EPI_ISL_1445177	SAUDE COLETIVA CAPAO BONITO	Instituto Butantan / Mendelics	Antonio Jorge Martins; Bibiana Santos; Claudia Renata dos Santos Barros; David Schlesinger; Debora Botequio Moretti; Dimas Tadeu Covas; Elaine Cristina Marqueze; Elaine Vieira dos Santos; Erika Freitas; Evandra Strazza Rodrigues; Flavia Aburjaile; José Salvatore Leister Patané; João Paulo Kitajima; Luiz Carlos Junior de Alcantara; Maria Carolina Elias; Marta Giovanetti; Rafael dos Santos Bezerra; Raul Machado Neto; Ricardo Haddad; Rodrigo Tocantins Calado.; Sandra Coccuzzo Sampaio; Simone Kashima; Svetoslav Nanev Slavov; Vagner Fonseca; Vincent Louis Viala
EPI_ISL_1509732 to 1509736, EPI_ISL_1509742 to 1509745, EPI_ISL_1509751, EPI_ISL_1510420 to 1510479	SC (UCO) Igiene e Sanità Pubblica (funzione integrata con SC Microbiologia e Virologia)	ARGO Laboratorio Genomica ed Epigenomica	D'Agaro P; Dal Monego S; De Rosa R; Degasperi M; Licastro D; Lombardo F; Marcello A; Pipan C; Piscianz E; Segat L
EPI_ISL_1509721 to 1509731, EPI_ISL_1509746, EPI_ISL_1509748 to 1509750, EPI_ISL_1509752 to 1509753, EPI_ISL_1509755 to 1509758	SC (UCO) Igiene e Sanità Pubblica (funzione integrata con SC Microbiologia e Virologia) e Az. Ospedaliero-Universitaria Udine	ARGO Laboratorio Genomica ed Epigenomica	D'Agaro P; Dal Monego S; De Rosa R; Degasperi M; Licastro D; Marcello A; Piscianz E; Segat L
EPI_ISL_1509737 to 1509741, EPI_ISL_1509747, EPI_ISL_1509754	SC (UCO) Igiene e Sanità Pubblica (funzione integrata con SC Microbiologia e Virologia) e Azienda Ospedaliera Pordenone	ARGO Laboratorio Genomica ed Epigenomica	D'Agaro P; Dal Monego S; Degasperi M; Licastro D; Marcello A; Piscianz E; Segat L
EPI_ISL_491097 to 491110	SC Department of Health and Environmental Control	SC Department of Health and Environmental Control	Flores, H.
EPI_ISL_1446706 to 1446711, EPI_ISL_1446735 to 1446737, EPI_ISL_1446976, EPI_ISL_1516269 to 1516275, EPI_ISL_1528270 to 1528271	SC Dept of Health and Env. Control-Bureau of Laboratories	Centers for Disease Control and Prevention Division of Viral Diseases, Pathogen Discovery	Alison Laufer Halpin; Ben L. Rambo-Martin; Clinton R. Paden; Dakota Howard; Darlene Wagner; Dave Wentworth; Dhwani Batra; Jasmine Padilla; Justin Lee; Katie Dillon; Krista Queen; Kristen Knipe; Kristine Lacek; Mark Burroughs; Matthew Schmerer; Mili Sheth; Peter Cook; Sam Shepard; Sarah Nobles; Shoshona Le; Suxiang Tong; Vivien Dugan; Yvette Unoarumhi
EPI_ISL_424841 to 424842, EPI_ISL_424888, EPI_ISL_424902 to 424905	SC Dept of Health and Env. Control-Bureau of Laboratories	Pathogen Discovery, Respiratory Viruses Branch, Division of Viral Diseases, Centers for Disease Control and Prevention	Alison S. Laufer Halpin; Anna Uehara; Christopher A. Elkins; Clinton R. Paden; Haibin Wang; Jing Zhang; Krista Queen; Mary S. Keckler; Rachel Marine; Suxiang Tong; Yan Li; Ying Tao
EPI_ISL_1446702 to 1446705, EPI_ISL_1446989 to 1446991, EPI_ISL_1516169 to 1516171, EPI_ISL_1528266 to 1528269	SD Public Health Laboratory	Centers for Disease Control and Prevention Division of Viral Diseases, Pathogen Discovery	Alison Laufer Halpin; Ben L. Rambo-Martin; Clinton R. Paden; Dakota Howard; Darlene Wagner; Dave Wentworth; Dhwani Batra; Jasmine Padilla; Justin Lee; Katie Dillon; Krista Queen; Kristen Knipe; Kristine Lacek; Mark Burroughs; Matthew Schmerer; Mili Sheth; Peter Cook; Sam Shepard; Sarah Nobles; Shoshona Le; Suxiang Tong; Vivien Dugan; Yvette Unoarumhi
EPI_ISL_569617	SD Urban Indian Health Pierre	South Dakota Public Health Laboratory	Jacob Garfin; Matt Plumb; Xiong Wang; and Chris Carlson
EPI_ISL_1445157 to 1445161, EPI_ISL_1445168 to 1445170, EPI_ISL_1445238 to 1445248, EPI_ISL_1445252 to 1445253, EPI_ISL_1445262	SECAO CENTRO DE DIAGNOSTICO SECEDI	Instituto Butantan / Mendelics	Antonio Jorge Martins; Bibiana Santos; Claudia Renata dos Santos Barros; David Schlesinger; Debora Botequio Moretti; Dimas Tadeu Covas; Elaine Cristina Marqueze; Elaine Vieira dos Santos; Erika Freitas; Evandra Strazza Rodrigues; Flavia Aburjaile; José Salvatore Leister Patané; João Paulo Kitajima; Luiz Carlos Junior de Alcantara; Maria Carolina Elias; Marta Giovanetti; Rafael dos Santos Bezerra; Raul Machado Neto; Ricardo Haddad; Rodrigo Tocantins Calado.; Sandra Coccuzzo Sampaio; Simone Kashima; Svetoslav Nanev Slavov; Vagner Fonseca; Vincent Louis Viala
EPI_ISL_1445066	SECRETARIA MUNICIPAL DA SAUDE DE GUARIBA	Instituto Butantan / Mendelics	Antonio Jorge Martins; Bibiana Santos; Claudia Renata dos Santos Barros; David Schlesinger; Debora Botequio Moretti; Dimas Tadeu Covas; Elaine Cristina Marqueze; Elaine Vieira dos Santos; Erika Freitas; Evandra Strazza Rodrigues; Flavia Aburjaile; José Salvatore Leister Patané; João Paulo Kitajima; Luiz Carlos Junior de Alcantara; Maria Carolina Elias; Marta Giovanetti; Rafael dos Santos Bezerra; Raul Machado Neto; Ricardo Haddad; Rodrigo Tocantins Calado.; Sandra Coccuzzo Sampaio; Simone Kashima; Svetoslav Nanev Slavov; Vagner Fonseca; Vincent Louis Viala
EPI_ISL_1445091 to 1445095, EPI_ISL_1445101	SECRETARIA MUNICIPAL DE SAUDE DE PIRACAIA	Instituto Butantan / Mendelics	Antonio Jorge Martins; Bibiana Santos; Claudia Renata dos Santos Barros; David Schlesinger; Debora Botequio Moretti; Dimas Tadeu Covas; Elaine Cristina Marqueze; Elaine Vieira dos Santos; Erika Freitas; Evandra Strazza Rodrigues; Flavia Aburjaile; José Salvatore Leister Patané; João Paulo Kitajima; Luiz Carlos Junior de Alcantara; Maria Carolina Elias; Marta Giovanetti; Rafael dos Santos Bezerra; Raul Machado Neto; Ricardo Haddad;

			Rodrigo Tocantins Calado.; Sandra Coccuzzo Sampaio; Simone Kashima; Svetoslav Nanev Slavov; Vagner Fonseca; Vincent Louis Viala
EPI_ISL_1469568, EPI_ISL_1469639, EPI_ISL_1469690, EPI_ISL_1469736, EPI_ISL_1469788, EPI_ISL_1469814	SECRETARIA MUNICIPAL DE SAUDE DE SAO LEOPOLDO	Epiclin	Ana Paula Muterle; Carolina Comerlato; Eliana Márcia Da Ros Wendland; Fernando Hayashi Sant'Anna; Janira Prichula; Juliana Comerlato
EPI_ISL_1469601, EPI_ISL_1469683, EPI_ISL_1469687, EPI_ISL_1469709, EPI_ISL_1479130	SECRETARIA MUNICIPAL DE SAUDE DE TAQUARA	Epiclin	Ana Paula Muterle; Carolina Comerlato; Eliana Márcia Da Ros Wendland; Fernando Hayashi Sant'Anna; Janira Prichula; Juliana Comerlato
EPI_ISL_1469645, EPI_ISL_1469678, EPI_ISL_1469740	SECRETARIA MUNICIPAL DE SAUDE DE TRES COROAS	Epiclin	Ana Paula Muterle; Carolina Comerlato; Eliana Márcia Da Ros Wendland; Fernando Hayashi Sant'Anna; Janira Prichula; Juliana Comerlato
EPI_ISL_1445082, EPI_ISL_1445087 to 1445088, EPI_ISL_1445133 to 1445135, EPI_ISL_1445137 to 1445139, EPI_ISL_1445171, EPI_ISL_1445176, EPI_ISL_1445178 to 1445180, EPI_ISL_1445182			
see above	SECRETARIA MUNICIPAL DE SAUDE SOROCABA	Instituto Butantan / Mendelics	Antonio Jorge Martins; Bibiana Santos; Claudia Renata dos Santos Barros; David Schlesinger; Debora Botequio Moretti; Dimas Tadeu Covas; Elaine Cristina Marqueze; Elaine Vieira dos Santos; Erika Freitas; Evandra Strazza Rodrigues; Flavia Aburjaile; José Salvatore Leister Patané; João Paulo Kitajima; Luiz Carlos Junior de Alcantara; Maria Carolina Elias; Marta Giovanetti; Rafael dos Santos Bezerra; Raul Machado Neto; Ricardo Haddad; Rodrigo Tocantins Calado.; Sandra Coccuzzo Sampaio; Simone Kashima; Svetoslav Nanev Slavov; Vagner Fonseca; Vincent Louis Viala
EPI_ISL_1433784, EPI_ISL_1433801, EPI_ISL_1433818, EPI_ISL_1433888, EPI_ISL_1433890, EPI_ISL_1433898, EPI_ISL_1433902, EPI_ISL_1433904, EPI_ISL_1433914, EPI_ISL_1433919, EPI_ISL_1433921, EPI_ISL_1433923, EPI_ISL_1434061, EPI_ISL_1434205, EPI_ISL_1434207, EPI_ISL_1434209, EPI_ISL_1434211, EPI_ISL_1434213, EPI_ISL_1434215, EPI_ISL_1434225 to 1434226, EPI_ISL_1434228, EPI_ISL_1434231, EPI_ISL_1434233, EPI_ISL_1434259, EPI_ISL_1434262, EPI_ISL_1457762, EPI_ISL_1457764, EPI_ISL_1457766 to 1457767			
see above	SELARL MIRIALIS CLUSES	CNR Virus des Infections Respiratoires - France SUD	Antonin Bal; Bruno Lina; Gregory Destras; Gwendolyne Burfin; Hadrien Regue; Laurence Josset; Martine Valette; Quentin Semanas
EPI_ISL_1445162 to 1445167, EPI_ISL_1445269, EPI_ISL_1445272	SERV DE VIG SANITARIA EPIDEMIO E CTRL DE ZOONOZES GUARUJA	Instituto Butantan / Mendelics	Antonio Jorge Martins; Bibiana Santos; Claudia Renata dos Santos Barros; David Schlesinger; Debora Botequio Moretti; Dimas Tadeu Covas; Elaine Cristina Marqueze; Elaine Vieira dos Santos; Erika Freitas; Evandra Strazza Rodrigues; Flavia Aburjaile; José Salvatore Leister Patané; João Paulo Kitajima; Luiz Carlos Junior de Alcantara; Maria Carolina Elias; Marta Giovanetti; Rafael dos Santos Bezerra; Raul Machado Neto; Ricardo Haddad; Rodrigo Tocantins Calado.; Sandra Coccuzzo Sampaio; Simone Kashima; Svetoslav Nanev Slavov; Vagner Fonseca; Vincent Louis Viala
EPI_ISL_435149, EPI_ISL_435153 to 435155, EPI_ISL_436726 to 436729	SERVIZIO DI IGIENE E SANITÀ PUBBLICA ASL Teramo	Istituto Zooprofilattico Sperimentale dell'Abruzzo e Molise "G.Caporale"	Ancora M; Cammà C; Curini V; Di Domenico M; Di Pasquale A; Lorusso A; Mangone I; Marcacci M; Puglia I; Rinaldi A; Savini G
EPI_ISL_515956	SHEKAR HOSPITAL	Department of Neurovirology, National Institute of Mental Health and Neuroscience (NIMHANS)	Anita Desai; Chitra Pattabiraman; Harsha PK; Manjunatha Venkataswamy; Pramada Prasad; Ravi Vasanthapuram; Risha Rasheed; Shafeeq S Hameed; Vijayalakshmi Reddy
EPI_ISL_1403534, EPI_ISL_1502441	SIESP CHIETI - DRIVE IN ATESSA ATESSA(CHIETI)	Istituto Zooprofilattico Sperimentale dell'Abruzzo e Molise "G. Caporale"	Ancora M; Calistri P; Cammà C; Caporale M; Curini V; Delli Compagni E; Di Domenico M; Di Lollo Valeria; Di Pasquale A; Lorusso A; Mangone I; Marcacci M; Puglia I; Rinaldi A; Savini G; Scialabba S
EPI_ISL_1391396, EPI_ISL_1391405	SIESP CHIETI - DRIVE IN CHIETI	Istituto Zooprofilattico Sperimentale dell'Abruzzo e Molise "G. Caporale"	Ancora M; Calistri P; Cammà C; Caporale M; Curini V; Di Domenico M; Di Lollo Valeria; Di Pasquale A; Lorusso A; Mangone I; Marcacci M; Puglia I; Rinaldi A; Savini G; Scialabba S
EPI_ISL_1391266 to 1391270, EPI_ISL_1392680, EPI_ISL_1392698 to 1392701, EPI_ISL_1392710, EPI_ISL_1403519, EPI_ISL_1403525, EPI_ISL_1403528, EPI_ISL_1403566, EPI_ISL_1412935, EPI_ISL_1502466, EPI_ISL_1502489 to 1502490, EPI_ISL_1502506, EPI_ISL_1502539, EPI_ISL_1524857, EPI_ISL_1524860			
see above	SIESP CHIETI - DRIVE IN CHIETI CHIETI(CHIETI)	Istituto Zooprofilattico Sperimentale dell'Abruzzo e Molise "G. Caporale"	Ancora M; Calistri P; Cammà C; Caporale M; Curini V; Delli Compagni E; Di Domenico M; Di Lollo Valeria; Di Pasquale A; Lorusso A; Mangone I; Marcacci M; Puglia I; Rinaldi A; Savini G; Scialabba S
EPI_ISL_1391275, EPI_ISL_1392691 to 1392693, EPI_ISL_1403503, EPI_ISL_1403505 to 1403507, EPI_ISL_1502450 to 1502451, EPI_ISL_1502487 to 1502488, EPI_ISL_1502531, EPI_ISL_1524859			
see above	SIESP CHIETI - DRIVE IN GISSI GISSI(CHIETI)	Istituto Zooprofilattico Sperimentale dell'Abruzzo e Molise "G. Caporale"	Ancora M; Calistri P; Cammà C; Caporale M; Curini V; Delli Compagni E; Di Domenico M; Di Lollo Valeria; Di Pasquale A; Lorusso A; Mangone I; Marcacci M; Puglia I; Rinaldi A; Savini G; Scialabba S
EPI_ISL_1415426	SIESP CHIETI - DRIVE IN LANCIANO	Istituto Zooprofilattico Sperimentale dell'Abruzzo e Molise "G. Caporale"	Ancora M; Calistri P; Cammà C; Caporale M; Curini V; Delli Compagni E; Di Domenico M; Di Lollo Valeria; Di Pasquale A; Lorusso A; Mangone I; Marcacci M; Puglia I; Rinaldi A; Savini G; Scialabba S
EPI_ISL_1391230 to 1391233, EPI_ISL_1391252, EPI_ISL_1391260 to 1391262, EPI_ISL_1391265, EPI_ISL_1392681, EPI_ISL_1392695 to 1392697, EPI_ISL_1403532, EPI_ISL_1403560, EPI_ISL_1403604, EPI_ISL_1403606, EPI_ISL_1412932, EPI_ISL_1502440, EPI_ISL_1502442 to 1502444, EPI_ISL_1502473, EPI_ISL_1502482 to 1502483, EPI_ISL_1524863 to 1524864, EPI_ISL_1524867			
see above	SIESP CHIETI - DRIVE IN LANCIANO LANCIANO(CHIETI)	Istituto Zooprofilattico Sperimentale dell'Abruzzo e Molise "G. Caporale"	Ancora M; Calistri P; Cammà C; Caporale M; Curini V; Delli Compagni E; Di Domenico M; Di Lollo Valeria; Di Pasquale A; Lorusso A; Mangone I; Marcacci M; Puglia I; Rinaldi A; Savini G; Scialabba S
EPI_ISL_1391397, EPI_ISL_1391399 to 1391400, EPI_ISL_1391408, EPI_ISL_1396650	SIESP CHIETI - DRIVE IN ORTONA	Istituto Zooprofilattico Sperimentale dell'Abruzzo e Molise "G. Caporale"	Ancora M; Calistri P; Cammà C; Caporale M; Curini V; Di Domenico M; Di Lollo Valeria; Di Pasquale A; Lorusso A; Mangone I; Marcacci M; Puglia I; Rinaldi A; Savini G; Scialabba S
EPI_ISL_1391271 to 1391273, EPI_ISL_1392702, EPI_ISL_1403509, EPI_ISL_1403521, EPI_ISL_1403543, EPI_ISL_1403568, EPI_ISL_1403581, EPI_ISL_1412929, EPI_ISL_1415423, EPI_ISL_1420829 to 1420830, EPI_ISL_1502447 to 1502448, EPI_ISL_1502471, EPI_ISL_1502485 to 1502486, EPI_ISL_1502507, EPI_ISL_1502538			
see above	SIESP CHIETI - DRIVE IN ORTONA ORTONA(CHIETI)	Istituto Zooprofilattico Sperimentale dell'Abruzzo e Molise "G. Caporale"	Ancora M; Calistri P; Cammà C; Caporale M; Curini V; Delli Compagni E; Di Domenico M; Di Lollo Valeria; Di Pasquale A; Lorusso A; Mangone I; Marcacci M; Puglia I; Rinaldi A; Savini G; Scialabba S
EPI_ISL_1392688 to 1392689, EPI_ISL_1392694, EPI_ISL_1403535, EPI_ISL_1403559, EPI_ISL_1502445 to 1502446, EPI_ISL_1502484, EPI_ISL_1524858			
see above	SIESP CHIETI - DRIVE IN VASTO VASTO(CHIETI)	Istituto Zooprofilattico Sperimentale dell'Abruzzo e Molise "G. Caporale"	Ancora M; Calistri P; Cammà C; Caporale M; Curini V; Delli Compagni E; Di Domenico M; Di Lollo Valeria; Di Pasquale A; Lorusso A; Mangone I; Marcacci M; Puglia I; Rinaldi A; Savini G; Scialabba S
EPI_ISL_1391393, EPI_ISL_1391404, EPI_ISL_1396653	SIESP DIPARTIMENTO DI PREVENZIONE CHIETI	Istituto Zooprofilattico Sperimentale dell'Abruzzo e Molise "G. Caporale"	Ancora M; Calistri P; Cammà C; Caporale M; Curini V; Di Domenico M; Di Lollo Valeria; Di Pasquale A; Lorusso A; Mangone I; Marcacci M; Puglia I; Rinaldi A; Savini G; Scialabba S
EPI_ISL_1391249 to 1391251, EPI_ISL_1391253 to 1391259, EPI_ISL_1391263 to 1391264, EPI_ISL_1392666, EPI_ISL_1392690, EPI_ISL_1403533, EPI_ISL_1403577, EPI_ISL_1502439, EPI_ISL_1502491, EPI_ISL_1502500 to 1502502, EPI_ISL_1502530			
see above	SIESP DIPARTIMENTO DI PREVENZIONE CHIETI CHIETI(CHIETI)	Istituto Zooprofilattico Sperimentale dell'Abruzzo e Molise "G. Caporale"	Ancora M; Calistri P; Cammà C; Caporale M; Curini V; Delli Compagni E; Di Domenico M; Di Lollo Valeria; Di Pasquale A; Lorusso A; Mangone I; Marcacci M; Puglia I; Rinaldi A; Savini G; Scialabba S
EPI_ISL_1391394, EPI_ISL_1391401 to 1391402, EPI_ISL_1391407, EPI_ISL_1396651 to 1396652	SIESP DIPARTIMENTO DI PREVENZIONE TERAMO	Istituto Zooprofilattico Sperimentale dell'Abruzzo e Molise "G. Caporale"	Ancora M; Calistri P; Cammà C; Caporale M; Curini V; Di Domenico M; Di Lollo Valeria; Di Pasquale A; Lorusso A; Mangone I; Marcacci M; Puglia I; Rinaldi A; Savini G; Scialabba S
EPI_ISL_1391204 to 1391229, EPI_ISL_1391274, EPI_ISL_1392652 to 1392662, EPI_ISL_1392682, EPI_ISL_1392703 to 1392709, EPI_ISL_1392713, EPI_ISL_1403502, EPI_ISL_1403518, EPI_ISL_1403520, EPI_ISL_1403522, EPI_ISL_1403540, EPI_ISL_1403549 to 1403551, EPI_ISL_1403553 to 1403554, EPI_ISL_1403556 to 1403558, EPI_ISL_1403561 to 1403565, EPI_ISL_1403567, EPI_ISL_1403569 to 1403575, EPI_ISL_1403578 to 1403580, EPI_ISL_1403582 to 1403583, EPI_ISL_1403592, EPI_ISL_1403602 to 1403603, EPI_ISL_1403605, EPI_ISL_1415420, EPI_ISL_1415424, EPI_ISL_1502435 to 1502438, EPI_ISL_1502452 to 1502459, EPI_ISL_1502461 to 1502465, EPI_ISL_1502467 to 1502470, EPI_ISL_1502480, EPI_ISL_1502492 to 1502497, EPI_ISL_1502499, EPI_ISL_1502503 to 1502505, EPI_ISL_1502518, EPI_ISL_1502532 to 1502533, EPI_ISL_1524852 to 1524856, EPI_ISL_1524861 to 1524862, EPI_ISL_1524869 to 1524870			
see above	SIESP DIPARTIMENTO DI PREVENZIONE TERAMO TERAMO(TERAMO)	Istituto Zooprofilattico Sperimentale dell'Abruzzo e Molise "G. Caporale"	Ancora M; Calistri P; Cammà C; Caporale M; Curini V; Delli Compagni E; Di Domenico M; Di Lollo Valeria; Di Pasquale A; Lorusso A; Mangone I; Marcacci M; Puglia I; Rinaldi A; Savini G; Scialabba S
EPI_ISL_1391398, EPI_ISL_1391406	SIESP SULMONA	Istituto Zooprofilattico Sperimentale dell'Abruzzo e Molise "G. Caporale"	Ancora M; Calistri P; Cammà C; Caporale M; Curini V; Di Domenico M; Di Lollo Valeria; Di Pasquale A; Lorusso A; Mangone I; Marcacci M; Puglia I; Rinaldi A; Savini G; Scialabba S
EPI_ISL_1391234 to 1391237, EPI_ISL_1391239 to 1391240, EPI_ISL_1391248, EPI_ISL_1392670 to 1392671, EPI_ISL_1392674, EPI_ISL_1403511, EPI_ISL_1403514, EPI_ISL_1403523 to 1403524, EPI_ISL_1403527, EPI_ISL_1403536, EPI_ISL_1403538 to 1403539, EPI_ISL_1403576, EPI_ISL_1502474, EPI_ISL_1502508 to 1502511, EPI_ISL_1502513, EPI_ISL_1524865 to 1524866, EPI_ISL_1524871			
see above	SIESP SULMONA SULMONA(L'AQUILA)	Istituto Zooprofilattico Sperimentale dell'Abruzzo e Molise "G. Caporale"	Ancora M; Calistri P; Cammà C; Caporale M; Curini V; Delli Compagni E; Di Domenico M; Di Lollo Valeria; Di Pasquale A; Lorusso A; Mangone I; Marcacci M; Puglia I; Rinaldi A; Savini G; Scialabba S
EPI_ISL_1478852, EPI_ISL_1478881	SIU Center for Family Medicine - Springfield Main	Illinois Department of Public Health - Springfield Lab	Bryan Sim; Gordon McCall

EPI_ISL_1478888 to 1478889, EPI_ISL_1478891 to 1478894, EPI_ISL_1478901, EPI_ISL_1478903 to 1478904	SIU Center for Family Medicine - Springfield SCDPH	Illinois Department of Public Health - Springfield Lab	Bryan Sim; Gordon McCall
EPI_ISL_1478925	SIU Center for Family Medicine- SPFLD Main	Illinois Department of Public Health - Springfield Lab	Bryan Sim; Gordon McCall
EPI_ISL_1500180 to 1500182, EPI_ISL_1500197 to 1500200, EPI_ISL_1500213 to 1500215	SIU Center for Family Medicine- SPFLD SCDPH	Illinois Department of Public Health - Springfield Lab	Bryan Sim; Gordon McCall
EPI_ISL_486393	SJMCH	Department of Neurovirology, National Institute of Mental Health and Neuroscience (NIMHANS)	Anita Desai; Chitra Pattabiraman; Harsha PK; Manjunatha Venkataswamy; Ravi Vasanthapuram; Risha Rasheed; Shafeeq S Hameed; Vijayalakshmi Reddy
EPI_ISL_1438583, EPI_ISL_1438642, EPI_ISL_1438655	SLK-Kliniken Heilbronn GmbH - Institut für Laboratoriumsmedizin	Robert Koch Institute	
EPI_ISL_1445136	SMS IPERO	Instituto Butantan / Mendelics	Antonio Jorge Martins; Bibiana Santos; Claudia Renata dos Santos Barros; David Schlesinger; Debora Botequio Moretti; Dimas Tadeu Covas; Elaine Cristina Marqueze; Elaine Vieira dos Santos; Erika Freitas; Evandra Strazza Rodrigues; Flavia Aburjaile; José Salvatore Leister Patané; João Paulo Kitajima; Luiz Carlos Junior de Alcantara; Maria Carolina Elias; Marta Giovanetti; Rafael dos Santos Bezerra; Raul Machado Neto; Ricardo Haddad; Rodrigo Tocantins Calado.; Sandra Coccuzzo Sampaio; Simone Kashima; Svetoslav Nanev Slavov; Vagner Fonseca; Vincent Louis Viala
EPI_ISL_454830 to 454833	SMS Medical College, Jaipur	CSIR Institute of Genomics and Integrative Biology	Abhinav Jain; Anshul Kumar; Bani Jolly; Disha Sharma; Gyan Ranjan; Mohammed Imran; Mohit Divakar; Paras Sehgal; Rahul Bhoyar; Rahul Sahlot; Sandeep K Mathur; Sridhar Sivasubbu; Sudhir Bhandari; Vinod Scaria
EPI_ISL_1445084	SMS SECRETARIA MUNICIPAL DE SAUDE DE BOITUVA	Instituto Butantan / Mendelics	Antonio Jorge Martins; Bibiana Santos; Claudia Renata dos Santos Barros; David Schlesinger; Debora Botequio Moretti; Dimas Tadeu Covas; Elaine Cristina Marqueze; Elaine Vieira dos Santos; Erika Freitas; Evandra Strazza Rodrigues; Flavia Aburjaile; José Salvatore Leister Patané; João Paulo Kitajima; Luiz Carlos Junior de Alcantara; Maria Carolina Elias; Marta Giovanetti; Rafael dos Santos Bezerra; Raul Machado Neto; Ricardo Haddad; Rodrigo Tocantins Calado.; Sandra Coccuzzo Sampaio; Simone Kashima; Svetoslav Nanev Slavov; Vagner Fonseca; Vincent Louis Viala
EPI_ISL_1544075 to 1544107	SOC Igiene ed Epidemiologia Clinica, ASUFC, Udine and SC (UCO) Igiene e Sanità Pubblica, ASUGI, Trieste	Istituto di Genomica Applicata	Corrado Pipan; Davide Scaglione; Eleonora Paparelli; Gabriele Magris; Irena Jurman; Michele Morgante; Pierlanfranco D'Agaro; Vera Vendramin
EPI_ISL_1393780 to 1393782, EPI_ISL_1393801, EPI_ISL_1538796, EPI_ISL_1539054, EPI_ISL_1539133	SP ZOZ Centralny Szpital Kliniczny UM w odzi Pracownia Diagnostyki Wirusów Oddechowych	1. National Institute of Public Health - National Institute of Hygiene; 2. Eurofins Genomics Europe Sequencing GmbH	ECDC COVID-19 WGS support team; Eurofins Genomics Europe Sequencing Team; Gierczyki Rafa; Sadkowska-Todys Magorzata; Wokowicz Tomasz; Zacharczuk Katarzyna
EPI_ISL_1538790 to 1538793	SP ZOZ USK nr 1 Zakad Diagnostyki Laboratoryjnej	1. National Institute of Public Health - National Institute of Hygiene; 2. Eurofins Genomics Europe Sequencing GmbH	ECDC COVID-19 WGS support team; Eurofins Genomics Europe Sequencing Team; Gierczyki Rafa; Sadkowska-Todys Magorzata; Wokowicz Tomasz; Zacharczuk Katarzyna
EPI_ISL_1393785 to 1393788	SP ZOZ Uniwersytecki Szpital Kliniczny nr 1 im. Norberta Barlickiego	1. National Institute of Public Health - National Institute of Hygiene; 2. Eurofins Genomics Europe Sequencing GmbH	ECDC COVID-19 WGS support team; Eurofins Genomics Europe Sequencing Team; Gierczyki Rafa; Sadkowska-Todys Magorzata; Wokowicz Tomasz; Zacharczuk Katarzyna
EPI_ISL_1393794	SP ZOZ Uniwersytecki Szpital Kliniczny nr 1 im. Norberta Barlickiego Uniwersytetu Medycznego w odzi	1. National Institute of Public Health - National Institute of Hygiene; 2. Eurofins Genomics Europe Sequencing GmbH	ECDC COVID-19 WGS support team; Eurofins Genomics Europe Sequencing Team; Gierczyki Rafa; Sadkowska-Todys Magorzata; Wokowicz Tomasz; Zacharczuk Katarzyna
EPI_ISL_1544142 to 1544165, EPI_ISL_1544170 to 1544178	SSG Medical College, Baroda	Gujarat Biotechnology Research Centre	Chaitanya Joshi; Dinesh Kumar; Janvi Raval; Madhvi Joshi; Nitesh Shah; Nitin Savaliya; Ramesh Pandit; Sonal Sharma; Tanuja B. Javadekar; Twinkle Soni; Umang Mishra; Zarna Patel; Zuber Saiyed
EPI_ISL_1482454	ST. FRANCIS HOSPITAL LABORATORY	Wadsworth Center, New York State Department of Health	Alexis Russell; Catharine Prussing; Daryl M. Lamson; Erasmus Schneider; Erica Lasek-Nesselquist; John Kelly; Jonathan Plitnick; Kirsten St. George; Matthew Shudt; Melissa A Leisner; Navjot Singh
EPI_ISL_1469437	ST. JOZEFKLINIEK-Campus Willebroek	UZA, Clinical Biology	Basil Britto Xavier; Christine Lammens; Herman Goossens; Jasmine Coppens; Marie Le Mercier; Veerle Matheeussen
EPI_ISL_1397831 to 1397832, EPI_ISL_1465838 to 1465847	STONY BROOK UNIVERSITY HOSPITAL	Wadsworth Center, New York State Department of Health	Alexis Russel; Alexis Russell; Catharine Prussing; Daryl M. Lamson; Erasmus Schneider; Erica Lasek-Nesselquist; John Kelly; Jonathan Plitnick; Kirsten St. George; Matthew Shudt; Melissa A Leisner; Navjot Singh
EPI_ISL_1416685 to 1416687	STZ	Jessa	Cruys et al. on behalf of the Jessa_cmdLab
EPI_ISL_1395751 to 1395752, EPI_ISL_1395755, EPI_ISL_1395759, EPI_ISL_1395761 to 1395762, EPI_ISL_1395764 to 1395765, EPI_ISL_1395767, EPI_ISL_1395770, EPI_ISL_1395772, EPI_ISL_1395774 to 1395776, EPI_ISL_1397389 to 1397392, EPI_ISL_1397394, EPI_ISL_1397396 to 1397402, EPI_ISL_1397404, EPI_ISL_1397406, EPI_ISL_1397408, EPI_ISL_1397410 to 1397423, EPI_ISL_1397459, EPI_ISL_1397592 to 1397611, EPI_ISL_1397629, EPI_ISL_1397706, EPI_ISL_1397784 to 1397787, EPI_ISL_1397968 to 1397970, EPI_ISL_1397983, EPI_ISL_1398002 to 1398004, EPI_ISL_1398156 to 1398162, EPI_ISL_1461294, EPI_ISL_1461310 to 1461317, EPI_ISL_1462500, EPI_ISL_1462502 to 1462503, EPI_ISL_1462505, EPI_ISL_1462536 to 1462537, EPI_ISL_1462539 to 1462540, EPI_ISL_1462542 to 1462543, EPI_ISL_1462545 to 1462546, EPI_ISL_1462548 to 1462549, EPI_ISL_1465638, EPI_ISL_1465665 to 1465666, EPI_ISL_1465689 to 1465692, EPI_ISL_1465697 to 1465700, EPI_ISL_1482274 to 1482276, EPI_ISL_1482406 to 1482407, EPI_ISL_1499437 to 1499456			
see above	SUNY UPSTATE MEDICAL UNIVERSITY	Wadsworth Center, New York State Department of Health	Alexis Russel; Alexis Russell; Catharine Prussing; Daryl M. Lamson; Erasmus Schneider; Erica Lasek-Nesselquist; John Kelly; Jonathan Plitnick; Kirsten St. George; Matthew Shudt; Melissa A Leisner; Navjot Singh
EPI_ISL_547575	SVO Jundiaí	Instituto Adolfo Lutz, Interdiciplinary Procedures Center, Strategic Laboratory	Claudia Regina Gonçalves; Claudio Tavares Sacchi; Erica Valessa Ramos Gomes; Karoline Rodrigues Campos
EPI_ISL_1492683 to 1492704, EPI_ISL_1492706, EPI_ISL_1492708 to 1492719, EPI_ISL_1492731, EPI_ISL_1492733 to 1492767, EPI_ISL_1492769 to 1492778, EPI_ISL_1492806 to 1492836, EPI_ISL_1492842 to 1492903, EPI_ISL_1492925 to 1492972, EPI_ISL_1493012 to 1493015, EPI_ISL_1493018 to 1493022, EPI_ISL_1493024 to 1493039, EPI_ISL_1533853 to 1533881, EPI_ISL_1533883 to 1533935			
see above	SYNLAB	GIGA Medical Genomics	Bouchra Boujemla; Cécile Meex; Keith Durkin; Maria Artesi; Marie-Pierre Hayette; Nathalie Renotte; Pierrette Melin; Raphaël Boreux; Sébastien Bontems; Vincent Bours
EPI_ISL_1434617, EPI_ISL_1434619, EPI_ISL_1434626, EPI_ISL_1434637, EPI_ISL_1434639, EPI_ISL_1434645, EPI_ISL_1434647, EPI_ISL_1434649, EPI_ISL_1434655, EPI_ISL_1434659, EPI_ISL_1434661, EPI_ISL_1434664, EPI_ISL_1434668, EPI_ISL_1434672, EPI_ISL_1434674, EPI_ISL_1434678, EPI_ISL_1434685 to 1434686, EPI_ISL_1434692, EPI_ISL_1434694, EPI_ISL_1434698, EPI_ISL_1434706, EPI_ISL_1434712, EPI_ISL_1434716, EPI_ISL_1434720 to 1434721, EPI_ISL_1434723, EPI_ISL_1434725, EPI_ISL_1434729, EPI_ISL_1434742, EPI_ISL_1434746, EPI_ISL_1434750, EPI_ISL_1434752, EPI_ISL_1434755, EPI_ISL_1434757, EPI_ISL_1434761, EPI_ISL_1434771, EPI_ISL_1434775, EPI_ISL_1434778, EPI_ISL_1434780, EPI_ISL_1434785, EPI_ISL_1434787, EPI_ISL_1434789, EPI_ISL_1434791, EPI_ISL_1434795 to 1434796, EPI_ISL_1434800, EPI_ISL_1434802, EPI_ISL_1434805, EPI_ISL_1434810, EPI_ISL_1434814, EPI_ISL_1434820, EPI_ISL_1434822, EPI_ISL_1434824, EPI_ISL_1434826, EPI_ISL_1434828 to 1434829, EPI_ISL_1434831, EPI_ISL_1434835, EPI_ISL_1434837, EPI_ISL_1434847, EPI_ISL_1434851, EPI_ISL_1434856 to 1434857, EPI_ISL_1434859, EPI_ISL_1434863, EPI_ISL_1434865, EPI_ISL_1434867, EPI_ISL_1434874, EPI_ISL_1434877, EPI_ISL_1434886, EPI_ISL_1434890, EPI_ISL_1434896, EPI_ISL_1434900, EPI_ISL_1434904, EPI_ISL_1434906, EPI_ISL_1434911, EPI_ISL_1434913, EPI_ISL_1434915, EPI_ISL_1434919, EPI_ISL_1434923, EPI_ISL_1434925, EPI_ISL_1434927, EPI_ISL_1434931, EPI_ISL_1434933, EPI_ISL_1434935, EPI_ISL_1434937, EPI_ISL_1434941, EPI_ISL_1434943, EPI_ISL_1434945, EPI_ISL_1434947, EPI_ISL_1434951, EPI_ISL_1434953, EPI_ISL_1434955, EPI_ISL_1434957 to 1434958, EPI_ISL_1434960, EPI_ISL_1434962, EPI_ISL_1434964, EPI_ISL_1434966, EPI_ISL_1434968, EPI_ISL_1434972, EPI_ISL_1434974, EPI_ISL_1434976, EPI_ISL_1434978, EPI_ISL_1434984, EPI_ISL_1434988, EPI_ISL_1434999, EPI_ISL_1435003, EPI_ISL_1435005, EPI_ISL_1435009, EPI_ISL_1435011, EPI_ISL_1435016, EPI_ISL_1435018, EPI_ISL_1435020, EPI_ISL_1435025, EPI_ISL_1435031, EPI_ISL_1435033, EPI_ISL_1435037, EPI_ISL_1435039, EPI_ISL_1435044, EPI_ISL_1435049 to 1435050, EPI_ISL_1435052, EPI_ISL_1435054 to 1435055, EPI_ISL_1435059, EPI_ISL_1435061, EPI_ISL_1435063, EPI_ISL_1435072, EPI_ISL_1435074, EPI_ISL_1435080, EPI_ISL_1435086 to 1435087, EPI_ISL_1435089, EPI_ISL_1435091, EPI_ISL_1435093, EPI_ISL_1435095, EPI_ISL_1435099, EPI_ISL_1435101, EPI_ISL_1435107, EPI_ISL_1435109, EPI_ISL_1435111, EPI_ISL_1435117, EPI_ISL_1435120, EPI_ISL_1435124, EPI_ISL_1435126, EPI_ISL_1435128, EPI_ISL_1435136, EPI_ISL_1435140, EPI_ISL_1435146, EPI_ISL_1435148, EPI_ISL_1435154, EPI_ISL_1435157, EPI_ISL_1435164, EPI_ISL_1435166, EPI_ISL_1435168, EPI_ISL_1435180, EPI_ISL_1435184, EPI_ISL_1435186, EPI_ISL_1435188, EPI_ISL_1435192, EPI_ISL_1435201, EPI_ISL_1435203, EPI_ISL_1435205, EPI_ISL_1435209, EPI_ISL_1435211, EPI_ISL_1435215 to 1435216, EPI_ISL_1435221 to 1435222, EPI_ISL_1435225 to 1435226, EPI_ISL_1435230, EPI_ISL_1435234, EPI_ISL_1435240, EPI_ISL_1435248, EPI_ISL_1435250, EPI_ISL_1435252, EPI_ISL_1435257, EPI_ISL_1435261, EPI_ISL_1435263, EPI_ISL_1435265, EPI_ISL_1435269, EPI_ISL_1435272, EPI_ISL_1435274, EPI_ISL_1435276, EPI_ISL_1435278 to 1435284, EPI_ISL_1436952, EPI_ISL_1436959 to 1436961, EPI_ISL_1436963, EPI_ISL_1436966, EPI_ISL_1436978, EPI_ISL_1436982 to 1436983, EPI_ISL_1436986, EPI_ISL_1437000, EPI_ISL_1437002, EPI_ISL_1437004 to 1437005, EPI_ISL_1437012, EPI_ISL_1437020, EPI_ISL_1437028 to 1437029, EPI_ISL_1437048, EPI_ISL_1437056, EPI_ISL_1437062, EPI_ISL_1437073, EPI_ISL_1437076, EPI_ISL_1437085, EPI_ISL_1437093, EPI_ISL_1437097, EPI_ISL_1437099, EPI_ISL_1437102 to 1437103, EPI_ISL_1437109, EPI_ISL_1437118, EPI_ISL_1437121, EPI_ISL_1437126, EPI_ISL_1437134, EPI_ISL_1437137 to 1437138, EPI_ISL_1437140 to 1437141, EPI_ISL_1437160, EPI_ISL_1437165, EPI_ISL_1437172, EPI_ISL_1437180, EPI_ISL_1437185, EPI_ISL_1437187 to 1437188, EPI_ISL_1437192, EPI_ISL_1437198, EPI_ISL_1437200 to 1437201, EPI_ISL_1437204, EPI_ISL_1437213, EPI_ISL_1437215, EPI_ISL_1437221, EPI_ISL_1437223, EPI_ISL_1437225, EPI_ISL_1437260, EPI_ISL_1437263, EPI_ISL_1437268, EPI_ISL_1438308, EPI_ISL_1438310, EPI_ISL_1438315, EPI_ISL_1438319 to 1438322, EPI_ISL_1438325 to 1438329, EPI_ISL_1438333 to 1438334, EPI_ISL_1438336 to 1438337, EPI_ISL_1438339 to 1438340, EPI_ISL_1438346 to 1438347, EPI_ISL_1438349, EPI_ISL_1438351, EPI_ISL_1438358, EPI_ISL_1438362, EPI_ISL_1438369 to 1438370, EPI_ISL_1438375, EPI_ISL_1438378 to 1438380, EPI_ISL_1438384 to 1438385, EPI_ISL_1438391, EPI_ISL_1438399, EPI_ISL_1438406 to 1438408, EPI_ISL_1438415, EPI_ISL_1438418, EPI_ISL_1438426, EPI_ISL_1438431 to 1438433, EPI_ISL_1438438, EPI_ISL_1438440, EPI_ISL_1438446, EPI_ISL_1438450, EPI_ISL_1438453, EPI_ISL_1438459, EPI_ISL_1438465, EPI_ISL_1438469, EPI_ISL_1438472, EPI_ISL_1438475, EPI_ISL_1438477, EPI_ISL_1438481, EPI_ISL_1438484, EPI_ISL_1438487, EPI_ISL_1438490, EPI_ISL_1438494 to 1438495, EPI_ISL_1438497, EPI_ISL_1438500, EPI_ISL_1438505, EPI_ISL_1438514, EPI_ISL_1438518 to 1438519, EPI_ISL_1438530, EPI_ISL_1438535 to 1438536, EPI_ISL_1438540, EPI_ISL_1438544, EPI_ISL_1438549, EPI_ISL_1438553, EPI_ISL_1438557, EPI_ISL_1438561, EPI_ISL_1438566, EPI_ISL_1438572, EPI_ISL_1438575, EPI_ISL_1438578, EPI_ISL_1441310, EPI_ISL_1441313, EPI_ISL_1441318, EPI_ISL_1441321, EPI_ISL_1441323, EPI_ISL_1441325 to 1441326, EPI_ISL_1441328, EPI_ISL_1441330, EPI_ISL_1441334, EPI_ISL_1441336 to 1441339, EPI_ISL_1441343, EPI_ISL_1441348, EPI_ISL_1441353, EPI_ISL_1441356, EPI_ISL_1441359, EPI_ISL_1441362, EPI_ISL_1441374, EPI_ISL_1441376, EPI_ISL_1441379, EPI_ISL_1441383, EPI_ISL_1441391, EPI_ISL_1441409, EPI_ISL_1441411, EPI_ISL_1441416, EPI_ISL_1441418, EPI_ISL_1441427 to 1441428, EPI_ISL_1441430, EPI_ISL_1441434, EPI_ISL_1441437, EPI_ISL_1441440, EPI_ISL_1441455, EPI_ISL_1441459, EPI_ISL_1441472 to 1441473, EPI_ISL_1441496, EPI_ISL_1441500, EPI_ISL_1441502, EPI_ISL_1441510, EPI_ISL_1441521, EPI_ISL_1441524, EPI_ISL_1441526, EPI_ISL_1441528, EPI_ISL_1441531, EPI_ISL_1441535, EPI_ISL_1441537, EPI_ISL_1441542, EPI_ISL_1441551, EPI_ISL_1441553, EPI_ISL_1441559, EPI_ISL_1441561, EPI_ISL_1441565, EPI_ISL_1441567 to 1441568, EPI_ISL_1441577, EPI_ISL_1441582, EPI_ISL_1441586, EPI_ISL_1441589, EPI_ISL_1441591 to 1441592, EPI_ISL_1441604, EPI_ISL_1441610, EPI_ISL_1441615, EPI_ISL_1441622, EPI_ISL_1441624, EPI_ISL_1441626, EPI_ISL_1441635, EPI_ISL_1441638, EPI_ISL_1441641 to 1441644, EPI_ISL_1442766, EPI_ISL_1442768 to 1442769, EPI_ISL_1442771, EPI_ISL_1442773, EPI_ISL_1442776, EPI_ISL_1442785 to 1442786, EPI_ISL_1442791, EPI_ISL_1442797 to 1442798, EPI_ISL_1442801, EPI_ISL_1442805, EPI_ISL_1442812, EPI_ISL_1442815, EPI_ISL_1442818 to 1442819, EPI_ISL_1442826 to 1442828, EPI_ISL_1442834, EPI_ISL_1442836, EPI_ISL_1442839 to 1442840, EPI_ISL_1442842, EPI_ISL_1442845, EPI_ISL_1442849, EPI_ISL_1442854 to 1442855, EPI_ISL_1442864 to 1442866, EPI_ISL_1442871, EPI_ISL_1442873 to 1442874, EPI_ISL_1442876 to 1442877, EPI_ISL_1442879, EPI_ISL_1442884 to 1442885, EPI_ISL_1442887			

see above	SYNLAB Jena Oncoscreen	Robert Koch Institute	
EPI_ISL_1438381, EPI_ISL_1438499, EPI_ISL_1438574	SYNLAB Labor München Zentrum LMZ	Robert Koch Institute	

EPI_ISL_1384774 to 1384801, EPI_ISL_1396404, EPI_ISL_1510083, EPI_ISL_1510100, EPI_ISL_1510111 to 1510118, EPI_ISL_1510192, EPI_ISL_1510194 to 1510195, EPI_ISL_1510203, EPI_ISL_1510208 to 1510209

see above	SYNLAB Lietuva UAB	Vilnius University Hospital Santaros Klinikos, Center of Laboratory Medicine	Daniel Naumovas; Dovile Ezerskyte; Gytis Dudas; Ingrida Olendraite; Laimonas Griskevicius; Ligita Raugaite; Mindaugas Stoskus; Monika Katenaite; Rimvydas Norvilas

EPI_ISL_1434615, EPI_ISL_1434657, EPI_ISL_1434666, EPI_ISL_1434683, EPI_ISL_1434688, EPI_ISL_1434714, EPI_ISL_1434731, EPI_ISL_1434737, EPI_ISL_1434744, EPI_ISL_1434765, EPI_ISL_1434818, EPI_ISL_1434888, EPI_ISL_1434892, EPI_ISL_1434898, EPI_ISL_1434970, EPI_ISL_1435023, EPI_ISL_1435070, EPI_ISL_1435076, EPI_ISL_1435084, EPI_ISL_1435115, EPI_ISL_1435130, EPI_ISL_1435134, EPI_ISL_1435160, EPI_ISL_1435172, EPI_ISL_1435178, EPI_ISL_1435190, EPI_ISL_1435197, EPI_ISL_1435213, EPI_ISL_1435228, EPI_ISL_1435242, EPI_ISL_1435254, EPI_ISL_1435259, EPI_ISL_1435267, EPI_ISL_1435271, EPI_ISL_1435273, EPI_ISL_1436974, EPI_ISL_1436979, EPI_ISL_1436992, EPI_ISL_1436996, EPI_ISL_1437031, EPI_ISL_1437038 to 1437039, EPI_ISL_1437110, EPI_ISL_1437119, EPI_ISL_1437135, EPI_ISL_1437145, EPI_ISL_1437147, EPI_ISL_1437151, EPI_ISL_1437156, EPI_ISL_1437171, EPI_ISL_1437176, EPI_ISL_1437179, EPI_ISL_1437184, EPI_ISL_1437207, EPI_ISL_1437216, EPI_ISL_1437219, EPI_ISL_1437227 to 1437228, EPI_ISL_1437231 to 1437232, EPI_ISL_1437238, EPI_ISL_1437240, EPI_ISL_1437247, EPI_ISL_1437249, EPI_ISL_1437252, EPI_ISL_1437255, EPI_ISL_1437259, EPI_ISL_1438364, EPI_ISL_1438524, EPI_ISL_1438539, EPI_ISL_1438558, EPI_ISL_1441312, EPI_ISL_1441320, EPI_ISL_1441324, EPI_ISL_1441331, EPI_ISL_1441350, EPI_ISL_1441360, EPI_ISL_1441370, EPI_ISL_1441375, EPI_ISL_1441382, EPI_ISL_1441386, EPI_ISL_1441388, EPI_ISL_1441394, EPI_ISL_1441396, EPI_ISL_1441400, EPI_ISL_1441405, EPI_ISL_1441410, EPI_ISL_1441413, EPI_ISL_1441415, EPI_ISL_1441420, EPI_ISL_1441425, EPI_ISL_1441439, EPI_ISL_1441465, EPI_ISL_1441467, EPI_ISL_1441476, EPI_ISL_1441506, EPI_ISL_1441508, EPI_ISL_1441515, EPI_ISL_1441529, EPI_ISL_1441534, EPI_ISL_1441540, EPI_ISL_1441546, EPI_ISL_1441557, EPI_ISL_1441563, EPI_ISL_1441571 to 1441572, EPI_ISL_1441574, EPI_ISL_1441576, EPI_ISL_1441579, EPI_ISL_1441587, EPI_ISL_1441599, EPI_ISL_1441606, EPI_ISL_1441612, EPI_ISL_1441623, EPI_ISL_1441630, EPI_ISL_1441632, EPI_ISL_1441634

see above	SYNLAB MVZ Berlin	Robert Koch Institute

EPI_ISL_1432055, EPI_ISL_1432089, EPI_ISL_1432125, EPI_ISL_1432155, EPI_ISL_1432265, EPI_ISL_1432312, EPI_ISL_1432432, EPI_ISL_1432678, EPI_ISL_1432686, EPI_ISL_1432706, EPI_ISL_1432713, EPI_ISL_1432717, EPI_ISL_1437434, EPI_ISL_1437440, EPI_ISL_1437456, EPI_ISL_1437460, EPI_ISL_1437483, EPI_ISL_1437487, EPI_ISL_1437496, EPI_ISL_1437517, EPI_ISL_1437563, EPI_ISL_1437590, EPI_ISL_1439972, EPI_ISL_1440218, EPI_ISL_1442368, EPI_ISL_1442374, EPI_ISL_1442376, EPI_ISL_1442381, EPI_ISL_1442449, EPI_ISL_1442456

see above	SYNLAB MVZ Bonn	Robert Koch Institute

EPI_ISL_1432026, EPI_ISL_1432031, EPI_ISL_1432038, EPI_ISL_1432042, EPI_ISL_1432046, EPI_ISL_1432050, EPI_ISL_1432053, EPI_ISL_1432057, EPI_ISL_1432062, EPI_ISL_1432066, EPI_ISL_1432074, EPI_ISL_1432084, EPI_ISL_1432086, EPI_ISL_1432091, EPI_ISL_1432097 to 1432098, EPI_ISL_1432107, EPI_ISL_1432109 to 1432110, EPI_ISL_1432114, EPI_ISL_1432118, EPI_ISL_1432123, EPI_ISL_1432128, EPI_ISL_1432134, EPI_ISL_1432138, EPI_ISL_1432145, EPI_ISL_1432153, EPI_ISL_1432161, EPI_ISL_1432163, EPI_ISL_1432166, EPI_ISL_1432173, EPI_ISL_1432175, EPI_ISL_1432181, EPI_ISL_1432186, EPI_ISL_1432190, EPI_ISL_1432201, EPI_ISL_1432205, EPI_ISL_1432209, EPI_ISL_1432211, EPI_ISL_1432217, EPI_ISL_1432221, EPI_ISL_1432225, EPI_ISL_1432227, EPI_ISL_1432229, EPI_ISL_1432231, EPI_ISL_1432234, EPI_ISL_1432239, EPI_ISL_1432241, EPI_ISL_1432246, EPI_ISL_1432248, EPI_ISL_1432252, EPI_ISL_1432257, EPI_ISL_1432260, EPI_ISL_1432278, EPI_ISL_1432281, EPI_ISL_1432286, EPI_ISL_1432292, EPI_ISL_1432300, EPI_ISL_1432305, EPI_ISL_1432309, EPI_ISL_1432315, EPI_ISL_1432328, EPI_ISL_1432338, EPI_ISL_1432341, EPI_ISL_1432345, EPI_ISL_1432349, EPI_ISL_1432351, EPI_ISL_1432355, EPI_ISL_1432362, EPI_ISL_1432365, EPI_ISL_1432373, EPI_ISL_1432382, EPI_ISL_1432390, EPI_ISL_1432392, EPI_ISL_1432397, EPI_ISL_1432399, EPI_ISL_1432402, EPI_ISL_1432411, EPI_ISL_1432417, EPI_ISL_1432421, EPI_ISL_1432423, EPI_ISL_1432430, EPI_ISL_1432436, EPI_ISL_1432438, EPI_ISL_1432445, EPI_ISL_1432453, EPI_ISL_1432457, EPI_ISL_1432466, EPI_ISL_1432475, EPI_ISL_1432479, EPI_ISL_1432489, EPI_ISL_1432494, EPI_ISL_1432496, EPI_ISL_1432516, EPI_ISL_1432523, EPI_ISL_1432528, EPI_ISL_1432534, EPI_ISL_1432537, EPI_ISL_1432539, EPI_ISL_1432543, EPI_ISL_1432549, EPI_ISL_1432554, EPI_ISL_1432558, EPI_ISL_1432562, EPI_ISL_1432566, EPI_ISL_1432571, EPI_ISL_1432573, EPI_ISL_1432575, EPI_ISL_1432581, EPI_ISL_1432583, EPI_ISL_1432586, EPI_ISL_1432592, EPI_ISL_1432594, EPI_ISL_1432601, EPI_ISL_1432609, EPI_ISL_1432611, EPI_ISL_1432620, EPI_ISL_1432626, EPI_ISL_1432635, EPI_ISL_1432637, EPI_ISL_1432645, EPI_ISL_1432652, EPI_ISL_1432654, EPI_ISL_1432661, EPI_ISL_1432663, EPI_ISL_1432667, EPI_ISL_1432669, EPI_ISL_1432676, EPI_ISL_1432688, EPI_ISL_1432690, EPI_ISL_1432692, EPI_ISL_1432694, EPI_ISL_1432698, EPI_ISL_1432702, EPI_ISL_1432705, EPI_ISL_1432711, EPI_ISL_1432715, EPI_ISL_1432722, EPI_ISL_1432729, EPI_ISL_1432731, EPI_ISL_1432735, EPI_ISL_1432740, EPI_ISL_1432748, EPI_ISL_1437429, EPI_ISL_1437435, EPI_ISL_1437437 to 1437439, EPI_ISL_1437444, EPI_ISL_1437448, EPI_ISL_1437455, EPI_ISL_1437457 to 1437459, EPI_ISL_1437461, EPI_ISL_1437463, EPI_ISL_1437467 to 1437468, EPI_ISL_1437471, EPI_ISL_1437478, EPI_ISL_1437482, EPI_ISL_1437486, EPI_ISL_1437488, EPI_ISL_1437490, EPI_ISL_1437492 to 1437493, EPI_ISL_1437495, EPI_ISL_1437500 to 1437501, EPI_ISL_1437507, EPI_ISL_1437511, EPI_ISL_1437521, EPI_ISL_1437524, EPI_ISL_1437526, EPI_ISL_1437531, EPI_ISL_1437533, EPI_ISL_1437535, EPI_ISL_1437542, EPI_ISL_1437547, EPI_ISL_1437549, EPI_ISL_1437551, EPI_ISL_1437556, EPI_ISL_1437560, EPI_ISL_1437567, EPI_ISL_1437570, EPI_ISL_1437572, EPI_ISL_1437575 to 1437577, EPI_ISL_1437580, EPI_ISL_1437582 to 1437583, EPI_ISL_1437587, EPI_ISL_1437598, EPI_ISL_1437600 to 1437601, EPI_ISL_1437604 to 1437605, EPI_ISL_1437607, EPI_ISL_1437609, EPI_ISL_1439902, EPI_ISL_1439904 to 1439905, EPI_ISL_1439909, EPI_ISL_1439911, EPI_ISL_1439915, EPI_ISL_1439929, EPI_ISL_1439933, EPI_ISL_1439935 to 1439936, EPI_ISL_1439940, EPI_ISL_1439946, EPI_ISL_1439951, EPI_ISL_1439957, EPI_ISL_1439966, EPI_ISL_1439977, EPI_ISL_1439980 to 1439981, EPI_ISL_1439986, EPI_ISL_1439988, EPI_ISL_1439994, EPI_ISL_1439997, EPI_ISL_1439999, EPI_ISL_1440008, EPI_ISL_1440012, EPI_ISL_1440014, EPI_ISL_1440018, EPI_ISL_1440022, EPI_ISL_1440026, EPI_ISL_1440028, EPI_ISL_1440034, EPI_ISL_1440038, EPI_ISL_1440040, EPI_ISL_1440044, EPI_ISL_1440047, EPI_ISL_1440049, EPI_ISL_1440054, EPI_ISL_1440059, EPI_ISL_1440061, EPI_ISL_1440063, EPI_ISL_1440067, EPI_ISL_1440072, EPI_ISL_1440074, EPI_ISL_1440079, EPI_ISL_1440085, EPI_ISL_1440097, EPI_ISL_1440101, EPI_ISL_1440107, EPI_ISL_1440111, EPI_ISL_1440117, EPI_ISL_1440123 to 1440124, EPI_ISL_1440138, EPI_ISL_1440159, EPI_ISL_1440166, EPI_ISL_1440169, EPI_ISL_1440173, EPI_ISL_1440187, EPI_ISL_1440189, EPI_ISL_1440194 to 1440195, EPI_ISL_1440198, EPI_ISL_1440200 to 1440201, EPI_ISL_1440204, EPI_ISL_1440213, EPI_ISL_1440224 to 1440225, EPI_ISL_1440231, EPI_ISL_1440235, EPI_ISL_1440237, EPI_ISL_1440242, EPI_ISL_1440247, EPI_ISL_1440250, EPI_ISL_1440255, EPI_ISL_1440258 to 1440259, EPI_ISL_1440262 to 1440263, EPI_ISL_1440265, EPI_ISL_1440267 to 1440268, EPI_ISL_1440270 to 1440271, EPI_ISL_1440273, EPI_ISL_1442371, EPI_ISL_1442377, EPI_ISL_1442379 to 1442380, EPI_ISL_1442382, EPI_ISL_1442386 to 1442387, EPI_ISL_1442390 to 1442391, EPI_ISL_1442394 to 1442396, EPI_ISL_1442398 to 1442401, EPI_ISL_1442404, EPI_ISL_1442407, EPI_ISL_1442410, EPI_ISL_1442415, EPI_ISL_1442419 to 1442420, EPI_ISL_1442423 to 1442425, EPI_ISL_1442428, EPI_ISL_1442431 to 1442432, EPI_ISL_1442435, EPI_ISL_1442437, EPI_ISL_1442443, EPI_ISL_1442446, EPI_ISL_1442450, EPI_ISL_1442452, EPI_ISL_1442454, EPI_ISL_1442457 to 1442459, EPI_ISL_1442461, EPI_ISL_1442467 to 1442468, EPI_ISL_1442470, EPI_ISL_1442472, EPI_ISL_1442477, EPI_ISL_1442486

see above	SYNLAB MVZ Ettlingen	Robert Koch Institute

EPI_ISL_1432070, EPI_ISL_1432093, EPI_ISL_1432192, EPI_ISL_1432360, EPI_ISL_1432386, EPI_ISL_1432428, EPI_ISL_1432440, EPI_ISL_1432447, EPI_ISL_1432487, EPI_ISL_1432491, EPI_ISL_1432500, EPI_ISL_1432556, EPI_ISL_1432588, EPI_ISL_1432596, EPI_ISL_1432622, EPI_ISL_1432742, EPI_ISL_1437286, EPI_ISL_1437329, EPI_ISL_1437331, EPI_ISL_1437365 to 1437366, EPI_ISL_1437371, EPI_ISL_1437379, EPI_ISL_1437384, EPI_ISL_1437595, EPI_ISL_1439922, EPI_ISL_1439971, EPI_ISL_1439993, EPI_ISL_1440000, EPI_ISL_1440005, EPI_ISL_1440015, EPI_ISL_1440020, EPI_ISL_1440042, EPI_ISL_1440121, EPI_ISL_1440130, EPI_ISL_1440163, EPI_ISL_1440175, EPI_ISL_1440185, EPI_ISL_1440253, EPI_ISL_1440579, EPI_ISL_1440585, EPI_ISL_1440587, EPI_ISL_1440610, EPI_ISL_1440616, EPI_ISL_1440619, EPI_ISL_1440621, EPI_ISL_1440623, EPI_ISL_1440644, EPI_ISL_1440647, EPI_ISL_1440652, EPI_ISL_1440657, EPI_ISL_1440660, EPI_ISL_1440666, EPI_ISL_1440690, EPI_ISL_1440699, EPI_ISL_1442367, EPI_ISL_1442385, EPI_ISL_1442389, EPI_ISL_1442392, EPI_ISL_1442409, EPI_ISL_1442412, EPI_ISL_1442414, EPI_ISL_1442418, EPI_ISL_1442463

see above	SYNLAB MVZ Hamburg	Robert Koch Institute

EPI_ISL_1432029, EPI_ISL_1432033, EPI_ISL_1432036, EPI_ISL_1432040, EPI_ISL_1432044, EPI_ISL_1432048, EPI_ISL_1432052, EPI_ISL_1432061, EPI_ISL_1432072, EPI_ISL_1432080, EPI_ISL_1432095, EPI_ISL_1432100, EPI_ISL_1432103, EPI_ISL_1432120, EPI_ISL_1432130, EPI_ISL_1432132, EPI_ISL_1432136, EPI_ISL_1432140, EPI_ISL_1432143, EPI_ISL_1432147, EPI_ISL_1432149, EPI_ISL_1432157, EPI_ISL_1432159, EPI_ISL_1432168, EPI_ISL_1432177, EPI_ISL_1432183, EPI_ISL_1432188, EPI_ISL_1432203, EPI_ISL_1432207, EPI_ISL_1432219, EPI_ISL_1432244, EPI_ISL_1432259, EPI_ISL_1432267, EPI_ISL_1432274 to 1432275, EPI_ISL_1432280, EPI_ISL_1432294, EPI_ISL_1432330, EPI_ISL_1432336, EPI_ISL_1432353, EPI_ISL_1432358, EPI_ISL_1432369, EPI_ISL_1432380, EPI_ISL_1432388, EPI_ISL_1432400, EPI_ISL_1432413, EPI_ISL_1432419, EPI_ISL_1432426, EPI_ISL_1432434, EPI_ISL_1432455, EPI_ISL_1432468, EPI_ISL_1432470, EPI_ISL_1432473, EPI_ISL_1432481, EPI_ISL_1432498, EPI_ISL_1432508, EPI_ISL_1432519, EPI_ISL_1432521, EPI_ISL_1432530, EPI_ISL_1432532, EPI_ISL_1432541, EPI_ISL_1432547, EPI_ISL_1432560, EPI_ISL_1432605, EPI_ISL_1432613, EPI_ISL_1432630, EPI_ISL_1432633, EPI_ISL_1432641, EPI_ISL_1432647, EPI_ISL_1432720, EPI_ISL_1432724, EPI_ISL_1432733, EPI_ISL_1432746, EPI_ISL_1437296, EPI_ISL_1437311, EPI_ISL_1437314, EPI_ISL_1437316, EPI_ISL_1437322, EPI_ISL_1437341, EPI_ISL_1437373, EPI_ISL_1437426, EPI_ISL_1437428, EPI_ISL_1439913, EPI_ISL_1439918, EPI_ISL_1439928, EPI_ISL_1439931, EPI_ISL_1439962, EPI_ISL_1440037, EPI_ISL_1440140, EPI_ISL_1440146, EPI_ISL_1440582, EPI_ISL_1440589, EPI_ISL_1440601, EPI_ISL_1440608 to 1440609, EPI_ISL_1440618, EPI_ISL_1440620, EPI_ISL_1440624, EPI_ISL_1440633, EPI_ISL_1440639 to 1440640, EPI_ISL_1440643, EPI_ISL_1440646, EPI_ISL_1440656, EPI_ISL_1440681, EPI_ISL_1440685, EPI_ISL_1440691, EPI_ISL_1440695 to 1440696, EPI_ISL_1440698, EPI_ISL_1440700, EPI_ISL_1440709, EPI_ISL_1440714, EPI_ISL_1440728

see above	SYNLAB MVZ Heidelberg	Robert Koch Institute

EPI_ISL_1434620, EPI_ISL_1434622, EPI_ISL_1434624, EPI_ISL_1434631, EPI_ISL_1434633, EPI_ISL_1434635, EPI_ISL_1434651, EPI_ISL_1434653, EPI_ISL_1434663, EPI_ISL_1434676, EPI_ISL_1434681, EPI_ISL_1434696, EPI_ISL_1434700, EPI_ISL_1434702, EPI_ISL_1434704, EPI_ISL_1434708, EPI_ISL_1434710, EPI_ISL_1434718, EPI_ISL_1434727, EPI_ISL_1434733, EPI_ISL_1434735, EPI_ISL_1434748, EPI_ISL_1434758, EPI_ISL_1434767, EPI_ISL_1434769, EPI_ISL_1434777, EPI_ISL_1434783, EPI_ISL_1434793, EPI_ISL_1434798, EPI_ISL_1434809, EPI_ISL_1434816, EPI_ISL_1434834, EPI_ISL_1434839, EPI_ISL_1434841, EPI_ISL_1434845, EPI_ISL_1434849, EPI_ISL_1434869, EPI_ISL_1434873, EPI_ISL_1434879, EPI_ISL_1434881, EPI_ISL_1434883 to 1434884, EPI_ISL_1434894, EPI_ISL_1434902, EPI_ISL_1434908, EPI_ISL_1434910, EPI_ISL_1434917, EPI_ISL_1434921, EPI_ISL_1434929, EPI_ISL_1434939, EPI_ISL_1434948, EPI_ISL_1434980, EPI_ISL_1434982, EPI_ISL_1434986, EPI_ISL_1434993, EPI_ISL_1434997, EPI_ISL_1435001, EPI_ISL_1435007, EPI_ISL_1435013 to 1435014, EPI_ISL_1435022, EPI_ISL_1435027, EPI_ISL_1435029, EPI_ISL_1435041 to 1435042, EPI_ISL_1435046, EPI_ISL_1435065, EPI_ISL_1435078, EPI_ISL_1435082, EPI_ISL_1435097, EPI_ISL_1435105, EPI_ISL_1435113, EPI_ISL_1435122, EPI_ISL_1435142, EPI_ISL_1435144, EPI_ISL_1435153, EPI_ISL_1435159, EPI_ISL_1435162, EPI_ISL_1435170, EPI_ISL_1435174, EPI_ISL_1435176, EPI_ISL_1435182, EPI_ISL_1435194, EPI_ISL_1435196, EPI_ISL_1435207, EPI_ISL_1435232, EPI_ISL_1435236, EPI_ISL_1435244, EPI_ISL_1435246, EPI_ISL_1436949 to 1436951, EPI_ISL_1436953 to 1436958, EPI_ISL_1436962, EPI_ISL_1436964 to 1436965, EPI_ISL_1436967 to 1436970, EPI_ISL_1436972 to 1436973, EPI_ISL_1436980, EPI_ISL_1436984 to 1436985, EPI_ISL_1436991, EPI_ISL_1436994 to 1436995, EPI_ISL_1436997 to 1436999, EPI_ISL_1437001, EPI_ISL_1437003, EPI_ISL_1437006 to 1437011, EPI_ISL_1437014 to 1437016, EPI_ISL_1437018 to 1437019, EPI_ISL_1437021 to 1437027, EPI_ISL_1437030, EPI_ISL_1437032 to 1437037, EPI_ISL_1437040, EPI_ISL_1437042 to 1437044, EPI_ISL_1437046, EPI_ISL_1437049 to 1437051, EPI_ISL_1437054 to 1437055, EPI_ISL_1437058, EPI_ISL_1437064, EPI_ISL_1437066 to 1437068, EPI_ISL_1437072, EPI_ISL_1437074, EPI_ISL_1437078, EPI_ISL_1437080, EPI_ISL_1437083, EPI_ISL_1437086 to 1437088, EPI_ISL_1437090, EPI_ISL_1437092, EPI_ISL_1437094 to 1437096, EPI_ISL_1437098, EPI_ISL_1437100 to 1437101, EPI_ISL_1437104 to 1437105, EPI_ISL_1437107 to 1437108, EPI_ISL_1437111 to 1437115, EPI_ISL_1437117, EPI_ISL_1437120, EPI_ISL_1437122 to 1437123, EPI_ISL_1437127 to 1437133, EPI_ISL_1437139, EPI_ISL_1437142 to 1437144, EPI_ISL_1437146, EPI_ISL_1437148 to 1437150, EPI_ISL_1437153 to 1437155, EPI_ISL_1437158 to 1437159, EPI_ISL_1437161 to 1437164, EPI_ISL_1437166 to 1437170, EPI_ISL_1437173 to 1437175, EPI_ISL_1437177 to 1437178, EPI_ISL_1437181 to 1437183, EPI_ISL_1437186, EPI_ISL_1437190 to 1437191, EPI_ISL_1437193 to 1437197, EPI_ISL_1437199, EPI_ISL_1437202, EPI_ISL_1437205 to 1437206, EPI_ISL_1437209 to 1437212, EPI_ISL_1437214, EPI_ISL_1437217 to 1437218, EPI_ISL_1437222, EPI_ISL_1437226, EPI_ISL_1437229 to 1437230, EPI_ISL_1437234 to 1437237, EPI_ISL_1437241 to 1437246, EPI_ISL_1437248, EPI_ISL_1437250 to 1437251, EPI_ISL_1437253 to 1437254, EPI_ISL_1437256, EPI_ISL_1437258, EPI_ISL_1437261 to 1437262, EPI_ISL_1437264 to 1437267, EPI_ISL_1437269 to 1437275, EPI_ISL_1437277 to 1437278, EPI_ISL_1437280, EPI_ISL_1438296 to 1438297, EPI_ISL_1438300 to 1438307, EPI_ISL_1438309, EPI_ISL_1438312 to 1438314, EPI_ISL_1438317 to 1438318, EPI_ISL_1438323 to 1438324, EPI_ISL_1438331 to 1438332, EPI_ISL_1438338, EPI_ISL_1438341 to 1438342, EPI_ISL_1438345, EPI_ISL_1438350, EPI_ISL_1438353, EPI_ISL_1438355 to 1438357, EPI_ISL_1438359 to 1438360, EPI_ISL_1438363, EPI_ISL_1438366 to 1438368, EPI_ISL_1438372 to 1438374, EPI_ISL_1438376 to 1438377, EPI_ISL_1438382 to 1438383, EPI_ISL_1438386 to 1438390, EPI_ISL_1438392, EPI_ISL_1438394 to 1438398, EPI_ISL_1438402 to 1438404, EPI_ISL_1438409 to 1438414, EPI_ISL_1438419 to 1438425, EPI_ISL_1438428 to 1438430, EPI_ISL_1438435 to 1438437, EPI_ISL_1438439, EPI_ISL_1438442, EPI_ISL_1438444, EPI_ISL_1438447 to 1438448, EPI_ISL_1438451 to 1438452, EPI_ISL_1438455, EPI_ISL_1438457, EPI_ISL_1438461, EPI_ISL_1438463, EPI_ISL_1438467 to 1438468, EPI_ISL_1438470 to 1438471, EPI_ISL_1438473, EPI_ISL_1438476, EPI_ISL_1438478, EPI_ISL_1438480, EPI_ISL_1438482 to 1438483, EPI_ISL_1438485, EPI_ISL_1438488 to 1438489, EPI_ISL_1438492 to 1438493, EPI_ISL_1438496, EPI_ISL_1438498, EPI_ISL_1438501, EPI_ISL_1438503 to 1438504, EPI_ISL_1438506 to 1438508, EPI_ISL_1438510 to 1438513, EPI_ISL_1438515, EPI_ISL_1438517, EPI_ISL_1438520 to 1438523, EPI_ISL_1438525 to 1438529, EPI_ISL_1438531 to 1438532, EPI_ISL_1438534, EPI_ISL_1438538, EPI_ISL_1438541, EPI_ISL_1438543, EPI_ISL_1438545, EPI_ISL_1438547 to 1438548, EPI_ISL_1438550, EPI_ISL_1438552, EPI_ISL_1438554 to 1438556, EPI_ISL_1438560, EPI_ISL_1438562 to 1438563, EPI_ISL_1438565, EPI_ISL_1438568, EPI_ISL_1438570 to 1438571, EPI_ISL_1438576 to 1438577, EPI_ISL_1438579 to 1438581, EPI_ISL_1441311, EPI_ISL_1441315, EPI_ISL_1441317, EPI_ISL_1441319, EPI_ISL_1441329, EPI_ISL_1441333, EPI_ISL_1441335, EPI_ISL_1441342, EPI_ISL_1441344 to 1441347, EPI_ISL_1441351 to 1441352, EPI_ISL_1441354, EPI_ISL_1441361, EPI_ISL_1441363, EPI_ISL_1441367, EPI_ISL_1441369, EPI_ISL_1441371 to 1441373, EPI_ISL_1441377, EPI_ISL_1441381, EPI_ISL_1441385, EPI_ISL_1441390, EPI_ISL_1441392 to 1441393, EPI_ISL_1441395, EPI_ISL_1441398, EPI_ISL_1441404, EPI_ISL_1441406 to 1441408, EPI_ISL_1441412, EPI_ISL_1441443 to 1441444, EPI_ISL_1441446, EPI_ISL_1441448, EPI_ISL_1441451, EPI_ISL_1441453, EPI_ISL_1441457, EPI_ISL_1441461, EPI_ISL_1441463, EPI_ISL_1441469, EPI_ISL_1441478 to 1441479, EPI_ISL_1441481, EPI_ISL_1441483, EPI_ISL_1441494, EPI_ISL_1441498, EPI_ISL_1441505, EPI_ISL_1441507, EPI_ISL_1441516, EPI_ISL_1441520, EPI_ISL_1441532, EPI_ISL_1441536, EPI_ISL_1441548, EPI_ISL_1441550, EPI_ISL_1441575, EPI_ISL_1441584, EPI_ISL_1441593, EPI_ISL_1441597, EPI_ISL_1441601 to 1441602, EPI_ISL_1441605, EPI_ISL_1441607, EPI_ISL_1441611, EPI_ISL_1441613 to 1441614, EPI_ISL_1441617 to 1441621, EPI_ISL_1441625, EPI_ISL_1441627 to 1441629, EPI_ISL_1441633, EPI_ISL_1441637, EPI_ISL_1441639, EPI_ISL_1442751 to 1442765, EPI_ISL_1442767, EPI_ISL_1442770, EPI_ISL_1442772, EPI_ISL_1442775, EPI_ISL_1442778 to 1442784, EPI_ISL_1442787 to 1442790, EPI_ISL_1442792 to 1442796, EPI_ISL_1442799 to 1442800, EPI_ISL_1442802 to 1442804, EPI_ISL_1442806 to 1442807, EPI_ISL_1442809 to 1442811, EPI_ISL_1442813, EPI_ISL_1442816, EPI_ISL_1442820 to 1442825, EPI_ISL_1442829 to 1442830, EPI_ISL_1442832, EPI_ISL_1442835, EPI_ISL_1442837 to 1442838, EPI_ISL_1442841, EPI_ISL_1442843 to 1442844, EPI_ISL_1442847, EPI_ISL_1442850 to 1442853, EPI_ISL_1442856 to 1442859, EPI_ISL_1442861 to 1442863, EPI_ISL_1442867, EPI_ISL_1442869 to 1442870, EPI_ISL_1442872, EPI_ISL_1442875, EPI_ISL_1442880, EPI_ISL_1442882 to 1442883, EPI_ISL_1442886, EPI_ISL_1442888 to 1442895

see above	SYNLAB MVZ Leinfelden-Echterdingen	Robert Koch Institute

EPI_ISL_1432059, EPI_ISL_1432065, EPI_ISL_1432068, EPI_ISL_1432075, EPI_ISL_1432082, EPI_ISL_1432122, EPI_ISL_1432151, EPI_ISL_1432170, EPI_ISL_1432179, EPI_ISL_1432194, EPI_ISL_1432196, EPI_ISL_1432213, EPI_ISL_1432236, EPI_ISL_1432250, EPI_ISL_1432255, EPI_ISL_1432269, EPI_ISL_1432271, EPI_ISL_1432284, EPI_ISL_1432290, EPI_ISL_1432296, EPI_ISL_1432302, EPI_ISL_1432307, EPI_ISL_1432317, EPI_ISL_1432321, EPI_ISL_1432323, EPI_ISL_1432325, EPI_ISL_1432334, EPI_ISL_1432343, EPI_ISL_1432347, EPI_ISL_1432371, EPI_ISL_1432375, EPI_ISL_1432378, EPI_ISL_1432395, EPI_ISL_1432405, EPI_ISL_1432407, EPI_ISL_1432409, EPI_ISL_1432415, EPI_ISL_1432449, EPI_ISL_1432451, EPI_ISL_1432460, EPI_ISL_1432462, EPI_ISL_1432503, EPI_ISL_1432510, EPI_ISL_1432512, EPI_ISL_1432514, EPI_ISL_1432525, EPI_ISL_1432545, EPI_ISL_1432564, EPI_ISL_1432569, EPI_ISL_1432579, EPI_ISL_1432590,

EPI_ISL_1432599, EPI_ISL_1432603, EPI_ISL_1432607, EPI_ISL_1432616, EPI_ISL_1432618, EPI_ISL_1432624, EPI_ISL_1432628, EPI_ISL_1432643, EPI_ISL_1432650, EPI_ISL_1432658, EPI_ISL_1432665, EPI_ISL_1432672, EPI_ISL_1432674, EPI_ISL_1432679, EPI_ISL_1432682, EPI_ISL_1432684, EPI_ISL_1432696, EPI_ISL_1432700, EPI_ISL_1432708, EPI_ISL_1432737, EPI_ISL_1437283 to 1437285, EPI_ISL_1437287 to 1437293, EPI_ISL_1437295, EPI_ISL_1437297 to 1437301, EPI_ISL_1437303 to 1437306, EPI_ISL_1437308 to 1437310, EPI_ISL_1437312 to 1437313, EPI_ISL_1437315, EPI_ISL_1437317 to 1437321, EPI_ISL_1437323 to 1437328, EPI_ISL_1437332 to 1437335, EPI_ISL_1437338 to 1437340, EPI_ISL_1437342 to 1437346, EPI_ISL_1437348 to 1437361, EPI_ISL_1437363 to 1437364, EPI_ISL_1437367, EPI_ISL_1437369 to 1437370, EPI_ISL_1437372, EPI_ISL_1437374, EPI_ISL_1437376 to 1437378, EPI_ISL_1437380 to 1437383, EPI_ISL_1437385 to 1437386, EPI_ISL_1437388 to 1437396, EPI_ISL_1437399 to 1437405, EPI_ISL_1437407 to 1437408, EPI_ISL_1437410 to 1437416, EPI_ISL_1437418 to 1437425, EPI_ISL_1437427, EPI_ISL_1437430, EPI_ISL_1437432 to 1437433, EPI_ISL_1437436, EPI_ISL_1437441 to 1437442, EPI_ISL_1437445 to 1437447, EPI_ISL_1437449 to 1437454, EPI_ISL_1437462, EPI_ISL_1437464 to 1437466, EPI_ISL_1437469 to 1437470, EPI_ISL_1437472 to 1437477, EPI_ISL_1437479 to 1437481, EPI_ISL_1437484 to 1437485, EPI_ISL_1437489, EPI_ISL_1437491, EPI_ISL_1437494, EPI_ISL_1437497 to 1437499, EPI_ISL_1437502 to 1437503, EPI_ISL_1437505, EPI_ISL_1437509, EPI_ISL_1437513, EPI_ISL_1437515, EPI_ISL_1437519, EPI_ISL_1437528, EPI_ISL_1437537, EPI_ISL_1437544, EPI_ISL_1437554, EPI_ISL_1437558, EPI_ISL_1437565, EPI_ISL_1437574, EPI_ISL_1437578 to 1437579, EPI_ISL_1437581, EPI_ISL_1437584 to 1437586, EPI_ISL_1437588 to 1437589, EPI_ISL_1437591 to 1437594, EPI_ISL_1437596 to 1437597, EPI_ISL_1437599, EPI_ISL_1437602 to 1437603, EPI_ISL_1437606, EPI_ISL_1437608, EPI_ISL_1437610 to 1437611, EPI_ISL_1439901, EPI_ISL_1439903, EPI_ISL_1439906 to 1439908, EPI_ISL_1439910, EPI_ISL_1439912, EPI_ISL_1439914, EPI_ISL_1439916 to 1439917, EPI_ISL_1439919, EPI_ISL_1439921, EPI_ISL_1439923, EPI_ISL_1439925 to 1439926, EPI_ISL_1439930, EPI_ISL_1439932, EPI_ISL_1439937 to 1439939, EPI_ISL_1439941 to 1439943, EPI_ISL_1439945, EPI_ISL_1439947, EPI_ISL_1439949 to 1439950, EPI_ISL_1439952 to 1439956, EPI_ISL_1439958 to 1439960, EPI_ISL_1439963 to 1439965, EPI_ISL_1439967 to 1439968, EPI_ISL_1439970, EPI_ISL_1439973 to 1439974, EPI_ISL_1439976, EPI_ISL_1439978 to 1439979, EPI_ISL_1439982, EPI_ISL_1439984 to 1439985, EPI_ISL_1439989, EPI_ISL_1439991, EPI_ISL_1439995 to 1439996, EPI_ISL_1439998, EPI_ISL_1440001 to 1440004, EPI_ISL_1440007, EPI_ISL_1440009 to 1440011, EPI_ISL_1440013, EPI_ISL_1440016 to 1440017, EPI_ISL_1440019, EPI_ISL_1440021, EPI_ISL_1440023 to 1440025, EPI_ISL_1440027, EPI_ISL_1440029 to 1440033, EPI_ISL_1440035 to 1440036, EPI_ISL_1440039, EPI_ISL_1440041, EPI_ISL_1440043, EPI_ISL_1440045 to 1440046, EPI_ISL_1440048, EPI_ISL_1440050 to 1440053, EPI_ISL_1440055 to 1440058, EPI_ISL_1440060, EPI_ISL_1440062, EPI_ISL_1440064 to 1440066, EPI_ISL_1440068 to 1440069, EPI_ISL_1440071, EPI_ISL_1440073, EPI_ISL_1440076 to 1440077, EPI_ISL_1440080, EPI_ISL_1440082, EPI_ISL_1440086, EPI_ISL_1440088 to 1440089, EPI_ISL_1440092, EPI_ISL_1440094 to 1440095, EPI_ISL_1440098, EPI_ISL_1440100, EPI_ISL_1440103 to 1440105, EPI_ISL_1440108, EPI_ISL_1440110, EPI_ISL_1440113 to 1440114, EPI_ISL_1440120, EPI_ISL_1440126 to 1440127, EPI_ISL_1440132 to 1440133, EPI_ISL_1440135, EPI_ISL_1440137, EPI_ISL_1440141, EPI_ISL_1440143 to 1440144, EPI_ISL_1440147, EPI_ISL_1440149 to 1440150, EPI_ISL_1440152 to 1440153, EPI_ISL_1440155 to 1440156, EPI_ISL_1440158, EPI_ISL_1440161 to 1440162, EPI_ISL_1440165, EPI_ISL_1440168, EPI_ISL_1440170, EPI_ISL_1440176 to 1440177, EPI_ISL_1440180, EPI_ISL_1440182 to 1440183, EPI_ISL_1440186, EPI_ISL_1440191 to 1440192, EPI_ISL_1440197, EPI_ISL_1440203, EPI_ISL_1440205, EPI_ISL_1440216, EPI_ISL_1440219, EPI_ISL_1440221 to 1440222, EPI_ISL_1440227 to 1440228, EPI_ISL_1440230, EPI_ISL_1440233 to 1440234, EPI_ISL_1440238 to 1440239, EPI_ISL_1440241, EPI_ISL_1440244, EPI_ISL_1440246, EPI_ISL_1440249, EPI_ISL_1440251, EPI_ISL_1440256, EPI_ISL_1440260, EPI_ISL_1440274, EPI_ISL_1440578, EPI_ISL_1440580 to 1440581, EPI_ISL_1440583 to 1440584, EPI_ISL_1440586, EPI_ISL_1440588, EPI_ISL_1440590 to 1440591, EPI_ISL_1440593 to 1440595, EPI_ISL_1440597 to 1440600, EPI_ISL_1440602 to 1440607, EPI_ISL_1440611 to 1440615, EPI_ISL_1440617, EPI_ISL_1440622, EPI_ISL_1440625 to 1440627, EPI_ISL_1440629 to 1440632, EPI_ISL_1440634 to 1440638, EPI_ISL_1440641 to 1440642, EPI_ISL_1440645, EPI_ISL_1440648, EPI_ISL_1440650 to 1440651, EPI_ISL_1440653 to 1440654, EPI_ISL_1440658 to 1440659, EPI_ISL_1440661 to 1440665, EPI_ISL_1440667 to 1440668, EPI_ISL_1440670 to 1440674, EPI_ISL_1440676, EPI_ISL_1440678 to 1440680, EPI_ISL_1440682 to 1440684, EPI_ISL_1440686 to 1440689, EPI_ISL_1440692, EPI_ISL_1440694, EPI_ISL_1440697, EPI_ISL_1440701 to 1440708, EPI_ISL_1440710 to 1440712, EPI_ISL_1440715 to 1440727, EPI_ISL_1440729 to 1440731, EPI_ISL_1441934 to 1441990, EPI_ISL_1442369 to 1442370, EPI_ISL_1442372 to 1442373, EPI_ISL_1442375, EPI_ISL_1442378, EPI_ISL_1442383 to 1442384, EPI_ISL_1442388, EPI_ISL_1442393, EPI_ISL_1442397, EPI_ISL_1442402 to 1442403, EPI_ISL_1442405 to 1442406, EPI_ISL_1442411, EPI_ISL_1442413, EPI_ISL_1442416 to 1442417, EPI_ISL_1442421 to 1442422, EPI_ISL_1442427, EPI_ISL_1442429, EPI_ISL_1442434, EPI_ISL_1442436, EPI_ISL_1442439, EPI_ISL_1442441, EPI_ISL_1442444, EPI_ISL_1442460, EPI_ISL_1442465, EPI_ISL_1442474, EPI_ISL_1442478, EPI_ISL_1442480, EPI_ISL_1442482, EPI_ISL_1442484			
see above	SYNLAB MVZ Leverkusen	Robert Koch Institute	
EPI_ISL_1432078, EPI_ISL_1432104, EPI_ISL_1432113, EPI_ISL_1432116, EPI_ISL_1432199, EPI_ISL_1432263, EPI_ISL_1432288, EPI_ISL_1432639, EPI_ISL_1432656, EPI_ISL_1440129, EPI_ISL_1440215, EPI_ISL_1440592, EPI_ISL_1440596, EPI_ISL_1440628, EPI_ISL_1440649, EPI_ISL_1440655, EPI_ISL_1440669, EPI_ISL_1440675, EPI_ISL_1440693, EPI_ISL_1440713			
see above	SYNLAB MVZ Neustadt a. d. Weinstraße	Robert Koch Institute	
EPI_ISL_1437614 to 1437617, EPI_ISL_1437619, EPI_ISL_1437622 to 1437633, EPI_ISL_1437635 to 1437641, EPI_ISL_1437643 to 1437644, EPI_ISL_1437646, EPI_ISL_1437648, EPI_ISL_1437651 to 1437652, EPI_ISL_1437654 to 1437662, EPI_ISL_1437665 to 1437666, EPI_ISL_1437669 to 1437680, EPI_ISL_1437682 to 1437685, EPI_ISL_1437687, EPI_ISL_1437689, EPI_ISL_1437691, EPI_ISL_1437693, EPI_ISL_1437695, EPI_ISL_1437697, EPI_ISL_1437700, EPI_ISL_1437702, EPI_ISL_1437705, EPI_ISL_1437708, EPI_ISL_1437710, EPI_ISL_1437712, EPI_ISL_1437714, EPI_ISL_1437716, EPI_ISL_1437721, EPI_ISL_1437724, EPI_ISL_1437726, EPI_ISL_1437731 to 1437733, EPI_ISL_1437735, EPI_ISL_1437738 to 1437741, EPI_ISL_1437743 to 1437745, EPI_ISL_1437747, EPI_ISL_1437752 to 1437753, EPI_ISL_1437755 to 1437756, EPI_ISL_1437758 to 1437761, EPI_ISL_1437763, EPI_ISL_1437766, EPI_ISL_1437768 to 1437771, EPI_ISL_1437773, EPI_ISL_1437775, EPI_ISL_1437778 to 1437779, EPI_ISL_1437782 to 1437785, EPI_ISL_1437787, EPI_ISL_1437789 to 1437809, EPI_ISL_1437811 to 1437813, EPI_ISL_1437817 to 1437818, EPI_ISL_1437820 to 1437836, EPI_ISL_1437838 to 1437842, EPI_ISL_1437844, EPI_ISL_1437849 to 1437858, EPI_ISL_1437860, EPI_ISL_1437862, EPI_ISL_1437864 to 1437868, EPI_ISL_1437870 to 1437880, EPI_ISL_1437883 to 1437889, EPI_ISL_1437891, EPI_ISL_1437894 to 1437900, EPI_ISL_1437902 to 1437903, EPI_ISL_1437906 to 1437908, EPI_ISL_1437910, EPI_ISL_1437912, EPI_ISL_1437914, EPI_ISL_1437916 to 1437922, EPI_ISL_1437924, EPI_ISL_1437926, EPI_ISL_1437928 to 1437931, EPI_ISL_1440280, EPI_ISL_1440288, EPI_ISL_1440293, EPI_ISL_1440299 to 1440300, EPI_ISL_1440302, EPI_ISL_1440306, EPI_ISL_1440311, EPI_ISL_1440315, EPI_ISL_1440321, EPI_ISL_1440328 to 1440331, EPI_ISL_1440333 to 1440334, EPI_ISL_1440342 to 1440344, EPI_ISL_1440347 to 1440348, EPI_ISL_1440353, EPI_ISL_1440362, EPI_ISL_1440365, EPI_ISL_1440368, EPI_ISL_1440372, EPI_ISL_1440374, EPI_ISL_1440378, EPI_ISL_1440380 to 1440381, EPI_ISL_1440383, EPI_ISL_1440385, EPI_ISL_1440387 to 1440388, EPI_ISL_1440390 to 1440391, EPI_ISL_1440395, EPI_ISL_1440397 to 1440398, EPI_ISL_1440405, EPI_ISL_1440407 to 1440408, EPI_ISL_1440410, EPI_ISL_1440415, EPI_ISL_1440419 to 1440420, EPI_ISL_1440430 to 1440431, EPI_ISL_1440433, EPI_ISL_1440435, EPI_ISL_1440445, EPI_ISL_1440447 to 1440448, EPI_ISL_1440450, EPI_ISL_1440453, EPI_ISL_1440455 to 1440456, EPI_ISL_1440461, EPI_ISL_1440466, EPI_ISL_1440473, EPI_ISL_1440476, EPI_ISL_1440482, EPI_ISL_1440484, EPI_ISL_1440491, EPI_ISL_1440495, EPI_ISL_1440497, EPI_ISL_1440499, EPI_ISL_1440501 to 1440506, EPI_ISL_1440508 to 1440509, EPI_ISL_1440511 to 1440514, EPI_ISL_1440517, EPI_ISL_1440519, EPI_ISL_1440522, EPI_ISL_1440526, EPI_ISL_1440528, EPI_ISL_1440539, EPI_ISL_1440541, EPI_ISL_1440543, EPI_ISL_1440547, EPI_ISL_1440549, EPI_ISL_1440552 to 1440553, EPI_ISL_1440555, EPI_ISL_1440564 to 1440565, EPI_ISL_1440575, EPI_ISL_1442586 to 1442587, EPI_ISL_1442589 to 1442592, EPI_ISL_1442594 to 1442597, EPI_ISL_1442599, EPI_ISL_1442601 to 1442603, EPI_ISL_1442605, EPI_ISL_1442607, EPI_ISL_1442610, EPI_ISL_1442615, EPI_ISL_1442618 to 1442621, EPI_ISL_1442623 to 1442625, EPI_ISL_1442627, EPI_ISL_1442629 to 1442630, EPI_ISL_1442632, EPI_ISL_1442635, EPI_ISL_1442637 to 1442638, EPI_ISL_1442640, EPI_ISL_1442642 to 1442643, EPI_ISL_1442645, EPI_ISL_1442647 to 1442649, EPI_ISL_1442651 to 1442653, EPI_ISL_1442655, EPI_ISL_1442657, EPI_ISL_1442659, EPI_ISL_1442665, EPI_ISL_1442668 to 1442670, EPI_ISL_1442672 to 1442675, EPI_ISL_1442677, EPI_ISL_1442679, EPI_ISL_1442681, EPI_ISL_1442683 to 1442696, EPI_ISL_1442698 to 1442706, EPI_ISL_1442708, EPI_ISL_1442710, EPI_ISL_1442713, EPI_ISL_1442716 to 1442724, EPI_ISL_1442726, EPI_ISL_1442729 to 1442730, EPI_ISL_1442733 to 1442743, EPI_ISL_1442745 to 1442750			
see above	SYNLAB MVZ Trier	Robert Koch Institute	
EPI_ISL_1437645, EPI_ISL_1437650, EPI_ISL_1437664, EPI_ISL_1437681, EPI_ISL_1437719, EPI_ISL_1437777, EPI_ISL_1437780, EPI_ISL_1437786, EPI_ISL_1437816, EPI_ISL_1437843, EPI_ISL_1437881 to 1437882, EPI_ISL_1437904, EPI_ISL_1440277, EPI_ISL_1440285 to 1440286, EPI_ISL_1440291, EPI_ISL_1440317 to 1440318, EPI_ISL_1440325 to 1440326, EPI_ISL_1440340 to 1440341, EPI_ISL_1440360, EPI_ISL_1440363, EPI_ISL_1440375 to 1440377, EPI_ISL_1440392, EPI_ISL_1440417, EPI_ISL_1440423 to 1440424, EPI_ISL_1440427 to 1440428, EPI_ISL_1440434, EPI_ISL_1440436 to 1440437, EPI_ISL_1440441, EPI_ISL_1440449, EPI_ISL_1440457, EPI_ISL_1440468 to 1440469, EPI_ISL_1440472, EPI_ISL_1440481, EPI_ISL_1440483, EPI_ISL_1440489, EPI_ISL_1440493, EPI_ISL_1440550, EPI_ISL_1440556, EPI_ISL_1440570, EPI_ISL_1440576, EPI_ISL_1442604, EPI_ISL_1442609, EPI_ISL_1442613 to 1442614, EPI_ISL_1442633, EPI_ISL_1442663, EPI_ISL_1442711, EPI_ISL_1442732, EPI_ISL_1442744			
see above	SYNLAB MVZ Weiden	Robert Koch Institute	
EPI_ISL_1468464 to 1468465, EPI_ISL_1468474, EPI_ISL_1493581	Sae servico de Atendimento Especializado	Instituto Adolfo Lutz, Interdiciplinary Procedures Center, Strategic Laboratory	Caio Vinicius Dias Lopes; Claudia Regina Gonçalves; Claudio Tavares Sacchi; Erica Valessa Ramos Gomes; Karoline Rodrigues Campos
EPI_ISL_479797 to 479798, EPI_ISL_479802 to 479804, EPI_ISL_479870 to 479871	Sagamihara City Public Health Research Institute	Pathogen Genomics Center, National Institute of Infectious Diseases	Hajime Kamiya; Hiroshi Nakamura; Kentaro Itokawa; Makoto Kuroda; Masanori Hashino; Motoi Suzuki; Rina Tanaka; Tsuyoshi Sekizuka
EPI_ISL_512072 to 512074	Saikrishna Hospital, Mehsana	Gujarat Biotechnology Research Centre	A M Kadri; Afzal Ansari; Apurvasinh Puvar; Chaitanya Joshi; Dinesh Kumar; Harsh Bakshi; Harshadbhai Parmar; Janvi Raval; Komal Patel; Labdhi Pandya; Madhvi Joshi; Maharshi Pandya; Monika Gandhi; Nidhi Patel; Nikha Trivedi; Nitin Savaliya; Pinal Trivedi; R D Dixit; Raghawendra Kumar; Zarna Patel; Zuber Saiyed
EPI_ISL_586567 to 586568	Saikrishna Hospital,Mehsana	Gujarat Biotechnology Research Centre	A M Kadri; Afzal Ansari; Apurvasinh Puvar; Chaitanya Joshi; Dinesh Kumar; Harsh Bakshi; Harshadbhai Parmar; Janvi Raval; Komal Patel; Labdhi Pandya; Madhvi Joshi; Maharshi Pandya; Monika Gandhi; Nidhi Patel; Nikha Trivedi; Nitin Savaliya; Pinal Trivedi; R D Dixit; Raghawendra Kumar; Zarna Patel; Zuber Saiyed
EPI_ISL_416525	Saitama Medical University	Saitama Medical University	Kazuo Imai
EPI_ISL_416524	Saitama Medical University Hospital	Saitama Medical University	Kazuo Imai
EPI_ISL_419301 to 419308, EPI_ISL_479805 to 479808, EPI_ISL_479928 to 479935, EPI_ISL_1425569 to 1425571, EPI_ISL_1425579 to 1425585, EPI_ISL_1425587, EPI_ISL_1425696, EPI_ISL_1425735 to 1425739, EPI_ISL_1425748, EPI_ISL_1426292 to 1426370, EPI_ISL_1426569, EPI_ISL_1426582 to 1426586, EPI_ISL_1426591, EPI_ISL_1426632, EPI_ISL_1426636 to 1426637, EPI_ISL_1426643 to 1426644, EPI_ISL_1426650 to 1426652, EPI_ISL_1426661, EPI_ISL_1426996 to 1427051, EPI_ISL_1427340 to 1427505, EPI_ISL_1427534 to 1427552, EPI_ISL_1428590, EPI_ISL_1430687 to 1430690, EPI_ISL_1430695 to 1430696, EPI_ISL_1430698, EPI_ISL_1430763 to 1430772, EPI_ISL_1431136 to 1431170, EPI_ISL_1431739 to 1431740, EPI_ISL_1431745 to 1431748, EPI_ISL_1437618			
see above	Saitama Prefectural Institute of Public Health	Pathogen Genomics Center, National Institute of Infectious Diseases	; Chang-Kweng Lim; Daisuke Kobayashi; Eri Nakayama; Hajime Kamiya; Hayato Ehara; Hussein H. Aly; Ken Miyazawa; Kentaro Itokawa; Kento Fukano; Kousho Wakae; Makoto Kuroda; Masanori Hashino; Michiyo Shinohara; Minoru Nagi; Motohiko Ogawa; Motoi Suzuki; Nozomu Hanaoka; Rina Tanaka; Shigeru Tanjima; Takahiro Maeki; Takanobu Kato; Takashi Sakudoh; Tsuguto Fujimoto; Tsuyoshi Kishimoto; Tsuyoshi Sekizuka
EPI_ISL_479925 to 479927, EPI_ISL_480065 to 480072, EPI_ISL_1426568, EPI_ISL_1426592 to 1426593, EPI_ISL_1427052 to 1427070, EPI_ISL_1427655, EPI_ISL_1427716, EPI_ISL_1428251 to 1428259			
see above	Sakai City Institute of Public Health	Pathogen Genomics Center, National Institute of Infectious Diseases	Hajime Kamiya; Kentaro Itokawa; Makoto Kuroda; Masanori Hashino; Motoi Suzuki; Rina Tanaka; Tatsuya Miyoshi; Tsuyoshi Sekizuka
EPI_ISL_1538799 to 1538802, EPI_ISL_1539134	Salve Medica Sp. Z o. o. sp. K.	1. National Institute of Public Health - National Institute of Hygiene; 2. Eurofins Genomics Europe Sequencing GmbH	ECDC COVID-19 WGS support team; Eurofins Genomics Europe Sequencing Team; Gierczyki Rafa; Sadkowska-Todys Magorzata; Wokowicz Tomasz; Zacharczuk Katarzyna
EPI_ISL_583173 to 583189	San Bernardino County Public Health Lab	Chan-Zuckerberg Biohub	CZB Cliahub Consortium
EPI_ISL_467950 to 467966, EPI_ISL_467968 to 467971, EPI_ISL_467973 to 467975, EPI_ISL_467977 to 467980, EPI_ISL_467982 to 467983, EPI_ISL_483158 to 483164, EPI_ISL_483531 to 483541, EPI_ISL_494372 to 494503, EPI_ISL_494568 to 494569, EPI_ISL_494571 to 494595, EPI_ISL_494620 to 494634, EPI_ISL_494714 to 494746, EPI_ISL_512158 to 512273, EPI_ISL_1468832, EPI_ISL_1468837, EPI_ISL_1468839, EPI_ISL_1468841, EPI_ISL_1468843 to 1468844, EPI_ISL_1468847, EPI_ISL_1468854, EPI_ISL_1468874 to 1468881, EPI_ISL_1468906 to 1468909, EPI_ISL_1468914 to 1468916, EPI_ISL_1468922 to 1468946, EPI_ISL_1468948 to 1468951, EPI_ISL_1468953, EPI_ISL_1468959, EPI_ISL_1468979 to 1469042, EPI_ISL_1531942 to 1532064			
see above	San Diego County Public Health Laboratory	Andersen lab at Scripps Research	Brett Austin; Ellen Stefanski; Ian Mchardy; Jovan Shephard; SEARCH Alliance San Diego with Michael Quigley; SEARCH Alliance San Diego with Tracy Basler
EPI_ISL_513856 to 513882, EPI_ISL_583190 to 583197	San Francisco Public Health Laboratory	Chan-Zuckerberg Biohub	CZB Cliahub Consortium
EPI_ISL_1492566, EPI_ISL_1492570, EPI_ISL_1492574	San Gallicano Dermatological Institute I.F.O.	INMI Lazzaro Spallanzani IRCCS	A Di Caro; A Mastrofrancesco; B Bartolini; CEM Gruber; E Giombini; EG Di Domenico; F Ensoli; F Messina; F Pimpinelli; F Santini; G Bonfiglio; G Prignano; M Rueca; MR Capobianchi; O Butera

EPI_ISL_468506 to 468559, EPI_ISL_486287 to 486338	San Joaquin County Public Health Lab	Chan-Zuckerberg Biohub	CZB Cliahub Consortium
EPI_ISL_1400982 to 1400983	San Juan Regional Medical Center	New Mexico Department of Health Scientific Laboratory	Anastacia Griego-Fisher; D'eldra Malone; Ellie Johnson; Jennifer Benoit
EPI_ISL_491465 to 491466, EPI_ISL_491471	San Lazaro Hospital	Research Institute for Tropical Medicine	Catalino Demetria; Criselda Bautista; Daria Manalo; Edelwisa Mercado; Francisco Gerardo Polotan; Inez Andrea Medado; Kirstyn Brunker; Ma. Angelica Tujan; Othoniel Jan Onza
EPI_ISL_582959 to 582974	San Luis Obispo Public Health Department	Chan-Zuckerberg Biohub	CZB Cliahub Consortium
EPI_ISL_542278 to 542399	San Matteo Hospital Pavia	Dep. Of Oncology and Hemato-Oncology University of Milan	Antonio Piralla; Carlo Federico Perno; Chiara Vismara; Claudia Alteri; Elisa Matarazzo; Fausto Baldanti; Federica Giardina; Federica Novazzi; Luna Colagrossi; Maria Antonello; Massimo Puoti; Monica Tallarita; Oscar Massimiliano Epis; Roberto Fumagalli; Silvia Renica; Stefano Gaiarsa; Valentino Costabile; Valeria Cento
EPI_ISL_603000 to 603003	Sanford South University Medical Center	Minnesota Department of Health, Public Health Laboratory	Alexandra Lorentz; Jacob Garfin; Matt Plumb; and Xiong Wang
EPI_ISL_1439597, EPI_ISL_1477047 to 1477049	Sanitary-Epidemiological And Public Health Department Of Tashkent Region, Uzbekistan	Center of Genomics and bioinformatics, Bioinformatics laboratory	Abdurakhmon N Yusupov; Ibrokhim Y Abdurakhmonov.; Mirzakamol S Ayubov; Mukhammadjon H Mirzakhmedov; Shukhrat E Shermatov; Zabardast T Buriev
EPI_ISL_583492	Santa Casa Anna Cintra	Instituto Adolfo Lutz, Interdiciplinary Procedures Center, Strategic Laboratory	Claudia Regina Gonçalves; Claudio Tavares Sacchi; Erica Valessa Ramos Gomes; Karoline Rodrigues Campos
EPI_ISL_547580, EPI_ISL_603026, EPI_ISL_603032	Santa Casa da Misericórdia de Presidente Prudente	Instituto Adolfo Lutz, Interdiciplinary Procedures Center, Strategic Laboratory	Claudia Regina Gonçalves; Claudio Tavares Sacchi; Erica Valessa Ramos Gomes; Karoline Rodrigues Campos
EPI_ISL_1468454	Santa Casa de Andradina	Instituto Adolfo Lutz, Interdiciplinary Procedures Center, Strategic Laboratory	Caio Vinicius Dias Lopes; Claudia Regina Gonçalves; Claudio Tavares Sacchi; Erica Valessa Ramos Gomes; Karoline Rodrigues Campos
EPI_ISL_1533721	Santa Casa de Aracatuba Hospital Sagrado Coracao De Jesus	Instituto Adolfo Lutz, Interdiciplinary Procedures Center, Strategic Laboratory	Caio Vinicius Dias Lopes; Claudia Regina Gonçalves; Claudio Tavares Sacchi; Erica Valessa Ramos Gomes; Karoline Rodrigues Campos; Leonardo Jose Tadeu de Araujo
EPI_ISL_1468417, EPI_ISL_1468419, EPI_ISL_1468424, EPI_ISL_1468449 to 1468450, EPI_ISL_1468461, EPI_ISL_1533715	Santa Casa de Aracatuba Hospital Sagrado Coracao de Jesus	Instituto Adolfo Lutz, Interdiciplinary Procedures Center, Strategic Laboratory	Caio Vinicius Dias Lopes; Claudia Regina Gonçalves; Claudio Tavares Sacchi; Erica Valessa Ramos Gomes; Karoline Rodrigues Campos; Leonardo Jose Tadeu de Araujo
EPI_ISL_1533704	Santa Casa de Atibaia Pro Saude	Instituto Adolfo Lutz, Interdiciplinary Procedures Center, Strategic Laboratory	Caio Vinicius Dias Lopes; Claudia Regina Gonçalves; Claudio Tavares Sacchi; Erica Valessa Ramos Gomes; Karoline Rodrigues Campos; Leonardo Jose Tadeu de Araujo
EPI_ISL_1468420 to 1468421, EPI_ISL_1468425, EPI_ISL_1468442, EPI_ISL_1468448, EPI_ISL_1468460, EPI_ISL_1533694	Santa Casa de Birigui	Instituto Adolfo Lutz, Interdiciplinary Procedures Center, Strategic Laboratory	Caio Vinicius Dias Lopes; Claudia Regina Gonçalves; Claudio Tavares Sacchi; Erica Valessa Ramos Gomes; Karoline Rodrigues Campos; Leonardo Jose Tadeu de Araujo
EPI_ISL_1493592	Santa Casa de Guaira	Instituto Adolfo Lutz, Interdiciplinary Procedures Center, Strategic Laboratory	Caio Vinicius Dias Lopes; Claudia Regina Gonçalves; Claudio Tavares Sacchi; Erica Valessa Ramos Gomes; Karoline Rodrigues Campos
EPI_ISL_603024, EPI_ISL_603027, EPI_ISL_603038	Santa Casa de Misericordia de Araçatuba	Instituto Adolfo Lutz, Interdiciplinary Procedures Center, Strategic Laboratory	Claudia Regina Gonçalves; Claudio Tavares Sacchi; Erica Valessa Ramos Gomes; Karoline Rodrigues Campos
EPI_ISL_1468445, EPI_ISL_1468453	Santa Casa de Misericordia de Pereira Barreto	Instituto Adolfo Lutz, Interdiciplinary Procedures Center, Strategic Laboratory	Caio Vinicius Dias Lopes; Claudia Regina Gonçalves; Claudio Tavares Sacchi; Erica Valessa Ramos Gomes; Karoline Rodrigues Campos
EPI_ISL_524469	Santa Casa de Misericordia de Sao Paulo	Instituto Adolfo Lutz, Interdiciplinary Procedures Center, Strategic Laboratory	Claudia Regina Gonçalves; Claudio Tavares Sacchi; Erica Valessa Ramos Gomes
EPI_ISL_547579	Santa Casa de Misericórdia de Araçatuba	Instituto Adolfo Lutz, Interdiciplinary Procedures Center, Strategic Laboratory	Claudia Regina Gonçalves; Claudio Tavares Sacchi; Erica Valessa Ramos Gomes; Karoline Rodrigues Campos
EPI_ISL_1533720	Santa Casa de Penapolis	Instituto Adolfo Lutz, Interdiciplinary Procedures Center, Strategic Laboratory	Caio Vinicius Dias Lopes; Claudia Regina Gonçalves; Claudio Tavares Sacchi; Erica Valessa Ramos Gomes; Karoline Rodrigues Campos; Leonardo Jose Tadeu de Araujo
EPI_ISL_603031	Santa Casa de Presidente Epitácio	Instituto Adolfo Lutz, Interdiciplinary Procedures Center, Strategic Laboratory	Claudia Regina Gonçalves; Claudio Tavares Sacchi; Erica Valessa Ramos Gomes; Karoline Rodrigues Campos
EPI_ISL_524464	Santa Casa de Santa Isabel	Instituto Adolfo Lutz, Interdiciplinary Procedures Center, Strategic Laboratory	Claudia Regina Gonçalves; Claudio Tavares Sacchi; Erica Valessa Ramos Gomes
EPI_ISL_1468466	Santa Casa de Sao Carlos	Instituto Adolfo Lutz, Interdiciplinary Procedures Center, Strategic Laboratory	Caio Vinicius Dias Lopes; Claudia Regina Gonçalves; Claudio Tavares Sacchi; Erica Valessa Ramos Gomes; Karoline Rodrigues Campos
EPI_ISL_1533692	Santa Casa de Sao Paulo	Instituto Adolfo Lutz, Interdiciplinary Procedures Center, Strategic Laboratory	Caio Vinicius Dias Lopes; Claudia Regina Gonçalves; Claudio Tavares Sacchi; Erica Valessa Ramos Gomes; Karoline Rodrigues Campos; Leonardo Jose Tadeu de Araujo
EPI_ISL_417317 to 417321, EPI_ISL_429879 to 429880, EPI_ISL_435580 to 435673	Santa Clara County Public Health Department	Chiu Laboratory, University of California, San Francisco	Brandon Bonin; Debra A. Wadford; Elsa Villarino; Scot Federman; Wei Gu; Xianding Deng; and Charles Y. Chiu
EPI_ISL_1477441, EPI_ISL_1477445, EPI_ISL_1477455, EPI_ISL_1477461, EPI_ISL_1477484, EPI_ISL_1477486, EPI_ISL_1477494, EPI_ISL_1477496, EPI_ISL_1477501, EPI_ISL_1477507, EPI_ISL_1477510, EPI_ISL_1477518 to 1477519, EPI_ISL_1477529, EPI_ISL_1477534 to 1477535, EPI_ISL_1477540 to 1477541, EPI_ISL_1477552, EPI_ISL_1477558, EPI_ISL_1477566, EPI_ISL_1477575, EPI_ISL_1477578, EPI_ISL_1477581, EPI_ISL_1477590, EPI_ISL_1477598, EPI_ISL_1477603, EPI_ISL_1477622, EPI_ISL_1477637, EPI_ISL_1477644, EPI_ISL_1477655, EPI_ISL_1477664, EPI_ISL_1477670, EPI_ISL_1477677, EPI_ISL_1477681 to 1477682, EPI_ISL_1477690, EPI_ISL_1477695, EPI_ISL_1477699, EPI_ISL_1477706, EPI_ISL_1477722, EPI_ISL_1477726 to 1477727, EPI_ISL_1477729 to 1477731, EPI_ISL_1477734, EPI_ISL_1477745, EPI_ISL_1477749 to 1477750, EPI_ISL_1477752, EPI_ISL_1477755, EPI_ISL_1477762, EPI_ISL_1477767 to 1477768, EPI_ISL_1477776, EPI_ISL_1477780, EPI_ISL_1477790, EPI_ISL_1477793, EPI_ISL_1477797, EPI_ISL_1477799 to 1477800, EPI_ISL_1477814, EPI_ISL_1477817 to 1477818, EPI_ISL_1477822, EPI_ISL_1477836, EPI_ISL_1477839, EPI_ISL_1477842, EPI_ISL_1477845 to 1477846, EPI_ISL_1477850, EPI_ISL_1477854, EPI_ISL_1477856, EPI_ISL_1477859 to 1477860, EPI_ISL_1477863, EPI_ISL_1477871, EPI_ISL_1477874, EPI_ISL_1477878 to 1477879, EPI_ISL_1477881 to 1477882, EPI_ISL_1477885, EPI_ISL_1477888, EPI_ISL_1477892 to 1477893, EPI_ISL_1477895, EPI_ISL_1477902, EPI_ISL_1477907, EPI_ISL_1477909, EPI_ISL_1477912, EPI_ISL_1477923, EPI_ISL_1477925 to 1477926, EPI_ISL_1477935, EPI_ISL_1477937, EPI_ISL_1477943, EPI_ISL_1477958, EPI_ISL_1477961, EPI_ISL_1477963, EPI_ISL_1477967, EPI_ISL_1477974, EPI_ISL_1477979, EPI_ISL_1477982 to 1477983, EPI_ISL_1478012 to 1478013, EPI_ISL_1478019, EPI_ISL_1478028, EPI_ISL_1478031, EPI_ISL_1478038, EPI_ISL_1478046, EPI_ISL_1478048, EPI_ISL_1478050, EPI_ISL_1478059, EPI_ISL_1478081, EPI_ISL_1478084, EPI_ISL_1478087, EPI_ISL_1478089, EPI_ISL_1478092, EPI_ISL_1478095, EPI_ISL_1478097, EPI_ISL_1478102 to 1478103, EPI_ISL_1478123 to 1478124, EPI_ISL_1478129, EPI_ISL_1478149, EPI_ISL_1478152 to 1478153, EPI_ISL_1478161, EPI_ISL_1478170 to 1478171, EPI_ISL_1478192, EPI_ISL_1478200, EPI_ISL_1529101 to 1529102, EPI_ISL_1529106 to 1529110, EPI_ISL_1529113, EPI_ISL_1529119, EPI_ISL_1529121, EPI_ISL_1529137 to 1529138, EPI_ISL_1529148, EPI_ISL_1529151 to 1529152, EPI_ISL_1529154, EPI_ISL_1529162, EPI_ISL_1529173, EPI_ISL_1529183, EPI_ISL_1529191, EPI_ISL_1529193, EPI_ISL_1529205 to 1529207, EPI_ISL_1529218, EPI_ISL_1529221, EPI_ISL_1529224, EPI_ISL_1529226, EPI_ISL_1529234, EPI_ISL_1529238, EPI_ISL_1529240 to 1529241, EPI_ISL_1529245, EPI_ISL_1529252, EPI_ISL_1529258 to 1529259, EPI_ISL_1529262 to 1529263, EPI_ISL_1529267, EPI_ISL_1529273, EPI_ISL_1529283 to 1529284, EPI_ISL_1529287, EPI_ISL_1529295, EPI_ISL_1529309, EPI_ISL_1529316 to 1529317, EPI_ISL_1529344, EPI_ISL_1529347, EPI_ISL_1529349, EPI_ISL_1529351			
see above	Santa Clara County Public Health Laboratory	Chan-Zuckerberg Biohub	CZB Cliahub Consortium
EPI_ISL_1406155 to 1406176, EPI_ISL_1483802, EPI_ISL_1508964 to 1508965, EPI_ISL_1532139 to 1532140, EPI_ISL_1532144	Santa Clara County Public Health Laboratory	Santa Clara County Public Health Laboratory	Santa Clara County Public Health Department
EPI_ISL_1483799, EPI_ISL_1532127 to 1532138	Santa Clara Valley Medical Center	Santa Clara County Public Health Laboratory	Santa Clara County Public Health Department
EPI_ISL_529718	Santo Borromeus Hospital	Laboratorium Kesehatan Provinsi Jawa Barat; School of Life Sciences and Technology & School of Pharmacy-Institut Teknologi Bandung; Molecular Genetics Laboratory-Faculty of Medicine-Universitas Padjadjaran	Adelina Khristiani Rahayu; Agung Eru Wibowo; Azzania Fibriani; Catur Riani; Cut Nur Cinthia Alamanda; Ema Rahmawati; Gusti Ayu Prani Pradani; Hammam Riza; Hesti Lina Wiraswati; Husna Nugrahapraja; Irvan Faizal; Lia Faridah; Marselina Irasonia Tan; Rifky Waluyajati Rachman; Ryan Bayusantika Ristandi; Savira Ekawardhani; Sony Solistia Wirawan; Tarwadi; Yunia Sribudiani

EPI_ISL_528751	Santo Borromeus Hospital	Molecular Genetics Laboratory-Faculty of Medicine-Universitas Padjadjaran; School of Life Sciences and Technology & School of Pharmacy-Institut Teknologi Bandung; Laboratorium Kesehatan Provinsi Jawa Barat	Agung Eru Wibowo; Azzania Fibriani; Catur Riani; Cut Nur Cinthia Alamanda; Ema Rahmawati; Fensi Amalina; Hammam Riza; Hesti Lina Wiraswati; Husna Nugrahapraja; Irvan Faizal; Lia Faridah; Marselina Irasonia Tan; Mas Rizky A.A. Syamsunarno; Rifky Waluyajati Rachman; Ryan Bayusantika Ristandi; Savira Ekawardhani; Sony Solistia Wirawan; Tarwadi; Tri Hanggono Achmad; Yunia Sribudiani
EPI_ISL_528750, EPI_ISL_528759	Santo Borromeus Hospital	School of Life Sciences and Technology & School of Pharmacy-Institut Teknologi Bandung; Molecular Genetics Laboratory-Faculty of Medicine-Universitas Padjadjaran; Laboratorium Kesehatan Provinsi Jawa Barat	Agung Eru Wibowo; Azzania Fibriani; Catur Riani; Cut Nur Cinthia Alamanda; Ema Rahmawati; Fensi Amalina; Hammam Riza; Hesti Lina Wiraswati; Husna Nugrahapraja; Irvan Faizal; Karimatu Khoirunnisa; Lia Faridah; Marselina Irasonia Tan; Mas Rizky A.A. Syamsunarno; Miftahul Faridl; Rifky Waluyajati Rachman; Ryan Bayusantika Ristandi; Savira Ekawardhani; Sony Solistia Wirawan; Tarwadi; Tri Hanggono Achmad; Yunia Sribudiani
EPI_ISL_528747	Santo Borromeus Hospital	School of Pharmacy & School of Life Sciences and Technology - Institut Teknologi Bandung; Molecular Genetics Laboratory-Faculty of Medicine-Universitas Padjadjaran; Laboratorium Kesehatan Provinsi Jawa Barat	Agung Eru Wibowo; Azzania Fibriani; Catur Riani; Cut Nur Cinthia Alamanda; Ema Rahmawati; Hammam Riza; Hesti Lina Wiraswati; Husna Nugrahapraja; Irvan Faizal; Karimatu Khoirunnisa; Lia Faridah; Marselina Irasonia Tan; Miftahul Faridl; Rifky Waluyajati Rachman; Ryan Bayusantika Ristandi; Savira Ekawardhani; Sony Solistia Wirawan; Tarwadi; Yunia Sribudiani
EPI_ISL_528749	Santosa Hospital Bandung Central	School of Life Sciences and Technology & School of Pharmacy-Institut Teknologi Bandung; Molecular Genetics Laboratory-Faculty of Medicine-Universitas Padjadjaran; Laboratorium Kesehatan Provinsi Jawa Barat	Agung Eru Wibowo; Azzania Fibriani; Catur Riani; Cut Nur Cinthia Alamanda; Davin H. E. Setiamarga; Ema Rahmawati; Hammam Riza; Hesti Lina Wiraswati; Husna Nugrahapraja; Irvan Faizal; Lia Faridah; Marselina Irasonia Tan; Rifky Waluyajati Rachman; Rizki Mardian; Ryan Bayusantika Ristandi; Savira Ekawardhani; Sony Solistia Wirawan; Tarwadi; Yunia Sribudiani
EPI_ISL_511878	Santosa Hospital Bandung Kopo	Laboratorium Kesehatan Provinsi Jawa Barat; Molecular Genetics Laboratory-Faculty of Medicine-Universitas Padjadjaran; School of Life Sciences and Technology & School of Pharmacy-Institut Teknologi Bandung	Agung Eru Wibowo; Azzania Fibriani; Catur Riani; Cut Nur Cinthia Alamanda; Ema Rahmawati; Hammam Riza; Hesti Lina Wiraswati; Husna Nugrahapraja; Lia Faridah; Marselina Irasonia Tan; Rifky Waluyajati Rachman; Ryan Bayusantika Ristandi; Savira Ekawardhani; Soni Solistia Wirawan; Tarwadi; Yunia Sribudiani
EPI_ISL_479799 to 479800, EPI_ISL_479826 to 479849, EPI_ISL_479872 to 479885	Sapporo City Institute of Public Health	Pathogen Genomics Center, National Institute of Infectious Diseases	Asami Ohnishi; Hajime Kamiya; Kentaro Itokawa; Makoto Kuroda; Masanori Hashino; Motoi Suzuki; Rina Tanaka; Tsuyoshi Sekizuka
EPI_ISL_1448409 to 1448423	Sapporo City Institute of Public Health	Sapporo City Institute of Public Health	Asami Ohnishi; Kentaro Itokawa; Makoto Kuroda; Masanori Hashino; Masayuki Kikuchi; Rie Shimazaki; Rina Tanaka; Tsuyoshi Sekizuka; Yusuke Oguchi
EPI_ISL_1443214, EPI_ISL_1443216 to 1443219, EPI_ISL_1443277 to 1443283	Sarah Busch Lincoln Health Center	Illinois Department of Public Health - Springfield Lab	Bryan Sim; Gordon McCall
EPI_ISL_512066 to 512069	Sardar Vallabhbhai Patel Institute of Medical Sciences & Research	Gujarat Biotechnology Research Centre	A M Kadri; Afzal Ansari; Apurvasinh Puvar; Chaitanya Joshi; Dinesh Kumar; Harsh Bakshi; Janvi Raval; Kamlesh J Upadhyay; Komal Patel; Labdhi Pandya; Madhvi Joshi; Maharshi Pandya; Monika Gandhi; Nidhi Patel; Nikha Trivedi; Nitin Savaliya; Pinal Trivedi; Pranay Shah; R D Dixit; Raghawendra Kumar; Sanjay Kapadia; Zarna Patel; Zuber Saiyed
EPI_ISL_434654, EPI_ISL_445222, EPI_ISL_445226, EPI_ISL_450808, EPI_ISL_450810, EPI_ISL_452231 to 452232	Saroledens Familjelakare	The Public Health Agency of Sweden	Anna Risberg; Anna-Malin Linde; Karin Tegmark-Wisell; Katarina Jarbur; Maria Lind Karlberg; Mia Brytting; Olov Svartstrom; Oskar Karlsson Lindsjo; Theresa Enkirch
EPI_ISL_455110	Scania Halsocenter, B288	The Public Health Agency of Sweden	Anna Risberg; Anna-Malin Linde; Christina Lergin; Karin Tegmark-Wisell; Maria Lind Karlberg; Mia Brytting; Olov Svartstrom; Oskar Karlsson Lindsjo; Theresa Enkirch
EPI_ISL_1527360 to 1527363, EPI_ISL_1527530 to 1527537, EPI_ISL_1531734, EPI_ISL_1531736 to 1531742	School of Pharmacy, Shenandoah University	School of Pharmacy, Shenandoah University	A.F.; Adams; G.W.; Harralson; Kidd; R.S.; S.M.; Sawyer
EPI_ISL_450403	School of Public Health, The University of Hong Kong	School of Public Health, The University of Hong Kong	Brackman; C.J.; Chu; D.K.W.; D.N.C.; L.D.; L.L.M. and Peiris, M.; Perera; Poon; R.A.P.M.; Sims; Sit; T.H.S.; Tsang
EPI_ISL_510529	School of Veterinary Medicine, Disease Control	School of Veterinary Medicine, Disease Control	A.L.; A.N.; Bates, M.; C. and Zumla, A.; Chambaro, H.; Chanda, D.; Changula, K.; Chilufya; Chipimo; Chitanga, S.; Fwoloshi, S.; K.S.; Kapata; Kapata, N.; Kapaya, F.; Kapin'a, M.; Kayeyi, N.; Liwewe; M.M.; Malama, K.; Masahiro, K.; Monze, M.; Morales; Mubemba, B.; Mukonka, V.; Mulenga, L.; Muleya, W.; Mupeta, F.; Musonda, K.; Nalubamba; Ngosa, W.; P.C.; P.J.; Saasa, N.; Sawa, H.; Shibemba; Simulundu, E.; Sinyange, N.; Takada, A.; Tembo, J.; Zulu, P.
EPI_ISL_437549 to 437600, EPI_ISL_445164 to 445168, EPI_ISL_450191 to 450192, EPI_ISL_458237 to 458284, EPI_ISL_467967, EPI_ISL_467972, EPI_ISL_494596 to 494619, EPI_ISL_494635 to 494713, EPI_ISL_1468842, EPI_ISL_1468849, EPI_ISL_1468882 to 1468888, EPI_ISL_1468947, EPI_ISL_1468955 to 1468956, EPI_ISL_1469043 to 1469060			
see above	Scripps Medical Laboratory	Andersen lab at Scripps Research	Brett Austin; Ellen Stefanski; Ian Mchardy; Jovan Shephard; SEARCH Alliance San Diego with Michael Quigley; SEARCH Alliance San Diego with Tracy Basler
EPI_ISL_413560, EPI_ISL_416459 to 416466, EPI_ISL_417163 to 417165, EPI_ISL_430113 to 430156, EPI_ISL_438140 to 438175, EPI_ISL_495654 to 495665, EPI_ISL_525699 to 525756, EPI_ISL_530124 to 530162, EPI_ISL_574648 to 574691, EPI_ISL_575028 to 575033, EPI_ISL_596387 to 596446, EPI_ISL_1468671 to 1468672, EPI_ISL_1468676 to 1468679, EPI_ISL_1468683, EPI_ISL_1468685 to 1468687, EPI_ISL_1468690 to 1468691, EPI_ISL_1468694, EPI_ISL_1468696, EPI_ISL_1468700, EPI_ISL_1468703, EPI_ISL_1468705, EPI_ISL_1468709, EPI_ISL_1468711, EPI_ISL_1468719, EPI_ISL_1468722, EPI_ISL_1468724, EPI_ISL_1468730 to 1468731, EPI_ISL_1468735, EPI_ISL_1468738 to 1468739, EPI_ISL_1468741 to 1468743, EPI_ISL_1468747, EPI_ISL_1468750 to 1468751, EPI_ISL_1468758 to 1468760, EPI_ISL_1468762, EPI_ISL_1468764, EPI_ISL_1468766, EPI_ISL_1468774 to 1468775, EPI_ISL_1468777, EPI_ISL_1468781, EPI_ISL_1468789, EPI_ISL_1468796, EPI_ISL_1468799, EPI_ISL_1468801, EPI_ISL_1468810, EPI_ISL_1468814 to 1468819, EPI_ISL_1503395 to 1503453, EPI_ISL_1516753			
see above	Seattle Flu Study	Seattle Flu Study	Amanda Adler; Barry R. Lutz; Benjamin Pelle; Brian Hiatt; Caitlin R. Wolf; Chris D. Frazar; Chu et al; Chu etl al; Deborah A. Nickerson; Elisabeth Brandstetter; Erica Ryke; Geoff Melly; Helen Y. Chu; Janet A. Englund; Jay Shendure; Jeff Duchin; Jover Lee; Kairsten Fay; Karen Cowgill; Kirsten Lacombe; Lea M. Starita; Mark J. Rieder; Matthew Richardson; Matthew Thompson; Melissa Truong; Michael Boeckh; Michael Famulare; Misja Ilcisin; Peter D. Han; Philip Dykema; Romesh Gautom; Scott Lindquist; Stephanie Schrag; Thomas R. Sibley; Trevor Bedford
EPI_ISL_413456	Seattle Flu Study, University of Washington Medical Center	Seattle Flu Study, University of Washington Medical Center	Chu et al
EPI_ISL_412026	Second Hospital of Anhui Medical University	Second Hospital of Anhui Medical University	Changtai Wang; Mengji Lu; Mengyuan Xua; Tengfei He; Xin Huang; Zhenhua Zhang; Zhongping Liua; Zixiang Chen
EPI_ISL_469253	Second Military Medical University, Department of Microbiology	Second Military Medical University, Department of Microbiology	Jiang, L.; Peng, H.; Qi; Tang, H.; Z. and Zhao, P.
EPI_ISL_1533722	Secretaria Municipal de Saude De Piracaia	Instituto Adolfo Lutz, Interdiciplinary Procedures Center, Strategic Laboratory	Caio Vinicius Dias Lopes; Claudia Regina Gonçalves; Claudio Tavares Sacchi; Erica Valessa Ramos Gomes; Karoline Rodrigues Campos; Leonardo Jose Tadeu de Araujo
EPI_ISL_1468467, EPI_ISL_1468470 to 1468471	Secretaria Municipal de Saude Descalvado	Instituto Adolfo Lutz, Interdiciplinary Procedures Center, Strategic Laboratory	Caio Vinicius Dias Lopes; Claudia Regina Gonçalves; Claudio Tavares Sacchi; Erica Valessa Ramos Gomes; Karoline Rodrigues Campos
EPI_ISL_1468469	Secretaria Municipal de Saude Porto Ferreira	Instituto Adolfo Lutz, Interdiciplinary Procedures Center, Strategic Laboratory	Caio Vinicius Dias Lopes; Claudia Regina Gonçalves; Claudio Tavares Sacchi; Erica Valessa Ramos Gomes; Karoline Rodrigues Campos
EPI_ISL_1468416, EPI_ISL_1468447	Secretaria Municipal de Saude de Andradina	Instituto Adolfo Lutz, Interdiciplinary Procedures Center, Strategic Laboratory	Caio Vinicius Dias Lopes; Claudia Regina Gonçalves; Claudio Tavares Sacchi; Erica Valessa Ramos Gomes; Karoline Rodrigues Campos
EPI_ISL_1468418, EPI_ISL_1468444, EPI_ISL_1468446, EPI_ISL_1533698	Secretaria Municipal de Saude de Birigui	Instituto Adolfo Lutz, Interdiciplinary Procedures Center, Strategic Laboratory	Caio Vinicius Dias Lopes; Claudia Regina Gonçalves; Claudio Tavares Sacchi; Erica Valessa Ramos Gomes; Karoline Rodrigues Campos; Leonardo Jose Tadeu de Araujo
EPI_ISL_468320	Secretaria Municipal de Saude de Hortolandia	Instituto Adolfo Lutz, Interdiciplinary Procedures Center, Strategic Laboratory	Claudia Regina Gonçalves; Claudio Tavares Sacchi; Erica Valessa Ramos Gomes
EPI_ISL_574583	Secretaria Municipal de Saude de Jandira	Instituto Adolfo Lutz, Interdiciplinary Procedures Center, Strategic Laboratory	Claudia Regina Gonçalves; Claudio Tavares Sacchi; Erica Valessa Ramos Gomes; Karoline Rodrigues Campos
EPI_ISL_574597	Secretaria Municipal de Saude de Jarinu	Instituto Adolfo Lutz, Interdiciplinary Procedures Center, Strategic Laboratory	Claudia Regina Gonçalves; Claudio Tavares Sacchi; Erica Valessa Ramos Gomes; Karoline Rodrigues Campos

EPI_ISL_1533693	Secretaria Municipal de Saude de Ubatuba	Instituto Adolfo Lutz, Interdiciplinary Procedures Center, Strategic Laboratory	Caio Vinicius Dias Lopes; Claudia Regina Gonçalves; Claudio Tavares Sacchi; Erica Valessa Ramos Gomes; Karoline Rodrigues Campos; Leonardo Jose Tadeu de Araujo
EPI_ISL_1468458 to 1468459	Secretaria Municipal de Saude de Valparaiso SP	Instituto Adolfo Lutz, Interdiciplinary Procedures Center, Strategic Laboratory	Caio Vinicius Dias Lopes; Claudia Regina Gonçalves; Claudio Tavares Sacchi; Erica Valessa Ramos Gomes; Karoline Rodrigues Campos
EPI_ISL_547576, EPI_ISL_603035	Secretaria Municipal de Saúde	Instituto Adolfo Lutz, Interdiciplinary Procedures Center, Strategic Laboratory	Claudia Regina Gonçalves; Claudio Tavares Sacchi; Erica Valessa Ramos Gomes; Karoline Rodrigues Campos
EPI_ISL_1469666	Secretaria Municipal de Saúde de Montenegro	Epiclin	Ana Paula Muterle; Carolina Comerlato; Eliana Márcia Da Ros Wendland; Fernando Hayashi Sant'Anna; Janira Prichula; Juliana Comerlato
EPI_ISL_1469551, EPI_ISL_1469556, EPI_ISL_1469727, EPI_ISL_1469757	Secretaria Municipal de Saúde de São Leopoldo	Epiclin	Ana Paula Muterle; Carolina Comerlato; Eliana Márcia Da Ros Wendland; Fernando Hayashi Sant'Anna; Janira Prichula; Juliana Comerlato
EPI_ISL_1469588, EPI_ISL_1469647, EPI_ISL_1469780	Secretaria Municipal de Saúde de Taquara	Epiclin	Ana Paula Muterle; Carolina Comerlato; Eliana Márcia Da Ros Wendland; Fernando Hayashi Sant'Anna; Janira Prichula; Juliana Comerlato
EPI_ISL_1469559, EPI_ISL_1469607, EPI_ISL_1469758, EPI_ISL_1469803, EPI_ISL_1469838, EPI_ISL_1469847	Secretaria Municipal de Saúde de Três Coroas	Epiclin	Ana Paula Muterle; Carolina Comerlato; Eliana Márcia Da Ros Wendland; Fernando Hayashi Sant'Anna; Janira Prichula; Juliana Comerlato
EPI_ISL_471542	Secretaria de Saude de Mogi das Cruzes	Instituto Adolfo Lutz, Interdiciplinary Procedures Center, Strategic Laboratory	Claudia Regina Gonçalves; Claudio Tavares Sacchi; Erica Valessa Ramos Gomes
EPI_ISL_1468468	Secretaria municipal de saude de Itapolis	Instituto Adolfo Lutz, Interdiciplinary Procedures Center, Strategic Laboratory	Caio Vinicius Dias Lopes; Claudia Regina Gonçalves; Claudio Tavares Sacchi; Erica Valessa Ramos Gomes; Karoline Rodrigues Campos
EPI_ISL_417924	Secretaría de Salud Medellín	Instituto Nacional de Salud, Universidad Cooperativa de Colombia, Instituto Alexander von Humboldt, Imperial College-London, London School of Hygiene & Tropical Medicine	Astrid C. Flórez; Carlos Franco-Muñoz; Christian Julian VillabonaArenas; Diana Marcela Walteros-Acero; Diego A. Álvarez-Díaz; Erika Ospitia; Gloria Puerto; Jose A. Usme-Ciro; Juliana Barbosa; Katherine Laiton-Donato; Liz Villabona-Arenas; Luz Dary Rodriguez; Mailyn A. Gonzalez; Marcela Mercado-Reyes; Martha Lucia Ospina Martinez; Nicolás D. Franco-Sierra; Sergio Gomez Rangel; Sussy Echeverria-Londoño; Zulma M. Cucunubá
EPI_ISL_1534339, EPI_ISL_1534356	Sedgefield Clinic wc SGE	NHLS/UCT	Arash Iranzadeh; Bruna Galvao; Carolyn Williamson; Deelan Doolabh; Diana Hardie; Emmanuel SJ; Innocent Mudau; Kruger Marais; Lynn Tyers; Marvin Hsiao; Stephen Korsman; Tegally H; de Oliveira T
EPI_ISL_1554887	Sentinelles Idf	National Reference Center for Viruses of Respiratory Infections, Institut Pasteur, Paris	Angela Brisebarre; Askin Dilek; Camille Capel; Christophe Malabat; Corinne Maufrais; Damien Mornico; Etienne Simon-Lorière; Frédéric Lemoine; Louise Lefrançois; Marion Barbet; Maud Vanpeene; Méline Bizard; Sylvie Behillil; Sylvie van der Werf; Vincent Enouf
EPI_ISL_420038, EPI_ISL_420045, EPI_ISL_420055, EPI_ISL_421514	Sentinelles network	National Reference Center for Viruses of Respiratory Infections, Institut Pasteur, Paris	Angela Brisebarre; Etienne Simon-Lorière; Flora Donati; Marion Barbet; Maud Vanpeene; Mélanie Albert; Méline Bizard; Sylvie Behillil; Sylvie van der Werf; Vincent Enouf
EPI_ISL_593934	Sentinelles, Chanteloup-En-Brie	National Reference Center for Viruses of Respiratory Infections, Institut Pasteur, Paris	Etienne Simon-Lorière; Fabiana Gambaro; Maud Vanpeene; Sylvie Behillil; Sylvie van der Werf; Vincent Enouf
EPI_ISL_593936	Sentinelles, Fondettes	National Reference Center for Viruses of Respiratory Infections, Institut Pasteur, Paris	Etienne Simon-Lorière; Fabiana Gambaro; Maud Vanpeene; Sylvie Behillil; Sylvie van der Werf; Vincent Enouf
EPI_ISL_593902	Sentinelles, Limay	National Reference Center for Viruses of Respiratory Infections, Institut Pasteur, Paris	Etienne Simon-Lorière; Fabiana Gambaro; Maud Vanpeene; Sylvie Behillil; Sylvie van der Werf; Vincent Enouf
EPI_ISL_593935	Sentinelles, Plessis-Trevise	National Reference Center for Viruses of Respiratory Infections, Institut Pasteur, Paris	Etienne Simon-Lorière; Fabiana Gambaro; Maud Vanpeene; Sylvie Behillil; Sylvie van der Werf; Vincent Enouf
EPI_ISL_408977	Serology, Virology and OTDS Laboratories (SAViD), NSW Health Pathology Randwick	NSW Health Pathology - Institute of Clinical Pathology and Medical Research; Centre for Infectious Diseases and Microbiology Laboratory Services; Westmead Hospital; University of Sydney	Carter I; Chen SC; Eden J-S; Holmes EC; Kok J and Dwyer DE for the 2019-nCoV Study Group*; Maddocks S; O'Sullivan MV; Rahman H; Rawlinson W; Rockett R; Sintchenko V
EPI_ISL_583502	Serv de Vig Sanitaria Epidemio e CTRL de Zoonoses Guaruja	Instituto Adolfo Lutz, Interdiciplinary Procedures Center, Strategic Laboratory	Claudia Regina Gonçalves; Claudio Tavares Sacchi; Erica Valessa Ramos Gomes; Karoline Rodrigues Campos
EPI_ISL_420048, EPI_ISL_420058 to 420060, EPI_ISL_420062, EPI_ISL_420064, EPI_ISL_421501, EPI_ISL_421504 to 421506, EPI_ISL_421512, EPI_ISL_428347, EPI_ISL_428349			
see above	Service de Biologie Médicale - BP 125	National Reference Center for Viruses of Respiratory Infections, Institut Pasteur, Paris	Angela Brisebarre; Christine Lambert; Etienne Simon-Lorière; Flora Donati; Marion Barbet; Maud Vanpeene; Mélanie Albert; Méline Bizard; Sylvie Behillil; Sylvie van der Werf; Vincent Enouf
EPI_ISL_420042, EPI_ISL_421513	Service de Biologie clinique	National Reference Center for Viruses of Respiratory Infections, Institut Pasteur, Paris	Angela Brisebarre; Christine Lambert; Etienne Simon-Lorière; Flora Donati; Marion Barbet; Maud Vanpeene; Mélanie Albert; Méline Bizard; Sylvie Behillil; Sylvie van der Werf; Vincent Enouf
EPI_ISL_469283 to 469284	Service de Virologie Hôpital Saint-Louis	Laboratory Cell Biology of Viral Infection-INSERM unit 944	Ali Amara; Constance Delaugerre; Laurent Meertens; Lucie Bonnet-Madin; Maud SALMONA; Séverine Mercier-Delarue
EPI_ISL_469282	Service de Virologie Hôpital Saint-Louis	Laboratory of Cell Biology of viral infection, Unit INSERM-U944	Ali Amara; Constance Delaugerre; Laurent Meertens; Lucie Bonnet-Madin
EPI_ISL_418232 to 418233	Service des Urgences	National Reference Center for Viruses of Respiratory Infections, Institut Pasteur, Paris	Angela Brisebarre; Boubkeur; Etienne Simon-Lorière; Flora Donati; Marion Barbet; Maud Vanpeene; Mélanie Albert; Méline Bizard; Sylvie Behillil; Sylvie van der Werf; Vincent Enouf
EPI_ISL_414598	Servicio Microbiologia, Hospital Clinico Universitario, Valencia	Sequencing and Bioinformatics Service and Molecular Epidemiology Research Group. FISABIO-Public Health.	David Navarro; Fernando Gonzalez-Candelas; Giuseppe D'Auria; Griselda De Marco; Maria Alma Bracho; Neris Garcia-Gonzalez
EPI_ISL_414496	Servicio Microbiologia. Hospital Clinico Universitario. Valencia.	Sequencing and Bioinformatics Service. Molecular Epidemiology Laboratory. FISABIO-Public Health	David Navarro; Fernando Gonzalez-Candelas; Giuseppe D'Auria; Griselda De Marco; María Alma Bracho; Neris Garcia-Gonzalez
EPI_ISL_414495	Servicio Microbiología. Hospital Clínico Universitario. Valencia.	Sequencing and Bioinformatics Service. Molecular Epidemiology Laboratory. FISABIO-Public Health	David Navarro; Fernando Gonzalez-Candelas; Giuseppe D'Auria; Griselda De Marco; Maria Alma Bracho; Neris Garcia-Gonzalez
EPI_ISL_420598 to 420600	Servicio Virosis Respiratorias-Departamento Virología-INEI	Instituto Nacional Enfermedades Infecciosas C.G.Malbran	Avaro M.; Baumeister E.; Benedetti E.; Campos J.; Cisterna D.; Dattero ME; Lorenzo F.; Molina V.; Perandones C.; Poklepovich T.; Pontoriero A.; Russo M.; Tuduri E.
EPI_ISL_416485, EPI_ISL_417206	Servicio de Microbiologia. Consorcio Hospital General Universitario de Valencia	Sequencing and Bioinformatics Service and Molecular Epidemiology Research Group. FISABIO-Public Health	Concepcion Gimeno; Fernando Gonzalez-Candelas; Giuseppe D'Auria; Griselda De Marco; Maria Alma Bracho; Maria Dolores Ocete; Neris Garcia-Gonzalez
EPI_ISL_537996 to 538002	Servicio de Microbiologia. Hospital Arnau de Vilanova	SeqCOVID-SPAIN consortium/IBV(CSIC)	Fernando Gonzalez-Candelas and SeqCOVID-SPAIN consortium; Giuseppe D'Auria; Griselda De Marco; Inma Galán Vendrell; Ivan Ansari; Lidia Ruiz Roldan; Llúcia Martínez-Priego; Loreto Ferrús Abad; Maria Alma Bracho; Mariana Reyes-Prieto; Neris Garcia-Gonzalez; Paula Ruiz-Hueso; Sandra Carbo; Vicente Soriano Chirona; Victoria Dominguez
EPI_ISL_539221 to 539233, EPI_ISL_539235 to 539239, EPI_ISL_539243 to 539244, EPI_ISL_539288	Servicio de Microbiologia. Hospital Clinico Universitario de Valencia	SeqCOVID-SPAIN consortium/IBV(CSIC)	Beatriz Beamud; Concepcion Gimeno; Fernando Gonzalez-Candelas and SeqCOVID-SPAIN consortium; Giuseppe D'Auria; Griselda De Marco; Inma Galán Vendrell; Lidia Ruiz Roldan; Llúcia Martínez-Priego; Loreto Ferrús Abad; Maria Alma Bracho; Maria Dolores Ocete; Mariana Reyes-Prieto; Marta Pla Diaz; Neris Garcia-Gonzalez; Paula Ruiz-Hueso; Vicente Soriano Chirona
EPI_ISL_420125 to 420132, EPI_ISL_421515 to 421520, EPI_ISL_425199 to 425211, EPI_ISL_436278 to 436360, EPI_ISL_436362 to 436371, EPI_ISL_436396 to 436411, EPI_ISL_447470 to 447531			
see above	Servicio de Microbiologia. Hospital Clinico Universitario de Valencia	Sequencing and Bioinformatics Service and Molecular Epidemiology Research Group. FISABIO-Public Health	Beatriz Beamud; Concepcion Gimeno; David Navarro; Eliseo Albert; Fernando Gonzalez-Candelas; Giuseppe 'Auria; Giuseppe D'Auria; Griselda De Marco; Inma Galán Vendrell; Ivan Ansari; Lidia Ruiz Roldan; Llúcia Martínez-Priego; Loreto Ferrús Abad; Maria Alma Bracho; Maria Dolores Ocete;

			Mariana Reyes-Prieto; Marta Pla Diaz; Neris Garcia-Gonzalez; Paula Ruiz-Hueso; Sandra Carbo; Vicente Soriano Chirona
EPI_ISL_455721 to 455734, EPI_ISL_455737 to 455744	Servicio de Microbiologia. Hospital Clinico Universitario de Valencia	Sequencing and Bioinformatics Service and Molecular Epidemiology Research Group. FISABIO-Public Health, and SeqCOVID-Spain Consortium	David Navarro; Eliseo Albert; Fernando Gonzalez-Candelas; Giuseppe 'Auria; Griselda De Marco; Inma Galán Vendrell; Ivan Ansari; Lidia Ruiz Roldan; Llúcia Martínez-Priego; Loreto Ferrús Abad; Maria Alma Bracho; Mariana Reyes-Prieto; Neris Garcia-Gonzalez; Paula Ruiz-Hueso; Sandra Carbo; Vicente Soriano Chirona
EPI_ISL_436235 to 436270, EPI_ISL_436372 to 436381	Servicio de Microbiologia. Hospital Universitario Doctor Peset	Sequencing and Bioinformatics Service and Molecular Epidemiology Research Group. FISABIO-Public Health	Beatriz Beamud; Fernando Gonzalez-Candelas; Giuseppe D'Auria; Griselda De Marco; Inma Galán Vendrell; Ivan Ansari; Jose Miguel Nogueira Coito; Juan Alberola Enguídanos; Juan Jose Camarena Miñana; Lidia Ruiz Roldan; Llúcia Martínez-Priego; Loreto Ferrús Abad; Maria Alma Bracho; Mariana Reyes-Prieto; Marta Pla Diaz; Neris Garcia-Gonzalez; Paula Ruiz-Hueso; Rosa González Pellicer; Sandra Carbo; Vicente Soriano Chirona
EPI_ISL_455735 to 455736	Servicio de Microbiologia. Hospital_Arnau_de_Vilanova	Sequencing and Bioinformatics Service and Molecular Epidemiology Research Group. FISABIO-Public Health, and SeqCOVID-Spain Consortium	Fernando Gonzalez-Candelas; Giuseppe 'Auria; Griselda De Marco; Inma Galán Vendrell; Ivan Ansari; Lidia Ruiz Roldan; Llúcia Martínez-Priego; Loreto Ferrús Abad; Maria Alma Bracho; Mariana Reyes-Prieto; Neris Garcia-Gonzalez; Paula Ruiz-Hueso; Sandra Carbo; Vicente Soriano Chirona; Victoria Dominguez
EPI_ISL_1392944 to 1393628	Servicio de Microbiología Clínica (Complejo Hospitalario de Navarra, Pamplona)	Centro de Secuenciación NASERTIC	Ana Miqueleiz; Ana Navascués; Carmen Ezpeleta Baquedano
EPI_ISL_1423633 to 1423680	Servicio de Microbiología Clínica (Complejo Hospitalario de Navarra, Pamplona), Instituto de Investigación Sanitaria de Navarra (IdiSNA)	SeqCOVID-SPAIN consortium/IBV(CSIC)	Ana Miqueleiz and SeqCOVID-SPAIN consortium; Ana Navascués; Carmen Ezpeleta Baquedano
EPI_ISL_538055 to 538061, EPI_ISL_538119 to 538230, EPI_ISL_538568 to 538607	Servicio de Microbiología y Parasitología clínica. UCEIMP. Hospital Universitario Virgen del Rocío/IBIS/CSIC/US	SeqCOVID-SPAIN consortium/IBV(CSIC)	Guillermo Martín Gutiérrez; Javier Aznar Martín and SeqCOVID-SPAIN consortium; Lidia Gálvez Benitez; Verónica González Galán; Ángel Rodríguez Villodres
EPI_ISL_452544 to 452616	Servicio de Microbiología y Parasitología clínica. UCEIMP. Hospital Universitario Virgen del Rocío/IBIS/CSIC/US.	SeqCOVID-SPAIN consortium/IBV(CSIC)	Guillermo Martí-n Gutiérrez; Javier Aznar Martí-n and SeqCOVID-SPAIN consortium; Lidia Gálvez Benitez; Verónica González Galán; Ángel Rodrí-guez Villodres
EPI_ISL_468765 to 468859, EPI_ISL_500287 to 500288, EPI_ISL_500290 to 500291, EPI_ISL_500336 to 500341, EPI_ISL_500343 to 500347, EPI_ISL_500349 to 500357, EPI_ISL_500359 to 500368, EPI_ISL_510333 to 510390, EPI_ISL_537729 to 537787			
see above	Servicio de Microbiología, Hospital Miguel Servet, Zaragoza	SeqCOVID-SPAIN consortium/IBV(CSIC)	Alexander Tristancho Baró; Ana Milagro; Antonio Rezusta López; Nieves Martínez Cameo and SeqCOVID-SPAIN consortium; Yolanda Gracia Grataloup
EPI_ISL_541008 to 541036	Servicio de Microbiología, Hospital Miguel Servet, Zaragoza	SeqCOVID-SPAIN consortium/Institute of Biomedicine of Valencia, IBV-CSIC	Alexander Tristancho Baró; Ana Milagro; Antonio Rezusta López; Nieves Martínez Cameo and SeqCOVID-SPAIN consortium; Yolanda Gracia Grataloup
EPI_ISL_582695 to 582766	Servicio de Microbiología, Hospital Universitario Cruces, Bilbao	SeqCOVID-SPAIN consortium/IBV(CSIC)	Maitane Aranzamendi Zaldumbide and SeqCOVID-SPAIN consortium
EPI_ISL_467055 to 467058, EPI_ISL_468952 to 469015, EPI_ISL_541949 to 541969	Servicio de Microbiología, Hospital Universitario Son Espases	SeqCOVID-SPAIN consortium/IBV(CSIC)	Antonio Oliver and SeqCOVID-SPAIN consortium; Carla López-Causapé; Jordi Reina; Jordi Reina y Antonio Oliver and SeqCOVID-SPAIN consortium
EPI_ISL_510510 to 510523	Servicio de Microbiología, Laboratori Clínic Metropolitana Nord. Hospital Universitari Germans Trias i Pujol. Institut d'Investigació en Ciències de la Salut Germans Trias i Pujol (IGTP)	SeqCOVID-SPAIN consortium/IBV(CSIC)	Adrián Antuori; Anabel Fernández; Anna Not; Antoni E. Bordoy; Elisa Martró; Nona Romaní and SeqCOVID-SPAIN consortium
EPI_ISL_537608, EPI_ISL_539234, EPI_ISL_539240 to 539242, EPI_ISL_539289 to 539298	Servicio de Microbiología. Consorcio Hospital General Universitario de Valencia	SeqCOVID-SPAIN consortium/IBV(CSIC)	Beatriz Beamud; David Navarro; Fernando Gonzalez-Candelas and SeqCOVID-SPAIN consortium; Giuseppe D'Auria; Griselda De Marco; Inma Galán Vendrell; Ivan Ansari; Lidia Ruiz Roldan; Llúcia Martínez-Priego; Loreto Ferrús Abad; Maria Alma Bracho; Mariana Reyes-Prieto; Marta Pla Diaz; Neris Garcia-Gonzalez; Paula Ruiz-Hueso; Sandra Carbo; Vicente Soriano Chirona
EPI_ISL_416483 to 416484, EPI_ISL_416486 to 416487, EPI_ISL_417205, EPI_ISL_419675 to 419690, EPI_ISL_420112 to 420124, EPI_ISL_425178 to 425198, EPI_ISL_425212 to 425223, EPI_ISL_436195 to 436234, EPI_ISL_436271 to 436277, EPI_ISL_436361, EPI_ISL_436382 to 436395			
see above	Servicio de Microbiología. Consorcio Hospital General Universitario de Valencia	Sequencing and Bioinformatics Service and Molecular Epidemiology Research Group. FISABIO-Public Health	; Beatriz Beamud; Concepcion Gimeno; David Navarro; Fernando Gonzalez-Candelas; Giuseppe D'Auria; Griselda De Marco; Inma Galán Vendrell; Ivan Ansari; Lidia Ruiz Roldan; Llúcia Martínez-Priego; Llúcia Martínez-PriegoGriselda De Marco; Loreto Ferrús Abad; Maria Alma Bracho; Maria Dolores Ocete; Mariana Reyes-Prieto; Marta Pla Diaz; Neris Garcia-Gonzalez; Paula Ruiz-Hueso; Sandra Carbo; Vicente Soriano Chirona
EPI_ISL_452365 to 452452, EPI_ISL_509602 to 509609, EPI_ISL_509611, EPI_ISL_509619 to 509632, EPI_ISL_510044 to 510063, EPI_ISL_565908 to 565919	Servicio de Microbiología. HRU de Málaga. Servicio Andaluz de Salud	SeqCOVID-SPAIN consortium/IBV(CSIC)	Begoña Palop Borrás and SeqCOVID-SPAIN consortium; Inmaculada de Toro Peinado; Inmaculada de Toro Peinado. MªConcepción Mediavilla Gradolph. Begoña Palop Borrás and SeqCOVID-SPAIN consortium; Ma Concepción Mediavilla Gradolph; Maria Concepción Mediavilla Gradolph
EPI_ISL_583917	Servicio de Microbiología. Hospital General Universitario de Castellón	SeqCOVID-SPAIN consortium/IBV (CSIC)	María Dolores Tirado and SeqCOVID-SPAIN consortium; Rosario Moreno
EPI_ISL_537946 to 537995, EPI_ISL_538613 to 538671, EPI_ISL_539284 to 539287, EPI_ISL_583918 to 583952	Servicio de Microbiología. Hospital General Universitario de Castellón	SeqCOVID-SPAIN consortium/IBV(CSIC)	María Dolores Tirado and SeqCOVID-SPAIN consortium; Rosario Moreno
EPI_ISL_1423737 to 1423761, EPI_ISL_1423793 to 1423815	Servicio de Microbiología. Hospital Ramón y Cajal. (CIBERESP)	SeqCOVID-SPAIN consortium/IBV(CSIC)	Jose Mª González-Alba; Juan C Galán and SeqCOVID-SPAIN consortium; L. Olavarrieta; Val Fernández
EPI_ISL_480952 to 480960, EPI_ISL_480962 to 480974, EPI_ISL_480976 to 480980, EPI_ISL_480982 to 480988, EPI_ISL_480990 to 480994, EPI_ISL_480996 to 481002, EPI_ISL_481004 to 481016, EPI_ISL_481018 to 481024, EPI_ISL_481026 to 481028, EPI_ISL_481030 to 481033, EPI_ISL_481036 to 481040, EPI_ISL_500292 to 500335, EPI_ISL_509601, EPI_ISL_509610, EPI_ISL_509612 to 509615, EPI_ISL_509633 to 509654, EPI_ISL_510391 to 510423, EPI_ISL_510467 to 510509, EPI_ISL_538062 to 538081, EPI_ISL_565920 to 565925, EPI_ISL_582034 to 582090, EPI_ISL_1423858 to 1423902			
see above	Servicio de Microbiología. Hospital Universitario Donostia. OSI Donostialdea. Área de Enfermedades Infecciosas, Grupo de Infección Respiratoria y Resistencia Antimicrobiana. Instituto de Investigación Sanitaria Biodonostia	SeqCOVID-SPAIN consortium/IBV(CSIC)	Ane Sorrarain; Gustavo Cilla; Gustavo Cilla Eguiluz; Jose Maria Marimón and SeqCOVID-SPAIN consortium; Luis Piñeiro; Luis Piñeiro Vázquez; Milagrosa Montes; Milagrosa Montes Ros
EPI_ISL_541083 to 541137	Servicio de Microbiología. Hospital Universitario Donostia. OSI Donostialdea. Área de Enfermedades Infecciosas, Grupo de Infección Respiratoria y Resistencia Antimicrobiana. Instituto de Investigación Sanitaria Biodonostia	SeqCOVID-SPAIN consortium/Institute of Biomedicine of Valencia, IBV-CSIC	Gustavo Cilla; Jose Maria Marimón and SeqCOVID-SPAIN consortium; Luis Piñeiro; Milagrosa Montes
EPI_ISL_452617 to 452691, EPI_ISL_468860 to 468913, EPI_ISL_468915 to 468951	Servicio de Microbiología. Hospital Universitario Donostia. OSI Donostialdea. Área de Enfermedades Infecciosas, Grupo de Infección Respiratoria y Resistencia Antimicrobiana. Instituto de Investigación Sanitaria Biodonostia.	SeqCOVID-SPAIN consortium/IBV(CSIC)	Gustavo Cilla; Jose Maria Marimón and SeqCOVID-SPAIN consortium; Luis Piñeiro; Milagrosa Montes
EPI_ISL_1468462	Servico Especial de Saude de Araraquara Sesa Araraquara	Instituto Adolfo Lutz, Interdiciplinary Procedures Center, Strategic Laboratory	Caio Vinicius Dias Lopes; Claudia Regina Gonçalves; Claudio Tavares Sacchi; Erica Valessa Ramos Gomes; Karoline Rodrigues Campos
EPI_ISL_574598	Servico de Verificacao de Obito SVO	Instituto Adolfo Lutz, Interdiciplinary Procedures Center, Strategic Laboratory	Claudia Regina Gonçalves; Claudio Tavares Sacchi; Erica Valessa Ramos Gomes; Karoline Rodrigues Campos
EPI_ISL_1533718	Servico de Verificacao de Obitos Svo Guarulhos	Instituto Adolfo Lutz, Interdiciplinary Procedures Center, Strategic Laboratory	Caio Vinicius Dias Lopes; Claudia Regina Gonçalves; Claudio Tavares Sacchi; Erica Valessa Ramos Gomes; Karoline Rodrigues Campos; Leonardo Jose Tadeu de Araujo
EPI_ISL_529010 to 529012	Servizio Igiene Epidemiologia e Sanità Pubblica (SIESP)-L'Aquila	Istitituto Zooprofilattico Sperimentale dell'Abruzzo e Molise "G.Caporale"	Ancora M; Cammà C; Curini V; Di Domenico M; Di Pasquale A; Lorusso A; Mangone I; Marcacci M; Puglia I; Rinaldi A; Savini G.
EPI_ISL_435152	Servizio di Igiene, Epidemiologia e Sanità Pubblica (SIESP) Avezzano	Istituto Zooprofilattico Sperimentale dell'Abruzzo e Molise "G.Caporale"	Ancora M; Cammà C; Curini V; Di Domenico M; Di Pasquale A; Lorusso A; Mangone I; Marcacci M; Puglia I; Rinaldi A; Savini G
EPI_ISL_529006, EPI_ISL_529008	Servizio di igiene e sanità pubblica (SIESP)-Teramo	Istitituto Zooprofilattico Sperimentale dell'Abruzzo e Molise	Ancora M; Cammà C; Curini V; Di Domenico M; Di Pasquale A; Lorusso A; Mangone I; Marcacci M; Puglia I; Rinaldi A; Savini G.

		"G.Caporale"	
EPI_ISL_528994 to 529005, EPI_ISL_529023 to 529025	Servizio di igiene epidemiologia e sanità pubblica (SIESP)-Chieti	Istituto Zooprofilattico Sperimentale dell'Abruzzo e Molise "G.Caporale"	Ancora M; Cammà C; Curini V; Di Domenico M; Di Pasquale A; Lorusso A; Mangone I; Marcacci M; Puglia I; Rinaldi A; Savini G.
EPI_ISL_436730	Servizio di igiene epidemiologia e sanità pubblica (Siesp) Chieti	Istituto Zooprofilattico Sperimentale dell'Abruzzo e Molise "G.Caporale"	Ancora M; Cammà C; Curini V; Di Domenico M; Di Pasquale A; Lorusso A; Mangone I; Marcacci M; Puglia I; Rinaldi A; Savini G
EPI_ISL_1543930	Servizo de Microbioloxía. Complexo Hospitalario de Santiago de Compostela	Servizo de Microbioloxía. Complexo Hospitalario de Santiago de Compostela21	Amparo Coira; Antonio Aguilera; Carlos García_Riestra; Daniel Navarro; Gema Barbeito; Iria Roca; José Llovo; Laura Millán; Manuela Hernández; María Luisa Pérez_del_Molino; Mercedes Treviño; Rocio Trastoy; Xosé Costa
EPI_ISL_534315, EPI_ISL_583495	Serviço de Verificação de Óbitos SVO Guaruulhos	Instituto Adolfo Lutz, Interdiciplinary Procedures Center, Strategic Laboratory	Claudia Regina Gonçalves; Claudio Tavares Sacchi; Erica Valessa Ramos Gomes; Karoline Rodrigues Campos
EPI_ISL_515543	Serviço de Vigilância Sanitária e Epidemiológica	Instituto Adolfo Lutz, Interdiciplinary Procedures Center, Strategic Laboratory	Claudia Regina Gonçalves; Claudio Tavares Sacchi; Erica Valessa Ramos Gomes
EPI_ISL_414934 to 414941	Shandong Provincial Center for Disease Control and Prevention	Beijing Institute of Microbiology and Epidemiology	Bo Pang; Can-Fang Lin; Cun-Bao Li; Dian-Ming Kang; Feng Gao; Guo-Lin Wang; Jie Lei; Li-Jun Duan; Lin Yao; Mai-Juan Ma; Ming-Xiao Yao; Sheng-Xiang Ji; Wen-Kui Sun; Xiang-Na Zhao; Xiao Wei; Xiao-Li Zhang; Xiao-Lin Jiang; Yang Hang; Zeng-Qiang Kou
EPI_ISL_416316 to 416409	Shanghai Public Health Clinical Center, Shanghai Medical College, Fudan University	National Research Center for Translational Medicine (Shanghai), Ruijin Hospital affiliated to Shanghai Jiao Tong University School of Medicine & Shanghai Public Health Clinical Center	Gang Lu; Hongzhou Lu; Saijuan Chen; Shengyue Wang; Xiaonan Zhang; Yun Ling; Yun Tan
EPI_ISL_497950	Shaoxing CDC	Zhejiang Provincial Center for Disease Control and Prevention	Haiyan Mao; Hao Yan; Junhang Pan; Wen Shi; Xiuyu Lou; Yanjun Zhang; Yi Sun; Yin Chen; Zhen Li
EPI_ISL_463889, EPI_ISL_463894 to 463902	Shaoxing Center for Disease Control and Prevention	Department of Pathology and Laboratory Medicine, University of California Los Angeles	Evann E. Hilt; Fan Li; Huan Wu; JiLing Wang; Jialiang Tang; Jinkun Chen; QinChao Zhang; Shangxin Yang; Yifang Wang; Zhuojing Jiang; Ziqin Li
EPI_ISL_1478854, EPI_ISL_1478856, EPI_ISL_1478886, EPI_ISL_1500235	Shapiro Center	Illinois Department of Public Health - Springfield Lab	Bryan Sim; Gordon McCall
EPI_ISL_1468829 to 1468831, EPI_ISL_1468833 to 1468836, EPI_ISL_1468838, EPI_ISL_1468840, EPI_ISL_1468845 to 1468846, EPI_ISL_1468848, EPI_ISL_1468850 to 1468853, EPI_ISL_1468855 to 1468873, EPI_ISL_1468889 to 1468905, EPI_ISL_1468910 to 1468913, EPI_ISL_1468917 to 1468921, EPI_ISL_1468952, EPI_ISL_1468954, EPI_ISL_1468957 to 1468958, EPI_ISL_1468960 to 1468978, EPI_ISL_1469061 to 1469087, EPI_ISL_1531777 to 1531806			
see above	Sharp HealthCare Laboratory	Andersen lab at Scripps Research	Art Mendoza; Cathy Woerle; Jacquelyn Berumen; Liam McGinnis; Omid Bakhtar; SEARCH Alliance San Diego with Aaron Harding
EPI_ISL_582125 to 582126	Sheikh Khalifa Medical City	Molecular Surveillance lab Sheikh Khalifa Medical City	Amirtharaj Francis; Hala Imambaccus; Hiba Saud; Sahar Almarzooqi; Sajeed Abdul; Stefan Weber
EPI_ISL_582608 to 582691	Sheikh Khalifa Medical City	Molecular/Surveillance lab Sheikh Khalifa Medical City	Amirtharaj Francis; Hala Imambaccus; Hiba Saud; Sahar Almarzooqi; Sajeed Abdul; Stefan Weber
EPI_ISL_406593 to 406595	Shenzhen Key Laboratory of Pathogen and Immunity, National Clinical Research Center for Infectious Disease, Shenzhen Third People's Hospital	Shenzhen Key Laboratory of Pathogen and Immunity, National Clinical Research Center for Infectious Disease, Shenzhen Third People's Hospital	Chenguang Shen; Haixia Zheng; Li Xing; Yang Yang; Yingxia Liu; Zhixiang Xu
EPI_ISL_406592	Shenzhen Third People's Hospital	Shenzhen Key Laboratory of Pathogen and Immunity, National Clinical Research Center for Infectious Disease,Shenzhen Third People's Hospital	Chenguang Shen; Haixia Zheng; Li Xing; Yang Yang; Yingxia Liu; Zhixiang Xu
EPI_ISL_1469987	Shimantik Pathology and Diagnostic Center	Child Health Research Foundation	CHRF Bangladesh Genomics Team; Md. Mobarok Karim; Md. Parvej Alam
EPI_ISL_1533610	Shiraz University	Shiraz University	Abozar Ghorbani; Ebrahim Eftekhar; Hakimeh Ahmadi; Hamed Gouklani
EPI_ISL_480074 to 480082	Shizuoka City Institute of Environmental Sciences and Public Health	Pathogen Genomics Center, National Institute of Infectious Diseases	Hajime Kamiya; Kenji Yagi; Kentaro Itokawa; Makoto Kuroda; Masanori Hashino; Motoi Suzuki; Rina Tanaka; Sou Okamura; Takaharu Maehata; Tsuyoshi Sekizuka; Yuji Kanazawa
EPI_ISL_437187	Siloam Hospitals	Institute of Tropical Disease, Universitas Airlangga	Aldise M Nastri; Gatot Soegiarto; Jezzy R Dewantari; Kazufumi Shimizu; Krisnoadi Rahardjo; Laksmi Wulandari; Maria I Lusida; Maria M Padmidewi; Mitsuhiro Nishimura; Resti Y Meliana; Retno A Setyoningrum; Rima R Prasetya; Soetjipto; Yasuko Mori; Yohko K Shimizu
EPI_ISL_470589 to 470597	Simile	Bioinformatics Laboratory / LNCC	Alexandra Gerber; Amilcar Tanuri; Ana Paula Guimarães; CADDE-group; Carolina Voloch; Ester Cerdeira Sabino; Filipe Romero; Ingra Morales Claro; Jaqueline Goes de Jesus; Laboratorio Hermes Pardini; Laboratorio Simile; Luiz Gonzaga Paula de Almeida; Mariane Talon; Nuno Rodrigues Faria; Renato Santana Aguiar e Ana Tereza Vasconcelos; Ronaldo da Silva Francisco Junior; Terezinha Marta Pereira; working group UFMG; working group UFRJ; Átila Duque Rossi
EPI_ISL_462085 to 462089, EPI_ISL_482672 to 482699, EPI_ISL_500539 to 500572, EPI_ISL_509372 to 509395, EPI_ISL_517996 to 518017, EPI_ISL_524445 to 524449, EPI_ISL_583893 to 583900, EPI_ISL_610152 to 610154			
see above	Singapore General Hospital	Department of Microbiology	Chenhao Li; Chenhao Li; Karrie Ko; Karrie Ko; Kenneth Xin Long Chan; Kern Rei Chng; Kern Rei Chng; Kian Sing Chan; Kian Sing Chan; Kun Lee Lim; Kun Lee Lim; Lynette Oon; Lynette Oon; Niranjan Nagarajan; Niranjan Nagarajan; Nurdyana Abdul Rahman; Nurdyana Abdul Rahman; Sui Sin Goh
EPI_ISL_406973	Singapore General Hospital	National Public Health Laboratory	Chavatte JM; Cui; L; Lin; Mak; Octavia S; RTP; TM; ZY; Zhou
EPI_ISL_407987	Singapore General Hospital	Programme in Emerging Infectious Diseases, Duke-NUS Medical School	Danielle E Anderson; Gavin JD Smith; Jayanthi Jayakumar; Jenny GH Low; Kian Sing Chan; Linfa Wang; Lynette LE Oon; Martin Linster; Yan Zhuang; Yvonne CF Su
EPI_ISL_410536 to 410537	Singapore General Hospital, Molecular Laboratory, Division of Pathology	Programme in Emerging Infectious Diseases, Duke-NUS Medical School	Danielle E Anderson; Gavin JD Smith; Jayanthi Jayakumar; Jenny GH Low; Kian Sing Chan; Lynette LE Oon; Martin Linster; Shirin Kalimuddin; Yan Zhuang; Yvonne CF Su
EPI_ISL_569611	Sioux Falls Urgent Care	South Dakota Public Health Laboratory	Jacob Garfin; Matt Plumb; Xiong Wang; and Chris Carlson
EPI_ISL_426414	Sir M P Shah Government Medical College	Gujarat Biotechnology Research Centre	Akanksha Verma; Amit Kanani; Ankit Hinsu; Apurvasinh Puvar; Bhavesh Modi; Binita Aring; Chaitanya Joshi; Dinesh Kumar; Dipa Kinariwala; Disha Patel; Geeta Vaghela; Janvi Raval; Kairavi Joshi; Madhvi Joshi; Maharshi Pandya; Monika Gandhi; Nidhi Sood; Nitin Savaliya; Pinal Trivedi; Pranay Shah; Pritesh Sabara; R D Dixit; Raghawendra Kumar; Ramesh Pandit; Snehal Bagatharia; Sonia Barve; Tejas Shah; Zubair Saiyed
EPI_ISL_524746 to 524751	Sir M P Shah Government Medical College, Jamnagar	Gujarat Biotechnology Research Centre	A M Kadri; Afzal Ansari; Apurvasinh Puvar; Binita Aring; Chaitanya Joshi; Dinesh Kumar; Harsh Bakshi; Janvi Raval; Komal Patel; Labdhi Pandya; Madhvi Joshi; Maharshi Pandya; Monika Gandhi; Nidhi Patel; Nikha Trivedi; Nitin Savaliya; Pinal Trivedi; R D Dixit; Raghawendra Kumar; Zarna Patel; Zuber Saiyed
EPI_ISL_426415	Sir M P Shah Government Medical College, Jamnagar	Gujarat Biotechnology Research Centre, Gandhinagar	Akanksha Verma; Amit Kanani; Ankit Hinsu; Apurvasinh Puvar; Bhavesh Modi; Binita Aring; Chaitanya Joshi; Dinesh Kumar; Dipa Kinariwala; Disha Patel; Geeta Vaghela; Janvi Raval; Kairavi Joshi; Madhvi Joshi; Maharshi Pandya; Monika Gandhi; Nidhi Sood; Nitin Savaliya; Pinal Trivedi; Pranay Shah; Pritesh Sabara; R D Dixit; Raghvendra Kumar; Ramesh Pandit; Snehal Bagatharia; Sonia Barve; Tejas Shah; Zuber Saiyed
EPI_ISL_455586	Siriraj hospital	National Institute of Health. Department of medical Sciences, Ministry of Public Health, Thailand	Chittaganpitch; Malinee; Okada; Parnmen; Phuygun; Pilailuk; Siripaporn; Sittiporn; Sunthareeya; Thanadachakul; Thanutsapa; Waicharoen; Warawan; Wongboot
EPI_ISL_534225 to 534229, EPI_ISL_560980	Skanes universitetssjukhus Lund	The Public Health Agency of Sweden	Anna Risberg; Anna-Malin Linde; Karin Tegmark-Wisell; Maria Lind Karlberg; Mattias Haukland; Mia Brytting; Olov Svartstrom; Oskar Karlsson Lindsjo; Petra Edquist; Reza Advani; Sandra Broddesson
EPI_ISL_454494 to 454496, EPI_ISL_455842 to 455845, EPI_ISL_475085, EPI_ISL_475092, EPI_ISL_475099, EPI_ISL_475105 to 475109, EPI_ISL_475111 to 475113, EPI_ISL_475140, EPI_ISL_475549 to 475550, EPI_ISL_475554 to 475555, EPI_ISL_476143 to 476144, EPI_ISL_510845 to 510851, EPI_ISL_548245 to 548246, EPI_ISL_615115 to 615116			
see above	Skovde/Unilabs	The Public Health Agency of Sweden	Anna Risberg; Anna-Malin Linde; Karin Tegmark-Wisell; Maria Lind Karlberg; Mattias Haukland; Mia Brytting; Olov Svartstrom; Oskar Karlsson Lindsjo; Petra Edquist; Reza Advani; Sandra Broddesson; Shamam Muradrasoli; Shaman Muradrasoli
EPI_ISL_455108	Smedby HC	The Public Health Agency of Sweden	Anna Risberg; Anna-Malin Linde; Karin Tegmark-Wisell; Maria Lind Karlberg; Mia Brytting; Olov Svartstrom; Oskar Karlsson Lindsjo; Susanne Brunby;

			Theresa Enkirch
EPI_ISL_1525252 to 1525253, EPI_ISL_1525255 to 1525258	Somedi laboratories	Imelda Hospital	Dagmar Obbels; Hanne Valgaeren; Johan Frans
EPI_ISL_1432928, EPI_ISL_1432941, EPI_ISL_1432956, EPI_ISL_1432972, EPI_ISL_1433010, EPI_ISL_1433027, EPI_ISL_1433057, EPI_ISL_1433070, EPI_ISL_1433082, EPI_ISL_1433104, EPI_ISL_1433109, EPI_ISL_1433126, EPI_ISL_1433164 to 1433165, EPI_ISL_1433169, EPI_ISL_1433171, EPI_ISL_1433187 to 1433188, EPI_ISL_1433190, EPI_ISL_1433201, EPI_ISL_1433222 to 1433223, EPI_ISL_1433225 to 1433227, EPI_ISL_1433237, EPI_ISL_1433243, EPI_ISL_1433246, EPI_ISL_1433263 to 1433264, EPI_ISL_1433269 to 1433270, EPI_ISL_1433290, EPI_ISL_1433294, EPI_ISL_1433296, EPI_ISL_1433313, EPI_ISL_1433347, EPI_ISL_1433354, EPI_ISL_1438701, EPI_ISL_1438738, EPI_ISL_1438771 to 1438772, EPI_ISL_1438861, EPI_ISL_1438870, EPI_ISL_1438887, EPI_ISL_1438900, EPI_ISL_1438904, EPI_ISL_1438986, EPI_ISL_1439019, EPI_ISL_1439025, EPI_ISL_1439127, EPI_ISL_1439135, EPI_ISL_1439141, EPI_ISL_1439218			
see above	Sonic - Bioscientia - MVZ Labor Saar GmbH	Robert Koch Institute	
EPI_ISL_1436016, EPI_ISL_1436053, EPI_ISL_1436092, EPI_ISL_1436108, EPI_ISL_1436110, EPI_ISL_1436117, EPI_ISL_1436119, EPI_ISL_1436127, EPI_ISL_1436140, EPI_ISL_1436142, EPI_ISL_1436155, EPI_ISL_1436165, EPI_ISL_1436211 to 1436212, EPI_ISL_1436226, EPI_ISL_1436300, EPI_ISL_1436377, EPI_ISL_1439065, EPI_ISL_1439070, EPI_ISL_1439098, EPI_ISL_1439114, EPI_ISL_1439177, EPI_ISL_1439202, EPI_ISL_1439209, EPI_ISL_1439220, EPI_ISL_1439233, EPI_ISL_1439272, EPI_ISL_1439282, EPI_ISL_1439342, EPI_ISL_1439357			
see above	Sonic - Dr. Staber & Kollegen GmbH München	Robert Koch Institute	
EPI_ISL_1436019, EPI_ISL_1436023, EPI_ISL_1436027, EPI_ISL_1436042, EPI_ISL_1436044, EPI_ISL_1436055, EPI_ISL_1436059, EPI_ISL_1436065, EPI_ISL_1436069, EPI_ISL_1436091, EPI_ISL_1436115, EPI_ISL_1436118, EPI_ISL_1436125, EPI_ISL_1436138, EPI_ISL_1436152, EPI_ISL_1436166, EPI_ISL_1436185, EPI_ISL_1436188, EPI_ISL_1436197, EPI_ISL_1436202, EPI_ISL_1436204, EPI_ISL_1436217, EPI_ISL_1436229, EPI_ISL_1436240, EPI_ISL_1436251 to 1436252, EPI_ISL_1436266 to 1436267, EPI_ISL_1436291, EPI_ISL_1436308 to 1436309, EPI_ISL_1436321, EPI_ISL_1436325, EPI_ISL_1436335, EPI_ISL_1436339, EPI_ISL_1436346, EPI_ISL_1436350, EPI_ISL_1436352, EPI_ISL_1436364, EPI_ISL_1436368, EPI_ISL_1436372, EPI_ISL_1436376, EPI_ISL_1436378, EPI_ISL_1436381, EPI_ISL_1436383, EPI_ISL_1436387, EPI_ISL_1436392, EPI_ISL_1438689 to 1438690, EPI_ISL_1438707 to 1438708, EPI_ISL_1438725, EPI_ISL_1438728, EPI_ISL_1438763, EPI_ISL_1438766, EPI_ISL_1438779, EPI_ISL_1438784 to 1438785, EPI_ISL_1438789, EPI_ISL_1438795, EPI_ISL_1438805, EPI_ISL_1438819, EPI_ISL_1438823 to 1438824, EPI_ISL_1438826, EPI_ISL_1438831, EPI_ISL_1438834, EPI_ISL_1438846, EPI_ISL_1438851, EPI_ISL_1438862, EPI_ISL_1438872, EPI_ISL_1438884, EPI_ISL_1438901, EPI_ISL_1438907 to 1438908, EPI_ISL_1438912, EPI_ISL_1438926, EPI_ISL_1438928, EPI_ISL_1438934 to 1438935, EPI_ISL_1438959, EPI_ISL_1438976, EPI_ISL_1439001 to 1439003, EPI_ISL_1439005, EPI_ISL_1439008, EPI_ISL_1439010, EPI_ISL_1439024, EPI_ISL_1439030			
see above	Sonic - Labor Dr. von Froreich GmbH	Robert Koch Institute	
EPI_ISL_1436062, EPI_ISL_1436071, EPI_ISL_1436098, EPI_ISL_1436167, EPI_ISL_1436268, EPI_ISL_1436270, EPI_ISL_1436286, EPI_ISL_1436292, EPI_ISL_1436322, EPI_ISL_1436324, EPI_ISL_1436332, EPI_ISL_1436337, EPI_ISL_1436389, EPI_ISL_1439043, EPI_ISL_1439077, EPI_ISL_1439172, EPI_ISL_1439197, EPI_ISL_1439201, EPI_ISL_1439224, EPI_ISL_1439246, EPI_ISL_1439252, EPI_ISL_1439285, EPI_ISL_1439302, EPI_ISL_1439321, EPI_ISL_1439361			
see above	Sonic - Labor Staber Bayreuth	Robert Koch Institute	
EPI_ISL_1436012, EPI_ISL_1436025, EPI_ISL_1436093, EPI_ISL_1436121, EPI_ISL_1436137, EPI_ISL_1436143, EPI_ISL_1436147, EPI_ISL_1436160, EPI_ISL_1436164, EPI_ISL_1436179, EPI_ISL_1436242 to 1436243, EPI_ISL_1436254, EPI_ISL_1436263, EPI_ISL_1436329, EPI_ISL_1436384, EPI_ISL_1436391, EPI_ISL_1439048, EPI_ISL_1439078, EPI_ISL_1439101, EPI_ISL_1439124, EPI_ISL_1439136, EPI_ISL_1439147, EPI_ISL_1439213, EPI_ISL_1439241, EPI_ISL_1439245, EPI_ISL_1439291, EPI_ISL_1439347			
see above	Sonic - Labor Staber Dresden (Klipphausen)	Robert Koch Institute	
EPI_ISL_1436114, EPI_ISL_1436182, EPI_ISL_1436189, EPI_ISL_1436208, EPI_ISL_1436271, EPI_ISL_1439125, EPI_ISL_1439193, EPI_ISL_1439320			
see above	Sonic - Labor Staber Heilbronn	Robert Koch Institute	
EPI_ISL_1433197, EPI_ISL_1433277, EPI_ISL_1438909, EPI_ISL_1438969	Sonic - Labor Staber MVZ Kassel	Robert Koch Institute	
EPI_ISL_1436007, EPI_ISL_1436060 to 1436061, EPI_ISL_1436126, EPI_ISL_1436156, EPI_ISL_1436175, EPI_ISL_1436178, EPI_ISL_1436186, EPI_ISL_1436228, EPI_ISL_1436280, EPI_ISL_1439046, EPI_ISL_1439059, EPI_ISL_1439080, EPI_ISL_1439095, EPI_ISL_1439228, EPI_ISL_1439281, EPI_ISL_1439353			
see above	Sonic - Labor Staber Nürnberg	Robert Koch Institute	
EPI_ISL_1436014, EPI_ISL_1436036, EPI_ISL_1436057, EPI_ISL_1436089, EPI_ISL_1436097, EPI_ISL_1436100, EPI_ISL_1436103, EPI_ISL_1436107, EPI_ISL_1436109, EPI_ISL_1436129 to 1436130, EPI_ISL_1436174, EPI_ISL_1436180 to 1436181, EPI_ISL_1436193, EPI_ISL_1436203, EPI_ISL_1436232, EPI_ISL_1436249, EPI_ISL_1436257, EPI_ISL_1436269, EPI_ISL_1436272, EPI_ISL_1436296, EPI_ISL_1436298, EPI_ISL_1436301, EPI_ISL_1436303 to 1436304, EPI_ISL_1436315, EPI_ISL_1436344, EPI_ISL_1436370 to 1436371, EPI_ISL_1436382, EPI_ISL_1436394, EPI_ISL_1438688, EPI_ISL_1438694, EPI_ISL_1438706, EPI_ISL_1438714, EPI_ISL_1438719, EPI_ISL_1438727, EPI_ISL_1438736 to 1438737, EPI_ISL_1438745, EPI_ISL_1438760, EPI_ISL_1438783, EPI_ISL_1438787, EPI_ISL_1438808 to 1438809, EPI_ISL_1438812 to 1438814, EPI_ISL_1438817, EPI_ISL_1438821, EPI_ISL_1438829, EPI_ISL_1438835 to 1438836, EPI_ISL_1438838, EPI_ISL_1438842, EPI_ISL_1438847, EPI_ISL_1438855, EPI_ISL_1438871, EPI_ISL_1438874, EPI_ISL_1438879, EPI_ISL_1438889, EPI_ISL_1438894, EPI_ISL_1438897, EPI_ISL_1438910, EPI_ISL_1438914, EPI_ISL_1438918 to 1438919, EPI_ISL_1438924, EPI_ISL_1438972 to 1438973, EPI_ISL_1438991, EPI_ISL_1438997 to 1438998, EPI_ISL_1439016, EPI_ISL_1439020 to 1439021, EPI_ISL_1439028			
see above	Sonic - MVZ Medizinisches Labor Bremen GmbH	Robert Koch Institute	
EPI_ISL_1436230, EPI_ISL_1438692, EPI_ISL_1438713, EPI_ISL_1439027, EPI_ISL_1439029	Sonic - MVZ Medizinisches Labor Oldenburg	Robert Koch Institute	
EPI_ISL_1392001 to 1392012, EPI_ISL_1392014 to 1392018, EPI_ISL_1392020, EPI_ISL_1392022 to 1392030, EPI_ISL_1392032 to 1392035, EPI_ISL_1392037 to 1392054, EPI_ISL_1392056 to 1392079, EPI_ISL_1392081 to 1392103, EPI_ISL_1392105 to 1392123, EPI_ISL_1392125 to 1392139, EPI_ISL_1392141 to 1392155, EPI_ISL_1392157 to 1392163, EPI_ISL_1392165 to 1392172, EPI_ISL_1392174 to 1392176, EPI_ISL_1392178 to 1392182, EPI_ISL_1392184 to 1392198, EPI_ISL_1392200 to 1392236, EPI_ISL_1392238, EPI_ISL_1392241, EPI_ISL_1392243, EPI_ISL_1392245, EPI_ISL_1392247, EPI_ISL_1392249, EPI_ISL_1392251, EPI_ISL_1392253, EPI_ISL_1392256 to 1392257, EPI_ISL_1392259, EPI_ISL_1392261, EPI_ISL_1392263, EPI_ISL_1392265, EPI_ISL_1392267, EPI_ISL_1392269, EPI_ISL_1392271, EPI_ISL_1392273, EPI_ISL_1392275, EPI_ISL_1392279, EPI_ISL_1392281, EPI_ISL_1392283, EPI_ISL_1392285, EPI_ISL_1392287, EPI_ISL_1392289, EPI_ISL_1392291, EPI_ISL_1392293, EPI_ISL_1392295, EPI_ISL_1392297, EPI_ISL_1392299, EPI_ISL_1392301, EPI_ISL_1392303, EPI_ISL_1392306, EPI_ISL_1392308, EPI_ISL_1392310, EPI_ISL_1392312, EPI_ISL_1392314, EPI_ISL_1392318, EPI_ISL_1392320, EPI_ISL_1392322, EPI_ISL_1392324, EPI_ISL_1392326, EPI_ISL_1392328, EPI_ISL_1392330, EPI_ISL_1392332, EPI_ISL_1392334, EPI_ISL_1392337, EPI_ISL_1392339, EPI_ISL_1392341 to 1392342, EPI_ISL_1392344, EPI_ISL_1392346, EPI_ISL_1392348, EPI_ISL_1392350, EPI_ISL_1392352, EPI_ISL_1392354, EPI_ISL_1392356, EPI_ISL_1392358, EPI_ISL_1392360, EPI_ISL_1392362, EPI_ISL_1392364, EPI_ISL_1392366, EPI_ISL_1392369, EPI_ISL_1392371, EPI_ISL_1392373, EPI_ISL_1392375, EPI_ISL_1392377, EPI_ISL_1392379, EPI_ISL_1392381, EPI_ISL_1392383, EPI_ISL_1392385, EPI_ISL_1392387, EPI_ISL_1392389, EPI_ISL_1392391, EPI_ISL_1392393, EPI_ISL_1392395, EPI_ISL_1392397, EPI_ISL_1392399, EPI_ISL_1392401, EPI_ISL_1392403, EPI_ISL_1392405, EPI_ISL_1392407, EPI_ISL_1392410 to 1392411, EPI_ISL_1392413, EPI_ISL_1392415, EPI_ISL_1392418 to 1392419, EPI_ISL_1392421, EPI_ISL_1392423, EPI_ISL_1392425, EPI_ISL_1392427, EPI_ISL_1392429, EPI_ISL_1392432 to 1392433, EPI_ISL_1392435, EPI_ISL_1392437 to 1392446, EPI_ISL_1392448 to 1392463, EPI_ISL_1392465 to 1392498, EPI_ISL_1392500 to 1392517, EPI_ISL_1392519 to 1392533, EPI_ISL_1392535 to 1392540, EPI_ISL_1392542 to 1392572, EPI_ISL_1392574 to 1392632, EPI_ISL_1392634 to 1392639, EPI_ISL_1464678 to 1464683, EPI_ISL_1464685 to 1464689, EPI_ISL_1464692, EPI_ISL_1464694, EPI_ISL_1464696 to 1464718, EPI_ISL_1464720 to 1464744, EPI_ISL_1464746 to 1464787, EPI_ISL_1464789 to 1464791, EPI_ISL_1464793 to 1464813, EPI_ISL_1464815 to 1464849, EPI_ISL_1464851 to 1464862, EPI_ISL_1464864 to 1464867, EPI_ISL_1464869 to 1464873, EPI_ISL_1464875 to 1464903, EPI_ISL_1464905 to 1464939, EPI_ISL_1464941 to 1464949, EPI_ISL_1464951 to 1464968, EPI_ISL_1464970 to 1465055, EPI_ISL_1465057 to 1465066, EPI_ISL_1465068 to 1465122, EPI_ISL_1465124 to 1465127, EPI_ISL_1465129 to 1465156, EPI_ISL_1465158 to 1465166, EPI_ISL_1465168, EPI_ISL_1465170 to 1465184, EPI_ISL_1465187, EPI_ISL_1465190, EPI_ISL_1465193, EPI_ISL_1465197, EPI_ISL_1465200, EPI_ISL_1465204, EPI_ISL_1465207, EPI_ISL_1465211, EPI_ISL_1465214, EPI_ISL_1465218, EPI_ISL_1465223, EPI_ISL_1465227, EPI_ISL_1465230, EPI_ISL_1465233, EPI_ISL_1465237, EPI_ISL_1465240, EPI_ISL_1465244, EPI_ISL_1465247, EPI_ISL_1465251, EPI_ISL_1465256, EPI_ISL_1465260, EPI_ISL_1465263, EPI_ISL_1465266, EPI_ISL_1465269, EPI_ISL_1465272, EPI_ISL_1465276, EPI_ISL_1465279 to 1465323, EPI_ISL_1465325 to 1465342, EPI_ISL_1465344 to 1465359, EPI_ISL_1465361, EPI_ISL_1465363 to 1465416, EPI_ISL_1465418 to 1465422, EPI_ISL_1465424 to 1465437, EPI_ISL_1500640 to 1500641, EPI_ISL_1500643, EPI_ISL_1500646, EPI_ISL_1500648 to 1500665, EPI_ISL_1500668 to 1500670, EPI_ISL_1500672 to 1500675, EPI_ISL_1500677, EPI_ISL_1500682 to 1500685, EPI_ISL_1500687 to 1500689, EPI_ISL_1500691 to 1500694, EPI_ISL_1500696 to 1500697, EPI_ISL_1500699 to 1500702, EPI_ISL_1500704, EPI_ISL_1500706, EPI_ISL_1500708 to 1500710, EPI_ISL_1500712 to 1500713, EPI_ISL_1500715 to 1500716, EPI_ISL_1500718 to 1500721, EPI_ISL_1500723 to 1500727, EPI_ISL_1500729 to 1500731, EPI_ISL_1500733, EPI_ISL_1500735 to 1500751, EPI_ISL_1500755 to 1500766, EPI_ISL_1500768 to 1500771, EPI_ISL_1500774 to 1500776, EPI_ISL_1500779, EPI_ISL_1500781 to 1500785, EPI_ISL_1500788, EPI_ISL_1500790 to 1500792, EPI_ISL_1500794 to 1500796, EPI_ISL_1500798 to 1500800, EPI_ISL_1500803 to 1500804, EPI_ISL_1500806 to 1500811, EPI_ISL_1500814 to 1500822, EPI_ISL_1500824 to 1500826, EPI_ISL_1500828 to 1500833, EPI_ISL_1500835 to 1500837, EPI_ISL_1500839 to 1500851, EPI_ISL_1500853 to 1500865, EPI_ISL_1500867 to 1500877, EPI_ISL_1500879 to 1500890, EPI_ISL_1500892 to 1500895, EPI_ISL_1500897 to 1500904, EPI_ISL_1500907 to 1500909, EPI_ISL_1500911, EPI_ISL_1500913 to 1500914, EPI_ISL_1500916 to 1500917, EPI_ISL_1500919 to 1500920, EPI_ISL_1500922 to 1500932, EPI_ISL_1500934 to 1500937, EPI_ISL_1500940 to 1500944, EPI_ISL_1500946 to 1500947, EPI_ISL_1525089, EPI_ISL_1530555 to 1530562, EPI_ISL_1530564 to 1530565, EPI_ISL_1530568 to 1530569, EPI_ISL_1530571 to 1530573, EPI_ISL_1530575, EPI_ISL_1530577 to 1530578, EPI_ISL_1530580 to 1530589, EPI_ISL_1530591 to 1530594, EPI_ISL_1530596, EPI_ISL_1530598, EPI_ISL_1530603, EPI_ISL_1530605 to 1530606, EPI_ISL_1530608 to 1530612, EPI_ISL_1530614 to 1530618, EPI_ISL_1530620 to 1530623, EPI_ISL_1530625 to 1530627, EPI_ISL_1530629, EPI_ISL_1530631 to 1530633, EPI_ISL_1530635 to 1530637, EPI_ISL_1530639, EPI_ISL_1530641, EPI_ISL_1530643 to 1530646, EPI_ISL_1530648 to 1530656, EPI_ISL_1530658 to 1530661, EPI_ISL_1530664 to 1530665, EPI_ISL_1530667, EPI_ISL_1530669 to 1530670, EPI_ISL_1530672 to 1530675, EPI_ISL_1530677 to 1530681, EPI_ISL_1530683 to 1530685, EPI_ISL_1530687, EPI_ISL_1530690, EPI_ISL_1530692 to 1530696, EPI_ISL_1530698, EPI_ISL_1530700 to 1530703, EPI_ISL_1530705 to 1530707, EPI_ISL_1530709 to 1530715, EPI_ISL_1530717 to 1530718, EPI_ISL_1530720, EPI_ISL_1530722, EPI_ISL_1530725 to 1530727, EPI_ISL_1530731, EPI_ISL_1530733, EPI_ISL_1530735, EPI_ISL_1530737 to 1530740, EPI_ISL_1530742, EPI_ISL_1530745 to 1530753, EPI_ISL_1530756 to 1530759, EPI_ISL_1530761 to 1530762, EPI_ISL_1530764 to 1530767, EPI_ISL_1530769 to 1530782, EPI_ISL_1530784, EPI_ISL_1530786 to 1530787, EPI_ISL_1530789 to 1530790, EPI_ISL_1530793, EPI_ISL_1530795 to 1530801, EPI_ISL_1530803 to 1530804, EPI_ISL_1530811 to 1530812, EPI_ISL_1530814 to 1530820, EPI_ISL_1530822, EPI_ISL_1530826 to 1530830, EPI_ISL_1530832, EPI_ISL_1530834 to 1530845, EPI_ISL_1530847 to 1530852, EPI_ISL_1530854, EPI_ISL_1530856 to 1530857, EPI_ISL_1530859 to 1530864, EPI_ISL_1530868, EPI_ISL_1530873 to 1530874			
see above	Sonora Quest Laboratories	TGen North	"Jolene Bowers; Ashlyn Pfeiffer; Chris French; Darrin Lemmer; Dave Engelthaler; Hayley Yaglom; Heather Centner; Jolene Bowers; The Arizona COVID Genomics Union (ACGU); The Arizona COVID Genomics Union (ACGU)"
EPI_ISL_408431	Sorbonne Université, Inserm et Assistance Publique-Hôpitaux de Paris (Pitié Salpétrière)	National Reference Center for Viruses of Respiratory Infections, Institut Pasteur, Paris	Angela Brisebarre; Anne-Geneviève Marcelin; David Boutolleau; Elise Klément; Eric Caumes.; Flora Donati; Marion Barbet; Maud Vanpeene; Mélanie Albert; Méline Bizard; Sonia Burrel; Sylvie Behillil; Sylvie van der Werf; Valérie Pourcher; Vincent Calvez; Vincent Enouf
EPI_ISL_529171 to 529178, EPI_ISL_529180 to 529199	South Carolina Department of Health and Environmental Control	South Carolina Department of Health and Environmental Control	Haley V. Flores
EPI_ISL_1401070 to 1401098	South Dakota Public Health Laboratory	South Dakota Public Health Laboratory	Jacob Garfin and Chris Carlson
EPI_ISL_451517 to 451519, EPI_ISL_451521, EPI_ISL_451632 to 451636, EPI_ISL_455036, EPI_ISL_455054, EPI_ISL_455075 to 455093, EPI_ISL_455096 to 455099			
see above	South Eastern Area Laboratory Services	NSW Health Pathology - Institute of Clinical Pathology and Medical Research; Westmead Hospital; University of Sydney	CIDM-PH et al.
EPI_ISL_478683 to 478705, EPI_ISL_478708 to 478711, EPI_ISL_490017 to 490019, EPI_ISL_490021 to 490035, EPI_ISL_490040 to 490042, EPI_ISL_490045, EPI_ISL_490105, EPI_ISL_498751, EPI_ISL_498768, EPI_ISL_513315 to 513317, EPI_ISL_513352, EPI_ISL_513377, EPI_ISL_526142, EPI_ISL_526144 to 526149, EPI_ISL_526191, EPI_ISL_526697, EPI_ISL_544970, EPI_ISL_544983 to 544984, EPI_ISL_545009 to 545016, EPI_ISL_545031, EPI_ISL_545048, EPI_ISL_547601, EPI_ISL_591502 to 591504, EPI_ISL_593683 to 593754, EPI_ISL_593771, EPI_ISL_1447288 to 1447291			

see above	South Eastern Area Laboratory Services (SEALS)	NSW Health Pathology - Institute of Clinical Pathology and Medical Research; Westmead Hospital; University of Sydney	CIDM-PH et al.
EPI_ISL_1443287	South Six Express Care Respiratory Clinic	Illinois Department of Public Health - Springfield Lab	Bryan Sim; Gordon McCall
EPI_ISL_1478847, EPI_ISL_1500239 to 1500240	South Sixth Express Care Respiratory Clinic	Illinois Department of Public Health - Springfield Lab	Bryan Sim; Gordon McCall
EPI_ISL_456158 to 456159, EPI_ISL_456162, EPI_ISL_456167, EPI_ISL_456181, EPI_ISL_456216 to 456220, EPI_ISL_456225 to 456240, EPI_ISL_456242 to 456254, EPI_ISL_456261 to 456293, EPI_ISL_456295 to 456299, EPI_ISL_456302 to 456318, EPI_ISL_456344 to 456347, EPI_ISL_536788 to 536789, EPI_ISL_579124 to 579128, EPI_ISL_579133 to 579155, EPI_ISL_579157 to 579189, EPI_ISL_579219			
see above	Southern Community Labs Dunedin	Institute of Environmental Science and Research (ESR)	Anja Werno; Antje van der Linden; Arlo Upton; Chris Mansell; David Hammer; Dragana Drinkovic; Erasmus Smit; Gary McAuliffe; Hana Sofia Andersson; Hermes Perez; James Ussher; Jill Sherwood; Jing Wang; Joep de Ligt; Josh Freeman; Julia Howard; Juliet Elvy; Lauren Jelly; Mary DeAlmeida; Matt Blakiston; Matt Storey; Matthew Rogers; Max Bloomfield; Michael Addidle; Michelle Balm; Muhammad Faisal; Nikki Freed; Olin Silander; Sally Roberts; Sarah Jefferies; Sharmini Muttaiyah; Susan Morpeth; Susan Taylor; Timothy Blackmore; Vani Sathyendran; Veronica Playle; Virginia Hope; Xiaoyun Ren
EPI_ISL_469241 to 469252	Special Infectious Agents Unit	Special Infectious Agents Unit	A.M.; Al-Sobahy; Azhar; E.I.; El-Kafrawy; Farraj; Hassan; N.A.; S.A.; T.L.; Tolah; Uthman
EPI_ISL_1553217 to 1553226	Springfield Clinic	Illinois Department of Public Health - Springfield Lab	Bryan Sim; Gordon McCall
EPI_ISL_1443220 to 1443221, EPI_ISL_1443233, EPI_ISL_1443275 to 1443276, EPI_ISL_1443285 to 1443286, EPI_ISL_1478844 to 1478846, EPI_ISL_1478884, EPI_ISL_1478922 to 1478924, EPI_ISL_1500219			
see above	Springfield Clinic Laboratory	Illinois Department of Public Health - Springfield Lab	Bryan Sim; Gordon McCall
EPI_ISL_1483207 to 1483209, EPI_ISL_1483232 to 1483234	Springfield-Greene Co. Health Dept.	Pathogen Discovery, Respiratory Viruses Branch, Division of Viral Diseases, Centers for Disease Control and Prevention	Anna Kelleher; Anna Uehara; Brian Lynch; Clinton R. Paden; Haibin Wang; Han Jia Justin Ng; Jing Zhang; Krista Queen; Peter Cook; Suxiang Tong; Yan Li; Ying Tao
EPI_ISL_1500241 to 1500242	St Margrets Hospital	Illinois Department of Public Health - Springfield Lab	Bryan Sim; Gordon McCall
EPI_ISL_513330, EPI_ISL_513347, EPI_ISL_513353 to 513356, EPI_ISL_513361 to 513365, EPI_ISL_513372, EPI_ISL_513379, EPI_ISL_513384 to 513385, EPI_ISL_513396 to 513399, EPI_ISL_526121 to 526123, EPI_ISL_526171, EPI_ISL_526177 to 526185, EPI_ISL_526187 to 526190, EPI_ISL_526194 to 526195, EPI_ISL_526200, EPI_ISL_544961, EPI_ISL_544968, EPI_ISL_544971 to 544973, EPI_ISL_544977 to 544981, EPI_ISL_544992 to 545001, EPI_ISL_545026, EPI_ISL_545030, EPI_ISL_547595 to 547596, EPI_ISL_591505 to 591506			
see above	St Vincent's Pathology (SydPath)	NSW Health Pathology - Institute of Clinical Pathology and Medical Research; Westmead Hospital; University of Sydney	CIDM-PH et al.
EPI_ISL_1553213	St. Clair County Health Department	Illinois Department of Public Health - Springfield Lab	Bryan Sim; Gordon McCall
EPI_ISL_514641, EPI_ISL_514646	St. Luke's Hospital	Minnesota Department of Health, Public Health Laboratory	Jacob Garfin; Matt Plumb; and Xiong Wang
EPI_ISL_1443207, EPI_ISL_1478848, EPI_ISL_1478882, EPI_ISL_1553227	St. Margaret's Hospital	Illinois Department of Public Health - Springfield Lab	Bryan Sim; Gordon McCall
EPI_ISL_1553245, EPI_ISL_1553249	St. Margarets Hospital	Illinois Department of Public Health - Springfield Lab	Bryan Sim; Gordon McCall
EPI_ISL_1492768, EPI_ISL_1493016 to 1493017, EPI_ISL_1493023, EPI_ISL_1493104, EPI_ISL_1533882	St. Nikolaus-Hospital Eupen	GIGA Medical Genomics	Bouchra Boujemla; Cécile Meex; Keith Durkin; Maria Artesi; Marie-Pierre Hayette; Nathalie Renotte; Pierrette Melin; Raphaël Boreux; Sébastien Bontems; Vincent Bours
EPI_ISL_591540	St.Johns Hospital	Department of Neurovirology, National Institute of Mental Health and Neuroscience (NIMHANS)	Anita Desai; Chitra Pattabiraman; Harsha PK; Manjunatha Venkataswamy; Pramada Prasad; Ravi Vasanthapuram; Risha Rasheed; Shafeeq S Hameed; Vijayalakshmi Reddy
EPI_ISL_450346 to 450352	St.Olavs hospital/NTNU	Institute of Genomics Core Facility, University of Tartu	Aleksandr Ianevski; Denis Kainov; Janne-Fossum Malmring; Svein Arne Nordbø; Tuuli Reisberg
EPI_ISL_596627 to 596676	St.Vincent's University Hospital	St.Vincent's University Hospital	Aisling Purcell; Gabriel Gonzalez; Guerrino Macori; Kirsten Schaffer; Lynda Fenelon; Mary Lucey; Niamh Mullane; Suzie Coughlan; Séamus Fanning; Una Sutton-Fitzpatrick
EPI_ISL_1483795 to 1483798, EPI_ISL_1508963	Stanford Clinical Virology Laboratory	Santa Clara County Public Health Laboratory	Santa Clara County Public Health Department
EPI_ISL_1424066 to 1424129	Stanford Health Care	Stanford University School of Medicine, Clinical Virology Laboratory	Becky Jiang; ChunHong Huang; Daniel Solis; Fumiko Yamamoto; James Zehnder; Malaya K. Sahoo; Mamdouh Sibai; Michelle Verghese; and Benjamin A. Pinsky
EPI_ISL_450445 to 450481, EPI_ISL_476767 to 476794	Stanford clinical virology lab	Chan-Zuckerberg Biohub	Benjamin Pinksy; Carlos Bustamante; Euan Ashley; Hannah N. DeJong; Jason Andrews; John Gorzynski; Katharine Walter; Manuel Rivas; Matthew T. Wheeler; Victoria N. Parikh; with CZB Cliahub Consortium
EPI_ISL_412912	State Health Office Baden-Württemberg	Charite Universitatsmedizin Berlin, Institute of Virology	Barbara Muhlemann; Christian Drosten; Julia Schneider; Jörn Beheim-Schwarzbach; Rainer Oehme; Silke Fischer; Talitha Veith; Terry Jones; Victor M Corman
EPI_ISL_1495111 to 1495143	State Institution «Public Health Center of Ministry of Health of Ukraine»	Robert Koch Institute, ZBS1 Highly Pathogenic Viruses, Berlin, Germany	Andreas Nitsche; Annika Brinkmann; Iryna Demchyshyna; Janine Michel; Liudmyla Chernenko; Roman Rodyna; Steven Uddin
EPI_ISL_416044, EPI_ISL_416046 to 416047, EPI_ISL_416425, EPI_ISL_416473 to 416474, EPI_ISL_418990 to 418991	State Key Laboratory for Diagnosis and Treatment of Infectious Diseases, National Clinical Research Center for Infectious Diseases, First Affiliated Hospital, Zhejiang University School of Medicine, Hangzhou, China 310003	State Key Laboratory for Diagnosis and Treatment of Infectious Diseases, National Clinical Research Center for Infectious Diseases, First Affiliated Hospital, Zhejiang University School of Medicine, Hangzhou, China 310003	Changzhong Jin; Chao Jiang; Fumin Liu; Haibo Wu; Hangping Yao; Lanjuan Li; Linfang Cheng; Min Zheng; Nanping Wu; Xiangyun Lu; Zhigang Wu
EPI_ISL_415709, EPI_ISL_415711, EPI_ISL_416042	State Key Laboratory for Diagnosis and Treatment of Infectious Diseases, National Clinical Research Center for Infectious Diseases, First Affiliated Hospital, Zhejiang University School of Medicine, Hangzhou, China. 310003	State Key Laboratory for Diagnosis and Treatment of Infectious Diseases, National Clinical Research Center for Infectious Diseases, First Affiliated Hospital, Zhejiang University School of Medicine, Hangzhou, China. 310003	Changzhong Jin; Chao Jiang; Fumin Liu; Haibo Wu; Hangping Yao; Lanjuan Li; Linfang Cheng; Min Zheng; Nanping Wu; Xiangyun Lu; Zhigang Wu
EPI_ISL_417443	State Key Laboratory for Emerging Infectious Diseases Department of Microbiology Li Ka Shing Faculty of Medicine The University of Hong Kong	State Key Laboratory for Emerging Infectious Diseases Department of Microbiology Li Ka Shing Faculty of Medicine The University of Hong Kong	Bobo Wing-Yee Mok; Honglin Chen; Kwok-Yung Yuen; Pui Wang; Shaofeng Deng; Siu-Ying Lau; Wenjun Song
EPI_ISL_444273	State Key Laboratory of Respiratory Disease, National Clinical Research Center for Respiratory Disease, Guangzhou Institute of Respiratory Health, the First Affiliated Hospital of Guangzhou Medical University	State Key Laboratory of Respiratory Disease, National Clinical Research Center for Respiratory Disease, Guangzhou Institute of Respiratory Health, the First Affiliated Hospital of Guangzhou Medical University	Huang; J. and Zhao, J.; Shi, Y.; Sun, J.; Zheng, K.
EPI_ISL_414663, EPI_ISL_414686, EPI_ISL_414688 to 414692	State Key Laboratory of Respiratory Disease, National Clinical Research Center for Respiratory Disease, Guangzhou Institute of Respiratory Health, the First Affiliated Hospital of Guangzhou Medical University	The First Affiliated Hospital of Guangzhou Medical University & BGI-Shenzhen	Zhao et al
EPI_ISL_414687	State Key Laboratory of Respiratory Disease, National Clinical Research Center for Respiratory Disease, Guangzhou Institute of Respiratory Health, the First Affiliated Hospital of Guangzhou Medical University	the First Affiliated Hospital of Guangzhou Medical University & BGI-Shenzhen	Zhao et al
EPI_ISL_1515801 to 1515821, EPI_ISL_1515823 to 1515835, EPI_ISL_1515837 to 1515839, EPI_ISL_1515842 to 1515994, EPI_ISL_1515996 to 1515997, EPI_ISL_1515999 to 1516006	State Laboratories Division, Hawaii State Department of Health	State Laboratories Division, Hawaii State Department of Health	Ayana Garnet; Drew Kuwazaki; Edward Desmond; Pamela O'Brien; Razvan Sultana
EPI_ISL_428860 to 428924	State Research Center of Virology and Biotechnology	State Research Center of Virology and Biotechnology	Alexander N. Shvalov; Anastasiya A. Nazarenko; Anastasiya M. Smirnova; Elena V. Gavrilova; Oleg V. Pyankov; Rinat A. Maksyutov; Sergey A. Bodnev;

	VECTOR, Department of Collection of Microorganisms	VECTOR, Department of Collection of Microorganisms	Tatyana V. Tregubchak
EPI_ISL_451655 to 451664	State Sanitary Inspectorate	Laboratory of Recombinant Vaccines	Boguslaw Szewczyk; Jaroslaw Pinkas; Krystyna Bienkowska-Szewczyk; Lukasz Rabalski
EPI_ISL_1497613 to 1497617	State Veterinary Institute Prague	State Veterinary Institute Prague	A; Cernikova; L; M; Nagy; Stara
EPI_ISL_420149	Stavanger University Hospital, Department of Medical Microbiology	Norwegian Institute of Public Health, Department of Virology	Hilde Elshaug; Kamilla Heddeland Instefjord; Karoline Bragstad; Kathrine Stene-Johansen; Olav Hungnes
EPI_ISL_1435385, EPI_ISL_1435391 to 1435392, EPI_ISL_1435406	Stiftung Krankenhaus Bethanien für die Grafschaft Moers	Robert Koch Institute	
EPI_ISL_1534280, EPI_ISL_1534301, EPI_ISL_1534311, EPI_ISL_1534335, EPI_ISL_1534368, EPI_ISL_1534417, EPI_ISL_1534428			
see above	Still Bay Sat Clinic wc SFV	NHLS/UCT	Arash Iranzadeh; Bruna Galvao; Carolyn Williamson; Deelan Doolabh; Diana Hardie; Emmanuel SJ; Innocent Mudau; Kruger Marais; Lynn Tyers; Marvin Hsiao; Stephen Korsman; Tegally H; de Oliveira T
EPI_ISL_582799, EPI_ISL_582839	Stockholm_KUL Solna	The Public Health Agency of Sweden	Anna Risberg; Anna-Malin Linde; Karin Tegmark-Wisell; Maria Lind Karlberg; Mattias Haukland; Mia Brytting; Olov Svartstrom; Oskar Karlsson Lindsjo; Petra Edquist; Reza Advani; Sandra Broddesson
EPI_ISL_1441247, EPI_ISL_1441254, EPI_ISL_1441290, EPI_ISL_1441305	Städtisches Klinikum Dresden	Robert Koch Institute	
EPI_ISL_1398267, EPI_ISL_1398355, EPI_ISL_1398359, EPI_ISL_1398375, EPI_ISL_1398561, EPI_ISL_1398924, EPI_ISL_1398927, EPI_ISL_1398930, EPI_ISL_1398981			
see above	Subang Public Health	West Java Health Laboratory; School of Life Sciences and Technology, Institut Teknologi Bandung	Azzania Fibriani; Cut Nur Cinthia Alamanda; Ema Rahmawati; Isak Solihin; Kamila Tania; Karimatu Khoirunnisa; Miftahul Faridl; Rifky Waluyajati Rachman; Rini Robiani; Ryan Bayusantika Ristandi
EPI_ISL_491036 to 491091	Suceava County Emergency Hospital	"Stefan cel Mare" University Metagenomics Lab	Antoniadis Panagiotis et al.; Lobiuc Andrei; Lobiuc Andrei et al.
EPI_ISL_486834	Suceava County Energency Hospital "Sf. Ioan cel Nou"	SMU Metagenomics lab	Antoniadis Panagiotis; Lobiuc Andrei
EPI_ISL_1398374	Sukabumi Public Health	West Java Health Laboratory; School of Life Sciences and Technology, Institut Teknologi Bandung	Azzania Fibriani; Cut Nur Cinthia Alamanda; Ema Rahmawati; Isak Solihin; Kamila Tania; Karimatu Khoirunnisa; Miftahul Faridl; Rifky Waluyajati Rachman; Rini Robiani; Ryan Bayusantika Ristandi
EPI_ISL_455072 to 455073	Sullivan Nicolaides Pathology	NSW Health Pathology - Institute of Clinical Pathology and Medical Research; Westmead Hospital; University of Sydney	CIDM-PH et al.
EPI_ISL_417033	Sullivan Nicolaides Pathology	Public Health Virology Laboratory	Alyssa Pyke; Amanda De Jong; Andrew Van Den Hurk; Bixing Huang; Carmel Taylor; David Warrilow; Doris Genge; Elisabeth Gamez; Glen Hewitson; Ian Maxwell Mackay; Inga Sultana; Jamie McMahon; Jean Barcelon; Judy Northill; Mitchell Finger; Natalie Simpson; Neelima Nair; Peter Burtonclay; Peter Moore; Sarah Wheatley; Sean Moody; Sonja Hall-Mendelin; Timothy Gardam; and Frederick Moore
EPI_ISL_1398365, EPI_ISL_1398980	Sumedang Public Health	West Java Health Laboratory; School of Life Sciences and Technology, Institut Teknologi Bandung	Azzania Fibriani; Cut Nur Cinthia Alamanda; Ema Rahmawati; Isak Solihin; Kamila Tania; Karimatu Khoirunnisa; Miftahul Faridl; Rifky Waluyajati Rachman; Rini Robiani; Ryan Bayusantika Ristandi
EPI_ISL_529719	Sumedang Regional General Hospital	Laboratorium Kesehatan Provinsi Jawa Barat; School of Life Sciences and Technology & School of Pharmacy-Institut Teknologi Bandung; Molecular Genetics Laboratory-Faculty of Medicine-Universitas Padjadjaran	Adelina Khristiani Rahayu; Agung Eru Wibowo; Azzania Fibriani; Catur Riani; Cut Nur Cinthia Alamanda; Ema Rahmawati; Gusti Ayu Prani Pradani; Hammam Riza; Hesti Lina Wiraswati; Husna Nugrahapraja; Irvan Faizal; Lia Faridah; Marselina Irasonia Tan; Rifky Waluyajati Rachman; Ryan Bayusantika Ristandi; Savira Ekawardhani; Sony Solistia Wirawan; Tarwadi; Yunia Sribudiani
EPI_ISL_534244, EPI_ISL_560986	Sundsvalls sjukhus	The Public Health Agency of Sweden	Anna Risberg; Anna-Malin Linde; Karin Tegmark-Wisell; Maria Lind Karlberg; Mattias Haukland; Mia Brytting; Olov Svartstrom; Oskar Karlsson Lindsjo; Petra Edquist; Reza Advani; Sandra Broddesson
EPI_ISL_450747	Sunnybrook Health Sciences Centre	Department of Laboratory Medicine and Molecular Diagnostics, Sunnybrook Health Sciences Centre	Amogelang R. Raphenya; Andrew G. McArthur; Arinjay Banerjee; Bo Wang; Brian P. Alcock; Finlay Maguire; Hamza Mbareche; Hassaan Maan; Jalees A. Nasir; Julian A. Hiscox; Karen Mossman; Kendrick M. Smith; Muhannad Alruwaili; Natalie C. Knox; Patryk Aftanas; Robert A. Kozak; Samira Mubareka
EPI_ISL_1544118 to 1544132, EPI_ISL_1544135 to 1544141, EPI_ISL_1544166 to 1544169	Supratech Micropath Laboratory Research Institute Pvt Ltd, Ahmedabad	Gujarat Biotechnology Research Centre	Chaitanya Joshi; Dinesh Kumar; Janvi Raval; Madhvi Joshi; Mahendra Parikh; Nitesh Shah; Nitin Savaliya; Ramesh Pandit; Sonal Sharma; Twinkle Soni; Umang Mishra; Zarna Patel; Zuber Saiyed
EPI_ISL_434663, EPI_ISL_434672, EPI_ISL_434675 to 434676, EPI_ISL_445225, EPI_ISL_455106, EPI_ISL_469066, EPI_ISL_469070, EPI_ISL_475543, EPI_ISL_475564, EPI_ISL_476136			
see above	Surbrunns VC	The Public Health Agency of Sweden	Anna Risberg; Anna-Malin Linde; Erik Embring; Karin Tegmark-Wisell; Maria Lind Karlberg; Mattias Haukland; Mia Brytting; Olov Svartstrom; Oskar Karlsson Lindsjo; Petra Edquist; Reza Advani; Sandra Broddesson; Shamam Muradrasoli; Theresa Enkirch
EPI_ISL_434645, EPI_ISL_434649	Svardsjo VC	The Public Health Agency of Sweden	Anna Risberg; Anna-Malin Linde; Karin Tegmark-Wisell; Maria Lind Karlberg; Mia Brytting; Olov Svartstrom; Oskar Karlsson Lindsjo; Theresa Enkirch; Tommy Janers
EPI_ISL_1417216 to 1417438, EPI_ISL_1417443 to 1418186, EPI_ISL_1418331 to 1419047, EPI_ISL_1419925 to 1420634	Swedish national genomic surveillance program of SARS-CoV-2	The Public Health Agency of Sweden	Swedish national genomic surveillance program of SARS-CoV-2
EPI_ISL_478717 to 478718, EPI_ISL_498765, EPI_ISL_526125, EPI_ISL_544956, EPI_ISL_545037, EPI_ISL_545044, EPI_ISL_545049, EPI_ISL_547598, EPI_ISL_591507			
see above	Sydney South West Pathology Service (SSWPS) - Concord Repatriation General Hospital - NSW Health Pathology	NSW Health Pathology - Institute of Clinical Pathology and Medical Research; Westmead Hospital; University of Sydney	CIDM-PH et al.
EPI_ISL_478675 to 478682, EPI_ISL_478706 to 478707, EPI_ISL_478712 to 478716, EPI_ISL_490020, EPI_ISL_490037, EPI_ISL_498763 to 498764, EPI_ISL_498778, EPI_ISL_498781 to 498782, EPI_ISL_513323, EPI_ISL_513327 to 513329, EPI_ISL_513333 to 513334, EPI_ISL_513336 to 513337, EPI_ISL_513366 to 513367, EPI_ISL_513373 to 513374, EPI_ISL_513381 to 513383, EPI_ISL_513401, EPI_ISL_513409 to 513412, EPI_ISL_526119 to 526120, EPI_ISL_526124, EPI_ISL_526126 to 526127, EPI_ISL_526131, EPI_ISL_526157 to 526160, EPI_ISL_526172 to 526176, EPI_ISL_526193, EPI_ISL_526197 to 526198, EPI_ISL_526206, EPI_ISL_526210 to 526211, EPI_ISL_544958 to 544959, EPI_ISL_544964 to 544965, EPI_ISL_544985, EPI_ISL_545003, EPI_ISL_545025, EPI_ISL_545042, EPI_ISL_547594, EPI_ISL_591508 to 591516, EPI_ISL_593755 to 593765			
see above	Sydney South West Pathology Service (SSWPS) - Liverpool Hospital - NSW Health Pathology	NSW Health Pathology - Institute of Clinical Pathology and Medical Research; Westmead Hospital; University of Sydney	CIDM-PH et al.
EPI_ISL_513400, EPI_ISL_526134 to 526137, EPI_ISL_526196, EPI_ISL_526201, EPI_ISL_526207 to 526209, EPI_ISL_544974, EPI_ISL_545004 to 545005, EPI_ISL_545017 to 545023, EPI_ISL_545028, EPI_ISL_545039, EPI_ISL_545041, EPI_ISL_545043, EPI_ISL_545045, EPI_ISL_547599, EPI_ISL_591517, EPI_ISL_593766 to 593768			
see above	Sydney South West Pathology Service (SSWPS) - Royal Prince Alfred Hospital - NSW Health Pathology	NSW Health Pathology - Institute of Clinical Pathology and Medical Research; Westmead Hospital; University of Sydney	CIDM-PH et al.
EPI_ISL_1385836	Syngene	INSACOG-KA, NIMHANS	Anita S Desai; Anson Kunjumon George; Chitra Pattabiraman; Darshan Sreenivas; Nakka Vijay Kiran Reddy; Pramada Prasad; V Ravi
EPI_ISL_1470001 to 1470058, EPI_ISL_1470078 to 1470121, EPI_ISL_1470137 to 1470296	Synlab Eesti OÜ	1. Laboratory of Communicable Diseases (Estonia); 2. Eurofins Genomics Europe Sequencing GmbH	Liidia Dotsenko et al.
EPI_ISL_1437612, EPI_ISL_1437621, EPI_ISL_1437653, EPI_ISL_1437667 to 1437668, EPI_ISL_1437699, EPI_ISL_1437715, EPI_ISL_1437728, EPI_ISL_1437742, EPI_ISL_1437746, EPI_ISL_1437748, EPI_ISL_1437750, EPI_ISL_1437757, EPI_ISL_1437762, EPI_ISL_1437764, EPI_ISL_1437767, EPI_ISL_1437772, EPI_ISL_1437776, EPI_ISL_1437810, EPI_ISL_1437815, EPI_ISL_1437819, EPI_ISL_1437845, EPI_ISL_1437859, EPI_ISL_1437861, EPI_ISL_1437863, EPI_ISL_1437892, EPI_ISL_1437901, EPI_ISL_1437905, EPI_ISL_1437913, EPI_ISL_1437915, EPI_ISL_1437927, EPI_ISL_1440305, EPI_ISL_1440312, EPI_ISL_1440338, EPI_ISL_1440345 to 1440346, EPI_ISL_1440350, EPI_ISL_1440352, EPI_ISL_1440355, EPI_ISL_1440359, EPI_ISL_1440394, EPI_ISL_1440416, EPI_ISL_1440421, EPI_ISL_1440429, EPI_ISL_1440446, EPI_ISL_1440452, EPI_ISL_1440460, EPI_ISL_1440462, EPI_ISL_1440475, EPI_ISL_1440492, EPI_ISL_1440518, EPI_ISL_1440524, EPI_ISL_1440527, EPI_ISL_1440531, EPI_ISL_1440551, EPI_ISL_1440568, EPI_ISL_1442588, EPI_ISL_1442598, EPI_ISL_1442608, EPI_ISL_1442616, EPI_ISL_1442697, EPI_ISL_1442707, EPI_ISL_1442709, EPI_ISL_1442714 to 1442715, EPI_ISL_1442728			
see above	Synlab MVZ Augsburg	Robert Koch Institute	
EPI_ISL_1538444, EPI_ISL_1538480, EPI_ISL_1538665 to 1538667, EPI_ISL_1538707 to 1538718, EPI_ISL_1538862, EPI_ISL_1538988 to 1538989, EPI_ISL_1539013			
see above	Szpital Uniwersytecki im. Karola Marcinkowskiego	1. National Institute of Public Health - National Institute of Hygiene; 2. Eurofins Genomics Europe Sequencing GmbH	ECDC COVID-19 WGS support team; Eurofins Genomics Europe Sequencing Team; Gierczyki Rafa; Sadkowska-Todys Magorzata; Wokowicz Tomasz; Zacharczuk Katarzyna

EPI_ISL_455719	T.C. Salk Bakanl Adyaman l Salk Müdürlüü Adyaman Eitim Ve Aratrma Hastanesi	VETAL Animal Health Products Company, BSL3+ Production Laboratuary /Turkey	Abidin Ercan Yonucu; Fatma Nilay Tutak; Fethiye Sevimli; Haluk Uluca; Mehmet Turgut; Muhittin Önderci; Murat Dönen; O. Ugur Sezerman
EPI_ISL_435057	T.C. Salk Bakanl Adyaman l Salk Müdürlüü Adyaman Eitim Ve Aratrma Hastanesi	VETAL Animal Health Products Company, BSL3+ Production Laboratuary, Turkey	Abidin Ercan Yonucu; Fatma Nilay Tutak; Fethiye Sevimli; Haluk Uluca; Mehmet Turgut; Muhittin Önderci; Murat Dönen; O. Ugur Sezerman
EPI_ISL_1397553 to 1397554, EPI_ISL_1397581 to 1397591, EPI_ISL_1397700 to 1397705, EPI_ISL_1397836, EPI_ISL_1397838 to 1397842, EPI_ISL_1397844 to 1397852, EPI_ISL_1397857, EPI_ISL_1397859 to 1397860, EPI_ISL_1397862 to 1397865, EPI_ISL_1461356 to 1461377, EPI_ISL_1462552, EPI_ISL_1465859 to 1465878			
see above	TEMPUS LABS INC	Wadsworth Center, New York State Department of Health	Alexis Russel; Alexis Russell; Catharine Prussing; Daryl M. Lamson; Erasmus Schneider; Erica Lasek-Nesselquist; John Kelly; Jonathan Plitnick; Kirsten St. George; Matthew Shudt; Melissa A Leisner; Navjot Singh
EPI_ISL_426499 to 426507, EPI_ISL_426509 to 426511, EPI_ISL_426520 to 426526, EPI_ISL_426532 to 426536, EPI_ISL_426556 to 426557, EPI_ISL_1392000, EPI_ISL_1392019, EPI_ISL_1392021, EPI_ISL_1392036, EPI_ISL_1392055, EPI_ISL_1392080, EPI_ISL_1392104, EPI_ISL_1392124, EPI_ISL_1392140, EPI_ISL_1392156, EPI_ISL_1392164, EPI_ISL_1392173, EPI_ISL_1392177, EPI_ISL_1392183, EPI_ISL_1392199, EPI_ISL_1392277, EPI_ISL_1392316, EPI_ISL_1392447, EPI_ISL_1392464, EPI_ISL_1392499, EPI_ISL_1392518, EPI_ISL_1392534, EPI_ISL_1392541, EPI_ISL_1392573, EPI_ISL_1392633, EPI_ISL_1464693, EPI_ISL_1464695, EPI_ISL_1464719, EPI_ISL_1464745, EPI_ISL_1464814, EPI_ISL_1464874, EPI_ISL_1464904, EPI_ISL_1464950, EPI_ISL_1464969, EPI_ISL_1465157, EPI_ISL_1465324, EPI_ISL_1465360, EPI_ISL_1465362, EPI_ISL_1465417, EPI_ISL_1465423, EPI_ISL_1500642, EPI_ISL_1500644 to 1500645, EPI_ISL_1500647, EPI_ISL_1500671, EPI_ISL_1500676, EPI_ISL_1500678 to 1500681, EPI_ISL_1500686, EPI_ISL_1500695, EPI_ISL_1500698, EPI_ISL_1500703, EPI_ISL_1500705, EPI_ISL_1500711, EPI_ISL_1500714, EPI_ISL_1500717, EPI_ISL_1500722, EPI_ISL_1500728, EPI_ISL_1500732, EPI_ISL_1500734, EPI_ISL_1500752 to 1500754, EPI_ISL_1500767, EPI_ISL_1500772 to 1500773, EPI_ISL_1500777 to 1500778, EPI_ISL_1500780, EPI_ISL_1500786 to 1500787, EPI_ISL_1500789, EPI_ISL_1500793, EPI_ISL_1500797, EPI_ISL_1500801, EPI_ISL_1500805, EPI_ISL_1500812 to 1500813, EPI_ISL_1500823, EPI_ISL_1500827, EPI_ISL_1500834, EPI_ISL_1500852, EPI_ISL_1500878, EPI_ISL_1500896, EPI_ISL_1500905 to 1500906, EPI_ISL_1500910, EPI_ISL_1500912, EPI_ISL_1500915, EPI_ISL_1500918, EPI_ISL_1500921, EPI_ISL_1500933, EPI_ISL_1500938 to 1500939, EPI_ISL_1500945, EPI_ISL_1530563, EPI_ISL_1530567, EPI_ISL_1530570, EPI_ISL_1530574, EPI_ISL_1530579, EPI_ISL_1530590, EPI_ISL_1530595, EPI_ISL_1530597, EPI_ISL_1530599 to 1530602, EPI_ISL_1530604, EPI_ISL_1530607, EPI_ISL_1530613, EPI_ISL_1530619, EPI_ISL_1530624, EPI_ISL_1530628, EPI_ISL_1530630, EPI_ISL_1530634, EPI_ISL_1530640, EPI_ISL_1530642, EPI_ISL_1530647, EPI_ISL_1530657, EPI_ISL_1530663, EPI_ISL_1530666, EPI_ISL_1530668, EPI_ISL_1530671, EPI_ISL_1530676, EPI_ISL_1530682, EPI_ISL_1530686, EPI_ISL_1530688 to 1530689, EPI_ISL_1530691, EPI_ISL_1530697, EPI_ISL_1530699, EPI_ISL_1530704, EPI_ISL_1530708, EPI_ISL_1530716, EPI_ISL_1530719, EPI_ISL_1530721, EPI_ISL_1530723 to 1530724, EPI_ISL_1530729 to 1530730, EPI_ISL_1530732, EPI_ISL_1530734, EPI_ISL_1530736, EPI_ISL_1530741, EPI_ISL_1530743 to 1530744, EPI_ISL_1530754 to 1530755, EPI_ISL_1530760, EPI_ISL_1530763, EPI_ISL_1530768, EPI_ISL_1530783, EPI_ISL_1530785, EPI_ISL_1530788, EPI_ISL_1530791 to 1530792, EPI_ISL_1530794, EPI_ISL_1530802, EPI_ISL_1530805 to 1530810, EPI_ISL_1530813, EPI_ISL_1530821, EPI_ISL_1530823 to 1530825, EPI_ISL_1530831, EPI_ISL_1530833, EPI_ISL_1530846, EPI_ISL_1530853, EPI_ISL_1530855, EPI_ISL_1530858, EPI_ISL_1530865 to 1530867, EPI_ISL_1530869 to 1530872, EPI_ISL_1550882, EPI_ISL_1550899, EPI_ISL_1550901			
see above	TGen North	TGen North	"Jolene Bowers; Ashlyn Pfeiffer; Chris French; Darrin Lemmer; Dave Engelthaler; Hayley Yaglom; Heather Centner; Jolene Bowers; Megan Folkerts; The Arizona COVID Genomics Union (ACGU); The Arizona COVID Genomics Union (ACGU)"
EPI_ISL_1395766, EPI_ISL_1395768 to 1395769, EPI_ISL_1395771, EPI_ISL_1395773, EPI_ISL_1397612 to 1397617, EPI_ISL_1397783, EPI_ISL_1397966 to 1397967, EPI_ISL_1397988, EPI_ISL_1398259 to 1398263, EPI_ISL_1462572 to 1462573, EPI_ISL_1462575, EPI_ISL_1465673 to 1465677, EPI_ISL_1465760			
see above	THE MARY IMOGENE BASSETT HOSPITAL	Wadsworth Center, New York State Department of Health	Alexis Russel; Alexis Russell; Catharine Prussing; Daryl M. Lamson; Erasmus Schneider; Erica Lasek-Nesselquist; John Kelly; Jonathan Plitnick; Kirsten St. George; Matthew Shudt; Melissa A Leisner; Navjot Singh
EPI_ISL_1446284, EPI_ISL_1446287, EPI_ISL_1446582, EPI_ISL_1446592, EPI_ISL_1446740, EPI_ISL_1446945, EPI_ISL_1516197, EPI_ISL_1528404 to 1528405			
see above	TN Division of Laboratory Services	Centers for Disease Control and Prevention Division of Viral Diseases, Pathogen Discovery	Alison Laufer Halpin; Ben L. Rambo-Martin; Clinton R. Paden; Dakota Howard; Darlene Wagner; Dave Wentworth; Dhwani Batra; Jasmine Padilla; Justin Lee; Katie Dillon; Krista Queen; Kristen Knipe; Kristine Lacek; Mark Burroughs; Matthew Schmerer; Mili Sheth; Peter Cook; Sam Shepard; Sarah Nobles; Shoshona Le; Suxiang Tong; Vivien Dugan; Yvette Unoarumhi
EPI_ISL_1408888 to 1408889, EPI_ISL_1408898, EPI_ISL_1448518, EPI_ISL_1448523, EPI_ISL_1509951	TN Division of Laboratory Services	Genomics and Discovery, Respiratory Viruses Branch, Division of Viral Diseases, Centers for Disease Control and Prevention	Adam Retchless; Anna Kelleher; Anna Montmayeur; Anna Uehara; Brian Lynch; Clinton R. Paden; Haibin Wang; Han Jia Justin Ng; Jing Zhang; Justin Lee; Krista Queen; Mark Burroughs; Peter Cook; Rachel Marine; Suxiang Tong; Yan Li; Ying Tao
EPI_ISL_540432 to 540434, EPI_ISL_576172, EPI_ISL_576175 to 576176	TN Division of Laboratory Services	Pathogen Discovery, Respiratory Viruses Branch, Division of Viral Diseases, Centers for Disease Control and Prevention	Anna Montmayeur; Anna Uehara; Brian Lynch; Clinton R. Paden; Haibin Wang; Jing Zhang; Krista Queen; Peter Cook; Rachel Marine; Suxiang Tong; Yan Li; Ying Tao
EPI_ISL_528419 to 528424	TNMC & BYL NAIR CH. HOSPITAL	Institute of Genomics and Integrative Biology - Council of Scientific and Industrial Research	Akshay Kanakan; Amol Borse; Janani Srinivasa Vasudevan; Jasmina Savak; Jayanthi Shastri; Mahesh Sangar; Manish Pathak; Mayuresh Vishwanathan; Nirjhar Chatterjee; Rajesh Pandey; Ranjeet Maurya; Sachee Agrawal; Shruthi Sachidanandan; Shrutika Pophale; Shweta Kawankar; Subrat Thanapati; Suresh Poojari; Swapneil Parikh; Utkarsha Yelve; Vasil Nachan; Vivekanand A
EPI_ISL_426629 to 426632, EPI_ISL_427392 to 427398, EPI_ISL_428229 to 428231, EPI_ISL_436099 to 436108, EPI_ISL_447252 to 447257, EPI_ISL_447591 to 447593, EPI_ISL_457726, EPI_ISL_457730, EPI_ISL_457733, EPI_ISL_458029			
see above	TSGH-CP molecular lab	TSGH-CP molecular lab	Cherng-Lih Perng; Chien-Wen Chen; Chih-Kai Chang; Feng-Yee Chang; Hsing-Yi Chung; Hung-Sheng Shang; Jung-Chung Lin; Kuo-Ming Yeh; Kuo-Sheng Hung; Ming-Jr JIAN; Ming-Jr Jian; Sheng-Kang Chiu; Shih-Hung Tsai; Tien-Yao Chang
EPI_ISL_437336	TSGH-CP molecular lab, Division of Clinical Pathology, Department of Pathology	TSGH-CP molecular lab, Division of Clinical Pathology, Department of Pathology	Cherng-Lih Perng; Chien-Wen Chen; Chih-Kai Chang; Feng-Yee Chang; Hsing-Yi Chung; Hung-Sheng Shang; Jung-Chung Lin; Kuo-Ming Yeh; Kuo-Sheng Hung; Ming-Jr JIAN; Sheng-Kang Chiu; Shih-Hung Tsai; Tien-Yao Chang
EPI_ISL_1446980 to 1446984, EPI_ISL_1447046	TX-Houston Health Department Laboratory	Centers for Disease Control and Prevention Division of Viral Diseases, Pathogen Discovery	Alison Laufer Halpin; Ben L. Rambo-Martin; Clinton R. Paden; Dakota Howard; Darlene Wagner; Dave Wentworth; Dhwani Batra; Jasmine Padilla; Justin Lee; Katie Dillon; Krista Queen; Kristen Knipe; Kristine Lacek; Mark Burroughs; Matthew Schmerer; Mili Sheth; Peter Cook; Sam Shepard; Sarah Nobles; Shoshona Le; Suxiang Tong; Vivien Dugan; Yvette Unoarumhi
EPI_ISL_1392714 to 1392799, EPI_ISL_1422238 to 1422326, EPI_ISL_1444262 to 1444346, EPI_ISL_1478933 to 1479015, EPI_ISL_1527538 to 1527629	TXDSHS	TXDSHS	Anita Pokharel; Bonnie Oh; Chun Wang; Grace Kubin; Jenny Zhang; Lorraine Rodriguez; Maliha Rahman; Mayela Pedrueza; Myong Koag; Rachel Lee; Rashmi Tuladhar
EPI_ISL_1443153 to 1443156	TYKS, Kliininen mikrobiologia	Expert Microbiology, National Institute for Health and Welfare	Carita Savolainen-Kopra; Haider al-Hello; Jani Halkilahti; Kirsi Liitsola; Niina Ikonen; Pekka Ellonen; Päivi Laurila; Sari Hannula; Soile Blomqvist; Teemu Smura
EPI_ISL_414518	Tai Lung Veterinary Laboratory, Agriculture, Fisheries and Conservation Department	School of Public Health, The University of Hong Kong	Christopher J Brackman; Daniel K.W. Chu; Leo L.M. Poon; Malik Peiris; Thomas S.H. Chung
EPI_ISL_411926 to 411927	Taiwan Centers for Disease Control	Taiwan Centers for Disease Control	Ji-Rong Yang; Jung-Jung Mu; Ming-Tsan-Liu; Yu-Chi-Lin
EPI_ISL_412968 to 412969, EPI_ISL_420889 to 420890, EPI_ISL_445183	Takayuki Hishiki Kanagawa Prefectural Institute of Public Health	Takayuki Hishiki Kanagawa Prefectural Institute of Public Health	An, T.; Hayashizaki; Hishiki, T.; Kawai, J.; Kogo, Y.; Matsuki, Y.; Sakuragi, J.; Suzuki, R.; Tanaka, Y.; Usui, K.; Y. and Takasaki, T.
EPI_ISL_447584 to 447587	Tamil Nadu Veterinary and Animal Sciences University	CSIR-Centre for Cellular and Molecular Biology	Archana Bharadwaj Siva; Dhiviya Vedagiri; Divya Gupta; Divya Tej Sowpati; G Dhinakar Raj; G Ravi Kumar; K Kaveri; Karthik Bharadwaj Tallapaka; Krishnan Harinivas Harshan; Kumarasamy Thangaraj; Lamuk Zaveri; Namami Gaur; P Padmapriya; Payel Mukherjee; Priya Singh; Purushotham Vodnala; R Kiruba; Rakesh K Mishra; S Magesh; S Sivasubramanian; S Vennila; Sakshi Shambhavi; Santosh Kumar Kuncha; Shagufta Khan; Sofia Banu; Tulasi Nagabandi; Vishal Sah
EPI_ISL_1483279 to 1483318, EPI_ISL_1492444 to 1492455	Tampa General Hospital Esoteric Lab	Tampa General Hospital Esoteric Research & Development Lab	Amorce Lima; Deanna Becker; Dominic Uy; Grant Vestal; Suzane Silbert; Vicki Healer
EPI_ISL_1455673	TaskForce Covid19-Ceinge Regione Campania	TaskForce Covid19-Ceinge Regione Campania	Massimo Zollo (1) Ettore Capoluongo (1) Veronica Ferrucci (1) Rossella Tufano (1) Angelo Boccia (1) Giovanni Paolella (1) 1) TaskForce Covid19-Ceinge Regione Campania
EPI_ISL_1443539 to 1443600	Teaching Institute for Public Health of Split-Dalmatia County	Croatian Institute of Public Health	Irena Tabain; Ivana Ferenak
EPI_ISL_1388124, EPI_ISL_1388568, EPI_ISL_1389060	Team W	University Hospital Basel, Clinical Bacteriology	Adrian Egli; Alfredo Mari; Hans Hirsch; Helena MB Seth-Smith; Julia Bielicki; Karoline Leuzinger; Madlen Stange; Manuel Battegay; Simon Fuchs; Tim Roloff
EPI_ISL_529149 to 529150	Technology Centre, Guangzhou Customs	Technology Centre, Guangzhou Customs	Dai, J.; Huang, J.; Huang, S.; Li, X.; Shi, Y.; Sun, F.; Sun, J.; Wang, Y.; Zhang, Z.; Zheng, K.; Zhu, .; Zhuang, Z.
EPI_ISL_412966 to 412967	Technology Centre, Guangzhou Customs	Technology Centre, Guangzhou Customs	Chen, Z.; Dai, J.; Huang; Huang, J.; J. and Zhao, J.; Li; Shi, Y.; Sun, F.; Sun, J.; X. and Wang, Y.; Zhang, Z.; Zheng, K.; Zhu, A.; Zhuang, Z.
EPI_ISL_514438 to 514439	Tewhey Lab, The Jackson Laboratory	Tewhey Lab, The Jackson Laboratory	Barter, M.; Dewey, H.; Lynch, R.; Matluk, N.; Munger, H.; Tewhey, R.
EPI_ISL_1446195 to 1446283, EPI_ISL_1446285 to 1446286, EPI_ISL_1446288 to 1446289, EPI_ISL_1446291 to 1446292, EPI_ISL_1446294 to 1446295, EPI_ISL_1446297 to 1446298, EPI_ISL_1446300 to 1446301, EPI_ISL_1446303, EPI_ISL_1446305 to 1446306, EPI_ISL_1446308 to 1446309, EPI_ISL_1446311 to 1446312, EPI_ISL_1446314, EPI_ISL_1446316 to 1446317, EPI_ISL_1446319 to 1446320, EPI_ISL_1446322 to 1446323, EPI_ISL_1446325 to 1446326, EPI_ISL_1446328 to 1446329, EPI_ISL_1446331, EPI_ISL_1446333 to 1446334, EPI_ISL_1446336 to 1446337, EPI_ISL_1446339 to 1446340, EPI_ISL_1446342 to 1446343, EPI_ISL_1446345 to 1446346, EPI_ISL_1446348 to 1446349, EPI_ISL_1446351, EPI_ISL_1446353 to 1446354, EPI_ISL_1446356 to 1446357, EPI_ISL_1446359 to 1446360, EPI_ISL_1446362 to 1446363, EPI_ISL_1446365 to 1446366, EPI_ISL_1446368 to 1446382, EPI_ISL_1446384 to 1446385, EPI_ISL_1446387 to 1446388, EPI_ISL_1446390 to 1446391, EPI_ISL_1446393 to 1446394, EPI_ISL_1446396 to 1446397, EPI_ISL_1446399 to 1446400, EPI_ISL_1446402 to 1446403, EPI_ISL_1446405, EPI_ISL_1446407 to 1446408, EPI_ISL_1446410 to 1446411, EPI_ISL_1446413 to 1446414, EPI_ISL_1446416 to 1446417, EPI_ISL_1446419,			

EPI_ISL_1446421 to 1446422, EPI_ISL_1446424, EPI_ISL_1446426, EPI_ISL_1446428 to 1446429, EPI_ISL_1446431 to 1446432, EPI_ISL_1446434 to 1446435, EPI_ISL_1446437 to 1446438, EPI_ISL_1446440, EPI_ISL_1446442 to 1446443, EPI_ISL_1446445 to 1446446, EPI_ISL_1446448 to 1446449, EPI_ISL_1446451 to 1446452, EPI_ISL_1446454 to 1446455, EPI_ISL_1446457 to 1446458, EPI_ISL_1446460 to 1446461, EPI_ISL_1446463 to 1446464, EPI_ISL_1446466 to 1446467, EPI_ISL_1446469 to 1446470, EPI_ISL_1446472, EPI_ISL_1446474 to 1446475, EPI_ISL_1446477 to 1446479, EPI_ISL_1446481, EPI_ISL_1446483 to 1446485, EPI_ISL_1446487 to 1446488, EPI_ISL_1446490, EPI_ISL_1446492 to 1446493, EPI_ISL_1446495 to 1446496, EPI_ISL_1446498, EPI_ISL_1446500 to 1446501, EPI_ISL_1446503 to 1446504, EPI_ISL_1446506 to 1446507, EPI_ISL_1446509 to 1446510, EPI_ISL_1446512, EPI_ISL_1446514 to 1446515, EPI_ISL_1446517 to 1446518, EPI_ISL_1446520 to 1446521, EPI_ISL_1446523, EPI_ISL_1446525 to 1446526, EPI_ISL_1446528 to 1446529, EPI_ISL_1446531 to 1446532, EPI_ISL_1446534, EPI_ISL_1446536 to 1446537, EPI_ISL_1446539 to 1446540, EPI_ISL_1446542, EPI_ISL_1482537 to 1482538			
see above	Texas Children's Hospital	Texas Children's Microbiome Center	Ila Singh; James Dunn; James Versalovic; Jennifer K. Spinler; Judith R. Campbell; Lea Fasciano; Lisa Tocco; Lucila Marquez; Ruth Ann Luna; Tjin Koy
EPI_ISL_420796 to 420798, EPI_ISL_452105, EPI_ISL_452112, EPI_ISL_452115	Texas DSHS Lab Services	Pathogen Discovery, Respiratory Viruses Branch, Division of Viral Diseases, Centers for Disease Control and Prevention	Alison S. Laufer Halpin; Anna Montmayeur; Anna Uehara; Anne Uehara; Christopher A. Elkins; Clinton R. Paden; Haibin Wang; Jasmine Padilla; Jing Zhang; Justin Lee; Krista Queen; Mary S. Keckler; Rachel Marine; Suxiang Tong; Yan Li; Ying Tao; Zachary Weiner
EPI_ISL_411956	Texas Department of State Health Services	Pathogen Discovery, Respiratory Viruses Branch, Division of Viral Diseases, Centers for Dieases Control and Prevention	Anna Uehara; Brett L. Whitaker; Brian Lynch; Clinton R. Paden; Haibin Wang; Janna' R. Murray; Jing Zhang; Krista Queen; Lijuan Wang; Senthil Kumar K. Sakthivel; Shifaq Kamili; Stephen Lindstrom; Susan I. Gerber; Suxiang Tong; Xiaoyan Lu; Yan Li; Ying Tao
EPI_ISL_515250 to 515261, EPI_ISL_517960 to 517995, EPI_ISL_522351 to 522395, EPI_ISL_522943 to 522977, EPI_ISL_524053 to 524073, EPI_ISL_525763 to 525789, EPI_ISL_527820 to 527855, EPI_ISL_534260 to 534302, EPI_ISL_538275 to 538320, EPI_ISL_541971 to 542011, EPI_ISL_542444 to 542485, EPI_ISL_560926 to 560970, EPI_ISL_569689 to 569734, EPI_ISL_574434 to 574478, EPI_ISL_576281 to 576362, EPI_ISL_576568, EPI_ISL_584123 to 584164, EPI_ISL_590838 to 590876, EPI_ISL_593559 to 593595, EPI_ISL_602210 to 602255, EPI_ISL_610000 to 610047, EPI_ISL_613966 to 614010, EPI_ISL_615122 to 615165			
see above	Texas Department of State Health Services	Texas Department of State Health Services	Anita Pokharel; Bonnie Oh; Cara Akrout; Chun Wang; Chung Wang; Grace Kubin; Jenny Zhang; Maliha Rahman; Mayela Pedrueza; Myong Koag; Rachel Lee; Rashmi Tuladhar
EPI_ISL_1405729 to 1405813, EPI_ISL_1503309 to 1503394	Texas Department of State Health Services (TXDSHS)	Texas Department of State Health Services (TXDSHS)	Anita Pokharel; Bonnie Oh; Chun Wang; Grace Kubin; Jenny Zhang; Lorraine Rodriguez; Maliha Rahman; Mayela Pedrueza; Myong Koag; Rachel Lee; Rashmi Tuladhar
EPI_ISL_594455, EPI_ISL_594464	Texas Department of State Health Services Public Health Laboratory	Pathogen Discovery, Respiratory Viruses Branch, Division of Viral Diseases, Centers for Disease Control and Prevention	Anna Uehara; Clinton Paden; Haibin Wang; Jing Zhang; Julu Bhatnagar; Krista Queen; Suxiang Tong; Yan Li; Ying Tao
EPI_ISL_434707	Thammasat University Hospital	National Institute of Health. Department of medical Sciences, Ministry of Public Health, Thailand	Chittaganpitch; Malinee; Okada; Parnmen; Phuygun; Pilailuk; Siripaporn; Sittiporn; Sunthareeya; Thanadachakul; Thanutsapa; Waicharoen; Warawan; Wongboot
EPI_ISL_1490223, EPI_ISL_1490225 to 1490226, EPI_ISL_1490228, EPI_ISL_1490246 to 1490250, EPI_ISL_1490252, EPI_ISL_1490254, EPI_ISL_1490653, EPI_ISL_1490661, EPI_ISL_1490666, EPI_ISL_1490714, EPI_ISL_1498095, EPI_ISL_1498262, EPI_ISL_1498379			
see above	The Caribbean Public Health Agency	Carrington Lab, Department of PreClinical Sciences, Faculty of Medical Sciences, The University of the West Indies	Adesh Ramsubhag; Arianne Brown-Jordan; Avery Hinds; Chinna Chinnadurai; Christine V. F. Carrington; Christopher Oura; Gabriel Escobar; Jaya Jayaraman; Jerome Foster; Karla Georges; Marsha Ivey; Naresh Nandram; Nikita S. D. Sahadeo; Nuno Faria; Oliver Pybus; Rahul Naidu; Rajini Haraksingh; Risha Singh; Roshan Parasram; Sarah Hill; Stanley Giddings; SueMin Nathaniel; Vernie Ramkissoon
EPI_ISL_412978	The Central Hospital Of Wuhan	Hubei Provincial Center for Disease Control and Prevention	Bin Fang; Bo Yang; Bo Yu; Faxian Zhan; Guojun Ye; Jing Li; Junqiang Xu; Kun Cai; Linlin Liu; Xiang Li; Xiao Yu; Xixiang Huo; Yongzhong Jiang.
EPI_ISL_419210	The Chaim Sheba Medical Center	Israel Institute for Biological Research	Adi Beth-Din; Anat Zvi; Boaz Politi; Dana Stein; Einat Vitner; Gadi Segal; Gili Regev-Yochay; Hadas Tamir; Hagit Achdout; Inbar Cohen-Gihon; Lilach Cherry; Michal Mandelboim; Nir Paran; Ofir Israeli; Ohad Shifman; Oran Erster; Orly Laskar; Sharon Melamed; Shay Weiss; Shmuel C. Shapira; Shmuel Yitzhaki; Tomer Israely; Yfat Yahalom Ronen
EPI_ISL_526129, EPI_ISL_526205, EPI_ISL_544963, EPI_ISL_545002, EPI_ISL_591518, EPI_ISL_593769	The Children's Hospital at Westmead	NSW Health Pathology - Institute of Clinical Pathology and Medical Research; Westmead Hospital; University of Sydney	CIDM-PH et al.
EPI_ISL_450442	The Department of Infectious Disease Prevention and Control, Henan Provincial Center for Disease Control and Prevention	The Department of Infectious Disease Prevention and Control, Henan Provincial Center for Disease Control and Prevention	Hu, X.; Huang; Li, D.; Li, X.; Lu, S.; Wu, B.; X. and Guo, W.
EPI_ISL_482057 to 482068, EPI_ISL_482120 to 482123, EPI_ISL_482125 to 482130	The Department of Microbiology, Torbay and South Devon NHS Foundation Trust	Wellcome Sanger Institute for the COVID-19 Genomics UK (COG-UK) consortium	Amy Hurd; Anthony Mogridge; Cheryl Bailiss; Cordelia Langford; David K. Jackson; Dominic Kwiatkowski; Ewan Harrison; Gary Booth; Helen Brown; Ian Johnston; Jack Howe; John Sillitoe on behalf of the Wellcome Sanger Institute COVID-19 Surveillance Team (http://www.sanger.ac.uk/covid-team); Mel Brown; Michelle Harrison and Alex Alderton; Roberto Amato; Sonia Goncalves; Sophie Lloyd
EPI_ISL_429074 to 429086, EPI_ISL_429088 to 429105	The First Affiliated Hospital of Guangzhou Medical University	BGI-shenzhen & The First Affiliated Hospital of Guangzhou Medical University	; Daxi Wang; Lu Zhang; Wanying Sun; Yanqun Wang; Zhaoyong Zhang et al.
EPI_ISL_457687 to 457698	The First Affiliated Hospital of Guangzhou Medical University, Guangzhou, China	BGI-shenzhen & The First Affiliated Hospital of Guangzhou Medical University	Daxi Wang; Lu Zhang; Wanying Sun; Yanqun Wang; Zhaoyong Zhang et al.
EPI_ISL_1401473 to 1401565, EPI_ISL_1470560 to 1470740, EPI_ISL_1540714 to 1540852	The Jackson Laboratory	The Jackson Laboratory	Adams M; Bergeron D; Kelly K; Li L; Long J; Omerza G; Renzette N; Uvalic J
EPI_ISL_491473	The Medical City	Research Institute for Tropical Medicine	Catalino Demetria; Criselda Bautista; Daria Manalo; Edelwisa Mercado; Francisco Gerardo Polotan; Inez Andrea Medado; Kirstyn Brunker; Ma. Angelica Tujan; Othoniel Jan Onza
EPI_ISL_491092, EPI_ISL_530347 to 530350, EPI_ISL_539493 to 539494, EPI_ISL_539777, EPI_ISL_541082, EPI_ISL_541138, EPI_ISL_541332 to 541334, EPI_ISL_541336 to 541337, EPI_ISL_545580, EPI_ISL_545954, EPI_ISL_545956 to 545957, EPI_ISL_546436, EPI_ISL_546935 to 546936, EPI_ISL_547966 to 547968, EPI_ISL_560408 to 560412, EPI_ISL_568577, EPI_ISL_577623 to 577638, EPI_ISL_584073 to 584082			
see above	The National Institute of Public Health	State Veterinary Institute Prague	A; D; H; J; Jirincova; L; Nagy; Novakova; Trnka; Vecerova
EPI_ISL_471528, EPI_ISL_471530, EPI_ISL_471540, EPI_ISL_471544, EPI_ISL_471547, EPI_ISL_471550, EPI_ISL_471553, EPI_ISL_471555, EPI_ISL_476067			
see above	The National Institute of Public Health	State Veterinary Institute Prague and The National Institute of Public Health	A; D; H; J; Jirincova; L; Nagy; Novakova; Trnka; Vecerova
EPI_ISL_541335	The National Institute of Public Health	Sídlištní 136/24 165 03, Prague Czech Republic	A; D; H; J; Jirincova; L; Nagy; Novakova; Trnka; Vecerova
EPI_ISL_489708 to 489709, EPI_ISL_490112, EPI_ISL_491093 to 491095, EPI_ISL_491117 to 491119, EPI_ISL_513314, EPI_ISL_513511 to 513512	The National Institute of Public Health	The National Institute of Public Health and State Veterinary Institute Prague	A; D; H; J; Jirincova; L; Nagy; Novakova; Trnka; Vecerova
EPI_ISL_414477, EPI_ISL_422636	The National Institute of Public Health Center for Epidemiology and Microbiology	State Veterinary Institute Prague	Alexander Nagy; Dusan Trnka; Helena Jirincova; Jaromira Vecerova; Klara Labska; Ludmila Novakova; Oldrich Bartos; Olga Storkanova
EPI_ISL_434572, EPI_ISL_437519	The National Institute of Public Health Center for Epidemiology and Microbiology	The National Institute of Public Health Center for Epidemiology and Microbiology	Alexander Nagy; Dusan Trnka; Helena Jirincova; Jaromira Vecerova; Ludmila Novakova
EPI_ISL_426379	The National Laboratory of Health, Environment and Food, Maribor, Slovenia	The National Laboratory of Health, Environment and Food, Maribor, Slovenia	Blazun Vosner H.; Duh D.; Hedzet S.; Janezic S.; Mahnic A.; Rupnik M.; Zavrsnik J.
EPI_ISL_1384692 to 1384701	The National Public Health Laboratory	Vilnius University Hospital Santaros Klinikos, Center of Laboratory Medicine	Daniel Naumovas; Dovile Ezerskyte; Gytis Dudas; Ingrida Olendraite; Laimonas Griskevicius; Ligita Raugaite; Mindaugas Stoskus; Monika Katenaite; Rimvydas Norvilas
EPI_ISL_417550 to 417551, EPI_ISL_417553 to 417589, EPI_ISL_417591 to 417617, EPI_ISL_417619 to 417654, EPI_ISL_417673 to 417675, EPI_ISL_417677 to 417876, EPI_ISL_424379 to 424532, EPI_ISL_424552, EPI_ISL_424554 to 424559, EPI_ISL_424563 to 424564, EPI_ISL_424571 to 424593, EPI_ISL_424595 to 424607, EPI_ISL_424610 to 424624			
see above	The National University Hospital of Iceland	deCODE genetics	Agnar Helgason; Alma Moller; Arna B Agustsdottir; Arnaldur Gylfason; Asgeir Sigurdsson; Aslaug Jonasdottir; Berglind Eiriksdottir; Bjarni Thorbjornsson; Brynjar O Jensson; Daniel F Gudbjartsson; Droplaug N Magnusdottir; Elisabet E Gardarsdottir; Emil A Thorarensen; Gardar Sveinbjornsson; Gisli Masson; Gudmundur Georgsson; Gudmundur L Norddahl; Gudrun Sigmundsdottir; Hakon Jonsson; Hilma Holm; Ingileif Jonsdottir; Jona Saemundsdottir; Kamilla S Josefsdottir; Kari Stefansson; Karl G Kristinsson; Kjartan R Gudmundsson; Kristin E Sveinsdottir; Louise le Roux; Maney Sveinsdottir; Olafia S Gretarsdottir; Olafur T Magnusson; Pall Melsted; Patrick Sulem; Run Fridriksdottir; Thora R Gunnarsdottir; Thordur Kristjansson; Thorolfur Gudnason; Unnur Thorsteinsdottir
EPI_ISL_421660, EPI_ISL_427288 to 427291	The Ohio State University	The Ohio State University-James Molecular Lab at Polaris	Dan Jones; Erica Vincent; Huolin Tu; Jason Garee; Joan-Miquel Balada-Llasat; Matt Hunt; Matthew Avenarius; Matthew Hunt; Preeti Panchioli; Preeti

			Pancholi; Rongqin Ren; Sean Caruthers; Weiqiang Zhao; Xiaokang Pan; Xiaokang Zhao
EPI_ISL_421675	The Ohio State University Wexner Medical Center	The Ohio State University James Molecular lab	Dan Jones; Huolin Tu; Jason Garee; Joan-Miquel Balada-Llasat; Matthew Avenarius; Matthew Hunt; Pam Snyder; Preeti Panchioli; Sean Caruthers; Xiaokang Pen
EPI_ISL_509436 to 509443	The Princess Alexandra Hospital	Wellcome Sanger Institute for the COVID-19 Genomics UK (COG-UK) consortium	Claudia McCrea and Alex Alderton; Cordelia Langford; David K. Jackson; Dominic Kwiatkowski; Ewan Harrison; Ian Johnston; Jessica Scott; John Sillitoe on behalf of the Wellcome Sanger Institute COVID-19 Surveillance Team (http://www.sanger.ac.uk/covid-team); Louise Lopez; Lynn Monaghan; Nick Levene; Roberto Amato; Sonia Goncalves
EPI_ISL_429161 to 429163, EPI_ISL_430862, EPI_ISL_430864	The Public Health Agency of Sweden	The Public Health Agency of Sweden	Anna Risberg; Anna-Malin Linde; Karin Tegmark-Wisell; Maria Lind Karlberg; Olov Svartstrom; Oskar Karlsson Lindsjo; Shaman Muradrasoli
EPI_ISL_1520159 to 1520164	The University of Chicago Medical Center	Illinois Department of Public Health - Chicago Lab	Ira Heimler; Vineet K. Dhiman
EPI_ISL_405839, EPI_ISL_406030	The University of Hong Kong - Shenzhen Hospital	Li Ka Shing Faculty of Medicine, The University of Hong Kong	C.C.-Y.; C.K.-M. and Yuen; Cai; Chan; Chen, H.; Cheng; Chu, H.; H.W.; Hui; Ip; J.D.; J.F.-W.; J.P.; K.H.; K.K.-W.; K.Y.; Kok; Liu, J.; Lo; Poon; R.W.-S.; S.K.-F.; To; Tsai; V.C.-C.; V.K.-M.; W.M.; Xing, F.; Yang, J.; Yip; Yuan, S.
EPI_ISL_1497597	The office of Disease Prevention and Control 6, Chonburi	National Institute of Health, Department of Medical Sciences, Ministry of Public Health, Thailand	Hathaikan Tanchai; Natchaya Khiadsang; Nuttida Thongpramul; Pakorn Piromtong; Pilailuk Okada; Ratana Tacharoenmuang; Siripaporn Phuygun; Sittiporn Parnmen; Sunthareeya Waicharoen.; Thanutsapa Thanadachakull
EPI_ISL_412983	Tianmen Center for Disease Control and Prevention	Hubei Provincial Center for Disease Control and Prevention	Bin Fang; Bo Yang; Bo Yu; Faxian Zhan; Guojun Ye; Jing Li; Junqiang Xu; Kun Cai; Linlin Liu; Xiang Li; Xiao Yu; Xierong Li; Xixiang Huo; Yangyang Tao; YiFa Zhu; Yongzhong Jiang.
EPI_ISL_576385	Tirta Medical Center Angsana, Banjarmasin Kalsel	National Institute of Health Research and Development	AA; HA; HD; Ikawati; KD; KNA; Nugraha; Paisal; Pangesti; Pawestri; Puspa; Setiawaty; Soekarso; Subangkit; T; V
EPI_ISL_480030 to 480041, EPI_ISL_480073	Tochigi Prefectural Institute of Public Health and Environmental Science	Pathogen Genomics Center, National Institute of Infectious Diseases	Ako Nakajima; Hajime Kamiya; Kentaro Itokawa; Makoto Kuroda; Masanori Hashino; Motoi Suzuki; Rina Tanaka; Tsuyoshi Sekizuka
EPI_ISL_479886 to 479895, EPI_ISL_479959 to 479965, EPI_ISL_480227, EPI_ISL_1430682	Tokyo Metropolitan Institute of Public Health	Pathogen Genomics Center, National Institute of Infectious Diseases	Chikako Shimokawa; Eunsil Park; Hajime Kamiya; Kenji Sadamasu; Kentaro Itokawa; Makoto Kuroda; Mami Nagashima; Masanori Hashino; Motoi Suzuki; Rina Tanaka; Takashi Chiba; Tsuguto Fujimoto; Tsuyoshi Sekizuka; Yasutaka Hoshino; Yoshihiro Kaku
EPI_ISL_463970 to 463994, EPI_ISL_586267 to 586428, EPI_ISL_586430 to 586438, EPI_ISL_586440 to 586449, EPI_ISL_586452 to 586453, EPI_ISL_586455 to 586456, EPI_ISL_586458 to 586461, EPI_ISL_586463, EPI_ISL_586466 to 586508, EPI_ISL_591100 to 591141, EPI_ISL_591143, EPI_ISL_591148, EPI_ISL_591150, EPI_ISL_591152, EPI_ISL_591154 to 591160, EPI_ISL_591164 to 591166, EPI_ISL_591168 to 591170, EPI_ISL_591175 to 591212, EPI_ISL_591216, EPI_ISL_591220, EPI_ISL_591222 to 591225, EPI_ISL_591237 to 591238, EPI_ISL_591240, EPI_ISL_591245 to 591247, EPI_ISL_591249, EPI_ISL_591253 to 591269			
see above	Toronto Invasive Bacterial Diseases Network	McMaster University	Ahmed Draia; Allison McGeer; Andrew G. McArthur; Angel Li; Emily Panousis; Hooman Derakhshani; Jalees Nasir; Kuganya Nirmalarajah; Michael Surette; Patryk Aftanas; Samira Mubareka
EPI_ISL_480196 to 480203, EPI_ISL_1429639, EPI_ISL_1430561 to 1430569, EPI_ISL_1431611, EPI_ISL_1431805, EPI_ISL_1432005, EPI_ISL_1432931, EPI_ISL_1432933, EPI_ISL_1432935, EPI_ISL_1432937 to 1432938, EPI_ISL_1432940, EPI_ISL_1432942			
see above	Toyama Institute of Health	Pathogen Genomics Center, National Institute of Infectious Diseases	Hajime Kamiya; Kazunori Oishi; Kentaro Itokawa; Makoto Kuroda; Masae Itamochi; Masanori Hashino; Motoi Suzuki; Rina Tanaka; Tsuyoshi Sekizuka
EPI_ISL_455588	Trang Hospital	National Institute of Health. Department of medical Sciences, Ministry of Public Health, Thailand	Chittaganpitch; Malinee; Okada; Parnmen; Phuygun; Pilailuk; Siripaporn; Sittiporn; Sunthareeya; Thanadachakul; Thanutsapa; Waicharoen; Warawan; Wongboot
EPI_ISL_508489 to 508509	Translational Health Science and Technology Institute	National Institute of Biomedical Genomics	Anbalagan Ananthraj; Arindam Maitra; Gagandeep Kang; Guruprasad Medigeshi; Imran Khan; Madhu Pareek; Saumitra Das; Sharanabasava Patil
EPI_ISL_511942 to 511944	Translational Health Science and Technology Institute	National Institute of Biomedical Genomics - DBT's PAN-INDIA 1000 SARS--CoV-2 RNA Genome Sequencing Consortium	Anbalagan Ananthraj; Arindam Maitra; Gagandeep Kang; Guruprasad Medigeshi; Imran Khan; Madhu Pareek; Saumitra Das; Sharanabasava Patil
EPI_ISL_454858 to 454867, EPI_ISL_528382 to 528385, EPI_ISL_1532786 to 1532791	Translational Health Science and Technology Institute -ESIC medical college and hospital, Faridabad	THSTI Bioassay laboratory	Akshay Kanakan; Anil Kumar Pandey; Anil Pandey; Asim Das; Guruprasad R Medigeshi; Guruprasad R. Medigeshi; Janani Srinivasa Vasudevan; Jigme Wangchuk; Naseem Ahmed Khan; Neha Jha; Priyanka Mehta; Rajesh Pandey; Ranjeet Maurya; Saurabh Kumar
EPI_ISL_538233 to 538264, EPI_ISL_542935 to 543006, EPI_ISL_560325 to 560362, EPI_ISL_582148 to 582203, EPI_ISL_582205 to 582219, EPI_ISL_610251 to 610252, EPI_ISL_610254 to 610267, EPI_ISL_610282			
see above	TriCore Reference Laboratories	Center for Global Health, University of New Mexico Health Sciences Center	Darrell Dinwiddie; Daryl Domman; Joseph Hicks; Kurt Schwalm; Michael Edwards; Twila Kunde
EPI_ISL_1530946 to 1530947	TriCore Reference Laboratories	New Mexico Department of Health Scientific Laboratory	Anastacia Griego-Fisher; D'eldra Malone; Ellie Johnson; Jennifer Benoit
EPI_ISL_1400964 to 1400966	TriCore Reference Laboratory Inc.	New Mexico Department of Health Scientific Laboratory	Anastacia Griego-Fisher; D'eldra Malone; Ellie Johnson; Jennifer Benoit
EPI_ISL_1499113, EPI_ISL_1499115	Trinidad Public Health Laboratory	Carrington Lab, Department of PreClinical Sciences, Faculty of Medical Sciences, The University of the West Indies	Adesh Ramsubhag; Arianne Brown-Jordan; Avery Hinds; Chinna Chinnadurai; Christine V. F. Carrington; Christopher Oura; Gabriel Escobar; Jaya Jayaraman; Jerome Foster; Karla Georges; Marsha Ivey; Naresh Nandram; Nikita S. D. Sahadeo; Nuno Faria; Oliver Pybus; Rahul Naidu; Rajini Haraksingh; Risha Singh; Roshan Parasram; Sarah Hill; Stanley Giddings; SueMin Nathaniel; Vernie Ramkissoon
EPI_ISL_434653, EPI_ISL_434656, EPI_ISL_475518	Trollbackens VC	The Public Health Agency of Sweden	Amelie Holmqvist; Anna Risberg; Anna-Malin Linde; Karin Tegmark-Wisell; Maria Lind Karlberg; Mattias Haukland; Mia Brytting; Olov Svartstrom; Oskar Karlsson Lindsjo; Reza Advani; Sandra Broddesson; Theresa Enkirch
EPI_ISL_539814, EPI_ISL_539818 to 539819	Tuen Mun Hospital	Hong Kong Department of Health	Alan K.L. Tsang; Dominic N.C. Tsang; Edman T.K. Lam; Peter C.W. Yip; Rickjason C.W. Chan
EPI_ISL_583171 to 583172	Tulare County Public Health Lab	Chan-Zuckerberg Biohub	CZB Cliahub Consortium
EPI_ISL_548363, EPI_ISL_548599, EPI_ISL_548626	Tuolumne County Public Health	Chan-Zuckerberg Biohub	CZB Cliahub Consortium
EPI_ISL_1448024, EPI_ISL_1448510, EPI_ISL_1470414	U.O. Microbiologia, Laboratorio Unico Centro Servizi - AUSL della Romagna	U.O. Microbiologia, Laboratorio Unico Centro Servizi - AUSL della Romagna	Giorgio Dirani; Silvia Zannoli; Vittorio Sambri
EPI_ISL_444610	U.S. Naval Medical Research Center Biological Defense Research Directorate	U.S. Naval Medical Research Center Biological Defense Research Directorate	A.C.; Balansy-Ames; C.A.; Cer; Christy; E.M.; Hollis; K.A.; L.J.; Long; M.S.; Myers; N.C. and Bishop-Lilly; Pan; Paskey; Pena-Gomez, D.; R.W.; R.Z.; Voegtly
EPI_ISL_1498097 to 1498120	UAB "Baltic Medics"	Institute of Biotechnology, Life Sciences Center, Vilnius University	Albertas Timinskas; Alma Gedvilaite; Danguole Ziogiene; Emilija Vasiliunaite; Milda Norkiene
EPI_ISL_1384641 to 1384664, EPI_ISL_1398395 to 1398430, EPI_ISL_1510088, EPI_ISL_1510092, EPI_ISL_1510101 to 1510110, EPI_ISL_1510193	UAB InMedica	Vilnius University Hospital Santaros Klinikos, Center of Laboratory Medicine	Daniel Naumovas; Dovile Ezerskyte; Gytis Dudas; Ingrida Olendraite; Laimonas Griskevicius; Ligita Raugaite; Mindaugas Stoskus; Monika Katenaite; Rimvydas Norvilas
EPI_ISL_1482354 to 1482392	UAB Medicina practica laboratorija	Lithuanian University of Health Sciences	Arnoldas Pautienius; Dovydas Gecys; Gediminas Alzbutas; Kamile Tamusauskaite; Lukas Zemaitis; Vaiva Lesauskaite
EPI_ISL_1510094, EPI_ISL_1510119 to 1510120	UAB SK Impeks Medicinos diagnostikos centras	Vilnius University Hospital Santaros Klinikos, Center of Laboratory Medicine	Daniel Naumovas; Dovile Ezerskyte; Gytis Dudas; Ingrida Olendraite; Laimonas Griskevicius; Ligita Raugaite; Mindaugas Stoskus; Monika Katenaite; Rimvydas Norvilas
EPI_ISL_1468455	UBS 02 Jardim Toselar Birigui	Instituto Adolfo Lutz, Interdiciplinary Procedures Center, Strategic Laboratory	Caio Vinicius Dias Lopes; Claudia Regina Gonçalves; Claudio Tavares Sacchi; Erica Valessa Ramos Gomes; Karoline Rodrigues Campos
EPI_ISL_1445206, EPI_ISL_1445208	UBS DARCY ALVES E ROBALINHO	Instituto Butantan / Mendelics	Antonio Jorge Martins; Bibiana Santos; Claudia Renata dos Santos Barros; David Schlesinger; Debora Botequio Moretti; Dimas Tadeu Covas; Elaine Cristina Marqueze; Elaine Vieira dos Santos; Erika Freitas; Evandra Strazza Rodrigues; Flavia Aburjaile; José Salvatore Leister Patané; João Paulo Kitajima; Luiz Carlos Junior de Alcantara; Maria Carolina Elias; Marta Giovanetti; Rafael dos Santos Bezerra; Raul Machado Neto; Ricardo Haddad; Rodrigo Tocantins Calado.; Sandra Coccuzzo Sampaio; Simone Kashima; Svetoslav Nanev Slavov; Vagner Fonseca; Vincent Louis Viala
EPI_ISL_1468456 to 1468457	UBS Dr Alfredo Dantas de Souza Umuarama	Instituto Adolfo Lutz, Interdiciplinary Procedures Center, Strategic Laboratory	Caio Vinicius Dias Lopes; Claudia Regina Gonçalves; Claudio Tavares Sacchi; Erica Valessa Ramos Gomes; Karoline Rodrigues Campos

EPI_ISL_1445217, EPI_ISL_1445219 to 1445220, EPI_ISL_1445227	UBS EMILIA COSME CERQUEIRA	Instituto Butantan / Mendelics	Antonio Jorge Martins; Bibiana Santos; Claudia Renata dos Santos Barros; David Schlesinger; Debora Botequio Moretti; Dimas Tadeu Covas; Elaine Cristina Marqueze; Elaine Vieira dos Santos; Erika Freitas; Evandra Strazza Rodrigues; Flavia Aburjaile; José Salvatore Leister Patané; João Paulo Kitajima; Luiz Carlos Junior de Alcantara; Maria Carolina Elias; Marta Giovanetti; Rafael dos Santos Bezerra; Raul Machado Neto; Ricardo Haddad; Rodrigo Tocantins Calado.; Sandra Coccuzzo Sampaio; Simone Kashima; Svetoslav Nanev Slavov; Vagner Fonseca; Vincent Louis Viala
EPI_ISL_1445205	UBS FRANCISCA LIMA DE LIRA	Instituto Butantan / Mendelics	Antonio Jorge Martins; Bibiana Santos; Claudia Renata dos Santos Barros; David Schlesinger; Debora Botequio Moretti; Dimas Tadeu Covas; Elaine Cristina Marqueze; Elaine Vieira dos Santos; Erika Freitas; Evandra Strazza Rodrigues; Flavia Aburjaile; José Salvatore Leister Patané; João Paulo Kitajima; Luiz Carlos Junior de Alcantara; Maria Carolina Elias; Marta Giovanetti; Rafael dos Santos Bezerra; Raul Machado Neto; Ricardo Haddad; Rodrigo Tocantins Calado.; Sandra Coccuzzo Sampaio; Simone Kashima; Svetoslav Nanev Slavov; Vagner Fonseca; Vincent Louis Viala
EPI_ISL_1445215 to 1445216, EPI_ISL_1445221 to 1445223	UBS HELENA MARREY	Instituto Butantan / Mendelics	Antonio Jorge Martins; Bibiana Santos; Claudia Renata dos Santos Barros; David Schlesinger; Debora Botequio Moretti; Dimas Tadeu Covas; Elaine Cristina Marqueze; Elaine Vieira dos Santos; Erika Freitas; Evandra Strazza Rodrigues; Flavia Aburjaile; José Salvatore Leister Patané; João Paulo Kitajima; Luiz Carlos Junior de Alcantara; Maria Carolina Elias; Marta Giovanetti; Rafael dos Santos Bezerra; Raul Machado Neto; Ricardo Haddad; Rodrigo Tocantins Calado.; Sandra Coccuzzo Sampaio; Simone Kashima; Svetoslav Nanev Slavov; Vagner Fonseca; Vincent Louis Viala
EPI_ISL_1520114	UBS III de Pariquera Acu Pariquera Acu	Instituto Adolfo Lutz, Interdiciplinary Procedures Center, Strategic Laboratory	Caio Vinicius Dias Lopes; Claudia Regina Gonçalves; Claudio Tavares Sacchi; Erica Valessa Ramos Gomes; Karoline Rodrigues Campos
EPI_ISL_1445212, EPI_ISL_1445228	UBS IRMA AGUEDA MARIA JAIME	Instituto Butantan / Mendelics	Antonio Jorge Martins; Bibiana Santos; Claudia Renata dos Santos Barros; David Schlesinger; Debora Botequio Moretti; Dimas Tadeu Covas; Elaine Cristina Marqueze; Elaine Vieira dos Santos; Erika Freitas; Evandra Strazza Rodrigues; Flavia Aburjaile; José Salvatore Leister Patané; João Paulo Kitajima; Luiz Carlos Junior de Alcantara; Maria Carolina Elias; Marta Giovanetti; Rafael dos Santos Bezerra; Raul Machado Neto; Ricardo Haddad; Rodrigo Tocantins Calado.; Sandra Coccuzzo Sampaio; Simone Kashima; Svetoslav Nanev Slavov; Vagner Fonseca; Vincent Louis Viala
EPI_ISL_1468451	UBS IV Guararapes	Instituto Adolfo Lutz, Interdiciplinary Procedures Center, Strategic Laboratory	Caio Vinicius Dias Lopes; Claudia Regina Gonçalves; Claudio Tavares Sacchi; Erica Valessa Ramos Gomes; Karoline Rodrigues Campos
EPI_ISL_1445214	UBS JOSE HILARIO DOS SANTOS	Instituto Butantan / Mendelics	Antonio Jorge Martins; Bibiana Santos; Claudia Renata dos Santos Barros; David Schlesinger; Debora Botequio Moretti; Dimas Tadeu Covas; Elaine Cristina Marqueze; Elaine Vieira dos Santos; Erika Freitas; Evandra Strazza Rodrigues; Flavia Aburjaile; José Salvatore Leister Patané; João Paulo Kitajima; Luiz Carlos Junior de Alcantara; Maria Carolina Elias; Marta Giovanetti; Rafael dos Santos Bezerra; Raul Machado Neto; Ricardo Haddad; Rodrigo Tocantins Calado.; Sandra Coccuzzo Sampaio; Simone Kashima; Svetoslav Nanev Slavov; Vagner Fonseca; Vincent Louis Viala
EPI_ISL_1445210 to 1445211	UBS JOSE SABINO FERREIRA	Instituto Butantan / Mendelics	Antonio Jorge Martins; Bibiana Santos; Claudia Renata dos Santos Barros; David Schlesinger; Debora Botequio Moretti; Dimas Tadeu Covas; Elaine Cristina Marqueze; Elaine Vieira dos Santos; Erika Freitas; Evandra Strazza Rodrigues; Flavia Aburjaile; José Salvatore Leister Patané; João Paulo Kitajima; Luiz Carlos Junior de Alcantara; Maria Carolina Elias; Marta Giovanetti; Rafael dos Santos Bezerra; Raul Machado Neto; Ricardo Haddad; Rodrigo Tocantins Calado.; Sandra Coccuzzo Sampaio; Simone Kashima; Svetoslav Nanev Slavov; Vagner Fonseca; Vincent Louis Viala
EPI_ISL_1445209, EPI_ISL_1445213	UBS OTACILIO FIRMINO LOPES	Instituto Butantan / Mendelics	Antonio Jorge Martins; Bibiana Santos; Claudia Renata dos Santos Barros; David Schlesinger; Debora Botequio Moretti; Dimas Tadeu Covas; Elaine Cristina Marqueze; Elaine Vieira dos Santos; Erika Freitas; Evandra Strazza Rodrigues; Flavia Aburjaile; José Salvatore Leister Patané; João Paulo Kitajima; Luiz Carlos Junior de Alcantara; Maria Carolina Elias; Marta Giovanetti; Rafael dos Santos Bezerra; Raul Machado Neto; Ricardo Haddad; Rodrigo Tocantins Calado.; Sandra Coccuzzo Sampaio; Simone Kashima; Svetoslav Nanev Slavov; Vagner Fonseca; Vincent Louis Viala
EPI_ISL_1445141	UBS SALTO DE SAO JOSE	Instituto Butantan / Mendelics	Antonio Jorge Martins; Bibiana Santos; Claudia Renata dos Santos Barros; David Schlesinger; Debora Botequio Moretti; Dimas Tadeu Covas; Elaine Cristina Marqueze; Elaine Vieira dos Santos; Erika Freitas; Evandra Strazza Rodrigues; Flavia Aburjaile; José Salvatore Leister Patané; João Paulo Kitajima; Luiz Carlos Junior de Alcantara; Maria Carolina Elias; Marta Giovanetti; Rafael dos Santos Bezerra; Raul Machado Neto; Ricardo Haddad; Rodrigo Tocantins Calado.; Sandra Coccuzzo Sampaio; Simone Kashima; Svetoslav Nanev Slavov; Vagner Fonseca; Vincent Louis Viala
EPI_ISL_1445196	UBS SAO JUDAS	Instituto Butantan / Mendelics	Antonio Jorge Martins; Bibiana Santos; Claudia Renata dos Santos Barros; David Schlesinger; Debora Botequio Moretti; Dimas Tadeu Covas; Elaine Cristina Marqueze; Elaine Vieira dos Santos; Erika Freitas; Evandra Strazza Rodrigues; Flavia Aburjaile; José Salvatore Leister Patané; João Paulo Kitajima; Luiz Carlos Junior de Alcantara; Maria Carolina Elias; Marta Giovanetti; Rafael dos Santos Bezerra; Raul Machado Neto; Ricardo Haddad; Rodrigo Tocantins Calado.; Sandra Coccuzzo Sampaio; Simone Kashima; Svetoslav Nanev Slavov; Vagner Fonseca; Vincent Louis Viala
EPI_ISL_1445218, EPI_ISL_1445226	UBS SYLVIO JOAO L DE LUCIA	Instituto Butantan / Mendelics	Antonio Jorge Martins; Bibiana Santos; Claudia Renata dos Santos Barros; David Schlesinger; Debora Botequio Moretti; Dimas Tadeu Covas; Elaine Cristina Marqueze; Elaine Vieira dos Santos; Erika Freitas; Evandra Strazza Rodrigues; Flavia Aburjaile; José Salvatore Leister Patané; João Paulo Kitajima; Luiz Carlos Junior de Alcantara; Maria Carolina Elias; Marta Giovanetti; Rafael dos Santos Bezerra; Raul Machado Neto; Ricardo Haddad; Rodrigo Tocantins Calado.; Sandra Coccuzzo Sampaio; Simone Kashima; Svetoslav Nanev Slavov; Vagner Fonseca; Vincent Louis Viala
EPI_ISL_523963	UBS Vila Silvia	Instituto Adolfo Lutz, Interdiciplinary Procedures Center, Strategic Laboratory	Claudia Regina Gonçalves; Claudio Tavares Sacchi; Erica Valessa Ramos Gomes
EPI_ISL_1509639, EPI_ISL_1509720	UBS de Auriflama	Instituto Adolfo Lutz, Interdiciplinary Procedures Center, Strategic Laboratory	Caio Vinicius Dias Lopes; Claudia Regina Gonçalves; Claudio Tavares Sacchi; Erica Valessa Ramos Gomes; Karoline Rodrigues Campos; Leonardo Jose Tadeu de Araujo
EPI_ISL_471648	UBS e Pronto Socorro Jd. Jacira	Instituto Adolfo Lutz, Interdiciplinary Procedures Center, Strategic Laboratory	Claudia Regina Gonçalves; Claudio Tavares Sacchi; Erica Valessa Ramos Gomes
EPI_ISL_445094 to 445117, EPI_ISL_483165 to 483530	UC San Diego Center for Advanced Laboratory Medicine	Andersen lab at Scripps Research	Allison Smither; Antoinette Bell; Arnaud Drouin; Dahlene Fusco; Gilberto Sabino-Santos; Ji H Shin; Kaylynn Genemaras; Lilia Melnik; Patricia Snarski; Robert Garry with SEARCH Alliance San Diego; SEARCH Alliance San Diego with David Pride
EPI_ISL_417432	UCD National Virus Reference Laboratory	Public Health Wales Microbiology Cardiff	; Catherine Dempsey; Cillian F De Gascun; Michael Prentice; Olive Murphy
EPI_ISL_414487, EPI_ISL_414584 to 414587, EPI_ISL_418516, EPI_ISL_418548, EPI_ISL_418580 to 418584	UCD National Virus Reference Laboratory	UCD National Virus Reference Laboratory	Alison Murphy; Brendan Loftus; Cillian F De Gascun; Gabriel Gonzalez; Jeff Connell; Jonathan Dean; Ken Wolfe; Kevin Byrne; Michael Carr; Suzie Coughlan
EPI_ISL_1393643 to 1393650, EPI_ISL_1538532, EPI_ISL_1539037	UCK	1. National Institute of Public Health - National Institute of Hygiene; 2. Eurofins Genomics Europe Sequencing GmbH	ECDC COVID-19 WGS support team; Eurofins Genomics Europe Sequencing Team; Gierczyki Rafa; Sadkowska-Todys Magorzata; Wokowicz Tomasz; Zacharczuk Katarzyna
EPI_ISL_1497541, EPI_ISL_1506114	UCLA Clinical Micro Lab	Los Angeles County PHL	P. Hemarajata et al.
EPI_ISL_537467 to 537607	UCLA Pathology Clinical Microbiology Lab	Kruglyak Lab	Guo et al.
EPI_ISL_1402586 to 1402654, EPI_ISL_1405050 to 1405060, EPI_ISL_1405599 to 1405679, EPI_ISL_1405814 to 1405897, EPI_ISL_1469120 to 1469121, EPI_ISL_1469852 to 1469936	UCSC Genomics Institute	UCSC Genomics Institute	A. Marm Kilpatrick; Angie Hinrichs; Beth Shapiro; Bryan Thornlow; David Haussler; Eric Beraut; Hugh Olsen; Ikenna Anigbogu; Isabel Bjork; Jakob McBroome; Jeremy Sanford; Joshua Kapp; Mark Akeson; Maximilian Haeussler; Michael Stone; Miten Jain; Molly Cassatt-Johnstone; Namrita Dhillon; Russell Corbett-Detig; Terren Chang; Yatish Turakhia
EPI_ISL_417931 to 417933, EPI_ISL_417935, EPI_ISL_417937 to 417939, EPI_ISL_428990 to 429073, EPI_ISL_430791 to 430792, EPI_ISL_444051 to 444078, EPI_ISL_445169 to 445182, EPI_ISL_454614 to 454634, EPI_ISL_486339 to 486365, EPI_ISL_513883 to 513910, EPI_ISL_516723 to 516747, EPI_ISL_548505, EPI_ISL_548513, EPI_ISL_548522, EPI_ISL_548555, EPI_ISL_548571, EPI_ISL_583199 to 583231			
see above	UCSF Clinical Microbiology Laboratory	Chan-Zuckerberg Biohub	Amy Kistler; Angela Detweiler; Angela Pisco; CZB Cliahub Consortium; Chaz Langelier; Chunyu Zhao; David Dynerman; G. Renuka Kumar; Gloria Castaneda; Jack Kamm; Josh Batson; Lucy Li; Lusajo Mwakibete; Maira Phelps; Michelle Tan; Norma Neff; Olga Botvinnik; Samantha Hao; Shaun Arevalo; Steve Miller
EPI_ISL_450232 to 450240	UCSF Clinical Microbiology Laboratory	Chiu Laboratory, University of California, San Francisco	Scot Federman; Wei Gu; Xianding Deng; and Charles Y. Chiu
EPI_ISL_1528885	UHC	WVU and Marshall University Combined Genomics Core Facilities	"James Denvir; Peter Perrotta; Peter Stoilov; Ryan Percifield"; Wesley Kimble
EPI_ISL_584440 to 584590	UHCW / University of Warwick	COVID-19 Genomics UK (COG-UK) Consortium	Chrystala Constantinidou; Dimitris Grammatopoulos; Grace Taylor-Joyce; Hannah Elizabeth Bridgewater; Jeff Cheng; Laura Baxter; Lucy Frost; Meera Unnikrishnan; Paul Brown; Richard Stark; Sarojini Pandey; Sascha Ott; Tauqeer Alam

EPI_ISL_460635	UHCW Pathology / University of Warwick	University of Warwick, for the COVID-19 Genomics (COG) UK Consortium	Chrystala Constantinidou; Dimitris Grammatopoulos; Grace Taylor-Joyce; Hannah Elizabeth Bridgewater; Jeff Cheng; Laura Baxter; Lucy Frost; Meera Unnikrishnan; Paul Brown; Richard Stark; Sarojini Pandey; Sascha Ott; Tauqeer Alam
EPI_ISL_1524386 to 1524387, EPI_ISL_1524430 to 1524432, EPI_ISL_1524452, EPI_ISL_1524648 to 1524649, EPI_ISL_1524651 to 1524653	ULSS 1 Dolomiti	Istituto Zooprofilattico Sperimentale delle Venezie	Adelaide Milani; Alessia Schivo; Alice Fusaro; Ambra Pastori; Annalisa Salviato; Antonia Ricci; Calogero Terregino; Edoardo Giussani; Elisa Palumbo; Erika Giorgia Quaranta; Isabella Monne; Luca Tassoni
EPI_ISL_1524470 to 1524483	ULSS 1 Dolomiti	Istituto di Genomica Applicata; Istituto Zooprofilattico Sperimentale delle Venezie	Alessia Schivo; Alice Fusaro; Ambra Pastori; Annalisa Salviato; Antonia Ricci; Calogero Terregino; Davide Scaglione; Edoardo Giussani; Eleonora Paparelli; Erika Giorgia Quaranta; Francesca Bruno; Gabriele Magris; Irena Jurman; Isabella Monne; Luca Tassoni; Maria Varotto; Michele Morgante; Silvia Ormelli; Valeria D'Amico; Vera Vendramin
EPI_ISL_1524696 to 1524697	ULSS 2 Marca Trevigiana	Istituto Zooprofilattico Sperimentale delle Venezie	Adelaide Milani; Alessia Schivo; Alice Fusaro; Ambra Pastori; Annalisa Salviato; Antonia Ricci; Calogero Terregino; Edoardo Giussani; Elisa Palumbo; Erika Giorgia Quaranta; Isabella Monne; Luca Tassoni
EPI_ISL_1524583 to 1524600	ULSS 2 Marca Trevigiana	Istituto di Genomica Applicata; Istituto Zooprofilattico Sperimentale delle Venezie	Alessia Schivo; Alice Fusaro; Ambra Pastori; Annalisa Salviato; Antonia Ricci; Calogero Terregino; Davide Scaglione; Edoardo Giussani; Eleonora Paparelli; Erika Giorgia Quaranta; Francesca Bruno; Gabriele Magris; Irena Jurman; Isabella Monne; Luca Tassoni; Maria Varotto; Michele Morgante; Silvia Ormelli; Valeria D'Amico; Vera Vendramin
EPI_ISL_1524437 to 1524445, EPI_ISL_1524451, EPI_ISL_1524457 to 1524461, EPI_ISL_1524676 to 1524678	ULSS 3 Venezia	Istituto Zooprofilattico Sperimentale delle Venezie	Adelaide Milani; Alessia Schivo; Alice Fusaro; Ambra Pastori; Annalisa Salviato; Antonia Ricci; Calogero Terregino; Edoardo Giussani; Elisa Palumbo; Erika Giorgia Quaranta; Isabella Monne; Luca Tassoni
EPI_ISL_1524601 to 1524619	ULSS 3 Venezia	Istituto di Genomica Applicata; Istituto Zooprofilattico Sperimentale delle Venezie	Alessia Schivo; Alice Fusaro; Ambra Pastori; Annalisa Salviato; Antonia Ricci; Calogero Terregino; Davide Scaglione; Edoardo Giussani; Eleonora Paparelli; Erika Giorgia Quaranta; Francesca Bruno; Gabriele Magris; Irena Jurman; Isabella Monne; Luca Tassoni; Maria Varotto; Michele Morgante; Silvia Ormelli; Valeria D'Amico; Vera Vendramin
EPI_ISL_1524453, EPI_ISL_1524664 to 1524669	ULSS 4 Veneto Orientale	Istituto Zooprofilattico Sperimentale delle Venezie	Adelaide Milani; Alessia Schivo; Alice Fusaro; Ambra Pastori; Annalisa Salviato; Antonia Ricci; Calogero Terregino; Edoardo Giussani; Elisa Palumbo; Erika Giorgia Quaranta; Isabella Monne; Luca Tassoni
EPI_ISL_1524464, EPI_ISL_1524507 to 1524513	ULSS 4 Veneto Orientale	Istituto di Genomica Applicata; Istituto Zooprofilattico Sperimentale delle Venezie	Alessia Schivo; Alice Fusaro; Ambra Pastori; Annalisa Salviato; Antonia Ricci; Calogero Terregino; Davide Scaglione; Edoardo Giussani; Eleonora Paparelli; Erika Giorgia Quaranta; Francesca Bruno; Gabriele Magris; Irena Jurman; Isabella Monne; Luca Tassoni; Maria Varotto; Michele Morgante; Silvia Ormelli; Valeria D'Amico; Vera Vendramin
EPI_ISL_1524404 to 1524405, EPI_ISL_1524436, EPI_ISL_1524454, EPI_ISL_1524654 to 1524659	ULSS 5 Polesana	Istituto Zooprofilattico Sperimentale delle Venezie	Adelaide Milani; Alessia Schivo; Alice Fusaro; Ambra Pastori; Annalisa Salviato; Antonia Ricci; Calogero Terregino; Edoardo Giussani; Elisa Palumbo; Erika Giorgia Quaranta; Isabella Monne; Luca Tassoni
EPI_ISL_1524484 to 1524497	ULSS 5 Polesana	Istituto di Genomica Applicata; Istituto Zooprofilattico Sperimentale delle Venezie	Alessia Schivo; Alice Fusaro; Ambra Pastori; Annalisa Salviato; Antonia Ricci; Calogero Terregino; Davide Scaglione; Edoardo Giussani; Eleonora Paparelli; Erika Giorgia Quaranta; Francesca Bruno; Gabriele Magris; Irena Jurman; Isabella Monne; Luca Tassoni; Maria Varotto; Michele Morgante; Silvia Ormelli; Valeria D'Amico; Vera Vendramin
EPI_ISL_1524385, EPI_ISL_1524388 to 1524392, EPI_ISL_1524412 to 1524418, EPI_ISL_1524424 to 1524429, EPI_ISL_1524446 to 1524447, EPI_ISL_1524631 to 1524646	ULSS 6 Euganea	Istituto Zooprofilattico Sperimentale delle Venezie	Adelaide Milani; Alessia Schivo; Alice Fusaro; Ambra Pastori; Annalisa Salviato; Antonia Ricci; Calogero Terregino; Edoardo Giussani; Elisa Palumbo; Erika Giorgia Quaranta; Isabella Monne; Luca Tassoni
EPI_ISL_1524548 to 1524560	ULSS 6 Euganea	Istituto di Genomica Applicata; Istituto Zooprofilattico Sperimentale delle Venezie	Alessia Schivo; Alice Fusaro; Ambra Pastori; Annalisa Salviato; Antonia Ricci; Calogero Terregino; Davide Scaglione; Edoardo Giussani; Eleonora Paparelli; Erika Giorgia Quaranta; Francesca Bruno; Gabriele Magris; Irena Jurman; Isabella Monne; Luca Tassoni; Maria Varotto; Michele Morgante; Silvia Ormelli; Valeria D'Amico; Vera Vendramin
EPI_ISL_1524448 to 1524449, EPI_ISL_1524462 to 1524463, EPI_ISL_1524670 to 1524672	ULSS 7 Pedemontana - Distretto 1	Istituto Zooprofilattico Sperimentale delle Venezie	Adelaide Milani; Alessia Schivo; Alice Fusaro; Ambra Pastori; Annalisa Salviato; Antonia Ricci; Calogero Terregino; Edoardo Giussani; Elisa Palumbo; Erika Giorgia Quaranta; Isabella Monne; Luca Tassoni
EPI_ISL_1524514 to 1524519	ULSS 7 Pedemontana - Distretto 1	Istituto di Genomica Applicata; Istituto Zooprofilattico Sperimentale delle Venezie	Alessia Schivo; Alice Fusaro; Ambra Pastori; Annalisa Salviato; Antonia Ricci; Calogero Terregino; Davide Scaglione; Edoardo Giussani; Eleonora Paparelli; Erika Giorgia Quaranta; Francesca Bruno; Gabriele Magris; Irena Jurman; Isabella Monne; Luca Tassoni; Maria Varotto; Michele Morgante; Silvia Ormelli; Valeria D'Amico; Vera Vendramin
EPI_ISL_1524419 to 1524423, EPI_ISL_1524673 to 1524675	ULSS 7 Pedemontana - Distretto 2	Istituto Zooprofilattico Sperimentale delle Venezie	Adelaide Milani; Alessia Schivo; Alice Fusaro; Ambra Pastori; Annalisa Salviato; Antonia Ricci; Calogero Terregino; Edoardo Giussani; Elisa Palumbo; Erika Giorgia Quaranta; Isabella Monne; Luca Tassoni
EPI_ISL_1524465, EPI_ISL_1524520 to 1524524	ULSS 7 Pedemontana - Distretto 2	Istituto di Genomica Applicata; Istituto Zooprofilattico Sperimentale delle Venezie	Alessia Schivo; Alice Fusaro; Ambra Pastori; Annalisa Salviato; Antonia Ricci; Calogero Terregino; Davide Scaglione; Edoardo Giussani; Eleonora Paparelli; Erika Giorgia Quaranta; Francesca Bruno; Gabriele Magris; Irena Jurman; Isabella Monne; Luca Tassoni; Maria Varotto; Michele Morgante; Silvia Ormelli; Valeria D'Amico; Vera Vendramin
EPI_ISL_1524401 to 1524403, EPI_ISL_1524433 to 1524435, EPI_ISL_1524450, EPI_ISL_1524692 to 1524695	ULSS 8 Berica	Istituto Zooprofilattico Sperimentale delle Venezie	Adelaide Milani; Alessia Schivo; Alice Fusaro; Ambra Pastori; Annalisa Salviato; Antonia Ricci; Calogero Terregino; Edoardo Giussani; Elisa Palumbo; Erika Giorgia Quaranta; Isabella Monne; Luca Tassoni
EPI_ISL_1524525 to 1524538	ULSS 8 Berica	Istituto di Genomica Applicata; Istituto Zooprofilattico Sperimentale delle Venezie	Alessia Schivo; Alice Fusaro; Ambra Pastori; Annalisa Salviato; Antonia Ricci; Calogero Terregino; Davide Scaglione; Edoardo Giussani; Eleonora Paparelli; Erika Giorgia Quaranta; Francesca Bruno; Gabriele Magris; Irena Jurman; Isabella Monne; Luca Tassoni; Maria Varotto; Michele Morgante; Silvia Ormelli; Valeria D'Amico; Vera Vendramin
EPI_ISL_422437 to 422438, EPI_ISL_452181 to 452189, EPI_ISL_522855, EPI_ISL_522860 to 522868	ULSS9 Distretto di Bussolengo	Istituto Zooprofilattico Sperimentale delle Venezie	Adelaide Milani; Alessia Schivo; Alice Fusaro; Ambra Pastori; Annalisa Salviato; Antonia Ricci; Bianca Zecchin; Calogero Terregino; Erika Giorgia Quaranta; Isabella Monne
EPI_ISL_452190 to 452191, EPI_ISL_522856, EPI_ISL_522858	ULSS9 Distretto di San Bonifacio	Istituto Zooprofilattico Sperimentale delle Venezie	Adelaide Milani; Alessia Schivo; Alice Fusaro; Ambra Pastori; Annalisa Salviato; Antonia Ricci; Bianca Zecchin; Calogero Terregino; Erika Giorgia Quaranta; Isabella Monne
EPI_ISL_522857, EPI_ISL_522859	ULSS9 Scaligera	Istituto Zooprofilattico Sperimentale delle Venezie	Adelaide Milani; Alessia Schivo; Alice Fusaro; Ambra Pastori; Annalisa Salviato; Antonia Ricci; Bianca Zecchin; Calogero Terregino; Erika Giorgia Quaranta; Isabella Monne
EPI_ISL_1402542 to 1402585, EPI_ISL_1511604 to 1511630, EPI_ISL_1559369 to 1559384	UMC Groningen, Clinical Virology, Department of Medical Microbiology and Infection Prevention	UMC Groningen, Clinical Virology, Department of Medical Microbiology and Infection Prevention	Alexander Friedrich; Coretta Van Leer-Buter; Erley Lizarazo-Forero; Hubert Niesters; Lilli Gard; Marjolein Knoester; Monika Fliss; Monika Flissikowska; Sigrid Rosema; Xuewei Zhou
EPI_ISL_560308 to 560317	UMMC-Health	WHO National Influenza Centre Russian Federation	Andrey Komissarov; Anna Ivanova; Artem Fadeev; Daria Danilenko; Tatiana Platonova
EPI_ISL_1445183 to 1445184	UNIDADE BASICA DE SAUDE DA FAMILIA BILAC	Instituto Butantan / Mendelics	Antonio Jorge Martins; Bibiana Santos; Claudia Renata dos Santos Barros; David Schlesinger; Debora Botequio Moretti; Dimas Tadeu Covas; Elaine Cristina Marqueze; Elaine Vieira dos Santos; Erika Freitas; Evandra Strazza Rodrigues; Flavia Aburjaile; José Salvatore Leister Patané; João Paulo Kitajima; Luiz Carlos Junior de Alcantara; Maria Carolina Elias; Marta Giovanetti; Rafael dos Santos Bezerra; Raul Machado Neto; Ricardo Haddad; Rodrigo Tocantins Calado.; Sandra Coccuzzo Sampaio; Simone Kashima; Svetoslav Nanev Slavov; Vagner Fonseca; Vincent Louis Viala
EPI_ISL_1469660, EPI_ISL_1469751	UNIDADE BASICA DE SAUDE DE RIOZINHO	Epiclin	Ana Paula Muterle; Carolina Comerlato; Eliana Márcia Da Ros Wendland; Fernando Hayashi Sant'Anna; Janira Prichula; Juliana Comerlato
EPI_ISL_1469694	UNIDADE BASICA DE SAUDE PARECI NOVO	Epiclin	Ana Paula Muterle; Carolina Comerlato; Eliana Márcia Da Ros Wendland; Fernando Hayashi Sant'Anna; Janira Prichula; Juliana Comerlato
EPI_ISL_1469680, EPI_ISL_1479126	UNIDADE DE ATENDIMENTO DST AIDS TB E HAN	Epiclin	Ana Paula Muterle; Carolina Comerlato; Eliana Márcia Da Ros Wendland; Fernando Hayashi Sant'Anna; Janira Prichula; Juliana Comerlato
EPI_ISL_1469589, EPI_ISL_1469646, EPI_ISL_1469719, EPI_ISL_1469733, EPI_ISL_1469745, EPI_ISL_1469768, EPI_ISL_1469804			
see above	UNIDADE DE PRONTO ATENDIMENTO DE SAPUCAIA DO	Epiclin	Ana Paula Muterle; Carolina Comerlato; Eliana Márcia Da Ros Wendland; Fernando Hayashi Sant'Anna; Janira Prichula; Juliana Comerlato

	SUL UPA		
EPI_ISL_1445120 to 1445126	UNIDADE DE PRONTO ATENDIMENTO UPA DRA ANA OLIVIA BENTIVOGLIO	Instituto Butantan / Mendelics	Antonio Jorge Martins; Bibiana Santos; Claudia Renata dos Santos Barros; David Schlesinger; Debora Botequio Moretti; Dimas Tadeu Covas; Elaine Cristina Marqueze; Elaine Vieira dos Santos; Erika Freitas; Evandra Strazza Rodrigues; Flavia Aburjaile; José Salvatore Leister Patané; João Paulo Kitajima; Luiz Carlos Junior de Alcantara; Maria Carolina Elias; Marta Giovanetti; Rafael dos Santos Bezerra; Raul Machado Neto; Ricardo Haddad; Rodrigo Tocantins Calado.; Sandra Coccuzzo Sampaio; Simone Kashima; Svetoslav Nanev Slavov; Vagner Fonseca; Vincent Louis Viala
EPI_ISL_1479124	UNIDADE DE SAUDE NOVA HARTZ	Epiclin	Ana Paula Muterle; Carolina Comerlato; Eliana Márcia Da Ros Wendland; Fernando Hayashi Sant'Anna; Janira Prichula; Juliana Comerlato
EPI_ISL_1445172	UNIDADE MISTA ARACOIABA DA SERRA	Instituto Butantan / Mendelics	Antonio Jorge Martins; Bibiana Santos; Claudia Renata dos Santos Barros; David Schlesinger; Debora Botequio Moretti; Dimas Tadeu Covas; Elaine Cristina Marqueze; Elaine Vieira dos Santos; Erika Freitas; Evandra Strazza Rodrigues; Flavia Aburjaile; José Salvatore Leister Patané; João Paulo Kitajima; Luiz Carlos Junior de Alcantara; Maria Carolina Elias; Marta Giovanetti; Rafael dos Santos Bezerra; Raul Machado Neto; Ricardo Haddad; Rodrigo Tocantins Calado.; Sandra Coccuzzo Sampaio; Simone Kashima; Svetoslav Nanev Slavov; Vagner Fonseca; Vincent Louis Viala
EPI_ISL_1445140	UNIDADE MISTA DE SAUDE CAPELA DO ALTO	Instituto Butantan / Mendelics	Antonio Jorge Martins; Bibiana Santos; Claudia Renata dos Santos Barros; David Schlesinger; Debora Botequio Moretti; Dimas Tadeu Covas; Elaine Cristina Marqueze; Elaine Vieira dos Santos; Erika Freitas; Evandra Strazza Rodrigues; Flavia Aburjaile; José Salvatore Leister Patané; João Paulo Kitajima; Luiz Carlos Junior de Alcantara; Maria Carolina Elias; Marta Giovanetti; Rafael dos Santos Bezerra; Raul Machado Neto; Ricardo Haddad; Rodrigo Tocantins Calado.; Sandra Coccuzzo Sampaio; Simone Kashima; Svetoslav Nanev Slavov; Vagner Fonseca; Vincent Louis Viala
EPI_ISL_1445065, EPI_ISL_1445198	UNIDADE MISTA DE TAIACU	Instituto Butantan / Mendelics	Antonio Jorge Martins; Bibiana Santos; Claudia Renata dos Santos Barros; David Schlesinger; Debora Botequio Moretti; Dimas Tadeu Covas; Elaine Cristina Marqueze; Elaine Vieira dos Santos; Erika Freitas; Evandra Strazza Rodrigues; Flavia Aburjaile; José Salvatore Leister Patané; João Paulo Kitajima; Luiz Carlos Junior de Alcantara; Maria Carolina Elias; Marta Giovanetti; Rafael dos Santos Bezerra; Raul Machado Neto; Ricardo Haddad; Rodrigo Tocantins Calado.; Sandra Coccuzzo Sampaio; Simone Kashima; Svetoslav Nanev Slavov; Vagner Fonseca; Vincent Louis Viala
EPI_ISL_1445113, EPI_ISL_1445119, EPI_ISL_1445127 to 1445129	UNIDADE RESPIRATORIA NOVA HORTOLANDIA	Instituto Butantan / Mendelics	Antonio Jorge Martins; Bibiana Santos; Claudia Renata dos Santos Barros; David Schlesinger; Debora Botequio Moretti; Dimas Tadeu Covas; Elaine Cristina Marqueze; Elaine Vieira dos Santos; Erika Freitas; Evandra Strazza Rodrigues; Flavia Aburjaile; José Salvatore Leister Patané; João Paulo Kitajima; Luiz Carlos Junior de Alcantara; Maria Carolina Elias; Marta Giovanetti; Rafael dos Santos Bezerra; Raul Machado Neto; Ricardo Haddad; Rodrigo Tocantins Calado.; Sandra Coccuzzo Sampaio; Simone Kashima; Svetoslav Nanev Slavov; Vagner Fonseca; Vincent Louis Viala
EPI_ISL_1469583, EPI_ISL_1469720, EPI_ISL_1469722, EPI_ISL_1469765	UNIDADE SANITARIA DE IGREJINHA	Epiclin	Ana Paula Muterle; Carolina Comerlato; Eliana Márcia Da Ros Wendland; Fernando Hayashi Sant'Anna; Janira Prichula; Juliana Comerlato
EPI_ISL_1433812, EPI_ISL_1433958, EPI_ISL_1433960, EPI_ISL_1433962, EPI_ISL_1433964, EPI_ISL_1433966 to 1433967, EPI_ISL_1433969, EPI_ISL_1433972, EPI_ISL_1433974, EPI_ISL_1433976 to 1433977, EPI_ISL_1433979, EPI_ISL_1434144, EPI_ISL_1434146 to 1434147, EPI_ISL_1434150, EPI_ISL_1434152, EPI_ISL_1434154, EPI_ISL_1434156, EPI_ISL_1434158, EPI_ISL_1434160, EPI_ISL_1434163, EPI_ISL_1434165, EPI_ISL_1434167, EPI_ISL_1434169, EPI_ISL_1434171 to 1434172, EPI_ISL_1434174, EPI_ISL_1434176, EPI_ISL_1434178, EPI_ISL_1434180, EPI_ISL_1434182, EPI_ISL_1434184, EPI_ISL_1434270, EPI_ISL_1434272, EPI_ISL_1457770			
see above	UNILIANS DECINES	CNR Virus des Infections Respiratoires - France SUD	Antonin Bal; Bruno Lina; Gregory Destras; Gwendolyne Burfin; Hadrien Regue; Laurence Josset; Martine Valette; Quentin Semanas
EPI_ISL_1517302	UNISANTE	Laboratory of genomics and metagenomics	Claire Bertelli; Damien Jacot; Gilbert Greub; SÃ©bastien Aeby; Trestan Pillonel
EPI_ISL_445317, EPI_ISL_445322	UNIV.DE CHILE HOSP.CLINICO	Instituto de Salud Publica de Chile	Alejandra Acevedo; Andrés E Castillo; Bárbara Parra; Carolina Tambley; Gabriel Leal; Jaime Lagos; Jorge Fernandez; Loredana Arata; Patricia Bustos; Paz Tapia; Rodrigo Fasce; Winston Andrade
EPI_ISL_445247	UNIVERSIDAD DE LOS ANDES	Instituto de Salud Publica de Chile	Alejandra Acevedo; Andrés E Castillo; Bárbara Parra; Carolina Tambley; Gabriel Leal; Jaime Lagos; Jorge Fernandez; Loredana Arata; Patricia Bustos; Paz Tapia; Rodrigo Fasce; Winston Andrade
EPI_ISL_582123, EPI_ISL_583954 to 583967, EPI_ISL_1408885 to 1408886, EPI_ISL_1416312 to 1416320, EPI_ISL_1500426 to 1500432	UOC Microbiologia e Virologia, Azienda Ospedaliera Universitaria Senese, Siena, Italy	Dipartimento di Biotecnologie Mediche	Claudia Gandolfo; David Pinzauti; Francesco Santoro; Gabriele Anichini; Gianni Gori Savellini; Gianni Pozzi; Maria Grazia Cusi
EPI_ISL_515522	UPA 24HS de Itatiba	Instituto Adolfo Lutz, Interdiciplinary Procedures Center, Strategic Laboratory	Claudia Regina Gonçalves; Claudio Tavares Sacchi; Erica Valessa Ramos Gomes
EPI_ISL_523983, EPI_ISL_523993	UPA Campo Limpo	Instituto Adolfo Lutz, Interdiciplinary Procedures Center, Strategic Laboratory	Claudia Regina Gonçalves; Claudio Tavares Sacchi; Erica Valessa Ramos Gomes
EPI_ISL_603025	UPA Central de Caraguatatuba	Instituto Adolfo Lutz, Interdiciplinary Procedures Center, Strategic Laboratory	Claudia Regina Gonçalves; Claudio Tavares Sacchi; Erica Valessa Ramos Gomes; Karoline Rodrigues Campos
EPI_ISL_534311	UPA III 26 de Agosto	Instituto Adolfo Lutz, Interdiciplinary Procedures Center, Strategic Laboratory	Claudia Regina Gonçalves; Claudio Tavares Sacchi; Erica Valessa Ramos Gomes
EPI_ISL_583496	UPA Jandira	Instituto Adolfo Lutz, Interdiciplinary Procedures Center, Strategic Laboratory	Claudia Regina Gonçalves; Claudio Tavares Sacchi; Erica Valessa Ramos Gomes; Karoline Rodrigues Campos
EPI_ISL_523975, EPI_ISL_523980	UPA Tito Lopes	Instituto Adolfo Lutz, Interdiciplinary Procedures Center, Strategic Laboratory	Claudia Regina Gonçalves; Claudio Tavares Sacchi; Erica Valessa Ramos Gomes
EPI_ISL_468316	UPA Vila Assis	Instituto Adolfo Lutz, Interdiciplinary Procedures Center, Strategic Laboratory	Claudia Regina Gonçalves; Claudio Tavares Sacchi; Erica Valessa Ramos Gomes
EPI_ISL_515550	UPA Vila Santa Catarina	Instituto Adolfo Lutz, Interdiciplinary Procedures Center, Strategic Laboratory	Claudia Regina Gonçalves; Claudio Tavares Sacchi; Erica Valessa Ramos Gomes
EPI_ISL_1491515, EPI_ISL_1491518 to 1491519, EPI_ISL_1491521	UPMC Children's Hospital of Pittsburgh	Genomics and Discovery, Respiratory Viruses Branch, Division of Viral Diseases, Centers for Disease Control and Prevention	Anna Kelleher; Anna Uehara; Brian Lynch; Clinton R. Paden; Haibin Wang; Han Jia Justin Ng; Jing Zhang; Krista Queen; Peter Cook; Suxiang Tong; Yan Li; Ying Tao
EPI_ISL_436466 to 436503	UPMC Clinical Laboratory	Microbial Genome Sequencing Center, Microbial Genomic Epidemiological Laboratory	Chinelo Ezeonwuku; Dan Snyder; Jane W. Marsh; Kady D Waggle; Lee H. Harrison; Marissa P Griffith; Mustapha M Mustapha; Stephanie L Mitchell; Vatsala R Srinivasa; Vaughn S. Cooper
EPI_ISL_454355 to 454368, EPI_ISL_454370 to 454376, EPI_ISL_454378 to 454384, EPI_ISL_454387, EPI_ISL_454399, EPI_ISL_454401 to 454404, EPI_ISL_454406 to 454411			
see above	UPMC Clinical Microbiology Laboratory	Microbial Genome Sequencing Center, Microbial Genomic Epidemiological Laboratory	Chinelo Ezeonwuku; Dan Snyder; Jane W. Marsh; Kady D. Waggle; Lee H. Harrison; Marissa P. Griffith; Mustapha M. Mustapha; Stephanie L. Mitchell; Vatsala R. Srinivasa; Vaughn S. Cooper
EPI_ISL_454354	UPMC Clinical Microbiology Laboratory	Microbial Genome Sequencing Center; Microbial Genomic Epidemiology Laboratory, University of Pittsburgh	Chinelo Ezeonwuku; Dan Snyder; Jane W. Marsh; Kady D. Waggle; Lee H. Harrison; Marissa P. Griffith; Mustapha M. Mustapha; Stephanie L. Mitchell; Vatsala R. Srinivasa; Vaughn S. Cooper
EPI_ISL_1394629 to 1394641, EPI_ISL_1394643, EPI_ISL_1397552, EPI_ISL_1397555 to 1397580, EPI_ISL_1397630 to 1397635, EPI_ISL_1397810 to 1397823, EPI_ISL_1397971 to 1397982, EPI_ISL_1397984 to 1397987, EPI_ISL_1397989 to 1398001, EPI_ISL_1398005 to 1398020, EPI_ISL_1398123 to 1398131, EPI_ISL_1398137 to 1398143, EPI_ISL_1398271 to 1398273, EPI_ISL_1398312 to 1398317, EPI_ISL_1461290 to 1461292, EPI_ISL_1461300 to 1461309, EPI_ISL_1461318, EPI_ISL_1462509 to 1462510, EPI_ISL_1462512 to 1462513, EPI_ISL_1462515 to 1462516, EPI_ISL_1462518 to 1462519, EPI_ISL_1462521, EPI_ISL_1462523 to 1462525, EPI_ISL_1462527 to 1462528, EPI_ISL_1462530 to 1462531, EPI_ISL_1462533 to 1462534, EPI_ISL_1465618 to 1465621, EPI_ISL_1465623 to 1465632, EPI_ISL_1465640 to 1465664, EPI_ISL_1465667, EPI_ISL_1465696, EPI_ISL_1465723 to 1465754, EPI_ISL_1482278 to 1482286, EPI_ISL_1482296 to 1482332, EPI_ISL_1482625 to 1482626, EPI_ISL_1499395 to 1499436			
see above	URMC LABS	Wadsworth Center, New York State Department of Health	Alexis Russel; Alexis Russell; Catharine Prussing; Daryl M. Lamson; Erasmus Schneider; Erica Lasek-Nesselquist; John Kelly; Jonathan Plitnick; Kirsten St. George; Matthew Shudt; Melissa A Leisner; Navjot Singh
EPI_ISL_1531431 to 1531530	US Air Force School of Aerospace Medicine	US Air Force School of Aerospace Medicine	Amanda Javorina; Anthony Fries; Clarise Starr; Elizabeth Macias; Jennifer Meyer; Sarah Purves; William Buggele; William Gruner
EPI_ISL_436045 to 436046	US VI Department of Health	Pathogen Discovery, Respiratory Viruses Branch, Division of Viral Diseases, Centers for Disease Control and Prevention	Anna Uehara; Bettina Bankamp; Clinton R. Paden; Haibin Wang; Jing Zhang; Krista Queen; Suxiang Tong; Yan Li; Ying Tao; Zachary Weiner
EPI_ISL_1549214	USA Health Care Authority	Synergy Laboratories	Megan Cornwell

EPI_ISL_1445203	USAFA GUILHERMINA	Instituto Butantan / Mendelics	Antonio Jorge Martins; Bibiana Santos; Claudia Renata dos Santos Barros; David Schlesinger; Debora Botequio Moretti; Dimas Tadeu Covas; Elaine Cristina Marqueze; Elaine Vieira dos Santos; Erika Freitas; Evandra Strazza Rodrigues; Flavia Aburjaile; José Salvatore Leister Patané; João Paulo Kitajima; Luiz Carlos Junior de Alcantara; Maria Carolina Elias; Marta Giovanetti; Rafael dos Santos Bezerra; Raul Machado Neto; Ricardo Haddad; Rodrigo Tocantins Calado.; Sandra Coccuzzo Sampaio; Simone Kashima; Svetoslav Nanev Slavov; Vagner Fonseca; Vincent Louis Viala
EPI_ISL_1445202, EPI_ISL_1445204	USAFA MARACANA	Instituto Butantan / Mendelics	Antonio Jorge Martins; Bibiana Santos; Claudia Renata dos Santos Barros; David Schlesinger; Debora Botequio Moretti; Dimas Tadeu Covas; Elaine Cristina Marqueze; Elaine Vieira dos Santos; Erika Freitas; Evandra Strazza Rodrigues; Flavia Aburjaile; José Salvatore Leister Patané; João Paulo Kitajima; Luiz Carlos Junior de Alcantara; Maria Carolina Elias; Marta Giovanetti; Rafael dos Santos Bezerra; Raul Machado Neto; Ricardo Haddad; Rodrigo Tocantins Calado.; Sandra Coccuzzo Sampaio; Simone Kashima; Svetoslav Nanev Slavov; Vagner Fonseca; Vincent Louis Viala
EPI_ISL_1445201	USAFA TUDE BASTOS	Instituto Butantan / Mendelics	Antonio Jorge Martins; Bibiana Santos; Claudia Renata dos Santos Barros; David Schlesinger; Debora Botequio Moretti; Dimas Tadeu Covas; Elaine Cristina Marqueze; Elaine Vieira dos Santos; Erika Freitas; Evandra Strazza Rodrigues; Flavia Aburjaile; José Salvatore Leister Patané; João Paulo Kitajima; Luiz Carlos Junior de Alcantara; Maria Carolina Elias; Marta Giovanetti; Rafael dos Santos Bezerra; Raul Machado Neto; Ricardo Haddad; Rodrigo Tocantins Calado.; Sandra Coccuzzo Sampaio; Simone Kashima; Svetoslav Nanev Slavov; Vagner Fonseca; Vincent Louis Viala
EPI_ISL_1497526 to 1497540, EPI_ISL_1502810 to 1502812, EPI_ISL_1503989 to 1503990, EPI_ISL_1503993 to 1503994, EPI_ISL_1503996 to 1503997, EPI_ISL_1504025, EPI_ISL_1504028 to 1504041, EPI_ISL_1506120 to 1506126, EPI_ISL_1508903 to 1508908, EPI_ISL_1508910 to 1508914, EPI_ISL_1508919 to 1508939, EPI_ISL_1509274 to 1509297, EPI_ISL_1515800			
see above	USC Clinical Lab	Los Angeles County PHL	P. Hemarajata et al.
EPI_ISL_1415422, EPI_ISL_1524851	USCA Avezzano AVEZZANO(L'AQUILA)	Istituto Zooprofilattico Sperimentale dell'Abruzzo e Molise "G. Caporale"	Ancora M; Calistri P; Cammà C; Caporale M; Curini V; Delli Compagni E; Di Domenico M; Di Lollo Valeria; Di Pasquale A; Lorusso A; Mangone I; Marcacci M; Puglia I; Rinaldi A; Savini G; Scialabba S
EPI_ISL_1403515 to 1403517	USCA Castel di Sangro CASTEL DI SANGRO(L'AQUILA)	Istituto Zooprofilattico Sperimentale dell'Abruzzo e Molise "G. Caporale"	Ancora M; Calistri P; Cammà C; Curini V; Delli Compagni E; Di Domenico M; Di Pasquale A; Lorusso A; Mangone I; Marcacci M; Puglia I; Rinaldi A; Savini G; Scialabba S
EPI_ISL_1392685 to 1392687, EPI_ISL_1403526, EPI_ISL_1403542, EPI_ISL_1403545, EPI_ISL_1403547, EPI_ISL_1502528 to 1502529	USCA Pescina PESCINA(L'AQUILA)	Istituto Zooprofilattico Sperimentale dell'Abruzzo e Molise "G. Caporale"	Ancora M; Calistri P; Cammà C; Caporale M; Curini V; Delli Compagni E; Di Domenico M; Di Lollo Valeria; Di Pasquale A; Lorusso A; Mangone I; Marcacci M; Puglia I; Rinaldi A; Savini G; Scialabba S
EPI_ISL_1403529, EPI_ISL_1403537	USCA Sulmona SULMONA(L'AQUILA)	Istituto Zooprofilattico Sperimentale dell'Abruzzo e Molise "G. Caporale"	Ancora M; Calistri P; Cammà C; Curini V; Delli Compagni E; Di Domenico M; Di Pasquale A; Lorusso A; Mangone I; Marcacci M; Puglia I; Rinaldi A; Savini G; Scialabba S
EPI_ISL_1392683 to 1392684, EPI_ISL_1403504, EPI_ISL_1420832	USCA Tagliacozzo TAGLIACOZZO(L'AQUILA)	Istituto Zooprofilattico Sperimentale dell'Abruzzo e Molise "G. Caporale"	Ancora M; Calistri P; Cammà C; Caporale M; Curini V; Delli Compagni E; Di Domenico M; Di Lollo Valeria; Di Pasquale A; Lorusso A; Mangone I; Marcacci M; Puglia I; Rinaldi A; Savini G; Scialabba S
EPI_ISL_1445070	USF ARCADAS	Instituto Butantan / Mendelics	Antonio Jorge Martins; Bibiana Santos; Claudia Renata dos Santos Barros; David Schlesinger; Debora Botequio Moretti; Dimas Tadeu Covas; Elaine Cristina Marqueze; Elaine Vieira dos Santos; Erika Freitas; Evandra Strazza Rodrigues; Flavia Aburjaile; José Salvatore Leister Patané; João Paulo Kitajima; Luiz Carlos Junior de Alcantara; Maria Carolina Elias; Marta Giovanetti; Rafael dos Santos Bezerra; Raul Machado Neto; Ricardo Haddad; Rodrigo Tocantins Calado.; Sandra Coccuzzo Sampaio; Simone Kashima; Svetoslav Nanev Slavov; Vagner Fonseca; Vincent Louis Viala
EPI_ISL_1445068	USF JARDIM DAS AVES MOREIRINHA	Instituto Butantan / Mendelics	Antonio Jorge Martins; Bibiana Santos; Claudia Renata dos Santos Barros; David Schlesinger; Debora Botequio Moretti; Dimas Tadeu Covas; Elaine Cristina Marqueze; Elaine Vieira dos Santos; Erika Freitas; Evandra Strazza Rodrigues; Flavia Aburjaile; José Salvatore Leister Patané; João Paulo Kitajima; Luiz Carlos Junior de Alcantara; Maria Carolina Elias; Marta Giovanetti; Rafael dos Santos Bezerra; Raul Machado Neto; Ricardo Haddad; Rodrigo Tocantins Calado.; Sandra Coccuzzo Sampaio; Simone Kashima; Svetoslav Nanev Slavov; Vagner Fonseca; Vincent Louis Viala
EPI_ISL_1445069, EPI_ISL_1445072	USF JARDIM SAO DIMAS	Instituto Butantan / Mendelics	Antonio Jorge Martins; Bibiana Santos; Claudia Renata dos Santos Barros; David Schlesinger; Debora Botequio Moretti; Dimas Tadeu Covas; Elaine Cristina Marqueze; Elaine Vieira dos Santos; Erika Freitas; Evandra Strazza Rodrigues; Flavia Aburjaile; José Salvatore Leister Patané; João Paulo Kitajima; Luiz Carlos Junior de Alcantara; Maria Carolina Elias; Marta Giovanetti; Rafael dos Santos Bezerra; Raul Machado Neto; Ricardo Haddad; Rodrigo Tocantins Calado.; Sandra Coccuzzo Sampaio; Simone Kashima; Svetoslav Nanev Slavov; Vagner Fonseca; Vincent Louis Viala
EPI_ISL_1445073	USF SANTA MARIA DO AMPARO	Instituto Butantan / Mendelics	Antonio Jorge Martins; Bibiana Santos; Claudia Renata dos Santos Barros; David Schlesinger; Debora Botequio Moretti; Dimas Tadeu Covas; Elaine Cristina Marqueze; Elaine Vieira dos Santos; Erika Freitas; Evandra Strazza Rodrigues; Flavia Aburjaile; José Salvatore Leister Patané; João Paulo Kitajima; Luiz Carlos Junior de Alcantara; Maria Carolina Elias; Marta Giovanetti; Rafael dos Santos Bezerra; Raul Machado Neto; Ricardo Haddad; Rodrigo Tocantins Calado.; Sandra Coccuzzo Sampaio; Simone Kashima; Svetoslav Nanev Slavov; Vagner Fonseca; Vincent Louis Viala
EPI_ISL_1445067, EPI_ISL_1445071, EPI_ISL_1445074	USF TRES PONTES	Instituto Butantan / Mendelics	Antonio Jorge Martins; Bibiana Santos; Claudia Renata dos Santos Barros; David Schlesinger; Debora Botequio Moretti; Dimas Tadeu Covas; Elaine Cristina Marqueze; Elaine Vieira dos Santos; Erika Freitas; Evandra Strazza Rodrigues; Flavia Aburjaile; José Salvatore Leister Patané; João Paulo Kitajima; Luiz Carlos Junior de Alcantara; Maria Carolina Elias; Marta Giovanetti; Rafael dos Santos Bezerra; Raul Machado Neto; Ricardo Haddad; Rodrigo Tocantins Calado.; Sandra Coccuzzo Sampaio; Simone Kashima; Svetoslav Nanev Slavov; Vagner Fonseca; Vincent Louis Viala
EPI_ISL_1446433, EPI_ISL_1446439, EPI_ISL_1446441, EPI_ISL_1446576 to 1446581, EPI_ISL_1446634, EPI_ISL_1446725 to 1446727, EPI_ISL_1516343 to 1516352, EPI_ISL_1528432 to 1528441			
see above	UT-Unified State Labs: Public Health Utah DOH	Centers for Disease Control and Prevention Division of Viral Diseases, Pathogen Discovery	Alison Laufer Halpin; Ben L. Rambo-Martin; Clinton R. Paden; Dakota Howard; Darlene Wagner; Dave Wentworth; Dhwani Batra; Jasmine Padilla; Justin Lee; Katie Dillon; Krista Queen; Kristen Knipe; Kristine Lacek; Mark Burroughs; Matthew Schmerer; Mili Sheth; Peter Cook; Sam Shepard; Sarah Nobles; Shoshona Le; Suxiang Tong; Vivien Dugan; Yvette Unoarumhi
EPI_ISL_424889 to 424890	UT-Unified State Labs: Public Health Utah DOH	Pathogen Discovery, Respiratory Viruses Branch, Division of Viral Diseases, Centers for Disease Control and Prevention	Alison S. Laufer Halpin; Anna Uehara; Christopher A. Elkins; Clinton R. Paden; Haibin Wang; Jing Zhang; Krista Queen; Mary S. Keckler; Rachel Marine; Suxiang Tong; Yan Li; Ying Tao
EPI_ISL_413562 to 413563, EPI_ISL_413649 to 413653, EPI_ISL_414363 to 414369, EPI_ISL_414591 to 414597, EPI_ISL_414616 to 414622, EPI_ISL_415591 to 415627, EPI_ISL_416433 to 416456, EPI_ISL_416635 to 416729, EPI_ISL_417341 to 417382, EPI_ISL_417448 to 417456, EPI_ISL_418028 to 418082, EPI_ISL_418866 to 418924, EPI_ISL_418926 to 418940, EPI_ISL_418942 to 418955, EPI_ISL_422961 to 422967, EPI_ISL_422969 to 423022, EPI_ISL_423024 to 423033, EPI_ISL_424166 to 424188, EPI_ISL_424190 to 424219, EPI_ISL_424239 to 424247, EPI_ISL_424249 to 424341, EPI_ISL_426052 to 426137, EPI_ISL_427161 to 427270, EPI_ISL_429597 to 429656, EPI_ISL_430867 to 430980, EPI_ISL_437803 to 437872, EPI_ISL_443184 to 443186, EPI_ISL_460621 to 460634, EPI_ISL_461399 to 461477, EPI_ISL_476900 to 476940, EPI_ISL_477673 to 477724, EPI_ISL_485917 to 485942, EPI_ISL_485944 to 485961, EPI_ISL_485963 to 485964, EPI_ISL_485967 to 485969, EPI_ISL_485971 to 486030, EPI_ISL_486032 to 486090, EPI_ISL_486093 to 486095, EPI_ISL_486097 to 486099, EPI_ISL_486102 to 486103, EPI_ISL_486105 to 486106, EPI_ISL_486108 to 486110, EPI_ISL_486112 to 486114, EPI_ISL_490979 to 490980, EPI_ISL_490982 to 490987, EPI_ISL_490989 to 490996, EPI_ISL_491001 to 491003, EPI_ISL_491005 to 491007, EPI_ISL_491009 to 491034, EPI_ISL_497872, EPI_ISL_511852 to 511861, EPI_ISL_570027 to 571015, EPI_ISL_576399 to 576405, EPI_ISL_576407 to 576517, EPI_ISL_576557 to 576564, EPI_ISL_603251 to 603265, EPI_ISL_610283 to 610285, EPI_ISL_1400350 to 1400443, EPI_ISL_1405061 to 1405403, EPI_ISL_1448051 to 1448397, EPI_ISL_1490861 to 1490896, EPI_ISL_1497767 to 1498093, EPI_ISL_1523794 to 1524014, EPI_ISL_1526795 to 1526835, EPI_ISL_1526910 to 1526989			
see above	UW Virology Lab	UW Virology Lab	Alexander Greninger; Amin Addetia; Hong Xie; Keith Jerome; Keith R Jerome; Lasata Shrestha; Margaret Mills; Meei-Li Huang; Michelle Lin; Noah Baker; Noah R. Baker; Pavitra Roychoudhury; Saraswathi Sathees; Sean Ellis; Shah Mohamed Bakhash; Truong Nguyen; Victoria M Rachleff
EPI_ISL_512086	UW Virology lab	UW Virology lab	Alexander Greninger; Amin Addetia; Hong Xie; Keith Jerome; Lasata Shrestha; Meei-Li Huang; Pavitra Roychoudhury; Truong Nguyen
EPI_ISL_451183 to 451202	Uganda Virus Research Institute	MRC/UVRI & LSHTM Uganda Research Unit	Beatrice Dhaala; Dan Lule Bugembe; Deogratius Ssemwanga; Henry Kyobe; Henry Mwebesa; Jane Aceng; John Kayiwa; Jonas Lexow; Julius Lutwama; Matthew Cotten; My V.T Phan; Phionah Tushabe; Pontiano Kaleebu; Stephen Balinandi
EPI_ISL_434646, EPI_ISL_434667, EPI_ISL_445228, EPI_ISL_445240, EPI_ISL_450824, EPI_ISL_455104, EPI_ISL_455109, EPI_ISL_469063, EPI_ISL_469072, EPI_ISL_475521, EPI_ISL_476138			
see above	Ulltuna Vardcentral	The Public Health Agency of Sweden	Anna Risberg; Anna-Malin Linde; Heidi Lindback; Karin Tegmark-Wisell; Maria Lind Karlberg; Mattias Haukland; Mia Brytting; Olov Svartstrom; Oskar Karlsson Lindsjo; Petra Edquist; Reza Advani; Sandra Broddesson; Shamam Muradrasoli; Theresa Enkirch
EPI_ISL_475122, EPI_ISL_475126 to 475127, EPI_ISL_475143, EPI_ISL_615092 to 615096	Umea klinisk mikrobiologi	The Public Health Agency of Sweden	Anna Risberg; Anna-Malin Linde; Karin Tegmark-Wisell; Maria Lind Karlberg; Mattias Haukland; Mia Brytting; Olov Svartstrom; Oskar Karlsson Lindsjo; Petra Edquist; Reza Advani; Sandra Broddesson; Shamam Muradrasoli
EPI_ISL_1423505	Unidad de Patologia Clinica	Instituto de Diagnostico y Referencia Epidemiologicos (INDRE)	Abril Rodriguez-Maldonado; Ariadna Medina-Benitez; Claudia Wong-Arambula; Ernesto Ramirez-Gonzalez.; Gisela Barrera-Badillo; Irma Lopez-Martinez; Joaquin Quiroz-Mercado; Lucia Hernandez-Rivas; Natividad Cruz-Ortiz; Sergio Rangel-Guerrero; Tatiana Nunez-Garcia; Vanessa Rivero-Arredondo
EPI_ISL_1469563, EPI_ISL_1469763	Unidade Básica de Saúde de Riozinho	Epiclin	Ana Paula Muterle; Carolina Comerlato; Eliana Márcia Da Ros Wendland; Fernando Hayashi Sant'Anna; Janira Prichula; Juliana Comerlato
EPI_ISL_1469552, EPI_ISL_1469567, EPI_ISL_1469576, EPI_ISL_1469614,	Unidade Sanitária de Igrejinha	Epiclin	Ana Paula Muterle; Carolina Comerlato; Eliana Márcia Da Ros Wendland; Fernando Hayashi Sant'Anna; Janira Prichula; Juliana Comerlato

EPI_ISL_1469716			
EPI_ISL_1469649, EPI_ISL_1469664, EPI_ISL_1469714	Unidade de Atendimento DST AIDS TB e Han	Epiclin	Ana Paula Muterle; Carolina Comerlato; Eliana Márcia Da Ros Wendland; Fernando Hayashi Sant'Anna; Janira Prichula; Juliana Comerlato
EPI_ISL_574590	Unidade de Printo Atendimento UPA I Santa Isabel	Instituto Adolfo Lutz, Interdiciplinary Procedures Center, Strategic Laboratory	Claudia Regina Gonçalves; Claudio Tavares Sacchi; Erica Valessa Ramos Gomes; Karoline Rodrigues Campos
EPI_ISL_1533713, EPI_ISL_1533717	Unidade de Pronto Atendimento Jd Amanda	Instituto Adolfo Lutz, Interdiciplinary Procedures Center, Strategic Laboratory	Caio Vinicius Dias Lopes; Claudia Regina Gonçalves; Claudio Tavares Sacchi; Erica Valessa Ramos Gomes; Karoline Rodrigues Campos; Leonardo Jose Tadeu de Araujo
EPI_ISL_1520115 to 1520116	Unidade de Pronto Atendimento UPA	Instituto Adolfo Lutz, Interdiciplinary Procedures Center, Strategic Laboratory	Caio Vinicius Dias Lopes; Claudia Regina Gonçalves; Claudio Tavares Sacchi; Erica Valessa Ramos Gomes; Karoline Rodrigues Campos
EPI_ISL_1469565, EPI_ISL_1469571, EPI_ISL_1469577, EPI_ISL_1469580, EPI_ISL_1469582, EPI_ISL_1469695, EPI_ISL_1469703, EPI_ISL_1469718, EPI_ISL_1469747, EPI_ISL_1469790, EPI_ISL_1469810			
see above	Unidade de Pronto Atendimento de Sapucaia do Sul	Epiclin	Ana Paula Muterle; Carolina Comerlato; Eliana Márcia Da Ros Wendland; Fernando Hayashi Sant'Anna; Janira Prichula; Juliana Comerlato
EPI_ISL_1493585, EPI_ISL_1493594	Unidade de Saude Dr Phebo de Oliveira Roge Ferreira	Instituto Adolfo Lutz, Interdiciplinary Procedures Center, Strategic Laboratory	Caio Vinicius Dias Lopes; Claudia Regina Gonçalves; Claudio Tavares Sacchi; Erica Valessa Ramos Gomes; Karoline Rodrigues Campos
EPI_ISL_534325	Unidade de Vigilancia em Saude de Guarulhos	Instituto Adolfo Lutz, Interdiciplinary Procedures Center, Strategic Laboratory	Claudia Regina Gonçalves; Claudio Tavares Sacchi; Erica Valessa Ramos Gomes
EPI_ISL_1441231, EPI_ISL_1441233, EPI_ISL_1441235, EPI_ISL_1441237, EPI_ISL_1441239, EPI_ISL_1441241, EPI_ISL_1441248, EPI_ISL_1441299			
see above	Uniklinikum Carl Gustav Carus an der TU Dresden; Institut für Virologie	Robert Koch Institute	
EPI_ISL_510827 to 510830, EPI_ISL_582794 to 582796, EPI_ISL_594154	Unilabs Eskilstuna	The Public Health Agency of Sweden	Anna Risberg; Anna-Malin Linde; Karin Tegmark-Wisell; Maria Lind Karlberg; Mattias Haukland; Mia Brytting; Olov Svartstrom; Oskar Karlsson Lindsjo; Petra Edquist; Reza Advani; Sandra Broddesson
EPI_ISL_420143, EPI_ISL_471172, EPI_ISL_549050, EPI_ISL_549123, EPI_ISL_549125, EPI_ISL_549172, EPI_ISL_549175, EPI_ISL_590882, EPI_ISL_590911, EPI_ISL_590917, EPI_ISL_591010, EPI_ISL_591013, EPI_ISL_1490256 to 1490257, EPI_ISL_1490272, EPI_ISL_1490334, EPI_ISL_1490459			
see above	Unilabs Laboratory Medicine	Norwegian Institute of Public Health, Department of Virology	Atiya R Ali; Debech Nadia; Engebretsen Serina Beate; Garcia Llorente Ignacio; Hilde Elshaug; Hilde Synnøve Vollan; Hilde Vollan; Jon Bråte; Kamilla Heddeland Instefjord; Karoline Bragstad; Kathrine Stene-Johansen; Marie Paulsen Madsen; Olav Hungnes; Pedersen Benedikte Nevjen; Rasmus Riis Kopperud
EPI_ISL_429126 to 429127, EPI_ISL_450835	Unilabs Skovde	The Public Health Agency of Sweden	Anna Risberg; Anna-Malin Linde; Helena Enroth; Karin Tegmark-Wisell; Maria Lind Karlberg; Mia Brytting; Olov Svartstrom; Oskar Karlsson Lindsjo; Shaman Muradrasoli; Theresa Enkirch; Tobias Kollberg
EPI_ISL_412979 to 412980	Union Hospital of Tongji Medical College, Huazhong University of Science and Technology	Hubei Provincial Center for Disease Control and Prevention	Bin Fang; Bo Yang; Bo Yu; Faxian Zhan; Guojun Ye; Jing Li; Junqiang Xu; Kun Cai; Linlin Liu; Xiang Li; Xiao Yu; Xixiang Huo; Yongzhong Jiang.
EPI_ISL_1534269, EPI_ISL_1534372	Uniondale Hospital wc UDH	NHLS/UCT	Arash Iranzadeh; Bruna Galvao; Carolyn Williamson; Deelan Doolabh; Diana Hardie; Emmanuel SJ; Innocent Mudau; Kruger Marais; Lynn Tyers; Marvin Hsiao; Stephen Korsman; Tegally H; de Oliveira T
EPI_ISL_411951	Unit for Laboratory Development and Technology Transfer, Public Health Agency of Sweden	Unit for Laboratory Development and Technology Transfer, Public Health Agency of Sweden	Appelberg, S.; Bengner, M.; Brave, A.; Lind Karlberg, M.; Lindsjo, O.; Monteil, V.; Muradrasoli; Palmerus, M.; S. and Tegmark-Wisell, K.
EPI_ISL_510527	Unite des virus emergents, UMR190	Unite des virus emergents, UMR190	Baronti, C.; Charrel; Coutard, B.; Piorkowski, G.; R. and de Lamballerie, X.
EPI_ISL_463995 to 464064, EPI_ISL_526253 to 526286, EPI_ISL_569949 to 570026, EPI_ISL_609801 to 609826, EPI_ISL_1490672 to 1490709, EPI_ISL_1490717 to 1490747	Unity Health Toronto	Ontario Institute for Cancer Research	Annette Gower; Bernard Lam; Felicia Vincelli; Ilinca Lungu; Jared Simpson; Jared T. Simpson; Jeremy Johns; Karel Boissinot; Larissa M. Matukas; Le Luu; Mark Downing; Paul Krzyzanowski; Philip Zuzarte; Ramzi Fattouh; Richard de Borja; Samira Mubareka; TIBDN; Trina Otterman; Wai Sum Siu; Yan Chen; Zhi Cui
EPI_ISL_1443234 to 1443236, EPI_ISL_1443238 to 1443245, EPI_ISL_1443247 to 1443267, EPI_ISL_1478864, EPI_ISL_1478866 to 1478876, EPI_ISL_1478883	Unity Point Methodist Hospital	Illinois Department of Public Health - Springfield Lab	Bryan Sim; Gordon McCall
EPI_ISL_1478865	Unity Point Proctor Hospital	Illinois Department of Public Health - Springfield Lab	Bryan Sim; Gordon McCall
EPI_ISL_525467 to 525471	Universidad Iberoamericana	International Centre for Genetic Engineering and Biotechnology (ICGEB) and ARGO Open Lab Platform	Alejandro Vallejo Degaudenzi; Danilo Licastro; Eileen Riego; Leandro Tapia; Patricia Leon; Robert Paulino-Ramirez; Simeone Dal Monego; Sreejith Rajasekharan and Alessandro Marcello.; Victor Virgilio Calderon
EPI_ISL_522406	Universidad Iberoamericana (UNIBE)	International Centre for Genetic Engineering and Biotechnology (ICGEB)and ARGO Open Lab Platform	Alejandro Vallejo; Danilo Licastro; Eileen Riego; Leandro Tapia; Robert Paulino-Ramirez; Simeone Dal Monego; Sreejith Rajasekharan; Victor Calderon; and Alessandro Marcello.
EPI_ISL_523811 to 523812	Universidad Iberoamericana, Instituto de Medicina Tropical & Salud Global	International Centre for Genetic Engineering and Biotechnology (ICGEB) and ARGO Open Lab Platform	Alejandro Vallejo Degaudenzi; Danilo Licastro; Eileen Riego; Leandro Tapia; Robert Paulino-Ramirez; Simeone Dal Monego; Sreejith Rajasekharan and Alessandro Marcello.; Victor Virgilio Calderon
EPI_ISL_539783 to 539784	Universidad Regional Amazonica IKIAM	Institute of Microbiology, Universidad San Francisco de Quito	Andrea Carrera; Belén Prado-Vivar; Bernardo Gutiérrez; Carolina Proaño-Bolaños; Fabian Aguilar; Gabriel Trueba; Giovanna Moran; Juan José Guadalupe; Katherine Apunte; Marcelo Ortiz; Michelle Grunauer; Monica Becerra-Wong; Nina Espinoza de los Monteros; Patricio Rojas-Silva; Paúl Cárdenas; Sonia Sislema; Sully Márquez; Verónica Barragán; Yeimy Rojas
EPI_ISL_1540707	Universidad Tecnica Particular de Loja	Institute of Microbiology, Universidad San Francisco de Quito	Belén Prado-Vivar; Bernardo Gutiérrez; David Zuñiga; Fernando Serrano; Gabriel Trueba; Juan José Guadalupe; Katherine Ojeda; Luis Flores; Melissa Ortega; Michelle Grunauer; Monica Becerra-Wong; Paola Dalgo; Patricio Rojas-Silva; Paúl Cárdenas; Raiza Briceño; Sully Márquez; Verónica Barragán
EPI_ISL_537288 to 537379, EPI_ISL_537609 to 537690	Universidad de León	SeqCOVID-SPAIN consortium/IBV(CSIC)	Ana Carvajal; Antonio J. Molina and SeqCOVID-SPAIN consortium; Héctor Argüello; Juan M. Fregeneda; Tania Fernández-Villa; Vicente Martín
EPI_ISL_1424054 to 1424056	Universidad del Magdalena	Instituto Nacional de Salud- Dirección de Investigación en Salud Pública	Carlos Franco-Muñoz; Diego A. Álvarez-Díaz; Diego Andrés Prada; Gerardo Santamaría; Hector Alejandro Ruiz-Moreno; Jhonnatan Reales-González; Julian Naizaque; Katherine Laiton-Donato; Magdalena Wiesner; Marcela Mercado-Reyes.; Maria T. Herrera-Sepúlveda; Martha Lucia Ospina Martínez; Sheryll Corchuelo
EPI_ISL_445219	Universidad del Valle, Laboratorio de Microbiologia, VIREM	Universidad del Valle, Universidad Nacional de Colombia-Sede Palmira, International Center for Tropical Agriculture	Beatriz Parra; Diana López-Alvarez; Wilmer J. Cuellar
EPI_ISL_417951, EPI_ISL_417953, EPI_ISL_417982 to 417985	Universidade Federal do Rio de Janeiro	Bioinformatics Laboratory - LNCC	Alexandra Gerber; Amilcar Tanuri; Ana Paula Guimarães; Cristiano Xavier Lima; Cássia Alves Gonçalves; Diana Mariani; Ester Cerdeira Sabino; Filipe Romero; Ingra Morales Claro; Isabela de Carvalho Leitão; Jaqueline Goes de Jesus; Laboratorio Hermes Pardini; Laboratorio Simile; Luciana Jesus Costa e André Felipe Santos; Luiz Gonzaga Paula de Almeida; Lídia Boullosa; Mariane Talon; Mauro Teixeira; Nuno Rodrigues Faria; Orlando Ferreira Jr.; Pinto Castiñeiras; Rafael de Mello Galliez; Renato Santana Aguiar e Ana Tereza Vasconcelos; Richard Araújo Maia; Rodrigo Brindeiro; Ronaldo da Silva Francisco Junior; Terezinha Marta Pereira; Érica Ramos dos Santos Nascimento
EPI_ISL_418959	Universidade Federal do Rio de Janeiro - UFRJ	Bioinformatics Laboratory - LNCC	Alexandra Gerber; Amilcar Tanuri; Ana Paula Guimarães; Cristiano Xavier Lima; Cássia Cristina Alves Gonçalves; Diana Mariani; Ester Cerdeira Sabino; Filipe Romero; Ingra Morales Claro; Isabela de Carvalho Leitão; Jaqueline Goes de Jesus; Laboratorio Hermes Pardini; Laboratorio Simile; Luciana Jesus Costa e André Felipe Santos; Luiz Gonzaga Paula de Almeida; Lídia Boullosa; Mariane Talon; Mauro Teixeira; Nuno Rodrigues Faria; Orlando Ferreira Jr.; Pinto Castiñeiras; Rafael de Mello Galliez; Renato Santana Aguiar; Richard Araújo Maia; Rodrigo Brindeiro; Ronaldo da Silva Francisco Junior; Terezinha Marta Pereira; e Ana Tereza Vasconcelos; Érica Ramos dos Santos Nascimento
EPI_ISL_437933 to 437973, EPI_ISL_475764 to	Universitaetsklinik für Innere Medizin II Innsbruck	Bergthaler laboratory, CeMM Research Center for Molecular	Adi Steinrigl; Alexander Lercher; Alexandra Popa; Andreas Bergthaler; Benedikt Agerer; Christian Paar; Christoph Bock; Daniela Schmid; Dorothee von

475768, EPI_ISL_475929 to 475936, EPI_ISL_583557 to 583562, EPI_ISL_583617 to 583618		Medicine of the Austrian Academy of Sciences	Laer; Elisabeth Puchhammer-Stoeckl; Franz Allerberger; Gernot Walder; Gregor Hörmann; Guenter Weiss; Gunther Vogl; Henrique Colaco; Jakob-Wendelin Genger; Jan Laine; Judith Aberle; Kinga Rigler-Hohenwarter; Lukas Endler; Manfred Nairz; Mark Smyth; Martin Senekowitsch; Michael Schuster; Peter Hufnagl; Peter Obrist; Rainer Gattringer; Sabine Sussitz-Rack; Stephan Aberle; Thomas Penz; Wegene Borena
EPI_ISL_458082, EPI_ISL_529962 to 529963	Universitas Airlangga Hospital	Institute of Tropical Disease, Universitas Airlangga	Aldise M Nastri; Gatot Soegiarto; Jezzy R Dewantari; Kazufumi Shimizu; Krisnoadi Rahardjo; Laksmi Wulandari; Maria I Lusida; Mitsuhiro Nishimura; Nasronudin; Resti Yudhawati; Retno A Setyoningrum; Rima R Prasetya; Soetjipto; Yasuko Mori; Yohko K Shimizu
EPI_ISL_534224, EPI_ISL_534246 to 534247, EPI_ISL_560977 to 560979	Universitetssjukhuset i Linkoping	The Public Health Agency of Sweden	Anna Risberg; Anna-Malin Linde; Karin Tegmark-Wisell; Maria Lind Karlberg; Mattias Haukland; Mia Brytting; Olov Svartstrom; Oskar Karlsson Lindsjo; Petra Edquist; Reza Advani; Sandra Broddesson
EPI_ISL_463893	University Clinical Center Tuzla	Alea Genetiki Centar	D; Goleti; Huki, M.; Konjhodži; L; Marjanovi; N; Pear; R; Salihefendi; T; Tihi
EPI_ISL_462990	University Clinical Centre of the Republic of Srpska	University of Sarajevo, Veterinary Faculty	Aida, K.; Alma; Maja, T.; Mirsada, H.; Teufik, G.; Toni, E.; Š.A.; Šejla, G.
EPI_ISL_462753	University Clinical Hospital of Mostar	University of Sarajevo Veterinary Faculty	Eterovic, T.; Goletic, S.; Goletic, T.; Hukic, M.; Ostojic, M.; Seho-Alic, A.; Softic, A.
EPI_ISL_507040 to 507109	University College London Hospital	COVID-19 Genomics UK (COG-UK) Consortium	Catherine Houlihan; Dan Frampton; Judith Heaney; Matthew Byott; Moira Spyer and Eleni Nastouli; Stuart Kirk
EPI_ISL_440951 to 441051, EPI_ISL_441350 to 441354, EPI_ISL_444079 to 444272, EPI_ISL_449626 to 449729, EPI_ISL_453460 to 453464, EPI_ISL_457270 to 457302, EPI_ISL_461763 to 461772, EPI_ISL_478404 to 478472, EPI_ISL_572684 to 572685, EPI_ISL_572709, EPI_ISL_572725, EPI_ISL_572770, EPI_ISL_572800, EPI_ISL_572807 to 572809, EPI_ISL_572828 to 572829, EPI_ISL_572837 to 572838, EPI_ISL_572957, EPI_ISL_573478 to 573525, EPI_ISL_573652 to 573685, EPI_ISL_573764, EPI_ISL_573800 to 573805, EPI_ISL_573856 to 573865, EPI_ISL_574258, EPI_ISL_576984 to 576985, EPI_ISL_584670 to 584679, EPI_ISL_584811 to 584846, EPI_ISL_585502 to 585503, EPI_ISL_594861 to 594888, EPI_ISL_611523, EPI_ISL_611527, EPI_ISL_611558 to 611559, EPI_ISL_611595 to 611597, EPI_ISL_611622 to 611623, EPI_ISL_611665, EPI_ISL_611822 to 611823, EPI_ISL_611996, EPI_ISL_612000, EPI_ISL_612014, EPI_ISL_612078 to 612079, EPI_ISL_612103, EPI_ISL_612105, EPI_ISL_612131, EPI_ISL_612198, EPI_ISL_612450 to 612457, EPI_ISL_1386813 to 1386925, EPI_ISL_1386927 to 1386931, EPI_ISL_1474800, EPI_ISL_1474829 to 1474892, EPI_ISL_1546229 to 1546238			
see above	University College London, Great Ormond Street Hospital for Children NHS Foundation Trust, Imperial College Healthcare NHS Trust	COVID-19 Genomics UK (COG-UK) Consortium	Alison Holmes; Charlotte Williams; Helena Tutill; Jacqueline Findlay; James Price; Judith Breuer; Julianne Brown; Kathryn Harris; Leysa Forrest; Mark Kristiansen; Paola Niola; Paola Resende Silva; Patricia Dyal; Paul Randell; Rachel Williams; Sam Weeks; Samuel Weeks; Sergi Castellano; Sunando Roy; Tony Brooks; Yasmin Panchbhaya
EPI_ISL_492495, EPI_ISL_492497, EPI_ISL_492746, EPI_ISL_492748, EPI_ISL_492750, EPI_ISL_492758	University College London, Great Ormond Street Hospital for Children NHS Foundation Trust, Imperial College Healthcare NHS Trust	Wellcome Sanger Institute for the COVID-19 Genomics UK (COG-UK) Consortium	Alison Holmes; Charlotte Williams; Cordelia Langford; David K. Jackson; Dominic Kwiatkowski; Ewan Harrison; Helena Tutill; Ian Johnston; Jacqueline Findlay; James Price; John Sillitoe on behalf of the Wellcome Sanger Institute COVID-19 Surveillance Team; Judith Breuer and Alex Alderton; Julianne Brown; Kathryn Harris; Leysa Forrest; Mark Kristiansen; Paola Niola; Paola Resende Silva; Patricia Dyal; Paul Randell; Rachel Williams; Roberto Amato; Sam Weeks; Sergi Castellano; Sonia Goncalves; Sunando Roy; Tony Brooks; Yasmin Panchbhaya
EPI_ISL_482160 to 482218, EPI_ISL_487525, EPI_ISL_487529, EPI_ISL_487534, EPI_ISL_487537, EPI_ISL_487540, EPI_ISL_487546, EPI_ISL_487550, EPI_ISL_487556 to 487559, EPI_ISL_487562, EPI_ISL_487564, EPI_ISL_487568, EPI_ISL_487572, EPI_ISL_487578, EPI_ISL_487581 to 487582, EPI_ISL_487584 to 487585, EPI_ISL_487589, EPI_ISL_487595, EPI_ISL_487597, EPI_ISL_487600, EPI_ISL_487606 to 487607, EPI_ISL_487614 to 487616, EPI_ISL_487621, EPI_ISL_487625 to 487628, EPI_ISL_487630 to 487633, EPI_ISL_487635 to 487646, EPI_ISL_492496, EPI_ISL_492498 to 492504, EPI_ISL_492747, EPI_ISL_492749, EPI_ISL_492751 to 492757, EPI_ISL_492759 to 492763			
see above	University College London, Great Ormond Street Hospital for Children NHS Foundation Trust, Imperial College Healthcare NHS Trust	Wellcome Sanger Institute for the COVID-19 Genomics UK (COG-UK) consortium	Alison Holmes; Charlotte Williams; Cordelia Langford; David K. Jackson; Dominic Kwiatkowski; Ewan Harrison; Helena Tutill; Ian Johnston; Jacqueline Findlay; James Price; John Sillitoe on behalf of the Wellcome Sanger Institute COVID-19 Surveillance Team (http://www.sanger.ac.uk/covid-team); Judith Breuer and Alex Alderton; Julianne Brown; Kathryn Harris; Leysa Forrest; Mark Kristiansen; Paola Niola; Paola Resende Silva; Patricia Dyal; Paul Randell; Rachel Williams; Roberto Amato; Sam Weeks; Sergi Castellano; Sonia Goncalves; Sunando Roy; Tony Brooks; Yasmin Panchbhaya
EPI_ISL_1498343 to 1498345, EPI_ISL_1498373	University Hospital Antwerp (UZA), Drie Eikenstraat 655, 2650 Edegem, Belgium	Labo Klinische Biologie, UZA	Basil Britto Xavier; Christine Lammens; Herman Goossens; Jasmine Coppens; Marie Le Mercier; Veerle Matheeussen
EPI_ISL_418274 to 418275, EPI_ISL_418277 to 418285, EPI_ISL_418433 to 418435, EPI_ISL_418437 to 418440, EPI_ISL_527916 to 528381, EPI_ISL_530122 to 530123, EPI_ISL_581668 to 582002, EPI_ISL_1388110, EPI_ISL_1388114, EPI_ISL_1388116, EPI_ISL_1388118 to 1388119, EPI_ISL_1388126, EPI_ISL_1388128, EPI_ISL_1388130, EPI_ISL_1388134, EPI_ISL_1388136 to 1388137, EPI_ISL_1388139, EPI_ISL_1388141, EPI_ISL_1388143, EPI_ISL_1388145, EPI_ISL_1388147, EPI_ISL_1388149, EPI_ISL_1388152, EPI_ISL_1388154, EPI_ISL_1388156, EPI_ISL_1388158, EPI_ISL_1388160, EPI_ISL_1388162, EPI_ISL_1388177, EPI_ISL_1388179, EPI_ISL_1388185, EPI_ISL_1388188, EPI_ISL_1388191, EPI_ISL_1388195, EPI_ISL_1388199, EPI_ISL_1388201, EPI_ISL_1388204, EPI_ISL_1388206, EPI_ISL_1388208, EPI_ISL_1388210, EPI_ISL_1388212, EPI_ISL_1388214, EPI_ISL_1388216, EPI_ISL_1388218, EPI_ISL_1388220, EPI_ISL_1388222, EPI_ISL_1388224, EPI_ISL_1388226, EPI_ISL_1388232, EPI_ISL_1388234 to 1388235, EPI_ISL_1388242, EPI_ISL_1388244, EPI_ISL_1388247, EPI_ISL_1388249, EPI_ISL_1388253, EPI_ISL_1388257, EPI_ISL_1388261, EPI_ISL_1388265, EPI_ISL_1388267, EPI_ISL_1388269, EPI_ISL_1388275, EPI_ISL_1388277, EPI_ISL_1388279, EPI_ISL_1388281, EPI_ISL_1388283, EPI_ISL_1388285, EPI_ISL_1388287, EPI_ISL_1388293, EPI_ISL_1388295, EPI_ISL_1388297, EPI_ISL_1388300 to 1388301, EPI_ISL_1388304, EPI_ISL_1388306, EPI_ISL_1388310, EPI_ISL_1388312, EPI_ISL_1388314, EPI_ISL_1388316, EPI_ISL_1388318, EPI_ISL_1388320, EPI_ISL_1388322, EPI_ISL_1388324, EPI_ISL_1388326, EPI_ISL_1388328, EPI_ISL_1388330, EPI_ISL_1388332, EPI_ISL_1388334, EPI_ISL_1388340, EPI_ISL_1388342, EPI_ISL_1388344, EPI_ISL_1388352, EPI_ISL_1388354, EPI_ISL_1388362, EPI_ISL_1388364, EPI_ISL_1388370, EPI_ISL_1388386, EPI_ISL_1388388, EPI_ISL_1388390, EPI_ISL_1388392, EPI_ISL_1388394, EPI_ISL_1388396 to 1388397, EPI_ISL_1388400, EPI_ISL_1388402, EPI_ISL_1388404, EPI_ISL_1388406, EPI_ISL_1388408, EPI_ISL_1388410, EPI_ISL_1388412, EPI_ISL_1388414, EPI_ISL_1388444, EPI_ISL_1388447, EPI_ISL_1388449, EPI_ISL_1388451, EPI_ISL_1388453, EPI_ISL_1388455, EPI_ISL_1388462, EPI_ISL_1388464, EPI_ISL_1388466, EPI_ISL_1388469, EPI_ISL_1388476, EPI_ISL_1388478, EPI_ISL_1388480, EPI_ISL_1388482, EPI_ISL_1388484, EPI_ISL_1388486, EPI_ISL_1388488, EPI_ISL_1388490, EPI_ISL_1388494, EPI_ISL_1388497, EPI_ISL_1388499, EPI_ISL_1388501, EPI_ISL_1388504, EPI_ISL_1388507, EPI_ISL_1388509, EPI_ISL_1388511, EPI_ISL_1388514, EPI_ISL_1388516, EPI_ISL_1388518, EPI_ISL_1388520, EPI_ISL_1388522, EPI_ISL_1388524, EPI_ISL_1388526, EPI_ISL_1388528, EPI_ISL_1388530, EPI_ISL_1388534, EPI_ISL_1388550, EPI_ISL_1388570, EPI_ISL_1388572, EPI_ISL_1388574, EPI_ISL_1388579 to 1388580, EPI_ISL_1388583, EPI_ISL_1388585, EPI_ISL_1388600, EPI_ISL_1388603 to 1388604, EPI_ISL_1388629, EPI_ISL_1388631, EPI_ISL_1388635, EPI_ISL_1388637, EPI_ISL_1388639, EPI_ISL_1388642, EPI_ISL_1388644, EPI_ISL_1388648, EPI_ISL_1388650, EPI_ISL_1388652 to 1388653, EPI_ISL_1388655, EPI_ISL_1388659, EPI_ISL_1388662, EPI_ISL_1388666, EPI_ISL_1388668 to 1388669, EPI_ISL_1388671, EPI_ISL_1388675, EPI_ISL_1388678 to 1388679, EPI_ISL_1388681, EPI_ISL_1388683, EPI_ISL_1388685, EPI_ISL_1388691, EPI_ISL_1388695, EPI_ISL_1388697, EPI_ISL_1388699, EPI_ISL_1388711, EPI_ISL_1388713, EPI_ISL_1388718, EPI_ISL_1388720, EPI_ISL_1388722 to 1388723, EPI_ISL_1388726 to 1388727, EPI_ISL_1388729, EPI_ISL_1388731, EPI_ISL_1388733, EPI_ISL_1388737, EPI_ISL_1388742, EPI_ISL_1388744, EPI_ISL_1388746, EPI_ISL_1388748, EPI_ISL_1388750 to 1388751, EPI_ISL_1388753, EPI_ISL_1388755, EPI_ISL_1388757, EPI_ISL_1388759, EPI_ISL_1388761, EPI_ISL_1388763, EPI_ISL_1388765, EPI_ISL_1388767, EPI_ISL_1388769, EPI_ISL_1388771, EPI_ISL_1388773 to 1388774, EPI_ISL_1388776, EPI_ISL_1388778, EPI_ISL_1388780 to 1388781, EPI_ISL_1388783, EPI_ISL_1388785, EPI_ISL_1388787, EPI_ISL_1388789, EPI_ISL_1388791, EPI_ISL_1388793, EPI_ISL_1388795, EPI_ISL_1388797, EPI_ISL_1388799, EPI_ISL_1388801, EPI_ISL_1388803, EPI_ISL_1388805, EPI_ISL_1388819, EPI_ISL_1388821, EPI_ISL_1388823, EPI_ISL_1388825, EPI_ISL_1388827, EPI_ISL_1388829 to 1388830, EPI_ISL_1388833 to 1388834, EPI_ISL_1388836, EPI_ISL_1388838, EPI_ISL_1388840, EPI_ISL_1388844 to 1388845, EPI_ISL_1388847, EPI_ISL_1388850, EPI_ISL_1388852, EPI_ISL_1388860, EPI_ISL_1388862, EPI_ISL_1388864, EPI_ISL_1388866, EPI_ISL_1388868 to 1388869, EPI_ISL_1388900, EPI_ISL_1388922, EPI_ISL_1388924, EPI_ISL_1388926, EPI_ISL_1388928, EPI_ISL_1388930, EPI_ISL_1388932 to 1388944, EPI_ISL_1388946 to 1388978, EPI_ISL_1388980 to 1388989, EPI_ISL_1388992 to 1389018, EPI_ISL_1389024 to 1389030, EPI_ISL_1389037, EPI_ISL_1389039, EPI_ISL_1389041, EPI_ISL_1389043, EPI_ISL_1389045, EPI_ISL_1389047, EPI_ISL_1389051, EPI_ISL_1389053, EPI_ISL_1389055, EPI_ISL_1389057, EPI_ISL_1389065, EPI_ISL_1389067, EPI_ISL_1389070, EPI_ISL_1389072, EPI_ISL_1389074, EPI_ISL_1389076, EPI_ISL_1389081, EPI_ISL_1389083, EPI_ISL_1389085, EPI_ISL_1389087, EPI_ISL_1389089, EPI_ISL_1389091, EPI_ISL_1389093, EPI_ISL_1389095, EPI_ISL_1389097, EPI_ISL_1389099, EPI_ISL_1389101, EPI_ISL_1389103, EPI_ISL_1389105, EPI_ISL_1389107, EPI_ISL_1389109, EPI_ISL_1389111, EPI_ISL_1389113 to 1389114, EPI_ISL_1389121, EPI_ISL_1389123 to 1389124, EPI_ISL_1389127, EPI_ISL_1389129, EPI_ISL_1389131, EPI_ISL_1389133, EPI_ISL_1389137			
see above	University Hospital Basel, Clinical Virology	University Hospital Basel, Clinical Bacteriology	A.University Hospital Basel; Adrian Egli; Alexander Gensch; Alfredo Mari; Christian Nickel; Clinical Bacteriology; Egli; Egli, A.; Hans Hirsch; Hans Pargger; Helena MB Seth-Smith; Hirsch, H.; Julia Bielicki; Karoline Leuzinger; Kirstine K. Soegaard; Leuzinger, K.; Madlen Stange; Manuel Battegay; Mari, A.; Martin Siegemund; Michael Osthoff; Michael Schweitzer; Myrta Brunner; Rita Schneider-Sliwa; Roland Bingisser; Roloff, T.; Sarah Tschudin-Sutter; Seth-Smith, H.; Simon Fuchs; Stefano Bassetti; Tim Roloff
EPI_ISL_418436	University Hospital Basel, Clinical Virology	University Hospital Basel, Clinical Virology	Egli, A.; Hirsch, H.; Leuzinger, K.; Mari, A.; Roloff, T.; Seth-Smith, H.
EPI_ISL_418271, EPI_ISL_418273	University Hospital Basel, Clinical Virology	University Hospital Basel, Labormedizin	Egli, A.; Hirsch, H.; Leuzinger, K.; Mari, A.; Roloff, T.; Seth-Smith, H.
EPI_ISL_1435908 to 1435909, EPI_ISL_1435919 to 1435920, EPI_ISL_1435933, EPI_ISL_1435966, EPI_ISL_1435968, EPI_ISL_1435970, EPI_ISL_1435972, EPI_ISL_1436022, EPI_ISL_1436024, EPI_ISL_1436033			
see above	University Hospital Centre Split	Croatian Institute of Public Health	Irena Tabain; Ivana Ferenak
EPI_ISL_483668 to 483685	University Hospital Zurich	Department of Biosystems Science and Engineering, ETH Zürich	Christian Beisel; Elodie Burcklen; Ina Nissen; Ivan Topolsky; Julia Martinez-Gomez; Mitch Levesque; Natascha Santacroce; Niko Beerenwinkel; Pedro Ferreira; Phil Cheng; Philipp Bosshard; Philipp Jablonski; Sarah Nadeau; Susana Posada-Céspedes; Tanja Stadler; Tobias Schär
EPI_ISL_524482, EPI_ISL_524782	University Hospital Zürich	Institute of Medical Virology, University of Zurich	Alexandra Trkola; Andrea Zbinden; Fiona Steiner; Gabriela Ziltener; Jon Huder; Jürg Böni; Maryam Zaheri; Michael Huber; Patrick Redli; Riccarda Capaul; Stefan Schmutz; Verena Kufner
EPI_ISL_454578, EPI_ISL_454581, EPI_ISL_454583, EPI_ISL_454588, EPI_ISL_454592, EPI_ISL_454595	University Hospital for Infectious Diseases "Dr. Fran Mihaljevi", Research Unit	University of Zagreb, Centre for research and knowledge transfer in biotechnology	Anamarija Slovic; Ivan-Christian Kurolt; Jelena Ivancic Jelecki
EPI_ISL_417485, EPI_ISL_420138, EPI_ISL_420152 to 420153, EPI_ISL_420310, EPI_ISL_590924 to 590926, EPI_ISL_590938 to 590944, EPI_ISL_590946 to 590947, EPI_ISL_590949 to 590950, EPI_ISL_1490215, EPI_ISL_1490258, EPI_ISL_1490263, EPI_ISL_1490301, EPI_ISL_1490380, EPI_ISL_1490406, EPI_ISL_1490424, EPI_ISL_1490429, EPI_ISL_1490443, EPI_ISL_1490446, EPI_ISL_1490448, EPI_ISL_1490456, EPI_ISL_1490554, EPI_ISL_1490605, EPI_ISL_1490607, EPI_ISL_1490622, EPI_ISL_1490627, EPI_ISL_1490632 to 1490633, EPI_ISL_1547571 to 1547572, EPI_ISL_1547574			
see above	University Hospital of Northern Norway, Department for Microbiology and Infectious Disease Control	Norwegian Institute of Public Health, Department of Virology	Atiya R Ali; Debech Nadia; Engebretsen Serina Beate; Garcia Llorente Ignacio; Hilde Elshaug; Hilde Vollan; Jon Bråte; Kamilla Heddeland Instefjord; Karoline Bragstad; Kathrine Stene-Johansen; Marie Paulsen Madsen; Olav Hungnes; Pedersen Benedikte Nevjen; Rasmus Riis Kopperud
EPI_ISL_478289 to 478371, EPI_ISL_484221 to 484253, EPI_ISL_484376 to 484389, EPI_ISL_499810, EPI_ISL_499828 to 499829, EPI_ISL_499858, EPI_ISL_499861, EPI_ISL_499869, EPI_ISL_499894 to 499895, EPI_ISL_499897, EPI_ISL_499910, EPI_ISL_499912 to 499913, EPI_ISL_499933, EPI_ISL_499950, EPI_ISL_499974 to 499983, EPI_ISL_1474645 to 1474653, EPI_ISL_1545973 to 1545983			
see above	University Hospitals Of Leicester NHS Trust and DeepSeq Nottingham	COVID-19 Genomics UK (COG-UK) Consortium	Christopher Holmes; Christopher Moore; Fei Sang; Joeseph Chappell; Johnny Debebe; Jonathan Ball; Julian Tang; Karlie Fallon; Matthew Carlisle; Matthew Loose; Nadine Holmes; Patrick McClure; Paul Bird; Thomas Helmer; Victoria Wright
EPI_ISL_1472386 to 1472407	University Hospitals Translational Laboratory (UHTL),	University Hospitals Translational Laboratory (UHTL),	Alouani, D.; Sadri, N.; Song, X.

	University Hospitals	University Hospitals	
EPI_ISL_415454 to 415459, EPI_ISL_415698 to 415708, EPI_ISL_429196 to 429197, EPI_ISL_429199 to 429201, EPI_ISL_429203 to 429204, EPI_ISL_429206 to 429215, EPI_ISL_429217 to 429223			
see above	University Hospitals of Geneva Laboratory of Virology	University Hospitals of Geneva Laboratory of Virology	Laubscher F.
EPI_ISL_1448529 to 1448787, EPI_ISL_1533102 to 1533422, EPI_ISL_1547472 to 1547501	University Hospitals of Geneva, Laboratory of Virology	HUG, Laboratory of Virology and the Health2030 Genome Center	Ana Rita Goncalves; Deborah Penet; Emmanouil Dermitzakis; Henri Pegeot; Ioannis Xenarios; Keith Harshman; Laurent Kaiser; Lorenzo Cerutti; Melyssa Elies; Samuel Cordey
EPI_ISL_1464109 to 1464113, EPI_ISL_1464588 to 1464589, EPI_ISL_1464591 to 1464600	University Medical Center Hamburg Eppendorf	Heinrich Pette Institute, Leibniz Institute for Experimental Virology	Adam Grundhoff; Alexis Robitaille; Johannes Knobloch; Martin Aepfelbacher; Nicole Fischer; Thomas Günther
EPI_ISL_492181 to 492182	University of Arkansas for Medical Sciences (UAMS)	Department of Biomedical Informatics, University of Arkansas for Medical Sciences (UAMS)	David W Ussery; Piroon Jenjaroenpun; Thidathip Wongsurawat
EPI_ISL_1469945 to 1469957, EPI_ISL_1469960, EPI_ISL_1469962 to 1469963, EPI_ISL_1520140, EPI_ISL_1520169, EPI_ISL_1520172, EPI_ISL_1520174, EPI_ISL_1520177, EPI_ISL_1520180, EPI_ISL_1520366, EPI_ISL_1520368, EPI_ISL_1520370 to 1520371, EPI_ISL_1520373, EPI_ISL_1520375, EPI_ISL_1520377 to 1520380			
see above	University of Bari Biomedical Sciences and Human Oncology	University of Bari Biomedical Sciences and Human Oncology	Accogli M.; Chironna M.; Loconsole D.; Sallustio A.; sallustio A.
EPI_ISL_444279 to 444312, EPI_ISL_447922 to 447944, EPI_ISL_456666 to 456676, EPI_ISL_473306 to 473450, EPI_ISL_477727 to 477782, EPI_ISL_483880 to 483912, EPI_ISL_484218 to 484220, EPI_ISL_493441 to 493451, EPI_ISL_526287 to 526336, EPI_ISL_529218 to 529223, EPI_ISL_529234 to 529235, EPI_ISL_529237, EPI_ISL_529239, EPI_ISL_529251 to 529252, EPI_ISL_529255 to 529256, EPI_ISL_529262, EPI_ISL_529268, EPI_ISL_529275, EPI_ISL_529277 to 529278, EPI_ISL_529289, EPI_ISL_529291, EPI_ISL_529298, EPI_ISL_529300, EPI_ISL_529312 to 529314, EPI_ISL_529320 to 529340, EPI_ISL_529357 to 529372, EPI_ISL_529384, EPI_ISL_529386, EPI_ISL_529390 to 529406, EPI_ISL_529408, EPI_ISL_529420 to 529421, EPI_ISL_529424 to 529430, EPI_ISL_529434 to 529435, EPI_ISL_529438 to 529444, EPI_ISL_529448, EPI_ISL_529453, EPI_ISL_529461 to 529462, EPI_ISL_529468, EPI_ISL_529470 to 529475, EPI_ISL_529480, EPI_ISL_529482, EPI_ISL_529485, EPI_ISL_529493 to 529497, EPI_ISL_529499, EPI_ISL_529508, EPI_ISL_529512, EPI_ISL_529514 to 529515, EPI_ISL_529517 to 529518, EPI_ISL_529520, EPI_ISL_529522, EPI_ISL_529590 to 529655, EPI_ISL_529661 to 529666, EPI_ISL_576877 to 576896, EPI_ISL_594484 to 594616, EPI_ISL_611550, EPI_ISL_611554, EPI_ISL_611556, EPI_ISL_611608, EPI_ISL_611612, EPI_ISL_611671, EPI_ISL_611692, EPI_ISL_611696, EPI_ISL_611700, EPI_ISL_611705, EPI_ISL_611776, EPI_ISL_611814, EPI_ISL_611825, EPI_ISL_611847, EPI_ISL_611855 to 611856, EPI_ISL_611858 to 611860, EPI_ISL_612011, EPI_ISL_612015, EPI_ISL_612047, EPI_ISL_612073, EPI_ISL_612207 to 612260, EPI_ISL_1386476 to 1386522, EPI_ISL_1386524 to 1386528, EPI_ISL_1386530 to 1386533, EPI_ISL_1386538, EPI_ISL_1386540 to 1386570, EPI_ISL_1474382 to 1474383, EPI_ISL_1474385 to 1474395, EPI_ISL_1474399 to 1474402, EPI_ISL_1474404, EPI_ISL_1474406, EPI_ISL_1474411 to 1474430, EPI_ISL_1474432 to 1474440, EPI_ISL_1474442, EPI_ISL_1474444, EPI_ISL_1474446 to 1474453, EPI_ISL_1474456 to 1474461, EPI_ISL_1474464 to 1474466, EPI_ISL_1474468 to 1474488, EPI_ISL_1474490 to 1474491, EPI_ISL_1474493 to 1474529			
see above	University of Birmingham	COVID-19 Genomics UK (COG-UK) Consortium	Alex Richter; Andrew Bosworth; Andrew Bosworth. Queen Elizabeth Hospital: Anna Casey; Andrew D Beggs; Andrew D Beggs // PHE Heartlands Lab: Husam Osman; Andrew D Beggs PHE Heartlands Lab: Husam Osman; Celina M Whalley; Charlotte Poxon; Claire McMurray; Husam Osman; Institute of Microbiology; Joanne Stockton; Josh Quick; Kasun Wanigasooriya; Loman Lab: Claire McMurray; Mike Kidd; Nicholas Loman; Nicholas Loman // UHB Lab: Celina M Whalley; Nicholas Loman. University of Birmingham Testing Laboratory: Celina M Whalley; Oliver Pickles; Radoslaw Poplawski; Samuel Nicholls; University of Birmingham Testing Laboratory: Celina M Whalley; University of Birmingham: Claire McMurray; Will Rowe
EPI_ISL_447887 to 447896, EPI_ISL_548374, EPI_ISL_548380, EPI_ISL_548382, EPI_ISL_548397, EPI_ISL_548414, EPI_ISL_548435, EPI_ISL_548447, EPI_ISL_548452, EPI_ISL_548458, EPI_ISL_548474, EPI_ISL_548477, EPI_ISL_548479, EPI_ISL_548486, EPI_ISL_548490			
see above	University of California, Davis	Chan-Zuckerberg Biohub	CZB Cliahub Consortium
EPI_ISL_476068 to 476077, EPI_ISL_477008 to 477013	University of Debrecen, Department of Medical Microbiology	National Laboratory of Virology, Szentágothai Research Centre	Balázs Somogyi; Brigitta Zana; Endre Gábor Tóth; Eszter Csoma; Ferenc Jakab; Gábor Kemenesi
EPI_ISL_457028 to 457029, EPI_ISL_457031 to 457136, EPI_ISL_457138 to 457147, EPI_ISL_457149 to 457151, EPI_ISL_457153 to 457258, EPI_ISL_471911 to 471992, EPI_ISL_478274 to 478279, EPI_ISL_478281 to 478288, EPI_ISL_512336 to 512341, EPI_ISL_540579, EPI_ISL_540582 to 540583, EPI_ISL_577220 to 577298, EPI_ISL_577346, EPI_ISL_577359 to 577380, EPI_ISL_577544, EPI_ISL_584883 to 584905, EPI_ISL_585533 to 585582, EPI_ISL_611539 to 611540, EPI_ISL_611551 to 611552, EPI_ISL_611599, EPI_ISL_611607, EPI_ISL_611615 to 611620, EPI_ISL_611641, EPI_ISL_611701 to 611703, EPI_ISL_611836 to 611838, EPI_ISL_611843 to 611844, EPI_ISL_611939 to 611940, EPI_ISL_611976, EPI_ISL_611987, EPI_ISL_612056 to 612057, EPI_ISL_612095 to 612096, EPI_ISL_612118, EPI_ISL_612130, EPI_ISL_612375 to 612393, EPI_ISL_612395 to 612400, EPI_ISL_1474530 to 1474536, EPI_ISL_1474538 to 1474541, EPI_ISL_1474543 to 1474552, EPI_ISL_1474554 to 1474563, EPI_ISL_1474603 to 1474643, EPI_ISL_1545766 to 1545767, EPI_ISL_1545770 to 1545773, EPI_ISL_1545775, EPI_ISL_1545777 to 1545778, EPI_ISL_1545780 to 1545781, EPI_ISL_1545783 to 1545793, EPI_ISL_1545795 to 1545820, EPI_ISL_1545822 to 1545841, EPI_ISL_1545844 to 1545845, EPI_ISL_1545944, EPI_ISL_1545946 to 1545959, EPI_ISL_1545969			
see above	University of Exeter	COVID-19 Genomics UK (COG-UK) Consortium	Aaron Jeffries; Audrey Farbos; Ben Temperton; Jane Masoli; Joanna Warwick-Dugdale; Michelle Michelsen; Robyn Manley; Stephen Michell
EPI_ISL_447903, EPI_ISL_447905, EPI_ISL_455682, EPI_ISL_468719 to 468723	University of Florida	University of Florida	Alam; C.-Y.; C.J.; Elbadry; Gibson; J.A.; J.C.; J.G.; J.G. Jr. and Lednicky; Lauzardo; Lauzardo, M.; Lednicky; M. and Morris; M.A.; M.M.; Morris; Stephenson; Subramaniam, K.; T.B.; Waltzek; Wu
EPI_ISL_447589	University of Florida, Lednicky Lab	University of Florida, Lednicky Lab	Alam; C.J.; Elbadry; Gibson; J.A.; J.C.; J.G. Jr. and Lednicky; M.A.; M.M.; Morris; Stephenson; Subramaniam, K.; T.B.; Waltzek
EPI_ISL_540442 to 540468, EPI_ISL_1492779 to 1492805, EPI_ISL_1492973 to 1493011, EPI_ISL_1533937 to 1533976	University of Liège COVID-19 testing center	GIGA Medical Genomics	Bouchra Boujemla; Cécile Meex; Emmanuel André; Fabrice Bureau; Keith Durkin; Laurent Gillet; Marc Van Ranst; Maria Artesi; Marie-Pierre Hayette; Nathalie Renotte; Pierrette Melin; Raphaël Boreux; Sébastien Bontems; Vincent Bours; Wouter Coppieters
EPI_ISL_1490186	University of Ljubljana, Faculty of Veterinary Medicine	Institute of Microbiology and Immunology, Faculty of Medicine, University of Ljubljana	Ana Koevar; Brigita Slavec; Jožko Ranik; Mario Poljak; Matic Brvar; Milan Matko; Miša Korva; Olga Zorman Rojs; Samo Zakotnik; Tatjana Avši - Županc; Tomaž Mark Zorec
EPI_ISL_479759 to 479775, EPI_ISL_513265 to 513293, EPI_ISL_527381 to 527399, EPI_ISL_534693 to 534698, EPI_ISL_602306 to 602328, EPI_ISL_605147	University of Miami Immunology and Histocompatibility Laboratory	University of Miami Immunology and Histocompatibility Laboratory	Emilio Margolles-Clark; MD; PhD; PhD and Phillip Ruiz
EPI_ISL_484709 to 484806, EPI_ISL_487229 to 487230, EPI_ISL_495563 to 495596, EPI_ISL_578082 to 578167, EPI_ISL_578169 to 578174, EPI_ISL_578185, EPI_ISL_583249 to 583425, EPI_ISL_584095 to 584097, EPI_ISL_590699 to 590752, EPI_ISL_596569 to 596626, EPI_ISL_610048 to 610117, EPI_ISL_610246 to 610248, EPI_ISL_1404249 to 1404373, EPI_ISL_1406251 to 1406271, EPI_ISL_1491390 to 1491514, EPI_ISL_1493040 to 1493058			
see above	University of Michigan Clinical Microbiology Laboratory	Lauring Lab, University of Michigan, Department of Microbiology and Immunology	Valesano; Valesano et al.
EPI_ISL_1527029 to 1527090, EPI_ISL_1527237 to 1527264, EPI_ISL_1527399 to 1527443	University of Mississippi Medical Center, Department of Pathology	University of Mississippi Medical Center, Molecular and Genomics Core Facility	Ashley C. Johnson; D. Ashley Robinson; Ithiel J. Frame; Krishna K. Ayyalasomayajula; Michael R. Garrett
EPI_ISL_1508853 to 1508889, EPI_ISL_1531745 to 1531775	University of Oregon COVID-19 MAP Laboratory	University of Oregon Genomics and Cell Characterization Core Facility (GC3F)	Ariana White; Douglas Turnbull; Jason Carriere; Jeff Bishop
EPI_ISL_476897	University of South Carolina Functional Genomics Core	University of South Carolina Functional Genomics Core	Michael Shtutman
EPI_ISL_476078, EPI_ISL_477615 to 477625	University of Szeged, Institute of Clinical Microbiology	National Laboratory of Virology, Szentágothai Research Centre	Balázs Somogyi; Brigitta Zana; Endre Gábor Tóth; Ferenc Jakab; Gábor Kemenesi; Terhes Gabriella
EPI_ISL_485001 to 485002, EPI_ISL_485388 to 485396	University of Ulsan College of Medicine and Asan Medical Center	University of Ulsan College of Medicine and Asan Medical Center	Heungsup Sung; Jaewoong Lee; Jina Lee; Jiwon Jung; Jongsik Chun; Kihyun Lee; Kuenyoul Park; Kyu-Hwa Hur; Mauricio Chalita; Mi-Na Kim; Seok-Hwan Yoon; Sung-Han Kim; and Hae Kyung Lee
EPI_ISL_524444	University of Washington Virology Lab	Fred Hutchinson Cancer Research Center	Greninger, A.; Jerome, K.; Roychoudhury, P.
EPI_ISL_524437 to 524443	University of Washington Virology Lab	Laboratory Medicine, University of Washington	Greninger, A.; Jerome, K.; Roychoudhury, P.
EPI_ISL_500500 to 500502, EPI_ISL_501083 to 501164, EPI_ISL_513632 to 513638, EPI_ISL_515270 to 515286	University of Washington Virology Lab	University of Washington Virology Lab	Alexander Greninger; Amin Addetia; Hong Xie; Keith R Jerome; Lasata Shrestha; Meei-Li Huang; Pavitra Roychoudhury; Truong Nguyen; Victoria M Rachleff
EPI_ISL_529155 to 529161, EPI_ISL_529170, EPI_ISL_529200	University of Washington, Laboratory Medicine	University of Washington, Laboratory Medicine	Greninger, A.; Jerome, K.; Roychoudhury, P.
EPI_ISL_418809	University of Wisconsin - Madison: Influenza Research Institute	University of Wisconsin Madison, AIDS Vaccine Research Laboratories	Gage Moreno; Katarina Braun; Peter Halfmann; et al.
EPI_ISL_416489, EPI_ISL_416491 to 416492, EPI_ISL_416523, EPI_ISL_417200 to 417204, EPI_ISL_417504 to 417510, EPI_ISL_417512 to 417517, EPI_ISL_421283 to 421336, EPI_ISL_421338 to 421343, EPI_ISL_425142 to 425176, EPI_ISL_427427 to 427462, EPI_ISL_428252 to 428345, EPI_ISL_428724 to 428732, EPI_ISL_428935 to 428936, EPI_ISL_434607 to 434615, EPI_ISL_436564 to 436640, EPI_ISL_450700 to 450722, EPI_ISL_480351 to 480413, EPI_ISL_484807 to 485000, EPI_ISL_491299 to 491426, EPI_ISL_495460 to 495509, EPI_ISL_509799 to 509998, EPI_ISL_510524 to 510525, EPI_ISL_516432 to 516549, EPI_ISL_522881 to 522939, EPI_ISL_527794 to 527808, EPI_ISL_536572 to 536655, EPI_ISL_536658 to 536784, EPI_ISL_536786 to 536787, EPI_ISL_541540 to 541550, EPI_ISL_541566 to 541567, EPI_ISL_541569 to 541648, EPI_ISL_547581 to 547583, EPI_ISL_605405 to 605501, EPI_ISL_605504 to 605779,			

EPI_ISL_1403624 to 1403642, EPI_ISL_1403646 to 1403664, EPI_ISL_1403810 to 1403826, EPI_ISL_1404106 to 1404129, EPI_ISL_1422216 to 1422236, EPI_ISL_1493065, EPI_ISL_1493110 to 1493122, EPI_ISL_1493125 to 1493144			
see above	University of Wisconsin-Madison AIDS Vaccine Research Laboratories	University of Wisconsin-Madison AIDS Vaccine Research Laboratories	Gage Moreno; Katarina Braun; et al. AIDS Vaccine Research Laboratories
[truncated: 3,760,201 more chars]
